# Mechanisms of hepatic steatosis in chickens: integrated analysis of the host genome, molecular phenomics and gut microbiome

**DOI:** 10.1093/gigascience/giae023

**Published:** 2024-06-05

**Authors:** Congjiao Sun, Fangren Lan, Qianqian Zhou, Xiaoli Guo, Jiaming Jin, Chaoliang Wen, Yanxin Guo, Zhuocheng Hou, Jiangxia Zheng, Guiqin Wu, Guangqi Li, Yiyuan Yan, Junying Li, Qiugang Ma, Ning Yang

**Affiliations:** State Key Laboratory of Animal Biotech Breeding, Department of Animal Genetics and Breeding, College of Animal Science and Technology, China Agricultural University, Beijing 100193, China; State Key Laboratory of Animal Biotech Breeding, Department of Animal Genetics and Breeding, College of Animal Science and Technology, China Agricultural University, Beijing 100193, China; State Key Laboratory of Animal Biotech Breeding, Department of Animal Genetics and Breeding, College of Animal Science and Technology, China Agricultural University, Beijing 100193, China; State Key Laboratory of Animal Biotech Breeding, Department of Animal Genetics and Breeding, College of Animal Science and Technology, China Agricultural University, Beijing 100193, China; State Key Laboratory of Animal Biotech Breeding, Department of Animal Genetics and Breeding, College of Animal Science and Technology, China Agricultural University, Beijing 100193, China; State Key Laboratory of Animal Biotech Breeding, Department of Animal Genetics and Breeding, College of Animal Science and Technology, China Agricultural University, Beijing 100193, China; State Key Laboratory of Animal Biotech Breeding, Department of Animal Genetics and Breeding, College of Animal Science and Technology, China Agricultural University, Beijing 100193, China; State Key Laboratory of Animal Biotech Breeding, Department of Animal Genetics and Breeding, College of Animal Science and Technology, China Agricultural University, Beijing 100193, China; State Key Laboratory of Animal Biotech Breeding, Department of Animal Genetics and Breeding, College of Animal Science and Technology, China Agricultural University, Beijing 100193, China; Beijing Engineering Research Centre of Layer, Beijing 101206, China; Beijing Engineering Research Centre of Layer, Beijing 101206, China; Beijing Engineering Research Centre of Layer, Beijing 101206, China; State Key Laboratory of Animal Biotech Breeding, Department of Animal Genetics and Breeding, College of Animal Science and Technology, China Agricultural University, Beijing 100193, China; State Key Laboratory of Animal Biotech Breeding, Department of Animal Genetics and Breeding, College of Animal Science and Technology, China Agricultural University, Beijing 100193, China; State Key Laboratory of Animal Biotech Breeding, Department of Animal Genetics and Breeding, College of Animal Science and Technology, China Agricultural University, Beijing 100193, China

**Keywords:** chickens, hepatic steatosis, genetics, microbiota, integrative analysis

## Abstract

Hepatic steatosis is the initial manifestation of abnormal liver functions and often leads to liver diseases such as nonalcoholic fatty liver disease in humans and fatty liver syndrome in animals. In this study, we conducted a comprehensive analysis of a large chicken population consisting of 705 adult hens by combining host genome resequencing; liver transcriptome, proteome, and metabolome analysis; and microbial 16S ribosomal RNA gene sequencing of each gut segment. The results showed the heritability (h^2^ = 0.25) and duodenal microbiability (m^2^ = 0.26) of hepatic steatosis were relatively high, indicating a large effect of host genetics and duodenal microbiota on chicken hepatic steatosis. Individuals with hepatic steatosis had low microbiota diversity and a decreased genetic potential to process triglyceride output from hepatocytes, fatty acid β-oxidation activity, and resistance to fatty acid peroxidation. Furthermore, we revealed a molecular network linking host genomic variants (GGA6: 5.59–5.69 Mb), hepatic gene/protein expression (*PEMT*, phosphatidyl-ethanolamine N-methyltransferase), metabolite abundances (folate, S-adenosylmethionine, homocysteine, phosphatidyl-ethanolamine, and phosphatidylcholine), and duodenal microbes (genus *Lactobacillus*) to hepatic steatosis, which could provide new insights into the regulatory mechanism of fatty liver development.

## Introduction

Lipid metabolism plays a crucial role in maintaining animal life and ensuring normal physiological functions. Dysregulations of fat metabolism can lead to fatty liver diseases, and hepatic steatosis is one of their first symptoms, which can progress to nonalcoholic fatty liver disease (NAFLD) in humans [1] or fatty liver syndrome (FLS) in farm animals with more severe cases developing blood clots and liver rupture [2, 3]. Robust evidence from human studies, especially twin-based studies, has provided in-depth knowledge that fatty liver is strongly influenced by host genetics with heritability ranging from 0.20 to 0.70 [4–7]. Additionally, genetic variations that strongly influence NAFLD in humans have been confirmed by many studies, such as the I148M mutation in the *PNPLA3* gene and the *E167K* mutation in the *TM6SF2* gene [8]. Experts have reached a consensus that metabolic (dysfunction) associated fatty liver disease (MAFLD) may be a more appropriate and inclusive definition than NAFLD in humans [9]. This change emphasizes the significance of lipid metabolism homeostasis, which is involved in hepatic *de novo* lipogenesis, β-oxidation, very low-density lipoprotein (VLDL) secretion, and gut absorption [10, 11]. Dysregulation of metabolism in any of these processes may cause the development of a fatty liver.

The liver and intestines communicate bidirectionally through the “gut–liver axis” [12, 13], finely tuning the host’s physiological state through reciprocal metabolite exchange and immune responses. Clinical studies have shown the association of microbial metabolites with the severity of NAFLD [14] and non-alcoholic steatohepatitis (NASH) [15]. Specifically, hepatic steatosis in experimental mice with NAFLD was induced by the commensal metabolite phenylacetate and exacerbated after fecal microbiota transplantation (FMT) from obese women [16]. Additionally, commensal-derived D-lactate, which supports pathogen clearance in hepatic Kupffer cells, was impaired after antibiotic therapy [17]. Gut microbiota ferments indigestible carbohydrates and proteins, producing metabolites, including short-chain fatty acids and succinate, that are vital for gut homeostasis and liver substrate metabolism [18–20]. Numerous studies indicated a positive impact of these metabolites in preventing and treating obesity [21, 22]. Notably, microbial diversity [23] and Bacteroidetes/Firmicutes ratio [24] were identified negatively correlated with obesity and NAFLD. However, elevated levels of *Lactobacillus* are commonly found in patients with MAFLD [25–27]. Besides, FLS frequently occurred in high-production hens, leading to noninfectious cause of death [28] and decreased egg production [29]. Therefore, it is important to establish a systematic chicken lipid metabolism regulation model that combines host genetics and gut microbiota to understand how various components, such as genomic variations, genes, proteins, metabolites, and gut microbes, interact to control lipid metabolism.

Compared with other animal models, studies on the regulatory mechanism of fatty liver are more prevalent in humans, especially the population-based design. For farm animals including chickens, research on molecular regulation of a fatty liver is limited [2, 30, 31]. Farm animals are more suitable to analyze the genetic and microbial involvement in a fatty liver by a population-based design. Farm animals have more genetic variations than mouse models, whose peculiarity was highly inbred, making farm animals more representative of the diversity seen in natural populations. Comparative gene mapping has revealed a closer genomic organization between humans and farm animals, compared to the mouse [32]. Additional, animal samples are more accessible than those from humans [33], such as obtaining microbiota from different gut segments in a large population. Chickens are ideal for large-scale studies due to their high reproductive capacity and low cost. Fatty liver in chickens leads to significant declines in egg production and quality, making it a major cause of noninfectious mortality [29, 34]. Therefore, studying the molecular control of fatty liver in chickens is scientifically valuable and economically important, offering potential for molecular breeding to reduce the condition.

In this study, we performed whole-genome resequencing, liver transcriptome, and 16S ribosomal RNA (rRNA) gene sequencing (duodenum, jejunum, ileum, cecum, and feces) on 705 Rhode Island Red chickens, coupled with proteomic and metabolomic sequencing on several selected individuals, to analyze the regulatory networks of hepatic steatosis. The genomic and microbial data were then used to systematically evaluate the contribution of the host genetics (h^2^ = 0.25) and gut microbial community (duodenal m^2^ = 0.26) to hepatic steatosis. We further identified gene PEMT and duodenal genus *Lactobacillus* involved in the methionine cycle as crucial contributors to hepatic steatosis, which would be beneficial to study NAFLD in farm animal or humans by providing innovative ideas and methods.

## Results

### Hepatic steatosis classification and phenotype characterization

**Figure 1:**
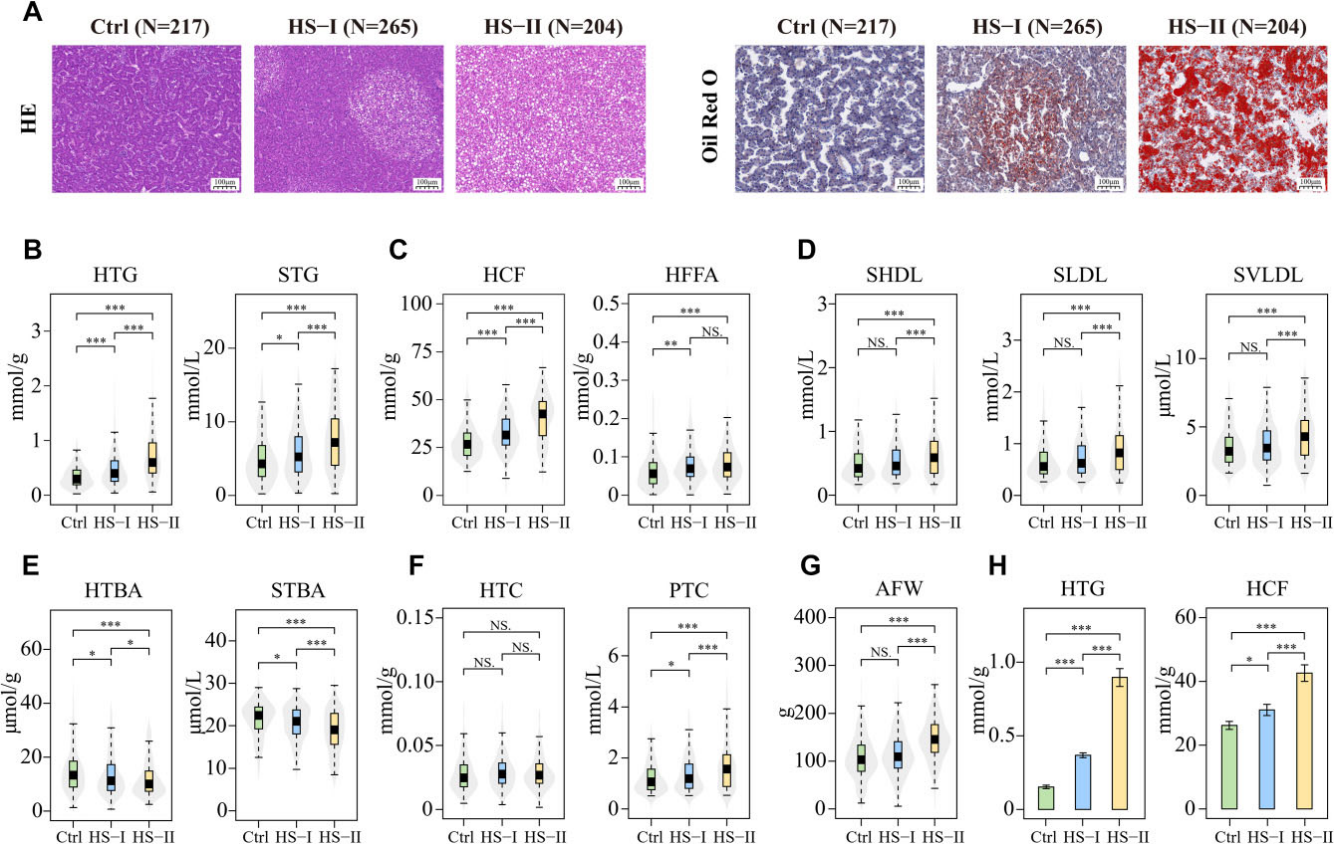
Phenotypic profiling of the histochemical stained sections of the chicken livers and hepatic and serum biochemical indicators among HSC (eHSC) groups. (A) Micrographs of H&E-stained and Oil Red O–stained whole sections of the chicken liver (scale bar: 100 μm). *n* = 673. (B–D) Boxplots of hepatic and plasmic triglyceride (HTG and STG, respectively) (B), hepatic crude fat (HCF) and free fatty acids (HFFAs) (C), serum high-density lipoprotein (SHDL), low-density lipoprotein (SLDL), and very low-density lipoprotein (SVLDL) (D), showing high levels in the hepatic steatosis group (*P*_adj_ < 0.05, post hoc Wilcoxon rank-sum test). *n* = 673. (E) Boxplots of hepatic and serum total bile acids (HTBAs and STBAs, respectively) showing a significant inverse correlation with hepatic steatosis (*P*_adj_ < 0.05, post hoc Wilcoxon rank-sum test). *n* = 673. (F) Boxplots of the association of hepatic and serum total cholesterol (HTC and STC, respectively) among HSC groups (*P*_adj_ < 0.05 for hepatic TC and *P*_adj_ < 0.05 for serum, post hoc Wilcoxon rank-sum test). *n* = 673. (G) Boxplots of the abdominal fat weight (AFW), showing a significant increase with hepatic steatosis (*P*_adj_ < 0.05, post hoc Wilcoxon rank-sum test). *n* = 673. (H) Bar plots of hepatic triglyceride (HTG) and crude fat (HCF), showing significant increases with hepatic steatosis in eHSC groups (*P*_adj_ < 0.05, post hoc Wilcoxon rank-sum test). Data are from *n* = 30 biological replicates. *P < 0.05, **P < 0.01, ***P < 0.001.

A total of 686 adult hens were used for hepatic steatosis classification (HSC) in livers with hematoxylin and eosin (H&E)–stained whole sections, and 1 control (Ctrl, *n* = 217, healthy) and 2 hepatic steatosis groups from mild (HS-Ⅰ, *n* = 265) to severe (HS-Ⅱ, *n* = 204) were classified in total (Fig. 1A). Hepatic triglyceride (HTG) and serum triglyceride (STG) contents increased with the severity of hepatic steatosis from 0.33 to 0.72 mmol/g in the liver (mean value, Ctrl vs. HS-Ⅱ, *P*_adj_ < 0.05, β = 0.32, post hoc Wilcoxon rank-sum test) and from 4.9 to 7.4 mmol/L in serum (mean value, Ctrl vs. HS-Ⅱ, *P*_adj_ < 0.05, β = 2.40, post hoc Wilcoxon rank-sum test, Fig. 1B). Hepatic crude fat (HCF) and hepatic free fatty acids (HFFAs), serum high-density lipoprotein (SHDL), low-density lipoprotein (SLDL), and very low-density lipoprotein (SVLDL) all displayed similar patterns (*P*_adj_ < 0.05, β_HCF_ = −14.08, β_HFFA_ = −0.02, β_SHDL_ = −0.12, β_SLDL_ = −0.18, β_SVLDL_ = −0.82, post hoc Wilcoxon rank-sum test, Fig. 1C and D). However, hepatic total bile acid (HTBA) exhibited the opposite trend, which decreased from 16.16 to 12.19 mmol/g (mean value, Ctrl vs. HS-Ⅱ, *P*_adj_ < 0.05, β = 2.91, post hoc Wilcoxon rank-sum test, Fig. 1E). Hepatic total cholesterol (HTC) did not change with a fatty liver, but serum total cholesterol (STC) increased significantly from 1.30 to 1.83 mmol/L (mean value, Ctrl vs. HS-Ⅱ, *P*_adj_ < 0.05, β = −0.34, post hoc Wilcoxon rank-sum test, Fig. 1F). With the severity of hepatic steatosis, the degree of obesity also increased with the abdominal fat weight (AFW) increasing from 106.6 to 145.8 g (mean value, Ctrl vs. HS-Ⅱ, *P*_adj_ < 0.05, β = 39.70, post hoc Wilcoxon rank-sum test, Fig. 1G).

The abovementioned quantitative phenotypes exhibited predominantly positive correlations with HSC, ranging from 0.176 (between HSC and HFFA) to 0.426 (between HSC and HCF) (*P*_adj_ < 0.05, Supplementary Fig. S1). STG had a relatively high correlation coefficient with SHDL (0.86), SLDL (0.87), SVLDL (0.82), serum total bile acid (STBA) (−0.95), and STC (0.89) (*P*_adj_ < 0.05, Supplementary Fig. S1). However, hepatic steatosis is in fact a quantitative trait, making artificial hepatic steatosis classification imprecise for intermediate individuals between each of 2 adjacent HSC groups. Hence, we further established an extreme hepatic steatosis classification (eHSC) model with more strict criteria, consisting of 3 groups: an eCtrl group (*n* = 30, without lipid droplets), eHS-Ⅰ group (*n* = 30, lipid droplets accounted for 30%−40% of H&E-stained images), and eHS-Ⅱ (*n* = 30, lipid droplets accounted for >90% of H&E-stained images), which were verified by Oil Red O staining. Among eHSC groups, lipid-related indicators, such as HTG and HCF quantities, exhibited even greater differences, that is, 2 to 3 times higher in the eHS-Ⅱ group than in the eCtrl group (Fig. 1H, *P*_adj_ < 0.01, β_HTG_ = −0.66, β_HCF_ = −18.29, post hoc Wilcoxon rank-sum test, other indicators see Supplementary Fig. S2). eHSC as a complementary model of HSC may aid in identifying crucial molecules for hepatic steatosis.

### Genetic determinants of hepatic steatosis

#### Genomic variants

**Figure 2:**
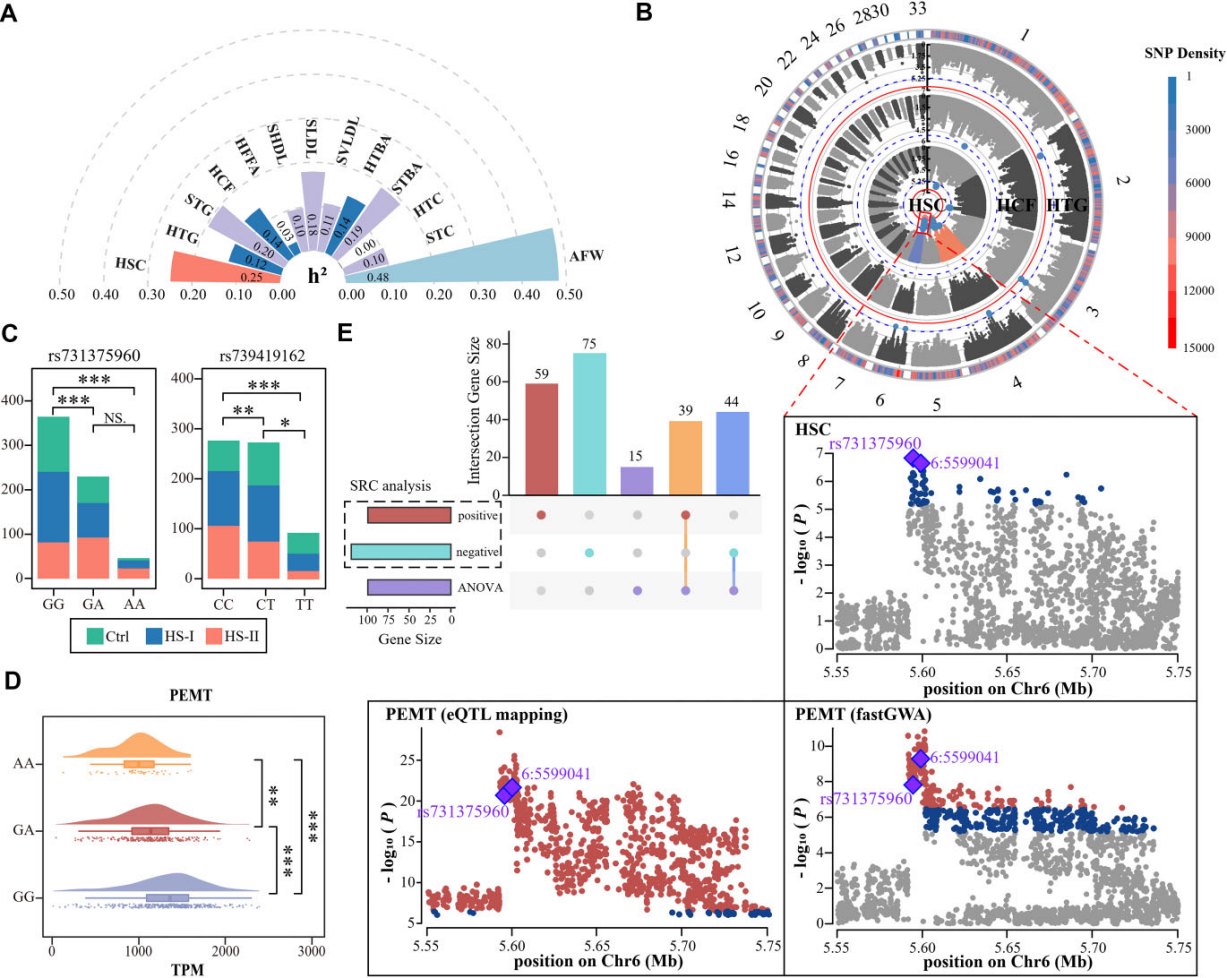
Genomic determinants of HSC and its related indicators. (A) The SNP-based heritability estimations of HSC and biochemical indicators. HSC: hepatic steatosis classification; HTG and STG: hepatic and serum triglyceride, respectively; HCF: hepatic crude fat; HFFA: hepatic free fatty acid; SHDL: serum high-density lipoprotein; SLDL: serum low-density lipoprotein; SVLDL: serum very low-density lipoprotein; HTBA and STBA: hepatic and serum total bile acid, respectively; HTC and STC: hepatic and serum total cholesterol, respectively; AFW: abdominal fat weight. *n* ≥ 673. (B) Circular Manhattan plots of GWAS for HSC, HCF, and HTG. Gray, dark blue, and reddish-brown dots indicate nonsignificant, suggestively significant, and significant SNPs, respectively. Colocalization of *trans*-eQTLs of the phosphatidylethanolamine N-methyltransferase (*PEMT*) gene in the liver and GWAS loci of HSC in chickens on chromosome 6 identified 2 colocalized SNPs, which were the significant *trans*-eQTL of *PEMT* and the top GWAS signals of HSC. *n* ≥ 668. (C) Stacked bar plots of the comparison of individuals distributing in different HSC levels across the 3 genotypes of these 2 eVariants (rs731375960 and rs731375960, *P*_adj_ < 0.05, chi-squared test). *n* = 673. (D) Raincloud plot shows the expression levels of the *PEMT* gene in the liver across the 3 genotypes of the top eVariant (rs731375960, *P*_adj_ < 0.05, post hoc Wilcoxon rank-sum test). *n* = 668. (E) Spearman’s rank-based correlation (SRC) analysis and analysis of variance (ANOVA) were performed to ascertain candidate genes in the liver for hepatics steatosis. *n* = 673. *P < 0.05, **P < 0.01, ***P < 0.001.

To investigate the influence of host genetics on hepatic steatosis, we performed whole-genome resequencing of 686 chickens. Up to 1.94 Tb of clean reads were generated, and each individual reached an 8.13-fold depth and 95.06% genome coverage. After stringent filtering, a final set of 5,904,820 single-nucleotide polymorphisms (SNPs) (6.17 SNPs per kb) was obtained (Supplementary Table S1). Estimation of SNP-based heritability (h^2^) was performed on fat metabolism and storage-related traits. AFW had the highest h^2^ (0.48), followed by HSC (0.25) and serum HTG (0.20), indicating that host genetics had a substantial role in determination of fat accumulation and storage (Fig. 2A). Next, we performed genome-wide analysis (GWA) analysis of HSC with the abdominal fat percentage (AFP) as an additional covariate and identified 2 genomic peaks (GGA6: 5.59–5.69 Mb; GGA4: 75.6–76.4 Mb) that were significantly associated with HSC. Additionally, the *P* -values of the top SNPs rs731375960 at GGA6 (6:5594550) and rs739419162 at GGA4 (4:75758710) were all less than 3.32 × 10^−7^ (Fig. 2B, Supplementary Table S2). Individuals with different genotypes of top variants had significantly different HSC ratios (*P*_adj_ < 0.05, chi-squared test, Fig. 2C).

#### eVariants of hepatic steatosis

Genes that harbor or are near to genomic peaks are candidate genes for target traits, which is known as *cis*-regulation. However, in many cases, genomic variants do not regulate their physically adjacent genes but genes far away and even in other chromosomes (*trans*-regulation). Thus, we used transcriptome data to further identify genes (eGenes) regulated by these 2 genomic regions. After quality control, the clean data of 668 liver samples varied between 5.24 and 10.51 G for each individual, and the expression of 12,191 genes was quantified in total, of which 10,171 genes were annotated successfully. After eQTL mapping by tensorQTL, 7,468 genes (217 *cis*- and 7,431 *trans*-regulated genes) were screened for their regulation by genomic variants (Supplementary Fig. S3). We then applied the summary data-based Mendelian randomization (SMR) method to genome-wide analysis (GWAS) summary datasets of HSC, and the SMR test showed that 2 genes (*NUDT14* and *EIF5A2*) were significantly associated with the genomic peak in GGA4 and 3 genes (*PEMT, TOM1L2*, and *GSTM3*) with their genomic peak in GGA6 (*P*_adj_ < 0.05, false discovery rate (FDR) correction for SMR, Supplementary Table S3). For verification, using the colocalization strategy, we performed thousands of fastGWA runs for all hepatic genes, and 4,171 genes (eGenes) were finally identified for expression that was significantly regulated by at least 1 genomic variant (eVariant, *P* value of the top SNPs <3.32 × 10^−7^). Among these genes, 2 protein-coding genes, *PEMT* (4,792,705–4,830,291 bp) and *TOM1L2* (4,914,681–4,925,733 bp), at GGA 14 were identified again for their significant association with the genomic region on GGA6, 5.59–5.69 Mb, harboring the same variant rs731375960 that was significantly associated with HSC (Fig. 2B, *P* < 3.32 × 10^−7^). However, no significantly genes were identified for their association with the genomic region of GGA4, 75.6–76.4 Mb. Furthermore, among the genotypes of the top variant rs731375960, only expression of the *PEMT* gene differed significantly (post hoc Wilcoxon rank-sum test, β = −371.91 for genotype GG to AA, Fig. 2D).

#### Crucial genes for hepatic steatosis

To ascertain the relationship between gene expression and hepatic steatosis, Spearman’s rank-based correlation (SRC) analysis was performed between the expression of each gene and hepatic steatosis classifications. We identified 98 and 119 genes that were significantly positively and negatively correlated with hepatic steatosis, respectively, after FDR correction (*P*_adj_ < 0.05, FDR correction, Supplementary Table S4), including *trans*-eGenes *PEMT* and *TOM1L2*. Considering that correlation analysis employs linear models, analysis of variance (ANOVA) was also performed among 3 hepatic steatosis groups. Then, 98 significantly differentially expressed genes were screened (*P*_adj_ < 0.05, FDR correction), 83 of which (84.7%) were consistent with the genes identified by SRC (Fig. 2E). Gene Ontology (GO) and pathway enrichment analyses indicated that these genes were mostly involved in the biological process of lipid localization and the biosynthetic process (Supplementary Fig. S4 and Table S5). Among 232 candidate genes derived from the union of SRC and ANOVA results, 48 were reported to be involved in hepatic steatosis, NAFLD, or hepatic lipid metabolism in humans and rats, indicating the reliability of our results (Table 1 and Supplementary Table S6), 15 of which were part of the 83 genes consistently identified by both Spearman’s rank correlation analysis and ANOVA. We further identified genomic variants (eVariants) that regulated the expression of these 48 genes in fastGWA datasets and found that 20 genes were significantly regulated by at least 1 eVariant, all of which were *trans*-regulated (Table 1). In eHSC groups, 8,632 annotated genes were identified for their significantly differential expression (*P*_adj_ < 0.05, FDR correction, Supplementary Table S7).

**Table 1:** Description of 18 *trans*-eGenes related to hepatic steatosis

Gene symbol	High presented in	Gene location	eVariants region	No. of significant SNPs	Top SNP (location)	Demonstration of the gene function in the references
*NAPEPLD*	Con	GGA1 13,065,354–13,085,319 bp	GGA10 10.2–17.9 Mb	19,044	rs313818251 (10:14,621,940)	Loss of *NAPEPLD* result in fat mass gain and hepatic steatosis in mouse [39]
*WNT5A*	HS	GGA12 8,303,804–8,315,223 bp	GGA5 30.1–36.4 Mb	8,259	- (5:34,210,278)	*WNT5A* may promote liver damage in human [85]
*IL15*	HS	GGA4 29,989,429–30,022,148 bp	GGA2 89.3–100.9 Mp	8,244	- (2:99,506,822)	Absence of *IL-15* or *IL-15Rα* protects from NAFL in mouse [86]
*ACOT13*	HS	GGA2 90,236,164–90,240,896 bp	GGA1 145.1–150.8 Mp	6,020	rs316486650 (1:148,449,556)	Regulate hepatic lipid and glucose metabolism in mouse [87]
*JAG1*	Con	GGA3 13,599,642–13,633,726 bp	GGA21 5.04–6.03 Mp	4428	rs313406743 (21:5,121,276)	Hepatocyte-specific *JAG1* knockout mice were protected from NASH-induced liver fibrosis [88]
*ISM1*	Con	GGA3 12,825,298–12,864,186 bp	GGA21 4.07–4.65 Mb	700	rs315660086 (21:4,319,591)	*ISM1* suppresses hepatocyte lipid synthesis in mouse [89]
*ECHDC1*	HS	GGA3 59,266,598–59,303,456 bp	GGA5 17.8–18.0 Mb	11	rs316727537 (5:17,943,618)	Involved in the occurrence and development of NAFLD by regulating hepatic lipid metabolism in human [90]
*ETV5*	Con	GGA9 5,289,877–5,299,258 bp	GGA4 61.15–61.17 Mb	4	rs315563554 (4:61,152,625)	Regulate hepatic fatty acid metabolism in mouse [91]
*GSTA3*	Con	GGA3 88,388,999–88,395,707 bp	GGA2 34.0–34.5 Mb	4	rs314345391 (2:34,475,071)	Play vital roles in hepatic iron metabolism and may be associated with NALFD in mouse [92]
*IFRD1*	Con	GGA1 27,057,994–27,068,635 bp	GGA11 11.0–11.7 Mb	13	- (11:11,142,255)	A regulator of lipid absorption and metabolism in mouse [93]
*SLITRK3*	Con	GGA9 21,273,430–21,276,090 bp	GGA4 79.7–80.0 Mb	38	- (4:79,863,187)	*SLITRK3* was downregulated in steatosis and NASH patients in human [94]
*PTPN2*	Con	GGA2 97,013,090–97,047,494 bp	GGA17 3.47–4.08 Mb	16	- (17:3,671,818)	Liver-specific *PTPN2* deficiency promotes hepatic steatosis, obesity, and insulin resistance in human and mouse [4, 95]
*UCHL1*	Con	GGA4 68,642,734–68,647,699 bp	GGA2 139.8–140.2 MB	15	rs312268369 (2:140,103,337)	*UCHL1* appears to be a functional tumor suppressor involved in the tumorigenesis of hepatocellular carcinoma in human [96]
*SLC16A10*	Con	GGA3 66,217,809–66,280,609 bp	GGA3 10,876,753–10,876,785 bp	3	rs739986067 (3:10,876,769)	*SLC16A10* is a transport carrier of aromatic amino acids, upregulated in NAFLD group in rats [97]
*DIO2*	Con	GGA5 40,752,235–40,769,122 bp	GGA3 8.69–8.86 Mb	2	rs315951384 (3:8,693,350)	Loss of the *DIO2* gene results in increased fat storage in adipose tissue and hepatic steatosis in mouse [98]
*MT3*	Con	GGA11 2,122,187–2,123,328 bp	GGA8 15,963,283 bp	1	rs314635449 (8:15,963,283)	*MT3* may be a potential intervention for hepatic steatosis by inhibit the generation of reactive oxygen species in human [99]
*RBP*	Con	GGA8 21,595,383–21,606,407 bp	GGA1 55,817,413 bp	1	- (3:55,817,413)	Lower expression of *RBP* may improve hepatic steatosis in mouse [100, 101]

*ACOT13*: acyl-CoA thioesterase 13; *DIO2*: deiodinase 2; *ECHDC1*: ethylmalonyl-CoA decarboxylase 1; *ETV5*: ETS variant transcription factor 7; *GSTA3*: glutathione S-transferase alpha 3; *IFRD1*: interferon-related developmental regulator 1; *IL15*: interleukin 15; *ISM1*: isthmin 1; *JAG1*: Jagged canonical Notch ligand 1; *MT3*: metallothionein 3; *NAPEPLD*: N-acyl phosphatidylethanolamine phospholipase D; *PTPN2*: protein tyrosine phosphatase nonreceptor type 2; *RBP*: retinol binding protein 1; *SLC16A10*: solute carrier family 16 member 10; *SLITRK3*: SLIT and NTRK like family member 3; *UCHL1*: ubiquitin C-terminal hydrolase L1; *WNT5A*: Wnt family member 5A.

### Multiple omics data reveal the molecular regulation mechanism for hepatic steatosis

**Figure 3:**
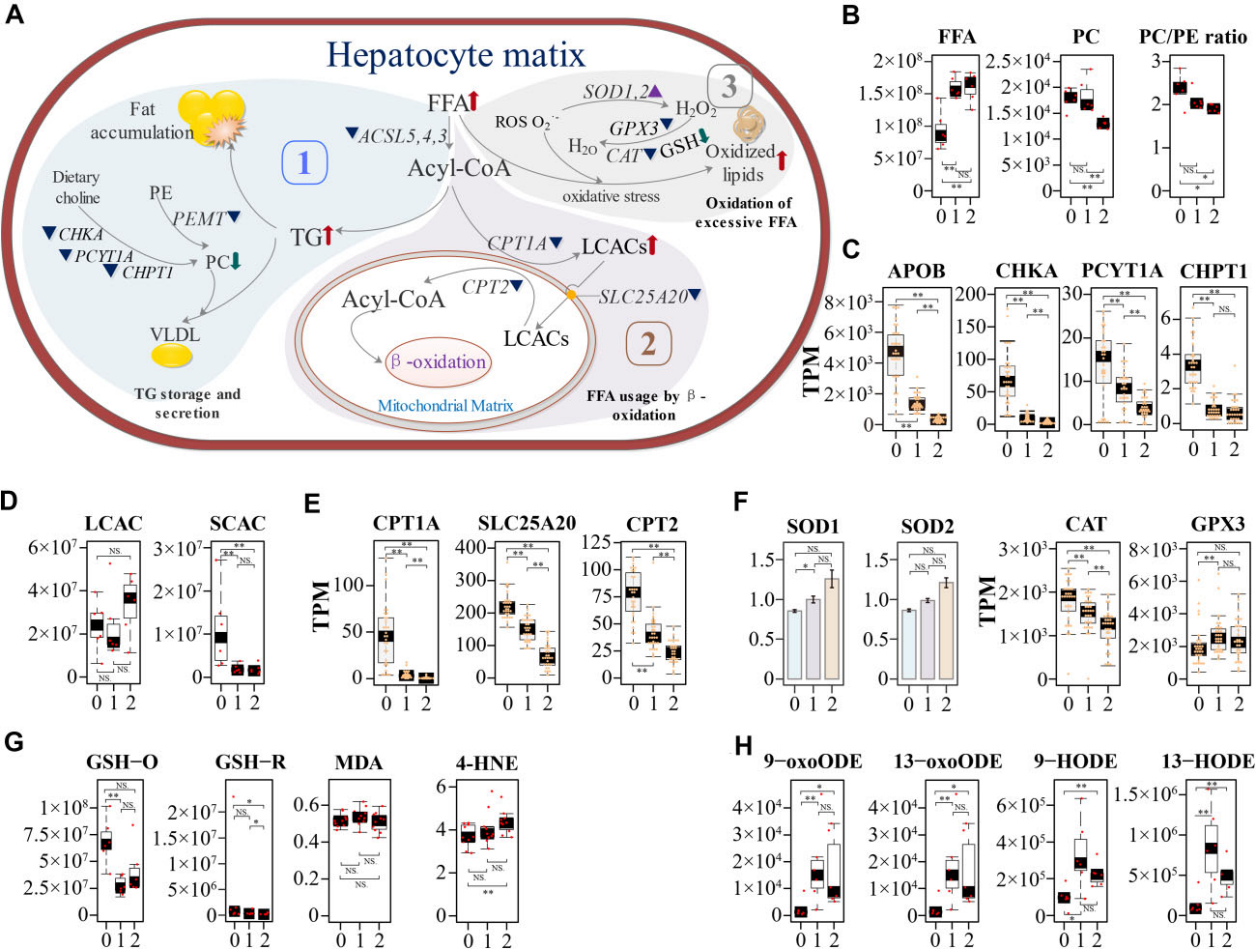
Multiomics data reveal the molecular regulation mechanism of hepatic steatosis. (A) Illustration summarizing 3 regulation routes to hepatic steatosis. To better demonstrate the results, not all enzymes and metabolites in the pathway are shown. (1) The severe accumulation of hepatic free fatty acids (HFFAs) and hepatic triglyceride (HTG), as well as the decrease of phosphatidylcholine (PC) partially caused by the low expression of phosphatidylethanolamine N-methyltransferase (*PEMT*), impeded the outward transport of TG from hepatocytes; Acyl-CoA synthetase long-chain family member 5, 4, 3: *ACSL5,4,3*, choline kinase alpha: *CHKA*, phosphate cytidylyltransferase 1A: *PCYT1A*, choline phosphotransferase 1: *CHPT1*. (2) Use of HFFA was inefficient because of the weak fatty acid β-oxidation activity; Carnitine palmitoyltransferase 1A, 2: *CPT1A* and *CPT2*, solute carrier family 25 member 20: *SLC25A20*. (3) The peroxidation of excessive HFFA accelerated the progression of hepatic steatosis; superoxide dismutase 1, 2: SOD1 and SOD2, glutathione peroxidase 3: *GPX3*, catalase: *CAT*. (B) HFFAs increased significantly with the severity of hepatic steatosis (*P*_adj_ < 0.05, post hoc Wilcoxon rank-sum test). The opposite change pattern applied to PC and the PC/phosphatidyl-ethnolamine (PE) ratio (*P*_adj_ < 0.05, post hoc Wilcoxon rank-sum test). (C) Expression of apolipoprotein B (*APOB*), *CHKA, PCYT1A*, and *CHPT1* in the liver shared the same change pattern, decreasing significantly with hepatic steatosis in the eHSC group (*P*_adj_ < 0.05, post hoc Wilcoxon rank-sum test). (D) The most severe hepatic steatosis group possessed the highest long-chain acylcarnitines (LCACs) but the least short-chain acylcarnitines (SCACs) (*P*_adj_ < 0.05, post hoc Wilcoxon rank-sum test). (E) Expression of *CPT1A, SLC25A20*, and *CPT2* in the liver was significantly negatively correlated to hepatic steatosis in the eHSC group (*P*_adj_ < 0.05, post hoc Wilcoxon rank-sum test). (F) Superoxide dismutase 1/2 (SOD1/2) were significantly upregulated in steatosis groups (*P*_adj_ < 0.05, post hoc Wilcoxon rank-sum test), *n* = 4, while GPX3 was more expressed in steatosis groups (*P*_adj_ < 0.05, post hoc Wilcoxon rank-sum test). (G) Oxidized and reduced glutathione (GSH-O and GSH-R, respectively) were also extremely low but 4-hydroxynonenal (HNE) was highly presented in steatotic livers (*P*_adj_ < 0.05, post hoc Wilcoxon rank-sum test). (H) 9- and 13-hydroxy-octadecadienoic acid (9- and 13-HODE) and 9- and 13-oxo-octadecadienoic acid (9- and 13-oxo ODE) were higher in the steatosis group than in the control group (*P*_adj_ < 0.05, post hoc Wilcoxon rank-sum test). For B–H, 0 refers to the eCtrl group, 1 for eHS-I, and 2 for eHS-II. Data of genes, metabolites, and proteins are from *n* = 30, 6, and 4 biological replicates, respectively. *P < 0.05, **P < 0.01.

To further ascertain the genetic mechanisms of hepatic steatosis, we complemented our molecular phenome coverage by profiling hepatic proteome and metabolome of individuals from eHSC groups (7 samples/group) and quantified 4,961 proteins and 1,005 metabolites in total (Supplementary Tables S8 and S9). On the basis of the integrative analysis from genome to metabolome data, we summarized the regulation routes from 3 aspects (Fig. 3A).

#### Inability of VLDL to effectively transport TG outward

Hepatic steatosis was characterized by the severe accumulation of triglyceride (TG). Our analysis uncovered that HTG increased significantly with the severity of hepatic steatosis (*P*_adj_ < 0.05, β = 0.32, post hoc Wilcoxon rank-sum test, Fig. 1B). In general, the generated TGs are transported extra-hepatically via VLDLs together with cholesterol and apolipoproteins, and impaired hepatic phosphatidylcholine (PC) biosynthesis can significantly reduce VLDL synthesis and secretion [35]. Our results showed the PC, phosphatidyl-ethanolamine (PE), and the PC/PE ratio significantly decreased with the severity of hepatic steatosis (*P*_adj_ < 0.05, β_PC_ = 5,282.95, β_PC/PE_ = 0.47, post hoc Wilcoxon rank-sum test, Fig. 3B). Endogenous PC is synthesized from PE by the *PEMT* gene, which was downregulated in hepatic steatosis groups and shared the same regulatory genomic variants with hepatic steatosis classification in our GWA analysis (Fig. 2B). Furthermore, PC can also be generated from dietary choline, which involves 3 genes, choline kinase (*CHKA*), phosphate cytidylyltransferase 1 (*PCYT1A*), and choline phosphotransferase 1 (*CHPT1*). The expression of these genes was significantly higher in the control than the steatosis group (3- to 5-fold change in eHSC, *P*_adj_ < 0.05, β*_CHKA_* = 63.84, β*_PCYT1A_* = 11.07, β*_CHPT1_* = 2.57, post hoc Wilcoxon rank-sum test, Fig. 3C), leading to a significant decrease of PC production in the steatosis group. Correspondingly, the *APOB* gene, which encodes the primary apolipoprotein for VLDL synthesis, was also dramatically decreased along with hepatic steatosis (13.4 times higher in the eCtrl group than eHS-II group, *P*_adj_ < 0.05, β = 4,483.77, post hoc Wilcoxon rank-sum test, Fig. 3C). Hence, we proposed that the downregulation of *PEMT, CHKA, PCYT1A*, and *CHPT1* collectively led to the reduction of PC levels, consequently affecting the synthesis of VLDL. This impaired VLDL production hindered TG outward transport, resulting in its excessive accumulation and the progression of hepatic steatosis (Fig. 3A, route 1).

#### Weak activity of β-oxidation inhibits fatty acid use

Notably, another characteristic of hepatic steatosis is the accumulation of free fatty acids (FFAs). Our metabolic profiles revealed that the quantity of HFFAs increased significantly with the severity of hepatic steatosis (*P*_adj_ < 0.05, β = −74,085,990, post hoc Wilcoxon rank-sum test, Fig. 3B), which was verified by an enzyme-linked immunosorbent assay (ELISA) (Fig. 1C). Besides being utilized in the synthesis of TGs, FFAs primarily serve as a source of energy supply through β-oxidation activity. Long-chain fatty acids (LCFAs) are first catalyzed by acyl-CoA synthetases (*ACSL5,4,3*) to form acyl-CoA, which are further synthesized to long-chain acylcarnitines (LCACs) by carnitine palmitoyltransferase 1A (*CPT1A*), a rate-limiting enzyme for β-oxidation. Our results showed the expression of *ACSL5,4,3* genes significantly decreased with the severity of hepatic steatosis, and the rate-limiting enzyme *CPT1A* was almost not expressed in steatotic livers (*P*_adj_ < 0.05, β = 45.17, post hoc Wilcoxon rank-sum test, 67 times higher in eCtrl than eHS-Ⅱ). Correspondingly, the metabolome data revealed a significantly high presence of LCACs in steatotic livers (*P*_adj_ < 0.05, β = −8,329,448, post hoc Wilcoxon rank-sum test, Fig. 3D), while short-chain acylcarnitines (SCACs) showed the opposite trend (*P*_adj_ < 0.05, β = 6,842,755, post hoc Wilcoxon rank-sum test, Fig. 3D). Additionally, the other 2 crucial β-oxidation–related genes showed similar expression patterns, including *SLC25A20* (solute carrier family 25 member 20), which is responsible for the transport of LCACs from cytosol to the mitochondrial matrix (*P*_adj_ < 0.05, β = 150.60, post hoc Wilcoxon rank-sum test, Fig. 3E), and *CPT2* (carnitine palmitoyltransferase 2), which catalyzes the opposite reaction with *CPT1A* from LCACs to acyl-CoA (*P*_adj_ < 0.05, β = 53.34, post hoc Wilcoxon rank-sum test, Fig. 3E). Therefore, the severely high LCACs and extremely low expression of *CPT1A, SLC25A20*, and *CPT2* indicated the suspension of β-oxidation activity in the HS group, leading to the accumulation of FFAs (Fig. 3A, route 2).

#### Oxidative stress of FFA accompanied by steatosis

When the synthesis of TGs from FFA and β-oxidation of FFA is blocked (Fig. 3A routes 1 and 2), we found excessive accumulated FFA might undergo the oxidation process, further aggravating the progression of hepatic steatosis. Specifically, the gene of superoxide dismutase 1/2 (SOD1/2) was highly expressed in the steatosis groups (*P* < 0.05, Fig. 3F); this gene can convert superoxide (O_2_^−^) to hydrogen peroxide (H_2_O_2_) and oxygen (O_2_). In addition, the catalase (*CAT*) gene responsible for conversion of H_2_O_2_ to H_2_O and O_2_, and the glutathione peroxidase 3 (*GPX3*) gene responsible for catalyzing the reduction of organic hydroperoxides and H_2_O_2_ by glutathione, were both significantly downregulated in steatotic livers (*P*_adj_ < 0.05, β*_CAT_* = 654.79, β*_GPX3_* = −383.55, post hoc Wilcoxon rank-sum test, Fig. 3F). Correspondingly, glutathione (GSH), as the most important antioxidant, was also extremely low in steatotic livers (*P*_adj_ < 0.05, β_GSH-O_ = 33,619,000, β_GSH-R_ = 441,270, post hoc Wilcoxon rank-sum test, Fig. 3G). Subsequently, we quantified the end products of lipid peroxidation, namely, malondialdehyde (MDA) and 4-hydroxynonenal (HNE), using ELISAs, and found their significantly higher presence in steatotic livers (*P*_adj_ < 0.05, β_MDA_ = 0.01, β_4-HNE_ = −0.64, post hoc Wilcoxon rank-sum test, Fig. 3G), verifying the high activity of lipid peroxidation. The metabolic data also revealed that oxidized lipids, such as 9- and 13-hydroxy-octadecadienoic acid (9-HODE, 13-HODE) and 9- and 13-oxo-octadecadienoic acid (9-oxoODE, 13-oxoODE) converted from linoleic acid, were 5 to 7 times higher in the steatosis group than in the control (*P*_adj_ < 0.05, β_9-HODE_ = −12,735.45, β_13-HODE_ = −389,758, β_9-oxoODE_ = −66,776.85, β_13-oxoHODE_ = −66,776.85, post hoc Wilcoxon rank-sum test, Fig. 3H), further illustrating the state of oxidative stress on fatty acids (FAs). The simultaneous upregulation of peroxidases *CAT* and *GPX3* and the downregulation of antioxidant enzymes GSH, accompanied by elevated levels of MDA, HNE, HODE, and oxoODE, jointly indicate a close relationship between the peroxidation of FFA and hepatic steatosis (Fig. 3A, route 3).

### Microbiome signatures of hepatic steatosis and their association with host genetics

Fat metabolism has long been thought to be regulated by both genetics and gut microbiota. Hence, 16S rRNA gene sequencing was performed in the duodenum, jejunum, ileum, cecum, and feces of 705 chickens, resulting in 174.2 million quality-filtered sequences from 3,430 samples with an average of 49,497 reads (Supplementary Table S10). Then, 6,087 (duodenum), 5,987 (jejunum), 3,751 (ileum), 3,215 (cecum), and 7,428 (faces) amplicon sequence variants (ASVs) were identified with 100% sequence identity in each gut segment. The α diversities exhibited significant differences among the 5 sampling sites, with the cecum displaying the highest diversity and the ileum the lowest. Subsequently, principal coordinates analysis (PCoA) was employed to visualize variations in microbial composition across these diverse sites. A distinct divergence in the gut microbial community was observed among the intestines, clustering separately. At the phylum level, the 3 segments of the small intestine (SI) harbored similar dominant microbial communities, with Firmicutes being the predominant phylum, followed by Proteobacteria, Bacteroidetes, and Actinobacteria. However, noticeable differences were evident in the cecum, where Bacteroidetes (53.91%) and Firmicutes (36.83%) constituted the most abundant phyla, respectively. At the genus level, *Lactobacillus* represented the majority of genera in the duodenum (46.06%), jejunum (54.25%), and feces (22.02%), while *Romboutsia* exhibited the highest abundance (30.32%) in the ileum, and *Bacteroides* constituted a notable fraction (30.04%) in the cecum.

#### Microbiability estimation

**Figure 4:**
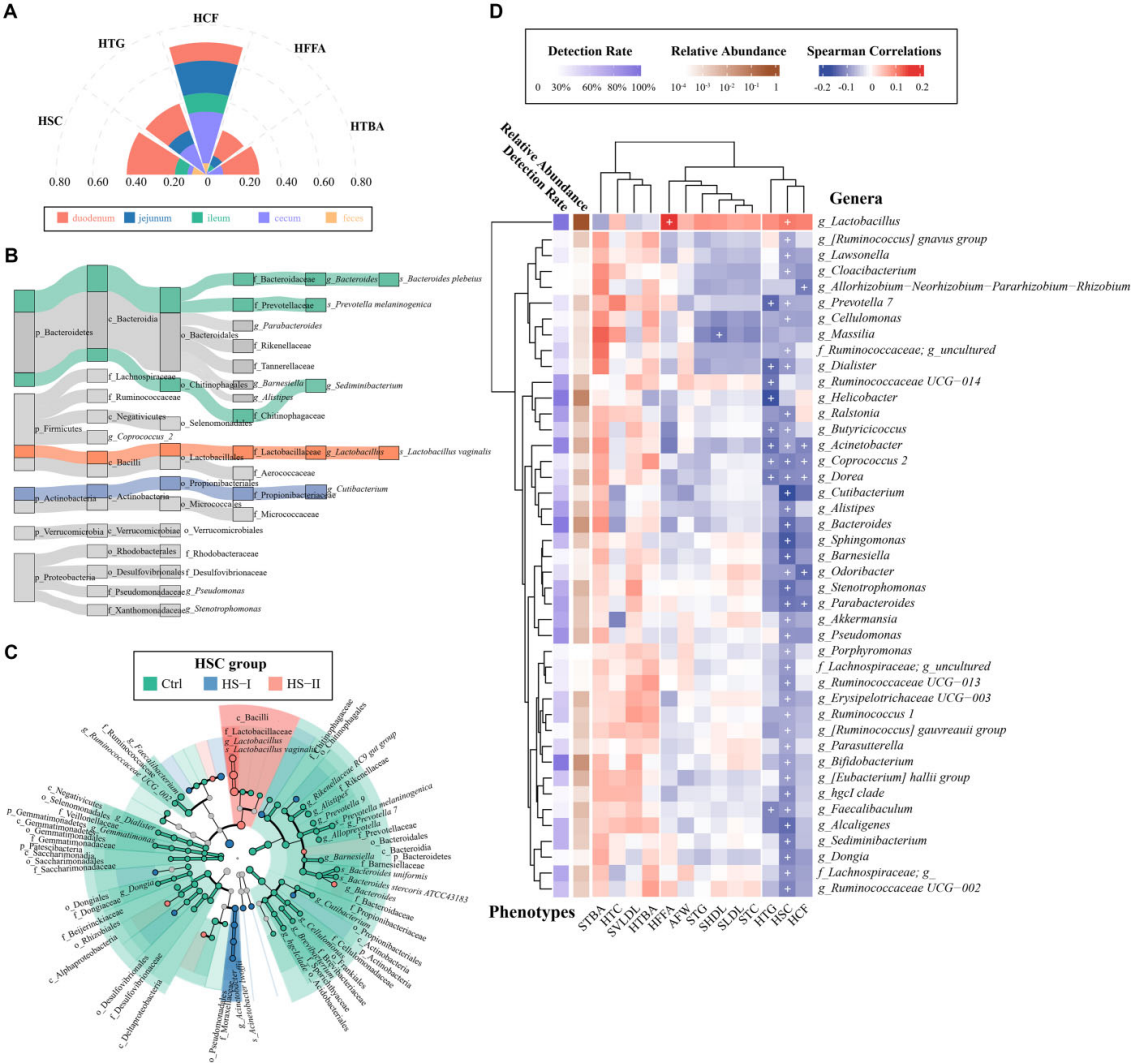
Contribution of the gut microbial community to fat deposition–related traits and their correlation with duodenal microbiota. (A) Microbiability of duodenal, jejunal, ileal, cecal, and fecal microbiota for hepatic steatosis classification (HSC), hepatic triglyceride (HTG), hepatic crude fat (HCF), hepatic free fatty acids (HFFAs), and hepatic total bile acids (HTBAs). (B) Four taxa chains from the phylum to genus level were generated on the basis of the taxa associated significantly with HSC by Spearman’s rank-based correlation (SRC) analysis. *n* = 673. (C) Linear discriminant analysis effect size (LEfSe) analysis identified differential taxa on the basis of their significantly differential presence among HSC groups (linear discriminant analysis >2). *n* = 673. (D) Heatmap of the association of genus abundance with all recorded phenotypes (SRC analysis, + indicates statistical significance *P*_adj_ < 0.05, FDR correction). HTC: hepatic total cholesterol; SHDL: serum high-density lipoprotein; SLDL: low-density lipoprotein; STBA: serum total bile acids; STC: serum total cholesterol; STG: serum triglyceride; SVLDL: very low-density lipoprotein. *n* ≥ 673. (E) The amount and cumulative relative abundance of duodenal taxa with different detection rates from phylum to species. *n* = 686.

Analogous to heritability, the relative proportion of the total variance due to the gut microbial community is defined as microbiability (m^2^), which allows estimation of the effect of microbiota as a whole on host traits. Hence, we first employed m^2^ with ASV data to dissect the contributions of microbiota in each gut segment to hepatic steatosis–related phenotypes. The m^2^ of HSC in the duodenum (0.26) was much higher than that in other anatomical sites (0.03 for jejunum, 0.08 for ileum, 0.02 for cecum, and 0.07 for feces), suggesting more important roles of duodenal microbes in the progression of hepatic steatosis (Supplementary Table S11 and Fig. 4A). Similar results were observed for HFFA and HTBA contents in the liver (0.15 and 0.19 for the duodenum, respectively, and almost zero in other segments). However, for HCF and HTG contents, cecal microbiota (m^2^ of 0.27 and 0.20 in cecum) played a more critical role than that in other segments (Fig. 4A). To validate the reliability of the estimated m^2^, a permutation test was performed for the relatively high m^2^ by randomly reordering the phenotypes 1,000 times. The results showed that the actual m^2^ (0.15∼0.27) was significantly higher than the simulated m^2^ (average m^2^ ranged from 0.02 to 0.04, *P* < 0.05, Supplementary Fig. S5A).

#### Identification of crucial microbes for hepatic steatosis

Using high-quality ASVs, 52 phyla, 161 classes, 467 orders, 1,003 families, 2,329 genera, and 3,930 species were successfully classified (Supplementary Fig. S5B). The Shannon and Simpson indices for α diversity revealed that the microbiota diversity decreased with steatosis progression (*P*_adj_ < 0.05, β_Shannon_ = 0.37, β_Simpson_ = 0.03, post hoc Wilcoxon rank-sum test, Supplementary Fig. S5C). To evaluate the association between gut microbiota and hepatic steatosis, SRC analysis was performed between the abundance of microbial taxa in each gut segment and HSC. Based on the significantly greater microbiability estimates in the duodenum, we focused on the duodenal microbiota, and significantly associated taxa (72 in total) with HSC were almost observed in the duodenum, including 6 phyla, 6 classes, 9 orders, 16 families, 14 genera, and 21 species (*P*_adj_ < 0.05, FDR correction, Supplementary Table S12). For other gut segments, only 2 taxa, the family Staphylococcaceae and genus *Staphylococcus* in the ileum, were significantly positively associated with HSC (*P*_adj_ < 0.05, FDR correction). Among these 72 taxa, 4 taxa chains from the phyla to genus level were identified for their association with HSC, including chains harboring the genera *Lactobacillus, Bacteroides, Sediminibacterium*, and *Cutibacterium* (Fig. 4B). Additionally, genera *Lactobacillus* and *Bacteroides* were verified by linear discriminant analysis effect size (LEfSe) analysis for their significantly differential presence among HSC groups (linear discriminant analysis [LDA] = 4.7 and 3.8, respectively, *P*_adj_ < 0.01, Kruskal–Wallis test, Fig. 4C). Genus *Lactobacillus* was positively correlated with HSC (41.1% in Ctrl, 47.5% and 52.0% in HS-Ⅰ and HS-Ⅱ, *P*_adj_ < 0.05), while genus *Bacteroides* showed the opposite trend (2.09% in Ctrl, 1.70% and 1.55% in HS-Ⅰ and HS-Ⅱ, *P*_adj_ < 0.05). Additionally, LEfSe analysis verified the differential presence of many other taxa (LDA > 3.0, *P*_adj_ < 0.01, Kruskal–Wallis test), including phyla Bacteroidetes, Firmicutes, and Actinobacteria; classes Bacilli, Actinobacteria, and Bacteroidia; orders Bacteroidales and Chitinophagales; families Lactobacillaceae, Ruminococcaceae, Bacteroidaceae, Prevotellaceae, and Chitinophagaceae; genera *Lactobacillus* and *Bacteroides*; and species *Lactobacillus vaginalis*. The abovementioned microbial taxa had a high detection rate, many of which exhibited a high correlation with the phenotypes (Fig. 4D and Supplementary Fig. S6). Except for HSC, many microbes were negatively correlated with HTG and HCF levels, including genera *Prevotella7, Dialister, Ruminococcaceae UCG-014, Helicobacter, Butyricicoccus, Acinetobacter, Coprococcus 2, Dorea, Odoribacter, Parabacteroides*, and *Faecalibaculum* (Fig. 4D, for other taxa levels, see Supplementary Fig. S6).

#### *Lactobacillus* might play compensatory roles in alleviating hepatic steatosis

**Figure 5:**
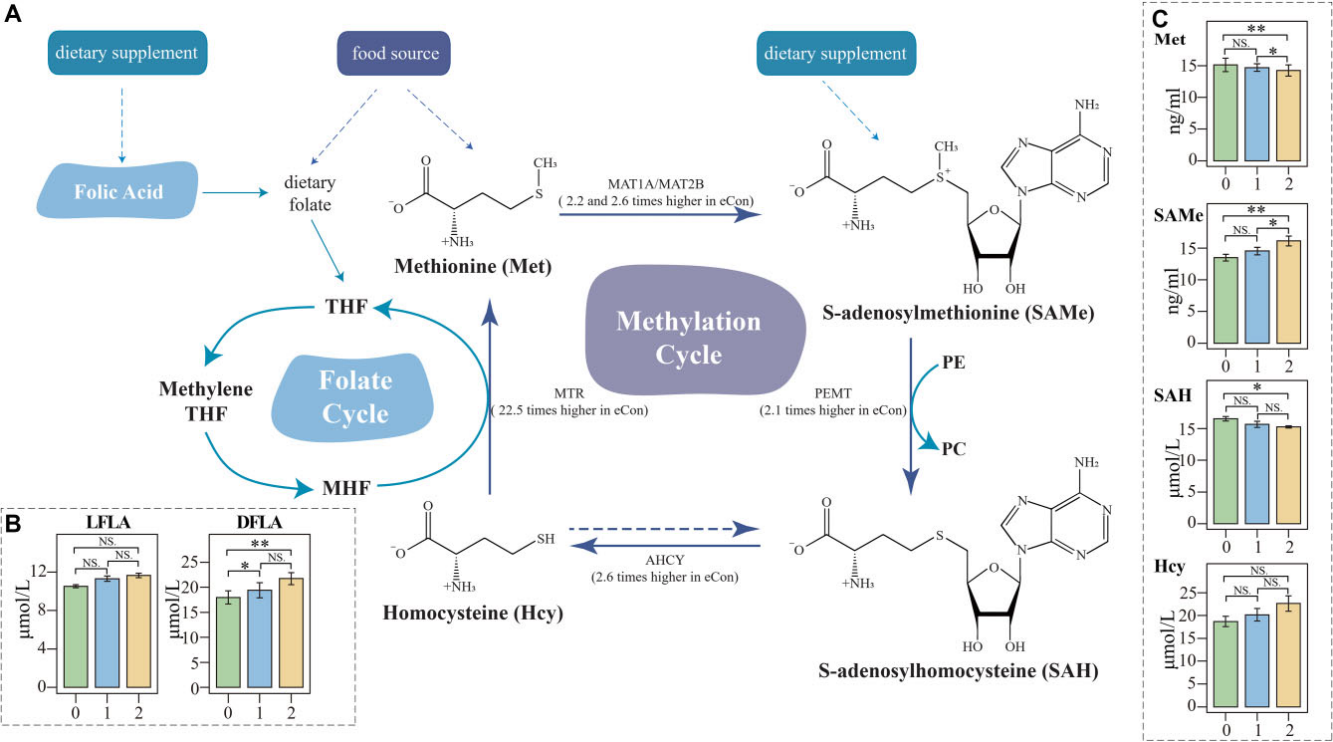
Chicken hepatic methionine cycle, folate cycle, and associated genes and metabolites. To better demonstrate the results, not all enzymes and metabolites in the pathway are shown. (A) Methionine cycle coupled with folate cycle. The scheme shows the main reactions and correlated genes involved in the methionine and folate cycle. Two main methionine adenosyltransferase isoforms (*MAT1A* and *MAT2B*), were respectively 2.2 and 2.6 times higher in the eCtrl group. Phosphatidylethanolamine N-methyltransferase (*PEMT*), S-adenosylhomocysteine hydrolase (*AHCY*), and methionine synthase (*MTR*) were significantly upregulated in the eCtrl group with 2.1, 2.6, and 22.5 times higher expression, respectively. (B) Comparison of the folic acid quantity in the liver (LFLA) and duodenal chyme (DFLA). 0 refers to the eCtrl group, 1 for eHS-I, and 2 for eHS-II. (C) Contents of methionine, S-adenosylmethionine (SAMe), S-adenosylhomocysteine (SAH), and homocysteine (Hcy) in the liver determined by ELISA assay. 0 refers to the eCtrl group, 1 for eHS-I, and 2 for eHS-II. Data are from *n* = 30 (A) and 10 (B and C) biological replicates, respectively. *P < 0.05, **P < 0.01.

By analyzing metabolome/transcriptome data from duodenum and liver samples, a significant enrichment of metabolites and genes associated with the methionine cycle was observed between the healthy and hepatic steatosis groups (Fig. 5A), suggesting a potential link between the abundance of *Lactobacillus* in the duodenum of individuals with hepatic steatosis and their involvement in the host methionine cycle. Notably, we observed a 1.2-fold increase in folic acid production by the duodenal microbiota in the eHS-II group compared to the eCtrl group (*P*_adj_ < 0.05, β = −1.08, post hoc Wilcoxon rank-sum test), as validated by an ELISA (Fig. 5B). Examining methionine cycle-related genes, including methionine adenosyltransferase (MAT), phosphatidyl-ethanolamine N-methyltransferase (PEMT), adenosylhomocysteinase (AHCY), and 5-methyltetrahydrofolate-homocysteine methyltransferase (MTR), revealed their significantly lower expression levels in individuals with hepatic steatosis. The eCtrl group exhibited 2.1–22.5 times higher expression than the eHS-II group. Furthermore, ELISA results unveiled a significant accumulation of S-adenosylmethionine (SAMe) and homocysteine (Hcy) in the liver samples of the eHS-II group compared to the eCtrl group (Fig. 5C, *P*_adj_ < 0.05, β_SAMe_ = −1.61, β_Hcy_ = 1.32, post hoc Wilcoxon rank-sum test). SAMe is converted to S-adenosyl homocysteine (SAH) by specific methyltransferases (MTs) such as glycine N-methyltransferase (GNMT), guanidinoacetate methyltransferase (GAMT), and PEMT. However, only the *PEMT* gene was expressed in the liver in our study, as demonstrated in Fig. 2. Interestingly, PEMT also catalyzes conversion of PE to PC, and its low expression led to accumulation of SAMe and PE. Hcy can be remethylated to regenerate methionine by *MTR* (22.5 times higher in eCtrl than eHS-II) with conversion of methyltetrahydrofolate (MTHF) to tetrahydrofolate (THF). Hence, additional folic acid is essential to prompt the final step of the methionine cycle. Although these results should be interpreted carefully, it can be hypothesized that the extra folate produced by duodenal microbiota may be absorbed by the host to reduce Hcy accumulation, resulting in comparable hepatic folic acid contents in healthy and hepatic steatosis groups (Fig. 5B).

## Discussion

In this study, we used a chicken population consisting of 705 well-phenotyped individuals to investigate genetic variants, crucial hepatic molecules, and their interactions with intestinal microbiota in the development of hepatic steatosis. Hepatic steatosis is a quantitative trait, making artificial hepatic steatosis classification imprecise for intermediate individuals between each of 2 adjacent HSC groups. Hence, in addition to the HSC model, we further established the eHSC model, which used more stringent criteria for hepatic steatosis classification with smaller variance in each eHSC group.

Many studies have estimated the heritability of hepatic steatosis or NAFLD in humans with the values ranging from 0.20 to 0.70 [5, 36, 37], which is comparable to our results (0.25). Hepatic steatosis is generally characterized by accumulation of lipid droplets [38], which is supported by the increased HTG and HCF along with hepatic steatosis in our study. However, our genetic analysis revealed that hepatic steatosis and fat accumulation did not share the same genetic determinants. We proposed that fat accumulation should be viewed as a symptom of hepatic steatosis rather than as its actual cause. With SRC and ANOVA analysis, 227 candidate genes were screened for their association with hepatic steatosis, 48 of which have been reported to be involved in NAFLD or hepatic lipid metabolism in humans and mice. For example, *NAPEPLD* in hepatocytes is an important regulator of liver bioactive lipid synthesis. The hepatocyte-specific *Napepld* deletion mouse develops a high-fat diet-like phenotype characterized by increased fat mass gain and hepatic steatosis [39]. *CYP1A1* metabolizes benzo[a]pyrene, resulting in either detoxication or metabolic activation in a context-dependent manner. Loss of the *CYP1A1* gene protects against nonalcoholic fatty liver disease caused by a Western diet containing benzo[a]pyrene in mice [40]. Interestingly, in contrast to previous studies of humans and cattle [41, 42], in which the majority of genes were *cis*-regulated by eVariants in most tissues, in this study, most genes expressed in the liver, including candidate genes for hepatic steatosis, were *trans*-regulated by genomic variants; that is, genes and their corresponding eVariants were on different chromosomes. This regulatory pattern was identified by both tensorQTL [43] and fastGWA [44] methods, indicating a unique gene regulation pattern in chickens, which requires additional study to determine whether this is the case in other species or tissues.

Combined with multiomics data and the validation of crucial metabolites, we found abnormalities in 3 regulatory routes in host hepatocytes that contribute to hepatic steatosis. First, low expression of the *PEMT* gene led to a decrease in PC production, which further reduced VLDL synthesis and secretion. Previous studies have demonstrated beneficial roles of PC in protecting against hepatic steatosis [45] and observed the clinical feature of a low PC/PE ratio in livers of patients with NAFLD [46]. In this study, we found not only a decrease in endogenous PC production due to the *PEMT* gene but also a dramatic decrease in the synthesis of exogenous PC from dietary choline, which further aggravated outward translocation of liver fat. Second, β-oxidation activity was very weak in mitochondria of HS individuals. A previous study has also demonstrated that lipid accumulation in the liver can be traced by impaired fatty acid β-oxidation [47]. Here, we found that the main genes in the β-oxidation pathway, including *ACSL5,4,3, CPT1A, SLC25A20*, and *CPT2*, were all expressed at extremely low levels in the HS-II group compared with the Ctrl group. This resulted in significantly elevated and decreased levels of LCACs and SCACs, respectively, in the HS-II group, which may serve as biomarkers for hepatic steatosis.

To the best of our knowledge, no study has reported the m^2^ of hepatic steatosis in humans or any animal model. Our study proposes that the crucial regulatory microbiota for hepatic steatosis mainly exist in the duodenum because of its much higher m^2^ of HSC (0.26) than other gut segments (near zero). Indeed, the anterior part of the small intestines is the main site for fat digestion and absorption [48]. Previous studies to identify hepatic steatosis or NAFLD-related microbiota in humans or mouse models have mainly focused on fecal microbiota. However, fecal microbiota cannot represent the composition and abundance of microorganisms in each gut segment [49]. In the duodenum, the abundance of genus *Lactobacillus* varies greatly among individuals of different hepatic steatosis grades. Genus *Lactobacillus*, which is commonly considered probiotic bacteria, provides numerous health benefits to the host, including folate production [50], which was consistent with our results, indicating that individuals with hepatic steatosis had a higher abundance of genus *Lactobacillus* and higher quantity of folate in their duodenum. Folate prompts the final step of the methionine cycle from Hcy to Met. Mice fed a methionine-choline–deficient diet develop nonalcoholic fatty liver disease with severe steatohepatitis [51], and addition of folate reduces the incidence of hepatic steatosis [52]. Moreover, SAMe and Hcy accumulation increases the incidence of fatty liver diseases [53], corroborating our results of higher Hcy abundances in individuals with hepatic steatosis.

## Conclusion

We identified regulatory networks among genomic variants (GGA6: 5.59–5.69 Mb), gene expression (*PEMT* and *TOM1L2*), protein presence (PEMT), and metabolite abundance (SAMe, SAH, PC, PE, and Hcy) in hepatic steatosis and proposed for the first time that duodenal microbiota (m^2^ = 0.26) played more important roles in hepatic steatosis than other gut segments, and genus *Lactobacillus* in the duodenum might perform compensatory roles in alleviating hepatic steatosis by production of extra folate to prompt the host methionine cycle. This integrated analysis of host genomic variations, the hepatic transcriptome, proteome, metabolome, and gut microbiome, provided a comprehensive understanding of the host genetic and gut microbial factors for hepatic steatosis and novel insights into mechanistic analysis of human NAFLD.

## Materials and Methods

### Experimental design

The experimental cohorts used in this study comprised a total of 705 hens from a pedigreed line of Rhode Island Red in Beijing Huadu Yukou Poultry Breeding Co., Ltd. Birds were generated from 2 batches with a 10-day interval and reared in individual cages under similar conditions with 16L:8D (16 hours of light and 8 hours of dark). These birds were fed a basic corn-based diet (details are listed in Table 2) and provided with free access to feed and water. No antibiotics were administered to the hens in our study.

**Table 2:** Ingredients and nutrient composition of diets

Item	Composition
**Ingredients, % as feed**	
Corn	61.7
Soybean meal	24
Wheat bran	3.8
Limestone	8
Premix^1^	2.5
**Metabolism energy (kcal/kg)**	2,630
**Nutrients, % DM**	
Crude protein	15.9
Calcium	3.5
Total phosphorus	0.36
Lysine	0.79
Methionine	0.38

1 Premix provided the following per kilogram of diet: vitamin A, 9,975 IU; vitamin B_12_, 0.03 mg; vitamin E, 78.4 IU; vitamin VD_3_, 4,200 IU; riboflavin, 3.8 mg; niacin, 50.1 mg; calcium pantopantolate, 18.3 mg; biotin, 0.35 mg; iron, 58 mg; zinc, 101 mg; copper, 10 mg; manganese, 96 mg; and choline chloride, 480 mg.

At 90 weeks of age, body weight was measured using an electronic scale to the nearest 5 g. Blood samples were collected from the wing vein and stored at −20°C. Serum was separated by centrifugation at 3,000 × *g* for 15 minutes and stored at −20°C until use. Fecal samples were manually collected from the rectum with sterile cotton swabs. Each bird was then euthanized by cervical dislocation followed by decapitation. The contents of the duodenum, jejunum, ileum, and cecum, including the chyme and mucosa, were immediately collected after opening the abdomen. All intestinal samples were dispensed into 2-mL tubes, snap frozen in liquid nitrogen, and stored at −80°C.

The weights of the liver and abdominal fat tissue surrounding the gizzard, cloaca, and adjacent abdominal muscles were to the nearest 1 g with the electronic scale. Subsequently, some the liver tissue sample was frozen in liquid nitrogen and stored at −80°C immediately after collection for genomic DNA, tissue RNA, protein, and metabolite extraction. Some of the liver was placed in a sterile plastic bag on dry ice using forceps and stored at −20°C to measure biochemical indicators. The remaining liver sample was fixed in formalin for 48–72 hours for histological observation.

All experiments involving animals were conducted according to the ethical policies and procedures approved by the Institutional Animal Care and Use Committee of China Agricultural University, China (Issue No. 32303202–1-1).

### Liver histological assessment

Liver histology was assessed in liver sections embedded in paraffin and stained with H&E using standard techniques. Whole-section images of each liver sample were obtained using a Canon EOS 7D digital camera and quantified using ImageJ (version 1.8.0; National Institutes of Health). The investigators were blind to the group allocations. Therefore, a veterinary science pathologist performed the hepatic steatosis assessment using the NASH Clinical Research Network Scoring System in humans [54]. All liver samples were graded from 0 to 2, representing healthy individuals, and mild to moderate and severe hepatic steatosis. Individuals were categorized into 3 levels based on the severity of fatty liver: lipid droplets constituting less than 10% of the area in the control group (Ctrl), 10% to 50% in the mild to moderate group (HS-I), and more than 50% in the severe group (HS-II). For extremely typical individuals that could represent each group (nearly no lipid droplets in Ctrl, 30%–40% in HS-I and over 90% lipid droplets in HS-II), Oil Red O staining was performed to verify lipid droplet accumulation.

### Measurement of biochemical indicators in serum

STG, STC, SHDL, SLDL, and STBAs were analyzed using commercial kits (Shanghai Kehua Bioengineering Co., Ltd.) with the KHB ZY-1280 automatic biochemical analyzer (Shanghai Kehua Bioengineering Co., Ltd.). SVLDL was measured using a chicken very low-density lipoprotein ELISA kit (JLC10779) in accordance with the manufacturer’s instructions (Shanghai Kehua Bioengineering Co., Ltd.).

### Measurement of biochemical indicators in the liver

**Crude fat (CF) content measurement**. The CF content was measured by the soxhlet extraction method (AOAC 920.85) and performed with a soxhlet apparatus by refluxing with petroleum ether to remove CF in the sample. The difference between the weights of the initial sample and residue was the CF content.
**Protein quantitation**. The livers were taken out of the −80°C freezer, temporarily stored in liquid nitrogen, trimmed into small pieces with scissors, and weighed ranging from 0.01 to 0.05 g on an electronic scale. Protein quantification was performed using a protein quantification kit (A045-2) from Nanjing Jiancheng Institute of Biological Engineering Ltd. according to the manufacturer’s instructions.
**Hepatic biochemical indicator assay**. On the basis of protein quantification, HTGs, HTC, HFFAs, and HTBAs were measured using the Triglyceride Assay Kit (A110-2-1) from Nanjing Jiancheng Institute of Biological Engineering Co., Ltd. according to the manufacturer’s instructions.

### Whole-genome resequencing and data processing

Genomic DNA was isolated from liver samples of 705 hens using a Tiangen DNA Extraction Kit (Tiangen Biotech, DP304-2) according to the manufacturer’s instructions. After purification and integrity verification of the DNA, a total of 686 DNA samples were used for subsequent whole-genome resequencing. Host DNAs were amplified using PCR with 500-bp inserts for library construction. Whole-genome resequencing was performed using the Illumina HiSeq 2500 Sequencer (RRID:SCR_016383) to generate 150-bp paired-end reads. To ensure the quality of data, the adaptor-polluted reads, low-quality reads, and reads with number of N bases accounting for more than 5% were removed. The clean reads were then mapped to the chicken reference genome (GRCg6a) using the Burrows–Wheeler aligner (BWA; RRID:SCR_010910; version 0.7.15) [55] with the default parameters. We subsequently used Samtools (RRID:SCR_002105; version 1.3.1) [56] to sort reads and remove low-quality reads with the parameter “-q 4.” Duplicate reads resulting from PCR were removed using Picard tools [57]. The HaplotypeCaller protocol in Genome Analysis Toolkit (GATK; RRID:SCR_001876; version 4.2.0.0) [58] was used for SNPs and indels calling. To obtain high-quality SNPs, the SNPs were filtered with the GATK VariantFiltration protocol as follows: QD < 2.0, ReadPosRankSum < −8.0, FS > 60.0, QUAL < 30.0, DP < 4.0, MQ < 40.0, MappingQualityRankSum < −12.5 and INDEL: QD < 2.0, ReadPosRankSum < −20.0, FS > 200.0, QUAL < 30.0, DP < 4.0. Finally, PLINK (RRID:SCR_001757; version 2.0) [59] was used for filtering annotated SNP data with the following parameters: sample call rate > 90%, SNP call rate > 90%, and minor allele frequencies > 1%. The remaining SNPs and individuals were used for imputation in BEAGLE (RRID:SCR_001789; version 5.1) [60], and the PLINK analysis was performed again using the same criteria as described above. After these steps, a total of 5,904,820 SNPs distributed across 32 chromosomes and 686 birds were retained for subsequent analysis.

### Genome-wide analysis study

To reveal the impact of host genetics on shaping the phenotypes, all valid individuals and SNPs were involved in GWAS with a univariate linear mixed model (LMM), which was performed using GEMMA (RRID:SCR_008007; version 0.98.4) [61]. The statistical model applied in this study is as follows:


\begin{eqnarray*}
{\mathrm{y}} = {\mathrm{W\alpha }} + {\mathrm{x}}\beta + {\mathrm{u}} + \varepsilon
\end{eqnarray*}

where y is the phenotypic values of 686 individuals, W is a matrix of covariates (fixed effects: top 5 principal components and batch effects) controlling for population structure, α refers to a vector of corresponding effects that compose the intercept, x denotes the marker genotypes, β is the corresponding marker’s effect, u is a vector of random polygenic effects with a covariance structure, and ε is vector of random residuals.

The likelihood ratio test *P* value was selected as a criterion for examining the significance of the association between SNPs and phenotypes. The genome-wide significant threshold was determined using a modified Bonferroni correction with an R package named simpleM, as previously described [62]. Using this approach, a total of 150,802 valid inspections were obtained, and thereby the genome-wide significance and suggestive significance thresholds were defined as 3.32 × 10^−7^ (0.05/150,802) and 6.63 × 10^−6^ (1/150,802), respectively.

For the sake of exploring the effects of host genetics on the gene expression in liver tissue, GWASs were conducted in Genome-Wide Complex Trait Analysis (GCTA) software (version 1.93.2) [63] with the support of fastGWA [44]. Out of 686 samples, 668 transcriptome-sequenced subjects were included for subsequent genome and gene expression association analysis in our study. Read counts were normalized using transcripts per million (TPM) initially. Genes were selected based on the expression thresholds of ≥0.1 TPM and ≥6 reads (unnormalized) in ≥20% samples. Afterward, read counts were normalized between samples using Trimmed Mean of M-values (TMM). Eventually, 12,191 genes remained in this part. Likewise, an LMM was employed throughout the analysis. For fastGWA, a full-dense genetic relationship matrix (GRM) was generated, based on which a sparse GRM was built at a cutoff value of 0.05. fastGWA was then run using this sparse GRM with expression level as the dependent variable and SNP genotype values as the independent variable. Significant and suggestively significant *P* value thresholds were 3.32 × 10^−7^ and 6.63 × 10^−6^ as described above.

### eQTL mapping and Mendelian randomization analysis

For each gene, we took all genetic variants into consideration and used the following covariates: top 5 genetic principal components, batch effects, and top 3 probabilistic estimation of expression residuals (PEER) factors. The number of PEER factors included in calculation equaling 60 was determined from the sample size corresponding to the previously reported research: 15 for *n* < 150, 30 for 150 ≤ *n* < 250, 45 for 250 ≤ *n* < 350, and 60 for *n* ≥ 350 [64]. Thereafter, the permutations of *cis*-QTL mapping were conducted to generate phenotype-level summary statistics with empirical *P* values and *trans*-QTL mapping to compute nominal associations between all phenotypes and genotypes. Notably, the *cis*-window referred to ranged from 1 Mbps upstream to 1 Mbps downstream of the transcription start sites (TSS), while for *trans*-QTL mapping, 5,315,471 common genetic variants passing strict quality control criteria (minor allele frequency (MAF) > 5% and outside of TSS ± 5 Mbps) were contained in the process. Correction for multiple testing was done using FDR for *cis*-eQTL analysis, resulting in a *P* value threshold of 8.05 × 10^−6^ for *cis*-eQTLs. Given that the remaining SNPs used in *trans*-QTL mapping were analogous to those in GWAS, we considered its *P* value threshold as 3.32 × 10^−7^.

**Figure 6:**
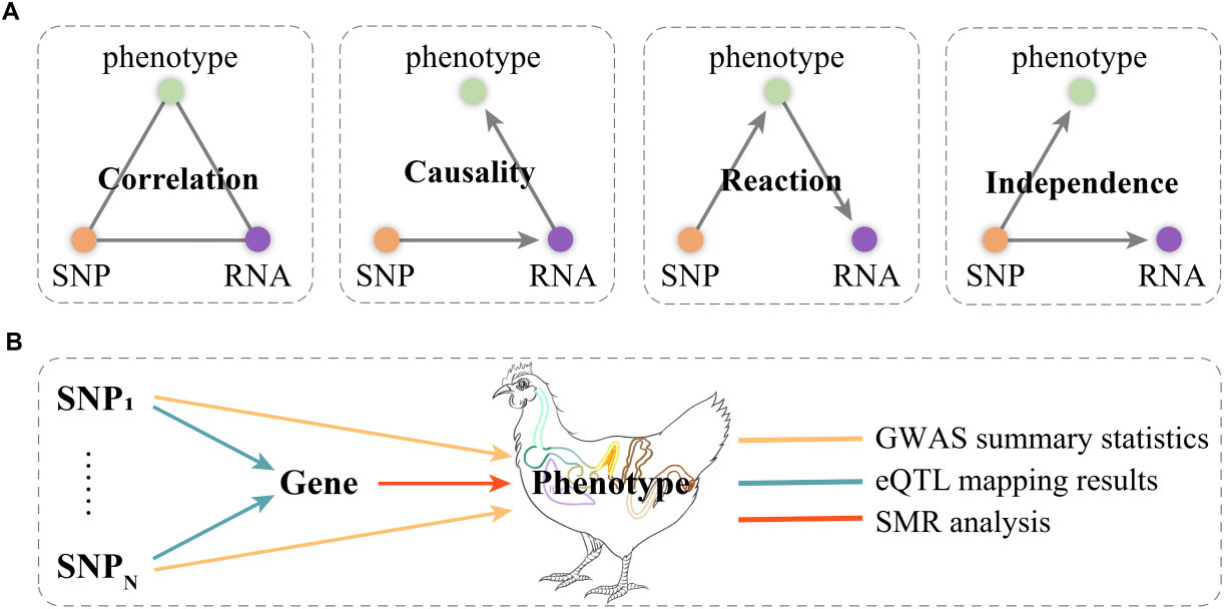
Principles of eQTL mapping and SMR analysis. (A) Differential RNA transcript abundance could potentially act as the causal link between a specific eQTL SNP and the phenotype connected to that SNP. Yet, the identification of a comparable correlation might also be attributed to 2 alternative scenarios: a reactive connection between RNA transcript abundance and the phenotype or an independence relationship involving the eQTL, RNA transcript abundance, and the phenotype. (B) SMR was employed to scrutinize the potential pleiotropic association between the expression level of a gene and a complex trait of interest, utilizing summary statistics from GWAS and results from eQTL mapping.

Subsequently, top eQTL-based SMR [65] analysis was performed to prioritize genes underlying GWAS associations. The panel in Fig. 6A illustrates possible causal relationships between an SNP, a transcript (RNA), and a phenotype [66]. Gene expression functions as the casual link between an eQTL genetic variant and the phenotype associated with that variant. Nonetheless, the observed correlation might arise from another 2 alternative relationships: a reactive connection between the phenotype and gene expression or an independent relationship between the genetic variant and both the phenotype and gene expression. Top eQTL-based SMR analysis uses SNPs as instrumental variables to assess causal relationships between a risk factor (gene expression levels) and an outcome (HSC). Large-scale eQTL mapping was conducted to identify SNPs associated with gene expression levels. From the pool of identified eQTLs, the most significant ones (top eQTLs) based on statistical criteria were selected. The independence of instrumental variables from each other is crucial to meet the assumptions of Mendelian randomization. To evaluate the degree of linkage disequilibrium (LD) between SNPs, SNPs in high LD are removed with the top associated eQTL. SMR uses an instrumental variable estimation in order to accurately integrate independent GWAS and eQTL summary data (Fig. 6B). The SMR procedure consists of 2 steps: (i) identification of variants independently associated with the exposure factor and (ii) calculation of causal estimates. Before that, we made a BESD file and updated coordinates of SNPs and genes, as well as the frequency of effect allele. For each GWAS summary statistic, SNPs significantly and suggestively significantly associated with the traits were selected as SMR input files to determine the connection with significant *cis*-eQTLs and *trans*-eQTLs. Finally, we selected only variants that showed an association at an FDR of 0.05 by adjusting *P* values using the Benjamini–Hochberg procedure.

## 16S rRNA gene sequencing

The gut digesta (duodenum, jejunum, ileum, and cecum) and fecal samples of 705 individuals were thawed on ice and homogenized, and ∼200 mg of each sample was used to extract the microbial genome DNA using the QIAamp Stool Mini Kit (QIAGEN, D4015-01) according to the manufacturer’s recommendations. The hypervariable V4 region of the 16S gene was amplified using the Ion Plus Fragment Library Kit 48 rxns (Thermo Scientific). Sequencing was performed on an Ion S5TM XL platform, and 400-bp single-end reads were generated, in accordance with the manufacturer’s instructions. Sequences were imported and processed using Quantitative Insights Into Microbial Ecology (QIIME2, version 2019.10) [67] for further bioinformatics analyses. After trimming the barcode and primer sequences, the preliminary quality screening was performed for the original high-throughput sequencing data using the QIIME2 plugin DADA2 [68], and the sequences were trimmed to a final length of 252 bp. The remaining high-quality sequences were clustered and classified by ASVs with 100% identity [69]. ASVs that presented in less than 1% (7) samples and had an average relative abundance below 10^−6^ were removed for subsequent analyses. Taxonomic assignments for each ASV were made via similarity searching against the SILVA 16S rRNA gene sequence reference database (Release 132) [70]. The α and β diversity were calculated with the vegan package [71].

### Heritability and microbiability estimation

The 5,904,820 filtered SNPs were used to construct a GRM using GCTA software (version 1.93.2) [63]. The GRM estimation model used was


\begin{eqnarray*}
{{\mathrm{g}}}_{{\mathrm{ij}}} = \frac{1}{N}\sum\nolimits_{V = 1}^N {\frac{{(Xiv - 2\overline {pv} )(xjv - 2\overline {pv} )}}{{2\overline {pv} (1 - \overline {pv} )}}}
\end{eqnarray*}

In this expression, g_ij_ denotes the genetic relationship between individuals i and j; x_iv_ and x_jv_ denote the number of reference alleles in hens i and j, respectively; p_v_ denotes the reference allele frequency; and N is the SNP number. The SNP-based heritability of the host phenotypes was estimated with the following model:


\begin{eqnarray*}
{\mathrm{Y}} = {\mathrm{Kc}} + {\mathrm{g}} + {\mathrm{e}}
\end{eqnarray*}

In this expression, y denotes a vector of the phenotype, c denotes a vector of fixed covariates (including batch effect and the first 10 host genetic principal components), K denotes the corresponding matrix for c, and g denotes a vector of the total effects of all SNPs with ∼ N (0, Gσ^2^A), where G and Gσ^2^A denote the GRM and genetic variance, respectively, and e denotes the residual effect.

The phenotypic variance explained by gut microbial variance is defined as microbiability (m^2^) in animals [72, 73], and it was estimated with GCTA software using the microbial relationship matrix (MRM). The construction of the microbial relationship matrix and phenotypic variance explained by the gut microbial variance were estimated as described in our previous study [74]. We corrected batch effects and the first 5 host genetic principal components in this analysis. All filtered ASVs in duodenum were normalized by zero-centering and scaling to unit variance to construct the MRM as previously described with an R script based on the following equation:


\begin{eqnarray*}
{m}_{sij} = \frac{1}{{{N}_S}}\sum\nolimits_{a = 1}^{{N}_S} {\frac{{({x}_{sia} - \overline {{x}_{sa}} )({x}_{sjo} - \overline {{x}_{sa}} )}}{{\sigma _S^2}}}
\end{eqnarray*}

where m_sij_ represents the estimated microbial relationship in the sampling site s between birds i and j; x_sia_ and x_sja_ denote the relative abundances of ASV a in the sampling site s in birds i and j, respectively; $\overline {{x}_{sa}} $ stands for the average relative abundance of the ASV a in the sampling site s in the population; $\sigma _s^2$ is the variance of the abundance of ASV a; and Ns is the total number of ASVs in the sampling site s used for the relatedness computation.

### Liver tissue transcriptome

A total of 686 samples were used for transcriptome sequencing. The Eastep Super Total RNA Extraction Kit (Promega, LS1040) was used to extract total RNA according to the manufacturer’s instructions. The RNA concentration and purity were determined using the NanoDrop ND-2000 spectrophotometer (Thermo Fisher Scientific). The integrity of the RNA was assessed using the RNA Nano 6000 Assay Kit of the Bioanalyzer 2100 system (Agilent Technologies). Libraries for transcriptome sequencing were constructed following the standard Illumina RNA sequencing instruction. The libraries were sequenced on an Illumina Novaseq platform and 150-bp paired-end reads were generated. Fastp (RRID:SCR_016962; version 0.20.1) [75] was used to remove the reads containing adaptor contamination, low-quality bases, and undetermined bases. Then, the quality-controlled sequencing data were aligned to the chicken reference genome (GRCg6a) using HISAT2 (RRID:SCR_015530; version 2.0.5) [76] with default parameters. After that, we employed featureCounts (RRID:SCR_012919; version 1.6.3) [77] to count the reads for each gene. The differentially expressed genes between different groups were identified with the assistance of DESeq2 (RRID:SCR_015687; version 3.16) [78]. The significance threshold for the differential expression was adjusted *P* value < 0.05 and a |log2 fold change | > 1.

### Liver tissue proteome

For protein extraction and digestion, chicken liver tissues (4 samples/group from eHSC groups) were ground with liquid nitrogen into cell powder and transferred to a 5-mL centrifuge tube. The protein concentration was determined with the BCA kit (Thermo Fisher Scientific, 23225) according to the manufacturer’s instructions, and protein digestion was conducted just as Song et al. [79] illustrated. Subsequent tandem mass tagging (TMT) labeling, high-performance liquid chromatography (HPLC) fractionation, and liquid chromatography-tandem mass spectrometry (LC-MS/MS) analysis of the TMT‐labeled peptides were performed as previously described, too [79]. Automatic gain control (AGC) target was set at 1 × 10^−5^, with an intensity threshold of 3.3 × 10^−4^ and a maximum injection time of 60 ms.

Downstream database search was performed using the MaxQuant search engine (v.1.6.15.0). Tandem mass spectra were searched against the *Gallus gallus* database (27,535 entries) concatenated with the reverse decoy database. Trypsin/P was specified as a cleavage enzyme, allowing up to 2 missing cleavages. The mass tolerance for precursor ions was set as 20 ppm in the first search and 5 ppm in the main search, and for fragment ions, it was set as 0.02 Da, respectively. Carbamidomethyl on Cys was specified as a fixed modification, and acetylation on protein N-terminal and oxidation on Met were specified as variable modifications. The threshold of FDR adjusted *P* value was set to 0.01.

### Widely targeted metabolome

Livers were thawed in a 50-μL ice-cold mixture (methanol/water = 7:3, v/v) and homogenized after adding a 150-μL solution (methanol/water = 7:3, v/v) containing internal standard. The sample was placed on ice for 15 minutes and centrifuged at 12,000 rpm for 10 minutes (4°C). The collected supernatant was placed at −20°C for 30 minutes and then centrifuged at 12,000 rpm for 3 minutes (4°C), followed by transferring around 120 μL aliquots of supernatant. The solution obtained was analyzed using an LC-ESI-MS/MS system (UPLC, ExionLC AD, MS, QTRAP System, [80]). The chromatographic separation was achieved by using water and acetonitrile (with 0.1% formic acid for each) as the mobile phase. The elution gradient program was 95:5 v/v at 0 minutes, 10:90 v/v at 11.0 minutes, 10:90 v/v at 12.0 minutes, 95:5 v/v at 12.1 minutes, and 95:5 v/v at 14.0 minutes with 0.40 mL/min of flow rate. The column temperature was 40°C, and the injection volume was 2 μL.

Linear ion trap (LIT) and triple quadrupole scans were acquired on a triple quadrupole–linear ion trap mass spectrometer (QTRAP), QTRAP LC-MS/MS System, equipped with an ESI Turbo Ion-Spray interface, operating in positive and negative ion mode and controlled by Analyst 1.6.3 software (Sciex). The ESI source operation parameters and successor operations followed the parameters by Chen et al. [81].

Significantly regulated metabolites between groups were determined by importance in the projection (VIP) (VIP ≥ 1), *P* value (*P* < 0.05), and absolute log2FC (|log2FC| ≥ 1.0). VIP values were generated from the orthogonal partial least-squares discriminant analysis (OPLS-DA) result using R package MetaboAnalystR. The data were log transformed (log_2_) and mean centered before OPLS-DA. In order to avoid overfitting, a permutation test (200 permutations) was performed.

### Liver tissue and duodenal mucosa assays

To validate the results of the metabolome, we randomly chose 10 individuals in each group (eCtrl, eHS-I, and eHS-II), and the levels of folic acid (FLA), homocysteine (Hcy), methionine (Met), S-adenosine homocysteine (SAH), and S-adenosylmethionine (SAMe) were determined in liver tissue and duodenal chyme samples by ELISA kits (Enzyme-Linked Biotechnology Co. Ltd.) in accordance with the manufacturer’s instructions.

### Statistical analysis

Differences in phenotypes among HSC groups were determined in R version 4.0.2 [82] using the Wilcoxon rank-sum test with a post hoc test to correct for multiple comparisons. ANOVA was performed to determine differences in liver gene expression (TPM) and biochemical indicators among HSC groups with FDR correction for multiple testing.

Spearman’s rank coefficient of correlations was calculated between the relative abundance of duodenal microbiota of various taxonomic levels ranging from phylum to species and HSC. Prior to this, the relative abundance of the microorganisms that presented in ≥60% of the population equaling 0 was converted to NA (not available), because it was considered to escape from detection. Otherwise, microorganisms detected in <60% and ≥30% of samples were dichotomized into presence/absence patterns, and we encoded the phenotype as a binary vector to prevent zero inflation, which led to a bimodal distribution. Microorganisms detected in <30% of samples were excluded from this analysis, as reported previously [74, 83]. Furthermore, LEfSe [84] was employed to identify differentially abundant microbial taxa from the phylum to genus level within HSC groups. The analysis incorporated LDA to quantify the effect size of each taxon. Taxa with high LDA scores were considered more influential in distinguishing between the HSC groups. The LEfSe analysis followed specific conditions: (i) the α value for the factorial Kruskal–Wallis test was set to less than 0.05, (ii) the α value for the pairwise Wilcoxon test in taxonomic compositions was set to less than 0.05, (iii) the threshold on the logarithmic LDA score for discriminative features was set to less than 2.0, and (iv) multiclass analysis was configured as all-against-all.

The relationship between hepatic biochemical indicators and the relative abundance of bacteria with a call rate ranging from 30% to 60% was measured using the Spearman correlation, whereas the Pearson correlation coefficient was employed to test the strength of the association between hepatic biochemical indicators and the relative abundance of bacteria in ≥60% of samples. Correlations between the TPM value and HSC were also calculated using the Spearman method. FDR corrections were carried out in all of the abovementioned analyses.

## Supplementary Material

giae023_Supplementary_Tables

giae023_GIGA_D_23_00122_Original_Submission

giae023_GIGA_D_23_00122_Revision_1

giae023_GIGA_D_23_00122_Revision_2

giae023_GIGA_D_23_00122_Revision_3

giae023_GIGA_D_23_00122_Revision_4

giae023_Response_to_Reviewer_Comments_Original_Submission

giae023_Response_to_Reviewer_Comments_Revision_1

giae023_Response_to_Reviewer_Comments_Revision_2

giae023_Response_to_Reviewer_Comments_Revision_3

giae023_Reviewer_1_Report_Original_Submission Huanzi Zhong, Ph.D. -- 11/15/2023

giae023_Reviewer_1_Report_Revision_1 Huanzi Zhong, Ph.D. -- 11/15/2023

giae023_Reviewer_1_Report_Revision_2 Huanzi Zhong, Ph.D. -- 3/12/2024

giae023_Reviewer_1_Report_Revision_3 Huanzi Zhong, Ph.D. -- 3/15/2024

giae023_Reviewer_2_Report_Original_Submission Gengjie Jia -- 12/17/2023

giae023_Reviewer_2_Report_Revision_1 Gengjie Jia -- 1/30/2024

## Data Availability

Whole-genome resequencing data are available on the NCBI Sequence Read Archive (SRA) under accession SUB13720715, and RNA-seq data are available on the SRA under the accession SUB13062074. 16S rRNA sequencing data can be accessed on SRA under the accession SUB12033010 (duodenum), SUB12035295 (jejunum), SUB12035349 (ileum), SUB12035378 (cecum), and SUB12035409 (feces). Raw data for metabolomics were submitted to MetaboLights at MTBLS7808, and raw data for proteomics were submitted to PRIDE at PXD042451. All supporting data and materials are available in the *GigaScience* GigaDB database [102].

## Additional Files

**Supplementary Fig. S1**. Correlations among all recorded phenotypes, including the hepatic steatosis classification (HSC), hepatic and serum triglyceride (HTG and STG, respectively), hepatic crude fat (HCF) and free fatty acids (HFFAs), serum high-density lipoprotein (SHDL), low-density lipoprotein (SLDL) and very low-density lipoprotein (SVLDL), hepatic and serum total bile acids (HTBAs and STBAs, respectively), hepatic and serum total cholesterol (HTC and STC, respectively), and abdominal fat weight (AFW). The lower panel shows scatterplots for each pair of observations. Each point represents an individual. *n* = 673. **P* < 0.05, ***P* < 0.01, ****P* < 0.001.

**Supplementary Fig. S2**. Violin plots of the change patterns of hepatic triglyceride (HTG), hepatic and serum total bile acids (HTBAs and STBAs, respectively), and abdominal fat weight (AFW) changed with hepatic steatosis in eHSC groups (*P*_adj_ < 0.05, post hoc Wilcoxon rank-sum test). Data are from *n* = 30 biological replicates. **P* < 0.05, ***P* < 0.01, ****P* < 0.001.

**Supplementary Fig. S3**. The amount and distribution of *cis*- and *trans*-regulated genes in liver.

**Supplementary Fig. S4**. GO and pathway enrichment analysis of 227 candidate genes identified many biological processes of lipid localization and biosynthetic processes.

**Supplementary Fig. S5**. (A) Permutation test of duodenal microbiability of hepatic steatosis classification (HSC), hepatic free fatty acids (HFFAs) and hepatic total bile acids (HTBAs), and cecal microbiability of hepatic crude fat (HCF) and hepatic triglyceride (HTG). The results indicated that the estimated m^2^ (0.15–0.27, blue lines) were significantly higher than the simulated ones (average m^2^ ranged from 0.02–0.04, gray lines). *n* ≥ 673. (B) The amount of taxa classified from phylum to species with high-quality ASVs. *n* = 705. (C) The α diversities, including Shannon and Simpson indices, both decreased with hepatic steatosis development (*P*_adj_ < 0.05, post hoc Wilcoxon rank-sum test). n = 673. **P* < 0.05, ***P* < 0.01.

**Supplementary Fig. S6**. Heatmaps of the association of the relative abundance of phyla, classes, orders, families, and species with all recorded phenotypes (Spearman’s rank-based correlation analysis, + indicates statistical significance *P*_adj_ < 0.05, FDR correction). AFW: abdominal fat weight; HCF: hepatic crude fat; HFFA: hepatic free fatty acid; HSC: hepatic steatosis classification; HTBAs: hepatic total bile acids; HTC: hepatic total cholesterol; HTG: hepatic triglyceride; SHDL: serum high-density lipoprotein; SLDL: serum low-density lipoprotein; STBAs: serum total bile acids; STC: serum total cholesterol; STG: serum triglyceride; SVLDL: serum very low-density lipoprotein. *n* ≥ 673.

**Supplementary Table S1**. Summary statistics of whole-genome resequencing.

**Supplementary Table S2**. Significant SNPs of GWAS on HSC with AFP as an additional covariate.

**Supplementary Table S3**. SMR test based on the GWAS result of HSC.

**Supplementary Table S4**. SRC analysis between the expression of each gene and HSC.

**Supplementary Table S5**. GO and pathway enrichment analysis based on the significant genes selected from SRC analysis and ANOVA.

**Supplementary Table S6**. Related reports of genes involved in hepatic steatosis, NAFLD, or hepatic lipid metabolism in human and rat.

**Supplementary Table S7**. Differential expression genes detected from eHSC groups.

**Supplementary Table S8**. Proteins quantified by hepatic proteome.

**Supplementary Table S9**. Metabolites quantified by hepatic metabolome.

**Supplementary Table S10**. Summary statistics of 16S rRNA gene sequencing.

**Supplementary Table S11**. Estimation of microbiability (m^2^) for 5 hepatic steatosis–related phenotypes in each gut segment.

**Supplementary Table S12**. SRC analysis performed between the abundance of microbial taxa in each gut segment and HSC.

## Abbreviations

ACSL: acyl-CoA synthetase; AFW: abdominal fat weight; AHCY: adenosylhomocysteinase; ANOVA: analysis of variance; ASV: amplicon sequence variant; CAT: catalase; CHKA: choline kinase; CHPT1: choline phosphotransferase 1; CPT1A: carnitine palmitoyltransferase 1A; CPT2: carnitine palmitoyltransferase 2; FA: fatty acid; FLA: folic acid; FLS: fatty liver syndrome; FMT: fecal microbiota transplantation; GCTA: genome-wide complex trait analysis; GPX3: peroxidase 3; GRM: genetic relationship matrix; GSH: glutathione; GWAS: genome-wide analysis study; H&E: hematoxylin and eosin; h2: heritability; H2O2: hydrogen peroxide; HCF: hepatic crude fat; Hcy: homocysteine; HFFA: hepatic free fatty acid; HNE: 4-hydroxynonenal; HODE: hydroxy-octadecadienoic acid; HSC: hepatic steatosis classification; HTBA: hepatic total bile acid; HTC: hepatic total cholesterol; HTG: hepatic triglyceride; LCAC: long-chain acylcarnitine; LCFA: long-chain fatty acid; LDA: linear discriminant analysis; LEfSe: linear discriminant analysis effect size; m2: microbiability; MAFLD: metabolic (dysfunction) associated fatty liver disease; MAT: methionine adenosyltransferase; MDA: malondialdehyde; Met: methionine; MRM: microbial relationship matrix; MT: methyltransferase; MTHF: methyltetrahydrofolate; MTR: 5-methyltetrahydrofolate-homocysteine methyltransferase; NAFLD: nonalcoholic fatty liver disease; O2−: superoxide; oxoODE: oxo-octadecadienoic acid; PC: phosphatidylcholine; PCYT1A: phosphate cytidylyltransferase 1; PE: phosphatidyl-ethanolamine; PEMT: phosphatidyl-ethanolamine N-methyltransferase; QIIME2: Quantitative Insights Into Microbial Ecology; SAH: S-adenosylhomocysteine; SAMe: S-adenosylmethionine; SCAC: short-chain acylcarnitine; SHDL: serum high-density lipoprotein; SI: small intestine; SLC25A20: solute carrier family 25 member 20; SLDL: serum low-density lipoprotein; SMR: summary data–based Mendelian randomization; SOD 1/2: superoxide dismutase 1/2; SRC: Spearman’s rank-based correlation; STBA: serum total bile acid; STC: serum total cholesterol; STG: serum triglyceride; SVLDL: serum very low-density lipoprotein; THF: tetrahydrofolate; TSS: transcription start sites; VLDL: very low-density lipoprotein.
