# Correction to: mechanisms of hepatic steatosis in chickens: integrated analysis of the host genome, molecular phenomics and gut microbiome

**DOI:** 10.1093/gigascience/giae069

**Published:** 2024-08-31

**Authors:** Congjiao Sun, Fangren Lan, Qianqian Zhou, Xiaoli Guo, Jiaming Jin, Chaoliang Wen, Yanxin Guo, Zhuocheng Hou, Jiangxia Zheng, Guiqin Wu, Guangqi Li, Yiyuan Yan, Junying Li, Qiugang Ma, Ning Yang

**Affiliations:** Department of Animal Genetics and Breeding, College of Animal Science and Technology, China Agricultural University, Beijing 100193, China; Department of Animal Genetics and Breeding, College of Animal Science and Technology, China Agricultural University, Beijing 100193, China; Department of Animal Genetics and Breeding, College of Animal Science and Technology, China Agricultural University, Beijing 100193, China; Department of Animal Genetics and Breeding, College of Animal Science and Technology, China Agricultural University, Beijing 100193, China; Department of Animal Genetics and Breeding, College of Animal Science and Technology, China Agricultural University, Beijing 100193, China; Department of Animal Genetics and Breeding, College of Animal Science and Technology, China Agricultural University, Beijing 100193, China; Department of Animal Genetics and Breeding, College of Animal Science and Technology, China Agricultural University, Beijing 100193, China; Department of Animal Genetics and Breeding, College of Animal Science and Technology, China Agricultural University, Beijing 100193, China; Department of Animal Genetics and Breeding, College of Animal Science and Technology, China Agricultural University, Beijing 100193, China; Beijing Engineering Research Centre of Layer, Beijing 101206, China; Beijing Engineering Research Centre of Layer, Beijing 101206, China; Beijing Engineering Research Centre of Layer, Beijing 101206, China; Department of Animal Genetics and Breeding, College of Animal Science and Technology, China Agricultural University, Beijing 100193, China; Department of Animal Genetics and Breeding, College of Animal Science and Technology, China Agricultural University, Beijing 100193, China; Department of Animal Genetics and Breeding, College of Animal Science and Technology, China Agricultural University, Beijing 100193, China

This is a correction to:

*GigaScience, Volume 13,2024, giae023*

https://doi.org/10.1093/gigascience/giae023

In the original version of the article “Mechanisms of hepatic steatosis in chickens: integrated analysis of the host genome, molecular phenomics and gut microbiome” by Congjiao Sun et al. [1], the first affiliation was incorrect, it should be changed from “Department of Animal Genetics and Breeding, College of Animal Science and Technology, China Agricultural University, Beijing 100193, China” to “State Key Laboratory of Animal Biotech Breeding, Department of Animal Genetics and Breeding, College of Animal Science and Technology, China Agricultural University, Beijing 100193, China.” The authors apologize for the errors.
