# Supplementary material for: Mechanisms of hepatic steatosis in chickens: integrated analysis of the host genome, molecular phenomics and gut microbiome
Source: Gigascience. 2024 Jun 5;13:giae023. doi: 10.1093/gigascience/giae023 (PMC11152177; doi:10.1093/gigascience/giae023)
Supplement: giae023_GIGA_D_23_00122_Original_Submission [file giae023_giga_d_23_00122_original_submission.pdf]

GigaScience

Mechanisms of hepatic steatosis in chickens: integrated analysis of the host genome, molecular phenomes and gut microbiome

--Manuscript Draft--

**Manuscript Number:**	GIGA-D-23-00122
**Full Title:**	Mechanisms of hepatic steatosis in chickens: integrated analysis of the host genome, molecular phenomes and gut microbiome
**Article Type:**	Research
**Funding Information:**	National Natural Science Foundation of China (31930105) — Professor Ning Yang<br>Agriculture Research System of China (CARS-40) — Professor Ning Yang<br>National Key Research and Development Programof China (2022YFF1000204) — Professor Ning Yang<br>2115 Talent Development Progarm of China Agricultural University — Associate professor Congjiao Sun
**Abstract:**	Hepatic steatosis is the initial manifestation of abnormal liver functions and often leads to liver diseases such as non-alcoholic fatty liver disease in humans and fatty liver syndrome in animals. In this study, we conducted a comprehensive analysis of a large chicken population consisting of 705 adult hens by combining host genome resequencing, liver transcriptome, proteome, and metabolome analysis, as well as microbial 16S rRNA gene sequencing of each gut segment. The results showed the heritability (h2=0.25) and duodenal microbiability (m2=0.26) of hepatic steatosis were relatively high, indicating a large effect of host genetics and duodenal microbiota on chicken hepatic steatosis. Individuals with hepatic steatosis had low microbiota diversity and a decreased genetic potential to process triglyceride output from hepatocytes, fatty acid β-oxidation activity, and resistance to fatty acid peroxidation. Furthermore, we revealed a molecular network linking host genomic variants (GGA6: 5.59–5.69 Mb), hepatic gene/protein expression (PEMT, phosphatidyl-ethanolamine N-methyltransferase), metabolite abundances (folate, S-adenosylmethionine, homocysteine, phosphatidyl-ethanolamine, and phosphatidylcholine) and duodenal microbes (genus Lactobacillus) to hepatic steatosis, which could provide new insights into the regulatory mechanism of fatty liver development.
**Corresponding Author:**	Congjiao Sun<br>China Agricultural University<br>Beijing, CHINA
**Corresponding Author Secondary Information:**	
**Corresponding Author's Institution:**	China Agricultural University
**Corresponding Author's Secondary Institution:**	
**First Author:**	Congjiao Sun
**First Author Secondary Information:**	
**Order of Authors:**	Congjiao Sun<br>Fangren Lan<br>Qianqian Zhou<br>Xiaoli Guo<br>Jiaming Jin<br>Chaoliang Wen

Powered by Editorial Manager® and ProduXion Manager® from Aries Systems Corporation

	Yanxin Guo
	Zhuocheng Hou
	Jiangxia Zheng
	Guiqin Wu
	Guangqi Li
	Yiyuan Yan
	Junying Li
	Qiugang Ma
	Ning Yang
**Order of Authors Secondary Information:**	
**Additional Information:**	
**Question**	**Response**
Are you submitting this manuscript to a special series or article collection?	No
**Experimental design and statistics**<br><br>Full details of the experimental design and statistical methods used should be given in the Methods section, as detailed in our Minimum Standards Reporting Checklist. Information essential to interpreting the data presented should be made available in the figure legends.<br><br>Have you included all the information requested in your manuscript?	Yes
**Resources**<br><br>A description of all resources used, including antibodies, cell lines, animals and software tools, with enough information to allow them to be uniquely identified, should be included in the Methods section. Authors are strongly encouraged to cite Research Resource Identifiers (RRIDs) for antibodies, model organisms and tools, where possible.<br><br>Have you included the information requested as detailed in our Minimum Standards Reporting Checklist?	Yes

Powered by Editorial Manager® and ProduXion Manager® from Aries Systems Corporation

**Availability of data and materials**<br><br>All datasets and code on which the conclusions of the paper rely must be either included in your submission or deposited in publicly available repositories (where available and ethically appropriate), referencing such data using a unique identifier in the references and in the “Availability of Data and Materials” section of your manuscript.<br><br>Have you have met the above requirement as detailed in our Minimum Standards Reporting Checklist?	Yes

Powered by Editorial Manager® and ProduXion Manager® from Aries Systems Corporation

Manuscript

Click here to access/download;Manuscript;manuscript_2023.5.9.docx

Research article

Manuscript title

Mechanisms of hepatic steatosis in chickens: integrated analysis of the host genome, molecular phenomes and gut microbiome

Running title: Regulatory mechanisms of hepatic steatosis in chickens

Authors

Congjiao Sun[1†*], Fangren Lan[1†], Qianqian Zhou[1†], Xiaoli Guo[1†], Jiaming Jin[1], Chaoliang Wen[1], Yanxin Guo[1], Zhuocheng Hou[1], Jiangxia Zheng[1], Guiqin Wu[2], Guangqi Li[2], Yiyuan Yan[2], Junying Li[1], Qiugang Ma[1] and Ning Yang[1*]

[1]Department of Animal Genetics and Breeding, College of Animal Science and Technology, China Agricultural University, Beijing 100193, China

[2]Beijing Engineering Research Centre of Layer, Beijing 101206, China

[†] These authors contributed equally to this work.

[*]To whom correspondence should be addressed. nyang@cau.edu.cn; cjsun@cau.edu.cn.

Abstract

Hepatic steatosis is the initial manifestation of abnormal liver functions and often leads to liver diseases such as non-alcoholic fatty liver disease in humans and fatty liver syndrome in animals. In this study, we conducted a comprehensive analysis of a large chicken population consisting of 705 adult hens by combining host genome resequencing, liver transcriptome, proteome, and metabolome analysis, as well as microbial 16S rRNA gene sequencing of each gut segment. The results showed the heritability (h^2=0.25) and duodenal microbiability (m^2=0.26) of hepatic steatosis were relatively high, indicating a large effect of host genetics and duodenal microbiota on chicken hepatic steatosis. Individuals with hepatic steatosis had low microbiota diversity and a decreased genetic potential to process triglyceride output from hepatocytes, fatty acid β-oxidation activity, and resistance to fatty acid peroxidation. Furthermore, we revealed a molecular network linking host genomic variants (GGA6: 5.59–5.69 Mb), hepatic gene/protein expression (*PEMT*, phosphatidyl-ethanolamine N-methyltransferase), metabolite abundances (folate, S-adenosylmethionine, homocysteine, phosphatidyl-ethanolamine, and phosphatidylcholine) and duodenal microbes (genus *Lactobacillus*) to hepatic steatosis, which could provide new insights into the regulatory mechanism of fatty liver development.

Key words: Chickens, Hepatic steatosis, Genetics, Microbiota, Integrative analysis

Introduction

Lipid metabolism plays a crucial role in maintaining animal life and ensuring normal physiological functions. Dysregulations of fat metabolism can lead to fatty liver

diseases, and hepatic steatosis is one of their first symptoms, which can progress to non-alcoholic fatty liver disease (NAFLD) in humans or fatty liver syndrome (FLS) in farm animals, such as chickens, with more severe cases developing blood clots and liver rupture. A fatty liver is strongly influenced by host genetics with heritability ranging from 0.38 to 0.70. Additionally, genetic variations that strongly influence NAFLD in humans have been confirmed by many studies, such as the I148M mutation in the *PNPLA3* gene and the *E167K* mutation in the *TM6SF2* gene. Experts have reached a consensus that metabolic (dysfunction) associated fatty liver disease (MAFLD) may be a more appropriate and inclusive definition than NAFLD in humans [1]. This change emphasizes the significance of lipid metabolism homeostasis, which is involved in hepatic de novo lipogenesis, β-oxidation, very low-density lipoprotein (VLDL) secretion, and gut absorption [2, 3]. Dysregulation of metabolism in any of these processes may cause the development of a fatty liver.

Metabolic disorders are not only regulated by the host itself but also influenced by gut microbiota. Growing evidence supports that gut microbiota play important roles in host lipid metabolism. The gut is linked to the liver through the gut–liver axis by which the gut transports gut microbiota-derived products directly to the liver and the liver feedback route of bile and antibody secretion to the gut [4]. Gut microbiota ferment indigestible carbohydrates and proteins, thereby yielding metabolites that play a crucial role in gut homeostasis and liver substrate metabolism and functions [5-7]. Dysregulation of gut microbiota influences the development of NAFLD/MAFLD, suggesting a potential causal role of gut microbiota in MAFLD development [8-10]. Therefore, a chicken lipid metabolism regulation model should be established systematically by combining host genetics and gut microbiota.

Compared with other animal models, studies on the regulatory mechanism of a fatty liver are more prevalent in humans, especially the population-based design. For farm animals including chickens, research on molecular regulation of a fatty liver is limited. Only a few studies have compared differences in mRNA, protein, and lncRNA expression and methylation in chickens with fatty liver syndrome [11-13]. Farm animals are more suitable to analyze the genetic and microbial involvement in a fatty liver by a population-based design. Farm animals have more genetic variations than mouse models, which are typically pure lines, and samples are more accessible than those from humans, such as obtaining microbiota from different gut segments in a large population. Chickens are patricianly suitable for large population analysis and can be used as model animals because of their strong reproductive ability and small individual economic value. Furthermore, a fatty liver is accompanied by a dramatic decrease in egg production and quality, and it is the main cause of non-infectious death of chickens [14, 15]. Hence, using chickens as a model to study molecular control of a fatty liver not only has high scientific value, but is also of great economic significance if a fatty liver can be reduced by molecular breeding.

In this study, we aimed to uncover the molecular regulatory mechanisms of hepatic steatosis. Host genome sequencing, liver transcriptome, proteome, and metabolome, and intestinal microbiome data were integrated to analyze the regulatory networks of host genetics, intestinal microbiota, and their interaction in hepatic steatosis. We generated a comprehensive and integrated host genetic–gut microbe interaction dataset that provides insight into the complex molecular control of hepatic steatosis, which would be beneficial to study human NAFLD by providing innovative ideas and methods for the application of multi-omics data to explore the genetic and microbial

regulatory mechanisms of important traits.

Results

Hepatic steatosis classification and phenotype characterization

A total of 686 adult hens were used for hepatic steatosis classification (HSC) in livers with hematoxylin and eosin (H&E)-stained whole sections, and one control (Con, N = 217, healthy) and two hepatic steatosis groups from mild (HS-Ⅰ, N = 265) to severe (HS-Ⅱ, N = 204) were classified in total (Fig. 1A). Hepatic triglyceride (HTG) and plasma triglyceride (PTG) contents increased with the severity of hepatic steatosis from 0.33 to 0.72 mmol/g in the liver (Con vs HS-Ⅱ, $P_{adj} < 0.05$, post-hoc Wilcoxon rank-sum test) and from 4.9 to 7.4 mmol/L in plasma (Con vs HS-Ⅱ, $P_{adj} < 0.05$, post-hoc Wilcoxon rank-sum test, Fig. 1B). Hepatic crude fat (HCF) and hepatic free fatty acids (HFFAs), plasma high-density lipoprotein (PHDL), low-density lipoprotein (PLDL) and very low-density lipoprotein (PVLDL) all displayed similar patterns ($P_{adj} < 0.05$, post-hoc Wilcoxon rank-sum test, Fig. 1C and D). However, hepatic total bile acid (HTBA) exhibited the opposite trend, which decreased from 16.16 to 12.19 mmol/g (Con vs HS-Ⅱ, $P_{adj} < 0.05$, post-hoc Wilcoxon rank-sum test, Fig. 1E). Hepatic total cholesterol did not change with a fatty liver, but plasma TC (PTC) increased significantly from 1.30 to 1.83 mmol/L (Con vs HS-Ⅱ, $P_{adj} < 0.05$, post-hoc Wilcoxon rank-sum test, Fig. 1F). With the severity of hepatic steatosis, the degree of obesity also increased with the abdominal fat weight (AFW) increasing from 106.6 to 145.8 g (Con

vs HS-Ⅱ, $P_{adj} < 0.05$, post-hoc Wilcoxon rank-sum test, Fig. 1G).

The abovementioned quantitative phenotypes mostly had positive correlations with HSC from 0.176 with HFFA to 0.426 with HCF ($P_{adj} < 0.05$, post-hoc Wilcoxon rank-sum test, Supplementary Figure S1). PTG had a relative high correlation coefficient with PHDL (0.86), PLDL (0.87), PVLDL (0.82), PTBA (−0.95), and PTC (0.89) ($P_{adj} < 0.05$, post-hoc Wilcoxon rank-sum test, Supplementary Figure S1). However, hepatic steatosis is in fact a quantitative trait, making artificial hepatic steatosis classification imprecise for intermediate individuals between each of two adjacent HSC groups. Hence, we further established an extreme hepatic steatosis classification (eHSC) model with more strict criteria, consisting of three groups: an eCon group (N = 30, without lipid droplets), eHS-Ⅰ group (N = 30, lipid droplets accounted for 30%−40% of HE-stained images), and eHS-Ⅱ (N = 30, lipid droplets accounted for > 90% of HE-stained images), which were verified by oil red O staining. Among eHSC groups, lipid-related indicators, such as HTG and HCF quantities, exhibited even greater differences; that is, two to three times higher in the eHS-Ⅱ group than in the eCon group (Fig. 1H, $P_{adj} < 0.01$, post-hoc Wilcoxon rank-sum test, other indicators see Supplementary Fig. S2). eHSC as a complementary model of HSC may aid in identifying crucial molecules for hepatic steatosis.

Genetic determinants of hepatic steatosis

Genomic variants

To investigate the influence of host genetics on hepatic steatosis, we performed whole genome resequencing of 686 chickens. Up to 1.94 Tb of clean reads were generated, and each individual reached an 8.13-fold depth and 95.06% genome coverage. After stringent filtering, a final set of 5,904,820 SNPs (6.17 SNPs per kb) was obtained (Supplementary Table S1). Estimation of SNP-based heritability (h^2) was performed on fat metabolism and storage-related traits. AFW had the highest h^2 (0.48), followed by HSC (0.25) and plasma HTG (0.20), indicating that host genetics had a substantial role in determination of fat accumulation and storage (Fig. 2A). Next, we performed GWA analysis of HSC with the abdominal fat percentage (AFP) as an additional covariate and identified two genomic peaks (GGA6: 5.59–5.69 Mb; GGA4: 75.6–76.4 Mb) that were significantly associated with HSC. Additionally, the P-values of the top SNPs rs731375960 at GGA6 (6:5594550) and rs739419162 at GGA4 (4:75758710) were all less than 3.32×10^{-7} (Fig. 2B, Supplementary Table S2). Individuals with different genotypes of top variants had significantly different HSC ratios ($P_{adj} < 0.05$, chi-squared test, Fig. 2C).

eVariants of hepatic steatosis

Genes that harbor or are near to genomic peaks are candidate genes for target traits, which is known as cis-regulation. However, in many cases, genomic variants do not regulate their physically adjacent genes, but genes far away and even in other chromosomes (trans-regulation). Thus, we used transcriptome data to further identify

genes (eGenes) regulated by these two genomic regions. After quality control, the clean data of 668 liver samples varied between 5.24 and 10.51 G for each individual, and the expression of 12,191 genes was quantified in total, of which 10,171 genes were annotated successfully. After eQTL mapping by tensorQTL, 7,468 genes (217 cis- and 7431 trans-regulated genes) were screened for their regulation by genomic variants (Supplementary Fig. S3). We then applied the summary data-based Mendelian randomization (SMR) method to GWAS summary datasets of HSC, and the SMR test showed that two genes (*NUDT14* and *EIF5A2*) were significantly associated with the genomic peak in GGA4 and three genes (*PEMT*, *TOM1L2*, and *GSTM3*) with their genomic peak in GGA6 ($P_{adj} < 0.05$, FDR correction for SMR, Supplementary Table S3). For verification, using the colocalization strategy, we performed thousands of fastGWA runs for all hepatic genes, and 4,171 genes (eGenes) were finally identified for expression that was significantly regulated by at least one genomic variant (eVariant, P-value of the top SNPs < 3.32×10^{-7}). Among these genes, two protein-coding genes, *PEMT* (4,792,705–4,830,291 bp) and *TOM1L2* (4,914,681–4,925,733 bp), at GGA 14 were identified again for their significant association with the genomic region on GGA6, 5.59–5.69 Mb, harboring the same variant rs731375960 that was significantly associated with HSC (Fig. 2B, $P < 3.32\times10^{-7}$). However, no significantly genes were identified for their association with the genomic region of GGA4, 75.6–76.4 Mb. Furthermore, among the genotypes of the top variant rs731375960, only expression of the *PEMT* gene differed significantly (post-hoc Wilcoxon rank-sum test, Fig. 2D).

Crucial genes for hepatic steatosis

To ascertain the relationship between gene expression and hepatic steatosis, Spearman’s rank-based correlation (SRC) analysis was performed between the expression of each gene and hepatic steatosis classifications. We identified 538 and 1797 genes that were significantly positively and negatively correlated with hepatic steatosis, respectively (P < 0.05), but the gene numbers decreased to 98 and 119, respectively, after FDR correction (P_{adj} < 0.05, FDR correction) (Supplementary Table S4), including trans-eGenes *PEMT* and *TOM1L2*. Considering that correlation analysis employs linear models, ANOVA analysis was also performed among three hepatic steatosis groups. Then, 98 significantly differentially expressed genes were screened (P_{adj} < 0.05, FDR correction), 83 of which (84.7%) were consistent with the genes identified by SRC (Fig. 2E). GO and pathway enrichment analyses indicated that these genes were mostly involved in the biological process of lipid localization and the biosynthetic process (Supplementary Fig. S4 and Table S5). Among 232 candidate genes, 48 were reported to be involved in hepatic steatosis, NAFLD, or hepatic lipid metabolism in humans and rats, indicating the reliability of our results (Table 1 and Supplementary Table S6). We further identified genomic variants (eVariants) that regulated the expression of these 48 genes in fastGWA datasets, and found that 20 genes were significantly regulated by at least one eVariant, all of which were trans-regulated (Table 1). In eHSC groups, 8,632 annotated genes were identified for their significantly differential expression (P_{adj} < 0.05, FDR correction, Supplementary Table S7), which

could be used as a DEG dataset to interpret molecular regulation of hepatic steatosis to some extent.

Multiple omics data reveal the molecular regulation mechanism for hepatic steatosis

To further ascertain the genetic mechanisms of hepatic steatosis, we complemented our molecular phenome coverage by profiling hepatic proteome and metabolome of individuals from eHSC groups (7 samples/group), and quantified 4,961 proteins and 1,005 metabolites in total (Supplementary Table S8 and S9). On the basis of the integrative analysis from genome to metabolome data, we summarized the regulation routes from three aspects (Fig. 3A): (1) Hepatic steatosis was characterized by the severe accumulation of free fatty acids (FFAs) and triglyceride (TG), and the decrease of phosphatidylcholine (PC) partially resulting from the low expression of *PEMT* that paly negative roles in TG output from hepatocytes; (2) The weak fatty acid β-oxidation activity reduced the utilization of FFA for energy supply; (3) The peroxidation of excessive FFAs aggravates the progression of hepatic steatosis.

Crucial role of *PEMT* in TG outward transport.

Metabolic profiles revealed that the quantity of HFFAs increased significantly (P_{adj} < 0.05, post-hoc Wilcoxon rank-sum test) with the severity of hepatic steatosis, which was verified by an ELISA (P_{adj} < 0.05, post-hoc Wilcoxon rank-sum test, Fig. 3B). This change in pattern also applied to HTG (P_{adj} < 0.05, post-hoc Wilcoxon rank-sum test, Fig. 1B). In general, the generated TGs are either stored in hepatic lipid droplets or

transported extra-hepatically via VLDLs, together with cholesterol and apolipoproteins. Because PC is by far the most abundant phospholipid component of all lipoprotein classes, impaired hepatic PC biosynthesis significantly reduces VLDL synthesis and secretion. Our results showed the PC content, phosphatidyl-ethanolamine (PE) and the PC/PE ratio significantly decreased with the severity of hepatic steatosis ($P_{adj} < 0.05$, post-hoc Wilcoxon rank-sum test, Fig. 3B). Endogenous PC is synthesized from PE by *PEMT*, which is encoded by a trans-eGene that shared the same regulatory genomic variants with hepatic steatosis classification in our GWA analysis. Correspondingly, the *APOB* gene, which encodes the primary apolipoprotein for VLDL, was also dramatically decreased by hepatic steatosis (13.4 times higher in the eCon group than eHS-II group, $P_{adj} < 0.05$, post-hoc Wilcoxon rank-sum test, Fig. 3C). The low expression of *PEMT* in the steatosis group may reduce endogenous production of PC. Furthermore, PC is generated from dietary choline, which involves three genes, choline kinase (*CHKA*), phosphate cytidylyltransferase 1 (*PCYT1A*), and choline phosphotransferase 1 (*CHPT1*). The expression of these genes was significantly higher in the control than the steatosis group (3–5 fold change in eHSC, $P_{adj} < 0.05$, post-hoc Wilcoxon rank-sum test, Fig. 3C). The expression of genes related to both endogenous and exogenous synthesis of PC was downregulated, leading to a significant decrease of PC in the steatosis group. However, the plasma VLDL concentration was 25% higher in the HS-II group than the control group (4.47 vs 3.57 μmol/L, $P_{adj} < 0.05$, post-hoc Wilcoxon rank-sum test, Fig. 1D). However, by considering liver weight (36.7 vs 46.3

g, 35% higher in HS-II than Con), the results became rational. Specifically, using liver weight as the covariate for AOV analysis of VLDL concentrations resulted in a p-value of 0.89 and the Con group had a slightly higher ratio of VLDL concentration/liver weight than the steatosis group (11% vs 9%, $P_{adj} > 0.05$, post-hoc Wilcoxon rank-sum test).

Weak activity of β-oxidation inhibits fatty acids (FAs) use

FAs, particularly long-chain fatty acids (LCFAs), can be used for energy supply by β-oxidation in mitochondria. LCFAs are first catalyzed by acyl-CoA synthetase (*ACSL5,4,3*) to form acyl-CoA, which are further synthesized to long-chain acylcarnitines (LCACs) by carnitine palmitoyltransferase 1A (*CPT1A*), a rate-limiting enzyme for β-oxidation. Our metabolome data revealed significantly high presence of LCACs in steatotic livers ($P_{adj} < 0.05$, post-hoc Wilcoxon rank-sum test, Fig. 3D), while short-chain acylcarnitines (SCACs) showed the opposite trend ($P_{adj} < 0.05$, post-hoc Wilcoxon rank-sum test, Fig. 3D). However, the rate-limiting enzyme *CPT1A* was almost not expressed in steatotic livers ($P_{adj} < 0.05$, post-hoc Wilcoxon rank-sum test, 67 times higher in eCon than eHS-Ⅱ). The severely high LCACs and extremely low expression of *CPT1A* in the HS group indicated suspension of β-oxidation activity, leading to accumulation of LCACs and low SCACs. Additionally, the other two crucial β-oxidation-related genes showed similar expression patterns, including *SLC25A20* (solute carrier family 25 member 20), which is responsible for the transport of LCACs

from cytosol to the mitochondrial matrix ($P_{adj} < 0.05$, post-hoc Wilcoxon rank-sum test, Fig. 3E), and *CPT2* (carnitine palmitoyltransferase 2), which catalyzes the opposite reaction with *CPT1A* from LCACs to acyl-CoA ($P_{adj} < 0.05$, post-hoc Wilcoxon rank-sum test, Fig. 3E).

Oxidative stress accompanied by steatosis

Oxidative stress is an imbalanced cellular state between the production of reactive oxygen species and normal free radical clearing mechanisms. Superoxide (O_2^-) is the precursor for most reactive oxygen species and can be converted to hydrogen peroxide (H_2O_2) and oxygen (O_2) by superoxide dismutase 1/2(SOD1/2), which was highly expressed in steatosis groups at the protein level ($P < 0.05$, Fig. 3F). However, the catalase (*CAT*) gene responsible for conversion of H_2O_2 to H_2O and O_2, and glutathione peroxidase 3 (*GPX3*) gene that catalyzes reduction of organic hydroperoxides and H_2O_2 by glutathione were significantly downregulated in steatotic livers ($P_{adj} < 0.05$, post-hoc Wilcoxon rank-sum test, Fig. 3F). Correspondingly, glutathione (GSH), as the most important antioxidant, was also extremely low in steatotic livers ($P_{adj} < 0.05$, post-hoc Wilcoxon rank-sum test, Fig. 3G). Finally, we measured the final products of lipid peroxidation, namely malondialdehyde (MDA) and 4-hydroxynonenal (HNE), by ELISAs, and found their high presence in steatotic livers ($P_{adj} < 0.05$, post-hoc Wilcoxon rank-sum test, Fig. 3G), verifying the high activity of lipid peroxidation. The metabolic data also revealed that oxidized lipids, such as 9- and 13-hydroxy-

octadecadienoic acid and 9- and 13-oxo-octadecadienoic acid converted from linoleic acid, were five to seven times higher in the steatosis group than in the control (P_{adj} < 0.05, post-hoc Wilcoxon rank-sum test, Fig. 3H), further illustrating the state of oxidative stress on FAs.

Microbiome signatures of hepatic steatosis and their association with host genetics

Fat metabolism has long been thought to be regulated by both genetics and gut microbiota. Hence, 16S rRNA gene sequencing was performed in the duodenum, jejunum, ileum, cecum, and feces of 705 chicken, resulting in 174.2 million quality-filtered sequences from 3,430 samples with an average of 49,497 reads (Supplementary Table S10). Then, 6,087 (duodenum), 5,987 (jejunum), 3,751 (ileum), 3,215 (cecum), and 7,428 (faces) ASVs were identified with 100% sequence identity in each gut segment.

Microbiability estimation

Analogous to heritability, the relative proportion of the total variance due to the gut microbial community is defined as microbiability (m^2), which allows estimation of the effect of microbiota as a whole on host traits. Hence, we first employed m^2 with ASV data to dissect the contributions of microbiota in each gut segment to hepatic steatosis-related phenotypes. The m^2 of HSC in the duodenum (0.26) was much higher than that in other anatomical sites (0.03 for the jejunum, 0.08 for ileum, 0.02 for cecum, and 0.07 for feces), suggesting more important roles of duodenal microbes in the

progression of hepatic steatosis (Supplementary Table S11 and Fig. 4A). Similar results were observed for FFA and TBA contents in the liver (0.15 and 0.19 for the duodenum respectively, and almost zero in other segments). However, for HCF and HTG contents, cecal microbiota (m^2 of 0.27 and 0.20 in cecum) played a more critical role than that in other segments (Fig. 4A). To validate the reliability of the estimated m^2, a permutation test was performed for the relatively high m^2 by randomly reordering the phenotypes 1,000 times. The results showed that the actual m^2 (0.15~0.27) was significantly higher than the simulated m^2 (average m^2 ranged from 0.02 to 0.04, $P < 0.05$, Supplementary Fig. S5A).

Identification of crucial microbes for hepatic steatosis

Using high-quality ASVs, 52 phyla, 161 classes, 467 orders, 1003 families, 2329 genera, and 3930 species were successfully classified (Supplementary Fig. S5B). The Shannon and Simpson Indices for alpha diversity revealed that the microbiota diversity decreased with steatosis progression ($P_{adj} < 0.05$, post-hoc Wilcoxon rank-sum test, Supplementary Fig. S5C). To evaluate the association between gut microbiota and hepatic steatosis, SRC analysis was performed between the abundance of microbial taxa in each gut segment and HSC. Consistent with the microbiability estimation, significantly associated taxa (72 in total) with HSC were almost observed in the duodenum, including six phyla, six classes, nine orders, 16 families, 14 genera, and 21 species ($P_{adj} < 0.05$, FDR correction, supplementary Table S12). For other gut segments,

only two taxa, the family Staphylococcaceae and genus *Staphylococcus* in the ileum, were significantly positively associated with HSC (P_{adj} < 0.05, FDR correction). Among these 72 taxa, four taxa chains from the phyla to genus level were identified for their association with HSC, including chains harboring the genera *Lactobacillus*, *Bacteroides*, *Sediminibacterium*, and *Cutibacterium* (Fig. 4B). Additionally, genera *Lactobacillus* and *Bacteroides* were verified by LEfSe analysis for their significantly differential presence among HSC groups (LDA = 4.7 and 3.8 respectively, P_{adj} < 0.01, Kruskal–Wallis test, Fig. 4C). Genus *Lactobacillus* was positively correlated with HSC (41.1% in Con, 47.5% and 52.0% in HS-Ⅰ and HS-Ⅱ, P_{adj} < 0.05), while genus *Bacteroides* showed the opposite trend (2.09% in Con, 1.70% and 1.55% in HS-Ⅰ and HS-Ⅱ, P_{adj} < 0.05, post-hoc Wilcoxon rank-sum test). Additionally, LEfSe analysis verified the differential presence of many other taxa (LDA > 3.0, P_{adj} < 0.01, Kruskal–Wallis test), including phyla Bacteroidetes, Firmicutes, and Actinobacteria, and classes Bacilli, Actinobacteria, and Bacteroidia, orders Bacteroidales and Chitinophagales, families Lactobacillaceae, Ruminococcaceae, Bacteroidaceae, Prevotellaceae, and Chitinophagaceae, genera *Lactobacillus* and *Bacteroides*, and species *Lactobacillus vaginalis*. The abovementioned microbial taxa had a high detection rate, many of which exhibited a high correlation with the phenotypes (Fig. 4D and Supplementary Fig. S6). Except for HSC, many microbes were negatively correlated with HTG and HCF levels, including genera *Prevotella7*, *Dialister*, *Ruminococcaceae UCG-014*, *Helicobacter*, *Butyricicoccus*, *Acinetobacter*, *Coprococcus 2*, *Dorea*, *Odoribacter*, *Parabacteroides*,

and *Faecalibaculum* (Fig. 4D, for other taxa levels, see Supplementary Fig. S6).

Lactobacillus might play compensatory roles in alleviating hepatic steatosis

By analyzing metabolome/transcriptome data from duodenum and liver samples, we found that the metabolites and genes related to the methionine cycle were significantly enriched for their differential presence/expression between healthy and hepatic steatosis groups (Fig. 5A). We hypothesized that highly abundant *Lactobacillus* in the duodenum of individuals with hepatic steatosis may participate in the host methionine cycle to alleviate their fatty liver status by producing extra folic acid (1.2 times higher in the duodenum of eHS-II group than the eCon group, Padj < 0.05, post-hoc Wilcoxon rank-sum test), which was verified by an ELISA (Fig. 5B). Methionine cycle-related genes, including methionine adenosyltransferase (*MAT*), *PEMT*, adenosylhomocysteinase (*AHCY*) and 5-methyltetrahydrofolate-homocysteine methyltransferase (*MTR*), were expressed at very low levels in hepatic steatosis individuals with 2.1–22.5 times higher expression in the eCon group than the eHS-II group (Fig. 5C, $P_{adj} < 0.05$, post-hoc Wilcoxon rank-sum test). Additionally, we found significant accumulation of S-adenosylmethionine (SAMe) and homocysteine (Hcy) in the livers of the eHS-II group compared with that in the eCon group by an ELISA (Fig. 5C). SAMe is converted to S-adenosylhomocysteine (SAH) by specific methyltransferases (MTs) such as GNMT, GAMT, and PEMT. However, only the *PEMT* gene was expressed in the liver in our study. Interestingly, PEMT also catalyzes

conversion of PE to PC, and its low expression leads to accumulation of SAMe and PE. Hcy can be remethylated to regenerate methionine by *MTR* (22.5 times higher in eCon than eHS-II) with conversion of methyl tetrahydrofolate (MTHF) to tetrahydrofolate (THF). Hence, additional folic acid is essential to prompt the final step of the methionine cycle. Although these results should be interpreted carefully, it can be hypothesized that the extra folate produced by duodenal microbiota may be absorbed by the host to reduce Hcy accumulation, resulting in comparable hepatic folic acid contents in healthy and hepatic steatosis groups (Fig. 5B).

Discussion

In this study, we used a chicken population consisting of 705 well-phenotyped individuals to investigate genetic variants, crucial hepatic molecules, and their interactions with intestinal microbiota in the development of hepatic steatosis. HSC and eHSC models were established to decipher the regulatory mechanism of hepatic steatosis. The HSC model allowed us to investigate genetic variants, eQTLs, and crucial genes for hepatic steatosis in the whole population with fewer but more accurate candidate genes or taxa being identified. However, hepatic steatosis is a quantitative trait, making artificial hepatic steatosis classification imprecise for intermediate individuals between each of two adjacent HSC groups. Hence, we further established the eHSC model, which used more stringent criteria for hepatic steatosis classification, leading to identification of more hepatic steatosis-related molecules because of the

smaller variance in each HSC group.

Through computational integration of individual genomes, hepatic transcriptomes, proteomes, metabolomes, and the gut microbiome, hepatic steatosis was found to be regulated by both host genetics and duodenal microbiota through various regulatory routes. The heritability (h^2) and duodenal microbiability (m^2) of HSC were comparable in this study (0.25 and 0.26, respectively), and explained the 51% phenotypic variance of hepatic steatosis. Many studies have estimated the heritability of hepatic steatosis or NAFLD in humans using a population-based design, and the values ranged from 0.20 to 0.34, which is comparable to our results [16-18]. However, to the best of our knowledge, no study has reported the m^2 of hepatic steatosis in humans or any animal model. Our study proposes that the crucial regulatory microbiota for hepatic steatosis mainly exist in the duodenum because of its much higher m^2 of HSC (0.26) than other gut segments (near zero).

Hepatic steatosis is generally characterized by accumulation of lipid droplets [19], which is supported by our observations that the quantities of HTG and HCF increased significantly with the severity of hepatic steatosis. However, our genetic analysis revealed that hepatic steatosis and fat accumulation did not share the same genetic determinants. The genomic region of GGA6, 5.59–5.69 Mb harboring the top SNP rs731375960 significantly influenced hepatic steatosis by regulating the expression of hepatic *PEMT* gene at GGA14 (Fig. 4B), but these variants had no effect on the quantity of HTG or HCF. We propose that fat accumulation should be viewed as a symptom of

hepatic steatosis rather than as its actual cause. SRC and ANOVA analysis of the HSC model identified 227 candidate genes for their association with hepatic steatosis, 48 of which have been reported to be involved in hepatic steatosis, NAFLD, or hepatic lipid metabolism in humans and mice. For example, *NAPEPLD* in hepatocytes is an important regulator of liver bioactive lipid synthesis. The hepatocyte-specific *Napepld* deletion mouse develops a high fat diet-like phenotype characterized by increased fat mass gain and hepatic steatosis [20]. *CYP1A1* metabolizes benzo[a]pyrene, resulting in either detoxication or metabolic activation in a context-dependent manner. Loss of the *CYP1A1* gene protects against non-alcoholic fatty liver disease caused by a Western diet containing benzo[a]pyrene in mice [21]. Interestingly, in contrast to previous studies of humans and cattle [22, 23], in which the majority of genes were cis-regulated by eVariants in most tissues, in this study, most genes expressed in the liver, including candidate genes for hepatic steatosis, were trans-regulated by genomic variants; that is, genes and their corresponding eVariants were on different chromosomes. This regulatory pattern was identified by both tensorQTL [24] and fastGWA [25] methods, indicating a unique gene regulation pattern in chickens, which requires additional study to determine whether this is the case in other species or tissues.

Combined with multi-omics data and the validation of crucial metabolites, we found abnormalities in three regulatory routes in host hepatocytes that contribute to hepatic steatosis. First, low expression of the *PEMT* gene led to a decrease in PC production, which further reduced VLDL synthesis and secretion, and ultimately led to

a decrease in hepatic lipid outward transport. Previous studies have demonstrated beneficial roles of PC in protecting against hepatic steatosis [26], and observed the clinical feature of a low PC/PE ratio in livers of NAFLD patients [27]. In this study, we not only found a decrease in endogenous PC production due to the *PEMT* gene, but also a dramatic decrease in the synthesis of exogenous PC from dietary choline, which further aggravated outward translocation of liver fat. Second, β-oxidation activity was very weak in mitochondria of HS individuals. A previous study has also demonstrated that lipid accumulation in the liver can be traced by impaired fatty acid β-oxidation [28]. Here, we found that the main genes in the β-oxidation pathway, including *ACSL5,4,3*, *CPT1A*, *SLC25A20*, and *CPT2*, were all expressed at extremely low levels in the HS-II group compared with the Con group. This resulted in significantly elevated and decreased levels of LCACs and SCACs, respectively, in the HS-II group, which may serve as biomarkers for hepatic steatosis.

Our results suggest that microbes in the duodenum, but not in other intestinal segments, are crucial regulators of hepatic steatosis. Indeed, the anterior part of the small intestines is the main site for fat digestion and absorption [29]. Previous studies to identify hepatic steatosis or NAFLD-related microbiota in humans or mouse models have mainly focused on fecal microbiota. However, fecal microbiota cannot represent the composition and abundance of microorganisms in each gut segment [30]. The connections between microbiota and the host were actually caused by metabolite-based signal transductions between individual microorganisms and gut epithelial cells.

Therefore, the contributing gut segment to different traits should first be identified.

In the duodenum, the abundance of genus *Lactobacillus* varies greatly among individuals of different hepatic steatosis grades. Genus *Lactobacillus*, which is commonly considered as probiotic bacteria, provides numerous health benefits to the host, including folate production [31], which was consistent with our results indicating that individuals with hepatic steatosis had a higher abundance of genus *Lactobacillus* and higher quantity of folate in their duodenum. Folate prompts the final step of the methionine cycle from Hcy to Met. Mice fed a methionine-choline-deficient diet develop non-alcoholic fatty liver disease with severe steatohepatitis [32], and addition of folate reduces the incidence of hepatic steatosis [33]. Moreover, SAMe and Hcy accumulation increases the incidence of fatty liver diseases [34], corroborating our results of higher Hcy abundances in individuals with hepatic steatosis. Although *Lactobacillus* has a compensatory effect on folate secretion, the expression of methionine cycle-related genes in individuals with hepatic steatosis was extremely low, which prevented normal activity of the methionine cycle and reduced PC production mediated by PEMT, leading to TG accumulation and hepatic steatosis.

Conclusion

We identified regulatory networks among genomic variants (GGA6: 5.59–5.69 Mb), gene expression (*PEMT* and *TOM1L2*), protein presence (PEMT), and metabolite abundance (SAMe, SAH, PC, PE, and Hcy) in hepatic steatosis and propose for the first

time that duodenal microbiota (m^2 = 0.21) plays more important roles in hepatic steatosis than other gut segments, and genus *Lactobacillus* in the duodenum might perform compensatory roles in alleviating hepatic steatosis by production of extra folate to prompt the host methionine cycle. This integrated analysis of host genomic variations, the hepatic transcriptome, proteome, metabolome, and gut microbiome provides a comprehensive understanding of the host genetic and gut microbial factors for hepatic steatosis and novel insights into mechanistic analysis of human NAFLD.

Material and Methods

Experimental Design

The experimental cohorts used in this study comprised a total of 705 hens from a pedigreed line of Rhode Island Red in Beijing Huadu Yukou Poultry Breeding Co., Ltd. (China). Birds were generated from two hatches and reared in individual cages under similar conditions. These birds were fed a basic diet and provided with free access to feed and water. No antibiotics were administered to the hens in our study. At 90 weeks of age, body weight was measured using an electronic scale to the nearest 5 g. Blood samples were collected from the wing vein and stored at −20 °C. Plasma was separated by centrifugation at 3,000 × *g* for 15 min and stored at −20 °C until use. Fecal samples were manually collected from the rectum with sterile cotton swabs. Each bird was then euthanized by cervical dislocation followed by decapitation. The contents of the duodenum, jejunum, ileum, and cecum, including the chyme and mucosa, were

immediately collected after opening the abdomen. All intestinal samples were dispensed into 2 ml tubes, snap frozen in liquid nitrogen, and stored at −80 °C.

The weights of the liver and abdominal fat tissue surrounding the gizzard, cloaca, and adjacent abdominal muscles were to the nearest 1 g with the electronic scale. Subsequently, some the liver tissue sample was frozen in liquid nitrogen and stored at −80 °C immediately after collection for genomic DNA, tissue RNA, protein, and metabolite extraction. Some of the liver was placed in a sterile plastic bag on dry ice using forceps and stored at −20 °C to measure biochemical indicators. The remaining liver sample was fixed in formalin for 48–72 h for histological observation.

All experiments involving animals were conducted according to the ethical policies and procedures approved by the Institutional Animal Care and Use Committee of China Agricultural University, China (Issue No.32303202-1-1)

Liver histological assessment

Liver histology was assessed in liver sections embedded in paraffin and stained with hematoxylin and eosin (H&E) using standard techniques. Whole section images of each liver sample were obtained using a Canon EOS 7D digital camera (Canon, Tokyo, Japan) and quantified using Image J (ver 1.8.0, National Institutes of Health, Bethesda, MD, USA). The investigators were blind to the group allocations. Therefore, a veterinary science pathologist performed the hepatic steatosis assessment using the NASH Clinical Research Network Scoring System in humans [35]. All liver samples were graded from 0 to 2, representing healthy individuals, and mild–moderate and

severe hepatic steatosis. For extremely typical individuals that could represent each group, oil red O staining was performed to verify lipid droplet accumulation.

Measurement of biochemical indicators in Plasma

Plasma triglycerides (PTGs), cholesterol (PTC), high-density lipoprotein cholesterol (PHDL), low-density lipoprotein cholesterol (PLDL), and total bile acids (PTBAs) were analyzed using commercial kits (Shanghai Kehua Bioengineering Co., Ltd., Shanghai, China) with the KHB ZY-1280 automatic biochemical analyzer (Shanghai Kehua Bioengineering Co., Ltd.). Plasma very low-density lipoprotein (PVLDL) was measured using a chicken very low-density lipoprotein ELISA kit (JLC10779) in accordance with the manufacturer's instructions (Shanghai Kehua Bioengineering Co., Ltd., Shanghai, China).

Measurement of biochemical indicators in the liver

Crude fat (CF) content measurement. The CF content was measured by the soxhlet extraction method (AOAC 920.85) and performed with a soxhlet apparatus by refluxing with petroleum ether to remove CF in the sample. The difference between the weights of the initial sample and residue was the CF content.

Protein quantitation. The livers were taken out of the −80°C freezer, temporarily stored in liquid nitrogen, trimmed into small pieces with scissors, and weighed ranging from 0.01g–0.05g on an electronic scale. Protein quantification was performed using a

protein quantification kit (A045-2) from Nanjing Jiancheng Institute of Biological Engineering Ltd. according to the manufacturer's instructions.

Hepatic biochemical indicator assay. On the basis of protein quantification, hepatic triglycerides (HTG), total cholesterol (HTC), free fatty acids (HFFAs), and total bile acids (HTBAs) were measured using the Triglyceride Assay Kit (A110-2-1) from Nanjing Jiancheng Institute of Biological Engineering Co., Ltd. according to the manufacturer's instructions.

Whole-genome resequencing and data processing

Genomic DNA was isolated from liver samples of 705 hens using a Tiangen DNA Extraction Kit (Tiangen Biotech, Beijing, China, DP304-2) according to the manufacturer's instructions. After purification and integrity verification of the DNA, a total of 686 DNA samples were used for subsequent whole-genome resequencing. Host DNAs were amplified using PCR with 500 bp inserts for library construction. Whole-genome resequencing was performed using the Illumina HiSeq 2500 Sequencer (Illumina, Inc., San Diego, CA, USA) to generate 150 bp paired-end reads. To ensure the quality of data, the adaptor-polluted reads, low-quality reads and reads with number of N bases accounting for more than 5 % were removed. The clean reads were then mapped to the chicken reference genome (GRCg6a) using the Burrows–Wheeler aligner (BWA, ver 0.7.15) [36] with the default parameters. We subsequently used Samtools (ver 1.3.1) [37] to sort reads and remove low quality reads with the parameter "-q 4". Duplicate reads resulting from PCR were removed using Picard tools

(http://broadinstitute.github.io/picard/). The HaplotypeCaller protocol in Genome Analysis Toolkit (GATK, ver 4.2.0.0) [38] was used for SNPs and indels calling. To obtain high-quality SNPs, the SNPs were filtered with GATK VariantFiltration protocol as follows: QD < 2.0, ReadPosRankSum < −8.0, FS > 60.0, QUAL < 30.0, DP < 4.0, MQ < 40.0, MappingQualityRankSum < −12.5 and INDEL: QD < 2.0, ReadPosRankSum < −20.0, FS > 200.0, QUAL < 30.0, DP < 4.0. Finally, the PLINK (ver 1.90) [39] was used for filtering annotated SNP data with the following parameters: sample call rate > 90%, SNP call rate > 90% and minor allele frequencies > 1%. The remaining SNPs and individuals were used for imputation in BEAGLE (ver 5.1) [40], and the PLINK analysis was reperformed using the same criteria as above-described. After these steps, a total of 5,904,820 SNPs distributed across 32 chromosomes and 686 birds were retained for subsequent analysis.

Genome-wide analysis study (GWAS)

To reveal the impact of host genetics on shaping the phenotypes, all valid individuals and SNPs were involved in GWAS with a univariate linear mixed model (LMM), which were performed using GEMMA (ver 0.98.4) [41]. The statistical model applied in this study is as follows:

$$y = W\alpha + x\beta + u + \varepsilon$$

where y is the phenotypic values of 686 individuals; W is a matrix of covariates (fixed effects: top five principal components and batch effects) controlling for population structure, while α refers to a vector of corresponding effects that compose

the intercept; x denotes the marker genotypes, while β is the corresponding marker's effect; u is a vector of random polygenic effects with a covariance structure; and ε is vector of random residuals.

The likelihood ratio test *P*-value was selected as a criterion for examining the significance of the association between SNPs and phenotypes. The genome-wide significant threshold was determined using a modified Bonferroni correction with an R package named simpleM as previously described [42]. Using this approach, a total of 150,802 valid inspections were obtained, and thereby the genome-wide significance and suggestive significance thresholds were defined as 3.32×10^{-7} (0.05/150802) and 6.63×10^{-6} (1/150802), respectively.

For the sake of exploring the effects of host genetics on the gene expression in liver tissue, GWAS were conducted in Genome-Wide Complex Trait Analysis (GCTA) software (ver 1.93.2) [43] with the support of fastGWA [25]. Out of 686 samples, 668 transcriptome-sequenced subjects were included for subsequent genome and gene expression association analysis in our study. Read counts were normalized using TPM initially. Genes were selected based on the expression thresholds of ≥ 0.1 TPM and ≥ 6 reads (unnormalized) in ≥ 20% samples. Afterwards, read counts were normalized between samples using TMM. Eventually, 12,191 genes were remained in this part. Likewise, a LMM was employed throughout the analysis. For fastGWA, a full-dense genetic relationship matrix (GRM) was generated, based on which a sparse GRM was built at a cutoff value of 0.05. fastGWA was then run using this sparse GRM with

expression level as the dependent variable and SNP genotype values as the independent variable. Significant and suggestively significant *P*-value thresholds were 3.32×10^{-7} and 6.63×10^{-6} as described above.

eQTL mapping and mendelian randomization analysis

For each gene, we took all genetic variants into consideration, and used the following covariates: top 5 genetic principal components, batch effects and top 3 PEER factors. The number of PEER factors included in calculation equaling to 60 was determined from the sample size corresponding to the previously reported researches: 15 for $n < 150$, 30 for $150 \leq n < 250$, 45 for $250 \leq n < 350$, 60 for $n \geq 350$ [44]. Thereafter, the permutations of cis-QTL mapping were conducted to generate phenotype-level summary statistics with empirical *P*-value, and trans-QTL mapping to compute nominal associations between all phenotypes and genotypes. Notably, the cis-window referred to ranging from 1Mbps upstream to 1Mbps downstream of the transcription start sites (TSS), while for trans-QTL mapping, 5,315,471 common genetic variants passing strict quality control criteria (MAF > 5% and outside of TSS ± 5 Mbps) were contained in the process. Correction for multiple testing was done using FDR for cis-eQTL analysis, resulting in a *P*-value threshold of 8.05×10^{-6} for cis-eQTLs. Given that the remaining SNPs used in trans-QTL mapping was analogous to those in GWAS, we considered its *P*-value threshold as 3.32×10^{-7}.

Subsequently, top-eQTL based Summary-data-based Mendelian Randomization (SMR) [45] analysis was performed to prioritize genes underlying GWAS associations.

The SMR procedure consists of two steps: i) identification of variants independently associated with the exposure factor, and ii) calculation of causal estimates. Before that, we made a BESD file and updated coordinates of SNPs and genes, frequency of effect allele. For each GWAS summary statistic, SNPs significantly and suggestively significantly associated with the traits were selected as SMR input files to determine the connection with significant cis-eQTLs and trans-eQTLs. Finally, we selected only variants that showed association at FDR corrected P-value < 0.05.

16S rRNA gene sequencing

The gut digesta (duodenum, jejunum, ileum and cecum) and fecal samples of 705 individuals were thawed on ice and homogenized, and ~200 mg of each sample was used to extract the microbial genome DNA using the QIAamp Stool Mini Kit (QIAGEN, Hilden, Germany, D4015-01) according to the manufacturer's recommendations. The hypervariable V4 region of the 16S gene was amplified using the Ion Plus Fragment Library Kit 48 rxns (Thermo Scientific). Sequencing was performed on an Ion S5TM XL platform, and 400 bp single-end reads were generated, in accordance with the manufacturer's instructions. Sequences were imported and processed using Quantitative Insights Into Microbial Ecology (QIIME2, ver 2019.10) [46] for further bioinformatics analyses. After trimming the barcode and primer sequences, the preliminary quality screening was performed for the original high-throughput sequencing data using the QIIME2 plugin DADA2 [47] and the sequences were trimmed to a final length of 252 bp. The remaining high-quality sequences were

clustered and classified by amplicon sequence variants (ASVs) with 100% identity [48]. ASVs that presented in less than 1% (seven) samples and had an average relative abundance below 10^{-6} were removed for subsequent analyses. Taxonomic assignments for each ASV were made via similarity searching against the SILVA 16S rRNA gene sequence reference database (Release 132) [49]. The alpha and beta diversity were calculated with the vegan package [50].

Heritability and microbiability estimation

The 5,904,820 filtered SNPs were used to construct genetic relatedness matrix (GRM) using GCTA software (ver 1.93.2) [43]: The GRM estimation model used was:

$$g_{ij} = \frac{1}{N}\sum_{V=1}^{N}\frac{(Xiv-2\overline{pv})(xjv-2\overline{pv})}{2\overline{pv}(1-\overline{pv})}$$

In this expression, g_{ij} denotes the genetic relationship between individuals i and j; x_{iv} and x_{jv} denote the number of reference alleles in hens i and j, respectively; p_v denotes the reference allele frequency; and N is the SNP number. The SNP-based heritability of the host phenotypes was estimated with the following model:

$$Y = Kc + g + e$$

In this expression, y denotes a vector of the phenotype; c denotes a vector of fixed covariates (including batch effect and the first ten host genetic principal components); K denotes the corresponding matrix for c; and g denotes a vector of the total effects of all SNPs with $\sim N(0, G\sigma^2 A)$, where G and $G\sigma^2 A$ denote the GRM and genetic variance, respectively; and e denotes the residual effect.

The phenotypic variance explained by gut microbial variance is defined as

microbiability (m^2) in animals [51, 52] and it was estimated with GCTA software using the microbial relationship matrix (MRM). The construction of the microbial relationship matrix and phenotypic variance explained by the gut microbial variance were estimated as described in our previous study [53]. We corrected batch effects and the first five host genetic principal components in this analysis.

Liver tissue transcriptome

A total of 686 samples were used for transcriptome sequencing. The Eastep ® Super Total RNA Extraction Kit (Promega, Shanghai, China, LS1040) was used to extract total RNA according to the manufacturer's instructions. The RNA concentration and purity were determined using the NanoDrop ND-2000 spectrophotometer (Thermo Fisher Scientific, Waltham, MA, USA). The integrity of the RNA as assessed using the RNA Nano 6000 Assay Kit of the Bioanalyzer 2100 system (Agilent Technologies, CA, USA). Libraries for transcriptome sequencing were constructed following the standard Illumina RNA-seq instruction. The libraries were sequenced on an Illumina Novaseq platform and 150 bp paired-end reads were generated. Fastp (ver 0.20.1) [54] was used to remove the reads containing adaptor contamination, low quality bases and undetermined bases. Then, the quality-controlled sequencing data were aligned to the chicken reference genome (GRCg6a) using HISAT2 (ver 2.0.5) [55] with default parameters. After that, we employed featureCounts (ver 1.6.3) [56] to count the reads for each gene. The differentially expressed genes between different groups were identified with the assistance of DESeq2 (ver 3.16) [57]. The significance threshold for

the differential expression was adjusted *P*-value < 0.05 and a |log2 fold change | > 1.

Liver tissue proteome

For protein extraction and digestion, chicken liver tissues (6 samples/group from eHSC groups) were ground with liquid nitrogen into cell powder and transferred to a 5-mL centrifuge tube. The protein concentration was determined with BCA kit (Thermo Fisher Scientific, 23225) according to the manufacturer's instructions and protein digestion was conducted just as Song et al. illustrated [58]. Subsequent TMT labeling, HPLC Fractionation and LC-MS/MS analysis of the TMT- labeled peptides were performed as previously described, too [58]. Automatic gain control (AGC) target was set at 1×10^{-5}, with an intensity threshold of 3.3×10^{-4} and a maximum injection time of 60 ms.

Downstream database search was performed using MaxQuant search engine (v.1.6.15.0). Tandem mass spectra were searched against the *Gallus gallus* database (27535 entries) concatenated with reverse decoy database. Trypsin/P was specified as cleavage enzyme allowing up to 2 missing cleavages. The mass tolerance for precursor ions was set as 20 ppm in first search and 5 ppm in main search, and for fragment ions was set as 0.02 Da, respectively. Carbamidomethyl on Cys was specified as fixed modification, and acetylation on protein N-terminal and oxidation on Met were specified as variable modifications. The threshold of FDR adjusted *P*-value was set to 0.01.

Widely targeted metabolome

Livers were thawed in a 50 μL ice-cold mixture (Methanol : Water = 7:3, V/V) and homogenized after adding a 150 μL solution (Methanol : Water = 7:3, V/V) containing internal standard. Placed the sample on ice for 15 min, and centrifuged it at 12000 rpm for 10 min (4 °C). The collected supernatant was placed in −20 °C for 30 min and then centrifuged at 12000 rpm for 3 min (4 °C), followed by transferring about 120 μL aliquots of supernatant. The solution obtained was analyzed using an LC-ESI-MS/MS system (UPLC, ExionLC AD, https://sciex.com.cn/; MS, QTRAP® System, https://sciex.com/). The chromatographic separation was achieved by using water and acetonitrile (with 0.1% formic acid for each) as the mobile phase. The elution gradient program was 95:5 V/V at 0 min, 10:90 V/V at 11.0 min, 10:90 V/V at 12.0 min, 95:5 V/V at 12.1 min, 95:5 V/V at 14.0 min with the 0.40 mL/min of flow rate. The column temperature was 40°C, and the injection volume was 2 μL.

LIT and triple quadrupole scans were acquired on a triple quadrupole-linear ion trap mass spectrometer (QTRAP), QTRAP® LC-MS/MS System, equipped with an ESI Turbo Ion-Spray interface, operating in positive and negative ion mode and controlled by Analyst 1.6.3 software (Sciex). The ESI source operation parameters and successor operations followed Chen's parameters [59].

Significantly regulated metabolites between groups were determined by VIP (VIP ≥ 1), *P*-value (*P*-value < 0.05) and absolute log2FC (|log2FC| ≥ 1.0). VIP values were generated from OPLS-DA result using R package MetaboAnalystR. The data was log

transform (log2) and mean centering before OPLS-DA. In order to avoid overfitting, a permutation test (200 permutations) was performed.

Liver tissue and duodenal mucosa assays

To validate the results of the metabolome, we randomly chose 10 individuals in each group (eCon, eHS-I, and eHS-II), and the levels of folic acid (FLA), homocysteine (Hcy), methionine (Met), S-adenosine homocysteine (SAH), and S-adenosylmethionine (SAM) were determined in liver tissue and duodenal mucosa samples by ELISA kits (Enzyme-linked Biotechnology Co. Ltd., Shanghai, China) in accordance with the manufacturer's instructions.

Statistical Analysis

Differences in phenotypes among HSC groups were determined in R version 4.0.2 [60] using the Wilcoxon rank sum test with a post-hoc test to correct for multiple comparisons. Analysis of variance (ANOVA) was performed to determine differences in gut microbiota, liver gene expression (TPM), and biochemical indicators among HSC groups with FDR correction for multiple testing.

Spearman's rank coefficient of correlations was calculated between the relative abundance of duodenal microbiota of various taxonomic levels ranging from phylum to species and HSC. Prior to this, the relative abundance of the microorganisms that presented in $\geq$ 60% of the population equaling to 0 was converted to NA, because it was considered to escape from detection. Otherwise, microorganisms detected in < 60%

and ≥ 30% of samples were dichotomized into presence/absence patterns and we encoded the phenotype as a binary vector to prevent zero inflation, which led to a bimodal distribution. Microorganisms detected in < 30% of samples were excluded from this analysis as reported previously [53, 61].

The relationship between hepatic biochemical indicators and the relative abundance of bacteria with a call rate ranging from 30% to 60% was measured using the Spearman correlation, whereas the Pearson correlation coefficient was employed to test the strength of the association between hepatic biochemical indicators and the relative abundance of bacteria in ≥ 60% of samples. Correlations between the TPM value and HSC were also calculated using the Spearman method. FDR corrections were carried out in all of the abovementioned analysis.

Declarations

Ethics approval and consent to participate

All experiments involving animals were conducted according to the ethical policies and procedures approved by the Institutional Animal Care and Use Committee of China Agricultural University, China (Issue No.32303202-1-1)

Availability of data and materials

Whole-genome resequencing data are submitting. RNA-Seq data are available on the NCBI Sequence Read Archive (SRA) under accession SUB13062074. 16S rRNA

sequencing data can be accessed on SRA under the accession SUB12033010 (duodenum), SUB12035295 (jejunum), SUB12035349 (ileum), SUB12035378 (cecum) and SUB12035409 (feces). Raw data for metabolomics were submitted to MetaboLights at MTBLS7808. And raw data for proteomics are still submitting.

Competing interests

The authors declare no competing interests.

Funding

This work was supported by the National Natural Science Foundation of China (No. 31930105), National Key Research and Development Program of China (2022YFF1000204), China Agriculture Research Systems [CARS-40] and the 2115 Talent Development Program of China Agricultural University.

Authors' contributions

NY and CS conceived the study, participated in the experiment design and critical discussion, and jointly supervised this work. CS, FL, QZ and XG performed the experiments and wrote the manuscript. CS, QZ, FL, CW, YG, ZH, JZ, GW, GL, YY, JL and QM participated in the management of the experimental animals and the sample collection. QZ, FL and JJ contributed to the measurement of biochemical traits, ELISA and qPCR analysis. CS, FL, QZ and XG conducted bioinformatics and statistical analysis. NY, CS and FL designed the figures and tables. NY and CS were responsible

for critical revisions of the manuscript drafts. All authors read and approved the final manuscript.

Acknowledgements

We are grateful to Mr. Hao Sun and Huadu Yukou Poultry Co., Ltd. for the assistance in providing experimental chickens.

Reference

1. Eslam M, Sanyal AJ, George J and Panel IC. MAFLD: A Consensus-Driven Proposed Nomenclature for Metabolic Associated Fatty Liver Disease. Gastroenterology. 2020;158 7:1999-+.
2. Kim H, Lee DS, An TH, Park HJ, Kim WK, Bae KH, et al. Metabolic Spectrum of Liver Failure in Type 2 Diabetes and Obesity: From NAFLD to NASH to HCC. Int J Mol Sci. 2021;22 9 doi:10.3390/ijms22094495.
3. Ramos LF, Silva CM, Pansa CC and Moraes KCM. Non-alcoholic fatty liver disease: molecular and cellular interplays of the lipid metabolism in a steatotic liver. Expert Rev Gastroent. 2021;15 1:25-40. doi:10.1080/17474124.2020.1820321.
4. Albillos A, de Gottardi A and Rescigno M. The gut-liver axis in liver disease: Pathophysiological basis for therapy. J Hepatol. 2020;72 3:558-77. doi:10.1016/j.jhep.2019.10.003.
5. Canfora EE, Meex RCR, Venema K and Blaak EE. Gut microbial metabolites in obesity, NAFLD and T2DM. Nat Rev Endocrinol. 2019;15 5:261-73. doi:10.1038/s41574-019-0156-z.
6. Dodd D, Spitzer MH, Van Treuren W, Merrill BD, Hryckowian AJ, Higginbottom SK, et al. A gut bacterial pathway metabolizes aromatic amino acids into nine circulating metabolites. Nature. 2017;551 7682:648-+. doi:10.1038/nature24661.
7. Zhao LP, Zhang F, Ding XY, Wu GJ, Lam YY, Wang XJ, et al. Gut bacteria selectively promoted by dietary fibers alleviate type 2 diabetes. Science. 2018;359 6380:1151-+. doi:10.1126/science.aao5774.
8. Hoyles L, Fernandez-Real JM, Federici M, Serino M, Abbott J, Charpentier J, et al. Molecular phenomics and metagenomics of hepatic steatosis in non-diabetic obese women (vol 24, pg 1070, 2018). Nat Med. 2018;24 10:1628-. doi:10.1038/s41591-018-0169-5.
9. Yao N, Yang YX, Li XT, Wang YX, Guo RR, Wang XH, et al. Effects of Dietary Nutrients on Fatty Liver Disease Associated With Metabolic Dysfunction (MAFLD): Based on the Intestinal-Hepatic Axis. Front Nutr. 2022;9 doi: 10.3389/fnut.2022.906511.
10. Zhao MM, Zhao L, Xiong XL, He Y, Huang W, Liu ZH, et al. TMAVA, a Metabolite of Intestinal Microbes, Is Increased in Plasma From Patients With Liver Steatosis, Inhibits gamma-

Butyrobetaine Hydroxylase, and Exacerbates Fatty Liver in Mice. Gastroenterology. 2020;158 8:2266-+. doi:10.1053/j.gastro.2020.02.033.

11. Tan XD, Liu RR, Xing SY, Zhang YH, Li QH, Zheng MQ, et al. Genome-Wide Detection of Key Genes and Epigenetic Markers for Chicken Fatty Liver. Int J Mol Sci. 2020;21 5 doi: 10.3390/ijms21051800.
12. Tan XD, Liu RR, Zhang YH, Wang XC, Wang J, Wang HL, et al. Integrated analysis of the methylome and transcriptome of chickens with fatty liver hemorrhagic syndrome. Bmc Genomics. 2021;22 1 doi: 10.1186/s12864-020-07305-3.
13. Tsai MT, Chen YJ, Chen CY, Tsai MH, Han CL, Chen YJ, et al. Identification of Potential Plasma Biomarkers for Nonalcoholic Fatty Liver Disease by Integrating Transcriptomics and Proteomics in Laying Hens. J Nutr. 2017;147 3:293-303. doi:10.3945/jn.116.240358.
14. Mete A, Giannitti F, Barr B, Woods L and Anderson M. Causes of Mortality in Backyard Chickens in Northern California: 2007-2011. Avian Dis. 2013;57 2:311-5. doi:DOI 10.1637/10382-092312-Case.1.
15. Trott KA, Giannitti F, Rimoldi G, Hill A, Woods L, Barr B, et al. Fatty liver hemorrhagic syndrome in the backyard chicken: a retrospective histopathologic case series. Vet Pathol. 2014;51 4:787-95. doi:10.1177/0300985813503569.
16. Palmer ND, Musani SK, Yerges-Armstrong LM, Feitosa MF, Bielak LF, Hernaez R, et al. Characterization of European Ancestry Nonalcoholic Fatty Liver Disease-Associated Variants in Individuals of African and Hispanic Descent. Hepatology. 2013;58 3:966-75. doi:10.1002/hep.26440.
17. Speliotes EK, Yerges-Armstrong LM, Wu J, Hernaez R, Kim LJ, Palmer CD, et al. Genome-Wide Association Analysis Identifies Variants Associated with Nonalcoholic Fatty Liver Disease That Have Distinct Effects on Metabolic Traits. Plos Genet. 2011;7 3 doi: 10.1371/journal.pgen.1001324.
18. Wagenknecht LE, Scherzinger AL, Stamm ER, Hanley AJG, Norris JM, Chen YDI, et al. Correlates and Heritability of Nonalcoholic Fatty Liver Disease in a Minority Cohort. Obesity. 2009;17 6:1240-6. doi:10.1038/oby.2009.4.
19. Liu Q, Bengmark S and Qu S. The role of hepatic fat accumulation in pathogenesis of non-alcoholic fatty liver disease (NAFLD). Lipids Health Dis. 2010;9 doi: 10.1186/1476-511x-9-42.
20. Lefort C, Roumain M, Van Hul M, Rastelli M, Manco R, Leclercq I, et al. Hepatic NAPE-PLD Is a Key Regulator of Liver Lipid Metabolism. Cells-Basel. 2020;9 5 doi:10.3390/cells9051247.
21. Uno S, Nebert DW and Makishima M. Cytochrome P450 1A1 (CYP1A1) protects against nonalcoholic fatty liver disease caused by Western diet containing benzo[a]pyrene in mice. Food Chem Toxicol. 2018;113:73-82. doi:10.1016/j.fct.2018.01.029.
22. Ardlie KG, DeLuca DS, Segre AV, Sullivan TJ, Young TR, Gelfand ET, et al. The Genotype-Tissue Expression (GTEx) pilot analysis: Multitissue gene regulation in humans. Science. 2015;348 6235:648-60. doi:10.1126/science.1262110.
23. Liu S, Gao Y, Canela-Xandri O, Wang S, Yu Y, Cai W, et al. A comprehensive catalogue of regulatory variants in the cattle transcriptome. bioRxiv. 2020:2020.12.01.406280. doi:10.1101/2020.12.01.406280.
24. Taylor-Weiner A, Aguet F, Haradhvala NJ, Gosai S, Anand S, Kim J, et al. Scaling computational genomics to millions of individuals with GPUs. Genome Biol. 2019;20 1:228. doi:10.1186/s13059-019-1836-7.

25. Jiang LD, Zheng ZL, Qi T, Kemper KE, Wray NR, Visscher PM, et al. A resource-efficient tool for mixed model association analysis of large-scale data. Nat Genet. 2019;51 12:1749-+. doi:10.1038/s41588-019-0530-8.
26. Niebergall LJ, Jacobs RL, Chaba T and Vance DE. Phosphatidylcholine protects against steatosis in mice but not non-alcoholic steatohepatitis. Biochim Biophys Acta. 2011;1811 12:1177-85. doi:10.1016/j.bbalip.2011.06.021.
27. Arendt BM, Ma DWL, Simons B, Noureldin SA, Therapondos G, Guindi M, et al. Nonalcoholic fatty liver disease is associated with lower hepatic and erythrocyte ratios of phosphatidylcholine to phosphatidylethanolamine. Appl Physiol Nutr Me. 2013;38 3:334-40. doi:10.1139/apnm-2012-0261.
28. Koo SH. Nonalcoholic fatty liver disease: molecular mechanisms for the hepatic steatosis. Clin Mol Hepatol. 2013;19 3:210-5. doi:10.3350/cmh.2013.19.3.210.
29. Feinle C, Rades T, Otto B and Fried M. Fat digestion modulates gastrointestinal sensations induced by gastric distention and duodenal lipid in humans. Gastroenterology. 2001;120 5:1100-7. doi:10.1053/gast.2001.23232.
30. Yan W, Sun CJ, Zheng JX, Wen CL, Ji CL, Zhang DX, et al. Efficacy of Fecal Sampling as a Gut Proxy in the Study of Chicken Gut Microbiota. Front Microbiol. 2019;10 doi: 10.3389/fmicb.2019.02126.
31. Rossi M, Amaretti A and Raimondi S. Folate production by probiotic bacteria. Nutrients. 2011;3 1:118-34. doi:10.3390/nu3010118.
32. Itagaki H, Shimizu K, Morikawa S, Ogawa K and Ezaki T. Morphological and functional characterization of non-alcoholic fatty liver disease induced by a methionine-choline-deficient diet in C57BL/6 mice. Int J Clin Exp Pathol. 2013;6 12:2683-96.
33. da Silva RP, Kelly KB, Al Rajabi A and Jacobs RL. Novel insights on interactions between folate and lipid metabolism. Biofactors. 2014;40 3:277-83. doi:10.1002/biof.1154.
34. Mato JM, Martinez-Chantar ML and Lu SC. Methionine metabolism and liver disease. Annu Rev Nutr. 2008;28:273-93. doi:10.1146/annurev.nutr.28.061807.155438.
35. Kleiner DE, Brunt EM, Van Natta M, Behling C, Contos MJ, Cummings OW, et al. Design and validation of a histological scoring system for nonalcoholic fatty liver disease. Hepatology. 2005;41 6:1313-21. doi:10.1002/hep.20701.
36. Li H and Durbin R. Fast and accurate short read alignment with Burrows-Wheeler transform. Bioinformatics. 2009;25 14:1754-60. doi:10.1093/bioinformatics/btp324.
37. Li H, Handsaker B, Wysoker A, Fennell T, Ruan J, Homer N, et al. The Sequence Alignment/Map format and SAMtools. Bioinformatics. 2009;25 16:2078-9. doi:10.1093/bioinformatics/btp352.
38. McKenna A, Hanna M, Banks E, Sivachenko A, Cibulskis K, Kernytsky A, et al. The Genome Analysis Toolkit: A MapReduce framework for analyzing next-generation DNA sequencing data. Genome Res. 2010;20 9:1297-303. doi:10.1101/gr.107524.110.
39. Purcell S, Neale B, Todd-Brown K, Thomas L, Ferreira MAR, Bender D, et al. PLINK: A tool set for whole-genome association and population-based linkage analyses. Am J Hum Genet. 2007;81 3:559-75. doi:10.1086/519795.
40. Browning SR and Browning BL. Rapid and accurate haplotype phasing and missing-data inference for whole-genome association studies by use of localized haplotype clustering. Am J Hum Genet. 2007;81 5:1084-97. doi:10.1086/521987.

41. Zhou X and Stephens M. Genome-wide efficient mixed-model analysis for association studies. Nat Genet. 2012;44 7:821-U136. doi:10.1038/ng.2310.
42. Gao XY, Stamier J and Martin ER. A multiple testing correction method for genetic association studies using correlated single nucleotide polymorphisms. Genet Epidemiol. 2008;32 4:361-9. doi:10.1002/gepi.20310.
43. Yang JA, Lee SH, Goddard ME and Visscher PM. GCTA: A Tool for Genome-wide Complex Trait Analysis. Am J Hum Genet. 2011;88 1:76-82. doi:10.1016/j.ajhg.2010.11.011.
44. Barbeira AN, Bonazzola R, Gamazon ER, Liang YY, Park Y, Kim-Hellmuth S, et al. Exploiting the GTEx resources to decipher the mechanisms at GWAS loci. Genome Biol. 2021;22 1 doi: 10.1186/s13059-020-02252-4.
45. Zhu ZH, Zhang FT, Hu H, Bakshi A, Robinson MR, Powell JE, et al. Integration of summary data from GWAS and eQTL studies predicts complex trait gene targets. Nat Genet. 2016;48 5:481-+. doi:10.1038/ng.3538.
46. Bolyen E, Rideout JR, Dillon MR, Bokulich NA, Abnet CC, Al-Ghalith GA, et al. Reproducible, interactive, scalable and extensible microbiome data science using QIIME 2 (vol 37, pg 852, 2019). Nat Biotechnol. 2019;37 9:1091-. doi:10.1038/s41587-019-0252-6.
47. Callahan BJ, McMurdie PJ, Rosen MJ, Han AW, Johnson AJA and Holmes SP. DADA2: High-resolution sample inference from Illumina amplicon data. Nat Methods. 2016;13 7:581-+. doi:10.1038/Nmeth.3869.
48. Callahan BJ, McMurdie PJ and Holmes SP. Exact sequence variants should replace operational taxonomic units in marker-gene data analysis. The ISME journal. 2017;11 12:2639-43.
49. Quast C, Pruesse E, Yilmaz P, Gerken J, Schweer T, Yarza P, et al. The SILVA ribosomal RNA gene database project: improved data processing and web-based tools. Nucleic Acids Res. 2013;41 D1:D590-D6. doi:10.1093/nar/gks1219.
50. Dixon P. VEGAN, a package of R functions for community ecology. J Veg Sci. 2003;14 6:927-30. doi:DOI 10.1111/j.1654-1103.2003.tb02228.x.
51. Camarinha-Silva A, Maushammer M, Wellmann R, Vital M, Preuss S and Bennewitz J. Host Genome Influence on Gut Microbial Composition and Microbial Prediction of Complex Traits in Pigs. Genetics. 2017;206 3:1637-44. doi:10.1534/genetics.117.200782.
52. Difford GF, Plichta DR, Lovendahl P, Lassen J, Noel SJ, Hojberg O, et al. Host genetics and the rumen microbiome jointly associate with methane emissions in dairy cows. Plos Genet. 2018;14 10 doi: 10.1371/journal.pgen.1007580.
53. Wen CL, Yan W, Sun CJ, Ji CL, Zhou QQ, Zhang DX, et al. The gut microbiota is largely independent of host genetics in regulating fat deposition in chickens. Isme J. 2019;13 6:1422-36. doi:10.1038/s41396-019-0367-2.
54. Chen SF, Zhou YQ, Chen YR and Gu J. fastp: an ultra-fast all-in-one FASTQ preprocessor. Bioinformatics. 2018;34 17:884-90. doi:10.1093/bioinformatics/bty560.
55. Kim D, Landmead B and Salzberg SL. HISAT: a fast spliced aligner with low memory requirements. Nat Methods. 2015;12 4:357-U121. doi:10.1038/Nmeth.3317.
56. Liao Y, Smyth GK and Shi W. featureCounts: an efficient general purpose program for assigning sequence reads to genomic features. Bioinformatics. 2014;30 7:923-30. doi:10.1093/bioinformatics/btt656.
57. Love MI, Huber W and Anders S. Moderated estimation of fold change and dispersion for RNA-

seq data with DESeq2. Genome Biol. 2014;15 12 doi: 10.1186/s13059-014-0550-8.
58. Song Y, Liu X, Stielow JB, de Hoog S and Li R. Post-translational changes in Phialophora verrucosa via lysine lactylation during prolonged presence in a patient with a CARD9-related immune disorder. Front Immunol. 2022;13:966457. doi:10.3389/fimmu.2022.966457.
59. Chen W, Gong L, Guo ZL, Wang WS, Zhang HY, Liu XQ, et al. A Novel Integrated Method for Large-Scale Detection, Identification, and Quantification of Widely Targeted Metabolites: Application in the Study of Rice Metabolomics. Mol Plant. 2013;6 6:1769-80. doi:10.1093/mp/sst080.
60. Team RC. R: A language and environment for statistical computing. MSOR connections. 2014;1.
61. Zierer J, Jackson MA, Kastenmüller G, Mangino M, Long T, Telenti A, et al. The fecal metabolome as a functional readout of the gut microbiome. Nat Genet. 2018;50 6:790-5. doi:10.1038/s41588-018-0135-7.
62. Bertran L, Portillo-Carrasquer M, Aguilar C, Porras JA, Riesco D, Martínez S, et al. Deregulation of Secreted Frizzled-Related Protein 5 in Nonalcoholic Fatty Liver Disease Associated with Obesity. Int J Mol Sci. 2021;22 13:6895.
63. Cepero-Donates Y, Lacraz G, Ghobadi F, Rakotoarivelo V, Orkhis S, Mayhue M, et al. Interleukin-15-mediated inflammation promotes non-alcoholic fatty liver disease. Cytokine. 2016;82:102-11. doi:10.1016/j.cyto.2016.01.020.
64. Kang HW, Niepel MW, Han S, Kawano Y and Cohen DE. Thioesterase superfamily member 2/acyl-CoA thioesterase 13 (Them2/Acot13) regulates hepatic lipid and glucose metabolism. FASEB J. 2012;26 5:2209-21. doi:10.1096/fj.11-202853.
65. Yu J, Zhu C, Wang X, Kim K, Bartolome A, Dongiovanni P, et al. Hepatocyte TLR4 triggers inter-hepatocyte Jagged1/Notch signaling to determine NASH-induced fibrosis. Sci Transl Med. 2021;13 599 doi:10.1126/scitranslmed.abe1692.
66. Jiang Z, Zhao M, Voilquin L, Jung Y, Aikio MA, Sahai T, et al. Isthmin-1 is an adipokine that promotes glucose uptake and improves glucose tolerance and hepatic steatosis. Cell metabolism. 2021;33 9:1836-52. e11.
67. Ma Y, Li Q, Chen G, Tan Z, Cao H, Bin Y, et al. Transcriptomic analysis reveals a novel regulatory factor of ECHDC1 involved in lipid metabolism of non-alcoholic fatty liver disease. Biochem Biophys Res Commun. 2022;605:1-8. doi:10.1016/j.bbrc.2022.03.055.
68. Mao Z, Feng M, Li Z, Zhou M, Xu L, Pan K, et al. ETV5 regulates hepatic fatty acid metabolism through PPAR signaling pathway. Diabetes. 2021;70 1:214-26.
69. Chen H, Gan Q, Yang C, Peng X, Qin J, Qiu S, et al. A novel role of glutathione S-transferase A3 in inhibiting hepatic stellate cell activation and rat hepatic fibrosis. J Transl Med. 2019;17 1:280. doi:10.1186/s12967-019-2027-8.
70. Yu C, Jiang S, Lu J, Coughlin CC, Wang Y, Swietlicki EA, et al. Deletion of Tis7 protects mice from high-fat diet-induced weight gain and blunts the intestinal adaptive response postresection. J Nutr. 2010;140 11:1907-14. doi:10.3945/jn.110.127084.
71. Ye J, Lin Y, Wang Q, Li Y, Zhao Y, Chen L, et al. Integrated Multichip Analysis Identifies Potential Key Genes in the Pathogenesis of Nonalcoholic Steatohepatitis. Front Endocrinol (Lausanne). 2020;11:601745. doi:10.3389/fendo.2020.601745.
72. Gurzov EN, Tran M, Fernandez-Rojo MA, Merry TL, Zhang X, Xu Y, et al. Hepatic oxidative stress promotes insulin-STAT-5 signaling and obesity by inactivating protein tyrosine phosphatase N2.

Cell Metab. 2014;20 1:85-102. doi:10.1016/j.cmet.2014.05.011.

73. Miele L, Giorgio V, Liguori A, Petta S, Pastorino R, Arzani D, et al. Genetic susceptibility of increased intestinal permeability is associated with progressive liver disease and diabetes in patients with non-alcoholic fatty liver disease. Nutrition, Metabolism and Cardiovascular Diseases. 2020;30 11:2103-10.

74. Yu J, Tao Q, Cheung KF, Jin H, Poon FF, Wang X, et al. Epigenetic identification of ubiquitin carboxyl- terminal hydrolase L1 as a functional tumor suppressor and biomarker for hepatocellular carcinoma and other digestive tumors. Hepatology. 2008;48 2:508-18.

75. Zhai R, Feng L, Zhang Y, Liu W, Li S and Hu Z. Combined Transcriptomic and Lipidomic Analysis Reveals Dysregulated Genes Expression and Lipid Metabolism Profiles in the Early Stage of Fatty Liver Disease in Rats. Front Nutr. 2021;8:733197. doi:10.3389/fnut.2021.733197.

76. Marsili A, Aguayo-Mazzucato C, Chen T, Kumar A, Chung M, Lunsford EP, et al. Mice with a targeted deletion of the type 2 deiodinase are insulin resistant and susceptible to diet induced obesity. PLoS One. 2011;6 6:e20832. doi:10.1371/journal.pone.0020832.

77. Sun H, Huang FF and Qu S. Melatonin: a potential intervention for hepatic steatosis. Lipids Health Dis. 2015;14:75. doi:10.1186/s12944-015-0081-7.

78. Matsuda A, Wang Z, Takahashi S, Tokuda T, Miura N and Hasegawa J. Upregulation of mRNA of retinoid binding protein and fatty acid binding protein by cholesterol enriched-diet and effect of ginger on lipid metabolism. Life Sci. 2009;84 25-26:903-7. doi:10.1016/j.lfs.2009.04.004.

79. Wang X, Hasegawa J, Kitamura Y, Wang Z, Matsuda A, Shinoda W, et al. Effects of hesperidin on the progression of hypercholesterolemia and fatty liver induced by high-cholesterol diet in rats. J Pharmacol Sci. 2011;117 3:129-38. doi:10.1254/jphs.11097fp.

Figure legends

Fig. 1. Phenotypic profiling of the histochemical stained sections of the chicken livers and hepatic and plasma biochemical indicators among HSC (eHSC) groups.

(**A**) Micrographs of H&E-stained and oil-red-O-stained whole sections of the chicken liver (scale bar: 100μm). n = 673. (**B-D**). Boxplots of hepatic and plasmic triglyceride (HTG and PTG, respectively) (B), hepatic crude fat (HCF) and free fatty acids (HFFAs) (C), plasma high-density lipoprotein (PHDL), low-density lipoprotein (PLDL) and very low-density lipoprotein (PVLDL) (D), showing high levels in the hepatic steatosis group (P_{adj} < 0.05, post-hoc Wilcoxon rank-sum test). n = 673. (**E**) Boxplots of hepatic and plasma total bile acids (HTBAs and PTBAs, respectively) showing a significant inverse correlation with hepatic steatosis (P_{adj} < 0.05, post-hoc Wilcoxon rank-sum test). n = 673. (**F**) Boxplots of the association of hepatic and plasma total cholesterol (HTC and PTC, respectively) among HSC groups (P_{adj} < 0.05 for hepatic TC and P_{adj} < 0.05 for plasma, post-hoc Wilcoxon rank-sum test). n = 673. (**G**) Boxplots of the abdominal fat weight (AFW), showing a significant increase with hepatic steatosis (P_{adj} < 0.05, post-hoc Wilcoxon rank-sum test). n = 673. (**H**) Bar plots of hepatic triglyceride (HTG) and crude fat (HCF), showing significant increases with hepatic steatosis in eHSC groups (P_{adj} < 0.05, post-hoc Wilcoxon rank-sum test). Data are from n = 30 biological replicates. *p < 0.05, **p < 0.01, ***p < 0.001.

Fig. 2. Genomic determinants of HSC and its related indicators.

(**A**) The SNP-based heritability estimations of HSC and biochemical indicators. HSC: hepatic steatosis classification; HTG and PTG: hepatic and plasma triglyceride, respectively; HCF: hepatic crude fat; HFFA: hepatic free fatty acids; PHDL: plasma high-density lipoprotein; PLDL: plasma low-density lipoprotein; PVLDL: plasma very low-density lipoprotein; HTBA and PTBA: hepatic and plasma total bile acid, respectively; HTC and PTC: hepatic and plasma total cholesterol, respectively; AFW: abdominal fat weight. $n \geq 673$. (**B**) Circular Manhattan plots of GWAS for HSC, HCF and HTG. Gray, dark blue and reddish-brown dots indicate non-significant, suggestively significant and significant SNPs, respectively. Colocalization of trans-eQTLs of phosphatidylethanolamine N-methyltransferase (*PEMT*) gene in the liver and GWAS loci of HSC in chickens on chromosome 6 identified two colocalized SNPs, which were the significant trans-eQTL of *PEMT* and the top GWAS signals of HSC. $n \geq 668$. (**C**) Stacked bar plots of the comparison of individuals distributing in different HSC levels across the three genotypes of these two eVariant (rs731375960 and rs731375960, $P_{adj} < 0.05$, chi-squared test). $n = 673$. (**D**) Raincloud plot shows the expression levels of the *PEMT* gene in the liver across the three genotypes of the top eVariant (rs731375960, $P_{adj} < 0.05$, post-hoc Wilcoxon rank-sum test). $n = 668$. (**E**) Spearman's rank-based correlation (SRC) analysis and analysis of variance (ANOVA) were performed to ascertain candidate genes in the liver for hepatics steatosis. $n = 673$. *$p < 0.05$, **$p < 0.01$, ***$p < 0.001$.

Fig. 3. Multi-omics data reveal the molecular regulation mechanism of hepatic steatosis.

(For Figure B-H, 0 refers to the eCon group, 1 for eHS-I, and 2 for eHS-II).

(**A**) Illustration summarizing three regulation routes to hepatic steatosis. To better demonstrate the results, not all enzymes and metabolites in the pathway are shown. (1) the severe accumulation of hepatic free fatty acids (HFFAs) and hepatic triglyceride (HTG), and the decrease of phosphatidylcholine (PC) partially caused by the low expression of phosphatidylethanolamine N-methyltransferase (*PEMT*), impeded the outward transport of TG from hepatocytes; Acyl-CoA synthetase long chain family member 5, 4, 3: *ACSL5*,*4*,*3*, choline kinase alpha: *CHKA*, phosphate cytidylyltransferase 1A: *PCYT1A*, choline phosphotransferase 1: *CHPT1*. (2) Use of HFFA was inefficient because of the weak fatty acid β-oxidation activity; Carnitine palmitoyltransferase 1A, 2: *CPT1A* and *CPT2*, solute carrier family 25 member 20: *SLC25A20*. (3) the peroxidation of excessive HFFA accelerated the progression of hepatic steatosis; Superoxide Dismutase 1, 2: SOD1 and SOD2, glutathione peroxidase 3: *GPX3*, catalase: *CAT*. (**B**) HFFAs increased significantly with the severity of hepatic steatosis (Padj < 0.05, post-hoc Wilcoxon rank-sum test). The opposite change pattern applied to PC and the PC/phosphatidyl-ethnolamine (PE) ratio ($P_{adj} < 0.05$, post-hoc Wilcoxon rank-sum test). (**C**) Expression of apolipoprotein B (*APOB*), *CHKA*, *PCYT1A* and *CHPT1* in the liver shared the same change pattern, decreasing significantly with hepatic steatosis in the eHSC group ($P_{adj} <$ 0.05, post-hoc Wilcoxon rank-sum test). (**D**) The most severe hepatic steatosis group possessed the highest long-chain acylcarnitines (LCACs) but the least short-chain acylcarnitines (SCACs) ($P_{adj} < 0.05$, post-hoc Wilcoxon rank-sum test). (**E**) Expression of *CPT1A*, *SLC25A20*, and *CPT2* in the liver were significantly negatively correlated to hepatic steatosis in the eHSC

group ($P_{adj} < 0.05$, post-hoc Wilcoxon rank-sum test). (**F**) Superoxide dismutase 1/2 (SOD1/2) were significantly up-regulated in steatosis groups ($P_{adj} < 0.05$, post-hoc Wilcoxon rank-sum test), n = 4; while GPX3 was more expressed in steatosis groups ($P_{adj} < 0.05$, post-hoc Wilcoxon rank-sum test). (**G**) Oxidized and reduced glutathione (GSH-O and GSH-R, respectively), were also extremely low but 4-hydroxynonenal (HNE) was highly presented in steatotic livers ($P_{adj} < 0.05$, post-hoc Wilcoxon rank-sum test). (**H**) 9- and 13-hydroxy-octadecadienoic acid (9- and 13-HODE) and 9- and 13-oxo-octadecadienoic acid (9- and 13-oxo ODE) were higher in the steatosis group than that in the control group ($P_{adj} < 0.05$, post-hoc Wilcoxon rank-sum test). Data of genes, metabolites and proteins are from n = 30, 6 and 4 biological replicates, respectively.

Fig. 4. Contribution of the gut microbial community to fat deposition related traits and their correlation with duodenal microbiota.

(**A**) Microbiability of duodenal, jejunal, ileal, cecal and fecal microbiota for hepatic steatosis classification (HSC), hepatic triglyceride (HTG), hepatic crude fat (HCF), hepatic free fatty acids (HFFA) and hepatic total bile acids (HTBA). (**B**) Four taxa chains from the phylum to genus level were generated on the basis of the taxa associated significantly with HSC by Spearman's rank-based correlation (SRC) analysis. n = 673. (**C**) Linear discriminant analysis Effect Size (LEfSe) analysis identified differential taxa on the basis of their significantly differential presence among HSC groups (LDA > 2). n = 673. (**D**) Heatmap of the association of genus abundance with all recorded phenotypes (SRC analysis, + indicates statistical

significance $P_{adj} < 0.05$, FDR correction); PTG: plasma triglyceride, PHDL: plasma high-density lipoprotein, PLDL: low-density lipoprotein, PVLDL: very low-density lipoprotein, PTBA: plasma total bile acids, HTC: hepatic total cholesterol, PTC: plasma total cholesterol. n ≥ 673. (**E**) The amount and cumulative relative abundance of duodenal taxa with different detection rates from phylum to species. n = 686.

Fig. 5. Chicken hepatic methionine cycle, folate cycle, and associated genes and metabolites. To better demonstrate the results, not all enzymes and metabolites in the pathway were shown.

(**A**) Methionine cycle coupled with folate cycle. The scheme shows the main reactions and correlated genes involved in the methionine and folate cycle. Two main methionine adenosyltransferase isoforms (*MAT1A* and *MAT2B*), were respectively 2.2 and 2.6 times higher in the eCon group. Phosphatidylethanolamine N-methyltransferase (*PEMT*), S-adenosylhomocysteine hydrolase (*AHCY*) and methionine synthase (*MTR*) were significantly upregulated in the eCon group with 2.1, 2.6 and 22.5 times higher expression, respectively. (**B**) Comparison of the folic acid quantity in the liver (LFLA) and duodenal chyme (DFLA). 0 refers to the eCon group, and 1 for eHS-I, 2 for eHS-II. (**C**) Contents of methionine, S-adenosylmethionine (SAMe), S-adenosylhomocysteine (SAH) and homocysteine (Hcy) in the liver determined by ELISAs assay. 0 refers to the eCon group, and 1 for eHS-I, 2 for eHS-II. Data are from n = 30 (A) and 10 (B and C) biological replicates, respectively.

Supplementary Fig. S1. Correlations among all recorded phenotypes, including the hepatic steatosis classification (HSC), hepatic and plasma triglyceride (HTG and PTG, respectively), hepatic crude fat (HCF) and free fatty acids (HFFAs), plasma high-density lipoprotein (PHDL), low-density lipoprotein (PLDL) and very low-density lipoprotein (PVLDL), hepatic and plasma total bile acids (HTBAs and PTBAs, respectively), hepatic and plasma total cholesterol (HTC and PTC, respectively) and abdominal fat weight (AFW). The lower panel shows scatterplots for each pair of observations. Each point represents an individual. n = 673.*p < 0.05, **p < 0.01, ***p < 0.001.

Supplementary Fig. S2. Violin plots of the change patterns of hepatic triglyceride (HTG), hepatic and plasma total bile acids (HTBAs and PTBAs, respectively) and abdominal fat weight (AFW) changed with hepatic steatosis in eHSC groups ($P_{adj} < 0.05$, post-hoc Wilcoxon rank-sum test). Data are from n = 30 biological replicates. *p < 0.05, **p < 0.01, ***p < 0.001.

Supplementary Fig. S3. The amount and distribution of cis- and trans-regulated genes in liver.

Supplementary Fig. S4. GO and pathway enrichment analysis of 227 candidate genes identified many biological processes of lipid localization and biosynthetic processes.

Supplementary Fig. S5. (**A**) Permutation test of duodenal microbiability of hepatic steatosis classification (HSC), hepatic free fatty acids (HFFAs) and hepatic total bile acids (HTBAs),

and cecal microbiability of hepatic crude fat (HCF) and hepatic triglyceride (HTG). The results indicated that the estimated m^2 (0.15-0.27, blue lines) were significantly higher than the simulated ones (average m2 ranged from 0.02-0.04, gray lines). $n \geq 673$. (**B**) The amount of taxa classified from phylum to species with high quality ASVs. n = 705. (**C**) Alpha diversities, including Shannon and Simpson Indices, both decreased with hepatic steatosis development ($P_{adj} < 0.05$, post-hoc Wilcoxon rank-sum test). n = 673. *$p < 0.05$, **$p < 0.01$.

Supplementary Fig. S6. Heatmaps of the association of the relative abundance of phyla, classes, orders, families and species with all recorded phenotypes (Spearman's rank-based correlation analysis, + indicates statistical significance $P_{adj} < 0.05$, FDR correction); HSC: hepatic steatosis classification, HTG: hepatic triglyceride, PTG: plasma triglyceride, HCF: hepatic crude fat, HFFA: hepatic free fatty acid, PHDL: plasma high-density lipoprotein, PLDL: plasma low-density lipoprotein, PVLDL: plasma very low-density lipoprotein, HTBAs: hepatic total bile acids, PTBAs: plasma total bile acids, HTC: hepatic total cholesterol, PTC: plasma total cholesterol, AFW: abdominal fat weight. $n \geq 673$.

Tables

Table 1 Description of 18 trans-eGenes related to hepatic steatosis

Gene symbol	High presented in	Gene location	eVariants region	No. of significant SNPs	Top SNP (Location)	Demonstration of the gene function in the references
NAPEPLD	Con	GGA1 13,065,354-13,085,319 bp	GGA10 10.2-17.9Mb	19044	rs313818251 (10:14,621,940)	Loss of *NAPEPLD* result in fat mass gain and hepatic steatosis in mouse [20]
WNT5A	HS	GGA12 8,303,804-8,315,223 bp	GGA5 30.1-36.4Mb	8259	- (5:34,210,278)	*WNT5A* may promote liver damage in human [62]
IL15	HS	GGA4 29,989,429-30,022,148 bp	GGA2 89.3-100.9Mp	8244	- (2:99,506,822)	Absence of *IL-15* or *IL-15Rα* protects from NAFL in mouse [63]
ACOT13	HS	GGA2 90,236,164-90,240,896 bp	GGA1 145.1-150.8Mp	6020	rs316486650 (1:148,449,556)	Regulate hepatic lipid and glucose metabolism in mouse [64]
JAG1	Con	GGA3 13,599,642-13,633,726 bp	GGA21 5.04-6.03Mp	4428	rs313406743 (21:5,121,276)	Hepatocyte-specific *JAG1* knockout mice were protected from NASH-induced liver fibrosis [65]
ISM1	Con	GGA3 12,825,298-12,864,186 bp	GGA21 4.07-4.65 Mb	700	rs315660086 (21:4,319,591)	*ISM1* suppresses hepatocyte lipid synthesis in mouse [66]
ECHDC1	HS	GGA3 59,266,598-59,303,456 bp	GGA5 17.8-18.0 Mb	11	rs316727537 (5:17,943,618)	Involved in the occurrence and development of NAFLD by regulating hepatic lipid metabolism in human [67]
ETV5	Con	GGA9 5,289,877-5,299,258 bp	GGA4 61.15-61.17 Mb	4	rs315563554 (4:61,152,625)	Regulate hepatic fatty acid metabolism in mouse [68]

GSTA3	Con	GGA3 88,388,999-88,395,707 bp	GGA2 34.0-34.5 Mb	4	rs314345391 (2:34,475,071)	Paly vital roles in hepatic iron metabolism, and may associated with NALFD in mouse [69]
IFRD1	Con	GGA1 27,057,994-27,068,635 bp	GGA11 11.0-11.7 Mb	13	- (11:11,142,255)	A regulator of lipid absorption and metabolism in mouse [70]
SLITRK3	Con	GGA9 21,273,430-21,276,090 bp	GGA4 79.7-80.0 Mb	38	- (4:79,863,187)	*SLITRK3* was down-regulated in steatosis and NASH patients in human [71]
PTPN2	Con	GGA2 97,013,090-97,047,494 bp	GGA17 3.47-4.08 Mb	16	- (17:3,671,818)	Liver-specific *PTPN2* deficiency promotes hepatic steatosis, obesity and insulin resistance in human and mouse [72, 73]
UCHL1	Con	GGA4 68,642,734-68,647,699 bp	GGA2 139.8-140.2 MB	15	rs312268369 (2:140,103,337)	*UCHL1* appears to be a functional tumor suppressor involved in the tumorigenesis of hepatocellular carcinoma in human [74]
SLC16A10	Con	GGA3 66,217,809-66,280,609 bp	GGA3 10876753-10876785 bp	3	rs739986067 (3:10,876,769)	*SLC16A10* is a transport carrier of aromatic amino acids, it up-regulated in NAFLD group in Rats [75]
DIO2	Con	GGA5 40,752,235-40,769,122 bp	GGA3 8.69-8.86 Mb	2	rs315951384 (3:8,693,350)	Loss of the *DIO2* gene results in increased fat storage in adipose tissue and hepatic steatosis in mouse [76]
MT3	Con	GGA11 2,122,187-2,123,328 bp	GGA8 15963283 bp	1	rs314635449 (8:15963283)	*MT3* may be a potential intervention for hepatic steatosis by inhibit the generation of ROS in human [77]
RBP	Con	GGA8 21,595,383-21,606,407 bp	GGA1 55817413 bp	1	- (3:55817413)	Lower expression of *RBP* may improve hepatic steatosis in mouse [78, 79]

NAPEPLD: N-acyl phosphatidylethanolamine phospholipase D, *WNT5A*: Wnt Family Member 5A, *IL15*: Interleukin 15, *ACOT13*: Acyl-CoA thioesterase 13, *JAG1*: Jagged canonical Notch ligand 1, *ISM1*: Isthmin 1, *ECHDC1*: Ethylmalonyl-CoA decarboxylase 1, *ETV5*: ETS variant transcription factor 7, *GSTA3*: Glutathione S-transferase alpha 3, *IFRD1*: Interferon related developmental regulator 1, *SLITRK3*: SLIT and NTRK like family member 3, *PTPN2*: Protein tyrosine phosphatase non-receptor type 2, *UCHL1*: Ubiquitin C-terminal hydrolase L1, *SLC16A10*: Solute carrier family 16 member 10, *DIO2*: Deiodinase 2, *MT3*: Metallothionein 3, *RBP*: Retinol binding protein 1

Figure 1 Click here to access/download;Figure;Figure 1.tif

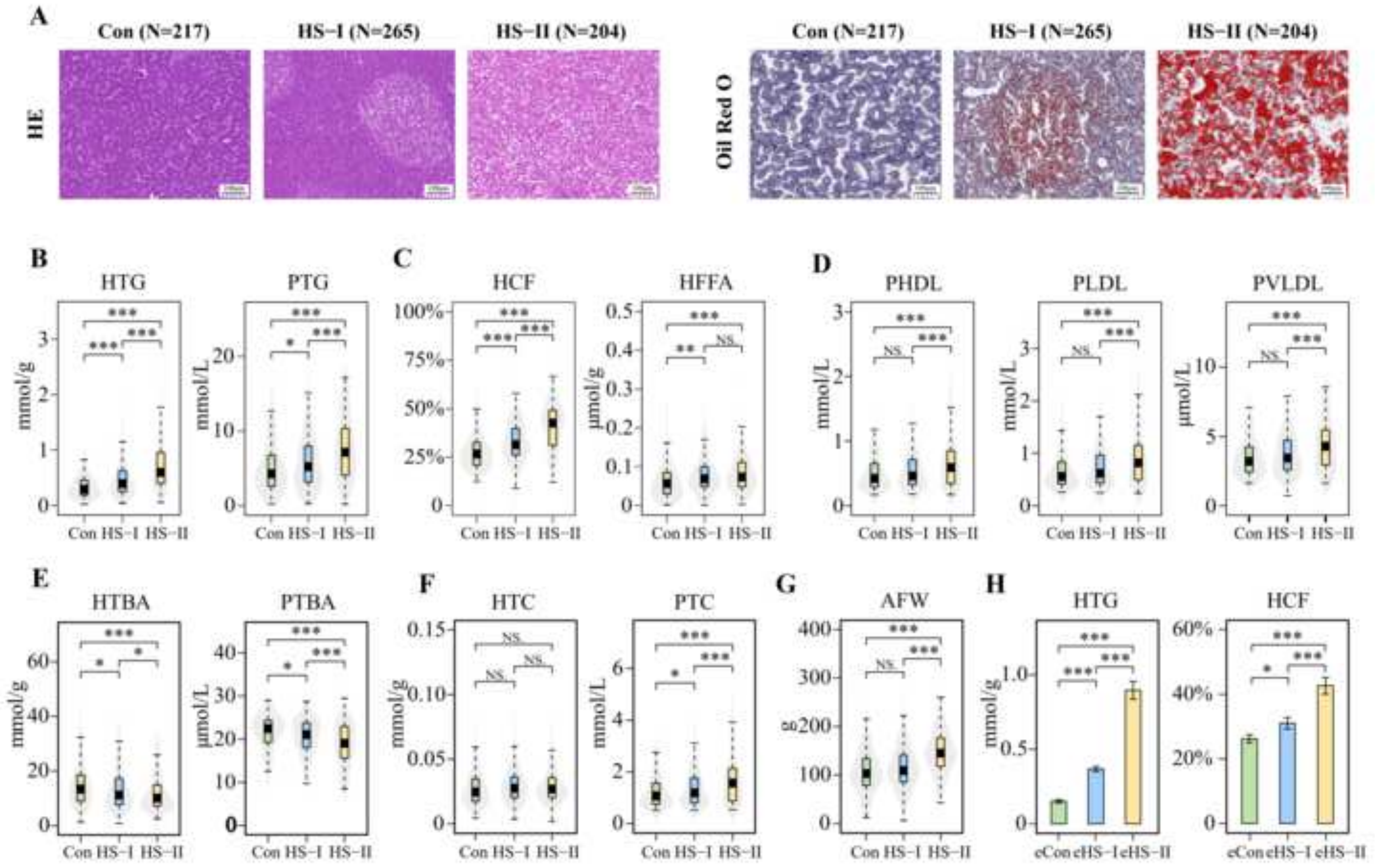

Figure 2

Click here to access/download;Figure;Figure 2.tif

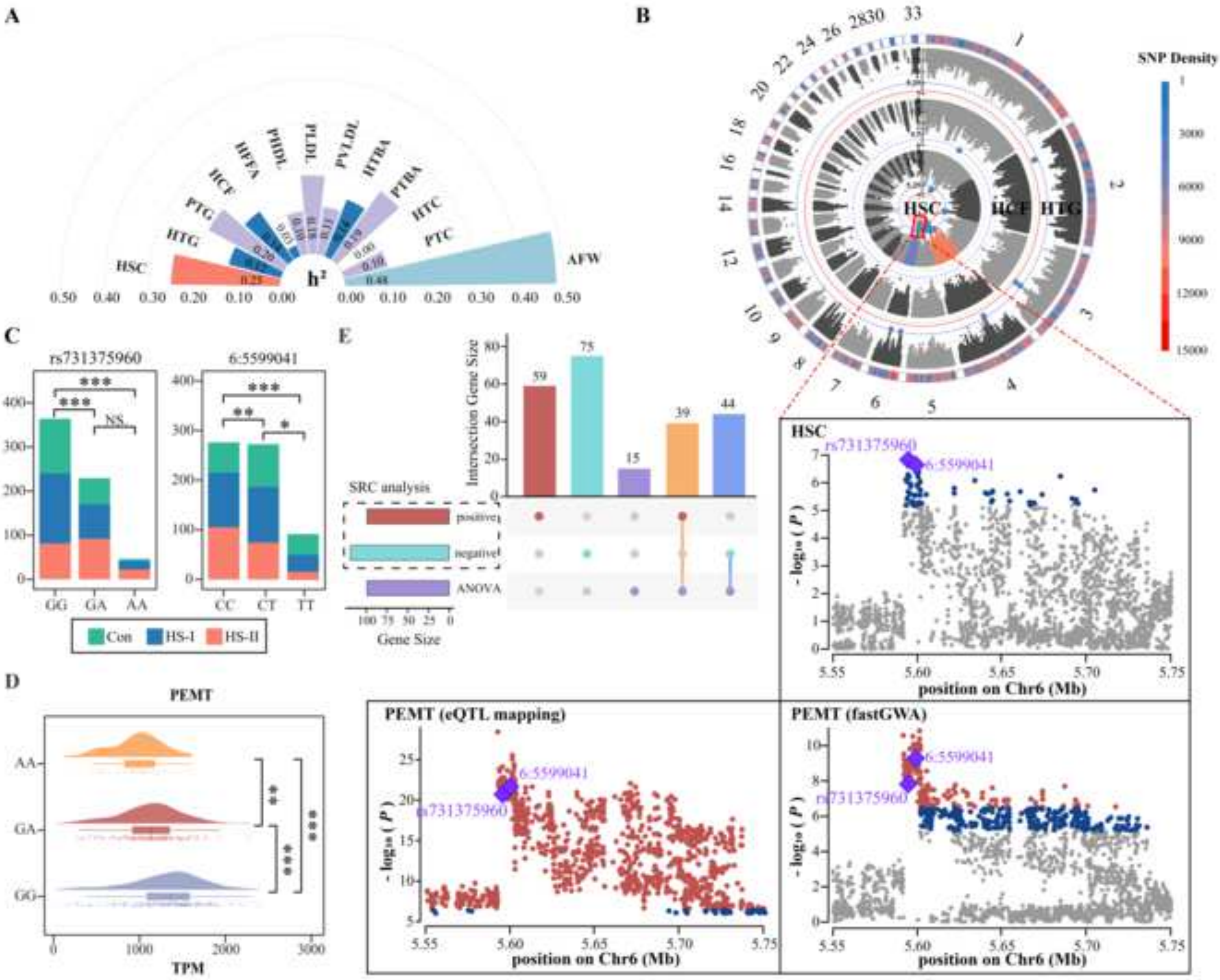

Figure 3

Click here to access/download;Figure;Figure 3.tif

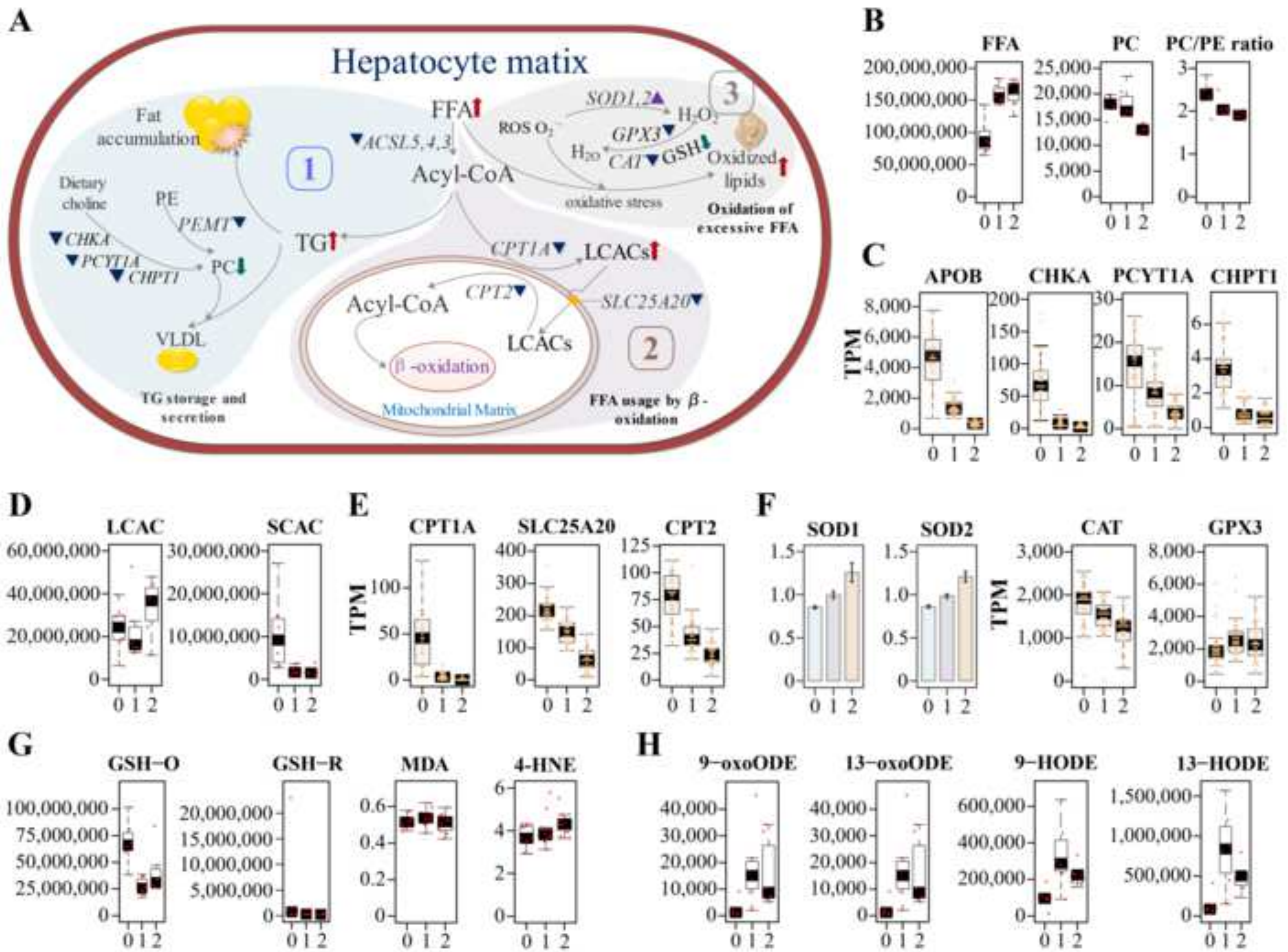

Figure 4

Click here to access/download;Figure;Figure 4.tif

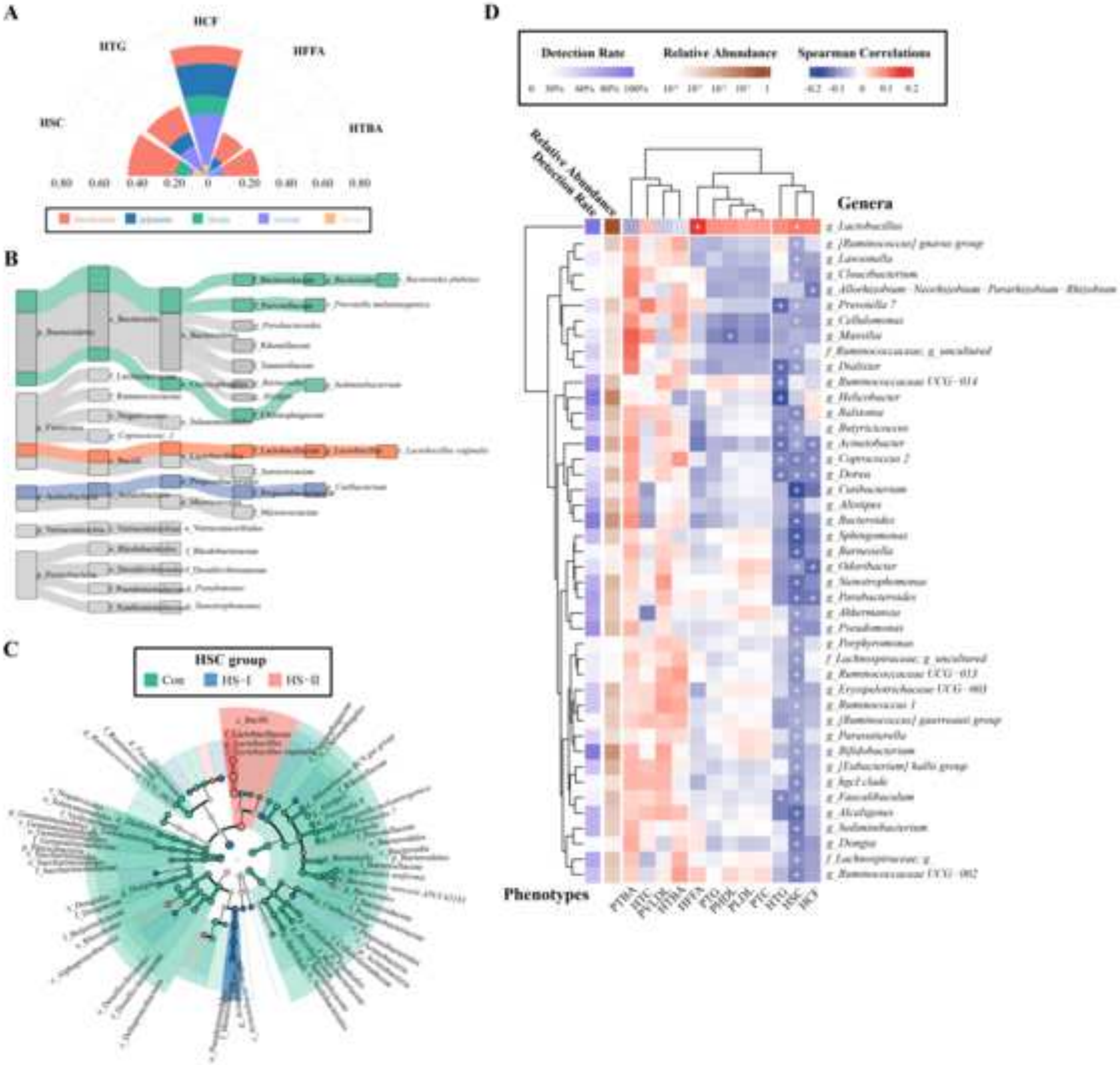

Figure 5

Click here to access/download;Figure;Figure 5.tif

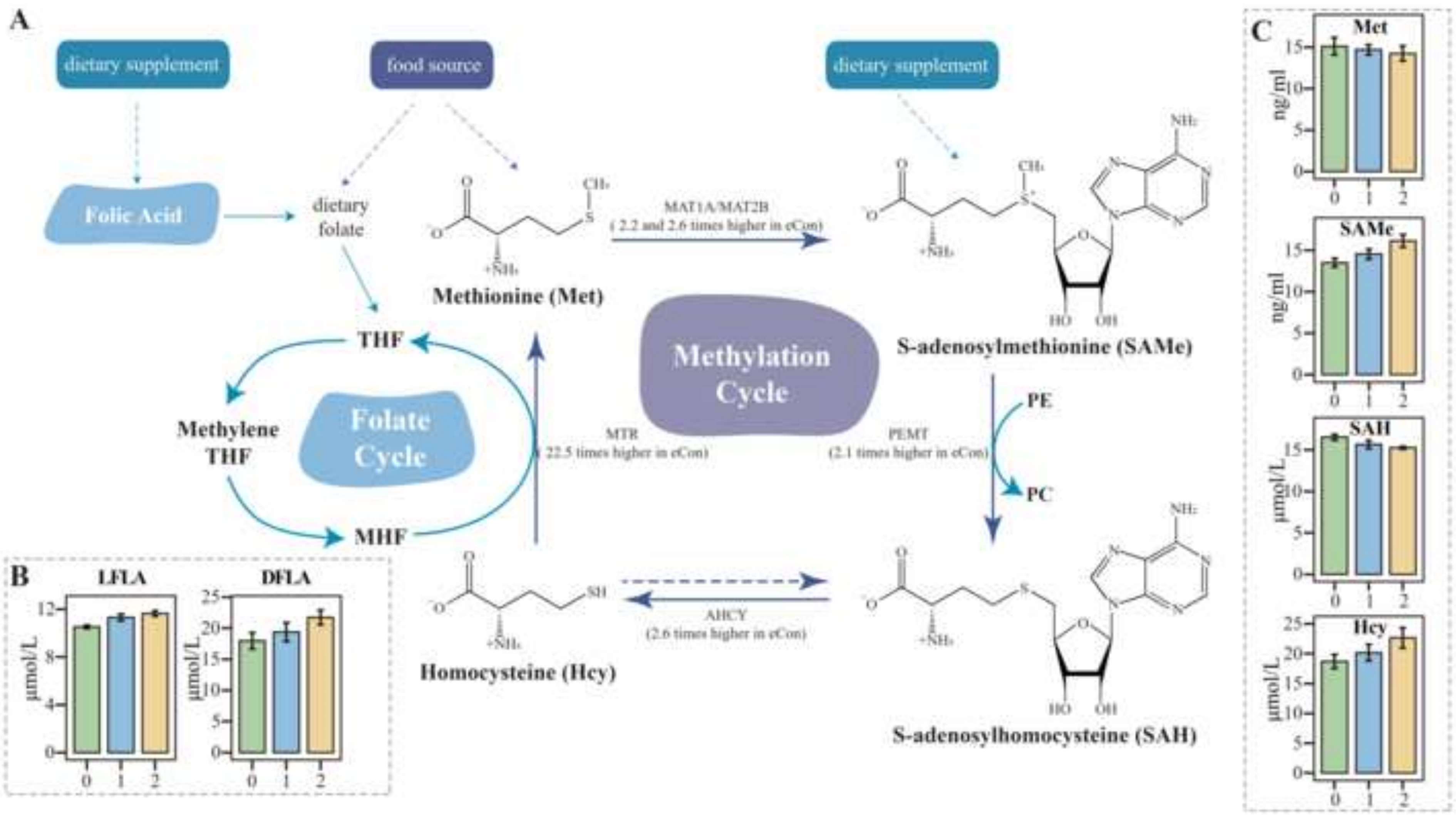

Supplementary Figure S1

Click here to access/download;Figure;Figure S1.tif

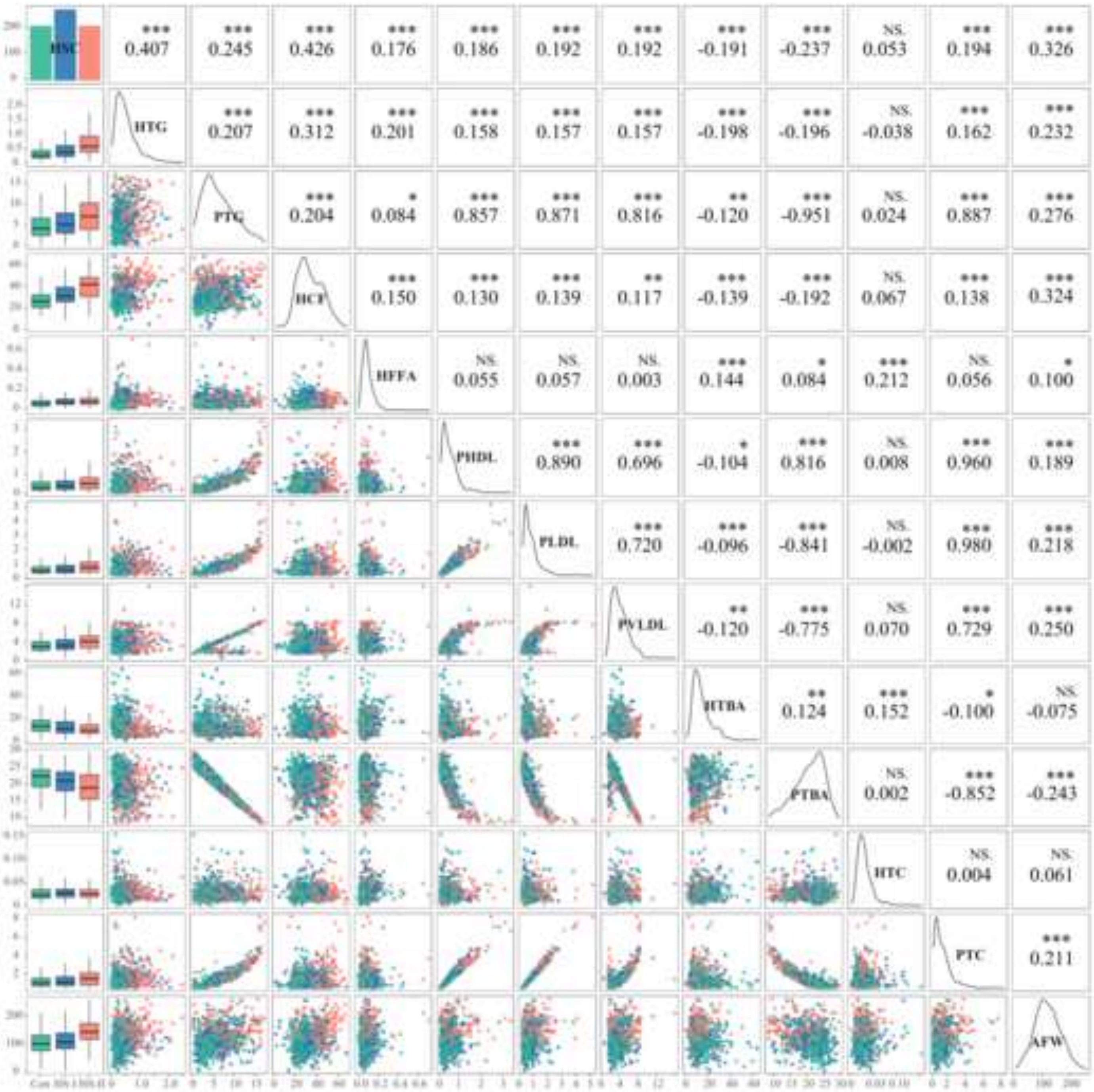

 Click here to access/download;Figure;Figure S2.tif

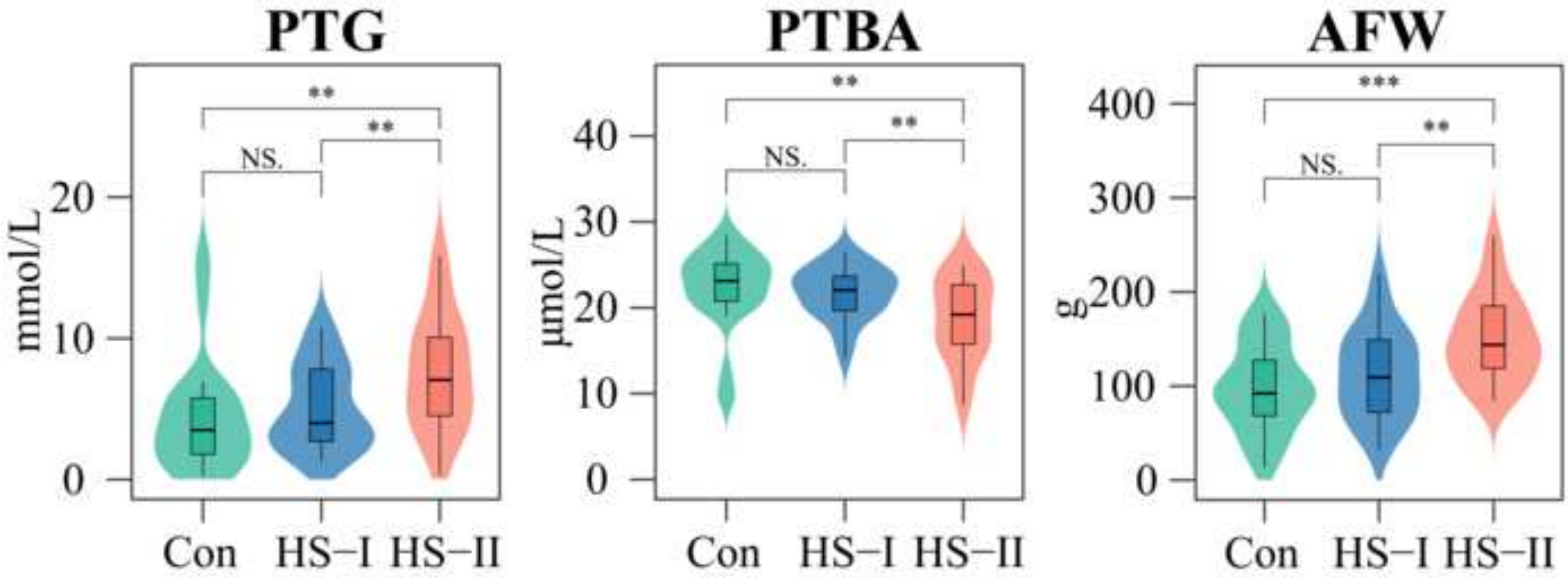

Supplementary Figure S3

Click here to access/download;Figure;Figure S3.tif

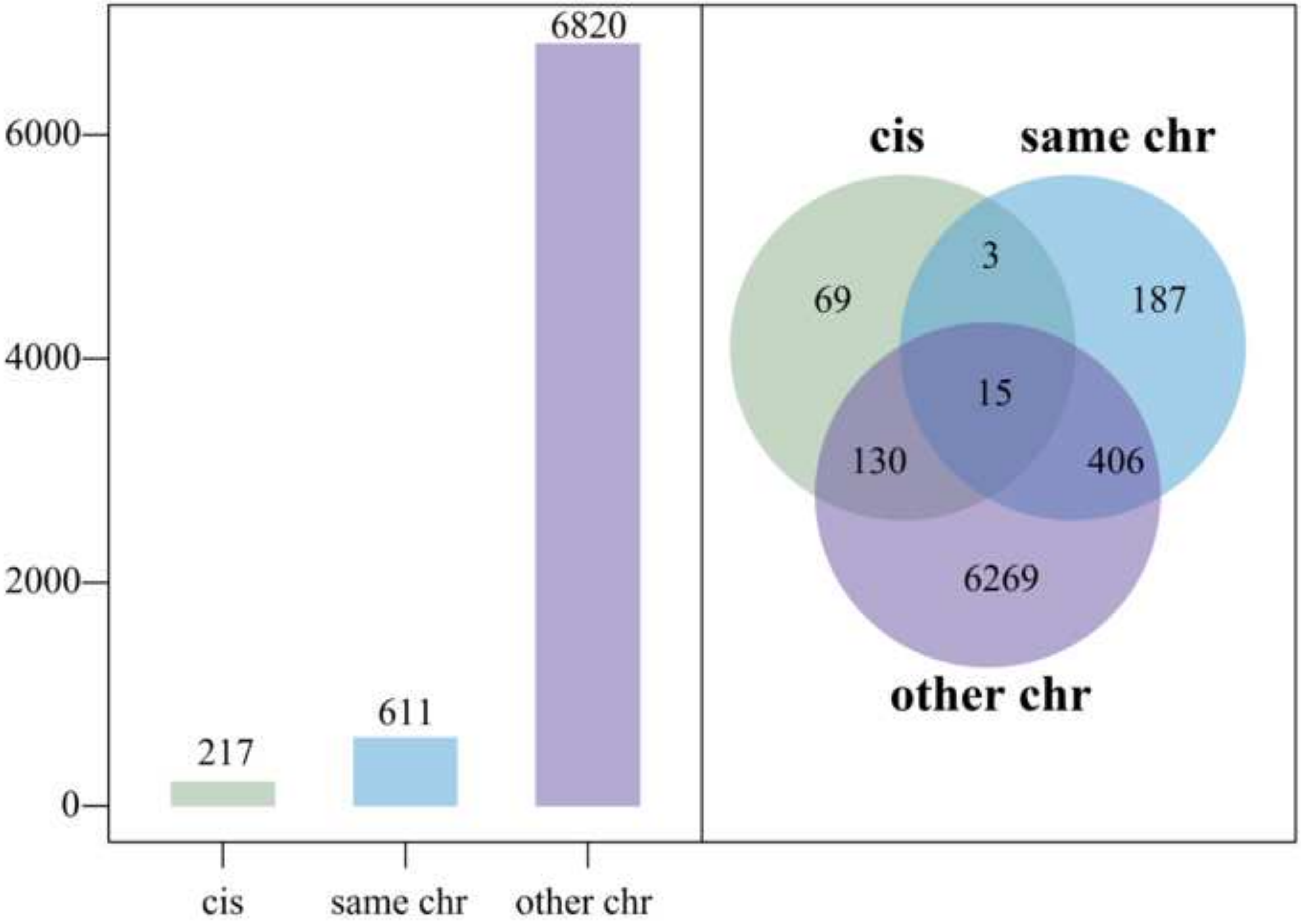

Supplementary Figure S4

Click here to access/download;Figure;Figure S4.tif

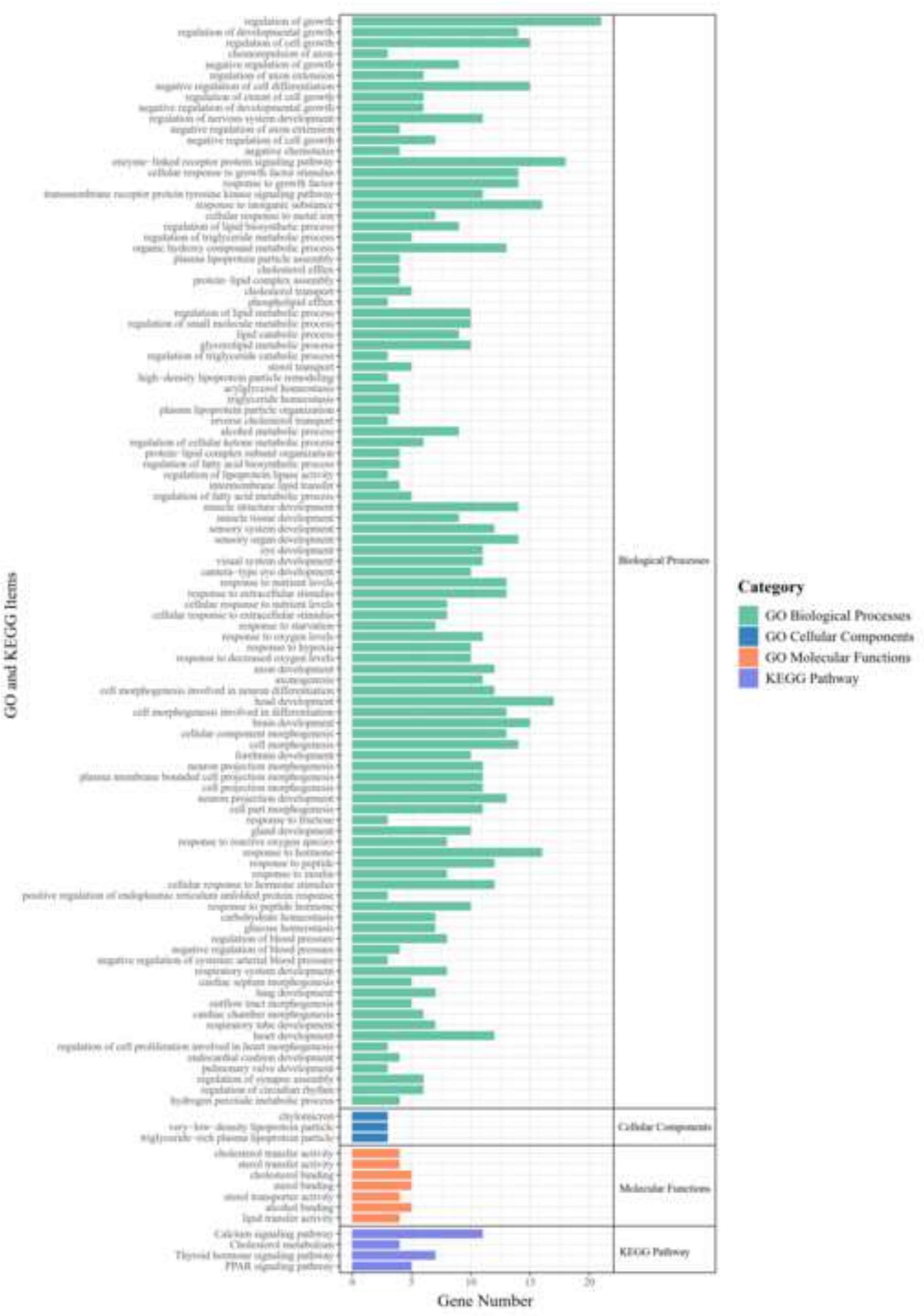

Supplementary Figure S5 Click here to access/download;Figure;Figure S5.tif

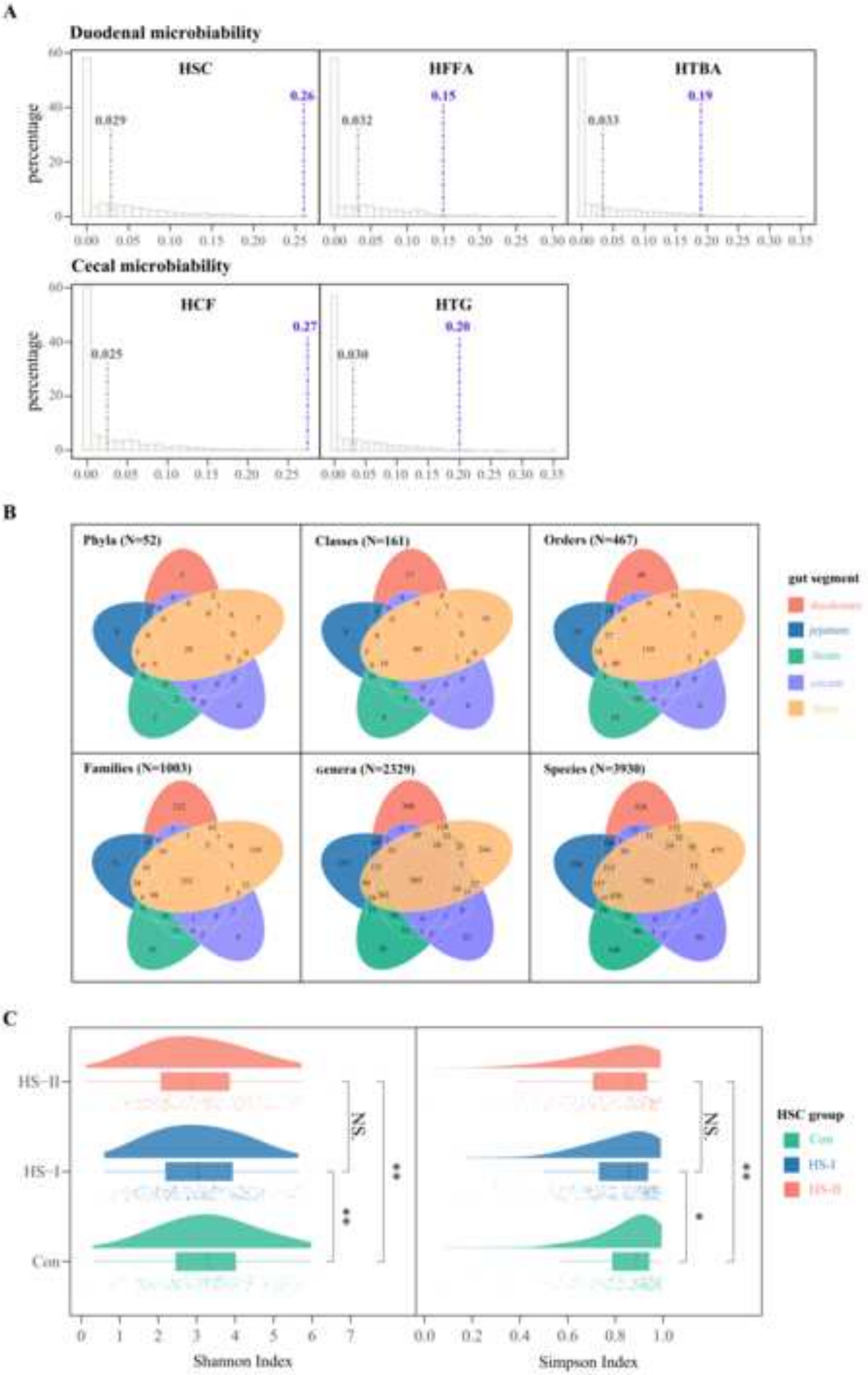

Supplementary Figure S6

Click here to access/download;Figure;Figure S6.tif

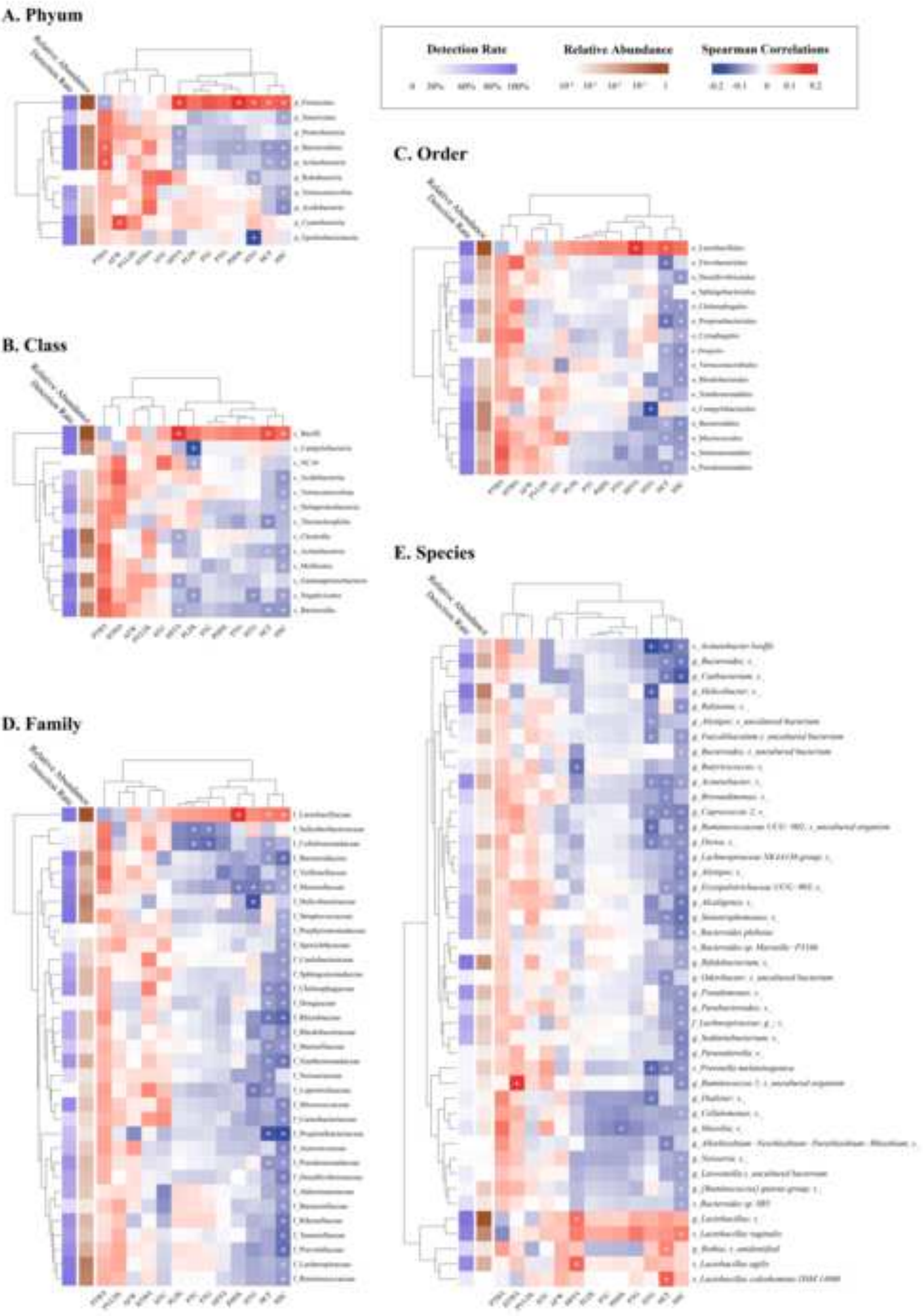

 Click here to access/download;Figure;PCA.tif

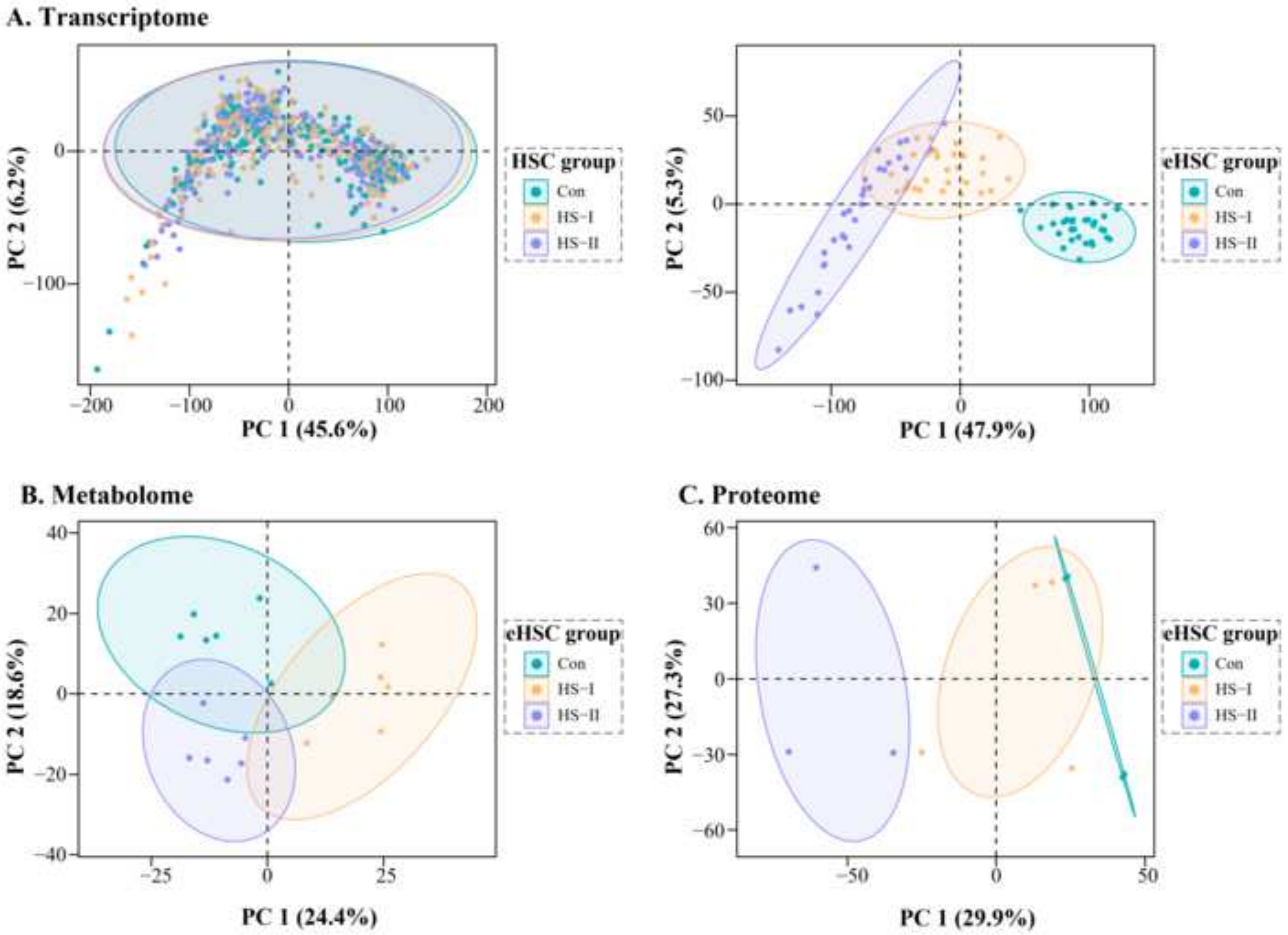

Supplementary Tables

Click here to access/download
Supplementary Material
Supplementary Tables.xlsx
