# Supplementary material for: Mechanisms of hepatic steatosis in chickens: integrated analysis of the host genome, molecular phenomics and gut microbiome
Source: Gigascience. 2024 Jun 5;13:giae023. doi: 10.1093/gigascience/giae023 (PMC11152177; doi:10.1093/gigascience/giae023)
Supplement: giae023_GIGA_D_23_00122_Revision_2 [file giae023_giga_d_23_00122_revision_2.pdf]

GigaScience

Mechanisms of hepatic steatosis in chickens: integrated analysis of the host genome, molecular phenomics and gut microbiome

--Manuscript Draft--

**Manuscript Number:**	GIGA-D-23-00122R2
**Full Title:**	Mechanisms of hepatic steatosis in chickens: integrated analysis of the host genome, molecular phenomics and gut microbiome
**Article Type:**	Research
**Funding Information:**	National Natural Science Foundation of China (31930105) — Professor Ning Yang<br>Agriculture Research System of China (CARS-40) — Professor Ning Yang<br>National Key Research and Development Programof China (2022YFF1000204) — Professor Ning Yang<br>2115 Talent Development Progarm of China Agricultural University — Associate professor Congjiao Sun
**Abstract:**	Hepatic steatosis is the initial manifestation of abnormal liver functions and often leads to liver diseases such as non-alcoholic fatty liver disease in humans and fatty liver syndrome in animals. In this study, we conducted a comprehensive analysis of a large chicken population consisting of 705 adult hens by combining host genome resequencing, liver transcriptome, proteome, and metabolome analysis, as well as microbial 16S rRNA gene sequencing of each gut segment. The results showed the heritability (h2=0.25) and duodenal microbiability (m2=0.26) of hepatic steatosis were relatively high, indicating a large effect of host genetics and duodenal microbiota on chicken hepatic steatosis. Individuals with hepatic steatosis had low microbiota diversity and a decreased genetic potential to process triglyceride output from hepatocytes, fatty acid β-oxidation activity, and resistance to fatty acid peroxidation. Furthermore, we revealed a molecular network linking host genomic variants (GGA6: 5.59–5.69 Mb), hepatic gene/protein expression (PEMT, phosphatidyl-ethanolamine N-methyltransferase), metabolite abundances (folate, S-adenosylmethionine, homocysteine, phosphatidyl-ethanolamine, and phosphatidylcholine) and duodenal microbes (genus Lactobacillus) to hepatic steatosis, which could provide new insights into the regulatory mechanism of fatty liver development.
**Corresponding Author:**	Congjiao Sun<br>China Agricultural University<br>Beijing, CHINA
**Corresponding Author Secondary Information:**	
**Corresponding Author's Institution:**	China Agricultural University
**Corresponding Author's Secondary Institution:**	
**First Author:**	Congjiao Sun
**First Author Secondary Information:**	
**Order of Authors:**	Congjiao Sun
	Fangren Lan
	Qianqian Zhou
	Xiaoli Guo
	Jiaming Jin
	Chaoliang Wen

Powered by Editorial Manager® and ProduXion Manager® from Aries Systems Corporation

	Yanxin Guo
	Zhuocheng Hou
	Jiangxia Zheng
	Guiqin Wu
	Guangqi Li
	Yiyuan Yan
	Junying Li
	Qiugang Ma
	Ning Yang
**Order of Authors Secondary Information:**	
**Response to Reviewers:**	Dear Editor,<br>We are grateful to you and reviewers for your valuable comments and suggestions on our manuscript (GIGA-D-23-00122). We have made all necessary revisions to improve the quality of our manuscript.<br>In the revised manuscript, we provided a more detailed interpretation regarding “gut-liver axis” at the introduction section, and shortened the lengthy description. With respect to results, we have added the specific meaning of the values in each group and interpreted the phenomenon derived from HTG and HCF. Furthermore, we have refined the depiction of the three regulatory routes deduced from multi-omics data for hepatic steatosis. Besides, we have deposited our scripts to Github at https://github.com/FangrenLan97/gigascience. Meanwhile, all figures and tables, including supplementary files, were double-checked.<br>The modifications are highlighted in the revised manuscript. The responses to the comments are listed below. We believe that we have carefully addressed all the comments and hope that the modifications meet with your approval.<br>Thank you again and we look forward to hearing from you soon.<br>Sincerely,<br>Ning Yang, Ph.D.<br>Professor of Poultry Genetics and Breeding<br>China Agricultural University<br>Beijing 100193, China<br>Tel: +86-10-6273 1351<br>E-mail: nyang@cau.edu.cn; cjsun@cau.edu.cn<br><br><br><br>Response to comments from Anonymous Referee #1<br>Introduction<br>1.1.Line56-73 —a very general description (not suitable for a research paper), consider replacing it with specific details on how gut microbiota are involved in gut-liver axis, promoting metabolic disorders, including liver disease (extending Line 74-Line 75).<br>A1.1: We have revised the introduction section to specifically describe the complex crosstalk along the gut-liver axis. See lines 60-68.<br>1.2.Line91-97 —modify the very lengthy description like " Chickens are ideal for large-scale studies due to their high reproductive capacity and low cost. Fatty liver in chickens leads to significant declines in egg production and quality, making it a major cause of non-infectious mortality. Therefore, studying the molecular control of fatty liver in chickens is scientifically valuable and economically important, offering potential for molecular breeding to reduce the condition."<br>A1.2: We have modified these sentences as the reviewer suggested. See lines 89-94.<br>1.3.Line98-104—provide specific details and key findings in your research, rather than the general description. (i.e., doi.org/10.1016/j.xcrm.2024.101431)<br>A1.3: This section has been revised. See lines 95-104.<br><br><br>Response to comments from Anonymous Referee #2<br>2.1.In addition, not sure, for example, “from 4.9 to 7.4”, is it mean or median value? This

applies to all the mentions that were based on Wilcoxon tests.
-----shall indicate in the manuscript.
A2.1: The specific meaning of the values in this aspect has been interpreted. See lines 112-126.
2.2.Figure 1H shows that HTG performed better in the eHSC model compared to the HSC model (Figure 1B). However, the results of eCon and eHS-I regarding HCF in the eHSC model performed worse than in the HSC model (Figure 1C). Please provide an explanation for this phenomenon.
-----what I meant was to interpret for biological findings.
A2.2: The phenomenon described could be attributed to several factors. The eHSC model's stricter criteria for classification may lead to a more accurate representation of extreme hepatic steatosis cases (eCtrl and eHS-II), as individuals in these groups exhibit more pronounced lipid droplet accumulation. However, this strictness may also result in a less precise classification of intermediate cases (eCtrl and eHS-I), where the boundaries between groups become more ambiguous. Moreover, HTG levels may be a more sensitive indicator of extreme hepatic steatosis compared to HCF. HTG specifically measures the amount of triglycerides in the liver, which are directly related to the presence of lipid droplets. In contrast, HCF may encompass a wider range of lipid species, some of which may not be as closely linked to the severity of hepatic steatosis.
2.3.At the first glance, I thought “it is quite a stretch to derive the three regulation routes from three aspects (fig. 3A), unless authors can provide more data evidence.” Then, I realized that authors explained the three aspects one by one afterwards. This arrangement is a bit strange and confusing, lack of signal words. Authors shall think a better way or order to represent the data.
-----still lack of signal words, and the three aspects are mingled with other sections. Authors shall definitely figure out a better way to present this in a structured manner.
A2.3: This part of results was restructured to enhance the clarity and logical coherence. See lines 219-297.
2.4.In fig. 3b, why to not use scientific notations.
----- on the basis of 10, using “e” to represent 10 is not a formal representation
A2.4: Fig. 3 was updated, and scientific notations were used in Fig. 3B to 3H.
2.5.In fig. 3, the representation of “0, 1, 2” on X-axis shall be put in the figure legend, and significance of involved comparisons shall be marked with “*” in the figure (this applies to fig 5B and C as well). This will make the figure easier to read.
---- the representation of “0, 1, 2” on X-axis shall be put in the figure legend, i.e., what do “0,1,2” represent?
A2.5: For Figure B-H, 0 refers to the eCtrl group, 1 for eHS-I, and 2 for eHS-II. The representation of “0, 1, 2” on X-axis has been demonstrated in the figure legend. See lines 1185-1186.
2.6.“FDR corrected P-value” is not a standard expression.
---- shall write like “at a FDR of 0.05 by adjusting p-values using the Benjamini-Hochberg (to check whether this is indeed your case) procedure”.
A2.6: We have revised this sentence. See line 644-645.
2.7.Suggest to deposit all the computational scripts to a publicly assessable platform, such as github and code ocean.
---- shall make it happen.
A2.7: Thank you for your suggestion. We have deposited our scripts of eQTL mapping and SMR at Github (https://github.com/FangrenLan97/gigascience).
2.8.It’s interesting that three molecular regulation routes for hepatic steatosis was elucidated through the application of multi-omics integrated analysis methods. However, it remains essential to ascertain the extent to which these pathways, individually or collectively, align with the actual biological context.
---- not properly addressed. This is also relevant to A2.1 where authors make excuse by saying this study merely focused on integrated analysis, however, some types of validation or verification shall be made, could be from literature or from another customized analysis.
A2.8: Thank you for your comments. In our study, we not only integrated multi-omics data to validate the findings but also utilized assay kits to measure diverse biochemical indicators and metabolites for experimental confirmation of the multi-omics results. Specifically, when exploring the relationship between hepatic steatosis and FFA, metabolic profilesrevealed that the quantity of HFFAs increased significantly with the severity of hepatic steatosis (Fig. 3B), which was verified by ELISA assay kits for 673 individuals (Fig. 1C). Moreover, we integrated metabolome data and hepatic ELISA to

	verify the relationship between hepatic steatosis and metabolites (MDA and 4-HNE) derived from the peroxidation of FFA. Besides, biochemical indicators (TG, TC, TBA and FFA) in liver and serum (686 samples for each), coupled with metabolite abundances involved in methionine cycle (folic acid, Met, SAMe, Hcy, SAH) in liver and duodenal chyme (10 samples/group) were determined with ELISA assay kits.<br>2.9.It's Table S2: what headers stand for, and it is sloppy to use "af, allele1", and "E-1" to represent numbers considering that this can be simply written as 0.xx.<br>A2.9: We have modified these headers and used "allele1, allele2, allel1 frequency" instead. See Table S2.
**Additional Information:**	
**Question**	**Response**
Are you submitting this manuscript to a special series or article collection?	No
**Experimental design and statistics**<br><br>Full details of the experimental design and statistical methods used should be given in the Methods section, as detailed in our Minimum Standards Reporting Checklist. Information essential to interpreting the data presented should be made available in the figure legends.<br><br>Have you included all the information requested in your manuscript?	Yes
**Resources**<br><br>A description of all resources used, including antibodies, cell lines, animals and software tools, with enough information to allow them to be uniquely identified, should be included in the Methods section. Authors are strongly encouraged to cite Research Resource Identifiers (RRIDs) for antibodies, model organisms and tools, where possible.<br><br>Have you included the information requested as detailed in our Minimum Standards Reporting Checklist?	Yes
**Availability of data and materials**<br><br>All datasets and code on which the conclusions of the paper rely must be	Yes

Powered by Editorial Manager® and ProduXion Manager® from Aries Systems Corporation

either included in your submission or deposited in publicly available repositories (where available and ethically appropriate), referencing such data using a unique identifier in the references and in the “Availability of Data and Materials” section of your manuscript.<br><br>Have you have met the above requirement as detailed in our Minimum Standards Reporting Checklist?	

Powered by Editorial Manager® and ProduXion Manager® from Aries Systems Corporation

Research article

Manuscript title

Mechanisms of hepatic steatosis in chickens: integrated analysis of the host genome, molecular phenomics and gut microbiome

Running title: Regulatory mechanisms of hepatic steatosis in chickens

Authors

Congjiao Sun[1†*], Fangren Lan[1†], Qianqian Zhou[1†], Xiaoli Guo[1†], Jiaming Jin[1], Chaoliang Wen[1], Yanxin Guo[1], Zhuocheng Hou[1], Jiangxia Zheng[1], Guiqin Wu[2], Guangqi Li[2], Yiyuan Yan[2], Junying Li[1], Qiugang Ma[1] and Ning Yang[1*]

[1]Department of Animal Genetics and Breeding, College of Animal Science and Technology, China Agricultural University, Beijing 100193, China

[2]Beijing Engineering Research Centre of Layer, Beijing 101206, China

[†] These authors contributed equally to this work.

[*]To whom correspondence should be addressed. nyang@cau.edu.cn; cjsun@cau.edu.cn.

Abstract

Hepatic steatosis is the initial manifestation of abnormal liver functions and often leads to liver diseases such as non-alcoholic fatty liver disease in humans and fatty liver syndrome in animals. In this study, we conducted a comprehensive analysis of a large chicken population consisting of 705 adult hens by combining host genome resequencing, liver transcriptome, proteome, and metabolome analysis, as well as microbial 16S rRNA gene sequencing of each gut segment. The results showed the heritability (h^2=0.25) and duodenal microbiability (m^2=0.26) of hepatic steatosis were relatively high, indicating a large effect of host genetics and duodenal microbiota on chicken hepatic steatosis. Individuals with hepatic steatosis had low microbiota diversity and a decreased genetic potential to process triglyceride output from hepatocytes, fatty acid β-oxidation activity, and resistance to fatty acid peroxidation. Furthermore, we revealed a molecular network linking host genomic variants (GGA6: 5.59–5.69 Mb), hepatic gene/protein expression (*PEMT*, phosphatidyl-ethanolamine N-methyltransferase), metabolite abundances (folate, S-adenosylmethionine, homocysteine, phosphatidyl-ethanolamine, and phosphatidylcholine) and duodenal microbes (genus *Lactobacillus*) to hepatic steatosis, which could provide new insights into the regulatory mechanism of fatty liver development.

Key words: Chickens, Hepatic steatosis, Genetics, Microbiota, Integrative analysis

Introduction

Lipid metabolism plays a crucial role in maintaining animal life and ensuring normal physiological functions. Dysregulations of fat metabolism can lead to fatty liver

diseases, and hepatic steatosis is one of their first symptoms, which can progress to non-alcoholic fatty liver disease (NAFLD) in humans [1] or fatty liver syndrome (FLS) in farm animals with more severe cases developing blood clots and liver rupture [2, 3]. Robust evidence from human studies, especially twin-based studies, has provided in-depth knowledge that fatty liver is strongly influenced by host genetics with heritability ranging from 0.2 to 0.70 [4-7]. Additionally, genetic variations that strongly influence NAFLD in humans have been confirmed by many studies, such as the I148M mutation in the *PNPLA3* gene and the *E167K* mutation in the *TM6SF2* gene [8]. Experts have reached a consensus that metabolic (dysfunction) associated fatty liver disease (MAFLD) may be a more appropriate and inclusive definition than NAFLD in humans [9]. This change emphasizes the significance of lipid metabolism homeostasis, which is involved in hepatic de novo lipogenesis, β-oxidation, very low-density lipoprotein (VLDL) secretion, and gut absorption [10, 11]. Dysregulation of metabolism in any of these processes may cause the development of a fatty liver.

Metabolic disorders are not only regulated by the host itself but also influenced by gut microbiota. Growing evidence supports that gut microbiota play important roles in host lipid metabolism. The liver and intestines communicate bi-directionally through three ways: the bile duct, portal vein and systemic circulation, collectively forming the "gut-liver axis" [12, 13]. Bidirectional crosstalk along the gut-liver axis finely tunes the host's physiological state through reciprocal metabolite exchange and immune responses. Specifically, hepatic steatosis in experimental mice NAFLD was induced by the commensal metabolite phenylacetate, and exacerbated after fecal microbiota transplantation (FMT) from obese women [14]. Trimethylamine N-oxide (TMAO) is

formed from the oxidation of TMA, which is produced first from the dietary precursors such as choline, carnitine by gut microbes and absorbed into the bloodstream [15, 16]. Many clinical studies have evidenced the association of higher TMAO levels with the severity of NAFLD [17], and NASH in T2D [18]. Additionally, commensal-derived D-lactate, supporting clearance of pathogens in hepatic Kupffer cells, was impaired after antibiotic therapy [19]. Gut microbiota ferment indigestible carbohydrates and proteins, thereby yielding metabolites including short-chain fatty acids and succinate that play a crucial role in gut homeostasis and liver substrate metabolism and functions [20-22]. Numerous studies indicated a positive impact of these metabolites in preventing and treating obesity [23, 24]. Dysregulation of gut microbiota influences the development of NAFLD/MAFLD, suggesting a potential causal role of gut microbiota in MAFLD development [25-27]. Therefore, a chicken lipid metabolism regulation model should be established systematically by combining host genetics and gut microbiota as a representation or description of how various components, such as host genetic variations, genes, proteins, metabolites and gut microbes interact and function to control or influence lipid metabolism.

Compared with other animal models, studies on the regulatory mechanism of a fatty liver are more prevalent in humans, especially the population-based design. For farm animals including chickens, research on molecular regulation of a fatty liver is limited [2, 28, 29]. Farm animals are more suitable to analyze the genetic and microbial involvement in a fatty liver by a population-based design. Farm animals have more genetic variations than mouse models whose peculiarity was highly inbred, making

farm animals more representative of the diversity seen in natural populations. Comparative gene mapping has revealed a closer genomic organization between humans and farm animals, compared to the mouse [30]. Additional, animal samples are more accessible than those from humans [31], such as obtaining microbiota from different gut segments in a large population. Chickens are ideal for large-scale studies due to their high reproductive capacity and low cost. Fatty liver in chickens leads to significant declines in egg production and quality, making it a major cause of non-infectious mortality [32, 33]. Therefore, studying the molecular control of fatty liver in chickens is scientifically valuable and economically important, offering potential for molecular breeding to reduce the condition.

In this study, we performed whole-genome resequencing, liver transcriptome and 16S rRNA gene sequencing (duodenum, jejunum, ileum, cecum and feces) on 705 Rhode Island Red chickens, coupled with proteomic and metabolomic sequencing on several selected individuals, to analyze the regulatory networks of hepatic steatosis. The genomic and microbial data were then used to systematically evaluate the contribution of the host genetics (h^2 = 0.25) and gut microbial community (duodenal m^2 = 0.26) to hepatic steatosis. We further identified gene PEMT and duodenal genus Lactobacillus involved in methionine cycle as crucial contributors to hepatic steatosis, which would be beneficial to study NAFLD in farm animal or humans by providing innovative ideas and methods.

Results

Hepatic steatosis classification and phenotype characterization

A total of 686 adult hens were used for hepatic steatosis classification (HSC) in livers with hematoxylin and eosin (H&E)-stained whole sections, and one control (Ctrl, N = 217, healthy) and two hepatic steatosis groups from mild (HS-Ⅰ, N = 265) to severe (HS-Ⅱ, N = 204) were classified in total (Fig. 1A). Hepatic triglyceride (HTG) and serum triglyceride (STG) contents increased with the severity of hepatic steatosis from 0.33 to 0.72 mmol/g in the liver (mean value, Ctrl vs HS-Ⅱ, $P_{adj} < 0.05$, beta = 0.32, post-hoc Wilcoxon rank-sum test) and from 4.9 to 7.4 mmol/L in serum (mean value, Ctrl vs HS-Ⅱ, $P_{adj} < 0.05$, beta = 2.40, post-hoc Wilcoxon rank-sum test, Fig. 1B). Hepatic crude fat (HCF) and hepatic free fatty acids (HFFAs), serum high-density lipoprotein (SHDL), low-density lipoprotein (SLDL) and very low-density lipoprotein (SVLDL) all displayed similar patterns ($P_{adj} < 0.05$, $beta_{HCF} = -14.08$, $beta_{HFFA} = -0.02$, $beta_{SHDL} = -0.12$, $beta_{SLDL} = -0.18$, $beta_{SVLDL} = -0.82$, post-hoc Wilcoxon rank-sum test, Fig. 1C and D). However, hepatic total bile acid (HTBA) exhibited the opposite trend, which decreased from 16.16 to 12.19 mmol/g (mean value, Ctrl vs HS-Ⅱ, $P_{adj} < 0.05$, beta = 2.91, post-hoc Wilcoxon rank-sum test, Fig. 1E). Hepatic total cholesterol did not change with a fatty liver, but serum TC (STC) increased significantly from 1.30 to 1.83 mmol/L (mean value, Ctrl vs HS-Ⅱ, $P_{adj} < 0.05$, beta = -0.34, post-hoc Wilcoxon rank-sum test, Fig. 1F). With the severity of hepatic steatosis, the degree of obesity also

increased with the abdominal fat weight (AFW) increasing from 106.6 to 145.8 g (mean value, Ctrl vs HS-Ⅱ, $P_{adj} < 0.05$, beta = 39.70, post-hoc Wilcoxon rank-sum test, Fig. 1G).

The abovementioned quantitative phenotypes exhibited predominantly positive correlations with HSC, ranging from 0.176 (between HSC and HFFA) to 0.426 (between HSC and HCF) ($P_{adj} < 0.05$, Supplementary Figure S1). STG had a relative high correlation coefficient with SHDL (0.86), SLDL (0.87), SVLDL (0.82), STBA (−0.95), and STC (0.89) ($P_{adj} < 0.05$, Supplementary Figure S1). However, hepatic steatosis is in fact a quantitative trait, making artificial hepatic steatosis classification imprecise for intermediate individuals between each of two adjacent HSC groups. Hence, we further established an extreme hepatic steatosis classification (eHSC) model with more strict criteria, consisting of three groups: an eCtrl group (N = 30, without lipid droplets), eHS-Ⅰ group (N = 30, lipid droplets accounted for 30%−40% of HE-stained images), and eHS-Ⅱ (N = 30, lipid droplets accounted for > 90% of HE-stained images), which were verified by oil red O staining. Among eHSC groups, lipid-related indicators, such as HTG and HCF quantities, exhibited even greater differences; that is, two to three times higher in the eHS-Ⅱ group than in the eCtrl group (Fig. 1H, $P_{adj} < 0.01$, $beta_{HTG} = -0.66$, $beta_{HCF} = -18.29$, post-hoc Wilcoxon rank-sum test, other indicators see Supplementary Fig. S2). eHSC as a complementary model of HSC may aid in identifying crucial molecules for hepatic steatosis.

Genetic determinants of hepatic steatosis

Genomic variants

To investigate the influence of host genetics on hepatic steatosis, we performed whole genome resequencing of 686 chickens. Up to 1.94 Tb of clean reads were generated, and each individual reached an 8.13-fold depth and 95.06% genome coverage. After stringent filtering, a final set of 5,904,820 SNPs (6.17 SNPs per kb) was obtained (Supplementary Table S1). Estimation of SNP-based heritability (h^2) was performed on fat metabolism and storage-related traits. AFW had the highest h^2 (0.48), followed by HSC (0.25) and serum HTG (0.20), indicating that host genetics had a substantial role in determination of fat accumulation and storage (Fig. 2A). Next, we performed GWA analysis of HSC with the abdominal fat percentage (AFP) as an additional covariate and identified two genomic peaks (GGA6: 5.59–5.69 Mb; GGA4: 75.6–76.4 Mb) that were significantly associated with HSC. Additionally, the P-values of the top SNPs rs731375960 at GGA6 (6:5594550) and rs739419162 at GGA4 (4:75758710) were all less than 3.32×10^{-7} (Fig. 2B, Supplementary Table S2). Individuals with different genotypes of top variants had significantly different HSC ratios ($P_{adj} < 0.05$, chi-squared test, Fig. 2C).

eVariants of hepatic steatosis

Genes that harbor or are near to genomic peaks are candidate genes for target traits, which is known as cis-regulation. However, in many cases, genomic variants do not

regulate their physically adjacent genes, but genes far away and even in other chromosomes (trans-regulation). Thus, we used transcriptome data to further identify genes (eGenes) regulated by these two genomic regions. After quality control, the clean data of 668 liver samples varied between 5.24 and 10.51 G for each individual, and the expression of 12,191 genes was quantified in total, of which 10,171 genes were annotated successfully. After eQTL mapping by tensorQTL, 7,468 genes (217 cis- and 7431 trans-regulated genes) were screened for their regulation by genomic variants (Supplementary Fig. S3). We then applied the summary data-based Mendelian randomization (SMR) method to GWAS summary datasets of HSC, and the SMR test showed that two genes (*NUDT14* and *EIF5A2*) were significantly associated with the genomic peak in GGA4 and three genes (*PEMT*, *TOM1L2*, and *GSTM3*) with their genomic peak in GGA6 ($P_{adj} < 0.05$, FDR correction for SMR, Supplementary Table S3). For verification, using the colocalization strategy, we performed thousands of fastGWA runs for all hepatic genes, and 4,171 genes (eGenes) were finally identified for expression that was significantly regulated by at least one genomic variant (eVariant, P-value of the top SNPs $< 3.32\times10^{-7}$). Among these genes, two protein-coding genes, *PEMT* (4,792,705–4,830,291 bp) and *TOM1L2* (4,914,681–4,925,733 bp), at GGA 14 were identified again for their significant association with the genomic region on GGA6, 5.59–5.69 Mb, harboring the same variant rs731375960 that was significantly associated with HSC (Fig. 2B, $P < 3.32\times10^{-7}$). However, no significantly genes were identified for their association with the genomic region of GGA4, 75.6–76.4 Mb.

Furthermore, among the genotypes of the top variant rs731375960, only expression of the *PEMT* gene differed significantly (post-hoc Wilcoxon rank-sum test, beta = -371.91 for genotype GG to AA, Fig. 2D).

Crucial genes for hepatic steatosis

To ascertain the relationship between gene expression and hepatic steatosis, Spearman's rank-based correlation (SRC) analysis was performed between the expression of each gene and hepatic steatosis classifications. We identified 98 and 119 genes that were significantly positively and negatively correlated with hepatic steatosis, respectively, after FDR correction ($P_{adj} < 0.05$, FDR correction, Supplementary Table S4), including trans-eGenes *PEMT* and *TOM1L2*. Considering that correlation analysis employs linear models, ANOVA analysis was also performed among three hepatic steatosis groups. Then, 98 significantly differentially expressed genes were screened ($P_{adj} < 0.05$, FDR correction), 83 of which (84.7%) were consistent with the genes identified by SRC (Fig. 2E). GO and pathway enrichment analyses indicated that these genes were mostly involved in the biological process of lipid localization and the biosynthetic process (Supplementary Fig. S4 and Table S5). Among 232 candidate genes derived from the union of SRC and ANOVA results, 48 were reported to be involved in hepatic steatosis, NAFLD, or hepatic lipid metabolism in humans and rats, indicating the reliability of our results (Table 1 and Supplementary Table S6), 15 of which were part of the 83 genes consistently identified by both Spearman's rank

correlation analysis and ANOVA. We further identified genomic variants (eVariants) that regulated the expression of these 48 genes in fastGWA datasets, and found that 20 genes were significantly regulated by at least one eVariant, all of which were trans-regulated (Table 1). In eHSC groups, 8,632 annotated genes were identified for their significantly differential expression ($P_{adj} < 0.05$, FDR correction, Supplementary Table S7).

Multiple omics data reveal the molecular regulation mechanism for hepatic steatosis

To further ascertain the genetic mechanisms of hepatic steatosis, we complemented our molecular phenome coverage by profiling hepatic proteome and metabolome of individuals from eHSC groups (7 samples/group), and quantified 4,961 proteins and 1,005 metabolites in total (Supplementary Table S8 and S9). On the basis of the integrative analysis from genome to metabolome data, we summarized the regulation routes from three aspects (Fig. 3A).

1) Inability of VLDL to effectively transport TG outward

Hepatic steatosis was characterized by the severe accumulation of triglyceride (TG). Our analysis uncovered that HTG increased significantly with the severity of hepatic steatosis ($P_{adj} < 0.05$, beta = 0.32, post-hoc Wilcoxon rank-sum test, Fig. 1B). In general, the generated TGs are transported extra-hepatically via VLDLs together with cholesterol and apolipoproteins, and impaired hepatic PC biosynthesis can significantly reduce VLDL synthesis and secretion [34]. Our results showed the PC,

phosphatidyl-ethanolamine (PE) and the PC/PE ratio significantly decreased with the severity of hepatic steatosis ($P_{adj} < 0.05$, $beta_{PC} = 5282.95$, $beta_{PC/PE} = 0.47$, post-hoc Wilcoxon rank-sum test, Fig. 3B). Endogenous PC is synthesized from PE by *PEMT* gene, which was down-regulated in hepatic steatosis groups and shared the same regulatory genomic variants with hepatic steatosis classification in our GWA analysis (Fig. 2B). Furthermore, PC can also be generated from dietary choline, which involves three genes, choline kinase (*CHKA*), phosphate cytidylyltransferase 1 (*PCYT1A*), and choline phosphotransferase 1 (*CHPT1*). The expression of these genes was significantly higher in the control than the steatosis group (3–5 fold change in eHSC, $P_{adj} < 0.05$, $beta_{CHKA} = 63.84$, $beta_{PCYT1A} = 11.07$, $beta_{CHPT1} = 2.57$, post-hoc Wilcoxon rank-sum test, Fig. 3C), leading to a significant decrease of PC production in the steatosis group. Correspondingly, the *APOB* gene, which encodes the primary apolipoprotein for VLDL synthesis, was also dramatically decreased along with hepatic steatosis (13.4 times higher in the eCtrl group than eHS-II group, $P_{adj} < 0.05$, beta = 4483.77, post-hoc Wilcoxon rank-sum test, Fig. 3C). Hence, we proposed that the downregulation of *PEMT*, *CHKA*, *PCYT1A* and *CHPT1* collectively led to the reduction of PC levels, consequently affecting the synthesis of VLDL. This impaired VLDL production hindered TG outward transport, resulting in its excessive accumulation and the progression of hepatic steatosis (Fig. 3A, route 1).

2) Weak activity of β-oxidation inhibits fatty acids (FAs) use

Notably, another characteristic of hepatic steatosis is the accumulation of FFAs. Our metabolic profiles revealed that the quantity of HFFAs increased significantly with the severity of hepatic steatosis ($P_{adj} < 0.05$, beta = -74085990, post-hoc Wilcoxon rank-sum test, Fig. 3B), which was verified by an ELISA (Fig. 1C). Besides being utilized in the synthesis of triglycerides (TG), FFAs primarily serve as a source of energy supply through β-oxidation activity. Long-chain fatty acids (LCFAs) are first catalyzed by acyl-CoA synthetases (*ACSL5,4,3*) to form acyl-CoA, which are further synthesized to long-chain acylcarnitines (LCACs) by carnitine palmitoyltransferase 1A (*CPT1A*), a rate-limiting enzyme for β-oxidation. Our results showed the expression of *ACSL5,4,3* genes significantly decreased with the severity of hepatic steatosis, and the rate-limiting enzyme *CPT1A* was almost not expressed in steatotic livers ($P_{adj} < 0.05$, beta = 45.17, post-hoc Wilcoxon rank-sum test, 67 times higher in eCtrl than eHS-Ⅱ). Correspondingly, the metabolome data revealed significantly high presence of LCACs in steatotic livers ($P_{adj} < 0.05$, beta = -8329448, post-hoc Wilcoxon rank-sum test, Fig. 3D), while short-chain acylcarnitines (SCACs) showed the opposite trend ($P_{adj} < 0.05$, beta = 6842755, post-hoc Wilcoxon rank-sum test, Fig. 3D). Additionally, the other two crucial β-oxidation-related genes showed similar expression patterns, including *SLC25A20* (solute carrier family 25 member 20), which is responsible for the transport of LCACs from cytosol to the mitochondrial matrix ($P_{adj} < 0.05$, beta = 150.60, post-hoc Wilcoxon rank-sum test, Fig. 3E), and *CPT2* (carnitine palmitoyltransferase 2), which catalyzes the opposite reaction with *CPT1A* from LCACs to acyl-CoA (P_{adj} <

0.05, beta = 53.34, post-hoc Wilcoxon rank-sum test, Fig. 3E). Therefore, the severely high LCACs and extremely low expression of *CPT1A*, *SLC25A20* and *CPT2* indicated the suspension of β-oxidation activity in the HS group, leading to the accumulation of FFAs (Fig. 3A, route 2).

3) Oxidative stress of FFA accompanied by steatosis

When the synthesis of TG from FFA and β-oxidation of FFA is blocked (Fig. 3A route 1 and 2), we found excessive accumulated FFA might undergo the oxidation process, further aggravating the progression of hepatic steatosis. Specifically, the gene of superoxide dismutase 1/2(SOD1/2) was highly expressed in steatosis groups ($P < 0.05$, Fig. 3F), this gene can convert superoxide (O_2^-) to hydrogen peroxide (H_2O_2) and oxygen (O_2). In addition, the catalase (*CAT*) gene responsible for conversion of H_2O_2 to H_2O and O_2, and glutathione peroxidase 3 (*GPX3*) gene responsible for catalyzing the reduction of organic hydroperoxides and H_2O_2 by glutathione were both significantly downregulated in steatotic livers ($P_{adj} < 0.05$, $beta_{CAT} = 654.79$, $beta_{GPX3} = -383.55$, post-hoc Wilcoxon rank-sum test, Fig. 3F). Correspondingly, glutathione (GSH), as the most important antioxidant, was also extremely low in steatotic livers ($P_{adj} < 0.05$, $beta_{GSH\text{-}O} = 33619000$, $beta_{GSH\text{-}R} = 441270$, post-hoc Wilcoxon rank-sum test, Fig. 3G). Subsequently, we quantified the end products of lipid peroxidation, namely malondialdehyde (MDA) and 4-hydroxynonenal (HNE), using ELISAs, and found their significantly higher presence in steatotic livers ($P_{adj} < 0.05$, $beta_{MDA} = 0.01$,

$\text{beta}_{4\text{-HNE}}$ = -0.64, post-hoc Wilcoxon rank-sum test, Fig. 3G), verifying the high activity of lipid peroxidation. The metabolic data also revealed that oxidized lipids, such as 9- and 13-hydroxy-octadecadienoic acid (9-HODE, 13-HODE) and 9- and 13-oxo-octadecadienoic acid (9-oxoODE, 13-oxoODE) converted from linoleic acid, were five to seven times higher in the steatosis group than in the control ($P_{adj} < 0.05$, $\text{beta}_{9\text{-HODE}}$ = -12735.45, $\text{beta}_{13\text{-HODE}}$ = -389758, $\text{beta}_{9\text{-oxoODE}}$ = -66776.85, $\text{beta}_{13\text{-oxoHODE}}$ = -66776.85, post-hoc Wilcoxon rank-sum test, Fig. 3H), further illustrating the state of oxidative stress on FAs. The simultaneous upregulation of peroxidases *CAT* and *GPX3* and the downregulation of antioxidant enzymes GSH, accompanied by elevated levels of MDA, HNE, HODE, and oxoODE, jointly indicate a close relationship between the peroxidation of FFA and hepatic steatosis (Fig. 3A, route 3).

Microbiome signatures of hepatic steatosis and their association with host genetics

Fat metabolism has long been thought to be regulated by both genetics and gut microbiota. Hence, 16S rRNA gene sequencing was performed in the duodenum, jejunum, ileum, cecum, and feces of 705 chicken, resulting in 174.2 million quality-filtered sequences from 3,430 samples with an average of 49,497 reads (Supplementary Table S10). Then, 6,087 (duodenum), 5,987 (jejunum), 3,751 (ileum), 3,215 (cecum), and 7,428 (faces) ASVs were identified with 100% sequence identity in each gut segment. Alpha diversities exhibited significant differences among the five sampling sites, with the cecum displaying the highest diversity and the ileum the lowest. Subsequently, Principal Coordinates Analysis (PCoA) was employed to visualize

variations in microbial composition across these diverse sites. A distinct divergence in the gut microbial community was observed among the intestines, clustering separately. At the phylum level, the three segments of the small intestine (SI) harbored similar dominant microbial communities, with Firmicutes being the predominant phylum, followed by Proteobacteria, Bacteroidetes, and Actinobacteria. However, noticeable differences were evident in the cecum, where Bacteroidetes (53.91%) and Firmicutes (36.83%) constituted the most abundant phyla, respectively. At the genus level, *Lactobacillus* represented the majority of genera in the duodenum (46.06%), jejunum (54.25%), and feces (22.02%), while *Romboutsia* exhibited the highest abundance (30.32%) in the ileum, and *Bacteroides* constituted a notable fraction (30.04%) in the cecum.

Microbiability estimation

Analogous to heritability, the relative proportion of the total variance due to the gut microbial community is defined as microbiability (m^2), which allows estimation of the effect of microbiota as a whole on host traits. Hence, we first employed m^2 with ASV data to dissect the contributions of microbiota in each gut segment to hepatic steatosis-related phenotypes. The m^2 of HSC in the duodenum (0.26) was much higher than that in other anatomical sites (0.03 for the jejunum, 0.08 for ileum, 0.02 for cecum, and 0.07 for feces), suggesting more important roles of duodenal microbes in the progression of hepatic steatosis (Supplementary Table S11 and Fig. 4A). Similar results

were observed for FFA and TBA contents in the liver (0.15 and 0.19 for the duodenum respectively, and almost zero in other segments). However, for HCF and HTG contents, cecal microbiota (m^2 of 0.27 and 0.20 in cecum) played a more critical role than that in other segments (Fig. 4A). To validate the reliability of the estimated m^2, a permutation test was performed for the relatively high m^2 by randomly reordering the phenotypes 1,000 times. The results showed that the actual m^2 (0.15~0.27) was significantly higher than the simulated m^2 (average m^2 ranged from 0.02 to 0.04, $P < 0.05$, Supplementary Fig. S5A).

Identification of crucial microbes for hepatic steatosis

Using high-quality ASVs, 52 phyla, 161 classes, 467 orders, 1003 families, 2329 genera, and 3930 species were successfully classified (Supplementary Fig. S5B). The Shannon and Simpson Indices for alpha diversity revealed that the microbiota diversity decreased with steatosis progression ($P_{adj} < 0.05$, $beta_{Shannon} = 0.37$, $beta_{Simpson} = 0.03$, post-hoc Wilcoxon rank-sum test, Supplementary Fig. S5C). To evaluate the association between gut microbiota and hepatic steatosis, SRC analysis was performed between the abundance of microbial taxa in each gut segment and HSC. Base on the significantly greater microbiability estimates in the duodenum, we focused on the duodenal microbiota and significantly associated taxa (72 in total) with HSC were almost observed in the duodenum, including 6 phyla, 6 classes, 9 orders, 16 families, 14 genera, and 21 species ($P_{adj} < 0.05$, FDR correction, supplementary Table S12). For

other gut segments, only two taxa, the family Staphylococcaceae and genus *Staphylococcus* in the ileum, were significantly positively associated with HSC ($P_{adj} <$ 0.05, FDR correction). Among these 72 taxa, four taxa chains from the phyla to genus level were identified for their association with HSC, including chains harboring the genera *Lactobacillus*, *Bacteroides*, *Sediminibacterium*, and *Cutibacterium* (Fig. 4B). Additionally, genera *Lactobacillus* and *Bacteroides* were verified by LEfSe analysis for their significantly differential presence among HSC groups (LDA = 4.7 and 3.8 respectively, $P_{adj} < 0.01$, Kruskal–Wallis test, Fig. 4C). Genus *Lactobacillus* was positively correlated with HSC (41.1% in Ctrl, 47.5% and 52.0% in HS-Ⅰ and HS-Ⅱ, $P_{adj} < 0.05$), while genus *Bacteroides* showed the opposite trend (2.09% in Ctrl, 1.70% and 1.55% in HS-Ⅰ and HS-Ⅱ, $P_{adj} < 0.05$). Additionally, LEfSe analysis verified the differential presence of many other taxa (LDA > 3.0, $P_{adj} < 0.01$, Kruskal–Wallis test), including phyla Bacteroidetes, Firmicutes, and Actinobacteria, and classes Bacilli, Actinobacteria, and Bacteroidia, orders Bacteroidales and Chitinophagales, families Lactobacillaceae, Ruminococcaceae, Bacteroidaceae, Prevotellaceae, and Chitinophagaceae, genera *Lactobacillus* and *Bacteroides*, and species *Lactobacillus vaginalis*. The abovementioned microbial taxa had a high detection rate, many of which exhibited a high correlation with the phenotypes (Fig. 4D and Supplementary Fig. S6). Except for HSC, many microbes were negatively correlated with HTG and HCF levels, including genera *Prevotella7*, *Dialister*, *Ruminococcaceae UCG-014*, *Helicobacter*, *Butyricicoccus*, *Acinetobacter*, *Coprococcus 2*, *Dorea*, *Odoribacter*, *Parabacteroides*,

and *Faecalibaculum* (Fig. 4D, for other taxa levels, see Supplementary Fig. S6).

Lactobacillus might play compensatory roles in alleviating hepatic steatosis

By analyzing metabolome/transcriptome data from duodenum and liver samples, a significant enrichment of metabolites and genes associated with the methionine cycle was observed between the healthy and hepatic steatosis groups (Fig. 5A), suggesting a potential link between the abundance of *Lactobacillus* in the duodenum of individuals with hepatic steatosis and their involvement in the host methionine cycle. Notably, we observed a 1.2-fold increase in folic acid production by the duodenal microbiota in the eHS-II group compared to the eCtrl group ($P_{adj} < 0.05$, beta = -1.08, post-hoc Wilcoxon rank-sum test), as validated by an ELISA (Fig. 5B). Examining methionine cycle-related genes, including methionine adenosyltransferase (MAT), PEMT, adenosylhomocysteinase (AHCY), and 5-methyltetrahydrofolate-homocysteine methyltransferase (MTR), revealed their significantly lower expression levels in hepatic steatosis individuals. The eCtrl group exhibited 2.1–22.5 times higher expression than the eHS-II group. Furthermore, ELISA results unveiled a significant accumulation of S-adenosylmethionine (SAMe) and homocysteine (Hcy) in the liver samples of the eHS-II group compared to the eCtrl group (Fig. 5C, $P_{adj} < 0.05$, $beta_{SAMe} = -1.61$, $beta_{Hcy} = 1.32$, post-hoc Wilcoxon rank-sum test). SAMe is converted to S-adenosylhomocysteine (SAH) by specific methyltransferases (MTs) such as GNMT, GAMT, and PEMT. However, only the *PEMT* gene was expressed in the liver in our

study as demonstrated in Figure 2. Interestingly, PEMT also catalyzes conversion of PE to PC, and its low expression led to accumulation of SAMe and PE. Hcy can be remethylated to regenerate methionine by *MTR* (22.5 times higher in eCtrl than eHS-II) with conversion of methyl tetrahydrofolate (MTHF) to tetrahydrofolate (THF). Hence, additional folic acid is essential to prompt the final step of the methionine cycle. Although these results should be interpreted carefully, it can be hypothesized that the extra folate produced by duodenal microbiota may be absorbed by the host to reduce Hcy accumulation, resulting in comparable hepatic folic acid contents in healthy and hepatic steatosis groups (Fig. 5B).

Discussion

In this study, we used a chicken population consisting of 705 well-phenotyped individuals to investigate genetic variants, crucial hepatic molecules, and their interactions with intestinal microbiota in the development of hepatic steatosis. Hepatic steatosis is a quantitative trait, making artificial hepatic steatosis classification imprecise for intermediate individuals between each of two adjacent HSC groups. Hence, in addition to HSC model, we further established the eHSC model, which used more stringent criteria for hepatic steatosis classification with smaller variance in each eHSC group.

Many studies have estimated the heritability of hepatic steatosis or NAFLD in humans with the values ranged from 0.20 to 0.70 [5, 35, 36], which is comparable to

our results (0.25). Hepatic steatosis is generally characterized by accumulation of lipid droplets [37], which is supported by the increased HTG and HCF along with hepatic steatosis in our study. However, our genetic analysis revealed that hepatic steatosis and fat accumulation did not share the same genetic determinants. We proposed that fat accumulation should be viewed as a symptom of hepatic steatosis rather than as its actual cause. With SRC and ANOVA analysis, 227 candidate genes were screened for their association with hepatic steatosis, 48 of which have been reported to be involved in NAFLD or hepatic lipid metabolism in humans and mice. For example, *NAPEPLD* in hepatocytes is an important regulator of liver bioactive lipid synthesis. The hepatocyte-specific *Napepld* deletion mouse develops a high fat diet-like phenotype characterized by increased fat mass gain and hepatic steatosis [38]. *CYP1A1* metabolizes benzo[a]pyrene, resulting in either detoxication or metabolic activation in a context-dependent manner. Loss of the *CYP1A1* gene protects against non-alcoholic fatty liver disease caused by a Western diet containing benzo[a]pyrene in mice [39]. Interestingly, in contrast to previous studies of humans and cattle [40, 41], in which the majority of genes were cis-regulated by eVariants in most tissues, in this study, most genes expressed in the liver, including candidate genes for hepatic steatosis, were trans-regulated by genomic variants; that is, genes and their corresponding eVariants were on different chromosomes. This regulatory pattern was identified by both tensorQTL [42] and fastGWA [43] methods, indicating a unique gene regulation pattern in chickens, which requires additional study to determine whether this is the case in other species or

tissues.

Combined with multi-omics data and the validation of crucial metabolites, we found abnormalities in three regulatory routes in host hepatocytes that contribute to hepatic steatosis. First, low expression of the *PEMT* gene led to a decrease in PC production, which further reduced VLDL synthesis and secretion. Previous studies have demonstrated beneficial roles of PC in protecting against hepatic steatosis [44], and observed the clinical feature of a low PC/PE ratio in livers of NAFLD patients [45]. In this study, we not only found a decrease in endogenous PC production due to the *PEMT* gene, but also a dramatic decrease in the synthesis of exogenous PC from dietary choline, which further aggravated outward translocation of liver fat. Second, β-oxidation activity was very weak in mitochondria of HS individuals. A previous study has also demonstrated that lipid accumulation in the liver can be traced by impaired fatty acid β-oxidation [46]. Here, we found that the main genes in the β-oxidation pathway, including *ACSL5,4,3*, *CPT1A*, *SLC25A20*, and *CPT2*, were all expressed at extremely low levels in the HS-II group compared with the Ctrl group. This resulted in significantly elevated and decreased levels of LCACs and SCACs, respectively, in the HS-II group, which may serve as biomarkers for hepatic steatosis.

To the best of our knowledge, no study has reported the m^2 of hepatic steatosis in humans or any animal model. Our study proposes that the crucial regulatory microbiota for hepatic steatosis mainly exist in the duodenum because of its much higher m^2 of HSC (0.26) than other gut segments (near zero). Indeed, the anterior part of the small

intestines is the main site for fat digestion and absorption [47]. Previous studies to identify hepatic steatosis or NAFLD-related microbiota in humans or mouse models have mainly focused on fecal microbiota. However, fecal microbiota cannot represent the composition and abundance of microorganisms in each gut segment [48].In the duodenum, the abundance of genus *Lactobacillus* varies greatly among individuals of different hepatic steatosis grades. Genus *Lactobacillus*, which is commonly considered as probiotic bacteria, provides numerous health benefits to the host, including folate production [49], which was consistent with our results indicating that individuals with hepatic steatosis had a higher abundance of genus *Lactobacillus* and higher quantity of folate in their duodenum. Folate prompts the final step of the methionine cycle from Hcy to Met. Mice fed a methionine-choline-deficient diet develop non-alcoholic fatty liver disease with severe steatohepatitis [50], and addition of folate reduces the incidence of hepatic steatosis [51]. Moreover, SAMe and Hcy accumulation increases the incidence of fatty liver diseases [52], corroborating our results of higher Hcy abundances in individuals with hepatic steatosis.

Conclusion

We identified regulatory networks among genomic variants (GGA6: 5.59–5.69 Mb), gene expression (*PEMT* and *TOM1L2*), protein presence (PEMT), and metabolite abundance (SAMe, SAH, PC, PE, and Hcy) in hepatic steatosis and proposed for the first time that duodenal microbiota (m^2 = 0.26) played more important roles in hepatic

steatosis than other gut segments, and genus *Lactobacillus* in the duodenum might perform compensatory roles in alleviating hepatic steatosis by production of extra folate to prompt the host methionine cycle. This integrated analysis of host genomic variations, the hepatic transcriptome, proteome, metabolome, and gut microbiome provided a comprehensive understanding of the host genetic and gut microbial factors for hepatic steatosis and novel insights into mechanistic analysis of human NAFLD.

Material and Methods

Experimental Design

The experimental cohorts used in this study comprised a total of 705 hens from a pedigreed line of Rhode Island Red in Beijing Huadu Yukou Poultry Breeding Co., Ltd. (China). Birds were generated from two batches with a ten-day interval and reared in individual cages under similar conditions with 16L:8D (16 hours of light and 8 hours of dark). These birds were fed a basic corn-based diet (details were listed in Table 2) and provided with free access to feed and water. No antibiotics were administered to the hens in our study.

Table 2. Ingredients and nutrient composition of diets

Item	Composition
**Ingredients, % as feed**	
Corn	61.7
Soybean meal	24
Wheat bran	3.8
Limestone	8
Premix[1]	2.5
**Metabolism energy (kcal/kg)**	2630

**Nutrients, % DM**	
Crude protein	15.9
Calcium	3.5
Total phosphorus	0.36
Lysine	0.79
Methionine	0.38

Note: [1] Premix provided the following per kilogram of diet: vitamin A 9975 IU, vitamin B_{12} 0.03 mg, vitamin E 78.4 IU, vitamin VD_3 4200 IU, riboflavin 3.8 mg, niacin 50.1 mg, calcium pantopantolate 18.3 mg, biotin 0.35 mg, Iron 58 mg, zinc 101 mg, copper 10 mg, manganese 96 mg and choline chloride 480 mg.

At 90 weeks of age, body weight was measured using an electronic scale to the nearest 5 g. Blood samples were collected from the wing vein and stored at −20 °C. Serum was separated by centrifugation at 3,000 × *g* for 15 min and stored at −20 °C until use. Fecal samples were manually collected from the rectum with sterile cotton swabs. Each bird was then euthanized by cervical dislocation followed by decapitation. The contents of the duodenum, jejunum, ileum, and cecum, including the chyme and mucosa, were immediately collected after opening the abdomen. All intestinal samples were dispensed into 2 ml tubes, snap frozen in liquid nitrogen, and stored at −80 °C.

The weights of the liver and abdominal fat tissue surrounding the gizzard, cloaca, and adjacent abdominal muscles were to the nearest 1 g with the electronic scale. Subsequently, some the liver tissue sample was frozen in liquid nitrogen and stored at −80 °C immediately after collection for genomic DNA, tissue RNA, protein, and metabolite extraction. Some of the liver was placed in a sterile plastic bag on dry ice using forceps and stored at −20 °C to measure biochemical indicators. The remaining liver sample was fixed in formalin for 48–72 h for histological observation.

All experiments involving animals were conducted according to the ethical

policies and procedures approved by the Institutional Animal Care and Use Committee of China Agricultural University, China (Issue No.32303202-1-1)

Liver histological assessment

Liver histology was assessed in liver sections embedded in paraffin and stained with hematoxylin and eosin (H&E) using standard techniques. Whole section images of each liver sample were obtained using a Canon EOS 7D digital camera (Canon, Tokyo, Japan) and quantified using Image J (ver 1.8.0, National Institutes of Health, Bethesda, MD, USA). The investigators were blind to the group allocations. Therefore, a veterinary science pathologist performed the hepatic steatosis assessment using the NASH Clinical Research Network Scoring System in humans [53]. All liver samples were graded from 0 to 2, representing healthy individuals, and mild–moderate and severe hepatic steatosis. Individuals were categorized into three levels based on the severity of fatty liver: lipid droplets constituting less than 10% of the area in the control group (Ctrl), 10% to 50% in mild–moderate group (HS-I), and more than 50% in severe group (HS-II). For extremely typical individuals that could represent each group (nearly no lipid droplets in Ctrl, 30% - 40% in HS-I and over 90% lipid droplets in HS-II), oil red O staining was performed to verify lipid droplet accumulation.

Measurement of biochemical indicators in Serum

Serum triglycerides (STGs), cholesterol (STC), high-density lipoprotein cholesterol (SHDL), low-density lipoprotein cholesterol (SLDL), and total bile acids

(STBAs) were analyzed using commercial kits (Shanghai Kehua Bioengineering Co., Ltd., Shanghai, China) with the KHB ZY-1280 automatic biochemical analyzer (Shanghai Kehua Bioengineering Co., Ltd.). Serum very low-density lipoprotein (SVLDL) was measured using a chicken very low-density lipoprotein ELISA kit (JLC10779) in accordance with the manufacturer's instructions (Shanghai Kehua Bioengineering Co., Ltd., Shanghai, China).

Measurement of biochemical indicators in the liver

Crude fat (CF) content measurement. The CF content was measured by the soxhlet extraction method (AOAC 920.85) and performed with a soxhlet apparatus by refluxing with petroleum ether to remove CF in the sample. The difference between the weights of the initial sample and residue was the CF content.

Protein quantitation. The livers were taken out of the −80°C freezer, temporarily stored in liquid nitrogen, trimmed into small pieces with scissors, and weighed ranging from 0.01g−0.05g on an electronic scale. Protein quantification was performed using a protein quantification kit (A045-2) from Nanjing Jiancheng Institute of Biological Engineering Ltd. according to the manufacturer's instructions.

Hepatic biochemical indicator assay. On the basis of protein quantification, hepatic triglycerides (HTG), total cholesterol (HTC), free fatty acids (HFFAs), and total bile acids (HTBAs) were measured using the Triglyceride Assay Kit (A110-2-1) from Nanjing Jiancheng Institute of Biological Engineering Co., Ltd. according to the manufacturer's instructions.

Whole-genome resequencing and data processing

Genomic DNA was isolated from liver samples of 705 hens using a Tiangen DNA Extraction Kit (Tiangen Biotech, Beijing, China, DP304-2) according to the manufacturer's instructions. After purification and integrity verification of the DNA, a total of 686 DNA samples were used for subsequent whole-genome resequencing. Host DNAs were amplified using PCR with 500 bp inserts for library construction. Whole-genome resequencing was performed using the Illumina HiSeq 2500 Sequencer (Illumina, Inc., San Diego, CA, USA) to generate 150 bp paired-end reads. To ensure the quality of data, the adaptor-polluted reads, low-quality reads and reads with number of N bases accounting for more than 5 % were removed. The clean reads were then mapped to the chicken reference genome (GRCg6a) using the Burrows–Wheeler aligner (BWA, ver 0.7.15) [54] with the default parameters. We subsequently used Samtools (ver 1.3.1) [55] to sort reads and remove low quality reads with the parameter "-q 4". Duplicate reads resulting from PCR were removed using Picard tools (http://broadinstitute.github.io/picard/). The HaplotypeCaller protocol in Genome Analysis Toolkit (GATK, ver 4.2.0.0) [56] was used for SNPs and indels calling. To obtain high-quality SNPs, the SNPs were filtered with GATK VariantFiltration protocol as follows: $QD < 2.0$, $ReadPosRankSum < -8.0$, $FS > 60.0$, $QUAL < 30.0$, $DP < 4.0$, $MQ < 40.0$, $MappingQualityRankSum < -12.5$ and INDEL: $QD < 2.0$, $ReadPosRankSum < -20.0$, $FS > 200.0$, $QUAL < 30.0$, $DP < 4.0$. Finally, the PLINK (ver 1.90) [57] was used for filtering annotated SNP data with the following parameters:

sample call rate > 90%, SNP call rate > 90% and minor allele frequencies > 1%. The remaining SNPs and individuals were used for imputation in BEAGLE (ver 5.1) [58], and the PLINK analysis was reperformed using the same criteria as above-described. After these steps, a total of 5,904,820 SNPs distributed across 32 chromosomes and 686 birds were retained for subsequent analysis.

Genome-wide analysis study (GWAS)

To reveal the impact of host genetics on shaping the phenotypes, all valid individuals and SNPs were involved in GWAS with a univariate linear mixed model (LMM), which were performed using GEMMA (ver 0.98.4) [59]. The statistical model applied in this study is as follows:

$$y = W\alpha + x\beta + u + \varepsilon$$

where y is the phenotypic values of 686 individuals; W is a matrix of covariates (fixed effects: top five principal components and batch effects) controlling for population structure, while α refers to a vector of corresponding effects that compose the intercept; x denotes the marker genotypes, while β is the corresponding marker's effect; u is a vector of random polygenic effects with a covariance structure; and ε is vector of random residuals.

The likelihood ratio test *P*-value was selected as a criterion for examining the significance of the association between SNPs and phenotypes. The genome-wide significant threshold was determined using a modified Bonferroni correction with an R package named simpleM as previously described [60]. Using this approach, a total of

150,802 valid inspections were obtained, and thereby the genome-wide significance and suggestive significance thresholds were defined as 3.32×10^{-7} (0.05/150802) and 6.63×10^{-6} (1/150802), respectively.

For the sake of exploring the effects of host genetics on the gene expression in liver tissue, GWAS were conducted in Genome-Wide Complex Trait Analysis (GCTA) software (ver 1.93.2) [61] with the support of fastGWA [43]. Out of 686 samples, 668 transcriptome-sequenced subjects were included for subsequent genome and gene expression association analysis in our study. Read counts were normalized using TPM initially. Genes were selected based on the expression thresholds of ≥ 0.1 TPM and ≥ 6 reads (unnormalized) in ≥ 20% samples. Afterwards, read counts were normalized between samples using TMM. Eventually, 12,191 genes were remained in this part. Likewise, a LMM was employed throughout the analysis. For fastGWA, a full-dense genetic relationship matrix (GRM) was generated, based on which a sparse GRM was built at a cutoff value of 0.05. fastGWA was then run using this sparse GRM with expression level as the dependent variable and SNP genotype values as the independent variable. Significant and suggestively significant P-value thresholds were 3.32×10^{-7} and 6.63×10^{-6} as described above.

eQTL mapping and mendelian randomization analysis

For each gene, we took all genetic variants into consideration, and used the following covariates: top 5 genetic principal components, batch effects and top 3 PEER factors. The number of PEER factors included in calculation equaling to 60 was

determined from the sample size corresponding to the previously reported researches: 15 for $n < 150$, 30 for $150 \leq n < 250$, 45 for $250 \leq n < 350$, 60 for $n \geq 350$ [62]. Thereafter, the permutations of cis-QTL mapping were conducted to generate phenotype-level summary statistics with empirical *P*-value, and trans-QTL mapping to compute nominal associations between all phenotypes and genotypes. Notably, the cis-window referred to ranging from 1Mbps upstream to 1Mbps downstream of the transcription start sites (TSS), while for trans-QTL mapping, 5,315,471 common genetic variants passing strict quality control criteria (MAF > 5% and outside of TSS ± 5 Mbps) were contained in the process. Correction for multiple testing was done using FDR for cis-eQTL analysis, resulting in a *P*-value threshold of 8.05×10^{-6} for cis-eQTLs. Given that the remaining SNPs used in trans-QTL mapping was analogous to those in GWAS, we considered its *P*-value threshold as 3.32×10^{-7}.

Subsequently, top-eQTL based Summary-data-based Mendelian Randomization (SMR) [63] analysis was performed to prioritize genes underlying GWAS associations. The panel in Fig. 6A illustrated possible causal relationships between a SNP, a transcript (RNA) and a phenotype [64]. Gene expression functions as the casual link between an eQTL genetic variant and the phenotype associated with that variant. Nonetheless, the observed correlation might arise from another two alternative relationships: a reactive connection between the phenotype and gene expression, or an independent relationship between the genetic variant and both the phenotype and gene expression. Top-eQTL based SMR analysis uses SNPs as instrumental variables to assess causal relationships

between a risk factor (gene expression levels) and an outcome (HSC). Large-scale eQTL mapping were conducted to identify SNPs associated with gene expression levels. From the pool of identified eQTLs, the most significant ones (top eQTLs) based on statistical criteria were selected. The independence of instrumental variables from each other is crucial to meet the assumptions of Mendelian Randomization. Evaluate the degree of LD between SNPs, removing SNPs in high LD with the top associated eQTL. SMR uses an instrumental variable estimation in order to accurately integrate independent GWAS and eQTL summary data (Fig. 6B). The SMR procedure consists of two steps: i) identification of variants independently associated with the exposure factor, and ii) calculation of causal estimates. Before that, we made a BESD file and updated coordinates of SNPs and genes, frequency of effect allele. For each GWAS summary statistic, SNPs significantly and suggestively significantly associated with the traits were selected as SMR input files to determine the connection with significant cis-eQTLs and trans-eQTLs. Finally, we selected only variants that showed association at a FDR of 0.05 by adjusting p-values using the Benjamini-Hochberg procedure.

16S rRNA gene sequencing

The gut digesta (duodenum, jejunum, ileum and cecum) and fecal samples of 705 individuals were thawed on ice and homogenized, and ~200 mg of each sample was used to extract the microbial genome DNA using the QIAamp Stool Mini Kit (QIAGEN, Hilden, Germany, D4015-01) according to the manufacturer's recommendations. The hypervariable V4 region of the 16S gene was amplified using the Ion Plus Fragment

Library Kit 48 rxns (Thermo Scientific). Sequencing was performed on an Ion S5TM XL platform, and 400 bp single-end reads were generated, in accordance with the manufacturer's instructions. Sequences were imported and processed using Quantitative Insights Into Microbial Ecology (QIIME2, ver 2019.10) [65] for further bioinformatics analyses. After trimming the barcode and primer sequences, the preliminary quality screening was performed for the original high-throughput sequencing data using the QIIME2 plugin DADA2 [66] and the sequences were trimmed to a final length of 252 bp. The remaining high-quality sequences were clustered and classified by amplicon sequence variants (ASVs) with 100% identity [67]. ASVs that presented in less than 1% (seven) samples and had an average relative abundance below 10^{-6} were removed for subsequent analyses. Taxonomic assignments for each ASV were made via similarity searching against the SILVA 16S rRNA gene sequence reference database (Release 132) [68]. The alpha and beta diversity were calculated with the vegan package [69].

Heritability and microbiability estimation

The 5,904,820 filtered SNPs were used to construct genetic relatedness matrix (GRM) using GCTA software (ver 1.93.2) [61]: The GRM estimation model used was:

$$g_{ij} = \frac{1}{N}\sum_{V=1}^{N}\frac{(Xiv-2\overline{pv})(xjv-2\overline{pv})}{2\overline{pv}(1-\overline{pv})}$$

In this expression, g_{ij} denotes the genetic relationship between individuals i and j; x_{iv} and x_{jv} denote the number of reference alleles in hens i and j, respectively; p_v denotes the reference allele frequency; and N is the SNP number. The SNP-based heritability of

the host phenotypes was estimated with the following model:

$$Y = Kc + g + e$$

In this expression, y denotes a vector of the phenotype; c denotes a vector of fixed covariates (including batch effect and the first ten host genetic principal components); K denotes the corresponding matrix for c; and g denotes a vector of the total effects of all SNPs with ~ N (0, Gσ^2A), where G and Gσ^2A denote the GRM and genetic variance, respectively; and e denotes the residual effect.

The phenotypic variance explained by gut microbial variance is defined as microbiability (m^2) in animals [70, 71] and it was estimated with GCTA software using the microbial relationship matrix (MRM). The construction of the microbial relationship matrix and phenotypic variance explained by the gut microbial variance were estimated as described in our previous study [72]. We corrected batch effects and the first five host genetic principal components in this analysis. All filtered ASVs in duodenum were normalized by zero-centering and scaling to unit variance to construct the MRM as previously described with an R script based on the following equation:

$$m_{sij} = \frac{1}{N_s}\sum_{a=1}^{N_s} \frac{(x_{sia}-\overline{x_{sa}})(x_{sjo}-\overline{x_{sa}})}{\sigma_s^2}$$

where m_{sij} represents the estimated microbial relationship in the sampling site *s* between birds *i* and *j*; x_{sia} and x_{sja} donate the relative abundances of ASV *a* in the sampling site *s* in birds *i* and *j*, respectively; $\overline{x_{sa}}$ stands for the average relative abundance of the ASV *a* in the sampling site *s* in the population; σ_s^2 is the variance of the abundance of ASV *a*; and *Ns* is the total number of ASVs in the sampling site *s* used

for the relatedness computation.

Liver tissue transcriptome

A total of 686 samples were used for transcriptome sequencing. The Eastep® Super Total RNA Extraction Kit (Promega, Shanghai, China, LS1040) was used to extract total RNA according to the manufacturer's instructions. The RNA concentration and purity were determined using the NanoDrop ND-2000 spectrophotometer (Thermo Fisher Scientific, Waltham, MA, USA). The integrity of the RNA as assessed using the RNA Nano 6000 Assay Kit of the Bioanalyzer 2100 system (Agilent Technologies, CA, USA). Libraries for transcriptome sequencing were constructed following the standard Illumina RNA-seq instruction. The libraries were sequenced on an Illumina Novaseq platform and 150 bp paired-end reads were generated. Fastp (ver 0.20.1) [73] was used to remove the reads containing adaptor contamination, low quality bases and undetermined bases. Then, the quality-controlled sequencing data were aligned to the chicken reference genome (GRCg6a) using HISAT2 (ver 2.0.5) [74] with default parameters. After that, we employed featureCounts (ver 1.6.3) [75] to count the reads for each gene. The differentially expressed genes between different groups were identified with the assistance of DESeq2 (ver 3.16) [76]. The significance threshold for the differential expression was adjusted P-value < 0.05 and a $|\log2 \text{ fold change }| > 1$.

Liver tissue proteome

For protein extraction and digestion, chicken liver tissues (4 samples/group from

eHSC groups) were ground with liquid nitrogen into cell powder and transferred to a 5-mL centrifuge tube. The protein concentration was determined with BCA kit (Thermo Fisher Scientific, 23225) according to the manufacturer's instructions and protein digestion was conducted just as Song et al. illustrated [77]. Subsequent TMT labeling, HPLC Fractionation and LC-MS/MS analysis of the TMT- labeled peptides were performed as previously described, too [77]. Automatic gain control (AGC) target was set at 1×10^{-5}, with an intensity threshold of 3.3×10^{-4} and a maximum injection time of 60 ms.

Downstream database search was performed using MaxQuant search engine (v.1.6.15.0). Tandem mass spectra were searched against the *Gallus gallus* database (27535 entries) concatenated with reverse decoy database. Trypsin/P was specified as cleavage enzyme allowing up to 2 missing cleavages. The mass tolerance for precursor ions was set as 20 ppm in first search and 5 ppm in main search, and for fragment ions was set as 0.02 Da, respectively. Carbamidomethyl on Cys was specified as fixed modification, and acetylation on protein N-terminal and oxidation on Met were specified as variable modifications. The threshold of FDR adjusted *P*-value was set to 0.01.

Widely targeted metabolome

Livers were thawed in a 50 μL ice-cold mixture (Methanol : Water = 7:3, V/V) and homogenized after adding a 150 μL solution (Methanol : Water = 7:3, V/V) containing internal standard. Placed the sample on ice for 15 min, and centrifuged it at 12000 rpm

for 10 min (4 °C). The collected supernatant was placed in −20 °C for 30 min and then centrifuged at 12000 rpm for 3 min (4 °C), followed by transferring about 120 μL aliquots of supernatant. The solution obtained was analyzed using an LC-ESI-MS/MS system (UPLC, ExionLC AD, https://sciex.com.cn/; MS, QTRAP® System, https://sciex.com/). The chromatographic separation was achieved by using water and acetonitrile (with 0.1% formic acid for each) as the mobile phase. The elution gradient program was 95:5 V/V at 0 min, 10:90 V/V at 11.0 min, 10:90 V/V at 12.0 min, 95:5 V/V at 12.1 min, 95:5 V/V at 14.0 min with the 0.40 mL/min of flow rate. The column temperature was 40°C, and the injection volume was 2 μL.

LIT and triple quadrupole scans were acquired on a triple quadrupole-linear ion trap mass spectrometer (QTRAP), QTRAP® LC-MS/MS System, equipped with an ESI Turbo Ion-Spray interface, operating in positive and negative ion mode and controlled by Analyst 1.6.3 software (Sciex). The ESI source operation parameters and successor operations followed Chen's parameters [78].

Significantly regulated metabolites between groups were determined by VIP (VIP ≥ 1), *P*-value (*P*-value < 0.05) and absolute log2FC (|log2FC| ≥ 1.0). VIP values were generated from OPLS-DA result using R package MetaboAnalystR. The data was log transform (log2) and mean centering before OPLS-DA. In order to avoid overfitting, a permutation test (200 permutations) was performed.

Liver tissue and duodenal mucosa assays

To validate the results of the metabolome, we randomly chose 10 individuals in

each group (eCtrl, eHS-I, and eHS-II), and the levels of folic acid (FLA), homocysteine (Hcy), methionine (Met), S-adenosine homocysteine (SAH), and S-adenosylmethionine (SAM) were determined in liver tissue and duodenal chyme samples by ELISA kits (Enzyme-linked Biotechnology Co. Ltd., Shanghai, China) in accordance with the manufacturer’s instructions.

Statistical Analysis

Differences in phenotypes among HSC groups were determined in R version 4.0.2 [79] using the Wilcoxon rank sum test with a post-hoc test to correct for multiple comparisons. Analysis of variance (ANOVA) was performed to determine differences in liver gene expression (TPM), and biochemical indicators among HSC groups with FDR correction for multiple testing.

Spearman’s rank coefficient of correlations was calculated between the relative abundance of duodenal microbiota of various taxonomic levels ranging from phylum to species and HSC. Prior to this, the relative abundance of the microorganisms that presented in ≥ 60% of the population equaling to 0 was converted to NA, because it was considered to escape from detection. Otherwise, microorganisms detected in < 60% and ≥ 30% of samples were dichotomized into presence/absence patterns and we encoded the phenotype as a binary vector to prevent zero inflation, which led to a bimodal distribution. Microorganisms detected in < 30% of samples were excluded from this analysis as reported previously [72, 80]. Furthermore, Linear discriminant analysis effect size (LEfSe) [81] was employed to identify differentially abundant

microbial taxa from the phylum to genus level within HSC groups. The analysis incorporated linear discriminant analysis (LDA) to quantify the effect size of each taxon. Taxa with high LDA scores were considered more influential in distinguishing between the HSC groups. The LEfSe analysis followed specific conditions: 1) the alpha value for the factorial Kruskal-Wallis test was set to less than 0.05; 2) the alpha value for the pairwise Wilcoxon test the taxonomic compositions was set to less than 0.05; 3) the threshold on the logarithmic LDA score for discriminative features was set to less than 2.0; 4) multiclass analysis was configured as all-against-all.

The relationship between hepatic biochemical indicators and the relative abundance of bacteria with a call rate ranging from 30% to 60% was measured using the Spearman correlation, whereas the Pearson correlation coefficient was employed to test the strength of the association between hepatic biochemical indicators and the relative abundance of bacteria in $\geq$ 60% of samples. Correlations between the TPM value and HSC were also calculated using the Spearman method. FDR corrections were carried out in all of the abovementioned analysis.

Declarations

Ethics approval and consent to participate

All experiments involving animals were conducted according to the ethical policies and procedures approved by the Institutional Animal Care and Use Committee of China Agricultural University, China (Issue No.32303202-1-1)

Availability of data and materials

Whole-genome resequencing data are available on the NCBI Sequence Read Archive (SRA) under accession SUB13720715, and RNA-Seq data are available on the SRA under the accession SUB13062074. 16S rRNA sequencing data can be accessed on SRA under the accession SUB12033010 (duodenum), SUB12035295 (jejunum), SUB12035349 (ileum), SUB12035378 (cecum) and SUB12035409 (feces). Raw data for metabolomics were submitted to MetaboLights at MTBLS7808. And raw data for proteomics were submitted toPRIDE at PXD042451.

Competing interests

The authors declare no competing interests.

Funding

This work was supported by the National Natural Science Foundation of China (No. 31930105), National Key Research and Development Program of China (2022YFF1000204), China Agriculture Research Systems [CARS-40] and the 2115 Talent Development Program of China Agricultural University.

Authors' contributions

NY and CS conceived the study, participated in the experiment design and critical discussion, and jointly supervised this work. CS, FL, QZ and XG performed the experiments and wrote the manuscript. CS, QZ, FL, CW, YG, ZH, JZ, GW, GL, YY, JL

and QM participated in the management of the experimental animals and the sample collection. QZ, FL and JJ contributed to the measurement of biochemical traits, ELISA and qPCR analysis. CS, FL, QZ and XG conducted bioinformatics and statistical analysis. NY, CS and FL designed the figures and tables. NY and CS were responsible for critical revisions of the manuscript drafts. All authors read and approved the final manuscript.

Acknowledgements

We are grateful to Mr. Hao Sun and Huadu Yukou Poultry Co., Ltd. for the assistance in providing experimental chickens.

Reference

[1] N Bhat, A Mani. Dysregulation of Lipid and Glucose Metabolism in Nonalcoholic Fatty Liver Disease. Nutrients 2023;15(10) https://doi.org/10.3390/nu15102323.

[2] X D Tan, R R Liu, Y H Zhang, X C Wang, J Wang, H L Wang, . . . J Wen. Integrated analysis of the methylome and transcriptome of chickens with fatty liver hemorrhagic syndrome. Bmc Genomics 2021;22(1) https://doi.org/ARTN 8
10.1186/s12864-020-07305-3.

[3] C W Lin, T W Huang, Y J Peng, Y Y Lin, H J Mersmann, S T Ding. A novel chicken model of fatty liver disease induced by high cholesterol and low choline diets. Poult Sci 2021;100(3) 100869. https://doi.org/10.1016/j.psj.2020.11.046.

[4] L Miele, V Giorgio, A Liguori, S Petta, R Pastorino, D Arzani, . . . M Biolato. Genetic susceptibility of increased intestinal permeability is associated with progressive liver disease and diabetes in patients with non-alcoholic fatty liver disease. Nutrition, Metabolism and Cardiovascular Diseases 2020;30(11) 2103-2110.

[5] E K Speliotes, L M Yerges-Armstrong, J Wu, R Hernaez, L J Kim, C D Palmer, . . . G Consortium. Genome-Wide Association Analysis Identifies Variants Associated with Nonalcoholic Fatty Liver Disease That Have Distinct Effects on Metabolic Traits. Plos Genet 2011;7(3) https://doi.org/ARTN e1001324
10.1371/journal.pgen.1001324.

[6] R Loomba, N Schork, C H Chen, R Bettencourt, A Bhatt, B Ang, . . . N i T C Genetics of. Heritability

of Hepatic Fibrosis and Steatosis Based on a Prospective Twin Study. Gastroenterology 2015;149(7) 1784-93. https://doi.org/10.1053/j.gastro.2015.08.011.

[7] M C Brouwers, M M van Greevenbroek, R M Cantor. Heritability of nonalcoholic fatty liver disease. Gastroenterology 2009;137(4) 1536. https://doi.org/10.1053/j.gastro.2009.03.065.

[8] J T Wu, S S Liu, X J Xie, Q Liu, Y N Xin, S Y Xuan. Independent and joint correlation of PNPLA3 I148M and TM6SF2 E167K variants with the risk of coronary heart disease in patients with non-alcoholic fatty liver disease. Lipids Health Dis 2020;19(1) 29. https://doi.org/10.1186/s12944-020-01207-9.

[9] M Eslam, A J Sanyal, J George, I C Panel. MAFLD: A Consensus-Driven Proposed Nomenclature for Metabolic Associated Fatty Liver Disease. Gastroenterology 2020;158(7) 1999-+.

[10] H Kim, D S Lee, T H An, H J Park, W K Kim, K H Bae, K J Oh. Metabolic Spectrum of Liver Failure in Type 2 Diabetes and Obesity: From NAFLD to NASH to HCC. Int J Mol Sci 2021;22(9) https://doi.org/ARTN 4495
10.3390/ijms22094495.

[11] L F Ramos, C M Silva, C C Pansa, K C M Moraes. Non-alcoholic fatty liver disease: molecular and cellular interplays of the lipid metabolism in a steatotic liver. Expert Rev Gastroent 2021;15(1) 25-40. https://doi.org/10.1080/17474124.2020.1820321.

[12] C L Hsu, B Schnabl. The gut-liver axis and gut microbiota in health and liver disease. Nat Rev Microbiol 2023;21(11) 719-733. https://doi.org/10.1038/s41579-023-00904-3.

[13] A Tripathi, J Debelius, D A Brenner, M Karin, R Loomba, B Schnabl, R Knight. The gut-liver axis and the intersection with the microbiome. Nat Rev Gastroenterol Hepatol 2018;15(7) 397-411. https://doi.org/10.1038/s41575-018-0011-z.

[14] L Hoyles, J M Fernandez-Real, M Federici, M Serino, J Abbott, J Charpentier, . . . M E Dumas. Molecular phenomics and metagenomics of hepatic steatosis in non-diabetic obese women. Nat Med 2018;24(7) 1070-1080. https://doi.org/10.1038/s41591-018-0061-3.

[15] Z Wang, E Klipfell, B J Bennett, R Koeth, B S Levison, B Dugar, . . . S L Hazen. Gut flora metabolism of phosphatidylcholine promotes cardiovascular disease. Nature 2011;472(7341) 57-63. https://doi.org/10.1038/nature09922.

[16] W H Tang, Z Wang, B S Levison, R A Koeth, E B Britt, X Fu, . . . S L Hazen. Intestinal microbial metabolism of phosphatidylcholine and cardiovascular risk. N Engl J Med 2013;368(17) 1575-84. https://doi.org/10.1056/NEJMoa1109400.

[17] Y M Chen, Y Liu, R F Zhou, X L Chen, C Wang, X Y Tan, . . . H L Zhu. Associations of gut-flora-dependent metabolite trimethylamine-N-oxide, betaine and choline with non-alcoholic fatty liver disease in adults. Sci Rep 2016;6 19076. https://doi.org/10.1038/srep19076.

[18] P Leon-Mimila, H Villamil-Ramirez, X S Li, D M Shih, S T Hui, E Ocampo-Medina, . . . S Canizales-Quinteros. Trimethylamine N-oxide levels are associated with NASH in obese subjects with type 2 diabetes. Diabetes Metab 2021;47(2) 101183. https://doi.org/10.1016/j.diabet.2020.07.010.

[19] B McDonald, A Z Zucoloto, I L Yu, R Burkhard, K Brown, M B Geuking, K D McCoy. Programing of an Intravascular Immune Firewall by the Gut Microbiota Protects against Pathogen Dissemination during Infection. Cell Host Microbe 2020;28(5) 660-668 e4. https://doi.org/10.1016/j.chom.2020.07.014.

[20] E E Canfora, R C R Meex, K Venema, E E Blaak. Gut microbial metabolites in obesity, NAFLD

and T2DM. Nat Rev Endocrinol 2019;15(5) 261-273. https://doi.org/10.1038/s41574-019-0156-z.

[21] D Dodd, M H Spitzer, W Van Treuren, B D Merrill, A J Hryckowian, S K Higginbottom, . . . J L Sonnenburg. A gut bacterial pathway metabolizes aromatic amino acids into nine circulating metabolites. Nature 2017;551(7682) 648-+. https://doi.org/10.1038/nature24661.

[22] L P Zhao, F Zhang, X Y Ding, G J Wu, Y Y Lam, X J Wang, . . . C H Zhang. Gut bacteria selectively promoted by dietary fibers alleviate type 2 diabetes. Science 2018;359(6380) 1151-+. https://doi.org/10.1126/science.aao5774.

[23] M C Dao, A Everard, J Aron-Wisnewsky, N Sokolovska, E Prifti, E O Verger, . . . K Clement. Akkermansia muciniphila and improved metabolic health during a dietary intervention in obesity: relationship with gut microbiome richness and ecology. Gut 2016;65(3) 426-36. https://doi.org/10.1136/gutjnl-2014-308778.

[24] E Corpeleijn, W H Saris, E E Blaak. Metabolic flexibility in the development of insulin resistance and type 2 diabetes: effects of lifestyle. Obes Rev 2009;10(2) 178-93. https://doi.org/10.1111/j.1467-789X.2008.00544.x.

[25] L Hoyles, J M Fernandez-Real, M Federici, M Serino, J Abbott, J Charpentier, . . . M E Dumas. Molecular phenomics and metagenomics of hepatic steatosis in non-diabetic obese women (vol 24, pg 1070, 2018). Nat Med 2018;24(10) 1628-1628. https://doi.org/10.1038/s41591-018-0169-5.

[26] N Yao, Y X Yang, X T Li, Y X Wang, R R Guo, X H Wang, . . . W W Cui. Effects of Dietary Nutrients on Fatty Liver Disease Associated With Metabolic Dysfunction (MAFLD): Based on the Intestinal-Hepatic Axis. Front Nutr 2022;9 https://doi.org/ARTN 906511
10.3389/fnut.2022.906511.

[27] M M Zhao, L Zhao, X L Xiong, Y He, W Huang, Z H Liu, . . . L M Zheng. TMAVA, a Metabolite of Intestinal Microbes, Is Increased in Plasma From Patients With Liver Steatosis, Inhibits gamma-Butyrobetaine Hydroxylase, and Exacerbates Fatty Liver in Mice. Gastroenterology 2020;158(8) 2266-+. https://doi.org/10.1053/j.gastro.2020.02.033.

[28] X D Tan, R R Liu, S Y Xing, Y H Zhang, Q H Li, M Q Zheng, . . . J Wen. Genome-Wide Detection of Key Genes and Epigenetic Markers for Chicken Fatty Liver. Int J Mol Sci 2020;21(5) https://doi.org/ARTN 1800
10.3390/ijms21051800.

[29] M T Tsai, Y J Chen, C Y Chen, M H Tsai, C L Han, Y J Chen, . . . S T Ding. Identification of Potential Plasma Biomarkers for Nonalcoholic Fatty Liver Disease by Integrating Transcriptomics and Proteomics in Laying Hens. J Nutr 2017;147(3) 293-303. https://doi.org/10.3945/jn.116.240358.

[30] L Andersson. Genetic dissection of phenotypic diversity in farm animals. Nat Rev Genet 2001;2(2) 130-8. https://doi.org/10.1038/35052563.

[31] D Dickson. Human tissue samples more difficult to obtain for academics. Nat Med 2002;8(6) 543. https://doi.org/10.1038/nm0602-543a.

[32] A Mete, F Giannitti, B Barr, L Woods, M Anderson. Causes of Mortality in Backyard Chickens in Northern California: 2007-2011. Avian Dis 2013;57(2) 311-315. https://doi.org/DOI 10.1637/10382-092312-Case.1.

[33] K A Trott, F Giannitti, G Rimoldi, A Hill, L Woods, B Barr, . . . A Mete. Fatty liver hemorrhagic syndrome in the backyard chicken: a retrospective histopathologic case series. Vet Pathol 2014;51(4) 787-95. https://doi.org/10.1177/0300985813503569.

[34] L K Cole, J E Vance, D E Vance. Phosphatidylcholine biosynthesis and lipoprotein metabolism.

Biochim Biophys Acta 2012;1821(5) 754-61. https://doi.org/10.1016/j.bbalip.2011.09.009.

[35] N D Palmer, S K Musani, L M Yerges-Armstrong, M F Feitosa, L F Bielak, R Hernaez, . . . E K Speliotes. Characterization of European Ancestry Nonalcoholic Fatty Liver Disease-Associated Variants in Individuals of African and Hispanic Descent. Hepatology 2013;58(3) 966-975. https://doi.org/10.1002/hep.26440.

[36] L E Wagenknecht, A L Scherzinger, E R Stamm, A J G Hanley, J M Norris, Y D I Chen, . . . J I Rotter. Correlates and Heritability of Nonalcoholic Fatty Liver Disease in a Minority Cohort. Obesity 2009;17(6) 1240-1246. https://doi.org/10.1038/oby.2009.4.

[37] Q Liu, S Bengmark, S Qu. The role of hepatic fat accumulation in pathogenesis of non-alcoholic fatty liver disease (NAFLD). Lipids Health Dis 2010;9 https://doi.org/Artn 42
10.1186/1476-511x-9-42.

[38] C Lefort, M Roumain, M Van Hul, M Rastelli, R Manco, I Leclercq, . . . P D Cani. Hepatic NAPE-PLD Is a Key Regulator of Liver Lipid Metabolism. Cells-Basel 2020;9(5) https://doi.org/10.3390/cells9051247.

[39] S Uno, D W Nebert, M Makishima. Cytochrome P450 1A1 (CYP1A1) protects against nonalcoholic fatty liver disease caused by Western diet containing benzo[a]pyrene in mice. Food Chem Toxicol 2018;113 73-82. https://doi.org/10.1016/j.fct.2018.01.029.

[40] K G Ardlie, D S DeLuca, A V Segre, T J Sullivan, T R Young, E T Gelfand, . . . G Consortium. The Genotype-Tissue Expression (GTEx) pilot analysis: Multitissue gene regulation in humans. Science 2015;348(6235) 648-660. https://doi.org/10.1126/science.1262110.

[41] S Liu, Y Gao, O Canela-Xandri, S Wang, Y Yu, W Cai, . . . L Fang. A comprehensive catalogue of regulatory variants in the cattle transcriptome. bioRxiv 2020; 2020.12.01.406280. https://doi.org/10.1101/2020.12.01.406280.

[42] A Taylor-Weiner, F Aguet, N J Haradhvala, S Gosai, S Anand, J Kim, . . . G Getz. Scaling computational genomics to millions of individuals with GPUs. Genome Biology 2019;20(1) 228. https://doi.org/10.1186/s13059-019-1836-7.

[43] L D Jiang, Z L Zheng, T Qi, K E Kemper, N R Wray, P M Visscher, J Yang. A resource-efficient tool for mixed model association analysis of large-scale data. Nat Genet 2019;51(12) 1749-+. https://doi.org/10.1038/s41588-019-0530-8.

[44] L J Niebergall, R L Jacobs, T Chaba, D E Vance. Phosphatidylcholine protects against steatosis in mice but not non-alcoholic steatohepatitis. Biochim Biophys Acta 2011;1811(12) 1177-85. https://doi.org/10.1016/j.bbalip.2011.06.021.

[45] B M Arendt, D W L Ma, B Simons, S A Noureldin, G Therapondos, M Guindi, . . . J P Allard. Nonalcoholic fatty liver disease is associated with lower hepatic and erythrocyte ratios of phosphatidylcholine to phosphatidylethanolamine. Appl Physiol Nutr Me 2013;38(3) 334-340. https://doi.org/10.1139/apnm-2012-0261.

[46] S H Koo. Nonalcoholic fatty liver disease: molecular mechanisms for the hepatic steatosis. Clin Mol Hepatol 2013;19(3) 210-5. https://doi.org/10.3350/cmh.2013.19.3.210.

[47] C Feinle, T Rades, B Otto, M Fried. Fat digestion modulates gastrointestinal sensations induced by gastric distention and duodenal lipid in humans. Gastroenterology 2001;120(5) 1100-7. https://doi.org/10.1053/gast.2001.23232.

[48] W Yan, C J Sun, J X Zheng, C L Wen, C L Ji, D X Zhang, . . . N Yang. Efficacy of Fecal Sampling as a Gut Proxy in the Study of Chicken Gut Microbiota. Front Microbiol 2019;10

https://doi.org/ARTN 2126
10.3389/fmicb.2019.02126.
[49] M Rossi, A Amaretti, S Raimondi. Folate production by probiotic bacteria. Nutrients 2011;3(1) 118-34. https://doi.org/10.3390/nu3010118.
[50] H Itagaki, K Shimizu, S Morikawa, K Ogawa, T Ezaki. Morphological and functional characterization of non-alcoholic fatty liver disease induced by a methionine-choline-deficient diet in C57BL/6 mice. Int J Clin Exp Pathol 2013;6(12) 2683-96.
[51] R P da Silva, K B Kelly, A Al Rajabi, R L Jacobs. Novel insights on interactions between folate and lipid metabolism. Biofactors 2014;40(3) 277-283. https://doi.org/10.1002/biof.1154.
[52] J M Mato, M L Martinez-Chantar, S C Lu. Methionine metabolism and liver disease. Annu Rev Nutr 2008;28 273-93. https://doi.org/10.1146/annurev.nutr.28.061807.155438.
[53] D E Kleiner, E M Brunt, M Van Natta, C Behling, M J Contos, O W Cummings, . . . N Nonalcoholic Steatohepatitis Clinical Research. Design and validation of a histological scoring system for nonalcoholic fatty liver disease. Hepatology 2005;41(6) 1313-21. https://doi.org/10.1002/hep.20701.
[54] H Li, R Durbin. Fast and accurate short read alignment with Burrows-Wheeler transform. Bioinformatics 2009;25(14) 1754-1760. https://doi.org/10.1093/bioinformatics/btp324.
[55] H Li, B Handsaker, A Wysoker, T Fennell, J Ruan, N Homer, . . . G P D Proc. The Sequence Alignment/Map format and SAMtools. Bioinformatics 2009;25(16) 2078-2079. https://doi.org/10.1093/bioinformatics/btp352.
[56] A McKenna, M Hanna, E Banks, A Sivachenko, K Cibulskis, A Kernytsky, . . . M A DePristo. The Genome Analysis Toolkit: A MapReduce framework for analyzing next-generation DNA sequencing data. Genome Res 2010;20(9) 1297-1303. https://doi.org/10.1101/gr.107524.110.
[57] S Purcell, B Neale, K Todd-Brown, L Thomas, M A R Ferreira, D Bender, . . . P C Sham. PLINK: A tool set for whole-genome association and population-based linkage analyses. Am J Hum Genet 2007;81(3) 559-575. https://doi.org/10.1086/519795.
[58] S R Browning, B L Browning. Rapid and accurate haplotype phasing and missing-data inference for whole-genome association studies by use of localized haplotype clustering. Am J Hum Genet 2007;81(5) 1084-1097. https://doi.org/10.1086/521987.
[59] X Zhou, M Stephens. Genome-wide efficient mixed-model analysis for association studies. Nat Genet 2012;44(7) 821-U136. https://doi.org/10.1038/ng.2310.
[60] X Y Gao, J Stamier, E R Martin. A multiple testing correction method for genetic association studies using correlated single nucleotide polymorphisms. Genet Epidemiol 2008;32(4) 361-369. https://doi.org/10.1002/gepi.20310.
[61] J A Yang, S H Lee, M E Goddard, P M Visscher. GCTA: A Tool for Genome-wide Complex Trait Analysis. Am J Hum Genet 2011;88(1) 76-82. https://doi.org/10.1016/j.ajhg.2010.11.011.
[62] A N Barbeira, R Bonazzola, E R Gamazon, Y Y Liang, Y Park, S Kim-Hellmuth, . . . G Consortium. Exploiting the GTEx resources to decipher the mechanisms at GWAS loci. Genome Biology 2021;22(1) https://doi.org/ARTN 49
10.1186/s13059-020-02252-4.
[63] Z H Zhu, F T Zhang, H Hu, A Bakshi, M R Robinson, J E Powell, . . . J Yang. Integration of summary data from GWAS and eQTL studies predicts complex trait gene targets. Nat Genet 2016;48(5) 481-+. https://doi.org/10.1038/ng.3538.

[64] W R MacLellan, Y Wang, A J Lusis. Systems-based approaches to cardiovascular disease. Nat Rev Cardiol 2012;9(3) 172-84. https://doi.org/10.1038/nrcardio.2011.208.

[65] E Bolyen, J R Rideout, M R Dillon, N A Bokulich, C C Abnet, G A Al-Ghalith, . . . J G Caporaso. Reproducible, interactive, scalable and extensible microbiome data science using QIIME 2 (vol 37, pg 852, 2019). Nat Biotechnol 2019;37(9) 1091-1091. https://doi.org/10.1038/s41587-019-0252-6.

[66] B J Callahan, P J McMurdie, M J Rosen, A W Han, A J A Johnson, S P Holmes. DADA2: High-resolution sample inference from Illumina amplicon data. Nat Methods 2016;13(7) 581-+. https://doi.org/10.1038/Nmeth.3869.

[67] B J Callahan, P J McMurdie, S P Holmes. Exact sequence variants should replace operational taxonomic units in marker-gene data analysis. The ISME journal 2017;11(12) 2639-2643.

[68] C Quast, E Pruesse, P Yilmaz, J Gerken, T Schweer, P Yarza, . . . F O Glockner. The SILVA ribosomal RNA gene database project: improved data processing and web-based tools. Nucleic Acids Res 2013;41(D1) D590-D596. https://doi.org/10.1093/nar/gks1219.

[69] P Dixon. VEGAN, a package of R functions for community ecology. J Veg Sci 2003;14(6) 927-930. https://doi.org/DOI 10.1111/j.1654-1103.2003.tb02228.x.

[70] A Camarinha-Silva, M Maushammer, R Wellmann, M Vital, S Preuss, J Bennewitz. Host Genome Influence on Gut Microbial Composition and Microbial Prediction of Complex Traits in Pigs. Genetics 2017;206(3) 1637-1644. https://doi.org/10.1534/genetics.117.200782.

[71] G F Difford, D R Plichta, P Lovendahl, J Lassen, S J Noel, O Hojberg, . . . G Sahana. Host genetics and the rumen microbiome jointly associate with methane emissions in dairy cows. Plos Genet 2018;14(10) https://doi.org/ARTN e1007580
10.1371/journal.pgen.1007580.

[72] C L Wen, W Yan, C J Sun, C L Ji, Q Q Zhou, D X Zhang, . . . N Yang. The gut microbiota is largely independent of host genetics in regulating fat deposition in chickens. Isme J 2019;13(6) 1422-1436. https://doi.org/10.1038/s41396-019-0367-2.

[73] S F Chen, Y Q Zhou, Y R Chen, J Gu. fastp: an ultra-fast all-in-one FASTQ preprocessor. Bioinformatics 2018;34(17) 884-890. https://doi.org/10.1093/bioinformatics/bty560.

[74] D Kim, B Landmead, S L Salzberg. HISAT: a fast spliced aligner with low memory requirements. Nat Methods 2015;12(4) 357-U121. https://doi.org/10.1038/Nmeth.3317.

[75] Y Liao, G K Smyth, W Shi. featureCounts: an efficient general purpose program for assigning sequence reads to genomic features. Bioinformatics 2014;30(7) 923-930. https://doi.org/10.1093/bioinformatics/btt656.

[76] M I Love, W Huber, S Anders. Moderated estimation of fold change and dispersion for RNA-seq data with DESeq2. Genome Biology 2014;15(12) https://doi.org/ARTN 550
10.1186/s13059-014-0550-8.

[77] Y Song, X Liu, J B Stielow, S de Hoog, R Li. Post-translational changes in Phialophora verrucosa via lysine lactylation during prolonged presence in a patient with a CARD9-related immune disorder. Front Immunol 2022;13 966457. https://doi.org/10.3389/fimmu.2022.966457.

[78] W Chen, L Gong, Z L Guo, W S Wang, H Y Zhang, X Q Liu, . . . J Luo. A Novel Integrated Method for Large-Scale Detection, Identification, and Quantification of Widely Targeted Metabolites: Application in the Study of Rice Metabolomics. Mol Plant 2013;6(6) 1769-1780. https://doi.org/10.1093/mp/sst080.

[79] R C Team. R: A language and environment for statistical computing. MSOR connections 2014;1

[80] J Zierer, M A Jackson, G Kastenmüller, M Mangino, T Long, A Telenti, . . . C Menni. The fecal metabolome as a functional readout of the gut microbiome. Nat Genet 2018;50(6) 790-795. https://doi.org/10.1038/s41588-018-0135-7.

[81] N Segata, J Izard, L Waldron, D Gevers, L Miropolsky, W S Garrett, C Huttenhower. Metagenomic biomarker discovery and explanation. Genome Biol 2011;12(6) R60. https://doi.org/10.1186/gb-2011-12-6-r60.

[82] L Bertran, M Portillo-Carrasquer, C Aguilar, J A Porras, D Riesco, S Martínez, . . . T Auguet. Deregulation of Secreted Frizzled-Related Protein 5 in Nonalcoholic Fatty Liver Disease Associated with Obesity. Int J Mol Sci 2021;22(13) 6895.

[83] Y Cepero-Donates, G Lacraz, F Ghobadi, V Rakotoarivelo, S Orkhis, M Mayhue, . . . S Ramanathan. Interleukin-15-mediated inflammation promotes non-alcoholic fatty liver disease. Cytokine 2016;82 102-11. https://doi.org/10.1016/j.cyto.2016.01.020.

[84] H W Kang, M W Niepel, S Han, Y Kawano, D E Cohen. Thioesterase superfamily member 2/acyl-CoA thioesterase 13 (Them2/Acot13) regulates hepatic lipid and glucose metabolism. FASEB J 2012;26(5) 2209-21. https://doi.org/10.1096/fj.11-202853.

[85] J Yu, C Zhu, X Wang, K Kim, A Bartolome, P Dongiovanni, . . . U B Pajvani. Hepatocyte TLR4 triggers inter-hepatocyte Jagged1/Notch signaling to determine NASH-induced fibrosis. Sci Transl Med 2021;13(599) https://doi.org/10.1126/scitranslmed.abe1692.

[86] Z Jiang, M Zhao, L Voilquin, Y Jung, M A Aikio, T Sahai, . . . J W Knowles. Isthmin-1 is an adipokine that promotes glucose uptake and improves glucose tolerance and hepatic steatosis. Cell metabolism 2021;33(9) 1836-1852. e11.

[87] Y Ma, Q Li, G Chen, Z Tan, H Cao, Y Bin, . . . Z Si. Transcriptomic analysis reveals a novel regulatory factor of ECHDC1 involved in lipid metabolism of non-alcoholic fatty liver disease. Biochem Biophys Res Commun 2022;605 1-8. https://doi.org/10.1016/j.bbrc.2022.03.055.

[88] Z Mao, M Feng, Z Li, M Zhou, L Xu, K Pan, . . . W Zhang. ETV5 regulates hepatic fatty acid metabolism through PPAR signaling pathway. Diabetes 2021;70(1) 214-226.

[89] H Chen, Q Gan, C Yang, X Peng, J Qin, S Qiu, . . . Y Peng. A novel role of glutathione S-transferase A3 in inhibiting hepatic stellate cell activation and rat hepatic fibrosis. J Transl Med 2019;17(1) 280. https://doi.org/10.1186/s12967-019-2027-8.

[90] C Yu, S Jiang, J Lu, C C Coughlin, Y Wang, E A Swietlicki, . . . D C Rubin. Deletion of Tis7 protects mice from high-fat diet-induced weight gain and blunts the intestinal adaptive response postresection. J Nutr 2010;140(11) 1907-14. https://doi.org/10.3945/jn.110.127084.

[91] J Ye, Y Lin, Q Wang, Y Li, Y Zhao, L Chen, . . . T Zhou. Integrated Multichip Analysis Identifies Potential Key Genes in the Pathogenesis of Nonalcoholic Steatohepatitis. Front Endocrinol (Lausanne) 2020;11 601745. https://doi.org/10.3389/fendo.2020.601745.

[92] E N Gurzov, M Tran, M A Fernandez-Rojo, T L Merry, X Zhang, Y Xu, . . . T Tiganis. Hepatic oxidative stress promotes insulin-STAT-5 signaling and obesity by inactivating protein tyrosine phosphatase N2. Cell Metab 2014;20(1) 85-102. https://doi.org/10.1016/j.cmet.2014.05.011.

[93] J Yu, Q Tao, K F Cheung, H Jin, F F Poon, X Wang, . . . M P Ebert. Epigenetic identification of ubiquitin carboxyl- terminal hydrolase L1 as a functional tumor suppressor and biomarker for hepatocellular carcinoma and other digestive tumors. Hepatology 2008;48(2) 508-518.

[94] R Zhai, L Feng, Y Zhang, W Liu, S Li, Z Hu. Combined Transcriptomic and Lipidomic Analysis Reveals Dysregulated Genes Expression and Lipid Metabolism Profiles in the Early Stage of Fatty

Liver Disease in Rats. Front Nutr 2021;8 733197. https://doi.org/10.3389/fnut.2021.733197.

[95] A Marsili, C Aguayo-Mazzucato, T Chen, A Kumar, M Chung, E P Lunsford, . . . A M Zavacki. Mice with a targeted deletion of the type 2 deiodinase are insulin resistant and susceptible to diet induced obesity. PLoS One 2011;6(6) e20832. https://doi.org/10.1371/journal.pone.0020832.

[96] H Sun, F F Huang, S Qu. Melatonin: a potential intervention for hepatic steatosis. Lipids Health Dis 2015;14 75. https://doi.org/10.1186/s12944-015-0081-7.

[97] A Matsuda, Z Wang, S Takahashi, T Tokuda, N Miura, J Hasegawa. Upregulation of mRNA of retinoid binding protein and fatty acid binding protein by cholesterol enriched-diet and effect of ginger on lipid metabolism. Life Sci 2009;84(25-26) 903-7. https://doi.org/10.1016/j.lfs.2009.04.004.

[98] X Wang, J Hasegawa, Y Kitamura, Z Wang, A Matsuda, W Shinoda, . . . K Kimura. Effects of hesperidin on the progression of hypercholesterolemia and fatty liver induced by high-cholesterol diet in rats. J Pharmacol Sci 2011;117(3) 129-38. https://doi.org/10.1254/jphs.11097fp.

Figure legends

Fig. 1. Phenotypic profiling of the histochemical stained sections of the chicken livers and hepatic and serum biochemical indicators among HSC (eHSC) groups.

(**A**) Micrographs of H&E-stained and oil-red-O-stained whole sections of the chicken liver (scale bar: 100μm). n = 673. (**B-D**). Boxplots of hepatic and plasmic triglyceride (HTG and STG, respectively) (B), hepatic crude fat (HCF) and free fatty acids (HFFAs) (C), serum high-density lipoprotein (SHDL), low-density lipoprotein (SLDL) and very low-density lipoprotein (SVLDL) (D), showing high levels in the hepatic steatosis group ($P_{adj} < 0.05$, post-hoc Wilcoxon rank-sum test). n = 673. (**E**) Boxplots of hepatic and serum total bile acids (HTBAs and STBAs, respectively) showing a significant inverse correlation with hepatic steatosis ($P_{adj} < 0.05$, post-hoc Wilcoxon rank-sum test). n = 673. (**F**) Boxplots of the association of hepatic and serum total cholesterol (HTC and STC, respectively) among HSC groups ($P_{adj} < 0.05$ for hepatic TC and $P_{adj} < 0.05$ for serum, post-hoc Wilcoxon rank-sum test). n = 673. (**G**) Boxplots of the abdominal fat weight (AFW), showing a significant increase with hepatic steatosis ($P_{adj} < 0.05$, post-hoc Wilcoxon rank-sum test). n = 673. (**H**) Bar plots of hepatic triglyceride (HTG) and crude fat (HCF), showing significant increases with hepatic steatosis in eHSC groups ($P_{adj} < 0.05$, post-hoc Wilcoxon rank-sum test). Data are from n = 30 biological replicates. *p < 0.05, **p < 0.01, ***p < 0.001.

Fig. 2. Genomic determinants of HSC and its related indicators.

(**A**) The SNP-based heritability estimations of HSC and biochemical indicators. HSC: hepatic steatosis classification; HTG and STG: hepatic and serum triglyceride, respectively; HCF: hepatic crude fat; HFFA: hepatic free fatty acids; SHDL: serum high-density lipoprotein; SLDL: serum low-density lipoprotein; SVLDL: serum very low-density lipoprotein; HTBA and STBA: hepatic and serum total bile acid, respectively; HTC and STC: hepatic and serum total cholesterol, respectively; AFW: abdominal fat weight. $n \geq 673$. (**B**) Circular Manhattan plots of GWAS for HSC, HCF and HTG. Gray, dark blue and reddish-brown dots indicate non-significant, suggestively significant and significant SNPs, respectively. Colocalization of trans-eQTLs of phosphatidylethanolamine N-methyltransferase (*PEMT*) gene in the liver and GWAS loci of HSC in chickens on chromosome 6 identified two colocalized SNPs, which were the significant trans-eQTL of *PEMT* and the top GWAS signals of HSC. $n \geq 668$. (**C**) Stacked bar plots of the comparison of individuals distributing in different HSC levels across the three genotypes of these two eVariant (rs731375960 and rs731375960, $P_{adj} < 0.05$, chi-squared test). $n = 673$. (**D**) Raincloud plot shows the expression levels of the *PEMT* gene in the liver across the three genotypes of the top eVariant (rs731375960, $P_{adj} < 0.05$, post-hoc Wilcoxon rank-sum test). $n = 668$. (**E**) Spearman's rank-based correlation (SRC) analysis and analysis of variance (ANOVA) were performed to ascertain candidate genes in the liver for hepatics steatosis. $n = 673$. $^{*}p < 0.05$, $^{**}p < 0.01$, $^{***}p < 0.001$.

Fig. 3. Multi-omics data reveal the molecular regulation mechanism of hepatic steatosis.

(**A**) Illustration summarizing three regulation routes to hepatic steatosis. To better demonstrate the results, not all enzymes and metabolites in the pathway are shown. (1) the severe accumulation of hepatic free fatty acids (HFFAs) and hepatic triglyceride (HTG), and the decrease of phosphatidylcholine (PC) partially caused by the low expression of phosphatidylethanolamine N-methyltransferase (*PEMT*), impeded the outward transport of TG from hepatocytes; Acyl-CoA synthetase long chain family member 5, 4, 3: *ACSL5*,*4*,*3*, choline kinase alpha: *CHKA*, phosphate cytidylyltransferase 1A: *PCYT1A*, choline phosphotransferase 1: *CHPT1*. (2) Use of HFFA was inefficient because of the weak fatty acid β-oxidation activity; Carnitine palmitoyltransferase 1A, 2: *CPT1A* and *CPT2*, solute carrier family 25 member 20: *SLC25A20*. (3) the peroxidation of excessive HFFA accelerated the progression of hepatic steatosis; Superoxide Dismutase 1, 2: SOD1 and SOD2, glutathione peroxidase 3: *GPX3*, catalase: *CAT*. (**B**) HFFAs increased significantly with the severity of hepatic steatosis (Padj < 0.05, post-hoc Wilcoxon rank-sum test). The opposite change pattern applied to PC and the PC/phosphatidyl-ethnolamine (PE) ratio ($P_{adj} < 0.05$, post-hoc Wilcoxon rank-sum test). (**C**) Expression of apolipoprotein B (*APOB*), *CHKA*, *PCYT1A* and *CHPT1* in the liver shared the same change pattern, decreasing significantly with hepatic steatosis in the eHSC group ($P_{adj} < 0.05$, post-hoc Wilcoxon rank-sum test). (**D**) The most severe hepatic steatosis group possessed the highest long-chain acylcarnitines (LCACs) but the least short-chain acylcarnitines (SCACs) ($P_{adj} < 0.05$, post-hoc Wilcoxon rank-sum test). (**E**) Expression of *CPT1A*, *SLC25A20*, and *CPT2* in the liver were significantly negatively correlated to hepatic steatosis in the eHSC group ($P_{adj} < 0.05$, post-hoc Wilcoxon rank-sum test). (**F**) Superoxide dismutase 1/2 (SOD1/2)

were significantly up-regulated in steatosis groups ($P_{adj} < 0.05$, post-hoc Wilcoxon rank-sum test), n = 4; while GPX3 was more expressed in steatosis groups ($P_{adj} < 0.05$, post-hoc Wilcoxon rank-sum test). (**G**) Oxidized and reduced glutathione (GSH-O and GSH-R, respectively), were also extremely low but 4-hydroxynonenal (HNE) was highly presented in steatotic livers ($P_{adj} < 0.05$, post-hoc Wilcoxon rank-sum test). (**H**) 9- and 13-hydroxy-octadecadienoic acid (9- and 13-HODE) and 9- and 13-oxo-octadecadienoic acid (9- and 13-oxo ODE) were higher in the steatosis group than that in the control group ($P_{adj} < 0.05$, post-hoc Wilcoxon rank-sum test). For Figure B-H, 0 refers to the eCtrl group, 1 for eHS-I, and 2 for eHS-II. Data of genes, metabolites and proteins are from n = 30, 6 and 4 biological replicates, respectively. *p < 0.05, **p < 0.01.

Fig. 4. Contribution of the gut microbial community to fat deposition related traits and their correlation with duodenal microbiota.

(**A**) Microbiability of duodenal, jejunal, ileal, cecal and fecal microbiota for hepatic steatosis classification (HSC), hepatic triglyceride (HTG), hepatic crude fat (HCF), hepatic free fatty acids (HFFA) and hepatic total bile acids (HTBA). (**B**) Four taxa chains from the phylum to genus level were generated on the basis of the taxa associated significantly with HSC by Spearman's rank-based correlation (SRC) analysis. n = 673. (**C**) Linear discriminant analysis Effect Size (LEfSe) analysis identified differential taxa on the basis of their significantly differential presence among HSC groups (LDA > 2). n = 673. (**D**) Heatmap of the association of genus abundance with all recorded phenotypes (SRC analysis, + indicates statistical

significance P_{adj} < 0.05, FDR correction); STG: serum triglyceride, SHDL: serum high-density lipoprotein, SLDL: low-density lipoprotein, SVLDL: very low-density lipoprotein, STBA: serum total bile acids, HTC: hepatic total cholesterol, STC: serum total cholesterol. n ≥ 673. (**E**) The amount and cumulative relative abundance of duodenal taxa with different detection rates from phylum to species. n = 686.

Fig. 5. Chicken hepatic methionine cycle, folate cycle, and associated genes and metabolites. To better demonstrate the results, not all enzymes and metabolites in the pathway were shown.

(**A**) Methionine cycle coupled with folate cycle. The scheme shows the main reactions and correlated genes involved in the methionine and folate cycle. Two main methionine adenosyltransferase isoforms (*MAT1A* and *MAT2B*), were respectively 2.2 and 2.6 times higher in the eCtrl group. Phosphatidylethanolamine N-methyltransferase (*PEMT*), S-adenosylhomocysteine hydrolase (*AHCY*) and methionine synthase (*MTR*) were significantly upregulated in the eCtrl group with 2.1, 2.6 and 22.5 times higher expression, respectively. (**B**) Comparison of the folic acid quantity in the liver (LFLA) and duodenal chyme (DFLA). 0 refers to the eCtrl group, and 1 for eHS-I, 2 for eHS-II. (**C**) Contents of methionine, S-adenosylmethionine (SAMe), S-adenosylhomocysteine (SAH) and homocysteine (Hcy) in the liver determined by ELISAs assay. 0 refers to the eCtrl group, and 1 for eHS-I, 2 for eHS-II. Data are from n = 30 (A) and 10 (B and C) biological replicates, respectively. *p < 0.05, **p < 0.01.

Fig. 6. Principles of eQTL mapping and SMR analysis.

(**A**) Differential RNA transcript abundance could potentially act as the causal link between a specific eQTL SNP and the phenotype connected to that SNP. Yet, the identification of a comparable correlation might also be attributed to two alternative scenarios: a reactive connection between RNA transcript abundance and the phenotype, or an independence relationship involving the eQTL, RNA transcript abundance, and the phenotype. (**B**) SMR was employed to scrutinize the potential pleiotropic association between the expression level of a gene and a complex trait of interest, utilizing summary statistics from GWAS and results from eQTL mapping.

Supplementary Fig. S1. Correlations among all recorded phenotypes, including the hepatic steatosis classification (HSC), hepatic and serum triglyceride (HTG and STG, respectively), hepatic crude fat (HCF) and free fatty acids (HFFAs), serum high-density lipoprotein (SHDL), low-density lipoprotein (SLDL) and very low-density lipoprotein (SVLDL), hepatic and serum total bile acids (HTBAs and STBAs, respectively), hepatic and serum total cholesterol (HTC and STC, respectively) and abdominal fat weight (AFW). The lower panel shows scatterplots for each pair of observations. Each point represents an individual. $n = 673$. *$p < 0.05$, **$p < 0.01$, ***$p < 0.001$.

Supplementary Fig. S2. Violin plots of the change patterns of hepatic triglyceride (HTG),

hepatic and serum total bile acids (HTBAs and STBAs, respectively) and abdominal fat weight (AFW) changed with hepatic steatosis in eHSC groups ($P_{adj} < 0.05$, post-hoc Wilcoxon rank-sum test). Data are from n = 30 biological replicates. *p < 0.05, **p < 0.01, ***p < 0.001.

Supplementary Fig. S3. The amount and distribution of cis- and trans-regulated genes in liver.

Supplementary Fig. S4. GO and pathway enrichment analysis of 227 candidate genes identified many biological processes of lipid localization and biosynthetic processes.

Supplementary Fig. S5. (**A**) Permutation test of duodenal microbiability of hepatic steatosis classification (HSC), hepatic free fatty acids (HFFAs) and hepatic total bile acids (HTBAs), and cecal microbiability of hepatic crude fat (HCF) and hepatic triglyceride (HTG). The results indicated that the estimated m^2 (0.15-0.27, blue lines) were significantly higher than the simulated ones (average m2 ranged from 0.02-0.04, gray lines). n ≥ 673. (**B**) The amount of taxa classified from phylum to species with high quality ASVs. n = 705. (**C**) Alpha diversities, including Shannon and Simpson Indices, both decreased with hepatic steatosis development ($P_{adj} < 0.05$, post-hoc Wilcoxon rank-sum test). n = 673. *p < 0.05, **p < 0.01.

Supplementary Fig. S6. Heatmaps of the association of the relative abundance of phyla, classes, orders, families and species with all recorded phenotypes (Spearman's rank-based correlation analysis, + indicates statistical significance $P_{adj} < 0.05$, FDR correction); HSC:

hepatic steatosis classification, HTG: hepatic triglyceride, STG: serum triglyceride, HCF: hepatic crude fat, HFFA: hepatic free fatty acid, SHDL: serum high-density lipoprotein, SLDL: serum low-density lipoprotein, SVLDL: serum very low-density lipoprotein, HTBAs: hepatic total bile acids, STBAs: serum total bile acids, HTC: hepatic total cholesterol, STC: serum total cholesterol, AFW: abdominal fat weight. $n \geq 673$.

Tables

Table 1 Description of 18 trans-eGenes related to hepatic steatosis

Gene symbol	High presented in	Gene location	eVariants region	No. of significant SNPs	Top SNP (Location)	Demonstration of the gene function in the references
NAPEPLD	Con	GGA1 13,065,354-13,085,319 bp	GGA10 10.2-17.9Mb	19044	rs313818251 (10:14,621,940)	Loss of *NAPEPLD* result in fat mass gain and hepatic steatosis in mouse [38]
WNT5A	HS	GGA12 8,303,804-8,315,223 bp	GGA5 30.1-36.4Mb	8259	- (5:34,210,278)	*WNT5A* may promote liver damage in human [82]
IL15	HS	GGA4 29,989,429-30,022,148 bp	GGA2 89.3-100.9Mp	8244	- (2:99,506,822)	Absence of *IL-15* or *IL-15Rα* protects from NAFL in mouse [83]
ACOT13	HS	GGA2 90,236,164-90,240,896 bp	GGA1 145.1-150.8Mp	6020	rs316486650 (1:148,449,556)	Regulate hepatic lipid and glucose metabolism in mouse [84]
JAG1	Con	GGA3 13,599,642-13,633,726 bp	GGA21 5.04-6.03Mp	4428	rs313406743 (21:5,121,276)	Hepatocyte-specific *JAG1* knockout mice were protected from NASH-induced liver fibrosis [85]
ISM1	Con	GGA3 12,825,298-12,864,186 bp	GGA21 4.07-4.65 Mb	700	rs315660086 (21:4,319,591)	*ISM1* suppresses hepatocyte lipid synthesis in mouse [86]
ECHDC1	HS	GGA3 59,266,598-59,303,456 bp	GGA5 17.8-18.0 Mb	11	rs316727537 (5:17,943,618)	Involved in the occurrence and development of NAFLD by regulating hepatic lipid metabolism in human [87]
ETV5	Con	GGA9 5,289,877-5,299,258 bp	GGA4 61.15-61.17 Mb	4	rs315563554 (4:61,152,625)	Regulate hepatic fatty acid metabolism in mouse [88]

GSTA3	Con	GGA3<br>88,388,999-88,395,707 bp	GGA2<br>34.0-34.5 Mb	4	rs314345391<br>(2:34,475,071)	Paly vital roles in hepatic iron metabolism, and may associated with NALFD in mouse [89]
IFRD1	Con	GGA1<br>27,057,994-27,068,635 bp	GGA11<br>11.0-11.7 Mb	13	-<br>(11:11,142,255)	A regulator of lipid absorption and metabolism in mouse [90]
SLITRK3	Con	GGA9<br>21,273,430-21,276,090 bp	GGA4<br>79.7-80.0 Mb	38	-<br>(4:79,863,187)	*SLITRK3* was down-regulated in steatosis and NASH patients in human [91]
PTPN2	Con	GGA2<br>97,013,090-97,047,494 bp	GGA17<br>3.47-4.08 Mb	16	-<br>(17:3,671,818)	Liver-specific *PTPN2* deficiency promotes hepatic steatosis, obesity and insulin resistance in human and mouse [4, 92]
UCHL1	Con	GGA4<br>68,642,734-68,647,699 bp	GGA2<br>139.8-140.2 MB	15	rs312268369<br>(2:140,103,337)	*UCHL1* appears to be a functional tumor suppressor involved in the tumorigenesis of hepatocellular carcinoma in human [93]
SLC16A10	Con	GGA3<br>66,217,809-66,280,609 bp	GGA3<br>10876753-<br>10876785 bp	3	rs739986067<br>(3:10,876,769)	*SLC16A10* is a transport carrier of aromatic amino acids, it up-regulated in NAFLD group in Rats [94]
DIO2	Con	GGA5<br>40,752,235-40,769,122 bp	GGA3<br>8.69-8.86 Mb	2	rs315951384<br>(3:8,693,350)	Loss of the *DIO2* gene results in increased fat storage in adipose tissue and hepatic steatosis in mouse [95]
MT3	Con	GGA11<br>2,122,187-2,123,328 bp	GGA8<br>15963283 bp	1	rs314635449<br>(8:15963283)	*MT3* may be a potential intervention for hepatic steatosis by inhibit the generation of ROS in human [96]
RBP	Con	GGA8<br>21,595,383-21,606,407 bp	GGA1<br>55817413 bp	1	-<br>(3:55817413)	Lower expression of *RBP* may improve hepatic steatosis in mouse [97, 98]

NAPEPLD: N-acyl phosphatidylethanolamine phospholipase D, *WNT5A*: Wnt Family Member 5A, *IL15*: Interleukin 15, *ACOT13*: Acyl-CoA thioesterase 13, *JAG1*: Jagged canonical Notch ligand 1, *ISM1*: Isthmin 1, *ECHDC1*: Ethylmalonyl-CoA decarboxylase 1, *ETV5*: ETS variant transcription factor 7, *GSTA3*: Glutathione S-transferase alpha 3, *IFRD1*: Interferon related developmental regulator 1, *SLITRK3*: SLIT and NTRK like family member 3, *PTPN2*: Protein tyrosine phosphatase non-receptor type 2, *UCHL1*: Ubiquitin C-terminal hydrolase L1, *SLC16A10*: Solute carrier family 16 member 10, *DIO2*: Deiodinase 2, *MT3*: Metallothionein 3, *RBP*: Retinol binding protein 1

 Click here to access/download;Table;Supplementary Tables.xlsx

Table S1. Summary statistics of whole-genome resequencing

Chromosome	Length	Variants	Variants rate	SNP density
1	197,608,386	1,253,547	157	6.369
2	149,682,049	891,415	167	5.988
3	110,838,418	684,557	161	6.211
4	91,315,245	616,721	148	6.757
5	59,809,098	391,270	152	6.579
6	36,374,701	231,498	157	6.369
7	36,742,308	238,840	153	6.536
8	30,219,446	162,237	186	5.376
9	24,153,086	177,911	135	7.407
10	21,119,840	116,804	180	5.556
11	20,200,042	111,727	180	5.556
12	20,387,278	114,119	178	5.618
13	19,166,714	115,089	166	6.024
14	16,219,308	111,232	145	6.897
15	13,062,184	80,399	162	6.173
16	2,844,601	5,667	501	1.996
17	10,762,512	61,843	174	5.747
18	11,373,140	79,457	143	6.993
19	10,323,212	67,391	153	6.536
20	13,897,287	79,011	175	5.714
21	6,844,979	43,677	156	6.41
22	5,459,462	17,294	315	3.175
23	6,149,580	39,737	154	6.494
24	6,491,222	47,846	135	7.407
25	3,980,610	18,184	218	4.587
26	6,055,710	39,936	151	6.623
27	8,080,432	35,492	227	4.405
28	5,116,882	29,276	174	5.747
30	1,818,525	3,719	488	2.049
31	6,153,034	9,915	620	1.613
32	725,831	1,732	419	2.387
33	7,821,666	27,277	286	3.497
Total	960,796,788	5,904,820	162	6.173

Table S2. significant SNPs of GWAS on

chromosome	position	n_miss	allele1	allele0	af	beta
1	83516996	0	G	T	0.023	-7.16E-01
1	83792529	0	G	A	0.027	-5.33E-01
2	129845750	0	A	G	0.236	-2.38E-01
2	129897204	0	T	C	0.245	-2.18E-01
2	129930691	0	G	A	0.237	-2.15E-01
2	129965320	0	G	C	0.22	-2.11E-01
4	34553496	0	T	C	0.391	-2.11E-01
4	34554151	0	C	A	0.391	-2.11E-01
4	75567863	0	A	G	0.378	-2.08E-01
4	75581602	0	G	A	0.377	-1.99E-01
4	75627113	0	T	C	0.38	-2.02E-01
4	75632945	0	C	A	0.38	-2.03E-01
4	75656076	0	C	A	0.387	-2.07E-01
4	75662049	0	A	G	0.378	-1.95E-01
4	75684578	0	A	G	0.443	-1.74E-01
4	75685964	0	C	A	0.411	-2.06E-01
4	75686442	0	C	T	0.405	-1.92E-01
4	75687831	0	C	G	0.399	-1.96E-01
4	75687838	0	T	C	0.375	-1.94E-01
4	75696705	0	C	T	0.373	-1.95E-01
4	75696745	0	G	A	0.41	-1.94E-01
4	75697628	0	T	A	0.377	-2.05E-01
4	75711936	0	T	C	0.416	-1.93E-01
4	75720493	0	T	C	0.378	-2.10E-01
4	75758710	0	T	C	0.356	-2.27E-01
4	75765168	0	C	A	0.356	-2.14E-01
4	75784652	0	T	G	0.348	-2.02E-01
4	75812652	0	T	C	0.41	-2.03E-01
4	75846402	0	A	G	0.434	-1.97E-01
4	75849038	0	C	T	0.436	-1.92E-01
4	75849291	0	A	G	0.438	-2.08E-01
4	75849576	0	A	G	0.439	-1.91E-01
4	75918373	0	G	A	0.406	-1.89E-01

4	75920070	0	T	C	0.403	-1.93E-01
4	75924204	0	A	G	0.395	-2.04E-01
4	75930994	0	C	G	0.386	-2.14E-01
4	75931184	0	T	G	0.384	-2.08E-01
4	75941787	0	G	A	0.361	-2.11E-01
4	75942314	0	T	C	0.389	-2.20E-01
4	75949490	0	A	G	0.391	-2.12E-01
4	75952489	0	T	C	0.377	-2.12E-01
4	75956383	0	G	A	0.382	-2.02E-01
4	75972585	0	A	T	0.391	-2.06E-01
4	75973636	0	G	A	0.373	-2.20E-01
4	75974299	0	G	A	0.391	-1.99E-01
4	75977013	0	G	A	0.388	-2.09E-01
4	75977098	0	T	C	0.375	-2.02E-01
4	75981329	0	C	T	0.359	-2.07E-01
4	75984316	0	T	C	0.389	-2.05E-01
4	75993261	0	A	G	0.384	-2.07E-01
4	75993304	0	A	G	0.384	-2.09E-01
4	75993479	0	A	G	0.404	-2.10E-01
4	76011918	0	C	A	0.396	-2.11E-01
4	76011920	0	C	T	0.402	-2.04E-01
4	76012006	0	T	C	0.398	-2.13E-01
4	76012033	0	T	C	0.398	-2.16E-01
4	76014590	0	C	T	0.402	-2.09E-01
4	76014793	0	C	T	0.402	-2.05E-01
4	76015796	0	G	A	0.387	-2.06E-01
4	76017707	0	G	C	0.397	-2.14E-01
4	76029335	0	T	C	0.373	-2.20E-01
4	76036639	0	G	A	0.431	-1.79E-01
4	76046632	0	T	C	0.365	-1.86E-01
4	76047356	0	T	G	0.349	-1.97E-01
4	76052698	0	C	T	0.348	-1.78E-01
4	76052813	0	T	C	0.356	-1.82E-01
4	76064052	0	G	A	0.4	-1.95E-01
4	76064202	0	A	T	0.403	-1.98E-01
4	76075176	0	G	T	0.398	-2.03E-01
4	76077316	0	A	C	0.401	-1.97E-01

4	76078049	0	G	T	0.393	-1.98E-01
4	76089787	0	C	G	0.409	-1.92E-01
4	76097440	0	A	G	0.358	-2.16E-01
4	76099099	0	T	C	0.348	-1.92E-01
4	76102412	0	G	A	0.378	-2.11E-01
4	76108074	0	T	C	0.384	-2.04E-01
4	76108411	0	C	T	0.348	-2.20E-01
4	76110602	0	A	G	0.35	-2.17E-01
4	76110884	0	T	C	0.353	-2.04E-01
4	76114368	0	G	A	0.394	-2.05E-01
4	76127442	0	G	A	0.399	-2.02E-01
4	76130502	0	C	T	0.392	-1.98E-01
4	76133653	0	G	A	0.405	-2.13E-01
4	76139794	0	G	A	0.382	-1.92E-01
4	76141988	0	C	T	0.355	-2.04E-01
4	76164811	0	A	C	0.382	-2.12E-01
4	76183477	0	T	C	0.388	-2.11E-01
4	76184054	0	A	G	0.389	-1.94E-01
4	76193178	0	C	G	0.391	-1.98E-01
4	76208160	0	C	T	0.391	-2.02E-01
4	76209175	0	A	G	0.363	-2.03E-01
4	76232936	0	C	T	0.362	-2.07E-01
4	76263548	0	C	T	0.398	-1.99E-01
4	76265313	0	T	C	0.36	-2.05E-01
4	76266812	0	T	C	0.382	-2.15E-01
4	76268021	0	T	C	0.394	-2.06E-01
4	76316140	0	A	G	0.402	-2.03E-01
4	76342528	0	C	T	0.393	-2.02E-01
4	76348471	0	A	G	0.359	-2.10E-01
4	76348821	0	C	T	0.362	-2.33E-01
4	76373195	0	A	G	0.387	-1.99E-01
4	76373450	0	T	A	0.389	-2.07E-01
4	76373977	0	C	A	0.39	-2.06E-01
4	76377549	0	A	G	0.377	-1.95E-01
4	76384869	0	C	T	0.388	-2.08E-01
4	76385276	0	A	G	0.359	-2.18E-01
4	76416923	0	T	C	0.396	-2.04E-01

4	76438296	0	A	T	0.389	-2.02E-01
4	76440762	0	A	G	0.397	-2.02E-01
4	76466486	0	T	A	0.355	-1.85E-01
4	76466670	0	A	G	0.379	-2.14E-01
4	76478523	0	A	C	0.387	-2.18E-01
4	76480932	0	A	G	0.347	-1.96E-01
4	76486416	0	A	G	0.362	-2.07E-01
4	76488920	0	C	T	0.362	-2.13E-01
4	76489652	0	C	T	0.359	-2.15E-01
4	76492306	0	G	A	0.351	-1.99E-01
4	76493573	0	G	A	0.359	-2.06E-01
4	84046002	0	A	C	0.013	8.14E-01
5	24251729	0	A	G	0.347	2.00E-01
6	5593161	0	T	C	0.252	2.41E-01
6	5593198	0	C	T	0.27	2.13E-01
6	5593674	0	C	T	0.251	2.34E-01
6	5593686	0	A	G	0.251	2.34E-01
6	5593894	0	T	C	0.273	2.11E-01
6	5594168	0	C	T	0.257	2.23E-01
6	5594388	0	T	C	0.271	2.34E-01
6	5594472	0	T	C	0.255	2.29E-01
6	5594550	0	A	G	0.252	2.55E-01
6	5595239	0	T	C	0.265	2.34E-01
6	5595589	0	C	T	0.277	2.11E-01
6	5595659	0	G	A	0.255	2.35E-01
6	5595978	0	C	G	0.255	2.31E-01
6	5596063	0	T	C	0.271	2.25E-01
6	5596289	0	A	G	0.255	2.19E-01
6	5597325	0	G	A	0.281	2.03E-01
6	5597813	0	A	T	0.261	2.06E-01
6	5597980	0	T	C	0.26	2.18E-01
6	5599033	0	C	T	0.259	2.25E-01
6	5599041	0	G	C	0.275	2.35E-01
6	5599107	0	A	G	0.277	2.31E-01
6	5599558	0	A	G	0.274	2.11E-01
6	5599772	0	T	A	0.273	2.27E-01
6	5600060	0	C	G	0.274	2.25E-01

6	5600131	0	C	A	0.274	2.25E-01
6	5600264	0	C	T	0.276	2.23E-01
6	5600306	0	G	A	0.275	2.31E-01
6	5600616	0	C	T	0.277	2.28E-01
6	5600837	0	C	T	0.258	2.18E-01
6	5601318	0	A	G	0.259	2.31E-01
6	5601454	0	T	C	0.275	2.26E-01
6	5601576	0	A	T	0.272	2.19E-01
6	5601618	0	G	C	0.269	2.32E-01
6	5601666	0	C	G	0.269	2.13E-01
6	5601735	0	A	G	0.274	2.19E-01
6	5602501	0	T	C	0.251	2.26E-01
6	5605128	0	C	T	0.269	2.18E-01
6	5621705	0	C	T	0.244	2.27E-01
6	5621741	0	T	C	0.255	2.29E-01
6	5633877	0	T	C	0.233	2.26E-01
6	5640110	0	A	G	0.236	2.27E-01
6	5641799	0	C	A	0.236	2.25E-01
6	5643678	0	C	T	0.244	2.02E-01
6	5644246	0	G	A	0.241	2.17E-01
6	5644302	0	A	G	0.247	2.26E-01
6	5644345	0	C	T	0.241	2.28E-01
6	5653114	0	G	T	0.268	2.21E-01
6	5654455	0	T	C	0.281	2.05E-01
6	5660719	0	A	T	0.247	2.33E-01
6	5666247	0	A	C	0.238	2.22E-01
6	5666425	0	G	A	0.24	2.25E-01
6	5668356	0	A	T	0.241	2.20E-01
6	5669244	0	C	T	0.248	2.21E-01
6	5673713	0	A	G	0.251	2.33E-01
6	5684495	0	A	C	0.241	2.45E-01
6	5691439	0	G	A	0.246	2.28E-01
6	5693788	0	A	G	0.242	2.28E-01
6	5693831	0	G	A	0.243	2.28E-01
6	5693905	0	C	G	0.244	2.30E-01
6	5694953	0	T	C	0.235	2.30E-01
6	5704817	0	A	G	0.216	2.36E-01

HSC with AFP as an additional covariates

se	logl_H1	l_remle	l_mle	p_wald	p_lrt
1.51E-01	-6.92E+02	4.31E-01	3.14E-01	2.44E-06	1.79E-06
1.15E-01	-6.92E+02	4.37E-01	3.22E-01	4.01E-06	3.00E-06
5.27E-02	-6.93E+02	3.64E-01	2.47E-01	7.75E-06	5.39E-06
4.79E-02	-6.92E+02	3.35E-01	2.24E-01	6.37E-06	4.49E-06
4.77E-02	-6.93E+02	3.50E-01	2.33E-01	7.48E-06	5.21E-06
4.55E-02	-6.92E+02	3.01E-01	1.95E-01	4.35E-06	3.21E-06
4.60E-02	-6.93E+02	6.16E-01	4.87E-01	5.18E-06	6.34E-06
4.60E-02	-6.93E+02	6.16E-01	4.87E-01	5.18E-06	6.34E-06
4.42E-02	-6.92E+02	4.27E-01	3.09E-01	3.10E-06	2.27E-06
4.40E-02	-6.93E+02	4.22E-01	3.06E-01	7.51E-06	5.53E-06
4.44E-02	-6.93E+02	4.22E-01	3.09E-01	6.63E-06	4.90E-06
4.40E-02	-6.92E+02	4.09E-01	2.96E-01	5.00E-06	3.61E-06
4.38E-02	-6.92E+02	4.15E-01	3.02E-01	2.70E-06	1.95E-06
4.35E-02	-6.93E+02	4.30E-01	3.15E-01	8.45E-06	6.32E-06
3.76E-02	-6.92E+02	4.17E-01	3.03E-01	4.41E-06	3.21E-06
4.23E-02	-6.91E+02	4.11E-01	2.98E-01	1.43E-06	1.02E-06
4.27E-02	-6.93E+02	4.23E-01	3.07E-01	8.09E-06	5.97E-06
4.26E-02	-6.92E+02	4.18E-01	3.02E-01	5.09E-06	3.71E-06
4.31E-02	-6.93E+02	4.15E-01	3.03E-01	7.88E-06	5.79E-06
4.29E-02	-6.93E+02	4.34E-01	3.18E-01	6.69E-06	5.02E-06
4.24E-02	-6.92E+02	4.20E-01	3.04E-01	5.85E-06	4.28E-06
4.39E-02	-6.92E+02	4.19E-01	3.03E-01	3.83E-06	2.78E-06
4.27E-02	-6.93E+02	4.13E-01	3.01E-01	7.11E-06	5.20E-06
4.44E-02	-6.92E+02	4.31E-01	3.17E-01	2.93E-06	2.16E-06
4.37E-02	-6.89E+02	4.52E-01	3.35E-01	2.66E-07	1.97E-07
4.44E-02	-6.91E+02	4.72E-01	3.54E-01	1.80E-06	1.42E-06
4.41E-02	-6.92E+02	4.85E-01	3.65E-01	5.38E-06	4.43E-06
4.28E-02	-6.92E+02	4.79E-01	3.60E-01	2.64E-06	2.13E-06
4.28E-02	-6.92E+02	4.40E-01	3.24E-01	5.36E-06	4.04E-06
4.25E-02	-6.93E+02	4.51E-01	3.37E-01	7.68E-06	5.97E-06
4.25E-02	-6.91E+02	4.69E-01	3.50E-01	1.20E-06	9.36E-07
4.24E-02	-6.93E+02	4.50E-01	3.33E-01	7.73E-06	5.96E-06
4.21E-02	-6.93E+02	4.31E-01	3.17E-01	8.65E-06	6.48E-06

4.25E-02	-6.93E+02	4.67E-01	3.54E-01	6.96E-06	5.58E-06
4.41E-02	-6.92E+02	4.60E-01	3.46E-01	4.50E-06	3.53E-06
4.60E-02	-6.92E+02	4.64E-01	3.49E-01	4.10E-06	3.23E-06
4.54E-02	-6.92E+02	4.59E-01	3.43E-01	5.69E-06	4.45E-06
4.59E-02	-6.92E+02	4.76E-01	3.61E-01	5.18E-06	4.20E-06
4.44E-02	-6.91E+02	4.88E-01	3.71E-01	8.72E-07	7.08E-07
4.59E-02	-6.92E+02	4.78E-01	3.62E-01	4.59E-06	3.73E-06
4.55E-02	-6.92E+02	4.75E-01	3.59E-01	3.84E-06	3.10E-06
4.47E-02	-6.93E+02	4.26E-01	3.13E-01	7.65E-06	5.69E-06
4.54E-02	-6.93E+02	4.78E-01	3.61E-01	6.72E-06	5.49E-06
4.31E-02	-6.90E+02	4.47E-01	3.32E-01	4.58E-07	3.39E-07
4.41E-02	-6.93E+02	4.87E-01	3.70E-01	7.56E-06	6.33E-06
4.47E-02	-6.92E+02	4.57E-01	3.40E-01	3.54E-06	2.73E-06
4.43E-02	-6.92E+02	4.50E-01	3.34E-01	6.04E-06	4.65E-06
4.46E-02	-6.92E+02	4.59E-01	3.48E-01	4.45E-06	3.49E-06
4.46E-02	-6.92E+02	4.68E-01	3.53E-01	5.27E-06	4.20E-06
4.54E-02	-6.93E+02	4.70E-01	3.53E-01	6.17E-06	4.94E-06
4.50E-02	-6.92E+02	4.83E-01	3.66E-01	4.41E-06	3.62E-06
4.34E-02	-6.91E+02	4.59E-01	3.40E-01	1.67E-06	1.28E-06
4.45E-02	-6.92E+02	4.75E-01	3.59E-01	2.74E-06	2.20E-06
4.42E-02	-6.92E+02	4.58E-01	3.44E-01	4.78E-06	3.73E-06
4.44E-02	-6.91E+02	4.55E-01	3.42E-01	1.97E-06	1.51E-06
4.44E-02	-6.91E+02	4.57E-01	3.44E-01	1.47E-06	1.13E-06
4.30E-02	-6.91E+02	4.84E-01	3.68E-01	1.46E-06	1.19E-06
4.29E-02	-6.92E+02	4.69E-01	3.55E-01	2.14E-06	1.69E-06
4.58E-02	-6.93E+02	4.75E-01	3.59E-01	7.73E-06	6.30E-06
4.42E-02	-6.91E+02	4.73E-01	3.57E-01	1.71E-06	1.36E-06
4.60E-02	-6.91E+02	4.61E-01	3.48E-01	2.14E-06	1.66E-06
3.86E-02	-6.92E+02	4.85E-01	3.67E-01	4.45E-06	3.67E-06
4.13E-02	-6.93E+02	4.52E-01	3.41E-01	7.98E-06	6.24E-06
4.39E-02	-6.93E+02	4.08E-01	3.01E-01	8.86E-06	6.51E-06
3.86E-02	-6.92E+02	4.34E-01	3.20E-01	5.22E-06	3.91E-06
3.85E-02	-6.92E+02	4.14E-01	3.04E-01	2.68E-06	1.95E-06
4.31E-02	-6.93E+02	4.42E-01	3.28E-01	7.57E-06	5.77E-06
4.33E-02	-6.92E+02	4.50E-01	3.36E-01	5.81E-06	4.48E-06
4.43E-02	-6.92E+02	4.46E-01	3.34E-01	5.75E-06	4.41E-06
4.28E-02	-6.92E+02	4.46E-01	3.33E-01	5.08E-06	3.89E-06

4.34E-02	-6.93E+02	4.80E-01	3.68E-01	6.16E-06	5.08E-06
4.19E-02	-6.92E+02	4.27E-01	3.18E-01	5.73E-06	4.28E-06
4.53E-02	-6.92E+02	4.41E-01	3.29E-01	2.29E-06	1.72E-06
4.25E-02	-6.93E+02	4.10E-01	3.03E-01	7.56E-06	5.56E-06
4.36E-02	-6.91E+02	4.70E-01	3.52E-01	1.59E-06	1.25E-06
4.53E-02	-6.93E+02	4.87E-01	3.68E-01	7.69E-06	6.41E-06
4.41E-02	-6.91E+02	4.45E-01	3.32E-01	8.11E-07	6.04E-07
4.38E-02	-6.91E+02	4.61E-01	3.48E-01	8.98E-07	6.91E-07
4.51E-02	-6.93E+02	4.67E-01	3.53E-01	7.40E-06	5.94E-06
4.41E-02	-6.92E+02	4.62E-01	3.49E-01	4.23E-06	3.33E-06
4.28E-02	-6.92E+02	4.43E-01	3.34E-01	2.86E-06	2.17E-06
4.38E-02	-6.93E+02	4.55E-01	3.42E-01	7.19E-06	5.63E-06
4.25E-02	-6.90E+02	4.75E-01	3.60E-01	7.13E-07	5.61E-07
4.23E-02	-6.93E+02	4.71E-01	3.56E-01	6.94E-06	5.61E-06
4.39E-02	-6.92E+02	4.54E-01	3.44E-01	3.95E-06	3.07E-06
4.65E-02	-6.92E+02	4.72E-01	3.56E-01	5.85E-06	4.71E-06
4.43E-02	-6.92E+02	4.60E-01	3.45E-01	2.33E-06	1.80E-06
4.17E-02	-6.92E+02	4.72E-01	3.56E-01	3.95E-06	3.17E-06
4.38E-02	-6.93E+02	4.22E-01	3.10E-01	7.19E-06	5.32E-06
4.15E-02	-6.91E+02	4.81E-01	3.65E-01	1.36E-06	1.10E-06
4.46E-02	-6.93E+02	4.73E-01	3.59E-01	6.49E-06	5.26E-06
4.58E-02	-6.93E+02	4.46E-01	3.33E-01	7.47E-06	5.74E-06
4.29E-02	-6.92E+02	4.59E-01	3.46E-01	4.21E-06	3.29E-06
4.42E-02	-6.92E+02	4.74E-01	3.62E-01	4.42E-06	3.58E-06
4.39E-02	-6.91E+02	4.56E-01	3.45E-01	1.22E-06	9.37E-07
4.46E-02	-6.92E+02	4.61E-01	3.47E-01	4.70E-06	3.70E-06
4.32E-02	-6.92E+02	4.95E-01	3.75E-01	3.24E-06	2.72E-06
4.45E-02	-6.93E+02	4.54E-01	3.40E-01	6.62E-06	5.15E-06
4.57E-02	-6.92E+02	4.64E-01	3.52E-01	5.07E-06	4.03E-06
4.55E-02	-6.90E+02	4.56E-01	3.44E-01	4.18E-07	3.15E-07
4.42E-02	-6.93E+02	4.67E-01	3.50E-01	7.74E-06	6.18E-06
4.52E-02	-6.92E+02	4.60E-01	3.45E-01	5.44E-06	4.26E-06
4.50E-02	-6.92E+02	4.63E-01	3.47E-01	5.66E-06	4.46E-06
4.30E-02	-6.93E+02	4.59E-01	3.44E-01	6.94E-06	5.45E-06
4.49E-02	-6.92E+02	4.76E-01	3.60E-01	4.35E-06	3.51E-06
4.64E-02	-6.92E+02	4.52E-01	3.39E-01	3.20E-06	2.45E-06
4.27E-02	-6.92E+02	4.50E-01	3.38E-01	2.25E-06	1.71E-06

4.48E-02	-6.93E+02	4.51E-01	3.39E-01	7.67E-06	5.97E-06
4.34E-02	-6.92E+02	4.55E-01	3.42E-01	4.06E-06	3.15E-06
4.05E-02	-6.93E+02	4.89E-01	3.73E-01	6.31E-06	5.30E-06
4.55E-02	-6.92E+02	4.72E-01	3.58E-01	2.97E-06	2.37E-06
4.47E-02	-6.91E+02	4.59E-01	3.46E-01	1.34E-06	1.03E-06
4.37E-02	-6.93E+02	4.64E-01	3.50E-01	8.16E-06	6.51E-06
4.58E-02	-6.93E+02	4.52E-01	3.39E-01	7.31E-06	5.69E-06
4.58E-02	-6.92E+02	4.45E-01	3.31E-01	4.11E-06	3.13E-06
4.58E-02	-6.92E+02	4.81E-01	3.65E-01	3.47E-06	2.83E-06
4.45E-02	-6.93E+02	4.03E-01	2.95E-01	8.83E-06	6.43E-06
4.56E-02	-6.93E+02	4.74E-01	3.59E-01	7.35E-06	5.97E-06
1.81E-01	-6.93E+02	4.59E-01	3.50E-01	8.12E-06	6.50E-06
4.30E-02	-6.92E+02	3.95E-01	2.75E-01	3.97E-06	2.79E-06
4.90E-02	-6.91E+02	3.23E-01	2.13E-01	1.10E-06	7.71E-07
4.75E-02	-6.93E+02	3.43E-01	2.33E-01	8.99E-06	6.39E-06
4.79E-02	-6.91E+02	3.50E-01	2.41E-01	1.30E-06	9.03E-07
4.79E-02	-6.91E+02	3.50E-01	2.41E-01	1.30E-06	9.03E-07
4.62E-02	-6.92E+02	3.65E-01	2.52E-01	5.58E-06	3.91E-06
4.83E-02	-6.92E+02	3.29E-01	2.21E-01	4.93E-06	3.52E-06
4.68E-02	-6.90E+02	3.52E-01	2.40E-01	7.50E-07	5.13E-07
4.87E-02	-6.92E+02	3.43E-01	2.31E-01	3.02E-06	2.10E-06
4.85E-02	-6.89E+02	3.12E-01	2.02E-01	2.13E-07	1.48E-07
4.72E-02	-6.91E+02	3.29E-01	2.18E-01	8.84E-07	6.12E-07
4.50E-02	-6.92E+02	3.47E-01	2.35E-01	3.50E-06	2.44E-06
4.84E-02	-6.91E+02	3.48E-01	2.35E-01	1.63E-06	1.12E-06
4.82E-02	-6.91E+02	3.45E-01	2.32E-01	1.99E-06	1.38E-06
4.63E-02	-6.91E+02	3.21E-01	2.12E-01	1.58E-06	1.11E-06
4.85E-02	-6.93E+02	3.42E-01	2.31E-01	7.35E-06	5.21E-06
4.47E-02	-6.92E+02	3.40E-01	2.31E-01	6.71E-06	4.76E-06
4.61E-02	-6.93E+02	3.13E-01	2.07E-01	8.95E-06	6.58E-06
4.68E-02	-6.92E+02	3.53E-01	2.43E-01	4.09E-06	2.88E-06
4.83E-02	-6.92E+02	3.42E-01	2.31E-01	3.97E-06	2.79E-06
4.57E-02	-6.90E+02	3.49E-01	2.38E-01	3.37E-07	2.28E-07
4.57E-02	-6.90E+02	3.44E-01	2.33E-01	5.89E-07	4.03E-07
4.72E-02	-6.93E+02	3.57E-01	2.46E-01	8.88E-06	6.28E-06
4.71E-02	-6.91E+02	3.31E-01	2.21E-01	1.83E-06	1.28E-06
4.66E-02	-6.91E+02	3.30E-01	2.20E-01	1.72E-06	1.20E-06

4.66E-02	-6.91E+02	3.30E-01	2.21E-01	1.71E-06	1.20E-06
4.60E-02	-6.91E+02	3.18E-01	2.11E-01	1.60E-06	1.14E-06
4.65E-02	-6.91E+02	3.20E-01	2.13E-01	8.65E-07	6.08E-07
4.53E-02	-6.90E+02	3.29E-01	2.18E-01	6.27E-07	4.32E-07
4.83E-02	-6.93E+02	3.67E-01	2.53E-01	7.27E-06	5.11E-06
4.86E-02	-6.92E+02	3.36E-01	2.26E-01	2.50E-06	1.75E-06
4.64E-02	-6.91E+02	3.45E-01	2.32E-01	1.38E-06	9.54E-07
4.72E-02	-6.92E+02	3.61E-01	2.49E-01	4.14E-06	2.90E-06
4.75E-02	-6.91E+02	3.50E-01	2.37E-01	1.30E-06	8.95E-07
4.73E-02	-6.93E+02	3.61E-01	2.49E-01	8.21E-06	5.80E-06
4.63E-02	-6.92E+02	3.39E-01	2.30E-01	2.71E-06	1.91E-06
5.00E-02	-6.93E+02	3.30E-01	2.21E-01	7.59E-06	5.44E-06
4.68E-02	-6.92E+02	3.37E-01	2.26E-01	3.77E-06	2.65E-06
4.83E-02	-6.92E+02	4.00E-01	2.88E-01	3.35E-06	2.40E-06
4.79E-02	-6.91E+02	3.41E-01	2.32E-01	2.26E-06	1.58E-06
4.61E-02	-6.91E+02	3.28E-01	2.22E-01	1.18E-06	8.28E-07
4.83E-02	-6.92E+02	3.58E-01	2.47E-01	3.33E-06	2.33E-06
4.81E-02	-6.92E+02	3.50E-01	2.42E-01	3.58E-06	2.52E-06
4.45E-02	-6.93E+02	3.65E-01	2.53E-01	6.98E-06	4.94E-06
4.76E-02	-6.92E+02	3.62E-01	2.52E-01	5.95E-06	4.20E-06
4.80E-02	-6.92E+02	3.54E-01	2.45E-01	3.14E-06	2.21E-06
4.83E-02	-6.92E+02	3.61E-01	2.50E-01	2.86E-06	2.00E-06
4.73E-02	-6.92E+02	3.38E-01	2.29E-01	3.62E-06	2.56E-06
4.50E-02	-6.92E+02	3.44E-01	2.36E-01	6.43E-06	4.57E-06
4.94E-02	-6.92E+02	3.64E-01	2.55E-01	2.98E-06	2.09E-06
4.90E-02	-6.93E+02	3.66E-01	2.56E-01	7.21E-06	5.11E-06
5.03E-02	-6.93E+02	3.73E-01	2.64E-01	8.82E-06	6.30E-06
4.87E-02	-6.93E+02	3.82E-01	2.72E-01	7.34E-06	5.24E-06
4.90E-02	-6.93E+02	3.91E-01	2.80E-01	7.78E-06	5.59E-06
4.99E-02	-6.92E+02	3.87E-01	2.77E-01	3.76E-06	2.66E-06
4.93E-02	-6.90E+02	3.46E-01	2.39E-01	8.42E-07	5.83E-07
4.96E-02	-6.92E+02	3.95E-01	2.81E-01	5.24E-06	3.73E-06
5.03E-02	-6.93E+02	3.90E-01	2.77E-01	6.86E-06	4.90E-06
5.04E-02	-6.93E+02	3.88E-01	2.74E-01	7.09E-06	5.05E-06
5.02E-02	-6.92E+02	3.93E-01	2.78E-01	5.74E-06	4.08E-06
5.09E-02	-6.93E+02	4.17E-01	3.03E-01	7.68E-06	5.64E-06
4.98E-02	-6.92E+02	3.68E-01	2.61E-01	2.58E-06	1.82E-06

p_score
2.87E-06
4.59E-06
9.67E-06
9.13E-06
9.90E-06
7.93E-06
1.01E-05
1.01E-05
3.60E-06
8.36E-06
7.43E-06
5.74E-06
3.19E-06
9.33E-06
5.06E-06
1.75E-06
8.96E-06
5.79E-06
8.80E-06
7.47E-06
6.60E-06
4.41E-06
7.99E-06
3.41E-06
3.60E-07
2.21E-06
6.37E-06
3.21E-06
6.04E-06
8.58E-06
1.50E-06
8.61E-06
9.53E-06

7.95E-06
5.20E-06
4.78E-06
6.47E-06
6.07E-06
1.15E-06
5.42E-06
4.57E-06
8.49E-06
7.80E-06
5.96E-07
8.89E-06
4.12E-06
6.81E-06
5.15E-06
6.10E-06
7.09E-06
5.27E-06
2.02E-06
3.32E-06
5.50E-06
2.36E-06
1.79E-06
1.86E-06
2.60E-06
8.88E-06
2.12E-06
2.57E-06
5.34E-06
8.92E-06
9.90E-06
5.90E-06
3.17E-06
8.41E-06
6.56E-06
6.49E-06
5.77E-06

7.24E-06
6.45E-06
2.70E-06
8.50E-06
1.96E-06
9.00E-06
1.02E-06
1.14E-06
8.43E-06
4.92E-06
3.35E-06
8.08E-06
9.31E-07
7.98E-06
4.58E-06
6.77E-06
2.78E-06
4.67E-06
8.02E-06
1.73E-06
7.50E-06
8.32E-06
4.88E-06
5.22E-06
1.51E-06
5.43E-06
4.02E-06
7.46E-06
5.87E-06
5.51E-07
8.77E-06
6.22E-06
6.47E-06
7.83E-06
5.14E-06
3.73E-06
2.67E-06

8.57E-06
4.69E-06
7.53E-06
3.56E-06
1.65E-06
9.21E-06
8.19E-06
4.71E-06
4.19E-06
9.94E-06
8.45E-06
9.20E-06
4.70E-06
1.84E-06
1.21E-05
1.88E-06
1.88E-06
7.03E-06
7.39E-06
1.11E-06
4.29E-06
4.24E-07
1.45E-06
4.84E-06
2.34E-06
2.86E-06
2.62E-06
1.01E-05
9.31E-06
1.44E-05
5.49E-06
5.59E-06
5.27E-07
9.14E-07
1.13E-05
2.85E-06
2.70E-06

2.68E-06
2.70E-06
1.49E-06
1.05E-06
8.98E-06
3.73E-06
2.03E-06
5.38E-06
1.88E-06
1.03E-05
3.96E-06
1.11E-05
5.46E-06
3.98E-06
3.29E-06
1.91E-06
4.42E-06
4.89E-06
8.72E-06
7.55E-06
4.25E-06
3.79E-06
5.23E-06
8.77E-06
3.89E-06
8.95E-06
1.06E-05
8.69E-06
8.99E-06
4.54E-06
1.27E-06
6.13E-06
7.99E-06
8.30E-06
6.71E-06
8.57E-06
3.36E-06

probeID	ProbeChr	Gene	Probe_bp	trans_chr
ENSGALG00000012196	4	MCUB	58154371	2
ENSGALG00000014026	4	DNAJB14	60128048	2
ENSGALG00000035619	4	MTTP	60028589	2
ENSGALG00000030614	4	ADH1C	59984686	2
ENSGALG00000020160	4	ADH6	59962436	2
ENSGALG00000012211	4	PDLIM5	58197414	2
ENSGALG00000012245	4	ADH4	59946344	2
ENSGALG00000011974	4	RPL7L1	54538589	2
ENSGALG00000002106	8	CLCC1	1724671	4
ENSGALG00000031122	8	NTNG1	1215190	4
ENSGALG00000002164	8	DDX59	1826039	4
ENSGALG00000002129	8	WDR47	1739634	4
ENSGALG00000002369	8	F13B	3032092	4
ENSGALG00000032398	8	ASPM	2987284	4
ENSGALG00000028940	5	NUDT14	52393790	4
ENSGALG00000009309	9	EIF5A2	19871778	4
ENSGALG00000004875	14	PEMT	5190747	6
ENSGALG00000004902	14	TOM1L2	5319981	6
ENSGALG00000042008	26	GSTM3	1269132	6

Table S3. SMR test based or

trans_leftBound	trans_rightBound	topSNP	topSNP_chr	topSNP_bp
128845750	129930691	2:129897204	2	129897204
128845750	129965320	2:129845750	2	129845750
128845750	129965320	2:129845750	2	129845750
128845750	129897204	2:129845750	2	129845750
128845750	129965320	2:129845750	2	129845750
128845750	129965320	2:129930691	2	129930691
128897204	129965320	2:129897204	2	129897204
129845750	130897204	2:129897204	2	129897204
33553496	34554151	4:34554151	4	34554151
33553496	34554151	4:34554151	4	34554151
33553496	34554151	4:34554151	4	34554151
33553496	34554151	4:34554151	4	34554151
33553496	34554151	4:34554151	4	34554151
33553496	34554151	4:34554151	4	34554151
75656076	75696705	4:75686442	4	75686442
75846402	76348471	4:76348471	4	76348471
4593161	5704817	6:5600306	6	5600306
4593161	5694953	6:5641799	6	5641799
4633877	5693831	6:5633877	6	5633877

n the GWAS result of HSC

A1	A2	Freq	b_GWAS	se_GWAS	p_GWAS	b_eQTL	se_eQTL
T	C	0.245509	-0.218	0.0479	4.49E-06	0.229971	0.0404727
A	G	0.235778	-0.238	0.0527	5.39E-06	0.543927	0.0492351
A	G	0.235778	-0.238	0.0527	5.39E-06	0.346674	0.0302353
A	G	0.235778	-0.238	0.0527	5.39E-06	0.288338	0.051504
A	G	0.235778	-0.238	0.0527	5.39E-06	-0.585188	0.0388398
G	A	0.236527	-0.215	0.0477	5.21E-06	-0.376409	0.0551412
T	C	0.245509	-0.218	0.0479	4.49E-06	0.234513	0.0432184
T	C	0.245509	-0.218	0.0479	4.49E-06	-0.214471	0.0406035
C	A	0.385479	-0.211	0.046	6.34E-06	-0.327155	0.0254351
C	A	0.385479	-0.211	0.046	6.34E-06	-0.291387	0.0504991
C	A	0.385479	-0.211	0.046	6.34E-06	-0.36311	0.0319782
C	A	0.385479	-0.211	0.046	6.34E-06	-0.413994	0.0475255
C	A	0.385479	-0.211	0.046	6.34E-06	-0.350215	0.043396
C	A	0.385479	-0.211	0.046	6.34E-06	-0.318039	0.0459616
C	T	0.403443	-0.192	0.0427	5.97E-06	-0.201815	0.0380047
A	G	0.359281	-0.21	0.0457	4.03E-06	-0.15567	0.030232
G	A	0.276946	0.231	0.0465	6.08E-07	-0.335565	0.0316287
C	A	0.235778	0.225	0.0481	2.52E-06	-0.346234	0.0515521
T	C	0.232036	0.226	0.0461	8.28E-07	0.165174	0.0303612

p_eQTL	b_SMR	se_SMR	p_SMR	p_SMR_FDR_adjusted
1.33E-08	-0.947947	0.266863	0.000382042	0.000518486
2.25E-28	-0.437558	0.104671	2.91E-05	9.22E-05
1.96E-30	-0.686524	0.163383	2.65E-05	9.22E-05
2.16E-08	-0.82542	0.234827	0.000439739	0.000557003
2.68E-51	0.406707	0.094015	1.52E-05	9.22E-05
8.72E-12	0.571188	0.151857	0.000168993	0.000291896
5.76E-08	-0.929587	0.266585	0.000488444	0.000580028
1.28E-07	1.01645	0.294808	0.000565071	0.000606971
7.33E-38	0.644955	0.14928	1.56E-05	9.22E-05
7.92E-09	0.724123	0.201669	0.000329861	0.000482104
7.01E-30	0.58109	0.136629	2.11E-05	9.22E-05
3.01E-18	0.50967	0.125576	4.94E-05	0.000133968
7.02E-16	0.602487	0.151082	6.67E-05	0.000158374
4.53E-12	0.663442	0.173529	0.000131713	0.000250255
1.09E-07	0.951368	0.277243	0.00060017	0.000606971
2.62E-07	1.34901	0.393472	0.000606971	0.000606971
2.69E-26	-0.688391	0.15301	6.83E-06	9.22E-05
1.87E-11	-0.64985	0.169298	0.000123795	0.000250255
5.32E-08	1.36826	0.375701	0.000270659	0.000428543

Table S4. SRC analysis between the expression of each

gene_id	gene name	SRC correlation coefficien	p_value
ENSGALG00000007114	APOA1	0.303	4.44E-15
ENSGALG00000007109	APOA4	0.293	3.97E-14
ENSGALG00000030920	APOC3	0.293	4.22E-14
ENSGALG00000045288	CAMK2N1	-0.279	7.02E-13
ENSGALG00000010225		-0.259	2.99E-11
ENSGALG00000004631	DRAXIN	-0.259	3.23E-11
ENSGALG00000004373	KCNJ16	-0.254	7.26E-11
ENSGALG00000044649	P2RX2	0.254	7.88E-11
ENSGALG00000011113	SGIP1	-0.233	2.70E-09
ENSGALG00000005411	MEIOB	-0.231	3.30E-09
ENSGALG00000016325	GSTA3	-0.229	5.12E-09
ENSGALG00000043903		0.224	1.09E-08
ENSGALG00000000318	CSRP1	0.222	1.36E-08
ENSGALG00000000645		-0.220	1.89E-08
ENSGALG00000001320	CYP1A2	-0.213	5.85E-08
ENSGALG00000027415	GRIN2C	0.211	7.40E-08
ENSGALG00000028376	FGF19	-0.204	1.91E-07
ENSGALG00000012322	KCTD16	-0.198	4.46E-07
ENSGALG00000034616	INHBA	0.196	5.64E-07
ENSGALG00000028294		-0.196	5.92E-07
ENSGALG00000050919		0.196	6.00E-07
ENSGALG00000050083	SYCP2L	-0.196	6.07E-07
ENSGALG00000051148		0.190	1.34E-06
ENSGALG00000048656		-0.189	1.56E-06
ENSGALG00000010170		0.187	1.85E-06
ENSGALG00000031069		0.186	2.22E-06
ENSGALG00000014795	TGIF1	-0.186	2.27E-06
ENSGALG00000010391	MMRN1	-0.185	2.35E-06
ENSGALG00000000120	BMP10	-0.185	2.35E-06
ENSGALG00000010139	SSTR1	-0.184	2.82E-06
ENSGALG00000023359	BTBD17	0.183	3.26E-06
ENSGALG00000046316	CFAP97D1	0.182	3.63E-06
ENSGALG00000015283	CEP57L1	0.181	3.92E-06

ENSGALG00000041078	MID1IP1	-0.180	4.47E-06
ENSGALG00000007786	DOK5	0.180	4.82E-06
ENSGALG00000017199	MAML2	0.179	5.30E-06
ENSGALG00000029670	CRYZL1	0.178	5.64E-06
ENSGALG00000042895	BCAR1	-0.178	5.79E-06
ENSGALG00000033591		-0.178	5.83E-06
ENSGALG00000020322		0.177	6.48E-06
ENSGALG00000034294	ATP6V0D2	0.177	6.61E-06
ENSGALG00000001325	CYP1A1	-0.177	7.04E-06
ENSGALG00000026384	PCSK4	0.176	7.35E-06
ENSGALG00000007220	FTH1	-0.176	7.36E-06
ENSGALG00000001319	CAMK1G	0.176	7.38E-06
ENSGALG00000008398	MPP4	0.175	8.44E-06
ENSGALG00000008201	FBXO21	0.175	9.04E-06
ENSGALG00000055056		0.174	9.86E-06
ENSGALG00000009448	IFRD1	-0.173	1.06E-05
ENSGALG00000034094	SP5	-0.173	1.13E-05
ENSGALG00000005768	ETV5	-0.172	1.22E-05
ENSGALG00000007596		-0.172	1.27E-05
ENSGALG00000002581		0.171	1.32E-05
ENSGALG00000046955		0.171	1.33E-05
ENSGALG00000023172		0.171	1.47E-05
ENSGALG00000051301	KHK	0.170	1.58E-05
ENSGALG00000002234	UNC13D	0.169	1.82E-05
ENSGALG00000030941	ELAPOR1	-0.168	2.05E-05
ENSGALG00000006027	TM6SF1	-0.167	2.14E-05
ENSGALG00000019934		0.167	2.20E-05
ENSGALG00000014365	RBPJ	-0.166	2.39E-05
ENSGALG00000014261	UCHL1	-0.166	2.42E-05
ENSGALG00000010343	TSPAN1	-0.166	2.46E-05
ENSGALG00000051871	INHBC	0.164	3.17E-05
ENSGALG00000009318	IFT140	0.163	3.33E-05
ENSGALG00000010507	FCRL4	0.163	3.38E-05
ENSGALG00000039528	PLCE1	0.163	3.43E-05
ENSGALG00000005360	CA4	-0.162	3.70E-05
ENSGALG00000017177	MSANTD4	0.161	4.12E-05
ENSGALG00000005090	SKA2	0.161	4.55E-05

ENSGALG00000026005	DIO2	-0.160	4.70E-05
ENSGALG00000046008	RSPH3	0.160	4.71E-05
ENSGALG00000009743	RPAP3	0.160	4.86E-05
ENSGALG00000050983		-0.160	4.96E-05
ENSGALG00000011353	TNNI3K	0.160	5.00E-05
ENSGALG00000020390		-0.159	5.65E-05
ENSGALG00000010293	RBP	-0.158	5.88E-05
ENSGALG00000005517	KIF9	0.158	5.95E-05
ENSGALG00000009830	MGAT4D	0.157	6.46E-05
ENSGALG00000038532		-0.157	6.47E-05
ENSGALG00000046326	IL1R2	-0.157	7.05E-05
ENSGALG00000041988	SIK1	-0.157	7.10E-05
ENSGALG00000008107	IRS4	-0.156	7.54E-05
ENSGALG00000004035	CRYBA1	0.156	7.61E-05
ENSGALG00000040905	NTAN1	0.156	7.85E-05
ENSGALG00000014828	ECHDC1	0.155	7.91E-05
ENSGALG00000045427	UPP2	-0.154	8.92E-05
ENSGALG00000003879	MFSD2A	0.154	8.92E-05
ENSGALG00000004369	PYGO1	-0.154	8.99E-05
ENSGALG00000021193	STARD5	-0.154	9.02E-05
ENSGALG00000015877	SH3BGRL2	-0.153	0.000101628
ENSGALG00000029235	CPNE4	-0.153	0.00010225
ENSGALG00000013867	PTPN2	-0.153	0.000103215
ENSGALG00000012505	LRFN5	-0.153	0.000106679
ENSGALG00000043399	EZH2	0.152	0.000113854
ENSGALG00000019543	OTULIN	0.152	0.000115323
ENSGALG00000002707	CHRNB2	-0.152	0.000116153
ENSGALG00000006919	POF1B	0.152	0.000118591
ENSGALG00000013090	LOXL4	0.151	0.000121584
ENSGALG00000008859	WDR31	0.151	0.000126195
ENSGALG00000023626	NTN1	-0.151	0.0001262
ENSGALG00000035978	CDCA7L	-0.151	0.000130739
ENSGALG00000036909	BEND5	-0.151	0.000132135
ENSGALG00000014997	PLA1A	-0.151	0.000133596
ENSGALG00000000521	SPIRE2	-0.151	0.00013381
ENSGALG00000011865	SMTN	0.150	0.000141571
ENSGALG00000028638	CNP1	0.150	0.000142179

ENSGALG00000004657	FBXO2	0.150	0.000145798
ENSGALG00000010232	VRTN	-0.150	0.000148375
ENSGALG00000004413	SLC39A11	-0.149	0.000154788
ENSGALG00000017652	TTC9	-0.149	0.000156003
ENSGALG00000007320	FXYD2	0.149	0.000158457
ENSGALG00000004189	MAFK	-0.149	0.000158636
ENSGALG00000009042	ISM1	-0.149	0.000161484
ENSGALG00000041555	COL1A1	0.149	0.000163662
ENSGALG00000000817	ZNF821	0.149	0.000163767
ENSGALG00000009538		-0.148	0.000165408
ENSGALG00000029083	NXPH2	-0.148	0.000175034
ENSGALG00000053278	SUCNR1	0.148	0.000175352
ENSGALG00000016287	NR0B1	-0.148	0.000178851
ENSGALG00000010237	NPC2	-0.147	0.000183646
ENSGALG00000002260	CISH	-0.147	0.000185029
ENSGALG00000040730	RXRG	-0.147	0.000186016
ENSGALG00000016761	LYG2	-0.147	0.000189749
ENSGALG00000016538	RAI2	-0.147	0.000191058
ENSGALG00000010847	VEGFC	-0.147	0.000197586
ENSGALG00000014250	PHOX2B	-0.147	0.000201024
ENSGALG00000036749	MRPS28	-0.146	0.000216058
ENSGALG00000012241	KLHDC1	0.146	0.000218241
ENSGALG00000026396	BG1	0.146	0.000219149
ENSGALG00000011141	ITGB6	-0.146	0.000219169
ENSGALG00000007014	PYROXD2	0.146	0.00021993
ENSGALG00000000745	SLC26A9	-0.145	0.000230765
ENSGALG00000016570		0.144	0.000255098
ENSGALG00000009068	ITPKB	-0.144	0.000256093
ENSGALG00000012812	SVOPL	-0.143	0.00028025
ENSGALG00000013155		-0.143	0.000281599
ENSGALG00000054657		0.143	0.00028215
ENSGALG00000022991	SMIM20	-0.143	0.000289
ENSGALG00000036293	EBAG9	-0.143	0.000289678
ENSGALG00000010811	LGMN	-0.143	0.000291723
ENSGALG00000008072	CHRDL1	-0.143	0.000301767
ENSGALG00000010108		0.142	0.00030807
ENSGALG00000032598	MYO1A	0.142	0.000312935

ENSGALG00000012638	CRY1	-0.142	0.000313224
ENSGALG00000049408	HIST1H2B8	0.142	0.000315176
ENSGALG00000015263		-0.142	0.000332479
ENSGALG00000014252		-0.141	0.000333631
ENSGALG00000039193	AGRN	0.141	0.000338716
ENSGALG00000028336	MED10	-0.141	0.000341732
ENSGALG00000049587		0.141	0.000343383
ENSGALG00000009020	JAG1	-0.141	0.00034569
ENSGALG00000050069		0.141	0.000348642
ENSGALG00000000918	CCDC103	0.141	0.00035583
ENSGALG00000002155	HS6ST1	-0.141	0.000358147
ENSGALG00000051290		-0.141	0.000362062
ENSGALG00000003464		0.141	0.000364309
ENSGALG00000051505	ZNF503	-0.141	0.000364859
ENSGALG00000044780		-0.140	0.000379492
ENSGALG00000051213	DUSP21	0.140	0.000381777
ENSGALG00000003476	ERN1	-0.140	0.000384784
ENSGALG00000026687	PLCD4	0.140	0.000389018
ENSGALG00000012219	UNC5C	-0.140	0.000389938
ENSGALG00000039022	PDE1B	0.140	0.000394725
ENSGALG00000008459	BMPR2	-0.139	0.000410526
ENSGALG00000034168	WNT5A	0.139	0.00041183
ENSGALG00000035242	RMDN3	-0.139	0.000445275
ENSGALG00000046409		-0.138	0.00045777
ENSGALG00000001475	STMN1	-0.138	0.000469564
ENSGALG00000007636	PCK1	0.138	0.000476915
ENSGALG00000007814		-0.138	0.000479423
ENSGALG00000009954	DYNC2LI1	-0.138	0.00048009
ENSGALG00000048343		0.138	0.000490671
ENSGALG00000029001	YRDC	-0.137	0.000502053
ENSGALG00000038145	DPP7	0.137	0.000505771
ENSGALG00000001333	MRRFP1	0.137	0.000507035
ENSGALG00000002121	PLEKHN1	0.137	0.00050945
ENSGALG00000008437	NET1	-0.137	0.000523493
ENSGALG00000014490	P3H3	0.137	0.000532452
ENSGALG00000030719	CLMN	-0.136	0.000542957
ENSGALG00000011327	NR2C1	0.136	0.000549191

ENSGALG00000040474	ITCH	-0.136	0.000549205
ENSGALG00000016932	MZT1	0.136	0.000568701
ENSGALG00000004047	GALE	0.136	0.0005712
ENSGALG00000052986		-0.136	0.000580022
ENSGALG00000005421		0.135	0.000594972
ENSGALG00000045814	CRLF2	-0.135	0.000601207
ENSGALG00000033271	PFKM	0.135	0.000604611
ENSGALG00000011266	PPM1K	-0.135	0.000617477
ENSGALG00000009870	IL15	0.135	0.000620103
ENSGALG00000010960	GALNT3	0.135	0.000621343
ENSGALG00000006662	BPIFB3	0.135	0.00062316
ENSGALG00000021451		-0.135	0.000632018
ENSGALG00000026380	UBE2C	0.135	0.000639666
ENSGALG00000040926	SAMSN1	0.135	0.000640773
ENSGALG00000000227	DPYSL2	-0.134	0.000656703
ENSGALG00000055023		0.134	0.000660484
ENSGALG00000043010	NR2F6	-0.134	0.000668975
ENSGALG00000004623	AGTRAP	-0.134	0.000678707
ENSGALG00000014616	MT3	-0.134	0.000702059
ENSGALG00000039983	EEPD1	-0.134	0.000703552
ENSGALG00000023576	PIM3	-0.133	0.000717648
ENSGALG00000016518	PHKA2	0.133	0.00071937
ENSGALG00000015692		0.133	0.000725656
ENSGALG00000016779	RNF149	-0.133	0.000725706
ENSGALG00000009807	FLVCR1	-0.133	0.000762932
ENSGALG00000000607	GPR37L1	-0.133	0.000765777
ENSGALG00000033144	COLEC10	-0.133	0.000780363
ENSGALG00000005796	XBP1	-0.133	0.000782979
ENSGALG00000054629	PDCD2L	0.133	0.000784684
ENSGALG00000006267	TPX2	0.132	0.000796816
ENSGALG00000034297	TBX2	-0.132	0.000804039
ENSGALG00000031164	WFDC2	-0.132	0.000807099
ENSGALG00000002311	GALK1	0.132	0.000816425
ENSGALG00000006189	CRYGN	0.132	0.000825714
ENSGALG00000025935	XPNPEP2	-0.132	0.000832492
ENSGALG00000008949	ADRB1	-0.132	0.00083423
ENSGALG00000050372	SYCN	-0.131	0.000897666

ENSGALG00000008285	NAPEPLD	-0.131	0.000902665
ENSGALG00000003702	CLINT1	-0.131	0.000915511
ENSGALG00000013624		-0.131	0.000920441
ENSGALG00000007610	CDC23	0.131	0.000922464
ENSGALG00000011880	BBS7	0.131	0.000926567
ENSGALG00000005707	IFT20	-0.131	0.000928035
ENSGALG00000015314	BEND3	-0.131	0.000937455
ENSGALG00000007999	MRPS7	-0.131	0.00093904
ENSGALG00000025884	NABP1	-0.131	0.00094317
ENSGALG00000042929		-0.130	0.000945663
ENSGALG00000051466	NDFIP2	-0.130	0.000958131
ENSGALG00000048285	TCN2	-0.130	0.000973516
ENSGALG00000007382	CDA	-0.130	0.000980662
ENSGALG00000044816		0.130	0.00098785
ENSGALG00000015233	ATP1B1	-0.130	0.001033118
ENSGALG00000014998	CABLES1	-0.129	0.001045384
ENSGALG00000039538	CLDND1	-0.129	0.001057745
ENSGALG00000035596	NT5DC2	-0.129	0.001058134
ENSGALG00000006153	EXOG	0.129	0.001061669
ENSGALG00000023517	AGPAT2	-0.129	0.001092253
ENSGALG00000031996		0.129	0.001120403
ENSGALG00000026663	CX3CL1	0.129	0.001126864
ENSGALG00000012882	KDSR	-0.128	0.001133908
ENSGALG00000013743	ENPP7	-0.128	0.001146832
ENSGALG00000052753		-0.128	0.001154207
ENSGALG00000048309		-0.128	0.001170109
ENSGALG00000003283		0.128	0.001179106
ENSGALG00000013632	ACOT13	0.128	0.001182803
ENSGALG00000025804	CLCN2	0.128	0.001184665
ENSGALG00000026607		-0.128	0.001188294
ENSGALG00000006805	PLSCR1	-0.128	0.00119874
ENSGALG00000048035	GCNT2	-0.128	0.001199863
ENSGALG00000040101		0.128	0.001203433
ENSGALG00000026161		0.128	0.001213194
ENSGALG00000004249	GRHL3	-0.128	0.001229787
ENSGALG00000037773	ST3GAL1	-0.128	0.001233696
ENSGALG00000049798	HDGFL3	-0.127	0.001240547

ENSGALG00000034434	IDH2	-0.127	0.001260581
ENSGALG00000014971	SLC2A9	-0.127	0.00128369
ENSGALG00000002764	ATP1B3	-0.127	0.001294515
ENSGALG00000007582	DPYSL3	-0.127	0.001300698
ENSGALG00000033941	RORC	0.127	0.001305059
ENSGALG00000003016	MASP2	-0.127	0.00132007
ENSGALG00000052318		0.127	0.001337753
ENSGALG00000012196	MCUB	-0.126	0.001369185
ENSGALG00000042159	SLITRK3	-0.126	0.001379437
ENSGALG00000030324	ELAPOR2	-0.126	0.001385315
ENSGALG00000039334		-0.126	0.001405181
ENSGALG00000027188	SREBF1	-0.126	0.001411633
ENSGALG00000049658		-0.125	0.001490397
ENSGALG00000036894	ABCA2	0.125	0.001494309
ENSGALG00000052088	SFR1	0.125	0.001505763
ENSGALG00000037261	RFXANK	-0.125	0.001520717
ENSGALG00000030024	USP15	-0.125	0.001535868
ENSGALG00000027920		-0.125	0.001539106
ENSGALG00000004220	SERINC3	-0.125	0.001539347
ENSGALG00000031238	SLIRP	0.125	0.001575133
ENSGALG00000010969	SPIA4	-0.125	0.001581358
ENSGALG00000013135	GALNT1	-0.125	0.001585608
ENSGALG00000048109		-0.125	0.001585734
ENSGALG00000035016	ID2	-0.125	0.001597405
ENSGALG00000001701		0.124	0.001625418
ENSGALG00000009276	LRAT	-0.124	0.001642941
ENSGALG00000047684		-0.124	0.001661105
ENSGALG00000039454	SP4	-0.124	0.001662966
ENSGALG00000026081	RBM38	0.124	0.00166855
ENSGALG00000042308	FZD3	-0.124	0.00167225
ENSGALG00000002804	RND2	0.124	0.001678456
ENSGALG00000017099	RASL11A	0.124	0.001684768
ENSGALG00000016885	STK24	-0.124	0.001685071
ENSGALG00000016459	LRATD1	-0.124	0.001688594
ENSGALG00000012877	CREB3L2	-0.124	0.001700183
ENSGALG00000028250	ANKRD22	-0.124	0.001712554
ENSGALG00000049421		-0.124	0.001713127

ENSGALG00000001616	CDC45	0.124	0.0017165
ENSGALG00000029168		-0.124	0.001720712
ENSGALG00000036725	ASCL3	0.124	0.001738796
ENSGALG00000003572	ARHGEF18	-0.123	0.001767243
ENSGALG00000005675		-0.123	0.001767772
ENSGALG00000048158		-0.123	0.001792101
ENSGALG00000008133	AS3MT	0.123	0.001794022
ENSGALG00000004860	RASD1	-0.123	0.001800338
ENSGALG00000033171	TGM4	0.123	0.001803408
ENSGALG00000001863	VTG2	-0.123	0.001809073
ENSGALG00000053601	LPXN	0.123	0.001828655
ENSGALG00000013863	PSMG2	-0.123	0.001832339
ENSGALG00000053245		-0.123	0.001833669
ENSGALG00000000949	HBEGF	0.123	0.001840991
ENSGALG00000026945	CIDEC	0.123	0.001842331
ENSGALG00000012823	TRIM24	-0.123	0.001845082
ENSGALG00000000545	REN	0.123	0.001856014
ENSGALG00000052887		-0.123	0.001860929
ENSGALG00000036190	AOC1	-0.123	0.001888912
ENSGALG00000037214		-0.122	0.001924246
ENSGALG00000004309	GDAP1L1	0.122	0.001936588
ENSGALG00000004637		0.122	0.001956444
ENSGALG00000012847		-0.122	0.001958231
ENSGALG00000052452		0.122	0.00195899
ENSGALG00000015219		-0.122	0.001963444
ENSGALG00000011419	DUSP4	-0.122	0.001976667
ENSGALG00000008953	AASS	-0.122	0.001983364
ENSGALG00000027369	DGCR6	0.122	0.002012584
ENSGALG00000034218		-0.122	0.002017611
ENSGALG00000010020	TTC7A	-0.122	0.002052269
ENSGALG00000010785	ACOT11	0.122	0.002064045
ENSGALG00000008091	PTPMT1	-0.122	0.002075138
ENSGALG00000030776		-0.122	0.002077751
ENSGALG00000043831	LRRC73	-0.122	0.002080453
ENSGALG00000038096	NOS2	0.122	0.002086169
ENSGALG00000043026		0.122	0.002089608
ENSGALG00000037784	DAG1	-0.121	0.002100566

ENSGALG00000016576	OFD1	0.121	0.002128082
ENSGALG00000010009	TTC29	-0.121	0.002184398
ENSGALG00000047713	QPRT	0.121	0.002191135
ENSGALG00000050224	GOLT1A	-0.121	0.002228322
ENSGALG00000035842	SLC37A2	0.121	0.002229288
ENSGALG00000053013		-0.121	0.002230993
ENSGALG00000011132	RBMS1	-0.121	0.002252343
ENSGALG00000008903	ITPRID2	-0.121	0.002261639
ENSGALG00000017462	FNIP1	-0.121	0.00226882
ENSGALG00000033920		0.120	0.002285077
ENSGALG00000008240	CFLAR	-0.120	0.002310975
ENSGALG00000030863		0.120	0.002336157
ENSGALG00000011254	SATB1	-0.120	0.002337099
ENSGALG00000004416	TTC19	0.120	0.0023436
ENSGALG00000005815	TMEM41B	-0.120	0.002352659
ENSGALG00000029811	BMPER	-0.120	0.002356371
ENSGALG00000015666	TNIP2	-0.120	0.002360888
ENSGALG00000042748		0.120	0.002362919
ENSGALG00000043044	IL1R1	-0.120	0.002375977
ENSGALG00000008631	AXL	-0.120	0.002378227
ENSGALG00000013846	ARFGEF3	-0.120	0.002413362
ENSGALG00000013001		-0.120	0.002423376
ENSGALG00000010401	EIF2A	0.120	0.002435282
ENSGALG00000010189		-0.120	0.002438032
ENSGALG00000037361	KLF8	-0.120	0.002447723
ENSGALG00000008604	TMEM255A	-0.120	0.002475754
ENSGALG00000042073		-0.120	0.002479146
ENSGALG00000000847	GARNL3	0.119	0.002486775
ENSGALG00000015708	FGFR3	-0.119	0.002486898
ENSGALG00000040947	CREG2	-0.119	0.002493701
ENSGALG00000003298	DDX55	0.119	0.002502617
ENSGALG00000006192	USH1C	-0.119	0.002507746
ENSGALG00000027872	STBD1	-0.119	0.002510205
ENSGALG00000053778		0.119	0.002539356
ENSGALG00000026253	TMEM144	0.119	0.002540103
ENSGALG00000053035		-0.119	0.002544918
ENSGALG00000034282		0.119	0.002602243

ENSGALG00000016931	BORA	0.119	0.002608887
ENSGALG00000029618		0.119	0.002640091
ENSGALG00000009424	FOXP2	-0.119	0.002657069
ENSGALG00000004449	CYP3A5	-0.119	0.002658277
ENSGALG00000007791	OTOP2	-0.119	0.002660609
ENSGALG00000038217	CD82	-0.119	0.002663081
ENSGALG00000012377	HNMT	-0.119	0.002665075
ENSGALG00000014233	FBLN1	-0.118	0.00269952
ENSGALG00000015040	SLC16A10	-0.118	0.00270442
ENSGALG00000017122	SGCG	-0.118	0.002724845
ENSGALG00000009639	DDX60	-0.118	0.002728769
ENSGALG00000004196	UGT1A1	-0.118	0.002729264
ENSGALG00000009682	VEPH1	-0.118	0.002745091
ENSGALG00000000011	C10orf88	0.118	0.002747604
ENSGALG00000026834	PLPP2	-0.118	0.002762337
ENSGALG00000007203	RAB3IL1	-0.118	0.002770529
ENSGALG00000052084		0.118	0.002771551
ENSGALG00000028622		-0.118	0.002772696
ENSGALG00000005078	NUP210	-0.118	0.002774982
ENSGALG00000007017	RBM7	-0.118	0.002777851
ENSGALG00000019844	AvBD8	0.118	0.002789344
ENSGALG00000042642	RARRES2	-0.118	0.002789577
ENSGALG00000044889	FYB2	-0.118	0.002791412
ENSGALG00000026210	POMK	-0.118	0.002806246
ENSGALG00000005572	NOXO1	-0.118	0.002823323
ENSGALG00000004902	TOM1L2	-0.118	0.002835672
ENSGALG00000011900	NAGA	-0.118	0.002853964
ENSGALG00000031255	FGF1	0.118	0.002867092
ENSGALG00000053585		0.118	0.002877817
ENSGALG00000051554		-0.118	0.002915108
ENSGALG00000037739	STK25	-0.118	0.00291519
ENSGALG00000017493		0.117	0.002936085
ENSGALG00000014060	EED	0.117	0.002939654
ENSGALG00000046049		0.117	0.002943211
ENSGALG00000033593	UBE2E2	-0.117	0.00294588
ENSGALG00000017392	SPDYA	0.117	0.002957501
ENSGALG00000035352	ZBTB17	0.117	0.002964151

ENSGALG00000054542		-0.117	0.002970828
ENSGALG00000007822	MRPL58	0.117	0.002971307
ENSGALG00000054779	FAM206A	0.117	0.00298309
ENSGALG00000020410	TMED8	-0.117	0.002985527
ENSGALG00000023395	PLIN1	0.117	0.003006109
ENSGALG00000017173	GUCY1A2	-0.117	0.003013097
ENSGALG00000021688	CD320	0.117	0.003022541
ENSGALG00000039170	LHFPL5	-0.117	0.00302583
ENSGALG00000026518	RUNDC3A	0.117	0.003031381
ENSGALG00000005981	GDF2	-0.117	0.003036456
ENSGALG00000006050	CYP2R1	-0.117	0.003055199
ENSGALG00000009855	TBC1D9	-0.117	0.00306484
ENSGALG00000005985	GDF10	-0.117	0.003080366
ENSGALG00000053550	ESPNL	0.117	0.003123913
ENSGALG00000005966	SEC61A1	-0.117	0.003130173
ENSGALG00000013969	ALDH8A1	0.117	0.003143
ENSGALG00000011205	SMOC2	-0.116	0.003203063
ENSGALG00000010303		0.116	0.003211224
ENSGALG00000013818	CITED2	-0.116	0.00321846
ENSGALG00000040969	PTP4A3	-0.116	0.003235559
ENSGALG00000002594	TFPI	0.116	0.003237399
ENSGALG00000007533	NPEPL1	-0.116	0.003245803
ENSGALG00000039839	HEXD	0.116	0.003273299
ENSGALG00000023444	APELA	-0.116	0.00328266
ENSGALG00000006880	SLC38A10	-0.116	0.003298278
ENSGALG00000034316	CNOT6	-0.116	0.003338369
ENSGALG00000004986	CERCAM	0.116	0.003375547
ENSGALG00000053299	KCNC3	-0.116	0.003377768
ENSGALG00000030007		0.116	0.003378389
ENSGALG00000042553	RAP2B	-0.116	0.003394183
ENSGALG00000011806		-0.116	0.003405102
ENSGALG00000028466		-0.116	0.003405208
ENSGALG00000004304	TCF12	-0.116	0.003422102
ENSGALG00000038933	FABP7	-0.116	0.003430677
ENSGALG00000009565	EIF2S1	-0.115	0.003463792
ENSGALG00000014589		-0.115	0.003469167
ENSGALG00000040619	BAAT	-0.115	0.003472768

ENSGALG00000010649	CNST	-0.115	0.003484708
ENSGALG00000012135	ATF4	-0.115	0.003486928
ENSGALG00000003777	NELL1	-0.115	0.003507684
ENSGALG00000008152	SPATS2L	-0.115	0.003520612
ENSGALG00000004590	CLCN6	-0.115	0.003533606
ENSGALG00000010864	EREG	0.115	0.003537296
ENSGALG00000050796		0.115	0.003542491
ENSGALG00000010762	OPN3	-0.115	0.003545564
ENSGALG00000023521	ADRA2A	-0.115	0.003553998
ENSGALG00000005293	GNG13	0.115	0.00356855
ENSGALG00000042621	HNF4G	-0.115	0.003570984
ENSGALG00000032975	UBE2QL1	-0.115	0.003576227
ENSGALG00000048024		0.115	0.003624327
ENSGALG00000036785	TRIM8	-0.115	0.003628661
ENSGALG00000001310	TRNAU1AP	0.115	0.003630779
ENSGALG00000000883	MAPKAPK2	-0.115	0.003637908
ENSGALG00000035819	TMEM51	-0.115	0.003648914
ENSGALG00000002647	PLS1	0.115	0.003671261
ENSGALG00000033338	GPT2	-0.115	0.003674369
ENSGALG00000015142	DSG2	-0.115	0.003712791
ENSGALG00000010805	USP24	-0.115	0.003713474
ENSGALG00000015136	ILDR1	-0.115	0.003718504
ENSGALG00000031186		0.115	0.003718558
ENSGALG00000011149	PLA2R1	-0.114	0.00377129
ENSGALG00000007778	PES1	-0.114	0.003775835
ENSGALG00000007419	MASP1	-0.114	0.003802582
ENSGALG00000010013	EDNRA	-0.114	0.003809823
ENSGALG00000039450	SOS1	-0.114	0.003826963
ENSGALG00000052192	TUBA1B	-0.114	0.003826994
ENSGALG00000037939	ADGRG1	-0.114	0.003830923
ENSGALG00000012185	PLA2G12A	-0.114	0.003832987
ENSGALG00000017715	TINAGL1	-0.114	0.003835236
ENSGALG00000052151		-0.114	0.003857486
ENSGALG00000029863	PHYHIPL	0.114	0.003868201
ENSGALG00000009977	SMAD1	-0.114	0.003885633
ENSGALG00000041650	DUSP7	-0.114	0.003886168
ENSGALG00000042454	DCDC2	-0.114	0.003913476

ENSGALG00000002262	ETF1	-0.114	0.003937482
ENSGALG00000009848	LPGAT1	-0.114	0.003949542
ENSGALG00000035883	ZMYND10	0.114	0.00394972
ENSGALG00000008520	EIF4G1	-0.114	0.003963168
ENSGALG00000049272		-0.114	0.003974353
ENSGALG00000004231		-0.114	0.003977194
ENSGALG00000010653	SMYD3	-0.114	0.003978174
ENSGALG00000042733		0.114	0.003994253
ENSGALG00000016690	CYP2AC1	0.114	0.004007859
ENSGALG00000009249	UBR1	-0.114	0.004046446
ENSGALG00000049504		0.114	0.004058222
ENSGALG00000012542	RASD2	-0.114	0.004059489
ENSGALG00000028530		-0.113	0.004099519
ENSGALG00000022909	PTPN3	-0.113	0.004105585
ENSGALG00000017524	DOHH	-0.113	0.004125334
ENSGALG00000007023	PRKAR2A	-0.113	0.004169496
ENSGALG00000028709	RNF144A	-0.113	0.004173273
ENSGALG00000015908	COL12A1	0.113	0.00418151
ENSGALG00000038582	SLC36A1	-0.113	0.004222977
ENSGALG00000020388		-0.113	0.004233124
ENSGALG00000016262	XK	-0.113	0.004245432
ENSGALG00000012691	ALDH1L2	0.113	0.004251298
ENSGALG00000047005		0.113	0.004256697
ENSGALG00000039200	AXIN2	-0.113	0.004286573
ENSGALG00000041238		-0.113	0.004286671
ENSGALG00000006062	ZBTB46	-0.113	0.004296078
ENSGALG00000031758	SUGCT	0.113	0.004327811
ENSGALG00000039549	SEL1L3	-0.113	0.004360245
ENSGALG00000008684	EIF4A2	-0.113	0.004378064
ENSGALG00000042325	CIZ1	0.113	0.004379592
ENSGALG00000054577		0.113	0.004381907
ENSGALG00000040241	NTRK3	0.113	0.004398407
ENSGALG00000015093	IMPACT	0.113	0.004403705
ENSGALG00000015895	HTR1B	0.112	0.004448681
ENSGALG00000041934	THOP1	-0.112	0.004458363
ENSGALG00000027287	HK3	0.112	0.004483529
ENSGALG00000014937	KPNA5	-0.112	0.004495215

ENSGALG00000039698	DRD1	-0.112	0.004505195
ENSGALG00000007075	ATP13A3	-0.112	0.004513509
ENSGALG00000037410	HEPH	-0.112	0.004531268
ENSGALG00000052087		-0.112	0.004539769
ENSGALG00000011805		-0.112	0.004542861
ENSGALG00000003932	SDC4	-0.112	0.004571806
ENSGALG00000007752	COPS7B	-0.112	0.004620227
ENSGALG00000003876	TIMD4	-0.112	0.004628278
ENSGALG00000041792	C26H6orf106	-0.112	0.004645231
ENSGALG00000028971	NKIRAS2	-0.112	0.004657448
ENSGALG00000031735	CD5	0.112	0.004681874
ENSGALG00000011917	PPM1A	-0.112	0.00469941
ENSGALG00000002945	TMEM266	0.112	0.004711508
ENSGALG00000017032	SLC25A15	0.112	0.004733855
ENSGALG00000046708	PTGES	0.112	0.004734193
ENSGALG00000005631	TRPM2	0.112	0.00475435
ENSGALG00000043087	MTF2	-0.112	0.004758176
ENSGALG00000011796		-0.112	0.004768929
ENSGALG00000035993	SUOX	-0.111	0.004779575
ENSGALG00000030008	HHEX	-0.111	0.004791952
ENSGALG00000052770		0.111	0.004794241
ENSGALG00000029829	RNLS	-0.111	0.004835231
ENSGALG00000011735	XCR1	-0.111	0.004872306
ENSGALG00000051980		-0.111	0.004893383
ENSGALG00000004282	RCAN3	-0.111	0.00491472
ENSGALG00000029599	ACVR1B	-0.111	0.004915766
ENSGALG00000005481	ARHGEF3	-0.111	0.004957175
ENSGALG00000047639		0.111	0.004957196
ENSGALG00000007130	RAC1	-0.111	0.004982466
ENSGALG00000007070		0.111	0.004989831
ENSGALG00000003085	CDK1	0.111	0.00499376
ENSGALG00000011124	ARHGAP24	-0.111	0.005006511
ENSGALG00000039742	CYP21A1	0.111	0.005028244
ENSGALG00000016300	TINAG	-0.111	0.005030026
ENSGALG00000009955	ABCG5	-0.111	0.005047317
ENSGALG00000006682	BEND7	-0.111	0.005066006
ENSGALG00000052983		-0.111	0.005080733

ENSGALG00000015699	NELFA	-0.111	0.00508831
ENSGALG00000020417	ISCA2	-0.111	0.005107713
ENSGALG00000034050		-0.111	0.005115895
ENSGALG00000052918		-0.111	0.005123434
ENSGALG00000035903	FAM46A	-0.111	0.005127451
ENSGALG00000014426	KPNA1	-0.111	0.005127785
ENSGALG00000047495	LRRC10	-0.111	0.005129556
ENSGALG00000040367	P2RY14	-0.111	0.005151068
ENSGALG00000037675	COL14A1	-0.110	0.005184038
ENSGALG00000010005	EPAS1	-0.110	0.005195593
ENSGALG00000036297	ABHD8	0.110	0.005211529
ENSGALG00000008728	PTER	-0.110	0.005213949
ENSGALG00000006156	COG7	0.110	0.005220352
ENSGALG00000052326	IRF2BP2	-0.110	0.005222655
ENSGALG00000012766		0.110	0.005231059
ENSGALG00000030151	LUZP2	0.110	0.005233561
ENSGALG00000053999	VPS37D	0.110	0.00531443
ENSGALG00000030886	PTGDS	-0.110	0.005340334
ENSGALG00000027096	LGALSL	-0.110	0.005356813
ENSGALG00000003092	ERLIN2	-0.110	0.005382748
ENSGALG00000007485	TFRC	-0.110	0.005384589
ENSGALG00000005314	APPBP2	-0.110	0.005396121
ENSGALG00000045196	LAPTM4B	-0.110	0.005401035
ENSGALG00000039887		-0.110	0.005405049
ENSGALG00000047321		0.110	0.005412494
ENSGALG00000011966	RANGAP1	-0.110	0.005428079
ENSGALG00000009119	THUMPD2	-0.110	0.0054385
ENSGALG00000000625	MAPT	0.110	0.00547599
ENSGALG00000033974	HGF	-0.110	0.005478255
ENSGALG00000008912	ABCB1	0.110	0.005484121
ENSGALG00000029691	NFKBIB	-0.110	0.005502808
ENSGALG00000015112	ZNF521	0.110	0.005506219
ENSGALG00000030877	CLUAP1	0.110	0.005523474
ENSGALG00000037238	PCCB	0.110	0.005554391
ENSGALG00000041927	FZD9	-0.110	0.00556917
ENSGALG00000005897	HMCES	-0.110	0.005572922
ENSGALG00000003578	FN1	-0.110	0.005581005

ENSGALG00000004215	WIPI1	-0.109	0.005594755
ENSGALG00000042492	PITX2	-0.109	0.005603669
ENSGALG00000023686	ZSWIM7	0.109	0.005606338
ENSGALG00000015795	ADAMTS5	-0.109	0.005608248
ENSGALG00000002709	MYO1C	-0.109	0.00563379
ENSGALG00000005000	COMTD1	-0.109	0.005653426
ENSGALG00000012021		-0.109	0.005683071
ENSGALG00000004701	SET	-0.109	0.005704027
ENSGALG00000033541	FRMPD4	-0.109	0.005705773
ENSGALG00000003015	SERPINF1	-0.109	0.005712245
ENSGALG00000032563	FBXO34	-0.109	0.00572737
ENSGALG00000036069	TCIRG1	-0.109	0.005753465
ENSGALG00000001030	SCAMP4	-0.109	0.005768918
ENSGALG00000030250	SV2B	-0.109	0.005810668
ENSGALG00000054836		0.109	0.005816755
ENSGALG00000039455	VAPA	-0.109	0.005842338
ENSGALG00000005971	RIC3	-0.109	0.005855226
ENSGALG00000049461	BG8	0.109	0.0058705
ENSGALG00000016912	EDNRB	-0.109	0.005888367
ENSGALG00000016555	HADHB	0.109	0.005902382
ENSGALG00000009842	RASSF3	-0.109	0.005933808
ENSGALG00000005784	CCDC117	-0.109	0.005976282
ENSGALG00000046739		-0.109	0.005983353
ENSGALG00000007025	CPNE8	-0.109	0.006003928
ENSGALG00000004612	MTHFR	-0.109	0.006017071
ENSGALG00000009920	COCH	-0.108	0.00606333
ENSGALG00000006437	NOM1	-0.108	0.00608328
ENSGALG00000019794	MSTO1	0.108	0.006091173
ENSGALG00000011237	DPH3P1	-0.108	0.006097207
ENSGALG00000004557	WIPI2	-0.108	0.006146622
ENSGALG00000042067	SPRY1	-0.108	0.006151824
ENSGALG00000001663	MAK16	-0.108	0.006181743
ENSGALG00000032882	EVA1C	0.108	0.006188373
ENSGALG00000049579		-0.108	0.006215408
ENSGALG00000040866	P3H2	-0.108	0.006219139
ENSGALG00000000409	CHMP7	-0.108	0.006234225
ENSGALG00000040360	DUSP1	-0.108	0.006240576

ENSGALG00000009971	CDC20	0.108	0.006265539
ENSGALG00000005763	VILL	-0.108	0.006280295
ENSGALG00000041139	PINX1	-0.108	0.006297
ENSGALG00000009782	NAA15	-0.108	0.006297886
ENSGALG00000011288	DACT2	-0.108	0.006331997
ENSGALG00000004969	TIMP4	-0.108	0.006337229
ENSGALG00000003767	NKD1	-0.108	0.006337957
ENSGALG00000028815		0.108	0.006378563
ENSGALG00000036125	SHE	-0.108	0.00638471
ENSGALG00000000806	LINGO3	-0.108	0.006424701
ENSGALG00000016461	DDX1	0.108	0.006426123
ENSGALG00000012985	SLC6A13	0.108	0.00643273
ENSGALG00000013804		-0.108	0.006464837
ENSGALG00000004882	PPCS	-0.108	0.006465057
ENSGALG00000038758	MMP15	-0.108	0.006470496
ENSGALG00000013865	IFNGR1	-0.108	0.006493627
ENSGALG00000006663	ARHGEF12	-0.107	0.006542033
ENSGALG00000027207	PERP2	-0.107	0.006636069
ENSGALG00000004376	KCNJ2	-0.107	0.006638112
ENSGALG00000001021	MYOCD	-0.107	0.006640098
ENSGALG00000050582		-0.107	0.006662002
ENSGALG00000049812	SLC66A2	-0.107	0.006690822
ENSGALG00000034528	SNTB1	-0.107	0.006694268
ENSGALG00000011432	CDC42BPB	-0.107	0.006732965
ENSGALG00000007478	SLC51A	0.107	0.006736825
ENSGALG00000016997	RB1	-0.107	0.006752285
ENSGALG00000009483	MARK1	-0.107	0.006762153
ENSGALG00000005807	IPO7	-0.107	0.006803747
ENSGALG00000003147	TRPC4AP	-0.107	0.006819662
ENSGALG00000012299	ANKRD54	-0.107	0.006856646
ENSGALG00000048098		-0.107	0.006878569
ENSGALG00000031527	ARL8BL	-0.107	0.006884974
ENSGALG00000007193		0.107	0.00689166
ENSGALG00000018976	LYSMD2	-0.107	0.006893746
ENSGALG00000029033		-0.107	0.006925448
ENSGALG00000027579	MRPS6	-0.107	0.006966569
ENSGALG00000000557	ETNK2	-0.107	0.006982399

ENSGALG00000008523	XPNPEP1	-0.107	0.007002999
ENSGALG00000000309		-0.107	0.007012525
ENSGALG00000020391	SERPINA10	-0.107	0.007047426
ENSGALG00000004285	HNF4A	-0.106	0.007051285
ENSGALG00000012816	SLC22A23	-0.106	0.007078105
ENSGALG00000011007	OSBPL3	-0.106	0.007081609
ENSGALG00000047859	TSPAN31	-0.106	0.007082039
ENSGALG00000015412	ARL13B	-0.106	0.007093039
ENSGALG00000029402	AXIN1	-0.106	0.007116112
ENSGALG00000009926	HAAO	0.106	0.007127393
ENSGALG00000050887	CACNB3	-0.106	0.007173774
ENSGALG00000015961	SUCLG1	-0.106	0.007195522
ENSGALG00000008932	ENTPD8	-0.106	0.007247481
ENSGALG00000005951	VPS29L	0.106	0.007279487
ENSGALG00000016511	ADGRG2	-0.106	0.007279516
ENSGALG00000033500	PDCD6IP	-0.106	0.007290632
ENSGALG00000030263	RABL6	-0.106	0.007297063
ENSGALG00000004661	SHISA5	-0.106	0.007399636
ENSGALG00000017378	CRTAC1	0.106	0.007423544
ENSGALG00000029971	PGLYRP2	-0.106	0.007437684
ENSGALG00000004700	NCF2	0.106	0.007456445
ENSGALG00000006889	TMC7	-0.106	0.007470476
ENSGALG00000044463		-0.106	0.007506556
ENSGALG00000047027		-0.106	0.00756134
ENSGALG00000054005	PPP1R37	-0.106	0.007591845
ENSGALG00000034761		0.105	0.007614288
ENSGALG00000006061	RAP2C	-0.105	0.007629146
ENSGALG00000011094	PDE4B	-0.105	0.007636031
ENSGALG00000054442		-0.105	0.00764828
ENSGALG00000043373	ARVCF	-0.105	0.007679454
ENSGALG00000005972	KBTBD12	-0.105	0.007682822
ENSGALG00000050250		0.105	0.007712332
ENSGALG00000026239	MPV17	0.105	0.007716531
ENSGALG00000016027	CBR3	-0.105	0.007741264
ENSGALG00000034438	GNB3	0.105	0.007752093
ENSGALG00000032159	FBXL14	-0.105	0.007768352
ENSGALG00000009438	SLC39A9	-0.105	0.00777613

ENSGALG00000007111	KCTD6	-0.105	0.007787598
ENSGALG00000026906	TMEM189	-0.105	0.007794989
ENSGALG00000033099	PAPOLB	-0.105	0.007816206
ENSGALG00000037301	ACVR1	-0.105	0.007824692
ENSGALG00000004019	MARS2	-0.105	0.00787029
ENSGALG00000038635		-0.105	0.007894479
ENSGALG00000004745	NATD1	-0.105	0.007906397
ENSGALG00000004198	PDZD11	-0.105	0.007907813
ENSGALG00000011175	TMEM150C	-0.105	0.007921241
ENSGALG00000026007	DOLK	-0.105	0.007933035
ENSGALG00000040000	ERO1B	-0.105	0.007939656
ENSGALG00000012420	CG-1B	0.105	0.007960833
ENSGALG00000005587	EIF4G2	-0.105	0.007963543
ENSGALG00000042077	HIC1	-0.105	0.007968922
ENSGALG00000012448	ORC4	0.105	0.008017181
ENSGALG00000052328		-0.105	0.008072471
ENSGALG00000001605	PPM1J	-0.105	0.008076145
ENSGALG00000015665	SH3BP2	-0.105	0.008088799
ENSGALG00000030460	TBC1D31	0.105	0.008089684
ENSGALG00000055035		0.105	0.008093896
ENSGALG00000044788	ZNF628	-0.105	0.008112862
ENSGALG00000021341	ADAM20	0.105	0.0081222
ENSGALG00000013838		0.105	0.008144888
ENSGALG00000048866	MARK2	-0.105	0.008173563
ENSGALG00000026983	CHRD	0.105	0.008177684
ENSGALG00000009732	PCDH18	-0.105	0.008187453
ENSGALG00000006425	SLC6A6	-0.104	0.008217497
ENSGALG00000040794	CCNE2	0.104	0.008222515
ENSGALG00000011421	HAL	-0.104	0.008237925
ENSGALG00000001054	C20H20ORF24	-0.104	0.008240197
ENSGALG00000034992	FRMD4A	-0.104	0.008246466
ENSGALG00000003560	SLC6A2	-0.104	0.008272168
ENSGALG00000012798	SSR1	-0.104	0.008278995
ENSGALG00000040244	SEPTIN9	-0.104	0.008284503
ENSGALG00000043675	ANTXR2	-0.104	0.008294249
ENSGALG00000008280	PMPCB	-0.104	0.008321301
ENSGALG00000040557	TFEC	-0.104	0.008406417

ENSGALG00000021627	IFI27L2	0.104	0.008415673
ENSGALG00000009222	FNDC3B	-0.104	0.008428359
ENSGALG00000003533	ZNF511	0.104	0.00843907
ENSGALG00000003536	KLHL11	-0.104	0.008498953
ENSGALG00000011379	SLC44A5	-0.104	0.008549999
ENSGALG00000014614	TNKS2	-0.104	0.008560114
ENSGALG00000040087	ZNF609	-0.104	0.008563355
ENSGALG00000011685	CSTF3	0.104	0.008564076
ENSGALG00000038097	EI24	0.104	0.0085722
ENSGALG00000011524	PPEF2	0.104	0.008588007
ENSGALG00000006426	PAK2	-0.104	0.008641994
ENSGALG00000001737	RABEP1	-0.104	0.008653909
ENSGALG00000052646	EXD3	0.104	0.008665741
ENSGALG00000053509	DDX11	0.104	0.008682604
ENSGALG00000055006	AHDC1	0.104	0.008698437
ENSGALG00000006158	ACVR2B	0.104	0.008714989
ENSGALG00000009429	RXFP1	0.104	0.008796007
ENSGALG00000001780	PROC	-0.104	0.008798042
ENSGALG00000002763	FRZB	-0.104	0.00880535
ENSGALG00000003374	ESRP2	-0.103	0.008858301
ENSGALG00000033925		-0.103	0.008867833
ENSGALG00000005823	HDLBP	-0.103	0.008912882
ENSGALG00000050350	HIST1H2B7	0.103	0.008924748
ENSGALG00000040005	PPP2R5D	-0.103	0.008938202
ENSGALG00000009676	METTL5	-0.103	0.008956061
ENSGALG00000001531	FN3K	0.103	0.008979547
ENSGALG00000028451	MT4	-0.103	0.008982933
ENSGALG00000046740		0.103	0.009037326
ENSGALG00000002598	TPST1	-0.103	0.009052946
ENSGALG00000005803	ZNRF3	-0.103	0.009061123
ENSGALG00000048331	LRFN1	-0.103	0.009130331
ENSGALG00000003424	LMX1A	-0.103	0.009142284
ENSGALG00000008752		-0.103	0.009147359
ENSGALG00000033083	ATOH8	-0.103	0.009174603
ENSGALG00000034007	ITGA6	-0.103	0.009182639
ENSGALG00000041808	ANO1	-0.103	0.009189238
ENSGALG00000006630	COMMD7	0.103	0.009196989

ENSGALG00000004948	GLA	-0.103	0.009203582
ENSGALG00000037067	EIF3E	0.103	0.009215608
ENSGALG00000004055	C7orf50	-0.103	0.009215784
ENSGALG00000041163	ABHD17A	-0.103	0.009233154
ENSGALG00000010835	DTX4	-0.103	0.009253928
ENSGALG00000012119	MARCO	-0.103	0.009265795
ENSGALG00000042148	FKBP5	-0.103	0.009267679
ENSGALG00000012889	ZCCHC2	-0.103	0.009278656
ENSGALG00000005668	WSB1	0.103	0.0092833
ENSGALG00000044204	ISG20	-0.103	0.009304411
ENSGALG00000034107	TRIM63	0.103	0.009322916
ENSGALG00000009170	NCEH1	-0.103	0.009327343
ENSGALG00000006937	P4HTM	0.103	0.009356904
ENSGALG00000043606		0.103	0.009367177
ENSGALG00000003929	SYS1	-0.103	0.009425902
ENSGALG00000038957	NAE1	0.103	0.009441979
ENSGALG00000035803	THRSP	0.103	0.009451495
ENSGALG00000005318	OVCH2	0.103	0.009466505
ENSGALG00000043493	IGFBP1	-0.103	0.009469277
ENSGALG00000006864		-0.103	0.00947118
ENSGALG00000009637	EMC7	-0.103	0.00951406
ENSGALG00000048401		-0.103	0.009517854
ENSGALG00000050750	NRN1L	0.102	0.009541208
ENSGALG00000032763	MECR	0.102	0.009545892
ENSGALG00000031328	PAK4	-0.102	0.00959383
ENSGALG00000026385	LYPLAL1	0.102	0.009648307
ENSGALG00000046360		-0.102	0.009650276
ENSGALG00000014974	MIB1	-0.102	0.009656267
ENSGALG00000043576	AGAP3	-0.102	0.009675641
ENSGALG00000039208	PHF2	-0.102	0.009681109
ENSGALG00000051392		0.102	0.009727222
ENSGALG00000031593	TMSB15B	-0.102	0.009739445
ENSGALG00000003014	CHRNA3	0.102	0.00974057
ENSGALG00000036244		0.102	0.009775867
ENSGALG00000011185	PLEKHA8	-0.102	0.009783333
ENSGALG00000042297	ANKRD13D	0.102	0.009829152
ENSGALG00000006048		0.102	0.009833237

ENSGALG00000006331	STAMBPL1	-0.102	0.009847651
ENSGALG00000042476	TEX9	0.102	0.009874073
ENSGALG00000010561	GPATCH11	-0.102	0.009888531
ENSGALG00000043009	WNT1	0.102	0.00989786
ENSGALG00000035825		-0.102	0.009909999
ENSGALG00000010901		-0.102	0.009967606
ENSGALG00000047665		-0.102	0.009988578
ENSGALG00000010026	PPP1CB	-0.102	0.009997497
ENSGALG00000015163	RNF138	-0.102	0.010054431
ENSGALG00000008348	PDIA3	-0.102	0.010063098
ENSGALG00000041833		0.102	0.01006864
ENSGALG00000035047		0.102	0.01011425
ENSGALG00000010674	DCTD	-0.102	0.010129036
ENSGALG00000042374	PDE11A	-0.102	0.010199071
ENSGALG00000014907	DCBLD1	-0.102	0.010215008
ENSGALG00000014115	SLAIN2	-0.102	0.01023651
ENSGALG00000031301	TULP1	-0.102	0.010245761
ENSGALG00000006982	EEF2K	-0.101	0.010248017
ENSGALG00000006857	SPATA20	-0.101	0.010253516
ENSGALG00000052767		0.101	0.010296694
ENSGALG00000032372	CYB5R2	0.101	0.010331749
ENSGALG00000016860	ERCC5	0.101	0.010335251
ENSGALG00000014831	TRMT11	-0.101	0.010343981
ENSGALG00000007766	KIAA1217	-0.101	0.010355057
ENSGALG00000016630	EXTL3	-0.101	0.010370032
ENSGALG00000002656	CRK	-0.101	0.010385038
ENSGALG00000035207	TDO2	-0.101	0.010398375
ENSGALG00000030407	IL6R	-0.101	0.01040626
ENSGALG00000020999		-0.101	0.010409377
ENSGALG00000009450	DUSP10	-0.101	0.010422171
ENSGALG00000044018	ISOC2	-0.101	0.010426019
ENSGALG00000045908		0.101	0.010447676
ENSGALG00000013102	COBL	-0.101	0.01045495
ENSGALG00000020741	HPS6	-0.101	0.010460942
ENSGALG00000050150	STX5	-0.101	0.010485072
ENSGALG00000035116		0.101	0.010487525
ENSGALG00000001838	XPO7	0.101	0.010511647

ENSGALG00000001596	MLLT1	-0.101	0.010523197
ENSGALG00000028084	FAAH2	0.101	0.010539391
ENSGALG00000010915	IL6	0.101	0.010553501
ENSGALG00000016911	OBI1	0.101	0.010582169
ENSGALG00000007731	DPCD	0.101	0.010647715
ENSGALG00000011414	AMDHD1	-0.101	0.01066349
ENSGALG00000054306	SRXN1	0.101	0.010667511
ENSGALG00000015480	POGLUT1	-0.101	0.010675442
ENSGALG00000039880	RHEB	0.101	0.010708502
ENSGALG00000009328	EIF3A	-0.101	0.010740963
ENSGALG00000006320		0.101	0.010787238
ENSGALG00000052296	MEX3D	-0.101	0.010816017
ENSGALG00000021443	PANX3	0.101	0.010910282
ENSGALG00000030254	METAP1D	0.101	0.010938049
ENSGALG00000016409	KIDINS220	-0.101	0.010975565
ENSGALG00000002900	NOTUM	-0.101	0.010983697
ENSGALG00000052371		-0.101	0.010987281
ENSGALG00000032181	LRP4	-0.101	0.011000102
ENSGALG00000012034	ADSL	-0.100	0.011036582
ENSGALG00000003299	NSD3	-0.100	0.011061774
ENSGALG00000009312	RPL22L1	0.100	0.011089894
ENSGALG00000012462	KIF5C	-0.100	0.011120818
ENSGALG00000039354	VTG1	-0.100	0.011126661
ENSGALG00000031597	HBAD	0.100	0.0111592
ENSGALG00000009704	CHST15	-0.100	0.011162108
ENSGALG00000000692	CDK18	-0.100	0.011162538
ENSGALG00000034229	ZNF703	-0.100	0.01118111
ENSGALG00000047327		-0.100	0.011213986
ENSGALG00000011319	ADGRL3	-0.100	0.011241448
ENSGALG00000008954	RUNDC3B	-0.100	0.011254424
ENSGALG00000009320	PSEN1	-0.100	0.011290079
ENSGALG00000006751	THY1	0.100	0.011299087
ENSGALG00000010491	PWWP2B	-0.100	0.011300704
ENSGALG00000040035	ADRB2	-0.100	0.011302124
ENSGALG00000010836	AHR	-0.100	0.011355947
ENSGALG00000008417	CFAP58	0.100	0.01143782
ENSGALG00000005181		-0.100	0.011440842

ENSGALG00000012171	GPR39	-0.100	0.011457435
ENSGALG00000011184	HNRNPD	0.100	0.011468147
ENSGALG00000018803	TMEM198	-0.100	0.011486802
ENSGALG00000009107	NRXN1	-0.100	0.011521502
ENSGALG00000028308	TNS4	-0.100	0.011524242
ENSGALG00000043198		-0.100	0.011549648
ENSGALG00000012144	MIEF1	-0.100	0.011562992
ENSGALG00000052072		-0.100	0.011572396
ENSGALG00000012259		-0.100	0.011589961
ENSGALG00000037807	NOP2	-0.100	0.011621386
ENSGALG00000028031	C1QL1	0.100	0.011621757
ENSGALG00000034132	CACFD1	-0.100	0.011632572
ENSGALG00000002182	NR5A2	-0.100	0.011678601
ENSGALG00000034675	XKR9	0.100	0.011702245
ENSGALG00000001983		0.100	0.011708993
ENSGALG00000036915	SQLE	-0.100	0.011734264
ENSGALG00000002055	HES4	0.100	0.011769214
ENSGALG00000015297		-0.100	0.011769464
ENSGALG00000011808	CCR9	-0.100	0.011770807
ENSGALG00000038219	NAA40	0.100	0.011812954
ENSGALG00000031262		0.100	0.011837059
ENSGALG00000006363	CABIN1	-0.099	0.01185879
ENSGALG00000044299	CFAP92	0.099	0.011908342
ENSGALG00000011465	STT3B	-0.099	0.01192144
ENSGALG00000009309	EIF5A2	-0.099	0.011934612
ENSGALG00000053184		-0.099	0.011934992
ENSGALG00000037935	RARA	-0.099	0.011967958
ENSGALG00000016880	UBAC2	-0.099	0.012028759
ENSGALG00000039420	TNPO3	-0.099	0.01203492
ENSGALG00000001521	VPS26B	-0.099	0.012076076
ENSGALG00000006465		-0.099	0.01207934
ENSGALG00000006080	GPC4	-0.099	0.012091005
ENSGALG00000007253	IL1RAP	-0.099	0.012109427
ENSGALG00000010202	ENTPD5	-0.099	0.012110989
ENSGALG00000010313		-0.099	0.012122923
ENSGALG00000011251	TBC1D5	-0.099	0.012176596
ENSGALG00000005476	PTH2R	-0.099	0.01218078

ENSGALG00000044026		0.099	0.012218328
ENSGALG00000005933	SLC5A11	0.099	0.012220194
ENSGALG00000038527	CARMIL3	0.099	0.012224262
ENSGALG00000029582	EVI5	0.099	0.012246213
ENSGALG00000044187		-0.099	0.012267768
ENSGALG00000014501	FGFBP2	0.099	0.012273628
ENSGALG00000007218	MB21D2	-0.099	0.012279392
ENSGALG00000000632	CHTF8	0.099	0.01227977
ENSGALG00000002919	MON1A	-0.099	0.012287109
ENSGALG00000008432	AP2M1	-0.099	0.01234163
ENSGALG00000016902	GPC5	-0.099	0.012349165
ENSGALG00000002959	NRBF2	-0.099	0.012368699
ENSGALG00000027608	PIGC	-0.099	0.012380219
ENSGALG00000009016	SLX4IP	-0.099	0.012421125
ENSGALG00000012834	AKR1D1	-0.099	0.012426819
ENSGALG00000027665	SYNGR1	-0.099	0.012447315
ENSGALG00000004814		-0.099	0.012451476
ENSGALG00000009266	FGA	-0.099	0.012471282
ENSGALG00000002519	SLC25A33	-0.099	0.012495795
ENSGALG00000036731	GAS2L1	-0.099	0.012540373
ENSGALG00000012287	ATP5S	-0.099	0.012570489
ENSGALG00000036286		-0.099	0.012588316
ENSGALG00000011697	SEC22A	-0.099	0.012626698
ENSGALG00000034973	FZD7	-0.099	0.012646114
ENSGALG00000001101	MBD3	-0.099	0.012647213
ENSGALG00000006702	MFGE8	-0.099	0.012661504
ENSGALG00000047011		-0.099	0.012693377
ENSGALG00000044199	CYP2AC7	-0.099	0.012713942
ENSGALG00000040834	LGR4	-0.099	0.012720549
ENSGALG00000010375	SLC30A2	-0.098	0.01285935
ENSGALG00000043920	OTUD3	0.098	0.012879438
ENSGALG00000003427	USP3	-0.098	0.012893564
ENSGALG00000053187		0.098	0.012929379
ENSGALG00000005408	BCO1	0.098	0.012941473
ENSGALG00000007341	MED19	-0.098	0.012953418
ENSGALG00000004900	RFLNB	-0.098	0.013003153
ENSGALG00000015802	N6AMT1	-0.098	0.013006812

ENSGALG00000015234		-0.098	0.013041754
ENSGALG00000001028		-0.098	0.013053714
ENSGALG00000007835	PUS10	0.098	0.013061414
ENSGALG00000030487		-0.098	0.013061725
ENSGALG00000017316	UCP3	0.098	0.013135155
ENSGALG00000004806	EDEM3	-0.098	0.013171354
ENSGALG00000008274	CRBN	-0.098	0.013192639
ENSGALG00000022980	CDKN1B	-0.098	0.013247701
ENSGALG00000000708	NUCKS1	-0.098	0.013250379
ENSGALG00000011331	CTH	-0.098	0.013271937
ENSGALG00000039687	NIPAL4	-0.098	0.013282993
ENSGALG00000045125	GP5	0.098	0.013313696
ENSGALG00000030351	ILF3	-0.098	0.013315727
ENSGALG00000008281	NEURL1	-0.098	0.01334825
ENSGALG00000055058		-0.098	0.013349868
ENSGALG00000042863	SMIM4	-0.098	0.013358294
ENSGALG00000009002	CPED1	-0.098	0.013375318
ENSGALG00000026638	RELL2	0.098	0.013383478
ENSGALG00000012655	POLR3B	0.098	0.013388918
ENSGALG00000003819		-0.098	0.013448804
ENSGALG00000016870	TMTC4	-0.098	0.013460058
ENSGALG00000028017	EFCAB2	0.098	0.013467758
ENSGALG00000003029	PLPP6	-0.098	0.013481912
ENSGALG00000016034	ATRN	-0.098	0.013502064
ENSGALG00000012894	CHRM2	-0.098	0.013511411
ENSGALG00000024486	C4orf48	-0.098	0.013526005
ENSGALG00000012748	ELOVL2	-0.098	0.01352683
ENSGALG00000041663	KLB	-0.098	0.013530898
ENSGALG00000023451	TRPM7	-0.098	0.013549348
ENSGALG00000033183	GABRQ	0.098	0.013553217
ENSGALG00000030054	PTPRN2	-0.098	0.01355532
ENSGALG00000036678	ETV4	-0.098	0.013572833
ENSGALG00000033179	TGFBR1	-0.098	0.013573111
ENSGALG00000041202	FBXO32	-0.098	0.013596163
ENSGALG00000010052	PPP2R3C	0.098	0.013603128
ENSGALG00000043352		-0.098	0.0136037
ENSGALG00000041193	NFYB	0.098	0.013609089

ENSGALG00000006461	UBE3C	-0.098	0.013613048
ENSGALG00000028866	COASY	-0.098	0.013617412
ENSGALG00000051135	BCL2L11	-0.098	0.013618529
ENSGALG00000044813	THUMPD3	-0.098	0.013620984
ENSGALG00000004332	PLA2G12B	-0.098	0.013625551
ENSGALG00000053516		0.098	0.013638277
ENSGALG00000049660		-0.098	0.013649198
ENSGALG00000029585	FUNDC2	-0.098	0.013656046
ENSGALG00000028967	DNAJC27	-0.098	0.013657485
ENSGALG00000041213	ARHGAP17	0.098	0.013672883
ENSGALG00000008731	DZANK1	0.097	0.013696642
ENSGALG00000001697		-0.097	0.013730059
ENSGALG00000049505		-0.097	0.013772255
ENSGALG00000004821	PTPN11	-0.097	0.013791771
ENSGALG00000013878	PCYOX1	-0.097	0.01379674
ENSGALG00000016480	SDC1	-0.097	0.01381847
ENSGALG00000008332	F2	-0.097	0.013825407
ENSGALG00000016265	LANCL3	-0.097	0.013897713
ENSGALG00000031872	AGMAT	-0.097	0.013906301
ENSGALG00000012956	TMTC1	-0.097	0.013911664
ENSGALG00000037957		-0.097	0.013912491
ENSGALG00000055117		0.097	0.013940683
ENSGALG00000048534		-0.097	0.013950205
ENSGALG00000004937	VGLL4	-0.097	0.013976083
ENSGALG00000016681	ZBED1	-0.097	0.013994202
ENSGALG00000034964	SYTL1	0.097	0.013994757
ENSGALG00000001849	BSN	-0.097	0.01400045
ENSGALG00000036683	PNPO	0.097	0.014003701
ENSGALG00000009485	IMMP2L	-0.097	0.014012042
ENSGALG00000041296		-0.097	0.014017163
ENSGALG00000042213	AP1B1	-0.097	0.014053914
ENSGALG00000040877	UBALD2	-0.097	0.01407897
ENSGALG00000009297	TELO2	-0.097	0.014119948
ENSGALG00000015889	PHIP	-0.097	0.01412092
ENSGALG00000011213	DEGS2	-0.097	0.014122438
ENSGALG00000010117	SLC25A21	0.097	0.014124002
ENSGALG00000005021	RAB43	-0.097	0.014154842

ENSGALG00000042080	PGPEP1	-0.097	0.014160663
ENSGALG00000004725		0.097	0.014195004
ENSGALG00000007720	AIFM3	0.097	0.014281715
ENSGALG00000001472	JAM3	-0.097	0.014286665
ENSGALG00000005350		-0.097	0.014301669
ENSGALG00000006286	USP31	-0.097	0.014327645
ENSGALG00000007492	YME1L1	-0.097	0.014330731
ENSGALG00000037246	RAMP3	-0.097	0.014348059
ENSGALG00000045251		-0.097	0.014373381
ENSGALG00000040006	TBL1X	-0.097	0.014380561
ENSGALG00000031368	PHF12	-0.097	0.01444683
ENSGALG00000001459	TNNC1	0.097	0.014456675
ENSGALG00000035599	CD24	-0.097	0.014461153
ENSGALG00000005957		-0.097	0.014510067
ENSGALG00000007308	RAB35	-0.097	0.01452428
ENSGALG00000006872	PISD	-0.097	0.014525832
ENSGALG00000009443	TMEM168	-0.097	0.014560157
ENSGALG00000011534	ZFYVE21	-0.097	0.014564409
ENSGALG00000050719		-0.097	0.014591646
ENSGALG00000010039	BRE	-0.097	0.014611303
ENSGALG00000016425	IAH1	0.097	0.014618765
ENSGALG00000043676		-0.097	0.014672809
ENSGALG00000003355	NOP16	-0.096	0.014828446
ENSGALG00000044874		0.096	0.014848765
ENSGALG00000012018	UGT8	-0.096	0.014909242
ENSGALG00000009268	FGG	-0.096	0.014928107
ENSGALG00000016491	APOB	-0.096	0.014981765
ENSGALG00000007971	SUMO2	-0.096	0.015015809
ENSGALG00000014468	MLF2	-0.096	0.015050206
ENSGALG00000006783	PLOD2	-0.096	0.015085991
ENSGALG00000028517	EIF1	-0.096	0.015087286
ENSGALG00000040109	LARP1	-0.096	0.015113139
ENSGALG00000053132		-0.096	0.015150487
ENSGALG00000028438	CLK1	0.096	0.015169591
ENSGALG00000008341	FURIN	-0.096	0.015178333
ENSGALG00000008675	SLC25A5	-0.096	0.015184788
ENSGALG00000008872	FAM199X	-0.096	0.01520972

ENSGALG00000005065	PLA2G4A	-0.096	0.015235036
ENSGALG00000005775		0.096	0.015256946
ENSGALG00000006956	ARIH2	-0.096	0.015280958
ENSGALG00000022720	GJB2	-0.096	0.015301372
ENSGALG00000007820	BTRC	-0.096	0.015311377
ENSGALG00000016456	LPIN1	-0.096	0.015312602
ENSGALG00000006357	XPC	-0.096	0.015325124
ENSGALG00000001117	PFKFB2	-0.096	0.015340205
ENSGALG00000047622		0.096	0.015343479
ENSGALG00000031493	BAK1	-0.096	0.015364954
ENSGALG00000050427	OSBPL10	-0.096	0.015369517
ENSGALG00000011320	TMCC3	-0.096	0.015384064
ENSGALG00000039346	ARHGAP39	0.096	0.015401275
ENSGALG00000002147	SIKE1	-0.096	0.015415345
ENSGALG00000003144	TRIM25	-0.096	0.01544682
ENSGALG00000005583	ALG14	-0.096	0.015449248
ENSGALG00000008676	RRBP1	-0.096	0.015485708
ENSGALG00000009287	ATF2	-0.096	0.015539077
ENSGALG00000033614	ATF1	-0.096	0.015571954
ENSGALG00000005683	ARHGAP29	-0.096	0.015574603
ENSGALG00000049001	ANKRD39	0.096	0.015610041
ENSGALG00000026817		0.096	0.015626203
ENSGALG00000006976		-0.096	0.015658075
ENSGALG00000023506		0.096	0.015676164
ENSGALG00000015623	HGFAC	-0.096	0.015678372
ENSGALG00000037854	TKFC	0.096	0.015684638
ENSGALG00000008771	SHOC2	-0.096	0.015697846
ENSGALG00000009305	LBR	-0.096	0.015699726
ENSGALG00000006350	DDTL	-0.096	0.015703262
ENSGALG00000040886		-0.096	0.015721021
ENSGALG00000007793	UPRT	-0.096	0.015725962
ENSGALG00000029653		-0.096	0.015728828
ENSGALG00000046984	M1AP	0.096	0.015734635
ENSGALG00000006543	PFKL	0.095	0.015754814
ENSGALG00000043971		-0.095	0.015773149
ENSGALG00000039883	PIGV	-0.095	0.015779233
ENSGALG00000021399		-0.095	0.015785212

ENSGALG00000008096	SUFU	-0.095	0.015808182
ENSGALG00000026316	TMEM136	-0.095	0.015824453
ENSGALG00000016717	GTPBP6	0.095	0.015856791
ENSGALG00000048775	TXLNA	-0.095	0.015863963
ENSGALG00000001779	KIAA0895L	0.095	0.015882333
ENSGALG00000033090	VDR	0.095	0.015914258
ENSGALG00000041834	AEBP1	0.095	0.015975925
ENSGALG00000003445	MGST3	0.095	0.016015039
ENSGALG00000029387	PURA	-0.095	0.016022284
ENSGALG00000037283	CUTC	0.095	0.016067202
ENSGALG00000002750	NCKAP1	-0.095	0.016087363
ENSGALG00000025948		-0.095	0.016096211
ENSGALG00000004098	STK4	-0.095	0.016119183
ENSGALG00000022557	CLDN3	-0.095	0.016140195
ENSGALG00000015001	LAMA4	0.095	0.016193896
ENSGALG00000003220	UPF1	-0.095	0.016211825
ENSGALG00000045747	REEP6	0.095	0.016225791
ENSGALG00000031296		-0.095	0.016239338
ENSGALG00000020788	CCDC141	0.095	0.016302862
ENSGALG00000032743	C1orf112	0.095	0.016305244
ENSGALG00000039140	CD14	-0.095	0.016336423
ENSGALG00000031294	MPLKIP	-0.095	0.01635638
ENSGALG00000007493	NSDHL	-0.095	0.016366719
ENSGALG00000037219	RNF2	-0.095	0.016387449
ENSGALG00000028683	SSR3	-0.095	0.016396448
ENSGALG00000054297		-0.095	0.016410074
ENSGALG00000015806	RARS2	0.095	0.016414797
ENSGALG00000007404	YIPF5	-0.095	0.016420023
ENSGALG00000010575	INTS12	-0.095	0.016440696
ENSGALG00000032400	UTP23	0.095	0.01644294
ENSGALG00000017153	RDX	-0.095	0.016466727
ENSGALG00000002969	MAU2	-0.095	0.01647239
ENSGALG00000050002	COLEC11	-0.095	0.016503687
ENSGALG00000003155	SRR	0.095	0.016505357
ENSGALG00000041564		-0.095	0.016521911
ENSGALG00000030899	ATXN7L3	-0.095	0.016524538
ENSGALG00000013037	BCL2L13	-0.095	0.016535304

ENSGALG00000000518	KLHL12	-0.095	0.016675641
ENSGALG00000011770	UMPS	-0.095	0.016696136
ENSGALG00000033469	KDM1A	-0.095	0.016724425
ENSGALG00000034024	RAPGEF5	-0.095	0.016781037
ENSGALG00000040956	CHIC1	-0.095	0.016840272
ENSGALG00000011296		-0.095	0.016851152
ENSGALG00000035057	SLC35B1	-0.094	0.016893673
ENSGALG00000016148	C2CD2	-0.094	0.016899802
ENSGALG00000000975	C1orf174	-0.094	0.016903755
ENSGALG00000043787	DEPDC6	-0.094	0.016904397
ENSGALG00000012888	PHLPP1	-0.094	0.016968888
ENSGALG00000012870	AGK	-0.094	0.016979542
ENSGALG00000003805		-0.094	0.01701356
ENSGALG00000011894	CYP2D6	-0.094	0.017055223
ENSGALG00000034971	SLC45A4	-0.094	0.017079671
ENSGALG00000011856	FMC1	0.094	0.01711955
ENSGALG00000005767	TRAF7	-0.094	0.017120734
ENSGALG00000040504	GGT5	-0.094	0.017122205
ENSGALG00000012277	MAFF	-0.094	0.017133668
ENSGALG00000002377	ENO1	-0.094	0.017192127
ENSGALG00000047480		-0.094	0.017255509
ENSGALG00000032145	B4GALT7	-0.094	0.017266955
ENSGALG00000012392	IYD	-0.094	0.017284269
ENSGALG00000016574	GEMIN8	-0.094	0.01728669
ENSGALG00000047617	DDA1	-0.094	0.017294037
ENSGALG00000051203		-0.094	0.017341111
ENSGALG00000039409	RELA	-0.094	0.017358959
ENSGALG00000039171	RAD52	0.094	0.017376082
ENSGALG00000006020	BTBD1	-0.094	0.01740109
ENSGALG00000006833	ITIH2	-0.094	0.017404698
ENSGALG00000016144	TMPRSS2	-0.094	0.017408255
ENSGALG00000010668	WWC2	-0.094	0.017511469
ENSGALG00000009021	ST6GALNAC5	-0.094	0.01752067
ENSGALG00000006632	CEP63	0.094	0.017546548
ENSGALG00000015879	HUNK	-0.094	0.017547714
ENSGALG00000001848	UBE2J2	-0.094	0.017562823
ENSGALG00000016510	KRTCAP3	-0.094	0.017567288

ENSGALG00000003845	MKNK2	-0.094	0.017569119
ENSGALG00000002699	VAV2	-0.094	0.017569916
ENSGALG00000005340	SLC35A3	-0.094	0.017589392
ENSGALG00000033926	CBX1	-0.094	0.017634984
ENSGALG00000020164	GAR1	-0.094	0.017673924
ENSGALG00000006186	RAD21L1	0.094	0.017729124
ENSGALG00000008378	SRGAP3	-0.094	0.017750553
ENSGALG00000042521	MPP3	0.094	0.017789685
ENSGALG00000039525		-0.094	0.017801845
ENSGALG00000012166	SLC35F5	-0.094	0.017810626
ENSGALG00000019552	SERPINB10	0.094	0.017838452
ENSGALG00000033570		0.094	0.017849067
ENSGALG00000004300	PTDSS2	-0.094	0.017853397
ENSGALG00000020260	PGRMC1	-0.094	0.01787062
ENSGALG00000001207	PLCH2	-0.094	0.017872364
ENSGALG00000022804		-0.094	0.017873768
ENSGALG00000002836	PEX14	-0.094	0.017876914
ENSGALG00000032430	IPO5	-0.094	0.017884346
ENSGALG00000001962	PTBP1	-0.094	0.017884977
ENSGALG00000017207	CWC15	-0.094	0.017921096
ENSGALG00000010079	HP1BP3	-0.094	0.01792811
ENSGALG00000013528		-0.094	0.017960895
ENSGALG00000011452	ARPC2	-0.094	0.017972224
ENSGALG00000001758	QSOX2	-0.093	0.018090124
ENSGALG00000001777	MFSD11	-0.093	0.018091717
ENSGALG00000034693	MLLT6	-0.093	0.018160924
ENSGALG00000036854	R3HCC1	-0.093	0.018172485
ENSGALG00000026322	ARMT1	-0.093	0.018208701
ENSGALG00000008187	EIF3J	-0.093	0.018266051
ENSGALG00000013369	NCLN	-0.093	0.018275817
ENSGALG00000009948	HHIP	-0.093	0.018278323
ENSGALG00000044278		-0.093	0.018291469
ENSGALG00000049378		-0.093	0.018312387
ENSGALG00000040687	SNX27	-0.093	0.018335014
ENSGALG00000001130	GALNT17	-0.093	0.018348503
ENSGALG00000017376		-0.093	0.018420953
ENSGALG00000007579	APBB1IP	-0.093	0.018433765

ENSGALG00000029788	CCK	-0.093	0.018444242
ENSGALG00000017627	C10orf2	-0.093	0.018448302
ENSGALG00000006198	LSS	-0.093	0.01853641
ENSGALG00000008359	CD248	-0.093	0.018565842
ENSGALG00000028302	UQCRQ	0.093	0.018646857
ENSGALG00000001790	METTL23	-0.093	0.018655759
ENSGALG00000014922	ROCK1	-0.093	0.018680337
ENSGALG00000032458	RABL2A	0.093	0.018685222
ENSGALG00000007291		-0.093	0.018690108
ENSGALG00000017156	DDX10	-0.093	0.018743731
ENSGALG00000016433	YWHAQ	-0.093	0.018780446
ENSGALG00000032713	FOXA1	-0.093	0.018791141
ENSGALG00000010125	CSGALNACT1	0.093	0.018874999
ENSGALG00000003370	KIAA1191	-0.093	0.018897851
ENSGALG00000030550	ATP2B1	-0.093	0.018958646
ENSGALG00000006613	CTSD	-0.093	0.018967799
ENSGALG00000031534	ARID5B	-0.093	0.01897305
ENSGALG00000005830	MNT	-0.093	0.018982083
ENSGALG00000014977	GSK3B	-0.093	0.019000459
ENSGALG00000024047	MYCL	0.093	0.019034182
ENSGALG00000006317	ING5	-0.093	0.019053702
ENSGALG00000040756	PI4KA	-0.093	0.019116995
ENSGALG00000005507	NONO	-0.093	0.019123561
ENSGALG00000006567	UBASH3B	-0.093	0.019124895
ENSGALG00000007381		-0.093	0.019127384
ENSGALG00000043754	GLUL	-0.093	0.019129742
ENSGALG00000041766	JOSD1	-0.093	0.019175425
ENSGALG00000041797		-0.093	0.019220827
ENSGALG00000006076	RASGEF1C	-0.093	0.019240553
ENSGALG00000039705	GFPT2	-0.093	0.019240963
ENSGALG00000050760	ADCY6	-0.093	0.019244114
ENSGALG00000041413	PPP3CC	-0.093	0.019295014
ENSGALG00000008045	SLC9A8	0.093	0.019307179
ENSGALG00000037642	SLC23A1	0.093	0.019347432
ENSGALG00000030812	CSNK1G1	-0.092	0.019364979
ENSGALG00000048496		-0.092	0.019403043
ENSGALG00000001905	DTX2	-0.092	0.019421217

ENSGALG00000031958	NABP2	0.092	0.019520805
ENSGALG00000050803	GIPC1	-0.092	0.019556553
ENSGALG00000004045	AGAP1	-0.092	0.019557217
ENSGALG00000014860	YES1	-0.092	0.019567541
ENSGALG00000030096		0.092	0.019572227
ENSGALG00000047908	ZFAND3	-0.092	0.019605078
ENSGALG00000032002	RPL34	0.092	0.019642139
ENSGALG00000006378	LIPA	-0.092	0.019731383
ENSGALG00000025904	WDR82	-0.092	0.019778902
ENSGALG00000016412	MBOAT2	-0.092	0.019856207
ENSGALG00000010406	TMEM63C	-0.092	0.019871835
ENSGALG00000035108	KDM7A	-0.092	0.019918379
ENSGALG00000001805		0.092	0.019924286
ENSGALG00000036456	TRIB1	-0.092	0.019943674
ENSGALG00000007215	KIF5B	-0.092	0.019947068
ENSGALG00000009868	ZNF330	0.092	0.019954992
ENSGALG00000014096	ARNTL2	0.092	0.01999605
ENSGALG00000036539	EFNB1	-0.092	0.02000005
ENSGALG00000026302	SRPRB	-0.092	0.020053623
ENSGALG00000040672	IGFBP4	-0.092	0.02005442
ENSGALG00000002878	CREBRF	-0.092	0.020060682
ENSGALG00000003798	GET4	-0.092	0.020069964
ENSGALG00000003336	DAPK2	-0.092	0.020080284
ENSGALG00000006167	LRRC8B	-0.092	0.020092434
ENSGALG00000010734	PPP4R3A	-0.092	0.020092627
ENSGALG00000009056	LCLAT1	-0.092	0.020102981
ENSGALG00000004476	SEMA3G	0.092	0.020125273
ENSGALG00000001017	WRAP73	0.092	0.020132618
ENSGALG00000006916	GDE1	-0.092	0.020177524
ENSGALG00000033246	ZNF740	-0.092	0.020208107
ENSGALG00000041687	SREBF2	-0.092	0.020246333
ENSGALG00000036502	CDIP1	-0.092	0.020260169
ENSGALG00000051274	B3GALT2	-0.092	0.020276978
ENSGALG00000005667	UBE2G2	-0.092	0.020285686
ENSGALG00000047187		-0.092	0.020353883
ENSGALG00000002232	RERE	-0.092	0.020377674
ENSGALG00000027534	GM2A	-0.092	0.020383702

ENSGALG00000007619	UBALD1	-0.092	0.020401693
ENSGALG00000000596	CDC27	-0.092	0.020430526
ENSGALG00000051711		0.092	0.020432544
ENSGALG00000003238	GNA11	-0.092	0.020502005
ENSGALG00000013625		-0.092	0.020506307
ENSGALG00000016266	PRRG1	-0.092	0.020510434
ENSGALG00000038541	VPS37C	-0.092	0.020522356
ENSGALG00000005456	SDR42E1	-0.092	0.020541248
ENSGALG00000005547	PIK3AP1	-0.092	0.020564163
ENSGALG00000038256		-0.092	0.020578911
ENSGALG00000041454	DNMT3A	0.092	0.020681432
ENSGALG00000038396		-0.092	0.020684916
ENSGALG00000042415	BBS2	-0.092	0.020699786
ENSGALG00000042741	GRK2	0.092	0.020701187
ENSGALG00000002024	COMT	-0.091	0.020733328
ENSGALG00000003628	TLN2	-0.091	0.020736253
ENSGALG00000012394		-0.091	0.020834662
ENSGALG00000043234	HBA1	0.091	0.020845666
ENSGALG00000039920	SNRK	-0.091	0.020866746
ENSGALG00000023806	PITPNA	-0.091	0.020877486
ENSGALG00000000619	ANGPTL4	0.091	0.020925048
ENSGALG00000030871	BCL2L10	-0.091	0.021004049
ENSGALG00000001433	B3GNTL1	0.091	0.021013996
ENSGALG00000004875	PEMT	-0.091	0.021066107
ENSGALG00000007489	CNNM1	-0.091	0.021098859
ENSGALG00000010227	KRR1	0.091	0.021104014
ENSGALG00000008016	GRB2	-0.091	0.021118253
ENSGALG00000001617	CAMKK1	-0.091	0.021147811
ENSGALG00000032595	NDUFB2	0.091	0.02121447
ENSGALG00000002863	ERGIC1	-0.091	0.021247896
ENSGALG00000025712	DEF8	-0.091	0.02125085
ENSGALG00000006241		-0.091	0.021286014
ENSGALG00000033497	FOXP4	-0.091	0.021296651
ENSGALG00000023070	SPIA1	-0.091	0.02130078
ENSGALG00000033695	CYP26A1	-0.091	0.021320869
ENSGALG00000041787	PLA2G15	-0.091	0.021364782
ENSGALG00000013438	TEX10	-0.091	0.021397895

ENSGALG00000051209		0.091	0.021401992
ENSGALG00000016281	DMD	-0.091	0.021415819
ENSGALG00000042485		-0.091	0.021423178
ENSGALG00000043405	BNIP3L	-0.091	0.021433334
ENSGALG00000007357	BCL6	0.091	0.021532741
ENSGALG00000008081	PIK3CG	-0.091	0.021556505
ENSGALG00000004192	SLTM	-0.091	0.021591444
ENSGALG00000012148	ATG14	-0.091	0.021617346
ENSGALG00000020249	RAPGEF2	-0.091	0.021618753
ENSGALG00000028230	SUN2	-0.091	0.021663528
ENSGALG00000005674	USP10	-0.091	0.021665212
ENSGALG00000020742	PPRC1	-0.091	0.021672567
ENSGALG00000036901	TSPAN18	-0.091	0.021675989
ENSGALG00000001332	RBL1	-0.091	0.021708787
ENSGALG00000047359	MAF1	-0.091	0.021718235
ENSGALG00000010234	MMACHC	-0.091	0.021722684
ENSGALG00000011229	SERBP1	-0.091	0.021727632
ENSGALG00000031546		-0.091	0.021753847
ENSGALG00000007590	MTMR8	-0.091	0.021776807
ENSGALG00000037968	API5	-0.091	0.021887196
ENSGALG00000008457	RIN2	-0.091	0.021897108
ENSGALG00000052268	SSBP4	-0.091	0.021901413
ENSGALG00000031518		0.091	0.021906369
ENSGALG00000031754	KCNG2	-0.091	0.021906637
ENSGALG00000039786	FGFR1	0.091	0.021938556
ENSGALG00000013057	USP18	-0.091	0.021947557
ENSGALG00000032632	SLC49A4	-0.091	0.021992048
ENSGALG00000016522	PPEF1	0.091	0.021994538
ENSGALG00000041030	PCBP2	-0.091	0.022043414
ENSGALG00000035282	IGF2	-0.091	0.022080551
ENSGALG00000010175	HSP90AB1	-0.091	0.02212309
ENSGALG00000007234	CLCN5	-0.091	0.022126243
ENSGALG00000054387		-0.091	0.022131405
ENSGALG00000001153	AUTS2	-0.091	0.022132371
ENSGALG00000014213	PRR5	-0.090	0.022145821
ENSGALG00000022718	FUT4	-0.090	0.022152734
ENSGALG00000006575	VIPR2	-0.090	0.022164606

ENSGALG00000033857	NAT8B	0.090	0.022251809
ENSGALG00000002550	TSHB	0.090	0.022313171
ENSGALG00000002170		-0.090	0.022351981
ENSGALG00000051335	INO80C	-0.090	0.022372481
ENSGALG00000008092	NXT2	-0.090	0.022481927
ENSGALG00000007821	DNAJA3	-0.090	0.022526075
ENSGALG00000007569	KBTBD8	-0.090	0.022531975
ENSGALG00000004503		0.090	0.022547906
ENSGALG00000008750	DPH7	-0.090	0.022579614
ENSGALG00000007409	SCN4B	-0.090	0.022606166
ENSGALG00000012404	ERO1A	-0.090	0.022638459
ENSGALG00000003838		0.090	0.022645635
ENSGALG00000029494	BGLAP	0.090	0.022709127
ENSGALG00000012966	ZBTB2	-0.090	0.022742072
ENSGALG00000011207	DUSP6	-0.090	0.022755001
ENSGALG00000045362		0.090	0.022755602
ENSGALG00000016124	HADH	-0.090	0.022811125
ENSGALG00000017186	BIRC3	-0.090	0.022865248
ENSGALG00000016059	ETS2	-0.090	0.022876839
ENSGALG00000047947		-0.090	0.022879263
ENSGALG00000031131	VIRMA	-0.090	0.022884791
ENSGALG00000016906	SPRY2	-0.090	0.022905353
ENSGALG00000005249	BICD2	-0.090	0.022915456
ENSGALG00000035919	EPN2	-0.090	0.022919967
ENSGALG00000042794	ZFAND1	0.090	0.022973596
ENSGALG00000003082	DPH1	-0.090	0.022975751
ENSGALG00000009859		-0.090	0.022985317
ENSGALG00000010702	ADSS	-0.090	0.023003431
ENSGALG00000011402	CNOT9	-0.090	0.023008637
ENSGALG00000008950	EHD4	-0.090	0.023015051
ENSGALG00000016261	CYBB	-0.090	0.023056047
ENSGALG00000039967	RP11-529K1.3	-0.090	0.023062454
ENSGALG00000030070	QDPR	-0.090	0.023167479
ENSGALG00000016897	GPR180	-0.090	0.023173475
ENSGALG00000004943	SS18L2	-0.090	0.023183586
ENSGALG00000026944	ARF6	-0.090	0.023232745
ENSGALG00000012321	MAP4K5	-0.090	0.023246054

ENSGALG00000039337	SH3BP5	-0.090	0.02328127
ENSGALG00000041471	TUBB	-0.090	0.023304865
ENSGALG00000017363	C11H16ORF87	-0.090	0.023320019
ENSGALG00000009192	SLITRK2	-0.090	0.023322165
ENSGALG00000016481	PUM2	-0.090	0.023342291
ENSGALG00000007346	JCAD	-0.090	0.02335515
ENSGALG00000011335	NHEJ1	0.090	0.023365688
ENSGALG00000027696		-0.090	0.023446175
ENSGALG00000009692	SH3RF1	-0.090	0.023600493
ENSGALG00000029343	TGM2	0.090	0.023616345
ENSGALG00000001536		-0.090	0.023627005
ENSGALG00000006033	GORASP1	-0.090	0.023628154
ENSGALG00000041373	ARAP2	-0.090	0.023629938
ENSGALG00000032832	DNAJC16	-0.090	0.023637038
ENSGALG00000025950	PRKCA	-0.090	0.023655694
ENSGALG00000038059	NR1H3	-0.089	0.023671066
ENSGALG00000003976	BCORL1	-0.089	0.023699496
ENSGALG00000001279	ZNF652	-0.089	0.023701329
ENSGALG00000008537	EPHB3	-0.089	0.023732983
ENSGALG00000000955	TMEM222	-0.089	0.023737499
ENSGALG00000032087	MC5R	-0.089	0.023796605
ENSGALG00000016863	TEX30	0.089	0.023872074
ENSGALG00000039011	DPYS	-0.089	0.023893091
ENSGALG00000007012	ARRDC4	-0.089	0.023914269
ENSGALG00000028423	HS2ST1	-0.089	0.023916742
ENSGALG00000011361	CRYZ	0.089	0.024066537
ENSGALG00000041514		0.089	0.024079767
ENSGALG00000051677		-0.089	0.024121317
ENSGALG00000017136	GJB6	-0.089	0.024132619
ENSGALG00000006627	UPB1	-0.089	0.024147445
ENSGALG00000040447	CDCP1	-0.089	0.02421067
ENSGALG00000019738	FBXO47	-0.089	0.024211626
ENSGALG00000031158	OAT	-0.089	0.02423657
ENSGALG00000038797	MANBAL4	-0.089	0.024240553
ENSGALG00000010165	TCTE1	0.089	0.024243189
ENSGALG00000039634	KRT80	-0.089	0.024243374
ENSGALG00000006871	SLCO3A1	-0.089	0.02424385

ENSGALG00000004893	TMEM119	-0.089	0.024261842
ENSGALG00000010884	DDX24	-0.089	0.024280827
ENSGALG00000042645	PARD3B	-0.089	0.024327376
ENSGALG00000000513	ZNF276	-0.089	0.024365249
ENSGALG00000011121	SLC35D1	-0.089	0.024436228
ENSGALG00000011347	IHH	-0.089	0.02443892
ENSGALG00000034502	SCD5	0.089	0.024446613
ENSGALG00000012059	OSBPL11	-0.089	0.024459503
ENSGALG00000030025	FABP4	0.089	0.02449602
ENSGALG00000011609		-0.089	0.024510278
ENSGALG00000052506	SLC25A44	-0.089	0.024533607
ENSGALG00000052355	PDE12	-0.089	0.024564587
ENSGALG00000040342	ADAMTS1	-0.089	0.024596145
ENSGALG00000005609	OGFR	-0.089	0.024638491
ENSGALG00000042890	PIN1	-0.089	0.024648339
ENSGALG00000010795	CRPPA	-0.089	0.024724707
ENSGALG00000034550	POLR2M	-0.089	0.024809209
ENSGALG00000037521	PRCP	-0.089	0.024824166
ENSGALG00000040549	HTR7	-0.089	0.024840856
ENSGALG00000020647	LDLRAD1	0.089	0.024849174
ENSGALG00000008414	DVL3	-0.089	0.024895113
ENSGALG00000003260	UTP6	0.089	0.024920992
ENSGALG00000009445	INPP5F	-0.089	0.024924718
ENSGALG00000049794		-0.089	0.024929387
ENSGALG00000009338	ZFYVE1	-0.089	0.024997751
ENSGALG00000041684	FBXO30	-0.089	0.025055208
ENSGALG00000037778	NAB1	-0.089	0.025141524
ENSGALG00000036076	UNC50	-0.089	0.025165157
ENSGALG00000002982	SLC2A6	-0.089	0.025179041
ENSGALG00000039590		0.089	0.025180935
ENSGALG00000048716	FBXL3	-0.089	0.025204567
ENSGALG00000004040	MFAP3	-0.089	0.025248807
ENSGALG00000016348	SAT1	-0.089	0.025269289
ENSGALG00000020747	MBD4	-0.089	0.025273462
ENSGALG00000041972	POP4	0.088	0.025297298
ENSGALG00000011294	THRB	-0.088	0.02534394
ENSGALG00000013785	GNAL	-0.088	0.025350029

ENSGALG00000024378	ZNF414	0.088	0.025381764
ENSGALG00000046494	CASP10	-0.088	0.025389566
ENSGALG00000037138	KCNQ1	-0.088	0.025462467
ENSGALG00000010000	PRKCE	-0.088	0.025506969
ENSGALG00000014236	ATP8A1	-0.088	0.025522406
ENSGALG00000052242		-0.088	0.025540715
ENSGALG00000032419	STARD8	-0.088	0.025653963
ENSGALG00000009510	PIGH	-0.088	0.025664556
ENSGALG00000040879	NUDCD1	-0.088	0.025666663
ENSGALG00000002883	SCAPER	-0.088	0.02567584
ENSGALG00000008700	PDCD4	-0.088	0.025695487
ENSGALG00000037316	OCLN	-0.088	0.025751192
ENSGALG00000008779	ACTR2	-0.088	0.025758531
ENSGALG00000009272		-0.088	0.025767456
ENSGALG00000005522	DYNLL2	-0.088	0.025792044
ENSGALG00000005612	CCM2	-0.088	0.025815248
ENSGALG00000050258		-0.088	0.025833617
ENSGALG00000020160	ADH6	0.088	0.025846356
ENSGALG00000054173		-0.088	0.025903894
ENSGALG00000016055	KCNJ15	-0.088	0.025947252
ENSGALG00000001602	NUTF2	0.088	0.02598452
ENSGALG00000001577	FOXK2	-0.088	0.025989141
ENSGALG00000053701		-0.088	0.026062891
ENSGALG00000027902	SYNGR2	-0.088	0.026095777
ENSGALG00000002882	BNIP1	0.088	0.026107417
ENSGALG00000009669	RSRC1	-0.088	0.026121611
ENSGALG00000035610	PPM1D	-0.088	0.026154002
ENSGALG00000000377	HNRNPM	-0.088	0.026163073
ENSGALG00000036836	SOSTDC1	-0.088	0.026194383
ENSGALG00000008127	SPI1	-0.088	0.026196979
ENSGALG00000014979	FRK	-0.088	0.02621607
ENSGALG00000008939	FUBP1	-0.088	0.026240414
ENSGALG00000029424	JPH3	-0.088	0.026302889
ENSGALG00000039573		-0.088	0.0263057
ENSGALG00000010539	RNF11	-0.088	0.026347817
ENSGALG00000024082	SMCR8	-0.088	0.026357602
ENSGALG00000033666	NHP2	0.088	0.026377019

ENSGALG00000009229	ATP6V0C	-0.088	0.026385528
ENSGALG00000006312	TSG101	-0.088	0.0264483
ENSGALG00000049517	CHMP2A	-0.088	0.026501814
ENSGALG00000020419	LIN52	-0.088	0.02655017
ENSGALG00000040380	RAD51C	-0.088	0.026577864
ENSGALG00000012685	KDM1B	-0.088	0.026596039
ENSGALG00000047125		-0.088	0.026596243
ENSGALG00000005412	SNAP47	-0.088	0.02660736
ENSGALG00000008791	NMT2	-0.088	0.026619899
ENSGALG00000009250	HNRNPA3	-0.088	0.026631868
ENSGALG00000005054	DKC1	-0.088	0.026662024
ENSGALG00000004569	UNC5B	-0.088	0.026672417
ENSGALG00000012186	CASP6	-0.088	0.026694127
ENSGALG00000001883	ST6GALNAC2	0.088	0.026728107
ENSGALG00000010001	EGLN3	-0.088	0.026750156
ENSGALG00000007473	MRPL28	-0.088	0.026761363
ENSGALG00000005997	WDR44	-0.088	0.026762259
ENSGALG00000052844		-0.088	0.026775011
ENSGALG00000009744	FAM98B	-0.088	0.02681627
ENSGALG00000028551		-0.088	0.026844244
ENSGALG00000008094	HSPD1	-0.088	0.026846655
ENSGALG00000008321	RNPEPL1	-0.088	0.026898517
ENSGALG00000010163	LGR5	-0.088	0.026938854
ENSGALG00000002085	FZR1	-0.088	0.026941993
ENSGALG00000048830	EIF4EBP3	0.088	0.026953621
ENSGALG00000021572	SSH2	-0.087	0.027026108
ENSGALG00000031551	ZDHHC9	-0.087	0.027033118
ENSGALG00000004477		-0.087	0.027148149
ENSGALG00000032418	RAB26	-0.087	0.027160824
ENSGALG00000033038	STARD3NL	0.087	0.027210531
ENSGALG00000012992	WNK1	-0.087	0.027222253
ENSGALG00000008023	TMEM94	-0.087	0.027270437
ENSGALG00000003969	EMC1	-0.087	0.027342662
ENSGALG00000010270	AREL1	-0.087	0.027364802
ENSGALG00000002437	DIPK1B	-0.087	0.0273694
ENSGALG00000052583		-0.087	0.027433368
ENSGALG00000045611		-0.087	0.027449326

ENSGALG00000031763	EDARADD	-0.087	0.027474935
ENSGALG00000044480	IL13RA1	-0.087	0.027478168
ENSGALG00000017047	SUPT20H	0.087	0.027490089
ENSGALG00000003925	MYPN	0.087	0.027494269
ENSGALG00000042400	CASK	-0.087	0.027497188
ENSGALG00000009722	DYNC1I1	0.087	0.027527798
ENSGALG00000017644	COTL1	-0.087	0.027611692
ENSGALG00000027484	C20orf85	0.087	0.027619683
ENSGALG00000016667	AvBD10	-0.087	0.027635656
ENSGALG00000016617	NCOA1	-0.087	0.027643897
ENSGALG00000005781	ARFGAP1	-0.087	0.027687764
ENSGALG00000028720		0.087	0.027697157
ENSGALG00000050420		-0.087	0.027727461
ENSGALG00000007396	TAOK3	-0.087	0.027795982
ENSGALG00000041752	CD164L2	-0.087	0.027808075
ENSGALG00000004193	ARSG	-0.087	0.027821498
ENSGALG00000004465	PALD1	0.087	0.027856446
ENSGALG00000040521	HOXA2	0.087	0.027860004
ENSGALG00000036300	SEC61A2	-0.087	0.027971179
ENSGALG00000005616	ARSI	-0.087	0.027972958
ENSGALG00000009236	AGPS	-0.087	0.028023332
ENSGALG00000014582	CADM1	-0.087	0.02804268
ENSGALG00000003205	EIF4EBP1	-0.087	0.028045439
ENSGALG00000041275	SLC25A38	-0.087	0.028074118
ENSGALG00000008551	CRELD2	-0.087	0.028089452
ENSGALG00000038574		0.087	0.028091013
ENSGALG00000039658	GGA1	-0.087	0.028102194
ENSGALG00000012929	DNM1L	-0.087	0.028106659
ENSGALG00000030613	PDGFRB	-0.087	0.028118808
ENSGALG00000011799		0.087	0.028155144
ENSGALG00000011221	GPAT3	0.087	0.028229168
ENSGALG00000030542	SEMA6D	-0.087	0.028247935
ENSGALG00000009441	TAF1A	0.087	0.028271362
ENSGALG00000001110		-0.087	0.028277416
ENSGALG00000011098	TAF5L	-0.087	0.028281064
ENSGALG00000012444	ACVR2A	-0.087	0.028310487
ENSGALG00000003959	AP1M1	-0.087	0.02832358

ENSGALG00000005809	SEPTIN2	-0.087	0.028377624
ENSGALG00000010103	FBXW7	0.087	0.028385297
ENSGALG00000006403	MESDC2	-0.087	0.028456975
ENSGALG00000035626	DAD1	-0.087	0.028479859
ENSGALG00000032231	C4	-0.087	0.028584096
ENSGALG00000009031	SESTD1	-0.087	0.028632433
ENSGALG00000029135	YEATS4	0.087	0.028636608
ENSGALG00000027262	CAB39	-0.087	0.028699569
ENSGALG00000005349	MSLN	0.087	0.028709136
ENSGALG00000006597	DZIP1L	-0.087	0.028716226
ENSGALG00000052162	PPM1L	-0.087	0.028739215
ENSGALG00000006379	SHH	0.087	0.028757749
ENSGALG00000008645	NKRF	-0.087	0.028764418
ENSGALG00000008907	PLCB4	0.086	0.028841284
ENSGALG00000051101		-0.086	0.028846394
ENSGALG00000000870	DCAKD	-0.086	0.028872162
ENSGALG00000005902	RAB7A	-0.086	0.028877421
ENSGALG00000005150	PTRH2	-0.086	0.028881539
ENSGALG00000014222	NUP50	-0.086	0.028885979
ENSGALG00000023542	ALPL	-0.086	0.028908798
ENSGALG00000006325		-0.086	0.028977769
ENSGALG00000040371		-0.086	0.0289972
ENSGALG00000038721	MAP1LC3A	-0.086	0.029006154
ENSGALG00000044151		-0.086	0.02901715
ENSGALG00000011902	TAGLN	0.086	0.029029829
ENSGALG00000010192	FBXO33	-0.086	0.029050423
ENSGALG00000030548	OSBP	-0.086	0.029051782
ENSGALG00000010136	HGSNAT	-0.086	0.029100142
ENSGALG00000005226	MYOZ1	0.086	0.029102371
ENSGALG00000009173	GFRA1	-0.086	0.029107166
ENSGALG00000000903	TERF2IP	-0.086	0.029115755
ENSGALG00000013569	SEC61B	-0.086	0.029176222
ENSGALG00000054900		0.086	0.029181823
ENSGALG00000030859	GNAS	0.086	0.02923445
ENSGALG00000032307	DDX23	-0.086	0.02925382
ENSGALG00000003203		0.086	0.029289789
ENSGALG00000002301	CNOT1	-0.086	0.029338101

ENSGALG00000048097		0.086	0.029344606
ENSGALG00000004689	PKN1	0.086	0.029361207
ENSGALG00000002563	TPM4	-0.086	0.029376215
ENSGALG00000004771	C1QB	-0.086	0.029390691
ENSGALG00000048849		-0.086	0.029390788
ENSGALG00000052702		0.086	0.029399795
ENSGALG00000013993	VNN1	-0.086	0.029532472
ENSGALG00000015308	SEC63	-0.086	0.029540918
ENSGALG00000003645		-0.086	0.029541134
ENSGALG00000007928	MLLT10	-0.086	0.029594382
ENSGALG00000034111	FOXF1	-0.086	0.029621149
ENSGALG00000014585		-0.086	0.029642661
ENSGALG00000002892		0.086	0.029650122
ENSGALG00000016053	DYRK1A	-0.086	0.029663117
ENSGALG00000008905	HABP2	-0.086	0.029666361
ENSGALG00000042900	GATAD2A	-0.086	0.029682948
ENSGALG00000001971	UBE2O	-0.086	0.029692064
ENSGALG00000003111	BFAR	-0.086	0.029711641
ENSGALG00000001168	POLR2C	-0.086	0.029721649
ENSGALG00000048020		0.086	0.029768584
ENSGALG00000011088	GSKIP	-0.086	0.029771558
ENSGALG00000006194	SLC52A3	-0.086	0.029772591
ENSGALG00000037423	PTPRK	-0.086	0.029784686
ENSGALG00000009426	AIDA	-0.086	0.029806241
ENSGALG00000010157	TRAPPC6A	0.086	0.029832681
ENSGALG00000040902	SACM1L	-0.086	0.029839042
ENSGALG00000007227	MSI1	-0.086	0.029892549
ENSGALG00000008222	SLC26A5	-0.086	0.029905946
ENSGALG00000024332	CDC34	-0.086	0.029951014
ENSGALG00000014286	PDS5A	-0.086	0.029963243
ENSGALG00000010708	ICA1	-0.086	0.029980345
ENSGALG00000053667		-0.086	0.030003312
ENSGALG00000033174	ZBTB45	-0.086	0.030008498
ENSGALG00000030918	RBM3	0.086	0.030026652
ENSGALG00000038978		-0.086	0.030062168
ENSGALG00000013683	ARID1B	-0.086	0.030137045
ENSGALG00000015916	SLC17A5	-0.086	0.030204868

ENSGALG00000039206	ARID5A	-0.086	0.030218683
ENSGALG00000003162		-0.086	0.030239391
ENSGALG00000023199	HNRNPDL	-0.086	0.030240569
ENSGALG00000006297	TRPC7	0.086	0.030255368
ENSGALG00000047988		-0.086	0.030265862
ENSGALG00000023364	CDC42EP4	-0.086	0.030318437
ENSGALG00000001019	BTBD2	-0.086	0.030333442
ENSGALG00000007030		0.086	0.030348156
ENSGALG00000016737	NIPA2	-0.086	0.03038047
ENSGALG00000041203	CRMP1	-0.086	0.030382708
ENSGALG00000017204	SESN3	-0.086	0.030385279
ENSGALG00000014258		-0.086	0.030408899
ENSGALG00000039141	CERS2	-0.086	0.030420949
ENSGALG00000003562	NPTX2	-0.086	0.030476204
ENSGALG00000016196		-0.086	0.030512543
ENSGALG00000006123		0.086	0.03051519
ENSGALG00000004070	MICALL2	0.086	0.030560481
ENSGALG00000028512	LCOR	-0.086	0.030577216
ENSGALG00000008163	PSME4	-0.086	0.030579284
ENSGALG00000016602	ARHGAP6	-0.086	0.030652829
ENSGALG00000037366	GRID1	-0.086	0.03065461
ENSGALG00000038154	YAP1	-0.086	0.030656659
ENSGALG00000047963	MECP2	-0.086	0.030668076
ENSGALG00000003208	GABARAPL2	-0.085	0.030734827
ENSGALG00000004341		-0.085	0.030767713
ENSGALG00000009505	PNPLA8	-0.085	0.030776148
ENSGALG00000012111	TMEM37	-0.085	0.030808027
ENSGALG00000005103	GDPD1	-0.085	0.030809491
ENSGALG00000027494	SPTSSB	-0.085	0.030831315
ENSGALG00000002870	LEMD2	-0.085	0.030845171
ENSGALG00000030580	RPS6KA5	-0.085	0.030854174
ENSGALG00000049811		-0.085	0.030861336
ENSGALG00000047610		-0.085	0.030861478
ENSGALG00000034948	UAP1L1	-0.085	0.030870278
ENSGALG00000032930	NCL	-0.085	0.030896856
ENSGALG00000007476	VAMP7	-0.085	0.030919338
ENSGALG00000051870		-0.085	0.030924218

ENSGALG00000037597	YOD1	-0.085	0.030930005
ENSGALG00000004379	BCL7A	-0.085	0.030959154
ENSGALG00000001389		-0.085	0.030965156
ENSGALG00000049673		-0.085	0.030977845
ENSGALG00000013814	IMPA2	-0.085	0.030992957
ENSGALG00000040869		-0.085	0.030998972
ENSGALG00000028496		0.085	0.031000998
ENSGALG00000036043	TBX3	-0.085	0.031058258
ENSGALG00000016805	SULT1C3	-0.085	0.031072117
ENSGALG00000001907	WAPL	-0.085	0.031074685
ENSGALG00000042612		-0.085	0.031084227
ENSGALG00000014408	LLGL1	-0.085	0.031086543
ENSGALG00000005802	FLT4	-0.085	0.031125419
ENSGALG00000005598	ZNF598	-0.085	0.031195232
ENSGALG00000007300	PYCR1	-0.085	0.031206168
ENSGALG00000039724	RHOF	-0.085	0.031257434
ENSGALG00000002943	PRPF8	-0.085	0.031276144
ENSGALG00000014248	TMEM33	-0.085	0.031289403
ENSGALG00000005856	TBC1D9B	-0.085	0.031339108
ENSGALG00000006910	IMPDH2	-0.085	0.031348298
ENSGALG00000035287	STAM	-0.085	0.031353007
ENSGALG00000005420	AATF	-0.085	0.031359025
ENSGALG00000029450	UBE2Q1	-0.085	0.031375948
ENSGALG00000004908		-0.085	0.031417952
ENSGALG00000033303		0.085	0.031450257
ENSGALG00000046995	NECTIN4	0.085	0.031462747
ENSGALG00000002832	IFI35	0.085	0.031497644
ENSGALG00000026421	PSMB2	-0.085	0.03149769
ENSGALG00000009402	LRRC34	-0.085	0.031506365
ENSGALG00000009303	ENAH	-0.085	0.031507498
ENSGALG00000031440	PTPRU	-0.085	0.03151941
ENSGALG00000017201	CEP57	-0.085	0.031553613
ENSGALG00000031786	BLVRA	-0.085	0.03159684
ENSGALG00000016406	RPS6KA3	-0.085	0.031625953
ENSGALG00000008918	LAMP5	0.085	0.031667901
ENSGALG00000030808		-0.085	0.031688479
ENSGALG00000023256	RNF8	-0.085	0.031696496

ENSGALG00000037744	CACNA1S	0.085	0.031700567
ENSGALG00000009275	TMEM63A	-0.085	0.031713676
ENSGALG00000045163	APH1A	-0.085	0.031715953
ENSGALG00000051040	TMEM251	-0.085	0.031720225
ENSGALG00000039532	PHC2	-0.085	0.031722552
ENSGALG00000015529	WFS1	-0.085	0.031776286
ENSGALG00000004918	RAB9B	-0.085	0.031818112
ENSGALG00000003818	CYFIP2	-0.085	0.031845348
ENSGALG00000054059		-0.085	0.031904142
ENSGALG00000009262	FGB	-0.085	0.03194407
ENSGALG00000030673		-0.085	0.03196569
ENSGALG00000041604	NPTXR	-0.085	0.0319953
ENSGALG00000036219	STK35	-0.085	0.032020044
ENSGALG00000011190	PLAC8	0.085	0.032076155
ENSGALG00000030530	HAND2	-0.085	0.032090805
ENSGALG00000002655	ITGAV	-0.085	0.032124908
ENSGALG00000001527	VWA1	-0.085	0.032139635
ENSGALG00000026163	TNFSF11	-0.085	0.032148566
ENSGALG00000016224	MAOA	0.085	0.032152028
ENSGALG00000006648	SPECC1L	-0.085	0.032161324
ENSGALG00000011801	ESR2	-0.085	0.032176608
ENSGALG00000030065	TENM3	-0.085	0.032181615
ENSGALG00000004927	COQ4	-0.085	0.032250484
ENSGALG00000011168	C12orf29	-0.085	0.032254711
ENSGALG00000005657	CRHR2	0.085	0.032282007
ENSGALG00000016377	PXDN	-0.085	0.032286135
ENSGALG00000050577	GRPEL2	-0.085	0.032315047
ENSGALG00000011174	PSMB1	-0.085	0.03233046
ENSGALG00000045642	TNFRSF11A	-0.085	0.032388177
ENSGALG00000011281	SGO1	0.085	0.032418363
ENSGALG00000002831	SH3PXD2B	-0.085	0.032476086
ENSGALG00000050943	RNF19B	0.085	0.032570986
ENSGALG00000003539	KIAA1522	-0.085	0.0325778
ENSGALG00000042051	SETD2	-0.085	0.032667889
ENSGALG00000001283	SRRM1	-0.085	0.032676894
ENSGALG00000026733	FFAR4	-0.085	0.032681043
ENSGALG00000008818	IDH1	-0.085	0.032685193

ENSGALG00000016917	CLN5	-0.084	0.032718975
ENSGALG00000001031		-0.084	0.032768371
ENSGALG00000027698	COX19	-0.084	0.032788981
ENSGALG00000026507	FDX1	-0.084	0.032797
ENSGALG00000042052	PPIL2	-0.084	0.032875378
ENSGALG00000004108		-0.084	0.032877558
ENSGALG00000003658	DHX35	-0.084	0.032908699
ENSGALG00000010290	VEGFA	-0.084	0.032957574
ENSGALG00000037787	MEF2D	-0.084	0.032973429
ENSGALG00000015285	TOMM70	-0.084	0.033007177
ENSGALG00000016537	SCML2	-0.084	0.033047422
ENSGALG00000016719	TNFRSF21	-0.084	0.033053106
ENSGALG00000016920	LMO7	-0.084	0.033079778
ENSGALG00000008231	PACSIN3	-0.084	0.033174765
ENSGALG00000051534		-0.084	0.033215001
ENSGALG00000005138	KCTD10	-0.084	0.033239719
ENSGALG00000040161	C7orf25	-0.084	0.033255157
ENSGALG00000001637	PSMD5	-0.084	0.033320475
ENSGALG00000024295		0.084	0.033322152
ENSGALG00000005820	RNF130	-0.084	0.033356103
ENSGALG00000011931	SNU13	-0.084	0.033383768
ENSGALG00000023740	HBZ	-0.084	0.033393128
ENSGALG00000032599	ZNF384	-0.084	0.033394075
ENSGALG00000054505		0.084	0.033397787
ENSGALG00000010051	MOCS1	-0.084	0.033408129
ENSGALG00000014834	NCOA7	-0.084	0.0334472
ENSGALG00000002913	BRI3BP	-0.084	0.03347677
ENSGALG00000047857		-0.084	0.033500528
ENSGALG00000047517		-0.084	0.033510642
ENSGALG00000033631	MYC	-0.084	0.033523388
ENSGALG00000052348		0.084	0.033604636
ENSGALG00000053680	HIST1H46L2	0.084	0.033608831
ENSGALG00000025764	FKBP10	-0.084	0.033643449
ENSGALG00000028681		-0.084	0.033648543
ENSGALG00000042446	ACSM5	-0.084	0.033671166
ENSGALG00000009800	SETD7	-0.084	0.033713704
ENSGALG00000038129	PTGES3L	0.084	0.033796507

ENSGALG00000026995	RHOG	-0.084	0.03379701
ENSGALG00000030147	AFF4	-0.084	0.03387807
ENSGALG00000010320	PAWR	-0.084	0.033916979
ENSGALG00000003358	UBIAD1	-0.084	0.033923587
ENSGALG00000048599		0.084	0.033937456
ENSGALG00000008160	CNNM2	-0.084	0.033944851
ENSGALG00000016102	CYP26B1	-0.084	0.033950504
ENSGALG00000040369	PFKFB3	-0.084	0.033968726
ENSGALG00000012489	KLHL28	-0.084	0.034052293
ENSGALG00000026957	SEMA4G	-0.084	0.034097827
ENSGALG00000002240	C3orf18	-0.084	0.034169816
ENSGALG00000019570	C1orf145	-0.084	0.034193182
ENSGALG00000015791	AKIRIN2	-0.084	0.03422606
ENSGALG00000004620	LAMC1	-0.084	0.034281842
ENSGALG00000029523	RAD21	-0.084	0.034290172
ENSGALG00000012874	DENND11	-0.084	0.034292984
ENSGALG00000048883		-0.084	0.034308665
ENSGALG00000040987	NDUFA8	-0.084	0.034367748
ENSGALG00000016844	RAB20	-0.084	0.034376254
ENSGALG00000035013	WDR11	-0.084	0.034378652
ENSGALG00000030554	KCNN3	-0.084	0.034394709
ENSGALG00000008811	SLC1A4	-0.084	0.034417393
ENSGALG00000001826	WWC1	-0.084	0.034422526
ENSGALG00000041037	YIPF3	-0.084	0.034485242
ENSGALG00000006237	PKN2	-0.084	0.034563308
ENSGALG00000039615	MSN	-0.084	0.034568874
ENSGALG00000016507	FNDC4	0.084	0.034628356
ENSGALG00000008860	LONRF3	-0.084	0.034636842
ENSGALG00000042357	ARHGAP1	-0.084	0.034705141
ENSGALG00000051895		-0.084	0.034727622
ENSGALG00000045350		-0.084	0.034747769
ENSGALG00000002524	UCHL5	-0.084	0.034784279
ENSGALG00000004930	HRH1	-0.083	0.034849424
ENSGALG00000042151		0.083	0.034904547
ENSGALG00000002973	BECN1	-0.083	0.0349363
ENSGALG00000034427	SCN4A	-0.083	0.03506243
ENSGALG00000008326		-0.083	0.035101842

ENSGALG00000005504	HNF1B	-0.083	0.035106432
ENSGALG00000044002		0.083	0.035130443
ENSGALG00000006420	ABHD17C	-0.083	0.035247094
ENSGALG00000029897	RPL30	-0.083	0.035268203
ENSGALG00000041112	TRAM1L1	-0.083	0.035301211
ENSGALG00000044224		0.083	0.03532516
ENSGALG00000016451	ROCK2	-0.083	0.035398717
ENSGALG00000008603	ITPKA	-0.083	0.035484191
ENSGALG00000030626	FZD6	-0.083	0.035493053
ENSGALG00000045122	AKR1B1	-0.083	0.035546963
ENSGALG00000042948	RNF126	-0.083	0.035561163
ENSGALG00000029646	CNNM4	-0.083	0.035616333
ENSGALG00000009944	KAT7	-0.083	0.03562473
ENSGALG00000001313	EIF4H	-0.083	0.035685339
ENSGALG00000007162	SELENOS	-0.083	0.03574773
ENSGALG00000038913	LDLRAD3	-0.083	0.035761286
ENSGALG00000016785	IL1RL1	-0.083	0.035794759
ENSGALG00000045776	CPN2	-0.083	0.03580573
ENSGALG00000048700	DDX28	-0.083	0.03581995
ENSGALG00000002408	NCDN	-0.083	0.035820292
ENSGALG00000005134	ZSWIM8	-0.083	0.035826531
ENSGALG00000008054		-0.083	0.035851701
ENSGALG00000010565	NRDC	-0.083	0.035867475
ENSGALG00000033888	IDH3B	0.083	0.035883499
ENSGALG00000003736	ABCB9	-0.083	0.035912576
ENSGALG00000010200	TRHDE	-0.083	0.035919364
ENSGALG00000042762	ZBTB26	-0.083	0.035962111
ENSGALG00000043447	LAS1L	-0.083	0.035965025
ENSGALG00000015070	FIG4	0.083	0.036089059
ENSGALG00000035406	DSN1	-0.083	0.036100212
ENSGALG00000006097	PLXNA1	-0.083	0.036136659
ENSGALG00000048064		-0.083	0.036178924
ENSGALG00000040275	RCSD1	-0.083	0.036187784
ENSGALG00000035845	JPH1	-0.083	0.036209249
ENSGALG00000038187	SLC35B4	-0.083	0.036249142
ENSGALG00000052425	BATF3	-0.083	0.036266536
ENSGALG00000028046	INSIG2	-0.083	0.036415026

ENSGALG00000054488		0.083	0.036506071
ENSGALG00000002661	YWHAE	-0.083	0.036530549
ENSGALG00000053025		-0.083	0.03653269
ENSGALG00000008545	SENP5	-0.083	0.036541233
ENSGALG00000001288		0.083	0.036541755
ENSGALG00000007246	ELMO2	0.083	0.036577312
ENSGALG00000033811	PTPRN	0.083	0.036610959
ENSGALG00000003936	PPT1	-0.083	0.036626079
ENSGALG00000008837	PELI3	-0.083	0.036635114
ENSGALG00000028032	MSRA	-0.083	0.036687184
ENSGALG00000040755	ANGPT4	-0.083	0.036690604
ENSGALG00000010183	RAB21	-0.083	0.036706902
ENSGALG00000016792	MFSD9	-0.083	0.036711246
ENSGALG00000000146	SUPT6H	-0.083	0.036714702
ENSGALG00000000919	PIGR	-0.083	0.036774892
ENSGALG00000022030	PGAP3	-0.083	0.0368401
ENSGALG00000040155	ZW10	-0.083	0.036865792
ENSGALG00000038550		-0.083	0.03689791
ENSGALG00000032618	DUSP5	-0.083	0.036907341
ENSGALG00000005684	FBXL15	-0.083	0.03693332
ENSGALG00000014122	CYB5R1	-0.083	0.036950224
ENSGALG00000016205	PDXK	-0.083	0.037002767
ENSGALG00000013356	IKBKE	0.083	0.03701107
ENSGALG00000016473	OSR1	0.083	0.03701273
ENSGALG00000007115	ASB7	-0.083	0.037022376
ENSGALG00000042976	UBTF	-0.082	0.03716628
ENSGALG00000011456	CDK17	-0.082	0.037171275
ENSGALG00000003201	FBXL8	0.082	0.037181528
ENSGALG00000016324	GSTA4	0.082	0.037267988
ENSGALG00000037262	HOXC8	-0.082	0.037281153
ENSGALG00000013941	PDE7B	-0.082	0.037296786
ENSGALG00000001976	TRIM33	-0.082	0.037314284
ENSGALG00000022874	RAB31	-0.082	0.037321784
ENSGALG00000034573	ABCF2	-0.082	0.037336336
ENSGALG00000003272	KHDRBS1	-0.082	0.03738265
ENSGALG00000015701	CXADR	-0.082	0.037485221
ENSGALG00000014899	NUS1	-0.082	0.037497608

ENSGALG00000028749	ACTG1	-0.082	0.037497665
ENSGALG00000026862	CLDN1	-0.082	0.03750711
ENSGALG00000008970	NRK	-0.082	0.037512398
ENSGALG00000003670	MAFB	-0.082	0.037520313
ENSGALG00000005120	FBLN2	-0.082	0.037578539
ENSGALG00000022875		0.082	0.037649423
ENSGALG00000010579	STON2	-0.082	0.037655597
ENSGALG00000030068	RAB3A	-0.082	0.037792195
ENSGALG00000005909	GATA2	-0.082	0.037823823
ENSGALG00000050619	SOCS2	-0.082	0.037833084
ENSGALG00000015519	ROBO2	-0.082	0.037853355
ENSGALG00000015762	LYRM2	0.082	0.037922951
ENSGALG00000010501	SNW1	-0.082	0.03798788
ENSGALG00000004365		-0.082	0.037999572
ENSGALG00000012076	TIFA	-0.082	0.038015559
ENSGALG00000011643	UTP20	-0.082	0.038074634
ENSGALG00000013034	CECR2	-0.082	0.038189216
ENSGALG00000010885	BMP2K	-0.082	0.038207139
ENSGALG00000006045	SH3GL3	-0.082	0.038214324
ENSGALG00000031410	ENPEP	-0.082	0.038215661
ENSGALG00000038589	ABHD14B	0.082	0.038253462
ENSGALG00000048205		-0.082	0.038270013
ENSGALG00000034258	C25H1ORF43	-0.082	0.038312751
ENSGALG00000015635	HTT	-0.082	0.038325326
ENSGALG00000001837	ORAI3	-0.082	0.038337408
ENSGALG00000002925	ETFA	0.082	0.038343553
ENSGALG00000001223	PEX10	-0.082	0.038358139
ENSGALG00000003322	TBC1D2B	-0.082	0.038421548
ENSGALG00000005241	DYNC1LI2	-0.082	0.038453401
ENSGALG00000027068	HDGFRP2	-0.082	0.038466605
ENSGALG00000003434	C17orf75	-0.082	0.03847771
ENSGALG00000002710	IPMK	-0.082	0.038483134
ENSGALG00000029166	UBTD1	0.082	0.038514242
ENSGALG00000033575	EIF2B4	0.082	0.038570028
ENSGALG00000001428	EFHD1	-0.082	0.038584422
ENSGALG00000004542	ZMYND8	-0.082	0.038611316
ENSGALG00000021301		-0.082	0.038614491

ENSGALG00000032234	MARCH5	-0.082	0.038624599
ENSGALG00000043193	TAF2	-0.082	0.038628996
ENSGALG00000011704	HACD2	-0.082	0.038733002
ENSGALG00000030396	HMGA1	-0.082	0.038744914
ENSGALG00000006568	TM9SF4	-0.082	0.038768783
ENSGALG00000013742	EZR	-0.082	0.038775583
ENSGALG00000007133		-0.082	0.038776313
ENSGALG00000016362	SH3YL1	0.082	0.038813952
ENSGALG00000049976	AMIGO1	-0.082	0.038851979
ENSGALG00000044388		0.082	0.038867394
ENSGALG00000006723		-0.082	0.038924619
ENSGALG00000005046	SHMT1	-0.082	0.038977851
ENSGALG00000030280	PABPC1	-0.082	0.03898767
ENSGALG00000042851	PPARGC1A	-0.082	0.039061598
ENSGALG00000013149	MOCOS	-0.082	0.039063004
ENSGALG00000001618	OGFOD3	-0.082	0.039116856
ENSGALG00000011621	IGF2R	-0.082	0.039123289
ENSGALG00000035973	YIPF6	-0.082	0.039173683
ENSGALG00000011930		-0.082	0.039197014
ENSGALG00000019228	SFT2D2	-0.082	0.039234378
ENSGALG00000043711	AGO3	-0.082	0.039256779
ENSGALG00000010857	DAB1	-0.082	0.039292608
ENSGALG00000004705	BUD31	-0.082	0.03932963
ENSGALG00000052647		-0.082	0.039336619
ENSGALG00000001968	SYT6	-0.082	0.039339935
ENSGALG00000012574	WDSUB1	0.082	0.039354161
ENSGALG00000029944	FAM222A	-0.082	0.039365469
ENSGALG00000008886	ZNF618	-0.082	0.039372823
ENSGALG00000007141	LRRK1	-0.082	0.039432053
ENSGALG00000005515	CRYBB3	0.081	0.03948135
ENSGALG00000011394	CYP2J21	-0.081	0.039583625
ENSGALG00000005708	HNF4beta	-0.081	0.039650561
ENSGALG00000004523	MFN2	-0.081	0.03967191
ENSGALG00000046687	EPS8L3	0.081	0.039684516
ENSGALG00000048773		-0.081	0.03970123
ENSGALG00000010448	LTBP1	-0.081	0.039701832
ENSGALG00000039643	TMEM79	-0.081	0.039717346

ENSGALG00000017185	TMEM123	-0.081	0.039781611
ENSGALG00000026896		-0.081	0.039826581
ENSGALG00000040307	MORN2	0.081	0.039894565
ENSGALG00000026465	TMEM100	-0.081	0.039958483
ENSGALG00000009365	CYP51A1	-0.081	0.039972926
ENSGALG00000014662	RBP5	-0.081	0.03997879
ENSGALG00000050256	TRPT1	0.081	0.040033443
ENSGALG00000015185	TAF13	-0.081	0.040069718
ENSGALG00000048930	CDC37	-0.081	0.040071873
ENSGALG00000053917	CD164	-0.081	0.040113309
ENSGALG00000016357	PRDX4	-0.081	0.04013698
ENSGALG00000006638	GRIK4	-0.081	0.04020348
ENSGALG00000038733	TRAK1	-0.081	0.040209751
ENSGALG00000034225	DEAF1	-0.081	0.040236952
ENSGALG00000043545	PNPT1	0.081	0.040289586
ENSGALG00000003880	STX10	-0.081	0.040325876
ENSGALG00000003941	FUBP3	-0.081	0.040356502
ENSGALG00000030569	TRIM41	-0.081	0.040397989
ENSGALG00000035115		-0.081	0.040445287
ENSGALG00000002667	OLFML2B	-0.081	0.040486333
ENSGALG00000035576	CDK9	-0.081	0.040502153
ENSGALG00000040097	UBE2W	-0.081	0.040510716
ENSGALG00000008435	SUMO1	-0.081	0.040539091
ENSGALG00000011569	SCYL2	-0.081	0.04056127
ENSGALG00000014935	GREB1L	-0.081	0.040577456
ENSGALG00000006494	GRAMD1B	-0.081	0.040649598
ENSGALG00000013594	PARD6G	-0.081	0.040651773
ENSGALG00000038528		0.081	0.040654949
ENSGALG00000008975	FAM3C	-0.081	0.040655315
ENSGALG00000006413	CEMIP	-0.081	0.040665809
ENSGALG00000042079	NID1	-0.081	0.040679275
ENSGALG00000003185	SCPEP1	-0.081	0.040688164
ENSGALG00000012230	LRR1	-0.081	0.04071151
ENSGALG00000020986		0.081	0.040719626
ENSGALG00000005087	FAM83G	-0.081	0.040726668
ENSGALG00000009790	RAB33B	-0.081	0.040770843
ENSGALG00000001369		0.081	0.040829078

ENSGALG00000036101	ARHGAP35	-0.081	0.040878998
ENSGALG00000007595	LRCH3	-0.081	0.040903362
ENSGALG00000026902	P2RX6	0.081	0.040951784
ENSGALG00000015958	EIF2AK3	-0.081	0.040979919
ENSGALG00000005698	HIF1AN	-0.081	0.041055596
ENSGALG00000010818	PLPP3	-0.081	0.041056333
ENSGALG00000053068	SMUG1	-0.081	0.041079292
ENSGALG00000027591	TSC22D2	-0.081	0.041086964
ENSGALG00000031062	BAG5	-0.081	0.041151831
ENSGALG00000005158	WDFY1	-0.081	0.041167898
ENSGALG00000029760	NRBP2	0.081	0.041223189
ENSGALG00000006755	USP2	0.081	0.041232079
ENSGALG00000004387	CCPG1	-0.081	0.041245357
ENSGALG00000041528	LMF2	-0.081	0.041276817
ENSGALG00000035816	FDXR	-0.081	0.041312562
ENSGALG00000029766	ITGB5	-0.081	0.041331278
ENSGALG00000011139	CCDC85C	-0.081	0.04139732
ENSGALG00000012216	BMPR1B	-0.081	0.041501606
ENSGALG00000049181	CHST14	-0.081	0.041516346
ENSGALG00000014031	ETFRF1	0.081	0.041531976
ENSGALG00000009314	CACUL1	-0.081	0.041544881
ENSGALG00000043641	JUND	-0.081	0.041545005
ENSGALG00000010246	OSBPL8	-0.081	0.041552678
ENSGALG00000047102	SAPCD2	-0.081	0.041574338
ENSGALG00000036412	TMEM108	0.081	0.04164695
ENSGALG00000024034	PI4KB	-0.081	0.041650492
ENSGALG00000007747	TMEM104	-0.081	0.041670729
ENSGALG00000010891	ABCB11	-0.081	0.041673786
ENSGALG00000000564	CCDC47	-0.081	0.041695919
ENSGALG00000016934		-0.081	0.041699645
ENSGALG00000004816		-0.081	0.04173493
ENSGALG00000001431	GUCA1A	-0.081	0.041764179
ENSGALG00000026820	RNF146	-0.081	0.041770883
ENSGALG00000016947	ELF1	-0.081	0.041772442
ENSGALG00000043909		-0.081	0.041803983
ENSGALG00000013424	TULP3	-0.081	0.041856667
ENSGALG00000049875		-0.081	0.041862615

ENSGALG00000001209	TP53INP2	-0.081	0.041867412
ENSGALG00000043733	TMEM50A	-0.080	0.041953108
ENSGALG00000003505	LACTB	-0.080	0.04206748
ENSGALG00000000707	PSMD7	0.080	0.042071054
ENSGALG00000006453	TF	-0.080	0.042082532
ENSGALG00000017018	INTS6	0.080	0.042122881
ENSGALG00000013064	NSUN2	-0.080	0.042125345
ENSGALG00000009856	GNS	-0.080	0.042164205
ENSGALG00000025898	SMAD6	-0.080	0.042200722
ENSGALG00000002908	TRAIP	0.080	0.042211533
ENSGALG00000004959	IRS1	-0.080	0.042294932
ENSGALG00000054637		-0.080	0.042299596
ENSGALG00000014656	RPRD2	-0.080	0.042324676
ENSGALG00000054017		-0.080	0.042330089
ENSGALG00000008473	PLXND1	-0.080	0.042399676
ENSGALG00000037193	HECW1	-0.080	0.042417432
ENSGALG00000050552		0.080	0.042470805
ENSGALG00000008103	CEP41	0.080	0.042489895
ENSGALG00000016582	PBK	0.080	0.04250422
ENSGALG00000008301	FAM185A	-0.080	0.042587922
ENSGALG00000016279	RAB23	-0.080	0.042600669
ENSGALG00000006564		-0.080	0.042660581
ENSGALG00000011570	ILF2	0.080	0.042733511
ENSGALG00000011280	EEA1	-0.080	0.042752026
ENSGALG00000013532	CAPRIN1	-0.080	0.042769299
ENSGALG00000008496	VPS18	-0.080	0.04278379
ENSGALG00000043290		-0.080	0.042803458
ENSGALG00000048200	MRPL33	-0.080	0.042814467
ENSGALG00000010718		-0.080	0.042823205
ENSGALG00000035923	MTMR11	-0.080	0.042830126
ENSGALG00000005475		-0.080	0.042865027
ENSGALG00000033277	PPM1M	-0.080	0.042937244
ENSGALG00000026722	UNC119B	-0.080	0.043040856
ENSGALG00000009523	CNTN1	-0.080	0.043084012
ENSGALG00000005328	IBA57	-0.080	0.043091825
ENSGALG00000003301	FAM167B	0.080	0.04312428
ENSGALG00000004696	LEO1	-0.080	0.043126103

ENSGALG00000002316	NUBP2	-0.080	0.043144711
ENSGALG00000001479	CYB5D2	-0.080	0.043166944
ENSGALG00000031843	ARHGAP18	-0.080	0.043174253
ENSGALG00000042491		0.080	0.043186751
ENSGALG00000001522	PHF20	-0.080	0.043196227
ENSGALG00000052199	SNRPD2	-0.080	0.043211504
ENSGALG00000005858	DR1	-0.080	0.043237326
ENSGALG00000029746	NT5DC3	-0.080	0.043248296
ENSGALG00000004347	NEDD4	-0.080	0.043254853
ENSGALG00000048082		-0.080	0.043318291
ENSGALG00000009990	SRBD1	-0.080	0.043347916
ENSGALG00000005237	FGD3	-0.080	0.043395281
ENSGALG00000014936	ESCO1	-0.080	0.043433637
ENSGALG00000006642	ADORA2A	-0.080	0.043442441
ENSGALG00000034187	SEPTIN8	-0.080	0.043456423
ENSGALG00000053130	MYOZ3	0.080	0.04346449
ENSGALG00000010542	PAPSS1	-0.080	0.043475859
ENSGALG00000007481	HACD3	-0.080	0.043498768
ENSGALG00000008306	FGL2	-0.080	0.043498983
ENSGALG00000031094	EDA2R	-0.080	0.043538949
ENSGALG00000010357	P2RY1	-0.080	0.043549633
ENSGALG00000040393	BORCS5	-0.080	0.043555948
ENSGALG00000019716	KRT40	-0.080	0.043578176
ENSGALG00000023812	CCNI2	-0.080	0.043632101
ENSGALG00000038688	ADAMTS17	-0.080	0.043642038
ENSGALG00000034044	TBC1D12	-0.080	0.043673332
ENSGALG00000001043	REXO1	-0.080	0.043684383
ENSGALG00000016487	LDAH	-0.080	0.043692119
ENSGALG00000039099	MAPK9	-0.080	0.043703419
ENSGALG00000026276	TCF15	0.080	0.04377248
ENSGALG00000050634		-0.080	0.043801325
ENSGALG00000004009		0.080	0.043853971
ENSGALG00000001376	ARID3B	-0.080	0.043890262
ENSGALG00000030357	ABL2	-0.080	0.043897728
ENSGALG00000026009	SRP9	-0.080	0.043901793
ENSGALG00000006252	LMO4	-0.080	0.043979728
ENSGALG00000031666	CCDC91	0.080	0.044001092

ENSGALG00000047230	SWI5	-0.080	0.044018075
ENSGALG00000006374	TBX6	-0.080	0.044039016
ENSGALG00000053490	FBXO45	-0.080	0.044043632
ENSGALG00000009260	TMEM62	-0.080	0.04404477
ENSGALG00000008780	CTBS	-0.080	0.044082104
ENSGALG00000007123		-0.080	0.044088058
ENSGALG00000040758	INSR	-0.080	0.044140191
ENSGALG00000005169	FUT11	-0.080	0.04419546
ENSGALG00000011298	RARB	-0.080	0.044245525
ENSGALG00000007721	ARHGAP21	-0.080	0.044312218
ENSGALG00000005269	INTS2	-0.080	0.044336661
ENSGALG00000006837	TSPAN4	-0.080	0.044347468
ENSGALG00000029212	PADI2	-0.080	0.044370504
ENSGALG00000048117	DNLZ	0.080	0.044396205
ENSGALG00000001708	MED1	-0.080	0.044425421
ENSGALG00000038529	PLPPR3	0.080	0.044458809
ENSGALG00000002615	DEF6	0.079	0.044564665
ENSGALG00000002761	SIRT1	-0.079	0.04461604
ENSGALG00000032993	FTSJ3	-0.079	0.044648896
ENSGALG00000016776	TBC1D8	-0.079	0.044657205
ENSGALG00000002496	SFTPA1	0.079	0.044682329
ENSGALG00000014749	EPHB6	-0.079	0.044754388
ENSGALG00000008122	SLC39A13	-0.079	0.044760734
ENSGALG00000002841	RFNG	-0.079	0.044770458
ENSGALG00000021653	IFT22	-0.079	0.044774943
ENSGALG00000035281	WAC	-0.079	0.044778165
ENSGALG00000014012	WASF2	-0.079	0.044799213
ENSGALG00000005872	BANP	-0.079	0.044887058
ENSGALG00000011633	SLC5A8	-0.079	0.044938125
ENSGALG00000007831	TLX1	0.079	0.044982526
ENSGALG00000008553	INO80	-0.079	0.044990119
ENSGALG00000054320		-0.079	0.045033867
ENSGALG00000029893	COG1	-0.079	0.045088277
ENSGALG00000002527		-0.079	0.045088603
ENSGALG00000051904	NLGN2	-0.079	0.045096532
ENSGALG00000040248	PLEKHF2	-0.079	0.045134179
ENSGALG00000032249	MAX	-0.079	0.045189657

ENSGALG00000015591	ACOX3	0.079	0.045193354
ENSGALG00000042462		-0.079	0.045224552
ENSGALG00000012300	RAB32	-0.079	0.045226903
ENSGALG00000013829	REPS1	-0.079	0.045233762
ENSGALG00000014355	STIM2	-0.079	0.045251222
ENSGALG00000021345	B3GNT9	-0.079	0.045256792
ENSGALG00000007044	METTL9	-0.079	0.045264151
ENSGALG00000015181	GAREM1	-0.079	0.045324097
ENSGALG00000039335	SLC25A22	-0.079	0.045353402
ENSGALG00000030081	SKAP2	-0.079	0.045460261
ENSGALG00000030826	CLCN7	-0.079	0.045462201
ENSGALG00000033058	FASTK	-0.079	0.045506479
ENSGALG00000040864	WDR77	-0.079	0.045590843
ENSGALG00000008405	RALBP1	-0.079	0.04568257
ENSGALG00000006788	RPRD1B	-0.079	0.045699175
ENSGALG00000053428		-0.079	0.045707707
ENSGALG00000047673		-0.079	0.045761095
ENSGALG00000031461	CTDP1	-0.079	0.045783632
ENSGALG00000040368	CALR	-0.079	0.045816497
ENSGALG00000031214	MED9	-0.079	0.045936772
ENSGALG00000012092	ESYT3	-0.079	0.045950985
ENSGALG00000033759	RAVER2	-0.079	0.045984601
ENSGALG00000014467	COPS7A	-0.079	0.045994069
ENSGALG00000006689	ABHD2	-0.079	0.046034234
ENSGALG00000008057	LLGL2	-0.079	0.046173092
ENSGALG00000048164	LHFPL4	-0.079	0.046174176
ENSGALG00000019976		0.079	0.04618285
ENSGALG00000023554	PIGW	-0.079	0.046203032
ENSGALG00000006092		-0.079	0.046218425
ENSGALG00000036942	KCNA3	-0.079	0.046237356
ENSGALG00000031928	SPTLC2	-0.079	0.046243115
ENSGALG00000039818	GLG1	-0.079	0.046257263
ENSGALG00000037203	CTNNB1	-0.079	0.046269154
ENSGALG00000017319	MRPL48	0.079	0.046330267
ENSGALG00000009978	DTD2	0.079	0.046331115
ENSGALG00000006272		0.079	0.04633593
ENSGALG00000042125	AP2B1	-0.079	0.046349101

ENSGALG00000016991	ESD	0.079	0.046365035
ENSGALG00000007326	PMM2	-0.079	0.046379465
ENSGALG00000003439	PLEKHM2	-0.079	0.0463803
ENSGALG00000016251		-0.079	0.04647281
ENSGALG00000042554	ABCD2	-0.079	0.046479903
ENSGALG00000034831	YTHDF2	-0.079	0.046506508
ENSGALG00000041384	SHKBP1	-0.079	0.046530454
ENSGALG00000016972	NUFIP1	-0.079	0.046665448
ENSGALG00000046787	NAGPA	-0.079	0.046675955
ENSGALG00000053468	ANKRD66	0.079	0.046705203
ENSGALG00000006421	SLC16A12	-0.079	0.046769242
ENSGALG00000005994	DERL3	-0.079	0.046776346
ENSGALG00000035950	NGFR	-0.079	0.046787177
ENSGALG00000012200	GCH1	-0.079	0.046814756
ENSGALG00000040156	CCDC88C	-0.079	0.046815763
ENSGALG00000050937		0.079	0.046857302
ENSGALG00000042511	PKDCC	-0.079	0.046861306
ENSGALG00000012089		-0.079	0.046912239
ENSGALG00000000761	TSKU	-0.079	0.0470371
ENSGALG00000001682	FBXL20	-0.079	0.047080304
ENSGALG00000026590	KLF13	-0.079	0.047097196
ENSGALG00000032783	SUMO3	-0.079	0.04713569
ENSGALG00000013889	RNMT	-0.079	0.04714745
ENSGALG00000010352	ARHGEF26	-0.079	0.047149344
ENSGALG00000007144	SIK3	-0.079	0.047167392
ENSGALG00000005530	VEZF1	-0.079	0.047195031
ENSGALG00000006546	CD81	-0.079	0.047207642
ENSGALG00000026572	RGS5	-0.079	0.047221049
ENSGALG00000001315	UBE2Z	-0.079	0.047242537
ENSGALG00000015117	DRD3	-0.079	0.047298988
ENSGALG00000050267	CALU	-0.078	0.047372398
ENSGALG00000003045	E2F1	-0.078	0.04737954
ENSGALG00000007169	EPC1	-0.078	0.047412463
ENSGALG00000008930	B3GNT2	-0.078	0.047433349
ENSGALG00000013091	EPS8	-0.078	0.047463896
ENSGALG00000010613	ZFYVE9	-0.078	0.047558041
ENSGALG00000012032	PRRG4	-0.078	0.047583881

ENSGALG00000002789	MAP2	-0.078	0.04763986
ENSGALG00000051520		0.078	0.047683229
ENSGALG00000051443	C1orf216	-0.078	0.047692091
ENSGALG00000035378	DNAJC5	-0.078	0.047745657
ENSGALG00000006407		0.078	0.047746811
ENSGALG00000004545	WDR72	-0.078	0.047818868
ENSGALG00000036833	PAXBP1	-0.078	0.047839659
ENSGALG00000049862		-0.078	0.047868764
ENSGALG00000040113	SFT2D3	-0.078	0.047876359
ENSGALG00000028988	ART7C	-0.078	0.047898306
ENSGALG00000036044		-0.078	0.047962581
ENSGALG00000006390	CRKL	-0.078	0.047965756
ENSGALG00000001434	STOM	-0.078	0.04802828
ENSGALG00000016820	GAS6	-0.078	0.048083193
ENSGALG00000014836	LPIN2	-0.078	0.048104532
ENSGALG00000002179	FBF1	0.078	0.048140854
ENSGALG00000054926		0.078	0.048162754
ENSGALG00000040771		0.078	0.048193593
ENSGALG00000007157	TENT5D	-0.078	0.048201811
ENSGALG00000054599		-0.078	0.048212206
ENSGALG00000030406	CDS2	-0.078	0.048265147
ENSGALG00000047993		-0.078	0.048288249
ENSGALG00000010842	UBR7	-0.078	0.048316563
ENSGALG00000014766	HAO2	-0.078	0.048328467
ENSGALG00000053846		0.078	0.048389743
ENSGALG00000027240	TSPAN13	-0.078	0.048390165
ENSGALG00000013830	PRELID3A	-0.078	0.048397499
ENSGALG00000003429	PBX1	-0.078	0.048409037
ENSGALG00000012295	CDKL1	-0.078	0.048412574
ENSGALG00000005998	RRAS	-0.078	0.048438438
ENSGALG00000015935	SMYD1	-0.078	0.048465325
ENSGALG00000001731	CDK12	-0.078	0.048477001
ENSGALG00000011835	FGF2	-0.078	0.048482053
ENSGALG00000005321	LAMA5	-0.078	0.048598279
ENSGALG00000027126	BLCAP	-0.078	0.048609552
ENSGALG00000027242	TTF1	-0.078	0.048623929
ENSGALG00000054086		-0.078	0.048644494

ENSGALG00000003765	A1CF	-0.078	0.048645154
ENSGALG00000013348	OTUD7B	-0.078	0.048697008
ENSGALG00000004859	ZNFX1	-0.078	0.048718707
ENSGALG00000005266	BEAN1	-0.078	0.048775674
ENSGALG00000003359	RAB5C	-0.078	0.048790762
ENSGALG00000001037	SCAI	0.078	0.048822187
ENSGALG00000008482	STAG2	-0.078	0.048829653
ENSGALG00000013452	ERP44	-0.078	0.048832544
ENSGALG00000012852	MKRN4P	-0.078	0.048833682
ENSGALG00000030160	DRC7	-0.078	0.048892927
ENSGALG00000036873	SIK2	-0.078	0.048921824
ENSGALG00000011068	COBLL1	-0.078	0.048931792
ENSGALG00000001621	CLDN5	-0.078	0.048961055
ENSGALG00000051276	GATAD2B	-0.078	0.048989708
ENSGALG00000005088	CAMK2G	-0.078	0.049026996
ENSGALG00000008074	AMMECR1	-0.078	0.049052375
ENSGALG00000003285	CDHR2	0.078	0.049054556
ENSGALG00000027231	NRARP	-0.078	0.049069216
ENSGALG00000004573	LRRC8A	-0.078	0.049111292
ENSGALG00000010333	CNOT6L	-0.078	0.049113102
ENSGALG00000050315	CLC2DL3	-0.078	0.049154591
ENSGALG00000002922	NOTCH2	-0.078	0.049264841
ENSGALG00000048876	LEAP2	0.078	0.049307107
ENSGALG00000003527	ARRDC2	-0.078	0.049309042
ENSGALG00000009234	AMDHD2	-0.078	0.049329539
ENSGALG00000027905	CSRNP1	-0.078	0.049418769
ENSGALG00000001600	DVL1	-0.078	0.049461815
ENSGALG00000049774		-0.078	0.049464907
ENSGALG00000039168	SCFD2	-0.078	0.049575461
ENSGALG00000032092	PPP4R1	-0.078	0.049578927
ENSGALG00000006385	UBXN7	-0.078	0.049590331
ENSGALG00000035094	CDH17	-0.078	0.049612474
ENSGALG00000052488	TMEM186	-0.078	0.049640562
ENSGALG00000015409	PROS1	-0.078	0.049739123
ENSGALG00000034575	MRI1	-0.078	0.049799069
ENSGALG00000035080	HARS	-0.078	0.049799711
ENSGALG00000022887	KBTBD11	-0.078	0.049816992

ENSGALG00000004669	ASPA	0.078	0.049833007
ENSGALG00000026981	NHSL1	-0.078	0.049846203
ENSGALG00000026328	MIEN1	-0.078	0.049847325
ENSGALG00000035774	PPFIA1	-0.078	0.049886375
ENSGALG00000044449	LAG3	0.078	0.049886516
ENSGALG00000051652		-0.078	0.049943561
ENSGALG00000054428	XXYLT1	-0.078	0.049960266
ENSGALG00000010826	PRKAA2	-0.078	0.049972239

gene and HSC

p_FDR	direction
5.41E-11	positive
1.71E-10	positive
1.71E-10	positive
2.14E-09	negative
6.56E-08	negative
6.56E-08	negative
1.20E-07	negative
1.20E-07	positive
3.66E-06	negative
4.03E-06	negative
5.68E-06	negative
1.11E-05	positive
1.27E-05	positive
1.65E-05	negative
4.75E-05	negative
5.64E-05	positive
0.000137032	negative
0.000302001	negative
0.000336272	positive
0.000336272	negative
0.000336272	positive
0.000336272	negative
0.000710572	positive
0.000794541	negative
0.00090162	positive
0.000989353	positive
0.000989353	negative
0.000989353	negative
0.000989353	negative
0.001147751	negative
0.00128386	positive
0.001381827	positive
0.001447322	positive

0.001601723	negative
0.001678554	positive
0.001793392	positive
0.001823463	positive
0.001823463	negative
0.001823463	negative
0.00196489	positive
0.00196489	positive
0.001998936	negative
0.001998936	positive
0.001998936	negative
0.001998936	positive
0.002235881	positive
0.002344141	positive
0.002504601	positive
0.002647808	negative
0.002754173	negative
0.002922869	negative
0.002974463	negative
0.003001732	positive
0.003001732	positive
0.00324873	positive
0.00344208	positive
0.00389051	positive
0.004309573	negative
0.004414055	negative
0.004475593	positive
0.004765037	negative
0.004765037	negative
0.004765037	negative
0.006039519	positive
0.006236246	positive
0.006236246	positive
0.006241875	positive
0.006630586	negative
0.00728331	positive
0.007923304	positive

0.007983229	negative
0.007983229	positive
0.008115838	positive
0.008133447	negative
0.008133447	positive
0.009069534	negative
0.009292204	negative
0.009292204	positive
0.009865485	positive
0.009865485	negative
0.010550353	negative
0.010550353	negative
0.011044122	negative
0.011044122	positive
0.011215899	positive
0.011215899	positive
0.012211824	negative
0.012211824	positive
0.012211824	negative
0.012211824	negative
0.013530029	negative
0.013530029	negative
0.013530029	negative
0.013835352	negative
0.014598104	positive
0.014598104	positive
0.014598104	negative
0.014752418	positive
0.014972009	positive
0.015232688	positive
0.015232688	negative
0.015535972	negative
0.015535972	negative
0.015535972	negative
0.015535972	negative
0.016199063	positive
0.016199063	positive

0.016457628	positive
0.016594885	negative
0.017114433	negative
0.017114433	negative
0.017114433	positive
0.017114433	negative
0.017211042	negative
0.017211042	positive
0.017211042	positive
0.017234991	negative
0.017964044	negative
0.017964044	positive
0.018169747	negative
0.0184368	negative
0.0184368	negative
0.0184368	negative
0.01863355	negative
0.01863355	negative
0.019117242	negative
0.01929676	negative
0.020311839	negative
0.020311839	positive
0.020311839	positive
0.020311839	negative
0.020311839	positive
0.021152338	negative
0.023126104	positive
0.023126104	negative
0.024925299	negative
0.024925299	negative
0.024925299	positive
0.025222629	negative
0.025222629	negative
0.025222629	negative
0.025907373	negative
0.026263471	positive
0.026317163	positive

0.026317163	negative
0.026317163	positive
0.027481767	negative
0.027481767	negative
0.027713302	positive
0.027723071	negative
0.027723071	positive
0.027725692	negative
0.027779743	positive
0.028151889	positive
0.028151889	negative
0.028151889	negative
0.028151889	positive
0.028151889	negative
0.029089002	negative
0.029089002	positive
0.02913607	negative
0.029163985	positive
0.029163985	negative
0.029342014	positive
0.030244667	negative
0.030244667	positive
0.032505104	negative
0.033218298	negative
0.033872508	negative
0.034027763	positive
0.034027763	negative
0.034027763	negative
0.03457674	positive
0.035088728	negative
0.035088728	positive
0.035088728	positive
0.035088728	positive
0.035853377	negative
0.036263256	positive
0.036773282	negative
0.036787709	positive

0.036787709	negative
0.037845104	positive
0.037845104	positive
0.038221872	negative
0.03899623	positive
0.039194211	negative
0.03920643	positive
0.039567408	negative
0.039567408	positive
0.039567408	positive
0.039567408	positive
0.039921917	negative
0.040059822	positive
0.040059822	positive
0.040846282	negative
0.040872909	positive
0.041189236	negative
0.04157849	negative
0.042671654	negative
0.042671654	negative
0.043156488	negative
0.043156488	positive
0.043156488	positive
0.043156488	negative
0.045099465	negative
0.045099465	negative
0.045552787	negative
0.045552787	negative
0.045552787	positive
0.046037836	positive
0.046194109	negative
0.046194109	negative
0.046509532	positive
0.046819907	positive
0.046866833	negative
0.046866833	negative
0.050199285	negative

0.050248326	negative
0.050507485	negative
0.050507485	negative
0.050507485	positive
0.050507485	positive
0.050507485	negative
0.050563948	negative
0.050563948	negative
0.050563948	negative
0.050563948	negative
0.051006879	negative
0.0516006	negative
0.051754323	negative
0.051908961	positive
0.054054664	negative
0.054462712	negative
0.054610991	negative
0.054610991	negative
0.054610991	positive
0.055948144	negative
0.057149909	positive
0.057240021	positive
0.057358798	negative
0.057772838	negative
0.057905077	negative
0.058413261	negative
0.058413261	positive
0.058413261	positive
0.058413261	positive
0.058413261	negative
0.0584504	negative
0.0584504	negative
0.0584504	positive
0.058690659	positive
0.059212549	negative
0.059212549	negative
0.059307859	negative

0.060030238	negative
0.060892862	negative
0.061168363	negative
0.061192216	negative
0.061192216	positive
0.061658915	negative
0.062246355	positive
0.063466668	negative
0.063699706	negative
0.063729726	negative
0.064400599	negative
0.064453978	negative
0.06772165	negative
0.06772165	positive
0.067987972	positive
0.068409823	negative
0.0684897	negative
0.0684897	negative
0.0684897	negative
0.069538408	positive
0.069538408	negative
0.069538408	negative
0.069538408	negative
0.069799147	negative
0.070769526	positive
0.071230624	negative
0.071230624	negative
0.071230624	negative
0.071230624	positive
0.071230624	negative
0.071230624	positive
0.071230624	positive
0.071230624	negative
0.071230624	negative
0.071351018	negative
0.071351018	negative
0.071351018	negative

0.071351018	positive
0.071351018	negative
0.071856497	positive
0.072562001	negative
0.072562001	negative
0.073027824	negative
0.073027824	positive
0.073027824	negative
0.073027824	positive
0.073027824	negative
0.073030517	positive
0.073030517	negative
0.073030517	negative
0.073030517	positive
0.073030517	positive
0.073030517	negative
0.073182515	positive
0.073182515	negative
0.074044121	negative
0.075187453	negative
0.075427924	positive
0.07550898	positive
0.07550898	negative
0.07550898	positive
0.07550898	negative
0.075778454	negative
0.075796826	negative
0.076625207	positive
0.076625207	negative
0.077665891	negative
0.077665891	positive
0.077665891	negative
0.077665891	negative
0.077665891	negative
0.077665891	positive
0.077665891	positive
0.077835856	negative

0.078616513	positive
0.080453173	negative
0.080458199	positive
0.081188151	negative
0.081188151	positive
0.081188151	negative
0.081721165	negative
0.08181495	negative
0.081831922	negative
0.082175153	positive
0.082862064	negative
0.083015413	positive
0.083015413	negative
0.083015413	positive
0.083015413	negative
0.083015413	negative
0.083015413	negative
0.083015413	positive
0.083074394	negative
0.083074394	negative
0.084060845	negative
0.084169153	negative
0.08419844	positive
0.08419844	negative
0.084294318	negative
0.08453567	negative
0.08453567	negative
0.08453567	positive
0.08453567	negative
0.08453567	negative
0.08453567	positive
0.08453567	negative
0.08453567	negative
0.085000259	positive
0.085000259	positive
0.085000259	negative
0.086661977	positive

0.086661977	positive
0.087104383	positive
0.087104383	negative
0.087104383	negative
0.087104383	negative
0.087104383	negative
0.087104383	negative
0.08748098	negative
0.08748098	negative
0.08748098	negative
0.08748098	negative
0.08748098	negative
0.08748098	negative
0.08748098	positive
0.08748098	negative
0.08748098	negative
0.08748098	positive
0.08748098	negative
0.08748098	negative
0.08748098	negative
0.08748098	positive
0.08748098	negative
0.08748098	negative
0.087720374	negative
0.088028454	negative
0.088187942	negative
0.088530985	negative
0.088712491	positive
0.088818909	positive
0.089426438	negative
0.089426438	negative
0.089426438	positive
0.089426438	positive
0.089426438	positive
0.089426438	negative
0.089426438	positive
0.089426438	positive

0.089426438	negative
0.089426438	positive
0.089426438	positive
0.089426438	negative
0.089630606	positive
0.089630606	negative
0.089630606	positive
0.089630606	negative
0.089630606	positive
0.089630606	negative
0.089966024	negative
0.090032432	negative
0.090271013	negative
0.091291716	positive
0.091291716	negative
0.091447056	positive
0.092972727	negative
0.092976873	positive
0.092976873	negative
0.093082849	negative
0.093082849	positive
0.093104898	negative
0.093673214	positive
0.093721095	negative
0.093946991	negative
0.094867254	negative
0.095337832	positive
0.095337832	negative
0.095337832	positive
0.09543194	negative
0.09543194	negative
0.09543194	negative
0.095685434	negative
0.095705686	negative
0.096174515	negative
0.096174515	negative
0.096174515	negative

0.096174515	negative
0.096174515	negative
0.096455282	negative
0.096455282	negative
0.096455282	negative
0.096455282	positive
0.096455282	positive
0.096455282	negative
0.096455282	negative
0.096455282	positive
0.096455282	negative
0.096455282	negative
0.097258191	positive
0.097258191	negative
0.097258191	positive
0.097258191	negative
0.097338981	negative
0.097590932	positive
0.097590932	negative
0.097911308	negative
0.097911308	negative
0.097911308	negative
0.097911308	positive
0.098991837	negative
0.098991837	negative
0.099057968	negative
0.099057968	negative
0.099057968	negative
0.099057968	negative
0.099057968	negative
0.099057968	negative
0.099057968	negative
0.099422019	negative
0.099487849	positive
0.099529994	negative
0.099529994	negative
0.100019255	negative

0.100202319	negative
0.100202319	negative
0.100202319	positive
0.100202319	negative
0.100202319	negative
0.100202319	negative
0.100202319	negative
0.100399881	positive
0.100534587	positive
0.10120496	negative
0.10120496	positive
0.10120496	negative
0.101937237	negative
0.101937237	negative
0.102219394	negative
0.102983408	negative
0.102983408	negative
0.102983408	positive
0.103786783	negative
0.103786783	negative
0.103786783	negative
0.103786783	positive
0.103786783	positive
0.104101208	negative
0.104101208	negative
0.104122229	negative
0.104683219	positive
0.10515714	negative
0.10515714	negative
0.10515714	positive
0.10515714	positive
0.10526583	positive
0.10526583	positive
0.106132817	positive
0.106156058	negative
0.106547178	positive
0.106617064	negative

0.106636035	negative
0.106636035	negative
0.106709098	negative
0.106709098	negative
0.106709098	negative
0.10718248	negative
0.108090678	negative
0.108090678	negative
0.108279186	negative
0.108356767	negative
0.108717575	positive
0.108917313	negative
0.108990491	positive
0.109101228	positive
0.109101228	positive
0.109240912	positive
0.109240912	negative
0.109245973	negative
0.109245973	negative
0.109245973	negative
0.109245973	positive
0.109974436	negative
0.110611322	negative
0.110883329	negative
0.110977965	negative
0.110977965	negative
0.111500329	negative
0.111500329	positive
0.111704465	negative
0.111704465	positive
0.111704465	positive
0.111784574	negative
0.111899713	positive
0.111899713	negative
0.112068838	negative
0.112068838	negative
0.112068838	negative

0.112068838	negative
0.112068838	negative
0.112068838	negative
0.112068838	negative
0.112068838	negative
0.112068838	negative
0.112068838	negative
0.112337515	negative
0.112526183	negative
0.112526183	negative
0.112526183	positive
0.112526183	negative
0.112526183	positive
0.112526183	negative
0.112526183	positive
0.112526183	positive
0.114063755	positive
0.114418297	negative
0.11455506	negative
0.11455506	negative
0.11455506	negative
0.11455506	negative
0.11455506	negative
0.11455506	negative
0.11455506	positive
0.114685817	negative
0.114707185	negative
0.115072142	positive
0.115072142	negative
0.115072142	positive
0.115139482	negative
0.115139482	positive
0.115302526	positive
0.115490119	positive
0.115490119	negative
0.115490119	negative
0.115490119	negative

0.115490119	negative
0.115490119	negative
0.115490119	positive
0.115490119	negative
0.115820461	negative
0.116028475	negative
0.116440874	negative
0.116451472	negative
0.116451472	negative
0.116451472	negative
0.116564878	negative
0.116900816	negative
0.117019775	negative
0.117598778	negative
0.117598778	positive
0.117920426	negative
0.117985216	negative
0.118097793	positive
0.118262082	negative
0.118348586	positive
0.118783344	negative
0.119383065	negative
0.119383065	negative
0.119597845	negative
0.119664135	negative
0.120387704	negative
0.120471727	negative
0.120471727	positive
0.120471727	negative
0.12115814	negative
0.12115814	negative
0.121485443	negative
0.121485443	positive
0.12169747	negative
0.12169747	negative
0.121726178	negative
0.121726178	negative

0.122017869	positive
0.122062849	negative
0.122062849	negative
0.122062849	negative
0.122256386	negative
0.122256386	negative
0.122256386	negative
0.122769724	positive
0.122769724	negative
0.123110542	negative
0.123110542	positive
0.123110542	positive
0.123252836	negative
0.123252836	negative
0.123252836	negative
0.123500474	negative
0.124227298	negative
0.125503006	negative
0.125503006	negative
0.125503006	negative
0.125722077	negative
0.125941075	negative
0.125941075	negative
0.12635174	negative
0.12635174	positive
0.126437742	negative
0.126437742	negative
0.127020637	negative
0.127123085	negative
0.127529068	negative
0.127529068	negative
0.127529068	negative
0.127529068	positive
0.127529068	negative
0.127921414	negative
0.128486294	negative
0.128583727	negative

0.128749536	negative
0.128749536	negative
0.129054009	negative
0.129054009	negative
0.129054009	negative
0.129054009	negative
0.129054009	negative
0.129061549	negative
0.129288404	negative
0.129300672	positive
0.129948709	negative
0.130149276	negative
0.130894879	negative
0.131013975	positive
0.131013975	negative
0.131013975	negative
0.131013975	negative
0.13266023	negative
0.132893421	positive
0.13295133	negative
0.133091541	positive
0.133147033	negative
0.133594776	negative
0.134373601	negative
0.13471934	negative
0.134914282	positive
0.134914282	negative
0.134914282	negative
0.134935147	negative
0.135153365	negative
0.135153365	negative
0.135355725	positive
0.135355725	positive
0.135561643	negative
0.135561643	positive
0.135561643	negative
0.135561643	negative

0.135561643	negative
0.135561643	negative
0.13569107	negative
0.13569107	negative
0.13625403	negative
0.13625403	negative
0.13625403	negative
0.13625403	negative
0.13625403	negative
0.13625403	negative
0.13625403	negative
0.13625403	positive
0.13625403	negative
0.13625403	negative
0.136887198	positive
0.137235998	negative
0.137235998	negative
0.137235998	negative
0.137235998	positive
0.137235998	positive
0.137333901	negative
0.137333901	positive
0.137526778	positive
0.137673437	negative
0.137673437	positive
0.137673437	negative
0.137715984	negative
0.137715984	positive
0.137715984	negative
0.137715984	negative
0.137715984	negative
0.137759116	negative
0.137759116	negative
0.137759116	negative
0.137759116	negative
0.138020374	negative
0.139206878	negative

0.139206878	positive
0.139216111	negative
0.139216111	positive
0.140014515	negative
0.140273416	negative
0.140273416	negative
0.140273416	negative
0.140273416	positive
0.140273416	positive
0.140343688	positive
0.141036874	negative
0.141042519	negative
0.141046788	positive
0.141132831	positive
0.141201932	positive
0.141282482	positive
0.142180166	positive
0.142180166	negative
0.142180166	negative
0.142810766	negative
0.142810766	negative
0.143346892	negative
0.143348624	positive
0.143375818	negative
0.143473501	negative
0.14352678	positive
0.14352678	negative
0.144206867	positive
0.144209068	negative
0.144209068	negative
0.144722133	negative
0.144722133	negative
0.144722133	negative
0.144722133	negative
0.144722133	negative
0.144722133	negative
0.144722133	positive

0.144722133	negative
0.144722133	positive
0.144722133	negative
0.144722133	negative
0.144722133	negative
0.144722133	negative
0.144722133	negative
0.144722133	negative
0.144722133	positive
0.144853036	negative
0.144853036	positive
0.144853036	negative
0.145101975	positive
0.145101975	positive
0.145602975	negative
0.145602975	positive
0.145602975	positive
0.145602975	positive
0.145602975	negative
0.145602975	negative
0.145952407	negative
0.145952407	negative
0.146015013	positive
0.146015013	positive
0.146564381	negative
0.146965737	positive
0.146965737	negative
0.146965737	negative
0.146976843	negative
0.146976843	negative
0.147329142	positive
0.147329142	negative
0.147329142	positive
0.147609672	positive
0.147609672	negative
0.147996284	positive
0.147996284	positive

0.14803048	negative
0.14823656	positive
0.14823656	negative
0.14823656	positive
0.14823656	negative
0.148915539	negative
0.14899692	negative
0.14899692	negative
0.149508884	negative
0.149508884	negative
0.149508884	positive
0.150003426	positive
0.150040196	negative
0.15078481	negative
0.15078481	negative
0.15078481	negative
0.15078481	negative
0.15078481	negative
0.15078481	negative
0.151101116	positive
0.151101116	positive
0.151101116	positive
0.151101116	negative
0.151101116	negative
0.151101116	negative
0.151101116	negative
0.151101116	negative
0.151101116	negative
0.151101116	negative
0.151101116	negative
0.151101116	negative
0.151101116	positive
0.151101116	negative
0.151101116	negative
0.151126967	negative
0.151126967	positive
0.151283371	positive

0.151283371	negative
0.151337705	positive
0.15136203	positive
0.151594859	positive
0.152215575	positive
0.152215575	negative
0.152215575	positive
0.152215575	negative
0.152508589	positive
0.152792389	negative
0.153271813	positive
0.153501822	negative
0.154659595	positive
0.154873115	positive
0.155030028	negative
0.155030028	negative
0.155030028	negative
0.155031489	negative
0.155366011	negative
0.155541049	negative
0.155756796	positive
0.155879152	negative
0.155879152	negative
0.155879152	positive
0.155879152	negative
0.155879152	negative
0.155959848	negative
0.156239665	negative
0.156395226	negative
0.156395226	negative
0.156395226	negative
0.156395226	positive
0.156395226	negative
0.156395226	negative
0.156961845	negative
0.157777491	positive
0.157777491	negative

0.157797042	negative
0.157797042	positive
0.157875541	negative
0.158033778	negative
0.158033778	negative
0.158160397	negative
0.158160397	negative
0.158160397	negative
0.158223085	negative
0.158273087	negative
0.158273087	positive
0.158273087	negative
0.15872221	negative
0.158781234	positive
0.158781234	positive
0.158912418	negative
0.158912418	positive
0.158912418	negative
0.158912418	negative
0.159305002	positive
0.159453683	positive
0.1595701	negative
0.159889545	positive
0.159889545	negative
0.159889545	negative
0.159889545	negative
0.160155191	negative
0.160698476	negative
0.160698476	negative
0.160816705	negative
0.160816705	negative
0.160816705	negative
0.160816705	negative
0.160816705	negative
0.160816705	negative
0.161066828	negative
0.161066828	negative

0.161066828	positive
0.161066828	positive
0.161066828	positive
0.161066828	positive
0.161066828	negative
0.161066828	positive
0.161066828	negative
0.161066828	positive
0.161066828	negative
0.161532913	negative
0.161532913	negative
0.161592348	negative
0.161592348	negative
0.161829359	negative
0.161829359	negative
0.161829359	negative
0.161829359	negative
0.161914158	negative
0.162059823	negative
0.162465132	negative
0.162682409	negative
0.162740359	negative
0.162983276	negative
0.162983276	negative
0.162983276	negative
0.162995135	negative
0.163233084	negative
0.163238121	negative
0.163238121	negative
0.16484578	negative
0.16492986	positive
0.164937499	negative
0.165183183	positive
0.165183183	positive
0.165183183	negative
0.165517792	negative
0.165517792	negative

0.165525453	negative
0.165525453	negative
0.165525453	positive
0.165525453	negative
0.16583506	positive
0.16583506	negative
0.16583506	negative
0.16583506	negative
0.16583506	negative
0.16583506	negative
0.16583506	negative
0.16583506	positive
0.16583506	negative
0.16583506	negative
0.16583506	negative
0.16583506	negative
0.16583506	negative
0.16583506	positive
0.16583506	positive
0.16583506	negative
0.16583506	negative
0.16583506	positive
0.16583506	negative
0.16583506	negative
0.16583506	negative
0.16583506	negative
0.16583506	negative
0.16583506	negative
0.16583506	negative
0.16583506	positive
0.16583506	negative
0.16583506	negative
0.16583506	negative
0.16583506	negative
0.16583506	positive
0.16583506	negative
0.16583506	positive

0.16583506	negative
0.16583506	negative
0.16583506	negative
0.16583506	negative
0.16583506	negative
0.16583506	positive
0.16583506	negative
0.16583506	negative
0.16583506	negative
0.165856835	positive
0.16597988	positive
0.166219616	negative
0.16653075	negative
0.16653075	negative
0.16653075	negative
0.166546974	negative
0.166546974	negative
0.166715355	negative
0.166715355	negative
0.166715355	negative
0.166715355	negative
0.166715355	positive
0.166715355	negative
0.166715355	negative
0.166715355	negative
0.166715355	positive
0.166715355	negative
0.166715355	positive
0.166715355	negative
0.166715355	negative
0.166989542	negative
0.167008451	negative
0.167008451	negative
0.167008451	negative
0.167008451	negative
0.167008451	positive
0.167117759	negative

0.167117759	negative
0.167361015	positive
0.168116534	positive
0.168116534	negative
0.168130807	negative
0.168148165	negative
0.168148165	negative
0.168189602	negative
0.168247041	negative
0.168247041	negative
0.168704222	negative
0.168704222	positive
0.168704222	negative
0.168973684	negative
0.168973684	negative
0.168973684	negative
0.169099722	negative
0.169099722	negative
0.169247263	negative
0.169247263	negative
0.169247263	positive
0.169711777	negative
0.171349375	negative
0.171421682	positive
0.171957023	negative
0.172011863	negative
0.172467135	negative
0.172695972	negative
0.172928424	negative
0.173028315	negative
0.173028315	negative
0.173161914	negative
0.173331225	negative
0.173331225	positive
0.173331225	negative
0.173331225	negative
0.173453416	negative

0.173490539	negative
0.173490539	positive
0.173490539	negative
0.173490539	negative
0.173490539	negative
0.173490539	negative
0.173490539	negative
0.173490539	negative
0.173490539	positive
0.173490539	negative
0.173490539	negative
0.173494103	negative
0.17352583	positive
0.17352583	negative
0.173586896	negative
0.173586896	negative
0.173836339	negative
0.17399414	negative
0.17399414	negative
0.17399414	negative
0.17399414	positive
0.17399414	positive
0.17399414	negative
0.17399414	positive
0.17399414	negative
0.17399414	positive
0.17399414	negative
0.17399414	negative
0.17399414	negative
0.17399414	negative
0.17399414	negative
0.17399414	negative
0.17399414	positive
0.17399414	positive
0.17399414	negative
0.17399414	negative
0.17399414	negative

0.174089923	negative
0.174111831	negative
0.174232046	positive
0.174232046	negative
0.174276802	positive
0.174470074	positive
0.174988767	positive
0.17518176	positive
0.17518176	negative
0.175515462	positive
0.175517809	negative
0.175517809	negative
0.175611222	negative
0.175683143	negative
0.176110425	positive
0.176133246	negative
0.176133246	positive
0.176133246	negative
0.176362114	positive
0.176362114	positive
0.176362114	negative
0.176362114	negative
0.176362114	negative
0.176362114	negative
0.176362114	negative
0.176362114	negative
0.176362114	positive
0.176362114	negative
0.176362114	negative
0.176362114	positive
0.176362114	negative
0.176362114	negative
0.176362114	negative
0.176362114	positive
0.176362114	negative
0.176362114	negative
0.176362114	negative

0.17770344	negative
0.177766462	negative
0.177912271	negative
0.178358865	negative
0.17873504	negative
0.17873504	negative
0.17873504	negative
0.17873504	negative
0.17873504	negative
0.17873504	negative
0.1792187	negative
0.1792187	negative
0.179422418	negative
0.179706334	negative
0.179756059	negative
0.179756059	positive
0.179756059	negative
0.179756059	negative
0.179756059	negative
0.180214292	negative
0.180506507	negative
0.180506507	negative
0.180506507	negative
0.180506507	negative
0.180506507	negative
0.18077005	negative
0.18077005	negative
0.18077005	positive
0.18077005	negative
0.18077005	negative
0.18077005	negative
0.181213916	negative
0.181213916	negative
0.181213916	positive
0.181213916	negative
0.181213916	negative
0.181213916	negative

0.181213916	negative
0.181213916	negative
0.181261433	negative
0.18157778	negative
0.181825158	negative
0.181848003	positive
0.181848003	negative
0.181848003	positive
0.181848003	negative
0.181848003	negative
0.181848003	positive
0.181848003	positive
0.181848003	negative
0.181848003	negative
0.181848003	negative
0.181848003	negative
0.181848003	negative
0.181848003	negative
0.181848003	negative
0.181983007	negative
0.181983007	negative
0.182127502	negative
0.182127502	negative
0.183034126	negative
0.183034126	negative
0.183546614	negative
0.183546614	negative
0.183760157	negative
0.183986224	negative
0.183986224	negative
0.183986224	negative
0.183986224	negative
0.184044773	negative
0.184104199	negative
0.184104199	negative
0.184498156	negative
0.184498156	negative

0.184498156	negative
0.184498156	negative
0.185227354	negative
0.185369521	negative
0.185849192	positive
0.185849192	negative
0.185849192	negative
0.185849192	positive
0.185849192	negative
0.186230497	negative
0.186397721	negative
0.186397721	negative
0.18707733	positive
0.187151671	negative
0.187376979	negative
0.187376979	negative
0.187376979	negative
0.187376979	negative
0.187406635	negative
0.187587477	positive
0.187619211	negative
0.187619211	negative
0.187619211	negative
0.187619211	negative
0.187619211	negative
0.187619211	negative
0.18791608	negative
0.187984774	negative
0.187984774	negative
0.187984774	negative
0.187984774	negative
0.188299056	negative
0.188299056	positive
0.1885408	positive
0.188561068	negative
0.188780925	negative
0.188807059	negative

0.189519478	positive
0.189519478	negative
0.189519478	negative
0.189519478	negative
0.189519478	positive
0.189686906	negative
0.189894782	positive
0.190606413	negative
0.190914164	negative
0.191448317	negative
0.191448317	negative
0.191448317	negative
0.191448317	positive
0.191448317	negative
0.191448317	negative
0.191448317	positive
0.191448317	positive
0.191448317	negative
0.191448317	negative
0.191448317	negative
0.191448317	negative
0.191448317	negative
0.191448317	negative
0.191448317	negative
0.191448317	negative
0.191448317	negative
0.191448317	positive
0.191448317	positive
0.191725796	negative
0.191866843	negative
0.192005281	negative
0.192005281	negative
0.192005281	negative
0.192005281	negative
0.192484671	negative
0.192484671	negative
0.192484671	negative

0.192498567	negative
0.192498567	negative
0.192498567	positive
0.192748876	negative
0.192748876	negative
0.192748876	negative
0.192748876	negative
0.192777796	negative
0.192834364	negative
0.192834364	negative
0.193385571	positive
0.193385571	negative
0.193385571	negative
0.193385571	positive
0.193416724	negative
0.193416724	negative
0.194139916	negative
0.194139916	positive
0.194139916	negative
0.194139916	negative
0.194433889	positive
0.194963185	negative
0.194963185	positive
0.195298029	negative
0.195335828	negative
0.195335828	positive
0.195335828	negative
0.195460926	negative
0.195928486	positive
0.19596756	negative
0.19596756	negative
0.195983255	negative
0.195983255	negative
0.195983255	negative
0.19602015	negative
0.196166493	negative
0.196166493	negative

0.196166493	positive
0.196166493	negative
0.196166493	negative
0.196166493	negative
0.196928468	positive
0.196937962	negative
0.196937962	negative
0.196937962	negative
0.196937962	negative
0.196937962	negative
0.196937962	negative
0.196937962	negative
0.196937962	negative
0.196937962	negative
0.196937962	negative
0.196937962	negative
0.196937962	negative
0.197029081	negative
0.197090612	negative
0.197532409	negative
0.197532409	negative
0.197532409	negative
0.197532409	positive
0.197532409	negative
0.19760906	positive
0.19760906	negative
0.197739978	negative
0.197739978	positive
0.197955098	negative
0.197955098	negative
0.197955098	negative
0.197955098	negative
0.197955098	negative
0.197955098	negative
0.197955098	negative
0.197955098	negative
0.197955098	negative

0.198588432	positive
0.198990391	positive
0.199190791	negative
0.199227841	negative
0.200056331	negative
0.200205038	negative
0.200205038	negative
0.200205038	positive
0.200340668	negative
0.200430377	negative
0.200488701	negative
0.200488701	positive
0.200878742	positive
0.200878742	negative
0.200878742	negative
0.200878742	positive
0.201130098	negative
0.201130098	negative
0.201130098	negative
0.201130098	negative
0.201130098	negative
0.201130098	negative
0.201130098	negative
0.201130098	negative
0.201130098	positive
0.201130098	negative
0.201130098	negative
0.201130098	negative
0.201130098	negative
0.201130098	negative
0.201255817	negative
0.201255817	negative
0.201879353	negative
0.201879353	negative
0.201879353	negative
0.202134552	negative
0.202134552	negative

0.202165441	negative
0.202165441	negative
0.202165441	negative
0.202165441	negative
0.202165441	negative
0.202165441	negative
0.202165441	positive
0.202717956	negative
0.20348092	negative
0.20348092	positive
0.20348092	negative
0.20348092	negative
0.20348092	negative
0.20348092	negative
0.20348092	negative
0.20348092	negative
0.20348092	negative
0.20348092	negative
0.20350482	negative
0.20350482	negative
0.20386817	negative
0.204323056	positive
0.204323056	negative
0.204323056	negative
0.204323056	negative
0.205390396	positive
0.205390396	positive
0.205390396	negative
0.205390396	negative
0.205390396	negative
0.205390396	negative
0.205390396	negative
0.205390396	negative
0.205390396	negative
0.205390396	positive
0.205390396	negative
0.205390396	negative

0.20540008	negative
0.205418152	negative
0.205669238	negative
0.205846677	negative
0.206071737	negative
0.206071737	negative
0.206071737	positive
0.206071737	negative
0.20621449	positive
0.20621449	negative
0.206268413	negative
0.206386546	negative
0.206509366	negative
0.206662927	negative
0.206662927	negative
0.207160758	negative
0.207632821	negative
0.207632821	negative
0.207632821	negative
0.207632821	positive
0.207733534	negative
0.207733534	positive
0.207733534	negative
0.207733534	negative
0.208160914	negative
0.208496958	negative
0.208972621	negative
0.208972621	negative
0.208972621	negative
0.208972621	positive
0.209026448	negative
0.209170925	negative
0.209170925	negative
0.209170925	negative
0.209226159	positive
0.209378185	negative
0.209378185	negative

0.209420974	positive
0.209420974	negative
0.209880284	negative
0.210090242	negative
0.210090242	negative
0.210099094	negative
0.210642105	negative
0.210642105	negative
0.210642105	negative
0.210642105	negative
0.210661518	negative
0.21082621	negative
0.21082621	negative
0.21082621	negative
0.210885858	negative
0.210905574	negative
0.210905574	negative
0.210905574	positive
0.211233693	negative
0.211445821	negative
0.211504419	positive
0.211504419	negative
0.211963111	negative
0.21201635	negative
0.21201635	positive
0.21201635	negative
0.212063324	negative
0.212063324	negative
0.212063324	negative
0.212063324	negative
0.21207705	negative
0.212133211	negative
0.212379334	negative
0.212379334	negative
0.212461012	negative
0.212461012	negative
0.212461012	positive

0.212461012	negative
0.212825888	negative
0.213115838	negative
0.213177349	negative
0.213177349	negative
0.213177349	negative
0.213177349	negative
0.213177349	negative
0.213177349	negative
0.213177349	negative
0.213203236	negative
0.213203236	negative
0.213203236	negative
0.213203236	positive
0.213203236	negative
0.213203236	negative
0.213203236	negative
0.213203236	negative
0.213355656	negative
0.213355656	negative
0.213355656	negative
0.21362855	negative
0.21364863	negative
0.21364863	negative
0.21364863	positive
0.214000484	negative
0.214000484	negative
0.214732556	negative
0.214732556	negative
0.214939432	positive
0.214939432	negative
0.215180514	negative
0.215542865	negative
0.215542865	negative
0.215542865	negative
0.215574416	negative
0.215574416	negative

0.215574416	negative
0.215574416	negative
0.215574416	positive
0.215574416	positive
0.215574416	negative
0.215675701	positive
0.216029965	negative
0.216029965	positive
0.216029965	negative
0.216029965	negative
0.216169043	negative
0.216169043	positive
0.2162671	negative
0.216584858	negative
0.216584858	negative
0.216584858	negative
0.216607979	positive
0.216607979	positive
0.217097144	negative
0.217097144	negative
0.217097144	negative
0.217097144	negative
0.217097144	negative
0.217097144	negative
0.217097144	negative
0.217097144	positive
0.217097144	negative
0.217097144	negative
0.217097144	negative
0.217240103	positive
0.217523312	positive
0.217523312	negative
0.217523312	positive
0.217523312	negative
0.217523312	negative
0.217575779	negative
0.217575779	negative

0.217775428	negative
0.217775428	positive
0.218188036	negative
0.21822625	negative
0.218887381	negative
0.219014358	negative
0.219014358	positive
0.219166887	negative
0.219166887	positive
0.219166887	negative
0.219166887	negative
0.219166887	positive
0.219166887	negative
0.219270843	positive
0.219270843	negative
0.219270843	negative
0.219270843	negative
0.219270843	negative
0.219270843	negative
0.219307506	negative
0.219375878	negative
0.219375878	negative
0.219375878	negative
0.219375878	negative
0.219375878	positive
0.219375878	negative
0.219375878	negative
0.219375878	negative
0.219375878	positive
0.219375878	negative
0.219375878	negative
0.219602224	negative
0.219602224	positive
0.219862541	positive
0.219872578	negative
0.219885216	positive
0.219885216	negative

0.219885216	positive
0.219885216	positive
0.219885216	negative
0.219885216	negative
0.219885216	negative
0.219885216	positive
0.220464549	negative
0.220464549	negative
0.220464549	negative
0.220464549	negative
0.220464549	negative
0.220464549	negative
0.220464549	positive
0.220464549	negative
0.220464549	negative
0.220464549	negative
0.220464549	negative
0.220464549	negative
0.220464549	negative
0.220464549	positive
0.220464549	negative
0.220464549	negative
0.220464549	negative
0.220465311	negative
0.220465311	positive
0.220465311	negative
0.220692122	negative
0.220692122	negative
0.220780685	negative
0.220780685	negative
0.220780685	negative
0.220780685	negative
0.220780685	negative
0.22078101	positive
0.220908914	negative
0.22132573	negative
0.221471261	negative

0.221471261	negative
0.221471261	negative
0.221471261	negative
0.221471261	positive
0.221471261	negative
0.221542285	negative
0.221542285	negative
0.221542285	positive
0.221542285	negative
0.221542285	negative
0.221542285	negative
0.221542285	negative
0.221542285	negative
0.221623416	negative
0.221623416	negative
0.221623416	positive
0.221623416	positive
0.221623416	negative
0.221623416	negative
0.221623416	negative
0.221623416	negative
0.221623416	negative
0.221623416	negative
0.221623416	negative
0.221623416	negative
0.221623416	negative
0.221623416	negative
0.221623416	negative
0.221623416	negative
0.221623416	negative
0.221623416	negative
0.221623416	negative
0.221623416	negative
0.221623416	negative
0.221623416	negative
0.221623416	negative
0.221623416	negative

0.221623416	negative
0.221623416	negative
0.221623416	negative
0.221623416	negative
0.221623416	negative
0.221623416	negative
0.221623416	positive
0.221623416	negative
0.221623416	negative
0.221623416	negative
0.221623416	negative
0.221623416	negative
0.221770883	negative
0.222086626	negative
0.222086626	negative
0.2222401	negative
0.2222401	negative
0.2222401	negative
0.2222401	negative
0.2222401	negative
0.2222401	negative
0.2222401	negative
0.2222401	negative
0.2222401	negative
0.2222401	positive
0.2222401	positive
0.2222401	positive
0.2222401	negative
0.2222401	negative
0.2222401	negative
0.2222401	negative
0.222258405	negative
0.222258405	negative
0.222258405	negative
0.222258405	positive
0.222258405	negative
0.222258405	negative

0.222258405	positive
0.222258405	negative
0.222258405	negative
0.222258405	negative
0.222258405	negative
0.222507011	negative
0.22267199	negative
0.222734733	negative
0.223018	negative
0.223169143	negative
0.223192281	negative
0.223271149	negative
0.223293151	negative
0.223293151	positive
0.223293151	negative
0.223293151	negative
0.223293151	negative
0.223293151	negative
0.223293151	positive
0.223293151	negative
0.223293151	negative
0.223293151	negative
0.223509521	negative
0.223509521	negative
0.223509521	positive
0.223509521	negative
0.223562473	negative
0.223562473	negative
0.223834616	negative
0.2239163	positive
0.224187972	negative
0.224635724	positive
0.224635724	negative
0.224867489	negative
0.224867489	negative
0.224867489	negative
0.224867489	negative

0.224972939	negative
0.225128505	negative
0.225128505	negative
0.225128505	negative
0.225427623	negative
0.225427623	negative
0.225514308	negative
0.225704138	negative
0.225704138	negative
0.22580836	negative
0.225869069	negative
0.225869069	negative
0.22592469	negative
0.226446564	negative
0.226594334	negative
0.226614653	negative
0.226614653	negative
0.226643574	negative
0.226643574	positive
0.226643574	negative
0.226643574	negative
0.226643574	negative
0.226643574	negative
0.226643574	positive
0.226643574	negative
0.226782436	negative
0.226794463	negative
0.226794463	negative
0.226794463	negative
0.226794463	negative
0.227120434	positive
0.227120434	positive
0.227136982	negative
0.227136982	negative
0.227163911	negative
0.22732509	negative
0.227634999	positive

0.227634999	negative
0.227910145	negative
0.227910145	negative
0.227910145	negative
0.227910145	positive
0.227910145	negative
0.227910145	negative
0.227910145	negative
0.22834516	negative
0.2285248	negative
0.228881442	negative
0.228912181	negative
0.229006527	negative
0.22905637	negative
0.22905637	negative
0.22905637	negative
0.22905637	negative
0.229063875	negative
0.229063875	negative
0.229063875	negative
0.229063875	negative
0.229063875	negative
0.229063875	negative
0.229356019	negative
0.229661661	negative
0.229661661	negative
0.229862676	positive
0.229862676	negative
0.230190628	negative
0.230214485	negative
0.230222852	negative
0.230339567	negative
0.230645671	negative
0.230885151	positive
0.230969869	negative
0.231678094	negative
0.231717654	negative

0.231717654	negative
0.231750667	positive
0.232394441	negative
0.232407925	negative
0.232499762	negative
0.232531872	positive
0.23289032	negative
0.233259194	negative
0.233259194	negative
0.233455108	negative
0.233455108	negative
0.233620809	negative
0.233620809	negative
0.233892458	negative
0.233917185	negative
0.233917185	negative
0.233917185	negative
0.233917185	negative
0.233917185	negative
0.233917185	negative
0.233917185	negative
0.233917185	negative
0.233917185	negative
0.233917185	positive
0.233917185	negative
0.233917185	negative
0.233964581	negative
0.233964581	negative
0.234593649	positive
0.234593649	negative
0.234705387	negative
0.234787267	negative
0.234787267	negative
0.234801572	negative
0.234923137	negative
0.234923137	negative
0.235759738	negative

0.235945665	positive
0.235945665	negative
0.235945665	negative
0.235945665	negative
0.235945665	positive
0.235945665	positive
0.235945665	positive
0.235945665	negative
0.235945665	negative
0.235945665	negative
0.235945665	negative
0.235945665	negative
0.235945665	negative
0.235945665	negative
0.236207962	negative
0.236502193	negative
0.23654256	negative
0.236551249	negative
0.236551249	negative
0.236551249	negative
0.236551249	negative
0.236551249	negative
0.236551249	positive
0.236551249	positive
0.236551249	negative
0.237195187	negative
0.237195187	negative
0.237195187	positive
0.237437286	positive
0.237437286	negative
0.237437286	negative
0.237437286	negative
0.237437286	negative
0.237437286	negative
0.237607865	negative
0.237739158	negative
0.237739158	negative

0.237739158	negative
0.237739158	negative
0.237739158	negative
0.237739158	negative
0.2379844	negative
0.238224903	positive
0.238224903	negative
0.238965064	negative
0.238975712	negative
0.238975712	negative
0.238979936	negative
0.239295389	positive
0.239507848	negative
0.239507848	negative
0.239507848	negative
0.239756126	negative
0.240148001	negative
0.240148001	negative
0.240148001	negative
0.240148001	negative
0.240176719	positive
0.240176719	negative
0.240176719	negative
0.240176719	negative
0.240176719	negative
0.240176719	positive
0.240176719	negative
0.240342154	negative
0.240342154	negative
0.240342154	negative
0.240342154	negative
0.240342154	negative
0.240391064	positive
0.240391064	positive
0.240391064	negative
0.240391064	negative
0.240391064	negative

0.240391064	negative
0.240391064	negative
0.240693502	negative
0.240693502	negative
0.240693502	negative
0.240693502	negative
0.240693502	negative
0.240804525	positive
0.240890897	negative
0.240890897	positive
0.24112298	negative
0.241268365	negative
0.241268365	negative
0.241489393	negative
0.241489393	negative
0.241617031	negative
0.241617031	negative
0.241805758	negative
0.241810625	negative
0.241810625	negative
0.241810625	negative
0.241810625	negative
0.241810625	negative
0.241810625	negative
0.241810625	negative
0.241810625	positive
0.241810625	negative
0.241810625	negative
0.242052445	negative
0.242233085	positive
0.242738418	negative
0.24282556	negative
0.24282556	negative
0.24282556	positive
0.24282556	negative
0.24282556	negative
0.24282556	negative

0.243096551	negative
0.243249421	negative
0.243542636	positive
0.243690715	negative
0.243690715	negative
0.243690715	negative
0.243892261	positive
0.243892261	negative
0.243892261	negative
0.244022632	negative
0.244044848	negative
0.244243687	negative
0.244243687	negative
0.244287193	negative
0.244484989	positive
0.244583459	negative
0.244647496	negative
0.244701239	negative
0.244701239	negative
0.244701239	negative
0.244701239	negative
0.244701239	negative
0.244701239	negative
0.244701239	negative
0.244701239	negative
0.244701239	negative
0.244701239	negative
0.244701239	positive
0.244701239	negative
0.244701239	negative
0.244701239	negative
0.244701239	negative
0.244701239	negative
0.244701239	positive
0.244701239	negative
0.244845985	negative
0.245074985	positive

0.245253869	negative
0.245279333	negative
0.24544897	positive
0.245496898	negative
0.245655313	negative
0.245655313	negative
0.245655313	negative
0.245655313	negative
0.245898015	negative
0.245898015	negative
0.245999092	positive
0.245999092	positive
0.245999092	negative
0.246066343	negative
0.246150272	negative
0.246150272	negative
0.24642321	negative
0.246609122	negative
0.246609122	negative
0.246609122	positive
0.246609122	negative
0.246609122	negative
0.246609122	negative
0.246609122	negative
0.246609122	positive
0.246609122	negative
0.246609122	negative
0.246609122	negative
0.246609122	negative
0.246609122	negative
0.246609122	negative
0.246609122	negative
0.246609122	negative
0.246609122	negative
0.246675875	negative
0.246691938	negative
0.246691938	negative

0.246691938	negative
0.247077458	negative
0.247481016	negative
0.247481016	positive
0.247481016	negative
0.247494014	positive
0.247494014	negative
0.247603001	negative
0.24764235	negative
0.24764235	positive
0.247860765	negative
0.247860765	negative
0.247860765	negative
0.247860765	negative
0.248133833	negative
0.248133833	negative
0.248284114	positive
0.248284114	positive
0.248284114	positive
0.248609263	negative
0.248609263	negative
0.248839781	negative
0.248876106	positive
0.248876106	negative
0.248876106	negative
0.248876106	negative
0.248876106	negative
0.248876106	negative
0.248876106	negative
0.248876106	negative
0.248960239	negative
0.249260922	negative
0.249441776	negative
0.249441776	negative
0.249441776	negative
0.249441776	positive
0.249441776	negative

0.249441776	negative
0.249441776	negative
0.249441776	negative
0.249441776	positive
0.249441776	negative
0.249441776	negative
0.249441776	negative
0.249441776	negative
0.249441776	negative
0.249642955	negative
0.249642955	negative
0.249642955	negative
0.249642955	negative
0.249642955	negative
0.249642955	negative
0.249642955	positive
0.249642955	negative
0.249642955	negative
0.249642955	negative
0.249642955	negative
0.249642955	negative
0.249642955	negative
0.249652979	negative
0.249666532	negative
0.249666532	negative
0.249666532	negative
0.249666532	negative
0.249666532	negative
0.249666532	negative
0.249943938	positive
0.249991549	negative
0.250096617	positive
0.250096617	negative
0.250096617	negative
0.250096617	negative
0.250209595	negative
0.250209595	positive

0.250209595	negative
0.250209595	negative
0.250209595	negative
0.250209595	negative
0.25022231	negative
0.25022231	negative
0.250401613	negative
0.250598534	negative
0.250765781	negative
0.250993491	negative
0.250993491	negative
0.250993491	negative
0.251007341	negative
0.251036243	positive
0.251084983	negative
0.25115725	positive
0.251638641	positive
0.251794559	negative
0.251794559	negative
0.251794559	negative
0.251794559	positive
0.251794559	negative
0.251794559	negative
0.251794559	negative
0.251794559	negative
0.251794559	negative
0.251796776	negative
0.25217425	negative
0.252344857	negative
0.252404296	positive
0.252404296	negative
0.252431504	negative
0.252431504	negative
0.252431504	negative
0.252431504	negative
0.252431504	negative
0.252431504	negative

0.252431504	positive
0.252431504	negative
0.252431504	negative
0.252431504	negative
0.252431504	negative
0.252431504	negative
0.252431504	negative
0.252650238	negative
0.252698044	negative
0.253072916	negative
0.253072916	negative
0.25320378	negative
0.253557465	negative
0.25385998	negative
0.25385998	negative
0.25385998	negative
0.25404076	negative
0.254050185	negative
0.254116884	negative
0.254631117	negative
0.254631117	negative
0.254638371	negative
0.254638371	negative
0.25474505	negative
0.254809479	negative
0.254809479	negative
0.254809479	positive
0.254809479	negative
0.254809479	negative
0.254809479	negative
0.254809479	negative
0.254809479	negative
0.254809479	negative
0.254809479	positive
0.254809479	positive
0.254809479	positive
0.254809479	negative

0.254809479	positive
0.254809479	negative
0.254809479	negative
0.255126741	negative
0.255126741	negative
0.255157891	negative
0.255174432	negative
0.255742279	negative
0.255742279	negative
0.255787571	positive
0.255818453	negative
0.255818453	negative
0.255818453	negative
0.255818453	negative
0.255818453	negative
0.255837969	positive
0.255837969	negative
0.256001388	negative
0.256538237	negative
0.256538237	negative
0.256538237	negative
0.256538237	negative
0.256538237	negative
0.256538237	negative
0.256538237	negative
0.256538237	negative
0.256538237	negative
0.256538237	negative
0.256540656	negative
0.25673284	negative
0.25694127	negative
0.25694127	negative
0.257004423	negative
0.257004423	negative
0.257055687	negative
0.257451188	negative
0.25747674	negative

0.257665275	negative
0.257719095	positive
0.257719095	negative
0.257786261	negative
0.257786261	positive
0.258028602	negative
0.258028602	negative
0.258028602	negative
0.258028602	negative
0.258032812	negative
0.258168004	negative
0.258168004	negative
0.258390451	negative
0.258549741	negative
0.258549741	negative
0.258549741	positive
0.258549741	positive
0.258549741	positive
0.258549741	negative
0.258549741	negative
0.258549741	negative
0.258549741	negative
0.258549741	negative
0.258549741	negative
0.258549741	positive
0.258549741	negative
0.258549741	negative
0.258549741	negative
0.258549741	negative
0.258549741	negative
0.258549741	negative
0.258549741	negative
0.258549741	negative
0.258853371	negative
0.258853371	negative
0.258853371	negative
0.258853371	negative

0.258853371	negative
0.258952334	negative
0.258952334	negative
0.258952334	negative
0.258952334	negative
0.258952334	positive
0.258952334	negative
0.258952334	negative
0.258952334	negative
0.259134439	negative
0.259134439	negative
0.259134439	negative
0.25917682	negative
0.259186659	negative
0.259186659	negative
0.259186659	negative
0.259186659	positive
0.259186659	negative
0.259193867	negative
0.259193867	negative
0.259300573	negative
0.259769757	negative
0.259773828	positive
0.259773828	negative
0.259773828	negative
0.260131354	negative
0.260149558	negative
0.260149558	negative
0.260472093	negative
0.260472093	negative
0.260472093	negative
0.260476172	negative
0.260511448	negative
0.260791819	negative
0.260791819	negative
0.260791819	negative
0.260791819	negative

0.260791819	positive
0.260791819	negative
0.260791819	negative
0.260791819	negative
0.260791819	positive
0.260904309	negative
0.260904309	negative
0.260904309	negative

Table S5. GO and pathway enrichment analysis based on the significant genes selected from SRC analysis and ANOVA

ID	Description	GeneNumber
GO:0040008	regulation of growth	21
GO:0048638	regulation of developmental growth	14
GO:0001558	regulation of cell growth	15
GO:0061643	chemorepulsion of axon	3
GO:0045926	negative regulation of growth	9
GO:0030516	regulation of axon extension	6
GO:0045596	negative regulation of cell differentiation	15
GO:0061387	regulation of extent of cell growth	6
GO:0048640	negative regulation of developmental growth	6
GO:0051960	regulation of nervous system development	11
GO:0030517	negative regulation of axon extension	4
GO:0030308	negative regulation of cell growth	7
GO:0050919	negative chemotaxis	4
GO:0007167	enzyme-linked receptor protein signaling pathway	18
GO:0071363	cellular response to growth factor stimulus	14
GO:0070848	response to growth factor	14
GO:0007169	transmembrane receptor protein tyrosine kinase signalir	11
GO:0010035	response to inorganic substance	16
GO:0071248	cellular response to metal ion	7
GO:0046890	regulation of lipid biosynthetic process	9
GO:0090207	regulation of triglyceride metabolic process	5
GO:1901615	organic hydroxy compound metabolic process	13
GO:0034377	plasma lipoprotein particle assembly	4
GO:0033344	cholesterol efflux	4
GO:0065005	protein-lipid complex assembly	4
GO:0030301	cholesterol transport	5
GO:0033700	phospholipid efflux	3
GO:0019216	regulation of lipid metabolic process	10
GO:0062012	regulation of small molecule metabolic process	10

GO:0016042	lipid catabolic process	9
GO:0046486	glycerolipid metabolic process	10
GO:0010896	regulation of triglyceride catabolic process	3
GO:0015918	sterol transport	5
GO:0034375	high-density lipoprotein particle remodeling	3
GO:0055090	acylglycerol homeostasis	4
GO:0070328	triglyceride homeostasis	4
GO:0071827	plasma lipoprotein particle organization	4
GO:0043691	reverse cholesterol transport	3
GO:0006066	alcohol metabolic process	9
GO:0010565	regulation of cellular ketone metabolic process	6
GO:0071825	protein-lipid complex subunit organization	4
GO:0042304	regulation of fatty acid biosynthetic process	4
GO:0051004	regulation of lipoprotein lipase activity	3
GO:0120009	intermembrane lipid transfer	4
GO:0019217	regulation of fatty acid metabolic process	5
GO:0061061	muscle structure development	14
GO:0060537	muscle tissue development	9
GO:0048880	sensory system development	12
GO:0007423	sensory organ development	14
GO:0001654	eye development	11
GO:0150063	visual system development	11
GO:0043010	camera-type eye development	10
GO:0031667	response to nutrient levels	13
GO:0009991	response to extracellular stimulus	13
GO:0031669	cellular response to nutrient levels	8
GO:0031668	cellular response to extracellular stimulus	8
GO:0042594	response to starvation	7
GO:0070482	response to oxygen levels	11
GO:0001666	response to hypoxia	10
GO:0036293	response to decreased oxygen levels	10
GO:0061564	axon development	12
GO:0007409	axonogenesis	11

GO:0048667	cell morphogenesis involved in neuron differentiation	12
GO:0060322	head development	17
GO:0000904	cell morphogenesis involved in differentiation	13
GO:0007420	brain development	15
GO:0032989	cellular component morphogenesis	13
GO:0000902	cell morphogenesis	14
GO:0030900	forebrain development	10
GO:0048812	neuron projection morphogenesis	11
GO:0120039	plasma membrane bounded cell projection morphogene	11
GO:0048858	cell projection morphogenesis	11
GO:0031175	neuron projection development	13
GO:0032990	cell part morphogenesis	11
GO:0009750	response to fructose	3
GO:0048732	gland development	10
GO:0000302	response to reactive oxygen species	8
GO:0009725	response to hormone	16
GO:1901652	response to peptide	12
GO:0032868	response to insulin	8
GO:0032870	cellular response to hormone stimulus	12
GO:1900103	positive regulation of endoplasmic reticulum unfolded [illegible]	3
GO:0043434	response to peptide hormone	10
GO:0033500	carbohydrate homeostasis	7
GO:0042593	glucose homeostasis	7
GO:0008217	regulation of blood pressure	8
GO:0045776	negative regulation of blood pressure	4
GO:0003085	negative regulation of systemic arterial blood pressure	3
GO:0060541	respiratory system development	8
GO:0060411	cardiac septum morphogenesis	5
GO:0030324	lung development	7
GO:0003151	outflow tract morphogenesis	5
GO:0003206	cardiac chamber morphogenesis	6
GO:0030323	respiratory tube development	7
GO:0007507	heart development	12

GO:2000136	regulation of cell proliferation involved in heart morpho	3
GO:0003197	endocardial cushion development	4
GO:0003177	pulmonary valve development	3
GO:0051963	regulation of synapse assembly	6
GO:0042752	regulation of circadian rhythm	6
GO:0042743	hydrogen peroxide metabolic process	4
GO:0042627	chylomicron	3
GO:0034361	very-low-density lipoprotein particle	3
GO:0034385	triglyceride-rich plasma lipoprotein particle	3
GO:0120020	cholesterol transfer activity	4
GO:0120015	sterol transfer activity	4
GO:0015485	cholesterol binding	5
GO:0032934	sterol binding	5
GO:0015248	sterol transporter activity	4
GO:0043178	alcohol binding	5
GO:0120013	lipid transfer activity	4
hsa04020	Calcium signaling pathway	11
hsa04979	Cholesterol metabolism	4
hsa04919	Thyroid hormone signaling pathway	7
hsa03320	PPAR signaling pathway	5

Table S6. Related reports of genes

Gene	High presented in	Species
APOA1	HS	Human
APOC3	HS	Human & Mouse
APOA4	HS	Human & Mouse
GSTA3	Con	Rat
CYP1A2	Con	Human
FGF19	Con	Human & Mouse
TGIF1	Con	Human & Mouse
FTH1	Con	Mouse
CYP1A1	Con	Mouse
IFRD1 (TIS7)	Con	Mouse
ETV5	Con	Mouse
KHK	HS	Human & Mouse
UCHL1	Con	Human
IFT140	HS	Human
DIO2	Con	Mouse
RBP	Con	Rat
SIK1	Con	Rat
ECHDC1	HS	Human
STARD5	Con	Rat
PYGO1	Con	Human
MFSD2A	HS	Mouse
PTPN2	Con	Human & Mouse
EZH2	HS	Human & Mouse
OTULIN	HS	Human & Mouse
ISM1	Con	Mouse
CRY1	Con	Mouse
JAG1	Con	Mouse
WNT5A	HS	Human
PCK1	HS	Human & Mouse

ITCH	Con	Human & Mouse
IL15	HS	Mouse
NR2F6	Con	Human & Mouse
XBP1	Con	Human & Mouse
COLEC10	Con	Human
TPX2	HS	Human
NAPEPLD	Con	Mouse
SLITRK3	Con	Human
ACOT13	HS	Mouse
HBA1	HS	Human
SLC16A10	Con	Rat
UCP3	HS	Human
PEMT	HS	Mouse
SREBF1	Con	Human & Mouse
VWA5B2	HS	Fish
MT3	Con	Human
TM6SF1	Con	Mouse
DPP4	HS	Mouse
TM6SF2	NS	Mouse

s involved in hepatic steatosis, NAFLD, c

Related reports in References
ApoA1 is highly associated with HDL-cholesterol and a negative association was also expected with steatosis
Some SNPs in APOC3 have been reported to be risk factor for NAFLD. Increase plasma APOC3 concentrations predispose to
APOA4 is closely linked to obesity, type 2 diabetes and hepatic steatosis
GSTA3 inhibited HSCs activation and liver fibrosis through suppression of the MAPK and GSK-3β signaling pathways
Decreased CYP1A2 activity has been associated with NAFLD progression
FGF19 protects from hepatic steatosis
High expression of TGIF1 have positive effect on lipid metabolism
TFR1 and FTH1 paly vital roles in hepatic iron metabolism, and may associated with NAFLD
CYP1A1 plays a protective role against NAFLD
Tis7 may be a regulator of lipid absorption and metabolism
ETV5 could regulate hepatic fatty acid metabolism
KHK inhibition to improve steatosis, fibrosis, and inflammation in the context of NAFLD
UCHL1 appears to be a functional tumor suppressor involved in the tumorigenesis of HCCs and other digestive cancers
IFI140 is a potential novel candidate BDM biomarker for liver fibrosis in NAFLD patients
Loss of the Dio2 gene results in increased fat storage in adipose tissue and hepatic steatosis
Lower expression of RBP may improve hepatic steatosis
Upregulating hepatic SIK1 may represent an efficient strategy for hepatic steatosis
ECHDC1 may be involved in the occurrence and development of NAFLD by regulating hepatic lipid metabolism
STARD5 modulate hepatic neutral lipid metabolism
PYGO1 is a novel steatosis modifier
Loss of Mfsd2a have normal liver lipid metabolism
liver-specific PTPN2 deficiency promotes hepatic steatosis, obesity and insulin resistance
aberrant activation of EZH2 promotes NAFLD progression
OTULIN prevents development of liver disease
ISM1 suppresses hepatocyte lipid synthesis
CRY1 may attenuates hyperglycaemia and liver steatosis
hepatocyte-specific Jag1 knockout mice were protected from NASH-induced liver fibrosis
WNT5A may promote liver damage
Loss of Pck1 gene coule develop hepatic steatosis

Loss of ITCH result in hepatic steatosis

Absence of IL-15 or IL-15Rα protects from NAFLD

NR2F6 contributes to liver steatosis

XBP1 contributes to ER stress-induced hepatic steatosis

COLEC10 may be a biomarker of HCC

TPX2 may suppress the growth of HCC

Loss of ITCH result in hepatic steatosis

SLITRK3 (SLIT and NTRK like family member 3) was down-regulated in steatosis and NASH patients

Acot13 could regulate hepatic lipid and glucose metabolism

serum HbA1c level is associated with NAFLD

Slc16a10 is a transport carrier of aromatic amino acids, it up-regulated in NAFLD group

UCP3 gene variants were associated with protection against NASH

PEMT deficiency promotes NAFLD progression

raise SREBF1 expression promotes the development of hepatic steatosis

upregulated in fish livers caused by hypoxia

MT3 may be a potential intervention for hepatic steatosis by inhibit the generation of reactive oxygen species (ROS)

Tm6sf1 is a widely expressed lysosomal transmembrane protein

DPP-4 inhibition has extrapancreatic protective effects against diet-induced adipose tissue inflammation and hepatic steatosis

Liver-Specific Deletion of Mouse Tm6sf2 Promotes Steatosis

Reference

Consumption of Spinach and Tomato Modifies Lipid Metabolism, Reducing Hepatic Steatosis in Rats

APOC3 rs2070666 is Associated with the Hepatic Steatosis Independently of PNPLA3 rs738409 in Chinese Han Patients with Nonalcoholic Fatty Liver Diseases;

Apolipoprotein A-IV expression in mouse liver enhances triglyceride secretion and reduces hepatic lipid content by promoting very low density lipoprotein particle

A novel role of glutathione S-transferase A3 in inhibiting hepatic stellate cell activation and rat hepatic fibrosis

Hepatic cytochrome P450 enzyme alterations in humans with progressive stages of nonalcoholic fatty liver disease;

Fibroblast growth factor 15/19 (FGF15/19) protects from diet-induced hepatic steatosis: development of an FGF19-based chimeric molecule to promote fatty liver

Effects on hepatic lipid metabolism in human hepatoma cells following overexpression of TGFβ induced factor homeobox 1 or 2

Study on the attenuated effect of Ginkgolide B on ferroptosis in high fat diet induced nonalcoholic fatty liver disease;

Cytochrome P450 1A1 (CYP1A1) protects against nonalcoholic fatty liver disease caused by Western diet containing benzo[a]pyrene in mice

Deletion of Tis7 protects mice from high-fat diet-induced weight gain and blunts the intestinal adaptive response postresection

ETV5 Regulates Hepatic Fatty Acid Metabolism Through PPAR Signaling Pathway.

Ketohexokinase inhibition improves NASH by reducing fructose-induced steatosis and fibrogenesis;

Epigenetic identification of ubiquitin carboxyl-terminal hydrolase L1 as a functional tumor suppressor and biomarker for hepatocellular carcinoma and other digestive

Potential Blood DNA Methylation Biomarker Genes for Diagnosis of Liver Fibrosis in Patients With Biopsy-Proven Non-alcoholic Fatty Liver Disease

Mice with a Targeted Deletion of the Type 2 Deiodinase Are Insulin Resistant and Susceptible to Diet Induced Obesity

Effects of Hesperidin on the Progression of Hypercholesterolemia and Fatty Liver Induced by High-Cholesterol Diet in Rats;

Zhenqing recipe attenuates non-alcoholic fatty liver disease by regulating the SIK1/CRTC2 signaling in experimental diabetic rats

Transcriptomic analysis reveals a novel regulatory factor of ECHDC1 involved in lipid metabolism of non-alcoholic fatty liver disease

Cytosolic lipid trafficking proteins STARD4 and STARD5 modulate hepatic neutral lipid metabolism: implications for diabetic dyslipidaemia and steatosis

Genome-wide association study of non-alcoholic fatty liver and steatohepatitis in a histologically characterised cohort

Major Facilitator Superfamily Domain-Containing Protein 2a (MFSD2A) Has Roles in Body Growth, Motor Function, and Lipid Metabolism

Hepatic oxidative stress promotes insulin-STAT-5 signaling and obesity by inactivating protein tyrosine phosphatase N2;

EZH2 as a Potential Target for NAFLD Therapy

OTULIN protects the liver against cell death, inflammation, fibrosis, and cancer

Isthmin-1 is an adipokine that promotes glucose uptake and improves glucose tolerance and hepatic steatosis

DDB1 E3 ligase controls dietary fructose-induced ChREBPα stabilization and liver steatosis via CRY1

Hepatocyte TLR4 triggers inter-hepatocyte Jagged1/Notch signaling to determine NASH-induced fibrosis

Deregulation of Secreted Frizzled-Related Protein 5 in Nonalcoholic Fatty Liver Disease Associated with Obesity

Disregulated glyceroneogenesis: *PCK1* as a candidate diabetes and obesity gene

ITCH E3 ubiquitin ligase downregulation compromises hepatic degradation of branched-chain amino acids

Interleukin-15-mediated inflammation promotes non-alcoholic fatty liver disease

The Nuclear Orphan Receptor NR2F6 Promotes Hepatic Steatosis through Upregulation of Fatty Acid Transporter CD36

Deacetylation of XBP1s by sirtuin 6 confers resistance to ER stress-induced hepatic steatosis

Transcriptional Landscape of Hepatocellular Carcinoma Reveals that Patient Ethnic-Origin Influences Patterns of Expression

TPX2 silencing exerts anti‑tumor effects on hepatocellular carcinoma by regulating the PI3K/AKT signaling pathway

Hepatic NAPE-PLD Is a Key Regulator of Liver Lipid Metabolism

Integrated Multichip Analysis Identifies Potential Key Genes in the Pathogenesis of Nonalcoholic Steatohepatitis

Thioesterase superfamily member 2/acyl-CoA thioesterase 13 (Them2/Acot13) regulates hepatic lipid and glucose metabolism

Independent association of HbA1c and nonalcoholic fatty liver disease in an elderly Chinese population

Combined Transcriptomic and Lipidomic Analysis Reveals Dysregulated Genes Expression and Lipid Metabolism Profiles in the Early Stage of Fatty Liver Disease in

Association of *UCP3* Polymorphisms with Nonalcoholic Steatohepatitis and Metabolic Syndrome in Nonalcoholic Fatty Liver Disease Brazilian Patients

Hepatic PEMT activity mediates liver health, weight gain, and insulin resistance

Downregulation of miR-192 causes hepatic steatosis and lipid accumulation by inducing SREBF1: Novel mechanism for bisphenol A-triggered non-alcoholic fatty

Hypoxia causes sex-specific hepatic toxicity at the transcriptome level in marine medaka (Oryzias melastigma)

Melatonin: a potential intervention for hepatic steatosis

Transmembrane 6 superfamily 1 (Tm6sf1) is a novel lysosomal transmembrane protein

Diet-Induced Adipose Tissue Inflammation and Liver Steatosis Are Prevented by DPP-4 Inhibition in Diabetic Mice

Liver-Specific Deletion of Mouse Tm6sf2 Promotes Steatosis, Fibrosis, and Hepatocellular Cancer

Table S7. Differential expression genes deteced from eHSC groups

gene id	gene name	Chromosome	p	p_FDR
ENSGALG00000050577	GRPEL2	13	2.31656E-44	9.41527E-41
ENSGALG00000014329	FOXJ2	1	7.17203E-44	2.18622E-40
ENSGALG00000005412	SNAP47	2	1.21757E-43	2.96917E-40
ENSGALG00000004098	STK4	20	1.91358E-43	3.88871E-40
ENSGALG00000000523	STK38	26	1.16241E-42	1.81188E-39
ENSGALG00000012720	NUP153	2	1.27988E-42	1.81188E-39
ENSGALG00000008097	CELF1	5	1.3374E-42	1.81188E-39
ENSGALG00000048138		11	4.32764E-42	4.83016E-39
ENSGALG00000039294	MSL2	9	4.35756E-42	4.83016E-39
ENSGALG00000054991		22	5.36962E-42	5.4557E-39
ENSGALG00000006697	TOLLIP	5	5.81679E-42	5.4557E-39
ENSGALG00000054086		14	7.86533E-42	6.85014E-39
ENSGALG00000008936		17	8.4356E-42	6.85702E-39
ENSGALG00000002489	PPP1R26	17	1.0989E-41	8.37434E-39
ENSGALG00000001950	PMPCA	17	1.4318E-41	1.02694E-38
ENSGALG00000005475		4	2.08327E-41	1.41119E-38
ENSGALG00000001600	DVL1	21	5.09203E-41	3.26775E-38
ENSGALG00000005856	TBC1D9B	13	5.95227E-41	3.6288E-38
ENSGALG00000012356	TAB2	3	8.69316E-41	5.04742E-38
ENSGALG00000008611	ZBTB33	4	1.22384E-40	6.78283E-38
ENSGALG00000004965	SLC27A4	17	1.47349E-40	7.81141E-38
ENSGALG00000004769	PSAP	6	1.7606E-40	8.9446E-38
ENSGALG00000016971		1	1.93486E-40	9.40963E-38
ENSGALG00000050605		9	2.00648E-40	9.40963E-38
ENSGALG00000033889	NAA50	1	2.1327E-40	9.63112E-38
ENSGALG00000008494	CREB1	7	2.25179E-40	9.80575E-38
ENSGALG00000047988		17	2.99318E-40	1.25848E-37
ENSGALG00000009338	ZFYVE1	5	3.72828E-40	1.5153E-37

ENSGALG00000016792	MFSD9	1	4.54024E-40	1.78578E-37
ENSGALG00000013628	C6orf62	2	4.79824E-40	1.82828E-37
ENSGALG00000001043	REXO1	28	9.71202E-40	3.58844E-37
ENSGALG00000008496	VPS18	5	1.0354E-39	3.71313E-37
ENSGALG00000021601	EPS15L1	28	1.57253E-39	5.39178E-37
ENSGALG00000012449	MBD5	7	1.59193E-39	5.39178E-37
ENSGALG00000001733	LRIG2	26	1.79699E-39	5.92181E-37
ENSGALG00000011863	POLDIP3	1	2.18077E-39	6.99739E-37
ENSGALG00000008653	MGA	5	2.38966E-39	7.47107E-37
ENSGALG00000005674	USP10	11	6.1546E-39	1.85581E-36
ENSGALG00000011878	TCF20	1	6.36564E-39	1.85581E-36
ENSGALG00000040844	GIT2	15	6.53579E-39	1.85581E-36
ENSGALG00000025904	WDR82	12	6.54474E-39	1.85581E-36
ENSGALG00000038926		2	7.12711E-39	1.97502E-36
ENSGALG00000030569	TRIM41	16	9.08853E-39	2.46259E-36
ENSGALG00000027132		28	9.61313E-39	2.54811E-36
ENSGALG00000008420	CAND2	12	1.02048E-38	2.64739E-36
ENSGALG00000010273	PPP2CB	4	1.04879E-38	2.66415E-36
ENSGALG00000000848	AP1G1	11	1.07784E-38	2.68206E-36
ENSGALG00000024082	SMCR8	14	1.13015E-38	2.71309E-36
ENSGALG00000039658	GGA1	1	1.13481E-38	2.71309E-36
ENSGALG00000015409	PROS1	1	1.24503E-38	2.91936E-36
ENSGALG00000022882	ZADH2	2	1.30423E-38	3.00047E-36
ENSGALG00000002863	ERGIC1	13	1.33402E-38	3.01217E-36
ENSGALG00000008023	TMEM94	18	1.40844E-38	3.12238E-36
ENSGALG00000006750		10	1.66377E-38	3.62257E-36
ENSGALG00000035211	KLHL21	21	1.94956E-38	4.17035E-36
ENSGALG00000015334	ZBTB11	1	1.99691E-38	4.19799E-36
ENSGALG00000012453	EPC2	7	2.11211E-38	4.3649E-36
ENSGALG00000041884	MFSD6	7	2.24392E-38	4.54531E-36
ENSGALG00000003358	UBIAD1	21	2.27396E-38	4.54531E-36
ENSGALG00000001099	ANKHD1	13	2.46193E-38	4.84166E-36
ENSGALG00000008122	SLC39A13	5	2.74129E-38	5.25276E-36

ENSGALG00000046769		2	2.75713E-38	5.25276E-36
ENSGALG00000014643		25	2.83497E-38	5.31797E-36
ENSGALG00000004667	SMG7	8	3.32948E-38	6.15096E-36
ENSGALG00000037597	YOD1	26	3.67276E-38	6.68387E-36
ENSGALG00000002139	CSDE1	26	4.28899E-38	7.69053E-36
ENSGALG00000031282	UBR5	2	5.01598E-38	8.86375E-36
ENSGALG00000042725	ZC4H2	4	5.20414E-38	9.06487E-36
ENSGALG00000000955	TMEM222	23	5.55956E-38	9.54756E-36
ENSGALG00000001420	RALGAPB	20	5.79387E-38	9.81176E-36
ENSGALG00000003875	ABL1	17	6.85405E-38	1.14481E-35
ENSGALG00000008026	SPATA2	20	7.07515E-38	1.16577E-35
ENSGALG00000008358	AMBRA1	5	7.30146E-38	1.18702E-35
ENSGALG00000031681	M6PR	1	1.01936E-37	1.6354E-35
ENSGALG00000003220	UPF1	28	1.07362E-37	1.66079E-35
ENSGALG00000031416	UBN1	14	1.07613E-37	1.66079E-35
ENSGALG00000044014		2	1.08121E-37	1.66079E-35
ENSGALG00000012037	ARID4A	5	1.08967E-37	1.66079E-35
ENSGALG00000009192	SLITRK2	4	1.11514E-37	1.67863E-35
ENSGALG00000007330	CTNND1	5	1.45223E-37	2.1594E-35
ENSGALG00000053997		7	1.54775E-37	2.27371E-35
ENSGALG00000007291		14	1.65314E-37	2.39962E-35
ENSGALG00000035669	ADNP2	2	1.79911E-37	2.58077E-35
ENSGALG00000001522	PHF20	20	1.94836E-37	2.76237E-35
ENSGALG00000015904	TMEM30A	3	2.06069E-37	2.88805E-35
ENSGALG00000001860	RSBN1	26	2.56375E-37	3.55225E-35
ENSGALG00000033521	FASTKD3	2	2.7964E-37	3.83106E-35
ENSGALG00000012489	KLHL28	5	3.0004E-37	4.06487E-35
ENSGALG00000042088	ATG2B	5	3.35857E-37	4.50012E-35
ENSGALG00000009835	XPOT	1	3.48416E-37	4.61764E-35
ENSGALG00000006829	SMG1	14	3.58077E-37	4.67358E-35
ENSGALG00000011329	AFDN	3	3.60302E-37	4.67358E-35
ENSGALG00000011992	EP300	1	4.34276E-37	5.57381E-35
ENSGALG00000008314		12	4.51039E-37	5.72867E-35

ENSGALG00000013635	SCAF8	3	4.84343E-37	6.08825E-35
ENSGALG00000007169	EPC1	2	5.05952E-37	6.29458E-35
ENSGALG00000006426	PAK2	9	5.11083E-37	6.29458E-35
ENSGALG00000042883	VCPIP1	2	5.19215E-37	6.33078E-35
ENSGALG00000006880	SLC38A10	18	5.48179E-37	6.61777E-35
ENSGALG00000047359	MAF1	2	5.58539E-37	6.65371E-35
ENSGALG00000007481	HACD3	10	5.6207E-37	6.65371E-35
ENSGALG00000047673		22	5.85911E-37	6.86925E-35
ENSGALG00000040109	LARP1	13	6.20443E-37	7.10841E-35
ENSGALG00000031303	FAM234A	14	6.2297E-37	7.10841E-35
ENSGALG00000004853	LRP3	11	6.23801E-37	7.10841E-35
ENSGALG00000031296		9	6.57833E-37	7.42681E-35
ENSGALG00000000875	PHLPP2	11	7.93906E-37	8.88082E-35
ENSGALG00000012888	PHLPP1	2	8.0594E-37	8.93348E-35
ENSGALG00000012386	LATS1	3	9.26162E-37	1.00897E-34
ENSGALG00000001377	POLRMT	28	9.26803E-37	1.00897E-34
ENSGALG00000036191		31	9.77355E-37	1.05459E-34
ENSGALG00000006261	SELENOF	8	1.00666E-36	1.07669E-34
ENSGALG00000002172	FAM168B	9	1.02157E-36	1.08313E-34
ENSGALG00000005708	HNF4beta	11	1.16919E-36	1.22896E-34
ENSGALG00000006201	TBC1D20	20	1.27944E-36	1.33335E-34
ENSGALG00000033954	TMEM248	19	1.31485E-36	1.35864E-34
ENSGALG00000034628	CCDC127	2	1.36463E-36	1.3936E-34
ENSGALG00000012986	KDM5A	1	1.37154E-36	1.3936E-34
ENSGALG00000007986	MOCS3	20	1.57169E-36	1.58377E-34
ENSGALG00000040035	ADRB2	13	1.82633E-36	1.82528E-34
ENSGALG00000010051	MOCS1	3	1.9404E-36	1.92352E-34
ENSGALG00000000838	THAP12	1	2.07304E-36	2.03276E-34
ENSGALG00000002726	KIF1B	21	2.09992E-36	2.03276E-34
ENSGALG00000011101	URB2	3	2.10062E-36	2.03276E-34
ENSGALG00000041790	SAMD4A	5	2.20766E-36	2.11953E-34
ENSGALG00000048829	CLPTM1	24	2.26748E-36	2.15995E-34
ENSGALG00000034250	HS6ST2	4	2.3189E-36	2.19181E-34

ENSGALG00000040607	MED23	3	2.3917E-36	2.24323E-34
ENSGALG00000003950	CNOT8	13	2.46575E-36	2.29503E-34
ENSGALG00000027591	TSC22D2	9	2.63104E-36	2.43033E-34
ENSGALG00000004168	FEM1A	28	2.70536E-36	2.48018E-34
ENSGALG00000045642	TNFRSF11A	2	2.84451E-36	2.58829E-34
ENSGALG00000016267	PHF3	3	2.92637E-36	2.64306E-34
ENSGALG00000003092	ERLIN2	22	2.99331E-36	2.67198E-34
ENSGALG00000014936	ESCO1	2	3.00223E-36	2.67198E-34
ENSGALG00000006411	CAMLG	13	3.12033E-36	2.75093E-34
ENSGALG00000002969	MAU2	28	3.13606E-36	2.75093E-34
ENSGALG00000015306	OSTM1	3	3.47301E-36	3.02475E-34
ENSGALG00000037837	ABCA3	14	3.78304E-36	3.23346E-34
ENSGALG00000014638	ASH1L	25	3.79279E-36	3.23346E-34
ENSGALG00000013853	AFG3L2	2	3.81357E-36	3.23346E-34
ENSGALG00000023256	RNF8	3	3.81874E-36	3.23346E-34
ENSGALG00000002656	CRK	19	4.25411E-36	3.55375E-34
ENSGALG00000006390	CRKL	15	4.27248E-36	3.55375E-34
ENSGALG00000009443	TMEM168	1	4.30665E-36	3.55375E-34
ENSGALG00000003771	SIN3B	28	4.31358E-36	3.55375E-34
ENSGALG00000031589	ZNF592	10	4.4684E-36	3.65659E-34
ENSGALG00000016853	ABHD13	1	4.53652E-36	3.68759E-34
ENSGALG00000037739	STK25	9	4.66298E-36	3.74219E-34
ENSGALG00000012852	MKRN4P	1	4.69148E-36	3.74219E-34
ENSGALG00000001275	PDCL	17	4.69577E-36	3.74219E-34
ENSGALG00000009946	HECTD1	5	5.37901E-36	4.25885E-34
ENSGALG00000010766	ATXN3L	5	5.80044E-36	4.56289E-34
ENSGALG00000037546	SRPK1	26	6.42691E-36	5.02329E-34
ENSGALG00000009502	ATE1	6	6.63821E-36	5.15539E-34
ENSGALG00000049668	MFSD4B	3	6.93061E-36	5.34841E-34
ENSGALG00000003596	TRRAP	14	7.0938E-36	5.43992E-34
ENSGALG00000010026	PPP1CB	3	7.22151E-36	5.50325E-34
ENSGALG00000033614	ATF1	33	7.69916E-36	5.79583E-34
ENSGALG00000016236		1	7.70232E-36	5.79583E-34

ENSGALG00000011548	PPP1R13B	5	7.74806E-36	5.79583E-34
ENSGALG00000039472	SART3	15	8.89856E-36	6.61586E-34
ENSGALG00000032734	RNF139	2	9.80224E-36	7.24356E-34
ENSGALG00000014656	RPRD2	25	1.00468E-35	7.37933E-34
ENSGALG00000053889		9	1.0107E-35	7.37933E-34
ENSGALG00000052354		1	1.03687E-35	7.52536E-34
ENSGALG00000016053	DYRK1A	1	1.05959E-35	7.64472E-34
ENSGALG00000034948	UAP1L1	17	1.10729E-35	7.94188E-34
ENSGALG00000010390	CIPC	5	1.11651E-35	7.96117E-34
ENSGALG00000017376		8	1.14609E-35	8.12461E-34
ENSGALG00000010734	PPP4R3A	5	1.19478E-35	8.42081E-34
ENSGALG00000049372		2	1.26007E-35	8.80277E-34
ENSGALG00000004040	MFAP3	13	1.26342E-35	8.80277E-34
ENSGALG00000008624	RPAP1	5	1.32058E-35	9.14878E-34
ENSGALG00000007575		20	1.35923E-35	9.36335E-34
ENSGALG00000014659	C1R	1	1.37618E-35	9.42682E-34
ENSGALG00000015958	EIF2AK3	4	1.42097E-35	9.67929E-34
ENSGALG00000001003		23	1.56056E-35	1.0571E-33
ENSGALG00000003641	KAT6A	22	1.74395E-35	1.17481E-33
ENSGALG00000005181		6	1.75434E-35	1.17531E-33
ENSGALG00000034528	SNTB1	2	1.79891E-35	1.19859E-33
ENSGALG00000005158	WDFY1	9	1.893E-35	1.25442E-33
ENSGALG00000003955	CEP350	8	1.97845E-35	1.30396E-33
ENSGALG00000016630	EXTL3	3	2.02E-35	1.32419E-33
ENSGALG00000017026	VPS36	1	2.09224E-35	1.36421E-33
ENSGALG00000004533	KIAA2013	21	2.23881E-35	1.45201E-33
ENSGALG00000007994	PARD6B	20	2.29777E-35	1.48237E-33
ENSGALG00000012098	NAA30	5	2.41546E-35	1.55009E-33
ENSGALG00000014267	RBM47	4	2.42986E-35	1.55117E-33
ENSGALG00000009409		9	2.50273E-35	1.58936E-33
ENSGALG00000003765	A1CF	6	2.85435E-35	1.80191E-33
ENSGALG00000013824	HECA	3	2.86698E-35	1.80191E-33
ENSGALG00000014083	INTS13	1	3.14143E-35	1.96428E-33

ENSGALG00000033675	BAHD1	5	3.1691E-35	1.97147E-33
ENSGALG00000008545	SENP5	9	3.34043E-35	2.06751E-33
ENSGALG00000042598	PUM1	23	3.40777E-35	2.09853E-33
ENSGALG00000038733	TRAK1	2	3.76826E-35	2.30886E-33
ENSGALG00000005138	KCTD10	15	3.80117E-35	2.31738E-33
ENSGALG00000010661	CDKN2AIP	4	4.1176E-35	2.49781E-33
ENSGALG00000007547	ABI1	2	4.14643E-35	2.50284E-33
ENSGALG00000011098	TAF5L	3	4.19072E-35	2.51711E-33
ENSGALG00000036101	ARHGAP35	32	4.53018E-35	2.70767E-33
ENSGALG00000016915	MYCBP2	1	4.61296E-35	2.7437E-33
ENSGALG00000037455	PBRM1	12	4.66528E-35	2.76135E-33
ENSGALG00000004255	SNX8	14	4.74768E-35	2.79654E-33
ENSGALG00000016284	TAB3	1	5.28118E-35	3.09584E-33
ENSGALG00000001647	PSKH1	11	5.33724E-35	3.11373E-33
ENSGALG00000016531	ASXL2	3	5.54155E-35	3.21753E-33
ENSGALG00000001758	QSOX2	17	5.63671E-35	3.25727E-33
ENSGALG00000002117	PARG	6	6.26687E-35	3.60434E-33
ENSGALG00000011833	SPATA5	4	6.5641E-35	3.75756E-33
ENSGALG00000014012	WASF2	23	6.59515E-35	3.7577E-33
ENSGALG00000003536	KLHL11	27	6.74612E-35	3.82584E-33
ENSGALG00000005716	SBF2	5	7.08328E-35	3.99845E-33
ENSGALG00000005107		6	7.38985E-35	4.15088E-33
ENSGALG00000035281	WAC	2	7.4214E-35	4.15088E-33
ENSGALG00000010084	EIF4G3	21	7.65281E-35	4.26076E-33
ENSGALG00000001939	HIPK1	26	9.22186E-35	5.111E-33
ENSGALG00000007663	KDM3B	13	9.2657E-35	5.11207E-33
ENSGALG00000040969	PTP4A3	2	1.20922E-34	6.64147E-33
ENSGALG00000043827	ZHX2	2	1.31292E-34	7.16315E-33
ENSGALG00000001157	DENND1A	17	1.31845E-34	7.16315E-33
ENSGALG00000008081	PIK3CG	1	1.32183E-34	7.16315E-33
ENSGALG00000012059	OSBPL11	7	1.41468E-34	7.63238E-33
ENSGALG00000005350		19	1.46582E-34	7.87346E-33
ENSGALG00000007541	STX16	20	1.48413E-34	7.93682E-33

ENSGALG00000027665	SYNGR1	1	1.50528E-34	8.01482E-33
ENSGALG00000029616	HCFC2	1	1.56631E-34	8.3035E-33
ENSGALG00000029295	AZIN1	2	1.64555E-34	8.68582E-33
ENSGALG00000012705	GMPR2	2	1.65676E-34	8.70729E-33
ENSGALG00000011642	ANKRD17	4	1.71633E-34	8.98165E-33
ENSGALG00000020741	HPS6	6	1.72937E-34	9.01119E-33
ENSGALG00000001262	CLIC4	23	1.81424E-34	9.3788E-33
ENSGALG00000013532	CAPRIN1	5	1.8153E-34	9.3788E-33
ENSGALG00000054686	PFN2	9	1.87438E-34	9.64318E-33
ENSGALG00000006237	PKN2	8	1.9624E-34	1.00459E-32
ENSGALG00000004332	PLA2G12B	6	1.96914E-34	1.00459E-32
ENSGALG00000000862	RLF	23	2.09781E-34	1.06578E-32
ENSGALG00000040248	PLEKHF2	2	2.15734E-34	1.09147E-32
ENSGALG00000041684	FBXO30	3	2.22742E-34	1.12227E-32
ENSGALG00000010359	LRRC41	8	2.26207E-34	1.13357E-32
ENSGALG00000012570	TANC1	7	2.26844E-34	1.13357E-32
ENSGALG00000009856	GNS	1	2.33767E-34	1.1634E-32
ENSGALG00000004506	URI1	11	2.40941E-34	1.19423E-32
ENSGALG00000006309	SMAD5	13	2.46249E-34	1.21559E-32
ENSGALG00000012889	ZCCHC2	2	2.66281E-34	1.30918E-32
ENSGALG00000039168	SCFD2	4	2.70579E-34	1.32497E-32
ENSGALG00000040059	C21orf62	1	2.81508E-34	1.36904E-32
ENSGALG00000040685		25	2.81824E-34	1.36904E-32
ENSGALG00000017370	ARPP19	10	2.87E-34	1.38865E-32
ENSGALG00000039558	TTC17	5	2.91569E-34	1.39979E-32
ENSGALG00000031130	GGH	2	2.91599E-34	1.39979E-32
ENSGALG00000001463	PDIK1L	23	2.94786E-34	1.40954E-32
ENSGALG00000033129	ZFP36L2	3	3.00868E-34	1.433E-32
ENSGALG00000011965	ZC3H7B	1	3.048E-34	1.44608E-32
ENSGALG00000008477	EXOC4	1	3.06358E-34	1.44784E-32
ENSGALG00000040834	LGR4	5	3.21962E-34	1.51571E-32
ENSGALG00000012024	MRTFA	1	3.30478E-34	1.54981E-32
ENSGALG00000007902	ATP7A	4	3.33581E-34	1.55837E-32

ENSGALG00000043193	TAF2	2	3.4124E-34	1.58807E-32
ENSGALG00000027922	ETAA1	3	3.53706E-34	1.63784E-32
ENSGALG00000016238	MED14	1	3.54621E-34	1.63784E-32
ENSGALG00000040189	RIPK4	1	3.71948E-34	1.71002E-32
ENSGALG00000052037		2	3.73054E-34	1.71002E-32
ENSGALG00000010385		3	3.93573E-34	1.79732E-32
ENSGALG00000007298	BACE1	24	3.98841E-34	1.81458E-32
ENSGALG00000001644	SIN3A	10	4.07167E-34	1.84557E-32
ENSGALG00000028191	GLCE	10	4.20254E-34	1.89784E-32
ENSGALG00000005530	VEZF1	19	4.50117E-34	2.02519E-32
ENSGALG00000028046	INSIG2	7	4.54457E-34	2.03721E-32
ENSGALG00000030966	MAPK13	26	4.58087E-34	2.04596E-32
ENSGALG00000010150	SEC23A	5	4.80246E-34	2.13709E-32
ENSGALG00000017185	TMEM123	1	4.97802E-34	2.20716E-32
ENSGALG00000002527		21	5.49052E-34	2.42558E-32
ENSGALG00000008320	EDEM1	12	5.5842E-34	2.45805E-32
ENSGALG00000041111	NUP214	17	5.6493E-34	2.47777E-32
ENSGALG00000013462	STX17	2	5.90443E-34	2.58038E-32
ENSGALG00000011645	CLASP1	7	6.0515E-34	2.63521E-32
ENSGALG00000011652	TSN	7	6.24103E-34	2.70807E-32
ENSGALG00000010192	FBXO33	5	6.7804E-34	2.93168E-32
ENSGALG00000055101	TASOR2	1	7.0319E-34	3.02968E-32
ENSGALG00000007625	FADD	5	7.20934E-34	3.09519E-32
ENSGALG00000001474	RAB14	17	7.65386E-34	3.27451E-32
ENSGALG00000033887	IL17RA	1	7.77639E-34	3.3153E-32
ENSGALG00000036391	GFPT1	22	7.84595E-34	3.3333E-32
ENSGALG00000007932	TRAF6	5	7.97722E-34	3.3773E-32
ENSGALG00000016813	CHAMP1	1	8.05313E-34	3.39764E-32
ENSGALG00000003691	RNF145	13	8.28913E-34	3.48515E-32
ENSGALG00000040474	ITCH	20	8.41176E-34	3.52456E-32
ENSGALG00000040917	PIK3R4	2	8.53578E-34	3.56427E-32
ENSGALG00000033126	DGCR8	15	8.8238E-34	3.67197E-32
ENSGALG00000011005	KCNK1	3	9.64333E-34	3.99936E-32

ENSGALG00000010699	MIOS	2	9.77573E-34	4.04052E-32
ENSGALG00000006663	ARHGEF12	24	9.97852E-34	4.11041E-32
ENSGALG00000006061	RAP2C	4	1.0125E-33	4.15669E-32
ENSGALG00000011456	CDK17	1	1.03898E-33	4.25109E-32
ENSGALG00000037410	HEPH	4	1.05045E-33	4.28365E-32
ENSGALG00000003674	DHTKD1	1	1.05684E-33	4.29535E-32
ENSGALG00000025858	LUZP1	23	1.07884E-33	4.37019E-32
ENSGALG00000016727	JADE3	1	1.08995E-33	4.40058E-32
ENSGALG00000031131	VIRMA	2	1.10134E-33	4.43189E-32
ENSGALG00000010678	DESI2	3	1.14536E-33	4.59387E-32
ENSGALG00000001777	MFSD11	18	1.31112E-33	5.24147E-32
ENSGALG00000029316		23	1.32832E-33	5.29286E-32
ENSGALG00000007569	KBTBD8	12	1.34126E-33	5.32703E-32
ENSGALG00000002301	CNOT1	11	1.39969E-33	5.54104E-32
ENSGALG00000032092	PPP4R1	2	1.43461E-33	5.66092E-32
ENSGALG00000009885	HELB	1	1.491E-33	5.86443E-32
ENSGALG00000034965	DIP2B	33	1.53783E-33	6.02919E-32
ENSGALG00000026188	PGAP6	14	1.58726E-33	6.20302E-32
ENSGALG00000011917	PPM1A	5	1.64266E-33	6.39901E-32
ENSGALG00000002137	PGAM5	15	1.702E-33	6.60907E-32
ENSGALG00000012849	SLC37A3	1	1.72429E-33	6.67439E-32
ENSGALG00000036017	NCOA2	2	1.80288E-33	6.95649E-32
ENSGALG00000020260	PGRMC1	4	2.02627E-33	7.79378E-32
ENSGALG00000007928	MLLT10	2	2.0709E-33	7.9404E-32
ENSGALG00000007435	RAB18	2	2.11771E-33	8.09444E-32
ENSGALG00000007750	RLIM	4	2.1573E-33	8.19617E-32
ENSGALG00000032280	NUP88	19	2.15777E-33	8.19617E-32
ENSGALG00000006286	USP31	14	2.30126E-33	8.71405E-32
ENSGALG00000014115	SLAIN2	4	2.33366E-33	8.8094E-32
ENSGALG00000034999	ZHX1	2	2.6092E-33	9.81913E-32
ENSGALG00000050210		5	2.64884E-33	9.93764E-32
ENSGALG00000029674		19	2.68418E-33	1.00393E-31
ENSGALG00000007934	KCTD2	18	2.73899E-33	1.0213E-31

ENSGALG00000001034	AAR2	20	2.79834E-33	1.04025E-31
ENSGALG00000009836	ELMOD2	4	2.8596E-33	1.05979E-31
ENSGALG00000008056	B4GALT5	20	2.95191E-33	1.09069E-31
ENSGALG00000009549	KPNA4	9	3.12081E-33	1.14961E-31
ENSGALG00000005854	CLUH	19	3.13035E-33	1.14965E-31
ENSGALG00000039774	YTHDF3	2	3.24202E-33	1.18709E-31
ENSGALG00000006898	EIF4ENIF1	15	3.35529E-33	1.22488E-31
ENSGALG00000015030	NPC1	2	3.45076E-33	1.25597E-31
ENSGALG00000005198	RPS6KB1	19	3.6081E-33	1.30933E-31
ENSGALG00000011541	BIN1	7	3.71266E-33	1.34012E-31
ENSGALG00000054430	GLYCTK	12	3.71492E-33	1.34012E-31
ENSGALG00000021572	SSH2	19	3.76635E-33	1.35466E-31
ENSGALG00000031796	SP3	7	3.94546E-33	1.41491E-31
ENSGALG00000024034	PI4KB	25	4.02151E-33	1.43796E-31
ENSGALG00000007685	BCL9L	24	4.17319E-33	1.48783E-31
ENSGALG00000009800	SETD7	4	4.30632E-33	1.53081E-31
ENSGALG00000042051	SETD2	2	4.65826E-33	1.65111E-31
ENSGALG00000032832	DNAJC16	21	4.72676E-33	1.67053E-31
ENSGALG00000042762	ZBTB26	17	4.81227E-33	1.69584E-31
ENSGALG00000004295	CPD	19	4.8797E-33	1.71465E-31
ENSGALG00000016795	TGFBRAP1	1	4.91562E-33	1.7223E-31
ENSGALG00000000525	MAP3K3	27	4.94491E-33	1.7276E-31
ENSGALG00000041505	PRRC2B	17	5.26531E-33	1.83428E-31
ENSGALG00000039920	SNRK	2	5.35946E-33	1.86176E-31
ENSGALG00000019286		1	5.58428E-33	1.93435E-31
ENSGALG00000043373	ARVCF	15	5.62632E-33	1.94339E-31
ENSGALG00000035940	SLC2A13	1	5.68458E-33	1.95797E-31
ENSGALG00000008645	NKRF	4	6.003E-33	2.06182E-31
ENSGALG00000048613	KDM4B	28	6.08827E-33	2.08041E-31
ENSGALG00000001501	MAPK3	15	6.09125E-33	2.08041E-31
ENSGALG00000005807	IPO7	5	6.21259E-33	2.11592E-31
ENSGALG00000010304	MLH3	5	6.85932E-33	2.32969E-31
ENSGALG00000006371	C5orf24	13	6.99778E-33	2.37011E-31

ENSGALG00000032659	ZBED4	1	7.18058E-33	2.42528E-31
ENSGALG00000026727	SLC30A10	3	7.41688E-33	2.49818E-31
ENSGALG00000012030	QSER1	5	7.51682E-33	2.52486E-31
ENSGALG00000009674	CLCN3	4	7.54841E-33	2.52851E-31
ENSGALG00000005468	SYNRG	19	7.92744E-33	2.6482E-31
ENSGALG00000015546	TBC1D14	4	7.9766E-33	2.65734E-31
ENSGALG00000012380	SPOPL	7	8.18612E-33	2.71971E-31
ENSGALG00000039500	GSPT2	14	8.4084E-33	2.78597E-31
ENSGALG00000004225	KDM2B	15	8.70029E-33	2.87487E-31
ENSGALG00000050380	TMPPE	2	9.00779E-33	2.96843E-31
ENSGALG00000012444	ACVR2A	7	9.16897E-33	3.0134E-31
ENSGALG00000008872	FAM199X	4	9.5335E-33	3.12478E-31
ENSGALG00000040663		21	1.00099E-32	3.27213E-31
ENSGALG00000004659	ZER1	17	1.04164E-32	3.39592E-31
ENSGALG00000040155	ZW10	24	1.07414E-32	3.49253E-31
ENSGALG00000009438	SLC39A9	5	1.10433E-32	3.58113E-31
ENSGALG00000009341	CRAMP1	14	1.11146E-32	3.59471E-31
ENSGALG00000004806	EDEM3	8	1.12405E-32	3.62582E-31
ENSGALG00000054178	CLDN12	2	1.13745E-32	3.65936E-31
ENSGALG00000016949	WBP4	1	1.17039E-32	3.75542E-31
ENSGALG00000002414	TSPAN14	6	1.18778E-32	3.8012E-31
ENSGALG00000017153	RDX	1	1.19665E-32	3.81958E-31
ENSGALG00000009847	STXBP6	5	1.25354E-32	3.99071E-31
ENSGALG00000041896	RFX7	10	1.2839E-32	4.07672E-31
ENSGALG00000010202	ENTPD5	5	1.33607E-32	4.23135E-31
ENSGALG00000001692	CAMSAP1	17	1.3538E-32	4.27639E-31
ENSGALG00000033074	ARID2	1	1.36126E-32	4.28885E-31
ENSGALG00000047847		13	1.3958E-32	4.38634E-31
ENSGALG00000005494	MLYCD	11	1.39968E-32	4.38724E-31
ENSGALG00000037423	PTPRK	3	1.4162E-32	4.42763E-31
ENSGALG00000000430	SPPL2C	28	1.44672E-32	4.51147E-31
ENSGALG00000005121	TAF4	20	1.45281E-32	4.51889E-31
ENSGALG00000002870	LEMD2	26	1.45965E-32	4.52863E-31

ENSGALG00000033064	DDX6	24	1.49418E-32	4.62399E-31
ENSGALG00000013685	EDRF1	6	1.50323E-32	4.64022E-31
ENSGALG00000039178	CHD9	11	1.51156E-32	4.65416E-31
ENSGALG00000006410	HTATSF1	4	1.52096E-32	4.6713E-31
ENSGALG00000029298	F5	1	1.55367E-32	4.75978E-31
ENSGALG00000043085	RNF14	13	1.57913E-32	4.82565E-31
ENSGALG00000005134	ZSWIM8	6	1.58575E-32	4.83376E-31
ENSGALG00000016034	ATRN	4	1.60413E-32	4.8776E-31
ENSGALG00000011400	USP44	1	1.63202E-32	4.95005E-31
ENSGALG00000038780	PRDM2	21	1.66803E-32	5.04672E-31
ENSGALG00000014582	CADM1	24	1.71731E-32	5.18296E-31
ENSGALG00000048716	FBXL3	1	1.79206E-32	5.39521E-31
ENSGALG00000009915	EML4	3	1.83695E-32	5.51672E-31
ENSGALG00000010136	HGSNAT	4	1.85676E-32	5.56251E-31
ENSGALG00000041939	FAM222B	19	1.89621E-32	5.66678E-31
ENSGALG00000016217		1	1.91421E-32	5.7066E-31
ENSGALG00000036322	MED12	4	1.97145E-32	5.86289E-31
ENSGALG00000008208	CASC4	10	2.05585E-32	6.09903E-31
ENSGALG00000013834	CCDC28A	3	2.08418E-32	6.16807E-31
ENSGALG00000054564	KLHL15	1	2.11277E-32	6.23752E-31
ENSGALG00000028148	PHF13	21	2.18586E-32	6.43774E-31
ENSGALG00000017112	MTMR6	1	2.23244E-32	6.55908E-31
ENSGALG00000036417	RNF10	15	2.25003E-32	6.59059E-31
ENSGALG00000002878	CREBRF	13	2.25683E-32	6.59059E-31
ENSGALG00000051957	PTPN12	1	2.25938E-32	6.59059E-31
ENSGALG00000006966	ZNF518A	6	2.29105E-32	6.667E-31
ENSGALG00000016327		1	2.31879E-32	6.73167E-31
ENSGALG00000004160	G3BP1	13	2.44607E-32	7.08431E-31
ENSGALG00000003129	RAB11FIP1	22	2.45734E-32	7.10008E-31
ENSGALG00000006424	JADE2	13	2.56335E-32	7.38888E-31
ENSGALG00000031917	ANKIB1	2	2.6471E-32	7.61228E-31
ENSGALG00000049609		31	2.6864E-32	7.70713E-31
ENSGALG00000015157	TRAPPC8	2	2.72464E-32	7.79849E-31

ENSGALG00000008016	GRB2	18	2.76996E-32	7.90964E-31
ENSGALG00000026468	CHSY1	10	2.84379E-32	8.10149E-31
ENSGALG00000003568	PPP1R16B	20	2.99726E-32	8.51878E-31
ENSGALG00000040018	NUP98	1	3.439E-32	9.73929E-31
ENSGALG00000042125	AP2B1	19	3.44266E-32	9.73929E-31
ENSGALG00000043243		2	3.61345E-32	1.01988E-30
ENSGALG00000032257	CSNK1E	1	3.71972E-32	1.04745E-30
ENSGALG00000014321	SMIM14	4	3.7958E-32	1.06641E-30
ENSGALG00000015197	SLC35A5	1	3.82526E-32	1.07222E-30
ENSGALG00000033469	KDM1A	23	3.83528E-32	1.07256E-30
ENSGALG00000016329	AGPAT5	3	3.94744E-32	1.1014E-30
ENSGALG00000022804		1	4.1412E-32	1.15282E-30
ENSGALG00000010246	OSBPL8	1	4.2359E-32	1.1765E-30
ENSGALG00000026258	MAF	11	4.36386E-32	1.20928E-30
ENSGALG00000004581	NDST1	13	4.70199E-32	1.29907E-30
ENSGALG00000001182	NCOA6	20	4.70917E-32	1.29907E-30
ENSGALG00000010541	BIRC6	3	4.96454E-32	1.36643E-30
ENSGALG00000004937	VGLL4	12	5.36631E-32	1.47368E-30
ENSGALG00000010372	ANGEL1	5	5.44245E-32	1.49123E-30
ENSGALG00000008457	RIN2	3	5.69967E-32	1.55821E-30
ENSGALG00000028253	ATXN1L	11	5.74663E-32	1.56502E-30
ENSGALG00000005882	LRCH2	4	5.75024E-32	1.56502E-30
ENSGALG00000001341	ZBTB44	24	6.03148E-32	1.6379E-30
ENSGALG00000039573		30	6.18172E-32	1.67497E-30
ENSGALG00000007266	ZNF438	2	6.23416E-32	1.68543E-30
ENSGALG00000044805	RNF26	24	6.42011E-32	1.73187E-30
ENSGALG00000005858	DR1	8	6.7226E-32	1.80946E-30
ENSGALG00000032289	IKZF5	6	7.05337E-32	1.89431E-30
ENSGALG00000007396	TAOK3	15	7.09324E-32	1.90083E-30
ENSGALG00000004228	USP40	7	7.36923E-32	1.97046E-30
ENSGALG00000011569	SCYL2	1	7.38948E-32	1.97155E-30
ENSGALG00000041953	KDM2A	5	7.41453E-32	1.97392E-30
ENSGALG00000027908	CYP2U1	4	7.78524E-32	2.06809E-30

ENSGALG00000006788	RPRD1B	20	7.87876E-32	2.08736E-30
ENSGALG00000047393	RAB11FIP5	4	7.89203E-32	2.08736E-30
ENSGALG00000015142	DSG2	2	8.15534E-32	2.15234E-30
ENSGALG00000005845	SLC7A5	11	8.44018E-32	2.2227E-30
ENSGALG00000049811		28	8.64852E-32	2.27266E-30
ENSGALG00000010629	TUT4	8	8.77161E-32	2.30005E-30
ENSGALG00000007554	RAB22A	20	8.81306E-32	2.30596E-30
ENSGALG00000001708	MED1	27	9.02048E-32	2.35517E-30
ENSGALG00000030268	NKTR	2	9.07392E-32	2.36407E-30
ENSGALG00000012882	KDSR	2	9.5415E-32	2.48059E-30
ENSGALG00000012256	UTRN	3	9.58785E-32	2.48733E-30
ENSGALG00000009328	EIF3A	6	9.6827E-32	2.50661E-30
ENSGALG00000044620		4	9.73337E-32	2.51438E-30
ENSGALG00000042816	TP53BP2	3	1.02258E-31	2.63601E-30
ENSGALG00000010569	TXNDC12	8	1.04555E-31	2.68953E-30
ENSGALG00000028423	HS2ST1	8	1.07645E-31	2.76272E-30
ENSGALG00000008162	FBXO18	1	1.07853E-31	2.76272E-30
ENSGALG00000002830	NUDT3	26	1.08103E-31	2.76332E-30
ENSGALG00000054428	XXYLT1	9	1.08417E-31	2.76554E-30
ENSGALG00000012813	PRPF4B	2	1.12057E-31	2.85243E-30
ENSGALG00000012144	MIEF1	1	1.15427E-31	2.9321E-30
ENSGALG00000042522	ZDHHC7	11	1.20716E-31	3.06007E-30
ENSGALG00000015340	TGS1	2	1.21143E-31	3.0645E-30
ENSGALG00000002262	ETF1	13	1.24542E-31	3.14129E-30
ENSGALG00000008491	BIRC8	4	1.24693E-31	3.14129E-30
ENSGALG00000041766	JOSD1	1	1.27919E-31	3.2159E-30
ENSGALG00000000902	KDF1	23	1.28667E-31	3.22805E-30
ENSGALG00000050995	LRRC47	21	1.38433E-31	3.46594E-30
ENSGALG00000005901	PCMTD2	20	1.39043E-31	3.47408E-30
ENSGALG00000005507	NONO	4	1.42183E-31	3.54006E-30
ENSGALG00000008238	MED13L	15	1.42264E-31	3.54006E-30
ENSGALG00000033270	KLHL26	28	1.43315E-31	3.55894E-30
ENSGALG00000034166	ZCCHC14	11	1.43609E-31	3.55899E-30

ENSGALG00000011898	PHETA2	1	1.4768E-31	3.65245E-30
ENSGALG00000010211	ALDH6A1	5	1.48188E-31	3.65762E-30
ENSGALG00000046787	NAGPA	14	1.50434E-31	3.70554E-30
ENSGALG00000009968	OTUD4	4	1.50861E-31	3.70858E-30
ENSGALG00000034003	ARID4B	3	1.52437E-31	3.73978E-30
ENSGALG00000005891	DGCR2	15	1.54354E-31	3.77919E-30
ENSGALG00000037787	MEF2D	25	1.54731E-31	3.78083E-30
ENSGALG00000002424	RECQL5	18	1.57641E-31	3.84423E-30
ENSGALG00000007215	KIF5B	2	1.58253E-31	3.85147E-30
ENSGALG00000005770	HMGXB3	13	1.60484E-31	3.89798E-30
ENSGALG00000032685	HECTD4	15	1.64551E-31	3.98092E-30
ENSGALG00000011216	BTD	2	1.64552E-31	3.98092E-30
ENSGALG00000026302	SRPRB	9	1.65432E-31	3.99429E-30
ENSGALG00000001577	FOXK2	18	1.66484E-31	4.01174E-30
ENSGALG00000039883	PIGV	23	1.6939E-31	4.07372E-30
ENSGALG00000004710	TMOD3	10	1.70398E-31	4.08989E-30
ENSGALG00000004856	NUP62	4	1.71595E-31	4.11053E-30
ENSGALG00000006382	PTPRJ	5	1.77797E-31	4.25075E-30
ENSGALG00000001596	MLLT1	28	1.82144E-31	4.34614E-30
ENSGALG00000036294	CAP1	23	1.82891E-31	4.35546E-30
ENSGALG00000011620	AKT1	5	1.84993E-31	4.39692E-30
ENSGALG00000049653	KMT2C	2	1.90214E-31	4.50369E-30
ENSGALG00000009013	MKKS	3	1.90224E-31	4.50369E-30
ENSGALG00000001329	EPB41	23	1.91056E-31	4.51268E-30
ENSGALG00000041123	DSTYK	26	1.91344E-31	4.51268E-30
ENSGALG00000008870	ZDHHC6	6	1.98845E-31	4.68053E-30
ENSGALG00000034340	ATP2C1	2	2.00208E-31	4.70353E-30
ENSGALG00000029585	FUNDC2	4	2.03467E-31	4.77091E-30
ENSGALG00000038154	YAP1	1	2.0482E-31	4.79341E-30
ENSGALG00000003047	SUGP2	28	2.12086E-31	4.95396E-30
ENSGALG00000008648	UBE2A	4	2.13567E-31	4.97902E-30
ENSGALG00000049421		7	2.14986E-31	5.00253E-30
ENSGALG00000002356	CMTR2	11	2.15659E-31	5.00863E-30

ENSGALG00000031474	ZRANB1	6	2.20824E-31	5.11883E-30
ENSGALG00000016854	LIG4	1	2.24122E-31	5.18543E-30
ENSGALG00000023648	PPP3R1	3	2.25297E-31	5.20273E-30
ENSGALG00000031163	ZNRF2	2	2.36417E-31	5.44921E-30
ENSGALG00000001691	INTS11	21	2.51356E-31	5.78261E-30
ENSGALG00000000855	ZMPSTE24	23	2.55196E-31	5.8599E-30
ENSGALG00000033470	ADGRA3	4	2.60976E-31	5.97981E-30
ENSGALG00000014426	KPNA1	1	2.61399E-31	5.97981E-30
ENSGALG00000029679	PLEKHA7	5	2.65997E-31	6.07361E-30
ENSGALG00000006787	CCDC71	12	2.67196E-31	6.08956E-30
ENSGALG00000002305	ZBTB41	8	2.68614E-31	6.11046E-30
ENSGALG00000041564		1	2.73915E-31	6.21945E-30
ENSGALG00000036128	ZNF407	2	2.94651E-31	6.67784E-30
ENSGALG00000003194	ATP6V0A2	15	3.00385E-31	6.79516E-30
ENSGALG00000001639	MTF1	23	3.07146E-31	6.93524E-30
ENSGALG00000009287	ATF2	7	3.11098E-31	7.01149E-30
ENSGALG00000008347	ATG13	5	3.16392E-31	7.11765E-30
ENSGALG00000003428		28	3.26588E-31	7.3335E-30
ENSGALG00000009992	ARHGAP5	5	3.27223E-31	7.33424E-30
ENSGALG00000040335	CEBPZ	3	3.30816E-31	7.40118E-30
ENSGALG00000001546	ZZEF1	19	3.36509E-31	7.51474E-30
ENSGALG00000034317	SYNJ1	1	3.40553E-31	7.59117E-30
ENSGALG00000053299	KCNC3	33	3.44371E-31	7.66225E-30
ENSGALG00000014922	ROCK1	2	3.45031E-31	7.66295E-30
ENSGALG00000002447	CTNNA1	13	3.46487E-31	7.6813E-30
ENSGALG00000010613	ZFYVE9	8	3.67537E-31	8.13316E-30
ENSGALG00000011373	TECPR2	5	3.72383E-31	8.22548E-30
ENSGALG00000002655	ITGAV	7	3.92482E-31	8.65376E-30
ENSGALG00000053597	MBNL3	4	3.98096E-31	8.74691E-30
ENSGALG00000007590	MTMR8	4	3.98141E-31	8.74691E-30
ENSGALG00000039130	TBC1D22B	26	4.0032E-31	8.77897E-30
ENSGALG00000010869	MYSM1	8	4.08319E-31	8.9383E-30
ENSGALG00000045168		1	4.11577E-31	8.99347E-30

ENSGALG00000035045	TASOR	12	4.31116E-31	9.40357E-30
ENSGALG00000004745	NATD1	14	4.33646E-31	9.44188E-30
ENSGALG00000015351	CBLB	1	4.3474E-31	9.44881E-30
ENSGALG00000006395	WDR53	9	4.60708E-31	9.99539E-30
ENSGALG00000010620	SPAST	3	4.65536E-31	1.00822E-29
ENSGALG00000000996		28	4.88619E-31	1.05634E-29
ENSGALG00000040464	STRN3	5	4.9427E-31	1.06666E-29
ENSGALG00000031973	ANKRD52	33	5.00414E-31	1.07801E-29
ENSGALG00000006108	PABIR2	4	5.02124E-31	1.07979E-29
ENSGALG00000009774	ELF2	4	5.18502E-31	1.11305E-29
ENSGALG00000042614	NBAS	3	5.24472E-31	1.12354E-29
ENSGALG00000010314	NEK9	5	5.25235E-31	1.12354E-29
ENSGALG00000008435	SUMO1	7	5.32966E-31	1.13808E-29
ENSGALG00000001536		20	5.38363E-31	1.1476E-29
ENSGALG00000008988	ZZZ3	8	5.54681E-31	1.18032E-29
ENSGALG00000031016	GTF3C4	17	5.65575E-31	1.2014E-29
ENSGALG00000004679	AKAP10	19	5.68561E-31	1.20565E-29
ENSGALG00000019228	SFT2D2	1	5.71999E-31	1.20875E-29
ENSGALG00000034179	SLC12A4	11	5.72105E-31	1.20875E-29
ENSGALG00000051654	YLPM1	5	5.73001E-31	1.20875E-29
ENSGALG00000051335	INO80C	2	5.74707E-31	1.21026E-29
ENSGALG00000000296	PCNX1	5	5.81932E-31	1.22336E-29
ENSGALG00000026820	RNF146	3	5.85861E-31	1.2295E-29
ENSGALG00000000708	NUCKS1	26	5.95366E-31	1.2473E-29
ENSGALG00000035490	MAPK1IP1L	5	6.11952E-31	1.27985E-29
ENSGALG00000049812	SLC66A2	2	6.15029E-31	1.28408E-29
ENSGALG00000004104	INTS1	14	6.23003E-31	1.2977E-29
ENSGALG00000040986	FAM76A	23	6.23681E-31	1.2977E-29
ENSGALG00000044788	ZNF628	31	6.38193E-31	1.32564E-29
ENSGALG00000010230	TESK2	8	6.4451E-31	1.33648E-29
ENSGALG00000010835	DTX4	5	6.59585E-31	1.36542E-29
ENSGALG00000011251	TBC1D5	2	6.68333E-31	1.38118E-29
ENSGALG00000043178	SBF1	1	6.76238E-31	1.39516E-29

ENSGALG00000053640	GZF1	3	6.86428E-31	1.41379E-29
ENSGALG00000014937	KPNA5	3	7.46093E-31	1.53408E-29
ENSGALG00000010270	AREL1	5	7.66805E-31	1.57151E-29
ENSGALG00000038152	ABCC6	14	7.66874E-31	1.57151E-29
ENSGALG00000029077	CBL	24	7.69243E-31	1.57372E-29
ENSGALG00000011718	ZBTB1	5	7.77323E-31	1.58759E-29
ENSGALG00000047559	DENND2B	5	8.04033E-31	1.63939E-29
ENSGALG00000001521	VPS26B	24	8.13905E-31	1.65409E-29
ENSGALG00000007410	IGDCC4	10	8.13955E-31	1.65409E-29
ENSGALG00000003405	BSDC1	23	8.23524E-31	1.67012E-29
ENSGALG00000034311	BTBD7	5	8.2458E-31	1.67012E-29
ENSGALG00000016728	RP2	1	8.30544E-31	1.67941E-29
ENSGALG00000006225	EFL1	10	8.72764E-31	1.76186E-29
ENSGALG00000036441	WIPF1	7	8.97335E-31	1.80835E-29
ENSGALG00000042533	TSPOAP1	19	8.9876E-31	1.80835E-29
ENSGALG00000005684	FBXL15	6	9.55704E-31	1.91975E-29
ENSGALG00000029484	RIC8B	1	9.61038E-31	1.92729E-29
ENSGALG00000042804	SAMD4B	32	9.65346E-31	1.93145E-29
ENSGALG00000010576	GTF2A1	5	9.66281E-31	1.93145E-29
ENSGALG00000004082	TAOK1	19	9.77076E-31	1.94983E-29
ENSGALG00000008370	TRAK2	7	9.79799E-31	1.95207E-29
ENSGALG00000010736	DIO1	8	9.91296E-31	1.96868E-29
ENSGALG00000014094	STK38L	1	9.91361E-31	1.96868E-29
ENSGALG00000052280	HEATR5A	5	1.0035E-30	1.98954E-29
ENSGALG00000036932	SPEN	21	1.04376E-30	2.06601E-29
ENSGALG00000007527	ADARB1	7	1.06722E-30	2.10901E-29
ENSGALG00000010207	LARP1B	4	1.07089E-30	2.11116E-29
ENSGALG00000020736	PHOSPHO2	7	1.07177E-30	2.11116E-29
ENSGALG00000007302	ATXN7	12	1.08861E-30	2.14087E-29
ENSGALG00000026322	ARMT1	3	1.12303E-30	2.205E-29
ENSGALG00000024094	UBAP2L	25	1.13816E-30	2.23112E-29
ENSGALG00000014614	TNKS2	6	1.1451E-30	2.24112E-29
ENSGALG00000050969	RBM15B	12	1.17598E-30	2.29787E-29

ENSGALG00000015889	PHIP	3	1.18928E-30	2.32013E-29
ENSGALG00000003204	MED22	17	1.21167E-30	2.36004E-29
ENSGALG00000003068	EZH1	27	1.21879E-30	2.37014E-29
ENSGALG00000006873		5	1.2469E-30	2.42094E-29
ENSGALG00000005241	DYNC1LI2	11	1.25182E-30	2.42662E-29
ENSGALG00000008482	STAG2	4	1.29241E-30	2.50133E-29
ENSGALG00000052583		1	1.361E-30	2.6299E-29
ENSGALG00000000470	LMNA	28	1.37725E-30	2.65709E-29
ENSGALG00000002695	SLC15A4	15	1.44206E-30	2.77772E-29
ENSGALG00000041911	AAK1	22	1.45607E-30	2.8003E-29
ENSGALG00000006867	QRICH1	12	1.4597E-30	2.80285E-29
ENSGALG00000049838		18	1.47345E-30	2.82481E-29
ENSGALG00000029238		8	1.47773E-30	2.82857E-29
ENSGALG00000013826	ABRACL	3	1.54118E-30	2.94539E-29
ENSGALG00000017076	B3GLCT	1	1.59576E-30	3.04493E-29
ENSGALG00000009098	MAMLD1	4	1.61631E-30	3.07933E-29
ENSGALG00000001419	DAB2IP	17	1.62332E-30	3.08503E-29
ENSGALG00000013793	CLOCK	4	1.62437E-30	3.08503E-29
ENSGALG00000030253	RBM12	20	1.67603E-30	3.17821E-29
ENSGALG00000020879	SLC30A7	8	1.72013E-30	3.25676E-29
ENSGALG00000033385	COPS8	7	1.77078E-30	3.34746E-29
ENSGALG00000030147	AFF4	13	1.79897E-30	3.39549E-29
ENSGALG00000039424	MTRR	2	1.8993E-30	3.57932E-29
ENSGALG00000031684	RCC2	21	1.9309E-30	3.63325E-29
ENSGALG00000010705	ZBTB18	3	1.94187E-30	3.64826E-29
ENSGALG00000004300	PTDSS2	5	1.95757E-30	3.6721E-29
ENSGALG00000002929	TRIP12	9	2.02918E-30	3.79738E-29
ENSGALG00000039815	MARF1	14	2.03058E-30	3.79738E-29
ENSGALG00000033500	PDCD6IP	2	2.08808E-30	3.89892E-29
ENSGALG00000026210	POMK	4	2.14002E-30	3.9898E-29
ENSGALG00000011065	SPRTN	3	2.20389E-30	4.1026E-29
ENSGALG00000003359	RAB5C	27	2.2159E-30	4.11753E-29
ENSGALG00000015259	RB1CC1	2	2.21867E-30	4.11753E-29

ENSGALG00000008974	USP33	8	2.27239E-30	4.21082E-29
ENSGALG00000052199	SNRPD2	32	2.28029E-30	4.21905E-29
ENSGALG00000054939	RAPH1	7	2.35301E-30	4.347E-29
ENSGALG00000011639		5	2.42458E-30	4.47246E-29
ENSGALG00000030904	LRP1	33	2.43113E-30	4.47776E-29
ENSGALG00000040000	ERO1B	3	2.49803E-30	4.59404E-29
ENSGALG00000034316	CNOT6	13	2.55958E-30	4.70014E-29
ENSGALG00000050634		32	2.56718E-30	4.70701E-29
ENSGALG00000016481	PUM2	3	2.58663E-30	4.7211E-29
ENSGALG00000034348	ATAD2	2	2.58991E-30	4.7211E-29
ENSGALG00000009782	NAA15	4	2.5907E-30	4.7211E-29
ENSGALG00000000726	ELAVL1	28	2.59209E-30	4.7211E-29
ENSGALG00000005415	TEAD1	5	2.59423E-30	4.7211E-29
ENSGALG00000053765		1	2.72281E-30	4.94773E-29
ENSGALG00000036076	UNC50	1	2.84262E-30	5.15775E-29
ENSGALG00000001374	SLC35E2B	21	2.8521E-30	5.16725E-29
ENSGALG00000051442	TSC22D1	1	2.92773E-30	5.2964E-29
ENSGALG00000006506	C13H5orf15	13	3.05203E-30	5.5131E-29
ENSGALG00000034001	SPAG9	18	3.09818E-30	5.58819E-29
ENSGALG00000009752	C1GALT1	2	3.13696E-30	5.64977E-29
ENSGALG00000009790	RAB33B	4	3.19175E-30	5.73997E-29
ENSGALG00000013731	SOCS6	2	3.24939E-30	5.83502E-29
ENSGALG00000015285	TOMM70	1	3.26125E-30	5.8477E-29
ENSGALG00000000611		26	3.42157E-30	6.12617E-29
ENSGALG00000040465	ZEB2	7	3.4324E-30	6.13655E-29
ENSGALG00000027159		3	3.45118E-30	6.16108E-29
ENSGALG00000031699	NEDD1	1	3.45818E-30	6.16456E-29
ENSGALG00000003195	TRADD	11	3.48403E-30	6.20157E-29
ENSGALG00000023806	PITPNA	19	3.49818E-30	6.21768E-29
ENSGALG00000052648	PRR14L	15	3.54037E-30	6.2835E-29
ENSGALG00000000146	SUPT6H	19	3.58717E-30	6.35255E-29
ENSGALG00000005021	RAB43	12	3.58969E-30	6.35255E-29
ENSGALG00000009304	RAB11FIP2	6	3.68423E-30	6.5104E-29

ENSGALG00000017000	RCBTB2	1	3.73462E-30	6.5899E-29
ENSGALG00000008529	FASTKD2	7	3.75531E-30	6.61683E-29
ENSGALG00000003238	GNA11	28	3.81595E-30	6.71398E-29
ENSGALG00000015791	AKIRIN2	3	4.09076E-30	7.18713E-29
ENSGALG00000015231	ZBTB24	3	4.13213E-30	7.2432E-29
ENSGALG00000003507	TECPR1	14	4.13456E-30	7.2432E-29
ENSGALG00000012894	CHRM2	1	4.20804E-30	7.36135E-29
ENSGALG00000012296	EIF3L	1	4.37638E-30	7.64487E-29
ENSGALG00000014391	CCDC149	4	4.38705E-30	7.65255E-29
ENSGALG00000020249	RAPGEF2	4	4.51778E-30	7.86932E-29
ENSGALG00000011557	UHRF1BP1L	1	4.56312E-30	7.93696E-29
ENSGALG00000011814	ATF7IP	1	4.70421E-30	8.17071E-29
ENSGALG00000040956	CHIC1	4	4.80771E-30	8.33861E-29
ENSGALG00000007970	PIAS1	10	4.921E-30	8.52298E-29
ENSGALG00000036502	CDIP1	14	4.9557E-30	8.5709E-29
ENSGALG00000041616	IRF2BPL	5	5.00545E-30	8.64468E-29
ENSGALG00000004798	MPRIP	14	5.01322E-30	8.64586E-29
ENSGALG00000001279	ZNF652	27	5.07658E-30	8.74277E-29
ENSGALG00000040428	MAVS	4	5.12092E-30	8.80669E-29
ENSGALG00000004133	SRSF10	23	5.28198E-30	9.07088E-29
ENSGALG00000038467	SELENOO	1	5.34829E-30	9.17184E-29
ENSGALG00000036677	ITSN1	1	5.37778E-30	9.20945E-29
ENSGALG00000016205	PDXK	1	5.50049E-30	9.40638E-29
ENSGALG00000007820	BTRC	6	5.66922E-30	9.68135E-29
ENSGALG00000010783	CPSF2	5	5.82348E-30	9.93087E-29
ENSGALG00000011193	C3H6orf120	3	5.89764E-30	1.00336E-28
ENSGALG00000008222	SLC26A5	1	5.90018E-30	1.00336E-28
ENSGALG00000001976	TRIM33	26	6.23886E-30	1.05948E-28
ENSGALG00000000755	ACER3	1	6.35772E-30	1.07816E-28
ENSGALG00000032273	POLR1B	3	6.40396E-30	1.08398E-28
ENSGALG00000010579	STON2	5	6.40981E-30	1.08398E-28
ENSGALG00000003589	VTN	19	6.60408E-30	1.11528E-28
ENSGALG00000021570	ZBTB6	17	6.82111E-30	1.15034E-28

ENSGALG00000027989	SBDS	19	6.94647E-30	1.16892E-28
ENSGALG00000032544	ALDH5A1	2	6.95044E-30	1.16892E-28
ENSGALG00000038772	RNF19A	2	7.09629E-30	1.1918E-28
ENSGALG00000000999	ZNF319	11	7.61605E-30	1.27734E-28
ENSGALG00000006672	LARP4B	2	7.82509E-30	1.31059E-28
ENSGALG00000040756	PI4KA	15	7.86399E-30	1.3153E-28
ENSGALG00000050637		32	7.93656E-30	1.32562E-28
ENSGALG00000026822	WDR81	19	7.97221E-30	1.32976E-28
ENSGALG00000028265	SURF4	17	7.98824E-30	1.33061E-28
ENSGALG00000047009		2	8.02816E-30	1.33543E-28
ENSGALG00000051672	NHLRC3	1	8.09574E-30	1.34484E-28
ENSGALG00000000336	PPP1R12B	26	8.1867E-30	1.3581E-28
ENSGALG00000037872	ATAD2B	3	8.24911E-30	1.3666E-28
ENSGALG00000011893	SLC38A6	5	8.31919E-30	1.37634E-28
ENSGALG00000010039	BRE	3	8.43668E-30	1.39388E-28
ENSGALG00000053657	LATS2	1	8.64673E-30	1.42639E-28
ENSGALG00000026364	ASAH1	4	8.65687E-30	1.42639E-28
ENSGALG00000005004	SAMD8	6	8.81412E-30	1.45034E-28
ENSGALG00000007115	ASB7	10	9.14128E-30	1.50215E-28
ENSGALG00000015590	MANEA	3	9.15433E-30	1.50227E-28
ENSGALG00000002267	DCUN1D3	14	9.18861E-30	1.50587E-28
ENSGALG00000016282	ZNF451	3	9.25662E-30	1.51498E-28
ENSGALG00000051135	BCL2L11	3	9.28267E-30	1.51721E-28
ENSGALG00000032632	SLC49A4	7	9.29995E-30	1.518E-28
ENSGALG00000017092	PAN3	1	9.3935E-30	1.53122E-28
ENSGALG00000032647	CLASP2	2	9.84924E-30	1.60336E-28
ENSGALG00000006837	TSPAN4	5	9.99939E-30	1.62563E-28
ENSGALG00000016265	LANCL3	1	1.00464E-29	1.63109E-28
ENSGALG00000004610	SH2B3	15	1.02346E-29	1.65945E-28
ENSGALG00000004143	YWHAB	20	1.0342E-29	1.67464E-28
ENSGALG00000051428		16	1.03716E-29	1.67721E-28
ENSGALG00000007254	ARHGAP12	2	1.04325E-29	1.68482E-28
ENSGALG00000038059	NR1H3	5	1.04879E-29	1.69152E-28

ENSGALG00000030802	SHROOM2	1	1.08864E-29	1.75347E-28
ENSGALG00000013878	PCYOX1	22	1.0918E-29	1.75624E-28
ENSGALG00000007804	GXYLT2	12	1.10946E-29	1.7823E-28
ENSGALG00000007272	RNF166	11	1.11176E-29	1.78364E-28
ENSGALG00000034550	POLR2M	10	1.12116E-29	1.79636E-28
ENSGALG00000035108	KDM7A	1	1.16107E-29	1.85786E-28
ENSGALG00000012265	MICALL1	1	1.18567E-29	1.89474E-28
ENSGALG00000039643	TMEM79	23	1.19427E-29	1.90599E-28
ENSGALG00000013026	HDHD5	1	1.20518E-29	1.92088E-28
ENSGALG00000027152	HELZ	18	1.23813E-29	1.97082E-28
ENSGALG00000051766		11	1.2746E-29	2.02623E-28
ENSGALG00000003322	TBC1D2B	10	1.28978E-29	2.04769E-28
ENSGALG00000015181	GAREM1	2	1.30719E-29	2.07263E-28
ENSGALG00000016984	ZC3H13	1	1.31203E-29	2.07761E-28
ENSGALG00000002106	CLCC1	8	1.31419E-29	2.07833E-28
ENSGALG00000028570	C6orf132	26	1.31695E-29	2.07999E-28
ENSGALG00000010048	SUPT7L	3	1.36636E-29	2.15524E-28
ENSGALG00000010885	BMP2K	4	1.37472E-29	2.16563E-28
ENSGALG00000006350	DDTL	15	1.37923E-29	2.16993E-28
ENSGALG00000012483	TMPRSS6	1	1.38736E-29	2.17991E-28
ENSGALG00000003604	SLC46A1	19	1.39164E-29	2.18382E-28
ENSGALG00000010333	CNOT6L	4	1.39484E-29	2.18603E-28
ENSGALG00000043636	YAE1	2	1.39736E-29	2.18716E-28
ENSGALG00000033099	PAPOLB	5	1.40303E-29	2.19323E-28
ENSGALG00000037697	LAMP1	1	1.40792E-29	2.19805E-28
ENSGALG00000008165	GPR75	3	1.45826E-29	2.27372E-28
ENSGALG00000033058	FASTK	2	1.46295E-29	2.27813E-28
ENSGALG00000032529	WDR43	3	1.4843E-29	2.30842E-28
ENSGALG00000012857	DENND2A	1	1.5527E-29	2.41172E-28
ENSGALG00000042320	GTF2E1	1	1.57901E-29	2.44948E-28
ENSGALG00000020813	CCDC50	9	1.63433E-29	2.53206E-28
ENSGALG00000049393		32	1.6912E-29	2.61685E-28
ENSGALG00000003299	NSD3	22	1.7642E-29	2.72635E-28

ENSGALG00000006372	RBM33	2	1.78834E-29	2.76016E-28
ENSGALG00000032156	TUBGCP6	1	1.8007E-29	2.77572E-28
ENSGALG00000002145	CAMSAP2	8	1.87608E-29	2.88827E-28
ENSGALG00000016617	NCOA1	3	1.89129E-29	2.908E-28
ENSGALG00000036028	GNA13	18	1.96699E-29	3.02059E-28
ENSGALG00000042838	TRAF2	17	2.05488E-29	3.1516E-28
ENSGALG00000013043	ATPSCKMT	2	2.06802E-29	3.16777E-28
ENSGALG00000016406	RPS6KA3	1	2.09172E-29	3.20004E-28
ENSGALG00000003314	RILPL1	15	2.10338E-29	3.21385E-28
ENSGALG00000015809	RNF103	4	2.13655E-29	3.26044E-28
ENSGALG00000043280	EXOC8	3	2.15739E-29	3.28813E-28
ENSGALG00000008443	ARHGDIA	18	2.2085E-29	3.36182E-28
ENSGALG00000009911	BICRAL	3	2.2265E-29	3.38499E-28
ENSGALG00000005230	MED13	19	2.23062E-29	3.38704E-28
ENSGALG00000010131	BTBD9	3	2.25701E-29	3.42285E-28
ENSGALG00000031461	CTDP1	2	2.29548E-29	3.47687E-28
ENSGALG00000029095	PDZD8	6	2.35089E-29	3.55637E-28
ENSGALG00000022866	ZNF654	1	2.35775E-29	3.56234E-28
ENSGALG00000014868	SERINC1	3	2.40203E-29	3.62474E-28
ENSGALG00000010006	SLC10A7	4	2.4212E-29	3.64917E-28
ENSGALG00000026692	N-RAS	26	2.43701E-29	3.66846E-28
ENSGALG00000015033	REV3L	3	2.4443E-29	3.67488E-28
ENSGALG00000032642	SMARCC1	2	2.46495E-29	3.70137E-28
ENSGALG00000011465	STT3B	2	2.47279E-29	3.70858E-28
ENSGALG00000035472	ZNF292	3	2.50184E-29	3.74754E-28
ENSGALG00000008507	THOC2	4	2.61669E-29	3.91476E-28
ENSGALG00000004542	ZMYND8	20	2.72103E-29	4.06587E-28
ENSGALG00000012659	CKAP4	1	2.80658E-29	4.18857E-28
ENSGALG00000011330	DYNC1H1	5	2.81719E-29	4.19927E-28
ENSGALG00000001907	WAPL	6	2.82689E-29	4.20858E-28
ENSGALG00000001425	SNX19	24	2.84776E-29	4.23448E-28
ENSGALG00000045246	GOLIM4	9	3.02696E-29	4.49546E-28
ENSGALG00000054273	RASSF8	1	3.22192E-29	4.77918E-28

ENSGALG00000013887	FAM210A	2	3.27634E-29	4.85295E-28
ENSGALG00000014954	ZNF518B	4	3.28396E-29	4.85295E-28
ENSGALG00000010005	EPAS1	3	3.28555E-29	4.85295E-28
ENSGALG00000035498	INPP5D	9	3.28757E-29	4.85295E-28
ENSGALG00000038954	LSM11	13	3.34518E-29	4.93201E-28
ENSGALG00000015664	ADD1	4	3.37988E-29	4.97716E-28
ENSGALG00000000934	ZBTB43	17	3.40299E-29	5.00515E-28
ENSGALG00000005878	MLST8	14	3.46432E-29	5.08921E-28
ENSGALG00000002841	RFNG	18	3.47016E-29	5.09165E-28
ENSGALG00000001619	ATG16L1	9	3.51719E-29	5.15446E-28
ENSGALG00000004169		4	3.67709E-29	5.38233E-28
ENSGALG00000014674	CLSTN3	1	3.72442E-29	5.44145E-28
ENSGALG00000048883		2	3.72641E-29	5.44145E-28
ENSGALG00000003661	SETX	17	3.90369E-29	5.6935E-28
ENSGALG00000032915	CHPF	7	3.94633E-29	5.74882E-28
ENSGALG00000009246	ACBD3	3	4.02875E-29	5.86188E-28
ENSGALG00000040161	C7orf25	2	4.07998E-29	5.92935E-28
ENSGALG00000001306	NFRKB	24	4.13987E-29	6.00922E-28
ENSGALG00000007308	RAB35	15	4.22284E-29	6.11903E-28
ENSGALG00000027682	KICS2	1	4.22556E-29	6.11903E-28
ENSGALG00000009085	MTMR1	4	4.30124E-29	6.22123E-28
ENSGALG00000007009	NCOA5	20	4.45106E-29	6.4303E-28
ENSGALG00000042177	GPR182	33	4.47697E-29	6.46008E-28
ENSGALG00000009608		7	4.75592E-29	6.85448E-28
ENSGALG00000002543	CSGALNACT2	6	5.09343E-29	7.33225E-28
ENSGALG00000002765	NBR1	27	5.21192E-29	7.49397E-28
ENSGALG00000049774		25	5.22175E-29	7.49483E-28
ENSGALG00000015059		1	5.22481E-29	7.49483E-28
ENSGALG00000048700	DDX28	11	5.33252E-29	7.64035E-28
ENSGALG00000012072		7	5.54275E-29	7.93225E-28
ENSGALG00000038981	CBLL1	1	5.56674E-29	7.95723E-28
ENSGALG00000009305	LBR	3	5.66547E-29	8.08888E-28
ENSGALG00000020805	LRRC8D	8	5.75325E-29	8.20461E-28

ENSGALG00000041124	NOLC1	6	5.82627E-29	8.29904E-28
ENSGALG00000032808		1	5.9221E-29	8.42568E-28
ENSGALG00000003753	CYLD	11	6.05302E-29	8.60192E-28
ENSGALG00000003483	GOLGA7	22	6.06308E-29	8.60619E-28
ENSGALG00000041403	DENND4A	10	6.23414E-29	8.83871E-28
ENSGALG00000039882	SLC25A32	2	6.29728E-29	8.91786E-28
ENSGALG00000047565		33	6.33049E-29	8.95449E-28
ENSGALG00000030031	TTPA	2	6.37926E-29	9.01302E-28
ENSGALG00000013710	SNX9	3	6.38703E-29	9.01354E-28
ENSGALG00000003743	VPS37B	15	6.43379E-29	9.06904E-28
ENSGALG00000003162		27	6.44421E-29	9.07323E-28
ENSGALG00000012865	BRAF	1	6.76651E-29	9.51603E-28
ENSGALG00000050416	SYDE1	30	6.78082E-29	9.52517E-28
ENSGALG00000004949	GARRE1	11	6.79218E-29	9.53016E-28
ENSGALG00000012075	DTX3L	7	6.81238E-29	9.54751E-28
ENSGALG00000010016	SOCS5	3	6.83202E-29	9.56404E-28
ENSGALG00000002501	VKORC1L1	19	6.89018E-29	9.6344E-28
ENSGALG00000016889	RAP2A	1	6.95254E-29	9.71046E-28
ENSGALG00000009942	MDM2	1	7.39575E-29	1.03177E-27
ENSGALG00000010668	WWC2	4	7.53328E-29	1.04975E-27
ENSGALG00000008053	PRDM11	5	7.54499E-29	1.05018E-27
ENSGALG00000040087	ZNF609	10	7.81265E-29	1.0862E-27
ENSGALG00000043693	ALKBH5	14	7.8572E-29	1.09115E-27
ENSGALG00000015887	URB1	1	7.91976E-29	1.09858E-27
ENSGALG00000002325	IGFALS	14	8.06666E-29	1.11769E-27
ENSGALG00000034693	MLLT6	27	8.1755E-29	1.1313E-27
ENSGALG00000008956	CCDC186	6	8.18343E-29	1.1313E-27
ENSGALG00000001042	MTMR4	19	8.47736E-29	1.17061E-27
ENSGALG00000007125	PARD3	2	8.69334E-29	1.19841E-27
ENSGALG00000050427	OSBPL10	2	8.69836E-29	1.19841E-27
ENSGALG00000031062	BAG5	5	8.89063E-29	1.22351E-27
ENSGALG00000027734	NAIF1	17	8.99755E-29	1.23683E-27
ENSGALG00000010214	JADE1	4	9.05731E-29	1.24365E-27

ENSGALG00000031560	USF3	1	9.08698E-29	1.24632E-27
ENSGALG00000004573	LRRC8A	17	9.20984E-29	1.26175E-27
ENSGALG00000003907	N4BP1	11	9.4337E-29	1.29097E-27
ENSGALG00000016781	MAP4K4	1	9.61148E-29	1.31378E-27
ENSGALG00000010276	GPBP1L1	8	9.62198E-29	1.31378E-27
ENSGALG00000006978	APOOL	4	9.8236E-29	1.33981E-27
ENSGALG00000004329		14	9.89848E-29	1.34757E-27
ENSGALG00000002182	NR5A2	8	9.90257E-29	1.34757E-27
ENSGALG00000009611	EIF2AK4	5	9.93777E-29	1.35085E-27
ENSGALG00000006357	XPC	12	1.00266E-28	1.3614E-27
ENSGALG00000029379	BRD1	1	1.03334E-28	1.4015E-27
ENSGALG00000037968	API5	5	1.03982E-28	1.40872E-27
ENSGALG00000013565		1	1.05769E-28	1.43135E-27
ENSGALG00000004523	MFN2	21	1.08573E-28	1.46766E-27
ENSGALG00000042833	MAPK14	26	1.08978E-28	1.4715E-27
ENSGALG00000042122	LARP4	33	1.10391E-28	1.48894E-27
ENSGALG00000026470		4	1.10994E-28	1.49542E-27
ENSGALG00000011395	SARAF	4	1.11323E-28	1.4982E-27
ENSGALG00000002943	PRPF8	19	1.11595E-28	1.5002E-27
ENSGALG00000042184	ALDH4A1	21	1.12873E-28	1.51571E-27
ENSGALG00000012739	HIVEP1	2	1.13302E-28	1.51979E-27
ENSGALG00000009859		1	1.13543E-28	1.52135E-27
ENSGALG00000000843	DOT1L	28	1.14526E-28	1.53284E-27
ENSGALG00000015858	DOP1A	3	1.15105E-28	1.5389E-27
ENSGALG00000043318	NMNAT1	21	1.16375E-28	1.55417E-27
ENSGALG00000006138	HELZ2	20	1.18287E-28	1.57798E-27
ENSGALG00000039734	CERS5	33	1.20387E-28	1.60424E-27
ENSGALG00000033179	TGFBR1	2	1.20814E-28	1.60818E-27
ENSGALG00000052355	PDE12	12	1.21393E-28	1.61412E-27
ENSGALG00000017388	YY2	5	1.25092E-28	1.66149E-27
ENSGALG00000012185	PLA2G12A	4	1.26331E-28	1.67612E-27
ENSGALG00000054496		31	1.27051E-28	1.68383E-27
ENSGALG00000015025	VANGL1	1	1.32797E-28	1.75809E-27

ENSGALG00000002158	GOLGA3	15	1.37161E-28	1.81389E-27
ENSGALG00000004587	RC3H1	8	1.41901E-28	1.87453E-27
ENSGALG00000003731	FBXO42	21	1.42891E-28	1.88558E-27
ENSGALG00000005660	NF1	19	1.43067E-28	1.88585E-27
ENSGALG00000010302		9	1.44768E-28	1.90467E-27
ENSGALG00000032502	HPS5	5	1.44807E-28	1.90467E-27
ENSGALG00000005823	HDLBP	9	1.45633E-28	1.91348E-27
ENSGALG00000002364	SEC16A	17	1.46539E-28	1.92331E-27
ENSGALG00000040181	EDC3	10	1.46919E-28	1.92622E-27
ENSGALG00000008603	ITPKA	5	1.49931E-28	1.9636E-27
ENSGALG00000029646	CNNM4	22	1.50247E-28	1.96563E-27
ENSGALG00000006363	CABIN1	15	1.50947E-28	1.97267E-27
ENSGALG00000042552	TJP1	10	1.51176E-28	1.97354E-27
ENSGALG00000005784	CCDC117	15	1.54212E-28	2.01102E-27
ENSGALG00000008587	NDUFAF1	5	1.56601E-28	2.03999E-27
ENSGALG00000039068	RSF1	1	1.57578E-28	2.05053E-27
ENSGALG00000016865	TPP2	1	1.57934E-28	2.05298E-27
ENSGALG00000050395		19	1.58772E-28	2.06167E-27
ENSGALG00000008199	CTDSPL2	10	1.62129E-28	2.10301E-27
ENSGALG00000002066	NCOA4	6	1.70997E-28	2.21343E-27
ENSGALG00000016806	GCC2	1	1.71004E-28	2.21343E-27
ENSGALG00000000377	HNRNPM	28	1.76342E-28	2.28011E-27
ENSGALG00000013348	OTUD7B	25	1.7707E-28	2.28709E-27
ENSGALG00000009082	COQ8A	3	1.7825E-28	2.29989E-27
ENSGALG00000043405	BNIP3L	22	1.8403E-28	2.37196E-27
ENSGALG00000000195	SLC23A2	22	1.84966E-28	2.3815E-27
ENSGALG00000019178	PRDM15	1	1.87845E-28	2.41602E-27
ENSGALG00000034610	ARFGEF1	2	2.01007E-28	2.58259E-27
ENSGALG00000005714	PKD1	14	2.0407E-28	2.61919E-27
ENSGALG00000015901	SENP6	3	2.09198E-28	2.68218E-27
ENSGALG00000009130	GPCPD1	3	2.09947E-28	2.68895E-27
ENSGALG00000033630	LRP12	2	2.18054E-28	2.78986E-27
ENSGALG00000014421	LCORL	4	2.23207E-28	2.85279E-27

ENSGALG00000008828	AFTPH	3	2.26517E-28	2.89206E-27
ENSGALG00000044260		1	2.27485E-28	2.90138E-27
ENSGALG00000006689	ABHD2	10	2.33654E-28	2.97695E-27
ENSGALG00000038598	TRIM28	31	2.47271E-28	3.14716E-27
ENSGALG00000001256	NLRC5	11	2.51509E-28	3.19775E-27
ENSGALG00000034264		5	2.58655E-28	3.28519E-27
ENSGALG00000001158	UIMC1	13	2.63357E-28	3.34143E-27
ENSGALG00000003995	ERAL1	19	2.70721E-28	3.43129E-27
ENSGALG00000035699	MARCHF6	2	2.71425E-28	3.43664E-27
ENSGALG00000010318	TMED10	5	2.71825E-28	3.43813E-27
ENSGALG00000036876	TMEM230	22	2.7596E-28	3.48682E-27
ENSGALG00000044187		14	2.79218E-28	3.52433E-27
ENSGALG00000001964	INPP5E	17	2.88645E-28	3.63955E-27
ENSGALG00000011149	PLA2R1	7	2.90725E-28	3.662E-27
ENSGALG00000033512	CPEB4	13	2.92588E-28	3.68165E-27
ENSGALG00000034773	TOGARAM1	5	3.16683E-28	3.98074E-27
ENSGALG00000008957	MSH6	3	3.20278E-28	4.02178E-27
ENSGALG00000004323	RASAL2	8	3.26312E-28	4.09334E-27
ENSGALG00000010104	DMAP1	8	3.28611E-28	4.11794E-27
ENSGALG00000052326	IRF2BP2	3	3.31655E-28	4.15182E-27
ENSGALG00000015710	TMEM129	4	3.37915E-28	4.22585E-27
ENSGALG00000035530	TFCP2	33	3.46995E-28	4.33494E-27
ENSGALG00000014974	MIB1	2	3.50096E-28	4.36921E-27
ENSGALG00000047617	DDA1	28	3.58939E-28	4.47499E-27
ENSGALG00000006385	UBXN7	9	3.60586E-28	4.49093E-27
ENSGALG00000007351	MTPAP	2	3.66863E-28	4.56445E-27
ENSGALG00000009081	ZNF106	5	3.74368E-28	4.65307E-27
ENSGALG00000030280	PABPC1	2	3.80368E-28	4.72284E-27
ENSGALG00000023407	ZBTB34	17	3.82919E-28	4.74968E-27
ENSGALG00000010018	CTSEAL	1	3.88311E-28	4.81166E-27
ENSGALG00000035774	PPFIA1	5	3.92487E-28	4.85847E-27
ENSGALG00000031542	EXT2	5	4.00322E-28	4.95044E-27
ENSGALG00000040948	ZMAT3	9	4.16656E-28	5.1472E-27

ENSGALG00000036201	GALK2	10	4.20694E-28	5.19182E-27
ENSGALG00000008160	CNNM2	6	4.24319E-28	5.23127E-27
ENSGALG00000006607	GUCD1	15	4.27484E-28	5.26496E-27
ENSGALG00000008479	PLEKHM3	7	4.31085E-28	5.30396E-27
ENSGALG00000052242		10	4.35996E-28	5.35897E-27
ENSGALG00000004550	PPTC7	15	4.4239E-28	5.43208E-27
ENSGALG00000005575	AKAP1	19	4.46981E-28	5.48011E-27
ENSGALG00000033376	APOH	18	4.472E-28	5.48011E-27
ENSGALG00000006612	ASXL1	20	4.48883E-28	5.49521E-27
ENSGALG00000015667		4	4.49978E-28	5.50309E-27
ENSGALG00000010733	CEP170	3	4.54872E-28	5.55736E-27
ENSGALG00000005146	PDP2	11	4.57704E-28	5.58637E-27
ENSGALG00000007528	TCERG1	13	4.69303E-28	5.72221E-27
ENSGALG00000007766	KIAA1217	2	4.74107E-28	5.77501E-27
ENSGALG00000010061	LRBA	4	5.00576E-28	6.09134E-27
ENSGALG00000004620	LAMC1	8	5.12291E-28	6.22759E-27
ENSGALG00000003937	GEMIN5	13	5.12795E-28	6.22759E-27
ENSGALG00000016920	LMO7	1	5.13392E-28	6.22864E-27
ENSGALG00000036833	PAXBP1	1	5.15315E-28	6.24576E-27
ENSGALG00000009222	FNDC3B	9	5.16511E-28	6.25404E-27
ENSGALG00000002542	GUSB	19	5.18142E-28	6.26757E-27
ENSGALG00000009097	SNAP23	5	5.21579E-28	6.30288E-27
ENSGALG00000012953	IPO8	1	5.26381E-28	6.35462E-27
ENSGALG00000008211	ACP2	5	5.34869E-28	6.4507E-27
ENSGALG00000001025	GOLGA1	17	5.66698E-28	6.82781E-27
ENSGALG00000009249	UBR1	5	5.86705E-28	7.06189E-27
ENSGALG00000001731	CDK12	27	5.90823E-28	7.10444E-27
ENSGALG00000000513	ZNF276	11	5.94724E-28	7.1443E-27
ENSGALG00000008837	PELI3	3	5.97491E-28	7.16924E-27
ENSGALG00000002690	PDPR	11	5.97975E-28	7.16924E-27
ENSGALG00000017071		1	6.21173E-28	7.44005E-27
ENSGALG00000016878	TM9SF2	1	6.36764E-28	7.6193E-27
ENSGALG00000013657	ZNF236	2	6.44092E-28	7.69942E-27

ENSGALG00000052195		33	6.76367E-28	8.07731E-27
ENSGALG00000008415	ALS2	7	6.8939E-28	8.21984E-27
ENSGALG00000041706	FAM13B	13	6.8965E-28	8.21984E-27
ENSGALG00000010614	SLC25A4	4	6.98737E-28	8.32002E-27
ENSGALG00000026007	DOLK	17	7.02777E-28	8.35997E-27
ENSGALG00000005840	DENND5A	5	7.1608E-28	8.50991E-27
ENSGALG00000000551	C26H6orf89	26	7.26186E-28	8.62161E-27
ENSGALG00000031495	TRIO	2	7.417E-28	8.79722E-27
ENSGALG00000000863	DYRK3	26	7.4937E-28	8.87956E-27
ENSGALG00000014467	COPS7A	1	7.60667E-28	9.00467E-27
ENSGALG00000015611	CASP8AP2	3	7.82633E-28	9.25446E-27
ENSGALG00000036163	ZNF710	10	7.83285E-28	9.25446E-27
ENSGALG00000015400	CGGBP1	1	7.85702E-28	9.27403E-27
ENSGALG00000000510	DCAF7	27	7.94555E-28	9.36945E-27
ENSGALG00000002762	UHRF1BP1	26	8.10963E-28	9.55369E-27
ENSGALG00000014998	CABLES1	2	8.13384E-28	9.57297E-27
ENSGALG00000044342	HINT3	3	8.19935E-28	9.64075E-27
ENSGALG00000038202	PWWP2A	13	8.57327E-28	1.00707E-26
ENSGALG00000005103	GDPD1	19	8.61634E-28	1.01116E-26
ENSGALG00000014603	C1S	1	8.70297E-28	1.02034E-26
ENSGALG00000006160	HNRNPA0	13	8.71167E-28	1.02038E-26
ENSGALG00000009466	SEC23IP	6	8.73638E-28	1.02229E-26
ENSGALG00000021301		13	8.76696E-28	1.02489E-26
ENSGALG00000007516		13	8.82333E-28	1.03049E-26
ENSGALG00000041568	AVL9	2	8.8393E-28	1.03136E-26
ENSGALG00000005782	GCLM	8	8.90212E-28	1.03762E-26
ENSGALG00000040212	FYCO1	2	8.9099E-28	1.03762E-26
ENSGALG00000014286	PDS5A	4	8.97823E-28	1.04458E-26
ENSGALG00000009645	ESRRG	3	8.99399E-28	1.04541E-26
ENSGALG00000013865	IFNGR1	3	9.03328E-28	1.04898E-26
ENSGALG00000033626		2	9.13461E-28	1.05974E-26
ENSGALG00000009570	ATP6V1D	5	9.29322E-28	1.07711E-26
ENSGALG00000001253	RC3H2	17	9.34603E-28	1.0822E-26

ENSGALG00000010199	MFSD8	4	9.44256E-28	1.09235E-26
ENSGALG00000006856	CD151	5	9.73349E-28	1.12493E-26
ENSGALG00000016147	ZBTB21	1	9.91841E-28	1.14522E-26
ENSGALG00000025819	UQCRFS1	11	9.96601E-28	1.14963E-26
ENSGALG00000017181	DCUN1D5	1	1.01016E-27	1.16416E-26
ENSGALG00000047248	DESI1	1	1.01176E-27	1.16491E-26
ENSGALG00000005612	CCM2	2	1.02429E-27	1.17822E-26
ENSGALG00000008725		3	1.03274E-27	1.18683E-26
ENSGALG00000000122	GNB2L1	16	1.03729E-27	1.19093E-26
ENSGALG00000006420	ABHD17C	10	1.05687E-27	1.21227E-26
ENSGALG00000009886	USP38	4	1.05871E-27	1.21324E-26
ENSGALG00000006682	BEND7	1	1.07049E-27	1.22558E-26
ENSGALG00000038278	TRIM7.2	16	1.08046E-27	1.23584E-26
ENSGALG00000030384		3	1.09571E-27	1.25211E-26
ENSGALG00000010581	SEL1L	5	1.12141E-27	1.28028E-26
ENSGALG00000011047	LEPROT	8	1.13673E-27	1.29655E-26
ENSGALG00000008707	KAT14	3	1.16036E-27	1.32226E-26
ENSGALG00000054703	BOLA1	25	1.17395E-27	1.33651E-26
ENSGALG00000012863	ADCK2	1	1.18177E-27	1.34415E-26
ENSGALG00000047807	DMBT1L	31	1.19431E-27	1.35715E-26
ENSGALG00000027886	TMEM165	4	1.19555E-27	1.3573E-26
ENSGALG00000034803		2	1.19867E-27	1.35958E-26
ENSGALG00000009056	LCLAT1	3	1.20326E-27	1.3631E-26
ENSGALG00000006161	CHMP1A	11	1.20402E-27	1.3631E-26
ENSGALG00000001028		19	1.20779E-27	1.3661E-26
ENSGALG00000004991	MKRN2	12	1.2201E-27	1.37874E-26
ENSGALG00000026722	UNC119B	15	1.23634E-27	1.39581E-26
ENSGALG00000010446	EFCAB14	8	1.25564E-27	1.41629E-26
ENSGALG00000039861	PLXNB2	1	1.26185E-27	1.42197E-26
ENSGALG00000010027	CFL2	5	1.26348E-27	1.4225E-26
ENSGALG00000012579	BAZ2B	7	1.28328E-27	1.44345E-26
ENSGALG00000010325	PPP1R12A	1	1.28549E-27	1.4446E-26
ENSGALG00000033884	EEF2	28	1.28716E-27	1.44515E-26

ENSGALG00000035769		3	1.38243E-27	1.55068E-26
ENSGALG00000000233	SP2	27	1.40291E-27	1.57221E-26
ENSGALG00000008241	TAF5	6	1.40647E-27	1.57476E-26
ENSGALG00000010543	EPS15	8	1.42245E-27	1.59118E-26
ENSGALG00000054198		4	1.4507E-27	1.6213E-26
ENSGALG00000011650	NIFK	7	1.45499E-27	1.62461E-26
ENSGALG00000040696	PRCC	25	1.47577E-27	1.6463E-26
ENSGALG00000010535	SGMS2	4	1.49757E-27	1.66909E-26
ENSGALG00000010630	AHCTF1	3	1.50957E-27	1.68093E-26
ENSGALG00000039221		5	1.55006E-27	1.72444E-26
ENSGALG00000031404	FHIP2A	6	1.55226E-27	1.72531E-26
ENSGALG00000037443	CRAT	17	1.56195E-27	1.7345E-26
ENSGALG00000007599	AMER1	4	1.57021E-27	1.74209E-26
ENSGALG00000005904	TMED5	8	1.57969E-27	1.74907E-26
ENSGALG00000011432	CDC42BPB	5	1.57993E-27	1.74907E-26
ENSGALG00000028886		3	1.5808E-27	1.74907E-26
ENSGALG00000039450	SOS1	3	1.59378E-27	1.76183E-26
ENSGALG00000012342	SASH1	3	1.62487E-27	1.79457E-26
ENSGALG00000001229	SKI	21	1.65315E-27	1.82415E-26
ENSGALG00000003205	EIF4EBP1	22	1.66286E-27	1.83321E-26
ENSGALG00000026542	FAM217B	20	1.69985E-27	1.87229E-26
ENSGALG00000008010	PTPN1	20	1.7593E-27	1.93602E-26
ENSGALG00000004052	GPR107	17	1.81408E-27	1.9945E-26
ENSGALG00000007861	MAGT1	4	1.82329E-27	2.00283E-26
ENSGALG00000006623	ATP11C	4	1.87074E-27	2.05135E-26
ENSGALG00000010074	KDM4A	8	1.87083E-27	2.05135E-26
ENSGALG00000032305	DHX8	27	1.90266E-27	2.08438E-26
ENSGALG00000040006	TBL1X	1	1.94317E-27	2.12685E-26
ENSGALG00000011443	EXOC3L4	5	1.97907E-27	2.1642E-26
ENSGALG00000042071	VPS53	19	2.00792E-27	2.19378E-26
ENSGALG00000005589	SYNGR3	14	2.04398E-27	2.23111E-26
ENSGALG00000007127		5	2.04654E-27	2.23111E-26
ENSGALG00000047203		18	2.04758E-27	2.23111E-26

ENSGALG00000013773	FUCA2	3	2.08652E-27	2.27152E-26
ENSGALG00000041619	MTPN	1	2.08975E-27	2.273E-26
ENSGALG00000006860	KIF3A	13	2.09304E-27	2.27455E-26
ENSGALG00000034971	SLC45A4	2	2.12718E-27	2.30959E-26
ENSGALG00000001872	SDF4	21	2.18988E-27	2.37555E-26
ENSGALG00000004107	ITM2A	4	2.28465E-27	2.47616E-26
ENSGALG00000017165	CUL5	1	2.29474E-27	2.48488E-26
ENSGALG00000049517	CHMP2A	31	2.3181E-27	2.50795E-26
ENSGALG00000003131	DGKE	18	2.36472E-27	2.55612E-26
ENSGALG00000016639	KIF13B	3	2.38305E-27	2.57346E-26
ENSGALG00000008561	SMNDC1	6	2.38498E-27	2.57346E-26
ENSGALG00000008847	VPS54	3	2.4118E-27	2.6001E-26
ENSGALG00000026298	SERTAD2	3	2.43985E-27	2.62801E-26
ENSGALG00000005745	YTHDF1	20	2.56348E-27	2.75694E-26
ENSGALG00000005042	IQSEC1	12	2.56407E-27	2.75694E-26
ENSGALG00000054191		1	2.57674E-27	2.76813E-26
ENSGALG00000003634	PTEN	6	2.59921E-27	2.78981E-26
ENSGALG00000037726	MARCKS	3	2.64467E-27	2.8361E-26
ENSGALG00000005269	INTS2	19	2.64906E-27	2.83831E-26
ENSGALG00000006848	ZSWIM3	20	2.67088E-27	2.85918E-26
ENSGALG00000001315	UBE2Z	27	2.69725E-27	2.88488E-26
ENSGALG00000002514	SFPQ	23	2.75704E-27	2.94624E-26
ENSGALG00000052210		9	2.77548E-27	2.96334E-26
ENSGALG00000004204	MINDY2	10	2.83178E-27	3.02081E-26
ENSGALG00000009428	SUSD6	5	2.84593E-27	3.03325E-26
ENSGALG00000015225	BLZF1	1	2.8486E-27	3.03345E-26
ENSGALG00000008485		7	2.91206E-27	3.09832E-26
ENSGALG00000028762	ZFYVE26	5	2.97122E-27	3.1585E-26
ENSGALG00000006799	AP2A2	5	3.00411E-27	3.19069E-26
ENSGALG00000002180	CHFR	15	3.02205E-27	3.20695E-26
ENSGALG00000032159	FBXL14	1	3.07508E-27	3.26039E-26
ENSGALG00000003976	BCORL1	4	3.08311E-27	3.26606E-26
ENSGALG00000007703		4	3.19755E-27	3.38436E-26

ENSGALG00000020793	UBE2E3	7	3.23243E-27	3.41831E-26
ENSGALG00000015402	C3orf38	1	3.31506E-27	3.50264E-26
ENSGALG00000040156	CCDC88C	5	3.37668E-27	3.56466E-26
ENSGALG00000001682	FBXL20	27	3.43448E-27	3.62254E-26
ENSGALG00000036867	CBX6	1	3.50824E-27	3.69715E-26
ENSGALG00000002090	GPSM2	8	3.52681E-27	3.71351E-26
ENSGALG00000003272	KHDRBS1	23	3.55966E-27	3.74486E-26
ENSGALG00000012336	STXBP5	3	3.56636E-27	3.74868E-26
ENSGALG00000015355	CD47	1	3.58811E-27	3.76829E-26
ENSGALG00000010536	FAF1	8	3.64366E-27	3.82333E-26
ENSGALG00000006033	GORASP1	2	3.65622E-27	3.83321E-26
ENSGALG00000009569	TLK1	7	3.77272E-27	3.95196E-26
ENSGALG00000011922	PCNX4	5	4.02619E-27	4.21286E-26
ENSGALG00000010019	TMEM184C	4	4.0287E-27	4.21286E-26
ENSGALG00000001720		12	4.06475E-27	4.24465E-26
ENSGALG00000026958	FPGT	8	4.06606E-27	4.24465E-26
ENSGALG00000004929	GID4	14	4.14757E-27	4.32603E-26
ENSGALG00000041641	HMG20A	10	4.31445E-27	4.49625E-26
ENSGALG00000005820	RNF130	13	4.34149E-27	4.52056E-26
ENSGALG00000005139	CLTC	19	4.35055E-27	4.52613E-26
ENSGALG00000029498	NUDT19	11	4.39913E-27	4.57277E-26
ENSGALG00000012966	ZBTB2	3	4.49648E-27	4.66998E-26
ENSGALG00000015877	SH3BGRL2	3	4.51051E-27	4.68057E-26
ENSGALG00000011904	TRMT5	5	4.526E-27	4.69265E-26
ENSGALG00000008180	SPG11	10	4.67899E-27	4.84714E-26
ENSGALG00000026728		9	4.72456E-27	4.8902E-26
ENSGALG00000011573	MOB1B	4	4.75427E-27	4.91678E-26
ENSGALG00000005999	ABTB1	12	4.80221E-27	4.96215E-26
ENSGALG00000044780		7	4.81154E-27	4.96758E-26
ENSGALG00000043711	AGO3	23	5.02522E-27	5.1838E-26
ENSGALG00000039228	ZFP36L1	5	5.10417E-27	5.26079E-26
ENSGALG00000039316	FAM120A	12	5.14926E-27	5.30278E-26
ENSGALG00000050582		5	5.2288E-27	5.38015E-26

ENSGALG00000002085	FZR1	28	5.26211E-27	5.40986E-26
ENSGALG00000006152		20	5.30317E-27	5.44748E-26
ENSGALG00000013102	COBL	2	5.33267E-27	5.47317E-26
ENSGALG00000039108	MAP1S	28	5.43792E-27	5.5765E-26
ENSGALG00000015699	NELFA	4	5.47421E-27	5.60899E-26
ENSGALG00000036789	ADGRL2	8	5.7E-27	5.83544E-26
ENSGALG00000014834	NCOA7	3	5.71371E-27	5.84457E-26
ENSGALG00000029836	GPLD1	2	5.72222E-27	5.84837E-26
ENSGALG00000011579		3	5.76821E-27	5.89044E-26
ENSGALG00000014935	GREB1L	2	5.83676E-27	5.95544E-26
ENSGALG00000000086	TIMM17B	26	5.85092E-27	5.96491E-26
ENSGALG00000041650	DUSP7	12	5.98703E-27	6.09857E-26
ENSGALG00000010612	PRKD3	3	6.03562E-27	6.14293E-26
ENSGALG00000020419	LIN52	5	6.12965E-27	6.23343E-26
ENSGALG00000005340	SLC35A3	8	6.14082E-27	6.23959E-26
ENSGALG00000013205	ETNK1	1	6.1578E-27	6.25163E-26
ENSGALG00000011621	IGF2R	3	6.19621E-27	6.28539E-26
ENSGALG00000015665	SH3BP2	4	6.22932E-27	6.31372E-26
ENSGALG00000031975	UBE2H	1	6.30961E-27	6.38979E-26
ENSGALG00000006437	NOM1	2	6.32265E-27	6.39768E-26
ENSGALG00000054017		12	6.35845E-27	6.42857E-26
ENSGALG00000036301	KIAA0895	2	6.37756E-27	6.44255E-26
ENSGALG00000001030	SCAMP4	28	6.58042E-27	6.64198E-26
ENSGALG00000012704	MYLIP	2	6.7468E-27	6.80428E-26
ENSGALG00000015304	SNX3	3	6.7629E-27	6.81488E-26
ENSGALG00000052837	GNA12	14	6.80044E-27	6.84705E-26
ENSGALG00000050948	TMEM115	12	6.82069E-27	6.86178E-26
ENSGALG00000001803	IWS1	9	6.86087E-27	6.8965E-26
ENSGALG00000015316	C3H6ORF203	3	6.94342E-27	6.97374E-26
ENSGALG00000032234	MARCH5	6	7.03076E-27	7.05564E-26
ENSGALG00000029387	PURA	13	7.40793E-27	7.42803E-26
ENSGALG00000003518	GPAT4	22	7.78064E-27	7.79534E-26
ENSGALG00000003144	TRIM25	18	7.80275E-27	7.81108E-26

ENSGALG00000049027		8	8.10996E-27	8.11195E-26
ENSGALG00000003819		14	8.21505E-27	8.21033E-26
ENSGALG00000022030	PGAP3	27	8.26487E-27	8.25336E-26
ENSGALG00000043092	MYH9	1	8.43723E-27	8.41859E-26
ENSGALG00000015538	KIAA0232	4	8.48453E-27	8.45887E-26
ENSGALG00000006982	EEF2K	14	8.53514E-27	8.49975E-26
ENSGALG00000026944	ARF6	5	8.53949E-27	8.49975E-26
ENSGALG00000000883	MAPKAPK2	26	8.65563E-27	8.60525E-26
ENSGALG00000025796	ADI1	3	8.65959E-27	8.60525E-26
ENSGALG00000012395	PLEKHG1	3	8.67617E-27	8.6147E-26
ENSGALG00000008934	PIK3CA	9	8.69617E-27	8.62753E-26
ENSGALG00000022739	AKAP11	1	9.09322E-27	9.01412E-26
ENSGALG00000038026	AEBP2	1	9.22626E-27	9.13857E-26
ENSGALG00000016732		3	9.25104E-27	9.15568E-26
ENSGALG00000001219	PIGU	20	9.37571E-27	9.27153E-26
ENSGALG00000031637	ICOSLG	1	9.44204E-27	9.32957E-26
ENSGALG00000035845	JPH1	2	9.68474E-27	9.56162E-26
ENSGALG00000004345	OIT3	6	1.02146E-26	1.00766E-25
ENSGALG00000046159	KNG1	9	1.04063E-26	1.02574E-25
ENSGALG00000041821	NAA25	15	1.04483E-26	1.02905E-25
ENSGALG00000013187	CLPTM1L	2	1.06544E-26	1.0485E-25
ENSGALG00000030691	PPM1B	3	1.07164E-26	1.05375E-25
ENSGALG00000012148	ATG14	5	1.08533E-26	1.06635E-25
ENSGALG00000006586	PLAGL2	20	1.08789E-26	1.06801E-25
ENSGALG00000017239	CTSC	1	1.10111E-26	1.08012E-25
ENSGALG00000004172	BET1L	5	1.10699E-26	1.08501E-25
ENSGALG00000005243	PPP3CB	6	1.12135E-26	1.0982E-25
ENSGALG00000035563	USP6NL	1	1.12358E-26	1.09912E-25
ENSGALG00000039300	NFAT5	11	1.12409E-26	1.09912E-25
ENSGALG00000006062	ZBTB46	20	1.14283E-26	1.11655E-25
ENSGALG00000004272	ASB1	7	1.16517E-26	1.13746E-25
ENSGALG00000040373	EXOSC2	17	1.20828E-26	1.1786E-25
ENSGALG00000008181	MADD	5	1.21862E-26	1.18774E-25

ENSGALG00000028301		18	1.25214E-26	1.21943E-25
ENSGALG00000035701	SMAD3	10	1.26321E-26	1.22833E-25
ENSGALG00000053990		12	1.2633E-26	1.22833E-25
ENSGALG00000033759	RAVER2	8	1.26526E-26	1.22833E-25
ENSGALG00000001730	DGKD	9	1.26531E-26	1.22833E-25
ENSGALG00000006403	MESDC2	10	1.27733E-26	1.23902E-25
ENSGALG00000039818	GLG1	11	1.28584E-26	1.24629E-25
ENSGALG00000016245	BCOR	1	1.29768E-26	1.25676E-25
ENSGALG00000003880	STX10	8	1.30713E-26	1.26491E-25
ENSGALG00000004918	RAB9B	4	1.30825E-26	1.26499E-25
ENSGALG00000032181	LRP4	5	1.3379E-26	1.29263E-25
ENSGALG00000001207	PLCH2	21	1.34545E-26	1.2989E-25
ENSGALG00000034376		2	1.35733E-26	1.30933E-25
ENSGALG00000006657	MAPRE1	20	1.35867E-26	1.30959E-25
ENSGALG00000004428		11	1.3684E-26	1.31792E-25
ENSGALG00000010840	SNX13	2	1.38497E-26	1.33283E-25
ENSGALG00000006929	ZNF711	4	1.39555E-26	1.34195E-25
ENSGALG00000038054	FAM175B	6	1.397E-26	1.34229E-25
ENSGALG00000039196	NECTIN1	24	1.40923E-26	1.35297E-25
ENSGALG00000016697	MMUT	3	1.44754E-26	1.38866E-25
ENSGALG00000032382	UBA5	2	1.45338E-26	1.39316E-25
ENSGALG00000007924	STK17B	7	1.47327E-26	1.41112E-25
ENSGALG00000029512	SPTBN1	3	1.5452E-26	1.47886E-25
ENSGALG00000035129	RBM12B	2	1.55039E-26	1.48266E-25
ENSGALG00000007716	SLC37A4	24	1.56283E-26	1.49339E-25
ENSGALG00000007595	LRCH3	9	1.58856E-26	1.51679E-25
ENSGALG00000009379	SEC62	9	1.61084E-26	1.53685E-25
ENSGALG00000040783		2	1.62208E-26	1.54636E-25
ENSGALG00000003325		15	1.65727E-26	1.57867E-25
ENSGALG00000017105	RNF6	1	1.66117E-26	1.58116E-25
ENSGALG00000014152	KMT5B	5	1.69453E-26	1.61166E-25
ENSGALG00000029435	CYP7B1	2	1.7159E-26	1.63071E-25
ENSGALG00000030598	BTAF1	6	1.72414E-26	1.63726E-25

ENSGALG00000003498	TAF8	26	1.7289E-26	1.6405E-25
ENSGALG00000006604	KIF3B	20	1.81655E-26	1.72233E-25
ENSGALG00000002904	REEP3	6	1.82561E-26	1.72958E-25
ENSGALG00000040475	ATMIN	11	1.83712E-26	1.73913E-25
ENSGALG00000039139	TNS3	2	1.86049E-26	1.75989E-25
ENSGALG00000004387	CCPG1	10	1.90171E-26	1.79749E-25
ENSGALG00000031281		3	1.93155E-26	1.82427E-25
ENSGALG00000034016		32	1.94326E-26	1.83391E-25
ENSGALG00000015353	BBX	1	1.96873E-26	1.85652E-25
ENSGALG00000028512	LCOR	6	2.00408E-26	1.88838E-25
ENSGALG00000030070	QDPR	4	2.02434E-26	1.90601E-25
ENSGALG00000040272	ALG10	1	2.03303E-26	1.91199E-25
ENSGALG00000039208	PHF2	12	2.03383E-26	1.91199E-25
ENSGALG00000038556	CDC42EP1	1	2.04354E-26	1.91963E-25
ENSGALG00000005753		4	2.14441E-26	2.01284E-25
ENSGALG00000009814	NSL1	3	2.18846E-26	2.0526E-25
ENSGALG00000004850	ZMIZ1	6	2.22366E-26	2.08402E-25
ENSGALG00000030487		12	2.24979E-26	2.10689E-25
ENSGALG00000017038	PROSER1	1	2.30048E-26	2.1527E-25
ENSGALG00000038704	KBTBD2	2	2.30481E-26	2.1551E-25
ENSGALG00000016779	RNF149	1	2.30813E-26	2.15655E-25
ENSGALG00000039583	MITF	12	2.33428E-26	2.17932E-25
ENSGALG00000005699	NLK	19	2.36112E-26	2.20216E-25
ENSGALG00000011168	C12orf29	1	2.36236E-26	2.20216E-25
ENSGALG00000027262	CAB39	9	2.39283E-26	2.22886E-25
ENSGALG00000012239	METAP1	4	2.51098E-26	2.33713E-25
ENSGALG00000003396	NFATC3	11	2.54238E-26	2.36455E-25
ENSGALG00000052601		3	2.55704E-26	2.37637E-25
ENSGALG00000012569	FBXO7	1	2.59503E-26	2.40984E-25
ENSGALG00000026850	TICAM1	28	2.60865E-26	2.42064E-25
ENSGALG00000003247	NFYA	26	2.61526E-26	2.42493E-25
ENSGALG00000015258	DCBLD2	1	2.66947E-26	2.47332E-25
ENSGALG00000016834	MCF2L	1	2.72389E-26	2.52182E-25

ENSGALG00000041104	USP20	17	2.77964E-26	2.57148E-25
ENSGALG00000009020	JAG1	3	2.78232E-26	2.57201E-25
ENSGALG00000001972		8	2.79252E-26	2.57949E-25
ENSGALG00000002141	UGGT1	9	2.79681E-26	2.58149E-25
ENSGALG00000002723	ANKS1A	26	3.05519E-26	2.81784E-25
ENSGALG00000030791	DIAPH2	4	3.11778E-26	2.8734E-25
ENSGALG00000008513	C1GALT1C1	4	3.14549E-26	2.89675E-25
ENSGALG00000007023	PRKAR2A	12	3.17192E-26	2.91889E-25
ENSGALG00000005251	CMTM4	11	3.20718E-26	2.94911E-25
ENSGALG00000033246	ZNF740	33	3.24467E-26	2.98134E-25
ENSGALG00000031987	MYO5A	10	3.3037E-26	3.03328E-25
ENSGALG00000008269	ZC3H8	3	3.40354E-26	3.1226E-25
ENSGALG00000009158	CDC42BPA	3	3.47224E-26	3.18324E-25
ENSGALG00000009217	TBC1D24	14	3.52382E-26	3.2281E-25
ENSGALG00000013036	ATP6V1E1	1	3.59345E-26	3.28941E-25
ENSGALG00000001725	TNRC6C	18	3.61827E-26	3.30965E-25
ENSGALG00000007771	FAM210B	20	3.6421E-26	3.32895E-25
ENSGALG00000012049	ZNF148	7	3.65606E-26	3.3392E-25
ENSGALG00000031212	MGAT2	5	3.70377E-26	3.38024E-25
ENSGALG00000041362	SECISBP2L	10	3.74192E-26	3.41251E-25
ENSGALG00000017145	MPHOSPH8	1	3.78263E-26	3.44519E-25
ENSGALG00000051398		2	3.78341E-26	3.44519E-25
ENSGALG00000016996	ITM2B	1	3.86199E-26	3.51412E-25
ENSGALG00000004079	ENOX2	4	3.87084E-26	3.51955E-25
ENSGALG00000035026	SLC22A4	13	3.88964E-26	3.53401E-25
ENSGALG00000001110		19	4.20627E-26	3.81885E-25
ENSGALG00000016547	TXLNG	1	4.27181E-26	3.87546E-25
ENSGALG00000043447	LAS1L	4	4.31035E-26	3.90752E-25
ENSGALG00000039720	MAP4K3	3	4.38926E-26	3.9761E-25
ENSGALG00000028967	DNAJC27	3	4.45534E-26	4.03296E-25
ENSGALG00000008011	SLC25A19	18	4.46919E-26	4.0425E-25
ENSGALG00000031415	SLC30A9	4	4.49489E-26	4.06272E-25
ENSGALG00000020003	MAP3K4	3	4.59867E-26	4.15345E-25

ENSGALG00000015163	RNF138	2	4.68768E-26	4.23071E-25
ENSGALG00000007332	PRICKLE2	12	4.72107E-26	4.25769E-25
ENSGALG00000007331	SVIL	2	4.77506E-26	4.3032E-25
ENSGALG00000036477	CHPF2	2	4.78636E-26	4.3102E-25
ENSGALG00000028994	SAP30L	13	4.86681E-26	4.37941E-25
ENSGALG00000019570	C1orf145	2	5.02144E-26	4.51522E-25
ENSGALG00000008341	FURIN	10	5.05857E-26	4.54526E-25
ENSGALG00000034358	RETSAT	22	5.08016E-26	4.5613E-25
ENSGALG00000041267	BMI1	2	5.10922E-26	4.58402E-25
ENSGALG00000006121	PIK3C2A	5	5.12326E-26	4.59323E-25
ENSGALG00000010412	WWTR1	9	5.18729E-26	4.64722E-25
ENSGALG00000017096	LNX2	1	5.20455E-26	4.65925E-25
ENSGALG00000029198	YDJC	15	5.22206E-26	4.6715E-25
ENSGALG00000012799	RREB1	2	5.25005E-26	4.6931E-25
ENSGALG00000010671	HNRNPU	3	5.27242E-26	4.70964E-25
ENSGALG00000031821	C15H12orf49	15	5.361E-26	4.78526E-25
ENSGALG00000026862	CLDN1	9	5.62799E-26	5.0199E-25
ENSGALG00000008439	CD36	1	5.65143E-26	5.03713E-25
ENSGALG00000036931	KLHL20	8	5.66988E-26	5.04988E-25
ENSGALG00000012392	IYD	3	5.67706E-26	5.05259E-25
ENSGALG00000032868	AHCYL2	1	5.71226E-26	5.0802E-25
ENSGALG00000037485	QTRT2	1	5.97844E-26	5.31306E-25
ENSGALG00000007130	RAC1	14	6.04991E-26	5.37266E-25
ENSGALG00000039611		14	6.37746E-26	5.65941E-25
ENSGALG00000044480	IL13RA1	4	6.46451E-26	5.73249E-25
ENSGALG00000007989	ADNP	20	6.46956E-26	5.7328E-25
ENSGALG00000011990	USP53	4	6.65202E-26	5.8902E-25
ENSGALG00000007224	HGS	18	6.68816E-26	5.9179E-25
ENSGALG00000006461	UBE3C	2	6.93193E-26	6.12916E-25
ENSGALG00000013452	ERP44	2	6.99855E-26	6.18358E-25
ENSGALG00000047283		31	7.07121E-26	6.24325E-25
ENSGALG00000001038	MAN1C1	23	7.10827E-26	6.27143E-25
ENSGALG00000038768	FAM20C	14	7.12232E-26	6.27928E-25

ENSGALG00000051534		18	7.14958E-26	6.29876E-25
ENSGALG00000051276	GATAD2B	25	7.18778E-26	6.32784E-25
ENSGALG00000046412		27	7.27728E-26	6.40201E-25
ENSGALG00000004220	SERINC3	20	7.28301E-26	6.40243E-25
ENSGALG00000008050	HBP1	1	7.29301E-26	6.4066E-25
ENSGALG00000005702	TMEM97	19	7.394E-26	6.49064E-25
ENSGALG00000002981	ERCC4	14	7.7528E-26	6.80071E-25
ENSGALG00000034230	SWT1	8	7.81779E-26	6.85279E-25
ENSGALG00000008405	RALBP1	2	7.86453E-26	6.88881E-25
ENSGALG00000006648	SPECC1L	15	7.96961E-26	6.97584E-25
ENSGALG00000003412		17	8.19747E-26	7.17014E-25
ENSGALG00000010287	IPP	8	8.21802E-26	7.18296E-25
ENSGALG00000007788	ABCB7	4	8.34996E-26	7.29306E-25
ENSGALG00000039037	PICALM	1	8.47039E-26	7.39295E-25
ENSGALG00000007078	ENTPD1	6	8.61606E-26	7.5138E-25
ENSGALG00000029935		2	8.62118E-26	7.5138E-25
ENSGALG00000004347	NEDD4	10	8.73661E-26	7.60896E-25
ENSGALG00000004701	SET	17	8.91237E-26	7.7565E-25
ENSGALG00000004597	ZBTB37	8	9.03472E-26	7.85737E-25
ENSGALG00000003663	RBL2	11	9.06589E-26	7.87886E-25
ENSGALG00000004352	PSMD9	15	9.1849E-26	7.9766E-25
ENSGALG00000003427	USP3	10	9.20921E-26	7.99202E-25
ENSGALG00000005850	FNBP1L	8	9.21939E-26	7.99517E-25
ENSGALG00000035013	WDR11	6	9.24316E-26	8.01008E-25
ENSGALG00000015635	HTT	4	9.54584E-26	8.26651E-25
ENSGALG00000036238	LUC7L2	1	9.67921E-26	8.37606E-25
ENSGALG00000007642	CTBPL	13	9.69557E-26	8.38426E-25
ENSGALG00000003788	BNIP2	10	9.91305E-26	8.56626E-25
ENSGALG00000006068	TNRC6A	14	1.00379E-25	8.66798E-25
ENSGALG00000027835	PCBD1	6	1.0173E-25	8.77841E-25
ENSGALG00000030843	COPS5	2	1.01901E-25	8.78698E-25
ENSGALG00000012689	KIF13A	2	1.03428E-25	8.91234E-25
ENSGALG00000004326	GOSR1	19	1.03958E-25	8.95166E-25

ENSGALG00000026221	ENDOG	17	1.04157E-25	8.9625E-25
ENSGALG00000003705	VPS13C	10	1.05984E-25	9.11326E-25
ENSGALG00000035610	PPM1D	19	1.071E-25	9.20272E-25
ENSGALG00000030263	RABL6	17	1.07977E-25	9.27156E-25
ENSGALG00000041760	CLTCL1	15	1.1E-25	9.43862E-25
ENSGALG00000033260	ST3GAL5	4	1.11571E-25	9.56674E-25
ENSGALG00000034831	YTHDF2	23	1.11776E-25	9.57755E-25
ENSGALG00000009260	TMEM62	5	1.12408E-25	9.6249E-25
ENSGALG00000004467	CLIP1	15	1.13191E-25	9.68519E-25
ENSGALG00000041139	PINX1	3	1.13697E-25	9.72169E-25
ENSGALG00000009858	SLC30A1	3	1.15356E-25	9.85657E-25
ENSGALG00000008005	RIPOR3	20	1.16931E-25	9.98417E-25
ENSGALG00000004363		7	1.17839E-25	1.00547E-24
ENSGALG00000038395	PDE7A	2	1.20074E-25	1.02382E-24
ENSGALG00000005622	ARF4	12	1.216E-25	1.03611E-24
ENSGALG00000005700	GID8	20	1.25228E-25	1.06628E-24
ENSGALG00000004140	SH3BP4	7	1.27881E-25	1.0881E-24
ENSGALG00000037943	PRKCB	14	1.31752E-25	1.12026E-24
ENSGALG00000030580	RPS6KA5	5	1.32291E-25	1.12406E-24
ENSGALG00000009637	EMC7	5	1.33125E-25	1.13036E-24
ENSGALG00000040128	SNAP29	15	1.3518E-25	1.14701E-24
ENSGALG00000012281	PLA2G6	1	1.37876E-25	1.16907E-24
ENSGALG00000008146	MORF4L2	10	1.38552E-25	1.17399E-24
ENSGALG00000037200		3	1.4228E-25	1.20473E-24
ENSGALG00000006127	FAM122A	4	1.42638E-25	1.20693E-24
ENSGALG00000007952	AMOT	4	1.42915E-25	1.20844E-24
ENSGALG00000010999	DICER1	5	1.45656E-25	1.23076E-24
ENSGALG00000046235		2	1.46508E-25	1.2371E-24
ENSGALG00000011533	XRCC3	5	1.48273E-25	1.25114E-24
ENSGALG00000034413	PIAS4	28	1.48483E-25	1.25204E-24
ENSGALG00000010047	SLC4A1AP	3	1.48628E-25	1.2524E-24
ENSGALG00000007370	CLP1	5	1.5291E-25	1.28759E-24
ENSGALG00000047929	SLC44A2	30	1.56139E-25	1.31387E-24

ENSGALG00000025864	WTIP	11	1.57027E-25	1.32044E-24
ENSGALG00000013129	RPRD1A	2	1.61006E-25	1.35296E-24
ENSGALG00000005626	TPST2	15	1.65935E-25	1.39342E-24
ENSGALG00000016631	INTS9	3	1.66597E-25	1.39802E-24
ENSGALG00000007748	SLC16A2	4	1.69756E-25	1.42355E-24
ENSGALG00000015709	TACC3	4	1.703E-25	1.42712E-24
ENSGALG00000041969	MTSS1	2	1.71658E-25	1.43751E-24
ENSGALG00000021611		19	1.73698E-25	1.4536E-24
ENSGALG00000005200	ADRM1	20	1.74274E-25	1.45742E-24
ENSGALG00000013197	GALNT12	2	1.7555E-25	1.46709E-24
ENSGALG00000037521	PRCP	1	1.7751E-25	1.48245E-24
ENSGALG00000033867	PCOLCE	30	1.80181E-25	1.50373E-24
ENSGALG00000036543	ZBTB38	9	1.82193E-25	1.51948E-24
ENSGALG00000000975	C1orf174	21	1.83657E-25	1.53064E-24
ENSGALG00000002478	MATR3	13	1.83791E-25	1.53071E-24
ENSGALG00000006925	CCP110	14	1.84628E-25	1.53663E-24
ENSGALG00000043265	ZC3H3	2	1.88359E-25	1.56661E-24
ENSGALG00000004957	RHBDD1	9	1.88978E-25	1.57069E-24
ENSGALG00000028587	TMX4	3	1.90719E-25	1.58409E-24
ENSGALG00000005982	KLHL9	4	1.91046E-25	1.58572E-24
ENSGALG00000016451	ROCK2	3	1.92107E-25	1.59345E-24
ENSGALG00000008983	GNB4	9	1.95103E-25	1.61719E-24
ENSGALG00000048159		17	1.98043E-25	1.64045E-24
ENSGALG00000016050	TTC3	1	2.01093E-25	1.66458E-24
ENSGALG00000046150	DIP2A	7	2.11045E-25	1.74578E-24
ENSGALG00000000816		26	2.11504E-25	1.74839E-24
ENSGALG00000009453	MARC1	3	2.12027E-25	1.75152E-24
ENSGALG00000035287	STAM	2	2.139E-25	1.7658E-24
ENSGALG00000011745		1	2.16231E-25	1.78383E-24
ENSGALG00000012272	TMEM184B	1	2.16608E-25	1.78573E-24
ENSGALG00000016675	PAQR8	3	2.17898E-25	1.79428E-24
ENSGALG00000002408	NCDN	23	2.17939E-25	1.79428E-24
ENSGALG00000008867	COMMD5	4	2.34058E-25	1.92569E-24

ENSGALG00000042201	TP53BP1	10	2.35717E-25	1.93803E-24
ENSGALG00000007025	CPNE8	1	2.36398E-25	1.94204E-24
ENSGALG00000009982	ZNF827	4	2.36524E-25	1.94204E-24
ENSGALG00000009796	RPS6KC1	3	2.44574E-25	2.00679E-24
ENSGALG00000011133	LIN54	4	2.46439E-25	2.02073E-24
ENSGALG00000002942	JMJD1C	6	2.51605E-25	2.06171E-24
ENSGALG00000049492	ARL2BP	11	2.65237E-25	2.17195E-24
ENSGALG00000021220	PPP1R3D	20	2.70952E-25	2.21726E-24
ENSGALG00000016568	MOSPD2	1	2.79683E-25	2.28717E-24
ENSGALG00000001479	CYB5D2	19	2.81625E-25	2.30151E-24
ENSGALG00000003695	MINPP1	6	2.84737E-25	2.32539E-24
ENSGALG00000033993	EIF6	20	2.86679E-25	2.33967E-24
ENSGALG00000046712	LMNA	25	2.90658E-25	2.37056E-24
ENSGALG00000032036		1	2.94005E-25	2.39626E-24
ENSGALG00000029971	PGLYRP2	30	2.95866E-25	2.40982E-24
ENSGALG00000032342	MIGA2	17	3.07005E-25	2.49887E-24
ENSGALG00000004294	CGNL1	10	3.07907E-25	2.50431E-24
ENSGALG00000009920	COCH	5	3.08083E-25	2.50431E-24
ENSGALG00000033879	CAND1	1	3.12639E-25	2.53965E-24
ENSGALG00000013920	SUV39H2	1	3.22625E-25	2.61902E-24
ENSGALG00000018819		24	3.23888E-25	2.62752E-24
ENSGALG00000000790	DNAJC8	23	3.26668E-25	2.64831E-24
ENSGALG00000003862	LRRFIP1	7	3.41146E-25	2.76385E-24
ENSGALG00000007092	LYSMD4	10	3.43149E-25	2.77673E-24
ENSGALG00000003845	MKNK2	28	3.43191E-25	2.77673E-24
ENSGALG00000031582	RAB1B	3	3.45004E-25	2.78954E-24
ENSGALG00000002219	WBP2	18	3.46841E-25	2.80181E-24
ENSGALG00000004655	SMURF1	14	3.46981E-25	2.80181E-24
ENSGALG00000048263		30	3.48718E-25	2.81398E-24
ENSGALG00000027242	TTF1	17	3.60607E-25	2.90799E-24
ENSGALG00000047461		2	3.62311E-25	2.9198E-24
ENSGALG00000000811	IST1	11	3.65636E-25	2.94465E-24
ENSGALG00000017006	SETDB2	1	3.67256E-25	2.95574E-24

ENSGALG00000014963		3	3.69191E-25	2.96936E-24
ENSGALG00000007779	ADCY9	14	3.8034E-25	3.05701E-24
ENSGALG00000007479	SLC25A28	6	3.88192E-25	3.11807E-24
ENSGALG00000024081	TGIF2	20	3.96716E-25	3.18443E-24
ENSGALG00000015529	WFS1	4	4.09852E-25	3.28771E-24
ENSGALG00000002846	NEURL1B	13	4.17236E-25	3.34475E-24
ENSGALG00000008886	ZNF618	17	4.2675E-25	3.41876E-24
ENSGALG00000040687	SNX27	25	4.30349E-25	3.44534E-24
ENSGALG00000041034	MIA3	3	4.31982E-25	3.45614E-24
ENSGALG00000001826	WWC1	13	4.34487E-25	3.4739E-24
ENSGALG00000008903	ITPRID2	7	4.43628E-25	3.54466E-24
ENSGALG00000010501	SNW1	5	4.45961E-25	3.56097E-24
ENSGALG00000016506	ZNF512	3	4.48655E-25	3.58014E-24
ENSGALG00000004809	PPIF	6	4.56487E-25	3.64025E-24
ENSGALG00000001637	PSMD5	17	4.5718E-25	3.64339E-24
ENSGALG00000039164		10	4.64097E-25	3.69611E-24
ENSGALG00000040265	USP30	15	4.65778E-25	3.70707E-24
ENSGALG00000026677	F10	1	4.67849E-25	3.72112E-24
ENSGALG00000008049	ARL3	6	4.7312E-25	3.7606E-24
ENSGALG00000003398	ADAM9	22	4.73438E-25	3.76067E-24
ENSGALG00000003496	TEX2	18	4.74305E-25	3.7651E-24
ENSGALG00000007683		4	4.83861E-25	3.83846E-24
ENSGALG00000030563		21	4.89592E-25	3.8814E-24
ENSGALG00000049638	GATD1	5	4.91506E-25	3.89404E-24
ENSGALG00000013683	ARID1B	3	4.97926E-25	3.94235E-24
ENSGALG00000016735	TUBGCP5	1	5.03236E-25	3.9818E-24
ENSGALG00000015842	TIAM1	1	5.13348E-25	4.05917E-24
ENSGALG00000017296	KLHL42	1	5.20961E-25	4.11671E-24
ENSGALG00000010891	ABCB11	7	5.28455E-25	4.17322E-24
ENSGALG00000005830	MNT	19	5.36462E-25	4.23371E-24
ENSGALG00000014346	TSPAN9	1	5.44782E-25	4.29659E-24
ENSGALG00000039099	MAPK9	13	5.59793E-25	4.41212E-24
ENSGALG00000007877	PIP4K2A	2	5.602E-25	4.41248E-24

ENSGALG00000034132	CACFD1	17	5.71906E-25	4.50177E-24
ENSGALG00000009873	LEMD3	1	5.75763E-25	4.52922E-24
ENSGALG00000011343	CNPPD1	7	5.7634E-25	4.53083E-24
ENSGALG00000039958		1	5.7944E-25	4.55226E-24
ENSGALG00000030406	CDS2	22	5.84188E-25	4.58661E-24
ENSGALG00000007697	MGEA5	6	6.08753E-25	4.7764E-24
ENSGALG00000012898	RELCH	2	6.29515E-25	4.93613E-24
ENSGALG00000005283	SLC25A36	9	6.35269E-25	4.97804E-24
ENSGALG00000009228	PARP1	3	6.45222E-25	5.05279E-24
ENSGALG00000034044	TBC1D12	6	6.51176E-25	5.09615E-24
ENSGALG00000005597	CNN3	8	6.63526E-25	5.18946E-24
ENSGALG00000002836	PEX14	21	6.64008E-25	5.1899E-24
ENSGALG00000011705	BRF1	5	6.66434E-25	5.20552E-24
ENSGALG00000047190		8	6.7735E-25	5.28741E-24
ENSGALG00000012992	WNK1	1	6.83893E-25	5.33506E-24
ENSGALG00000014992	TMEM128	4	7.05239E-25	5.49807E-24
ENSGALG00000022166	PNKD	7	7.06726E-25	5.50614E-24
ENSGALG00000041216	RPUSD2	5	7.08021E-25	5.51271E-24
ENSGALG00000010975	TMTC2	1	7.29309E-25	5.67483E-24
ENSGALG00000008990	RADX	4	7.29826E-25	5.67524E-24
ENSGALG00000009229	ATP6V0C	14	7.44503E-25	5.78568E-24
ENSGALG00000011831	ANKRD50	4	7.45214E-25	5.78751E-24
ENSGALG00000000401	RBM15	26	7.84147E-25	6.086E-24
ENSGALG00000010806	RIN3	5	7.85837E-25	6.09524E-24
ENSGALG00000013352	SF3B4	25	7.95066E-25	6.1629E-24
ENSGALG00000004842	NT5M	14	7.98534E-25	6.18584E-24
ENSGALG00000010090	FHIP1A	4	8.34747E-25	6.46227E-24
ENSGALG00000008946	NHLRC2	6	8.35747E-25	6.4659E-24
ENSGALG00000051040	TMEM251	5	8.38042E-25	6.47955E-24
ENSGALG00000014084	FGFR1OP2	1	8.71165E-25	6.73137E-24
ENSGALG00000032992	NR3C1	13	9.02524E-25	6.96927E-24
ENSGALG00000028530		8	9.10898E-25	7.02948E-24
ENSGALG00000039811	NIPA1	1	9.2385E-25	7.12493E-24

ENSGALG00000004156	KIF1BP	6	9.35902E-25	7.21331E-24
ENSGALG00000038904	ARL6IP5	12	9.58232E-25	7.38075E-24
ENSGALG00000017065	STARD13	1	9.81718E-25	7.55687E-24
ENSGALG00000034834	WWC3	1	9.9512E-25	7.6552E-24
ENSGALG00000033927	MFSD5	33	9.98027E-25	7.67272E-24
ENSGALG00000016799	NCK1	1	1.00474E-24	7.71948E-24
ENSGALG00000010350	GPATCH2L	5	1.01399E-24	7.78564E-24
ENSGALG00000053363	CTNNBIP1	21	1.01678E-24	7.80211E-24
ENSGALG00000012426	DDHD1	5	1.02023E-24	7.82366E-24
ENSGALG00000013408	TMF1	12	1.02543E-24	7.85861E-24
ENSGALG00000037177	TSNAX	3	1.03649E-24	7.9384E-24
ENSGALG00000000672	SCMH1	23	1.04483E-24	7.99721E-24
ENSGALG00000007008	POLR3E	14	1.04808E-24	8.01705E-24
ENSGALG00000010183	RAB21	1	1.05178E-24	8.04037E-24
ENSGALG00000017309	RNF169	1	1.08403E-24	8.28167E-24
ENSGALG00000028227	SLC25A20	12	1.10016E-24	8.39969E-24
ENSGALG00000008621	NRP2	7	1.10471E-24	8.42913E-24
ENSGALG00000007381		5	1.12062E-24	8.54518E-24
ENSGALG00000005079	VCL	6	1.13476E-24	8.64757E-24
ENSGALG00000013935	BCLAF1	3	1.14246E-24	8.7008E-24
ENSGALG00000008021	RNF114	20	1.17468E-24	8.94065E-24
ENSGALG00000002922	NOTCH2	8	1.22044E-24	9.2827E-24
ENSGALG00000005647	ABCD3	8	1.22115E-24	9.2827E-24
ENSGALG00000033618	MYO9A	10	1.22706E-24	9.32181E-24
ENSGALG00000005722	SEC31B	6	1.22871E-24	9.32853E-24
ENSGALG00000043036	VASH1	5	1.26172E-24	9.57323E-24
ENSGALG00000030139	SIDT2	24	1.26671E-24	9.60509E-24
ENSGALG00000014410	CSTB	1	1.27282E-24	9.64545E-24
ENSGALG00000026424	TOR2A	17	1.2794E-24	9.68929E-24
ENSGALG00000020410	TMED8	5	1.3126E-24	9.93457E-24
ENSGALG00000008942	FOXN2	3	1.31929E-24	9.97894E-24
ENSGALG00000004902	TOM1L2	14	1.32068E-24	9.98329E-24
ENSGALG00000011342	FGD6	1	1.3298E-24	1.0046E-23

ENSGALG00000001995	LIMS2	9	1.35052E-24	1.01962E-23
ENSGALG00000015713	FAM53A	4	1.4436E-24	1.08922E-23
ENSGALG00000006312	TSG101	5	1.45402E-24	1.09641E-23
ENSGALG00000040489		33	1.46789E-24	1.10618E-23
ENSGALG00000038338	GLYR1	14	1.49497E-24	1.12589E-23
ENSGALG00000008487	TMCC1	12	1.51016E-24	1.13663E-23
ENSGALG00000036380	SLC48A1	33	1.51974E-24	1.14313E-23
ENSGALG00000012484	RIF1	7	1.58531E-24	1.19172E-23
ENSGALG00000016475		3	1.59833E-24	1.20076E-23
ENSGALG00000038924	IDE	6	1.62288E-24	1.21846E-23
ENSGALG00000009813	SCOC	4	1.64379E-24	1.2334E-23
ENSGALG00000014192	ATP10D	4	1.69898E-24	1.27403E-23
ENSGALG00000006613	CTSD	5	1.71078E-24	1.28209E-23
ENSGALG00000042400	CASK	1	1.73043E-24	1.29584E-23
ENSGALG00000034396	TMEM50B	1	1.73126E-24	1.29584E-23
ENSGALG00000030379	LMBR1	2	1.73421E-24	1.29725E-23
ENSGALG00000040028	GLB1	2	1.74088E-24	1.30144E-23
ENSGALG00000014902		3	1.75324E-24	1.30988E-23
ENSGALG00000001441	UBE2G1	19	1.75592E-24	1.31108E-23
ENSGALG00000012730	GFOD1	2	1.7756E-24	1.32496E-23
ENSGALG00000039222	DPY19L4	2	1.78417E-24	1.33054E-23
ENSGALG00000012345	UST	3	1.79923E-24	1.34095E-23
ENSGALG00000009901	DYRK2	1	1.82272E-24	1.35763E-23
ENSGALG00000005979	OXSR1	2	1.82924E-24	1.36165E-23
ENSGALG00000006097	PLXNA1	12	1.87262E-24	1.39309E-23
ENSGALG00000031893	RALA	2	1.90716E-24	1.41793E-23
ENSGALG00000030016	CTSB	3	1.98012E-24	1.47127E-23
ENSGALG00000001239	SLC26A2	13	1.98475E-24	1.47382E-23
ENSGALG00000009282	MIDEAS	5	2.01413E-24	1.49472E-23
ENSGALG00000007651	STAT1	7	2.10219E-24	1.55912E-23
ENSGALG00000032732	WASHC5	2	2.11837E-24	1.57017E-23
ENSGALG00000041202	FBXO32	2	2.13992E-24	1.58518E-23
ENSGALG00000014971	SLC2A9	4	2.2026E-24	1.63062E-23

ENSGALG00000029142	ORMDL3	27	2.2189E-24	1.64169E-23
ENSGALG00000016323	PDK3	1	2.22979E-24	1.64874E-23
ENSGALG00000005815	TMEM41B	5	2.25588E-24	1.66702E-23
ENSGALG00000029653		23	2.27129E-24	1.6774E-23
ENSGALG00000005676	TTC28	15	2.27926E-24	1.68227E-23
ENSGALG00000037698	ZFAT	2	2.42218E-24	1.78649E-23
ENSGALG00000010014	CRIPT	3	2.42341E-24	1.78649E-23
ENSGALG00000004578	RABGAP1L	8	2.44304E-24	1.79988E-23
ENSGALG00000004191	RIC8A	5	2.45442E-24	1.80717E-23
ENSGALG00000036567	ARMC1	2	2.5209E-24	1.855E-23
ENSGALG00000015342		1	2.52453E-24	1.85655E-23
ENSGALG00000032830	SEPT7	2	2.5337E-24	1.86217E-23
ENSGALG00000005972	KBTBD12	12	2.54334E-24	1.86813E-23
ENSGALG00000043935	AFF1	4	2.5704E-24	1.88687E-23
ENSGALG00000011798	HEBP1	1	2.58923E-24	1.89955E-23
ENSGALG00000040840	MPST	1	2.59284E-24	1.90105E-23
ENSGALG00000005609	OGFR	20	2.60948E-24	1.9121E-23
ENSGALG00000009065	VMA21	4	2.64349E-24	1.93586E-23
ENSGALG00000041962	POMT2	5	2.65778E-24	1.94516E-23
ENSGALG00000028851	RAB8A	28	2.70328E-24	1.97727E-23
ENSGALG00000001428	EFHD1	9	2.70842E-24	1.97984E-23
ENSGALG00000036114	SMO	1	2.76137E-24	2.01734E-23
ENSGALG00000042308	FZD3	3	2.87118E-24	2.09583E-23
ENSGALG00000009817	ZNF770	5	2.87225E-24	2.09583E-23
ENSGALG00000009821	NENF	3	2.87997E-24	2.10021E-23
ENSGALG00000001943	RHBDF2	18	2.93396E-24	2.1383E-23
ENSGALG00000010677	NRDE2	5	2.93724E-24	2.13942E-23
ENSGALG00000039134		33	2.94878E-24	2.14654E-23
ENSGALG00000048712	RNF115	25	2.95224E-24	2.14777E-23
ENSGALG00000032850	STK3	2	3.01002E-24	2.1885E-23
ENSGALG00000014468	MLF2	1	3.02469E-24	2.19786E-23
ENSGALG00000034944	UBE3A	1	3.05969E-24	2.22196E-23
ENSGALG00000012952	BASP1	2	3.06545E-24	2.22482E-23

ENSGALG00000008306	FGL2	1	3.1106E-24	2.25625E-23
ENSGALG00000016003	DDRGK1	4	3.12342E-24	2.2642E-23
ENSGALG00000054637		25	3.36653E-24	2.43898E-23
ENSGALG00000021231	TMEM254	6	3.45386E-24	2.50076E-23
ENSGALG00000010886	PAQR3	4	3.46266E-24	2.50565E-23
ENSGALG00000008054		10	3.54208E-24	2.5616E-23
ENSGALG00000054325		31	3.54997E-24	2.56579E-23
ENSGALG00000031286	APIP	5	3.56382E-24	2.57427E-23
ENSGALG00000003226	APOBEC2	26	3.68176E-24	2.65789E-23
ENSGALG00000011139	CCDC85C	5	3.74019E-24	2.69847E-23
ENSGALG00000014567	PHB2	1	3.75229E-24	2.7056E-23
ENSGALG00000041792	C26H6orf106	26	3.76668E-24	2.71437E-23
ENSGALG00000043081	WDR26	3	3.7944E-24	2.73273E-23
ENSGALG00000042182	OXR1	2	3.79997E-24	2.73512E-23
ENSGALG00000002705	CISD1	6	3.86023E-24	2.77686E-23
ENSGALG00000012003	XPNPEP3	1	3.9385E-24	2.8315E-23
ENSGALG00000006908	DLG1	9	4.04604E-24	2.9071E-23
ENSGALG00000041183	SGPP1	5	4.06019E-24	2.91554E-23
ENSGALG00000045856	BBOF1	5	4.06263E-24	2.91558E-23
ENSGALG00000053428		5	4.10574E-24	2.94478E-23
ENSGALG00000041114	GOLGA4	2	4.14014E-24	2.96771E-23
ENSGALG00000000156	BRD2	16	4.15742E-24	2.97834E-23
ENSGALG00000002967	ZNF365	6	4.19613E-24	3.00431E-23
ENSGALG00000011426	RNASET2	3	4.21357E-24	3.01503E-23
ENSGALG00000007190	ATP6V1G1	17	4.33169E-24	3.09773E-23
ENSGALG00000008559	CUL4B	4	4.36966E-24	3.12305E-23
ENSGALG00000011088	GSKIP	5	4.45828E-24	3.18452E-23
ENSGALG00000010149	CMTR1	3	4.46561E-24	3.18789E-23
ENSGALG00000013829	REPS1	3	4.47395E-24	3.19198E-23
ENSGALG00000001905	DTX2	19	4.48333E-24	3.1968E-23
ENSGALG00000002861	TMEM170A	11	4.55634E-24	3.24696E-23
ENSGALG00000039011	DPYS	2	4.64311E-24	3.30686E-23
ENSGALG00000034040	ZNF318	3	4.93448E-24	3.51233E-23

ENSGALG00000005947	MYD88	2	5.03684E-24	3.58309E-23
ENSGALG00000010542	PAPSS1	4	5.05624E-24	3.59422E-23
ENSGALG00000011840	PPP2R5E	5	5.05838E-24	3.59422E-23
ENSGALG00000040528	AP5Z1	14	5.08397E-24	3.6103E-23
ENSGALG00000004750	NOL8	12	5.08773E-24	3.61087E-23
ENSGALG00000028801	YPEL2	19	5.12337E-24	3.63405E-23
ENSGALG00000010001	EGLN3	5	5.17469E-24	3.66832E-23
ENSGALG00000001376	ARID3B	10	5.20892E-24	3.69043E-23
ENSGALG00000011770	UMPS	7	5.22827E-24	3.70199E-23
ENSGALG00000012473	MMADHC	7	5.24562E-24	3.71212E-23
ENSGALG00000015779	PM20D2	3	5.27959E-24	3.73399E-23
ENSGALG00000006001	PRPF6	20	5.36947E-24	3.79536E-23
ENSGALG00000000845	UVRAG	1	5.3827E-24	3.80251E-23
ENSGALG00000010818	PLPP3	8	5.4156E-24	3.82353E-23
ENSGALG00000048754		23	5.42945E-24	3.83109E-23
ENSGALG00000041685	CAPN1	33	5.47989E-24	3.86445E-23
ENSGALG00000052101		32	5.52931E-24	3.89705E-23
ENSGALG00000040741	DNM2	30	5.58636E-24	3.93498E-23
ENSGALG00000007937	SF3A1	15	5.63749E-24	3.9687E-23
ENSGALG00000032419	STARD8	4	5.66672E-24	3.9856E-23
ENSGALG00000011379	SLC44A5	8	5.66803E-24	3.9856E-23
ENSGALG00000029588		33	5.70933E-24	4.01232E-23
ENSGALG00000003160	CERS1	28	5.87082E-24	4.12344E-23
ENSGALG00000037371	TMEM65	2	5.93653E-24	4.16719E-23
ENSGALG00000014569	PNPLA2	5	6.03183E-24	4.23165E-23
ENSGALG00000053000	DNAAF9	4	6.05345E-24	4.24438E-23
ENSGALG00000003913	MCEE	10	6.15221E-24	4.31114E-23
ENSGALG00000013002	DAP	2	6.24117E-24	4.37097E-23
ENSGALG00000009177	FMR1	4	6.27436E-24	4.39169E-23
ENSGALG00000042554	ABCD2	1	6.31583E-24	4.41818E-23
ENSGALG00000039615	MSN	4	6.36397E-24	4.4493E-23
ENSGALG00000001467	ANKFY1	19	6.37918E-24	4.45739E-23
ENSGALG00000016196		1	6.54547E-24	4.57095E-23

ENSGALG00000009791	PROX1	3	6.55486E-24	4.57489E-23
ENSGALG00000002739	INPP5K	19	6.56957E-24	4.58254E-23
ENSGALG00000008555	MXI1	6	6.60534E-24	4.60485E-23
ENSGALG00000003165	CCDC6	6	6.69827E-24	4.66697E-23
ENSGALG00000001792	MRPS34	14	6.79434E-24	4.7312E-23
ENSGALG00000008745	APLF	3	6.8162E-24	4.74371E-23
ENSGALG00000005280	DBT	8	7.05196E-24	4.905E-23
ENSGALG00000008328	RSBN1L	1	7.16442E-24	4.98038E-23
ENSGALG00000004663	ARFGEF2	20	7.17092E-24	4.98205E-23
ENSGALG00000005456	SDR42E1	11	7.34699E-24	5.10147E-23
ENSGALG00000012050	TNRC6B	1	7.4082E-24	5.14104E-23
ENSGALG00000015459	ZFP92	1	7.4859E-24	5.19201E-23
ENSGALG00000047145	SMARCA4	32	7.65852E-24	5.30872E-23
ENSGALG00000006887	FAM174B	10	7.80661E-24	5.4083E-23
ENSGALG00000016148	C2CD2	1	7.82107E-24	5.41523E-23
ENSGALG00000016001	LZTS3	4	7.85446E-24	5.43527E-23
ENSGALG00000009356	SKIL	9	7.97942E-24	5.51861E-23
ENSGALG00000028790	DNASE2B	8	8.14076E-24	5.627E-23
ENSGALG00000037712	FCHO1	28	8.40956E-24	5.8095E-23
ENSGALG00000015377	PHLDB2	1	8.43039E-24	5.8206E-23
ENSGALG00000033301	DCAF1	12	8.44149E-24	5.82496E-23
ENSGALG00000006345	TMEM43	12	8.48331E-24	5.85051E-23
ENSGALG00000007171		14	8.55661E-24	5.89772E-23
ENSGALG00000001971	UBE2O	18	8.59946E-24	5.92391E-23
ENSGALG00000014071	ITPR2	1	8.74578E-24	6.0213E-23
ENSGALG00000007144	SIK3	24	8.8702E-24	6.10352E-23
ENSGALG00000004529	VPS29	15	8.99215E-24	6.18394E-23
ENSGALG00000004045	AGAP1	7	9.09472E-24	6.25096E-23
ENSGALG00000003794	SLC35E1	28	9.37118E-24	6.43734E-23
ENSGALG00000012526	PRPF40A	7	9.58841E-24	6.58285E-23
ENSGALG00000054599		32	9.60483E-24	6.59041E-23
ENSGALG00000011121	SLC35D1	8	9.61404E-24	6.59302E-23
ENSGALG00000005524	NTSR1	20	9.83774E-24	6.74264E-23

ENSGALG00000005698	HIF1AN	6	9.86461E-24	6.75726E-23
ENSGALG00000010969	SPIA4	5	9.87918E-24	6.76344E-23
ENSGALG00000008057	LLGL2	18	1.01092E-23	6.91704E-23
ENSGALG00000005637	GBF1	6	1.02118E-23	6.98333E-23
ENSGALG00000030599	TYK2	30	1.02747E-23	7.02242E-23
ENSGALG00000016574	GEMIN8	1	1.03208E-23	7.04993E-23
ENSGALG00000016535	RAB10	3	1.0349E-23	7.06526E-23
ENSGALG00000011031	JAK1	8	1.0429E-23	7.11585E-23
ENSGALG00000016065	BRWD1	1	1.05329E-23	7.18276E-23
ENSGALG00000001317		28	1.05767E-23	7.2086E-23
ENSGALG00000030550	ATP2B1	1	1.09373E-23	7.45022E-23
ENSGALG00000003689	PAPSS2	6	1.11392E-23	7.58351E-23
ENSGALG00000041787	PLA2G15	11	1.12507E-23	7.65514E-23
ENSGALG00000011870	HIF1A	5	1.14282E-23	7.77154E-23
ENSGALG00000003349	PLEKHA2	22	1.14538E-23	7.78465E-23
ENSGALG00000014408	LLGL1	14	1.14802E-23	7.79824E-23
ENSGALG00000050441		1	1.15892E-23	7.86789E-23
ENSGALG00000013369	NCLN	28	1.16982E-23	7.93745E-23
ENSGALG00000041423	MAP7D3	4	1.18779E-23	8.05491E-23
ENSGALG00000039239	SERPIND1	15	1.24033E-23	8.40652E-23
ENSGALG00000017312	PPME1	1	1.25902E-23	8.52448E-23
ENSGALG00000027696		28	1.25913E-23	8.52448E-23
ENSGALG00000008115	ATXN7L1	1	1.26139E-23	8.53503E-23
ENSGALG00000004180	TOR1B	17	1.27236E-23	8.60449E-23
ENSGALG00000021345	B3GNT9	11	1.28361E-23	8.67574E-23
ENSGALG00000011132	RBMS1	7	1.29007E-23	8.71459E-23
ENSGALG00000011618	ADSS1	5	1.31029E-23	8.84624E-23
ENSGALG00000054256	HAUS3	4	1.33603E-23	9.01503E-23
ENSGALG00000040005	PPP2R5D	3	1.37081E-23	9.24464E-23
ENSGALG00000003628	TLN2	10	1.37432E-23	9.26316E-23
ENSGALG00000015580	UFL1	3	1.37523E-23	9.2642E-23
ENSGALG00000034383	TRMT2B	4	1.38726E-23	9.3401E-23
ENSGALG00000016433	YWHAQ	3	1.41436E-23	9.51727E-23

ENSGALG00000048759		33	1.42542E-23	9.58643E-23
ENSGALG00000014122	CYB5R1	1	1.42731E-23	9.59383E-23
ENSGALG00000014236	ATP8A1	4	1.43562E-23	9.64437E-23
ENSGALG00000014248	TMEM33	4	1.43764E-23	9.65259E-23
ENSGALG00000008321	RNPEPL1	9	1.45922E-23	9.79211E-23
ENSGALG00000009330	DEGS1	3	1.46399E-23	9.81869E-23
ENSGALG00000002336	EP400	15	1.48189E-23	9.93329E-23
ENSGALG00000004839	GPATCH1	11	1.48951E-23	9.9789E-23
ENSGALG00000008136	C7H2ORF69	7	1.49056E-23	9.98046E-23
ENSGALG00000040902	SACM1L	2	1.5126E-23	1.01224E-22
ENSGALG00000001605	PPM1J	26	1.52465E-23	1.01975E-22
ENSGALG00000053335		4	1.5562E-23	1.04028E-22
ENSGALG00000015314	BEND3	3	1.57857E-23	1.05466E-22
ENSGALG00000010357	P2RY1	9	1.5812E-23	1.05584E-22
ENSGALG00000012808		2	1.58571E-23	1.05827E-22
ENSGALG00000033663	ATP6V1B2	22	1.59127E-23	1.0614E-22
ENSGALG00000011297	CRADD	1	1.59672E-23	1.06445E-22
ENSGALG00000032599	ZNF384	1	1.60031E-23	1.06626E-22
ENSGALG00000031702		33	1.63624E-23	1.08961E-22
ENSGALG00000011007	OSBPL3	2	1.64754E-23	1.09653E-22
ENSGALG00000005407	AGL	8	1.65283E-23	1.09945E-22
ENSGALG00000008500	RBSN	12	1.6689E-23	1.10954E-22
ENSGALG00000017075	FRY	1	1.70542E-23	1.1332E-22
ENSGALG00000036829	RECK	2	1.72017E-23	1.14237E-22
ENSGALG00000000777	EYA3	23	1.73117E-23	1.14906E-22
ENSGALG00000008318	SLK	6	1.77956E-23	1.18053E-22
ENSGALG00000006392	RNF168	9	1.82445E-23	1.20965E-22
ENSGALG00000015547	TADA2B	4	1.82879E-23	1.21187E-22
ENSGALG00000008389	VBP1	4	1.8393E-23	1.21818E-22
ENSGALG00000014979	FRK	3	1.86524E-23	1.23468E-22
ENSGALG00000016491	APOB	3	1.88065E-23	1.24421E-22
ENSGALG00000046739		16	1.9282E-23	1.27498E-22
ENSGALG00000036085		2	1.96499E-23	1.2986E-22

ENSGALG00000015344	NXPE3	1	1.9771E-23	1.30589E-22
ENSGALG00000031070	MAIP1	7	1.98659E-23	1.31145E-22
ENSGALG00000002818	VAT1	27	1.99119E-23	1.31377E-22
ENSGALG00000054297		1	2.05185E-23	1.35307E-22
ENSGALG00000009520	MARCH1	4	2.06324E-23	1.35985E-22
ENSGALG00000000919	PIGR	26	2.08103E-23	1.37083E-22
ENSGALG00000010611	CFAP97	4	2.09692E-23	1.38055E-22
ENSGALG00000002003	BMPR1A	6	2.13324E-23	1.4037E-22
ENSGALG00000004816		14	2.2576E-23	1.48473E-22
ENSGALG00000051212		30	2.2682E-23	1.4909E-22
ENSGALG00000012866	SERPINB6	2	2.27234E-23	1.49281E-22
ENSGALG00000013709	TIMM21	2	2.29381E-23	1.50611E-22
ENSGALG00000007557	RUBCN	9	2.29793E-23	1.508E-22
ENSGALG00000052223	YPEL1	15	2.36748E-23	1.55281E-22
ENSGALG00000033603	METTL22	14	2.37501E-23	1.55654E-22
ENSGALG00000007615	RAB11A	10	2.37692E-23	1.55654E-22
ENSGALG00000011937	NFE2L1	27	2.377E-23	1.55654E-22
ENSGALG00000008519	NR2C2	12	2.38408E-23	1.56034E-22
ENSGALG00000016563	ASB9	1	2.39224E-23	1.56484E-22
ENSGALG00000012227	TSPAN5	4	2.41593E-23	1.57949E-22
ENSGALG00000003026		14	2.4458E-23	1.59816E-22
ENSGALG00000002510	IP6K1	12	2.45444E-23	1.60294E-22
ENSGALG00000043437	CAPZA2	1	2.49536E-23	1.6288E-22
ENSGALG00000000657	TERF2	11	2.55095E-23	1.66419E-22
ENSGALG00000027096	LGALSL	3	2.57943E-23	1.68187E-22
ENSGALG00000044053	NLRX1	24	2.59503E-23	1.69114E-22
ENSGALG00000004171	RNF111	10	2.61922E-23	1.70599E-22
ENSGALG00000041445	ANKRD46	2	2.71132E-23	1.76504E-22
ENSGALG00000006254	ERCC6	6	2.76459E-23	1.79875E-22
ENSGALG00000009346	HN1L	14	2.77789E-23	1.8064E-22
ENSGALG00000007199	TRIAP1	15	2.77931E-23	1.8064E-22
ENSGALG00000017242	FZD4	1	2.78462E-23	1.80889E-22
ENSGALG00000049505		32	2.79742E-23	1.81564E-22

ENSGALG00000045584		4	2.79799E-23	1.81564E-22
ENSGALG00000032148	CANX	13	2.98983E-23	1.93909E-22
ENSGALG00000008579	OIP5	5	3.01723E-23	1.95583E-22
ENSGALG00000008357	TPCN1	15	3.12211E-23	2.02274E-22
ENSGALG00000034777	VPS13D	21	3.12838E-23	2.0255E-22
ENSGALG00000010795	CRPPA	2	3.1297E-23	2.0255E-22
ENSGALG00000009224	TTC30B	7	3.21409E-23	2.07902E-22
ENSGALG00000001988	TXNRD2	15	3.24318E-23	2.09672E-22
ENSGALG00000007246	ELMO2	20	3.34123E-23	2.15896E-22
ENSGALG00000006052	WDR48	2	3.37762E-23	2.18132E-22
ENSGALG00000004532	ZNF507	11	3.45242E-23	2.22844E-22
ENSGALG00000031475	MAFG	18	3.49812E-23	2.25675E-22
ENSGALG00000037441		25	3.50432E-23	2.25889E-22
ENSGALG00000007664	PSMD1	9	3.50514E-23	2.25889E-22
ENSGALG00000007304	DNMBP	6	3.56043E-23	2.29331E-22
ENSGALG00000014907	DCBLD1	3	3.58187E-23	2.3059E-22
ENSGALG00000016427	ADAM17	3	3.60199E-23	2.31763E-22
ENSGALG00000034493	DCLK3	2	3.62215E-23	2.32937E-22
ENSGALG00000008092	NXT2	4	3.68777E-23	2.37032E-22
ENSGALG00000016418	BCLAF3	1	3.70871E-23	2.38252E-22
ENSGALG00000008604	TMEM255A	4	3.71941E-23	2.38814E-22
ENSGALG00000008797	WASL	1	3.78109E-23	2.42646E-22
ENSGALG00000039363	TXNRD3	12	3.85389E-23	2.47189E-22
ENSGALG00000010279	TMEM69	8	3.87753E-23	2.48574E-22
ENSGALG00000009966	FRS2	1	3.9716E-23	2.5447E-22
ENSGALG00000035182	CMKLR1	15	3.98137E-23	2.54962E-22
ENSGALG00000026449		12	4.04057E-23	2.58618E-22
ENSGALG00000014335	ITFG2	1	4.12963E-23	2.64179E-22
ENSGALG00000008265	PDCD11	6	4.1338E-23	2.64308E-22
ENSGALG00000009272		4	4.18304E-23	2.67315E-22
ENSGALG00000009303	ENAH	3	4.34599E-23	2.77583E-22
ENSGALG00000004684	IPPK	12	4.3679E-23	2.78837E-22
ENSGALG00000017070	PDS5B	1	4.47506E-23	2.85528E-22

ENSGALG00000009075	CD99L2	4	4.5975E-23	2.93125E-22
ENSGALG00000009865	RNF150	4	4.59893E-23	2.93125E-22
ENSGALG00000046580	MGARP	4	4.61325E-23	2.93884E-22
ENSGALG00000000636	MDM4	26	4.66038E-23	2.96731E-22
ENSGALG00000010046	FAM177A1	5	4.81094E-23	3.06158E-22
ENSGALG00000004833	P3H1	21	4.90574E-23	3.12027E-22
ENSGALG00000003294	ATAD5	18	5.00865E-23	3.18407E-22
ENSGALG00000007111	KCTD6	12	5.06048E-23	3.21534E-22
ENSGALG00000013981	TBPL1	3	5.24672E-23	3.33194E-22
ENSGALG00000004570	NCOA3	20	5.54625E-23	3.52032E-22
ENSGALG00000034007	ITGA6	7	5.57206E-23	3.53487E-22
ENSGALG00000046862	UBE2Q2	10	5.78641E-23	3.66793E-22
ENSGALG00000002759	PEAK1	10	5.78783E-23	3.66793E-22
ENSGALG00000044642	SENP7	1	5.7933E-23	3.66949E-22
ENSGALG00000007260	ZEB1	2	5.83262E-23	3.69248E-22
ENSGALG00000029332	C5H15orf57	5	5.85631E-23	3.70555E-22
ENSGALG00000009320	PSEN1	5	5.91603E-23	3.7414E-22
ENSGALG00000038217	CD82	5	5.98762E-23	3.78471E-22
ENSGALG00000034100	UACA	10	6.03594E-23	3.81328E-22
ENSGALG00000014209	GPR155	7	6.06468E-23	3.82945E-22
ENSGALG00000040879	NUDCD1	2	6.09023E-23	3.84359E-22
ENSGALG00000001750	MAT2B	13	6.0957E-23	3.84505E-22
ENSGALG00000011309		1	6.21468E-23	3.91808E-22
ENSGALG00000017196	ARHGAP42	1	6.323E-23	3.98431E-22
ENSGALG00000006840		10	6.33271E-23	3.98836E-22
ENSGALG00000008629	MRC1	2	6.35024E-23	3.99734E-22
ENSGALG00000011377	METAP2	1	6.45303E-23	4.05995E-22
ENSGALG00000004661	SHISA5	12	6.48925E-23	4.08063E-22
ENSGALG00000042052	PPIL2	15	6.56234E-23	4.12446E-22
ENSGALG00000028683	SSR3	9	6.5812E-23	4.13418E-22
ENSGALG00000053068	SMUG1	33	6.64545E-23	4.1724E-22
ENSGALG00000036854	R3HCC1	22	6.65736E-23	4.17773E-22
ENSGALG00000034625	TCEB3	23	6.6863E-23	4.19373E-22

ENSGALG00000016321	CILK1	3	6.78269E-23	4.252E-22
ENSGALG00000049026	MAP2K7	31	6.94659E-23	4.35251E-22
ENSGALG00000012973	ESR1	3	6.9843E-23	4.37389E-22
ENSGALG00000035575	SLC7A6OS	11	7.07892E-23	4.43087E-22
ENSGALG00000016768	LONRF2	1	7.09634E-23	4.43949E-22
ENSGALG00000005093		19	7.20906E-23	4.5077E-22
ENSGALG00000038187	SLC35B4	1	7.33772E-23	4.58579E-22
ENSGALG00000002363	HSPA9	13	7.34738E-23	4.58948E-22
ENSGALG00000030554	KCNN3	25	7.53642E-23	4.70515E-22
ENSGALG00000010547	YIPF4	3	7.60872E-23	4.74786E-22
ENSGALG00000003378		14	7.6222E-23	4.75383E-22
ENSGALG00000012289	SHPRH	3	7.67842E-23	4.78645E-22
ENSGALG00000015770	APP	1	7.72426E-23	4.81256E-22
ENSGALG00000041456	SLC35G1	6	7.7917E-23	4.8521E-22
ENSGALG00000016036	DOP1B	1	7.81475E-23	4.86397E-22
ENSGALG00000015086	OSBPL1A	2	7.85903E-23	4.88904E-22
ENSGALG00000002304	ADPRHL2	23	7.95471E-23	4.94604E-22
ENSGALG00000004355	MCU	6	8.13292E-23	5.05427E-22
ENSGALG00000010932	NUDT9	4	8.13856E-23	5.0552E-22
ENSGALG00000053670	MEGF9	17	8.16795E-23	5.07087E-22
ENSGALG00000012179	SOCS4	5	8.20036E-23	5.0884E-22
ENSGALG00000012919	YARS2	1	8.21933E-23	5.09758E-22
ENSGALG00000013535	CYP4V2	4	8.23246E-23	5.10312E-22
ENSGALG00000028133	UBE2V2	2	8.27122E-23	5.12454E-22
ENSGALG00000001564	ATP2A3	19	8.3524E-23	5.17221E-22
ENSGALG00000036666	ZCCHC10	13	8.57529E-23	5.30754E-22
ENSGALG00000015077		1	8.66443E-23	5.35999E-22
ENSGALG00000006655	MAP3K13	9	8.89074E-23	5.4972E-22
ENSGALG00000008014	CEBPB	20	8.95978E-23	5.53708E-22
ENSGALG00000016796	C1H2ORF49	1	9.16767E-23	5.66269E-22
ENSGALG00000008842	PIKFYVE	7	9.27457E-23	5.72581E-22
ENSGALG00000042333	DDB1	5	9.28279E-23	5.72799E-22
ENSGALG00000017358	YWHAG	19	9.384E-23	5.78751E-22

ENSGALG00000013218	C3AR1	1	9.60596E-23	5.92141E-22
ENSGALG00000002147	SIKE1	26	9.80396E-23	6.04041E-22
ENSGALG00000007443	TMLHE	4	9.85996E-23	6.07184E-22
ENSGALG00000011229	SERBP1	8	9.87335E-23	6.07702E-22
ENSGALG00000040856	CPNE1	20	1.00177E-22	6.16274E-22
ENSGALG00000031410	ENPEP	4	1.00466E-22	6.17745E-22
ENSGALG00000004908		4	1.00987E-22	6.20634E-22
ENSGALG00000046494	CASP10	7	1.01909E-22	6.2598E-22
ENSGALG00000015273	TBC1D23	1	1.02756E-22	6.30866E-22
ENSGALG00000010371	FAM13A	4	1.04549E-22	6.41551E-22
ENSGALG00000004065	SLC25A16	6	1.05548E-22	6.47357E-22
ENSGALG00000004412	DIABLO	15	1.06684E-22	6.53997E-22
ENSGALG00000008514	DLL4	5	1.07188E-22	6.56757E-22
ENSGALG00000013861	TNFAIP3	3	1.07326E-22	6.57271E-22
ENSGALG00000013594	PARD6G	2	1.10749E-22	6.77892E-22
ENSGALG00000052506	SLC25A44	23	1.10923E-22	6.7862E-22
ENSGALG00000002831	SH3PXD2B	13	1.12089E-22	6.85406E-22
ENSGALG00000012101	AP5M1	5	1.13145E-22	6.91519E-22
ENSGALG00000015828	BACH1	1	1.14032E-22	6.96591E-22
ENSGALG00000037713	FNDC10	21	1.14988E-22	7.02075E-22
ENSGALG00000001603	NARF	18	1.15205E-22	7.02767E-22
ENSGALG00000013265	GYS2	1	1.15243E-22	7.02767E-22
ENSGALG00000047187		9	1.15274E-22	7.02767E-22
ENSGALG00000011823	FAT4	4	1.18242E-22	7.20504E-22
ENSGALG00000029341	PPP6R2	1	1.18576E-22	7.22176E-22
ENSGALG00000036539	EFNB1	4	1.19172E-22	7.25447E-22
ENSGALG00000005725	CSF1R	13	1.19672E-22	7.28122E-22
ENSGALG00000037301	ACVR1	7	1.20652E-22	7.33722E-22
ENSGALG00000014988	POPDC2	1	1.21318E-22	7.37404E-22
ENSGALG00000003297	HDAC1	23	1.22555E-22	7.44553E-22
ENSGALG00000001144	ZNF346	13	1.23109E-22	7.47546E-22
ENSGALG00000003381	HERC1	10	1.24013E-22	7.52658E-22
ENSGALG00000017268	KCTD21	1	1.24144E-22	7.53078E-22

ENSGALG00000002469	CDC73	8	1.26814E-22	7.68894E-22
ENSGALG00000013310	MAEA	4	1.2697E-22	7.69459E-22
ENSGALG00000042080	PGPEP1	28	1.27365E-22	7.71469E-22
ENSGALG00000047837		2	1.30731E-22	7.9146E-22
ENSGALG00000038018	DNMT1	30	1.33704E-22	8.09061E-22
ENSGALG00000035478	FAM91A1	2	1.36237E-22	8.23976E-22
ENSGALG00000040758	INSR	28	1.36573E-22	8.25599E-22
ENSGALG00000035696	RRP1B	1	1.37746E-22	8.32276E-22
ENSGALG00000009653	CASD1	2	1.40398E-22	8.47879E-22
ENSGALG00000041663	KLB	4	1.40484E-22	8.47981E-22
ENSGALG00000035115		14	1.4263E-22	8.60511E-22
ENSGALG00000017389	SIX4	5	1.44419E-22	8.70871E-22
ENSGALG00000028876	SNN	14	1.46558E-22	8.83331E-22
ENSGALG00000037171	ZNF622	2	1.49644E-22	9.01486E-22
ENSGALG00000053490	FBXO45	9	1.51944E-22	9.14892E-22
ENSGALG00000009718	FAM53B	6	1.52554E-22	9.1811E-22
ENSGALG00000010539	RNF11	8	1.53778E-22	9.24778E-22
ENSGALG00000027905	CSRNP1	2	1.53814E-22	9.24778E-22
ENSGALG00000044716	KIAA1109	4	1.55101E-22	9.31697E-22
ENSGALG00000031328	PAK4	32	1.55117E-22	9.31697E-22
ENSGALG00000013764	AASDH	4	1.56491E-22	9.39487E-22
ENSGALG00000006092		20	1.56663E-22	9.40056E-22
ENSGALG00000015378	ABHD10	1	1.63958E-22	9.83347E-22
ENSGALG00000008667	DSTN	3	1.6414E-22	9.83953E-22
ENSGALG00000013573	ALG2	2	1.64225E-22	9.83981E-22
ENSGALG00000051049		31	1.64326E-22	9.84102E-22
ENSGALG00000010853	C8B	8	1.6705E-22	9.99919E-22
ENSGALG00000016459	LRATD1	3	1.69773E-22	1.01572E-21
ENSGALG00000037778	NAB1	7	1.71432E-22	1.02494E-21
ENSGALG00000041275	SLC25A38	2	1.71482E-22	1.02494E-21
ENSGALG00000030179		6	1.72688E-22	1.03164E-21
ENSGALG00000014878	MAN1A1	3	1.75269E-22	1.04655E-21
ENSGALG00000002775	FA2H	11	1.7809E-22	1.06287E-21

ENSGALG00000040546		20	1.85286E-22	1.10528E-21
ENSGALG00000036743	MGAT3	1	1.85582E-22	1.1065E-21
ENSGALG00000048158		25	1.86888E-22	1.11375E-21
ENSGALG00000009896	TRERF1	3	1.89564E-22	1.12914E-21
ENSGALG00000041253	MYRF	5	1.90124E-22	1.13193E-21
ENSGALG00000010491	PWWP2B	6	1.93771E-22	1.15308E-21
ENSGALG00000012139	ARL14EP	5	1.9626E-22	1.16728E-21
ENSGALG00000010580	STRN	3	1.96349E-22	1.16728E-21
ENSGALG00000006826	KLHL25	10	2.00469E-22	1.19119E-21
ENSGALG00000007695	SNAPC5	10	2.03275E-22	1.20727E-21
ENSGALG00000008546	FGD5	12	2.05344E-22	1.21897E-21
ENSGALG00000037991	H1-10	12	2.16435E-22	1.28418E-21
ENSGALG00000033174	ZBTB45	31	2.18003E-22	1.29285E-21
ENSGALG00000043053	DAO	15	2.18448E-22	1.29487E-21
ENSGALG00000001642	GLT8D1	12	2.18815E-22	1.29641E-21
ENSGALG00000009551	DYNC1I2	7	2.2074E-22	1.30718E-21
ENSGALG00000015185	TAF13	1	2.20954E-22	1.30781E-21
ENSGALG00000007519	ACBD5	2	2.26432E-22	1.33958E-21
ENSGALG00000049181	CHST14	5	2.37012E-22	1.4015E-21
ENSGALG00000015473	ADPRH	1	2.38095E-22	1.40667E-21
ENSGALG00000047947		22	2.38118E-22	1.40667E-21
ENSGALG00000011894	CYP2D6	1	2.38388E-22	1.40759E-21
ENSGALG00000037339	SMARCAD1	4	2.39393E-22	1.41229E-21
ENSGALG00000009692	SH3RF1	4	2.39416E-22	1.41229E-21
ENSGALG00000007044	METTL9	14	2.40316E-22	1.41691E-21
ENSGALG00000051301	KHK	3	2.41669E-22	1.4242E-21
ENSGALG00000035966	EDA	4	2.46619E-22	1.45267E-21
ENSGALG00000027579	MRPS6	1	2.47905E-22	1.45954E-21
ENSGALG00000012909	C5orf22	2	2.49402E-22	1.46765E-21
ENSGALG00000009044		2	2.53736E-22	1.49243E-21
ENSGALG00000015215	MAPRE2	2	2.54834E-22	1.49816E-21
ENSGALG00000038684	EEFSEC	12	2.5526E-22	1.49995E-21
ENSGALG00000005105	TPR	8	2.55935E-22	1.50319E-21

ENSGALG00000009728	SLC25A13	2	2.61516E-22	1.53523E-21
ENSGALG00000007867	SLC9A5	11	2.62897E-22	1.54259E-21
ENSGALG00000012688	NHLRC1	2	2.64332E-22	1.55026E-21
ENSGALG00000041797		25	2.67443E-22	1.56776E-21
ENSGALG00000047142		4	2.7283E-22	1.59857E-21
ENSGALG00000002598	TPST1	19	2.74704E-22	1.60878E-21
ENSGALG00000007643	DIS3L	10	2.76863E-22	1.62064E-21
ENSGALG00000013037	BCL2L13	1	2.77809E-22	1.6254E-21
ENSGALG00000047665		16	2.85882E-22	1.67183E-21
ENSGALG00000006049	STK26	4	2.86444E-22	1.67431E-21
ENSGALG00000010313		8	2.93716E-22	1.71599E-21
ENSGALG00000009955	ABCG5	3	2.96042E-22	1.72875E-21
ENSGALG00000043628	UBE2M	31	2.96751E-22	1.73207E-21
ENSGALG00000039200	AXIN2	18	2.97006E-22	1.73272E-21
ENSGALG00000002706	XRN1	9	2.97324E-22	1.73375E-21
ENSGALG00000010352	ARHGEF26	9	2.9819E-22	1.73797E-21
ENSGALG00000034859	COMMD9	5	3.0289E-22	1.76452E-21
ENSGALG00000002167	PATL2	10	3.05365E-22	1.77809E-21
ENSGALG00000009730	SLC38A4	1	3.08427E-22	1.79429E-21
ENSGALG00000051466	NDFIP2	1	3.08441E-22	1.79429E-21
ENSGALG00000034077	KCNK5	3	3.10026E-22	1.80265E-21
ENSGALG00000011414	AMDHD1	1	3.10433E-22	1.80415E-21
ENSGALG00000001684	SEC14L1	18	3.12989E-22	1.81814E-21
ENSGALG00000002750	NCKAP1	7	3.15439E-22	1.8315E-21
ENSGALG00000034500	CIDEA	2	3.23488E-22	1.87734E-21
ENSGALG00000002277	KLHL24	9	3.26804E-22	1.89568E-21
ENSGALG00000039420	TNPO3	1	3.34361E-22	1.93859E-21
ENSGALG00000033932	BF1	16	3.3717E-22	1.95395E-21
ENSGALG00000006399	TLNRD1	10	3.42166E-22	1.98196E-21
ENSGALG00000049001	ANKRD39	22	3.46533E-22	2.00631E-21
ENSGALG00000010763	TRIP11	5	3.47604E-22	2.01155E-21
ENSGALG00000007097	RPS6KA6	4	3.50102E-22	2.02505E-21
ENSGALG00000015051	CDK19	3	3.58905E-22	2.07498E-21

ENSGALG00000017210	ANKRD49	1	3.62262E-22	2.09339E-21
ENSGALG00000002369	F13B	8	3.68443E-22	2.1281E-21
ENSGALG00000009900	PRKD1	5	3.69682E-22	2.13425E-21
ENSGALG00000015805		4	3.76527E-22	2.17274E-21
ENSGALG00000008944	FBXO11	3	3.77683E-22	2.17838E-21
ENSGALG00000012300	RAB32	3	3.81586E-22	2.19985E-21
ENSGALG00000046331		31	4.00742E-22	2.30919E-21
ENSGALG00000052842	ANKRD11	11	4.01324E-22	2.31145E-21
ENSGALG00000000962	PUS3	24	4.07678E-22	2.34694E-21
ENSGALG00000005900	E4F1	14	4.07899E-22	2.3471E-21
ENSGALG00000012425	FERMT2	5	4.11472E-22	2.36654E-21
ENSGALG00000010746	TMEM59	8	4.16649E-22	2.39519E-21
ENSGALG00000032249	MAX	5	4.17443E-22	2.39862E-21
ENSGALG00000016285		1	4.18453E-22	2.40228E-21
ENSGALG00000034253	ZNF644	8	4.18472E-22	2.40228E-21
ENSGALG00000012214	RAB3GAP1	7	4.18944E-22	2.40385E-21
ENSGALG00000008107	IRS4	4	4.23802E-22	2.43058E-21
ENSGALG00000050288	DCP1B	1	4.27206E-22	2.44895E-21
ENSGALG00000005803	ZNRF3	15	4.35035E-22	2.49266E-21
ENSGALG00000028989		1	4.37948E-22	2.50774E-21
ENSGALG00000001055	NSF	27	4.38079E-22	2.50774E-21
ENSGALG00000005268	SEC22C	2	4.41227E-22	2.52458E-21
ENSGALG00000029810	RPS19BP1	1	4.41996E-22	2.5278E-21
ENSGALG00000009802	ANGEL2	3	4.48181E-22	2.56197E-21
ENSGALG00000001525		27	4.49225E-22	2.56673E-21
ENSGALG00000000557	ETNK2	26	4.49688E-22	2.56817E-21
ENSGALG00000030481	RAB40C	14	4.52994E-22	2.58584E-21
ENSGALG00000007712	MYO1B	7	4.53453E-22	2.58725E-21
ENSGALG00000047814		17	4.5483E-22	2.59389E-21
ENSGALG00000006542	ESYT2	2	4.57662E-22	2.60883E-21
ENSGALG00000005509	DPYD	8	4.58456E-22	2.61213E-21
ENSGALG00000011131	TANK	7	4.63564E-22	2.64E-21
ENSGALG00000011446	TNFAIP2	5	4.7066E-22	2.67916E-21

ENSGALG00000013851	UBE3B	15	4.81594E-22	2.74012E-21
ENSGALG00000050065	CYSLTR2	1	4.86577E-22	2.76718E-21
ENSGALG00000052154		9	4.90096E-22	2.78589E-21
ENSGALG00000009532	GXYLT1	1	5.03603E-22	2.86134E-21
ENSGALG00000009071	HMGB3	4	5.13042E-22	2.91361E-21
ENSGALG00000016504	ITSN2	3	5.29968E-22	3.00833E-21
ENSGALG00000006425	SLC6A6	12	5.32125E-22	3.01915E-21
ENSGALG00000010571	HEATR5B	3	5.32369E-22	3.01915E-21
ENSGALG00000016059	ETS2	1	5.39177E-22	3.05634E-21
ENSGALG00000010674	DCTD	4	5.40617E-22	3.06308E-21
ENSGALG00000003601	ATP2B4	26	5.42573E-22	3.07273E-21
ENSGALG00000040594	GNPDA1	13	5.51829E-22	3.1237E-21
ENSGALG00000000606	ARL8A	26	5.79357E-22	3.278E-21
ENSGALG00000001726	NUDCD2	13	5.98931E-22	3.38718E-21
ENSGALG00000029402	AXIN1	14	6.04116E-22	3.41492E-21
ENSGALG00000013135	GALNT1	2	6.08466E-22	3.43792E-21
ENSGALG00000004078		13	6.09389E-22	3.44154E-21
ENSGALG00000023131	PDXP	1	6.11132E-22	3.44948E-21
ENSGALG00000007843	ATRX	4	6.11361E-22	3.44948E-21
ENSGALG00000050166		14	6.20377E-22	3.49873E-21
ENSGALG00000035623	LIMD1	2	6.45972E-22	3.6414E-21
ENSGALG00000014290	UBE2K	4	6.60183E-22	3.71978E-21
ENSGALG00000038964		9	6.62784E-22	3.73272E-21
ENSGALG00000005998	RRAS	5	6.70079E-22	3.77206E-21
ENSGALG00000027860	CISD2	4	6.85492E-22	3.85704E-21
ENSGALG00000042612		9	6.86806E-22	3.86265E-21
ENSGALG00000014799	METTL4	2	6.91909E-22	3.88955E-21
ENSGALG00000006147	NUCB2	5	6.97602E-22	3.91975E-21
ENSGALG00000038636	LAPTM5	23	7.25876E-22	4.0752E-21
ENSGALG00000050760	ADCY6	33	7.25937E-22	4.0752E-21
ENSGALG00000010395	SIAH2	9	7.32034E-22	4.10755E-21
ENSGALG00000002553	PCDH1	13	7.36828E-22	4.13254E-21
ENSGALG00000049865	RPL18A	1	7.4617E-22	4.18301E-21

ENSGALG00000032173	TADA1	8	7.56539E-22	4.23919E-21
ENSGALG00000003370	KIAA1191	13	7.58232E-22	4.24673E-21
ENSGALG00000032223		32	7.58641E-22	4.24707E-21
ENSGALG00000010023	CALM2	3	7.62749E-22	4.2681E-21
ENSGALG00000017462	FNIP1	13	7.63498E-22	4.27033E-21
ENSGALG00000005476	PTH2R	2	7.75203E-22	4.33381E-21
ENSGALG00000038089	EGFR	2	7.78653E-22	4.35111E-21
ENSGALG00000011442	TGFBR2	2	7.94582E-22	4.43809E-21
ENSGALG00000043675	ANTXR2	4	8.01074E-22	4.4723E-21
ENSGALG00000006167	LRRC8B	8	8.08171E-22	4.50985E-21
ENSGALG00000012341	SAV1	5	8.09373E-22	4.51449E-21
ENSGALG00000003228	OARD1	26	8.1318E-22	4.53196E-21
ENSGALG00000043669	WDFY3	4	8.13247E-22	4.53196E-21
ENSGALG00000007334	ABAT	14	8.13747E-22	4.53267E-21
ENSGALG00000002783	SLC43A2	19	8.19442E-22	4.56231E-21
ENSGALG00000013695	ZDHHC14	3	8.25313E-22	4.5929E-21
ENSGALG00000031874	HIST1H101	1	8.32334E-22	4.62986E-21
ENSGALG00000037496	USP46	4	8.38148E-22	4.66007E-21
ENSGALG00000029893	COG1	18	8.48092E-22	4.71321E-21
ENSGALG00000008089	KBTBD4	5	8.60208E-22	4.77837E-21
ENSGALG00000016579	RAB9A	1	8.67367E-22	4.81594E-21
ENSGALG00000007757	CSTF1	20	8.76688E-22	4.86548E-21
ENSGALG00000001375	COX10	18	8.81528E-22	4.89012E-21
ENSGALG00000006763	EXOC6	6	8.82309E-22	4.89222E-21
ENSGALG00000010665	FOXN3	5	9.09101E-22	5.03849E-21
ENSGALG00000009456	EXD2	5	9.33387E-22	5.17074E-21
ENSGALG00000015330	PCNP	1	9.39654E-22	5.20309E-21
ENSGALG00000038943	RASSF5	26	9.47633E-22	5.24489E-21
ENSGALG00000001618	OGFOD3	18	9.50168E-22	5.25653E-21
ENSGALG00000006454	SLC22A18	5	9.66767E-22	5.34594E-21
ENSGALG00000006916	GDE1	14	9.77623E-22	5.40352E-21
ENSGALG00000004671	ANKRD27	11	1.00445E-21	5.54926E-21
ENSGALG00000029652	OPHN1	4	1.01443E-21	5.60187E-21

ENSGALG00000041558		3	1.03088E-21	5.69013E-21
ENSGALG00000003977	TWF2	12	1.03564E-21	5.7138E-21
ENSGALG00000008325	CKAP5	5	1.03903E-21	5.72992E-21
ENSGALG00000007694	PRTFDC1	2	1.05944E-21	5.83984E-21
ENSGALG00000007777	SLC39A10	7	1.06223E-21	5.8526E-21
ENSGALG00000023581	UTS2R	18	1.06407E-21	5.86009E-21
ENSGALG00000030719	CLMN	5	1.06529E-21	5.86416E-21
ENSGALG00000028935	BCL10	8	1.08886E-21	5.9912E-21
ENSGALG00000008631	AXL	5	1.09663E-21	6.03119E-21
ENSGALG00000002095	CHMP4B	20	1.10041E-21	6.04929E-21
ENSGALG00000002639	TRPC1	9	1.10259E-21	6.05854E-21
ENSGALG00000008778	SPRED2	3	1.11223E-21	6.10801E-21
ENSGALG00000053100	FITM2	20	1.1126E-21	6.10801E-21
ENSGALG00000002621	SLC25A42	28	1.13952E-21	6.25298E-21
ENSGALG00000004199	TMEM184A	14	1.15355E-21	6.32712E-21
ENSGALG00000051415	TM2D1	8	1.1723E-21	6.42711E-21
ENSGALG00000015706	LETM1	4	1.17853E-21	6.45833E-21
ENSGALG00000051383		4	1.18728E-21	6.50335E-21
ENSGALG00000008991	VPS39	5	1.19474E-21	6.54131E-21
ENSGALG00000028167	RBM18	17	1.19668E-21	6.54896E-21
ENSGALG00000015405	PREP	3	1.20387E-21	6.58534E-21
ENSGALG00000019277	SLCO1B3	1	1.21556E-21	6.64634E-21
ENSGALG00000014381	HNRNPAB	13	1.25111E-21	6.83764E-21
ENSGALG00000000209	PRNP	22	1.25772E-21	6.8707E-21
ENSGALG00000037316	OCLN	28	1.26457E-21	6.90502E-21
ENSGALG00000011813	HEG1	7	1.27008E-21	6.93197E-21
ENSGALG00000002143	ANKLE2	15	1.27169E-21	6.93766E-21
ENSGALG00000002492	H6PD	21	1.27419E-21	6.94823E-21
ENSGALG00000006462	PPP2CA	13	1.28516E-21	7.00488E-21
ENSGALG00000005035	KAT6B	6	1.2925E-21	7.04177E-21
ENSGALG00000000660	CYB5B	11	1.33249E-21	7.25638E-21
ENSGALG00000049613		14	1.3333E-21	7.25755E-21
ENSGALG00000008807	DCUN1D1	9	1.36353E-21	7.41881E-21

ENSGALG00000008916	DNAJB4	8	1.37123E-21	7.45738E-21
ENSGALG00000005703	SLC30A4	10	1.3755E-21	7.47725E-21
ENSGALG00000006502	PCGF5	6	1.38245E-21	7.51166E-21
ENSGALG00000050615	CKAP2L	22	1.40946E-21	7.65503E-21
ENSGALG00000026182	CTTNBP2NL	26	1.44844E-21	7.86322E-21
ENSGALG00000014277	RHOH	4	1.45062E-21	7.87159E-21
ENSGALG00000054366		31	1.45621E-21	7.8984E-21
ENSGALG00000011827	WDR89	5	1.45966E-21	7.91357E-21
ENSGALG00000005652	DIDO1	20	1.46568E-21	7.94268E-21
ENSGALG00000003434	C17orf75	18	1.4694E-21	7.95929E-21
ENSGALG00000011170	WIPF3	2	1.47183E-21	7.96895E-21
ENSGALG00000036920	SLC22A5	13	1.48806E-21	8.05323E-21
ENSGALG00000013138	TMEM245	2	1.50029E-21	8.11341E-21
ENSGALG00000016047	HLCS	1	1.50051E-21	8.11341E-21
ENSGALG00000034932	BACE2	1	1.50953E-21	8.15856E-21
ENSGALG00000008187	EIF3J	10	1.53446E-21	8.28963E-21
ENSGALG00000016662	GATA4	3	1.54464E-21	8.34091E-21
ENSGALG00000015763	GABPA	1	1.59119E-21	8.58848E-21
ENSGALG00000005328	IBA57	2	1.59982E-21	8.63127E-21
ENSGALG00000038128	UBE2D2	13	1.66377E-21	8.97231E-21
ENSGALG00000005855	CA5B	11	1.68938E-21	9.10637E-21
ENSGALG00000012856	WRNIP1	2	1.69322E-21	9.12303E-21
ENSGALG00000004216	TOR3A	8	1.71139E-21	9.21686E-21
ENSGALG00000003161	GDF3	28	1.71944E-21	9.25612E-21
ENSGALG00000016387	TRAPPC12	3	1.72344E-21	9.27355E-21
ENSGALG00000017186	BIRC3	1	1.72833E-21	9.2958E-21
ENSGALG00000049475	S1PR2	30	1.74104E-21	9.35998E-21
ENSGALG00000007151	BRWD3	4	1.7506E-21	9.40727E-21
ENSGALG00000033926	CBX1	27	1.77317E-21	9.52434E-21
ENSGALG00000048698		3	1.78955E-21	9.60808E-21
ENSGALG00000008520	EIF4G1	9	1.79109E-21	9.61215E-21
ENSGALG00000001313	EIF4H	19	1.86099E-21	9.98289E-21
ENSGALG00000004696	LEO1	10	1.86963E-21	1.00248E-20

ENSGALG00000011608		5	1.87614E-21	1.00546E-20
ENSGALG00000026865	XRCC2	2	1.87683E-21	1.00546E-20
ENSGALG00000016014	RCAN1	1	2.01119E-21	1.07696E-20
ENSGALG00000011117	AGT	3	2.01435E-21	1.07818E-20
ENSGALG00000051043		1	2.02216E-21	1.08188E-20
ENSGALG00000037375	MAN1B1	17	2.03909E-21	1.09047E-20
ENSGALG00000047480		1	2.06939E-21	1.10618E-20
ENSGALG00000006780	ARL6IP1	14	2.07899E-21	1.11083E-20
ENSGALG00000003024	ARMC6	28	2.1178E-21	1.13107E-20
ENSGALG00000011068	COBLL1	7	2.1343E-21	1.13939E-20
ENSGALG00000010643	ZYG11B	8	2.1532E-21	1.14897E-20
ENSGALG00000010616	PTPN21	5	2.20545E-21	1.17634E-20
ENSGALG00000043787	DEPDC6	2	2.2319E-21	1.18992E-20
ENSGALG00000049272		33	2.2359E-21	1.19112E-20
ENSGALG00000005005	MIEF2	14	2.2361E-21	1.19112E-20
ENSGALG00000010649	CNST	3	2.24518E-21	1.19544E-20
ENSGALG00000008154	SRPK2	1	2.25541E-21	1.20036E-20
ENSGALG00000039454	SP4	2	2.25697E-21	1.20067E-20
ENSGALG00000017129	SAP18	1	2.31263E-21	1.22974E-20
ENSGALG00000015649	NOP14	4	2.34941E-21	1.24875E-20
ENSGALG00000001143	ETS1	24	2.37183E-21	1.26012E-20
ENSGALG00000006611	KIF7	10	2.391E-21	1.26929E-20
ENSGALG00000008076	TMEM164	4	2.39117E-21	1.26929E-20
ENSGALG00000039673	GALNS	11	2.39837E-21	1.27255E-20
ENSGALG00000011612	GC	4	2.41279E-21	1.27965E-20
ENSGALG00000053823	FAM219B	10	2.42051E-21	1.28319E-20
ENSGALG00000026189		18	2.48602E-21	1.31734E-20
ENSGALG00000001728	NACC2	17	2.48975E-21	1.31874E-20
ENSGALG00000012944	DENND5B	1	2.50119E-21	1.32423E-20
ENSGALG00000010929	SPARCL1	4	2.52586E-21	1.33671E-20
ENSGALG00000011854	UBN2	1	2.57153E-21	1.36014E-20
ENSGALG00000013297	BBOX1	5	2.57236E-21	1.36014E-20
ENSGALG00000016633	HMBOX1	3	2.58909E-21	1.36839E-20

ENSGALG00000015438	MPZL1	1	2.62826E-21	1.38849E-20
ENSGALG00000004144		6	2.63917E-21	1.39365E-20
ENSGALG00000047167	DNAJC30	19	2.66785E-21	1.40819E-20
ENSGALG00000035605		7	2.73321E-21	1.44206E-20
ENSGALG00000009480	DOCK4	1	2.76217E-21	1.45671E-20
ENSGALG00000012187	MGAT5	7	2.82651E-21	1.49E-20
ENSGALG00000019077		1	2.87186E-21	1.51325E-20
ENSGALG00000039704	SEC22B	8	2.89048E-21	1.5224E-20
ENSGALG00000011695	HIPK3	5	2.89884E-21	1.52615E-20
ENSGALG00000017249	PCF11	1	2.94873E-21	1.55174E-20
ENSGALG00000041200	ZNF706	2	2.97554E-21	1.56518E-20
ENSGALG00000012837	EXOC2	2	3.02096E-21	1.58838E-20
ENSGALG00000008418	LARGE2	5	3.05403E-21	1.60507E-20
ENSGALG00000011294	THRB	2	3.10366E-21	1.63046E-20
ENSGALG00000002606	CLSTN1	21	3.12061E-21	1.63866E-20
ENSGALG00000026591	GNG12	8	3.1388E-21	1.6475E-20
ENSGALG00000015951	OGFRL1	3	3.14759E-21	1.6514E-20
ENSGALG00000008274	CRBN	12	3.14914E-21	1.6515E-20
ENSGALG00000048022		2	3.1756E-21	1.66466E-20
ENSGALG00000004165	TOR1A	17	3.20054E-21	1.67702E-20
ENSGALG00000012018	UGT8	4	3.24769E-21	1.70099E-20
ENSGALG00000010592	TET2	4	3.30926E-21	1.73196E-20
ENSGALG00000034575	MRI1	30	3.30966E-21	1.73196E-20
ENSGALG00000031843	ARHGAP18	3	3.32353E-21	1.73847E-20
ENSGALG00000011958	TEF	1	3.35323E-21	1.75326E-20
ENSGALG00000015803	KDM3A	4	3.36876E-21	1.76062E-20
ENSGALG00000007702	XYLT2	18	3.3955E-21	1.77384E-20
ENSGALG00000039173	RGMA	10	3.41001E-21	1.78065E-20
ENSGALG00000015916	SLC17A5	3	3.44591E-21	1.79863E-20
ENSGALG00000032410	CBFA2T3	11	3.51387E-21	1.83332E-20
ENSGALG00000009957	TIE1	8	3.51744E-21	1.8344E-20
ENSGALG00000006020	BTBD1	10	3.52836E-21	1.8393E-20
ENSGALG00000001335	FAM18B1	18	3.53301E-21	1.84094E-20

ENSGALG00000004088	FNBP1	17	3.59296E-21	1.87138E-20
ENSGALG00000008760	WDR92	3	3.74818E-21	1.95139E-20
ENSGALG00000040388	PUDP	1	3.85484E-21	2.00606E-20
ENSGALG00000000613	RAB11B	28	3.87152E-21	2.01388E-20
ENSGALG00000030055	RIPOR1	11	3.87436E-21	2.0145E-20
ENSGALG00000039141	CERS2	25	3.90365E-21	2.02887E-20
ENSGALG00000032546	MYEF2	10	3.9384E-21	2.04605E-20
ENSGALG00000000235	PPP2R2A	22	3.99964E-21	2.07698E-20
ENSGALG00000039380	TBC1D1	4	4.06776E-21	2.11073E-20
ENSGALG00000032563	FBXO34	5	4.06808E-21	2.11073E-20
ENSGALG00000011402	CNOT9	7	4.17084E-21	2.16313E-20
ENSGALG00000014537	BMF	5	4.24025E-21	2.19819E-20
ENSGALG00000014443	IFFO1	1	4.34771E-21	2.25294E-20
ENSGALG00000012245	ADH4	4	4.37646E-21	2.26687E-20
ENSGALG00000051152		6	4.39694E-21	2.27651E-20
ENSGALG00000001407	ADAMTS15	24	4.461E-21	2.3087E-20
ENSGALG00000002124	ZDHHC8	15	4.47787E-21	2.31645E-20
ENSGALG00000001693	SLC18A1	22	4.49141E-21	2.32247E-20
ENSGALG00000033178	KLF6	2	4.51793E-21	2.33519E-20
ENSGALG00000003999	NAV2	5	4.60218E-21	2.37676E-20
ENSGALG00000036556	MORC4	1	4.60226E-21	2.37676E-20
ENSGALG00000004901	CORO1C	15	4.74866E-21	2.45133E-20
ENSGALG00000015878	LCA5	3	4.75419E-21	2.45314E-20
ENSGALG00000008786	NBEAL1	7	4.88381E-21	2.51896E-20
ENSGALG00000008034	CASKIN2	18	4.89883E-21	2.52564E-20
ENSGALG00000042766	TRAPPC9	2	4.95668E-21	2.55439E-20
ENSGALG00000012748	ELOVL2	2	5.04465E-21	2.59862E-20
ENSGALG00000011020	CYCS	2	5.11896E-21	2.63579E-20
ENSGALG00000016651		3	5.28985E-21	2.72263E-20
ENSGALG00000048802		2	5.31924E-21	2.7366E-20
ENSGALG00000002417	GMPPA	7	5.35867E-21	2.75573E-20
ENSGALG00000053836	SIPA1	33	5.36278E-21	2.75668E-20
ENSGALG00000042553	RAP2B	9	5.3874E-21	2.76817E-20

ENSGALG00000038320		8	5.47983E-21	2.81447E-20
ENSGALG00000009627	GPATCH2	3	5.48361E-21	2.81523E-20
ENSGALG00000042641	PATL1	5	5.51099E-21	2.8281E-20
ENSGALG00000017040		1	5.51494E-21	2.82893E-20
ENSGALG00000011811	SYNE2	5	5.54772E-21	2.84455E-20
ENSGALG00000008431	SMARCA1	4	5.55239E-21	2.84575E-20
ENSGALG00000015246	TIPRL	1	5.58092E-21	2.85917E-20
ENSGALG00000044018	ISOC2	22	5.59398E-21	2.86466E-20
ENSGALG00000003183	C11H16orf70	11	5.6152E-21	2.87431E-20
ENSGALG00000040823	CHRAC1	2	5.70882E-21	2.92101E-20
ENSGALG00000001121	SH3GL1	28	5.75382E-21	2.9428E-20
ENSGALG00000009381	SIPA1L1	5	5.76035E-21	2.9449E-20
ENSGALG00000031160	GRSF1	4	5.76833E-21	2.94775E-20
ENSGALG00000011280	EEA1	1	5.8304E-21	2.97822E-20
ENSGALG00000007150	OPA1	9	5.84163E-21	2.98271E-20
ENSGALG00000008025	SLF2	6	5.90428E-21	3.01343E-20
ENSGALG00000016769	CHST10	1	6.06025E-21	3.09174E-20
ENSGALG00000016289	DST	3	6.06949E-21	3.09516E-20
ENSGALG00000013602	PCM1	4	6.12808E-21	3.12373E-20
ENSGALG00000016350	ARHGEF10	3	6.21559E-21	3.16702E-20
ENSGALG00000034347	KIF21B	26	6.32065E-21	3.2192E-20
ENSGALG00000002232	RERE	21	6.33898E-21	3.22719E-20
ENSGALG00000009036	GANC	5	6.36328E-21	3.23821E-20
ENSGALG00000050253		22	6.39408E-21	3.25237E-20
ENSGALG00000031294	MPLKIP	2	6.39644E-21	3.25237E-20
ENSGALG00000003693	MACF1	23	6.8038E-21	3.45806E-20
ENSGALG00000029017	ZNRF1	11	6.98009E-21	3.54618E-20
ENSGALG00000016870	TMTC4	1	7.03868E-21	3.57446E-20
ENSGALG00000016468	GEN1	3	7.09437E-21	3.60124E-20
ENSGALG00000025780	ITPRIP	6	7.17297E-21	3.63962E-20
ENSGALG00000015537	MAN2B2	4	7.19555E-21	3.64956E-20
ENSGALG00000011481	GPD1L	2	7.2281E-21	3.66454E-20
ENSGALG00000012561	PKP4	7	7.25486E-21	3.67658E-20

ENSGALG00000000106	LRIF1	26	7.32114E-21	3.70863E-20
ENSGALG00000039575	AMD1	3	7.59302E-21	3.84475E-20
ENSGALG00000010768	PIGM	3	7.94254E-21	4.02007E-20
ENSGALG00000007128	SH3BGRL	4	8.01525E-21	4.05519E-20
ENSGALG00000007364	TOB1	18	8.14979E-21	4.12046E-20
ENSGALG00000000783	PTAFR	23	8.15102E-21	4.12046E-20
ENSGALG00000009424	FOXP2	1	8.43183E-21	4.26064E-20
ENSGALG00000007650	SLC9A3R1	18	8.55534E-21	4.32126E-20
ENSGALG00000010839	SPCS3	4	8.6319E-21	4.35813E-20
ENSGALG00000036751		14	8.76107E-21	4.42151E-20
ENSGALG00000007132	ACOX2	12	8.97437E-21	4.52729E-20
ENSGALG00000005061	PPM1E	19	8.99075E-21	4.53367E-20
ENSGALG00000002431		8	9.0025E-21	4.53772E-20
ENSGALG00000001610	PIP4K2B	27	9.108E-21	4.589E-20
ENSGALG00000048321		3	9.28635E-21	4.67693E-20
ENSGALG00000042645	PARD3B	7	9.33468E-21	4.69933E-20
ENSGALG00000001689	SCAMP2	10	9.3692E-21	4.71476E-20
ENSGALG00000009967	LRPPRC	3	9.42028E-21	4.73851E-20
ENSGALG00000005966	SEC61A1	12	9.43253E-21	4.74272E-20
ENSGALG00000011276	BTG1	1	9.61868E-21	4.83432E-20
ENSGALG00000010221	ZSWIM5	8	9.72196E-21	4.88422E-20
ENSGALG00000013941	PDE7B	3	9.72729E-21	4.88488E-20
ENSGALG00000036456	TRIB1	2	9.77353E-21	4.90608E-20
ENSGALG00000030261	SMIM12	23	9.80383E-21	4.91926E-20
ENSGALG00000007483	LRIG1	12	9.9593E-21	4.99522E-20
ENSGALG00000054795		1	1.00468E-20	5.03702E-20
ENSGALG00000010030		5	1.00662E-20	5.04469E-20
ENSGALG00000033068	TLR21	11	1.00987E-20	5.05887E-20
ENSGALG00000046805	LMAN2L	22	1.02225E-20	5.11882E-20
ENSGALG00000041934	THOP1	28	1.02891E-20	5.15007E-20
ENSGALG00000049229		16	1.05616E-20	5.28426E-20
ENSGALG00000041512		6	1.0637E-20	5.3198E-20
ENSGALG00000043613	TXNDC16	5	1.07146E-20	5.35644E-20

ENSGALG00000012080		2	1.0924E-20	5.45889E-20
ENSGALG00000035242	RMDN3	5	1.098E-20	5.4846E-20
ENSGALG00000048239		10	1.12136E-20	5.59902E-20
ENSGALG00000004193	ARSG	18	1.18095E-20	5.8941E-20
ENSGALG00000011522	TRMT61A	5	1.19417E-20	5.95766E-20
ENSGALG00000007021	PCNT	7	1.20523E-20	6.01036E-20
ENSGALG00000010109	RNF220	8	1.21076E-20	6.0355E-20
ENSGALG00000045350		4	1.21882E-20	6.07317E-20
ENSGALG00000015623	HGFAC	4	1.22035E-20	6.07832E-20
ENSGALG00000036219	STK35	20	1.22674E-20	6.10766E-20
ENSGALG00000003670	MAFB	20	1.29075E-20	6.42372E-20
ENSGALG00000050596		31	1.30336E-20	6.48384E-20
ENSGALG00000008218	DDB2	5	1.31752E-20	6.55059E-20
ENSGALG00000017034	FOXO1	1	1.31785E-20	6.55059E-20
ENSGALG00000013092	STRAP	1	1.33381E-20	6.6272E-20
ENSGALG00000014395	DHX15	4	1.35549E-20	6.73219E-20
ENSGALG00000007181	PGS1	18	1.36944E-20	6.7987E-20
ENSGALG00000014910	COLEC12	2	1.37815E-20	6.83916E-20
ENSGALG00000008504	HNRNPLL	3	1.38058E-20	6.84841E-20
ENSGALG00000004557	WIPI2	14	1.38813E-20	6.88307E-20
ENSGALG00000005248	CDC14A	8	1.40451E-20	6.96144E-20
ENSGALG00000016261	CYBB	1	1.41174E-20	6.99445E-20
ENSGALG00000039013	EXT1	2	1.41998E-20	7.03242E-20
ENSGALG00000016487	LDAH	3	1.43451E-20	7.10149E-20
ENSGALG00000012111	TMEM37	7	1.46532E-20	7.25108E-20
ENSGALG00000036286		5	1.46792E-20	7.26098E-20
ENSGALG00000015247	GPR161	1	1.48586E-20	7.34616E-20
ENSGALG00000012877	CREB3L2	1	1.48634E-20	7.34616E-20
ENSGALG00000014800	ATP6V1A	1	1.49383E-20	7.38019E-20
ENSGALG00000001439	RANBP10	11	1.49508E-20	7.38338E-20
ENSGALG00000009096	MTM1	4	1.50178E-20	7.41102E-20
ENSGALG00000005377	WWOX	11	1.5019E-20	7.41102E-20
ENSGALG00000001389		17	1.51765E-20	7.48574E-20

ENSGALG00000003308	ERLIN1	6	1.52441E-20	7.51604E-20
ENSGALG00000012932	FGD4	1	1.55178E-20	7.64789E-20
ENSGALG00000012171	GPR39	7	1.58024E-20	7.78497E-20
ENSGALG00000012401	GPR137C	5	1.58207E-20	7.79085E-20
ENSGALG00000028155	DUSP16	1	1.62391E-20	7.9937E-20
ENSGALG00000008651	APMAP	3	1.63766E-20	8.05809E-20
ENSGALG00000008525	ABHD12	3	1.63832E-20	8.05812E-20
ENSGALG00000011368	ZNF839	5	1.64156E-20	8.07079E-20
ENSGALG00000003053	PRDX6	8	1.64693E-20	8.09393E-20
ENSGALG00000048331	LRFN1	32	1.65124E-20	8.11183E-20
ENSGALG00000011957	TOB2	1	1.65663E-20	8.13503E-20
ENSGALG00000039140	CD14	13	1.66075E-20	8.15198E-20
ENSGALG00000001209	TP53INP2	20	1.66506E-20	8.16987E-20
ENSGALG00000037601	TBC1D25	30	1.67861E-20	8.23304E-20
ENSGALG00000010805	USP24	8	1.69684E-20	8.31908E-20
ENSGALG00000013968	SGCB	4	1.72225E-20	8.44029E-20
ENSGALG00000001250	APLP2	24	1.75448E-20	8.59478E-20
ENSGALG00000039536	C2H8ORF22	2	1.75947E-20	8.61577E-20
ENSGALG00000006378	LIPA	6	1.8147E-20	8.88264E-20
ENSGALG00000038160		4	1.81549E-20	8.88291E-20
ENSGALG00000051870		33	1.84344E-20	9.01609E-20
ENSGALG00000011079	GLRX5	5	1.85416E-20	9.06488E-20
ENSGALG00000001833	PRKRIP1	19	1.85749E-20	9.07752E-20
ENSGALG00000037655	BCL9	1	1.86831E-20	9.12675E-20
ENSGALG00000007227	MSI1	15	1.88369E-20	9.19816E-20
ENSGALG00000014510	FBXL5	4	1.8859E-20	9.2053E-20
ENSGALG00000033683	PHOSPHO1	27	1.89045E-20	9.22377E-20
ENSGALG00000004498	SLC2A10	20	1.8992E-20	9.26279E-20
ENSGALG00000007001	TLR4	17	1.90067E-20	9.26626E-20
ENSGALG00000037326	MAP3K9	5	1.91559E-20	9.33525E-20
ENSGALG00000008939	FUBP1	8	1.99342E-20	9.71064E-20
ENSGALG00000007832	PEX13	3	1.99697E-20	9.72406E-20
ENSGALG00000003340	GBGT1	17	2.0108E-20	9.7875E-20

ENSGALG00000029190	MBNL1	9	2.01181E-20	9.7885E-20
ENSGALG00000009805	PPM1H	1	2.01405E-20	9.79548E-20
ENSGALG00000048103	TBCC	3	2.01774E-20	9.80954E-20
ENSGALG00000000835	PLEKHJ1	28	2.03472E-20	9.88814E-20
ENSGALG00000015286	SESN1	3	2.04099E-20	9.91465E-20
ENSGALG00000015375	PLCXD2	1	2.04805E-20	9.94499E-20
ENSGALG00000005314	APPBP2	19	2.0633E-20	1.00151E-19
ENSGALG00000041337	OTUD6A	2	2.06506E-20	1.00196E-19
ENSGALG00000038222		18	2.06959E-20	1.00367E-19
ENSGALG00000003741	MYO18A	19	2.07022E-20	1.00367E-19
ENSGALG00000008332	F2	5	2.08013E-20	1.00807E-19
ENSGALG00000006642	ADORA2A	15	2.10259E-20	1.01855E-19
ENSGALG00000003495	ALDH9A1	8	2.11443E-20	1.02388E-19
ENSGALG00000011502		7	2.11538E-20	1.02393E-19
ENSGALG00000009064	FZD1	2	2.13268E-20	1.0319E-19
ENSGALG00000026167	PIK3R5	18	2.16744E-20	1.0483E-19
ENSGALG00000015195	RPS15	28	2.17868E-20	1.05332E-19
ENSGALG00000007431	MAGI1	12	2.19171E-20	1.0592E-19
ENSGALG00000038420	HSF2	3	2.20417E-20	1.06479E-19
ENSGALG00000007747	TMEM104	18	2.22312E-20	1.07352E-19
ENSGALG00000039538	CLDND1	1	2.23169E-20	1.07724E-19
ENSGALG00000030943	KRAS	1	2.23542E-20	1.07861E-19
ENSGALG00000013209	SLC39A6	2	2.23901E-20	1.07992E-19
ENSGALG00000043468	LRRC23	1	2.24048E-20	1.0802E-19
ENSGALG00000035219	ALB	4	2.24769E-20	1.08324E-19
ENSGALG00000049579		28	2.27405E-20	1.09552E-19
ENSGALG00000036909	BEND5	8	2.31048E-20	1.11263E-19
ENSGALG00000020927	ARPC5	8	2.33662E-20	1.12477E-19
ENSGALG00000034910	ZDHHC18	23	2.34E-20	1.12595E-19
ENSGALG00000008684	EIF4A2	9	2.36894E-20	1.13943E-19
ENSGALG00000000729	PPP1R8	23	2.39058E-20	1.14938E-19
ENSGALG00000010127	GLO1	3	2.39438E-20	1.15076E-19
ENSGALG00000014464	MTR	3	2.42698E-20	1.16597E-19

ENSGALG00000017333	LCMT2	1	2.4565E-20	1.17968E-19
ENSGALG00000006818		24	2.47863E-20	1.18984E-19
ENSGALG00000050893	CCR2	2	2.50592E-20	1.20247E-19
ENSGALG00000010476	BNIP3	6	2.53153E-20	1.21428E-19
ENSGALG00000041680	KCNT2	8	2.54791E-20	1.22165E-19
ENSGALG00000027757	ARL4A	2	2.55345E-20	1.22383E-19
ENSGALG00000006402	NRROS	9	2.58735E-20	1.23959E-19
ENSGALG00000020390		5	2.62668E-20	1.25794E-19
ENSGALG00000016990	LRCH1	1	2.67192E-20	1.2791E-19
ENSGALG00000021676		21	2.69494E-20	1.28961E-19
ENSGALG00000012874	DENND11	1	2.70323E-20	1.29308E-19
ENSGALG00000009170	NCEH1	9	2.72311E-20	1.30207E-19
ENSGALG00000034618	DCAF13	2	2.73978E-20	1.30953E-19
ENSGALG00000010813	GOLGA5	5	2.81489E-20	1.3449E-19
ENSGALG00000012662	NUAK1	1	2.86195E-20	1.36685E-19
ENSGALG00000032640	CRIM1	3	2.87419E-20	1.37216E-19
ENSGALG00000049882	SMPDL3A	3	2.90521E-20	1.38616E-19
ENSGALG00000048164	LHFPL4	12	2.90579E-20	1.38616E-19
ENSGALG00000032366	MEF2A	10	2.91447E-20	1.38976E-19
ENSGALG00000030879	AR	4	2.92495E-20	1.39421E-19
ENSGALG00000049588		8	3.01059E-20	1.43447E-19
ENSGALG00000009547	PPHLN1	1	3.02003E-20	1.43828E-19
ENSGALG00000025747	NQO2	2	3.02095E-20	1.43828E-19
ENSGALG00000012994	ERC1	1	3.04256E-20	1.44801E-19
ENSGALG00000042624	CEP85	23	3.07806E-20	1.46433E-19
ENSGALG00000013882	MXD1	22	3.09104E-20	1.46993E-19
ENSGALG00000011320	TMCC3	1	3.11939E-20	1.48283E-19
ENSGALG00000034050		2	3.12627E-20	1.48553E-19
ENSGALG00000040999	EIF3B	14	3.13411E-20	1.48867E-19
ENSGALG00000033597		17	3.14835E-20	1.49485E-19
ENSGALG00000005472	NAT	11	3.23592E-20	1.53583E-19
ENSGALG00000003476	ERN1	18	3.25304E-20	1.54336E-19
ENSGALG00000005750	SWAP70	5	3.27362E-20	1.55252E-19

ENSGALG00000036807	NOD1	2	3.28694E-20	1.55823E-19
ENSGALG00000035636	PHYH	1	3.31431E-20	1.57059E-19
ENSGALG00000019171	GATD3A	1	3.39497E-20	1.60819E-19
ENSGALG00000008163	PSME4	3	3.4034E-20	1.61156E-19
ENSGALG00000010595	GPR65	5	3.51897E-20	1.66564E-19
ENSGALG00000038797	MANBAL4	20	3.54155E-20	1.67567E-19
ENSGALG00000029761	SIRT2	32	3.54883E-20	1.67847E-19
ENSGALG00000003759	RORA	10	3.56165E-20	1.68388E-19
ENSGALG00000016896	ABCC4	1	3.56491E-20	1.68476E-19
ENSGALG00000039830	DHX30	2	3.56677E-20	1.68499E-19
ENSGALG00000055010	MAP1LC3B	11	3.57444E-20	1.68796E-19
ENSGALG00000033956	SFXN1	13	3.59077E-20	1.69502E-19
ENSGALG00000029083	NXPH2	7	3.62337E-20	1.70974E-19
ENSGALG00000002725	UAP1	8	3.64954E-20	1.72097E-19
ENSGALG00000013672	TSHZ1	2	3.64998E-20	1.72097E-19
ENSGALG00000010658	RWDD4	4	3.6902E-20	1.73926E-19
ENSGALG00000053799		8	3.75016E-20	1.76683E-19
ENSGALG00000007707	TRAPPC4	24	3.76613E-20	1.77367E-19
ENSGALG00000007963	NFATC2	20	3.80804E-20	1.79272E-19
ENSGALG00000008217	RNFT2	15	3.81445E-20	1.79499E-19
ENSGALG00000040886		20	3.8158E-20	1.79499E-19
ENSGALG00000047642	TMEM42	2	3.81925E-20	1.79592E-19
ENSGALG00000009252	PLD1	9	3.82818E-20	1.79942E-19
ENSGALG00000035824	ARSA	1	3.83211E-20	1.80057E-19
ENSGALG00000023571	TRIM59	9	3.86508E-20	1.81537E-19
ENSGALG00000028565	PCDHGC3	13	3.88888E-20	1.82584E-19
ENSGALG00000031375	CHST7	1	3.91924E-20	1.83939E-19
ENSGALG00000003185	SCPEP1	18	3.92385E-20	1.84084E-19
ENSGALG00000035752	PEX2	2	3.95032E-20	1.85255E-19
ENSGALG00000009505	PNPLA8	1	4.11444E-20	1.92877E-19
ENSGALG00000012530	FOXRED2	1	4.12085E-20	1.93104E-19
ENSGALG00000008730	CYP20A1	7	4.1316E-20	1.93533E-19
ENSGALG00000035065	FAM110B	2	4.1496E-20	1.94301E-19

ENSGALG00000036295	DFFB	21	4.16915E-20	1.95142E-19
ENSGALG00000013134	PLEKHA5	1	4.19247E-20	1.96158E-19
ENSGALG00000043052	CD1B	16	4.22599E-20	1.9765E-19
ENSGALG00000029896		13	4.23886E-20	1.98176E-19
ENSGALG00000029292	PCNA	22	4.25105E-20	1.9867E-19
ENSGALG00000026421	PSMB2	23	4.25649E-20	1.98848E-19
ENSGALG00000016817	RASA3	1	4.38668E-20	2.04852E-19
ENSGALG00000008689		4	4.39263E-20	2.05012E-19
ENSGALG00000008589	CMKLR2	7	4.39347E-20	2.05012E-19
ENSGALG00000006695	BCR	15	4.40291E-20	2.05374E-19
ENSGALG00000003578	FN1	7	4.41372E-20	2.05799E-19
ENSGALG00000011106	GALNT2	3	4.45303E-20	2.07553E-19
ENSGALG00000032250	COQ10A	33	4.5103E-20	2.10142E-19
ENSGALG00000012978	CDYL2	11	4.52864E-20	2.10915E-19
ENSGALG00000013438	TEX10	2	4.53603E-20	2.11179E-19
ENSGALG00000030363	IFNAR1	1	4.54177E-20	2.11342E-19
ENSGALG00000015605	BACH2	3	4.54298E-20	2.11342E-19
ENSGALG00000004413	SLC39A11	18	4.54562E-20	2.11383E-19
ENSGALG00000016463	FAM49A	3	4.55911E-20	2.1193E-19
ENSGALG00000006298	ATG4B	9	4.61277E-20	2.14343E-19
ENSGALG00000007758	OSBP2	15	4.68912E-20	2.17808E-19
ENSGALG00000005504	HNF1B	19	4.7849E-20	2.22172E-19
ENSGALG00000012791	TBXAS1	1	4.81878E-20	2.2366E-19
ENSGALG00000011312	OXSM	2	4.90175E-20	2.27424E-19
ENSGALG00000042482	RAB5B	33	4.93955E-20	2.29091E-19
ENSGALG00000047230	SWI5	17	4.96179E-20	2.30034E-19
ENSGALG00000044889	FYB2	8	5.09356E-20	2.36054E-19
ENSGALG00000017171	CWF19L2	1	5.16954E-20	2.39484E-19
ENSGALG00000000186	MTCH1	26	5.19236E-20	2.4045E-19
ENSGALG00000012801	LY86	2	5.23382E-20	2.42278E-19
ENSGALG00000000564	CCDC47	27	5.24424E-20	2.42668E-19
ENSGALG00000029523	RAD21	2	5.26658E-20	2.43609E-19
ENSGALG00000003736	ABCB9	15	5.40243E-20	2.49798E-19

ENSGALG00000008875	PLCB1	3	5.52237E-20	2.55247E-19
ENSGALG00000047610		31	5.73844E-20	2.65134E-19
ENSGALG00000005534	TM9SF3	6	5.79013E-20	2.67421E-19
ENSGALG00000026360	SOWAHC	1	5.82488E-20	2.68924E-19
ENSGALG00000010351	POMGNT1	8	5.86404E-20	2.70629E-19
ENSGALG00000033806	ITPRIPL2	14	5.87795E-20	2.71169E-19
ENSGALG00000012784	TXNDC5	2	5.88027E-20	2.71173E-19
ENSGALG00000004166	VPS51	5	5.89399E-20	2.71703E-19
ENSGALG00000039541	SH3GLB2	17	5.89985E-20	2.7187E-19
ENSGALG00000010073	DDOST	21	6.23657E-20	2.87278E-19
ENSGALG00000007098	KIF21A	1	6.35486E-20	2.92617E-19
ENSGALG00000026995	RHOG	1	6.41803E-20	2.95413E-19
ENSGALG00000007119	DAGLB	14	6.44969E-20	2.96759E-19
ENSGALG00000038618	ONECUT1	10	6.54694E-20	3.01083E-19
ENSGALG00000040921	RRM2B	2	6.54953E-20	3.01083E-19
ENSGALG00000001788	ADPGK	10	6.55108E-20	3.01083E-19
ENSGALG00000031448	MAPK4	10	6.60691E-20	3.03438E-19
ENSGALG00000008473	PLXND1	12	6.60729E-20	3.03438E-19
ENSGALG00000015022	FYN	3	6.67012E-20	3.06208E-19
ENSGALG00000043129	DEDD	25	6.76164E-20	3.10292E-19
ENSGALG00000012298	SLC39A8	4	6.76566E-20	3.1036E-19
ENSGALG00000016415	MAP7D2	1	6.84433E-20	3.13851E-19
ENSGALG00000010049	PTPRB	1	6.87361E-20	3.15075E-19
ENSGALG00000004378	ASB6	17	6.89864E-20	3.16103E-19
ENSGALG00000027199	MMGT1	4	6.99098E-20	3.20214E-19
ENSGALG00000013063	SRD5A1	2	6.99381E-20	3.20223E-19
ENSGALG00000042990	IFT27	1	7.07066E-20	3.23621E-19
ENSGALG00000009686	PPP1R9A	2	7.11375E-20	3.25471E-19
ENSGALG00000032930	NCL	9	7.16266E-20	3.27537E-19
ENSGALG00000027190	COX11	18	7.16427E-20	3.27537E-19
ENSGALG00000002488	PAIP2B	13	7.26572E-20	3.3205E-19
ENSGALG00000000311	DOCK5	22	7.38446E-20	3.3735E-19
ENSGALG00000004675	RGS9BP	11	7.40226E-20	3.38036E-19

ENSGALG00000010481	CMPK1	8	7.6797E-20	3.50575E-19
ENSGALG00000054442		12	7.73491E-20	3.52963E-19
ENSGALG00000003409	SOGA1	20	7.82427E-20	3.56908E-19
ENSGALG00000008953	AASS	1	7.93415E-20	3.61784E-19
ENSGALG00000028262		26	8.05268E-20	3.67052E-19
ENSGALG00000006956	ARIH2	12	8.0775E-20	3.68046E-19
ENSGALG00000030889	FOXA2	3	8.12444E-20	3.70046E-19
ENSGALG00000008779	ACTR2	3	8.15616E-20	3.71352E-19
ENSGALG00000003520	POLG2	18	8.20945E-20	3.73639E-19
ENSGALG00000009309	EIF5A2	9	8.23E-20	3.74434E-19
ENSGALG00000012699	MCUR1	2	8.30548E-20	3.77728E-19
ENSGALG00000013086	IKZF1	2	8.36614E-20	3.80344E-19
ENSGALG00000038978		2	8.54534E-20	3.88347E-19
ENSGALG00000035295	AP4B1	26	8.62664E-20	3.91895E-19
ENSGALG00000005744	EMC8	11	8.81873E-20	4.00472E-19
ENSGALG00000011448	ERI1	4	8.83763E-20	4.01181E-19
ENSGALG00000048687		1	8.86599E-20	4.02319E-19
ENSGALG00000011323	SNAPC3	1	8.93981E-20	4.05517E-19
ENSGALG00000000259	PSMF1	20	9.19535E-20	4.16954E-19
ENSGALG00000025851	GPRIN3	4	9.50257E-20	4.30724E-19
ENSGALG00000021128	FSCN1	14	9.71319E-20	4.40107E-19
ENSGALG00000000830		1	9.72407E-20	4.40437E-19
ENSGALG00000034187	SEPTIN8	13	9.81182E-20	4.44246E-19
ENSGALG00000047027		2	9.93045E-20	4.49451E-19
ENSGALG00000011581	KIF26A	5	9.95788E-20	4.50525E-19
ENSGALG00000014508	CD38	4	1.02605E-19	4.64044E-19
ENSGALG00000035626	DAD1	27	1.03506E-19	4.67946E-19
ENSGALG00000006118	ZC3H18	11	1.06914E-19	4.83174E-19
ENSGALG00000030759	SRPRA	24	1.07512E-19	4.85695E-19
ENSGALG00000016687	P2RY8	1	1.08614E-19	4.90491E-19
ENSGALG00000013932	NAPG	2	1.08946E-19	4.91808E-19
ENSGALG00000004743	MAPKAPK5	15	1.09207E-19	4.92807E-19
ENSGALG00000016862	POGLUT2	1	1.11295E-19	5.02041E-19

ENSGALG00000028979	FN3KRP	18	1.12207E-19	5.05968E-19
ENSGALG00000004711	PTCD1	14	1.12703E-19	5.08019E-19
ENSGALG00000014336	RELL1	4	1.13379E-19	5.10876E-19
ENSGALG00000035630	UBP1	2	1.13686E-19	5.1207E-19
ENSGALG00000010716	AGA	4	1.14703E-19	5.16461E-19
ENSGALG00000045163	APH1A	25	1.1477E-19	5.1657E-19
ENSGALG00000010957	USP1	8	1.15527E-19	5.19787E-19
ENSGALG00000053399		3	1.16498E-19	5.2396E-19
ENSGALG00000041102		17	1.18078E-19	5.30871E-19
ENSGALG00000035718	DOK1	4	1.18606E-19	5.33049E-19
ENSGALG00000007999	MRPS7	18	1.18691E-19	5.33234E-19
ENSGALG00000033568	TMEM70	2	1.18932E-19	5.34122E-19
ENSGALG00000038000	CX3CR1	2	1.19327E-19	5.35698E-19
ENSGALG00000002272	NOC4L	15	1.21555E-19	5.45497E-19
ENSGALG00000006546	CD81	5	1.23E-19	5.51779E-19
ENSGALG00000023898		11	1.23213E-19	5.52534E-19
ENSGALG00000005953	RPAP2	8	1.23534E-19	5.5377E-19
ENSGALG00000002028	GPRC5B	14	1.23862E-19	5.55033E-19
ENSGALG00000002347	DIPK2A	9	1.24047E-19	5.5566E-19
ENSGALG00000007022	PGPEP1L	10	1.24172E-19	5.56017E-19
ENSGALG00000007145	ITGB1	2	1.24261E-19	5.56207E-19
ENSGALG00000051904	NLGN2	15	1.26061E-19	5.64059E-19
ENSGALG00000002018	CAPN15	14	1.26625E-19	5.66375E-19
ENSGALG00000008128	MOB4	7	1.27828E-19	5.71546E-19
ENSGALG00000038551		1	1.28101E-19	5.72556E-19
ENSGALG00000001024	TPRG1L	21	1.28521E-19	5.74225E-19
ENSGALG00000041547	DCP1A	12	1.29593E-19	5.78802E-19
ENSGALG00000002761	SIRT1	6	1.32248E-19	5.90441E-19
ENSGALG00000012559	LARGE1	1	1.32787E-19	5.92631E-19
ENSGALG00000009944	KAT7	27	1.34033E-19	5.97912E-19
ENSGALG00000032190	MICAL1	26	1.34068E-19	5.97912E-19
ENSGALG00000022980	CDKN1B	1	1.34363E-19	5.99008E-19
ENSGALG00000002240	C3orf18	12	1.34439E-19	5.99129E-19

ENSGALG00000048524	SHARPIN	2	1.34655E-19	5.99874E-19
ENSGALG00000009581	MPP5	5	1.35995E-19	6.0562E-19
ENSGALG00000005533	ZMYM3	4	1.36175E-19	6.06202E-19
ENSGALG00000015055	SLC22A16	3	1.37718E-19	6.12845E-19
ENSGALG00000020251	FNIP2	4	1.39091E-19	6.18731E-19
ENSGALG00000004591	SERPINC1	8	1.4191E-19	6.31039E-19
ENSGALG00000011403	SRP72	4	1.42183E-19	6.31934E-19
ENSGALG00000045557	MTTPL	6	1.42215E-19	6.31934E-19
ENSGALG00000000895	NUDC	23	1.42371E-19	6.32397E-19
ENSGALG00000048814		16	1.43689E-19	6.38018E-19
ENSGALG00000041037	YIPF3	3	1.4561E-19	6.46312E-19
ENSGALG00000012321	MAP4K5	5	1.47598E-19	6.549E-19
ENSGALG00000011278	KAT2B	2	1.50713E-19	6.68319E-19
ENSGALG00000037791	FRMD4B	12	1.50732E-19	6.68319E-19
ENSGALG00000010884	DDX24	5	1.54802E-19	6.86114E-19
ENSGALG00000001816	MAGI3	26	1.5495E-19	6.8652E-19
ENSGALG00000038646	EIF1B	2	1.55118E-19	6.87014E-19
ENSGALG00000032349	CDH5	11	1.56468E-19	6.92744E-19
ENSGALG00000005902	RAB7A	12	1.57632E-19	6.97644E-19
ENSGALG00000028568	DNAL1	5	1.59205E-19	7.04351E-19
ENSGALG00000006906	CHM	4	1.63015E-19	7.20942E-19
ENSGALG00000053398	TMEM80	5	1.63099E-19	7.21055E-19
ENSGALG00000016385	TSSC1	3	1.65453E-19	7.31194E-19
ENSGALG00000007821	DNAJA3	14	1.6658E-19	7.35908E-19
ENSGALG00000016649	MTMR9	3	1.67631E-19	7.40286E-19
ENSGALG00000005667	UBE2G2	9	1.68418E-19	7.43491E-19
ENSGALG00000037506	SIPA1L2	3	1.68724E-19	7.44572E-19
ENSGALG00000036870	ZNF767	2	1.69915E-19	7.49555E-19
ENSGALG00000031763	EDARADD	3	1.70742E-19	7.52933E-19
ENSGALG00000011950	ACO2	1	1.7164E-19	7.56618E-19
ENSGALG00000006417	SEC24A	13	1.73927E-19	7.66423E-19
ENSGALG00000036901	TSPAN18	5	1.74091E-19	7.6687E-19
ENSGALG00000018700	PLPP7	17	1.74778E-19	7.69615E-19

ENSGALG00000044251	RASSF7	5	1.80516E-19	7.94596E-19
ENSGALG00000011155	ARV1	3	1.8373E-19	8.08454E-19
ENSGALG00000050425		18	1.85033E-19	8.13891E-19
ENSGALG00000049137	SHLD1	3	1.85288E-19	8.14721E-19
ENSGALG00000013427	SORBS2	4	1.86457E-19	8.19563E-19
ENSGALG00000044326		31	1.87157E-19	8.22344E-19
ENSGALG00000029909	DHDDS	23	1.89776E-19	8.33551E-19
ENSGALG00000006766		15	1.91738E-19	8.41593E-19
ENSGALG00000033816	KCTD12	1	1.91745E-19	8.41593E-19
ENSGALG00000003778	TMEM38A	28	1.93265E-19	8.47959E-19
ENSGALG00000039391	ZC3H12B	4	1.94765E-19	8.54233E-19
ENSGALG00000006912	CPEB3	6	1.95408E-19	8.56746E-19
ENSGALG00000015412	ARL13B	1	2.00682E-19	8.79551E-19
ENSGALG00000001201	GFOD2	11	2.00968E-19	8.8049E-19
ENSGALG00000054057		3	2.0156E-19	8.82767E-19
ENSGALG00000010320	PAWR	1	2.03475E-19	8.90832E-19
ENSGALG00000021180	PAQR9	9	2.03578E-19	8.90963E-19
ENSGALG00000009445	INPP5F	6	2.04689E-19	8.95504E-19
ENSGALG00000031105	SLC4A4	4	2.07744E-19	9.08546E-19
ENSGALG00000031270	CSK	10	2.11968E-19	9.26688E-19
ENSGALG00000005579	GFER	14	2.15372E-19	9.41228E-19
ENSGALG00000005077	F8	4	2.19361E-19	9.58317E-19
ENSGALG00000015249		1	2.21102E-19	9.65578E-19
ENSGALG00000014226	KIAA0930	1	2.22261E-19	9.70292E-19
ENSGALG00000040729		5	2.2285E-19	9.72515E-19
ENSGALG00000005034	ACAD9	12	2.23091E-19	9.73221E-19
ENSGALG00000011609		5	2.23801E-19	9.7597E-19
ENSGALG00000034992	FRMD4A	1	2.25612E-19	9.83514E-19
ENSGALG00000013795	HIVEP2	3	2.29779E-19	1.00132E-18
ENSGALG00000008572	LAMP2	4	2.31907E-19	1.01023E-18
ENSGALG00000015458	ILDR2	1	2.33502E-19	1.01682E-18
ENSGALG00000003293	MBTPS1	11	2.33825E-19	1.01786E-18
ENSGALG00000035035		19	2.35039E-19	1.02278E-18

ENSGALG00000042016	ATG101	33	2.35775E-19	1.02562E-18
ENSGALG00000034303	C1orf115	3	2.39968E-19	1.04349E-18
ENSGALG00000010364	AADAC	9	2.41225E-19	1.04858E-18
ENSGALG00000026600	C1orf198	3	2.42013E-19	1.05163E-18
ENSGALG00000006652	DIP2C	2	2.42694E-19	1.05421E-18
ENSGALG00000032041		31	2.45875E-19	1.06765E-18
ENSGALG00000010284	GMPS	9	2.4842E-19	1.07831E-18
ENSGALG00000005924		13	2.57406E-19	1.11692E-18
ENSGALG00000010055	DAAM2	3	2.60168E-19	1.1285E-18
ENSGALG00000013468	TLR3	4	2.65761E-19	1.15235E-18
ENSGALG00000032840		3	2.72539E-19	1.18132E-18
ENSGALG00000026590	KLF13	10	2.75159E-19	1.19226E-18
ENSGALG00000047977	NANP	3	2.79433E-19	1.21035E-18
ENSGALG00000011241	RFTN1	2	2.7989E-19	1.2119E-18
ENSGALG00000003078	MMD	18	2.8002E-19	1.21203E-18
ENSGALG00000038722	SORBS1	6	2.92215E-19	1.26437E-18
ENSGALG00000033379	EAF1	2	2.97053E-19	1.28484E-18
ENSGALG00000042001		4	3.0608E-19	1.32342E-18
ENSGALG00000017084	UBL3	1	3.07057E-19	1.32717E-18
ENSGALG00000028081	ONECUT3	28	3.11636E-19	1.34648E-18
ENSGALG00000014159	PACSIN2	1	3.20016E-19	1.3822E-18
ENSGALG00000042599	TMEM64	2	3.21558E-19	1.38837E-18
ENSGALG00000031265	MTIF2	3	3.22388E-19	1.39146E-18
ENSGALG00000012308	MANBA	4	3.31385E-19	1.42979E-18
ENSGALG00000031210		8	3.3167E-19	1.43051E-18
ENSGALG00000029429	TGFBI	13	3.39075E-19	1.46193E-18
ENSGALG00000047316		9	3.4583E-19	1.49053E-18
ENSGALG00000001547	CAPZA1	26	3.57823E-19	1.54167E-18
ENSGALG00000032570	LANCL2	2	3.60124E-19	1.55104E-18
ENSGALG00000002959	NRBF2	6	3.69295E-19	1.58998E-18
ENSGALG00000003392	SBNO1	15	3.69579E-19	1.59064E-18
ENSGALG00000016382	MBTPS2	1	3.70607E-19	1.5945E-18
ENSGALG00000006117	UBFD1	14	3.71002E-19	1.59563E-18

ENSGALG00000009678	KCTD3	3	3.75465E-19	1.61426E-18
ENSGALG00000031117	STK17A	2	3.79437E-19	1.63076E-18
ENSGALG00000042137	ELK4	26	3.81573E-19	1.63937E-18
ENSGALG00000033965	SCAF4	1	3.884E-19	1.6671E-18
ENSGALG00000011488	CMTM7	2	3.88407E-19	1.6671E-18
ENSGALG00000002786	PSTPIP1	10	3.88439E-19	1.6671E-18
ENSGALG00000041722	STX1A	19	3.89999E-19	1.67321E-18
ENSGALG00000004727	ULK2	19	3.92027E-19	1.68132E-18
ENSGALG00000051438	EXTL2	8	3.95699E-19	1.69637E-18
ENSGALG00000011683	CENPC	4	3.95815E-19	1.69637E-18
ENSGALG00000003559	ACTR5	20	3.97622E-19	1.70352E-18
ENSGALG00000020262	NKAPL	4	3.98998E-19	1.70881E-18
ENSGALG00000050205		6	4.01464E-19	1.71877E-18
ENSGALG00000034188	PAG1	2	4.037E-19	1.72773E-18
ENSGALG00000015151	B4GALT6	2	4.03985E-19	1.72835E-18
ENSGALG00000005997	WDR44	4	4.0449E-19	1.7299E-18
ENSGALG00000012452	MIS18BP1	5	4.05858E-19	1.73498E-18
ENSGALG00000017111	NUP58	1	4.05963E-19	1.73498E-18
ENSGALG00000044813	THUMPD3	12	4.12885E-19	1.76395E-18
ENSGALG00000002504	RO60	8	4.22474E-19	1.80428E-18
ENSGALG00000012275	EPM2A	3	4.22944E-19	1.80566E-18
ENSGALG00000002855	SARDH	17	4.2314E-19	1.80586E-18
ENSGALG00000028696	FTL	5	4.24132E-19	1.80946E-18
ENSGALG00000039072		25	4.27566E-19	1.82348E-18
ENSGALG00000054412		4	4.32307E-19	1.84305E-18
ENSGALG00000009801	C5H15orf41	5	4.33195E-19	1.84619E-18
ENSGALG00000033828	PYCR3	2	4.37453E-19	1.86369E-18
ENSGALG00000010934	PATJ	8	4.38881E-19	1.86911E-18
ENSGALG00000011172		7	4.41277E-19	1.87866E-18
ENSGALG00000050058	REEP1	4	4.45426E-19	1.89566E-18
ENSGALG00000041419		12	4.64534E-19	1.97629E-18
ENSGALG00000011375	RNF25	7	4.67663E-19	1.98891E-18
ENSGALG00000005232	USP54	6	4.69162E-19	1.99459E-18

ENSGALG00000012823	TRIM24	1	4.74787E-19	2.0178E-18
ENSGALG00000051117	GIMD1	4	4.83328E-19	2.05339E-18
ENSGALG00000054723	AMOTL2	9	4.8575E-19	2.06296E-18
ENSGALG00000041112	TRAM1L1	2	4.93062E-19	2.09328E-18
ENSGALG00000009202	RBM45	7	4.98588E-19	2.116E-18
ENSGALG00000031380	DENND3	2	5.02829E-19	2.13326E-18
ENSGALG00000005078	NUP210	12	5.03453E-19	2.13517E-18
ENSGALG00000011805		4	5.07433E-19	2.15068E-18
ENSGALG00000029324		11	5.07464E-19	2.15068E-18
ENSGALG00000039190	CDK2AP2	5	5.09167E-19	2.15715E-18
ENSGALG00000040724	AKAP9	2	5.11279E-19	2.16535E-18
ENSGALG00000011384	RCOR1	5	5.13234E-19	2.17287E-18
ENSGALG00000031440	PTPRU	23	5.1857E-19	2.1947E-18
ENSGALG00000050069		22	5.19785E-19	2.19908E-18
ENSGALG00000037784	DAG1	12	5.32881E-19	2.2537E-18
ENSGALG00000051101		23	5.34318E-19	2.259E-18
ENSGALG00000027950	TXNDC17	19	5.34725E-19	2.25993E-18
ENSGALG00000054081		4	5.37064E-19	2.26903E-18
ENSGALG00000036883	MET	1	5.38715E-19	2.27522E-18
ENSGALG00000035492	ELMO1	2	5.48549E-19	2.31595E-18
ENSGALG00000040275	RCSD1	1	5.50154E-19	2.32192E-18
ENSGALG00000010315	GTPBP2	3	5.50623E-19	2.3231E-18
ENSGALG00000013235	PDK1	7	5.56922E-19	2.34886E-18
ENSGALG00000027791	FGF23	1	5.60538E-19	2.36329E-18
ENSGALG00000028988	ART7C	1	5.63071E-19	2.37315E-18
ENSGALG00000008359	CD248	3	5.63745E-19	2.37517E-18
ENSGALG00000001565	C5	17	5.64678E-19	2.37828E-18
ENSGALG00000010079	HP1BP3	21	5.6656E-19	2.38538E-18
ENSGALG00000016556		3	5.68083E-19	2.39097E-18
ENSGALG00000029789	PPP1R2	9	5.8381E-19	2.45631E-18
ENSGALG00000026299	C3orf52	1	5.88036E-19	2.47324E-18
ENSGALG00000041746	ZMAT2	13	5.90222E-19	2.48158E-18
ENSGALG00000041408		11	5.93667E-19	2.4952E-18

ENSGALG00000016855	ARGLU1	1	6.09322E-19	2.56012E-18
ENSGALG00000001938	SLC25A24	8	6.09947E-19	2.56186E-18
ENSGALG00000019867	WDCP	3	6.11467E-19	2.56736E-18
ENSGALG00000015568	SH3TC1	4	6.1597E-19	2.58538E-18
ENSGALG00000004981	TSEN2	12	6.16682E-19	2.58748E-18
ENSGALG00000043596	SNED1	9	6.16968E-19	2.58778E-18
ENSGALG00000014708	GSTK1	1	6.17301E-19	2.58829E-18
ENSGALG00000040244	SEPTIN9	18	6.18386E-19	2.59195E-18
ENSGALG00000050803	GIPC1	30	6.31623E-19	2.64652E-18
ENSGALG00000035533	ADIPOR2	1	6.44472E-19	2.69866E-18
ENSGALG00000029701	INO80D	7	6.44509E-19	2.69866E-18
ENSGALG00000016925	TBC1D4	1	6.53605E-19	2.73581E-18
ENSGALG00000017009	KPNA3	1	6.59777E-19	2.76069E-18
ENSGALG00000033092	TMEM127	22	6.62217E-19	2.76995E-18
ENSGALG00000015396	CD200	1	6.63253E-19	2.77334E-18
ENSGALG00000008862	DNAJC10	7	6.66651E-19	2.78659E-18
ENSGALG00000009306	SLC2A2	9	6.67065E-19	2.78736E-18
ENSGALG00000001817	CTCF	11	6.68758E-19	2.79348E-18
ENSGALG00000016548	SYAP1	1	6.7272E-19	2.80907E-18
ENSGALG00000015435		1	6.77659E-19	2.82872E-18
ENSGALG00000038776	SYDE2	8	6.81391E-19	2.84333E-18
ENSGALG00000026507	FDX1	1	6.8477E-19	2.85645E-18
ENSGALG00000029576	PDHB	12	6.85958E-19	2.86042E-18
ENSGALG00000004771	C1QB	21	6.91983E-19	2.88456E-18
ENSGALG00000036365	POGZ	25	6.9231E-19	2.88494E-18
ENSGALG00000000067	SPR	4	6.95091E-19	2.89554E-18
ENSGALG00000002888	CASZ1	21	7.0124E-19	2.92016E-18
ENSGALG00000054005	PPP1R37	32	7.04969E-19	2.93468E-18
ENSGALG00000036064	PAXIP1	2	7.11257E-19	2.95985E-18
ENSGALG00000011562	PRKN	3	7.26105E-19	3.02061E-18
ENSGALG00000000055	LAMTOR3	4	7.28629E-19	3.03007E-18
ENSGALG00000030542	SEMA6D	10	7.29277E-19	3.03173E-18
ENSGALG00000023510	ERP29	15	7.42504E-19	3.08567E-18

ENSGALG00000034011	PMS1	7	7.66378E-19	3.1838E-18
ENSGALG00000001895	PANK3	13	7.69639E-19	3.19626E-18
ENSGALG00000042481	FARS2	2	7.7411E-19	3.21373E-18
ENSGALG00000007798	SHQ1	12	7.76922E-19	3.22431E-18
ENSGALG00000010097	GATB	4	7.8733E-19	3.26639E-18
ENSGALG00000009927	RAP1B	1	7.92752E-19	3.28776E-18
ENSGALG00000012119	MARCO	7	8.04684E-19	3.33612E-18
ENSGALG00000030112		2	8.05764E-19	3.33946E-18
ENSGALG00000049318		3	8.08393E-19	3.34921E-18
ENSGALG00000004959	IRS1	9	8.12757E-19	3.36615E-18
ENSGALG00000008764	FAM171A1	2	8.15626E-19	3.37689E-18
ENSGALG00000035068	DNAJC13	2	8.27617E-19	3.42537E-18
ENSGALG00000009898	GAB1	4	8.32418E-19	3.44407E-18
ENSGALG00000008836	ATP6AP1	1	8.50038E-19	3.51578E-18
ENSGALG00000004650	BRAP	15	8.51625E-19	3.52115E-18
ENSGALG00000010428	FAM98A	3	8.65381E-19	3.57681E-18
ENSGALG00000011796		1	8.69636E-19	3.59318E-18
ENSGALG00000052488	TMEM186	14	8.78113E-19	3.6261E-18
ENSGALG00000010764	FBXO8	4	8.78198E-19	3.6261E-18
ENSGALG00000003447	FRS3	26	8.82027E-19	3.64068E-18
ENSGALG00000010594	FEZ2	3	8.86124E-19	3.65635E-18
ENSGALG00000016602	ARHGAP6	1	9.11574E-19	3.76009E-18
ENSGALG00000017493		1	9.20946E-19	3.79746E-18
ENSGALG00000010925	FAM126A	2	9.28075E-19	3.82556E-18
ENSGALG00000009850	NOVA1	5	9.35707E-19	3.85572E-18
ENSGALG00000016409	KIDINS220	3	9.44083E-19	3.88892E-18
ENSGALG00000042067	SPRY1	4	9.55656E-19	3.93526E-18
ENSGALG00000006457	RBMXL1	4	9.62146E-19	3.96065E-18
ENSGALG00000011225	SLC25A47	5	9.71562E-19	3.99806E-18
ENSGALG00000029514	MAPK8IP3	14	9.77156E-19	4.01973E-18
ENSGALG00000000814	HNRNPR	23	9.81255E-19	4.03523E-18
ENSGALG00000054812		1	9.87274E-19	4.05861E-18
ENSGALG00000006428	UBE2I	14	9.99811E-19	4.10825E-18

ENSGALG00000009314	CACUL1	6	1.00002E-18	4.10825E-18
ENSGALG00000032893	SHISA4	26	1.03694E-18	4.25848E-18
ENSGALG00000017132	XPO4	1	1.05004E-18	4.31083E-18
ENSGALG00000013754	PLAGL1	3	1.06394E-18	4.36643E-18
ENSGALG00000052873	MAPKBP1	5	1.07584E-18	4.41375E-18
ENSGALG00000024053	ICMT	21	1.08649E-18	4.45595E-18
ENSGALG00000007996	ANKRD44	7	1.10929E-18	4.54793E-18
ENSGALG00000008860	LONRF3	4	1.11718E-18	4.57877E-18
ENSGALG00000001117	PFKFB2	26	1.11972E-18	4.58763E-18
ENSGALG00000009028	BTBD3	3	1.13228E-18	4.6375E-18
ENSGALG00000013098	MGST1	1	1.14185E-18	4.67515E-18
ENSGALG00000033077	TRMT1L	8	1.15137E-18	4.71253E-18
ENSGALG00000035447		14	1.1617E-18	4.75321E-18
ENSGALG00000038834	MAGI2	1	1.20653E-18	4.935E-18
ENSGALG00000017156	DDX10	1	1.20801E-18	4.93938E-18
ENSGALG00000014949	RABL3	1	1.21307E-18	4.95842E-18
ENSGALG00000018803	TMEM198	7	1.22452E-18	5.00352E-18
ENSGALG00000013034	CECR2	1	1.2282E-18	5.01691E-18
ENSGALG00000035903	FAM46A	3	1.25732E-18	5.13415E-18
ENSGALG00000034769	USP12-like	4	1.26399E-18	5.15963E-18
ENSGALG00000001863	VTG2	8	1.2655E-18	5.16406E-18
ENSGALG00000050049	C3orf70	9	1.26601E-18	5.16443E-18
ENSGALG00000037396	ASB8	33	1.27987E-18	5.21923E-18
ENSGALG00000004625	ATXN2	15	1.30611E-18	5.32445E-18
ENSGALG00000052558		4	1.31004E-18	5.33868E-18
ENSGALG00000031134	UBE4A	24	1.31743E-18	5.36699E-18
ENSGALG00000040875	EDC4	11	1.33038E-18	5.41793E-18
ENSGALG00000043039	HSBP1	11	1.3425E-18	5.46546E-18
ENSGALG00000002021	ZC3H12A	23	1.35889E-18	5.53037E-18
ENSGALG00000029360		14	1.36199E-18	5.54114E-18
ENSGALG00000004379	BCL7A	15	1.39202E-18	5.6614E-18
ENSGALG00000047797		2	1.41245E-18	5.74257E-18
ENSGALG00000013993	VNN1	3	1.43103E-18	5.81619E-18

ENSGALG00000043701	ELK3	1	1.44822E-18	5.88408E-18
ENSGALG00000005237	FGD3	12	1.47115E-18	5.97527E-18
ENSGALG00000040393	BORCS5	1	1.47357E-18	5.98311E-18
ENSGALG00000030081	SKAP2	2	1.48198E-18	6.01524E-18
ENSGALG00000019201	HSPA13	1	1.49382E-18	6.06129E-18
ENSGALG00000026119	MN1	15	1.50338E-18	6.09803E-18
ENSGALG00000011178	ENOPH1	4	1.54937E-18	6.28249E-18
ENSGALG00000008127	SPI1	5	1.59417E-18	6.46095E-18
ENSGALG00000042784	TPPP	2	1.59444E-18	6.46095E-18
ENSGALG00000007076	CCNY	2	1.59574E-18	6.46407E-18
ENSGALG00000014901	METTL16	1	1.60046E-18	6.48102E-18
ENSGALG00000024391	CACTIN	28	1.60339E-18	6.49077E-18
ENSGALG00000003800	PABPC4	23	1.62636E-18	6.58154E-18
ENSGALG00000013803	ADGRG6	3	1.63445E-18	6.6121E-18
ENSGALG00000007620	GFRA3	13	1.6399E-18	6.63195E-18
ENSGALG00000010652	SCP2	8	1.6467E-18	6.65724E-18
ENSGALG00000004848	YBX1	21	1.64887E-18	6.66378E-18
ENSGALG00000026443	RNF44	13	1.66703E-18	6.73495E-18
ENSGALG00000016271	PTP4A1	3	1.67258E-18	6.75512E-18
ENSGALG00000042511	PKDCC	3	1.6852E-18	6.80386E-18
ENSGALG00000009795	MON2	1	1.68853E-18	6.81503E-18
ENSGALG00000053245		8	1.69739E-18	6.84854E-18
ENSGALG00000044141	THAP9	4	1.70504E-18	6.87713E-18
ENSGALG00000047352		3	1.74285E-18	7.02732E-18
ENSGALG00000016673	TRAM2	3	1.84588E-18	7.44028E-18
ENSGALG00000028622		25	1.87495E-18	7.55495E-18
ENSGALG00000006532	FOPNL	14	1.87827E-18	7.56581E-18
ENSGALG00000005024		12	1.8821E-18	7.57875E-18
ENSGALG00000013889	RNMT	2	1.88585E-18	7.59136E-18
ENSGALG00000010744	GALNT7	4	1.90025E-18	7.64679E-18
ENSGALG00000008372	XRN2	3	1.90198E-18	7.65122E-18
ENSGALG00000017323	FAM168A	1	1.92691E-18	7.74896E-18
ENSGALG00000012117	STEAP3	7	1.94823E-18	7.8321E-18

ENSGALG00000032417		20	1.98404E-18	7.97345E-18
ENSGALG00000009147	NCSTN	25	1.99934E-18	8.03226E-18
ENSGALG00000016861	BIVM	1	2.00379E-18	8.04751E-18
ENSGALG00000000390	ENTPD4	22	2.01854E-18	8.10408E-18
ENSGALG00000052964		2	2.02737E-18	8.13684E-18
ENSGALG00000027871	MAD2L1BP	3	2.03831E-18	8.17804E-18
ENSGALG00000011365	WDR20	5	2.04254E-18	8.19234E-18
ENSGALG00000039409	RELA	33	2.04598E-18	8.20343E-18
ENSGALG00000015939	THNSL2	4	2.0686E-18	8.29142E-18
ENSGALG00000007373	PRKAB1	15	2.07142E-18	8.29999E-18
ENSGALG00000016198	U2AF1	1	2.11641E-18	8.47748E-18
ENSGALG00000000703	SLC45A3	26	2.13556E-18	8.55135E-18
ENSGALG00000014201	PARVB	1	2.16112E-18	8.64964E-18
ENSGALG00000040131		15	2.16152E-18	8.64964E-18
ENSGALG00000016708	ENPP4	3	2.20364E-18	8.81527E-18
ENSGALG00000011515	ERCC3	7	2.21993E-18	8.87753E-18
ENSGALG00000007511	ITGB2	7	2.25731E-18	9.02407E-18
ENSGALG00000004192	SLTM	10	2.29128E-18	9.15685E-18
ENSGALG00000006094	PHF6	4	2.29787E-18	9.18017E-18
ENSGALG00000038667	MRPS12	32	2.30669E-18	9.21241E-18
ENSGALG00000016132	IGSF5	1	2.31886E-18	9.25797E-18
ENSGALG00000034065		4	2.31984E-18	9.25888E-18
ENSGALG00000013768	PHACTR2	3	2.33156E-18	9.30258E-18
ENSGALG00000034111	FOXF1	11	2.33344E-18	9.30703E-18
ENSGALG00000003035	FAM78B	8	2.33893E-18	9.3259E-18
ENSGALG00000005150	PTRH2	19	2.3487E-18	9.3618E-18
ENSGALG00000037633	WIPF2	27	2.35184E-18	9.37122E-18
ENSGALG00000040746	CHMP1B	4	2.35819E-18	9.39345E-18
ENSGALG00000020742	PPRC1	6	2.38577E-18	9.50024E-18
ENSGALG00000003658	DHX35	20	2.41644E-18	9.6192E-18
ENSGALG00000013481	IKZF3	27	2.43439E-18	9.68752E-18
ENSGALG00000002699	VAV2	17	2.4396E-18	9.70508E-18
ENSGALG00000002484	CRYM	14	2.51255E-18	9.99201E-18

ENSGALG00000021040	HAGHL	14	2.5289E-18	1.00538E-17
ENSGALG00000032004	CSRNP2	33	2.54072E-18	1.00975E-17
ENSGALG00000050806		6	2.54433E-18	1.01085E-17
ENSGALG00000029476	XPO1	3	2.55524E-18	1.01486E-17
ENSGALG00000003461	USP49	26	2.56267E-18	1.01747E-17
ENSGALG00000006534	PEX11A	10	2.5742E-18	1.02172E-17
ENSGALG00000012709	ATXN1	2	2.63324E-18	1.04481E-17
ENSGALG00000036021	MTMR7	4	2.63972E-18	1.04704E-17
ENSGALG00000003377	SIMC1	13	2.64092E-18	1.04718E-17
ENSGALG00000007430	ARCN1	24	2.64697E-18	1.04924E-17
ENSGALG00000016605	EFR3B	3	2.65237E-18	1.05104E-17
ENSGALG00000005796	XBP1	15	2.65334E-18	1.05108E-17
ENSGALG00000023576	PIM3	1	2.66379E-18	1.05487E-17
ENSGALG00000016917	CLN5	1	2.67212E-18	1.05783E-17
ENSGALG00000004048	PTPRS	28	2.74164E-18	1.085E-17
ENSGALG00000031457	TDG	1	2.75367E-18	1.0894E-17
ENSGALG00000012232	DNAAF2	5	2.79945E-18	1.10716E-17
ENSGALG00000009556	PRICKLE1	1	2.82335E-18	1.11625E-17
ENSGALG00000011180	FAM120B	3	2.84189E-18	1.12321E-17
ENSGALG00000006038	TGFBR3	8	2.85013E-18	1.12611E-17
ENSGALG00000016805	SULT1C3	1	2.8813E-18	1.13805E-17
ENSGALG00000054034	TNKS1BP1	5	2.91766E-18	1.15204E-17
ENSGALG00000023691	ENTPD7	6	2.9451E-18	1.1625E-17
ENSGALG00000013149	MOCOS	2	2.97549E-18	1.17412E-17
ENSGALG00000037030	CREBL2	1	2.99411E-18	1.18108E-17
ENSGALG00000005002	VDAC2	6	3.01481E-18	1.18886E-17
ENSGALG00000007492	YME1L1	2	3.03146E-18	1.19504E-17
ENSGALG00000009322	NVL	3	3.05557E-18	1.20415E-17
ENSGALG00000009018	TMEM87A	5	3.14873E-18	1.24047E-17
ENSGALG00000014085	TM7SF3	1	3.17691E-18	1.25116E-17
ENSGALG00000017049	ALG5	1	3.22461E-18	1.26954E-17
ENSGALG00000000407		28	3.28917E-18	1.29454E-17
ENSGALG00000005646	TSC2	14	3.32586E-18	1.30856E-17

ENSGALG00000012769	CHPT1	1	3.35835E-18	1.32091E-17
ENSGALG00000041857	ZC3H15	7	3.44E-18	1.35259E-17
ENSGALG00000031534	ARID5B	6	3.45905E-18	1.35965E-17
ENSGALG00000016681	ZBED1	1	3.4656E-18	1.36178E-17
ENSGALG00000006968	HNF1A	15	3.47619E-18	1.3655E-17
ENSGALG00000006035	PDE3B	5	3.48673E-18	1.3692E-17
ENSGALG00000008627	TTBK1	3	3.5207E-18	1.3821E-17
ENSGALG00000009426	AIDA	3	3.52564E-18	1.38359E-17
ENSGALG00000009905	MDM1	1	3.55334E-18	1.39401E-17
ENSGALG00000041783	TMEM19	1	3.57767E-18	1.40311E-17
ENSGALG00000008844	SLC31A1	17	3.61404E-18	1.41691E-17
ENSGALG00000012790	DSP	2	3.62258E-18	1.4198E-17
ENSGALG00000013420	FOXM1	1	3.6761E-18	1.44032E-17
ENSGALG00000042446	ACSM5	14	3.71026E-18	1.45324E-17
ENSGALG00000041717	KLF3	4	3.7954E-18	1.4861E-17
ENSGALG00000004963	TIMM8A	4	3.90979E-18	1.5304E-17
ENSGALG00000012778	SLC35B3	2	3.9701E-18	1.55299E-17
ENSGALG00000032499	ANAPC15	1	3.97063E-18	1.55299E-17
ENSGALG00000034811	RPLP2	5	3.97131E-18	1.55299E-17
ENSGALG00000006069	SMTNL2	19	3.99476E-18	1.56166E-17
ENSGALG00000008845	HAO1	3	4.03439E-18	1.57664E-17
ENSGALG00000012311	UBE2D3	4	4.08718E-18	1.59674E-17
ENSGALG00000008734	PCSK2	3	4.08842E-18	1.59674E-17
ENSGALG00000027165	RSFR	6	4.14254E-18	1.61704E-17
ENSGALG00000007041	HDX	4	4.14307E-18	1.61704E-17
ENSGALG00000038627	FBXO38	13	4.21491E-18	1.64456E-17
ENSGALG00000006622	FRA10AC1	6	4.29604E-18	1.67568E-17
ENSGALG00000012121		5	4.3021E-18	1.6775E-17
ENSGALG00000015040	SLC16A10	3	4.34561E-18	1.69393E-17
ENSGALG00000052268	SSBP4	28	4.37113E-18	1.70333E-17
ENSGALG00000043456	SMIM7	28	4.39465E-18	1.71195E-17
ENSGALG00000017133	EEF1AKMT1	1	4.39694E-18	1.71229E-17
ENSGALG00000053848		1	4.41114E-18	1.71727E-17

ENSGALG00000028627	TIMP3	1	4.42464E-18	1.72198E-17
ENSGALG00000002866	KANSL1L	7	4.4283E-18	1.72286E-17
ENSGALG00000047569		2	4.44385E-18	1.72835E-17
ENSGALG00000006520	MYH11	14	4.44917E-18	1.72987E-17
ENSGALG00000017208	AMOTL1	1	4.4829E-18	1.74243E-17
ENSGALG00000012159	ACTR3	7	4.51237E-18	1.75332E-17
ENSGALG00000044213	METTL3	31	4.52189E-18	1.75646E-17
ENSGALG00000045776	CPN2	9	4.55092E-18	1.76718E-17
ENSGALG00000005057	ADK	6	4.59676E-18	1.78441E-17
ENSGALG00000001364	CSNK1A1	13	4.63245E-18	1.79769E-17
ENSGALG00000033997	ZNF800	1	4.64949E-18	1.80373E-17
ENSGALG00000039349	DISC1	3	4.6909E-18	1.81922E-17
ENSGALG00000039563	CNOT11	1	4.69438E-18	1.81999E-17
ENSGALG00000021636	CUX1	19	4.7089E-18	1.82504E-17
ENSGALG00000033137	TLDC1	11	4.80821E-18	1.86293E-17
ENSGALG00000007153	DYL1	15	4.84272E-18	1.87571E-17
ENSGALG00000011091	NUP133	3	4.88152E-18	1.89014E-17
ENSGALG00000004593	SULF2	20	4.9807E-18	1.92793E-17
ENSGALG00000050332		14	5.09348E-18	1.97095E-17
ENSGALG00000038502	CCNF	14	5.0958E-18	1.97123E-17
ENSGALG00000007054	TBC1D16	18	5.20635E-18	2.01336E-17
ENSGALG00000026834	PLPP2	28	5.25838E-18	2.03283E-17
ENSGALG00000038589	ABHD14B	12	5.26602E-18	2.03514E-17
ENSGALG00000001774	NEO1	10	5.28977E-18	2.04367E-17
ENSGALG00000011153	LY75	7	5.31909E-18	2.05435E-17
ENSGALG00000046409		5	5.42536E-18	2.09473E-17
ENSGALG00000009403	GRK5	6	5.43034E-18	2.09598E-17
ENSGALG00000005430	ALS2CL	2	5.44496E-18	2.10096E-17
ENSGALG00000011008	MAP3K21	3	5.4822E-18	2.11466E-17
ENSGALG00000015480	POGLUT1	1	5.58428E-18	2.15336E-17
ENSGALG00000003074		28	5.77268E-18	2.2253E-17
ENSGALG00000005036	SH2D3C	17	5.8089E-18	2.23856E-17
ENSGALG00000010702	ADSS	3	5.89892E-18	2.27253E-17

ENSGALG00000017202	FAM76B	1	5.91825E-18	2.27926E-17
ENSGALG00000034586		2	5.92405E-18	2.28077E-17
ENSGALG00000029012		3	5.95832E-18	2.29324E-17
ENSGALG00000007203	RAB3IL1	5	6.10898E-18	2.35048E-17
ENSGALG00000000903	TERF2IP	11	6.15267E-18	2.36655E-17
ENSGALG00000043572	FAM89A	3	6.16098E-18	2.36884E-17
ENSGALG00000034328	MCOLN1	30	6.16252E-18	2.36884E-17
ENSGALG00000028849	SCAF11	1	6.19242E-18	2.37958E-17
ENSGALG00000033608	RAB12	2	6.32716E-18	2.4306E-17
ENSGALG00000008084	NDUFS3	5	6.33289E-18	2.43203E-17
ENSGALG00000031167		2	6.38825E-18	2.45251E-17
ENSGALG00000014860	YES1	2	6.44984E-18	2.47488E-17
ENSGALG00000012796		1	6.45055E-18	2.47488E-17
ENSGALG00000036459	THAP7	31	6.46041E-18	2.47788E-17
ENSGALG00000009402	LRRC34	9	6.58424E-18	2.52458E-17
ENSGALG00000029746	NT5DC3	1	6.59451E-18	2.52772E-17
ENSGALG00000029793	CNEP1R1	11	6.62182E-18	2.53739E-17
ENSGALG00000004005	AKR7L	21	6.66992E-18	2.55502E-17
ENSGALG00000045671		14	6.76006E-18	2.58874E-17
ENSGALG00000004205	SOAT1	8	6.88971E-18	2.63756E-17
ENSGALG00000041494	GOLGB1	1	6.91541E-18	2.64657E-17
ENSGALG00000005522	DYNLL2	19	6.9571E-18	2.66169E-17
ENSGALG00000033677	AHCY	20	7.07276E-18	2.70509E-17
ENSGALG00000046776		1	7.10166E-18	2.71529E-17
ENSGALG00000030419	GLS2	7	7.12092E-18	2.7218E-17
ENSGALG00000054048		19	7.15457E-18	2.73381E-17
ENSGALG00000037138	KCNQ1	5	7.30863E-18	2.7918E-17
ENSGALG00000035482	SEC24B	4	7.31773E-18	2.7944E-17
ENSGALG00000047908	ZFAND3	3	7.3258E-18	2.7966E-17
ENSGALG00000002506	SPSB1	21	7.34129E-18	2.80164E-17
ENSGALG00000008436	CRY2	5	7.38145E-18	2.81608E-17
ENSGALG00000007139	FAM107A	12	7.4297E-18	2.8336E-17
ENSGALG00000005737	TNFAIP1	19	7.48546E-18	2.85398E-17

ENSGALG00000001175	C4BPA	26	7.49356E-18	2.85617E-17
ENSGALG00000039284	RCN2	10	7.52272E-18	2.86639E-17
ENSGALG00000034973	FZD7	7	7.6545E-18	2.91569E-17
ENSGALG00000006487	TM9SF2L	4	7.73366E-18	2.94493E-17
ENSGALG00000003304	CRTC1	28	7.83551E-18	2.98278E-17
ENSGALG00000004266	TNFAIP8L1	28	7.94958E-18	3.02526E-17
ENSGALG00000005363	CHDH	12	8.1081E-18	3.08462E-17
ENSGALG00000013810	VTA1	3	8.1306E-18	3.09222E-17
ENSGALG00000005206	ACSL3	9	8.14812E-18	3.09791E-17
ENSGALG00000041630	NFIX	30	8.21014E-18	3.12052E-17
ENSGALG00000042611		1	8.26081E-18	3.1388E-17
ENSGALG00000006413	CEMIP	10	8.313E-18	3.15764E-17
ENSGALG00000003206	METTL13	8	8.32764E-18	3.16222E-17
ENSGALG00000003732	UCK1	17	8.33199E-18	3.16289E-17
ENSGALG00000004738	GLE1	17	8.41494E-18	3.19338E-17
ENSGALG00000010253	ZDHHC17	1	8.58941E-18	3.25858E-17
ENSGALG00000005096	B9D1	14	8.63529E-18	3.27496E-17
ENSGALG00000011856	FMC1	1	8.70771E-18	3.3014E-17
ENSGALG00000037618	ASAP1	2	8.79751E-18	3.33441E-17
ENSGALG00000007382	CDA	21	8.91729E-18	3.37876E-17
ENSGALG00000054059		3	9.01932E-18	3.41636E-17
ENSGALG00000028543	FGFR4	13	9.02807E-18	3.41861E-17
ENSGALG00000038400	TOMM40L	25	9.12908E-18	3.45579E-17
ENSGALG00000007131		4	9.21054E-18	3.48554E-17
ENSGALG00000008408	GAB3	4	9.3177E-18	3.525E-17
ENSGALG00000027363	C1orf159	21	9.50171E-18	3.5935E-17
ENSGALG00000036969	KLHL14	2	9.57454E-18	3.61992E-17
ENSGALG00000010718		2	9.7618E-18	3.68957E-17
ENSGALG00000042471	IL2RA	1	9.7963E-18	3.70147E-17
ENSGALG00000003916	SIAH1	11	9.94433E-18	3.75623E-17
ENSGALG00000030276	SHTN1	6	1.00144E-17	3.78153E-17
ENSGALG00000042839	FAM214A	10	1.00736E-17	3.8027E-17
ENSGALG00000011162	CYP46A1	5	1.01377E-17	3.82573E-17

ENSGALG00000014023	H2AFZ	4	1.01556E-17	3.8313E-17
ENSGALG00000007248		10	1.02096E-17	3.85047E-17
ENSGALG00000003029	PLPP6	8	1.03742E-17	3.91133E-17
ENSGALG00000001146	UBXN6	28	1.044E-17	3.93492E-17
ENSGALG00000010013	EDNRA	4	1.05151E-17	3.96202E-17
ENSGALG00000026906	TMEM189	20	1.05789E-17	3.98483E-17
ENSGALG00000008074	AMMECR1	4	1.065E-17	4.00967E-17
ENSGALG00000035378	DNAJC5	20	1.06515E-17	4.00967E-17
ENSGALG00000022440	EVA1B	23	1.07116E-17	4.03108E-17
ENSGALG00000016849	ING1	1	1.07518E-17	4.04496E-17
ENSGALG00000020331		4	1.07754E-17	4.05259E-17
ENSGALG00000011931	SNU13	1	1.08507E-17	4.07965E-17
ENSGALG00000031263	TBL2	19	1.11144E-17	4.17749E-17
ENSGALG00000014324	SLC2A14	1	1.11353E-17	4.18405E-17
ENSGALG00000013689	TMEM242	3	1.14302E-17	4.29354E-17
ENSGALG00000038286	AP4E1	10	1.14464E-17	4.29829E-17
ENSGALG00000003487	RAB8B	10	1.14675E-17	4.30489E-17
ENSGALG00000027635	NME3	14	1.15516E-17	4.33514E-17
ENSGALG00000008373	STRADB	7	1.16839E-17	4.38343E-17
ENSGALG00000026154		12	1.19343E-17	4.47599E-17
ENSGALG00000017248	RAB30	1	1.19847E-17	4.49297E-17
ENSGALG00000012814	PXDC1	2	1.19869E-17	4.49297E-17
ENSGALG00000039177		4	1.20534E-17	4.5165E-17
ENSGALG00000009364	PRKCI	9	1.21347E-17	4.54558E-17
ENSGALG00000011084	CCSAP	3	1.22914E-17	4.60285E-17
ENSGALG00000004531	PLOD1	21	1.25222E-17	4.68785E-17
ENSGALG00000007677	THNSL1	2	1.26592E-17	4.73768E-17
ENSGALG00000051401	RASIP1	22	1.27865E-17	4.78386E-17
ENSGALG00000005809	SEPTIN2	9	1.29062E-17	4.82715E-17
ENSGALG00000007174	TNFSF15	17	1.30117E-17	4.86513E-17
ENSGALG00000004200	DPP9	28	1.31249E-17	4.90596E-17
ENSGALG00000020057	PIGF	3	1.31579E-17	4.91676E-17
ENSGALG00000013160	CHMP5	2	1.31945E-17	4.92895E-17

ENSGALG00000042321	LYN	2	1.34128E-17	5.00894E-17
ENSGALG00000012543	GPD2	7	1.34459E-17	5.01978E-17
ENSGALG00000002285	SPSB3	14	1.3474E-17	5.02872E-17
ENSGALG00000005169	FUT11	6	1.34913E-17	5.03365E-17
ENSGALG00000037629	TRANK1	2	1.35913E-17	5.06939E-17
ENSGALG00000010681	CPT2	8	1.41344E-17	5.27036E-17
ENSGALG00000015626		4	1.4586E-17	5.43709E-17
ENSGALG00000023451	TRPM7	10	1.48222E-17	5.52343E-17
ENSGALG00000004111	GPR174	4	1.51377E-17	5.63927E-17
ENSGALG00000004534	DCTN4	13	1.5242E-17	5.67642E-17
ENSGALG00000002062	SRP68	18	1.53134E-17	5.70126E-17
ENSGALG00000015303	AFG1L	3	1.53811E-17	5.72473E-17
ENSGALG00000008818	IDH1	7	1.55345E-17	5.78005E-17
ENSGALG00000039535	D2HGDH	9	1.55821E-17	5.79599E-17
ENSGALG00000016559	PIGA	1	1.56397E-17	5.81566E-17
ENSGALG00000030511	SLC19A1	7	1.5844E-17	5.88982E-17
ENSGALG00000025743	CDR2	14	1.58662E-17	5.89626E-17
ENSGALG00000029599	ACVR1B	33	1.60244E-17	5.95324E-17
ENSGALG00000005768	ETV5	9	1.62918E-17	6.05073E-17
ENSGALG00000053875		4	1.63037E-17	6.05332E-17
ENSGALG00000007140	NRP1	2	1.67831E-17	6.22941E-17
ENSGALG00000005905	ZDHHC4	14	1.6882E-17	6.26422E-17
ENSGALG00000028230	SUN2	1	1.70212E-17	6.31395E-17
ENSGALG00000008312	GSAP	1	1.70621E-17	6.3272E-17
ENSGALG00000010793	SLC24A4	5	1.71359E-17	6.35264E-17
ENSGALG00000041422		3	1.74778E-17	6.47743E-17
ENSGALG00000013715	DLC1	4	1.75758E-17	6.51176E-17
ENSGALG00000042948	RNF126	28	1.77924E-17	6.59001E-17
ENSGALG00000005147	AMY1A	8	1.7817E-17	6.59712E-17
ENSGALG00000036754	CHKA	5	1.78331E-17	6.60106E-17
ENSGALG00000001697		12	1.79051E-17	6.62569E-17
ENSGALG00000045199		5	1.7987E-17	6.65397E-17
ENSGALG00000002151	PEX12	19	1.84584E-17	6.82631E-17

ENSGALG00000021262	GHITM	6	1.86818E-17	6.90683E-17
ENSGALG00000050315	CLC2DL3	1	1.87355E-17	6.9246E-17
ENSGALG00000054978		25	1.89773E-17	7.01183E-17
ENSGALG00000023070	SPIA1	5	1.91074E-17	7.05775E-17
ENSGALG00000052396		5	1.93785E-17	7.15573E-17
ENSGALG00000006526	PLXNB3	12	2.00505E-17	7.40164E-17
ENSGALG00000049068	ZNF831	20	2.04024E-17	7.52927E-17
ENSGALG00000004621	PREX1	20	2.04156E-17	7.53184E-17
ENSGALG00000029709	INPP4A	1	2.04414E-17	7.53906E-17
ENSGALG00000014647	RIT1	25	2.07132E-17	7.63701E-17
ENSGALG00000040770		14	2.08352E-17	7.67968E-17
ENSGALG00000000615	NWD1	23	2.09569E-17	7.72219E-17
ENSGALG00000031835	TMEM68	2	2.12153E-17	7.81505E-17
ENSGALG00000052286	MGAT1	1	2.12403E-17	7.82189E-17
ENSGALG00000017079	USPL1	1	2.12497E-17	7.82299E-17
ENSGALG00000023189	UMAD1	2	2.1613E-17	7.95434E-17
ENSGALG00000043164	TNKS	4	2.1844E-17	8.03692E-17
ENSGALG00000009598	EPRS	3	2.2419E-17	8.24599E-17
ENSGALG00000016705	SUPT3H	3	2.24884E-17	8.26684E-17
ENSGALG00000032199	FGR	23	2.24892E-17	8.26684E-17
ENSGALG00000015388	PRDM1	3	2.31289E-17	8.49942E-17
ENSGALG00000016022	RUNX1	1	2.33041E-17	8.56121E-17
ENSGALG00000008811	SLC1A4	3	2.3649E-17	8.68529E-17
ENSGALG00000003860	MID2	4	2.3852E-17	8.75723E-17
ENSGALG00000000201	PLEKHM1	27	2.415E-17	8.86396E-17
ENSGALG00000005598	ZNF598	14	2.44564E-17	8.97371E-17
ENSGALG00000041418	NDUFS2	25	2.45512E-17	9.00579E-17
ENSGALG00000008750	DPH7	17	2.47117E-17	9.06194E-17
ENSGALG00000051954	RBM43	7	2.47954E-17	9.0899E-17
ENSGALG00000001022	CCDC43	27	2.52046E-17	9.23714E-17
ENSGALG00000046983		33	2.52201E-17	9.24003E-17
ENSGALG00000009495	FGFR2	6	2.55075E-17	9.34252E-17
ENSGALG00000054002	G6PC3	27	2.56666E-17	9.39799E-17

ENSGALG00000000731	THEMIS2	23	2.61908E-17	9.58704E-17
ENSGALG00000008193	RELN	1	2.62329E-17	9.59956E-17
ENSGALG00000012760	WASHC3	1	2.65804E-17	9.72383E-17
ENSGALG00000010427	TM4SF4	9	2.68293E-17	9.81054E-17
ENSGALG00000009540	YAF2	1	2.68336E-17	9.81054E-17
ENSGALG00000031009	ARHGEF7	1	2.70199E-17	9.87572E-17
ENSGALG00000007448	SPRY3	4	2.73175E-17	9.98148E-17
ENSGALG00000030672	TRIP4	10	2.78241E-17	1.01636E-16
ENSGALG00000009239	TLR2B	4	2.7884E-17	1.01824E-16
ENSGALG00000016906	SPRY2	1	2.81644E-17	1.02817E-16
ENSGALG00000008240	CFLAR	7	2.86958E-17	1.04726E-16
ENSGALG00000007346	JCAD	2	2.87067E-17	1.04734E-16
ENSGALG00000005417	JMJD4	2	2.8864E-17	1.05276E-16
ENSGALG00000005571	HCCS	8	2.89026E-17	1.05338E-16
ENSGALG00000012946	FAM60A	1	2.89039E-17	1.05338E-16
ENSGALG00000036990	DECR1	2	2.89069E-17	1.05338E-16
ENSGALG00000028304	MMR1L4	2	2.91913E-17	1.06343E-16
ENSGALG00000011175	TMEM150C	4	2.9627E-17	1.07898E-16
ENSGALG00000002450	WDR24	14	3.07524E-17	1.11963E-16
ENSGALG00000047879		5	3.08401E-17	1.12249E-16
ENSGALG00000033635	PTGS2	8	3.12428E-17	1.13681E-16
ENSGALG00000038385	MTFR1	2	3.13176E-17	1.13911E-16
ENSGALG00000006170	LRRC8C	8	3.13249E-17	1.13911E-16
ENSGALG00000016922	COMMD6	1	3.13977E-17	1.14142E-16
ENSGALG00000038721	MAP1LC3A	20	3.14552E-17	1.14317E-16
ENSGALG00000011069	FIGN	7	3.17834E-17	1.15475E-16
ENSGALG00000013603	MYCT1	3	3.21589E-17	1.16778E-16
ENSGALG00000008950	EHD4	5	3.2161E-17	1.16778E-16
ENSGALG00000007141	LRRK1	10	3.23027E-17	1.17257E-16
ENSGALG00000053766		22	3.3223E-17	1.20562E-16
ENSGALG00000011872		1	3.36468E-17	1.22064E-16
ENSGALG00000005369	BTBD10	5	3.48989E-17	1.26568E-16
ENSGALG00000004224	MAD1L1	14	3.49776E-17	1.26816E-16

ENSGALG00000042371	ANO10	2	3.52376E-17	1.2772E-16
ENSGALG00000047827	TMEM86A	5	3.52868E-17	1.27861E-16
ENSGALG00000027768	CTDSP1	7	3.53864E-17	1.28184E-16
ENSGALG00000004629	RNF216	14	3.54464E-17	1.28363E-16
ENSGALG00000016227	GPR34	1	3.58038E-17	1.29619E-16
ENSGALG00000037241	KCNAB2	21	3.5873E-17	1.29831E-16
ENSGALG00000042739	TACC1	22	3.61075E-17	1.30641E-16
ENSGALG00000037377	TULP4	3	3.61888E-17	1.30896E-16
ENSGALG00000034163	CRTAP	2	3.64279E-17	1.31721E-16
ENSGALG00000038515	LFNG	14	3.71867E-17	1.34425E-16
ENSGALG00000008544	SLC8A1	3	3.77885E-17	1.3656E-16
ENSGALG00000007810	PPP4R2	12	3.82939E-17	1.38346E-16
ENSGALG00000015474	CD80	1	3.88284E-17	1.40236E-16
ENSGALG00000041626	MEIS1	3	3.93646E-17	1.4213E-16
ENSGALG00000012885	BCL2	2	3.94066E-17	1.4224E-16
ENSGALG00000011908	WBP2NL	1	3.99647E-17	1.44211E-16
ENSGALG00000024332	CDC34	28	4.01556E-17	1.44857E-16
ENSGALG00000026009	SRP9	3	4.0416E-17	1.45754E-16
ENSGALG00000030351	ILF3	30	4.08791E-17	1.4738E-16
ENSGALG00000029857		2	4.17605E-17	1.50513E-16
ENSGALG00000001283	SRRM1	23	4.20045E-17	1.51348E-16
ENSGALG00000043372	RUNX3	23	4.23431E-17	1.52523E-16
ENSGALG00000039394	PGM2	4	4.265E-17	1.53583E-16
ENSGALG00000013907	KDR	4	4.27265E-17	1.53813E-16
ENSGALG00000053991	SERF2	10	4.28651E-17	1.54266E-16
ENSGALG00000013785	GNAL	2	4.28817E-17	1.5428E-16
ENSGALG00000003876	TIMD4	13	4.31054E-17	1.55039E-16
ENSGALG00000003091	PARN	14	4.32269E-17	1.55431E-16
ENSGALG00000030120	ITGB1BP3	10	4.36468E-17	1.56894E-16
ENSGALG00000040367	P2RY14	9	4.53532E-17	1.6298E-16
ENSGALG00000028373	GAS2L3	1	4.59419E-17	1.65047E-16
ENSGALG00000011998	SEC24D	4	4.61089E-17	1.65598E-16
ENSGALG00000002057	POLR2D	9	4.71398E-17	1.69251E-16

ENSGALG00000009496	NAF1	4	4.73903E-17	1.701E-16
ENSGALG00000016105		4	4.78412E-17	1.71668E-16
ENSGALG00000023135	SRP54	5	4.7858E-17	1.71678E-16
ENSGALG00000005893	ZFAND4	6	4.79147E-17	1.71782E-16
ENSGALG00000035749		10	4.79151E-17	1.71782E-16
ENSGALG00000007162	SELENOS	10	4.80327E-17	1.72153E-16
ENSGALG00000006901	AATK	18	4.85792E-17	1.7406E-16
ENSGALG00000004244	TTPAL	20	4.8766E-17	1.74678E-16
ENSGALG00000005802	FLT4	13	4.90795E-17	1.75749E-16
ENSGALG00000017011	TRIM13	1	4.92402E-17	1.76273E-16
ENSGALG00000003321	RILPL2	15	4.93391E-17	1.76575E-16
ENSGALG00000031928	SPTLC2	5	4.94801E-17	1.77028E-16
ENSGALG00000053304		14	4.96876E-17	1.77718E-16
ENSGALG00000025996		6	5.00286E-17	1.78885E-16
ENSGALG00000041635	CAMK2D	4	5.06013E-17	1.8088E-16
ENSGALG00000030185	PTDSS1	2	5.08161E-17	1.81595E-16
ENSGALG00000006421	SLC16A12	6	5.22388E-17	1.86624E-16
ENSGALG00000049976	AMIGO1	26	5.29324E-17	1.89047E-16
ENSGALG00000010200	TRHDE	1	5.42538E-17	1.93709E-16
ENSGALG00000033106	TMTC3	1	5.45722E-17	1.94789E-16
ENSGALG00000033025	RYBP	12	5.62987E-17	2.00893E-16
ENSGALG00000023554	PIGW	19	5.68239E-17	2.02707E-16
ENSGALG00000010189		5	5.68859E-17	2.02869E-16
ENSGALG00000007646		4	5.7248E-17	2.04087E-16
ENSGALG00000012170	LIN7C	5	5.72608E-17	2.04087E-16
ENSGALG00000052338	SELENOW	32	5.75143E-17	2.04931E-16
ENSGALG00000047875		1	5.75495E-17	2.04996E-16
ENSGALG00000042023		1	5.86205E-17	2.0875E-16
ENSGALG00000010068	KCNK17	3	5.89124E-17	2.09728E-16
ENSGALG00000031729	MOB3A	28	5.93231E-17	2.11129E-16
ENSGALG00000042858	FAM8A1	2	6.02655E-17	2.1442E-16
ENSGALG00000008623	TOR4A	17	6.02857E-17	2.14429E-16
ENSGALG00000014892	CEP85L	3	6.13499E-17	2.18151E-16

ENSGALG00000003067	MRPL44	9	6.26942E-17	2.22866E-16
ENSGALG00000003919	MRPL22	13	6.311E-17	2.24217E-16
ENSGALG00000027240	TSPAN13	2	6.3111E-17	2.24217E-16
ENSGALG00000026971	RRAGD	3	6.35102E-17	2.25569E-16
ENSGALG00000031693	KCNB2	2	6.36962E-17	2.26164E-16
ENSGALG00000049446	SLC29A3	6	6.37178E-17	2.26175E-16
ENSGALG00000012207		7	6.45189E-17	2.28952E-16
ENSGALG00000002282	TMEM200A	3	6.55852E-17	2.32668E-16
ENSGALG00000039455	VAPA	2	6.59066E-17	2.3374E-16
ENSGALG00000052333		21	6.63304E-17	2.35175E-16
ENSGALG00000037686	NBN	2	6.65903E-17	2.36028E-16
ENSGALG00000030871	BCL2L10	10	6.67011E-17	2.36352E-16
ENSGALG00000033278	NLRC3	14	6.68889E-17	2.36949E-16
ENSGALG00000000511	KCTD20	26	6.69124E-17	2.36963E-16
ENSGALG00000004818	RPL6	15	6.73995E-17	2.38619E-16
ENSGALG00000007439	PRELID3B	20	6.7501E-17	2.38909E-16
ENSGALG00000040737		6	6.88737E-17	2.43696E-16
ENSGALG00000038639	AGBL5	3	6.96693E-17	2.46408E-16
ENSGALG00000042079	NID1	3	6.96805E-17	2.46408E-16
ENSGALG00000005651	KLHL36	11	6.97855E-17	2.46708E-16
ENSGALG00000009731	ARHGAP11B	5	7.00586E-17	2.47601E-16
ENSGALG00000009315	PAPLN	5	7.02416E-17	2.48176E-16
ENSGALG00000034742	LIMA1	33	7.0365E-17	2.4854E-16
ENSGALG00000042357	ARHGAP1	5	7.06947E-17	2.49632E-16
ENSGALG00000011452	ARPC2	7	7.10511E-17	2.50818E-16
ENSGALG00000028471	CNPY3	3	7.14866E-17	2.52283E-16
ENSGALG00000002056	TANGO2	15	7.22592E-17	2.54935E-16
ENSGALG00000034014	C2CD2L	24	7.30938E-17	2.57805E-16
ENSGALG00000012956	TMTC1	1	7.47628E-17	2.63616E-16
ENSGALG00000013661	MICU3	4	7.56398E-17	2.66631E-16
ENSGALG00000016824	TMCO3	1	7.62258E-17	2.68619E-16
ENSGALG00000016897	GPR180	1	7.6795E-17	2.70546E-16
ENSGALG00000043421		1	7.72072E-17	2.7192E-16

ENSGALG00000016266	PRRG1	1	7.80607E-17	2.74847E-16
ENSGALG00000035017		21	7.822E-17	2.75328E-16
ENSGALG00000004948	GLA	4	7.83777E-17	2.75803E-16
ENSGALG00000011436	TMBIM1	7	7.86214E-17	2.76581E-16
ENSGALG00000003485	CCND3	26	7.86725E-17	2.76681E-16
ENSGALG00000001331	ST14	24	8.09454E-17	2.84592E-16
ENSGALG00000004821	PTPN11	15	8.19626E-17	2.88086E-16
ENSGALG00000041573	BPNT2	2	8.20242E-17	2.88219E-16
ENSGALG00000044799	VSIG4	4	8.31146E-17	2.91967E-16
ENSGALG00000043535	RIPK2	2	8.57903E-17	3.01279E-16
ENSGALG00000016883	DOCK9	1	8.61181E-17	3.02343E-16
ENSGALG00000035993	SUOX	33	8.64894E-17	3.03559E-16
ENSGALG00000034772		31	8.75078E-17	3.07045E-16
ENSGALG00000004719	SPTAN1	17	8.76702E-17	3.07449E-16
ENSGALG00000003717	MYO9B	28	8.76734E-17	3.07449E-16
ENSGALG00000009974	MMAA	4	8.78062E-17	3.07827E-16
ENSGALG00000026428	CHMP6	18	8.85579E-17	3.10373E-16
ENSGALG00000006833	ITIH2	1	8.93178E-17	3.12946E-16
ENSGALG00000030005	IGSF1	20	8.98381E-17	3.14679E-16
ENSGALG00000000806	LINGO3	28	8.98659E-17	3.14685E-16
ENSGALG00000003408	RP1-27O5.3	23	9.05088E-17	3.16846E-16
ENSGALG00000017118	MIPEP	1	9.06914E-17	3.17394E-16
ENSGALG00000035537	SYNJ2BP	5	9.11932E-17	3.19059E-16
ENSGALG00000032028	SERTM1	1	9.1326E-17	3.19432E-16
ENSGALG00000011529	NAAA	4	9.14731E-17	3.19854E-16
ENSGALG00000004108		6	9.15972E-17	3.20196E-16
ENSGALG00000015898	MYO6	3	9.45557E-17	3.30444E-16
ENSGALG00000017272	VWF	1	9.61929E-17	3.36069E-16
ENSGALG00000007149	CYTH3	14	9.70029E-17	3.38802E-16
ENSGALG00000026303	MRPS2	17	9.73876E-17	3.40048E-16
ENSGALG00000047813		4	9.89149E-17	3.45282E-16
ENSGALG00000039878	TFEB	26	9.92976E-17	3.46518E-16
ENSGALG00000000905	PSMB4	25	1.01095E-16	3.52691E-16

ENSGALG00000010053	PTPRF	8	1.01982E-16	3.55682E-16
ENSGALG00000001621	CLDN5	15	1.02957E-16	3.5898E-16
ENSGALG00000027698	COX19	14	1.03267E-16	3.59958E-16
ENSGALG00000006002	WHAMM	10	1.0446E-16	3.64014E-16
ENSGALG00000028807	AP1AR	4	1.07328E-16	3.73902E-16
ENSGALG00000008120	TLE3	10	1.08098E-16	3.76475E-16
ENSGALG00000002763	FRZB	7	1.08551E-16	3.77944E-16
ENSGALG00000008285	NAPEPLD	1	1.09394E-16	3.8077E-16
ENSGALG00000001334	GNB1	21	1.09571E-16	3.81278E-16
ENSGALG00000006080	GPC4	4	1.10845E-16	3.85601E-16
ENSGALG00000043010	NR2F6	28	1.10977E-16	3.85951E-16
ENSGALG00000010924	NFIA	8	1.12572E-16	3.91387E-16
ENSGALG00000001462	ADORA3	26	1.14549E-16	3.98148E-16
ENSGALG00000014222	NUP50	1	1.15377E-16	4.00909E-16
ENSGALG00000005779	FBXO31	11	1.15786E-16	4.02216E-16
ENSGALG00000030076	PCSK6	10	1.16749E-16	4.05447E-16
ENSGALG00000014873	GJA1	3	1.17878E-16	4.09249E-16
ENSGALG00000013091	EPS8	1	1.19256E-16	4.13917E-16
ENSGALG00000022887	KBTBD11	3	1.19857E-16	4.15884E-16
ENSGALG00000005125	LSM14B	20	1.19891E-16	4.15884E-16
ENSGALG00000004618	TAX1BP3	19	1.20451E-16	4.17706E-16
ENSGALG00000015407	PLAG1	2	1.21356E-16	4.20725E-16
ENSGALG00000007564		2	1.21554E-16	4.21292E-16
ENSGALG00000004215	WIPI1	18	1.21929E-16	4.22473E-16
ENSGALG00000016187	WDR4	1	1.22444E-16	4.24135E-16
ENSGALG00000008951	MIGA1	8	1.24151E-16	4.29927E-16
ENSGALG00000048042		8	1.24296E-16	4.30305E-16
ENSGALG00000040342	ADAMTS1	1	1.25068E-16	4.32856E-16
ENSGALG00000037043	PIK3C2B	26	1.25577E-16	4.34494E-16
ENSGALG00000054319	ELOVL6	4	1.26345E-16	4.37028E-16
ENSGALG00000008382	GNAI1	1	1.28309E-16	4.43696E-16
ENSGALG00000039685	GMEB2	20	1.29561E-16	4.47899E-16
ENSGALG00000012124	PELI2	5	1.30353E-16	4.50507E-16

ENSGALG00000008475	ARRDC1	17	1.30751E-16	4.51758E-16
ENSGALG00000014831	TRMT11	3	1.31457E-16	4.54066E-16
ENSGALG00000009336	FAM45A	6	1.31605E-16	4.5445E-16
ENSGALG00000002170		26	1.33037E-16	4.59265E-16
ENSGALG00000041036	FTO	11	1.35718E-16	4.68385E-16
ENSGALG00000027126	BLCAP	20	1.36482E-16	4.70888E-16
ENSGALG00000036785	TRIM8	6	1.36639E-16	4.71298E-16
ENSGALG00000008459	BMPR2	7	1.37045E-16	4.72564E-16
ENSGALG00000052250	HJV	25	1.37945E-16	4.75535E-16
ENSGALG00000009485	IMMP2L	1	1.38521E-16	4.77386E-16
ENSGALG00000015416	SDR16C5	2	1.38763E-16	4.78084E-16
ENSGALG00000003081	SUCO	8	1.39565E-16	4.80711E-16
ENSGALG00000034722	TLR2A	4	1.41337E-16	4.86678E-16
ENSGALG00000010139	SSTR1	5	1.41685E-16	4.87736E-16
ENSGALG00000041808	ANO1	5	1.42214E-16	4.8942E-16
ENSGALG00000003681	TOP1	20	1.4247E-16	4.90163E-16
ENSGALG00000015570	GPR63	3	1.44242E-16	4.9612E-16
ENSGALG00000043724	NFX1	2	1.44459E-16	4.96725E-16
ENSGALG00000038532		21	1.45434E-16	4.99936E-16
ENSGALG00000007403	PEBP1	15	1.46351E-16	5.02947E-16
ENSGALG00000031570	WDR54	4	1.47396E-16	5.06397E-16
ENSGALG00000007489	CNNM1	6	1.48525E-16	5.10132E-16
ENSGALG00000007522		18	1.50578E-16	5.17038E-16
ENSGALG00000041914	BRI3	14	1.50703E-16	5.1732E-16
ENSGALG00000037816		4	1.56834E-16	5.38214E-16
ENSGALG00000033012	SPRY4	13	1.58216E-16	5.42805E-16
ENSGALG00000036240	AKAP12	3	1.58944E-16	5.45148E-16
ENSGALG00000037549	EML3	33	1.61057E-16	5.52239E-16
ENSGALG00000030162	IQGAP1	10	1.61978E-16	5.55242E-16
ENSGALG00000016564	PTK2B	3	1.62266E-16	5.56011E-16
ENSGALG00000000913	CHST6	11	1.62293E-16	5.56011E-16
ENSGALG00000042077	HIC1	19	1.64571E-16	5.63656E-16
ENSGALG00000005108	CUL3	9	1.66785E-16	5.71079E-16

ENSGALG00000047269	TMEM135	1	1.66943E-16	5.71459E-16
ENSGALG00000001161	FLI1	24	1.68241E-16	5.75742E-16
ENSGALG00000036965	PTPA	17	1.69735E-16	5.80691E-16
ENSGALG00000036095	BICC1	6	1.72321E-16	5.8937E-16
ENSGALG00000021143	RGS18	8	1.72528E-16	5.89915E-16
ENSGALG00000010290	VEGFA	3	1.73315E-16	5.92438E-16
ENSGALG00000038527	CARMIL3	1	1.73928E-16	5.94367E-16
ENSGALG00000033404	RARG	33	1.74245E-16	5.95283E-16
ENSGALG00000051852		1	1.75067E-16	5.97925E-16
ENSGALG00000014276	CIR1	7	1.75337E-16	5.98679E-16
ENSGALG00000003101	PLPBP	22	1.7546E-16	5.98933E-16
ENSGALG00000017334		1	1.77022E-16	6.04094E-16
ENSGALG00000002709	MYO1C	19	1.77395E-16	6.05197E-16
ENSGALG00000008301	FAM185A	1	1.79113E-16	6.10889E-16
ENSGALG00000006422		15	1.81211E-16	6.17872E-16
ENSGALG00000007476	VAMP7	4	1.83059E-16	6.23998E-16
ENSGALG00000005695	CHEK2	15	1.83318E-16	6.24649E-16
ENSGALG00000010217		8	1.83353E-16	6.24649E-16
ENSGALG00000042895	BCAR1	11	1.84509E-16	6.28412E-16
ENSGALG00000009331	FBXO28	3	1.84839E-16	6.29363E-16
ENSGALG00000003420	ZBTB8A	23	1.85165E-16	6.30294E-16
ENSGALG00000021475	ADAR	25	1.89189E-16	6.43812E-16
ENSGALG00000047163	TSEN54	18	1.89348E-16	6.44175E-16
ENSGALG00000010017	MCFD2	3	1.89461E-16	6.44378E-16
ENSGALG00000007961	ARMC7	18	1.89514E-16	6.44379E-16
ENSGALG00000003301	FAM167B	23	1.91737E-16	6.51758E-16
ENSGALG00000002155	HS6ST1	9	1.92462E-16	6.54037E-16
ENSGALG00000011291	NR1D2	2	1.93776E-16	6.58321E-16
ENSGALG00000040549	HTR7	6	1.94261E-16	6.59784E-16
ENSGALG00000016947	ELF1	1	1.99903E-16	6.78758E-16
ENSGALG00000010761	MRPL37	8	2.01486E-16	6.83942E-16
ENSGALG00000055058		1	2.05398E-16	6.97029E-16
ENSGALG00000025950	PRKCA	18	2.06889E-16	7.0189E-16

ENSGALG00000009119	THUMPD2	3	2.08583E-16	7.07442E-16
ENSGALG00000016622	STS	1	2.09239E-16	7.09468E-16
ENSGALG00000003475	ACLY	27	2.10486E-16	7.135E-16
ENSGALG00000008466		7	2.12133E-16	7.18881E-16
ENSGALG00000012525	FMNL2	7	2.1284E-16	7.21076E-16
ENSGALG00000017366	DPM1	20	2.13835E-16	7.24247E-16
ENSGALG00000001607	CYBC1	18	2.15902E-16	7.31043E-16
ENSGALG00000001722	E2F4	11	2.19682E-16	7.43638E-16
ENSGALG00000006477	DNAJB6	2	2.20135E-16	7.44964E-16
ENSGALG00000030810		3	2.22817E-16	7.5383E-16
ENSGALG00000012442	MFNG	1	2.23628E-16	7.56364E-16
ENSGALG00000004611	FBXL18	14	2.24249E-16	7.58254E-16
ENSGALG00000031762	TMEM98	27	2.24349E-16	7.58382E-16
ENSGALG00000052515		25	2.2447E-16	7.58581E-16
ENSGALG00000008432	AP2M1	9	2.30335E-16	7.78185E-16
ENSGALG00000050734	METTL27	19	2.30875E-16	7.79794E-16
ENSGALG00000008179	NT5C2	6	2.3183E-16	7.82803E-16
ENSGALG00000005992	PDE8A	10	2.33053E-16	7.86714E-16
ENSGALG00000014182	ADORA2B	19	2.33641E-16	7.88482E-16
ENSGALG00000011468	IGFBP5	7	2.33745E-16	7.88614E-16
ENSGALG00000006328	CHCHD4	12	2.39684E-16	8.08426E-16
ENSGALG00000021569		21	2.40522E-16	8.1103E-16
ENSGALG00000007822	MRPL58	18	2.41417E-16	8.13822E-16
ENSGALG00000003082	DPH1	19	2.41622E-16	8.14288E-16
ENSGALG00000010326	FLVCR2	5	2.41921E-16	8.15071E-16
ENSGALG00000035057	SLC35B1	27	2.42532E-16	8.16904E-16
ENSGALG00000002583	PIK3CD	21	2.42758E-16	8.17441E-16
ENSGALG00000029915	RBM34	3	2.44831E-16	8.24192E-16
ENSGALG00000005249	BICD2	12	2.45308E-16	8.25571E-16
ENSGALG00000007845	REL	3	2.4678E-16	8.30296E-16
ENSGALG00000009086	EHD3	3	2.50394E-16	8.42223E-16
ENSGALG00000003959	AP1M1	28	2.56337E-16	8.61972E-16
ENSGALG00000041365	SALL4	20	2.57474E-16	8.65559E-16

ENSGALG00000003906	MPHOSPH10	10	2.57589E-16	8.65707E-16
ENSGALG00000033498		5	2.5811E-16	8.67219E-16
ENSGALG00000052329	PEX11B	25	2.58902E-16	8.69639E-16
ENSGALG00000004449	CYP3A5	14	2.602E-16	8.7376E-16
ENSGALG00000052052		6	2.6097E-16	8.76103E-16
ENSGALG00000015490	FBXL4	3	2.63598E-16	8.84683E-16
ENSGALG00000036492	DAGLA	5	2.67332E-16	8.96968E-16
ENSGALG00000002192	PTPRC	8	2.68193E-16	8.99608E-16
ENSGALG00000047782		32	2.68439E-16	9.00185E-16
ENSGALG00000016912	EDNRB	1	2.69565E-16	9.03713E-16
ENSGALG00000008557	ARL5B	2	2.698E-16	9.04253E-16
ENSGALG00000007808	HID1	18	2.69927E-16	9.04428E-16
ENSGALG00000004336	INPPL1	4	2.7191E-16	9.10824E-16
ENSGALG00000037360	DDX17	1	2.73462E-16	9.1577E-16
ENSGALG00000022720	GJB2	1	2.79521E-16	9.35805E-16
ENSGALG00000041978	K123	1	2.82224E-16	9.44593E-16
ENSGALG00000016988	RUBCNL	1	2.86885E-16	9.59931E-16
ENSGALG00000016926	KLF12	1	2.88169E-16	9.63963E-16
ENSGALG00000048115	OLFM2	30	2.92453E-16	9.78026E-16
ENSGALG00000007581	FYTTD1	9	2.92543E-16	9.78059E-16
ENSGALG00000052856		2	2.93326E-16	9.80407E-16
ENSGALG00000033226	FAM107B	1	2.94272E-16	9.833E-16
ENSGALG00000017126	ZDHHC20	1	2.95481E-16	9.8707E-16
ENSGALG00000010186	CAPN11	3	2.97087E-16	9.92161E-16
ENSGALG00000009932	THADA	3	2.97587E-16	9.93416E-16
ENSGALG00000041433	MSL3	1	2.97626E-16	9.93416E-16
ENSGALG00000034024	RAPGEF5	2	3.01119E-16	1.0048E-15
ENSGALG00000054304		5	3.02318E-16	1.00853E-15
ENSGALG00000014428	TAPBPL	1	3.03431E-16	1.01196E-15
ENSGALG00000000713	ZFHX3	11	3.04111E-16	1.01395E-15
ENSGALG00000011504	TMPO	1	3.05334E-16	1.01775E-15
ENSGALG00000025884	NABP1	7	3.05734E-16	1.01881E-15
ENSGALG00000016328	GSTA2	3	3.11583E-16	1.03802E-15

ENSGALG00000051947	OPA3	32	3.12265E-16	1.04E-15
ENSGALG00000042485		23	3.14851E-16	1.04833E-15
ENSGALG00000007920	PRR5L	5	3.16034E-16	1.05198E-15
ENSGALG00000010565	NRDC	8	3.16968E-16	1.05461E-15
ENSGALG00000008009	BCAP29	1	3.16997E-16	1.05461E-15
ENSGALG00000013911	ZC3HAV1	1	3.18385E-16	1.05894E-15
ENSGALG00000053626		31	3.22192E-16	1.07131E-15
ENSGALG00000005867	MARCH8	6	3.24229E-16	1.07779E-15
ENSGALG00000009907	G2E3	5	3.26628E-16	1.08547E-15
ENSGALG00000032847	HDGF	25	3.31057E-16	1.09988E-15
ENSGALG00000006743	YWHAH	15	3.32623E-16	1.10479E-15
ENSGALG00000034380	C7orf26	14	3.35326E-16	1.11346E-15
ENSGALG00000045596		1	3.36922E-16	1.11846E-15
ENSGALG00000016480	SDC1	3	3.39929E-16	1.12813E-15
ENSGALG00000006083		10	3.41981E-16	1.13463E-15
ENSGALG00000008785	MCCC1	9	3.48703E-16	1.15662E-15
ENSGALG00000026981	NHSL1	3	3.50567E-16	1.16249E-15
ENSGALG00000007404	YIPF5	13	3.5166E-16	1.16579E-15
ENSGALG00000013846	ARFGEF3	3	3.546E-16	1.17522E-15
ENSGALG00000001434	STOM	17	3.57216E-16	1.18312E-15
ENSGALG00000054044		32	3.57249E-16	1.18312E-15
ENSGALG00000042269	METTL25	1	3.57273E-16	1.18312E-15
ENSGALG00000012007	ST13P5	1	3.60084E-16	1.1921E-15
ENSGALG00000038102	TBRG4	2	3.68183E-16	1.21858E-15
ENSGALG00000033813	BTBD6	5	3.7259E-16	1.23283E-15
ENSGALG00000013569	SEC61B	2	3.75438E-16	1.24192E-15
ENSGALG00000016304	LRRC1	3	3.7642E-16	1.24483E-15
ENSGALG00000008537	EPHB3	9	3.7908E-16	1.25329E-15
ENSGALG00000004122	PNRC2	23	3.85474E-16	1.27408E-15
ENSGALG00000015061	CDC40	3	3.85937E-16	1.27527E-15
ENSGALG00000005120	FBLN2	12	3.89682E-16	1.28729E-15
ENSGALG00000049473		18	3.92367E-16	1.29581E-15
ENSGALG00000014262		4	3.94752E-16	1.30333E-15

ENSGALG00000009809		4	4.02328E-16	1.32799E-15
ENSGALG00000054244		12	4.09484E-16	1.35124E-15
ENSGALG00000040817	NEMP2	7	4.10792E-16	1.35519E-15
ENSGALG00000035734	IKZF4	33	4.16013E-16	1.37204E-15
ENSGALG00000041013	TNS1	7	4.421E-16	1.45769E-15
ENSGALG00000034029	FBXO3	5	4.48143E-16	1.47721E-15
ENSGALG00000053708		31	4.48718E-16	1.47871E-15
ENSGALG00000000943	WDTC1	23	4.49036E-16	1.47936E-15
ENSGALG00000016734	SLC9A7	1	4.57277E-16	1.5061E-15
ENSGALG00000041528	LMF2	1	4.60438E-16	1.5161E-15
ENSGALG00000001332	RBL1	20	4.72503E-16	1.55541E-15
ENSGALG00000015923	MTO1	3	4.75556E-16	1.56504E-15
ENSGALG00000004346	P2RY4	4	4.77905E-16	1.57234E-15
ENSGALG00000041285	MAP2K3	14	4.80439E-16	1.58025E-15
ENSGALG00000041296		3	4.89984E-16	1.61121E-15
ENSGALG00000002710	IPMK	6	4.96854E-16	1.63336E-15
ENSGALG00000031094	EDA2R	4	4.97404E-16	1.63473E-15
ENSGALG00000038229	OTUB1	33	5.02858E-16	1.65221E-15
ENSGALG00000010573	GSTCD	4	5.05425E-16	1.6602E-15
ENSGALG00000004196	UGT1A1	7	5.13182E-16	1.68522E-15
ENSGALG00000008469		5	5.19627E-16	1.70593E-15
ENSGALG00000008164	ADAMTS7	10	5.20246E-16	1.7075E-15
ENSGALG00000005256	EPHA4	9	5.22158E-16	1.71331E-15
ENSGALG00000009040	TBC1D8B	4	5.23511E-16	1.71729E-15
ENSGALG00000000275	KCTD9	22	5.23781E-16	1.71771E-15
ENSGALG00000010010	RHOQ	3	5.2434E-16	1.71908E-15
ENSGALG00000002631	LRRC75A	19	5.3061E-16	1.73918E-15
ENSGALG00000040557	TFEC	1	5.30831E-16	1.73943E-15
ENSGALG00000012532	TXN2	1	5.31883E-16	1.74241E-15
ENSGALG00000006791	TTI1	20	5.48748E-16	1.79718E-15
ENSGALG00000007780	EIF4E3	12	5.53324E-16	1.81168E-15
ENSGALG00000031786	BLVRA	2	5.55939E-16	1.81975E-15
ENSGALG00000036043	TBX3	15	5.57587E-16	1.82465E-15

ENSGALG00000034701		33	5.62063E-16	1.83881E-15
ENSGALG00000046828		25	5.80838E-16	1.89936E-15
ENSGALG00000008501	KLF7	7	5.80884E-16	1.89936E-15
ENSGALG00000019894	TMEM14A	3	5.81538E-16	1.90099E-15
ENSGALG00000026776		1	5.8207E-16	1.90222E-15
ENSGALG00000011428	AZI2	2	5.83774E-16	1.90685E-15
ENSGALG00000043797	DBR1	9	5.8393E-16	1.90685E-15
ENSGALG00000042621	HNF4G	2	5.83955E-16	1.90685E-15
ENSGALG00000029220	PCSK7	24	5.87073E-16	1.91651E-15
ENSGALG00000013409		23	5.87493E-16	1.91737E-15
ENSGALG00000009173	GFRA1	6	5.87976E-16	1.91843E-15
ENSGALG00000010502	SPATA6	8	6.05067E-16	1.97367E-15
ENSGALG00000015233	ATP1B1	1	6.07146E-16	1.97992E-15
ENSGALG00000000637	ZBTB48	21	6.12528E-16	1.99694E-15
ENSGALG00000002643		8	6.13787E-16	2.00051E-15
ENSGALG00000002544	PCDH12	13	6.15719E-16	2.00627E-15
ENSGALG00000004397	MLXIP	15	6.20253E-16	2.0205E-15
ENSGALG00000036252	SAP30	4	6.31443E-16	2.0564E-15
ENSGALG00000016479	LAPTM4A	3	6.322E-16	2.05832E-15
ENSGALG00000050797	SOCS7	27	6.3555E-16	2.06868E-15
ENSGALG00000017219	C1H11ORF54	1	6.42463E-16	2.09062E-15
ENSGALG00000004186	NSRP1	19	6.48378E-16	2.1093E-15
ENSGALG00000006284	SH3GLB1	8	6.62249E-16	2.15385E-15
ENSGALG00000006270	SCNN1G	14	6.64789E-16	2.16114E-15
ENSGALG00000003574	LPCAT2	11	6.64845E-16	2.16114E-15
ENSGALG00000015297		3	6.66259E-16	2.16516E-15
ENSGALG00000045611		23	6.94085E-16	2.25499E-15
ENSGALG00000008398	MPP4	7	7.00195E-16	2.27423E-15
ENSGALG00000004969	TIMP4	12	7.04981E-16	2.28917E-15
ENSGALG00000012394		5	7.13847E-16	2.31734E-15
ENSGALG00000001586	NEIL1	10	7.1569E-16	2.32271E-15
ENSGALG00000008293	SH3PXD2A	6	7.1943E-16	2.33422E-15
ENSGALG00000039698	DRD1	13	7.23285E-16	2.34611E-15

ENSGALG00000015549	GRPEL1	4	7.31787E-16	2.37305E-15
ENSGALG00000004322		7	7.43816E-16	2.41087E-15
ENSGALG00000002661	YWHAE	19	7.43846E-16	2.41087E-15
ENSGALG00000003572	ARHGEF18	28	7.5564E-16	2.44845E-15
ENSGALG00000001021	MYOCD	18	7.56669E-16	2.45113E-15
ENSGALG00000054173		4	7.75592E-16	2.51176E-15
ENSGALG00000012092	ESYT3	7	7.77881E-16	2.51851E-15
ENSGALG00000006018		2	7.80347E-16	2.52582E-15
ENSGALG00000016279	RAB23	3	7.9957E-16	2.58736E-15
ENSGALG00000028009		12	8.14109E-16	2.6337E-15
ENSGALG00000009887	DIEXF	3	8.14537E-16	2.63439E-15
ENSGALG00000012550	HMOX1	1	8.25115E-16	2.66789E-15
ENSGALG00000001800	DUS3L	28	8.26059E-16	2.67024E-15
ENSGALG00000005020		12	8.27372E-16	2.67377E-15
ENSGALG00000043744	NDFIP1	13	8.3056E-16	2.68337E-15
ENSGALG00000027688	NDUFB4	1	8.4247E-16	2.72112E-15
ENSGALG00000035317	ID3	23	8.5149E-16	2.74953E-15
ENSGALG00000017120	SACS	1	8.65008E-16	2.79244E-15
ENSGALG00000027231	NRARP	17	8.68142E-16	2.80181E-15
ENSGALG00000003818	CYFIP2	13	8.72196E-16	2.81415E-15
ENSGALG00000047000		4	8.76123E-16	2.82608E-15
ENSGALG00000016654		1	8.90322E-16	2.87112E-15
ENSGALG00000001658	RPL19	27	8.91133E-16	2.87297E-15
ENSGALG00000016191	PKNOX1	1	9.1354E-16	2.94444E-15
ENSGALG00000030324	ELAPOR2	1	9.315E-16	3.00153E-15
ENSGALG00000049884		17	9.32567E-16	3.00417E-15
ENSGALG00000008954	RUNDC3B	2	9.36109E-16	3.01478E-15
ENSGALG00000001985	PIGQ	14	9.36969E-16	3.01676E-15
ENSGALG00000050752		5	9.43561E-16	3.03718E-15
ENSGALG00000038913	LDLRAD3	5	9.53309E-16	3.06775E-15
ENSGALG00000006013	ZNF512B	20	9.78111E-16	3.14673E-15
ENSGALG00000053132		1	9.81208E-16	3.15586E-15
ENSGALG00000011528		5	9.9638E-16	3.20381E-15

ENSGALG00000008997	STEAP4	2	9.99351E-16	3.21252E-15
ENSGALG00000004932	ATG7	12	1.00404E-15	3.22673E-15
ENSGALG00000004110		20	1.01074E-15	3.24664E-15
ENSGALG00000009300	NUMB	5	1.01076E-15	3.24664E-15
ENSGALG00000033529	BRPF1	12	1.01578E-15	3.2619E-15
ENSGALG00000009107	NRXN1	3	1.01793E-15	3.26792E-15
ENSGALG00000002816	STK10	13	1.03076E-15	3.30826E-15
ENSGALG00000013931	CHIC2	4	1.03166E-15	3.31027E-15
ENSGALG00000000864	FBXW2	17	1.04133E-15	3.34043E-15
ENSGALG00000001473	MIB2	21	1.04422E-15	3.34882E-15
ENSGALG00000007409	SCN4B	24	1.04667E-15	3.3558E-15
ENSGALG00000008172	ERLEC1	3	1.05714E-15	3.38846E-15
ENSGALG00000029450	UBE2Q1	25	1.05987E-15	3.39632E-15
ENSGALG00000007155	RMI2	14	1.06036E-15	3.39698E-15
ENSGALG00000008728	PTER	2	1.06244E-15	3.40276E-15
ENSGALG00000009297	TELO2	14	1.06789E-15	3.41932E-15
ENSGALG00000015012	STX18	4	1.06844E-15	3.42018E-15
ENSGALG00000006562	MCF2	4	1.07812E-15	3.45027E-15
ENSGALG00000002246	DENND1B	8	1.10027E-15	3.52023E-15
ENSGALG00000011994	SYNPO2	4	1.10188E-15	3.52444E-15
ENSGALG00000029583	PLBD1	1	1.11427E-15	3.56314E-15
ENSGALG00000005559	MRPS23	19	1.13046E-15	3.61399E-15
ENSGALG00000009422	RGS10	6	1.13208E-15	3.6182E-15
ENSGALG00000008346	CASP18	7	1.13444E-15	3.62407E-15
ENSGALG00000045511	PARP9	7	1.13451E-15	3.62407E-15
ENSGALG00000007251	PCYT2	18	1.13517E-15	3.62522E-15
ENSGALG00000014766	HAO2	1	1.15371E-15	3.68348E-15
ENSGALG00000024171	HRH2	13	1.15537E-15	3.6878E-15
ENSGALG00000012827	RIPK1	2	1.15812E-15	3.69563E-15
ENSGALG00000035134	UBOX5	4	1.17763E-15	3.75689E-15
ENSGALG00000008614	TTL	3	1.19562E-15	3.81329E-15
ENSGALG00000016704	CDC5L	3	1.20153E-15	3.83114E-15
ENSGALG00000047517		30	1.23245E-15	3.92871E-15

ENSGALG00000054320		32	1.23606E-15	3.93917E-15
ENSGALG00000008582	ADAM23	7	1.24541E-15	3.96793E-15
ENSGALG00000010081	SH3D19	4	1.25086E-15	3.98427E-15
ENSGALG00000005425	OGT	4	1.26394E-15	4.02458E-15
ENSGALG00000003339	MTOR	21	1.26418E-15	4.02458E-15
ENSGALG00000005521	PER2	9	1.26761E-15	4.03445E-15
ENSGALG00000000820		23	1.26991E-15	4.04072E-15
ENSGALG00000004486	ZCCHC8	15	1.27243E-15	4.04769E-15
ENSGALG00000042796	DNAJC7	27	1.30041E-15	4.13561E-15
ENSGALG00000015465	ASCC3	3	1.30796E-15	4.15852E-15
ENSGALG00000008701	XDH	3	1.30958E-15	4.1626E-15
ENSGALG00000034213	TNFRSF1B	21	1.31566E-15	4.18084E-15
ENSGALG00000041192	IFIH1	7	1.3232E-15	4.20368E-15
ENSGALG00000051644		15	1.33894E-15	4.25259E-15
ENSGALG00000005028	TOP3A	14	1.34743E-15	4.27843E-15
ENSGALG00000042741	GRK2	5	1.36861E-15	4.34456E-15
ENSGALG00000004263	MYDGF	28	1.37319E-15	4.35798E-15
ENSGALG00000000721	SLC41A1	26	1.37812E-15	4.37139E-15
ENSGALG00000002985	UTP18	18	1.37814E-15	4.37139E-15
ENSGALG00000013430		2	1.41845E-15	4.49809E-15
ENSGALG00000042679	RETREG1	2	1.42274E-15	4.51053E-15
ENSGALG00000038858	ZFP64	20	1.44483E-15	4.57936E-15
ENSGALG00000008354	HARBI1	5	1.45127E-15	4.59858E-15
ENSGALG00000012166	SLC35F5	7	1.46007E-15	4.62527E-15
ENSGALG00000033660	PRDM10	24	1.48979E-15	4.71818E-15
ENSGALG00000009092	STON1	3	1.4987E-15	4.74518E-15
ENSGALG00000006516	MTHFS	10	1.52025E-15	4.81215E-15
ENSGALG00000006757	XYLT1	14	1.52597E-15	4.829E-15
ENSGALG00000037077	CCR5	2	1.53258E-15	4.84867E-15
ENSGALG00000014118	MRPS26	4	1.54363E-15	4.88234E-15
ENSGALG00000002099		12	1.54602E-15	4.88864E-15
ENSGALG00000011266	PPM1K	4	1.55383E-15	4.91207E-15
ENSGALG00000011531	APAF1	1	1.59594E-15	5.04389E-15

ENSGALG00000005326	EIF3F	6	1.59697E-15	5.04584E-15
ENSGALG00000010392	CCSER1	4	1.6067E-15	5.07526E-15
ENSGALG00000001717	SLC16A1	26	1.62076E-15	5.11835E-15
ENSGALG00000043363	KATNIP	14	1.62555E-15	5.13215E-15
ENSGALG00000029632	CHST13	12	1.63105E-15	5.14819E-15
ENSGALG00000034352	MRPL43	6	1.65861E-15	5.2338E-15
ENSGALG00000011688	TCP11L1	5	1.66404E-15	5.2496E-15
ENSGALG00000008784		8	1.66731E-15	5.25853E-15
ENSGALG00000001729	CD276	10	1.66924E-15	5.26328E-15
ENSGALG00000003243	RAD54L2	12	1.67617E-15	5.28374E-15
ENSGALG00000036355	C7orf73	1	1.67861E-15	5.29008E-15
ENSGALG00000015124	KCTD1	2	1.68888E-15	5.32107E-15
ENSGALG00000027872	STBD1	4	1.71804E-15	5.41155E-15
ENSGALG00000040672	IGFBP4	27	1.73325E-15	5.45805E-15
ENSGALG00000029322	FBXO39	19	1.75785E-15	5.53406E-15
ENSGALG00000016259	DYNLT3	1	1.75853E-15	5.53479E-15
ENSGALG00000048409	TXNIP	25	1.76576E-15	5.55611E-15
ENSGALG00000006465		12	1.77026E-15	5.56882E-15
ENSGALG00000013100	GRB10	2	1.80488E-15	5.67628E-15
ENSGALG00000030925	LHPP	6	1.83717E-15	5.77632E-15
ENSGALG00000043587	CCDC88A	3	1.84951E-15	5.81363E-15
ENSGALG00000035561	CPNE3	2	1.86895E-15	5.87323E-15
ENSGALG00000010493	INPP5A	6	1.87084E-15	5.87764E-15
ENSGALG00000035578	RBM6	12	1.88259E-15	5.91302E-15
ENSGALG00000035576	CDK9	17	1.90415E-15	5.97771E-15
ENSGALG00000050267	CALU	1	1.90416E-15	5.97771E-15
ENSGALG00000000720		23	1.92314E-15	6.03574E-15
ENSGALG00000003356	TM2D2	22	1.92757E-15	6.04807E-15
ENSGALG00000014386	SEPSECS	4	1.93767E-15	6.07822E-15
ENSGALG00000000787	DHX38	11	1.9385E-15	6.07927E-15
ENSGALG00000022874	RAB31	2	1.94145E-15	6.08692E-15
ENSGALG00000041604	NPTXR	1	1.96983E-15	6.17432E-15
ENSGALG00000033787		31	1.98321E-15	6.21466E-15

ENSGALG00000030673		5	1.98763E-15	6.22691E-15
ENSGALG00000003503	MYBL2	20	1.99898E-15	6.26087E-15
ENSGALG00000004590	CLCN6	21	2.01582E-15	6.31199E-15
ENSGALG00000003849	TRPM1	10	2.02856E-15	6.35025E-15
ENSGALG00000006647	DUSP8	5	2.04948E-15	6.4141E-15
ENSGALG00000005673	DTWD1	10	2.051E-15	6.4172E-15
ENSGALG00000052983		33	2.07509E-15	6.4909E-15
ENSGALG00000023142	PTGER3	8	2.07686E-15	6.49479E-15
ENSGALG00000050692	CBX8	18	2.08398E-15	6.51536E-15
ENSGALG00000045684		28	2.10706E-15	6.58586E-15
ENSGALG00000050742		4	2.11034E-15	6.59441E-15
ENSGALG00000009704	CHST15	6	2.17853E-15	6.80574E-15
ENSGALG00000036792	TIGD5	2	2.19345E-15	6.85059E-15
ENSGALG00000016444	ODC1	3	2.22044E-15	6.93312E-15
ENSGALG00000012200	GCH1	5	2.23681E-15	6.98244E-15
ENSGALG00000008791	NMT2	2	2.30265E-15	7.18612E-15
ENSGALG00000001837	ORAI3	19	2.33886E-15	7.29728E-15
ENSGALG00000016513	PPM1G	3	2.34556E-15	7.3163E-15
ENSGALG00000026639	SMCO4	1	2.35229E-15	7.33541E-15
ENSGALG00000037072	CCDC90B	1	2.39524E-15	7.46744E-15
ENSGALG00000034069	TMEM106C	33	2.40066E-15	7.48242E-15
ENSGALG00000008425	MAP1A	10	2.40944E-15	7.50788E-15
ENSGALG00000001387	SPNS2	19	2.41822E-15	7.53332E-15
ENSGALG00000028840	CDPF1	1	2.43329E-15	7.57832E-15
ENSGALG00000005894	USP8	10	2.45351E-15	7.63935E-15
ENSGALG00000051992	CYP2J23	8	2.49089E-15	7.75375E-15
ENSGALG00000007077	CPT1A	5	2.5047E-15	7.79473E-15
ENSGALG00000006942	PIK3IP1	15	2.52733E-15	7.86317E-15
ENSGALG00000002774	TSPAN3	10	2.53314E-15	7.87921E-15
ENSGALG00000012361	NIN	5	2.55179E-15	7.93522E-15
ENSGALG00000005706	HSCB	15	2.55614E-15	7.94671E-15
ENSGALG00000007395		6	2.56019E-15	7.95728E-15
ENSGALG00000008625	ANAPC2	17	2.59191E-15	8.05381E-15

ENSGALG00000040662	HSF3	4	2.60861E-15	8.10363E-15
ENSGALG00000009583	GORASP2	7	2.62099E-15	8.14002E-15
ENSGALG00000003672	UBLCP1	13	2.62329E-15	8.14509E-15
ENSGALG00000016300	TINAG	3	2.66461E-15	8.27127E-15
ENSGALG00000008932	ENTPD8	17	2.67622E-15	8.30519E-15
ENSGALG00000010000	PRKCE	3	2.6943E-15	8.35918E-15
ENSGALG00000049853	VAMP1	1	2.70497E-15	8.39015E-15
ENSGALG00000015795	ADAMTS5	1	2.73217E-15	8.47238E-15
ENSGALG00000010570	BTF3L4	8	2.77257E-15	8.59547E-15
ENSGALG00000003416	FHIP1B	1	2.78789E-15	8.64076E-15
ENSGALG00000023670	NAALADL2	9	2.80544E-15	8.69295E-15
ENSGALG00000037193	HECW1	2	2.81054E-15	8.70654E-15
ENSGALG00000005794	BLOC1S2	6	2.81325E-15	8.71271E-15
ENSGALG00000014054	GRK6	13	2.82263E-15	8.73956E-15
ENSGALG00000034081	AKT3	3	2.84609E-15	8.80995E-15
ENSGALG00000005704	NDUFB8	6	2.84708E-15	8.81077E-15
ENSGALG00000039682	HCLS1	1	2.861E-15	8.8516E-15
ENSGALG00000016766	REV1	1	2.87291E-15	8.88619E-15
ENSGALG00000014899	NUS1	3	2.88396E-15	8.91812E-15
ENSGALG00000032316	CDV3	2	2.9495E-15	9.11847E-15
ENSGALG00000001852	LRWD1	19	3.01241E-15	9.3106E-15
ENSGALG00000008182	CHAC2	3	3.04241E-15	9.40093E-15
ENSGALG00000015263		1	3.0766E-15	9.50416E-15
ENSGALG00000014355	STIM2	4	3.08201E-15	9.51848E-15
ENSGALG00000038488	GMEB1	23	3.1012E-15	9.57531E-15
ENSGALG00000014069	TP53I11	5	3.11313E-15	9.60973E-15
ENSGALG00000001371	HS3ST3A1	18	3.1145E-15	9.61151E-15
ENSGALG00000034289	SLC41A3	12	3.13674E-15	9.6777E-15
ENSGALG00000029175	EFNB2	1	3.14643E-15	9.70513E-15
ENSGALG00000053675		2	3.1482E-15	9.70815E-15
ENSGALG00000003652	ATAD1	6	3.17321E-15	9.78279E-15
ENSGALG00000033515	STK11IP	7	3.18133E-15	9.80533E-15
ENSGALG00000029485		20	3.20275E-15	9.86888E-15

ENSGALG00000029378	ITGA9	2	3.23508E-15	9.96597E-15
ENSGALG00000008474	CARF	7	3.26762E-15	1.00637E-14
ENSGALG00000007931	DLD	1	3.27252E-15	1.00762E-14
ENSGALG00000015348	ALCAM	1	3.29537E-15	1.01422E-14
ENSGALG00000006724		9	3.2956E-15	1.01422E-14
ENSGALG00000006868	CRACR2B	5	3.35385E-15	1.03188E-14
ENSGALG00000007721	ARHGAP21	2	3.42575E-15	1.05374E-14
ENSGALG00000006453	TF	9	3.4298E-15	1.05472E-14
ENSGALG00000009594	RARRES1	9	3.43522E-15	1.05612E-14
ENSGALG00000026367	FAM43B	21	3.44418E-15	1.05861E-14
ENSGALG00000010196	FAM161B	5	3.55946E-15	1.09376E-14
ENSGALG00000002663	ATR	9	3.62397E-15	1.11331E-14
ENSGALG00000002632	CALCRL	7	3.62989E-15	1.11484E-14
ENSGALG00000010589	FLRT2	5	3.70374E-15	1.13724E-14
ENSGALG00000003227	RAB11FIP4	18	3.7105E-15	1.13903E-14
ENSGALG00000040854	CPPED1	14	3.72449E-15	1.14303E-14
ENSGALG00000033259	SRSF5A	5	3.73104E-15	1.14476E-14
ENSGALG00000049239		7	3.75566E-15	1.15202E-14
ENSGALG00000010843	GPR137B	3	3.78398E-15	1.16041E-14
ENSGALG00000042890	PIN1	30	3.81417E-15	1.16938E-14
ENSGALG00000021399		18	3.85031E-15	1.18016E-14
ENSGALG00000008123	RINT1	1	3.99282E-15	1.22353E-14
ENSGALG00000008326		4	4.01699E-15	1.23063E-14
ENSGALG00000004741	EPHB2	21	4.03828E-15	1.23684E-14
ENSGALG00000017135	CRYL1	1	4.04283E-15	1.23793E-14
ENSGALG00000005279	BRIP1	19	4.05407E-15	1.24106E-14
ENSGALG00000017004	CDADC1	1	4.05835E-15	1.24205E-14
ENSGALG00000027789	RAB37	18	4.13009E-15	1.26369E-14
ENSGALG00000031841	SLX4	14	4.19505E-15	1.28321E-14
ENSGALG00000028225	SLC17A9	20	4.19597E-15	1.28321E-14
ENSGALG00000048704		13	4.24976E-15	1.29933E-14
ENSGALG00000040371		5	4.27772E-15	1.30755E-14
ENSGALG00000004907	KCTD15	11	4.46363E-15	1.36404E-14

ENSGALG00000016486	HS1BP3	3	4.53675E-15	1.38603E-14
ENSGALG00000031350	KCNE4	9	4.56557E-15	1.39449E-14
ENSGALG00000004246	SLC6A4	19	4.57026E-15	1.39557E-14
ENSGALG00000009943	FAM117A	27	4.57239E-15	1.39587E-14
ENSGALG00000028174	CAVIN2	7	4.57547E-15	1.39646E-14
ENSGALG00000011423	CEP43	3	4.59942E-15	1.40342E-14
ENSGALG00000004234	PGGHG	5	4.64808E-15	1.41791E-14
ENSGALG00000030065	TENM3	4	4.67288E-15	1.42512E-14
ENSGALG00000006527	CRTAM	24	4.7136E-15	1.43718E-14
ENSGALG00000052952		15	4.72233E-15	1.43949E-14
ENSGALG00000034487	IMPA1	2	4.77366E-15	1.45477E-14
ENSGALG00000040993	BAMBI	2	4.79437E-15	1.46071E-14
ENSGALG00000034534	FAAP24	11	4.81897E-15	1.46784E-14
ENSGALG00000031947	FBXL7	2	4.88533E-15	1.48768E-14
ENSGALG00000007133		14	4.90485E-15	1.49325E-14
ENSGALG00000031934	TES	1	5.08281E-15	1.54705E-14
ENSGALG00000005006	UBA2	11	5.21525E-15	1.58696E-14
ENSGALG00000028942	ZBTB25	5	5.2533E-15	1.59814E-14
ENSGALG00000008890	ADGRL4	8	5.38345E-15	1.63732E-14
ENSGALG00000011255	MCCC2L	8	5.41006E-15	1.64501E-14
ENSGALG00000007793	UPRT	4	5.46997E-15	1.66281E-14
ENSGALG00000026864	ZCCHC24	6	5.56362E-15	1.69086E-14
ENSGALG00000008445	SLC24A3	3	5.62626E-15	1.70947E-14
ENSGALG00000007287		5	5.63407E-15	1.71141E-14
ENSGALG00000030732	MAPK8IP1	5	5.63847E-15	1.71233E-14
ENSGALG00000015995	PTPRA	4	5.72718E-15	1.73883E-14
ENSGALG00000007549	VAPB	20	5.74122E-15	1.74266E-14
ENSGALG00000017089	SLC46A3	1	5.75494E-15	1.74639E-14
ENSGALG00000048936		17	5.76698E-15	1.74961E-14
ENSGALG00000007393	SUDS3	15	5.79016E-15	1.7562E-14
ENSGALG00000004845	TIMM10	5	5.82599E-15	1.76663E-14
ENSGALG00000041298		14	5.84478E-15	1.77189E-14
ENSGALG00000032090	TXNDC9	1	5.89381E-15	1.78631E-14

ENSGALG00000011660	WTAP	3	5.90219E-15	1.78816E-14
ENSGALG00000015182	NEPRO	1	5.90286E-15	1.78816E-14
ENSGALG00000006783	PLOD2	9	6.02209E-15	1.82383E-14
ENSGALG00000004055	C7orf50	14	6.17413E-15	1.86941E-14
ENSGALG00000039751	PANX1	1	6.20195E-15	1.87737E-14
ENSGALG00000016885	STK24	1	6.27524E-15	1.89908E-14
ENSGALG00000000621	KANK3	28	6.30463E-15	1.9075E-14
ENSGALG00000030450	CLIP2	19	6.33584E-15	1.91647E-14
ENSGALG00000037733		28	6.34475E-15	1.91869E-14
ENSGALG00000008281	NEURL1	6	6.36232E-15	1.92352E-14
ENSGALG00000038753	SAR1B	13	6.39432E-15	1.93272E-14
ENSGALG00000008962	MSH2	3	6.54929E-15	1.97907E-14
ENSGALG00000016128	B3GALT5	1	6.59564E-15	1.99258E-14
ENSGALG00000009709	MUM1L1	28	6.65817E-15	2.01098E-14
ENSGALG00000039895	EPHA2	21	6.68592E-15	2.01886E-14
ENSGALG00000015009	ZBTB49	4	6.86517E-15	2.07247E-14
ENSGALG00000028016		1	6.89858E-15	2.08204E-14
ENSGALG00000017125	MICU2	1	6.94326E-15	2.09501E-14
ENSGALG00000003107	AMFR	11	6.9694E-15	2.10237E-14
ENSGALG00000013583	FAM114A1	4	6.99077E-15	2.1083E-14
ENSGALG00000014883	MCM9	3	7.08788E-15	2.13705E-14
ENSGALG00000001882	TNFRSF18	21	7.16316E-15	2.15922E-14
ENSGALG00000025946	MOB3C	8	7.21781E-15	2.17515E-14
ENSGALG00000012113	C7H2ORF76	7	7.24263E-15	2.1821E-14
ENSGALG00000001153	AUTS2	19	7.28896E-15	2.19551E-14
ENSGALG00000012658	TCP11X2	1	7.48383E-15	2.25365E-14
ENSGALG00000053254	EHHADH	9	7.49077E-15	2.25518E-14
ENSGALG00000010103	FBXW7	4	7.52217E-15	2.26408E-14
ENSGALG00000011744	KALRN	7	7.54486E-15	2.27035E-14
ENSGALG00000009073	PSEN2	3	7.68046E-15	2.31058E-14
ENSGALG00000002579	RIMBP2	15	8.00747E-15	2.40836E-14
ENSGALG00000003789		17	8.0602E-15	2.42363E-14
ENSGALG00000006715	OAF	24	8.08056E-15	2.42915E-14

ENSGALG00000015556	AFAP1	4	8.14526E-15	2.448E-14
ENSGALG00000002651	ST3GAL2	11	8.16035E-15	2.45193E-14
ENSGALG00000031982	SDF2L1	15	8.18499E-15	2.45872E-14
ENSGALG00000029669	C1QC	21	8.20556E-15	2.4643E-14
ENSGALG00000041471	TUBB	16	8.28814E-15	2.48848E-14
ENSGALG00000043357	ANKRD13A	15	8.35386E-15	2.5076E-14
ENSGALG00000048926	WAS	30	8.35766E-15	2.50812E-14
ENSGALG00000015094	ZBTB20	1	8.38712E-15	2.51634E-14
ENSGALG00000004333	ABCA5	18	8.41156E-15	2.52305E-14
ENSGALG00000007711	RPL4	10	8.47881E-15	2.5426E-14
ENSGALG00000030780	MGAT4A	1	8.59882E-15	2.57796E-14
ENSGALG00000041236	CBX7	1	8.66127E-15	2.59604E-14
ENSGALG00000003429	PBX1	8	8.70446E-15	2.60834E-14
ENSGALG00000012847		1	8.72526E-15	2.61393E-14
ENSGALG00000029610	RGS9	18	8.75128E-15	2.62108E-14
ENSGALG00000012034	ADSL	1	8.80404E-15	2.63624E-14
ENSGALG00000011248	PLCL2	2	8.86717E-15	2.65449E-14
ENSGALG00000016144	TMPRSS2	1	8.86955E-15	2.65455E-14
ENSGALG00000014176	PLA2G2E	21	9.04311E-15	2.7057E-14
ENSGALG00000050757	ZDHHC12	17	9.0449E-15	2.7057E-14
ENSGALG00000008094	HSPD1	7	9.063E-15	2.71045E-14
ENSGALG00000039337	SH3BP5	2	9.2466E-15	2.76468E-14
ENSGALG00000042949	MGMT	6	9.55974E-15	2.85761E-14
ENSGALG00000006753	ATP5C1	1	9.62222E-15	2.87558E-14
ENSGALG00000014186	MPPED1	1	9.64564E-15	2.88188E-14
ENSGALG00000039549	SEL1L3	4	9.80148E-15	2.92772E-14
ENSGALG00000006233	GALNT11	2	9.82793E-15	2.9349E-14
ENSGALG00000034210	RPTOR	18	9.85817E-15	2.94321E-14
ENSGALG00000006822	PCDH19	4	9.90519E-15	2.95652E-14
ENSGALG00000002659	WDR5B	17	1.00004E-14	2.98422E-14
ENSGALG00000010622	ZC3H14	5	1.00579E-14	3.00064E-14
ENSGALG00000024485	BET1	2	1.01664E-14	3.03226E-14
ENSGALG00000006474	SKP1	13	1.03207E-14	3.07735E-14

ENSGALG00000014551		1	1.03226E-14	3.07735E-14
ENSGALG00000049673		8	1.03597E-14	3.08765E-14
ENSGALG00000052737		2	1.03882E-14	3.0954E-14
ENSGALG00000005315	RPUSD1	14	1.05191E-14	3.13364E-14
ENSGALG00000010286	DLST	5	1.05549E-14	3.14351E-14
ENSGALG00000004276	CHST12	14	1.06825E-14	3.18074E-14
ENSGALG00000041867	IFNAR2	1	1.07421E-14	3.19771E-14
ENSGALG00000013818	CITED2	3	1.07717E-14	3.20548E-14
ENSGALG00000025764	FKBP10	27	1.07734E-14	3.20548E-14
ENSGALG00000016361	FAM110C	3	1.0782E-14	3.20724E-14
ENSGALG00000038541	VPS37C	5	1.08297E-14	3.22064E-14
ENSGALG00000038540	ZBTB32	24	1.08542E-14	3.22715E-14
ENSGALG00000010554	RMDN2	3	1.10396E-14	3.28146E-14
ENSGALG00000029276	PPP1R9B	27	1.10845E-14	3.29401E-14
ENSGALG00000028295	MFAP1	10	1.11162E-14	3.30262E-14
ENSGALG00000034950	SMPD3	11	1.11926E-14	3.32451E-14
ENSGALG00000028505	PSMD4	25	1.12723E-14	3.34738E-14
ENSGALG00000004019	MARS2	4	1.13606E-14	3.37276E-14
ENSGALG00000026422		7	1.15216E-14	3.41973E-14
ENSGALG00000012025	TA3	5	1.1597E-14	3.44127E-14
ENSGALG00000012023	DACT1	5	1.17251E-14	3.47844E-14
ENSGALG00000033780		10	1.17422E-14	3.48266E-14
ENSGALG00000040008	STK11	28	1.17515E-14	3.48458E-14
ENSGALG00000012879	VPS4B	2	1.20342E-14	3.56754E-14
ENSGALG00000010836	AHR	2	1.20934E-14	3.58421E-14
ENSGALG00000002693	FAM149B1	6	1.21175E-14	3.5905E-14
ENSGALG00000009958	ABCG8	3	1.21844E-14	3.60943E-14
ENSGALG00000011566	GYPC	7	1.24959E-14	3.70083E-14
ENSGALG00000005909	GATA2	12	1.25847E-14	3.7262E-14
ENSGALG00000001223	PEX10	21	1.26731E-14	3.75146E-14
ENSGALG00000020747	MBD4	12	1.26908E-14	3.7558E-14
ENSGALG00000015064	TTC39C	2	1.28138E-14	3.7913E-14
ENSGALG00000005805	PLCD1	2	1.31221E-14	3.88155E-14

ENSGALG00000048865		10	1.35742E-14	4.0143E-14
ENSGALG00000043058	TBC1D22A	1	1.35804E-14	4.01518E-14
ENSGALG00000003893	XPR1	8	1.36373E-14	4.03103E-14
ENSGALG00000023444	APELA	4	1.38053E-14	4.07969E-14
ENSGALG00000031205	GNGT2	27	1.39178E-14	4.11194E-14
ENSGALG00000001054	C20H20ORF24	20	1.4038E-14	4.14646E-14
ENSGALG00000052196		23	1.42604E-14	4.21112E-14
ENSGALG00000010035	NR3C2	4	1.43491E-14	4.23627E-14
ENSGALG00000023338	CBX2	18	1.43987E-14	4.24991E-14
ENSGALG00000037669	FKBP9	2	1.44416E-14	4.26154E-14
ENSGALG00000005757	IRF8	11	1.47005E-14	4.33687E-14
ENSGALG00000008999	AFAP1L2	6	1.47476E-14	4.34971E-14
ENSGALG00000030899	ATXN7L3	27	1.50771E-14	4.44583E-14
ENSGALG00000038421	RWDD3	8	1.51061E-14	4.45329E-14
ENSGALG00000035060	FKBP11	33	1.51725E-14	4.4718E-14
ENSGALG00000053353	BOP1	2	1.54472E-14	4.55165E-14
ENSGALG00000010953	KANK4	8	1.56571E-14	4.61131E-14
ENSGALG00000006179	RPL13	11	1.56572E-14	4.61131E-14
ENSGALG00000001545	RAB40B	18	1.59995E-14	4.71099E-14
ENSGALG00000026838	MDFIC	1	1.6375E-14	4.82038E-14
ENSGALG00000014103	FRYL	4	1.64449E-14	4.83979E-14
ENSGALG00000033753	PDHX	5	1.66374E-14	4.89525E-14
ENSGALG00000036288	OAZ2	10	1.67305E-14	4.92147E-14
ENSGALG00000008873	POLE3	17	1.6745E-14	4.92456E-14
ENSGALG00000010653	SMYD3	3	1.68498E-14	4.95417E-14
ENSGALG00000014940	ZUP1	3	1.6912E-14	4.97127E-14
ENSGALG00000033083	ATOH8	4	1.7027E-14	5.00387E-14
ENSGALG00000037773	ST3GAL1	2	1.72123E-14	5.05709E-14
ENSGALG00000015152	BOC	1	1.73607E-14	5.09946E-14
ENSGALG00000013872	SEH1L	2	1.74287E-14	5.11822E-14
ENSGALG00000007769	FOXP1	12	1.74411E-14	5.12062E-14
ENSGALG00000016322		3	1.74858E-14	5.1325E-14
ENSGALG00000011483	MPC1L	3	1.75728E-14	5.1568E-14

ENSGALG00000006824	USP19	12	1.76792E-14	5.18677E-14
ENSGALG00000001838	XPO7	22	1.78378E-14	5.23204E-14
ENSGALG00000008975	FAM3C	1	1.80083E-14	5.28078E-14
ENSGALG00000038714	NDUFB7	30	1.8034E-14	5.28706E-14
ENSGALG00000038608	HSPB11	8	1.80428E-14	5.28836E-14
ENSGALG00000017715	TINAGL1	23	1.80486E-14	5.2888E-14
ENSGALG00000005438	PARVA	5	1.81399E-14	5.31427E-14
ENSGALG00000006934	PATZ1	15	1.81956E-14	5.3293E-14
ENSGALG00000006690	ECHDC3	1	1.82494E-14	5.34377E-14
ENSGALG00000001171	SEMA6B	28	1.8571E-14	5.43665E-14
ENSGALG00000035487	SP1	33	1.89254E-14	5.53906E-14
ENSGALG00000007752	COPS7B	9	1.92294E-14	5.62667E-14
ENSGALG00000039887		25	1.9243E-14	5.6293E-14
ENSGALG00000000581	DDX42	27	1.9293E-14	5.64259E-14
ENSGALG00000002260	CISH	12	1.94511E-14	5.68745E-14
ENSGALG00000033575	EIF2B4	3	1.95538E-14	5.71611E-14
ENSGALG00000050781		7	1.96078E-14	5.73053E-14
ENSGALG00000009029	TSPAN12	1	1.97603E-14	5.77372E-14
ENSGALG00000006143		6	1.98641E-14	5.80265E-14
ENSGALG00000009544	ZCRB1	1	1.9924E-14	5.81876E-14
ENSGALG00000033110	SNX33	10	2.00549E-14	5.85559E-14
ENSGALG00000037223		9	2.03541E-14	5.94152E-14
ENSGALG00000050631		14	2.09224E-14	6.10595E-14
ENSGALG00000006229		25	2.09567E-14	6.1145E-14
ENSGALG00000008039	MFSD13A	6	2.14582E-14	6.25932E-14
ENSGALG00000016934		1	2.15891E-14	6.296E-14
ENSGALG00000007362	ZDHHC5	5	2.17489E-14	6.34109E-14
ENSGALG00000033186	LRRC58	1	2.18072E-14	6.35657E-14
ENSGALG00000002160	B2M	10	2.18241E-14	6.35997E-14
ENSGALG00000052945		33	2.18562E-14	6.36782E-14
ENSGALG00000053701		27	2.18994E-14	6.37887E-14
ENSGALG00000041373	ARAP2	4	2.19917E-14	6.40423E-14
ENSGALG00000044177	FAN1	10	2.20107E-14	6.40824E-14

ENSGALG00000013757	TMX3	2	2.21128E-14	6.43641E-14
ENSGALG00000037869		25	2.2439E-14	6.52981E-14
ENSGALG00000010485	ALKBH1	5	2.2808E-14	6.63559E-14
ENSGALG00000005683	ARHGAP29	8	2.28466E-14	6.64525E-14
ENSGALG00000038599	PMM1	1	2.29363E-14	6.66975E-14
ENSGALG00000032029	SRF	3	2.35305E-14	6.84089E-14
ENSGALG00000051068		4	2.35524E-14	6.84564E-14
ENSGALG00000004781	ZBTB40	21	2.38581E-14	6.93282E-14
ENSGALG00000032386	DCLK2	4	2.38659E-14	6.93346E-14
ENSGALG00000040725		11	2.39925E-14	6.96858E-14
ENSGALG00000050814		2	2.40322E-14	6.97845E-14
ENSGALG00000012916	DROSHA	2	2.42589E-14	7.04258E-14
ENSGALG00000039245	STAB2	1	2.43854E-14	7.07762E-14
ENSGALG00000001429	TTC1	13	2.50755E-14	7.27618E-14
ENSGALG00000033573		26	2.54252E-14	7.37592E-14
ENSGALG00000005772	BOK	9	2.54752E-14	7.38866E-14
ENSGALG00000041993	UGDH	4	2.58837E-14	7.50535E-14
ENSGALG00000038582	SLC36A1	13	2.59265E-14	7.51588E-14
ENSGALG00000007491	VWA9	10	2.59324E-14	7.51588E-14
ENSGALG00000006015	COPB1	5	2.65547E-14	7.69443E-14
ENSGALG00000017291	FAR2	1	2.72493E-14	7.8938E-14
ENSGALG00000002237	ATP6V0D1	11	2.72618E-14	7.89556E-14
ENSGALG00000026445	CALM1	5	2.74649E-14	7.95249E-14
ENSGALG00000039327	VAV3	8	2.76959E-14	8.01748E-14
ENSGALG00000041086	MRPL15	2	2.857E-14	8.26856E-14
ENSGALG00000008086	ACTR1A	6	2.87251E-14	8.31148E-14
ENSGALG00000028290	C1H21ORF91	1	2.90714E-14	8.40968E-14
ENSGALG00000035619	MTTP	4	2.93542E-14	8.48946E-14
ENSGALG00000012488	TST	1	3.01122E-14	8.7066E-14
ENSGALG00000011966	RANGAP1	1	3.01893E-14	8.72685E-14
ENSGALG00000008302	CIB4	10	3.02802E-14	8.75105E-14
ENSGALG00000049138	TAF6	15	3.05841E-14	8.83677E-14
ENSGALG00000026973	KIF3C	3	3.07687E-14	8.88801E-14

ENSGALG00000051983	LETMD1	33	3.08523E-14	8.91004E-14
ENSGALG00000053058	LAMTOR2	25	3.09865E-14	8.94669E-14
ENSGALG00000002781	BRCA1	27	3.10208E-14	8.95445E-14
ENSGALG00000037031		17	3.15275E-14	9.09856E-14
ENSGALG00000010209	PGRMC2	4	3.1973E-14	9.22495E-14
ENSGALG00000011493	TMEM169	7	3.22369E-14	9.2989E-14
ENSGALG00000015596	MAP3K7	3	3.29356E-14	9.49821E-14
ENSGALG00000010234	MMACHC	8	3.39624E-14	9.79199E-14
ENSGALG00000002980	RTKN2	6	3.41853E-14	9.85393E-14
ENSGALG00000054336	CDC25B	4	3.42303E-14	9.86456E-14
ENSGALG00000045122	AKR1B1	1	3.4673E-14	9.9898E-14
ENSGALG00000002820	DFFA	21	3.47342E-14	1.00051E-13
ENSGALG00000033754	SURF1	17	3.51273E-14	1.01159E-13
ENSGALG00000003933	QSOX1	8	3.52813E-14	1.01578E-13
ENSGALG00000006252	LMO4	8	3.53575E-14	1.01774E-13
ENSGALG00000017244	PRSS23	1	3.54547E-14	1.02018E-13
ENSGALG00000001737	RABEP1	19	3.5459E-14	1.02018E-13
ENSGALG00000030475	SCO1	18	3.54807E-14	1.02056E-13
ENSGALG00000002313	SLC38A7	11	3.57187E-14	1.02716E-13
ENSGALG00000031002		25	3.63775E-14	1.04586E-13
ENSGALG00000030613	PDGFRB	13	3.64342E-14	1.04725E-13
ENSGALG00000049402		26	3.65305E-14	1.04977E-13
ENSGALG00000048705	PFDN6	16	3.69981E-14	1.06295E-13
ENSGALG00000040079	ZFPM2	2	3.73134E-14	1.07176E-13
ENSGALG00000044155	TMEM140	1	3.74354E-14	1.07501E-13
ENSGALG00000005587	EIF4G2	5	3.7827E-14	1.086E-13
ENSGALG00000003837	ADCY7	11	3.79016E-14	1.08789E-13
ENSGALG00000012105	EXOC5	5	3.81173E-14	1.09382E-13
ENSGALG00000010118	MIPOL1	5	3.83206E-14	1.0994E-13
ENSGALG00000035282	IGF2	5	3.87676E-14	1.11196E-13
ENSGALG00000005872	BANP	11	3.88647E-14	1.11448E-13
ENSGALG00000017644	COTL1	11	3.9199E-14	1.1238E-13
ENSGALG00000054287		1	3.95618E-14	1.13394E-13

ENSGALG00000016314	ELOVL5	3	4.02318E-14	1.15287E-13
ENSGALG00000040368	CALR	30	4.03266E-14	1.15532E-13
ENSGALG00000010940	ACSS3	1	4.05341E-14	1.16099E-13
ENSGALG00000047483	C1QTNF6	1	4.05579E-14	1.1614E-13
ENSGALG00000010979		4	4.1055E-14	1.17518E-13
ENSGALG00000023541	SPHK1	18	4.10586E-14	1.17518E-13
ENSGALG00000031723	BANF1	33	4.13509E-14	1.18327E-13
ENSGALG00000011692	CDCA4	5	4.19407E-14	1.19986E-13
ENSGALG00000004400		19	4.21728E-14	1.20622E-13
ENSGALG00000031932	AGPAT3	1	4.2826E-14	1.22462E-13
ENSGALG00000009483	MARK1	3	4.29135E-14	1.22683E-13
ENSGALG00000005074	FAM102A	17	4.30391E-14	1.23013E-13
ENSGALG00000016440	KLF11	3	4.35685E-14	1.24497E-13
ENSGALG00000014042	SSPN	1	4.3872E-14	1.25335E-13
ENSGALG00000030515	UBE2N	1	4.39518E-14	1.25534E-13
ENSGALG00000044204	ISG20	10	4.47411E-14	1.27758E-13
ENSGALG00000011419	DUSP4	4	4.52983E-14	1.29303E-13
ENSGALG00000008753	FBXO48	3	4.53033E-14	1.29303E-13
ENSGALG00000005713	NCKIPSD	12	4.59857E-14	1.3122E-13
ENSGALG00000040864	WDR77	26	4.61351E-14	1.31616E-13
ENSGALG00000007419	MASP1	9	4.62073E-14	1.31791E-13
ENSGALG00000009268	FGG	4	4.76574E-14	1.35895E-13
ENSGALG00000052712		22	4.79506E-14	1.36699E-13
ENSGALG00000053464	CCDC191	1	4.83856E-14	1.37883E-13
ENSGALG00000004728	RGL1	8	4.83886E-14	1.37883E-13
ENSGALG00000020884		8	4.88432E-14	1.39146E-13
ENSGALG00000006812	TTC36	24	4.95212E-14	1.41045E-13
ENSGALG00000001392	MMP23B	21	4.96908E-14	1.41495E-13
ENSGALG00000004227	FAM20B	8	4.98761E-14	1.41989E-13
ENSGALG00000027093	TLR1B	4	5.01308E-14	1.42681E-13
ENSGALG00000036942	KCNA3	26	5.02273E-14	1.42922E-13
ENSGALG00000014026	DNAJB14	4	5.03284E-14	1.43176E-13
ENSGALG00000001823	HIKESHI	1	5.09365E-14	1.44872E-13

ENSGALG00000044463		13	5.10481E-14	1.45156E-13
ENSGALG00000047290	KIAA1755	20	5.13559E-14	1.45997E-13
ENSGALG00000013505	SYNE1	3	5.14351E-14	1.46188E-13
ENSGALG00000050150	STX5	33	5.17521E-14	1.47055E-13
ENSGALG00000041181		14	5.23599E-14	1.48747E-13
ENSGALG00000000692	CDK18	26	5.24518E-14	1.48974E-13
ENSGALG00000004324	SETD1A	15	5.25112E-14	1.49108E-13
ENSGALG00000037076	GART	1	5.25683E-14	1.49235E-13
ENSGALG00000012206	CGRRF1	5	5.26973E-14	1.49567E-13
ENSGALG00000032701	LY96	2	5.33461E-14	1.51373E-13
ENSGALG00000012414	GNPNAT1	5	5.37564E-14	1.52501E-13
ENSGALG00000039269	RNF213	18	5.37902E-14	1.52562E-13
ENSGALG00000011046	SNX10	2	5.56625E-14	1.57836E-13
ENSGALG00000044210	QPCTL	32	5.61159E-14	1.59084E-13
ENSGALG00000017204	SESN3	1	5.62026E-14	1.59293E-13
ENSGALG00000014854	MYL12A	2	5.71142E-14	1.61839E-13
ENSGALG00000052296	MEX3D	28	5.73681E-14	1.62521E-13
ENSGALG00000003124	BRF2	22	5.85014E-14	1.65693E-13
ENSGALG00000010881	ASB2	5	5.85719E-14	1.65854E-13
ENSGALG00000005298		17	5.88764E-14	1.66678E-13
ENSGALG00000016880	UBAC2	1	5.94836E-14	1.68322E-13
ENSGALG00000013952	B3GALNT2	3	5.94915E-14	1.68322E-13
ENSGALG00000010973	TRA2A	2	5.94988E-14	1.68322E-13
ENSGALG00000034507	CHST2	9	5.95399E-14	1.68399E-13
ENSGALG00000038758	MMP15	11	6.01727E-14	1.7015E-13
ENSGALG00000037483	ANP32B	28	6.12222E-14	1.73077E-13
ENSGALG00000046639	CYP2AC2	3	6.13431E-14	1.73379E-13
ENSGALG00000002707	CHRNB2	25	6.14632E-14	1.73678E-13
ENSGALG00000001048	OLFML2A	17	6.17339E-14	1.74403E-13
ENSGALG00000051554		6	6.40691E-14	1.80958E-13
ENSGALG00000041923	ESPL1	33	6.55044E-14	1.84969E-13
ENSGALG00000013094	DDC	2	6.62435E-14	1.87013E-13
ENSGALG00000012816	SLC22A23	2	6.6921E-14	1.88881E-13

ENSGALG00000047622		25	6.869E-14	1.9383E-13
ENSGALG00000005294	PPFIBP2	5	6.89675E-14	1.94568E-13
ENSGALG00000042941	MRPL9	25	6.92654E-14	1.95363E-13
ENSGALG00000037519	C1orf35	2	6.97035E-14	1.96553E-13
ENSGALG00000008902	GIPC2	8	6.97676E-14	1.96688E-13
ENSGALG00000004623	AGTRAP	21	6.99084E-14	1.9704E-13
ENSGALG00000027654	C5orf49	2	7.03946E-14	1.98364E-13
ENSGALG00000030994	MRPL24	25	7.0541E-14	1.98731E-13
ENSGALG00000002138	HYAL2	12	7.07638E-14	1.99312E-13
ENSGALG00000006955	CCNJ	6	7.08384E-14	1.99476E-13
ENSGALG00000007253	IL1RAP	9	7.11285E-14	2.00247E-13
ENSGALG00000027198	TMEM47	1	7.17854E-14	2.02034E-13
ENSGALG00000009292		5	7.17964E-14	2.02034E-13
ENSGALG00000012076	TIFA	4	7.25071E-14	2.03987E-13
ENSGALG00000025755	SPTSSA	5	7.26031E-14	2.0421E-13
ENSGALG00000040755	ANGPT4	20	7.26238E-14	2.04221E-13
ENSGALG00000028254	WASHC4	1	7.29077E-14	2.04972E-13
ENSGALG00000007337	LPP	9	7.30911E-14	2.0544E-13
ENSGALG00000009440	BMT2	1	7.33484E-14	2.06116E-13
ENSGALG00000016830	CUL4A	1	7.34052E-14	2.06228E-13
ENSGALG00000013747	TAGAP	3	7.3438E-14	2.06273E-13
ENSGALG00000012448	ORC4	7	7.40198E-14	2.07859E-13
ENSGALG00000052529	RNF4	4	7.4258E-14	2.0848E-13
ENSGALG00000033149	GCC1	1	7.42785E-14	2.08489E-13
ENSGALG00000009639	DDX60	4	7.44112E-14	2.08814E-13
ENSGALG00000008088	ACSL4	4	7.46533E-14	2.09445E-13
ENSGALG00000035667	NOC3L	6	7.51095E-14	2.10676E-13
ENSGALG00000016412	MBOAT2	3	7.54282E-14	2.11522E-13
ENSGALG00000040784	RAB39B	4	7.6144E-14	2.1348E-13
ENSGALG00000008794	RPP38	2	7.62975E-14	2.13861E-13
ENSGALG00000013229	CMAS	1	7.74362E-14	2.17003E-13
ENSGALG00000043118	ATP10A	1	7.86329E-14	2.20306E-13
ENSGALG00000043400	BCDIN3D	33	7.9112E-14	2.21597E-13

ENSGALG00000009266	FGA	4	7.92407E-14	2.21907E-13
ENSGALG00000039334		5	7.98857E-14	2.23662E-13
ENSGALG00000003474	MED20	26	7.99837E-14	2.23885E-13
ENSGALG00000002499		23	8.15E-14	2.28076E-13
ENSGALG00000039775	SHC1	25	8.1859E-14	2.29029E-13
ENSGALG00000004222	HK1	6	8.27076E-14	2.3135E-13
ENSGALG00000016994	NUDT15	1	8.30344E-14	2.32211E-13
ENSGALG00000006473	PLXNA4	1	8.51391E-14	2.37994E-13
ENSGALG00000040007	IL17D	1	8.51416E-14	2.37994E-13
ENSGALG00000016410	EIF1AX	1	8.55558E-14	2.39097E-13
ENSGALG00000016772	PDCL3	1	8.58222E-14	2.39787E-13
ENSGALG00000038155	AP3M2	22	8.65823E-14	2.41855E-13
ENSGALG00000037553	SEC11A	10	8.68783E-14	2.42626E-13
ENSGALG00000004555	RBM22	13	8.84091E-14	2.46845E-13
ENSGALG00000015813	USP16	1	8.87324E-14	2.47691E-13
ENSGALG00000005393	ARF1	2	9.07259E-14	2.53198E-13
ENSGALG00000011226	ANKRD28	2	9.19891E-14	2.56664E-13
ENSGALG00000013980	SLC2A12	3	9.2696E-14	2.58578E-13
ENSGALG00000050420		2	9.56275E-14	2.66647E-13
ENSGALG00000007652	SUCLG2	12	9.56324E-14	2.66647E-13
ENSGALG00000011207	DUSP6	1	9.6081E-14	2.67836E-13
ENSGALG00000027372	PIP5K1A	25	9.65629E-14	2.69118E-13
ENSGALG00000032526	B3GNT5	9	9.858E-14	2.74677E-13
ENSGALG00000020454	C5H14ORF169	5	9.92253E-14	2.76412E-13
ENSGALG00000010190	TBC1D15	1	1.00388E-13	2.79587E-13
ENSGALG00000016377	PXDN	3	1.00414E-13	2.79597E-13
ENSGALG00000009475	FAM133B	2	1.01621E-13	2.8289E-13
ENSGALG00000051677		1	1.01699E-13	2.83043E-13
ENSGALG00000047207		4	1.02449E-13	2.85066E-13
ENSGALG00000009784	PTPN14	3	1.02697E-13	2.85692E-13
ENSGALG00000026578		2	1.03355E-13	2.87457E-13
ENSGALG00000003111	BFAR	14	1.03477E-13	2.87731E-13
ENSGALG00000002375	NOTCH1	17	1.04226E-13	2.89746E-13

ENSGALG00000000991	TAF12	23	1.05142E-13	2.92225E-13
ENSGALG00000017299	RPS11	1	1.05884E-13	2.94222E-13
ENSGALG00000008066	UQCR10	15	1.06892E-13	2.96955E-13
ENSGALG00000047327		23	1.06931E-13	2.96996E-13
ENSGALG00000005473	PNAT3	11	1.07077E-13	2.97321E-13
ENSGALG00000012680	E2F3	2	1.07097E-13	2.97321E-13
ENSGALG00000046464	RUSC1	25	1.08603E-13	3.01432E-13
ENSGALG00000011185	PLEKHA8	2	1.09293E-13	3.0328E-13
ENSGALG00000035138	UBL5	30	1.09833E-13	3.04708E-13
ENSGALG00000027991	TTLL4	7	1.10269E-13	3.05847E-13
ENSGALG00000033471	CALD1	1	1.10463E-13	3.06316E-13
ENSGALG00000024083	TCIM	22	1.10831E-13	3.07266E-13
ENSGALG00000041927	FZD9	19	1.11154E-13	3.08094E-13
ENSGALG00000046000	ICE2	10	1.126E-13	3.1203E-13
ENSGALG00000012211	PDLIM5	4	1.13892E-13	3.15537E-13
ENSGALG00000004646	FBXO44	21	1.14543E-13	3.17269E-13
ENSGALG00000004783	C17H9orf16	17	1.15253E-13	3.19165E-13
ENSGALG00000003473	SFRP1	22	1.15695E-13	3.20316E-13
ENSGALG00000012355	DARS	7	1.16508E-13	3.22493E-13
ENSGALG00000038678	EPB41L3	2	1.18175E-13	3.27034E-13
ENSGALG00000004604		17	1.20058E-13	3.32167E-13
ENSGALG00000041420		5	1.20283E-13	3.32715E-13
ENSGALG00000016691	SLC25A6	1	1.21303E-13	3.35462E-13
ENSGALG00000005080	C8H1orf27	8	1.21384E-13	3.35609E-13
ENSGALG00000014516	CPEB2	4	1.22173E-13	3.37713E-13
ENSGALG00000031896	GSE1	11	1.22285E-13	3.37947E-13
ENSGALG00000042534	NFATC1	2	1.23921E-13	3.4239E-13
ENSGALG00000004759	DHRS7B	14	1.24142E-13	3.42924E-13
ENSGALG00000011982	PDE5A	4	1.25502E-13	3.46601E-13
ENSGALG00000028958	NOL9	21	1.26878E-13	3.50322E-13
ENSGALG00000006631	TICRR	10	1.27064E-13	3.50757E-13
ENSGALG00000009681	BUB3	6	1.27225E-13	3.51121E-13
ENSGALG00000012377	HNMT	7	1.28791E-13	3.55363E-13

ENSGALG00000008838	FKBP15	17	1.2898E-13	3.55804E-13
ENSGALG00000002463	SUMF2	19	1.30628E-13	3.60269E-13
ENSGALG00000007018	SLC26A11	18	1.30666E-13	3.60292E-13
ENSGALG00000002159	ACOX1	18	1.309E-13	3.60856E-13
ENSGALG00000005295	BCAS3	19	1.31289E-13	3.61845E-13
ENSGALG00000026493	RALB	7	1.31633E-13	3.62712E-13
ENSGALG00000016062	PSMG1	1	1.31718E-13	3.62864E-13
ENSGALG00000009399	GATAD1	2	1.32596E-13	3.65201E-13
ENSGALG00000008714	ABI2	7	1.34181E-13	3.69483E-13
ENSGALG00000012850	RAB19	1	1.34692E-13	3.70806E-13
ENSGALG00000000404	COPS2	10	1.35868E-13	3.73959E-13
ENSGALG00000012087	CEP70	7	1.36945E-13	3.76838E-13
ENSGALG00000015327	TXNL4B	1	1.39482E-13	3.83732E-13
ENSGALG00000045136	POLG	10	1.39918E-13	3.84846E-13
ENSGALG00000004900	RFLNB	19	1.40347E-13	3.85938E-13
ENSGALG00000013571	F11	4	1.41493E-13	3.89001E-13
ENSGALG00000011794	DDX47	1	1.42789E-13	3.92476E-13
ENSGALG00000007416	CD3E	24	1.42892E-13	3.92672E-13
ENSGALG00000027247	EOMES	2	1.43232E-13	3.93517E-13
ENSGALG00000008911	RNF208	17	1.4364E-13	3.94548E-13
ENSGALG00000014298	RFC1	4	1.44326E-13	3.96344E-13
ENSGALG00000034137		3	1.45194E-13	3.98586E-13
ENSGALG00000014982	RBBP8	2	1.45208E-13	3.98586E-13
ENSGALG00000004707	CSE1L	20	1.45927E-13	4.00469E-13
ENSGALG00000035505	AOAH	2	1.46001E-13	4.00582E-13
ENSGALG00000041687	SREBF2	1	1.46892E-13	4.02937E-13
ENSGALG00000009841	FASTKD1	7	1.49847E-13	4.10951E-13
ENSGALG00000006583	LSP1P1	5	1.52735E-13	4.18777E-13
ENSGALG00000038340	ZBTB14	2	1.53167E-13	4.19867E-13
ENSGALG00000005888	PGP	14	1.54126E-13	4.224E-13
ENSGALG00000026733	FFAR4	6	1.59161E-13	4.361E-13
ENSGALG00000007904	DLAT	24	1.60183E-13	4.38804E-13
ENSGALG00000017327	P2RY6	1	1.60759E-13	4.40282E-13

ENSGALG00000028203	SPRED1	5	1.61347E-13	4.41794E-13
ENSGALG00000038041	PHF14	2	1.62124E-13	4.43822E-13
ENSGALG00000003891	ZNF423	11	1.62496E-13	4.4474E-13
ENSGALG00000040877	UBALD2	18	1.62782E-13	4.45422E-13
ENSGALG00000006949	CHD2	10	1.63279E-13	4.46681E-13
ENSGALG00000009988	ELOVL1	8	1.65367E-13	4.52293E-13
ENSGALG00000028506	NHSL2	4	1.6576E-13	4.53265E-13
ENSGALG00000003832	TEX264	12	1.6622E-13	4.54421E-13
ENSGALG00000021637	WDR18	28	1.67708E-13	4.58386E-13
ENSGALG00000000870	DCAKD	27	1.68387E-13	4.60139E-13
ENSGALG00000020391	SERPINA10	5	1.71489E-13	4.68511E-13
ENSGALG00000016058	ERG	1	1.72206E-13	4.70364E-13
ENSGALG00000008744	MCF2L2	9	1.76597E-13	4.8225E-13
ENSGALG00000009533	NMD3	9	1.77215E-13	4.83831E-13
ENSGALG00000039121	PPM1F	15	1.78046E-13	4.85991E-13
ENSGALG00000014698	CASP2	1	1.79783E-13	4.90621E-13
ENSGALG00000048775	TXLNA	23	1.81814E-13	4.96052E-13
ENSGALG00000035635	ACVRL1	33	1.8477E-13	5.04006E-13
ENSGALG00000037327	DCAF17	7	1.85507E-13	5.05901E-13
ENSGALG00000029408	BAZ1B	19	1.87734E-13	5.1186E-13
ENSGALG00000051072		16	1.88566E-13	5.13971E-13
ENSGALG00000005918	DIPK1A	8	1.88592E-13	5.13971E-13
ENSGALG00000010804	ANKMY2	2	1.90209E-13	5.18261E-13
ENSGALG00000000371	CRHR1	27	1.90775E-13	5.19686E-13
ENSGALG00000002497	RHOT2	14	1.91158E-13	5.20614E-13
ENSGALG00000040504	GGT5	15	1.91747E-13	5.22101E-13
ENSGALG00000041898	FAM43A	9	1.94001E-13	5.28121E-13
ENSGALG00000001741	NPTN	10	1.95116E-13	5.31039E-13
ENSGALG00000009642	AVEN	5	1.95662E-13	5.32405E-13
ENSGALG00000039299	LASP1	27	1.97579E-13	5.37502E-13
ENSGALG00000017042	UFM1	1	1.98927E-13	5.41048E-13
ENSGALG00000011697	SEC22A	7	1.99053E-13	5.41269E-13
ENSGALG00000046729		30	1.99498E-13	5.4236E-13

ENSGALG00000029867	MROH1	2	2.0004E-13	5.4371E-13
ENSGALG00000004369	PYGO1	10	2.00805E-13	5.45668E-13
ENSGALG00000037370	NAV1	26	2.00853E-13	5.45677E-13
ENSGALG00000029659	BPHL	2	2.08453E-13	5.66198E-13
ENSGALG00000001267	MAP2K2	28	2.09089E-13	5.678E-13
ENSGALG00000040030	PHLDB1	24	2.11099E-13	5.73131E-13
ENSGALG00000008319	PLBD2	15	2.14667E-13	5.82688E-13
ENSGALG00000013874	PEX7	3	2.15084E-13	5.8369E-13
ENSGALG00000036069	TCIRG1	5	2.17687E-13	5.90623E-13
ENSGALG00000016810	SH3RF3	1	2.19235E-13	5.94689E-13
ENSGALG00000016510	KRTCAP3	3	2.1929E-13	5.94708E-13
ENSGALG00000009063		4	2.20628E-13	5.98202E-13
ENSGALG00000006076	RASGEF1C	13	2.22672E-13	6.03611E-13
ENSGALG00000000668	RBBP5	26	2.24143E-13	6.07462E-13
ENSGALG00000006355	SPTY2D1	5	2.28762E-13	6.19843E-13
ENSGALG00000000284	HOXB4	27	2.28959E-13	6.20238E-13
ENSGALG00000029831	IFFO2	21	2.30994E-13	6.25612E-13
ENSGALG00000002554	TSPAN2	26	2.31477E-13	6.26781E-13
ENSGALG00000004304	TCF12	10	2.35569E-13	6.37721E-13
ENSGALG00000004841	APLNR	5	2.41817E-13	6.54489E-13
ENSGALG00000032974	ADAMTS2	13	2.45986E-13	6.65626E-13
ENSGALG00000038141	NCF4	1	2.49396E-13	6.74703E-13
ENSGALG00000031209	GINS3	11	2.5008E-13	6.76404E-13
ENSGALG00000029266	ZDHHC3	2	2.5062E-13	6.77713E-13
ENSGALG00000043684	PSMD13	5	2.52522E-13	6.82706E-13
ENSGALG00000015977	SMOX	4	2.54072E-13	6.86743E-13
ENSGALG00000032887	CHIR-B3	31	2.55987E-13	6.91768E-13
ENSGALG00000023612	LZTS2	6	2.5814E-13	6.9743E-13
ENSGALG00000038256		33	2.59216E-13	7.00181E-13
ENSGALG00000017021	ATP7B	1	2.60374E-13	7.03153E-13
ENSGALG00000040267	IVD	5	2.6139E-13	7.05736E-13
ENSGALG00000013579	FAT1	4	2.61446E-13	7.05736E-13
ENSGALG00000007217	BEST1	5	2.61646E-13	7.06119E-13

ENSGALG00000016936	TDRD3	1	2.64395E-13	7.1338E-13
ENSGALG00000048303		10	2.68218E-13	7.23535E-13
ENSGALG00000014772	WARS2	1	2.68907E-13	7.25234E-13
ENSGALG00000030401	MED31	19	2.71101E-13	7.30989E-13
ENSGALG00000041463		22	2.71825E-13	7.32778E-13
ENSGALG00000001101	MBD3	28	2.73364E-13	7.36765E-13
ENSGALG00000030125	ANKRD10	1	2.75485E-13	7.42318E-13
ENSGALG00000008675	SLC25A5	4	2.7662E-13	7.45212E-13
ENSGALG00000042851	PPARGC1A	4	2.77264E-13	7.46781E-13
ENSGALG00000021117	RNF7	9	2.7834E-13	7.49513E-13
ENSGALG00000050796		27	2.79203E-13	7.51672E-13
ENSGALG00000020078	H3F3C	3	2.80922E-13	7.56134E-13
ENSGALG00000003798	GET4	14	2.88681E-13	7.76847E-13
ENSGALG00000017205	ENDOD1	1	2.89823E-13	7.79746E-13
ENSGALG00000049356		14	2.90144E-13	7.80438E-13
ENSGALG00000009504		6	2.90959E-13	7.82457E-13
ENSGALG00000016497	UBXN2A	3	2.93744E-13	7.89772E-13
ENSGALG00000015464	PTGFRN	1	2.99283E-13	8.04487E-13
ENSGALG00000013609	MTRF1L	3	2.99485E-13	8.04854E-13
ENSGALG00000002531	SF3B3	11	3.00923E-13	8.08539E-13
ENSGALG00000038559	CD8A	4	3.03589E-13	8.15522E-13
ENSGALG00000042759	PRKX	1	3.09547E-13	8.31344E-13
ENSGALG00000003575		20	3.13847E-13	8.42708E-13
ENSGALG00000040308	BAHCC1	18	3.1493E-13	8.4543E-13
ENSGALG00000004474	PLEKHF1	11	3.16287E-13	8.48885E-13
ENSGALG00000003901	P2RX4	15	3.17598E-13	8.52109E-13
ENSGALG00000008286	SUMF1	12	3.17628E-13	8.52109E-13
ENSGALG00000003929	SYS1	20	3.19443E-13	8.5679E-13
ENSGALG00000004974	PPARG	12	3.19565E-13	8.56928E-13
ENSGALG00000031751	SCRN3	7	3.27503E-13	8.78022E-13
ENSGALG00000016881	GPR183	1	3.30067E-13	8.84703E-13
ENSGALG00000044654	C22orf39	15	3.31552E-13	8.88487E-13
ENSGALG00000043208	TRAFD1	15	3.32743E-13	8.91482E-13

ENSGALG00000003709	PLAT	22	3.34529E-13	8.96072E-13
ENSGALG00000019355	CPSF6	1	3.36417E-13	9.00856E-13
ENSGALG00000034427	SCN4A	27	3.36463E-13	9.00856E-13
ENSGALG00000037407	FAM118B	24	3.39566E-13	9.08963E-13
ENSGALG00000009848	LPGAT1	3	3.44908E-13	9.23061E-13
ENSGALG00000052511		9	3.47033E-13	9.28545E-13
ENSGALG00000010610	SNX25	4	3.49199E-13	9.34135E-13
ENSGALG00000009689	PON1	2	3.52134E-13	9.4178E-13
ENSGALG00000019397	CERK	1	3.52574E-13	9.42748E-13
ENSGALG00000042986	RNASEL	8	3.54388E-13	9.47391E-13
ENSGALG00000016357	PRDX4	1	3.55516E-13	9.50197E-13
ENSGALG00000042564	UBAC1	17	3.57133E-13	9.54311E-13
ENSGALG00000026715	MIF4GD	18	3.62394E-13	9.68157E-13
ENSGALG00000034225	DEAF1	5	3.62762E-13	9.68927E-13
ENSGALG00000004756	CEP89	11	3.65845E-13	9.76949E-13
ENSGALG00000047514		11	3.66822E-13	9.79344E-13
ENSGALG00000027365	HIVEP3	23	3.67762E-13	9.81639E-13
ENSGALG00000009732	PCDH18	4	3.69583E-13	9.86282E-13
ENSGALG00000012052	SNX4	7	3.73673E-13	9.96978E-13
ENSGALG00000012638	CRY1	1	3.74119E-13	9.97917E-13
ENSGALG00000000764	SSR2	25	3.74188E-13	9.97917E-13
ENSGALG00000003935	PBLD	6	3.79728E-13	1.01247E-12
ENSGALG00000017329	FCHSD2	1	3.81967E-13	1.01822E-12
ENSGALG00000048534		11	3.845E-13	1.02475E-12
ENSGALG00000009052	LBH	3	3.88567E-13	1.03536E-12
ENSGALG00000006404	DDX46	13	3.89051E-13	1.03642E-12
ENSGALG00000042771	GBE1	1	3.90074E-13	1.03892E-12
ENSGALG00000010448	LTBP1	3	3.90158E-13	1.03892E-12
ENSGALG00000005092	HDAC11	12	3.9606E-13	1.0544E-12
ENSGALG00000004569	UNC5B	6	3.99076E-13	1.0622E-12
ENSGALG00000032207	HMGN4	23	4.0199E-13	1.06972E-12
ENSGALG00000048091		27	4.03584E-13	1.07373E-12
ENSGALG00000002703	TFAM	6	4.04883E-13	1.07695E-12

ENSGALG00000037854	TKFC	5	4.0746E-13	1.08357E-12
ENSGALG00000049589		26	4.09326E-13	1.08829E-12
ENSGALG00000044661		28	4.10792E-13	1.09195E-12
ENSGALG00000048656		7	4.15011E-13	1.10293E-12
ENSGALG00000002403	ULK1	15	4.1824E-13	1.11127E-12
ENSGALG00000006539	C9H21orf2	9	4.19637E-13	1.11473E-12
ENSGALG00000016969		1	4.26411E-13	1.13248E-12
ENSGALG00000006646	ABCC1	14	4.27966E-13	1.13637E-12
ENSGALG00000008569	CHP1	5	4.31732E-13	1.14612E-12
ENSGALG00000049024	CD7	18	4.3721E-13	1.16041E-12
ENSGALG00000036821	HSF1	2	4.43977E-13	1.17811E-12
ENSGALG00000027561	GNG5	8	4.46542E-13	1.18466E-12
ENSGALG00000041454	DNMT3A	3	4.53945E-13	1.20403E-12
ENSGALG00000010575	INTS12	4	4.59186E-13	1.21767E-12
ENSGALG00000052629	TWSG1	2	4.59873E-13	1.21923E-12
ENSGALG00000012685	KDM1B	2	4.60313E-13	1.22013E-12
ENSGALG00000016516	ZNF513	3	4.65175E-13	1.23275E-12
ENSGALG00000011518	POMGNT2	2	4.67748E-13	1.2393E-12
ENSGALG00000033807	TYSND1	6	4.75286E-13	1.259E-12
ENSGALG00000011038	CBX3	2	4.7658E-13	1.26198E-12
ENSGALG00000051980		2	4.7662E-13	1.26198E-12
ENSGALG00000034374	STX7	3	4.77174E-13	1.26317E-12
ENSGALG00000014953	RWDD1	3	4.84023E-13	1.28103E-12
ENSGALG00000012968	RMND1	3	4.84973E-13	1.28326E-12
ENSGALG00000052918		28	4.86493E-13	1.28701E-12
ENSGALG00000000410	TLK2	27	4.8911E-13	1.29365E-12
ENSGALG00000012974	WASH1	1	4.90397E-13	1.29677E-12
ENSGALG00000044505		25	4.90658E-13	1.29718E-12
ENSGALG00000001797	GPSM1	17	4.92106E-13	1.30073E-12
ENSGALG00000005944	ZNF639	9	4.95288E-13	1.30885E-12
ENSGALG00000035919	EPN2	14	5.00677E-13	1.32281E-12
ENSGALG00000043128	ARHGEF10L	21	5.02322E-13	1.32687E-12
ENSGALG00000035417	TCEB1	2	5.07615E-13	1.34056E-12

ENSGALG00000048144		8	5.0993E-13	1.34638E-12
ENSGALG00000043379	RPL14	2	5.10922E-13	1.3487E-12
ENSGALG00000000775	CTSS	25	5.11994E-13	1.35124E-12
ENSGALG00000009169	TRMT6	3	5.15067E-13	1.35906E-12
ENSGALG00000036790	RPS27A	3	5.17118E-13	1.36418E-12
ENSGALG00000028032	MSRA	3	5.27931E-13	1.3924E-12
ENSGALG00000048773		33	5.41558E-13	1.42803E-12
ENSGALG00000004477		19	5.48987E-13	1.44731E-12
ENSGALG00000039523	MRM1	19	5.52995E-13	1.45756E-12
ENSGALG00000035502	ZBTB39	33	5.53845E-13	1.45948E-12
ENSGALG00000006494	GRAMD1B	24	5.56906E-13	1.46723E-12
ENSGALG00000016537	SCML2	1	5.68651E-13	1.49785E-12
ENSGALG00000013143	PDE3A	1	5.74011E-13	1.51164E-12
ENSGALG00000016567	EPHX2	3	5.74766E-13	1.51331E-12
ENSGALG00000010311	NAV3	1	5.76091E-13	1.51647E-12
ENSGALG00000041072	TATDN1	2	6.07556E-13	1.59895E-12
ENSGALG00000004212	ADAM10	10	6.09764E-13	1.60441E-12
ENSGALG00000017154	ZC3H12C	1	6.14755E-13	1.6172E-12
ENSGALG00000010689	MAGOH	8	6.30351E-13	1.65787E-12
ENSGALG00000013018	TPRKB	1	6.32904E-13	1.66422E-12
ENSGALG00000033557	CTSH	10	6.37773E-13	1.67666E-12
ENSGALG00000006843	NCBP2	9	6.38285E-13	1.67765E-12
ENSGALG00000000724	PM20D1	26	6.39584E-13	1.6807E-12
ENSGALG00000050258		15	6.44567E-13	1.69343E-12
ENSGALG00000030677	ABCG2	6	6.50959E-13	1.70985E-12
ENSGALG00000028971	NKIRAS2	27	6.54024E-13	1.71754E-12
ENSGALG00000031996		33	6.56984E-13	1.72494E-12
ENSGALG00000052408		17	6.71253E-13	1.76202E-12
ENSGALG00000004170	ADA	20	6.74514E-13	1.7702E-12
ENSGALG00000005321	LAMA5	20	6.79571E-13	1.78309E-12
ENSGALG00000010811	LGMN	5	6.85427E-13	1.79806E-12
ENSGALG00000026891	NINJ1	12	6.88586E-13	1.80597E-12
ENSGALG00000028866	COASY	27	6.92448E-13	1.8157E-12

ENSGALG00000001978	MED16	28	7.1191E-13	1.86633E-12
ENSGALG00000000596	CDC27	27	7.12771E-13	1.86819E-12
ENSGALG00000005420	AATF	19	7.13566E-13	1.86987E-12
ENSGALG00000015017		3	7.28289E-13	1.90804E-12
ENSGALG00000001035		28	7.31491E-13	1.91602E-12
ENSGALG00000012074	ALPK1	4	7.37969E-13	1.93257E-12
ENSGALG00000011124	ARHGAP24	4	7.39525E-13	1.93623E-12
ENSGALG00000009528	TMEM192	4	7.4261E-13	1.94389E-12
ENSGALG00000016590	TLR7	1	7.57186E-13	1.98162E-12
ENSGALG00000012787	BMP6	2	7.63798E-13	1.99849E-12
ENSGALG00000011304	NGLY1	2	7.66507E-13	2.00515E-12
ENSGALG00000030886	PTGDS	17	7.68489E-13	2.00991E-12
ENSGALG00000003118	ADGRA2	22	7.73873E-13	2.02355E-12
ENSGALG00000011953	PHF5A	1	7.75654E-13	2.02778E-12
ENSGALG00000008542	ADD3	6	7.77339E-13	2.03175E-12
ENSGALG00000045132		31	7.81545E-13	2.0423E-12
ENSGALG00000034917	GCDH	30	7.83715E-13	2.04753E-12
ENSGALG00000006629	RBP4A	6	7.90151E-13	2.0639E-12
ENSGALG00000036582		14	7.90694E-13	2.06488E-12
ENSGALG00000010285	MRPL14	3	7.90864E-13	2.06488E-12
ENSGALG00000017201	CEP57	1	7.93975E-13	2.0725E-12
ENSGALG00000015666	TNIP2	4	7.9412E-13	2.0725E-12
ENSGALG00000029743	MED6	5	7.98584E-13	2.0837E-12
ENSGALG00000031503	LRP6	1	8.07307E-13	2.10601E-12
ENSGALG00000031214	MED9	14	8.35908E-13	2.18015E-12
ENSGALG00000045815		1	8.367E-13	2.18175E-12
ENSGALG00000033447	H2AZ2	22	8.41805E-13	2.1946E-12
ENSGALG00000003602	RPGRIP1L	11	8.46661E-13	2.20678E-12
ENSGALG00000008207	FBXW8	15	8.4815E-13	2.21019E-12
ENSGALG00000034876	PDP1	2	8.51521E-13	2.2185E-12
ENSGALG00000034504		1	8.58596E-13	2.23646E-12
ENSGALG00000005643	LYVE1	5	8.59736E-13	2.23895E-12
ENSGALG00000054667		16	8.60039E-13	2.23926E-12

ENSGALG00000011171	TBP	3	8.6457E-13	2.25058E-12
ENSGALG00000009493	TMEM229B	5	8.8167E-13	2.2946E-12
ENSGALG00000034171	EPB41L1	20	8.8831E-13	2.31139E-12
ENSGALG00000004259	BLMH	19	8.98123E-13	2.33642E-12
ENSGALG00000009622		4	8.9971E-13	2.34005E-12
ENSGALG00000001325	CYP1A1	10	9.0514E-13	2.35367E-12
ENSGALG00000034806		2	9.28548E-13	2.41403E-12
ENSGALG00000005397	GGNBP2	19	9.42823E-13	2.45062E-12
ENSGALG00000020582	SLC39A3	28	9.47478E-13	2.46219E-12
ENSGALG00000036067	RBFA	2	9.48695E-13	2.46483E-12
ENSGALG00000009476	CDK6	2	9.50511E-13	2.46902E-12
ENSGALG00000027188	SREBF1	14	9.59245E-13	2.49118E-12
ENSGALG00000006157		4	9.67955E-13	2.51326E-12
ENSGALG00000012761	DRAM1	1	9.69981E-13	2.51798E-12
ENSGALG00000017139		1	9.74737E-13	2.52979E-12
ENSGALG00000037940	IGFBP3	2	9.81431E-13	2.54663E-12
ENSGALG00000030068	RAB3A	28	9.84042E-13	2.55286E-12
ENSGALG00000037480	ENSA	25	9.85819E-13	2.55688E-12
ENSGALG00000004174	VPS26A	6	9.86012E-13	2.55688E-12
ENSGALG00000013642	KAZN	21	9.93658E-13	2.57616E-12
ENSGALG00000028083	THSD1	1	9.9897E-13	2.58938E-12
ENSGALG00000030176	SLC31A2	17	1.01672E-12	2.63483E-12
ENSGALG00000023920	PHYKPL	13	1.02327E-12	2.65123E-12
ENSGALG00000052072		2	1.0356E-12	2.68262E-12
ENSGALG00000021647	NRTN	28	1.03807E-12	2.68844E-12
ENSGALG00000008914	NRAP	6	1.0528E-12	2.72601E-12
ENSGALG00000052371		16	1.05505E-12	2.73126E-12
ENSGALG00000011499	SCARB2	4	1.06065E-12	2.74518E-12
ENSGALG00000014347		1	1.06114E-12	2.74586E-12
ENSGALG00000002415	SAP30BP	18	1.06167E-12	2.74664E-12
ENSGALG00000028499	MINDY1	25	1.09223E-12	2.82511E-12
ENSGALG00000002900	NOTUM	18	1.10579E-12	2.85957E-12
ENSGALG00000039320	ADGRF5	3	1.11139E-12	2.87344E-12

ENSGALG00000016823	TFDP1	1	1.11376E-12	2.87896E-12
ENSGALG00000004662	DISP3	21	1.12118E-12	2.89752E-12
ENSGALG00000001476		26	1.1294E-12	2.91816E-12
ENSGALG00000040947	CREG2	1	1.13399E-12	2.92939E-12
ENSGALG00000005547	PIK3AP1	6	1.1481E-12	2.96521E-12
ENSGALG00000004386	SOX9	18	1.16804E-12	3.01607E-12
ENSGALG00000008220	BZW1	7	1.17048E-12	3.02175E-12
ENSGALG00000002764	ATP1B3	9	1.17386E-12	3.02983E-12
ENSGALG00000007955	SLC16A5	18	1.17526E-12	3.0328E-12
ENSGALG00000014575	SEPHS2L	1	1.1905E-12	3.07148E-12
ENSGALG00000012178	PDGFB	1	1.2165E-12	3.13788E-12
ENSGALG00000004128	CYSLTR1	4	1.2196E-12	3.14522E-12
ENSGALG00000009016	SLX4IP	3	1.22673E-12	3.16293E-12
ENSGALG00000008139	EML6	3	1.23407E-12	3.18119E-12
ENSGALG00000043573	CHMP4C	2	1.23453E-12	3.18169E-12
ENSGALG00000039172	ASPSCR1	18	1.2541E-12	3.23146E-12
ENSGALG00000040905	NTAN1	14	1.25453E-12	3.23187E-12
ENSGALG00000037525	FGFRL1	4	1.25507E-12	3.23259E-12
ENSGALG00000026957	SEMA4G	6	1.2772E-12	3.28889E-12
ENSGALG00000029918		23	1.2984E-12	3.34278E-12
ENSGALG00000036925	CADM3	25	1.30823E-12	3.36738E-12
ENSGALG00000032660	IFNGR2	1	1.3203E-12	3.39774E-12
ENSGALG00000043650	GFI1	8	1.32899E-12	3.41936E-12
ENSGALG00000035493	POC1B	1	1.35857E-12	3.49472E-12
ENSGALG00000001107	GOSR2	27	1.36338E-12	3.50637E-12
ENSGALG00000002524	UCHL5	8	1.37333E-12	3.53121E-12
ENSGALG00000031058		18	1.39485E-12	3.58578E-12
ENSGALG00000011593	TMEM177	7	1.40628E-12	3.61441E-12
ENSGALG00000009698	SLC38A1	1	1.42454E-12	3.66057E-12
ENSGALG00000038207		22	1.44056E-12	3.70097E-12
ENSGALG00000036395		27	1.47757E-12	3.79524E-12
ENSGALG00000030432	RASSF2	22	1.47932E-12	3.79893E-12
ENSGALG00000049538	RPS28	28	1.51911E-12	3.9003E-12

ENSGALG00000034271	UHRF1	28	1.55863E-12	4.00092E-12
ENSGALG00000012684	DEK	2	1.58093E-12	4.05731E-12
ENSGALG00000013061	PAPD7	2	1.59276E-12	4.08682E-12
ENSGALG00000003702	CLINT1	13	1.60278E-12	4.11164E-12
ENSGALG00000031021	ERICH1	3	1.63622E-12	4.19655E-12
ENSGALG00000008979	TRMT61B	3	1.63764E-12	4.19931E-12
ENSGALG00000032087	MC5R	2	1.64211E-12	4.20988E-12
ENSGALG00000017091	FLT1	1	1.6565E-12	4.24589E-12
ENSGALG00000011881	CCNA2	4	1.65744E-12	4.2474E-12
ENSGALG00000030710		20	1.6648E-12	4.26538E-12
ENSGALG00000005378	ARNTL	5	1.67354E-12	4.28687E-12
ENSGALG00000053788	TNFSF8	17	1.68683E-12	4.32001E-12
ENSGALG00000005552	PTBP2	8	1.69931E-12	4.35105E-12
ENSGALG00000046992	MCRIP2	14	1.71087E-12	4.37973E-12
ENSGALG00000041380	BF2	16	1.72815E-12	4.42304E-12
ENSGALG00000009948	HHIP	4	1.73879E-12	4.44933E-12
ENSGALG00000030626	FZD6	2	1.7994E-12	4.60345E-12
ENSGALG00000005549	CUEDC1	19	1.81128E-12	4.63288E-12
ENSGALG00000048701		5	1.82727E-12	4.67279E-12
ENSGALG00000001595	PHF19	17	1.83834E-12	4.70012E-12
ENSGALG00000030269	CNIH4	3	1.84337E-12	4.71199E-12
ENSGALG00000017485	TLR1A	4	1.84389E-12	4.71233E-12
ENSGALG00000007537	INCENP	5	1.84646E-12	4.71792E-12
ENSGALG00000046413	PRR15L	27	1.86282E-12	4.75871E-12
ENSGALG00000008682	SLC25A43	4	1.87699E-12	4.79391E-12
ENSGALG00000002249	AGO1	23	1.90746E-12	4.87071E-12
ENSGALG00000007819	PDZRN3	12	1.92888E-12	4.92437E-12
ENSGALG00000026999	SNRPB2	3	1.93857E-12	4.94807E-12
ENSGALG00000000318	CSRP1	26	1.94527E-12	4.96414E-12
ENSGALG00000001118	NEK6	17	1.9734E-12	5.03487E-12
ENSGALG00000017039	STOML3	1	1.97475E-12	5.03727E-12
ENSGALG00000027330	SYNPO	13	1.97647E-12	5.04061E-12
ENSGALG00000016088	CCT7	4	1.9896E-12	5.07302E-12

ENSGALG00000032287	ATP2A2	15	2.00058E-12	5.09996E-12
ENSGALG00000006109	MAPK8	6	2.00331E-12	5.10585E-12
ENSGALG00000053667		1	2.02184E-12	5.152E-12
ENSGALG00000003863	P2RX7	15	2.02928E-12	5.16987E-12
ENSGALG00000002038	DENND2C	26	2.0497E-12	5.22081E-12
ENSGALG00000030303	PPP1R3B	4	2.05289E-12	5.22783E-12
ENSGALG00000009510	PIGH	5	2.05867E-12	5.24147E-12
ENSGALG00000005054	DKC1	4	2.08389E-12	5.30457E-12
ENSGALG00000003249	CHUK	6	2.09233E-12	5.32493E-12
ENSGALG00000046085	FAM110D	23	2.10188E-12	5.34813E-12
ENSGALG00000048552		2	2.11471E-12	5.37966E-12
ENSGALG00000026940	TTYH2	18	2.16392E-12	5.50368E-12
ENSGALG00000054634		28	2.17793E-12	5.53816E-12
ENSGALG00000017090	POMP	1	2.19679E-12	5.58496E-12
ENSGALG00000006889	TMC7	14	2.20433E-12	5.60295E-12
ENSGALG00000010669	TDP1	5	2.20725E-12	5.60921E-12
ENSGALG00000039781	TXNDC15	13	2.27313E-12	5.77544E-12
ENSGALG00000006575	VIPR2	2	2.29298E-12	5.82464E-12
ENSGALG00000016447	PDIA6	3	2.30335E-12	5.84978E-12
ENSGALG00000014754		1	2.3932E-12	6.07669E-12
ENSGALG00000005030	DOCK10	9	2.40318E-12	6.10077E-12
ENSGALG00000005086	PLAU	6	2.4435E-12	6.20183E-12
ENSGALG00000012697	SLC41A2	1	2.45995E-12	6.24229E-12
ENSGALG00000012542	RASD2	1	2.49325E-12	6.32546E-12
ENSGALG00000009947	PLEKHH2	3	2.50171E-12	6.3456E-12
ENSGALG00000011208	HELQ	4	2.51135E-12	6.36873E-12
ENSGALG00000007218	MB21D2	9	2.57194E-12	6.52104E-12
ENSGALG00000002653	NCBP3	19	2.57375E-12	6.52426E-12
ENSGALG00000049875		15	2.6498E-12	6.71566E-12
ENSGALG00000006567	UBASH3B	24	2.66702E-12	6.75788E-12
ENSGALG00000006933	MOSMO	14	2.68729E-12	6.80784E-12
ENSGALG00000016241	ATP6AP2	1	2.69111E-12	6.8161E-12
ENSGALG00000021883	C19orf44	28	2.71182E-12	6.86712E-12

ENSGALG00000017022	ALG11	1	2.75019E-12	6.96284E-12
ENSGALG00000009759	SLC16A7	1	2.75335E-12	6.9694E-12
ENSGALG00000036873	SIK2	24	2.76167E-12	6.98901E-12
ENSGALG00000023909		17	2.78188E-12	7.03869E-12
ENSGALG00000035345	TXNRD1	1	2.78322E-12	7.04063E-12
ENSGALG00000007874	IL18	24	2.78991E-12	7.05468E-12
ENSGALG00000006165		6	2.78993E-12	7.05468E-12
ENSGALG00000002204	SLC7A4	15	2.80881E-12	7.10094E-12
ENSGALG00000035856	CD48	25	2.81255E-12	7.10892E-12
ENSGALG00000015240		1	2.82083E-12	7.12838E-12
ENSGALG00000040426	CREG1	1	2.86631E-12	7.24181E-12
ENSGALG00000011099	FAP	7	2.87881E-12	7.27186E-12
ENSGALG00000005442	PALMD	8	2.89424E-12	7.30933E-12
ENSGALG00000009048	ABLIM1	6	2.89487E-12	7.30942E-12
ENSGALG00000054937		13	2.90577E-12	7.33543E-12
ENSGALG00000031424	SDC2	2	2.92334E-12	7.37824E-12
ENSGALG00000006300	LDHA	5	2.94325E-12	7.42695E-12
ENSGALG00000036616	NUAK2	26	2.98116E-12	7.52106E-12
ENSGALG00000009112	LRRC57	5	3.04318E-12	7.67594E-12
ENSGALG00000000535	ERRFI1	21	3.0688E-12	7.73896E-12
ENSGALG00000010043	RBKS	3	3.0817E-12	7.76987E-12
ENSGALG00000009378	PDGFC	4	3.08438E-12	7.77504E-12
ENSGALG00000004480	C11H19orf12	11	3.09313E-12	7.79549E-12
ENSGALG00000041556	GPATCH8	27	3.09553E-12	7.79991E-12
ENSGALG00000011921	DHRS7	5	3.10041E-12	7.8106E-12
ENSGALG00000037944	LNPK	7	3.10192E-12	7.81278E-12
ENSGALG00000041666	ASNSD1	7	3.12416E-12	7.86717E-12
ENSGALG00000032916		15	3.13004E-12	7.88036E-12
ENSGALG00000028685	SEMA5A	2	3.13352E-12	7.8875E-12
ENSGALG00000038747	TM4SF18	9	3.14204E-12	7.90732E-12
ENSGALG00000037164	ZFHX4	2	3.14358E-12	7.90956E-12
ENSGALG00000017036	LHFPL6	1	3.1444E-12	7.90999E-12
ENSGALG00000019921	NDUFAF4	3	3.15691E-12	7.9398E-12

ENSGALG00000023844		20	3.17162E-12	7.97515E-12
ENSGALG00000014263	PHC1	1	3.18235E-12	8.0005E-12
ENSGALG00000016251		1	3.18579E-12	8.0075E-12
ENSGALG00000006598	SORL1	24	3.20992E-12	8.06648E-12
ENSGALG00000033941	RORC	25	3.24819E-12	8.16096E-12
ENSGALG00000031175	LZTR1	26	3.25928E-12	8.18714E-12
ENSGALG00000016089	PRADC1	4	3.26581E-12	8.20185E-12
ENSGALG00000047792	SELENOM	15	3.27951E-12	8.23449E-12
ENSGALG00000024326	RPUSD4	24	3.28015E-12	8.23449E-12
ENSGALG00000008230	PCGF6	6	3.32197E-12	8.33776E-12
ENSGALG00000054971	ARHGAP30	25	3.32672E-12	8.34795E-12
ENSGALG00000009135	PLEKHA3	7	3.36073E-12	8.43156E-12
ENSGALG00000011655	IQCB1	7	3.36793E-12	8.44789E-12
ENSGALG00000006596	SNRPD3	15	3.41711E-12	8.56949E-12
ENSGALG00000020164	GAR1	4	3.42318E-12	8.58293E-12
ENSGALG00000010468		5	3.4474E-12	8.64188E-12
ENSGALG00000002042		14	3.4556E-12	8.66068E-12
ENSGALG00000031384		14	3.48373E-12	8.72937E-12
ENSGALG00000011357	VEZT	1	3.49045E-12	8.74441E-12
ENSGALG00000048951		32	3.51542E-12	8.80515E-12
ENSGALG00000002891	ACADL	7	3.52343E-12	8.8234E-12
ENSGALG00000004980	KCNMA1	6	3.53864E-12	8.85968E-12
ENSGALG00000016583	ESCO2	3	3.55053E-12	8.88763E-12
ENSGALG00000035152	ALKBH2	15	3.61945E-12	9.05828E-12
ENSGALG00000040484	SEC31A	4	3.66953E-12	9.18172E-12
ENSGALG00000005379	SGSM1	15	3.73112E-12	9.33392E-12
ENSGALG00000031275	TUBB6	2	3.74344E-12	9.36282E-12
ENSGALG00000037203	CTNNB1	2	3.75418E-12	9.38777E-12
ENSGALG00000039498	PPP3CA	4	3.78592E-12	9.4652E-12
ENSGALG00000038946	KLHL38	2	3.78988E-12	9.47315E-12
ENSGALG00000048210		32	3.79442E-12	9.48254E-12
ENSGALG00000039724	RHOF	15	3.83099E-12	9.57197E-12
ENSGALG00000043569	NME5	13	3.84002E-12	9.59257E-12

ENSGALG00000051669	ARL5A	7	3.84843E-12	9.61162E-12
ENSGALG00000028439	SHLD2	6	3.8709E-12	9.66576E-12
ENSGALG00000004121	LPAR4	4	3.87507E-12	9.67419E-12
ENSGALG00000003712	RAPGEF1	17	3.89943E-12	9.73301E-12
ENSGALG00000008385	PPIP5K1	10	3.9013E-12	9.73568E-12
ENSGALG00000014167	TTLL12	1	3.92518E-12	9.79327E-12
ENSGALG00000007048	SYNM	10	3.9348E-12	9.81527E-12
ENSGALG00000037699		4	3.9768E-12	9.918E-12
ENSGALG00000034548	PLEKHA1	6	3.99691E-12	9.96613E-12
ENSGALG00000014847	MYOM1	2	4.0261E-12	1.00368E-11
ENSGALG00000009593	MFSD1	9	4.03637E-12	1.00604E-11
ENSGALG00000052573	UBE2S	22	4.04003E-12	1.00674E-11
ENSGALG00000007698		15	4.04085E-12	1.00674E-11
ENSGALG00000033631	MYC	2	4.04373E-12	1.00726E-11
ENSGALG00000024486	C4orf48	4	4.05609E-12	1.01013E-11
ENSGALG00000007045	ADGRA1	6	4.09078E-12	1.01856E-11
ENSGALG00000028937	TIMM13	28	4.09379E-12	1.0191E-11
ENSGALG00000013651	RNF217	3	4.12376E-12	1.02635E-11
ENSGALG00000054783	NDRG1	2	4.14357E-12	1.03107E-11
ENSGALG00000004342	ORC6	11	4.154E-12	1.03346E-11
ENSGALG00000037273		1	4.22984E-12	1.05211E-11
ENSGALG00000036490	CYRIB	2	4.26382E-12	1.06035E-11
ENSGALG00000014362	CD86	1	4.26983E-12	1.06162E-11
ENSGALG00000033855	GOLT1B	1	4.29111E-12	1.0667E-11
ENSGALG00000008747	ITGA8	2	4.43182E-12	1.10145E-11
ENSGALG00000011360	PRKAG3	7	4.4736E-12	1.11161E-11
ENSGALG00000010837	ASB5	4	4.50053E-12	1.11807E-11
ENSGALG00000001527	VWA1	21	4.57773E-12	1.13702E-11
ENSGALG00000032430	IPO5	1	4.61058E-12	1.14495E-11
ENSGALG00000052056		14	4.62642E-12	1.14865E-11
ENSGALG00000036471	RFTN2	7	4.66462E-12	1.15789E-11
ENSGALG00000008795	GPAM	6	4.75802E-12	1.18084E-11
ENSGALG00000007619	UBALD1	14	4.77617E-12	1.1851E-11

ENSGALG00000053595	CXXC4	4	4.78322E-12	1.18661E-11
ENSGALG00000005331	GJC2	2	4.79428E-12	1.18911E-11
ENSGALG00000012269		5	4.82527E-12	1.19655E-11
ENSGALG00000052641	PLD6	14	4.83378E-12	1.19842E-11
ENSGALG00000009372	PHC3	9	4.86028E-12	1.20474E-11
ENSGALG00000010335		8	4.88792E-12	1.21135E-11
ENSGALG00000004749	TMEM11	14	4.91744E-12	1.21842E-11
ENSGALG00000007000	NR2F2	10	4.9276E-12	1.22069E-11
ENSGALG00000009002	CPED1	1	4.93169E-12	1.22145E-11
ENSGALG00000037716	UBA52	28	4.94218E-12	1.2238E-11
ENSGALG00000008098	NAMPTP1	1	4.95295E-12	1.22622E-11
ENSGALG00000033338	GPT2	11	4.95651E-12	1.22685E-11
ENSGALG00000006113	ZNF326	8	5.00812E-12	1.23937E-11
ENSGALG00000003781	SUN1	14	5.04726E-12	1.24881E-11
ENSGALG00000010471	PPP2R2D	6	5.06586E-12	1.25313E-11
ENSGALG00000052676	CYP2J22	8	5.06679E-12	1.25313E-11
ENSGALG00000004645	TBC1D13	17	5.10234E-12	1.26167E-11
ENSGALG00000027834	ERGIC3	20	5.16305E-12	1.27642E-11
ENSGALG00000040962	CPSF7	5	5.17351E-12	1.27875E-11
ENSGALG00000013728	PPAT	4	5.23364E-12	1.29335E-11
ENSGALG00000031851	TENT4B	11	5.25618E-12	1.29865E-11
ENSGALG00000040097	UBE2W	2	5.27148E-12	1.30217E-11
ENSGALG00000044525	NFKBIE	3	5.41056E-12	1.33626E-11
ENSGALG00000011623	ADAMTS3	4	5.43597E-12	1.34226E-11
ENSGALG00000007465	COX15	6	5.44583E-12	1.34442E-11
ENSGALG00000008226	NIF3L1	7	5.47664E-12	1.35175E-11
ENSGALG00000011434	SLC11A1	7	5.63003E-12	1.38933E-11
ENSGALG00000023742	AHR2	7	5.64026E-12	1.39158E-11
ENSGALG00000021656	PTPN22	26	5.66059E-12	1.39631E-11
ENSGALG00000016930	DIS3	1	5.67357E-12	1.39923E-11
ENSGALG00000023294	RPLP0	15	5.83852E-12	1.43962E-11
ENSGALG00000023953		26	5.85839E-12	1.44395E-11
ENSGALG00000039696	ENTPD2	17	5.85844E-12	1.44395E-11

ENSGALG00000031348	SPPL2A	10	6.11654E-12	1.50726E-11
ENSGALG00000041625	LSM5	2	6.18963E-12	1.52496E-11
ENSGALG00000039595	BTBD11	1	6.28397E-12	1.54789E-11
ENSGALG00000006430		15	6.30117E-12	1.55181E-11
ENSGALG00000038032	DUT	10	6.34978E-12	1.56347E-11
ENSGALG00000052604		2	6.47971E-12	1.59514E-11
ENSGALG00000042330	PPP1R9B	7	6.48224E-12	1.59544E-11
ENSGALG00000011616	NPFFR2	4	6.49088E-12	1.59724E-11
ENSGALG00000004364	ABR	19	6.49701E-12	1.59843E-11
ENSGALG00000026492	EMC6	19	6.67374E-12	1.64158E-11
ENSGALG00000003855	SRC	20	6.80659E-12	1.67392E-11
ENSGALG00000008632	ACSS1	3	6.92363E-12	1.70236E-11
ENSGALG00000005470	PLPPR5	8	6.95667E-12	1.71013E-11
ENSGALG00000017262	NARS2	1	6.97936E-12	1.71537E-11
ENSGALG00000005229	DPH5	8	6.98648E-12	1.71646E-11
ENSGALG00000008368	RAD18	12	6.98662E-12	1.71646E-11
ENSGALG00000016425	IAH1	3	6.99537E-12	1.71826E-11
ENSGALG00000011812		4	7.02239E-12	1.72455E-11
ENSGALG00000000745	SLC26A9	26	7.05775E-12	1.73289E-11
ENSGALG00000011427	USP37	7	7.06474E-12	1.73425E-11
ENSGALG00000041238		17	7.10894E-12	1.74475E-11
ENSGALG00000012763	GNPTAB	1	7.11921E-12	1.74692E-11
ENSGALG00000014836	LPIN2	2	7.22066E-12	1.77146E-11
ENSGALG00000016017	SLC4A11	4	7.27608E-12	1.7847E-11
ENSGALG00000001568	NFS1	20	7.2996E-12	1.7901E-11
ENSGALG00000003273	HSDL1	11	7.31332E-12	1.79311E-11
ENSGALG00000010899	G6PC2	7	7.3459E-12	1.80074E-11
ENSGALG00000011305	SRSF11	8	7.35585E-12	1.80281E-11
ENSGALG00000051974		31	7.42251E-12	1.81878E-11
ENSGALG00000041417	ACADSB	6	7.4817E-12	1.83292E-11
ENSGALG00000036310		13	7.52497E-12	1.84315E-11
ENSGALG00000040957	AGO2	2	7.59955E-12	1.86104E-11
ENSGALG00000039080	CD44	5	7.63602E-12	1.8696E-11

ENSGALG00000003881	ERCC6L	4	7.83023E-12	1.91676E-11
ENSGALG00000035841	PRRC2C	8	7.88487E-12	1.92975E-11
ENSGALG00000004613	P2RX5	19	7.94872E-12	1.94499E-11
ENSGALG00000043044	IL1R1	1	7.95533E-12	1.94621E-11
ENSGALG00000026392	PIPOX	19	8.01397E-12	1.96017E-11
ENSGALG00000049798	HDGFL3	10	8.08481E-12	1.9771E-11
ENSGALG00000009463	DCAF5	5	8.16441E-12	1.99616E-11
ENSGALG00000006149	MORN4	6	8.20141E-12	2.00481E-11
ENSGALG00000007017	RBM7	24	8.25774E-12	2.01817E-11
ENSGALG00000033300	CORO1B	5	8.26879E-12	2.02047E-11
ENSGALG00000031519	KHDRBS3	2	8.27484E-12	2.02154E-11
ENSGALG00000050464		11	8.30483E-12	2.02846E-11
ENSGALG00000005087	FAM83G	14	8.31956E-12	2.03165E-11
ENSGALG00000011661	SOD2	3	8.32568E-12	2.03274E-11
ENSGALG00000009823	PTPRE	6	8.50446E-12	2.07597E-11
ENSGALG00000016893	UGGT2	1	8.56452E-12	2.09021E-11
ENSGALG00000027207	PERP2	3	8.63269E-12	2.10643E-11
ENSGALG00000013625		3	8.6415E-12	2.10816E-11
ENSGALG00000011181	FKBP14	2	8.70783E-12	2.12392E-11
ENSGALG00000049204	BLOC1S6	10	8.72117E-12	2.12674E-11
ENSGALG00000039264	SLA	2	8.72904E-12	2.12824E-11
ENSGALG00000012177	NCKAP5	7	8.7449E-12	2.13168E-11
ENSGALG00000054562		28	8.861E-12	2.15955E-11
ENSGALG00000013042	MICAL3	1	8.9687E-12	2.18536E-11
ENSGALG00000051517		4	9.07044E-12	2.20971E-11
ENSGALG00000009990	SRBD1	3	9.10949E-12	2.21878E-11
ENSGALG00000009479		2	9.16212E-12	2.23115E-11
ENSGALG00000011490	CMTM6	2	9.23448E-12	2.24832E-11
ENSGALG00000044519	COA8	5	9.39173E-12	2.28615E-11
ENSGALG00000014509	BST1	4	9.47089E-12	2.30496E-11
ENSGALG00000047356	VASN	14	9.49504E-12	2.31038E-11
ENSGALG00000012938	AMN1	1	9.51523E-12	2.31483E-11
ENSGALG00000033416	HIC2	15	9.57201E-12	2.32818E-11

ENSGALG00000008429	CRNKL1	3	9.63484E-12	2.34299E-11
ENSGALG00000017234	FOLH1	1	9.67161E-12	2.35147E-11
ENSGALG00000029247	SMARCD1	33	9.67801E-12	2.35255E-11
ENSGALG00000002803	MTA1	8	9.70594E-12	2.35887E-11
ENSGALG00000027793	SCN9A	7	9.71908E-12	2.36159E-11
ENSGALG00000016758	LIPT1	1	9.81516E-12	2.38446E-11
ENSGALG00000009981	PREPL	3	9.87776E-12	2.39919E-11
ENSGALG00000052538		2	9.94094E-12	2.41406E-11
ENSGALG00000031368	PHF12	19	1.0002E-11	2.42831E-11
ENSGALG00000027533	RNF24	4	1.00036E-11	2.42831E-11
ENSGALG00000029494	BGLAP	23	1.00186E-11	2.43148E-11
ENSGALG00000004285	HNF4A	20	1.00424E-11	2.43676E-11
ENSGALG00000012759	NUP37	1	1.02415E-11	2.48458E-11
ENSGALG00000021135	HAPLN3	10	1.03598E-11	2.51276E-11
ENSGALG00000005582	KLHL18	2	1.04859E-11	2.54285E-11
ENSGALG00000008858	MDH1	3	1.05232E-11	2.55138E-11
ENSGALG00000046507		12	1.05261E-11	2.55159E-11
ENSGALG00000028879	MRPS33	1	1.07503E-11	2.60504E-11
ENSGALG00000008381	TMEM237	7	1.07509E-11	2.60504E-11
ENSGALG00000030902		2	1.07722E-11	2.60967E-11
ENSGALG00000005553	NLGN3	4	1.0998E-11	2.66387E-11
ENSGALG00000000712	RAB29	26	1.10575E-11	2.67774E-11
ENSGALG00000022909	PTPN3	2	1.10628E-11	2.67849E-11
ENSGALG00000016890	MBNL2	1	1.12828E-11	2.73122E-11
ENSGALG00000023812	CCNI2	13	1.13501E-11	2.74695E-11
ENSGALG00000013627	SLC7A2	4	1.14096E-11	2.76082E-11
ENSGALG00000043733	TMEM50A	23	1.14216E-11	2.76316E-11
ENSGALG00000051500		33	1.15332E-11	2.78961E-11
ENSGALG00000003505	LACTB	10	1.15634E-11	2.79636E-11
ENSGALG00000016518	PHKA2	1	1.16475E-11	2.81615E-11
ENSGALG00000034975	FUT7	17	1.17436E-11	2.83882E-11
ENSGALG00000007936	PGK2	4	1.18359E-11	2.86055E-11
ENSGALG00000041812		1	1.19582E-11	2.88955E-11

ENSGALG00000034140	ZNF395	3	1.20126E-11	2.90212E-11
ENSGALG00000033356	FAHD2A	22	1.20252E-11	2.90457E-11
ENSGALG00000029540		6	1.21781E-11	2.94094E-11
ENSGALG00000004975		4	1.22351E-11	2.95411E-11
ENSGALG00000009047	YPEL5	3	1.22413E-11	2.95502E-11
ENSGALG00000011387	POLR2B	4	1.22966E-11	2.96778E-11
ENSGALG00000028520	CST3	3	1.23099E-11	2.97004E-11
ENSGALG00000029882	MYADML2	18	1.23108E-11	2.97004E-11
ENSGALG00000004375	VPS35	11	1.23272E-11	2.9734E-11
ENSGALG00000038820		2	1.2349E-11	2.97808E-11
ENSGALG00000034397		3	1.27327E-11	3.07001E-11
ENSGALG00000006513	F9	4	1.28063E-11	3.08713E-11
ENSGALG00000001480	INKA2	26	1.29518E-11	3.12159E-11
ENSGALG00000008551	CRELD2	1	1.30385E-11	3.14187E-11
ENSGALG00000030933	C1H12ORF73	1	1.30709E-11	3.14905E-11
ENSGALG00000004947	POLR3A	6	1.33797E-11	3.22282E-11
ENSGALG00000040553	SLC27A1	30	1.35628E-11	3.26628E-11
ENSGALG00000046023	NAXE	25	1.35677E-11	3.26679E-11
ENSGALG00000005781	ARFGAP1	20	1.39515E-11	3.35855E-11
ENSGALG00000022891		1	1.40455E-11	3.38051E-11
ENSGALG00000015969	FAM135A	3	1.41417E-11	3.40299E-11
ENSGALG00000010988	PALS2	2	1.41661E-11	3.40819E-11
ENSGALG00000011319	ADGRL3	4	1.41786E-11	3.41053E-11
ENSGALG00000042505	DSEL	2	1.42133E-11	3.4182E-11
ENSGALG00000013814	IMPA2	2	1.42955E-11	3.43729E-11
ENSGALG00000034630	PPIL1	26	1.4583E-11	3.50572E-11
ENSGALG00000017158	POGLUT3	1	1.46361E-11	3.5178E-11
ENSGALG00000034573	ABCF2	2	1.46872E-11	3.52939E-11
ENSGALG00000031741	PTK2	2	1.48465E-11	3.56696E-11
ENSGALG00000015836	CEP162	3	1.49242E-11	3.58492E-11
ENSGALG00000017314		1	1.49304E-11	3.5857E-11
ENSGALG00000032713	FOXA1	5	1.49748E-11	3.59565E-11
ENSGALG00000034030	L3MBTL3	3	1.49868E-11	3.59784E-11

ENSGALG00000035625	CEP192	2	1.50666E-11	3.61627E-11
ENSGALG00000006011	KIAA0753	19	1.51017E-11	3.62399E-11
ENSGALG00000007971	SUMO2	18	1.52052E-11	3.64811E-11
ENSGALG00000005257		8	1.53689E-11	3.68667E-11
ENSGALG00000003499	MAST3	28	1.54718E-11	3.71062E-11
ENSGALG00000029381		16	1.55336E-11	3.72471E-11
ENSGALG00000041008		30	1.60121E-11	3.83869E-11
ENSGALG00000033709	LTA4H	1	1.60904E-11	3.8567E-11
ENSGALG00000053244		33	1.61708E-11	3.87522E-11
ENSGALG00000037219	RNF2	8	1.62371E-11	3.89033E-11
ENSGALG00000003196	RNASE6	6	1.64161E-11	3.93244E-11
ENSGALG00000041010	WBP1L	6	1.64231E-11	3.93335E-11
ENSGALG00000013424	TULP3	1	1.64364E-11	3.93577E-11
ENSGALG00000002564	RGS4	8	1.68494E-11	4.03386E-11
ENSGALG00000023547	PCIF1	20	1.68666E-11	4.03718E-11
ENSGALG00000016325	GSTA3	3	1.69332E-11	4.05233E-11
ENSGALG00000004594	CD74	13	1.7022E-11	4.0728E-11
ENSGALG00000003130	DDX49	28	1.70358E-11	4.07528E-11
ENSGALG00000033974	HGF	1	1.70538E-11	4.07879E-11
ENSGALG00000007484	GOT1	6	1.75266E-11	4.19105E-11
ENSGALG00000038330	ATG9B	2	1.77121E-11	4.23458E-11
ENSGALG00000015190	GTPBP8	1	1.77577E-11	4.24465E-11
ENSGALG00000002623	PCOLCE2	9	1.77853E-11	4.25042E-11
ENSGALG00000008096	SUFU	6	1.78209E-11	4.25808E-11
ENSGALG00000009168	AFF2	4	1.79137E-11	4.27943E-11
ENSGALG00000015363	HJURP	1	1.80949E-11	4.32185E-11
ENSGALG00000008073	NF2	15	1.82328E-11	4.35394E-11
ENSGALG00000009253	SDE2	3	1.83855E-11	4.38955E-11
ENSGALG00000022718	FUT4	1	1.84174E-11	4.39631E-11
ENSGALG00000011889		5	1.84672E-11	4.40733E-11
ENSGALG00000046960		1	1.87432E-11	4.47232E-11
ENSGALG00000011285		1	1.87797E-11	4.48016E-11
ENSGALG00000032577	MECOM	9	1.89166E-11	4.51193E-11

ENSGALG00000013708	CYB5A	2	1.90024E-11	4.53152E-11
ENSGALG00000047208		8	1.91292E-11	4.56085E-11
ENSGALG00000009959		1	1.93178E-11	4.60493E-11
ENSGALG00000017137	GJA3	1	1.93668E-11	4.6157E-11
ENSGALG00000030054	PTPRN2	2	1.94829E-11	4.64247E-11
ENSGALG00000039967	RP11-529K1.3	21	1.95938E-11	4.66798E-11
ENSGALG00000030826	CLCN7	14	1.9617E-11	4.67258E-11
ENSGALG00000045021		2	1.96452E-11	4.67839E-11
ENSGALG00000016313	GCLC	3	1.96884E-11	4.68777E-11
ENSGALG00000000685	MAP3K14	27	1.98861E-11	4.73391E-11
ENSGALG00000026278		4	1.98914E-11	4.73425E-11
ENSGALG00000008022	TAPBP	16	1.99006E-11	4.73552E-11
ENSGALG00000034829	PDXDC1	14	2.01574E-11	4.79569E-11
ENSGALG00000008670	C8H1orf52	8	2.01719E-11	4.7982E-11
ENSGALG00000029286		4	2.03103E-11	4.83019E-11
ENSGALG00000007123		4	2.03252E-11	4.83278E-11
ENSGALG00000046656	PMVK	25	2.04316E-11	4.85714E-11
ENSGALG00000041226		4	2.06718E-11	4.91329E-11
ENSGALG00000009977	SMAD1	4	2.09389E-11	4.97579E-11
ENSGALG00000004562	HVCN1	15	2.09713E-11	4.98252E-11
ENSGALG00000005974	COL6A1	7	2.10696E-11	5.00491E-11
ENSGALG00000007753	POLL	6	2.12363E-11	5.04352E-11
ENSGALG00000011145	TRIL	2	2.14975E-11	5.10457E-11
ENSGALG00000005569	USP47	5	2.17704E-11	5.16834E-11
ENSGALG00000004097	LRRC38	21	2.17877E-11	5.17145E-11
ENSGALG00000032957	NOCT	4	2.1811E-11	5.17598E-11
ENSGALG00000008952	NOXA1	17	2.20965E-11	5.2427E-11
ENSGALG00000012208	GMFB	5	2.22957E-11	5.28895E-11
ENSGALG00000054926		11	2.23113E-11	5.29161E-11
ENSGALG00000014138	NFXL1	4	2.24864E-11	5.33211E-11
ENSGALG00000013765		3	2.26676E-11	5.37402E-11
ENSGALG00000002354	PUS1	15	2.27033E-11	5.38143E-11
ENSGALG00000042297	ANKRD13D	5	2.27951E-11	5.40214E-11

ENSGALG00000011340	ZRANB2	8	2.30145E-11	5.45308E-11
ENSGALG00000008938	DCLRE1A	6	2.32485E-11	5.50746E-11
ENSGALG00000002421	FAM213A	6	2.34393E-11	5.55159E-11
ENSGALG00000002954	MBTD1	18	2.3748E-11	5.62361E-11
ENSGALG00000011492	XRCC5	7	2.39214E-11	5.66356E-11
ENSGALG00000007490	DDX54	15	2.41052E-11	5.70597E-11
ENSGALG00000031408	SORD	10	2.4115E-11	5.70719E-11
ENSGALG00000012929	DNM1L	1	2.43072E-11	5.75156E-11
ENSGALG00000024056	CHST3	6	2.46636E-11	5.83477E-11
ENSGALG00000003645		15	2.46866E-11	5.83906E-11
ENSGALG00000016803	ST6GAL2	1	2.47687E-11	5.85735E-11
ENSGALG00000034615	ANO8	28	2.48038E-11	5.86452E-11
ENSGALG00000009995	AKAP6	5	2.54161E-11	6.00812E-11
ENSGALG00000044071	ABI3	27	2.5462E-11	6.0178E-11
ENSGALG00000050750	NRN1L	11	2.56369E-11	6.05797E-11
ENSGALG00000006775	TGM2	20	2.5799E-11	6.09509E-11
ENSGALG00000028810	CHCHD10	15	2.60555E-11	6.1545E-11
ENSGALG00000029343	TGM2	32	2.63146E-11	6.21448E-11
ENSGALG00000010174	SLC35B2	3	2.64395E-11	6.24277E-11
ENSGALG00000002486	RNF123	12	2.70126E-11	6.37686E-11
ENSGALG00000047077	SLC25A1	15	2.71333E-11	6.4041E-11
ENSGALG00000001046	DLGAP4	20	2.72861E-11	6.43894E-11
ENSGALG00000007676	EME1	18	2.75204E-11	6.49296E-11
ENSGALG00000004310	TTYH3	14	2.76899E-11	6.53169E-11
ENSGALG00000052328		2	2.77019E-11	6.53326E-11
ENSGALG00000005787	ZNF143	5	2.79879E-11	6.59942E-11
ENSGALG00000003439	PLEKHM2	21	2.83516E-11	6.68389E-11
ENSGALG00000012067	LMLN	7	2.83907E-11	6.69183E-11
ENSGALG00000046032	CD83	2	2.84073E-11	6.69406E-11
ENSGALG00000002495	CHCHD2P9	19	2.84112E-11	6.69406E-11
ENSGALG00000002029	TMEM259	28	2.86105E-11	6.73971E-11
ENSGALG00000006098	HPRT1	4	2.86686E-11	6.7521E-11
ENSGALG00000003786	BRD7	11	2.90187E-11	6.83323E-11

ENSGALG00000000599	MARCHF2	28	2.90275E-11	6.83398E-11
ENSGALG00000007905	LAMB1	1	2.90483E-11	6.83756E-11
ENSGALG00000021653	IFT22	19	2.90701E-11	6.84139E-11
ENSGALG00000007315	PSMD6	12	2.91519E-11	6.85931E-11
ENSGALG00000009846	BBS5	7	2.92861E-11	6.88954E-11
ENSGALG00000005632	IRAG1	5	2.95257E-11	6.94457E-11
ENSGALG00000037682	CCSER2	6	3.02211E-11	7.10678E-11
ENSGALG00000043034	KIFC3	11	3.04278E-11	7.154E-11
ENSGALG00000010792	AGMO	2	3.05984E-11	7.19271E-11
ENSGALG00000037540	PLEKHO1	25	3.07624E-11	7.22989E-11
ENSGALG00000005128	PTPDC1	12	3.09153E-11	7.26442E-11
ENSGALG00000015787	RNGTT	3	3.11557E-11	7.31948E-11
ENSGALG00000015073	TMEM39A	1	3.12414E-11	7.33821E-11
ENSGALG00000032191		27	3.1404E-11	7.37498E-11
ENSGALG00000052284	H2A-VIII	1	3.14527E-11	7.385E-11
ENSGALG00000027868	AMER2	1	3.15524E-11	7.40697E-11
ENSGALG00000005687	CUEDC2	6	3.16351E-11	7.42495E-11
ENSGALG00000031631	SDHAF4	3	3.177E-11	7.45519E-11
ENSGALG00000015016	SLC22A15	1	3.19862E-11	7.50448E-11
ENSGALG00000009450	DUSP10	3	3.213E-11	7.53677E-11
ENSGALG00000016379	SMS	1	3.22048E-11	7.55281E-11
ENSGALG00000011797	MTHFD1	5	3.22108E-11	7.55281E-11
ENSGALG00000005778		15	3.23034E-11	7.57306E-11
ENSGALG00000007579	APBB1IP	2	3.25855E-11	7.63775E-11
ENSGALG00000006530	TSSC4	5	3.2644E-11	7.64998E-11
ENSGALG00000011555	QKI	3	3.28755E-11	7.70276E-11
ENSGALG00000033446	COX5B	22	3.29009E-11	7.70721E-11
ENSGALG00000031862	BCL11B	5	3.33661E-11	7.81469E-11
ENSGALG00000049484		31	3.39439E-11	7.94849E-11
ENSGALG00000048401		19	3.40802E-11	7.97888E-11
ENSGALG00000007310	CEP164	24	3.48113E-11	8.14847E-11
ENSGALG00000011296		3	3.49787E-11	8.18609E-11
ENSGALG00000049176		1	3.50012E-11	8.18978E-11

ENSGALG00000050983		6	3.52796E-11	8.25334E-11
ENSGALG00000047664		3	3.52871E-11	8.25352E-11
ENSGALG00000009492	DNAJB9	1	3.5329E-11	8.26173E-11
ENSGALG00000026460		7	3.54188E-11	8.28114E-11
ENSGALG00000004893	TMEM119	15	3.55903E-11	8.31963E-11
ENSGALG00000012387	NUP43	3	3.59119E-11	8.3932E-11
ENSGALG00000016719	TNFRSF21	3	3.60456E-11	8.42283E-11
ENSGALG00000001309	SNF8	27	3.61211E-11	8.43887E-11
ENSGALG00000032371		8	3.63088E-11	8.48109E-11
ENSGALG00000003430	PMS2	14	3.72553E-11	8.7005E-11
ENSGALG00000004240	ORAI1	15	3.73052E-11	8.7105E-11
ENSGALG00000029047	TMEM204	14	3.76076E-11	8.77942E-11
ENSGALG00000009442	ETFDH	4	3.76318E-11	8.7834E-11
ENSGALG00000029664	SAMD12	2	3.7788E-11	8.81817E-11
ENSGALG00000005478	SNX7	8	3.78247E-11	8.82504E-11
ENSGALG00000014723	GABARAPL1	1	3.78756E-11	8.83523E-11
ENSGALG00000005345	CABLES2	20	3.85176E-11	8.98327E-11
ENSGALG00000002467	MTSS2	11	3.85577E-11	8.99089E-11
ENSGALG00000040269		20	3.88213E-11	9.05064E-11
ENSGALG00000015949	ASRGL1	3	3.90319E-11	9.09799E-11
ENSGALG00000003336	DAPK2	10	3.90635E-11	9.10362E-11
ENSGALG00000010847	VEGFC	4	3.92412E-11	9.14329E-11
ENSGALG00000005656	DENND6A	12	3.93717E-11	9.17193E-11
ENSGALG00000016844	RAB20	1	3.95545E-11	9.21277E-11
ENSGALG00000034918		28	3.96961E-11	9.24398E-11
ENSGALG00000038971	CFAP418	2	4.00419E-11	9.32271E-11
ENSGALG00000049794		30	4.0115E-11	9.33771E-11
ENSGALG00000051505	ZNF503	6	4.01216E-11	9.33771E-11
ENSGALG00000025759	TFDP2	9	4.03789E-11	9.3958E-11
ENSGALG00000002679	GLT1D1	15	4.07198E-11	9.47332E-11
ENSGALG00000031122	NTNG1	8	4.07702E-11	9.48324E-11
ENSGALG00000010538	TTC27	3	4.09637E-11	9.52642E-11
ENSGALG00000039872	PDLIM4	13	4.10708E-11	9.5495E-11

ENSGALG00000053457	PCDHGC5	13	4.1334E-11	9.60887E-11
ENSGALG00000029304	SMARCD2	27	4.14761E-11	9.64007E-11
ENSGALG00000053296		3	4.16127E-11	9.66998E-11
ENSGALG00000005439	ACACA	19	4.22681E-11	9.82041E-11
ENSGALG00000014011	LRMP	1	4.2324E-11	9.83153E-11
ENSGALG00000041762	STIM1	1	4.24861E-11	9.86729E-11
ENSGALG00000043618	SLC40A1	7	4.33777E-11	1.00724E-10
ENSGALG00000001848	UBE2J2	21	4.3671E-11	1.01386E-10
ENSGALG00000005012		12	4.37896E-11	1.01642E-10
ENSGALG00000029892	LMO2	5	4.41108E-11	1.02368E-10
ENSGALG00000007205	COX6A1	15	4.44487E-11	1.03133E-10
ENSGALG00000005266	BEAN1	11	4.46024E-11	1.0347E-10
ENSGALG00000005860	ACAA1	2	4.46168E-11	1.03483E-10
ENSGALG00000005205	RNFT1	19	4.46958E-11	1.03647E-10
ENSGALG00000005595	HPS4	15	4.61484E-11	1.06995E-10
ENSGALG00000029837	OST4	3	4.64498E-11	1.07673E-10
ENSGALG00000010526	OSTC	4	4.64949E-11	1.07758E-10
ENSGALG00000013964	SPATA18	4	4.69421E-11	1.08773E-10
ENSGALG00000055035		4	4.69542E-11	1.08781E-10
ENSGALG00000025811		2	4.77142E-11	1.1052E-10
ENSGALG00000000476	LRRTM4	22	4.79961E-11	1.11152E-10
ENSGALG00000035047		3	4.81493E-11	1.11481E-10
ENSGALG00000005046	SHMT1	14	4.81563E-11	1.11481E-10
ENSGALG00000039048	ZNF282	2	4.81773E-11	1.11508E-10
ENSGALG00000031700	TRPS1	2	4.82003E-11	1.1154E-10
ENSGALG00000007080		13	4.82487E-11	1.11631E-10
ENSGALG00000003260	UTP6	18	4.8562E-11	1.12335E-10
ENSGALG00000042411	GASK1B	4	4.86814E-11	1.1259E-10
ENSGALG00000012639	MTERF2	1	4.89261E-11	1.13134E-10
ENSGALG00000043451	DNAL4	1	4.91436E-11	1.13615E-10
ENSGALG00000001051		23	4.93565E-11	1.14086E-10
ENSGALG00000016864	METTL21C	1	4.94346E-11	1.14245E-10
ENSGALG00000010370	MED12L	9	4.94509E-11	1.14261E-10

ENSGALG00000004435	RAB27A	10	4.95711E-11	1.14517E-10
ENSGALG00000006871	SLCO3A1	10	4.97082E-11	1.14812E-10
ENSGALG00000009410	PEX1	2	4.97965E-11	1.14994E-10
ENSGALG00000048866	MARK2	33	5.0645E-11	1.16931E-10
ENSGALG00000007326	PMM2	14	5.07779E-11	1.17216E-10
ENSGALG00000022991	SMIM20	4	5.09681E-11	1.17633E-10
ENSGALG00000000707	PSMD7	11	5.14628E-11	1.18752E-10
ENSGALG00000012045		7	5.15944E-11	1.19033E-10
ENSGALG00000007161	LRRK2	1	5.1723E-11	1.19307E-10
ENSGALG00000003727	HAUS8	28	5.19616E-11	1.19835E-10
ENSGALG00000055053	PRSS35	3	5.19831E-11	1.19862E-10
ENSGALG00000004017	TOR1AIP2	8	5.20679E-11	1.20035E-10
ENSGALG00000006180	SLC9A6	4	5.23322E-11	1.20621E-10
ENSGALG00000016419	ASAP2	3	5.23685E-11	1.20682E-10
ENSGALG00000036117	TENT5B	23	5.30473E-11	1.22223E-10
ENSGALG00000032595	NDUFB2	1	5.30829E-11	1.22282E-10
ENSGALG00000017142	PSPC1	1	5.31246E-11	1.22355E-10
ENSGALG00000025822	CYP1B1	3	5.32346E-11	1.22585E-10
ENSGALG00000005531	MRGBP	20	5.32697E-11	1.22643E-10
ENSGALG00000000478	TANC2	27	5.33333E-11	1.22766E-10
ENSGALG00000045721	ISG20L2	25	5.3393E-11	1.22881E-10
ENSGALG00000016421	ITGB1BP1	3	5.3627E-11	1.23396E-10
ENSGALG00000031112	COL5A2	7	5.38562E-11	1.239E-10
ENSGALG00000012429	BMP4	5	5.47211E-11	1.25866E-10
ENSGALG00000032765		16	5.47702E-11	1.25955E-10
ENSGALG00000008255	C11orf49	5	5.52016E-11	1.26923E-10
ENSGALG00000007234	CLCN5	4	5.52657E-11	1.27046E-10
ENSGALG00000007608	ARHGEF9	4	5.56766E-11	1.27964E-10
ENSGALG00000008272	TMED3	10	5.56856E-11	1.27964E-10
ENSGALG00000048830	EIF4EBP3	13	5.59524E-11	1.28552E-10
ENSGALG00000047380		16	5.60233E-11	1.28691E-10
ENSGALG00000036284	RPGR	1	5.80644E-11	1.33354E-10
ENSGALG00000003969	EMC1	21	5.85524E-11	1.3445E-10

ENSGALG00000010031	ARHGAP10	4	5.89588E-11	1.35358E-10
ENSGALG00000002278	UNK	18	5.93116E-11	1.36142E-10
ENSGALG00000050359		27	5.94837E-11	1.36511E-10
ENSGALG00000037860		22	5.963E-11	1.36801E-10
ENSGALG00000003566	SMURF2	18	5.96326E-11	1.36801E-10
ENSGALG00000029970	LIMS1	1	6.03942E-11	1.38523E-10
ENSGALG00000007609	ARHGAP19	6	6.04965E-11	1.38731E-10
ENSGALG00000038757	RBMS2	33	6.16283E-11	1.413E-10
ENSGALG00000030007		26	6.17951E-11	1.41656E-10
ENSGALG00000054150	GPR27	12	6.25516E-11	1.43363E-10
ENSGALG00000031284	PTPRM	2	6.51564E-11	1.49305E-10
ENSGALG00000003939	KLF2	28	6.56738E-11	1.50462E-10
ENSGALG00000032628	SRCIN1	27	6.57189E-11	1.50537E-10
ENSGALG00000000629		28	6.60233E-11	1.51206E-10
ENSGALG00000043971		13	6.78556E-11	1.55373E-10
ENSGALG00000041732	NOL4L	20	6.83229E-11	1.56414E-10
ENSGALG00000013742	EZR	3	6.84961E-11	1.56781E-10
ENSGALG00000006756		20	6.94165E-11	1.58858E-10
ENSGALG00000037904	EIF4EBP2	6	7.05546E-11	1.61432E-10
ENSGALG00000052302		8	7.1345E-11	1.6321E-10
ENSGALG00000000461	MRC2	27	7.15603E-11	1.63672E-10
ENSGALG00000011118	MIER1	8	7.19134E-11	1.64449E-10
ENSGALG00000005675		9	7.19614E-11	1.64527E-10
ENSGALG00000014976	GATA6	2	7.22179E-11	1.65083E-10
ENSGALG00000032418	RAB26	14	7.22491E-11	1.65123E-10
ENSGALG00000006566	TBCCD1	9	7.2679E-11	1.66075E-10
ENSGALG00000032590	NDE1	14	7.29707E-11	1.6671E-10
ENSGALG00000048496		4	7.32647E-11	1.6735E-10
ENSGALG00000008759	LAMP3	9	7.54632E-11	1.7234E-10
ENSGALG00000012472	IL2RB	1	7.60893E-11	1.73737E-10
ENSGALG00000031388	KLHL5	4	7.71871E-11	1.76211E-10
ENSGALG00000004115	DNAJA2	11	7.77714E-11	1.77511E-10
ENSGALG00000051088		16	7.85333E-11	1.79217E-10

ENSGALG00000014166	TSPO	1	7.95066E-11	1.81404E-10
ENSGALG00000016843	COL4A2	1	7.96777E-11	1.8176E-10
ENSGALG00000017357	SRSF2	18	7.97118E-11	1.81804E-10
ENSGALG00000035973	YIPF6	4	7.97965E-11	1.81964E-10
ENSGALG00000032420	DPAGT1	24	8.11942E-11	1.85116E-10
ENSGALG00000043754	GLUL	8	8.1297E-11	1.85316E-10
ENSGALG00000017064	RFC3	1	8.14249E-11	1.85573E-10
ENSGALG00000046098		5	8.15275E-11	1.85772E-10
ENSGALG00000006132	MOSPD1	4	8.18391E-11	1.86447E-10
ENSGALG00000002479	MAT1A	6	8.22937E-11	1.87448E-10
ENSGALG00000028691	MPG	14	8.26955E-11	1.88328E-10
ENSGALG00000012747	NEDD9	2	8.35363E-11	1.90207E-10
ENSGALG00000035194	ARHGAP25	22	8.56145E-11	1.94902E-10
ENSGALG00000008013	KLHDC10	1	8.69659E-11	1.97942E-10
ENSGALG00000048671		13	8.84122E-11	2.01196E-10
ENSGALG00000017173	GUCY1A2	1	8.84994E-11	2.01357E-10
ENSGALG00000003120	NCOR2	15	8.92838E-11	2.03104E-10
ENSGALG00000012165	DLGAP5	5	8.95283E-11	2.03622E-10
ENSGALG00000015959	SMAP1	3	9.03141E-11	2.05371E-10
ENSGALG00000016710	CYP39A1	3	9.04079E-11	2.05546E-10
ENSGALG00000010093	BRMS1L	5	9.07764E-11	2.06345E-10
ENSGALG00000039212	NDUFA13	28	9.10277E-11	2.06878E-10
ENSGALG00000010204	KCNC2	1	9.16702E-11	2.083E-10
ENSGALG00000007170	SNRPA1	10	9.20538E-11	2.09132E-10
ENSGALG00000008201	FBXO21	15	9.29717E-11	2.11178E-10
ENSGALG00000049288		2	9.30781E-11	2.1138E-10
ENSGALG00000002906	TARDBP	21	9.44448E-11	2.14444E-10
ENSGALG00000011347	IHH	7	9.48952E-11	2.15412E-10
ENSGALG00000009747	ATP23	1	9.49064E-11	2.15412E-10
ENSGALG00000010025	SNX32	5	9.54125E-11	2.1652E-10
ENSGALG00000010976	CCDC126	2	9.81154E-11	2.22613E-10
ENSGALG00000036111	ITM2C	9	9.83896E-11	2.23193E-10
ENSGALG00000011213	DEGS2	5	9.93238E-11	2.25271E-10

ENSGALG00000014485	LDB2	4	1.00541E-10	2.27989E-10
ENSGALG00000014964	NT5DC1	3	1.01278E-10	2.29619E-10
ENSGALG00000026229	PIGY	4	1.01513E-10	2.30108E-10
ENSGALG00000039528	PLCE1	6	1.01564E-10	2.30179E-10
ENSGALG00000008713	BIRC5	3	1.01592E-10	2.302E-10
ENSGALG00000038043	HECTD2	6	1.01961E-10	2.30995E-10
ENSGALG00000040418	DGAT2	1	1.021E-10	2.31266E-10
ENSGALG00000051479	CDK5	2	1.02892E-10	2.33017E-10
ENSGALG00000038710	MANSC1	1	1.03353E-10	2.34017E-10
ENSGALG00000043163	MSANTD2	24	1.0342E-10	2.34125E-10
ENSGALG00000011682	CNOT4	1	1.04199E-10	2.35845E-10
ENSGALG00000003745	NECAP2	21	1.04508E-10	2.36502E-10
ENSGALG00000007358	CILP	10	1.0462E-10	2.36711E-10
ENSGALG00000040519	PEF1	23	1.05322E-10	2.38254E-10
ENSGALG00000053916	ABCB1	2	1.05843E-10	2.39388E-10
ENSGALG00000010641	SCCPDH	3	1.06064E-10	2.39844E-10
ENSGALG00000053964	IMAP1-GIMAI	2	1.06665E-10	2.41158E-10
ENSGALG00000008834		8	1.07337E-10	2.42632E-10
ENSGALG00000046867	GET3	30	1.07768E-10	2.43562E-10
ENSGALG00000053035		14	1.08389E-10	2.44919E-10
ENSGALG00000001073	NR6A1	17	1.11059E-10	2.50905E-10
ENSGALG00000012462	KIF5C	7	1.12496E-10	2.54105E-10
ENSGALG00000047135		1	1.13488E-10	2.56298E-10
ENSGALG00000047215		11	1.1464E-10	2.58853E-10
ENSGALG00000032496	SIRT3	5	1.14965E-10	2.59539E-10
ENSGALG00000049843		31	1.16536E-10	2.63037E-10
ENSGALG00000005670	SLMAP	12	1.16577E-10	2.6308E-10
ENSGALG00000002189	SNX1	10	1.19241E-10	2.69041E-10
ENSGALG00000035816	FDXR	18	1.19649E-10	2.69912E-10
ENSGALG00000000104		26	1.21135E-10	2.73215E-10
ENSGALG00000016895	DZIP1	1	1.21327E-10	2.73596E-10
ENSGALG00000009179	TNFSF10	9	1.22592E-10	2.76399E-10
ENSGALG00000014795	TGIF1	2	1.22694E-10	2.76578E-10

ENSGALG00000035187	FHL3	23	1.23529E-10	2.78408E-10
ENSGALG00000013660	ZNF516	2	1.23705E-10	2.78754E-10
ENSGALG00000012287	ATP5S	5	1.24648E-10	2.80826E-10
ENSGALG00000038494	CMIP	11	1.25281E-10	2.822E-10
ENSGALG00000037888	TBXA2R	28	1.26064E-10	2.83912E-10
ENSGALG00000019290	SLC6A12	1	1.27155E-10	2.86315E-10
ENSGALG00000026328	MIEN1	27	1.28601E-10	2.89519E-10
ENSGALG00000029445	FADS6	18	1.28915E-10	2.90171E-10
ENSGALG00000016420	SH3KBP1	1	1.29351E-10	2.911E-10
ENSGALG00000016759	MITD1	1	1.30235E-10	2.93035E-10
ENSGALG00000021362	GPRC5C	18	1.30847E-10	2.94358E-10
ENSGALG00000053730		16	1.3311E-10	2.99394E-10
ENSGALG00000008949	ADRB1	6	1.35177E-10	3.03987E-10
ENSGALG00000051203		5	1.3557E-10	3.04814E-10
ENSGALG00000001264	PLXNA2	26	1.35967E-10	3.05649E-10
ENSGALG00000026396	BG1	16	1.36258E-10	3.06248E-10
ENSGALG00000011506	NUP54	4	1.36519E-10	3.06779E-10
ENSGALG00000047403	PIH1D2	24	1.3714E-10	3.08116E-10
ENSGALG00000013777		3	1.38038E-10	3.10077E-10
ENSGALG00000008727		5	1.39152E-10	3.12523E-10
ENSGALG00000030002	KRT18	33	1.40484E-10	3.15454E-10
ENSGALG00000003146	CCDC92	15	1.41054E-10	3.16678E-10
ENSGALG00000016456	LPIN1	3	1.41983E-10	3.18704E-10
ENSGALG00000032664	UBE2B	13	1.42788E-10	3.20452E-10
ENSGALG00000004273	RALGPS2	8	1.43035E-10	3.20946E-10
ENSGALG00000005065	PLA2G4A	8	1.43134E-10	3.21111E-10
ENSGALG00000044985		28	1.43368E-10	3.21576E-10
ENSGALG00000011286	UBE2E1	2	1.45844E-10	3.27069E-10
ENSGALG00000055081	SEMA4C	22	1.46358E-10	3.28161E-10
ENSGALG00000012186	CASP6	4	1.4782E-10	3.31378E-10
ENSGALG00000033286	ARSJ	4	1.48304E-10	3.32402E-10
ENSGALG00000034067	FMOD	26	1.49919E-10	3.3596E-10
ENSGALG00000014962	CALHM6	3	1.5E-10	3.3608E-10

ENSGALG00000032922	GSTM2	26	1.51168E-10	3.38635E-10
ENSGALG00000017365	REEP2	13	1.51487E-10	3.39288E-10
ENSGALG00000047125		5	1.51855E-10	3.40049E-10
ENSGALG00000016443	HPCAL1	3	1.52052E-10	3.40428E-10
ENSGALG00000031606	NDC1	8	1.5275E-10	3.41929E-10
ENSGALG00000010770	SCIN	2	1.5327E-10	3.43029E-10
ENSGALG00000006864		8	1.53851E-10	3.44265E-10
ENSGALG00000010854		2	1.54719E-10	3.46144E-10
ENSGALG00000047011		20	1.56545E-10	3.50166E-10
ENSGALG00000009275	TMEM63A	3	1.57908E-10	3.53149E-10
ENSGALG00000008736	PNPLA7	17	1.57968E-10	3.53218E-10
ENSGALG00000017200	MTMR2	1	1.59663E-10	3.56943E-10
ENSGALG00000041384	SHKBP1	32	1.60883E-10	3.59605E-10
ENSGALG00000028940	NUDT14	5	1.62298E-10	3.62701E-10
ENSGALG00000005961	DGUOK	13	1.62397E-10	3.62857E-10
ENSGALG00000036659	NDUFAF8	18	1.63167E-10	3.64454E-10
ENSGALG00000012529	EIF3D	1	1.63172E-10	3.64454E-10
ENSGALG00000014203	PARVG	1	1.63568E-10	3.65271E-10
ENSGALG00000007555	CCND1	5	1.64637E-10	3.67592E-10
ENSGALG00000035850	CSAD	33	1.66031E-10	3.70637E-10
ENSGALG00000002536	STX2	15	1.67264E-10	3.7332E-10
ENSGALG00000053220		2	1.67929E-10	3.74737E-10
ENSGALG00000004282	RCAN3	23	1.67964E-10	3.74746E-10
ENSGALG00000030038	C3	30	1.68356E-10	3.75551E-10
ENSGALG00000005474	PNAT10	11	1.68506E-10	3.75818E-10
ENSGALG00000043018	DLG5	6	1.68883E-10	3.76589E-10
ENSGALG00000015688	MXD4	4	1.71345E-10	3.8201E-10
ENSGALG00000008676	RRBP1	3	1.72186E-10	3.83815E-10
ENSGALG00000004714	CPSF4	14	1.72938E-10	3.85419E-10
ENSGALG00000048015		1	1.73293E-10	3.86141E-10
ENSGALG00000014413	CCDC58	1	1.73635E-10	3.86832E-10
ENSGALG00000000693	NOB1	11	1.73826E-10	3.87187E-10
ENSGALG00000003560	SLC6A2	11	1.7476E-10	3.89196E-10

ENSGALG00000010110	TMEM53	8	1.75886E-10	3.91632E-10
ENSGALG00000026363	FIGNL1	2	1.76088E-10	3.9201E-10
ENSGALG00000014213	PRR5	1	1.77626E-10	3.95362E-10
ENSGALG00000005769	WEE1	5	1.7785E-10	3.95788E-10
ENSGALG00000024041	FAHD1	14	1.78216E-10	3.9653E-10
ENSGALG00000000871	ART1	1	1.79577E-10	3.99486E-10
ENSGALG00000049891	GPD1	33	1.80382E-10	4.01203E-10
ENSGALG00000051459		22	1.81302E-10	4.03176E-10
ENSGALG00000016986	LCP1	1	1.84105E-10	4.09334E-10
ENSGALG00000025738	RHOU	3	1.88156E-10	4.18265E-10
ENSGALG00000039354	VTG1	8	1.88551E-10	4.19067E-10
ENSGALG00000008780	CTBS	8	1.9042E-10	4.23143E-10
ENSGALG00000010857	DAB1	8	1.91441E-10	4.25336E-10
ENSGALG00000003344	CLTB	13	1.91791E-10	4.26034E-10
ENSGALG00000007324	TP63	9	1.94267E-10	4.31457E-10
ENSGALG00000043006	PDIA4	2	1.94396E-10	4.31665E-10
ENSGALG00000047821		1	1.94896E-10	4.32695E-10
ENSGALG00000031518		33	1.95519E-10	4.34001E-10
ENSGALG00000041140	HTRA1	6	1.95756E-10	4.34447E-10
ENSGALG00000046125	PECAM1	18	1.97968E-10	4.39263E-10
ENSGALG00000036787	HSD17B12	5	1.97998E-10	4.39263E-10
ENSGALG00000052151		28	1.98656E-10	4.40642E-10
ENSGALG00000002667	OLFML2B	8	1.99339E-10	4.42078E-10
ENSGALG00000006899		24	1.99474E-10	4.42297E-10
ENSGALG00000035733	CCR7	27	2.00983E-10	4.45554E-10
ENSGALG00000037253		30	2.01017E-10	4.45554E-10
ENSGALG00000009015	PIGK	8	2.01453E-10	4.46406E-10
ENSGALG00000002362	MANF	12	2.01474E-10	4.46406E-10
ENSGALG00000033885	RAP1GAP2	19	2.02062E-10	4.47628E-10
ENSGALG00000030703	CYBRD1	7	2.02339E-10	4.4816E-10
ENSGALG00000052753		9	2.08252E-10	4.61172E-10
ENSGALG00000007744	CASS4	20	2.08858E-10	4.62431E-10
ENSGALG00000007768	AURKA	20	2.10569E-10	4.66134E-10

ENSGALG00000034118	SOX18	20	2.1435E-10	4.74418E-10
ENSGALG00000000608	CDH1	11	2.1702E-10	4.80241E-10
ENSGALG00000014331	FKBP4	1	2.17213E-10	4.8058E-10
ENSGALG00000030836	PSD2	13	2.17921E-10	4.82059E-10
ENSGALG00000034346	RDH10	2	2.18246E-10	4.82691E-10
ENSGALG00000027501	ANPEP	10	2.19591E-10	4.85577E-10
ENSGALG00000026846	JMJD7	5	2.20793E-10	4.88146E-10
ENSGALG00000004293		3	2.2106E-10	4.88649E-10
ENSGALG00000006489	CHCHD3	1	2.21962E-10	4.90553E-10
ENSGALG00000009067	GPR50	4	2.23696E-10	4.94297E-10
ENSGALG00000054235		3	2.24026E-10	4.94934E-10
ENSGALG00000026970		5	2.24601E-10	4.96117E-10
ENSGALG00000043074		5	2.2743E-10	5.02273E-10
ENSGALG00000002397	PSPH	19	2.27605E-10	5.0257E-10
ENSGALG00000047857		21	2.29637E-10	5.06964E-10
ENSGALG00000023521	ADRA2A	6	2.34046E-10	5.16605E-10
ENSGALG00000042853	GGPS1	3	2.35183E-10	5.19021E-10
ENSGALG00000013738	OPTN	1	2.35813E-10	5.20317E-10
ENSGALG00000001563	WDR45B	18	2.37306E-10	5.23516E-10
ENSGALG00000036044		5	2.38052E-10	5.25067E-10
ENSGALG00000045555	IVNS1ABP	8	2.39054E-10	5.27182E-10
ENSGALG00000003059	HLF	18	2.4038E-10	5.30009E-10
ENSGALG00000009825	SRGAP1	1	2.40956E-10	5.31184E-10
ENSGALG00000001124	LHX2	17	2.41469E-10	5.32219E-10
ENSGALG00000017362	HAVCR1	13	2.43307E-10	5.36173E-10
ENSGALG00000014828	ECHDC1	3	2.44507E-10	5.38719E-10
ENSGALG00000027633	RASSF9	1	2.45056E-10	5.39832E-10
ENSGALG00000007700	CTTN	5	2.45771E-10	5.4131E-10
ENSGALG00000029268	CIAPIN1	11	2.4681E-10	5.43498E-10
ENSGALG00000005654	PITPNB	15	2.48176E-10	5.46408E-10
ENSGALG00000037361	KLF8	4	2.50374E-10	5.51149E-10
ENSGALG00000010749	TCEANC2	8	2.50673E-10	5.51707E-10
ENSGALG00000035950	NGFR	27	2.51925E-10	5.54363E-10

ENSGALG00000014970	FSTL1	1	2.5251E-10	5.5555E-10
ENSGALG00000028709	RNF144A	3	2.53129E-10	5.5681E-10
ENSGALG00000009128		4	2.54163E-10	5.58985E-10
ENSGALG00000035980	REXO2	24	2.58726E-10	5.68916E-10
ENSGALG00000011000	TARBP1	3	2.64597E-10	5.81722E-10
ENSGALG00000040606	RAC3	18	2.66363E-10	5.855E-10
ENSGALG00000016185	SLC37A1	1	2.67604E-10	5.88122E-10
ENSGALG00000012712	RBM24	2	2.70556E-10	5.94503E-10
ENSGALG00000052156		4	2.73959E-10	6.01871E-10
ENSGALG00000014585		19	2.76079E-10	6.06418E-10
ENSGALG00000017002	FNDC3A	1	2.77913E-10	6.10336E-10
ENSGALG00000006418	PDPK1	14	2.82625E-10	6.20574E-10
ENSGALG00000016898	TGDS	1	2.82973E-10	6.21225E-10
ENSGALG00000053833	C1orf210	8	2.89644E-10	6.35757E-10
ENSGALG00000037129	FAM241A	4	2.90295E-10	6.3707E-10
ENSGALG00000000927	ANGPTL2	17	2.91649E-10	6.39927E-10
ENSGALG00000053703		2	2.94026E-10	6.45026E-10
ENSGALG00000010567	RAB3B	8	2.94296E-10	6.45503E-10
ENSGALG00000010560	EIF2AK2	3	2.95483E-10	6.47989E-10
ENSGALG00000026263	RGS8	8	2.95992E-10	6.4899E-10
ENSGALG00000004112	FUCA1	23	3.00233E-10	6.58169E-10
ENSGALG00000012219	UNC5C	4	3.05027E-10	6.68559E-10
ENSGALG00000000071		1	3.0829E-10	6.7559E-10
ENSGALG00000040793	KDELR2	14	3.1507E-10	6.90323E-10
ENSGALG00000002618	KCTD7	19	3.15304E-10	6.90712E-10
ENSGALG00000041030	PCBP2	33	3.18627E-10	6.97866E-10
ENSGALG00000007478	SLC51A	9	3.19443E-10	6.99526E-10
ENSGALG00000008113	PLCL1	7	3.20362E-10	7.01415E-10
ENSGALG00000042500		31	3.21183E-10	7.03084E-10
ENSGALG00000008313	NAPB	3	3.24649E-10	7.10544E-10
ENSGALG00000011457	CCNI	4	3.25822E-10	7.12984E-10
ENSGALG00000030552		2	3.26116E-10	7.135E-10
ENSGALG00000035429	XKR4	2	3.27426E-10	7.16237E-10

ENSGALG00000015362	TRAT1	1	3.28007E-10	7.1738E-10
ENSGALG00000010737	PLD5	3	3.29719E-10	7.20995E-10
ENSGALG00000037414	DNAJC28	1	3.29794E-10	7.2103E-10
ENSGALG00000035007	USP35	1	3.33009E-10	7.27926E-10
ENSGALG00000008969	EPCAM	3	3.34257E-10	7.30523E-10
ENSGALG00000022531		5	3.3497E-10	7.31952E-10
ENSGALG00000025948		20	3.36959E-10	7.36166E-10
ENSGALG00000004960	FBN1	10	3.47874E-10	7.59875E-10
ENSGALG00000004997	FLII	14	3.52111E-10	7.68993E-10
ENSGALG00000002519	SLC25A33	21	3.52378E-10	7.69439E-10
ENSGALG00000038189		28	3.55968E-10	7.77139E-10
ENSGALG00000026989	HOXA7	2	3.59512E-10	7.84735E-10
ENSGALG00000017073	BRCA2	1	3.61582E-10	7.89113E-10
ENSGALG00000024482	LEPROTL1	4	3.6806E-10	8.03105E-10
ENSGALG00000053682		16	3.68173E-10	8.03208E-10
ENSGALG00000040084	MRS2	2	3.68671E-10	8.04151E-10
ENSGALG00000007510	CETN1	4	3.71152E-10	8.09418E-10
ENSGALG00000011861	SYT16	5	3.72976E-10	8.1325E-10
ENSGALG00000047495	LRRC10	1	3.73169E-10	8.13526E-10
ENSGALG00000033091	RUNX1T1	2	3.7337E-10	8.13819E-10
ENSGALG00000002263	KLHL6	9	3.73892E-10	8.14811E-10
ENSGALG00000033497	FOXP4	26	3.75199E-10	8.17513E-10
ENSGALG00000009628		4	3.80443E-10	8.28763E-10
ENSGALG00000008015	UBE2V1	20	3.80498E-10	8.28763E-10
ENSGALG00000025773	SMIM29	26	3.80975E-10	8.29653E-10
ENSGALG00000030291	IRF9	20	3.82405E-10	8.32619E-10
ENSGALG00000002987	SERPINF2	19	3.85862E-10	8.39995E-10
ENSGALG00000004190	RNF34	15	3.90148E-10	8.49173E-10
ENSGALG00000002123	OSCP1	23	3.92516E-10	8.54176E-10
ENSGALG00000054339		7	3.93652E-10	8.56495E-10
ENSGALG00000000901		11	3.94837E-10	8.58921E-10
ENSGALG00000032836		1	3.94981E-10	8.5908E-10
ENSGALG00000009241	SFRP2	4	3.95989E-10	8.61119E-10

ENSGALG00000014258		4	3.96354E-10	8.61758E-10
ENSGALG00000000436	RABIF	26	3.98584E-10	8.66453E-10
ENSGALG00000012619	PRDM4	1	4.02083E-10	8.73904E-10
ENSGALG00000000533	SRSF3	26	4.0469E-10	8.79412E-10
ENSGALG00000006544		12	4.06847E-10	8.83943E-10
ENSGALG00000008618	UPF3B	4	4.14621E-10	9.00672E-10
ENSGALG00000011254	SATB1	2	4.16657E-10	9.04933E-10
ENSGALG00000013503	FAM149A	4	4.19174E-10	9.10239E-10
ENSGALG00000030789	PGAP1	7	4.24526E-10	9.21696E-10
ENSGALG00000011393	ZNF142	7	4.25129E-10	9.22841E-10
ENSGALG00000003345		27	4.28902E-10	9.30864E-10
ENSGALG00000006937	P4HTM	12	4.32768E-10	9.39088E-10
ENSGALG00000035911	CBX4	18	4.34868E-10	9.43477E-10
ENSGALG00000034373	KATNB1	11	4.36366E-10	9.46558E-10
ENSGALG00000009207	TRIM2	4	4.37668E-10	9.49215E-10
ENSGALG00000011237	DPH3P1	2	4.37843E-10	9.49426E-10
ENSGALG00000016592	PRPS2	1	4.38824E-10	9.51215E-10
ENSGALG00000012396	PTGER2	5	4.38824E-10	9.51215E-10
ENSGALG00000030603	ANTXR1	22	4.41025E-10	9.55816E-10
ENSGALG00000014791	WDR3	1	4.41536E-10	9.56753E-10
ENSGALG00000000997	GJC3	27	4.41708E-10	9.56955E-10
ENSGALG00000008404		4	4.43264E-10	9.60156E-10
ENSGALG00000001204	ENKD1	11	4.47153E-10	9.68409E-10
ENSGALG00000037051		19	4.52615E-10	9.80062E-10
ENSGALG00000030530	HAND2	4	4.56493E-10	9.88285E-10
ENSGALG00000006096	RPS13	5	4.57049E-10	9.89312E-10
ENSGALG00000006459	CCDC174	12	4.65437E-10	1.00729E-09
ENSGALG00000003924	ACBD6	8	4.77074E-10	1.03229E-09
ENSGALG00000009587	GPHN	5	4.85427E-10	1.05018E-09
ENSGALG00000010022	PRMT9	4	4.85913E-10	1.05105E-09
ENSGALG00000015313	PDSS2	3	4.90254E-10	1.06025E-09
ENSGALG00000044662		33	4.94559E-10	1.06937E-09
ENSGALG00000027416	ROMO1	20	4.95746E-10	1.07174E-09

ENSGALG00000004882	PPCS	21	4.98113E-10	1.07667E-09
ENSGALG00000052702		9	4.98979E-10	1.07835E-09
ENSGALG00000037839	ARFIP2	1	5.03004E-10	1.08686E-09
ENSGALG00000039525		21	5.07049E-10	1.0954E-09
ENSGALG00000004372	MICU1	6	5.08176E-10	1.09764E-09
ENSGALG00000030455	JAZF1	2	5.12802E-10	1.10744E-09
ENSGALG00000034281	TMCC2	26	5.1298E-10	1.10763E-09
ENSGALG00000035504	ETV1	2	5.1533E-10	1.1125E-09
ENSGALG00000001606	INPP5B	23	5.16267E-10	1.11433E-09
ENSGALG00000012792	HIPK2	1	5.17373E-10	1.11652E-09
ENSGALG00000016164	ABCG1	1	5.18892E-10	1.1196E-09
ENSGALG00000009576	BPNT1	3	5.20368E-10	1.12258E-09
ENSGALG00000004528	TSHZ3	11	5.29013E-10	1.14103E-09
ENSGALG00000006327	FBXL21	13	5.29419E-10	1.14171E-09
ENSGALG00000043287	CAV1	1	5.31829E-10	1.1467E-09
ENSGALG00000054435		4	5.31974E-10	1.14681E-09
ENSGALG00000012998	WNT5B	1	5.35577E-10	1.15437E-09
ENSGALG00000030548	OSBP	5	5.38828E-10	1.16117E-09
ENSGALG00000002832	IFI35	27	5.40164E-10	1.16385E-09
ENSGALG00000015219		2	5.4162E-10	1.16678E-09
ENSGALG00000042651	KRIT1	2	5.44143E-10	1.17201E-09
ENSGALG00000015134	APOV1	1	5.56331E-10	1.19805E-09
ENSGALG00000009024	CLIP4	3	5.57406E-10	1.20015E-09
ENSGALG00000035978	CDCA7L	2	5.62067E-10	1.20997E-09
ENSGALG00000009427	TIAL1	6	5.6327E-10	1.21235E-09
ENSGALG00000026653	ARL9	4	5.65701E-10	1.21737E-09
ENSGALG00000002129	WDR47	8	5.68572E-10	1.22333E-09
ENSGALG00000004348	BRAT1	14	5.7007E-10	1.22633E-09
ENSGALG00000010900	SPC25	7	5.70692E-10	1.22745E-09
ENSGALG00000001304		21	5.76471E-10	1.23967E-09
ENSGALG00000000550	STRADA	27	5.77836E-10	1.24238E-09
ENSGALG00000005450	CCDC66	12	5.78832E-10	1.24431E-09
ENSGALG00000054787		2	5.84272E-10	1.25578E-09

ENSGALG00000004270	ALDH1A2	10	5.85923E-10	1.2591E-09
ENSGALG00000022875		2	5.87307E-10	1.26186E-09
ENSGALG00000032260	IL10RA	24	5.8759E-10	1.26224E-09
ENSGALG00000007438	IFT46	24	5.88991E-10	1.26503E-09
ENSGALG00000011497	PECR	7	5.92497E-10	1.27233E-09
ENSGALG00000043681	DAPP1	4	6.0009E-10	1.28841E-09
ENSGALG00000044521		31	6.06067E-10	1.30102E-09
ENSGALG00000001409	IL12B	13	6.07187E-10	1.30319E-09
ENSGALG00000026231	ARID3A	28	6.09227E-10	1.30734E-09
ENSGALG00000008835	THAP10	8	6.13678E-10	1.31666E-09
ENSGALG00000028336	MED10	2	6.21278E-10	1.33273E-09
ENSGALG00000007473	MRPL28	14	6.22675E-10	1.33549E-09
ENSGALG00000043570	SGK3	2	6.30829E-10	1.35274E-09
ENSGALG00000001058	ARHGEF16	21	6.34281E-10	1.35991E-09
ENSGALG00000038745	LACTB2	2	6.39297E-10	1.37042E-09
ENSGALG00000006219	PRKAG2	2	6.40842E-10	1.37349E-09
ENSGALG00000054931		33	6.4765E-10	1.38784E-09
ENSGALG00000015356	IFT57	1	6.52914E-10	1.39887E-09
ENSGALG00000014455	LPAR5	1	6.56185E-10	1.40563E-09
ENSGALG00000002229	FBXW11	13	6.64935E-10	1.42413E-09
ENSGALG00000013916	HYPK	10	6.67226E-10	1.42878E-09
ENSGALG00000000424	CMTM3	11	6.69332E-10	1.43304E-09
ENSGALG00000001103	PSMB7	17	6.7958E-10	1.45472E-09
ENSGALG00000029330		32	6.82913E-10	1.46161E-09
ENSGALG00000003742	CPAMD8	28	6.88139E-10	1.47253E-09
ENSGALG00000033569		21	6.92075E-10	1.48069E-09
ENSGALG00000012640	TMEM263	1	6.95462E-10	1.48768E-09
ENSGALG00000007084	DUSP12	1	6.96771E-10	1.49022E-09
ENSGALG00000001006	TP73	21	6.99644E-10	1.4961E-09
ENSGALG00000005907	RPN1	12	7.02633E-10	1.50223E-09
ENSGALG00000011206	KITLG	1	7.03889E-10	1.50465E-09
ENSGALG00000009195	MCM8	3	7.07375E-10	1.51184E-09
ENSGALG00000000998	MTFR1L	23	7.22066E-10	1.54296E-09

ENSGALG00000023468	VPS9D1	11	7.28218E-10	1.55584E-09
ENSGALG00000003458	CCZ1	14	7.35195E-10	1.57047E-09
ENSGALG00000002445	KIAA0319L	23	7.39592E-10	1.57958E-09
ENSGALG00000008905	HABP2	6	7.40829E-10	1.58195E-09
ENSGALG00000002458	VAC14	11	7.47246E-10	1.59537E-09
ENSGALG00000043772		27	7.50435E-10	1.6019E-09
ENSGALG00000054212		6	7.52501E-10	1.60603E-09
ENSGALG00000007792	PFDN4	20	7.58179E-10	1.61786E-09
ENSGALG00000007059	IGSF6	14	7.67791E-10	1.63809E-09
ENSGALG00000013177	BCAT1	1	7.68332E-10	1.63896E-09
ENSGALG00000003433	DPEP2	11	7.70344E-10	1.64296E-09
ENSGALG00000023632		2	7.75614E-10	1.65391E-09
ENSGALG00000010967	DOCK7	8	7.77661E-10	1.65798E-09
ENSGALG00000007012	ARRDC4	10	7.84577E-10	1.67244E-09
ENSGALG00000054397		16	7.96579E-10	1.69772E-09
ENSGALG00000009879		1	7.97861E-10	1.70016E-09
ENSGALG00000047720	KLHDC7A	21	8.12694E-10	1.73147E-09
ENSGALG00000026152		12	8.14711E-10	1.73546E-09
ENSGALG00000042797		3	8.14856E-10	1.73546E-09
ENSGALG00000004683	TPD52L2	20	8.21396E-10	1.74909E-09
ENSGALG00000013632	ACOT13	2	8.309E-10	1.76902E-09
ENSGALG00000007341	MED19	5	8.34399E-10	1.77616E-09
ENSGALG00000004033	GALNT10	13	8.45584E-10	1.79965E-09
ENSGALG00000001948		28	8.47211E-10	1.8028E-09
ENSGALG00000049034	MAP10	3	8.49249E-10	1.80682E-09
ENSGALG00000042929		7	8.5477E-10	1.81825E-09
ENSGALG00000010902	CERS6	7	8.62954E-10	1.83534E-09
ENSGALG00000010564	TBCK	4	8.8961E-10	1.8917E-09
ENSGALG00000009494	THAP5	1	8.91919E-10	1.89612E-09
ENSGALG00000043285	CNTD1	27	8.91998E-10	1.89612E-09
ENSGALG00000037950	ALPK3	10	9.06095E-10	1.92575E-09
ENSGALG00000013881	C22H2ORF42	22	9.07765E-10	1.92896E-09
ENSGALG00000013863	PSMG2	2	9.16818E-10	1.94786E-09

ENSGALG00000052481		26	9.2196E-10	1.95844E-09
ENSGALG00000037737		20	9.22897E-10	1.96009E-09
ENSGALG00000026373	KCNJ8	1	9.62095E-10	2.04299E-09
ENSGALG00000014801	NDC80	2	9.63595E-10	2.04581E-09
ENSGALG00000016293	BMP5	3	9.66401E-10	2.05142E-09
ENSGALG00000036731	GAS2L1	15	9.89018E-10	2.09906E-09
ENSGALG00000027492	TEDC1	8	9.9285E-10	2.10683E-09
ENSGALG00000050207	EMP2	14	1.00623E-09	2.13484E-09
ENSGALG00000012199	WDHD1	5	1.01738E-09	2.15813E-09
ENSGALG00000012089		5	1.01966E-09	2.16258E-09
ENSGALG00000015662		1	1.01999E-09	2.1629E-09
ENSGALG00000013727	SYNJ2	3	1.04132E-09	2.20762E-09
ENSGALG00000016667	AvBD10	3	1.04143E-09	2.20762E-09
ENSGALG00000005505	PIK3CB	9	1.05121E-09	2.22795E-09
ENSGALG00000008376	EIF2B5	9	1.06115E-09	2.24863E-09
ENSGALG00000049851		8	1.06426E-09	2.25482E-09
ENSGALG00000015387	ATG5	3	1.07038E-09	2.26739E-09
ENSGALG00000031069		7	1.07359E-09	2.2738E-09
ENSGALG00000002877	LRRC45	18	1.07414E-09	2.27457E-09
ENSGALG00000000812	OAZ1	28	1.07503E-09	2.27606E-09
ENSGALG00000042462		20	1.07758E-09	2.28106E-09
ENSGALG00000041179		2	1.07885E-09	2.28335E-09
ENSGALG00000029811	BMPER	2	1.0804E-09	2.28623E-09
ENSGALG00000012127	ELP4	5	1.0844E-09	2.29427E-09
ENSGALG00000050875	ITGB7	33	1.08457E-09	2.29427E-09
ENSGALG00000034297	TBX2	19	1.0869E-09	2.2988E-09
ENSGALG00000005305	ACKR2	2	1.09201E-09	2.3092E-09
ENSGALG00000010651	TFB2M	3	1.09247E-09	2.30978E-09
ENSGALG00000017283	CCND2	1	1.0971E-09	2.31917E-09
ENSGALG00000007641	LDB1	6	1.10802E-09	2.34184E-09
ENSGALG00000030609	REXO4	17	1.10931E-09	2.34417E-09
ENSGALG00000002686	ECD	6	1.13468E-09	2.39736E-09
ENSGALG00000003022		8	1.14261E-09	2.41369E-09

ENSGALG00000011389	TRAF3	5	1.15837E-09	2.44655E-09
ENSGALG00000008169	GDI1	1	1.16007E-09	2.44972E-09
ENSGALG00000020729	NDUFAF5	3	1.17097E-09	2.47231E-09
ENSGALG00000013210	C2CD5	1	1.17148E-09	2.47296E-09
ENSGALG00000030357	ABL2	8	1.1725E-09	2.4747E-09
ENSGALG00000045352	EIF4B	33	1.17509E-09	2.47973E-09
ENSGALG00000037780	PMEPA1	20	1.1785E-09	2.48649E-09
ENSGALG00000016737	NIPA2	1	1.17935E-09	2.48786E-09
ENSGALG00000037479	IGSF21	21	1.18586E-09	2.50115E-09
ENSGALG00000013999	FBXO40	1	1.191E-09	2.51157E-09
ENSGALG00000017010	SPRYD7	1	1.19283E-09	2.515E-09
ENSGALG00000040836	INSYN2A	6	1.19609E-09	2.52143E-09
ENSGALG00000029346		13	1.1971E-09	2.52311E-09
ENSGALG00000005662	AMPD3	5	1.20431E-09	2.53788E-09
ENSGALG00000015218	SLC19A2	1	1.21536E-09	2.56071E-09
ENSGALG00000014759	STYK1	1	1.22135E-09	2.57289E-09
ENSGALG00000010747	FBLN5	5	1.2242E-09	2.57846E-09
ENSGALG00000033943	HYI	8	1.23189E-09	2.59421E-09
ENSGALG00000016262	XK	1	1.23296E-09	2.596E-09
ENSGALG00000012061		7	1.23559E-09	2.60109E-09
ENSGALG00000052334		31	1.24324E-09	2.61674E-09
ENSGALG00000034708	USP12	1	1.24524E-09	2.6205E-09
ENSGALG00000047963	MECP2	30	1.24828E-09	2.62646E-09
ENSGALG00000012230	LRR1	5	1.24941E-09	2.62838E-09
ENSGALG00000003072	RPA1	19	1.25823E-09	2.64648E-09
ENSGALG00000022622	HOXA4	2	1.25949E-09	2.64866E-09
ENSGALG00000010185	HSPA4L	4	1.26666E-09	2.66329E-09
ENSGALG00000002532	GJA4	23	1.26931E-09	2.66839E-09
ENSGALG00000032854	ACP5	30	1.28146E-09	2.69332E-09
ENSGALG00000014081	OCIAD1	4	1.28161E-09	2.69332E-09
ENSGALG00000034590	CELF2	1	1.30045E-09	2.73244E-09
ENSGALG00000003360	ARL10	13	1.30166E-09	2.73451E-09
ENSGALG00000007177	PTPRG	12	1.31132E-09	2.75433E-09

ENSGALG00000002634	HDAC3	13	1.31157E-09	2.75433E-09
ENSGALG00000016320	FBXO9	3	1.31177E-09	2.75433E-09
ENSGALG00000041900	DLG3	4	1.31651E-09	2.7638E-09
ENSGALG00000010060	PSMA6	5	1.31714E-09	2.76466E-09
ENSGALG00000037453	TERF1	2	1.32174E-09	2.77384E-09
ENSGALG00000003033	STXBP4	18	1.32383E-09	2.77775E-09
ENSGALG00000054209		17	1.3286E-09	2.78727E-09
ENSGALG00000046223	GPR21	17	1.33372E-09	2.79753E-09
ENSGALG00000002164	DDX59	8	1.33934E-09	2.80883E-09
ENSGALG00000043268	DUSP19	7	1.34535E-09	2.82096E-09
ENSGALG00000030783	GTPBP1	1	1.34949E-09	2.82915E-09
ENSGALG00000003013		6	1.3563E-09	2.84293E-09
ENSGALG00000051652		33	1.36046E-09	2.85116E-09
ENSGALG00000007958	GTF3C3	7	1.371E-09	2.87277E-09
ENSGALG00000011332	ANKZF1	7	1.37396E-09	2.87847E-09
ENSGALG00000001231	SAMHD1	20	1.38631E-09	2.90385E-09
ENSGALG00000005062	FOXN4	15	1.38886E-09	2.90868E-09
ENSGALG00000009595	TWF1	1	1.40905E-09	2.95046E-09
ENSGALG00000004261	AQP9	10	1.41826E-09	2.96923E-09
ENSGALG00000004885	SLC24A5	10	1.43477E-09	3.00328E-09
ENSGALG00000005527	APPL1	12	1.43504E-09	3.00334E-09
ENSGALG00000000442	KCNA2	26	1.4381E-09	3.00923E-09
ENSGALG00000034560	SCARF2	15	1.44913E-09	3.03179E-09
ENSGALG00000006630	COMMD7	20	1.45391E-09	3.04125E-09
ENSGALG00000007956	PLXDC2	2	1.46153E-09	3.05668E-09
ENSGALG00000003826	ANAPC7	15	1.46415E-09	3.06163E-09
ENSGALG00000002944	CPS1	7	1.46957E-09	3.07244E-09
ENSGALG00000012480	NMI	7	1.48603E-09	3.10631E-09
ENSGALG00000015026		3	1.48848E-09	3.11091E-09
ENSGALG00000006078	PALB2	14	1.49717E-09	3.12854E-09
ENSGALG00000006048		19	1.50851E-09	3.15169E-09
ENSGALG00000003625	NDUFS5	23	1.52789E-09	3.19163E-09
ENSGALG00000026153	FOXO6	23	1.52831E-09	3.19197E-09

ENSGALG00000033971		28	1.53701E-09	3.20958E-09
ENSGALG00000017392	SPDYA	3	1.54971E-09	3.23555E-09
ENSGALG00000040089	HPS3	9	1.58527E-09	3.30923E-09
ENSGALG00000037879	PAF1	32	1.58705E-09	3.31237E-09
ENSGALG00000002982	SLC2A6	17	1.59168E-09	3.32147E-09
ENSGALG00000009312	RPL22L1	9	1.59274E-09	3.32312E-09
ENSGALG00000012172	RPL3	1	1.59521E-09	3.3277E-09
ENSGALG00000007052	PXK	12	1.60944E-09	3.35681E-09
ENSGALG00000015221	CCDC181	1	1.61967E-09	3.37758E-09
ENSGALG00000014561	PTPN6	1	1.62447E-09	3.38699E-09
ENSGALG00000015308	SEC63	3	1.63766E-09	3.41391E-09
ENSGALG00000053041		4	1.63911E-09	3.41636E-09
ENSGALG00000003208	GABARAPL2	11	1.64912E-09	3.43662E-09
ENSGALG00000016485	RHOB	3	1.6727E-09	3.48516E-09
ENSGALG00000015925	CGAS	3	1.70157E-09	3.54473E-09
ENSGALG00000035369		27	1.71833E-09	3.57902E-09
ENSGALG00000049929	LIAS	4	1.72502E-09	3.59234E-09
ENSGALG00000005353	FAR1	5	1.73749E-09	3.61769E-09
ENSGALG00000014233	FBLN1	1	1.75004E-09	3.64321E-09
ENSGALG00000008447	SH2D1A	4	1.75655E-09	3.65614E-09
ENSGALG00000032400	UTP23	2	1.75815E-09	3.65883E-09
ENSGALG00000052375		4	1.76286E-09	3.66802E-09
ENSGALG00000016541		1	1.77514E-09	3.69294E-09
ENSGALG00000020340	TMX1	5	1.7898E-09	3.7228E-09
ENSGALG00000010861	OMA1	8	1.79195E-09	3.72663E-09
ENSGALG00000010740	YIPF1	8	1.80159E-09	3.74604E-09
ENSGALG00000046779		10	1.80323E-09	3.74881E-09
ENSGALG00000005481	ARHGEF3	12	1.81976E-09	3.78252E-09
ENSGALG00000034251	SLC9A1	23	1.84466E-09	3.83363E-09
ENSGALG00000032097	TBX20	2	1.86114E-09	3.86722E-09
ENSGALG00000041592	SMARCC2	33	1.86372E-09	3.87194E-09
ENSGALG00000003106	TOX2	20	1.86897E-09	3.88217E-09
ENSGALG00000012009	JKAMP	5	1.86933E-09	3.88226E-09

ENSGALG00000031244	ADCY6	7	1.88149E-09	3.90684E-09
ENSGALG00000032681		1	1.90244E-09	3.94969E-09
ENSGALG00000013615	MTUS1	4	1.9036E-09	3.95141E-09
ENSGALG00000033079	RRAGC	23	1.90481E-09	3.95325E-09
ENSGALG00000051260		30	1.91356E-09	3.97074E-09
ENSGALG00000049414	CYP2C23a	6	1.92472E-09	3.99322E-09
ENSGALG00000016902	GPC5	1	1.93903E-09	4.02222E-09
ENSGALG00000042105	PLCB2	5	1.94959E-09	4.04343E-09
ENSGALG00000011649	ATG9A	7	1.95395E-09	4.05179E-09
ENSGALG00000002234	UNC13D	18	1.95823E-09	4.05996E-09
ENSGALG00000010708	ICA1	2	1.96811E-09	4.07977E-09
ENSGALG00000044293		9	1.97585E-09	4.09511E-09
ENSGALG00000014924	POLQ	1	1.98446E-09	4.11226E-09
ENSGALG00000033666	NHP2	13	2.00434E-09	4.15275E-09
ENSGALG00000004670	ATRIP	12	2.01407E-09	4.17177E-09
ENSGALG00000044705	TIMM50	32	2.0142E-09	4.17177E-09
ENSGALG00000008003	ZC3HC1	1	2.04125E-09	4.22707E-09
ENSGALG00000003854	IFT81	15	2.07217E-09	4.29037E-09
ENSGALG00000014555	C1H12ORF57	1	2.09919E-09	4.34558E-09
ENSGALG00000002169	PLEKHB2	9	2.10507E-09	4.35701E-09
ENSGALG00000012259		7	2.13291E-09	4.41388E-09
ENSGALG00000011619	SIVA1	5	2.18714E-09	4.52534E-09
ENSGALG00000008771	SHOC2	6	2.18933E-09	4.52909E-09
ENSGALG00000007809	TSHZ2	20	2.1924E-09	4.53467E-09
ENSGALG00000050481		3	2.21384E-09	4.57824E-09
ENSGALG00000039687	NIPAL4	13	2.22691E-09	4.60449E-09
ENSGALG00000008757	CNRIP1	3	2.2273E-09	4.60451E-09
ENSGALG00000046811	STIP1	33	2.25058E-09	4.65185E-09
ENSGALG00000002107	RANBP1	15	2.25871E-09	4.6671E-09
ENSGALG00000017122	SGCG	1	2.25872E-09	4.6671E-09
ENSGALG00000003584	KPNA2	18	2.26296E-09	4.67507E-09
ENSGALG00000012216	BMPR1B	4	2.27478E-09	4.6987E-09
ENSGALG00000034725		30	2.27825E-09	4.70506E-09

ENSGALG00000008722	MCOLN2	8	2.3006E-09	4.75041E-09
ENSGALG00000030866	CPQ	2	2.30776E-09	4.7644E-09
ENSGALG00000026558	ZNF362	21	2.31539E-09	4.77934E-09
ENSGALG00000004087	THSD4	10	2.3193E-09	4.7866E-09
ENSGALG00000005544	GPR55	9	2.33137E-09	4.81069E-09
ENSGALG00000000695	MFSD4A	26	2.33549E-09	4.81838E-09
ENSGALG00000018557	SOD3	4	2.35167E-09	4.85094E-09
ENSGALG00000050372	SYCN	32	2.36435E-09	4.87627E-09
ENSGALG00000014210	GNPDA2	4	2.3648E-09	4.87638E-09
ENSGALG00000033195	IGF2BP2	9	2.39465E-09	4.93709E-09
ENSGALG00000029296	MPI	10	2.39698E-09	4.94107E-09
ENSGALG00000005885	ESS2	15	2.40882E-09	4.96464E-09
ENSGALG00000004935	LSM14A	11	2.43402E-09	5.01572E-09
ENSGALG00000041577	ITGA4	7	2.43615E-09	5.01927E-09
ENSGALG00000014365	RBPJ	4	2.44828E-09	5.04341E-09
ENSGALG00000023780	TREM-B2	26	2.50475E-09	5.15886E-09
ENSGALG00000002688	NME4	14	2.56561E-09	5.28332E-09
ENSGALG00000032239	GCHFR	5	2.57208E-09	5.29574E-09
ENSGALG00000029027	SEPTIN12	14	2.57449E-09	5.2998E-09
ENSGALG00000006936	WDR6	12	2.57667E-09	5.30216E-09
ENSGALG00000029354	GMIP	30	2.5769E-09	5.30216E-09
ENSGALG00000054512		1	2.57694E-09	5.30216E-09
ENSGALG00000008085	NEFH	15	2.5818E-09	5.31127E-09
ENSGALG00000005638	IL2RG	4	2.58542E-09	5.31782E-09
ENSGALG00000001572	COMMD4	10	2.58783E-09	5.32187E-09
ENSGALG00000008038	SF3B1	7	2.58933E-09	5.32407E-09
ENSGALG00000017350	RRM1	1	2.59777E-09	5.34052E-09
ENSGALG00000003594	ANK1	22	2.60068E-09	5.3456E-09
ENSGALG00000010995	ITGB3BP	8	2.60707E-09	5.35771E-09
ENSGALG00000028821	GLIPR2	2	2.60745E-09	5.35771E-09
ENSGALG00000004858	FICD	15	2.61371E-09	5.36966E-09
ENSGALG00000010504	ADCK1	5	2.61812E-09	5.37783E-09
ENSGALG00000050374		21	2.62337E-09	5.38769E-09

ENSGALG00000052064	MRPS24	22	2.62777E-09	5.39582E-09
ENSGALG00000020999		15	2.63464E-09	5.40901E-09
ENSGALG00000006806	HINFP	24	2.66312E-09	5.46656E-09
ENSGALG00000026734	GABPB2	25	2.67206E-09	5.484E-09
ENSGALG00000010769	HPGD	4	2.74077E-09	5.62406E-09
ENSGALG00000032458	RABL2A	1	2.74696E-09	5.63583E-09
ENSGALG00000003767	NKD1	11	2.77641E-09	5.69529E-09
ENSGALG00000009828	PPP2R5A	3	2.77838E-09	5.69836E-09
ENSGALG00000029102	PXYLP1	9	2.81446E-09	5.77139E-09
ENSGALG00000003441	GTF3C5	17	2.81719E-09	5.77602E-09
ENSGALG00000055092		25	2.85549E-09	5.85356E-09
ENSGALG00000007240	TRAIL-LIKE	4	2.86519E-09	5.87247E-09
ENSGALG00000041251	HDAC10	1	2.88451E-09	5.91106E-09
ENSGALG00000044574	PLPP5	22	2.88757E-09	5.91633E-09
ENSGALG00000014758	ARHGAP28	2	2.89287E-09	5.9262E-09
ENSGALG00000002883	SCAPER	10	2.89396E-09	5.92743E-09
ENSGALG00000017229	FAT3	1	2.90889E-09	5.95702E-09
ENSGALG00000026055	PALM	28	2.91484E-09	5.96819E-09
ENSGALG00000032373		6	2.92378E-09	5.98551E-09
ENSGALG00000034768	ESF1	3	2.94114E-09	6.02003E-09
ENSGALG00000003383	KPNA6	23	2.98594E-09	6.11069E-09
ENSGALG00000036258	PCDH8	1	2.99598E-09	6.13022E-09
ENSGALG00000039288	RBM10	12	3.0083E-09	6.1544E-09
ENSGALG00000007967	FDXACB1	24	3.04268E-09	6.22368E-09
ENSGALG00000012584	MARCHF7	7	3.04976E-09	6.23712E-09
ENSGALG00000035365		33	3.07149E-09	6.28052E-09
ENSGALG00000015478	USP45	3	3.08109E-09	6.29908E-09
ENSGALG00000000906	RALGPS1	17	3.09952E-09	6.33571E-09
ENSGALG00000049256		5	3.10599E-09	6.34785E-09
ENSGALG00000001130	GALNT17	19	3.14275E-09	6.42191E-09
ENSGALG00000052763	BTG2	26	3.19175E-09	6.52095E-09
ENSGALG00000008304	STN1	6	3.20624E-09	6.54946E-09
ENSGALG00000027064	HIST1H3H	1	3.21734E-09	6.57103E-09

ENSGALG00000037656	NECAP1	1	3.26374E-09	6.66468E-09
ENSGALG00000051538		4	3.30598E-09	6.7498E-09
ENSGALG00000054176		33	3.32561E-09	6.78875E-09
ENSGALG00000054420		2	3.32801E-09	6.79251E-09
ENSGALG00000012033	SGSM3	1	3.34821E-09	6.83259E-09
ENSGALG00000035028	NAB2	33	3.41889E-09	6.97566E-09
ENSGALG00000019553	SERPINB10B	2	3.42513E-09	6.98721E-09
ENSGALG00000033570		24	3.43667E-09	7.00959E-09
ENSGALG00000014453	FAM46C	1	3.45168E-09	7.03903E-09
ENSGALG00000009839	PPIG	7	3.46949E-09	7.07416E-09
ENSGALG00000005613		6	3.51423E-09	7.16419E-09
ENSGALG00000033593	UBE2E2	2	3.52123E-09	7.17628E-09
ENSGALG00000013167	SDHA	2	3.52134E-09	7.17628E-09
ENSGALG00000042387	CRELD1	12	3.52243E-09	7.17731E-09
ENSGALG00000044279		3	3.52538E-09	7.18211E-09
ENSGALG00000004460	FOXK1	14	3.58455E-09	7.30145E-09
ENSGALG00000002253	HEMK1	12	3.58989E-09	7.3111E-09
ENSGALG00000011314	LRRC3B	2	3.6003E-09	7.33107E-09
ENSGALG00000034855	HK2	22	3.60967E-09	7.34892E-09
ENSGALG00000042469	PRKDC	2	3.67077E-09	7.47208E-09
ENSGALG00000038671		18	3.68465E-09	7.49907E-09
ENSGALG00000003298	DDX55	15	3.72049E-09	7.57076E-09
ENSGALG00000001126	CHAF1A	28	3.7549E-09	7.6395E-09
ENSGALG00000039983	EEPD1	2	3.83307E-09	7.79723E-09
ENSGALG00000039982	IRF5	1	3.84511E-09	7.82043E-09
ENSGALG00000038951	RASSF1	12	3.85333E-09	7.83583E-09
ENSGALG00000004064	GPER1	14	3.90772E-09	7.94511E-09
ENSGALG00000003261	PTRF	27	3.90885E-09	7.94608E-09
ENSGALG00000010282	TMEM63B	3	3.9158E-09	7.95889E-09
ENSGALG00000004637		19	3.91682E-09	7.95964E-09
ENSGALG00000016586	CCDC25	3	3.96723E-09	8.06074E-09
ENSGALG00000004812	FAM129A	8	4.07566E-09	8.27966E-09
ENSGALG00000014128	A4GALT	1	4.09218E-09	8.31183E-09

ENSGALG00000029536	VPS11	24	4.11124E-09	8.34915E-09
ENSGALG00000027534	GM2A	13	4.11202E-09	8.34934E-09
ENSGALG00000000541	ZC3H11B	26	4.11492E-09	8.35386E-09
ENSGALG00000040942	EFCAB5	19	4.12495E-09	8.37281E-09
ENSGALG00000030245		5	4.1482E-09	8.41755E-09
ENSGALG00000017524	DOHH	28	4.14837E-09	8.41755E-09
ENSGALG00000002973	BECN1	27	4.15061E-09	8.4207E-09
ENSGALG00000047446		27	4.1718E-09	8.4614E-09
ENSGALG00000031387	YWHAZ	2	4.17206E-09	8.4614E-09
ENSGALG00000000396	METTL2B	27	4.20925E-09	8.5354E-09
ENSGALG00000011735	XCR1	2	4.21205E-09	8.53965E-09
ENSGALG00000035099	LRRN1	12	4.24086E-09	8.59663E-09
ENSGALG00000003063	HYKK	10	4.29182E-09	8.69849E-09
ENSGALG00000035963	METTL18	8	4.30657E-09	8.72694E-09
ENSGALG00000041763	ADPRM	18	4.35425E-09	8.82211E-09
ENSGALG00000038003	EDF1	17	4.36361E-09	8.83912E-09
ENSGALG00000005329	MFSD14A	8	4.3641E-09	8.83912E-09
ENSGALG00000016027	CBR3	1	4.39027E-09	8.88936E-09
ENSGALG00000011801	ESR2	5	4.3904E-09	8.88936E-09
ENSGALG00000029691	NFKBIB	32	4.3911E-09	8.88936E-09
ENSGALG00000002569	RAN	15	4.41862E-09	8.94359E-09
ENSGALG00000002734	UHMK1	8	4.43456E-09	8.97436E-09
ENSGALG00000007434	RHBDF1	14	4.44015E-09	8.9842E-09
ENSGALG00000004598	CUX2	15	4.44144E-09	8.98532E-09
ENSGALG00000003870	MED7	13	4.49326E-09	9.08863E-09
ENSGALG00000052471	MRPS21	25	4.62476E-09	9.35307E-09
ENSGALG00000016847	CARS2	1	4.64178E-09	9.38452E-09
ENSGALG00000009690	CENPF	3	4.64185E-09	9.38452E-09
ENSGALG00000015004	RIOK3	2	4.65573E-09	9.41102E-09
ENSGALG00000004455	USP34	3	4.66718E-09	9.4326E-09
ENSGALG00000010758		8	4.6983E-09	9.49394E-09
ENSGALG00000015978	PANK2	4	4.71987E-09	9.53594E-09
ENSGALG00000027608	PIGC	8	4.73054E-09	9.5559E-09

ENSGALG00000016932	MZT1	1	4.75341E-09	9.60051E-09
ENSGALG00000009332		5	4.81825E-09	9.72987E-09
ENSGALG00000002616	LZIC	21	4.83421E-09	9.76047E-09
ENSGALG00000035079	RPSAP58	2	4.84272E-09	9.77604E-09
ENSGALG00000053653	ZNF467	2	4.84598E-09	9.781E-09
ENSGALG00000002955	ADAMTSL2	17	4.92732E-09	9.94354E-09
ENSGALG00000006560	SEMA3A	1	4.94392E-09	9.97538E-09
ENSGALG00000006156	COG7	14	4.95534E-09	9.99676E-09
ENSGALG00000020775		6	4.98506E-09	1.00551E-08
ENSGALG00000045758	SEC14L5	14	4.99249E-09	1.00684E-08
ENSGALG00000010224	GLIPR1L	1	5.00163E-09	1.00851E-08
ENSGALG00000008335	PHTF2	1	5.01851E-09	1.01168E-08
ENSGALG00000033144	COLEC10	2	5.019E-09	1.01168E-08
ENSGALG00000004814		11	5.02497E-09	1.01272E-08
ENSGALG00000039974	AKAP8L	30	5.03979E-09	1.01554E-08
ENSGALG00000039942	CTBP1	4	5.11608E-09	1.03074E-08
ENSGALG00000038440	COQ10B	7	5.12565E-09	1.0325E-08
ENSGALG00000003245	LSM1	22	5.13133E-09	1.03347E-08
ENSGALG00000037186		33	5.1332E-09	1.03368E-08
ENSGALG00000043828	HEXA	10	5.15066E-09	1.03702E-08
ENSGALG00000001634	RPL23	27	5.17034E-09	1.04081E-08
ENSGALG00000015193	CCDC80	1	5.20904E-09	1.04843E-08
ENSGALG00000029706	RUFY1	13	5.27666E-09	1.06186E-08
ENSGALG00000019514	CNDP2	2	5.27778E-09	1.06191E-08
ENSGALG00000008419	SEC13	12	5.28463E-09	1.06312E-08
ENSGALG00000011847	TTC26	1	5.33282E-09	1.07263E-08
ENSGALG00000048771		19	5.35625E-09	1.07717E-08
ENSGALG00000002960	HMGCS2	8	5.42673E-09	1.09116E-08
ENSGALG00000040873	MRPL3	2	5.43501E-09	1.09265E-08
ENSGALG00000036190	AOC1	2	5.43964E-09	1.0934E-08
ENSGALG00000015681	NRIP1	1	5.4803E-09	1.10139E-08
ENSGALG00000006325		12	5.52422E-09	1.11003E-08
ENSGALG00000031325	TGFB1	32	5.64968E-09	1.13506E-08

ENSGALG00000038550		10	5.74486E-09	1.15399E-08
ENSGALG00000006388	IL16	10	5.76218E-09	1.15728E-08
ENSGALG00000005490	RPS2	14	5.77448E-09	1.15956E-08
ENSGALG00000012782	BLOC1S5	2	5.81315E-09	1.16713E-08
ENSGALG00000035023	CNIH1	5	5.81892E-09	1.16809E-08
ENSGALG00000004267	SIGIRR	5	5.86143E-09	1.17644E-08
ENSGALG00000011210	FAM175A	4	5.94057E-09	1.19212E-08
ENSGALG00000009276	LRAT	4	5.94537E-09	1.19289E-08
ENSGALG00000031604		6	5.96932E-09	1.1975E-08
ENSGALG00000013794	MPPE1	2	6.00067E-09	1.20359E-08
ENSGALG00000045288	CAMK2N1	21	6.13063E-09	1.22945E-08
ENSGALG00000040098	EEF1D	2	6.13671E-09	1.23047E-08
ENSGALG00000005834	PAFAH1B1	19	6.16976E-09	1.23689E-08
ENSGALG00000012147	DDX18	7	6.18686E-09	1.24012E-08
ENSGALG00000013722	SEMA3B	12	6.19445E-09	1.2413E-08
ENSGALG00000019555	SERPINB1	2	6.19481E-09	1.2413E-08
ENSGALG00000007507	MASTL	2	6.20253E-09	1.24265E-08
ENSGALG00000054821	NAALAD2	1	6.20829E-09	1.2436E-08
ENSGALG00000007195		15	6.23212E-09	1.24817E-08
ENSGALG00000034788		1	6.24221E-09	1.24998E-08
ENSGALG00000028749	ACTG1	18	6.296E-09	1.26054E-08
ENSGALG00000006158	ACVR2B	2	6.38865E-09	1.27888E-08
ENSGALG00000053184		8	6.42086E-09	1.28502E-08
ENSGALG00000052932	SHROOM3	4	6.42141E-09	1.28502E-08
ENSGALG00000015358	MYH15	1	6.43685E-09	1.2879E-08
ENSGALG00000049254		11	6.47711E-09	1.29574E-08
ENSGALG00000013962	HBS1L	3	6.50167E-09	1.30044E-08
ENSGALG00000000818		1	6.53957E-09	1.30781E-08
ENSGALG00000033196	EIF2S2	20	6.54909E-09	1.3095E-08
ENSGALG00000001093	TCF3	28	6.59407E-09	1.31827E-08
ENSGALG00000015136	ILDR1	1	6.61711E-09	1.32266E-08
ENSGALG00000002012		6	6.64873E-09	1.32877E-08
ENSGALG00000008530	EIF4A3	3	6.66835E-09	1.33247E-08

ENSGALG00000011394	CYP2J21	8	6.67145E-09	1.33287E-08
ENSGALG00000008857	PRPF4	17	6.68266E-09	1.33489E-08
ENSGALG00000032057		33	6.74711E-09	1.34754E-08
ENSGALG00000003201	FBXL8	11	6.83384E-09	1.36464E-08
ENSGALG00000012770		2	6.91962E-09	1.38155E-08
ENSGALG00000025929	HOXA5	2	6.9229E-09	1.38197E-08
ENSGALG00000038512	AGTR1	9	7.01362E-09	1.39985E-08
ENSGALG00000007447	TUBB1	20	7.03686E-09	1.40426E-08
ENSGALG00000005299	ANXA7	6	7.12875E-09	1.42237E-08
ENSGALG00000008646		9	7.24883E-09	1.44609E-08
ENSGALG00000050002	COLEC11	3	7.25789E-09	1.44766E-08
ENSGALG00000000357		22	7.3087E-09	1.45746E-08
ENSGALG00000008933	CASP7	6	7.30943E-09	1.45746E-08
ENSGALG00000007178	FADS2	5	7.32312E-09	1.45995E-08
ENSGALG00000006253	ILKAP	9	7.40278E-09	1.47559E-08
ENSGALG00000015461		1	7.42495E-09	1.47977E-08
ENSGALG00000004198	PDZD11	4	7.46988E-09	1.48831E-08
ENSGALG00000036527	SYBU	2	7.47024E-09	1.48831E-08
ENSGALG00000001962	PTBP1	28	7.50845E-09	1.49568E-08
ENSGALG00000039124	KLHL3	13	7.51954E-09	1.49764E-08
ENSGALG00000030863		10	7.52244E-09	1.49798E-08
ENSGALG00000043829		23	7.52757E-09	1.49875E-08
ENSGALG00000015117	DRD3	1	7.57678E-09	1.5083E-08
ENSGALG00000037769	NEBL	2	7.60912E-09	1.5145E-08
ENSGALG00000015687		4	7.69365E-09	1.53107E-08
ENSGALG00000041491	ACKR4	2	7.73407E-09	1.53886E-08
ENSGALG00000032889	RBFOX3	18	7.73627E-09	1.53905E-08
ENSGALG00000005618	CTR9	5	7.75534E-09	1.54259E-08
ENSGALG00000022750	GPR18	1	7.79524E-09	1.55028E-08
ENSGALG00000009185	ROM1	5	7.87855E-09	1.56659E-08
ENSGALG00000011464	EIF5	5	7.90469E-09	1.57153E-08
ENSGALG00000012721	C2H6orf52	2	7.92097E-09	1.5745E-08
ENSGALG00000044816		4	7.92223E-09	1.5745E-08

ENSGALG00000009062	CDK14	2	7.95026E-09	1.57982E-08
ENSGALG00000016024	SETD4	1	8.02423E-09	1.59426E-08
ENSGALG00000006976		9	8.02864E-09	1.59487E-08
ENSGALG00000003805		6	8.03668E-09	1.59621E-08
ENSGALG00000028648	CCDC59	1	8.05623E-09	1.59983E-08
ENSGALG00000053917	CD164	3	8.12733E-09	1.61369E-08
ENSGALG00000009352	DPF3	5	8.15463E-09	1.6186E-08
ENSGALG00000029508	SMIM19	4	8.15474E-09	1.6186E-08
ENSGALG00000027056		1	8.19903E-09	1.62713E-08
ENSGALG00000007546		4	8.26334E-09	1.63962E-08
ENSGALG00000009214	CRLS1	3	8.27165E-09	1.64101E-08
ENSGALG00000041346	CXCL12	6	8.27346E-09	1.6411E-08
ENSGALG00000041370	ZNF335	20	8.34651E-09	1.65532E-08
ENSGALG00000038730	GIGYF2	9	8.45191E-09	1.67595E-08
ENSGALG00000026383	TMSB4X	1	8.53616E-09	1.69238E-08
ENSGALG00000047265		28	8.56421E-09	1.69767E-08
ENSGALG00000016055	KCNJ15	1	8.78983E-09	1.74211E-08
ENSGALG00000035916	SHANK2	5	8.98756E-09	1.78101E-08
ENSGALG00000016457	TRIB2	3	9.00351E-09	1.78388E-08
ENSGALG00000015415	HACE1	3	9.05377E-09	1.79354E-08
ENSGALG00000020488		5	9.11384E-09	1.80515E-08
ENSGALG00000023296	TAL1	8	9.17301E-09	1.81657E-08
ENSGALG00000012173	LGALS3	5	9.32327E-09	1.84603E-08
ENSGALG00000009230	TMEM131L	4	9.34456E-09	1.84995E-08
ENSGALG00000009788	SMYD2	3	9.39947E-09	1.86052E-08
ENSGALG00000006443	MADPRT1	1	9.49574E-09	1.87926E-08
ENSGALG00000010628	ACSL1	4	9.49819E-09	1.87945E-08
ENSGALG00000038405	CASR	1	9.5358E-09	1.88658E-08
ENSGALG00000034673	HTRA3	4	9.6622E-09	1.91128E-08
ENSGALG00000011238	WLS	8	9.7086E-09	1.92015E-08
ENSGALG00000040787	FAM193B	13	9.75773E-09	1.92955E-08
ENSGALG00000037603	SESN2	23	9.79955E-09	1.9375E-08
ENSGALG00000005132	CIAO2B	11	9.82293E-09	1.94181E-08

ENSGALG00000010593	GALC	5	9.87093E-09	1.95099E-08
ENSGALG00000026607		10	9.88075E-09	1.95261E-08
ENSGALG00000027435	MED21	1	1.00005E-08	1.97595E-08
ENSGALG00000008663	DDAH1	8	1.00452E-08	1.98447E-08
ENSGALG00000050224	GOLT1A	26	1.0096E-08	1.99399E-08
ENSGALG00000029694	SNTA1	20	1.00967E-08	1.99399E-08
ENSGALG00000035952	KIAA1143	2	1.02374E-08	2.02146E-08
ENSGALG00000041331	RRP12	6	1.02551E-08	2.02462E-08
ENSGALG00000052448	STYX	5	1.02834E-08	2.02989E-08
ENSGALG00000001036	ELAC2	18	1.03156E-08	2.03591E-08
ENSGALG00000006861	COQ7	14	1.03212E-08	2.03668E-08
ENSGALG00000050720		1	1.03412E-08	2.0403E-08
ENSGALG00000000737	SMPDL3B	23	1.03724E-08	2.04611E-08
ENSGALG00000047952		1	1.04651E-08	2.06407E-08
ENSGALG00000009057	TASP1	3	1.05031E-08	2.07124E-08
ENSGALG00000049986		2	1.05261E-08	2.07542E-08
ENSGALG00000042810		14	1.0632E-08	2.09598E-08
ENSGALG00000047924		10	1.06674E-08	2.10262E-08
ENSGALG00000004517	PARP3	12	1.07004E-08	2.10879E-08
ENSGALG00000038585	NOA1	4	1.07139E-08	2.1111E-08
ENSGALG00000015367	NECTIN3	1	1.07338E-08	2.11467E-08
ENSGALG00000033857	NAT8B	4	1.08125E-08	2.12983E-08
ENSGALG00000013039	BID	1	1.09016E-08	2.14703E-08
ENSGALG00000005414	MSRB1	14	1.10084E-08	2.16772E-08
ENSGALG00000037172	IK	13	1.10395E-08	2.1735E-08
ENSGALG00000013784	CDT1	11	1.11675E-08	2.19833E-08
ENSGALG00000039773	AKAP8	30	1.12311E-08	2.21051E-08
ENSGALG00000002222	NOC2L	21	1.1373E-08	2.23808E-08
ENSGALG00000015436	SDCBP	2	1.15209E-08	2.26682E-08
ENSGALG00000037325	SERP1	9	1.16045E-08	2.2829E-08
ENSGALG00000032365	CEBPG	11	1.16565E-08	2.29275E-08
ENSGALG00000008652	STEEP1	4	1.16883E-08	2.29864E-08
ENSGALG00000001328	BSG	28	1.17293E-08	2.30632E-08

ENSGALG00000002562	ARAP3	13	1.18165E-08	2.3231E-08
ENSGALG00000008355	CASP8	7	1.18325E-08	2.32587E-08
ENSGALG00000000669	ETV3	25	1.18407E-08	2.32711E-08
ENSGALG00000033609	NACA	33	1.19008E-08	2.33853E-08
ENSGALG00000000723	STX12	23	1.19033E-08	2.33865E-08
ENSGALG00000003696		15	1.19164E-08	2.34084E-08
ENSGALG00000001850	RARS	13	1.19415E-08	2.3454E-08
ENSGALG00000003972	FAXDC2	13	1.19622E-08	2.34909E-08
ENSGALG00000014031	ETFRF1	1	1.19986E-08	2.35586E-08
ENSGALG00000031525	TSTA3	2	1.23093E-08	2.41647E-08
ENSGALG00000015226	SPIDR	2	1.23438E-08	2.42285E-08
ENSGALG00000002002	BCAS2	26	1.23546E-08	2.42458E-08
ENSGALG00000004943	SS18L2	11	1.25244E-08	2.45752E-08
ENSGALG00000010309	ZC2HC1C	5	1.25829E-08	2.46861E-08
ENSGALG00000054596		3	1.26466E-08	2.4807E-08
ENSGALG00000002729	GK5	9	1.2699E-08	2.49058E-08
ENSGALG00000004971	URM1	17	1.27251E-08	2.4953E-08
ENSGALG00000011300	TOP2B	2	1.27422E-08	2.49825E-08
ENSGALG00000008738		7	1.27692E-08	2.50313E-08
ENSGALG00000001620		17	1.27735E-08	2.50358E-08
ENSGALG00000003675	PRKAR1B	14	1.28318E-08	2.51459E-08
ENSGALG00000009669	RSRC1	9	1.28582E-08	2.51936E-08
ENSGALG00000030998	MTA2	33	1.28976E-08	2.52669E-08
ENSGALG00000040878	TMEM208	11	1.29455E-08	2.53566E-08
ENSGALG00000004554	CACYBP	8	1.30068E-08	2.54725E-08
ENSGALG00000032031	MMP16	2	1.30386E-08	2.55306E-08
ENSGALG00000029199	WDR55	13	1.30798E-08	2.56072E-08
ENSGALG00000029419	MRPL16	5	1.30941E-08	2.56311E-08
ENSGALG00000053510		1	1.33284E-08	2.60857E-08
ENSGALG00000048082		4	1.34016E-08	2.62247E-08
ENSGALG00000009431	PLEKHD1	5	1.35108E-08	2.64341E-08
ENSGALG00000004035	CRYBA1	19	1.35253E-08	2.64581E-08
ENSGALG00000052784	BBS12	4	1.35852E-08	2.65711E-08

ENSGALG00000029569		2	1.36487E-08	2.66911E-08
ENSGALG00000027838	HMG20B	28	1.36955E-08	2.67783E-08
ENSGALG00000034434	IDH2	10	1.37368E-08	2.68547E-08
ENSGALG00000032702	ITGA3	27	1.37885E-08	2.69514E-08
ENSGALG00000000081	IL4I1	16	1.38648E-08	2.70962E-08
ENSGALG00000042708		16	1.38855E-08	2.71324E-08
ENSGALG00000033522		27	1.39036E-08	2.71635E-08
ENSGALG00000011331	CTH	8	1.39575E-08	2.72644E-08
ENSGALG00000034616	INHBA	2	1.39731E-08	2.72903E-08
ENSGALG00000009248	HSPA12A	6	1.4104E-08	2.75417E-08
ENSGALG00000035476	MRNIP	13	1.45802E-08	2.84671E-08
ENSGALG00000004046	LARP6	10	1.4733E-08	2.87608E-08
ENSGALG00000034802	SETD3	5	1.47708E-08	2.88298E-08
ENSGALG00000037593	PINK1	21	1.49138E-08	2.91044E-08
ENSGALG00000039335	SLC25A22	5	1.49766E-08	2.92223E-08
ENSGALG00000033428	CA13	2	1.50773E-08	2.94139E-08
ENSGALG00000033953	SHROOM4	4	1.519E-08	2.96291E-08
ENSGALG00000042422	GLI1	33	1.53111E-08	2.98606E-08
ENSGALG00000016860	ERCC5	1	1.56201E-08	3.04583E-08
ENSGALG00000002457	SIL1	13	1.56386E-08	3.04895E-08
ENSGALG00000028302	UQCRQ	13	1.569E-08	3.05849E-08
ENSGALG00000028629	TM4SF1a	1	1.5723E-08	3.06443E-08
ENSGALG00000010042	CNOT2	1	1.5853E-08	3.08927E-08
ENSGALG00000038146		19	1.62333E-08	3.16287E-08
ENSGALG00000017627	C10orf2	6	1.6383E-08	3.19153E-08
ENSGALG00000040266	GLI3	2	1.67257E-08	3.25776E-08
ENSGALG00000040802	SOWAHA	13	1.67969E-08	3.27112E-08
ENSGALG00000029015	TM6SF2	28	1.68367E-08	3.27835E-08
ENSGALG00000037734	PIGB	10	1.68788E-08	3.28602E-08
ENSGALG00000024051	SETDB1	25	1.6911E-08	3.29176E-08
ENSGALG00000031158	OAT	6	1.69836E-08	3.30537E-08
ENSGALG00000015069	B4GALT4	1	1.70909E-08	3.32571E-08
ENSGALG00000000589	BTN1A1	28	1.71687E-08	3.34032E-08

ENSGALG00000013646	VPS37A	4	1.73096E-08	3.36719E-08
ENSGALG00000036497	SOX4	2	1.74581E-08	3.39554E-08
ENSGALG00000027925	HOXA3	2	1.77026E-08	3.44256E-08
ENSGALG00000051144	EVI5L	30	1.77632E-08	3.45378E-08
ENSGALG00000003883	MTMR10	10	1.78833E-08	3.47658E-08
ENSGALG00000009936	AP4S1	5	1.80535E-08	3.5091E-08
ENSGALG00000017274	CD9	1	1.81072E-08	3.51899E-08
ENSGALG00000051443	C1orf216	23	1.82422E-08	3.54466E-08
ENSGALG00000009293	CHN1	7	1.82589E-08	3.54734E-08
ENSGALG00000041404	SALL3	2	1.83053E-08	3.55578E-08
ENSGALG00000033888	IDH3B	4	1.83345E-08	3.56088E-08
ENSGALG00000001343	LAMB3	26	1.8369E-08	3.56702E-08
ENSGALG00000042351	PIK3R6	18	1.84608E-08	3.58428E-08
ENSGALG00000015322	IMPG2	1	1.84965E-08	3.59064E-08
ENSGALG00000006627	UPB1	15	1.88301E-08	3.65481E-08
ENSGALG00000010680	TTC7B	5	1.90065E-08	3.68846E-08
ENSGALG00000037522	SBNO2	28	1.90606E-08	3.69838E-08
ENSGALG00000034509	DYNLRB1	20	1.91966E-08	3.72417E-08
ENSGALG00000030034	G6PC1	27	1.92281E-08	3.72969E-08
ENSGALG00000023621	SFXN3	6	1.92924E-08	3.74157E-08
ENSGALG00000011205	SMOC2	3	1.94369E-08	3.76899E-08
ENSGALG00000037322	HIST1H46	1	1.95399E-08	3.78836E-08
ENSGALG00000007912	ATP9A	20	1.96126E-08	3.80186E-08
ENSGALG00000040017	THRA	27	1.9643E-08	3.80713E-08
ENSGALG00000010826	PRKAA2	8	1.96631E-08	3.81043E-08
ENSGALG00000028744	TMEM18	3	1.96699E-08	3.81113E-08
ENSGALG00000002813	RPS10	26	1.97093E-08	3.81816E-08
ENSGALG00000038902	PIM1	26	1.97632E-08	3.82801E-08
ENSGALG00000006334	LAP3	4	1.9948E-08	3.86318E-08
ENSGALG00000009139	TMEM185A	4	1.9996E-08	3.87159E-08
ENSGALG00000007252	ANKDD1A	10	1.99977E-08	3.87159E-08
ENSGALG00000040173	NDUFS8	5	2.00165E-08	3.8746E-08
ENSGALG00000001780	PROC	9	2.03654E-08	3.94152E-08

ENSGALG00000011404	HOPX	4	2.04207E-08	3.95159E-08
ENSGALG00000012692	SIRT5	2	2.04334E-08	3.95343E-08
ENSGALG00000004953	DRG2	14	2.05134E-08	3.96827E-08
ENSGALG00000001849	BSN	12	2.05631E-08	3.97725E-08
ENSGALG00000050072	EMID1	15	2.05811E-08	3.98009E-08
ENSGALG00000008864	FXR1	9	2.0618E-08	3.98661E-08
ENSGALG00000010561	GPATCH11	3	2.07532E-08	4.0121E-08
ENSGALG00000001651	TTI2	22	2.07992E-08	4.02037E-08
ENSGALG00000047713	QPRT	26	2.08808E-08	4.0355E-08
ENSGALG00000015414	NSUN3	1	2.103E-08	4.06368E-08
ENSGALG00000005857	ALOX5	6	2.11535E-08	4.08692E-08
ENSGALG00000044230	ARHGAP27	27	2.11824E-08	4.09185E-08
ENSGALG00000054624		7	2.13928E-08	4.13184E-08
ENSGALG00000006568	TM9SF4	20	2.16111E-08	4.17333E-08
ENSGALG00000010020	TTC7A	3	2.18521E-08	4.2192E-08
ENSGALG00000051358		9	2.18653E-08	4.22107E-08
ENSGALG00000009061	ACTL6A	9	2.27563E-08	4.3924E-08
ENSGALG00000005071	MPP1	4	2.28277E-08	4.40545E-08
ENSGALG00000001617	CAMKK1	19	2.28312E-08	4.40545E-08
ENSGALG00000040101		16	2.29444E-08	4.4266E-08
ENSGALG00000052647		19	2.30246E-08	4.44137E-08
ENSGALG00000029657		25	2.31442E-08	4.46373E-08
ENSGALG00000011605	EPB41L5	7	2.34832E-08	4.5284E-08
ENSGALG00000012611	RTCB	1	2.35716E-08	4.54473E-08
ENSGALG00000013817	SPIRE1	2	2.3586E-08	4.54678E-08
ENSGALG00000031332	NPEPPS	27	2.36404E-08	4.55654E-08
ENSGALG00000003684		14	2.37041E-08	4.56811E-08
ENSGALG00000013232	RHBG	25	2.37128E-08	4.56876E-08
ENSGALG00000044996	TMEM71	2	2.3715E-08	4.56876E-08
ENSGALG00000015804	LTN1	1	2.39397E-08	4.61132E-08
ENSGALG00000015708	FGFR3	4	2.39692E-08	4.61629E-08
ENSGALG00000009739	AMIGO2	1	2.39928E-08	4.62009E-08
ENSGALG00000042794	ZFAND1	2	2.45734E-08	4.73115E-08

ENSGALG00000004571	PPP1CC	15	2.49654E-08	4.80587E-08
ENSGALG00000000521	SPIRE2	11	2.50262E-08	4.8168E-08
ENSGALG00000053196		33	2.5115E-08	4.83313E-08
ENSGALG00000016543	NHS	1	2.5468E-08	4.90029E-08
ENSGALG00000040511	MTERF3	2	2.65504E-08	5.10774E-08
ENSGALG00000001802	JMJD6	18	2.67189E-08	5.13935E-08
ENSGALG00000007610	CDC23	13	2.67476E-08	5.14407E-08
ENSGALG00000003701	SGMS1	6	2.69828E-08	5.18848E-08
ENSGALG00000002507	MBL2	6	2.72191E-08	5.23309E-08
ENSGALG00000038838	NSMAF	2	2.73194E-08	5.25154E-08
ENSGALG00000012299	ANKRD54	1	2.7365E-08	5.25948E-08
ENSGALG00000001067	TMEM57	23	2.74639E-08	5.27765E-08
ENSGALG00000037214		4	2.76622E-08	5.31492E-08
ENSGALG00000029670	CRYZL1	1	2.78173E-08	5.34389E-08
ENSGALG00000021079	RGS19	20	2.79545E-08	5.36939E-08
ENSGALG00000011200	THBS2	3	2.80718E-08	5.39107E-08
ENSGALG00000015437	MPC2	1	2.83519E-08	5.444E-08
ENSGALG00000000978	CEP104	21	2.83932E-08	5.45107E-08
ENSGALG00000011288	DACT2	3	2.89488E-08	5.55687E-08
ENSGALG00000051205		3	2.91874E-08	5.6018E-08
ENSGALG00000011534	ZFYVE21	5	2.93784E-08	5.63756E-08
ENSGALG00000039713	AGO4	23	2.95259E-08	5.66499E-08
ENSGALG00000026092		2	2.96194E-08	5.68201E-08
ENSGALG00000047542	COA4	1	2.96749E-08	5.69177E-08
ENSGALG00000004360		13	2.97912E-08	5.71318E-08
ENSGALG00000047498	HNRNPUL1	32	3.03222E-08	5.8141E-08
ENSGALG00000015283	CEP57L1	3	3.04093E-08	5.82988E-08
ENSGALG00000013879	TIA1	22	3.10614E-08	5.95396E-08
ENSGALG00000009451	PPID	4	3.10805E-08	5.95668E-08
ENSGALG00000004465	PALD1	6	3.11787E-08	5.97457E-08
ENSGALG00000005095	SLC25A25	17	3.14716E-08	6.02976E-08
ENSGALG00000001981	PARP6	10	3.21504E-08	6.15883E-08
ENSGALG00000043671		16	3.23681E-08	6.19956E-08

ENSGALG00000027777	CNN2	28	3.24907E-08	6.22207E-08
ENSGALG00000028501	DCK	4	3.26617E-08	6.25383E-08
ENSGALG00000028960	NEU3	1	3.29016E-08	6.29877E-08
ENSGALG00000042764	COG5	1	3.31557E-08	6.34643E-08
ENSGALG00000036916		12	3.34314E-08	6.39819E-08
ENSGALG00000021031	CXCR7	7	3.34671E-08	6.40402E-08
ENSGALG00000040141	H2AFY	13	3.34964E-08	6.40863E-08
ENSGALG00000014038	CDC123	1	3.38384E-08	6.47304E-08
ENSGALG00000043088		24	3.40359E-08	6.5098E-08
ENSGALG00000005884		2	3.47775E-08	6.65061E-08
ENSGALG00000050553		3	3.4857E-08	6.66475E-08
ENSGALG00000012870	AGK	1	3.49227E-08	6.67627E-08
ENSGALG00000011209	EVL	5	3.49309E-08	6.6768E-08
ENSGALG00000004376	KCNJ2	18	3.50982E-08	6.70771E-08
ENSGALG00000036547		4	3.55141E-08	6.78601E-08
ENSGALG00000010363	HERC3	4	3.5519E-08	6.78601E-08
ENSGALG00000007944	HECW2	7	3.56956E-08	6.81868E-08
ENSGALG00000051163		30	3.5742E-08	6.82591E-08
ENSGALG00000003553	ABCA12	7	3.57446E-08	6.82591E-08
ENSGALG00000006872	PISD	15	3.58435E-08	6.84371E-08
ENSGALG00000006351	FAS	6	3.65805E-08	6.98335E-08
ENSGALG00000038657	ZNF777	2	3.71321E-08	7.08753E-08
ENSGALG00000048306		26	3.74601E-08	7.14902E-08
ENSGALG00000010007	EAPP	5	3.77478E-08	7.2028E-08
ENSGALG00000029672	ABCB6	7	3.7853E-08	7.22175E-08
ENSGALG00000015705		4	3.82951E-08	7.30495E-08
ENSGALG00000003777	NELL1	5	3.84217E-08	7.32795E-08
ENSGALG00000052204	ENY2	2	3.89247E-08	7.42218E-08
ENSGALG00000001000	HSPA5	17	3.8928E-08	7.42218E-08
ENSGALG00000011016	PGM1	8	3.90725E-08	7.44859E-08
ENSGALG00000007606	PGAM1	6	3.94687E-08	7.52294E-08
ENSGALG00000010478	STIL	8	3.97842E-08	7.58189E-08
ENSGALG00000046830		22	3.97999E-08	7.5837E-08

ENSGALG00000039032	FJX1	5	3.98301E-08	7.58825E-08
ENSGALG00000038915	E2F5	2	4.0339E-08	7.68401E-08
ENSGALG00000046393	SLAMF1	25	4.04702E-08	7.7078E-08
ENSGALG00000003042	PXMP4	20	4.08243E-08	7.77403E-08
ENSGALG00000013732	SERAC1	3	4.08388E-08	7.77558E-08
ENSGALG00000053042		31	4.1104E-08	7.82484E-08
ENSGALG00000007791	OTOP2	18	4.11447E-08	7.83136E-08
ENSGALG00000031593	TMSB15B	4	4.12991E-08	7.85952E-08
ENSGALG00000010228	TOE1	8	4.13691E-08	7.87161E-08
ENSGALG00000020392	PRIMA1	5	4.18884E-08	7.96919E-08
ENSGALG00000044768		1	4.19392E-08	7.97761E-08
ENSGALG00000040651	IGF1R	10	4.26358E-08	8.10885E-08
ENSGALG00000030643	VANGL2	25	4.28903E-08	8.15598E-08
ENSGALG00000016919	ACOD1	1	4.30206E-08	8.17949E-08
ENSGALG00000050216		19	4.3179E-08	8.20831E-08
ENSGALG00000001740	MGAT5B	18	4.32364E-08	8.21796E-08
ENSGALG00000050946		31	4.3398E-08	8.24738E-08
ENSGALG00000034421	SENP1	33	4.38333E-08	8.3288E-08
ENSGALG00000009264	MTX2	7	4.38408E-08	8.32894E-08
ENSGALG00000007079	PAPPA	17	4.38693E-08	8.33305E-08
ENSGALG00000011659	ACAT2	3	4.39206E-08	8.34149E-08
ENSGALG00000054542		22	4.45549E-08	8.46064E-08
ENSGALG00000011663	FNDC1	3	4.46912E-08	8.48521E-08
ENSGALG00000012322	KCTD16	13	4.48334E-08	8.51087E-08
ENSGALG00000040380	RAD51C	19	4.52462E-08	8.5879E-08
ENSGALG00000006979	SPPL3	15	4.55564E-08	8.64543E-08
ENSGALG00000006318	IL21R	14	4.60139E-08	8.73089E-08
ENSGALG00000003147	TRPC4AP	20	4.62761E-08	8.77928E-08
ENSGALG00000032610	NAGLU	27	4.63134E-08	8.78382E-08
ENSGALG00000043087	MTF2	8	4.63144E-08	8.78382E-08
ENSGALG00000011211	HACL1	2	4.65243E-08	8.82224E-08
ENSGALG00000040561	GPR171	9	4.67628E-08	8.8661E-08
ENSGALG00000037467	NEU2	9	4.70253E-08	8.91449E-08

ENSGALG00000044199	CYP2AC7	14	4.70643E-08	8.92049E-08
ENSGALG00000002932	NME2	18	4.75227E-08	9.00597E-08
ENSGALG00000049065	ZFPM1	11	4.76133E-08	9.02174E-08
ENSGALG00000010133	PTCH2	8	4.77317E-08	9.04276E-08
ENSGALG00000047621	YBEY	7	4.79207E-08	9.07716E-08
ENSGALG00000003454	ZNF207	18	4.83593E-08	9.15881E-08
ENSGALG00000016833	F7	1	4.85856E-08	9.20025E-08
ENSGALG00000041390	TMEM141	17	4.86428E-08	9.20965E-08
ENSGALG00000011855	RHOJ	5	4.90679E-08	9.28869E-08
ENSGALG00000006409	PODXL	1	4.91237E-08	9.29782E-08
ENSGALG00000030775	HIGD1C	2	4.92001E-08	9.31083E-08
ENSGALG00000042215	FAAP100	18	4.94555E-08	9.35771E-08
ENSGALG00000027778		5	4.96223E-08	9.38781E-08
ENSGALG00000035599	CD24	3	4.96556E-08	9.39265E-08
ENSGALG00000009838	AQR	5	4.96988E-08	9.39937E-08
ENSGALG00000034860	BCHE	9	4.99141E-08	9.43862E-08
ENSGALG00000004763	ALDH3A2	19	5.00785E-08	9.46824E-08
ENSGALG00000001316	CPNE2	11	5.01629E-08	9.48274E-08
ENSGALG00000027918	PAFAH1B2	24	5.05961E-08	9.56315E-08
ENSGALG00000013155		2	5.06083E-08	9.56397E-08
ENSGALG00000001529	THYN1	24	5.09445E-08	9.626E-08
ENSGALG00000001270	PRKCZ	21	5.11699E-08	9.6671E-08
ENSGALG00000003355	NOP16	13	5.14461E-08	9.71778E-08
ENSGALG00000052192	TUBA1B	33	5.17712E-08	9.77626E-08
ENSGALG00000009842	RASSF3	1	5.17718E-08	9.77626E-08
ENSGALG00000050153		2	5.17833E-08	9.77692E-08
ENSGALG00000008340	FES	10	5.19999E-08	9.8163E-08
ENSGALG00000011544	BCL2L14	1	5.24614E-08	9.90189E-08
ENSGALG00000045925		1	5.25301E-08	9.91332E-08
ENSGALG00000002316	NUBP2	14	5.27343E-08	9.94895E-08
ENSGALG00000012655	POLR3B	1	5.27352E-08	9.94895E-08
ENSGALG00000047909		3	5.28329E-08	9.96584E-08
ENSGALG00000003861		6	5.29744E-08	9.99098E-08

ENSGALG00000014683	PEX5	1	5.30387E-08	1.00013E-07
ENSGALG00000007408	MPP7	2	5.30453E-08	1.00013E-07
ENSGALG00000009441	TAF1A	3	5.31121E-08	1.00123E-07
ENSGALG00000006174	VSTM4	6	5.42956E-08	1.02338E-07
ENSGALG00000015034	ANKRD29	2	5.46772E-08	1.03042E-07
ENSGALG00000049273	MICOS13	28	5.50539E-08	1.03735E-07
ENSGALG00000054387		1	5.50927E-08	1.03792E-07
ENSGALG00000039652		13	5.51089E-08	1.03807E-07
ENSGALG00000004705	BUD31	14	5.53494E-08	1.04244E-07
ENSGALG00000013726	PAICS	4	5.54755E-08	1.04465E-07
ENSGALG00000010646	STOX2	4	5.57344E-08	1.04937E-07
ENSGALG00000009683	NEK1	4	5.59232E-08	1.05276E-07
ENSGALG00000006842	ACOT8	20	5.66192E-08	1.0657E-07
ENSGALG00000052849		25	5.6731E-08	1.06763E-07
ENSGALG00000007307	USP7	14	5.68034E-08	1.06883E-07
ENSGALG00000008111	MEST	1	5.72391E-08	1.07687E-07
ENSGALG00000009262	FGB	4	5.73493E-08	1.07875E-07
ENSGALG00000037065	SC5D	24	5.73567E-08	1.07875E-07
ENSGALG00000015293	TMEM45A	1	5.79539E-08	1.08981E-07
ENSGALG00000015056	LAMA3	2	5.83374E-08	1.09685E-07
ENSGALG00000015594	TRMT44	4	5.8613E-08	1.10186E-07
ENSGALG00000040498	SLC26A8	26	5.93868E-08	1.11624E-07
ENSGALG00000054271	MED28	4	5.94058E-08	1.11642E-07
ENSGALG00000053575	DAZAP2	33	5.95014E-08	1.11805E-07
ENSGALG00000006050	CYP2R1	5	5.95939E-08	1.11961E-07
ENSGALG00000011036	HNRNPA2B1	2	5.9911E-08	1.1254E-07
ENSGALG00000009153	COPA	25	6.10809E-08	1.1472E-07
ENSGALG00000041372		2	6.15493E-08	1.15569E-07
ENSGALG00000035717	HSPG2	21	6.15519E-08	1.15569E-07
ENSGALG00000006022	PSMA1	5	6.16336E-08	1.15704E-07
ENSGALG00000012754	PAH	1	6.18191E-08	1.16035E-07
ENSGALG00000011418	CCR6	3	6.22108E-08	1.16752E-07
ENSGALG00000017166	RAB39A	1	6.25382E-08	1.17348E-07

ENSGALG00000008812	CCT4	3	6.26507E-08	1.17541E-07
ENSGALG00000030605	B4GALT3	25	6.30389E-08	1.18251E-07
ENSGALG00000039001	FILIP1	3	6.3181E-08	1.185E-07
ENSGALG00000005776		8	6.33303E-08	1.18761E-07
ENSGALG00000035535	GYG1	9	6.33943E-08	1.18863E-07
ENSGALG00000012687	TPMT	2	6.36598E-08	1.19342E-07
ENSGALG00000054505		19	6.39895E-08	1.19942E-07
ENSGALG00000031594		33	6.40951E-08	1.20122E-07
ENSGALG00000053246	OCM2	14	6.42441E-08	1.20382E-07
ENSGALG00000052162	PPM1L	9	6.45878E-08	1.21008E-07
ENSGALG00000029827		14	6.47405E-08	1.21275E-07
ENSGALG00000050406		2	6.47895E-08	1.21348E-07
ENSGALG00000007687	MAP2K1	10	6.50657E-08	1.21847E-07
ENSGALG00000015675		1	6.5766E-08	1.2314E-07
ENSGALG00000000142	CEPT1	26	6.57801E-08	1.23147E-07
ENSGALG00000050719		13	6.62737E-08	1.24052E-07
ENSGALG00000032768	SOX5	1	6.69933E-08	1.2538E-07
ENSGALG00000005682		10	6.72366E-08	1.25816E-07
ENSGALG00000011024	CACHD1	8	6.75575E-08	1.26397E-07
ENSGALG00000017304	PGM2L1	1	6.80866E-08	1.27367E-07
ENSGALG00000051582		11	6.82731E-08	1.27696E-07
ENSGALG00000006356	MKLN1	1	6.85943E-08	1.28278E-07
ENSGALG00000008175	ORC5	1	6.89712E-08	1.28963E-07
ENSGALG00000032679	GCSH	11	6.91342E-08	1.29248E-07
ENSGALG00000033339	FIGNL2	33	6.92091E-08	1.29368E-07
ENSGALG00000031056	NEMP1	33	6.97919E-08	1.30437E-07
ENSGALG00000054657		17	6.98241E-08	1.30477E-07
ENSGALG00000002053	POR	19	6.9895E-08	1.3059E-07
ENSGALG00000016761	LYG2	1	6.99525E-08	1.30677E-07
ENSGALG00000000887	NR0B2	23	7.02469E-08	1.31207E-07
ENSGALG00000037957		3	7.05095E-08	1.31678E-07
ENSGALG00000043080	CCDC85A	3	7.05883E-08	1.31804E-07
ENSGALG00000002722	MST1	12	7.08332E-08	1.32242E-07

ENSGALG00000012106	SCTR	7	7.11614E-08	1.32834E-07
ENSGALG00000012947	CAPRIN2	1	7.14192E-08	1.33295E-07
ENSGALG00000049378		33	7.16799E-08	1.33761E-07
ENSGALG00000004801	PHKA1	4	7.24344E-08	1.35148E-07
ENSGALG00000013031	CECR1	1	7.2997E-08	1.36177E-07
ENSGALG00000026736	OGN	12	7.30167E-08	1.36193E-07
ENSGALG00000006964	SDR42E2	14	7.30995E-08	1.36326E-07
ENSGALG00000036005	TIAM2	3	7.45485E-08	1.39007E-07
ENSGALG00000021232	TGM6	20	7.49138E-08	1.39667E-07
ENSGALG00000007389	ANKRD40	18	7.54504E-08	1.40646E-07
ENSGALG00000005923	NPY4R	6	7.60368E-08	1.41696E-07
ENSGALG00000023661		1	7.60369E-08	1.41696E-07
ENSGALG00000011560	PACRG	3	7.63074E-08	1.42179E-07
ENSGALG00000034562	CAPN10	9	7.65974E-08	1.42697E-07
ENSGALG00000007638	HMOX2	14	7.66739E-08	1.42818E-07
ENSGALG00000025792	FRRS1L	2	7.71906E-08	1.43758E-07
ENSGALG00000050831		11	7.75949E-08	1.44489E-07
ENSGALG00000049622		2	7.76253E-08	1.44524E-07
ENSGALG00000003979	NEK8	19	7.76838E-08	1.4461E-07
ENSGALG00000046754		23	7.83812E-08	1.45887E-07
ENSGALG00000016852	TNFSF13B	1	7.89264E-08	1.46879E-07
ENSGALG00000004197	SUPV3L1	6	7.89519E-08	1.46904E-07
ENSGALG00000010577	ARHGEF38	4	7.90473E-08	1.47059E-07
ENSGALG00000009577	PUS7L	1	7.92597E-08	1.47431E-07
ENSGALG00000026638	RELL2	13	7.94389E-08	1.47742E-07
ENSGALG00000040254	UQCC1	20	8.00319E-08	1.48822E-07
ENSGALG00000001206		13	8.01913E-08	1.49096E-07
ENSGALG00000005897	HMCES	12	8.0336E-08	1.49342E-07
ENSGALG00000002312	TMEM132E	19	8.0501E-08	1.49626E-07
ENSGALG00000007462	CTSZ	20	8.0659E-08	1.49897E-07
ENSGALG00000010106	ERI3	8	8.11381E-08	1.50764E-07
ENSGALG00000047102	SAPCD2	17	8.11624E-08	1.50787E-07
ENSGALG00000036133	KLF15	12	8.14441E-08	1.51287E-07

ENSGALG00000051274	B3GALT2	8	8.24229E-08	1.53082E-07
ENSGALG00000014749	EPHB6	1	8.28677E-08	1.53884E-07
ENSGALG00000006374	TBX6	15	8.31286E-08	1.54346E-07
ENSGALG00000004074	KCNS1	20	8.39958E-08	1.55932E-07
ENSGALG00000040925	RNF215	15	8.42249E-08	1.56333E-07
ENSGALG00000053013		2	8.42972E-08	1.56444E-07
ENSGALG00000034229	ZNF703	22	8.48161E-08	1.57383E-07
ENSGALG00000010444	ATPAF1	8	8.55682E-08	1.58754E-07
ENSGALG00000045581		2	8.57525E-08	1.59072E-07
ENSGALG00000040908	HIP1	19	8.59543E-08	1.59422E-07
ENSGALG00000053999	VPS37D	19	8.65004E-08	1.60411E-07
ENSGALG00000042213	AP1B1	15	8.66275E-08	1.60622E-07
ENSGALG00000002060	TAF15	19	8.67366E-08	1.608E-07
ENSGALG00000039238	SALL1	11	8.73471E-08	1.61907E-07
ENSGALG00000049658		14	8.74592E-08	1.6209E-07
ENSGALG00000041054		4	8.83858E-08	1.63782E-07
ENSGALG00000049684		2	8.86446E-08	1.64237E-07
ENSGALG00000004897	GTSE1	1	8.91205E-08	1.65094E-07
ENSGALG00000006301	KDM8	14	8.92211E-08	1.65255E-07
ENSGALG00000017553	SNX11	27	8.95839E-08	1.65902E-07
ENSGALG00000032699	CDK2	33	8.98233E-08	1.6632E-07
ENSGALG00000053274	PPP1R14B	33	8.99337E-08	1.66499E-07
ENSGALG00000018942	NRGN	24	9.03875E-08	1.67314E-07
ENSGALG00000052526		20	9.09603E-08	1.68348E-07
ENSGALG00000003039	SRM	21	9.12993E-08	1.6895E-07
ENSGALG00000043826	ANXA11	6	9.18328E-08	1.69912E-07
ENSGALG00000013725	LONRF1	4	9.25002E-08	1.7112E-07
ENSGALG00000002113	LCP2	13	9.25531E-08	1.71192E-07
ENSGALG00000037946		33	9.27566E-08	1.71543E-07
ENSGALG00000046741		26	9.30063E-08	1.71978E-07
ENSGALG00000006919	POF1B	4	9.32481E-08	1.72399E-07
ENSGALG00000030140	CCDC3	1	9.40132E-08	1.73788E-07
ENSGALG00000036627	CXCR1	7	9.53851E-08	1.76297E-07

ENSGALG00000010956	TTC21B	7	9.66458E-08	1.786E-07
ENSGALG00000031551	ZDHHC9	4	9.70413E-08	1.79304E-07
ENSGALG00000055127		31	9.73214E-08	1.79794E-07
ENSGALG00000000349	PSMC5	27	9.76991E-08	1.80464E-07
ENSGALG00000048394	AP2S1	32	9.94983E-08	1.8376E-07
ENSGALG00000039169	MLH1	2	1.00025E-07	1.84681E-07
ENSGALG00000005580	TMEM56	8	1.00027E-07	1.84681E-07
ENSGALG00000047515		30	1.00656E-07	1.85813E-07
ENSGALG00000010978	ANGPTL3	8	1.01272E-07	1.86923E-07
ENSGALG00000027620		2	1.01871E-07	1.88E-07
ENSGALG00000007167	CLEC16A	14	1.02179E-07	1.88539E-07
ENSGALG00000011696	JAG2	5	1.03049E-07	1.90117E-07
ENSGALG00000026301		3	1.03136E-07	1.90248E-07
ENSGALG00000037274	SNPH	20	1.03613E-07	1.91098E-07
ENSGALG00000007668	BMP7	20	1.04646E-07	1.92976E-07
ENSGALG00000005203	OLFM3	8	1.05106E-07	1.93793E-07
ENSGALG00000042836	KCNH2	2	1.05679E-07	1.94821E-07
ENSGALG00000010517	TCTN3	6	1.05813E-07	1.95038E-07
ENSGALG00000001942	SPDL1	13	1.06518E-07	1.96308E-07
ENSGALG00000051199		23	1.07828E-07	1.98692E-07
ENSGALG00000010036	FOSL2	3	1.0901E-07	2.00839E-07
ENSGALG00000035406	DSN1	30	1.09094E-07	2.00964E-07
ENSGALG00000040031	SLC35G2	9	1.09414E-07	2.01524E-07
ENSGALG00000011708	MYLK	7	1.09951E-07	2.02483E-07
ENSGALG00000043694	RAPGEF4	7	1.10468E-07	2.03404E-07
ENSGALG00000008880	VTI1A	6	1.10488E-07	2.03409E-07
ENSGALG00000016981	SPERT	1	1.10537E-07	2.03469E-07
ENSGALG00000042259	DOCK1	6	1.11037E-07	2.04359E-07
ENSGALG00000016689	ASMTL	1	1.11756E-07	2.0565E-07
ENSGALG00000032997	EMILIN2	2	1.11802E-07	2.05704E-07
ENSGALG00000006808	TSPAN6	4	1.12715E-07	2.07353E-07
ENSGALG00000003824	CHERP	28	1.13222E-07	2.08255E-07
ENSGALG00000042423		13	1.13358E-07	2.08474E-07

ENSGALG00000029944	FAM222A	15	1.13387E-07	2.08495E-07
ENSGALG00000001901	ARIH1	10	1.14259E-07	2.10066E-07
ENSGALG00000014957	WDR1	4	1.14899E-07	2.11211E-07
ENSGALG00000054561		5	1.15269E-07	2.1186E-07
ENSGALG00000026316	TMEM136	24	1.15389E-07	2.12048E-07
ENSGALG00000005359		6	1.15571E-07	2.1235E-07
ENSGALG00000015645	GRK4	4	1.15755E-07	2.12656E-07
ENSGALG00000006401	OSBPL5	5	1.15973E-07	2.13025E-07
ENSGALG00000043603	CCL5	19	1.16015E-07	2.1307E-07
ENSGALG00000029766	ITGB5	7	1.1636E-07	2.13671E-07
ENSGALG00000048109		1	1.16446E-07	2.13797E-07
ENSGALG00000032780	TTC13	3	1.16611E-07	2.14068E-07
ENSGALG00000040424	SCNN1A	1	1.16978E-07	2.14709E-07
ENSGALG00000003065	NECAB3	20	1.17348E-07	2.15356E-07
ENSGALG00000036019	POLR2K	2	1.17668E-07	2.15911E-07
ENSGALG00000033567		3	1.17983E-07	2.16457E-07
ENSGALG00000030859	GNAS	20	1.19712E-07	2.19595E-07
ENSGALG00000013624		2	1.19731E-07	2.19596E-07
ENSGALG00000041731	CAPN7	2	1.20666E-07	2.21278E-07
ENSGALG00000008813	CROT	2	1.20711E-07	2.21328E-07
ENSGALG00000047861	TAMALIN	33	1.21276E-07	2.2233E-07
ENSGALG00000040297	NBL1	21	1.21604E-07	2.22899E-07
ENSGALG00000048178	SLC27A3	25	1.23752E-07	2.26801E-07
ENSGALG00000005616	ARSI	13	1.25122E-07	2.29278E-07
ENSGALG00000002374	SLC9A9	9	1.25326E-07	2.29617E-07
ENSGALG00000003318	SNRNP35	15	1.25562E-07	2.30014E-07
ENSGALG00000011930		1	1.25581E-07	2.30014E-07
ENSGALG00000048332	GPR135	5	1.26088E-07	2.3091E-07
ENSGALG00000053697	TMEM125	8	1.27422E-07	2.33317E-07
ENSGALG00000051589		20	1.27788E-07	2.33952E-07
ENSGALG00000032588	ARPC1B	14	1.27822E-07	2.33978E-07
ENSGALG00000012821	TUBB2B	2	1.28031E-07	2.34326E-07
ENSGALG00000029006	SYPL1	1	1.28926E-07	2.3593E-07

ENSGALG00000012421	ARHGAP15	7	1.29123E-07	2.36254E-07
ENSGALG00000006609	EPHB1	9	1.29583E-07	2.37061E-07
ENSGALG00000016808		1	1.30299E-07	2.38334E-07
ENSGALG00000042168		8	1.30348E-07	2.38388E-07
ENSGALG00000008597		3	1.31031E-07	2.39602E-07
ENSGALG00000008152	SPATS2L	7	1.31549E-07	2.40513E-07
ENSGALG00000001624	CWC25	27	1.3175E-07	2.40844E-07
ENSGALG00000011455	CL2	4	1.32514E-07	2.42204E-07
ENSGALG00000007365	PARP16	10	1.34412E-07	2.45627E-07
ENSGALG00000012013	SLC25A17	1	1.34427E-07	2.45627E-07
ENSGALG00000048315		22	1.3451E-07	2.45742E-07
ENSGALG00000003217	LITAF	14	1.36484E-07	2.49312E-07
ENSGALG00000027122	APPL2	1	1.36562E-07	2.49416E-07
ENSGALG00000009279	SFMBT2	1	1.37927E-07	2.51871E-07
ENSGALG00000009913	SMARCA5	4	1.397E-07	2.55071E-07
ENSGALG00000004436	CYP3A4	14	1.39958E-07	2.55503E-07
ENSGALG00000017414	JUP	27	1.40076E-07	2.55681E-07
ENSGALG00000000639	LRRN2	26	1.41692E-07	2.58591E-07
ENSGALG00000003595		19	1.4294E-07	2.6083E-07
ENSGALG00000009291	EPHX1	3	1.43269E-07	2.61392E-07
ENSGALG00000004648	PDCD5	11	1.43774E-07	2.62274E-07
ENSGALG00000032975	UBE2QL1	2	1.45423E-07	2.65241E-07
ENSGALG00000007493	NSDHL	4	1.47989E-07	2.69882E-07
ENSGALG00000014477	CD4	1	1.4836E-07	2.70519E-07
ENSGALG00000003748	ANO5	5	1.50691E-07	2.74728E-07
ENSGALG00000039084	EVI2A	19	1.52597E-07	2.7816E-07
ENSGALG00000054718	FLNA	27	1.53062E-07	2.78966E-07
ENSGALG00000011664	RASSF6	4	1.54299E-07	2.8118E-07
ENSGALG00000041863	MRPL13	2	1.55471E-07	2.83272E-07
ENSGALG00000016551	AP1S2	1	1.56673E-07	2.85419E-07
ENSGALG00000005052		4	1.56865E-07	2.85726E-07
ENSGALG00000005303		2	1.57495E-07	2.86819E-07
ENSGALG00000015491	GPR89A	1	1.57512E-07	2.86819E-07

ENSGALG00000002964	DHX37	15	1.59378E-07	2.90175E-07
ENSGALG00000012921	ETV6	1	1.62957E-07	2.96646E-07
ENSGALG00000033277	PPM1M	12	1.63908E-07	2.98333E-07
ENSGALG00000007556	ORAOV1	5	1.64841E-07	2.99986E-07
ENSGALG00000053315		3	1.65378E-07	3.00918E-07
ENSGALG00000027920		13	1.65682E-07	3.01426E-07
ENSGALG00000000853	SLC2A8	17	1.66372E-07	3.02636E-07
ENSGALG00000039708		28	1.68062E-07	3.05665E-07
ENSGALG00000006231	SERGEF	5	1.68591E-07	3.06582E-07
ENSGALG00000034671	WDR74	33	1.68628E-07	3.06603E-07
ENSGALG00000003397	RHOT1	18	1.72034E-07	3.12749E-07
ENSGALG00000004288	HDAC4	7	1.73494E-07	3.15356E-07
ENSGALG00000033957	SPATS2	33	1.73735E-07	3.15747E-07
ENSGALG00000035883	ZMYND10	12	1.74149E-07	3.16454E-07
ENSGALG00000016278	PRIM2	3	1.78074E-07	3.23536E-07
ENSGALG00000008308	BHLHE40	12	1.78761E-07	3.24737E-07
ENSGALG00000050882		11	1.78946E-07	3.25024E-07
ENSGALG00000021634	MAP7D1	23	1.81481E-07	3.2958E-07
ENSGALG00000017235	NOX4	1	1.82098E-07	3.30652E-07
ENSGALG00000038044	NME6	1	1.82598E-07	3.3151E-07
ENSGALG00000047486		5	1.82677E-07	3.31603E-07
ENSGALG00000053074		1	1.83664E-07	3.33346E-07
ENSGALG00000023348	HPDL	8	1.83901E-07	3.33727E-07
ENSGALG00000002201	PPL	14	1.84045E-07	3.33938E-07
ENSGALG00000004614	NPL	8	1.84105E-07	3.33998E-07
ENSGALG00000011806		7	1.85408E-07	3.3631E-07
ENSGALG00000004334	MYLK3	11	1.85952E-07	3.37246E-07
ENSGALG00000000884	CXXC5	13	1.86131E-07	3.37522E-07
ENSGALG00000054364		27	1.88254E-07	3.4132E-07
ENSGALG00000008317	SDSL	15	1.88431E-07	3.41591E-07
ENSGALG00000039170	LHFPL5	26	1.91105E-07	3.46386E-07
ENSGALG00000008486	CACNA2D1	1	1.91778E-07	3.47556E-07
ENSGALG00000007593	JAKMIP2	13	1.92941E-07	3.4961E-07

ENSGALG00000044811		6	1.93263E-07	3.50143E-07
ENSGALG00000009906	UBR2	3	1.9477E-07	3.5282E-07
ENSGALG00000042875	HMGB1	1	1.9494E-07	3.53075E-07
ENSGALG00000007993	DCX	4	1.95416E-07	3.53886E-07
ENSGALG00000040655	FAM20A	18	1.97389E-07	3.57404E-07
ENSGALG00000040626	NDUFA2	13	1.97918E-07	3.58309E-07
ENSGALG00000016720	CD2AP	3	1.98978E-07	3.60174E-07
ENSGALG00000014887	ASF1A	3	2.01134E-07	3.64023E-07
ENSGALG00000009807	FLVCR1	3	2.06838E-07	3.74292E-07
ENSGALG00000024172	THOC3	13	2.0692E-07	3.74385E-07
ENSGALG00000041823	PICK1	1	2.08879E-07	3.77872E-07
ENSGALG00000008827	FERMT1	3	2.09435E-07	3.78822E-07
ENSGALG00000001431	GUCA1A	26	2.11201E-07	3.81961E-07
ENSGALG00000019979	DYNLT1	3	2.11714E-07	3.82832E-07
ENSGALG00000041926	HEXIM1	27	2.12818E-07	3.8477E-07
ENSGALG00000026070		25	2.15095E-07	3.88829E-07
ENSGALG00000043576	AGAP3	2	2.15326E-07	3.89188E-07
ENSGALG00000013117	PIK3C2G	1	2.15459E-07	3.89371E-07
ENSGALG00000048002	CCS	33	2.16265E-07	3.9077E-07
ENSGALG00000016464	VSNL1	3	2.16824E-07	3.91723E-07
ENSGALG00000031343	ECE1	21	2.21018E-07	3.99241E-07
ENSGALG00000023950	CR1L	26	2.23098E-07	4.02907E-07
ENSGALG00000010316		4	2.23114E-07	4.02907E-07
ENSGALG00000038782	ZC3H12D	3	2.23235E-07	4.03065E-07
ENSGALG00000038799		6	2.23391E-07	4.03288E-07
ENSGALG00000008752		5	2.24359E-07	4.04976E-07
ENSGALG00000013756	ZC2HC1B	3	2.25684E-07	4.07307E-07
ENSGALG00000008613	NDUFA1	4	2.27034E-07	4.09682E-07
ENSGALG00000000643	ANKS3	14	2.27177E-07	4.0988E-07
ENSGALG00000010256	TIPARP	9	2.29438E-07	4.13899E-07
ENSGALG00000000293		27	2.30388E-07	4.15551E-07
ENSGALG00000037249	KCNQ5	3	2.3187E-07	4.18162E-07
ENSGALG00000003521	TPM1	10	2.32218E-07	4.18727E-07

ENSGALG00000040369	PFKFB3	1	2.33211E-07	4.20456E-07
ENSGALG00000046914		4	2.3408E-07	4.2196E-07
ENSGALG00000053566		31	2.37091E-07	4.27325E-07
ENSGALG00000000627	DNAJC11	21	2.38072E-07	4.29029E-07
ENSGALG00000039216	COL6A2	7	2.39999E-07	4.32439E-07
ENSGALG00000007057	LRRC28	10	2.40364E-07	4.33032E-07
ENSGALG00000026780	TPGS1	28	2.44859E-07	4.41065E-07
ENSGALG00000011686	SEMA5B	7	2.45732E-07	4.42571E-07
ENSGALG00000011626	COX18	4	2.45795E-07	4.4262E-07
ENSGALG00000002205	FBRSL1	15	2.46877E-07	4.44502E-07
ENSGALG00000013489	CCDC82	1	2.47123E-07	4.4488E-07
ENSGALG00000008539	ALG12	1	2.52323E-07	4.54174E-07
ENSGALG00000013828	GALM	3	2.53755E-07	4.56685E-07
ENSGALG00000003738	EMC3	12	2.54918E-07	4.58709E-07
ENSGALG00000025862	TMEM26	6	2.56108E-07	4.60783E-07
ENSGALG00000048309		1	2.56846E-07	4.62043E-07
ENSGALG00000016231	DDX3X	1	2.58098E-07	4.64227E-07
ENSGALG00000002827	GPS1	18	2.59457E-07	4.66602E-07
ENSGALG00000037246	RAMP3	2	2.61645E-07	4.70467E-07
ENSGALG00000009564	METTL8	7	2.63674E-07	4.74045E-07
ENSGALG00000015854	PGM3	3	2.6556E-07	4.77365E-07
ENSGALG00000026572	RGS5	8	2.66884E-07	4.79675E-07
ENSGALG00000052592	TMEM82	21	2.68122E-07	4.81829E-07
ENSGALG00000008616	DDR1	17	2.72056E-07	4.88826E-07
ENSGALG00000009205		14	2.72364E-07	4.89308E-07
ENSGALG00000010391	MMRN1	4	2.72637E-07	4.89726E-07
ENSGALG00000007536		9	2.73994E-07	4.92091E-07
ENSGALG00000028187	FUNDC1	1	2.74104E-07	4.92217E-07
ENSGALG00000027407		5	2.77309E-07	4.97898E-07
ENSGALG00000002350		14	2.77406E-07	4.97999E-07
ENSGALG00000002442	ANKS4B	14	2.79017E-07	5.00817E-07
ENSGALG00000016529	CDKL5	1	2.79435E-07	5.01494E-07
ENSGALG00000049652		11	2.84587E-07	5.10665E-07

ENSGALG00000016692	CENPQ	3	2.86671E-07	5.14329E-07
ENSGALG00000013064	NSUN2	2	2.87532E-07	5.15797E-07
ENSGALG00000053105		21	2.88529E-07	5.1751E-07
ENSGALG00000048338		33	2.91624E-07	5.22984E-07
ENSGALG00000034473	PARP4	1	2.92222E-07	5.2398E-07
ENSGALG00000051993		2	2.92792E-07	5.24925E-07
ENSGALG00000051290		22	2.95556E-07	5.29801E-07
ENSGALG00000013437		11	2.95827E-07	5.30209E-07
ENSGALG00000003936	PPT1	23	2.96636E-07	5.31582E-07
ENSGALG00000003991	MRTO4	21	2.97727E-07	5.33458E-07
ENSGALG00000026902	P2RX6	15	2.98182E-07	5.34195E-07
ENSGALG00000052541		33	2.99313E-07	5.36143E-07
ENSGALG00000040573	FMO3	8	3.00302E-07	5.37835E-07
ENSGALG00000031497		6	3.07126E-07	5.49976E-07
ENSGALG00000012702	JARID2	2	3.08576E-07	5.52492E-07
ENSGALG00000035629	GRAMD1C	1	3.09156E-07	5.53448E-07
ENSGALG00000013244	ABCC9	1	3.09412E-07	5.53826E-07
ENSGALG00000009586	IRAK4	1	3.10184E-07	5.55126E-07
ENSGALG00000004817	GLOD4	19	3.10726E-07	5.56015E-07
ENSGALG00000003199	MLX	27	3.10963E-07	5.56356E-07
ENSGALG00000043352		28	3.12989E-07	5.599E-07
ENSGALG00000001019	BTBD2	28	3.14215E-07	5.6201E-07
ENSGALG00000042073		7	3.14476E-07	5.62394E-07
ENSGALG00000002010	SNIP1	23	3.15162E-07	5.6354E-07
ENSGALG00000005525	SRSF1	19	3.15611E-07	5.64258E-07
ENSGALG00000005710	CTDSPL	2	3.18573E-07	5.69471E-07
ENSGALG00000008108	PSMC3	5	3.22213E-07	5.75892E-07
ENSGALG00000016363	ACP1	3	3.23033E-07	5.77273E-07
ENSGALG00000006558	DYNC2I1	2	3.28816E-07	5.87522E-07
ENSGALG00000004519	TRPV2	19	3.31692E-07	5.92574E-07
ENSGALG00000024335		18	3.34172E-07	5.96918E-07
ENSGALG00000002881	C26H6ORF125	26	3.36627E-07	6.01215E-07
ENSGALG00000007972	TRPC5	4	3.37787E-07	6.03199E-07

ENSGALG00000008885	PDE1A	7	3.38531E-07	6.04439E-07
ENSGALG00000028354	METRNL	18	3.38995E-07	6.05178E-07
ENSGALG00000021285	UNG	15	3.44978E-07	6.15769E-07
ENSGALG00000008563	ENTPD6	3	3.52211E-07	6.28588E-07
ENSGALG00000005066	DPM2	17	3.54161E-07	6.31976E-07
ENSGALG00000045093		1	3.60159E-07	6.42584E-07
ENSGALG00000033205	PHF20L1	2	3.60317E-07	6.42771E-07
ENSGALG00000010077	RPS3A	4	3.61236E-07	6.44317E-07
ENSGALG00000017394	INSIG1	2	3.63403E-07	6.48088E-07
ENSGALG00000011283		2	3.66026E-07	6.52669E-07
ENSGALG00000050240	TBX21	27	3.66931E-07	6.54188E-07
ENSGALG00000005374	MYO19	19	3.69404E-07	6.58501E-07
ENSGALG00000005985	GDF10	6	3.6984E-07	6.59181E-07
ENSGALG00000034262	VPS25	27	3.73581E-07	6.65752E-07
ENSGALG00000016545	REPS2	1	3.7509E-07	6.68342E-07
ENSGALG00000054223		2	3.77256E-07	6.72104E-07
ENSGALG00000004058	GPR146	14	3.79965E-07	6.76831E-07
ENSGALG00000008716	FLRT3	3	3.83187E-07	6.82472E-07
ENSGALG00000037996	COX7A2L	3	3.91089E-07	6.96443E-07
ENSGALG00000011596	PTPN4	7	3.91827E-07	6.97656E-07
ENSGALG00000001350	RSPRY1	11	3.9407E-07	7.01548E-07
ENSGALG00000007474	PCYT1A	9	3.95121E-07	7.03315E-07
ENSGALG00000016340	EIF2S3	1	3.95587E-07	7.04041E-07
ENSGALG00000046258	CIBAR1	2	3.98289E-07	7.08748E-07
ENSGALG00000000918	CCDC103	27	4.00755E-07	7.13032E-07
ENSGALG00000009615	RRP15	3	4.02212E-07	7.15519E-07
ENSGALG00000042840	OSGIN2	2	4.05578E-07	7.21402E-07
ENSGALG00000005964		4	4.07283E-07	7.24329E-07
ENSGALG00000016972	NUFIP1	1	4.07605E-07	7.24795E-07
ENSGALG00000003052	AGFG1	9	4.08156E-07	7.25671E-07
ENSGALG00000028031	C1QL1	27	4.12214E-07	7.32779E-07
ENSGALG00000009651	LXN	9	4.15983E-07	7.3937E-07
ENSGALG00000015812	SMIM8	3	4.16943E-07	7.40969E-07

ENSGALG00000038399	PLEKHA6	26	4.17099E-07	7.41137E-07
ENSGALG00000020253		4	4.1957E-07	7.4542E-07
ENSGALG00000014646	SYT11	25	4.20991E-07	7.47835E-07
ENSGALG00000009560	MSMO1	4	4.24065E-07	7.53164E-07
ENSGALG00000021126	SLC2A11L1	15	4.24114E-07	7.53164E-07
ENSGALG00000029308	PNPLA3	1	4.26584E-07	7.57439E-07
ENSGALG00000037314		19	4.27171E-07	7.58371E-07
ENSGALG00000037807	NOP2	1	4.28884E-07	7.61303E-07
ENSGALG00000013069	BPGM	1	4.29197E-07	7.61747E-07
ENSGALG00000036847	PLAC9	6	4.30892E-07	7.64601E-07
ENSGALG00000006911	TMC5	14	4.3093E-07	7.64601E-07
ENSGALG00000014234	ATXN10	1	4.31645E-07	7.65757E-07
ENSGALG00000042908	CTC-575C13.4	2	4.32258E-07	7.66732E-07
ENSGALG00000034716	HEY2	3	4.33038E-07	7.68004E-07
ENSGALG00000015775	UBE2J1	3	4.39216E-07	7.78848E-07
ENSGALG00000026385	LYPLAL1	3	4.40299E-07	7.80656E-07
ENSGALG00000052612	RPS27L	10	4.40692E-07	7.81238E-07
ENSGALG00000027983		9	4.40973E-07	7.81623E-07
ENSGALG00000015768	ANKRD6	3	4.43816E-07	7.86548E-07
ENSGALG00000006877	KLHL4	4	4.44883E-07	7.88214E-07
ENSGALG00000016538	RAI2	1	4.44885E-07	7.88214E-07
ENSGALG00000031797	JAGN1	12	4.46074E-07	7.90204E-07
ENSGALG00000010469	CYP4B7	8	4.46855E-07	7.91406E-07
ENSGALG00000036234	RFWD3	11	4.46882E-07	7.91406E-07
ENSGALG00000002570		13	4.47869E-07	7.9304E-07
ENSGALG00000054625		4	4.50906E-07	7.98301E-07
ENSGALG00000033062	PDE6D	9	4.51881E-07	7.9991E-07
ENSGALG00000040541	SLC5A6	3	4.54983E-07	8.05284E-07
ENSGALG00000050467		8	4.59165E-07	8.1257E-07
ENSGALG00000004268	NIPAL3	23	4.59692E-07	8.13383E-07
ENSGALG00000009935		27	4.60331E-07	8.14396E-07
ENSGALG00000021539	PRPSAP1	18	4.62719E-07	8.18501E-07
ENSGALG00000033781	FEN1	5	4.63459E-07	8.19692E-07

ENSGALG00000036806	GDF11	33	4.64582E-07	8.21558E-07
ENSGALG00000012798	SSR1	2	4.72371E-07	8.35211E-07
ENSGALG00000042900	GATAD2A	28	4.74673E-07	8.39161E-07
ENSGALG00000016921	UCHL3	1	4.76642E-07	8.4252E-07
ENSGALG00000048873	C3orf14	12	4.76913E-07	8.42876E-07
ENSGALG00000018976	LYSMD2	10	4.8346E-07	8.54323E-07
ENSGALG00000051082		3	4.88236E-07	8.62637E-07
ENSGALG00000028897	WDR25	5	4.89828E-07	8.65325E-07
ENSGALG00000054449	SDHC	25	4.94193E-07	8.72909E-07
ENSGALG00000001714	PUSL1	21	4.95321E-07	8.74775E-07
ENSGALG00000003135	ANK3	6	4.95916E-07	8.75698E-07
ENSGALG00000042863	SMIM4	12	4.97463E-07	8.78303E-07
ENSGALG00000050897	TNIK	9	4.99987E-07	8.82632E-07
ENSGALG00000008681	NSMF	17	5.06535E-07	8.94062E-07
ENSGALG00000005259	VIPR1	2	5.06694E-07	8.94214E-07
ENSGALG00000051871	INHBC	33	5.06997E-07	8.94618E-07
ENSGALG00000015029	EVC2	4	5.09989E-07	8.99768E-07
ENSGALG00000027046	INTS3	25	5.10336E-07	9.00251E-07
ENSGALG00000001370	ADAMTS8	24	5.10995E-07	9.01283E-07
ENSGALG00000025906	HS6ST3	1	5.13593E-07	9.05733E-07
ENSGALG00000053973		33	5.14033E-07	9.06378E-07
ENSGALG00000026741	CETN2	4	5.14173E-07	9.06493E-07
ENSGALG00000011406	NTN4	1	5.14475E-07	9.06896E-07
ENSGALG00000042055	RXRA	17	5.16802E-07	9.10865E-07
ENSGALG00000006087	GPC3	4	5.21425E-07	9.18881E-07
ENSGALG00000001174	RCC1L	19	5.23693E-07	9.22744E-07
ENSGALG00000038536	SH3BGRL3	23	5.24789E-07	9.24541E-07
ENSGALG00000028659	BLEC1	16	5.27715E-07	9.29563E-07
ENSGALG00000003942	LONP2	11	5.32061E-07	9.37082E-07
ENSGALG00000031035	PCMTD1	2	5.33471E-07	9.39431E-07
ENSGALG00000035923	MTMR11	25	5.34366E-07	9.40869E-07
ENSGALG00000030247	TMOD4	25	5.35461E-07	9.42662E-07
ENSGALG00000033513	ELL	28	5.37791E-07	9.46627E-07

ENSGALG00000016070	WRB	1	5.39615E-07	9.497E-07
ENSGALG00000004398	CARD11	14	5.42454E-07	9.54559E-07
ENSGALG00000045169	LRRC10B	5	5.44257E-07	9.57594E-07
ENSGALG00000016067	HMGN1	1	5.47427E-07	9.63032E-07
ENSGALG00000039432	P2RY13	9	5.51042E-07	9.69252E-07
ENSGALG00000021692		28	5.52178E-07	9.71111E-07
ENSGALG00000005467	HSD17B2	11	5.53373E-07	9.73072E-07
ENSGALG00000043513	TEAD3	26	5.54035E-07	9.74096E-07
ENSGALG00000030254	METAP1D	7	5.80799E-07	1.021E-06
ENSGALG00000024049	RAB33A	4	5.81185E-07	1.02153E-06
ENSGALG00000015519	ROBO2	1	5.93224E-07	1.04249E-06
ENSGALG00000007592	ZDHHC16	6	5.93277E-07	1.04249E-06
ENSGALG00000017012	KCNRG	1	6.01009E-07	1.05592E-06
ENSGALG00000002225	RFFL	19	6.0509E-07	1.06294E-06
ENSGALG00000002925	ETFA	10	6.05891E-07	1.06419E-06
ENSGALG00000000907	KARS	11	6.06109E-07	1.06442E-06
ENSGALG00000012000	METTL14	4	6.08088E-07	1.06774E-06
ENSGALG00000051278		28	6.09405E-07	1.0699E-06
ENSGALG00000033966	TENM4	1	6.12327E-07	1.07488E-06
ENSGALG00000008060	PRKAR2B	1	6.13839E-07	1.07738E-06
ENSGALG00000003045	E2F1	20	6.16647E-07	1.08215E-06
ENSGALG00000015471	CCNC	3	6.18148E-07	1.08463E-06
ENSGALG00000053867		2	6.21533E-07	1.09041E-06
ENSGALG00000045212	PLCD3	27	6.28674E-07	1.10278E-06
ENSGALG00000035299	TOMM20	3	6.37485E-07	1.11807E-06
ENSGALG00000009723	FMN1	5	6.37816E-07	1.11849E-06
ENSGALG00000053742		28	6.42338E-07	1.12626E-06
ENSGALG00000003265	PTP4A2	23	6.44212E-07	1.12939E-06
ENSGALG00000048440		14	6.47215E-07	1.13449E-06
ENSGALG00000033950	DERL1	2	6.52127E-07	1.14293E-06
ENSGALG00000003806	RHOA	12	6.56651E-07	1.1507E-06
ENSGALG00000009298		9	6.57063E-07	1.15125E-06
ENSGALG00000036642	RAD9A	5	6.61624E-07	1.15908E-06

ENSGALG00000001475	STMN1	23	6.69119E-07	1.17204E-06
ENSGALG00000015783	IMMT	4	6.76035E-07	1.18398E-06
ENSGALG00000043499	EGFL7	17	6.79484E-07	1.18985E-06
ENSGALG00000010237	NPC2	5	6.83151E-07	1.1961E-06
ENSGALG00000037777	MSC	2	6.84407E-07	1.19813E-06
ENSGALG00000012574	WDSUB1	7	7.07498E-07	1.23838E-06
ENSGALG00000045529	RAB15	5	7.10307E-07	1.24311E-06
ENSGALG00000016628	NLGN4Y	1	7.11889E-07	1.2457E-06
ENSGALG00000000776	BRPF3	26	7.15435E-07	1.25173E-06
ENSGALG00000035677	RPL35	17	7.16876E-07	1.25407E-06
ENSGALG00000016569	FANCB	1	7.18117E-07	1.25606E-06
ENSGALG00000013131	C18orf21	2	7.20656E-07	1.26032E-06
ENSGALG00000041163	ABHD17A	28	7.23065E-07	1.26435E-06
ENSGALG00000005135	SERPINE2	9	7.28229E-07	1.2732E-06
ENSGALG00000019336	CEP83	1	7.35316E-07	1.28541E-06
ENSGALG00000023725	IER5	8	7.35633E-07	1.28578E-06
ENSGALG00000014501	FGFBP2	4	7.39962E-07	1.29316E-06
ENSGALG00000001191	MEOX1	27	7.41962E-07	1.29647E-06
ENSGALG00000004083	GRIA1	13	7.4471E-07	1.30108E-06
ENSGALG00000006921	DRG1	15	7.47181E-07	1.30521E-06
ENSGALG00000015961	SUCLG1	4	7.55567E-07	1.31967E-06
ENSGALG00000051798		2	7.58933E-07	1.32536E-06
ENSGALG00000030376	DHRS3	21	7.61131E-07	1.32901E-06
ENSGALG00000006181	NUB1	2	7.66962E-07	1.339E-06
ENSGALG00000017099	RASL11A	1	7.67279E-07	1.33933E-06
ENSGALG00000040808		1	7.67369E-07	1.33933E-06
ENSGALG00000008091	PTPMT1	5	7.73499E-07	1.34974E-06
ENSGALG00000008518		17	7.73555E-07	1.34974E-06
ENSGALG00000011974	RPL7L1	4	7.74552E-07	1.35128E-06
ENSGALG00000008058	PAK3	4	7.79931E-07	1.36047E-06
ENSGALG00000034009	AMPD2	26	7.80654E-07	1.36154E-06
ENSGALG00000011689	PDIA5	7	7.84016E-07	1.36721E-06
ENSGALG00000004106	DHCR7	5	7.84355E-07	1.3676E-06

ENSGALG00000005253	C1QTNF8	14	7.87336E-07	1.3726E-06
ENSGALG00000005977	BTBD8	8	7.87623E-07	1.37291E-06
ENSGALG00000026163	TNFSF11	1	7.89982E-07	1.37682E-06
ENSGALG00000047441	TRARG1	19	7.90293E-07	1.37717E-06
ENSGALG00000008393	CREB3L1	5	7.90553E-07	1.37742E-06
ENSGALG00000003878	PIN4	4	7.91076E-07	1.37814E-06
ENSGALG00000028822	RNF152	2	7.91753E-07	1.37912E-06
ENSGALG00000047289	TNFAIP8L3	10	8.05669E-07	1.40316E-06
ENSGALG00000026948	ADD2	22	8.1281E-07	1.41539E-06
ENSGALG00000017211	MRE11	1	8.17821E-07	1.42392E-06
ENSGALG00000027632	ACYP2	3	8.25584E-07	1.43723E-06
ENSGALG00000008254	ANAPC1	3	8.28668E-07	1.44239E-06
ENSGALG00000003539	KIAA1522	23	8.3634E-07	1.45554E-06
ENSGALG00000002210	PPIB	10	8.37286E-07	1.45698E-06
ENSGALG00000049721		26	8.39167E-07	1.46004E-06
ENSGALG00000010360	NSUN4	8	8.43396E-07	1.46719E-06
ENSGALG00000016992	HTR2A	1	8.58976E-07	1.49408E-06
ENSGALG00000045842		22	8.59629E-07	1.49485E-06
ENSGALG00000039155	ZHX3	20	8.59662E-07	1.49485E-06
ENSGALG00000045473	CHIR-B6	31	8.63715E-07	1.50168E-06
ENSGALG00000033087	STAT4	7	8.67686E-07	1.50837E-06
ENSGALG00000002558	ASL1	19	8.68664E-07	1.50985E-06
ENSGALG00000007477	NELFCD	20	8.68812E-07	1.5099E-06
ENSGALG00000010197	COQ6	5	8.70724E-07	1.513E-06
ENSGALG00000051595		31	8.74107E-07	1.51866E-06
ENSGALG00000004113		4	8.8178E-07	1.53178E-06
ENSGALG00000021027	PDCD7	10	8.83755E-07	1.53499E-06
ENSGALG00000003172	SLC16A9	6	8.8742E-07	1.54113E-06
ENSGALG00000037289	ATL2	3	8.88344E-07	1.54252E-06
ENSGALG00000031872	AGMAT	21	8.88919E-07	1.5433E-06
ENSGALG00000002719	CSNK1D	18	8.9E-07	1.54496E-06
ENSGALG00000016755	CRACDL	1	8.90179E-07	1.54505E-06
ENSGALG00000012418	KYNU	7	8.95927E-07	1.5548E-06

ENSGALG00000051773	JHY	24	8.98383E-07	1.55884E-06
ENSGALG00000055075	FMNL3	33	9.02459E-07	1.56569E-06
ENSGALG00000013537	TPM3	25	9.02966E-07	1.56635E-06
ENSGALG00000033051	CAMK1D	1	9.04045E-07	1.568E-06
ENSGALG00000013253	INSRR	25	9.04376E-07	1.56835E-06
ENSGALG00000008470	WDR12	7	9.18599E-07	1.59279E-06
ENSGALG00000010638	CASP3	4	9.26847E-07	1.60686E-06
ENSGALG00000011475	RPL37A	7	9.29396E-07	1.61105E-06
ENSGALG00000005931	RUVBL1	12	9.32862E-07	1.61683E-06
ENSGALG00000032736	MGLL	12	9.34612E-07	1.61963E-06
ENSGALG00000011094	PDE4B	8	9.36622E-07	1.62288E-06
ENSGALG00000011657	EAF2	7	9.36752E-07	1.62288E-06
ENSGALG00000015486	COQ3	3	9.38932E-07	1.62642E-06
ENSGALG00000039461	TNFRSF1A	1	9.39903E-07	1.62788E-06
ENSGALG00000010435	RASGRP3	3	9.47995E-07	1.64161E-06
ENSGALG00000010930	STK39	7	9.48105E-07	1.64161E-06
ENSGALG00000000678	CITED4	23	9.54622E-07	1.65266E-06
ENSGALG00000015299	TFG	1	9.57013E-07	1.65657E-06
ENSGALG00000011808	CCR9	2	9.59285E-07	1.66026E-06
ENSGALG00000007350		5	9.59481E-07	1.66037E-06
ENSGALG00000029586	RNF141	5	9.66362E-07	1.67204E-06
ENSGALG00000011635	TFCP2L1	7	9.85774E-07	1.70538E-06
ENSGALG00000001645	KCNT1	17	9.87433E-07	1.70801E-06
ENSGALG00000019934		3	9.88228E-07	1.70914E-06
ENSGALG00000015360	KIAA1524	1	9.89574E-07	1.71123E-06
ENSGALG00000004777	AIFM2	6	9.90823E-07	1.71315E-06
ENSGALG00000007015	CD40	20	9.95323E-07	1.72068E-06
ENSGALG00000016326		1	1.00039E-06	1.7292E-06
ENSGALG00000005001	CNBP	12	1.00555E-06	1.73787E-06
ENSGALG00000008270	CALHM2	6	1.01167E-06	1.7482E-06
ENSGALG00000016709	RCAN2	3	1.01193E-06	1.7484E-06
ENSGALG00000019932		3	1.01503E-06	1.7535E-06
ENSGALG00000017326	ARHGEF17	1	1.01978E-06	1.76147E-06

ENSGALG00000047431		31	1.02439E-06	1.76918E-06
ENSGALG00000012965	FAM105A	2	1.02586E-06	1.77146E-06
ENSGALG00000054822		11	1.02856E-06	1.77588E-06
ENSGALG00000008072	CHRDL1	4	1.03136E-06	1.78045E-06
ENSGALG00000043148	TMEM101	27	1.03482E-06	1.78618E-06
ENSGALG00000038290	P4HB	18	1.03777E-06	1.79101E-06
ENSGALG00000005934		2	1.04635E-06	1.80557E-06
ENSGALG00000039764		1	1.04685E-06	1.80618E-06
ENSGALG00000050725	SVBP	21	1.07682E-06	1.85763E-06
ENSGALG00000031512	PAN2	33	1.08249E-06	1.86714E-06
ENSGALG00000004373	KCNJ16	18	1.08553E-06	1.87211E-06
ENSGALG00000039995		13	1.08717E-06	1.87468E-06
ENSGALG00000000972	PBX3	17	1.08933E-06	1.87814E-06
ENSGALG00000001486	ZAP70	28	1.1105E-06	1.91436E-06
ENSGALG00000053073	NTPCR	3	1.11065E-06	1.91436E-06
ENSGALG00000013743	ENPP7	18	1.11601E-06	1.92333E-06
ENSGALG00000033920		17	1.11944E-06	1.92897E-06
ENSGALG00000023626	NTN1	18	1.12408E-06	1.93668E-06
ENSGALG00000015880	HMGN2P46	3	1.1275E-06	1.9423E-06
ENSGALG00000005515	CRYBB3	15	1.13248E-06	1.95061E-06
ENSGALG00000001779	KIAA0895L	11	1.13858E-06	1.96084E-06
ENSGALG00000048639		3	1.14305E-06	1.96825E-06
ENSGALG00000012424	GTDC1	7	1.15077E-06	1.98127E-06
ENSGALG00000030396	HMGA1	26	1.15982E-06	1.99657E-06
ENSGALG00000007710		4	1.16538E-06	2.00585E-06
ENSGALG00000006626	PDE6C	6	1.17128E-06	2.01573E-06
ENSGALG00000006950	LIMK2	15	1.17337E-06	2.01905E-06
ENSGALG00000009744	FAM98B	5	1.17671E-06	2.02449E-06
ENSGALG00000010238	NAP1L1	1	1.182E-06	2.03332E-06
ENSGALG00000026781	ALOX5AP	1	1.18264E-06	2.03413E-06
ENSGALG00000034919	CDK2AP1	15	1.18714E-06	2.04158E-06
ENSGALG00000017025	CKAP2	1	1.18861E-06	2.04382E-06
ENSGALG00000048691		30	1.18963E-06	2.04528E-06

ENSGALG00000040360	DUSP1	13	1.19048E-06	2.04646E-06
ENSGALG00000015271		1	1.19381E-06	2.05189E-06
ENSGALG00000010301	EIF2B2	5	1.19436E-06	2.05255E-06
ENSGALG00000049511	AMIGO3	12	1.20791E-06	2.07554E-06
ENSGALG00000012241	KLHDC1	5	1.21453E-06	2.08662E-06
ENSGALG00000043583	SCG3	10	1.21729E-06	2.09108E-06
ENSGALG00000016358	PTCHD1	1	1.21917E-06	2.094E-06
ENSGALG00000031734	AQP11	1	1.22432E-06	2.10255E-06
ENSGALG00000037781	GSR	4	1.22458E-06	2.1027E-06
ENSGALG00000011203	HPSE	4	1.22787E-06	2.10806E-06
ENSGALG00000000226	TMEM9	26	1.23361E-06	2.11762E-06
ENSGALG00000004370	MAP2K6	18	1.23614E-06	2.12167E-06
ENSGALG00000010659	ING2	4	1.23868E-06	2.12572E-06
ENSGALG00000048989		31	1.23984E-06	2.12741E-06
ENSGALG00000001017	WRAP73	21	1.24435E-06	2.13485E-06
ENSGALG00000006172	ABCC8	5	1.24728E-06	2.13957E-06
ENSGALG00000012505	LRFN5	5	1.25897E-06	2.15932E-06
ENSGALG00000035589		19	1.26142E-06	2.16322E-06
ENSGALG00000039480		16	1.26569E-06	2.17023E-06
ENSGALG00000051337		18	1.26684E-06	2.1719E-06
ENSGALG00000003169	CBFB	11	1.27633E-06	2.18787E-06
ENSGALG00000008051	MTMR3	15	1.27686E-06	2.18847E-06
ENSGALG00000036836	SOSTDC1	2	1.28128E-06	2.19574E-06
ENSGALG00000009864	TRAF5	3	1.28357E-06	2.19934E-06
ENSGALG00000054550		1	1.28563E-06	2.20258E-06
ENSGALG00000054619	GAB2	1	1.29413E-06	2.21681E-06
ENSGALG00000049122		2	1.29557E-06	2.21898E-06
ENSGALG00000028294		9	1.30209E-06	2.22983E-06
ENSGALG00000015450	RAB2A	2	1.30893E-06	2.24123E-06
ENSGALG00000040120		5	1.31676E-06	2.25432E-06
ENSGALG00000036798	COL4A1	1	1.32349E-06	2.26552E-06
ENSGALG00000052643	C15orf48	10	1.33887E-06	2.29134E-06
ENSGALG00000052865		30	1.33895E-06	2.29134E-06

ENSGALG00000005933	SLC5A11	14	1.34036E-06	2.29344E-06
ENSGALG00000000638	UTP4	11	1.3461E-06	2.30293E-06
ENSGALG00000003722	THG1L	13	1.34629E-06	2.30293E-06
ENSGALG00000037097	GRK3	15	1.35148E-06	2.31149E-06
ENSGALG00000043929		32	1.36056E-06	2.3267E-06
ENSGALG00000015066	WASF1	3	1.37584E-06	2.35249E-06
ENSGALG00000038957	NAE1	11	1.38268E-06	2.36386E-06
ENSGALG00000021273	TMPRSS13	24	1.39869E-06	2.3909E-06
ENSGALG00000015802	N6AMT1	1	1.41461E-06	2.41777E-06
ENSGALG00000006439	ARHGEF6	4	1.41684E-06	2.42124E-06
ENSGALG00000038504	MID1	1	1.41748E-06	2.42198E-06
ENSGALG00000044814	CCDC14	7	1.42095E-06	2.42757E-06
ENSGALG00000033532	RAD23A	30	1.42244E-06	2.42978E-06
ENSGALG00000043223	COL26A1	19	1.42448E-06	2.43293E-06
ENSGALG00000034216	TK2	11	1.44117E-06	2.46109E-06
ENSGALG00000051777		8	1.44325E-06	2.46429E-06
ENSGALG00000048448		6	1.46104E-06	2.49433E-06
ENSGALG00000008497	SLC38A2	1	1.46139E-06	2.49457E-06
ENSGALG00000007030		14	1.46702E-06	2.50383E-06
ENSGALG00000039163	ARHGEF11	25	1.47804E-06	2.52228E-06
ENSGALG00000038918		4	1.48257E-06	2.52967E-06
ENSGALG00000000374	RNPEP	26	1.48459E-06	2.53276E-06
ENSGALG00000012834	AKR1D1	1	1.48919E-06	2.54024E-06
ENSGALG00000032530	SASH3	4	1.49104E-06	2.54306E-06
ENSGALG00000052373		31	1.50832E-06	2.57215E-06
ENSGALG00000041344	FABP5	2	1.51698E-06	2.58656E-06
ENSGALG00000021092		9	1.5199E-06	2.59117E-06
ENSGALG00000017085	SLC7A1	1	1.54506E-06	2.6337E-06
ENSGALG00000039263	OXLD1	18	1.55451E-06	2.64944E-06
ENSGALG00000034645	USMG5	6	1.56978E-06	2.6751E-06
ENSGALG00000036189	ANGPT2	3	1.57273E-06	2.67976E-06
ENSGALG00000029596	HAS2	2	1.60183E-06	2.72895E-06
ENSGALG00000000419	LAMTOR5	26	1.60375E-06	2.73184E-06

ENSGALG00000040646		4	1.60673E-06	2.73653E-06
ENSGALG00000029212	PADI2	21	1.61142E-06	2.74414E-06
ENSGALG00000006649	TMEM41A	9	1.62565E-06	2.76799E-06
ENSGALG00000043938	LIG1	31	1.6476E-06	2.80497E-06
ENSGALG00000030781	CA2	2	1.65893E-06	2.82387E-06
ENSGALG00000001031		19	1.65975E-06	2.82486E-06
ENSGALG00000008291	AP3S2	10	1.67329E-06	2.84752E-06
ENSGALG00000019211	MAEL	1	1.67363E-06	2.84769E-06
ENSGALG00000011571	AGPAT4	3	1.67597E-06	2.85127E-06
ENSGALG00000042005		3	1.6791E-06	2.8561E-06
ENSGALG00000054130	PPOX	25	1.67928E-06	2.8561E-06
ENSGALG00000012412	MTHFD1L	3	1.6822E-06	2.86067E-06
ENSGALG00000045427	UPP2	7	1.68454E-06	2.86426E-06
ENSGALG00000012809	ECI2	2	1.70055E-06	2.89108E-06
ENSGALG00000038458	LOXL1	10	1.71329E-06	2.91232E-06
ENSGALG00000009429	RXFP1	4	1.71448E-06	2.91395E-06
ENSGALG00000010762	OPN3	3	1.74265E-06	2.96141E-06
ENSGALG00000003464		27	1.74986E-06	2.97324E-06
ENSGALG00000034274	GRAMD4	1	1.76436E-06	2.99747E-06
ENSGALG00000020322		4	1.76686E-06	3.0013E-06
ENSGALG00000015851	RWDD2A	3	1.77233E-06	3.01018E-06
ENSGALG00000030831	FBXW5	17	1.77644E-06	3.01641E-06
ENSGALG00000039990	TNC	17	1.7765E-06	3.01641E-06
ENSGALG00000016142	MX1	1	1.7838E-06	3.02838E-06
ENSGALG00000038634	KIT	4	1.79155E-06	3.04111E-06
ENSGALG00000019719	KRT24	27	1.80787E-06	3.06839E-06
ENSGALG00000023472		11	1.8102E-06	3.07193E-06
ENSGALG00000033880	CRISPLD1	2	1.82124E-06	3.09023E-06
ENSGALG00000015123	ZDHHC23	1	1.8254E-06	3.09685E-06
ENSGALG00000011116	VRK1	5	1.8356E-06	3.11374E-06
ENSGALG00000005263	SOX8	14	1.8672E-06	3.16689E-06
ENSGALG00000036293	EBAG9	2	1.87178E-06	3.17422E-06
ENSGALG00000008166	TLR15	3	1.87256E-06	3.1751E-06

ENSGALG00000016549	CTPS2	1	1.87508E-06	3.17893E-06
ENSGALG00000006891	ITIH5	1	1.8759E-06	3.17988E-06
ENSGALG00000054158		31	1.88238E-06	3.19042E-06
ENSGALG00000016125	LCA5L	1	1.90192E-06	3.22308E-06
ENSGALG00000005969	ANTXRL	6	1.90826E-06	3.23338E-06
ENSGALG00000009448	IFRD1	1	1.9163E-06	3.24656E-06
ENSGALG00000007101	ZPR1	24	1.93697E-06	3.28112E-06
ENSGALG00000047117		31	1.93745E-06	3.28147E-06
ENSGALG00000035596	NT5DC2	12	1.94429E-06	3.2926E-06
ENSGALG00000011662	NUP205	1	1.94557E-06	3.2943E-06
ENSGALG00000011680	EPHA5	4	1.97574E-06	3.34494E-06
ENSGALG00000012273	SOS2	5	1.99366E-06	3.37481E-06
ENSGALG00000036263		12	2.00871E-06	3.3998E-06
ENSGALG00000006910	IMPDH2	12	2.01191E-06	3.40475E-06
ENSGALG00000009837	DTL	3	2.01353E-06	3.40702E-06
ENSGALG00000017053	SPART	1	2.03101E-06	3.43612E-06
ENSGALG00000011220	SLC25A29	5	2.03618E-06	3.44439E-06
ENSGALG00000003947	HNRNPH3	6	2.06691E-06	3.49589E-06
ENSGALG00000008401	ASB13	1	2.07343E-06	3.50642E-06
ENSGALG00000012404	ERO1A	5	2.0935E-06	3.53988E-06
ENSGALG00000049293		27	2.0976E-06	3.54631E-06
ENSGALG00000007675	CXCR5	24	2.1292E-06	3.59924E-06
ENSGALG00000009855	TBC1D9	4	2.1339E-06	3.60668E-06
ENSGALG00000050329		16	2.14832E-06	3.63031E-06
ENSGALG00000009870	IL15	4	2.14847E-06	3.63031E-06
ENSGALG00000016736	CYFIP1	1	2.15057E-06	3.63335E-06
ENSGALG00000011019	ROR1	8	2.17273E-06	3.67028E-06
ENSGALG00000053262	HIST2H4B	1	2.18975E-06	3.69852E-06
ENSGALG00000041205		4	2.19177E-06	3.70142E-06
ENSGALG00000031590	B3GAT2	3	2.20093E-06	3.71637E-06
ENSGALG00000013929	PDGFRA	4	2.22081E-06	3.74943E-06
ENSGALG00000016514		3	2.222E-06	3.75092E-06
ENSGALG00000015203		28	2.23813E-06	3.77763E-06

ENSGALG00000014387	MRPL10	27	2.25006E-06	3.79723E-06
ENSGALG00000047273	DRAP1	33	2.25822E-06	3.81048E-06
ENSGALG00000008452	IFT122	12	2.26163E-06	3.8157E-06
ENSGALG00000016894	DNAJC3	1	2.26209E-06	3.81595E-06
ENSGALG00000030164	ECH1	32	2.26634E-06	3.82258E-06
ENSGALG00000051739		3	2.26844E-06	3.82559E-06
ENSGALG00000046969		7	2.27907E-06	3.84299E-06
ENSGALG00000014078	DCUN1D4	4	2.2857E-06	3.85363E-06
ENSGALG00000042309	CHURC1	5	2.29687E-06	3.87193E-06
ENSGALG00000016454	E2F6	3	2.31808E-06	3.90716E-06
ENSGALG00000011421	HAL	1	2.32187E-06	3.913E-06
ENSGALG00000002576	ASL2	19	2.33121E-06	3.9282E-06
ENSGALG00000016904	SLITRK6	1	2.33257E-06	3.92994E-06
ENSGALG00000004042	NUFIP2	19	2.35511E-06	3.96738E-06
ENSGALG00000049287	ATP6V0E1	13	2.35841E-06	3.97238E-06
ENSGALG00000049399	DCAF15	30	2.36312E-06	3.97978E-06
ENSGALG00000010163	LGR5	1	2.37921E-06	4.00631E-06
ENSGALG00000006190	FHL1	4	2.38456E-06	4.01477E-06
ENSGALG00000028774	S100A10	25	2.38861E-06	4.02103E-06
ENSGALG00000016174	LMBRD1	3	2.42797E-06	4.08672E-06
ENSGALG00000029724	MTURN	2	2.43268E-06	4.09409E-06
ENSGALG00000031718	CSNK2A2	11	2.43408E-06	4.09589E-06
ENSGALG00000043555	NPNT	4	2.4538E-06	4.1285E-06
ENSGALG00000002624	BMS1	6	2.4665E-06	4.14929E-06
ENSGALG00000006617	BCCIP	6	2.47139E-06	4.15693E-06
ENSGALG00000027587		28	2.52783E-06	4.25129E-06
ENSGALG00000011835	FGF2	4	2.53064E-06	4.25543E-06
ENSGALG00000036680		10	2.53386E-06	4.26025E-06
ENSGALG00000015608	ADRA2C	4	2.55641E-06	4.29757E-06
ENSGALG00000000966	NPHP4	21	2.57101E-06	4.32152E-06
ENSGALG00000042538	BAIAP2	18	2.58956E-06	4.3521E-06
ENSGALG00000036738	IGSF3	1	2.59085E-06	4.35366E-06
ENSGALG00000037256	GTPBP3	28	2.59807E-06	4.3652E-06

ENSGALG00000004958	BTK	4	2.60342E-06	4.37359E-06
ENSGALG00000052825	BIN3	22	2.61922E-06	4.39952E-06
ENSGALG00000008574	CLNS1A	1	2.62517E-06	4.40891E-06
ENSGALG00000012915	MCM4	2	2.62779E-06	4.4127E-06
ENSGALG00000031238	SLIRP	5	2.63421E-06	4.42287E-06
ENSGALG00000010440	MKNK1	8	2.63599E-06	4.42525E-06
ENSGALG00000009236	AGPS	7	2.6426E-06	4.43574E-06
ENSGALG00000004494	CCNE1	11	2.706E-06	4.54154E-06
ENSGALG00000026674	CIB2	10	2.72095E-06	4.56599E-06
ENSGALG00000002682	BRD3	17	2.72485E-06	4.57191E-06
ENSGALG00000012103		5	2.74002E-06	4.59673E-06
ENSGALG00000007926	ATP5PD	18	2.74247E-06	4.60021E-06
ENSGALG00000011214	SERPINH1	1	2.78924E-06	4.67802E-06
ENSGALG00000043219	HOXB3	27	2.7947E-06	4.68654E-06
ENSGALG00000003391	EIF2AK1	14	2.80718E-06	4.70681E-06
ENSGALG00000006519	KIAA1024	10	2.81061E-06	4.71192E-06
ENSGALG00000030858	FRMD5	10	2.86061E-06	4.79508E-06
ENSGALG00000009939	SPOP	27	2.88047E-06	4.8277E-06
ENSGALG00000010551	CDC42EP3	3	2.88591E-06	4.83617E-06
ENSGALG00000011900	NAGA	1	2.88751E-06	4.83818E-06
ENSGALG00000031128	SENP2	9	2.89035E-06	4.84227E-06
ENSGALG00000016501	SF3B6	3	2.89822E-06	4.85479E-06
ENSGALG00000014811	SMCHD1	2	2.9009E-06	4.85861E-06
ENSGALG00000035726	FLT3	1	2.90432E-06	4.86368E-06
ENSGALG00000012552	TOM1	1	2.90718E-06	4.86779E-06
ENSGALG00000023122	SULT1B	4	2.9352E-06	4.91404E-06
ENSGALG00000004860	RASD1	14	2.95278E-06	4.94279E-06
ENSGALG00000033789	HSP90B1	1	2.95869E-06	4.952E-06
ENSGALG00000001252	CREB3L3	28	2.96323E-06	4.95892E-06
ENSGALG00000006305	DTYMK	9	2.97813E-06	4.98316E-06
ENSGALG00000048205		30	3.02343E-06	5.05827E-06
ENSGALG00000005747		4	3.02551E-06	5.06105E-06
ENSGALG00000008963	DBF4	2	3.0525E-06	5.1055E-06

ENSGALG00000042939	HMBS	24	3.06421E-06	5.12438E-06
ENSGALG00000039120	NBEAL2	2	3.0768E-06	5.14473E-06
ENSGALG00000005733	WNT8B	6	3.08123E-06	5.15144E-06
ENSGALG00000041814	DYNC2H1	1	3.08181E-06	5.1517E-06
ENSGALG00000038948	KDM5B	26	3.11027E-06	5.19857E-06
ENSGALG00000036443	FUT8	5	3.13798E-06	5.24416E-06
ENSGALG00000006965	C12orf43	15	3.14535E-06	5.25575E-06
ENSGALG00000044485	F2RL3	28	3.14816E-06	5.25973E-06
ENSGALG00000043002	TOMM6	26	3.1594E-06	5.27778E-06
ENSGALG00000005483	CDH13	11	3.18565E-06	5.3209E-06
ENSGALG00000050442		1	3.21156E-06	5.36345E-06
ENSGALG00000038601	LIPH	9	3.22063E-06	5.37787E-06
ENSGALG00000016776	TBC1D8	1	3.23062E-06	5.3938E-06
ENSGALG00000008508	PYGB	3	3.30413E-06	5.51577E-06
ENSGALG00000017220		1	3.31435E-06	5.53209E-06
ENSGALG00000002893	STC2	13	3.32253E-06	5.54498E-06
ENSGALG00000010177	ZFC3H1	1	3.34201E-06	5.57672E-06
ENSGALG00000004612	MTHFR	21	3.3477E-06	5.58545E-06
ENSGALG00000023708	UBXN10	21	3.36737E-06	5.61751E-06
ENSGALG00000007596		12	3.44678E-06	5.74919E-06
ENSGALG00000015815	RMND5A	4	3.44818E-06	5.75074E-06
ENSGALG00000011227	CDS1	4	3.45778E-06	5.76596E-06
ENSGALG00000011076	SYNE3	5	3.46642E-06	5.77958E-06
ENSGALG00000030908		12	3.4729E-06	5.78959E-06
ENSGALG00000032348	LTB4R	16	3.50923E-06	5.84936E-06
ENSGALG00000006507	CLMP	24	3.51664E-06	5.8609E-06
ENSGALG00000003443	LRRC52	8	3.54299E-06	5.90401E-06
ENSGALG00000009233	LIN9	3	3.55091E-06	5.9164E-06
ENSGALG00000038666	FBXL12	30	3.56952E-06	5.9466E-06
ENSGALG00000050125		32	3.61435E-06	6.02046E-06
ENSGALG00000005014		4	3.66811E-06	6.10917E-06
ENSGALG00000052087		3	3.71131E-06	6.18028E-06
ENSGALG00000008465	SORCS1	6	3.71696E-06	6.18884E-06

ENSGALG00000033017	C2orf88	7	3.75855E-06	6.25723E-06
ENSGALG00000029168		1	3.76022E-06	6.25916E-06
ENSGALG00000052581	ARL16	18	3.78563E-06	6.30061E-06
ENSGALG00000011822	CANT1	18	3.79429E-06	6.31416E-06
ENSGALG00000030590	COA6	3	3.80903E-06	6.33781E-06
ENSGALG00000030941	ELAPOR1	26	3.817E-06	6.35022E-06
ENSGALG00000033460	TMCO1	8	3.83871E-06	6.38546E-06
ENSGALG00000029996	PPP2R5C	5	3.88909E-06	6.46838E-06
ENSGALG00000048165		33	3.92422E-06	6.52591E-06
ENSGALG00000002913	BRI3BP	15	3.92995E-06	6.53455E-06
ENSGALG00000003016	MASP2	21	3.93384E-06	6.54013E-06
ENSGALG00000054915		19	3.94278E-06	6.5541E-06
ENSGALG00000010642	IRF2	4	3.95099E-06	6.56685E-06
ENSGALG00000027166	ART7B	1	3.97415E-06	6.60445E-06
ENSGALG00000042008	GSTM3	26	3.98062E-06	6.6143E-06
ENSGALG00000007237	ALYREF	18	3.98685E-06	6.62374E-06
ENSGALG00000000583	SOX13	26	3.99602E-06	6.63808E-06
ENSGALG00000048474		28	3.99993E-06	6.64367E-06
ENSGALG00000041643	NFIC	28	4.0176E-06	6.6721E-06
ENSGALG00000038336	SLC20A2	4	4.0237E-06	6.68133E-06
ENSGALG00000041907	CDK5R1	27	4.06044E-06	6.74141E-06
ENSGALG00000046965	NNF1	23	4.07036E-06	6.75696E-06
ENSGALG00000010863	TWISTNB	2	4.13044E-06	6.85576E-06
ENSGALG00000000158	DMA	16	4.1566E-06	6.89825E-06
ENSGALG00000040023	MAPK11	1	4.22438E-06	7.00977E-06
ENSGALG00000052684		1	4.24628E-06	7.04517E-06
ENSGALG00000023881	PLXDC1	27	4.26638E-06	7.07755E-06
ENSGALG00000011800	FAM234B	1	4.31046E-06	7.1497E-06
ENSGALG00000033244	DNM3	8	4.32084E-06	7.16593E-06
ENSGALG00000046826	GYS1	33	4.32224E-06	7.16729E-06
ENSGALG00000039532	PHC2	21	4.33981E-06	7.19544E-06
ENSGALG00000000584		26	4.36023E-06	7.22832E-06
ENSGALG00000031331	PKIG	20	4.36527E-06	7.23569E-06

ENSGALG00000012277	MAFF	1	4.36773E-06	7.23879E-06
ENSGALG00000011594	NR1H4	1	4.37034E-06	7.24212E-06
ENSGALG00000016828	GRTP1	1	4.38389E-06	7.26267E-06
ENSGALG00000038875	TMC6	18	4.38393E-06	7.26267E-06
ENSGALG00000006947	BDH1A	9	4.43112E-06	7.33986E-06
ENSGALG00000007930	MAP2K5	10	4.43254E-06	7.3412E-06
ENSGALG00000041949	AAMDC	1	4.43934E-06	7.35147E-06
ENSGALG00000016292		3	4.45391E-06	7.3746E-06
ENSGALG00000033925		4	4.45833E-06	7.38091E-06
ENSGALG00000044628		4	4.45952E-06	7.38188E-06
ENSGALG00000003287	TMEM39B	23	4.48035E-06	7.41535E-06
ENSGALG00000036728	PRDM16	21	4.48615E-06	7.4233E-06
ENSGALG00000039468		9	4.48637E-06	7.4233E-06
ENSGALG00000005981	GDF2	6	4.49267E-06	7.43271E-06
ENSGALG00000006074	SOX6	5	4.49363E-06	7.4333E-06
ENSGALG00000017134	IFT88	1	4.49795E-06	7.43944E-06
ENSGALG00000040291		4	4.52741E-06	7.48714E-06
ENSGALG00000008808	DTD1	3	4.53143E-06	7.49277E-06
ENSGALG00000009467	RBM48	2	4.54019E-06	7.50624E-06
ENSGALG00000013969	ALDH8A1	3	4.55322E-06	7.52676E-06
ENSGALG00000008970	NRK	4	4.58544E-06	7.579E-06
ENSGALG00000009903	IFNG	1	4.60691E-06	7.61346E-06
ENSGALG00000017177	MSANTD4	1	4.61326E-06	7.62292E-06
ENSGALG00000003267	STAT3	27	4.62626E-06	7.64337E-06
ENSGALG00000016929	PIBF1	1	4.64372E-06	7.67117E-06
ENSGALG00000005070	AP3M1	6	4.64559E-06	7.67321E-06
ENSGALG00000039710	HDAC7	33	4.67853E-06	7.72657E-06
ENSGALG00000048063		16	4.68594E-06	7.73776E-06
ENSGALG00000027138	ETV3L	25	4.69796E-06	7.75656E-06
ENSGALG00000025788	CACNB1	27	4.72721E-06	7.80379E-06
ENSGALG00000009289	SLC18A2	6	4.73608E-06	7.81739E-06
ENSGALG00000019543	OTULIN	2	4.7616E-06	7.85845E-06
ENSGALG00000049862		10	4.76787E-06	7.86772E-06

ENSGALG00000008615		5	4.76872E-06	7.86807E-06
ENSGALG00000035819	TMEM51	21	4.76959E-06	7.86844E-06
ENSGALG00000010700	RPA3	2	4.8019E-06	7.92067E-06
ENSGALG00000030888		14	4.80471E-06	7.92423E-06
ENSGALG00000005464		4	4.84745E-06	7.99363E-06
ENSGALG00000000518	KLHL12	26	4.85024E-06	7.99716E-06
ENSGALG00000008231	PACSIN3	5	4.86588E-06	8.02186E-06
ENSGALG00000045753	MRPL12	18	4.90355E-06	8.08287E-06
ENSGALG00000009500	VPS50	2	4.92438E-06	8.1161E-06
ENSGALG00000031624		31	5.04171E-06	8.30773E-06
ENSGALG00000042454	DCDC2	2	5.04201E-06	8.30773E-06
ENSGALG00000008167	KMT2E	1	5.05342E-06	8.32445E-06
ENSGALG00000026239	MPV17	3	5.05352E-06	8.32445E-06
ENSGALG00000013967	NUDT5	1	5.05757E-06	8.33E-06
ENSGALG00000016945	LECT1	1	5.06108E-06	8.33465E-06
ENSGALG00000042197	WDR75	7	5.08538E-06	8.37354E-06
ENSGALG00000016693	CSF2RA	1	5.08707E-06	8.37518E-06
ENSGALG00000038219	NAA40	33	5.10276E-06	8.39989E-06
ENSGALG00000032440	QPCT	3	5.1055E-06	8.40325E-06
ENSGALG00000016951	MTRF1	1	5.14336E-06	8.46444E-06
ENSGALG00000046794		16	5.14923E-06	8.47295E-06
ENSGALG00000013740	SLC20A1	22	5.17817E-06	8.51942E-06
ENSGALG00000011704	HACD2	7	5.19323E-06	8.54305E-06
ENSGALG00000008195	NPHP1	3	5.23095E-06	8.60394E-06
ENSGALG00000016511	ADGRG2	1	5.26125E-06	8.65261E-06
ENSGALG00000025712	DEF8	11	5.26991E-06	8.66567E-06
ENSGALG00000003920		4	5.28004E-06	8.68117E-06
ENSGALG00000008553	INO80	5	5.28535E-06	8.68872E-06
ENSGALG00000007112	LINS1	10	5.3622E-06	8.81387E-06
ENSGALG00000003307	TMED2	15	5.37242E-06	8.82948E-06
ENSGALG00000024047	MYCL	23	5.42786E-06	8.9194E-06
ENSGALG00000010889	HOOK1	8	5.45439E-06	8.96179E-06
ENSGALG00000009714	CBR4	4	5.50268E-06	9.0399E-06

ENSGALG00000039803	LSM12	27	5.50845E-06	9.04816E-06
ENSGALG00000055094	CD63	33	5.51418E-06	9.05635E-06
ENSGALG00000047662	NR1D1	27	5.52637E-06	9.07516E-06
ENSGALG00000031922	TRMU	1	5.53946E-06	9.09542E-06
ENSGALG00000012295	CDKL1	5	5.55283E-06	9.11616E-06
ENSGALG00000045726		1	5.56829E-06	9.14029E-06
ENSGALG00000029279		2	5.63569E-06	9.2497E-06
ENSGALG00000053351		27	5.67049E-06	9.30556E-06
ENSGALG00000008462	CDK3	18	5.68466E-06	9.32756E-06
ENSGALG00000029502	CLIC2	4	5.69147E-06	9.33747E-06
ENSGALG00000031538	PI4K2B	4	5.69981E-06	9.34968E-06
ENSGALG00000006446	NAP1L4	5	5.70044E-06	9.34968E-06
ENSGALG00000050405		18	5.72647E-06	9.39111E-06
ENSGALG00000005678	FLNB	12	5.73309E-06	9.40069E-06
ENSGALG00000032469	ARHGAP45	28	5.73883E-06	9.40884E-06
ENSGALG00000004889	SLC2A1	21	5.81387E-06	9.5306E-06
ENSGALG00000012830	IRF4	2	5.83576E-06	9.56519E-06
ENSGALG00000009743	RPAP3	1	5.89084E-06	9.65417E-06
ENSGALG00000001702	EXOC3L1	11	5.91965E-06	9.70008E-06
ENSGALG00000030773	CP	9	5.92321E-06	9.70461E-06
ENSGALG00000011058	LEPR	8	5.93319E-06	9.71966E-06
ENSGALG00000001013	PPP6C	17	5.93478E-06	9.72095E-06
ENSGALG00000008590	EEF1B2	7	5.95926E-06	9.75973E-06
ENSGALG00000038637	SHF	10	6.07461E-06	9.94732E-06
ENSGALG00000000091	INTS4	1	6.07986E-06	9.95458E-06
ENSGALG00000032501		22	6.08576E-06	9.9629E-06
ENSGALG00000001225	RABGAP1	17	6.10034E-06	9.98543E-06
ENSGALG00000002466	SLC2A5	21	6.10404E-06	9.99014E-06
ENSGALG00000013926	DCLRE1C	1	6.12417E-06	1.00217E-05
ENSGALG00000042798	GDAP1	2	6.1331E-06	1.0035E-05
ENSGALG00000040493	PTN	1	6.13842E-06	1.00424E-05
ENSGALG00000011353	TNNI3K	8	6.16397E-06	1.00828E-05
ENSGALG00000004852	DNM1	17	6.20473E-06	1.01481E-05

ENSGALG00000033157	ADAMTS10	28	6.22314E-06	1.01769E-05
ENSGALG00000050787	PORCN	13	6.24998E-06	1.02194E-05
ENSGALG00000036647	BCL7B	19	6.2537E-06	1.02241E-05
ENSGALG00000045814	CRLF2	1	6.26897E-06	1.02477E-05
ENSGALG00000040003	STXBP1	17	6.29323E-06	1.0286E-05
ENSGALG00000017195	PGR	1	6.32689E-06	1.03396E-05
ENSGALG00000025881		1	6.33042E-06	1.0344E-05
ENSGALG00000005391	SELENOK	12	6.36107E-06	1.03927E-05
ENSGALG00000051621	SCAMP5	10	6.3902E-06	1.04389E-05
ENSGALG00000043167	TNFRSF11B	2	6.40857E-06	1.04667E-05
ENSGALG00000003005	CHRNB4	10	6.40896E-06	1.04667E-05
ENSGALG00000006561	MRPL23	5	6.43165E-06	1.0501E-05
ENSGALG00000002769	DNAJC12	6	6.43165E-06	1.0501E-05
ENSGALG00000023517	AGPAT2	17	6.46701E-06	1.05573E-05
ENSGALG00000035506	FAU	33	6.55633E-06	1.07016E-05
ENSGALG00000046885	SCO2	1	6.60527E-06	1.07801E-05
ENSGALG00000013365		28	6.62807E-06	1.08158E-05
ENSGALG00000030250	SV2B	10	6.63709E-06	1.08291E-05
ENSGALG00000013057	USP18	1	6.66467E-06	1.08727E-05
ENSGALG00000002216	NEK7	8	6.67187E-06	1.0883E-05
ENSGALG00000030931	HTRA2	4	6.67998E-06	1.08947E-05
ENSGALG00000012388	PCMT1	3	6.71839E-06	1.09559E-05
ENSGALG00000038813	KLHL41	7	6.73148E-06	1.09758E-05
ENSGALG00000006512	HSPA8	24	6.73667E-06	1.09828E-05
ENSGALG00000004431	SAR1A	6	6.80262E-06	1.10879E-05
ENSGALG00000041621	LY6E	2	6.80295E-06	1.10879E-05
ENSGALG00000009035	GTPBP10	2	6.83115E-06	1.11323E-05
ENSGALG00000004693	ARPC1A	14	6.86921E-06	1.11929E-05
ENSGALG00000023709		17	6.90144E-06	1.12439E-05
ENSGALG00000001892	CYGB	18	6.91921E-06	1.12713E-05
ENSGALG00000029983	ANGPT1	2	6.92144E-06	1.12735E-05
ENSGALG00000040889	ARMC8	9	6.92697E-06	1.1281E-05
ENSGALG00000011328	GLB1L	7	6.94338E-06	1.13062E-05

ENSGALG00000004237	PRKAR1A	18	6.96171E-06	1.13345E-05
ENSGALG00000006480	TCF7	13	7.04546E-06	1.14693E-05
ENSGALG00000013403	MSANTD3	2	7.0824E-06	1.15279E-05
ENSGALG00000036791	COA5	1	7.10233E-06	1.15588E-05
ENSGALG00000017019	WDFY2	1	7.10847E-06	1.15673E-05
ENSGALG00000042210	RABEPK	17	7.24988E-06	1.17958E-05
ENSGALG00000032783	SUMO3	9	7.27377E-06	1.18331E-05
ENSGALG00000011272	RAB5A	2	7.28562E-06	1.18508E-05
ENSGALG00000005975	ZNF488	6	7.28836E-06	1.18537E-05
ENSGALG00000010954	MALSU1	2	7.30696E-06	1.18823E-05
ENSGALG00000033760	PTGES2	17	7.35811E-06	1.19639E-05
ENSGALG00000011511	CKB	5	7.37025E-06	1.19821E-05
ENSGALG00000030576		10	7.43696E-06	1.20889E-05
ENSGALG00000034453	SAMD11	21	7.43972E-06	1.20918E-05
ENSGALG00000046360		2	7.48446E-06	1.21629E-05
ENSGALG00000051829		1	7.50757E-06	1.21988E-05
ENSGALG00000009676	METTL5	7	7.5133E-06	1.22065E-05
ENSGALG00000029856	TOMM7	2	7.55091E-06	1.22659E-05
ENSGALG00000010942	NUP42	2	7.60312E-06	1.23491E-05
ENSGALG00000032398	ASPM	8	7.61683E-06	1.23697E-05
ENSGALG00000010459	VIPAS39	5	7.64625E-06	1.24159E-05
ENSGALG00000010120	KIF2C	8	7.69608E-06	1.24951E-05
ENSGALG00000035853	SCRN2	27	7.7177E-06	1.25285E-05
ENSGALG00000040644	C8G	17	7.72116E-06	1.25325E-05
ENSGALG00000011228	WARS1	5	7.76675E-06	1.26048E-05
ENSGALG00000044313		5	7.9265E-06	1.28624E-05
ENSGALG00000008365	CHRM4	5	7.94772E-06	1.28951E-05
ENSGALG00000003136	IKZF2	7	7.96262E-06	1.29175E-05
ENSGALG00000034076	PBX4	30	8.07449E-06	1.30973E-05
ENSGALG00000002119	VPS35L	14	8.08832E-06	1.3118E-05
ENSGALG00000030011	ZNF704	2	8.21229E-06	1.33161E-05
ENSGALG00000009134	CTTNBP2	1	8.21269E-06	1.33161E-05
ENSGALG00000001538	ST7L	26	8.29706E-06	1.34511E-05

ENSGALG00000028438	CLK1	7	8.39331E-06	1.36054E-05
ENSGALG00000042458	ACTN1	5	8.42465E-06	1.36544E-05
ENSGALG00000031983	MPHOSPH6	11	8.45501E-06	1.37017E-05
ENSGALG00000036625	NDUFV2	2	8.47783E-06	1.37369E-05
ENSGALG00000015122	TAF4B	2	8.49284E-06	1.37594E-05
ENSGALG00000038292	TMEM258	5	8.50116E-06	1.37711E-05
ENSGALG00000016449	SLC66A3	3	8.64083E-06	1.39954E-05
ENSGALG00000037401	IDH3A	10	8.64982E-06	1.40081E-05
ENSGALG00000005914	FAM21C	6	8.69428E-06	1.40783E-05
ENSGALG00000054321		31	8.69654E-06	1.40801E-05
ENSGALG00000026253	TMEM144	4	8.72297E-06	1.4121E-05
ENSGALG00000036955	NEDD8	25	8.75839E-06	1.41764E-05
ENSGALG00000011469	IGFBP2	7	8.8029E-06	1.42466E-05
ENSGALG00000005659	SNX12	4	8.87778E-06	1.43659E-05
ENSGALG00000011783	HIST1H111R	1	8.89762E-06	1.43961E-05
ENSGALG00000053194		1	8.90072E-06	1.43992E-05
ENSGALG00000006239		6	8.92379E-06	1.44346E-05
ENSGALG00000000379	ITGB3	27	8.92608E-06	1.44364E-05
ENSGALG00000042460	FPGS	17	8.94645E-06	1.44674E-05
ENSGALG00000016281	DMD	1	9.11197E-06	1.47331E-05
ENSGALG00000032402	VSTM2B	11	9.14733E-06	1.47883E-05
ENSGALG00000031726	MAPRE3	3	9.16634E-06	1.48171E-05
ENSGALG00000008740	POT1	1	9.23817E-06	1.49312E-05
ENSGALG00000000650	NIP7	11	9.25122E-06	1.49503E-05
ENSGALG00000042214	UBQLN4	25	9.28283E-06	1.49994E-05
ENSGALG00000012229	RPS29	5	9.34844E-06	1.51034E-05
ENSGALG00000040113	SFT2D3	9	9.3539E-06	1.51102E-05
ENSGALG00000032627	C9orf69	17	9.38237E-06	1.51542E-05
ENSGALG00000000645		21	9.38485E-06	1.51562E-05
ENSGALG00000037595	USF1	25	9.44358E-06	1.52491E-05
ENSGALG00000007778	PES1	15	9.47217E-06	1.52932E-05
ENSGALG00000007863	COX7B	4	9.56305E-06	1.54379E-05
ENSGALG00000007467	SLC25A26	12	9.58184E-06	1.54662E-05

ENSGALG00000041470	PPARA	1	9.58929E-06	1.54761E-05
ENSGALG00000003108	SMG6	19	9.59709E-06	1.54867E-05
ENSGALG00000035842	SLC37A2	24	9.61953E-06	1.55208E-05
ENSGALG00000006817	CTNNBL1	20	9.62945E-06	1.55348E-05
ENSGALG00000038495	TAF1B	3	9.63095E-06	1.55351E-05
ENSGALG00000040205		13	9.64378E-06	1.55538E-05
ENSGALG00000037166		5	9.69983E-06	1.56421E-05
ENSGALG00000009021	ST6GALNAC5	8	9.78249E-06	1.57733E-05
ENSGALG00000048781		3	9.78453E-06	1.57745E-05
ENSGALG00000006047	TPRA1	12	9.81002E-06	1.58135E-05
ENSGALG00000044278		3	9.82825E-06	1.58408E-05
ENSGALG00000010151	CCDC167	3	9.8602E-06	1.58902E-05
ENSGALG00000034020	RHOBTB2	22	9.86431E-06	1.58948E-05
ENSGALG00000011491	DYNC1LI1	2	9.86779E-06	1.58982E-05
ENSGALG00000010874	UNC79	5	9.87429E-06	1.59066E-05
ENSGALG00000004189	MAFK	14	9.98063E-06	1.60758E-05
ENSGALG00000047072		4	9.99179E-06	1.60917E-05
ENSGALG00000010601	UFSP2	4	1.00231E-05	1.614E-05
ENSGALG00000043103	PKNOX2	24	1.01068E-05	1.62726E-05
ENSGALG00000027981	AASDHPPT	1	1.01458E-05	1.63332E-05
ENSGALG00000027980	NANOS1	6	1.02164E-05	1.64446E-05
ENSGALG00000050619	SOCS2	1	1.02897E-05	1.65605E-05
ENSGALG00000049536		1	1.02944E-05	1.65659E-05
ENSGALG00000033938	ARHGAP26	13	1.03406E-05	1.66381E-05
ENSGALG00000003478	GFI1B	17	1.03898E-05	1.6715E-05
ENSGALG00000037646	BRD8	13	1.0404E-05	1.67337E-05
ENSGALG00000042275		28	1.04042E-05	1.67337E-05
ENSGALG00000003932	SDC4	20	1.04143E-05	1.67477E-05
ENSGALG00000006714	CCDC61	9	1.04409E-05	1.67883E-05
ENSGALG00000012367	TRIM9	5	1.05103E-05	1.68977E-05
ENSGALG00000016445	NOL10	3	1.06612E-05	1.7138E-05
ENSGALG00000012357	CXCR4	7	1.07202E-05	1.72305E-05
ENSGALG00000016955	VWA8	1	1.07972E-05	1.7352E-05

ENSGALG00000031390	STAU2	2	1.08185E-05	1.7384E-05
ENSGALG00000011128	PSMD14	7	1.08486E-05	1.743E-05
ENSGALG00000016991	ESD	1	1.08665E-05	1.74565E-05
ENSGALG00000052650	RBIS	2	1.10679E-05	1.77778E-05
ENSGALG00000031754	KCNG2	2	1.10855E-05	1.78036E-05
ENSGALG00000004837	CCNDBP1	20	1.11352E-05	1.78811E-05
ENSGALG00000009307	FAM204A	6	1.12635E-05	1.80848E-05
ENSGALG00000007466	EDNRB2	4	1.13483E-05	1.82186E-05
ENSGALG00000028407	GDF9	13	1.13603E-05	1.82353E-05
ENSGALG00000006194	SLC52A3	20	1.13982E-05	1.82938E-05
ENSGALG00000006367	PCBD2	13	1.1422E-05	1.83297E-05
ENSGALG00000043089	VWCE	5	1.14965E-05	1.84468E-05
ENSGALG00000005707	IFT20	19	1.15129E-05	1.84707E-05
ENSGALG00000046843		24	1.15332E-05	1.85007E-05
ENSGALG00000052568		31	1.15639E-05	1.85475E-05
ENSGALG00000000150	RPL9	4	1.16009E-05	1.86044E-05
ENSGALG00000013605	CARMIL1	2	1.16136E-05	1.86224E-05
ENSGALG00000013951	AHI1	3	1.16478E-05	1.86747E-05
ENSGALG00000034735		6	1.16496E-05	1.86752E-05
ENSGALG00000039741	TNFRSF8	21	1.16947E-05	1.8745E-05
ENSGALG00000051012	USP21	25	1.16984E-05	1.87484E-05
ENSGALG00000011138	CCNK	5	1.19178E-05	1.90975E-05
ENSGALG00000040152	CCNJL	13	1.20089E-05	1.92411E-05
ENSGALG00000008239	NDUFB3	7	1.20842E-05	1.93591E-05
ENSGALG00000040421	LYPLA1	2	1.21302E-05	1.94303E-05
ENSGALG00000003025		9	1.2172E-05	1.94947E-05
ENSGALG00000002199	KLHL17	21	1.22665E-05	1.96434E-05
ENSGALG00000005162	RNPC3	8	1.24585E-05	1.99483E-05
ENSGALG00000028880	FDPS	25	1.25836E-05	2.0146E-05
ENSGALG00000026271	TM2D3	10	1.26833E-05	2.03029E-05
ENSGALG00000047488		2	1.27884E-05	2.04685E-05
ENSGALG00000015712	SLBP	4	1.3011E-05	2.0822E-05
ENSGALG00000003562	NPTX2	14	1.30372E-05	2.08613E-05

ENSGALG00000028906	FEM1B	10	1.3095E-05	2.09509E-05
ENSGALG00000019003	PRAG1	4	1.31111E-05	2.0974E-05
ENSGALG00000013674	TUSC3	4	1.31597E-05	2.10486E-05
ENSGALG00000031182	FANCM	5	1.31612E-05	2.10486E-05
ENSGALG00000046858	MOGS	4	1.3211E-05	2.11255E-05
ENSGALG00000000417	SLC16A4	26	1.32263E-05	2.11472E-05
ENSGALG00000046740		22	1.35708E-05	2.16952E-05
ENSGALG00000045036		4	1.36119E-05	2.1758E-05
ENSGALG00000040567	AVPR1A	1	1.3614E-05	2.17584E-05
ENSGALG00000054868		20	1.36559E-05	2.18226E-05
ENSGALG00000031890	YBX3	1	1.3665E-05	2.18342E-05
ENSGALG00000023886	ZNF804A	7	1.36834E-05	2.18594E-05
ENSGALG00000015183		2	1.36843E-05	2.18594E-05
ENSGALG00000012011	L3HYPDH	5	1.3693E-05	2.18705E-05
ENSGALG00000008176	ASB3	3	1.37577E-05	2.19709E-05
ENSGALG00000005043		15	1.38084E-05	2.20489E-05
ENSGALG00000000856	EIF2D	26	1.38391E-05	2.20951E-05
ENSGALG00000050392		25	1.38515E-05	2.21119E-05
ENSGALG00000026043		11	1.40005E-05	2.2347E-05
ENSGALG00000011643	UTP20	1	1.40377E-05	2.24033E-05
ENSGALG00000038393	DMB2	16	1.41459E-05	2.2573E-05
ENSGALG00000001459	TNNC1	12	1.41534E-05	2.25821E-05
ENSGALG00000016560	SELENOI	3	1.42084E-05	2.26669E-05
ENSGALG00000016985	CPB2	1	1.43185E-05	2.28395E-05
ENSGALG00000038688	ADAMTS17	10	1.4348E-05	2.28836E-05
ENSGALG00000008206	TESC	15	1.43922E-05	2.29511E-05
ENSGALG00000015513	MMS22L	3	1.45061E-05	2.31279E-05
ENSGALG00000001561	MXRA8	21	1.45069E-05	2.31279E-05
ENSGALG00000011967	MAD2L1	4	1.45114E-05	2.31322E-05
ENSGALG00000005465	NDUFB10	14	1.45135E-05	2.31324E-05
ENSGALG00000014938	ABHD3	2	1.45328E-05	2.31597E-05
ENSGALG00000001245	SIRT6	28	1.45362E-05	2.31597E-05
ENSGALG00000017330	RPS3	1	1.45363E-05	2.31597E-05

ENSGALG00000001163	NDEL1	18	1.45703E-05	2.32108E-05
ENSGALG00000004066	HTR6	21	1.49535E-05	2.38182E-05
ENSGALG00000049665	BLEC3	16	1.50094E-05	2.39041E-05
ENSGALG00000052798		27	1.50432E-05	2.39548E-05
ENSGALG00000004325	P4HA1	6	1.51076E-05	2.40542E-05
ENSGALG00000016098	EXOC6B	4	1.53414E-05	2.44232E-05
ENSGALG00000037811	NRSN1	2	1.53914E-05	2.44997E-05
ENSGALG00000049218	DYX1C1	10	1.55133E-05	2.46904E-05
ENSGALG00000016618	PNPLA4	1	1.55246E-05	2.47051E-05
ENSGALG00000040364	HERPUD2	2	1.58076E-05	2.51523E-05
ENSGALG00000000397	IPO9	26	1.58969E-05	2.52911E-05
ENSGALG00000009601	NELL2	1	1.62006E-05	2.57709E-05
ENSGALG00000008036	TMEM209	1	1.62615E-05	2.58644E-05
ENSGALG00000028982	CMPK2	3	1.64365E-05	2.61394E-05
ENSGALG00000000329	AHCYL1	26	1.66004E-05	2.63966E-05
ENSGALG00000004736	STAU1	20	1.66273E-05	2.64359E-05
ENSGALG00000013950		3	1.6797E-05	2.67021E-05
ENSGALG00000002550	TSHB	26	1.68391E-05	2.67656E-05
ENSGALG00000009830	MGAT4D	4	1.68522E-05	2.6783E-05
ENSGALG00000009525	HAT1	7	1.69101E-05	2.68715E-05
ENSGALG00000008930	B3GNT2	3	1.69171E-05	2.68791E-05
ENSGALG00000005583	ALG14	8	1.69359E-05	2.69055E-05
ENSGALG00000001333	MRRFP1	17	1.70343E-05	2.70583E-05
ENSGALG00000003720	FANCD2	12	1.71137E-05	2.71808E-05
ENSGALG00000001169	CRB2	17	1.71813E-05	2.72847E-05
ENSGALG00000005986	TUB	5	1.71853E-05	2.72875E-05
ENSGALG00000041093	MMP24	20	1.74204E-05	2.76571E-05
ENSGALG00000030614	ADH1C	4	1.74928E-05	2.77684E-05
ENSGALG00000023364	CDC42EP4	18	1.75715E-05	2.78897E-05
ENSGALG00000029022	NPAS3	5	1.76748E-05	2.80501E-05
ENSGALG00000012408	PSMC6	5	1.77293E-05	2.8133E-05
ENSGALG00000050785		31	1.80314E-05	2.86086E-05
ENSGALG00000006702	MFGE8	10	1.813E-05	2.87613E-05

ENSGALG00000027440	COX20	3	1.81349E-05	2.87653E-05
ENSGALG00000037744	CACNA1S	26	1.82253E-05	2.89049E-05
ENSGALG00000008155	KCTD18	7	1.83599E-05	2.91146E-05
ENSGALG00000006331	STAMBPL1	6	1.85318E-05	2.93834E-05
ENSGALG00000001405		26	1.86089E-05	2.95018E-05
ENSGALG00000021213	UBE2D1	6	1.86465E-05	2.95575E-05
ENSGALG00000001959	HENMT1	8	1.86502E-05	2.95595E-05
ENSGALG00000009725	SCG5	5	1.8762E-05	2.97329E-05
ENSGALG00000053106	FAM181B	1	1.88548E-05	2.98761E-05
ENSGALG00000013568	NR4A3	2	1.88892E-05	2.99268E-05
ENSGALG00000038506	NXNL1	30	1.89121E-05	2.99591E-05
ENSGALG00000012833	VPS33B	10	1.89892E-05	3.00773E-05
ENSGALG00000039917	EXOSC1	6	1.9017E-05	3.01174E-05
ENSGALG00000008409	GSTO2	6	1.90218E-05	3.01211E-05
ENSGALG00000044002		24	1.9293E-05	3.05466E-05
ENSGALG00000004366	BAP1	12	1.95153E-05	3.08946E-05
ENSGALG00000007974	NUP85	18	1.95499E-05	3.09453E-05
ENSGALG00000001181	KCNJ5	24	1.95579E-05	3.09539E-05
ENSGALG00000015119	SS18	2	1.95698E-05	3.09688E-05
ENSGALG00000017189	C1H11ORF70	1	1.95827E-05	3.09851E-05
ENSGALG00000007282	CNGA2	4	1.95942E-05	3.09994E-05
ENSGALG00000005171	AP1S3	9	1.96991E-05	3.11612E-05
ENSGALG00000005460	SLCO4A1	20	1.98147E-05	3.134E-05
ENSGALG00000007919	EIF4E2	9	1.98669E-05	3.14186E-05
ENSGALG00000029898	YKT6	22	1.99859E-05	3.16026E-05
ENSGALG00000005968	MMP11	15	2.00143E-05	3.16434E-05
ENSGALG00000015338	QRSL1	3	2.00186E-05	3.16461E-05
ENSGALG00000000667	EDN2	23	2.00408E-05	3.16771E-05
ENSGALG00000012876	SSBP1	1	2.05657E-05	3.25025E-05
ENSGALG00000030526		1	2.08214E-05	3.29025E-05
ENSGALG00000038635		2	2.11001E-05	3.33386E-05
ENSGALG00000006292		2	2.12259E-05	3.3533E-05
ENSGALG00000033541	FRMPD4	1	2.12323E-05	3.35387E-05

ENSGALG00000036004	MRPL34	28	2.12427E-05	3.35509E-05
ENSGALG00000035375	LRTOMT	1	2.14505E-05	3.38746E-05
ENSGALG00000014794	GDAP2	1	2.14712E-05	3.3903E-05
ENSGALG00000030920	APOC3	24	2.1479E-05	3.39109E-05
ENSGALG00000007992	GGA3	18	2.14826E-05	3.39122E-05
ENSGALG00000032413	NADK	21	2.15342E-05	3.39892E-05
ENSGALG00000007040	ACAP2	9	2.1579E-05	3.40554E-05
ENSGALG00000034461	TOPBP1	2	2.16682E-05	3.41918E-05
ENSGALG00000047235		9	2.17311E-05	3.42867E-05
ENSGALG00000006723		2	2.17387E-05	3.42942E-05
ENSGALG00000054998		4	2.18933E-05	3.45336E-05
ENSGALG00000001263	GTF2IRD1	19	2.19563E-05	3.46285E-05
ENSGALG00000001513	CNTRL	17	2.20271E-05	3.47357E-05
ENSGALG00000010384	CLRN1	9	2.20594E-05	3.47821E-05
ENSGALG00000009257	PLRG1	4	2.23487E-05	3.52337E-05
ENSGALG00000002993	CA10	18	2.26952E-05	3.57754E-05
ENSGALG00000008823	IQUB	1	2.27162E-05	3.58038E-05
ENSGALG00000008257	MERTK	3	2.2851E-05	3.60116E-05
ENSGALG00000017199	MAML2	1	2.28706E-05	3.60379E-05
ENSGALG00000012683	RNF144B	2	2.29517E-05	3.6161E-05
ENSGALG00000003885		8	2.31421E-05	3.64563E-05
ENSGALG00000047993		3	2.3168E-05	3.64924E-05
ENSGALG00000003374	ESRP2	11	2.34299E-05	3.69001E-05
ENSGALG00000011572	ASPG	5	2.37293E-05	3.73644E-05
ENSGALG00000008145	TYW5	7	2.37308E-05	3.73644E-05
ENSGALG00000010619	CC2D1B	8	2.37853E-05	3.74454E-05
ENSGALG00000048069	LIX1L	25	2.39862E-05	3.77567E-05
ENSGALG00000051885	TTC4	8	2.40107E-05	3.77905E-05
ENSGALG00000048869		2	2.40224E-05	3.78039E-05
ENSGALG00000003973	TLCD1	19	2.42075E-05	3.80904E-05
ENSGALG00000011910	MNAT1	5	2.44093E-05	3.84029E-05
ENSGALG00000038896		23	2.44233E-05	3.84199E-05
ENSGALG00000040703	FLOT1	16	2.44557E-05	3.84659E-05

ENSGALG00000030024	USP15	1	2.44979E-05	3.85274E-05
ENSGALG00000031323	ATP9B	2	2.45188E-05	3.85553E-05
ENSGALG00000030187	CAT	5	2.4533E-05	3.85726E-05
ENSGALG00000030270	CD1C	16	2.46281E-05	3.87172E-05
ENSGALG00000008584	NUSAP1	5	2.47573E-05	3.89152E-05
ENSGALG00000009455	MCMBP	6	2.48694E-05	3.90864E-05
ENSGALG00000044554	RIDA	2	2.50009E-05	3.9288E-05
ENSGALG00000037587	ENPP3	3	2.50809E-05	3.94087E-05
ENSGALG00000030701	IQCC	23	2.5406E-05	3.99144E-05
ENSGALG00000010618	SLC30A6	3	2.55978E-05	4.02105E-05
ENSGALG00000039145	CA8	2	2.56063E-05	4.02187E-05
ENSGALG00000029747	ST3GAL4	24	2.56541E-05	4.02885E-05
ENSGALG00000046904		1	2.56706E-05	4.03093E-05
ENSGALG00000014688	ZYX	1	2.56968E-05	4.03452E-05
ENSGALG00000006017	DOCK11	4	2.57259E-05	4.03858E-05
ENSGALG00000036142	LOXL3	4	2.59751E-05	4.07717E-05
ENSGALG00000035352	ZBTB17	21	2.59833E-05	4.07792E-05
ENSGALG00000032882	EVA1C	1	2.60855E-05	4.09345E-05
ENSGALG00000005795	CYP2C23b	6	2.61256E-05	4.09922E-05
ENSGALG00000009815	TMEM5	1	2.63384E-05	4.13206E-05
ENSGALG00000008548	MDH1B	7	2.63765E-05	4.1375E-05
ENSGALG00000006110	PLK1	14	2.65387E-05	4.16242E-05
ENSGALG00000016609	CENPO	3	2.68269E-05	4.20707E-05
ENSGALG00000048048		9	2.69374E-05	4.22386E-05
ENSGALG00000030099		33	2.69657E-05	4.22777E-05
ENSGALG00000039448	NCAPD2	1	2.69766E-05	4.22893E-05
ENSGALG00000015132	CDH2	2	2.6981E-05	4.22907E-05
ENSGALG00000002525	RASGEF1A	6	2.71274E-05	4.25147E-05
ENSGALG00000002880	AARSD1	27	2.71352E-05	4.25215E-05
ENSGALG00000010323	BATF	5	2.73036E-05	4.27798E-05
ENSGALG00000000674	MYO1D	27	2.74763E-05	4.30449E-05
ENSGALG00000046210		33	2.76377E-05	4.32915E-05
ENSGALG00000016536	HADHA	3	2.76408E-05	4.32915E-05

ENSGALG00000010798	DHCR24	8	2.77471E-05	4.34524E-05
ENSGALG00000048200	MRPL33	3	2.77513E-05	4.34534E-05
ENSGALG00000052067		1	2.78817E-05	4.36519E-05
ENSGALG00000009100	FSHR	3	2.81883E-05	4.41264E-05
ENSGALG00000049408	HIST1H2B8	1	2.83843E-05	4.44274E-05
ENSGALG00000033232	LMAN2	13	2.84311E-05	4.4495E-05
ENSGALG00000050887	CACNB3	33	2.84818E-05	4.45687E-05
ENSGALG00000011947	POLR3H	1	2.86862E-05	4.48826E-05
ENSGALG00000009740	RASGRP1	5	2.88432E-05	4.51225E-05
ENSGALG00000011356		8	2.88857E-05	4.51833E-05
ENSGALG00000054536		32	2.89479E-05	4.52748E-05
ENSGALG00000050131		16	2.89883E-05	4.53322E-05
ENSGALG00000048325	GBP	12	2.90204E-05	4.53764E-05
ENSGALG00000007357	BCL6	9	2.91871E-05	4.56312E-05
ENSGALG00000006563	SEMA3D	1	2.92433E-05	4.57132E-05
ENSGALG00000046964	CPLANE2	21	2.9338E-05	4.58555E-05
ENSGALG00000012735	EDN1	2	2.93458E-05	4.58617E-05
ENSGALG00000026490	RPS16	1	2.95556E-05	4.61838E-05
ENSGALG00000008656	ICOS	7	2.95859E-05	4.62251E-05
ENSGALG00000016646		3	2.96109E-05	4.62583E-05
ENSGALG00000012348	MCM6	7	2.97258E-05	4.64317E-05
ENSGALG00000005951	VPS29L	15	2.9776E-05	4.65042E-05
ENSGALG00000014124	TEC	4	2.99562E-05	4.67798E-05
ENSGALG00000010347	CCNG2	4	3.00759E-05	4.69606E-05
ENSGALG00000041309	ALKBH3	5	3.03524E-05	4.7384E-05
ENSGALG00000003445	MGST3	8	3.03548E-05	4.7384E-05
ENSGALG00000007565	PPP2R2B	13	3.04712E-05	4.75595E-05
ENSGALG00000010938	KLHL7	2	3.04792E-05	4.75659E-05
ENSGALG00000016775	RPL31	1	3.06763E-05	4.78675E-05
ENSGALG00000037556	SLC1A2	5	3.08127E-05	4.80741E-05
ENSGALG00000013494	CENPN	11	3.10388E-05	4.84206E-05
ENSGALG00000017290		1	3.11418E-05	4.85752E-05
ENSGALG00000047956		7	3.12946E-05	4.88072E-05

ENSGALG00000050586	ILVBL	30	3.16542E-05	4.93617E-05
ENSGALG00000012540	RBFOX2	1	3.18353E-05	4.96378E-05
ENSGALG00000041977	RNF128	4	3.18414E-05	4.96411E-05
ENSGALG00000021869	PODN	8	3.22299E-05	5.02403E-05
ENSGALG00000016927	KLF5	1	3.2646E-05	5.08824E-05
ENSGALG00000039634	KRT80	33	3.26659E-05	5.09068E-05
ENSGALG00000003683	MED27	17	3.27321E-05	5.10035E-05
ENSGALG00000017282	NDUFA9	1	3.27894E-05	5.10862E-05
ENSGALG00000050204	MRPL4	30	3.28447E-05	5.11659E-05
ENSGALG00000003099	PCTP	18	3.29569E-05	5.13341E-05
ENSGALG00000041428	NEK4	12	3.31309E-05	5.15986E-05
ENSGALG00000008918	LAMP5	3	3.33386E-05	5.19154E-05
ENSGALG00000022926	GPR162	1	3.35557E-05	5.22467E-05
ENSGALG00000008506	MRPS25	12	3.38888E-05	5.27586E-05
ENSGALG00000008669	CD28	7	3.39375E-05	5.28278E-05
ENSGALG00000005403	DHRS11	19	3.3946E-05	5.28343E-05
ENSGALG00000029999	CCDC102A	11	3.41772E-05	5.31873E-05
ENSGALG00000004758	KCNB1	20	3.44229E-05	5.35628E-05
ENSGALG00000043367	LHFPL3	1	3.45181E-05	5.37041E-05
ENSGALG00000030718		4	3.47487E-05	5.4056E-05
ENSGALG00000019835	TRIM27.2	16	3.5076E-05	5.45583E-05
ENSGALG00000031260		2	3.51431E-05	5.46556E-05
ENSGALG00000012696	RANBP9	2	3.52327E-05	5.47879E-05
ENSGALG00000008132	PUS7	1	3.52657E-05	5.48323E-05
ENSGALG00000011219	GCGR	18	3.53759E-05	5.49966E-05
ENSGALG00000003899	RAB17	7	3.54124E-05	5.50463E-05
ENSGALG00000046823	SNX22	10	3.57406E-05	5.55494E-05
ENSGALG00000000540	GAS8	11	3.58746E-05	5.57506E-05
ENSGALG00000039705	GFPT2	13	3.60437E-05	5.60062E-05
ENSGALG00000015906	COX7A2	3	3.61295E-05	5.61324E-05
ENSGALG00000004203	PSMG3	14	3.61989E-05	5.6233E-05
ENSGALG00000032815	ATP13A1	30	3.62702E-05	5.63364E-05
ENSGALG00000003624	KLHL10	27	3.62746E-05	5.63364E-05

ENSGALG00000023454	C9ORF152	2	3.63412E-05	5.64325E-05
ENSGALG00000048365	GPATCH4	25	3.65911E-05	5.68133E-05
ENSGALG00000021395		18	3.70302E-05	5.74878E-05
ENSGALG00000012454	CYTH4	1	3.73646E-05	5.79995E-05
ENSGALG00000017136	GJB6	1	3.75211E-05	5.82351E-05
ENSGALG00000006323	LECT2	13	3.7571E-05	5.83051E-05
ENSGALG00000004871	RPS24	6	3.76311E-05	5.83909E-05
ENSGALG00000043025		5	3.77332E-05	5.8542E-05
ENSGALG00000015275	NIT2	1	3.7775E-05	5.85993E-05
ENSGALG00000017104	CDK8	1	3.77819E-05	5.86025E-05
ENSGALG00000007513		7	3.78198E-05	5.86538E-05
ENSGALG00000001593	UFD1L	15	3.79519E-05	5.88513E-05
ENSGALG00000035641		32	3.80691E-05	5.90255E-05
ENSGALG00000011392	MSH4	8	3.81319E-05	5.91153E-05
ENSGALG00000000603	KANSL1	27	3.82081E-05	5.9226E-05
ENSGALG00000021171		10	3.83214E-05	5.9394E-05
ENSGALG00000011489	TBXT	3	3.85935E-05	5.98082E-05
ENSGALG00000035094	CDH17	2	3.89241E-05	6.03128E-05
ENSGALG00000039897	FBXO22	10	3.8929E-05	6.03128E-05
ENSGALG00000035236	BCAP31	30	3.89537E-05	6.03433E-05
ENSGALG00000004475		4	3.90469E-05	6.04801E-05
ENSGALG00000004747		6	3.92399E-05	6.07713E-05
ENSGALG00000003766	KIAA0100	19	3.94265E-05	6.10525E-05
ENSGALG00000009592	SRP14	5	3.95695E-05	6.12661E-05
ENSGALG00000016052	VPS26C	1	3.96076E-05	6.13174E-05
ENSGALG00000012677	CDKAL1	2	3.98585E-05	6.16979E-05
ENSGALG00000015010	NSG1	4	4.07521E-05	6.30731E-05
ENSGALG00000003648	SVIP	5	4.08872E-05	6.32742E-05
ENSGALG00000008037	HORMAD2	15	4.10036E-05	6.34463E-05
ENSGALG00000011293	MRPL42	1	4.11346E-05	6.3641E-05
ENSGALG00000014189	SULT4A1	1	4.13417E-05	6.39532E-05
ENSGALG00000007367	WFIKKN2	18	4.13765E-05	6.3999E-05
ENSGALG00000054588		31	4.1682E-05	6.44633E-05

ENSGALG00000011867	SERHL	1	4.2002E-05	6.49499E-05
ENSGALG00000046384		27	4.23144E-05	6.54247E-05
ENSGALG00000022758	GGACT	1	4.24572E-05	6.56372E-05
ENSGALG00000002563	TPM4	28	4.25705E-05	6.58041E-05
ENSGALG00000015733	MDN1	3	4.26519E-05	6.59214E-05
ENSGALG00000015879	HUNK	1	4.28988E-05	6.62948E-05
ENSGALG00000006594	POFUT1	20	4.30185E-05	6.64712E-05
ENSGALG00000002622	FCHSD1	13	4.34322E-05	6.7102E-05
ENSGALG00000012126	CFI	4	4.35593E-05	6.72899E-05
ENSGALG00000040926	SAMSN1	1	4.36647E-05	6.7444E-05
ENSGALG00000048599		6	4.39479E-05	6.7873E-05
ENSGALG00000005788	FARP2	9	4.41427E-05	6.81651E-05
ENSGALG00000003319	ADAP2	18	4.42053E-05	6.82531E-05
ENSGALG00000054944	GULP1	7	4.42306E-05	6.82835E-05
ENSGALG00000017050	SMAD9	1	4.43E-05	6.8382E-05
ENSGALG00000004695	ECM2	12	4.43628E-05	6.84703E-05
ENSGALG00000044404	FAM83D	20	4.44297E-05	6.85649E-05
ENSGALG00000017380	UQCRHL	8	4.50696E-05	6.95437E-05
ENSGALG00000026491	TMEM17	3	4.55382E-05	7.02577E-05
ENSGALG00000033170	GMDS	2	4.57442E-05	7.05667E-05
ENSGALG00000006131	FTCD	7	4.58676E-05	7.0748E-05
ENSGALG00000004859	ZNFX1	20	4.59102E-05	7.08049E-05
ENSGALG00000005808	KREMEN1	15	4.60639E-05	7.10329E-05
ENSGALG00000008438	SLC35C1	5	4.61323E-05	7.11294E-05
ENSGALG00000012694		1	4.62416E-05	7.12889E-05
ENSGALG00000032146	SLC4A5	22	4.62723E-05	7.13272E-05
ENSGALG00000046455		26	4.6461E-05	7.1609E-05
ENSGALG00000045424		33	4.65201E-05	7.1691E-05
ENSGALG00000041299	E2F8	5	4.66002E-05	7.18054E-05
ENSGALG00000012903	PIGN	2	4.66861E-05	7.19286E-05
ENSGALG00000004927	COQ4	17	4.6802E-05	7.20981E-05
ENSGALG00000053601	LPXN	5	4.73949E-05	7.30023E-05
ENSGALG00000047706		8	4.74681E-05	7.31059E-05

ENSGALG00000006010	FAM103A1	10	4.76934E-05	7.34354E-05
ENSGALG00000048910		1	4.76942E-05	7.34354E-05
ENSGALG00000025774	HOXB2	27	4.7782E-05	7.35614E-05
ENSGALG00000011017	KLHL8	4	4.79249E-05	7.37721E-05
ENSGALG00000039903	VSIG10	15	4.79825E-05	7.38514E-05
ENSGALG00000008677	VIM	2	4.81438E-05	7.40904E-05
ENSGALG00000000740	APBA3	28	4.81937E-05	7.41577E-05
ENSGALG00000003527	ARRDC2	28	4.82622E-05	7.42537E-05
ENSGALG00000001688	SEPT5	15	4.83585E-05	7.43925E-05
ENSGALG00000048011	SCLT1	4	4.85422E-05	7.46657E-05
ENSGALG00000030969	CLEC3B	2	4.86254E-05	7.47842E-05
ENSGALG00000008620	RPL39L	4	4.86771E-05	7.48543E-05
ENSGALG00000000011	C10orf88	6	4.876E-05	7.49723E-05
ENSGALG00000032803		5	4.89463E-05	7.52487E-05
ENSGALG00000031832	IKBKB	22	4.89521E-05	7.52487E-05
ENSGALG00000007387	GTF3C1	14	4.90823E-05	7.54394E-05
ENSGALG00000031298		22	4.91934E-05	7.56005E-05
ENSGALG00000009634	GFM1	9	4.94299E-05	7.59545E-05
ENSGALG00000042322	NAA60	14	4.94624E-05	7.59949E-05
ENSGALG00000001183	MYH10	18	4.96885E-05	7.63325E-05
ENSGALG00000012055		7	4.98539E-05	7.65771E-05
ENSGALG00000005957		4	4.99024E-05	7.66419E-05
ENSGALG00000031425	PMP22	18	5.02278E-05	7.71319E-05
ENSGALG00000002517	TMEM35B	23	5.07171E-05	7.78736E-05
ENSGALG00000010507	FCRL4	25	5.12636E-05	7.87027E-05
ENSGALG00000003331	FAF2	13	5.15276E-05	7.90981E-05
ENSGALG00000007669	EGR1	13	5.16039E-05	7.92053E-05
ENSGALG00000005849	PPDPF	20	5.16209E-05	7.92214E-05
ENSGALG00000039951	TRAP1	14	5.16988E-05	7.93309E-05
ENSGALG00000013830	PRELID3A	2	5.1802E-05	7.94792E-05
ENSGALG00000033295	CAV2	1	5.20904E-05	7.99118E-05
ENSGALG00000006189	CRYGN	2	5.21225E-05	7.99509E-05
ENSGALG00000047119		1	5.22488E-05	8.01345E-05

ENSGALG00000041634	ACTG2	22	5.23011E-05	8.02047E-05
ENSGALG00000043775	RAPGEF3	33	5.23637E-05	8.02905E-05
ENSGALG00000017024	NEK3	1	5.24232E-05	8.03717E-05
ENSGALG00000043435	CARNS1	5	5.24342E-05	8.03784E-05
ENSGALG00000007878	SDHD	24	5.25082E-05	8.04817E-05
ENSGALG00000006034	PITPNM3	19	5.2551E-05	8.05372E-05
ENSGALG00000023936		21	5.29006E-05	8.10628E-05
ENSGALG00000033854	CSF1	26	5.32609E-05	8.16047E-05
ENSGALG00000002379	MRPS17	19	5.32729E-05	8.16129E-05
ENSGALG00000010307	ACYP1	5	5.34242E-05	8.18343E-05
ENSGALG00000040433		1	5.3688E-05	8.22282E-05
ENSGALG00000027506	DNAJB2	7	5.37619E-05	8.23309E-05
ENSGALG00000028376	FGF19	5	5.38978E-05	8.25286E-05
ENSGALG00000005930	PLS3	4	5.4094E-05	8.28186E-05
ENSGALG00000042492	PITX2	4	5.43621E-05	8.32187E-05
ENSGALG00000010745	HMGB2	4	5.45075E-05	8.34308E-05
ENSGALG00000040002		25	5.47132E-05	8.37351E-05
ENSGALG00000041988	SIK1	1	5.48254E-05	8.38964E-05
ENSGALG00000050676		2	5.48856E-05	8.3978E-05
ENSGALG00000004874	PPIH	21	5.48942E-05	8.39806E-05
ENSGALG00000003923	COL6A3	7	5.50214E-05	8.41646E-05
ENSGALG00000003203		12	5.50301E-05	8.41673E-05
ENSGALG00000003233	ASH2L	22	5.53325E-05	8.46193E-05
ENSGALG00000011865	SMTN	15	5.5652E-05	8.50911E-05
ENSGALG00000006441	GNPTG	14	5.5655E-05	8.50911E-05
ENSGALG00000053483		13	5.60468E-05	8.56793E-05
ENSGALG00000007075	ATP13A3	9	5.63702E-05	8.61629E-05
ENSGALG00000007028		4	5.65931E-05	8.64929E-05
ENSGALG00000005572	NOXO1	14	5.67883E-05	8.67802E-05
ENSGALG00000015722	TMED11	4	5.68057E-05	8.6796E-05
ENSGALG00000007582	DPYSL3	13	5.69433E-05	8.69954E-05
ENSGALG00000046226	SCARB1	15	5.69542E-05	8.70011E-05
ENSGALG00000011884	EXOSC9	4	5.71358E-05	8.72676E-05

ENSGALG00000054981		1	5.72714E-05	8.74636E-05
ENSGALG00000032658	FMNL1	27	5.74157E-05	8.76731E-05
ENSGALG00000009070	HSPE1	7	5.82724E-05	8.89701E-05
ENSGALG00000050083	SYCP2L	2	5.83154E-05	8.90247E-05
ENSGALG00000029571	TMEM55A	2	5.87259E-05	8.964E-05
ENSGALG00000007353	MTFMT	10	5.90473E-05	9.01193E-05
ENSGALG00000006503	VDAC1	13	5.90552E-05	9.01202E-05
ENSGALG00000041222	CYP7A1	2	5.90769E-05	9.0142E-05
ENSGALG00000009677	SSB	7	5.915E-05	9.02423E-05
ENSGALG00000028001	COMMD2	9	5.9491E-05	9.07511E-05
ENSGALG00000030776		33	5.95343E-05	9.08058E-05
ENSGALG00000003750	PLCG1	20	5.97505E-05	9.11242E-05
ENSGALG00000003323	NECAB2	11	5.9823E-05	9.12233E-05
ENSGALG00000013441	INVS	2	6.00721E-05	9.15918E-05
ENSGALG00000044801	CHIR-B5	31	6.04227E-05	9.21148E-05
ENSGALG00000046607	SCNM1	25	6.07168E-05	9.25515E-05
ENSGALG00000023872	NIBAN2	17	6.08615E-05	9.27605E-05
ENSGALG00000027482	SNTB2	11	6.1598E-05	9.38713E-05
ENSGALG00000030175	FBXL2	2	6.19416E-05	9.43831E-05
ENSGALG00000037366	GRID1	6	6.2291E-05	9.49036E-05
ENSGALG00000037283	CUTC	6	6.26611E-05	9.54556E-05
ENSGALG00000014687	EPHA1	1	6.28811E-05	9.57788E-05
ENSGALG00000044763	GPR82	1	6.31022E-05	9.61035E-05
ENSGALG00000000747	CHD5	21	6.31655E-05	9.61879E-05
ENSGALG00000053424		2	6.35983E-05	9.68349E-05
ENSGALG00000031000	APRT	11	6.37101E-05	9.6993E-05
ENSGALG00000034511		1	6.37216E-05	9.69984E-05
ENSGALG00000002612	U2SURP	9	6.38404E-05	9.71672E-05
ENSGALG00000000003	PANX2	1	6.38692E-05	9.71989E-05
ENSGALG00000049422	ATF3	3	6.42668E-05	9.77917E-05
ENSGALG00000035935		10	6.45012E-05	9.81361E-05
ENSGALG00000016359	FBXO25	3	6.45897E-05	9.82586E-05
ENSGALG00000040891	RSU1	2	6.46162E-05	9.82866E-05

ENSGALG00000009365	CYP51A1	2	6.47064E-05	9.84115E-05
ENSGALG00000032467	C11orf52	24	6.49688E-05	9.87982E-05
ENSGALG00000009068	ITPKB	3	6.54496E-05	9.9517E-05
ENSGALG00000002013	MEAF6	23	6.55869E-05	9.97133E-05
ENSGALG00000004230	LIPC	10	6.63661E-05	0.000100885
ENSGALG00000051412	PEA15	23	6.68423E-05	0.000101597
ENSGALG00000003757	POMT1	17	6.76904E-05	0.000102873
ENSGALG00000000785	HYAL1	12	6.77619E-05	0.000102969
ENSGALG00000053846		1	6.78471E-05	0.000103085
ENSGALG00000012048	LARP7	4	6.79569E-05	0.000103239
ENSGALG00000013867	PTPN2	2	6.81936E-05	0.000103586
ENSGALG00000001889		17	6.82152E-05	0.000103606
ENSGALG00000004509		19	6.8248E-05	0.000103643
ENSGALG00000009477	PDCD10	9	6.87347E-05	0.000104369
ENSGALG00000047176		3	6.88793E-05	0.000104575
ENSGALG00000019794	MSTO1	25	6.89691E-05	0.000104699
ENSGALG00000009553	SMC4	9	6.92328E-05	0.000105086
ENSGALG00000048035	GCNT2	2	6.93344E-05	0.000105227
ENSGALG00000006639	CEP55	6	6.98678E-05	0.000106023
ENSGALG00000013637	TDP2	2	6.99549E-05	0.000106142
ENSGALG00000002081	MMP28	19	7.00392E-05	0.000106257
ENSGALG00000033520	DCSTAMP	2	7.00822E-05	0.000106309
ENSGALG00000042340	GTF2H1	5	7.01785E-05	0.000106442
ENSGALG00000046379		1	7.02971E-05	0.000106609
ENSGALG00000002784	APEH	12	7.06565E-05	0.00010714
ENSGALG00000050508	NOS3	2	7.12409E-05	0.000108001
ENSGALG00000015235	XCL1	1	7.12418E-05	0.000108001
ENSGALG00000046008	RSPH3	3	7.13223E-05	0.0001081
ENSGALG00000011668	IL8L1	4	7.1325E-05	0.0001081
ENSGALG00000016348	SAT1	1	7.151E-05	0.000108367
ENSGALG00000052844		30	7.18671E-05	0.00010888
ENSGALG00000044299	CFAP92	12	7.18676E-05	0.00010888
ENSGALG00000049360	PPP1R10	16	7.1875E-05	0.00010888

ENSGALG00000003014	CHRNA3	10	7.23585E-05	0.000109598
ENSGALG00000016785	IL1RL1	1	7.27468E-05	0.000110173
ENSGALG00000003259	EIF2B1	15	7.27558E-05	0.000110173
ENSGALG00000015037	EVC	4	7.30377E-05	0.000110586
ENSGALG00000010961	IGF2BP3	2	7.34168E-05	0.000111146
ENSGALG00000002804	RND2	27	7.34267E-05	0.000111147
ENSGALG00000006352	CH25H	6	7.51317E-05	0.000113714
ENSGALG00000035930	PHACTR3	20	7.52236E-05	0.000113839
ENSGALG00000046326	IL1R2	1	7.53454E-05	0.000114003
ENSGALG00000012032	PRRG4	5	7.53505E-05	0.000114003
ENSGALG00000010193	ZNF410	5	7.55673E-05	0.000114317
ENSGALG00000034741	ETNPPL	4	7.57107E-05	0.000114519
ENSGALG00000005215	CACNA1H	14	7.57201E-05	0.000114519
ENSGALG00000016232	RPL8	1	7.57662E-05	0.000114575
ENSGALG00000049448		16	7.67586E-05	0.000116061
ENSGALG00000009733	SHFM1	2	7.70652E-05	0.00011651
ENSGALG00000002105	FOXI1	13	7.81479E-05	0.000118133
ENSGALG00000028204	GPX1	12	7.81825E-05	0.00011817
ENSGALG00000009523	CNTN1	1	7.92286E-05	0.000119737
ENSGALG00000006571	SETD5	12	7.99425E-05	0.0001208
ENSGALG00000032335	MARCKSL1	23	8.03918E-05	0.000121464
ENSGALG00000011543	SDAD1	4	8.05916E-05	0.000121751
ENSGALG00000002675	SCUBE3	26	8.08654E-05	0.00012215
ENSGALG00000048098		23	8.11023E-05	0.000122492
ENSGALG00000016102	CYP26B1	4	8.16457E-05	0.000123298
ENSGALG00000005541	GJB1	4	8.1878E-05	0.000123633
ENSGALG00000013748	CD226	2	8.22909E-05	0.000124241
ENSGALG00000045090		31	8.23402E-05	0.0001243
ENSGALG00000011651	ARL1	1	8.28479E-05	0.000125051
ENSGALG00000014589		28	8.3035E-05	0.000125318
ENSGALG00000000560		26	8.31055E-05	0.000125409
ENSGALG00000019844	AvBD8	3	8.3246E-05	0.000125606
ENSGALG00000030602	ADAM33	4	8.46106E-05	0.000127649

ENSGALG00000004930	HRH1	12	8.52735E-05	0.000128633
ENSGALG00000027823	TFAP4	14	8.54442E-05	0.000128874
ENSGALG00000003966		19	8.58828E-05	0.00012952
ENSGALG00000005226	MYOZ1	6	8.65294E-05	0.000130479
ENSGALG00000012339	ATL1	5	8.67663E-05	0.00013082
ENSGALG00000000120	BMP10	22	8.68014E-05	0.000130857
ENSGALG00000040620	LSAMP	1	8.70327E-05	0.000131187
ENSGALG00000026896		1	8.70417E-05	0.000131187
ENSGALG00000040768	MAL2	2	8.73089E-05	0.000131573
ENSGALG00000010346	TGFB3	5	8.75942E-05	0.000131987
ENSGALG00000005452	TADA2A	19	8.77587E-05	0.000132218
ENSGALG00000007220	FTH1	5	8.80245E-05	0.000132602
ENSGALG00000001504	DDX20	26	8.82922E-05	0.000132989
ENSGALG00000001951		8	8.86659E-05	0.000133536
ENSGALG00000021685	SERINC2	23	8.90256E-05	0.000134061
ENSGALG00000000881	GPATCH3	23	8.91908E-05	0.000134293
ENSGALG00000016815		1	9.05344E-05	0.000136299
ENSGALG00000001533		11	9.05535E-05	0.000136311
ENSGALG00000052452		1	9.06507E-05	0.00013644
ENSGALG00000010721	GLIS1	8	9.223E-05	0.0001388
ENSGALG00000047166		1	9.22592E-05	0.000138827
ENSGALG00000011182	DLL1	3	9.24897E-05	0.000139157
ENSGALG00000021683	RER1	21	9.4035E-05	0.000141464
ENSGALG00000017072		1	9.41659E-05	0.000141644
ENSGALG00000037136	RPS6KA1	23	9.4313E-05	0.000141848
ENSGALG00000008633	SEPT6	4	9.48712E-05	0.000142669
ENSGALG00000051184		1	9.49085E-05	0.000142708
ENSGALG00000009682	VEPH1	9	9.50943E-05	0.00014297
ENSGALG00000019042	ALG8	1	9.57956E-05	0.000144006
ENSGALG00000034591		2	9.62672E-05	0.000144698
ENSGALG00000040478	CAPN5	1	9.74331E-05	0.000146432
ENSGALG00000049268	H2A-VIII	1	9.75866E-05	0.000146645
ENSGALG00000005102	CDH4	20	9.79843E-05	0.000147224

ENSGALG00000010312	PLCH1	9	9.85231E-05	0.000148015
ENSGALG00000033811	PTPRN	7	9.89045E-05	0.00014857
ENSGALG00000020386	BDKRB1	5	9.93037E-05	0.000149151
ENSGALG00000006294	PSMD2	9	0.000100751	0.000151307
ENSGALG00000028924	S100A16	25	0.000101006	0.00015167
ENSGALG00000047202		33	0.000101109	0.000151807
ENSGALG00000028872	TPT1	1	0.000101251	0.000152001
ENSGALG00000004221	IL22RA1	23	0.000101296	0.00015205
ENSGALG00000031216	FLOT2	19	0.000101334	0.000152088
ENSGALG00000012220	CDKN3	5	0.000102431	0.000153716
ENSGALG00000041775	ESRRB	5	0.000103387	0.000155132
ENSGALG00000007186	TMEM268	17	0.000103773	0.000155692
ENSGALG00000016608	ADCY3	3	0.000104629	0.000156957
ENSGALG00000008937	PPP1R21	3	0.000104715	0.000157066
ENSGALG00000003425	GALR1L	11	0.000104941	0.000157385
ENSGALG00000053321	DOK2	22	0.000105468	0.000158156
ENSGALG00000029678	SNX16	2	0.000105778	0.000158602
ENSGALG00000040238	IMP3	3	0.000105978	0.000158882
ENSGALG00000022702	CHRNA10	1	0.000106638	0.000159853
ENSGALG00000015410	BVES	3	0.000106884	0.000160201
ENSGALG00000012422		1	0.00010703	0.0001604
ENSGALG00000021193	STARD5	10	0.000107236	0.000160683
ENSGALG00000006053	SLC2A11	15	0.000107245	0.000160683
ENSGALG00000003058		28	0.000107436	0.00016095
ENSGALG00000000225	MGAT4C	26	0.000107745	0.000161393
ENSGALG00000027232	C1QTNF2	13	0.000107843	0.000161519
ENSGALG00000005037		19	0.000108141	0.000161946
ENSGALG00000039387	PSMA2	2	0.000108617	0.000162639
ENSGALG00000050532	B3GNT3	28	0.000108881	0.000163014
ENSGALG00000040833		7	0.000108988	0.000163154
ENSGALG00000015093	IMPACT	2	0.000109461	0.000163842
ENSGALG00000003357	FKBP8	28	0.000109498	0.000163878
ENSGALG00000033678	EHMT1	17	0.000109541	0.000163922

ENSGALG00000005088	CAMK2G	6	0.00010971	0.000164154
ENSGALG00000043290		3	0.000109749	0.000164192
ENSGALG00000004940	TAMM41	12	0.000110659	0.000165534
ENSGALG00000006455	SMPD4	15	0.000110815	0.000165747
ENSGALG00000036390	PPP1R17	2	0.000111008	0.000166015
ENSGALG00000028830		15	0.000111214	0.000166303
ENSGALG00000029528	KIRREL1	25	0.000111645	0.000166927
ENSGALG00000000619	ANGPTL4	28	0.00011273	0.000168528
ENSGALG00000035419	CDON	24	0.000113206	0.000169218
ENSGALG00000050400		33	0.000113564	0.000169734
ENSGALG00000037709	SDF2	19	0.000114224	0.000170699
ENSGALG00000048223		1	0.000114674	0.000171351
ENSGALG00000034232		6	0.000114928	0.000171709
ENSGALG00000027629	GNG2	5	0.000116687	0.000174315
ENSGALG00000032489	ELOF1	30	0.000116872	0.000174571
ENSGALG00000036725	ASCL3	5	0.00011744	0.000175397
ENSGALG00000016820	GAS6	1	0.000117545	0.000175533
ENSGALG00000055006	AHDC1	23	0.000118107	0.00017635
ENSGALG00000006830	CHID1	5	0.000118444	0.000176832
ENSGALG00000032984	MEX3B	10	0.000119575	0.000178498
ENSGALG00000042254		6	0.000120048	0.000179183
ENSGALG00000004849	WSCD2	15	0.000120076	0.000179203
ENSGALG00000001465	RPL38	18	0.000120131	0.000179262
ENSGALG00000050158		18	0.000120384	0.000179619
ENSGALG00000029226	ZFAND2B	7	0.000120577	0.000179884
ENSGALG00000043736		33	0.000120855	0.000180278
ENSGALG00000050256	TRPT1	33	0.000121091	0.000180607
ENSGALG00000007767	DIS3L2	9	0.000121123	0.000180633
ENSGALG00000007689	CENATAC	24	0.000121156	0.00018066
ENSGALG00000028341		28	0.000121406	0.000181011
ENSGALG00000027036	PKIB	3	0.000121905	0.000181733
ENSGALG00000024318	TMEM200B	23	0.000122283	0.000182274
ENSGALG00000002850	RASA2	9	0.00012245	0.0001825

ENSGALG00000009822	PACC1	3	0.000122719	0.000182863
ENSGALG00000009973	SLC3A1	3	0.000122723	0.000182863
ENSGALG00000002671		15	0.000122789	0.000182939
ENSGALG00000039891	ANP32E	25	0.000123185	0.000183506
ENSGALG00000043654		27	0.000123475	0.000183915
ENSGALG00000011721		5	0.00012402	0.000184704
ENSGALG00000002200	ACSM3	14	0.000124131	0.000184847
ENSGALG00000004057	HMGCL	23	0.000125531	0.00018691
ENSGALG00000000990	SRSF6	20	0.000125869	0.00018739
ENSGALG00000008756	PLEK	3	0.000125962	0.000187505
ENSGALG00000020005	SFT2D1	3	0.000127375	0.000189586
ENSGALG00000030900	NCMAP	23	0.00012751	0.000189763
ENSGALG00000002118	KCNMB1	13	0.000129647	0.000192921
ENSGALG00000043026		24	0.000129956	0.000193356
ENSGALG00000001968	SYT6	26	0.000130202	0.000193698
ENSGALG00000005160	VMP1	19	0.000130302	0.000193823
ENSGALG00000009157	ECT2	9	0.000131912	0.000196195
ENSGALG00000047242	MAST1	30	0.000132281	0.00019672
ENSGALG00000042112	EIF4E	4	0.000133971	0.000199208
ENSGALG00000003361	TREM-B1	26	0.000134101	0.000199377
ENSGALG00000031860		2	0.000134385	0.000199776
ENSGALG00000016684	RHAG	3	0.000134784	0.000200344
ENSGALG00000051567	MRPL41	17	0.000135035	0.000200674
ENSGALG00000016294	HMGCLL1	3	0.000135046	0.000200674
ENSGALG00000014680		1	0.000135056	0.000200674
ENSGALG00000012319		4	0.000136034	0.000202103
ENSGALG00000000378	SLC25A37	22	0.0001361	0.000202177
ENSGALG00000036218	RAB4A	3	0.000137257	0.000203871
ENSGALG00000010654	TRAPPC11	4	0.000137665	0.000204452
ENSGALG00000015328	TRMT10C	1	0.000138132	0.00020512
ENSGALG00000001472	JAM3	24	0.000138418	0.000205519
ENSGALG00000002708	LINGO1	10	0.000138693	0.000205903
ENSGALG00000050677	BRICD5	14	0.000139052	0.000206411

ENSGALG00000023193	SOCS1L	1	0.000139826	0.000207534
ENSGALG00000028056		3	0.000140112	0.000207934
ENSGALG00000015701	CXADR	1	0.000140296	0.000208174
ENSGALG00000005408	BCO1	11	0.000140308	0.000208174
ENSGALG00000050539		33	0.000141285	0.000209599
ENSGALG00000023279	EPN3	18	0.00014159	0.000210025
ENSGALG00000005749	COX4I1	11	0.000142033	0.000210656
ENSGALG00000008018	SNAI1	20	0.000142125	0.000210767
ENSGALG00000011388	RABGGTB	8	0.000142392	0.000211137
ENSGALG00000016570		3	0.000142913	0.000211885
ENSGALG00000023191	MRPS18C	4	0.00014428	0.000213885
ENSGALG00000029261	RTN4	3	0.000144395	0.00021403
ENSGALG00000033894	MEA1	3	0.000144606	0.000214317
ENSGALG00000008765	C1D	3	0.00014555	0.000215689
ENSGALG00000009012	ZNF385B	7	0.000145964	0.000216277
ENSGALG00000004274	TWIST2	7	0.000146867	0.000217589
ENSGALG00000008414	DVL3	9	0.000147215	0.000218077
ENSGALG00000028448	SH2D1B	1	0.000148155	0.000219443
ENSGALG00000007717	GCNT7	20	0.000148252	0.00021956
ENSGALG00000029001	YRDC	23	0.000148431	0.000219799
ENSGALG00000010497	SLC29A4	14	0.000149053	0.000220693
ENSGALG00000001540	SF3A3	23	0.000150722	0.000223137
ENSGALG00000008550	MRPS5	3	0.000151028	0.000223563
ENSGALG00000043676		2	0.000152946	0.000226373
ENSGALG00000016215	DIPK2B	1	0.000153383	0.000226993
ENSGALG00000051905		5	0.00015399	0.000227856
ENSGALG00000030862	MED30	2	0.000154003	0.000227856
ENSGALG00000001654	C1QBP	19	0.000154619	0.000228739
ENSGALG00000012021		5	0.000154887	0.000229107
ENSGALG00000001542	MRPL20	21	0.000155271	0.000229633
ENSGALG00000050119	LAMTOR1	1	0.00015528	0.000229633
ENSGALG00000030027	MCAM	24	0.000155396	0.000229777
ENSGALG00000050314	SRSF4	23	0.000155562	0.000229996

ENSGALG00000046955		14	0.000156057	0.000230698
ENSGALG00000042365	CARD10	1	0.000156945	0.000231984
ENSGALG00000000946	PFDN1	13	0.00015821	0.000233825
ENSGALG00000028308	TNS4	27	0.000158738	0.000234577
ENSGALG00000054674		23	0.000158878	0.000234756
ENSGALG00000007090	CPN1	6	0.000160289	0.000236812
ENSGALG00000055056		19	0.000160572	0.0002372
ENSGALG00000011130	COPS4	4	0.000162205	0.000239585
ENSGALG00000017159	ATM	1	0.000163598	0.000241612
ENSGALG00000040366	NDUFAF6	2	0.000164534	0.000242965
ENSGALG00000003354	KCNH4	27	0.000164584	0.00024301
ENSGALG00000035184	SDC3	23	0.000164756	0.000243234
ENSGALG00000012237	POLE2	5	0.000164887	0.000243398
ENSGALG00000014442	GAPDH	1	0.000165478	0.000244241
ENSGALG00000042181	DDHD2	22	0.000165961	0.000244925
ENSGALG00000003153	COPE	28	0.000166188	0.000245229
ENSGALG00000045540		1	0.000166261	0.000245307
ENSGALG00000005516	HEATR6	19	0.000167239	0.000246721
ENSGALG00000007426	MKX	2	0.000167411	0.000246945
ENSGALG00000009610	PSTK	6	0.000168208	0.00024809
ENSGALG00000019384	MAPK12	1	0.000169697	0.000250255
ENSGALG00000030705	TRABD	1	0.000170393	0.000251252
ENSGALG00000022850	TMEM214	3	0.000170488	0.000251361
ENSGALG00000016389	RNASEH1	3	0.000170993	0.000252076
ENSGALG00000016845	NAXD	1	0.000172268	0.000253925
ENSGALG00000052122		31	0.000172463	0.000254182
ENSGALG00000016731	MRPL19	3	0.000173094	0.00025508
ENSGALG00000004492	PIGL	19	0.000173374	0.000255463
ENSGALG00000051206	RBMX2	4	0.000173828	0.0002561
ENSGALG00000044098		2	0.000173867	0.000256127
ENSGALG00000041277	UBB	15	0.000174626	0.000257214
ENSGALG00000033591		13	0.000174864	0.000257533
ENSGALG00000011013	PCNX2	3	0.00017526	0.00025808

ENSGALG00000011366	MOK	5	0.000175278	0.00025808
ENSGALG00000000352	TIMM44	28	0.000175317	0.000258106
ENSGALG00000013804		4	0.000175425	0.000258235
ENSGALG00000043831	LRRC73	3	0.000175516	0.000258337
ENSGALG00000017384	ERH	5	0.000175945	0.000258937
ENSGALG00000050824		31	0.000176017	0.000259012
ENSGALG00000037913	KLF10	2	0.000176252	0.000259327
ENSGALG00000038884	SRL	14	0.000177105	0.000260551
ENSGALG00000040270	ZNF687	25	0.000177283	0.00026078
ENSGALG00000004760	TMEM116	15	0.000177756	0.000261444
ENSGALG00000013688	UROS	6	0.000178239	0.000262123
ENSGALG00000034939		5	0.00017917	0.000263445
ENSGALG00000003282	STAT5A	27	0.00017918	0.000263445
ENSGALG00000002112	CSF3R	23	0.000179505	0.00026389
ENSGALG00000002853	CFDP1	11	0.00017986	0.000264381
ENSGALG00000005118	FAM124B	9	0.000179951	0.000264481
ENSGALG00000039146	CCR4	2	0.00018074	0.00026561
ENSGALG00000025907	ZBTB7A	28	0.000182104	0.000267582
ENSGALG00000000791		26	0.000183485	0.000269578
ENSGALG00000053778		1	0.000184161	0.000270539
ENSGALG00000027902	SYNGR2	18	0.000185986	0.000273187
ENSGALG00000034688	TIMELESS	33	0.000186108	0.000273334
ENSGALG00000048102		4	0.000186231	0.000273481
ENSGALG00000050937		30	0.000187094	0.000274715
ENSGALG00000008819	LRSAM1	17	0.000187134	0.000274741
ENSGALG00000046674	CTU2	11	0.000188321	0.000276451
ENSGALG00000008840	ACSL5	6	0.000188468	0.000276633
ENSGALG00000007543	APCDD1L	20	0.000189729	0.000278451
ENSGALG00000005022	GP9	12	0.000189938	0.000278723
ENSGALG00000005000	COMTD1	6	0.000190029	0.000278824
ENSGALG00000002284	TRAPPC3	23	0.000190153	0.000278972
ENSGALG00000050668		8	0.000190879	0.000280003
ENSGALG00000004424	SEC16B	8	0.000191148	0.000280364

ENSGALG00000007072	ACADS	15	0.000191954	0.000281513
ENSGALG00000042894	SLC25A39	27	0.000193513	0.000283765
ENSGALG00000029416	TRPC6	1	0.000193716	0.000284028
ENSGALG00000033772	UPF2	1	0.00019397	0.000284367
ENSGALG00000003655	GAS2	5	0.000194939	0.000285753
ENSGALG00000052425	BATF3	3	0.000197511	0.000289489
ENSGALG00000015343	RTN4IP1	3	0.000199039	0.000291693
ENSGALG00000011880	BBS7	4	0.000199104	0.000291753
ENSGALG00000014981	COX17	1	0.000199215	0.00029188
ENSGALG00000042356	FSTL3	28	0.000199385	0.000292095
ENSGALG00000036491	DRAM2	26	0.000199668	0.000292471
ENSGALG00000020615	STK32C	6	0.00019969	0.000292471
ENSGALG00000008803		7	0.00020044	0.000293534
ENSGALG00000006794		13	0.000202511	0.000296531
ENSGALG00000010009	TTC29	4	0.000202712	0.00029679
ENSGALG00000005140	RRAD	11	0.000202967	0.000297098
ENSGALG00000037497	CABP2	5	0.000202971	0.000297098
ENSGALG00000054240		30	0.000203306	0.000297553
ENSGALG00000009288	RNPS1	14	0.000203816	0.000298263
ENSGALG00000004952	RPL36AL	4	0.000204028	0.000298538
ENSGALG00000015143	TTR	2	0.000204519	0.00029922
ENSGALG00000012197	ACMSD	7	0.000205305	0.000300334
ENSGALG00000016686	AKAP17A	1	0.00020569	0.000300862
ENSGALG00000004740	TMOD2	10	0.000207431	0.000303337
ENSGALG00000009871	RCOR3	3	0.000207432	0.000303337
ENSGALG00000016816	CDC16	1	0.000207532	0.000303447
ENSGALG00000016713	PLA2G7	3	0.000210007	0.000307028
ENSGALG00000001749	ACSBG2	28	0.000210104	0.000307133
ENSGALG00000001977	LDB3	6	0.000212476	0.000310563
ENSGALG00000014250	PHOX2B	4	0.000213094	0.00031143
ENSGALG00000007160		4	0.000213493	0.000311976
ENSGALG00000007193		9	0.000213732	0.000312287
ENSGALG00000026284	IKBIP	1	0.000214243	0.000312996

ENSGALG00000006056		12	0.00021585	0.000315306
ENSGALG00000035223	NDUFA6	1	0.000216019	0.000315515
ENSGALG00000031255	FGF1	13	0.000216724	0.000316507
ENSGALG00000006564		9	0.000217511	0.000317618
ENSGALG00000012410		1	0.000217887	0.000318129
ENSGALG00000046212	MILR1	18	0.000219825	0.00032092
ENSGALG00000000544		26	0.000220278	0.000321543
ENSGALG00000011306	DES	7	0.000220553	0.000321905
ENSGALG00000002288	LIG3	19	0.000220923	0.000322407
ENSGALG00000011189	DYNLT2	3	0.000222443	0.000324587
ENSGALG00000013893	MNS1	10	0.000223188	0.000325635
ENSGALG00000000950	MVB12B	17	0.000223249	0.000325685
ENSGALG00000016286	TASL	1	0.000224792	0.000327897
ENSGALG00000003517	ZBTB8OS	23	0.000224915	0.000328036
ENSGALG00000038162	FAM26E	3	0.000225474	0.000328812
ENSGALG00000036747	TTC31	4	0.000227406	0.000331591
ENSGALG00000037015	DACH1	1	0.000229165	0.000334116
ENSGALG00000049362		31	0.000229295	0.000334265
ENSGALG00000017307	POLD3	1	0.000229464	0.000334471
ENSGALG00000011570	ILF2	25	0.000230414	0.000335816
ENSGALG00000051311		22	0.000234164	0.000341221
ENSGALG00000043728	SCN8A	33	0.000234179	0.000341221
ENSGALG00000049514	C16orf89	14	0.000236395	0.00034441
ENSGALG00000046593		5	0.000236988	0.000345233
ENSGALG00000047632		33	0.000237711	0.000346245
ENSGALG00000007841		7	0.000237911	0.000346493
ENSGALG00000002666	RAB11FIP3	14	0.00023819	0.000346859
ENSGALG00000040632	SDHAF2	5	0.00023873	0.000347604
ENSGALG00000037170	TRIM39.2	16	0.000239784	0.000349097
ENSGALG00000038242	CACNA2D2	12	0.000240393	0.000349941
ENSGALG00000014486	TAPT1	4	0.000241988	0.000352222
ENSGALG00000031813	CASC3	27	0.000242287	0.000352614
ENSGALG00000054154		27	0.000242921	0.000353495

ENSGALG00000031563	ECI1	14	0.000243215	0.000353881
ENSGALG00000051325		1	0.000244498	0.000355704
ENSGALG00000043416		25	0.000244834	0.000356151
ENSGALG00000003034	SS2	21	0.000245241	0.000356701
ENSGALG00000038672		23	0.000245626	0.000357218
ENSGALG00000032307	DDX23	33	0.000245781	0.000357401
ENSGALG00000039624	PSMA5	26	0.000245985	0.000357655
ENSGALG00000015612	LRPAP1	4	0.00024643	0.000358259
ENSGALG00000011625	MRPL18	3	0.000249023	0.000361985
ENSGALG00000014540	KANSL3	22	0.000250505	0.000364097
ENSGALG00000044581	MIIP	21	0.000252079	0.000366341
ENSGALG00000008282	ATG4A	4	0.000252824	0.00036738
ENSGALG00000003015	SERPINF1	19	0.000254029	0.000369087
ENSGALG00000039783	MRPL57	1	0.000254987	0.000370435
ENSGALG00000005594	OMG	19	0.000255294	0.000370836
ENSGALG00000040648	RGS11	14	0.000259753	0.000377268
ENSGALG00000022137	CRP	25	0.000260793	0.000378734
ENSGALG00000009111	HAUS2	5	0.000261244	0.000379343
ENSGALG00000041955	LRRC56	5	0.000261283	0.000379356
ENSGALG00000028871	SLC38A3	12	0.000261854	0.000380139
ENSGALG00000043493	IGFBP1	2	0.000262667	0.000381273
ENSGALG00000011386	BCS1L	7	0.000263083	0.000381833
ENSGALG00000036432	DNAJC17	5	0.000263466	0.000382342
ENSGALG00000016886	FARP1	1	0.000263981	0.000383045
ENSGALG00000041744	OTOS	9	0.000264646	0.000383963
ENSGALG00000000162	DMB1	16	0.000265134	0.000384626
ENSGALG00000005411	MEIOB	14	0.000267403	0.000387871
ENSGALG00000049461	BG8	16	0.000268109	0.000388849
ENSGALG00000003283		28	0.000268257	0.000388975
ENSGALG00000006431	PANK1	6	0.00026826	0.000388975
ENSGALG00000004998	RAF1	12	0.000269291	0.000390424
ENSGALG00000038069	CCR8	2	0.000269737	0.000391024
ENSGALG00000039863	DDX25	19	0.000270135	0.000391555

ENSGALG00000001416	ADRA1B	13	0.00027074	0.000392385
ENSGALG00000052653	PCDHAC1	13	0.000270784	0.000392401
ENSGALG00000027068	HDGFRP2	28	0.000271034	0.000392718
ENSGALG00000041754	N4BP3	13	0.000272058	0.000394155
ENSGALG00000003815	RPN2	20	0.000272958	0.000395411
ENSGALG00000019316	TOMM22	1	0.000273701	0.00039644
ENSGALG00000051779	PRORSD1P	3	0.000275029	0.000398316
ENSGALG00000013956	MYB	3	0.000275698	0.000399238
ENSGALG00000029621	SEMA3F	12	0.000276004	0.000399633
ENSGALG00000041129	TMEM173	13	0.000279156	0.00040415
ENSGALG00000029927	STRBP	17	0.000280667	0.000406289
ENSGALG00000051266		20	0.000280807	0.000406444
ENSGALG00000005739	SCD	6	0.000281255	0.00040701
ENSGALG00000015884	MIS18A	1	0.000281265	0.00040701
ENSGALG00000050799		30	0.000282208	0.000408326
ENSGALG00000030917	CCDC65	33	0.000282442	0.000408616
ENSGALG00000013033		2	0.000285445	0.000412912
ENSGALG00000023359	BTBD17	18	0.000287396	0.000415684
ENSGALG00000031853	PIGBOS1	10	0.000287657	0.000416013
ENSGALG00000003703	USE1	28	0.000288691	0.000417458
ENSGALG00000043825	NISCH	12	0.00028979	0.000418997
ENSGALG00000006773	TRMT12	4	0.00028999	0.000419237
ENSGALG00000012703	DTNBP1	2	0.000292075	0.000422202
ENSGALG00000054037		19	0.000292547	0.000422834
ENSGALG00000009031	SESTD1	7	0.000292616	0.000422884
ENSGALG00000015830	SYNCRIP	3	0.000292742	0.000423015
ENSGALG00000007027	SGSH	18	0.000293158	0.000423566
ENSGALG00000004920	TRUB2	17	0.000294185	0.000424999
ENSGALG00000026279	COMMD8	4	0.000295736	0.000427189
ENSGALG00000002789	MAP2	7	0.000295875	0.000427339
ENSGALG00000003242	STAR	22	0.00029596	0.000427412
ENSGALG00000009286	ATP5G3	7	0.000299703	0.000432766
ENSGALG00000002082	AMPD1	26	0.000300027	0.000433182

ENSGALG00000007736	ZWILCH	10	0.000302014	0.000436
ENSGALG00000010707	GPR68	5	0.000303086	0.000437496
ENSGALG00000012039	PSMA3	5	0.000307484	0.000443792
ENSGALG00000023546		9	0.000310162	0.000447604
ENSGALG00000020922	C1orf21	8	0.000310397	0.00044789
ENSGALG00000013371	S1PR4	28	0.000310852	0.000448494
ENSGALG00000010034		5	0.000311485	0.000449353
ENSGALG00000041989	RRN3	14	0.000312293	0.000450466
ENSGALG00000044649	P2RX2	15	0.000316913	0.000457076
ENSGALG00000050127		1	0.000319153	0.000460253
ENSGALG00000044069		33	0.000319687	0.000460968
ENSGALG00000049903		1	0.000319851	0.00046115
ENSGALG00000007907	CCDC157	15	0.000321511	0.000463488
ENSGALG00000015917	EEF1A1	3	0.00032201	0.000464153
ENSGALG00000017217	VSTM5	1	0.000322209	0.000464385
ENSGALG00000007109	APOA4	24	0.000322637	0.000464947
ENSGALG00000006731	TAF3	1	0.000323033	0.000465462
ENSGALG00000029172	NDUFC2	1	0.000323295	0.000465785
ENSGALG00000003986	PITHD1	23	0.000323687	0.000466294
ENSGALG00000028141	C15orf61	10	0.000326889	0.000470852
ENSGALG00000038865	PRMT7	11	0.000328064	0.000472488
ENSGALG00000023689	ASS1	17	0.000330766	0.000476323
ENSGALG00000033694	IFI30	28	0.000332044	0.000478108
ENSGALG00000049174		16	0.000332377	0.00047853
ENSGALG00000013639	SYF2	23	0.000334104	0.000480959
ENSGALG00000011961	TNIP3	4	0.000336406	0.000484217
ENSGALG00000008920	NDOR1	17	0.000336778	0.000484694
ENSGALG00000013971	SGK1	3	0.000340106	0.000489427
ENSGALG00000006019	EPHX4	8	0.000341382	0.000491205
ENSGALG00000044733		2	0.000341848	0.000491818
ENSGALG00000000243	RDM1	27	0.000342944	0.000493335
ENSGALG00000034458	S100A1	25	0.000343222	0.000493677
ENSGALG00000023542	ALPL	21	0.000343647	0.000494231

ENSGALG00000040730	RXRG	8	0.000345382	0.000496667
ENSGALG00000015902	CD8BP	4	0.000346928	0.000498832
ENSGALG00000010636	PRIMPOL	4	0.000349956	0.000503126
ENSGALG00000000734	RPA2	23	0.000350043	0.000503192
ENSGALG00000007661	MYCBPAP	18	0.000350195	0.000503351
ENSGALG00000040316	GPC1	9	0.000352836	0.000507088
ENSGALG00000036621	ZCCHC4	4	0.000357021	0.000513041
ENSGALG00000030847	FZD2	27	0.000357715	0.000513979
ENSGALG00000038145	DPP7	17	0.000361662	0.000519588
ENSGALG00000036749	MRPS28	2	0.000363287	0.000521861
ENSGALG00000055014		1	0.000363845	0.000522601
ENSGALG00000002024	COMT	15	0.000365419	0.000524801
ENSGALG00000009700	PDK4	2	0.000365479	0.000524824
ENSGALG00000052388	METRN	14	0.000367507	0.000527675
ENSGALG00000046217	PIT54	31	0.000367952	0.000528251
ENSGALG00000010225		5	0.000369733	0.000530746
ENSGALG00000047981		32	0.000371316	0.000532955
ENSGALG00000011259	RPE65	8	0.000379142	0.000544123
ENSGALG00000037437	EGR3	22	0.000379733	0.000544908
ENSGALG00000027700	FLAD1	25	0.000379955	0.000545162
ENSGALG00000035994		2	0.000382679	0.000549006
ENSGALG00000041080	CHD7	2	0.000385012	0.000552289
ENSGALG00000027786	SOCS3	18	0.000386174	0.000553889
ENSGALG00000040182		28	0.000386523	0.000554325
ENSGALG00000010921	PKD2	4	0.000386837	0.000554711
ENSGALG00000001571	MYO1F	28	0.000387255	0.000555245
ENSGALG00000005608	SLC9A3R2	14	0.00038824	0.000556591
ENSGALG00000013617	PDGFRL	4	0.000391882	0.000561747
ENSGALG00000010083	ST3GAL3	8	0.000393929	0.000564615
ENSGALG00000012464	SOUL	5	0.000395058	0.000566167
ENSGALG00000026401	FAM104A	18	0.00039631	0.000567894
ENSGALG00000002144	THRAP3	23	0.000396558	0.000568183
ENSGALG00000002742	TMEM132B	15	0.000396841	0.00056852

ENSGALG00000043327		21	0.000397579	0.000569511
ENSGALG00000013112	RERGL	1	0.000397898	0.000569901
ENSGALG00000009654		9	0.000399288	0.000571825
ENSGALG00000010087	IPO13	8	0.000399581	0.000572177
ENSGALG00000007191	TK1	18	0.000402945	0.000576927
ENSGALG00000035063		3	0.000405795	0.00058094
ENSGALG00000005188	MINDY4	2	0.000407743	0.00058366
ENSGALG00000053876		12	0.000408395	0.000584524
ENSGALG00000036089	NICN1	12	0.000408918	0.000585204
ENSGALG00000003533	ZNF511	6	0.000409202	0.000585542
ENSGALG00000040866	P3H2	9	0.00041061	0.000587488
ENSGALG00000014931	RAB28	4	0.000413756	0.000591919
ENSGALG00000055023		33	0.000415759	0.000594716
ENSGALG00000001490	KCNJ13	9	0.000416024	0.000595025
ENSGALG00000033203	TRIM54	3	0.000417846	0.000597561
ENSGALG00000008901	PTGFR	8	0.000417993	0.0005977
ENSGALG00000037955	INTS8	2	0.000418048	0.000597709
ENSGALG00000016471		3	0.000420303	0.000600863
ENSGALG00000010985	ATG4C	8	0.000420674	0.000601322
ENSGALG00000002198	FHOD1	11	0.000420917	0.000601598
ENSGALG00000043021	HORMAD1	25	0.000423781	0.000605622
ENSGALG00000049299	TMEM170B	2	0.000427471	0.000610823
ENSGALG00000053250	ESYT1	33	0.000427984	0.000611484
ENSGALG00000001037	SCAI	17	0.000429131	0.000613051
ENSGALG00000010283	GTF2E2	4	0.000433247	0.000618858
ENSGALG00000053585		14	0.000434208	0.000620159
ENSGALG00000032247	ATP1A1	1	0.000444965	0.000635449
ENSGALG00000038325	CORO7	14	0.000446238	0.000637191
ENSGALG00000002515	OLFM1	17	0.000446379	0.000637318
ENSGALG00000033730		5	0.000447915	0.000639401
ENSGALG00000047199		6	0.000447943	0.000639401
ENSGALG00000027960	GRPR	1	0.000449665	0.000641785
ENSGALG00000031527	ARL8BL	1	0.000449849	0.000641973

ENSGALG00000013776	CEP135	4	0.000453175	0.000646643
ENSGALG00000009703	NDUFS7	28	0.000453553	0.000647106
ENSGALG00000002899	AACS	15	0.000456978	0.000651917
ENSGALG00000026384	PCSK4	28	0.000457541	0.000652643
ENSGALG00000011677	HSPBAP1	7	0.000458419	0.00065382
ENSGALG00000036739	UBE2L3	15	0.000460445	0.000656632
ENSGALG00000039118	MEIS2	5	0.000462135	0.000658965
ENSGALG00000003990		6	0.000462272	0.000659084
ENSGALG00000010293	RBP	8	0.000462645	0.000659538
ENSGALG00000036125	SHE	25	0.000464268	0.000661775
ENSGALG00000048562		31	0.000465363	0.000663258
ENSGALG00000030457	CHCHD5	30	0.000466331	0.00066456
ENSGALG00000042491		1	0.000467807	0.000666586
ENSGALG00000011537	PDE10A	3	0.000468291	0.000667197
ENSGALG00000039336	OTX5	32	0.0004702	0.000669839
ENSGALG00000004049	ITFG1	11	0.000470329	0.000669945
ENSGALG00000021627	IFI27L2	23	0.00047254	0.000673015
ENSGALG00000005209	AQP1	2	0.000473224	0.000673911
ENSGALG00000023749	DARS2	8	0.000476136	0.000677978
ENSGALG00000002638		6	0.000477204	0.000679419
ENSGALG00000027645		7	0.00047773	0.000680089
ENSGALG00000037962	C1H21ORF59	1	0.000479479	0.000682499
ENSGALG00000048815		1	0.000480461	0.000683818
ENSGALG00000027716		5	0.000481397	0.000685069
ENSGALG00000013741	SYTL3	3	0.000484557	0.000689486
ENSGALG00000034756		23	0.000485037	0.000690089
ENSGALG00000037929	BIN2	33	0.000485329	0.000690423
ENSGALG00000050390	CYB561D2	12	0.000489736	0.00069661
ENSGALG00000026313	RND3	7	0.00049348	0.000701855
ENSGALG00000010291	RBPMS	4	0.000493595	0.000701936
ENSGALG00000038933	FABP7	3	0.000494176	0.000702681
ENSGALG00000031635	VPS41	2	0.000494807	0.000703497
ENSGALG00000013745	RTTN	2	0.000495332	0.000704161

ENSGALG00000007929	DIXDC1	24	0.000495549	0.000704386
ENSGALG00000036837	PTGES3	33	0.000496646	0.000705864
ENSGALG00000011902	TAGLN	24	0.000497495	0.000706987
ENSGALG00000033245	SMPD2	26	0.000497893	0.000707471
ENSGALG00000001165	PANK4	21	0.000499757	0.000710037
ENSGALG00000032002	RPL34	4	0.000505683	0.000718372
ENSGALG00000046169	PYM1	33	0.000506664	0.000719682
ENSGALG00000046461	LRRC15	9	0.000508624	0.000722383
ENSGALG00000020716	ARMH1	8	0.000509485	0.000723521
ENSGALG00000041909		33	0.000512716	0.000728025
ENSGALG00000007485	TFRC	9	0.000515578	0.000732002
ENSGALG00000010088	RALGAPA1	5	0.000522861	0.000742257
ENSGALG00000039727		6	0.000523498	0.000743075
ENSGALG00000013892	MAP3K5	3	0.000523697	0.00074327
ENSGALG00000008881	RGS3	17	0.000523775	0.000743295
ENSGALG00000009355		9	0.000524237	0.000743865
ENSGALG00000037998	GLTP	15	0.000524552	0.000744224
ENSGALG00000028803	B3GNT4	15	0.00052605	0.000746262
ENSGALG00000054344		9	0.00052846	0.000749594
ENSGALG00000011176	SCRN1	2	0.000530857	0.000752907
ENSGALG00000040180	CEBPD	2	0.000540867	0.000767015
ENSGALG00000028985		2	0.000544394	0.000771927
ENSGALG00000034395	NUDT7	11	0.000544685	0.00077225
ENSGALG00000008926	CADPS2	1	0.000545904	0.000773887
ENSGALG00000006670	PPP1R3C	6	0.000546149	0.000774145
ENSGALG00000011298	RARB	2	0.000546358	0.000774351
ENSGALG00000006044	MAB21L4	9	0.000546671	0.000774705
ENSGALG00000012374	FRMD6	5	0.000549912	0.000779207
ENSGALG00000019087	RFXAP	1	0.000551326	0.00078112
ENSGALG00000033303		12	0.000556603	0.000788505
ENSGALG00000008288	ARMC10	1	0.000557475	0.000789649
ENSGALG00000046939		30	0.000559001	0.000791718
ENSGALG00000005550	ST6GAL1	9	0.000561773	0.000795551

ENSGALG00000012338	BDH2	4	0.000562703	0.000796776
ENSGALG00000004767	ODF2	17	0.000564949	0.000799864
ENSGALG00000002043		9	0.000568438	0.00080471
ENSGALG00000040832	CFD	28	0.000569818	0.00080657
ENSGALG00000021225	CDCA9	11	0.000570229	0.000807058
ENSGALG00000028643	ANAPC10	4	0.000572248	0.000809821
ENSGALG00000042227	GNLY	22	0.000574591	0.000813042
ENSGALG00000014297	IRF7	5	0.000575086	0.000813649
ENSGALG00000014217	GUF1	4	0.000575989	0.000814831
ENSGALG00000012068		4	0.000579856	0.000820206
ENSGALG00000014106	PPFIBP1	1	0.000581645	0.000822642
ENSGALG00000008591	CACNB2	2	0.000581994	0.00082304
ENSGALG00000007016		10	0.000582688	0.000823926
ENSGALG00000006703	GTPBP4	2	0.000583908	0.000825555
ENSGALG00000028517	EIF1	27	0.000585518	0.000827736
ENSGALG00000003549	MTG1	6	0.000588277	0.00083154
ENSGALG00000006027	TM6SF1	10	0.000588655	0.000831977
ENSGALG00000034751	TRPA1	2	0.000589307	0.000832802
ENSGALG00000051375		23	0.000591953	0.000836445
ENSGALG00000000761	TSKU	1	0.000592222	0.000836728
ENSGALG00000004729	SLC7A10	11	0.00059296	0.000837673
ENSGALG00000017015	RNASEH2B	1	0.000594754	0.000840111
ENSGALG00000007701	B3GNT7	9	0.000596612	0.000842637
ENSGALG00000001373	TRAF3IP3	26	0.000597633	0.000843981
ENSGALG00000000528	DBNDD1	11	0.000599081	0.000845928
ENSGALG00000033183	GABRQ	4	0.000600207	0.00084742
ENSGALG00000004881		17	0.000605359	0.000854596
ENSGALG00000048501		17	0.00061202	0.0008639
ENSGALG00000008256	BLM	10	0.000613158	0.000865405
ENSGALG00000005567	MSI2	19	0.000615923	0.000869206
ENSGALG00000008480	ABCF3	9	0.000616969	0.000870582
ENSGALG00000039589		2	0.000617603	0.000871377
ENSGALG00000001289		28	0.000619407	0.00087382

ENSGALG00000026499	TNFRSF25	21	0.000622478	0.000878051
ENSGALG00000008889	TTC14	9	0.000625323	0.000881962
ENSGALG00000005309	TKT	12	0.000625665	0.000882342
ENSGALG00000013406	EOGT	12	0.000625831	0.000882474
ENSGALG00000006479	CCM2L	20	0.000629332	0.000887309
ENSGALG00000003031	SMYD4	19	0.00063017	0.000888388
ENSGALG00000006543	PFKL	9	0.000630937	0.000889366
ENSGALG00000006427	CARS	5	0.00063344	0.000892791
ENSGALG00000045228		3	0.000634326	0.000893937
ENSGALG00000009705	RYR3	5	0.000638869	0.000900235
ENSGALG00000015379	TAGLN3	1	0.000639239	0.000900652
ENSGALG00000049392		33	0.000640657	0.000902546
ENSGALG00000006963	ENDOV	18	0.000648062	0.000912872
ENSGALG00000002496	SFTPA1	6	0.000648227	0.000912999
ENSGALG00000051765		2	0.000648535	0.000913327
ENSGALG00000004899	PEPD	11	0.000648837	0.000913646
ENSGALG00000039102	TOX	2	0.000654549	0.000921584
ENSGALG00000001965	AARS	11	0.000656386	0.000924064
ENSGALG00000010971	CSRNP3	7	0.00066157	0.000931255
ENSGALG00000003665	PIGS	19	0.000662083	0.000931868
ENSGALG00000008263	CNTN4	12	0.000670952	0.000944243
ENSGALG00000006405	CEP19	9	0.000673448	0.000947646
ENSGALG00000002919	MON1A	12	0.000675716	0.000950727
ENSGALG00000000618	ZCCHC17	23	0.000677639	0.000953324
ENSGALG00000044383	MCL1	25	0.000680187	0.000956797
ENSGALG00000043460	SOX2	9	0.000682521	0.00095997
ENSGALG00000032819	RNF41	33	0.000690717	0.000971385
ENSGALG00000015468		3	0.000692197	0.000973354
ENSGALG00000002487	SFSWAP	15	0.000695874	0.000978412
ENSGALG00000016312	POLA1	1	0.000696264	0.000978848
ENSGALG00000030255	AMBP	17	0.000697986	0.000981155
ENSGALG00000053517	CDC26	17	0.000705786	0.000992006
ENSGALG00000038969		1	0.000708381	0.000995539

ENSGALG00000014155	OGDH	22	0.000711293	0.000999515
ENSGALG00000035111	PDRG1	20	0.000713189	0.001002064
ENSGALG00000011932	XRCC6	1	0.000716458	0.001006542
ENSGALG00000047800		27	0.00071695	0.001007117
ENSGALG00000004309	GDAP1L1	20	0.000717337	0.001007544
ENSGALG00000009413	BROX	3	0.000724812	0.001017925
ENSGALG00000004129	SPP2	7	0.000725423	0.001018666
ENSGALG00000006043	RBBP6	14	0.000726492	0.001019995
ENSGALG00000038109	MRPL45	27	0.000726536	0.001019995
ENSGALG00000038787	ADRA1D	4	0.000727328	0.001020988
ENSGALG00000005540	MICAL2	5	0.000731215	0.001026327
ENSGALG00000011976	L3MBTL2	1	0.000735336	0.001031993
ENSGALG00000006777	MRPS11	10	0.000738917	0.001036871
ENSGALG00000010820	CHGA	5	0.000738982	0.001036871
ENSGALG00000009738	CTBP2	6	0.000747101	0.001048142
ENSGALG00000000625	MAPT	27	0.000748257	0.001049628
ENSGALG00000032518	NCALD	2	0.000748332	0.001049628
ENSGALG00000011327	NR2C1	1	0.000748928	0.001050343
ENSGALG00000009076	NDUFB5	9	0.000749627	0.001051202
ENSGALG00000041094	SYT1	1	0.000753229	0.001056131
ENSGALG00000006197	CSNK2A1	20	0.000756192	0.001060165
ENSGALG00000054891	RTKN	4	0.000763606	0.001070436
ENSGALG00000043690	ECRG4	1	0.000769221	0.001078088
ENSGALG00000048592		32	0.000769242	0.001078088
ENSGALG00000039347	UNC93B1	5	0.000770254	0.001079382
ENSGALG00000030220	ELMO3	11	0.000770802	0.001080026
ENSGALG00000044632		28	0.000771154	0.001080395
ENSGALG00000015253	COL8A1	1	0.000773122	0.001083028
ENSGALG00000054779	FAM206A	2	0.000773338	0.001083206
ENSGALG00000009961	ABCE1	4	0.000777028	0.00108825
ENSGALG00000006460	TTLL9	20	0.000777345	0.001088569
ENSGALG00000004420	SDK1	14	0.000778252	0.001089713
ENSGALG00000012695	NOL7	2	0.000783426	0.001096832

ENSGALG00000003154	TXNDC11	14	0.000783622	0.001096981
ENSGALG00000050608		30	0.000784099	0.001097523
ENSGALG00000036915	SQLE	2	0.000784369	0.001097774
ENSGALG00000020417	ISCA2	5	0.000784658	0.001098052
ENSGALG00000003021	CHRNA5	10	0.000786983	0.001101181
ENSGALG00000030739	MSL1	27	0.000788551	0.001103247
ENSGALG00000009993	MED8	8	0.000795336	0.001112612
ENSGALG00000013272	PRMT8	1	0.000795843	0.001113194
ENSGALG00000007964	CASTOR1	15	0.000797857	0.001115883
ENSGALG00000041101	GALNT6	33	0.00079961	0.001118207
ENSGALG00000030378	RPL11	23	0.000805717	0.001126618
ENSGALG00000048124		1	0.000805913	0.001126762
ENSGALG00000000048		25	0.000806523	0.001127486
ENSGALG00000017218	MED17	1	0.000807075	0.001128128
ENSGALG00000023172		4	0.000809119	0.001130856
ENSGALG00000003985	TARSL2	10	0.000816942	0.001141659
ENSGALG00000016345	APOO	1	0.000820664	0.00114673
ENSGALG00000025977	GADD45A	8	0.000827467	0.001156103
ENSGALG00000002549	RGS1	8	0.000831903	0.001162167
ENSGALG00000049587		2	0.000832116	0.001162331
ENSGALG00000032618	DUSP5	6	0.00083495	0.001166156
ENSGALG00000026559	SNRNP25	14	0.000835865	0.001167301
ENSGALG00000044479		3	0.000836067	0.001167448
ENSGALG00000044418		5	0.000839781	0.001172501
ENSGALG00000045747	REEP6	28	0.000840267	0.001173045
ENSGALG00000006508	FGF13	4	0.00084079	0.001173641
ENSGALG00000048508		30	0.00084215	0.001175405
ENSGALG00000011113	SGIP1	8	0.000843746	0.001177498
ENSGALG00000002856	RUNDC1	27	0.000846604	0.00118135
ENSGALG00000051041	NFE2	33	0.000847833	0.00118293
ENSGALG00000038411	CENPL	8	0.000850008	0.00118583
ENSGALG00000000573	PER3	21	0.000852249	0.001188819
ENSGALG00000036042		33	0.000854207	0.001191415

ENSGALG00000040522	EIF3H	2	0.000855839	0.001193554
ENSGALG00000004257	TSPAN15	6	0.000856365	0.001194152
ENSGALG00000027050	SENP8	10	0.000859037	0.00119774
ENSGALG00000028602	HMP19	13	0.000859466	0.001198201
ENSGALG00000019489	CHST9	2	0.000860052	0.001198881
ENSGALG00000044866		22	0.000860711	0.001199662
ENSGALG00000026225	PSMD12	18	0.00086412	0.001204277
ENSGALG00000017319	MRPL48	1	0.000870579	0.001213139
ENSGALG00000003734	SZRD1	21	0.000873384	0.001216909
ENSGALG00000027641	DUSP14	19	0.000873534	0.001216979
ENSGALG00000009748	ASNS	2	0.000873652	0.001217004
ENSGALG00000030040	CDH15	11	0.000878836	0.001224086
ENSGALG00000009325	OLA1	7	0.000879016	0.001224196
ENSGALG00000012112	DBI	7	0.000890163	0.00123958
ENSGALG00000016974	GTF2F2	1	0.000893322	0.001243836
ENSGALG00000042030	P4HA3	1	0.000898254	0.00125056
ENSGALG00000006681	BRSK1	5	0.000898504	0.001250766
ENSGALG00000004081	TMCO4	21	0.000901258	0.001254457
ENSGALG00000052202		32	0.000907932	0.001263602
ENSGALG00000000681	PAK1	1	0.000911342	0.001268203
ENSGALG00000030597	KIF11	6	0.000914464	0.001272402
ENSGALG00000006960	RNF5	15	0.000915467	0.001273653
ENSGALG00000020688	CYP4A22	8	0.000917146	0.001275842
ENSGALG00000011681	DEPDC7	5	0.000919097	0.001278411
ENSGALG00000051213	DUSP21	15	0.00092024	0.001279855
ENSGALG00000010870	JUN	8	0.000921209	0.001281056
ENSGALG00000002689	NUP35	7	0.000922661	0.001282929
ENSGALG00000026518	RUNDC3A	27	0.000925891	0.001287273
ENSGALG00000043256		4	0.000931631	0.001295106
ENSGALG00000037867	MRPL53	2	0.000934995	0.001299635
ENSGALG00000001597	PSMB3	27	0.000937569	0.001303002
ENSGALG00000026663	CX3CL1	11	0.000937631	0.001303002
ENSGALG00000006343	ACTA2	6	0.000938403	0.001303925

ENSGALG00000034986	HEYL	23	0.00093896	0.001304551
ENSGALG00000027035	RPL21	1	0.000939514	0.001305171
ENSGALG00000029940	IL1B	22	0.000943552	0.001310632
ENSGALG00000053574		14	0.00094588	0.001313717
ENSGALG00000001492	NDRG3	20	0.000953949	0.001324773
ENSGALG00000025778	SIRPA	20	0.000954834	0.001325851
ENSGALG00000000699	WWP2	11	0.000956656	0.001328228
ENSGALG00000034898	ITPK1	5	0.000959181	0.001331583
ENSGALG00000006488	RYK	9	0.000961566	0.001334742
ENSGALG00000016661	BLK	3	0.000963628	0.001337453
ENSGALG00000038129	PTGES3L	27	0.000965129	0.001339383
ENSGALG00000012468	PRPF39	5	0.000967822	0.001342967
ENSGALG00000053631	KIF12	19	0.000968914	0.00134433
ENSGALG00000026038	ERI2	14	0.000972758	0.001349509
ENSGALG00000038374	SNX20	11	0.000977149	0.001355383
ENSGALG00000000751	ARNT	25	0.000977214	0.001355383
ENSGALG00000006838		15	0.000978728	0.001357328
ENSGALG00000001555	AURKAIP1	21	0.000979811	0.001358675
ENSGALG00000009361	GUCY1B1	4	0.000982104	0.001361701
ENSGALG00000041795	WWP1	2	0.000988722	0.001370721
ENSGALG00000032549	TYW3	8	0.000990218	0.001372639
ENSGALG00000007356	MAP3K8	2	0.000993068	0.001376433
ENSGALG00000048441		25	0.000993332	0.001376642
ENSGALG00000036214	PARK7	21	0.00099544	0.001379407
ENSGALG00000015496	CHMP2B	1	0.000997351	0.001381898
ENSGALG00000054469	DISP1	3	0.001004323	0.0013914
ENSGALG00000004870	DNAAF6	4	0.001006593	0.001394387
ENSGALG00000021314	PLA2G10	14	0.001015669	0.001406799
ENSGALG00000044536	WDR13	30	0.001019148	0.001411458
ENSGALG00000026517	RFC5	15	0.001020534	0.001413217
ENSGALG00000016811	SEPTIN10	1	0.001021254	0.001414054
ENSGALG00000041338		12	0.001024796	0.001418797
ENSGALG00000041802	LYRM9	19	0.001029348	0.001424936

ENSGALG00000009001	CWC22	7	0.001036374	0.001434499
ENSGALG00000035818		33	0.00103747	0.001435853
ENSGALG00000009250	HNRNPA3	7	0.001044606	0.001445566
ENSGALG00000011633	SLC5A8	1	0.001045849	0.001447121
ENSGALG00000006601	MTMR14	12	0.001051463	0.001454725
ENSGALG00000008289	RBM19	15	0.001055108	0.001459602
ENSGALG00000001168	POLR2C	11	0.001055517	0.001460002
ENSGALG00000002194	MRPL38	18	0.001058773	0.001464339
ENSGALG00000003882	SMARCE1	27	0.001060543	0.001466621
ENSGALG00000029244	SMAP2	23	0.001070255	0.001479884
ENSGALG00000013200	GABBR2	2	0.001070777	0.001480438
ENSGALG00000003400	AIMP2	14	0.001071162	0.001480802
ENSGALG00000007533	NPEPL1	20	0.001072638	0.001482675
ENSGALG00000007070		14	0.001084527	0.001498938
ENSGALG00000053980		25	0.0010875	0.001502878
ENSGALG00000001841	ROGDI	14	0.001089266	0.001505148
ENSGALG00000042819		19	0.001091855	0.001508554
ENSGALG00000034399	TNS2	33	0.001097861	0.001516681
ENSGALG00000004631	DRAXIN	21	0.001102232	0.001522547
ENSGALG00000016224	MAOA	1	0.001105364	0.0015267
ENSGALG00000054103		13	0.00110569	0.001526977
ENSGALG00000038529	PLPPR3	28	0.001113284	0.00153729
ENSGALG00000048625		31	0.001120578	0.001547188
ENSGALG00000046691		32	0.001127306	0.001556196
ENSGALG00000051297		1	0.001127358	0.001556196
ENSGALG00000054577		28	0.001128093	0.001556912
ENSGALG00000045762		1	0.001128133	0.001556912
ENSGALG00000000577	RANBP3	28	0.001129309	0.001558359
ENSGALG00000027462	P3H4	27	0.00113559	0.00156685
ENSGALG00000007954		15	0.001136034	0.001567284
ENSGALG00000011242	OBSL1	7	0.001137589	0.001569252
ENSGALG00000020160	ADH6	4	0.001137821	0.001569395
ENSGALG00000002802	PACSIN1	26	0.001138325	0.001569912

ENSGALG00000011041	DNAJC6	8	0.001140779	0.00157312
ENSGALG00000001894		21	0.001142006	0.001574633
ENSGALG00000054306	SRXN1	20	0.001146163	0.001580186
ENSGALG00000014736		1	0.001147341	0.001581631
ENSGALG00000043072	POU6F1	33	0.001165081	0.001605905
ENSGALG00000003385	LSM4	28	0.00117144	0.001614487
ENSGALG00000008534	POLR2H	9	0.001179664	0.001625638
ENSGALG00000053680	HIST1H46L2	1	0.001181246	0.001627634
ENSGALG00000005976	TCEA2	20	0.001190089	0.001639634
ENSGALG00000020726		6	0.001195797	0.001647311
ENSGALG00000048067	DUS2	11	0.001197005	0.001648789
ENSGALG00000036730	MRPS35	1	0.00120024	0.001653059
ENSGALG00000036300	SEC61A2	1	0.001206292	0.001661205
ENSGALG00000016946	SUGT1	1	0.001213786	0.001671337
ENSGALG00000045898		7	0.001216561	0.001674969
ENSGALG00000005302	SASS6	8	0.001219738	0.001679154
ENSGALG00000024295		23	0.00122731	0.001689387
ENSGALG00000001773	RFX2	28	0.00122898	0.001691495
ENSGALG00000001136		21	0.001230911	0.001693961
ENSGALG00000016202	HSF2BP	1	0.001231828	0.001695032
ENSGALG00000012490		1	0.001235842	0.001700364
ENSGALG00000008815	LRRN4	3	0.001236158	0.001700585
ENSGALG00000048624	SGPP2	9	0.001236282	0.001700585
ENSGALG00000004628	CTNS	19	0.001238656	0.001703658
ENSGALG00000045478		5	0.00124322	0.001709743
ENSGALG00000033185	TAFAZZIN	30	0.001248678	0.001717056
ENSGALG00000034572		12	0.001249455	0.001717888
ENSGALG00000003348		13	0.001249565	0.001717888
ENSGALG00000049660		31	0.001251721	0.001720657
ENSGALG00000016899	DCT	1	0.001252523	0.001721567
ENSGALG00000007158	SOCS1	14	0.001260882	0.00173286
ENSGALG00000012244	KLHDC2	5	0.001273248	0.001749657
ENSGALG00000043035	SHISA2	1	0.001273587	0.001749926

ENSGALG00000007412	MPZL2	24	0.0012843	0.001764448
ENSGALG00000015937	FABP1	4	0.001287874	0.001769159
ENSGALG00000037851	KK34	13	0.001295926	0.001780019
ENSGALG00000023904	FIBIN	5	0.001297754	0.001782329
ENSGALG00000003904	MLPH	7	0.001298974	0.001783803
ENSGALG00000050805		30	0.001299448	0.001784253
ENSGALG00000001056	ABHD11	19	0.001305996	0.001793043
ENSGALG00000010743	LRRC42	8	0.001306403	0.001793399
ENSGALG00000051280	DNAJB1	30	0.001308209	0.001795676
ENSGALG00000001394	UBL7	10	0.00130908	0.00179667
ENSGALG00000002127	LSM10	23	0.001314635	0.001804091
ENSGALG00000030890	RRS1	2	0.001318099	0.00180864
ENSGALG00000001362	CLK3	10	0.001327666	0.001821564
ENSGALG00000030022	FANCL	3	0.001344303	0.001844182
ENSGALG00000008077		10	0.001347644	0.001848376
ENSGALG00000011287	SULT	3	0.001347664	0.001848376
ENSGALG00000026311	C12orf75	1	0.001350763	0.001852419
ENSGALG00000026332	EEF1AKMT4	9	0.00135235	0.001854386
ENSGALG00000003651	NT5C3B	27	0.001356229	0.001859497
ENSGALG00000031120	HNRNPH2	13	0.001356938	0.001860259
ENSGALG00000028908	HOXA6	2	0.001373282	0.001882454
ENSGALG00000008124	BORCS7	6	0.001375262	0.001884956
ENSGALG00000000409	CHMP7	22	0.00137816	0.001888716
ENSGALG00000025999	NDUFA4	2	0.001386947	0.001900544
ENSGALG00000006728	SLC5A1	15	0.001388409	0.001902334
ENSGALG00000031312	ANAPC13	9	0.001392383	0.00190735
ENSGALG00000032145	B4GALT7	13	0.001392383	0.00190735
ENSGALG00000023436	HDC	10	0.00139583	0.001911857
ENSGALG00000033328	PACS2	8	0.001396919	0.001913135
ENSGALG00000003941	FUBP3	17	0.001398887	0.001915614
ENSGALG00000047323		4	0.001405743	0.001924787
ENSGALG00000034474	CRISP3	3	0.001408304	0.001928077
ENSGALG00000005994	DERL3	15	0.00140881	0.001928553

ENSGALG00000037167	HIST1H103	1	0.001412865	0.001933887
ENSGALG00000005401	WNT9A	2	0.001416254	0.001938307
ENSGALG00000009621	ACTB	14	0.001418891	0.001941699
ENSGALG00000032988	PCMT1L	5	0.001421214	0.00194466
ENSGALG00000035453		2	0.001425552	0.001950377
ENSGALG00000031958	NABP2	33	0.00142595	0.001950703
ENSGALG00000012137	KTN1	5	0.001431662	0.001958296
ENSGALG00000042748		22	0.001434984	0.00196262
ENSGALG00000027805		10	0.001444228	0.001975042
ENSGALG00000041121	SLIT2	4	0.001444921	0.001975768
ENSGALG00000004038	ADAL	10	0.001447156	0.001978602
ENSGALG00000007835	PUS10	3	0.001448797	0.001980624
ENSGALG00000047704		33	0.001455163	0.001989103
ENSGALG00000016826		1	0.001461093	0.00199683
ENSGALG00000029135	YEATS4	1	0.001461143	0.00199683
ENSGALG00000012292	BANK1	4	0.001463742	0.002000157
ENSGALG00000026661	MSRB3	1	0.001466419	0.002003591
ENSGALG00000045037	CYP2W1	14	0.001473513	0.002013058
ENSGALG00000010494	SLC5A9	8	0.001474715	0.002014475
ENSGALG00000036736	KXD1	28	0.00147678	0.002017069
ENSGALG00000034048	BCL11A	3	0.00148096	0.002022553
ENSGALG00000002490	UQCRC2	14	0.001485061	0.002027926
ENSGALG00000027909		23	0.001494047	0.002039969
ENSGALG00000006511	BCL2A1	10	0.001494654	0.002040569
ENSGALG00000047954		22	0.001496568	0.002042953
ENSGALG00000006939	PFKP	2	0.001497449	0.002043927
ENSGALG00000004319	NUDT13	6	0.001498269	0.002044817
ENSGALG00000015252	ST3GAL6	1	0.001513794	0.002065773
ENSGALG00000008369	ABCC5	9	0.001521568	0.00207615
ENSGALG00000052957		14	0.001528389	0.002085207
ENSGALG00000012160	KIF18A	5	0.001528547	0.002085207
ENSGALG00000047024		11	0.001528973	0.002085554
ENSGALG00000009538		5	0.001532946	0.002090739

ENSGALG00000027947	GHSR	9	0.001534062	0.002092027
ENSGALG00000030585	UBXN2B	2	0.001534556	0.002092468
ENSGALG00000006886	DACH2	4	0.001543302	0.002104158
ENSGALG00000010129	PLK3	8	0.001546172	0.002107835
ENSGALG00000038740	AMY2A	8	0.001546952	0.002108662
ENSGALG00000043126		11	0.001554594	0.002118734
ENSGALG00000012379	C5H14orf166	5	0.001554688	0.002118734
ENSGALG00000010096	MBIP	5	0.001558688	0.002123948
ENSGALG00000017119	TNFRSF19	1	0.001559773	0.002125189
ENSGALG00000003644		10	0.001571539	0.002140981
ENSGALG00000003258	DNAAF1	11	0.001574172	0.002144329
ENSGALG00000039322	DDR2	8	0.001581967	0.002154706
ENSGALG00000035153	STX3	5	0.001590747	0.002166423
ENSGALG00000001678	PRKAB2	8	0.001604518	0.002184933
ENSGALG00000027520		5	0.001612703	0.002195833
ENSGALG00000026151	CEP250	20	0.001613595	0.002196803
ENSGALG00000013173	PYROXD1	1	0.00162903	0.002217568
ENSGALG00000040168	GPR61	26	0.001630554	0.002219395
ENSGALG00000026207	CNTF	5	0.001634498	0.002224516
ENSGALG00000013383	PPIE	23	0.001638496	0.002229708
ENSGALG00000021658	PAFAH2	23	0.001643583	0.00223638
ENSGALG00000021972	PLIN4	28	0.001645561	0.002238822
ENSGALG00000030940	BLB2	16	0.001647848	0.002241684
ENSGALG00000041510	DUOX2	10	0.001648291	0.002242036
ENSGALG00000039182	TBL1XR1	9	0.00165756	0.002254392
ENSGALG00000037261	RFXANK	28	0.001666899	0.002266841
ENSGALG00000005535	TCOF1	13	0.001669505	0.002270071
ENSGALG00000008703	RFC4	9	0.001669646	0.002270071
ENSGALG00000038143		5	0.001673384	0.0022749
ENSGALG00000013001		2	0.001683765	0.002288757
ENSGALG00000054799	SPIC	1	0.001688813	0.002295362
ENSGALG00000012362	THSD7B	7	0.0017026	0.002313816
ENSGALG00000003197	RPL7A	17	0.00170277	0.002313816

ENSGALG00000008247	ARFGAP2	5	0.001719513	0.002336308
ENSGALG00000047285	BD7	3	0.001731924	0.002352907
ENSGALG00000030101	CSDC2	1	0.001738378	0.002361413
ENSGALG00000015001	LAMA4	3	0.001738789	0.002361709
ENSGALG00000048349	PRC1	10	0.001745533	0.002370605
ENSGALG00000008709	MCOLN3	8	0.001750091	0.00237653
ENSGALG00000011350	NEGR1	8	0.001759566	0.00238913
ENSGALG00000051748	NTMT2	8	0.001761383	0.002391331
ENSGALG00000002377	ENO1	21	0.001765307	0.002396391
ENSGALG00000052769		8	0.001768448	0.002400388
ENSGALG00000054865		12	0.001768863	0.002400685
ENSGALG00000047412		30	0.001769296	0.002401005
ENSGALG00000024488	CD99	1	0.001770747	0.002402706
ENSGALG00000041078	MID1IP1	1	0.001774422	0.002407424
ENSGALG00000029270	GATA3	1	0.001782996	0.002418788
ENSGALG00000044641	DAPL1	7	0.001787132	0.002424078
ENSGALG00000005418	FRRS1	8	0.001787293	0.002424078
ENSGALG00000002581		8	0.00178822	0.002424907
ENSGALG00000004034	CAPZB	21	0.001788301	0.002424907
ENSGALG00000016324	GSTA4	3	0.001790084	0.002427053
ENSGALG00000002594	TFPI	7	0.001793193	0.002430998
ENSGALG00000041700	GNAI3	26	0.001804725	0.00244636
ENSGALG00000016512	NRBP1	3	0.001805141	0.002446652
ENSGALG00000033090	VDR	33	0.001805431	0.002446773
ENSGALG00000040363	ABHD4	2	0.001809787	0.002452405
ENSGALG00000001949	MDH2	19	0.001810103	0.00245256
ENSGALG00000015346	NFKBIZ	1	0.001815204	0.002459198
ENSGALG00000004039	MINOS1	21	0.001816796	0.002461082
ENSGALG00000006486	RPP30	6	0.001819564	0.002464557
ENSGALG00000016124	HADH	4	0.001826363	0.002473492
ENSGALG00000050260		22	0.001827248	0.002474415
ENSGALG00000003529	SYNC	23	0.00183266	0.002481469
ENSGALG00000026836		23	0.001840798	0.002492211

ENSGALG00000010540	TTC39A	8	0.00184366	0.002495809
ENSGALG00000036244		2	0.001845505	0.002498029
ENSGALG00000010195		4	0.001845864	0.002498237
ENSGALG00000019215	ARL6	1	0.001847961	0.002500797
ENSGALG00000009004	ZNF804B	2	0.001853804	0.002508427
ENSGALG00000043606		33	0.001856068	0.002511211
ENSGALG00000034512		9	0.001856861	0.002512005
ENSGALG00000015751	ATP5PF	1	0.001857355	0.002512395
ENSGALG00000039533		2	0.001858907	0.002514215
ENSGALG00000009719	PCDH10	4	0.001863275	0.002519843
ENSGALG00000013848	MVK	15	0.001863503	0.002519872
ENSGALG00000051895		23	0.00186422	0.002520562
ENSGALG00000034083		1	0.001870325	0.002528537
ENSGALG00000024481	CENPS	21	0.001876518	0.002536628
ENSGALG00000028175	GJA9	23	0.001878587	0.002539143
ENSGALG00000029820	NMRAL1	14	0.00188197	0.002543434
ENSGALG00000029424	JPH3	11	0.001891972	0.002556668
ENSGALG00000011222	IL12RB2	8	0.0018932	0.002558044
ENSGALG00000052682		20	0.001917148	0.002590115
ENSGALG00000015196	ASXL3	2	0.001919924	0.002593578
ENSGALG00000003677	NTNG2	17	0.001923861	0.002598609
ENSGALG00000034967	GDPD5	1	0.001927758	0.002603584
ENSGALG00000016610	PTRHD1	3	0.001930841	0.002607459
ENSGALG00000011281	SGO1	2	0.00193464	0.0026123
ENSGALG00000006608		5	0.001936575	0.002614623
ENSGALG00000036993	RNF214	24	0.001941091	0.00262043
ENSGALG00000007183	ERGIC2	1	0.001953613	0.002637042
ENSGALG00000009978	DTD2	5	0.001972556	0.002662318
ENSGALG00000008168	ANKRD16	1	0.001999549	0.002698451
ENSGALG00000002283	MAPKAPK3	12	0.002007946	0.002709483
ENSGALG00000028214	OTOP3	18	0.002009133	0.002710785
ENSGALG00000004320	FAT2	13	0.002015496	0.002719069
ENSGALG00000045362		5	0.002028616	0.002736466

ENSGALG00000002048	GPRIN2	6	0.002034115	0.00274358
ENSGALG00000024272	S100A12	25	0.002048346	0.002762469
ENSGALG00000043947		1	0.0020525	0.002767765
ENSGALG00000010866	AREG	4	0.002064845	0.002783866
ENSGALG00000026031	ZNHIT3	19	0.002064897	0.002783866
ENSGALG00000031149		26	0.002074258	0.002796178
ENSGALG00000007289	CLPX	10	0.002074708	0.002796475
ENSGALG00000042011	HNRNPA1	33	0.002085663	0.002810931
ENSGALG00000041361	ARFIP1	4	0.002089021	0.002815145
ENSGALG00000028345	RHBDL1	14	0.002093557	0.002820946
ENSGALG00000011866	RRP7A	1	0.00212425	0.002861986
ENSGALG00000035031	HEY1	2	0.002130618	0.00287025
ENSGALG00000046649		23	0.002136623	0.00287802
ENSGALG00000042242	NDUFB9	2	0.002149032	0.002894415
ENSGALG00000026098	IL8	4	0.002149841	0.002895186
ENSGALG00000006317	ING5	9	0.002151423	0.002896996
ENSGALG00000045658	ABHD14A	12	0.002167694	0.002918584
ENSGALG00000000795	MFSD12	28	0.002185032	0.00294146
ENSGALG00000008234	ORC2	7	0.002185168	0.00294146
ENSGALG00000013207	CTNNAL1	2	0.002189231	0.002946605
ENSGALG00000013397	EXOC3	2	0.002225081	0.002994526
ENSGALG00000042773	VOPP1	2	0.002234514	0.00300689
ENSGALG00000004254	TMEM120B	15	0.002238504	0.003011926
ENSGALG00000028600	PPIA	22	0.002255037	0.003033838
ENSGALG00000035202	AP3B2	10	0.002260477	0.003040573
ENSGALG00000012017	DAAM1	5	0.002260543	0.003040573
ENSGALG00000010349	POLR1C	3	0.00226214	0.003042387
ENSGALG00000039869	PYURF	4	0.002262728	0.003042842
ENSGALG00000004134	GLRA1	13	0.002265002	0.003045563
ENSGALG00000008698	MGME1	3	0.002265335	0.003045675
ENSGALG00000033365	ALDH1A3	10	0.002268308	0.003049336
ENSGALG00000053281	PKIA	2	0.0022726	0.003054769
ENSGALG00000045165	C1orf109	23	0.002296418	0.003086444

ENSGALG00000044726	SNAPC4	17	0.002334778	0.003137655
ENSGALG00000047591		13	0.002337664	0.003141188
ENSGALG00000006531	TRA2B	9	0.002348262	0.003155081
ENSGALG00000020067	VASH2	3	0.002351259	0.00315876
ENSGALG00000043130		6	0.002354436	0.003162679
ENSGALG00000036683	PNPO	27	0.002369539	0.003182615
ENSGALG00000023760	CHIA	26	0.002385515	0.003203721
ENSGALG00000012304	NFKB1	4	0.002387706	0.003206311
ENSGALG00000011838	GUCY2C	1	0.002388573	0.003207121
ENSGALG00000054873	NPPC	9	0.0023984	0.003219962
ENSGALG00000015140		2	0.002412178	0.003238103
ENSGALG00000006188	KYAT3	8	0.002413856	0.003239998
ENSGALG00000020438	DPH6	5	0.002416408	0.003243067
ENSGALG00000041255	ADAM12	6	0.002426052	0.003255652
ENSGALG00000045499	TCFL5	20	0.002427835	0.003257686
ENSGALG00000004782	TSEN15	8	0.002428179	0.003257788
ENSGALG00000028095	HOXA1	2	0.002451981	0.003289361
ENSGALG00000051065	PYGO2	25	0.002462866	0.0033036
ENSGALG00000039206	ARID5A	22	0.002514012	0.003371834
ENSGALG00000037315	KLHDC4	11	0.002523627	0.003384358
ENSGALG00000004245	NUDT1	14	0.002533815	0.003397648
ENSGALG00000026849	SIAH3	1	0.002536955	0.003401484
ENSGALG00000007082	CREM	2	0.002549833	0.003418374
ENSGALG00000012987	CCDC77	1	0.002556385	0.003426781
ENSGALG00000005826	DLEC1	2	0.002558656	0.003429449
ENSGALG00000007649	TIPIN	10	0.002568585	0.003442378
ENSGALG00000004150	MYO1E	10	0.002572362	0.003447062
ENSGALG00000037082	ASCL1	1	0.002575534	0.003450933
ENSGALG00000031978	TARP	2	0.002578351	0.003454327
ENSGALG00000017035	COG6	1	0.002581026	0.003457532
ENSGALG00000004793		19	0.002583294	0.003460189
ENSGALG00000019873	RDH14	3	0.002623369	0.003513482
ENSGALG00000049105		5	0.00263414	0.00352752

ENSGALG00000008686	TRDMT1	2	0.002637755	0.003531973
ENSGALG00000000107	TRIM7.1	16	0.002641237	0.003536247
ENSGALG00000054999		23	0.002648701	0.003545851
ENSGALG00000014496	PROM1	4	0.002653803	0.003552291
ENSGALG00000053516		26	0.002654152	0.003552368
ENSGALG00000006521	TRPM5	5	0.002661368	0.003561635
ENSGALG00000049270	FKBP2	33	0.002675408	0.003580032
ENSGALG00000039839	HEXD	18	0.002687433	0.003595728
ENSGALG00000034124	NLRP3	5	0.002710416	0.00362608
ENSGALG00000007114	APOA1	24	0.002712704	0.003628744
ENSGALG00000050459	DUSP23	25	0.002719284	0.003637147
ENSGALG00000053114		1	0.002729303	0.003650147
ENSGALG00000033054	RMC1	2	0.002730526	0.003651383
ENSGALG00000050206		18	0.002739979	0.003663621
ENSGALG00000031679	POLE4	28	0.002751055	0.003677942
ENSGALG00000006805	PLSCR1	9	0.002751292	0.003677942
ENSGALG00000047425		30	0.002755241	0.003682817
ENSGALG00000023435	GATM	10	0.002756226	0.003683729
ENSGALG00000040447	CDCP1	2	0.002765767	0.003696076
ENSGALG00000011274	DCN	1	0.002777951	0.003711951
ENSGALG00000043009	WNT1	33	0.002783029	0.00371833
ENSGALG00000029367	CYBA	11	0.002788455	0.00372517
ENSGALG00000009104	ANKRD26	1	0.002810227	0.003753845
ENSGALG00000040909	WRN	4	0.002830798	0.003780909
ENSGALG00000034850	GLRX2	8	0.002832672	0.003782998
ENSGALG00000040896	FASN	18	0.002847309	0.003802128
ENSGALG00000034675	XKR9	2	0.002849993	0.003805296
ENSGALG00000009172	OSBPL6	7	0.002854821	0.003811044
ENSGALG00000008896	PPP1R1C	7	0.002854923	0.003811044
ENSGALG00000035386	NCKAP1L	33	0.00286352	0.003822101
ENSGALG00000036949	ASAP3	23	0.002899865	0.003870189
ENSGALG00000008945	NEXN	8	0.002900712	0.003870896
ENSGALG00000006758	C1QTNF5	24	0.002902905	0.003873399

ENSGALG00000010294	RPS6KL1	5	0.002904427	0.003875006
ENSGALG00000054523	LZTFL1	2	0.002910019	0.003882042
ENSGALG00000036962	KPNB1	27	0.002929433	0.003907229
ENSGALG00000030051	TOP3B	15	0.00292954	0.003907229
ENSGALG00000037802		25	0.002935584	0.003914861
ENSGALG00000046657		2	0.002942832	0.003924098
ENSGALG00000025898	SMAD6	10	0.002945032	0.003926602
ENSGALG00000005236	FARSB	9	0.002956913	0.003942012
ENSGALG00000044406		27	0.002973186	0.003963273
ENSGALG00000016595	TRIM35	3	0.002988341	0.003983039
ENSGALG00000003084	NUDT21	11	0.002990311	0.003985229
ENSGALG00000049949		4	0.002991611	0.003986525
ENSGALG00000006522	HCK	20	0.003007464	0.004007213
ENSGALG00000037995	STRIP1	26	0.003012191	0.004012891
ENSGALG00000007713	CD300LG	18	0.003012384	0.004012891
ENSGALG00000013737	TMEM181	3	0.003017076	0.004018704
ENSGALG00000053734		31	0.003026807	0.004031225
ENSGALG00000003987	TRAF4	19	0.003034542	0.004041085
ENSGALG00000007965	HN1	18	0.003043389	0.004052423
ENSGALG00000006016	PASK	9	0.003049132	0.004059627
ENSGALG00000049347		4	0.003052349	0.004063467
ENSGALG00000008262	RASGRF1	10	0.003059383	0.004072387
ENSGALG00000031735	CD5	5	0.003064096	0.004078215
ENSGALG00000009152	ATRNL1	6	0.003079175	0.004097836
ENSGALG00000003922	TOP2A	27	0.003111658	0.004140615
ENSGALG00000006153	EXOG	2	0.003127972	0.00416158
ENSGALG00000001096	UQCR11	28	0.003128097	0.00416158
ENSGALG00000054913		4	0.003132103	0.004166271
ENSGALG00000013591	BLOC1S4	2	0.003132306	0.004166271
ENSGALG00000005762	BIRC7	20	0.003158343	0.004200444
ENSGALG00000005214	TERB1	11	0.003174074	0.004220906
ENSGALG00000009916	SCFD1	5	0.003183728	0.004233282
ENSGALG00000006101	ARFRP1	20	0.003184408	0.004233725

ENSGALG00000030706	MAP3K6	23	0.00318773	0.004237678
ENSGALG00000005173	TUBD1	19	0.003191127	0.004241355
ENSGALG00000036638	TACR1	22	0.003191191	0.004241355
ENSGALG00000052976		4	0.003203889	0.004257768
ENSGALG00000037675	COL14A1	2	0.003205703	0.004259714
ENSGALG00000037336	PRTG	10	0.003208534	0.004263011
ENSGALG00000022536	GPR142	18	0.003211192	0.004266078
ENSGALG00000001412	SEMA7A	10	0.003211939	0.004266606
ENSGALG00000015591	ACOX3	4	0.003220232	0.004277155
ENSGALG00000007371	IGDCC3	10	0.003224476	0.004282326
ENSGALG00000040055		6	0.003239423	0.004301708
ENSGALG00000017032	SLC25A15	1	0.003247179	0.004311538
ENSGALG00000044841		19	0.00324768	0.004311734
ENSGALG00000037939	ADGRG1	11	0.00324895	0.00431295
ENSGALG00000009387		5	0.003252735	0.004317505
ENSGALG00000028900	CENPT	11	0.003262107	0.004329473
ENSGALG00000046299	CNKSR1	23	0.003271153	0.004340662
ENSGALG00000026687	PLCD4	7	0.003271249	0.004340662
ENSGALG00000017128	SKA3	1	0.003273184	0.004342757
ENSGALG00000009301	CHRNA1	7	0.003277729	0.004348314
ENSGALG00000050157		1	0.003289106	0.004362932
ENSGALG00000016442	RRM2	3	0.003310354	0.004390639
ENSGALG00000048876	LEAP2	13	0.003313435	0.004394247
ENSGALG00000044678		13	0.003326243	0.004410754
ENSGALG00000011235	GALNT15	2	0.003326635	0.004410794
ENSGALG00000045085	IFIT5	6	0.00335716	0.004450783
ENSGALG00000014378	ANAPC4	4	0.003357845	0.004451207
ENSGALG00000048285	TCN2	15	0.003405896	0.004514414
ENSGALG00000054766	ITGA2B	27	0.003406326	0.004514493
ENSGALG00000033063	GINS2	11	0.003414877	0.004525334
ENSGALG00000047126		1	0.003424521	0.00453762
ENSGALG00000043359	RRP9	12	0.0034533	0.004575257
ENSGALG00000010822	SPATA4	4	0.003458039	0.004581037

ENSGALG00000007665	POLE	15	0.003464263	0.004588784
ENSGALG00000037983	LUC7L	14	0.003469074	0.004594658
ENSGALG00000012129	CCDC93	7	0.003512216	0.004651293
ENSGALG00000007699	RPS25	24	0.003514092	0.004653271
ENSGALG00000009954	DYNC2LI1	3	0.003516024	0.004655324
ENSGALG00000020939	SLC35C2	20	0.00352018	0.00466032
ENSGALG00000049063	INAFM2	5	0.003546269	0.004693926
ENSGALG00000051392		4	0.003546333	0.004693926
ENSGALG00000013095	DERA	1	0.003551039	0.004699643
ENSGALG00000016918	GATD3AL1	1	0.003556489	0.004706346
ENSGALG00000016642		3	0.003559828	0.004710254
ENSGALG00000026161		28	0.003567785	0.00472027
ENSGALG00000008297	SEMA4B	10	0.00357743	0.004732276
ENSGALG00000021275	LTC4S	13	0.003577636	0.004732276
ENSGALG00000010130	INTS10	4	0.00360429	0.004767014
ENSGALG00000002070	SAP130	9	0.003614224	0.004779634
ENSGALG00000035360	XPO6	14	0.003621299	0.004788471
ENSGALG00000037112		2	0.003622556	0.004789615
ENSGALG00000039193	AGRN	21	0.003623351	0.004790146
ENSGALG00000002790	ABLIM3	13	0.003644876	0.004818081
ENSGALG00000015746	JAM2	1	0.003654828	0.004830712
ENSGALG00000003071	OGFOD1	11	0.003662458	0.004840272
ENSGALG00000032717	CCL4	19	0.003666699	0.004845351
ENSGALG00000004875	PEMT	14	0.003691646	0.00487779
ENSGALG00000010094	B4GALT2	8	0.003699174	0.004887207
ENSGALG00000006760	CSTF2	4	0.003710287	0.004901357
ENSGALG00000034964	SYTL1	23	0.003728859	0.004925357
ENSGALG00000042159	SLITRK3	9	0.003730379	0.004926832
ENSGALG00000028327		5	0.003736281	0.004934092
ENSGALG00000002882	BNIP1	13	0.003748862	0.00495017
ENSGALG00000033461		7	0.003762703	0.004967909
ENSGALG00000031966	TIMM9	5	0.003798643	0.005014797
ENSGALG00000052611		1	0.003799039	0.005014797

ENSGALG00000028537	NDUFB1	5	0.003831416	0.005056987
ENSGALG00000046487		33	0.003834958	0.005061115
ENSGALG00000050815		4	0.003840725	0.005068178
ENSGALG00000013780	AIG1	3	0.003843154	0.005070834
ENSGALG00000046708	PTGES	17	0.003849794	0.005079045
ENSGALG00000008658	ZNHIT6	8	0.003858882	0.005090485
ENSGALG00000039452	GJA5	1	0.003874555	0.005110607
ENSGALG00000040492	LONP1	28	0.003897811	0.005140726
ENSGALG00000006904	RNH1	5	0.003903971	0.005148293
ENSGALG00000031129	HMCN1	8	0.003914896	0.005162142
ENSGALG00000003597	AK2	23	0.003949195	0.005206652
ENSGALG00000046180	TDRD1	6	0.003949506	0.005206652
ENSGALG00000016997	RB1	1	0.003957005	0.005215974
ENSGALG00000003484	BARD1	7	0.003960073	0.005219454
ENSGALG00000011445	AAMP	7	0.003969412	0.005231197
ENSGALG00000033199		1	0.003986252	0.005252823
ENSGALG00000008070	ZMAT5	15	0.004016226	0.005291748
ENSGALG00000002242	GALNT9	15	0.004021266	0.005297817
ENSGALG00000012264	TRMT10A	4	0.004031371	0.005310556
ENSGALG00000046049		10	0.004055463	0.005341715
ENSGALG00000015821	CCT8	1	0.004067211	0.005356611
ENSGALG00000016832	PROZ	1	0.00407417	0.005365197
ENSGALG00000036175	CAPG	22	0.004082592	0.005375707
ENSGALG00000029390	C15H12ORF65	15	0.00409152	0.005386881
ENSGALG00000012015	NDST4	4	0.004100733	0.005398428
ENSGALG00000011003	SLC35F3	3	0.004132299	0.005439395
ENSGALG00000009034	ALK	3	0.004135446	0.00544295
ENSGALG00000042963		27	0.004147082	0.005457676
ENSGALG00000000993	GAPVD1	17	0.004162348	0.005477176
ENSGALG00000005696	ABHD6	12	0.004170762	0.005487655
ENSGALG00000036093	PTPN7	26	0.0041775	0.005495927
ENSGALG00000016702		1	0.00419825	0.005522631
ENSGALG00000048961		26	0.00419957	0.005523771

ENSGALG00000011174	PSMB1	3	0.004205634	0.005531151
ENSGALG00000031629	RPS6KB2	5	0.004227531	0.005559349
ENSGALG00000052084		2	0.004231152	0.005563511
ENSGALG00000011494	CNOT10	2	0.004249353	0.005586841
ENSGALG00000029795	FAM131A	9	0.004272105	0.005616149
ENSGALG00000001445	FABP6	13	0.004279584	0.005625374
ENSGALG00000040157	CHTF18	14	0.004287191	0.005634766
ENSGALG00000016993	SUCLA2	1	0.004314331	0.005669825
ENSGALG00000011035		8	0.004315121	0.005670252
ENSGALG00000027484	C20orf85	20	0.004324421	0.005681777
ENSGALG00000010340	TTLL5	5	0.004324824	0.005681777
ENSGALG00000016309	KLHL31	3	0.004335074	0.00569463
ENSGALG00000009890	MRPS10	3	0.004341049	0.005701865
ENSGALG00000035946	GUCY1A1	4	0.004365192	0.005732958
ENSGALG00000022733	ARL11	1	0.004368376	0.005736522
ENSGALG00000048261	HES6	9	0.00438207	0.005753886
ENSGALG00000030350		9	0.004384009	0.005755812
ENSGALG00000006073	NDUFAB1	14	0.004389798	0.005762791
ENSGALG00000028197	IQCK	14	0.004399796	0.005775294
ENSGALG00000011275	DEPDC1	8	0.004409531	0.005787451
ENSGALG00000016552	ZRSR2	1	0.004419883	0.005800412
ENSGALG00000000836	RFX5	25	0.004438257	0.005823899
ENSGALG00000003839	GRM2	12	0.004444785	0.005831837
ENSGALG00000051711		6	0.00444544	0.005832069
ENSGALG00000015441	CD247	1	0.004465865	0.005857837
ENSGALG00000052063	CEBPZOS	3	0.004466042	0.005857837
ENSGALG00000007979	CALML4	10	0.004478407	0.005873423
ENSGALG00000010645	ENPP6	4	0.004501634	0.00590325
ENSGALG00000020479	SLC43A3	5	0.004502821	0.005904172
ENSGALG00000052868		25	0.004512247	0.005915895
ENSGALG00000035362		21	0.004545063	0.005958278
ENSGALG00000040444	SEMA3C	1	0.004576488	0.00599883
ENSGALG00000051148		28	0.004625075	0.006061866

ENSGALG00000030397	TRIM27.1	16	0.004647197	0.006090206
ENSGALG00000007042	CCDC40	18	0.004651498	0.006095187
ENSGALG00000054032		1	0.004656696	0.006101343
ENSGALG00000043607	CENPX	18	0.004662195	0.006107891
ENSGALG00000042063	EPDR1	2	0.004666469	0.006112834
ENSGALG00000033653		25	0.00466726	0.006113213
ENSGALG00000030251	ADCY8	2	0.004688005	0.006139725
ENSGALG00000043015	FOXS1	20	0.004690761	0.006142675
ENSGALG00000049581		31	0.004692799	0.006144685
ENSGALG00000026875		21	0.004719039	0.006178379
ENSGALG00000011141	ITGB6	7	0.004720018	0.006178997
ENSGALG00000046616	CHGB	3	0.004727166	0.006187691
ENSGALG00000010343	TSPAN1	8	0.004744606	0.006209852
ENSGALG00000008585	SMC3	6	0.004746553	0.006211733
ENSGALG00000034899	NKAIN3	2	0.004747866	0.006212786
ENSGALG00000002588	PPARD	26	0.004752136	0.006217705
ENSGALG00000028561	SZT2	8	0.004761364	0.00622911
ENSGALG00000010621	DPY30	3	0.004778667	0.006251077
ENSGALG00000001711	PPCDC	10	0.004799757	0.006277992
ENSGALG00000050352	BCL2L15	26	0.004801543	0.006279654
ENSGALG00000040713		11	0.004808983	0.00628871
ENSGALG00000004231		23	0.004810408	0.006289899
ENSGALG00000047139		26	0.004816054	0.006296606
ENSGALG00000052051	COPS9	9	0.004821875	0.006303541
ENSGALG00000023757	TMEM199	19	0.004826855	0.006309374
ENSGALG00000048013	HTD2	12	0.00483901	0.006324584
ENSGALG00000017295	PTHLH	1	0.004845267	0.006332084
ENSGALG00000030086		33	0.004855908	0.00634531
ENSGALG00000009360	CAPN2	3	0.004858943	0.006348596
ENSGALG00000043047	SOBP	3	0.004868906	0.006360931
ENSGALG00000047103	UTP3	4	0.004873648	0.006366444
ENSGALG00000033729		31	0.00489096	0.006388375
ENSGALG00000052306		24	0.00490648	0.00640796

ENSGALG00000014997	PLA1A	1	0.004918593	0.006423092
ENSGALG00000022950	MRPL35	4	0.004919708	0.00642386
ENSGALG00000036742	GATSL2	19	0.004923776	0.006428483
ENSGALG00000028770	INHBB	7	0.004935541	0.006443153
ENSGALG00000009219	C3H1orf95	3	0.004957604	0.006471263
ENSGALG00000048064		2	0.004962582	0.006477067
ENSGALG00000014515	C1QTNF7	4	0.004981201	0.006500672
ENSGALG00000008378	SRGAP3	12	0.004998876	0.006523041
ENSGALG00000048707		25	0.005002577	0.006527171
ENSGALG00000051187	YJU2B	30	0.005005401	0.006530158
ENSGALG00000047574		16	0.005019239	0.006547511
ENSGALG00000008855	UGP2	3	0.005068272	0.006610766
ENSGALG00000033783	FER1L6	2	0.005097726	0.006648473
ENSGALG00000035466	CACNB4	7	0.005101926	0.006653239
ENSGALG00000016356	TDRP	3	0.005107719	0.006660081
ENSGALG00000021364	FAM173A	14	0.005116768	0.006671167
ENSGALG00000035645	GNAI2	12	0.005129638	0.006687231
ENSGALG00000019690	WDR86	2	0.005134011	0.006691853
ENSGALG00000039302	MINA	1	0.005134281	0.006691853
ENSGALG00000030918	RBM3	28	0.005135813	0.006693134
ENSGALG00000047794		16	0.005152459	0.006714111
ENSGALG00000042339		1	0.005173817	0.006741222
ENSGALG00000033320		19	0.005198648	0.006772852
ENSGALG00000027375	NR2C2AP	28	0.00520717	0.006783229
ENSGALG00000051860		22	0.005208025	0.006783619
ENSGALG00000053371		5	0.005208768	0.006783861
ENSGALG00000015693	USP25	1	0.005212504	0.006788002
ENSGALG00000036447		1	0.005222806	0.006800067
ENSGALG00000043903		23	0.005223034	0.006800067
ENSGALG00000017227	SLC36A4	1	0.005223442	0.006800067
ENSGALG00000039865	HAX1	25	0.005230798	0.006808916
ENSGALG00000050173	TCEA3	23	0.005253662	0.006837948
ENSGALG00000029721	C1orf232	23	0.005275597	0.006865766

ENSGALG00000040869		15	0.00528985	0.006883579
ENSGALG00000045795		8	0.005301011	0.006897367
ENSGALG00000030342	SARNP	33	0.005318497	0.00691938
ENSGALG00000041750	ADAMTS9	12	0.005358191	0.006970278
ENSGALG00000000309		26	0.005381185	0.006999444
ENSGALG00000005512	KIAA1671	15	0.005399374	0.007022354
ENSGALG00000013858	CEP76	2	0.005408807	0.007033217
ENSGALG00000014525	USP5	1	0.005408881	0.007033217
ENSGALG00000036678	ETV4	27	0.005455939	0.007093651
ENSGALG00000041826	S100A6	25	0.005458685	0.007096465
ENSGALG00000005195	OSBPL2	20	0.005500465	0.007150019
ENSGALG00000029794	AKAP7	3	0.005510988	0.007162933
ENSGALG00000005506	HESX1	12	0.005544284	0.007205442
ENSGALG00000002197	NPM1	13	0.005546865	0.007208027
ENSGALG00000001282	GABRD	21	0.005548594	0.007209507
ENSGALG00000016565	CHRNA2	3	0.005553831	0.007215542
ENSGALG00000004410	ANAPC16	6	0.005595272	0.007268608
ENSGALG00000055021	GREM2	3	0.005616378	0.007295248
ENSGALG00000001925	GNB1L	15	0.005625611	0.007306464
ENSGALG00000007311	CLDN2	4	0.005650625	0.007338169
ENSGALG00000037953	TUBA1A	33	0.005660437	0.007350128
ENSGALG00000010939	LIN7A	1	0.005662965	0.007352502
ENSGALG00000006120	PKD2L2	13	0.005663471	0.007352502
ENSGALG00000014918	RFX6	3	0.005677075	0.007369379
ENSGALG00000023686	ZSWIM7	19	0.005691909	0.007387848
ENSGALG00000027069	TSC22D4	4	0.005696409	0.007392903
ENSGALG00000021271	MPZL3	24	0.005700384	0.007397273
ENSGALG00000052797		18	0.005713822	0.007413923
ENSGALG00000000988	EFTUD2	27	0.005721936	0.00742366
ENSGALG00000006211	BCL2L1	20	0.005723354	0.007424711
ENSGALG00000014342	TEAD4	1	0.005766763	0.007480228
ENSGALG00000008493	METTL21A	7	0.005800991	0.007523826
ENSGALG00000016968	CCDC122	1	0.005822635	0.007551094

ENSGALG00000011498	TCAIM	2	0.00583835	0.007570669
ENSGALG00000000817	ZNF821	11	0.005843143	0.007576079
ENSGALG00000009512	TFPI2	2	0.005864813	0.007603367
ENSGALG00000036566		4	0.005873351	0.007613627
ENSGALG00000036378	TBR1	7	0.0058753	0.007615344
ENSGALG00000005936	AGTR2	4	0.005882132	0.007623388
ENSGALG00000038328	ANKRD33B	2	0.005893563	0.007637391
ENSGALG00000009318	IFT140	14	0.005925464	0.007677849
ENSGALG00000040060	NRF1	1	0.005926043	0.007677849
ENSGALG00000042476	TEX9	10	0.005928669	0.007680436
ENSGALG00000011803	EMP1	1	0.005930382	0.007681838
ENSGALG00000030008	HHEX	6	0.00596082	0.007720446
ENSGALG00000003802	OTUD7A	10	0.005961826	0.007720929
ENSGALG00000009491	RAD51B	5	0.005965232	0.007724519
ENSGALG00000045127		7	0.005982092	0.00774553
ENSGALG00000002990	PRELID1	13	0.006002391	0.007770987
ENSGALG00000007037	PPP6R3	5	0.006025486	0.007800059
ENSGALG00000009392	TLR5	3	0.006036194	0.00781309
ENSGALG00000049190		14	0.006038754	0.007814752
ENSGALG00000005983	SMARCB1	15	0.00603876	0.007814752
ENSGALG00000015833	NT5E	3	0.006057464	0.007838125
ENSGALG00000028119	CDC42SE1	25	0.006061254	0.007842198
ENSGALG00000004162	SLC16A6	18	0.006102129	0.007894245
ENSGALG00000004514	PDE6G	18	0.006107252	0.007900034
ENSGALG00000010317	POLH	3	0.006111788	0.007905064
ENSGALG00000003526	TUBGCP2	6	0.006117884	0.007912109
ENSGALG00000002157	RPS17	10	0.006148058	0.007950289
ENSGALG00000037526		28	0.006154257	0.007957461
ENSGALG00000000847	GARNL3	17	0.006175986	0.00798471
ENSGALG00000001862	ST6GALNAC1	18	0.006206912	0.008023439
ENSGALG00000001946	RSPO1	23	0.006207258	0.008023439
ENSGALG00000042984		12	0.006212828	0.008029787
ENSGALG00000039061	PHPT1	17	0.006230415	0.008051664

ENSGALG00000005451	HELLS	6	0.00625427	0.008081635
ENSGALG00000013183	TERT	2	0.006265759	0.008095624
ENSGALG00000040276	RUNX2	3	0.006285559	0.008120345
ENSGALG00000007673	LRRC59	18	0.006305408	0.008144282
ENSGALG00000015446	POU2F1	1	0.006305423	0.008144282
ENSGALG00000004917	DOC2B	19	0.006320721	0.008163176
ENSGALG00000042220	PRUNE1	25	0.006342235	0.008190094
ENSGALG00000031960	LEF1	4	0.00635316	0.008203334
ENSGALG00000014934	HS3ST1	4	0.006361622	0.00821339
ENSGALG00000003457	CNP	27	0.006372524	0.008226595
ENSGALG00000020626		9	0.006373456	0.008226927
ENSGALG00000001220	HERPUD1	11	0.006382575	0.008237825
ENSGALG00000016296	GFRAL	3	0.006392391	0.008249622
ENSGALG00000036552	PDAP1	14	0.006400294	0.008258947
ENSGALG00000038175	NAPRT	2	0.006434411	0.008302093
ENSGALG00000050919		9	0.006435497	0.008302615
ENSGALG00000052829	MAMDC4	17	0.006437979	0.008304938
ENSGALG00000004243		5	0.00645377	0.008324428
ENSGALG00000038528		3	0.006461538	0.008333566
ENSGALG00000005767	TRAF7	14	0.006466266	0.008338782
ENSGALG00000003362	ACSS2	20	0.006476603	0.008351229
ENSGALG00000036049	LETM2	22	0.006513266	0.008397616
ENSGALG00000037402	NOV	3	0.006526779	0.008414148
ENSGALG00000016875	CLYBL	1	0.006530224	0.0084177
ENSGALG00000017077	HSPH1	1	0.006585995	0.008488694
ENSGALG00000021074	MYOC	8	0.006590882	0.008494095
ENSGALG00000036550		33	0.006636508	0.008551992
ENSGALG00000005398	HAGH	14	0.006713943	0.008650862
ENSGALG00000047233		28	0.006724319	0.008663316
ENSGALG00000002963	PID1	9	0.006729838	0.00866951
ENSGALG00000010963		4	0.006758849	0.008705964
ENSGALG00000023355	TMEM154	4	0.006787346	0.008741746
ENSGALG00000052095		25	0.006800804	0.008758153

ENSGALG00000031487	FSTL4	13	0.006813403	0.008773453
ENSGALG00000011221	GPAT3	4	0.006820214	0.008781295
ENSGALG00000014950		3	0.006824865	0.008786356
ENSGALG00000046550		4	0.006833946	0.008797118
ENSGALG00000052038	MBLAC1	15	0.006859656	0.008829281
ENSGALG00000053641		25	0.006866955	0.008837744
ENSGALG00000016576	OFD1	1	0.006870083	0.008840836
ENSGALG00000001557	ACAD8	24	0.006878528	0.00885077
ENSGALG00000004252		28	0.006884574	0.008857615
ENSGALG00000031493	BAK1	26	0.006890567	0.008864389
ENSGALG00000004239	IFITM5	5	0.006894409	0.008868396
ENSGALG00000002276	EME2	14	0.006908441	0.008885508
ENSGALG00000040971	EGR2	6	0.006929526	0.008911688
ENSGALG00000040279	RHCE	23	0.006963108	0.008953931
ENSGALG00000003256	ATP6V0A1	27	0.007033012	0.009042868
ENSGALG00000052971		31	0.007176013	0.009225762
ENSGALG00000014701	RAP1GAP1	1	0.007177289	0.009226429
ENSGALG00000014915	THOC1	2	0.007220341	0.009280794
ENSGALG00000014463	ACTN2	3	0.007228212	0.009289932
ENSGALG00000047357		3	0.007231187	0.009292776
ENSGALG00000012513	STAM2	7	0.007239488	0.009302463
ENSGALG00000005619	F3	8	0.007249916	0.009314882
ENSGALG00000053063	MRPL40	15	0.007322345	0.009406949
ENSGALG00000040453	SRA1	13	0.007327994	0.009413214
ENSGALG00000053526	NTF3	1	0.007347283	0.009436999
ENSGALG00000037353	TMEM131	1	0.007361023	0.00945365
ENSGALG00000004383	TIMM22	19	0.007416475	0.009523863
ENSGALG00000050440	APOF	33	0.007435213	0.00954692
ENSGALG00000010296	MRPS18A	3	0.007462794	0.009581325
ENSGALG00000021686		23	0.007485046	0.009608882
ENSGALG00000017148	CENPJ	1	0.007533323	0.00966984
ENSGALG00000023396	ASPHD2	15	0.007537515	0.009674202
ENSGALG00000008850	TTLL7	8	0.007555716	0.009696542

ENSGALG00000006691	ARPC4	12	0.007578508	0.009724768
ENSGALG00000005503	WFDC1	11	0.007586982	0.009734617
ENSGALG00000031159	HIST1H110	1	0.007591128	0.009738912
ENSGALG00000043386	PEX5L	9	0.007612519	0.009765329
ENSGALG00000020316	IL13RA2	4	0.007625301	0.009780696
ENSGALG00000000652	ACOT7	21	0.00762825	0.009783449
ENSGALG00000042994	DDX41	13	0.007666975	0.009832081
ENSGALG00000021442	CARMIL2	11	0.007689326	0.009859706
ENSGALG00000044125	MLLT11	25	0.007691431	0.009860816
ENSGALG00000001091		26	0.007691808	0.009860816
ENSGALG00000015057	DDO	3	0.007705125	0.009876849
ENSGALG00000053478	CDK20	13	0.007713274	0.009886256
ENSGALG00000005422	PDLIM1	6	0.007731141	0.009908114
ENSGALG00000008523	XPNPEP1	6	0.007732154	0.009908371
ENSGALG00000042607	RSPO3	3	0.007788804	0.009979917
ENSGALG00000019848	AvBD13	3	0.007791696	0.009982573
ENSGALG00000013778	EXOC1	4	0.00785369	0.010060942
ENSGALG00000035237		7	0.00785817	0.010065623
ENSGALG00000052240		16	0.007859805	0.010066659
ENSGALG00000003313		23	0.007883521	0.010095974
ENSGALG00000004416	TTC19	19	0.007940931	0.010168428
ENSGALG00000029980	QARS	12	0.007987527	0.010227021
ENSGALG00000007909	IQCH	10	0.007989217	0.01022811
ENSGALG00000043016		5	0.007998463	0.010238873
ENSGALG00000054900		24	0.008008168	0.010250219
ENSGALG00000010842	UBR7	5	0.00802832	0.010274935
ENSGALG00000037989	IL10RB	1	0.008061229	0.01031597
ENSGALG00000038624		12	0.008099699	0.010364113
ENSGALG00000051209		1	0.00810057	0.01036414
ENSGALG00000015274	TCEA1	2	0.00810842	0.010373094
ENSGALG00000001502	UTP11	23	0.0081524	0.010428064
ENSGALG00000042452	DPP4	7	0.008153099	0.010428064
ENSGALG00000009470	SERPINI1	9	0.008193738	0.010478944

ENSGALG00000002108		18	0.008219076	0.010510246
ENSGALG00000001664	PTPN9	10	0.008302899	0.010616323
ENSGALG00000019094	PHF11	1	0.008378573	0.010711957
ENSGALG00000035113	DEXI	14	0.008392145	0.010728184
ENSGALG00000044344		4	0.008418024	0.01076014
ENSGALG00000035207	TDO2	4	0.008437868	0.010784373
ENSGALG00000010232	VRTN	5	0.008447335	0.010795341
ENSGALG00000045846	PDK2	27	0.008498196	0.010859202
ENSGALG00000004328	IGHMBP2	5	0.008509291	0.01087224
ENSGALG00000033769		1	0.008516362	0.010880134
ENSGALG00000008437	NET1	1	0.008519955	0.010883585
ENSGALG00000011473	RPS6KA2	3	0.008544369	0.010913628
ENSGALG00000016522	PPEF1	1	0.008613982	0.011000529
ENSGALG00000022871	HHLA2	1	0.008614209	0.011000529
ENSGALG00000003435	PGC	26	0.008653513	0.011049563
ENSGALG00000002892		6	0.008660236	0.011056991
ENSGALG00000050250		1	0.008716923	0.0111282
ENSGALG00000013647	TFB1M	3	0.0087182	0.011128666
ENSGALG00000008133	AS3MT	6	0.008751853	0.011170454
ENSGALG00000011738	ARHGDIB	1	0.008757369	0.011176323
ENSGALG00000049193	IQGAP3	25	0.008760357	0.011178009
ENSGALG00000030003	TUBG1	27	0.008760522	0.011178009
ENSGALG00000010341	XPO5	3	0.008777165	0.011198071
ENSGALG00000042325	CIZ1	17	0.008806624	0.01123448
ENSGALG00000011592	MRAS	7	0.008810217	0.011237514
ENSGALG00000008586	TUBB4B	17	0.008810845	0.011237514
ENSGALG00000028084	FAAH2	4	0.008815113	0.011241781
ENSGALG00000007785	PROK2	12	0.008850373	0.011285568
ENSGALG00000007848	PTS	24	0.008855195	0.011290536
ENSGALG00000019063	MMP10	1	0.008877882	0.011318278
ENSGALG00000052743	ARL4C	7	0.008902584	0.011348584
ENSGALG00000041845	ZNF692	16	0.008924308	0.011375087
ENSGALG00000011551	JCHAIN	4	0.008953167	0.011410679

ENSGALG00000008865	ALAD	17	0.00898804	0.011453927
ENSGALG00000032770	VRK2	3	0.009004	0.011473067
ENSGALG00000034605		5	0.009014449	0.01148518
ENSGALG00000007914	DNAJC1	2	0.00901961	0.011489615
ENSGALG00000003329	GORAB	8	0.009019814	0.011489615
ENSGALG00000034275	MCAT	1	0.009030552	0.011502091
ENSGALG00000030702	KIN	1	0.009031498	0.011502094
ENSGALG00000016872	PCCA	1	0.009036931	0.011507813
ENSGALG00000019961	SLC18B1	3	0.009039145	0.011509429
ENSGALG00000027053	AKAIN1	2	0.009049682	0.011521643
ENSGALG00000048954	DMP1	4	0.00905329	0.011525032
ENSGALG00000037776	TMCO6	13	0.009088579	0.011568749
ENSGALG00000037863	SEC61G	2	0.009123728	0.011612277
ENSGALG00000012466	FKBP3	5	0.009169778	0.01166967
ENSGALG00000006332	RBM17	1	0.009200356	0.011707361
ENSGALG00000042396	SLC12A7	2	0.00923054	0.011744545
ENSGALG00000017083	KATNAL1	1	0.009253921	0.011773065
ENSGALG00000051520		19	0.009255918	0.011774377
ENSGALG00000041353	TP53INP1	2	0.009291977	0.011819015
ENSGALG00000031222	SPRYD3	33	0.009352568	0.011894843
ENSGALG00000013157	BAG1	2	0.009378162	0.011926053
ENSGALG00000012766		1	0.009379063	0.011926053
ENSGALG00000032903		5	0.009389583	0.011937373
ENSGALG00000040249	BHLHE22	2	0.009389924	0.011937373
ENSGALG00000042589		26	0.009411945	0.011964121
ENSGALG00000017023	NEK5	1	0.009425072	0.011979558
ENSGALG00000015742	MRPL39	1	0.009498122	0.012071149
ENSGALG00000037238	PCCB	9	0.00954117	0.012124595
ENSGALG00000046358	FBLN7	3	0.009552317	0.012137495
ENSGALG00000006802	LAMB2	12	0.009580497	0.012172033
ENSGALG00000003774	ATP13A2	21	0.009611758	0.012210478
ENSGALG00000027986	NIPAL1	4	0.009615557	0.012214032
ENSGALG00000035140	TMEM106B	2	0.009682301	0.012297531

ENSGALG00000050229		23	0.009764751	0.012400959
ENSGALG00000006637		4	0.009772996	0.012410138
ENSGALG00000017363	C11H16ORF87	11	0.009780616	0.01241852
ENSGALG00000012109	EIF3M	5	0.009782539	0.012419668
ENSGALG00000015692		4	0.009834106	0.012483837
ENSGALG00000003879	MFSD2A	23	0.009851224	0.012504265
ENSGALG00000010219	UROD	8	0.009876673	0.012535263
ENSGALG00000050868		33	0.009939674	0.01261391
ENSGALG00000046248	ELMOD3	22	0.009977367	0.012660426
ENSGALG00000002346	CLSPN	23	0.010053431	0.012755617
ENSGALG00000042976	UBTF	27	0.010074463	0.012780973
ENSGALG00000043765		14	0.01010173	0.012814232
ENSGALG00000050047		33	0.01010696	0.012819532
ENSGALG00000021230	EPB42	20	0.01012408	0.012839911
ENSGALG00000014977	GSK3B	1	0.01013945	0.012858067
ENSGALG00000000109	AFMID	18	0.010152618	0.012873426
ENSGALG00000041873	MFN1	9	0.010159268	0.012880519
ENSGALG00000019231	CD200R1	1	0.010165856	0.012887532
ENSGALG00000041924	PFDN5	33	0.010171843	0.012893781
ENSGALG00000029624	DHRS4	1	0.010181035	0.012904092
ENSGALG00000012802	F13A1	2	0.010189871	0.012913949
ENSGALG00000030148	AUP1	4	0.010226228	0.012958678
ENSGALG00000003337	EIF3I	23	0.010412636	0.013193523
ENSGALG00000003212	TSPO2	26	0.010480654	0.013278327
ENSGALG00000010949	GPNMB	2	0.010491975	0.013291288
ENSGALG00000010331	MME	9	0.010531932	0.013340521
ENSGALG00000036171		2	0.010569227	0.01338637
ENSGALG00000009971	CDC20	8	0.010659981	0.013499911
ENSGALG00000049516		25	0.010671903	0.013513606
ENSGALG00000019262		1	0.01069429	0.013540548
ENSGALG00000049208		16	0.010696517	0.013541962
ENSGALG00000009626	THBS1	5	0.010704775	0.01355101
ENSGALG00000008749	POLR3F	3	0.010707086	0.013552528

ENSGALG00000009693		6	0.010724095	0.013572648
ENSGALG00000008298	LRRC17	1	0.010732243	0.013581551
ENSGALG00000047573		8	0.010750202	0.013602866
ENSGALG00000040260	TUBA1C	33	0.010769672	0.013626088
ENSGALG00000008866	WDPCP	3	0.010795151	0.013656908
ENSGALG00000010960	GALNT3	7	0.010826411	0.013695033
ENSGALG00000039733		28	0.010841959	0.013713279
ENSGALG00000020388		5	0.01085825	0.013732459
ENSGALG00000009345		5	0.010945376	0.013841213
ENSGALG00000012263	NEMF	5	0.010957941	0.013855665
ENSGALG00000003181	SURF2	17	0.011027662	0.013942376
ENSGALG00000046727		33	0.011117657	0.014054702
ENSGALG00000052628		11	0.011132324	0.014071783
ENSGALG00000029834	AIF1L	17	0.011222679	0.014184527
ENSGALG00000048539	BHLHE41	1	0.011275499	0.01424981
ENSGALG00000045196	LAPTM4B	2	0.011376906	0.014376476
ENSGALG00000030305	XKR8	23	0.011388308	0.014389393
ENSGALG00000051635		17	0.01139292	0.014393728
ENSGALG00000016774	NPAS2	1	0.011411068	0.014415163
ENSGALG00000045574	CAMK2N2	9	0.011434841	0.014443698
ENSGALG00000008386	KIZ	3	0.011454194	0.014466645
ENSGALG00000014818	TRIM45	1	0.011472443	0.014488193
ENSGALG00000013869	IL20RA	3	0.011478503	0.014494344
ENSGALG00000022920	SMYD5	4	0.011492422	0.014510418
ENSGALG00000032763	MECR	23	0.011499667	0.014518062
ENSGALG00000054272		14	0.011502815	0.014520533
ENSGALG00000016690	CYP2AC1	3	0.011506088	0.01452316
ENSGALG00000048097		1	0.011558198	0.014587425
ENSGALG00000030587		3	0.011559494	0.014587551
ENSGALG00000040184	RIMKLB	1	0.011576491	0.014607487
ENSGALG00000011409	SNRPF	1	0.011607187	0.014644706
ENSGALG00000019738	FBXO47	27	0.011712294	0.014775789
ENSGALG00000048452		25	0.011763004	0.014838228

ENSGALG00000024112	PSMD11	27	0.011768512	0.01484364
ENSGALG00000048152	SAE1	32	0.011773378	0.014848242
ENSGALG00000038939		33	0.011786025	0.014862654
ENSGALG00000013045	TUBA8B	1	0.011788862	0.014864695
ENSGALG00000043137		2	0.011807495	0.014886649
ENSGALG00000007531	ZFYVE27	6	0.01183353	0.014917931
ENSGALG00000001433	B3GNTL1	18	0.011853247	0.014941243
ENSGALG00000027655	TRABD2B	8	0.011912869	0.015014845
ENSGALG00000011875	TRPC3	4	0.011943087	0.015051375
ENSGALG00000009519	ARG2	5	0.011948583	0.015056745
ENSGALG00000004568	CELA2A	21	0.011952042	0.015059548
ENSGALG00000029558		27	0.011959323	0.015067165
ENSGALG00000027151	FKBP1A	20	0.012114203	0.015260597
ENSGALG00000002945	TMEM266	10	0.01211536	0.015260597
ENSGALG00000012056	GRAP2	1	0.012150954	0.01530385
ENSGALG00000047878		27	0.012181045	0.015340165
ENSGALG00000012370	GINM1	3	0.012220008	0.015387644
ENSGALG00000034354		5	0.012226301	0.015393978
ENSGALG00000043771	DENND6B	1	0.012243772	0.015414385
ENSGALG00000008144	COPG2	1	0.012245626	0.015415127
ENSGALG00000049755	IL22RA2	3	0.01224749	0.015415882
ENSGALG00000032178	MAP2K4	18	0.01229407	0.015472914
ENSGALG00000032645	H2A-VIII	1	0.012314497	0.015497023
ENSGALG00000000545	REN	26	0.012335562	0.015521931
ENSGALG00000006037	MCM2	12	0.012370065	0.015563739
ENSGALG00000001489	SSU72	21	0.012391525	0.015589131
ENSGALG00000009501	TMA16	4	0.012413905	0.015615675
ENSGALG00000040471	MVP	28	0.012435739	0.015641528
ENSGALG00000037941	ZC3H10	33	0.012448255	0.015655655
ENSGALG00000008787	CEP68	3	0.012508176	0.015729392
ENSGALG00000004123	PABPC1L	20	0.012546395	0.015775828
ENSGALG00000053276		2	0.012576946	0.015812611
ENSGALG00000004832	RBM41	4	0.01259661	0.015835701

ENSGALG00000021017	DUSP15	20	0.012624535	0.015869171
ENSGALG00000036871		8	0.0126791	0.015934673
ENSGALG00000015652		4	0.012679258	0.015934673
ENSGALG00000011450		13	0.012703192	0.015963106
ENSGALG00000019761		18	0.012785456	0.016064826
ENSGALG00000009503	RAB3GAP2	3	0.01280349	0.016085827
ENSGALG00000006841	POLR2L	5	0.012826933	0.01611362
ENSGALG00000033001	ATP5J2	14	0.012898597	0.016201978
ENSGALG00000050840	APCDD1	2	0.012925579	0.016234197
ENSGALG00000042020	CG-16	4	0.012931247	0.016239643
ENSGALG00000001973	GNL2	23	0.012950491	0.016262135
ENSGALG00000037714	BBIP1	6	0.012995189	0.016316544
ENSGALG00000008606	NDUFS1	7	0.012996496	0.016316544
ENSGALG00000033102	LRRFIP2	2	0.013008087	0.016329414
ENSGALG00000053247	SFTPC	22	0.013043099	0.016371681
ENSGALG00000025937	FXYD6	24	0.013052478	0.016381767
ENSGALG00000051142	FAM180B	5	0.013063558	0.016393986
ENSGALG00000028608	BTC	4	0.013163996	0.016518329
ENSGALG00000049547		31	0.013185449	0.016543545
ENSGALG00000002649	UBE4B	21	0.013191443	0.016549364
ENSGALG00000003842	GHRH	20	0.013221069	0.016584825
ENSGALG00000003799	USP4	12	0.013244093	0.016611997
ENSGALG00000039171	RAD52	1	0.013245931	0.016612594
ENSGALG00000000576	METTL25B	25	0.013269702	0.016640694
ENSGALG00000054226		16	0.013271626	0.016641396
ENSGALG00000016724	RGN	1	0.01328388	0.016655049
ENSGALG00000008635		1	0.013311307	0.016687721
ENSGALG00000042761	MUL1	21	0.0133346	0.016715202
ENSGALG00000026359	SERTAD4	3	0.013338185	0.016717978
ENSGALG00000012115	WT1	5	0.013341027	0.016719821
ENSGALG00000002591	POLR2E	28	0.013352463	0.016732434
ENSGALG00000034456	PRELP	26	0.013379044	0.01676402
ENSGALG00000001310	TRNAU1AP	23	0.013398664	0.016786879

ENSGALG00000012082	FAIM	7	0.013470922	0.016875676
ENSGALG00000026471	CRIP2	8	0.013513212	0.016926915
ENSGALG00000032220	ELN	19	0.013526775	0.016942164
ENSGALG00000003096	CBFA2T2	20	0.013531749	0.016946653
ENSGALG00000032843	AAAS	33	0.013556445	0.016975837
ENSGALG00000008503	ALG3	9	0.013562985	0.016982284
ENSGALG00000006662	BPIFB3	20	0.013569573	0.016988788
ENSGALG00000006792	VSTM2L	20	0.01358188	0.01700245
ENSGALG00000020342		5	0.013635614	0.017067965
ENSGALG00000014194	SAMM50	1	0.013705354	0.017153498
ENSGALG00000042577	CFAP44	1	0.013726237	0.017177872
ENSGALG00000004796	CDC42	21	0.013798534	0.017266577
ENSGALG00000049433		31	0.01383306	0.017308004
ENSGALG00000014973	GPR156	1	0.013840843	0.017315965
ENSGALG00000002315		9	0.013845209	0.017319651
ENSGALG00000006182	NSFL1C	20	0.013872599	0.017352134
ENSGALG00000034134	ZNF302	18	0.013972003	0.017474677
ENSGALG00000034094	SP5	7	0.013991514	0.017497285
ENSGALG00000010719	NEIL3	4	0.013998242	0.017503903
ENSGALG00000016964		1	0.014051604	0.017568827
ENSGALG00000003978	CIB3	28	0.014068116	0.017586275
ENSGALG00000012006	RTN1	5	0.014068443	0.017586275
ENSGALG00000003647	SLC13A2	19	0.014086074	0.017606509
ENSGALG00000017611	NTMT1	17	0.014103176	0.017626079
ENSGALG00000033150	MIDN	28	0.014105241	0.017626853
ENSGALG00000003614	AZIN2	23	0.014169849	0.017705777
ENSGALG00000005517	KIF9	2	0.014305024	0.017872851
ENSGALG00000041407		2	0.014339015	0.017913485
ENSGALG00000029731	TPP1	1	0.014417225	0.018009346
ENSGALG00000003537	SGK2	20	0.014495645	0.01810545
ENSGALG00000052088	SFR1	6	0.014513576	0.01812599
ENSGALG00000007709	RTFDC1	20	0.014583824	0.018211857
ENSGALG00000010703	DGLUCY	5	0.014667179	0.018314072

ENSGALG00000031381	VAMP4	8	0.014754281	0.018420945
ENSGALG00000003241	CCDC28B	23	0.014764677	0.018432037
ENSGALG00000006704	VPS8	9	0.014771652	0.018438857
ENSGALG00000012587	NAA20	7	0.014844073	0.01852736
ENSGALG00000035676	PDDC1	5	0.014853978	0.018537825
ENSGALG00000054252	TM4SF1	9	0.014862562	0.01854664
ENSGALG00000014372	SLC34A2	4	0.014912296	0.018606797
ENSGALG00000001437	NTM	24	0.014921495	0.01861637
ENSGALG00000003250	ITPA	8	0.0149308	0.018626074
ENSGALG00000008185	AOX1	7	0.014939874	0.018635487
ENSGALG00000009240	NFE2L2	7	0.0150407	0.018759335
ENSGALG00000009468		9	0.015052893	0.018772622
ENSGALG00000033039	ENG	17	0.015092389	0.018819953
ENSGALG00000002849	DCXR	18	0.01509617	0.018822743
ENSGALG00000006306	UROC1	12	0.015164837	0.018906427
ENSGALG00000007262	SPG21	10	0.015267122	0.019032003
ENSGALG00000026446	CPLX3	10	0.015271594	0.019035631
ENSGALG00000036418	ANXA13	2	0.015306421	0.019077092
ENSGALG00000003548	BAIAP2L1	14	0.015346216	0.019124735
ENSGALG00000010609	NDUFAF7	3	0.01534861	0.019125764
ENSGALG00000053112		6	0.015455234	0.019256659
ENSGALG00000030461	ADIRF	6	0.015487088	0.019294377
ENSGALG00000037332	IGFBP7	4	0.015549876	0.019370621
ENSGALG00000033204	PQLC2	21	0.015590411	0.019419132
ENSGALG00000007759	TMEFF2	7	0.015593022	0.01941921
ENSGALG00000034475	EIF3G	30	0.015593659	0.01941921
ENSGALG00000049741	SMIM18	4	0.015642101	0.019477546
ENSGALG00000006572	TNNT3	5	0.015701524	0.019549544
ENSGALG00000001945	RFT1	12	0.015748139	0.019605581
ENSGALG00000027287	HK3	13	0.015767504	0.019627685
ENSGALG00000011271	LUM	1	0.015772792	0.019632263
ENSGALG00000008149	EWSR1	15	0.015809639	0.019674722
ENSGALG00000047148	LIPT2	1	0.015810131	0.019674722

ENSGALG00000041153	PHETA1	15	0.01588038	0.019760126
ENSGALG00000002448	CCT6A	19	0.015893139	0.019773984
ENSGALG00000033535	RGS16	8	0.015899567	0.019779963
ENSGALG00000023882	MALL	3	0.015947159	0.019837146
ENSGALG00000002595	GPX4	28	0.01598557	0.019882898
ENSGALG00000050753		3	0.016002068	0.019901389
ENSGALG00000055069		5	0.016044228	0.019951787
ENSGALG00000029829	RNLS	6	0.016053831	0.019961693
ENSGALG00000004286	COL13A1	6	0.016089234	0.020003674
ENSGALG00000040348	EHBP1	3	0.016166565	0.02009777
ENSGALG00000010850	C8A	8	0.016177957	0.020109881
ENSGALG00000042733		25	0.016191816	0.020125057
ENSGALG00000031918	RAD51D	19	0.016203184	0.020137134
ENSGALG00000016228	NYX	1	0.016223237	0.020160001
ENSGALG00000008985	SRI	2	0.016261127	0.020205026
ENSGALG00000044026		3	0.016290778	0.020239806
ENSGALG00000042642	RARRES2	2	0.01632478	0.020279984
ENSGALG00000019147		1	0.016404611	0.020377081
ENSGALG00000047890	PCP4	1	0.016416883	0.020390247
ENSGALG00000035803	THRSP	1	0.016421491	0.020393892
ENSGALG00000003211	POLDIP2	19	0.016433882	0.020407202
ENSGALG00000009091	TRUB1	6	0.016489535	0.020474226
ENSGALG00000029897	RPL30	2	0.016547439	0.020544031
ENSGALG00000048236		33	0.016571317	0.02057158
ENSGALG00000017018	INTS6	1	0.016613259	0.020621548
ENSGALG00000004153	NADSYN1	5	0.016623175	0.02063072
ENSGALG00000042555	STAMBP	22	0.016624032	0.02063072
ENSGALG00000005584	GALNT18	5	0.016692909	0.020714089
ENSGALG00000035029		14	0.016711229	0.020734712
ENSGALG00000002921	LANCL1	7	0.016721323	0.020745126
ENSGALG00000002247	SETD6	11	0.016768216	0.020801186
ENSGALG00000000868	RNF121	1	0.01678704	0.020822419
ENSGALG00000008236	PSMC2	1	0.016802726	0.020839755

ENSGALG00000003590	C17orf58	18	0.016866699	0.020916971
ENSGALG00000040521	HOXA2	2	0.016903568	0.020960562
ENSGALG00000006857	SPATA20	18	0.01695676	0.021024382
ENSGALG00000034218		33	0.016962514	0.021029379
ENSGALG00000035156	DCTN1	4	0.017001226	0.021075229
ENSGALG00000028250	ANKRD22	6	0.017037353	0.021117866
ENSGALG00000007762	CREBBP	14	0.017046333	0.021126849
ENSGALG00000043597	RAD54B	2	0.017048315	0.021127157
ENSGALG00000042103	C5H11ORF96	5	0.017095784	0.02118383
ENSGALG00000016676	MCM3	3	0.017120561	0.021212377
ENSGALG00000016723		3	0.017194623	0.021301975
ENSGALG00000042129		25	0.0172087	0.021317249
ENSGALG00000038096	NOS2	19	0.017269051	0.021389836
ENSGALG00000016446	ATP6V1C2	3	0.017321159	0.021452198
ENSGALG00000009234	AMDHD2	14	0.017337294	0.02147
ENSGALG00000034761		21	0.017359495	0.02149531
ENSGALG00000006051	CDC7	8	0.017370081	0.021506234
ENSGALG00000054378	LRRC43	15	0.017419361	0.021565059
ENSGALG00000027184	OLFML1	5	0.017458787	0.021611674
ENSGALG00000051899	DND1	13	0.017490792	0.021649094
ENSGALG00000034989	CEP112	18	0.017567092	0.021741327
ENSGALG00000010882	G3BP2	4	0.017607033	0.021788547
ENSGALG00000041098		5	0.017625668	0.021809394
ENSGALG00000005922	RPL5	8	0.017653706	0.021841871
ENSGALG00000000784	PIP5K1C	28	0.017712376	0.021911172
ENSGALG00000041413	PPP3CC	22	0.017713313	0.021911172
ENSGALG00000011184	HNRNPD	4	0.017724495	0.021922781
ENSGALG00000029382	BARX2	24	0.017746827	0.021948175
ENSGALG00000039978	SLC4A1	27	0.017765463	0.021968995
ENSGALG00000031143	DCPS	24	0.017779068	0.02198359
ENSGALG00000023506		3	0.017782536	0.021985648
ENSGALG00000002461		15	0.017785274	0.021986804
ENSGALG00000003807	GPN3	15	0.0178593	0.022076079

ENSGALG00000007267	MVD	11	0.017966021	0.022205747
ENSGALG00000050257		1	0.017992558	0.022236292
ENSGALG00000031659		1	0.017997307	0.022239908
ENSGALG00000033118	FAM83H	2	0.01806318	0.022319046
ENSGALG00000002644	RPL10A	26	0.018132991	0.022403036
ENSGALG00000049236	PEBP4	22	0.018224317	0.022513587
ENSGALG00000001320	CYP1A2	10	0.018263871	0.022560164
ENSGALG00000044592		31	0.018309631	0.022614397
ENSGALG00000009926	HAAO	3	0.018327097	0.022633677
ENSGALG00000010208	CAPS2	1	0.018380374	0.022697174
ENSGALG00000009840	TBK1	1	0.018396835	0.022715201
ENSGALG00000005421		14	0.018404194	0.022721987
ENSGALG00000053052		31	0.018411279	0.022728432
ENSGALG00000022815	AvBD1	3	0.018441512	0.02276345
ENSGALG00000008549	TUBGCP4	10	0.01847414	0.022801416
ENSGALG00000007825	KIAA1841	3	0.018479887	0.0228062
ENSGALG00000010124	RPS8	8	0.018508439	0.022839126
ENSGALG00000017320	RAB6A	1	0.018527453	0.022860275
ENSGALG00000029319	UQCRB	2	0.018592616	0.022938356
ENSGALG00000009940	SLC35E3	1	0.018598316	0.022943066
ENSGALG00000013060	ADCY2	2	0.018605657	0.0229498
ENSGALG00000049672		33	0.018763013	0.023141555
ENSGALG00000004825		4	0.018900705	0.023309021
ENSGALG00000038604	PHB	27	0.018933511	0.023347118
ENSGALG00000047979	EBF4	4	0.019035535	0.023470551
ENSGALG00000024480	TIMMDC1	1	0.019041832	0.023475941
ENSGALG00000015244	TBX19	1	0.019070058	0.023508363
ENSGALG00000047320	MFAP5	1	0.019072009	0.023508391
ENSGALG00000001244		18	0.019084037	0.02352084
ENSGALG00000036297	ABHD8	28	0.019086722	0.023521771
ENSGALG00000030411	PSMA7	20	0.019093272	0.023527465
ENSGALG00000004367	AWAT1	4	0.019146148	0.023590237
ENSGALG00000009670	SGCE	2	0.019229884	0.023691015

ENSGALG00000006599	ZMYND11	2	0.019244346	0.023706437
ENSGALG00000035882	TANGO6	11	0.019252867	0.023714538
ENSGALG00000030492	ASCL2	5	0.019301862	0.023772485
ENSGALG00000046156		1	0.01937643	0.023861914
ENSGALG00000013495	GSDMA	27	0.01938574	0.023870968
ENSGALG00000003076	FASLG	8	0.019490603	0.02399767
ENSGALG00000004500	COP1	8	0.019531142	0.024045155
ENSGALG00000005204		15	0.019557508	0.024075184
ENSGALG00000040987	NDUFA8	17	0.019565256	0.02408229
ENSGALG00000006250	TRAPPC2L	11	0.019569216	0.024084733
ENSGALG00000012691	ALDH1L2	1	0.019583469	0.024099842
ENSGALG00000028573		3	0.019607185	0.024126592
ENSGALG00000042232	PSMG4	2	0.01961106	0.024128926
ENSGALG00000050086	RNF207	21	0.019623505	0.024141802
ENSGALG00000004657	FBXO2	21	0.019631975	0.024149785
ENSGALG00000020788	CCDC141	7	0.019648718	0.024167943
ENSGALG00000005843	EEF1A2	20	0.01970672	0.02423684
ENSGALG00000000616	ELF3	26	0.019713681	0.024242957
ENSGALG00000010855	MTHFD2L	4	0.019745835	0.024280049
ENSGALG00000006057	CLK4	13	0.01974802	0.024280287
ENSGALG00000003948	ALAS1	12	0.019757691	0.024289729
ENSGALG00000025938	KIAA0513	11	0.019799627	0.024338829
ENSGALG00000050503	TNFRSF4	21	0.019903267	0.024463764
ENSGALG00000011301	LRRC40	8	0.019917146	0.024478355
ENSGALG00000032740	HOXB7	27	0.01992974	0.024491364
ENSGALG00000008460	ZFYVE19	5	0.019946308	0.024509255
ENSGALG00000024490		4	0.020007436	0.024581889
ENSGALG00000031940	ORMDL2	33	0.020031519	0.024608998
ENSGALG00000028017	EFCAB2	3	0.020066303	0.024649247
ENSGALG00000003580	MMP2	11	0.020092963	0.024677521
ENSGALG00000033589	SYN3	1	0.020093367	0.024677521
ENSGALG00000025959	MKRN2OS	12	0.020099275	0.02468229
ENSGALG00000004251	ARR3	4	0.020196545	0.024799242

ENSGALG00000025935	XPNPEP2	4	0.020471707	0.025134581
ENSGALG00000000102	UBE2T	26	0.020492413	0.02515747
ENSGALG00000000748	TJP3	28	0.020561617	0.025239887
ENSGALG00000006710	RAB36	15	0.020639947	0.025333488
ENSGALG00000014514	CC2D2A	4	0.020658289	0.025353449
ENSGALG00000029145	DCUN1D2	1	0.020669252	0.025364351
ENSGALG00000016721	PLCXD1	1	0.020751105	0.025462235
ENSGALG00000035799	SHISA9	14	0.020753613	0.02546275
ENSGALG00000031293	SLCO1A2	1	0.020881421	0.02561698
ENSGALG00000003424	LMX1A	8	0.020989688	0.02574721
ENSGALG00000036935		5	0.021073077	0.0258469
ENSGALG00000035116		9	0.021132371	0.025917019
ENSGALG00000038097	EI24	24	0.021216682	0.026017802
ENSGALG00000000466	LCK	23	0.021246017	0.026051155
ENSGALG00000010859	EPGN	4	0.021250208	0.026053674
ENSGALG00000033695	CYP26A1	6	0.02142592	0.026266463
ENSGALG00000046316	CFAP97D1	27	0.02146713	0.026314338
ENSGALG00000000474	RPL36	28	0.021508406	0.026362283
ENSGALG00000050235		30	0.021520702	0.026374703
ENSGALG00000001798	BBS4	10	0.021537517	0.026392658
ENSGALG00000050965		18	0.021579022	0.026440862
ENSGALG00000015404		2	0.021595681	0.026458615
ENSGALG00000017652	TTC9	5	0.021739277	0.02663187
ENSGALG00000039817	NOG2	14	0.021767898	0.026664254
ENSGALG00000032925	STK40	23	0.021798745	0.026699356
ENSGALG00000010203		8	0.02181523	0.026716864
ENSGALG00000033452		7	0.021818075	0.026717664
ENSGALG00000035675		18	0.021923407	0.026843955
ENSGALG00000005959	GLMN	8	0.021991081	0.026924114
ENSGALG00000004794	H2AFY2	6	0.022031306	0.026970654
ENSGALG00000011080	BDKRB2	5	0.022044765	0.026984421
ENSGALG00000021665		19	0.022190442	0.027160014
ENSGALG00000031839	SUV39H1	30	0.022240306	0.027218313

ENSGALG00000045953		2	0.022348069	0.027347451
ENSGALG00000042572		1	0.02238155	0.027385673
ENSGALG00000045514	PEX6	3	0.022447071	0.027463088
ENSGALG00000006207	HM13	20	0.022559025	0.02759729
ENSGALG00000054713		30	0.022565114	0.02760197
ENSGALG00000001470	STOML1	10	0.022621615	0.027668306
ENSGALG00000031139	LSM3	12	0.022661693	0.027714546
ENSGALG00000035604	USB1	11	0.022679511	0.027733556
ENSGALG00000007681	HTR2B	9	0.022763398	0.027833345
ENSGALG00000007216	TVP23A	14	0.022804668	0.027881011
ENSGALG00000052652		26	0.022824371	0.027902302
ENSGALG00000001109	GTSF1	27	0.022828237	0.02790423
ENSGALG00000005287	ZBTB47	2	0.023017231	0.028132427
ENSGALG00000001483		21	0.023048831	0.028168226
ENSGALG00000039606	CLN8	3	0.02313218	0.028267256
ENSGALG00000041449	EXD1	5	0.023305278	0.028475924
ENSGALG00000033212	HSP90AA1	5	0.023313387	0.028482979
ENSGALG00000017068	KL	1	0.023443773	0.028639408
ENSGALG00000010647	ECHDC2	8	0.023456896	0.028652568
ENSGALG00000013943		4	0.023757839	0.029017262
ENSGALG00000032149	OSBPL7	27	0.023780309	0.029041797
ENSGALG00000026816		18	0.023808592	0.029073426
ENSGALG00000040702	BCKDHB	3	0.023874904	0.029151483
ENSGALG00000007653	RAE1	20	0.023898743	0.029177668
ENSGALG00000005332	CACNA1D	12	0.024003604	0.029302758
ENSGALG00000048929		31	0.024299654	0.029661196
ENSGALG00000043823	ECSIT	30	0.024304524	0.02966417
ENSGALG00000026005	DIO2	5	0.02432287	0.029683591
ENSGALG00000052894		1	0.024508403	0.029907021
ENSGALG00000034104	DNAJB12	6	0.024770487	0.030223812
ENSGALG00000030907	CSF3	27	0.024827187	0.030289963
ENSGALG00000038520	STRIP2	1	0.024894609	0.030369182
ENSGALG00000035049		15	0.024898471	0.030370855

ENSGALG00000001189	NCF1C	19	0.024923547	0.0303984
ENSGALG00000002735	TAF11	26	0.025072704	0.030577263
ENSGALG00000030153		5	0.025202477	0.030732453
ENSGALG00000003143	ZC3H7A	14	0.025229608	0.030762461
ENSGALG00000017418	NPTX1	18	0.025293361	0.030837111
ENSGALG00000008233	BUB1	3	0.025356583	0.0309111
ENSGALG00000047516		1	0.025363215	0.030916093
ENSGALG00000006326	MIF	15	0.02539814	0.03095557
ENSGALG00000037852	HSD17B7	8	0.025413713	0.030971455
ENSGALG00000033790	MAN2A2	10	0.02556827	0.031155914
ENSGALG00000003793	GTF2A2	10	0.025570182	0.031155914
ENSGALG00000018815	PCGF2	27	0.025587642	0.031174073
ENSGALG00000027864	NFKBIA	5	0.025670394	0.031271766
ENSGALG00000011335	NHEJ1	7	0.025774662	0.03139565
ENSGALG00000042814	MCRIP1	18	0.025821149	0.031449133
ENSGALG00000054834		1	0.025860636	0.031494081
ENSGALG00000013414	PDLIM3	4	0.025898277	0.031536772
ENSGALG00000014841	HDDC2	3	0.025916244	0.031555499
ENSGALG00000032683	ADTRP	2	0.025941476	0.031583067
ENSGALG00000003820	ARPC3	15	0.026025918	0.03168271
ENSGALG00000046724	PRRG3	4	0.026047264	0.03170553
ENSGALG00000052251		33	0.026098452	0.031764666
ENSGALG00000047034		31	0.026105785	0.03177042
ENSGALG00000006045	SH3GL3	10	0.026119516	0.031783959
ENSGALG00000043082	DHX36	9	0.026136243	0.031801138
ENSGALG00000028145	BORCS6	3	0.026282838	0.031976317
ENSGALG00000035836	LDHB	1	0.0266139	0.032375864
ENSGALG00000002286	H3F3B	18	0.026709848	0.032489343
ENSGALG00000007658	ARMH3	6	0.026767515	0.03255624
ENSGALG00000006707	NOX1	4	0.026837678	0.032638321
ENSGALG00000045606		1	0.02694717	0.03276821
ENSGALG00000049290		15	0.027078545	0.032924681
ENSGALG00000019845	AvBD9	3	0.027092519	0.032938387

ENSGALG00000010091	ATP6V0B	8	0.027115889	0.032963512
ENSGALG00000006958		17	0.027154349	0.033006955
ENSGALG00000011646	PLD4	5	0.027157039	0.033006955
ENSGALG00000038396		6	0.027206483	0.033063754
ENSGALG00000031430		8	0.02725275	0.033116681
ENSGALG00000014060	EED	1	0.027261661	0.033124209
ENSGALG00000044849		6	0.027283715	0.033147702
ENSGALG00000007723	NAT9	18	0.027298306	0.033160342
ENSGALG00000031553	KIF18B	27	0.027299559	0.033160342
ENSGALG00000050491	SLC35E4	15	0.027385955	0.033261974
ENSGALG00000005277	RTCA	8	0.027548109	0.033455587
ENSGALG00000013356	IKBKE	26	0.027608513	0.033525605
ENSGALG00000028638	CNP1	1	0.027706229	0.033640913
ENSGALG00000016500	FKBP1B	3	0.027713863	0.033646832
ENSGALG00000032730	NOP56	4	0.027783942	0.033728554
ENSGALG00000016979	SLC25A30	1	0.027829178	0.033780106
ENSGALG00000007276	GCN1	15	0.027839157	0.033788855
ENSGALG00000006547	PROCR	9	0.027847064	0.033795088
ENSGALG00000005869	COPG1	12	0.027872207	0.033822236
ENSGALG00000037558		28	0.027875134	0.033822421
ENSGALG00000035465	WDR76	10	0.02793881	0.03389631
ENSGALG00000010794	MEOX2	2	0.027972664	0.033934006
ENSGALG00000008538	GINS1	3	0.028126055	0.034116692
ENSGALG00000015844	SOD1	1	0.028178202	0.034176546
ENSGALG00000009369	PRDX3	6	0.02819563	0.034194283
ENSGALG00000004141	LBFABP	23	0.028200876	0.034197243
ENSGALG00000039071		25	0.028297848	0.034311422
ENSGALG00000049437		8	0.028393413	0.034423872
ENSGALG00000005694	GARS	2	0.028451486	0.03449085
ENSGALG00000037935	RARA	27	0.02846888	0.034508505
ENSGALG00000003254	BAG4	22	0.02862227	0.034689566
ENSGALG00000002121	PLEKHN1	21	0.028623942	0.034689566
ENSGALG00000013189	LPCAT1	2	0.028649858	0.034717523

ENSGALG00000012246	R3HDM1	7	0.028708401	0.034785008
ENSGALG00000048190		19	0.028753284	0.034835929
ENSGALG00000053241		7	0.028791454	0.034878708
ENSGALG00000010122	SH2D4A	4	0.02881078	0.034898653
ENSGALG00000030194	SCLY	9	0.028837092	0.034927054
ENSGALG00000044151		17	0.028900112	0.034999907
ENSGALG00000003467	BYSL	26	0.02897121	0.035082527
ENSGALG00000008595	RTF1	5	0.028975767	0.035084561
ENSGALG00000016739	HERC2	1	0.02899619	0.035105803
ENSGALG00000030025	FABP4	2	0.029087204	0.035212498
ENSGALG00000029446		1	0.029134991	0.035266847
ENSGALG00000051336	ANKLE1	28	0.029220079	0.035366332
ENSGALG00000034655	ALDH18A1	6	0.029242156	0.035389539
ENSGALG00000005090	SKA2	19	0.029273092	0.035423462
ENSGALG00000047761		31	0.029281651	0.035430304
ENSGALG00000030808		19	0.029516725	0.035711195
ENSGALG00000035260	RBM14	33	0.029538487	0.035733979
ENSGALG00000014897		2	0.029571652	0.035770551
ENSGALG00000012731	TBC1D7	2	0.029579091	0.035776
ENSGALG00000037408	ADMP	28	0.029623576	0.035826251
ENSGALG00000000249	GH	27	0.029628893	0.035829127
ENSGALG00000005367	MRPS22	9	0.029662423	0.035866117
ENSGALG00000015339	RPL24	1	0.029670599	0.035872446
ENSGALG00000051564		19	0.029859189	0.036096877
ENSGALG00000015287	LNP1	1	0.029905184	0.036148895
ENSGALG00000038313	EFHD2	21	0.029983492	0.03623996
ENSGALG00000020415	IFT43	5	0.029991009	0.036245453
ENSGALG00000051665	LYRM4	2	0.03023896	0.036541491
ENSGALG00000000141	BLB1	16	0.03042294	0.036760174
ENSGALG00000049557		33	0.030595611	0.036965149
ENSGALG00000054310		18	0.030680443	0.03706397
ENSGALG00000007508	HPSE2	6	0.030761561	0.037158283
ENSGALG00000052939		25	0.030773324	0.03716881

ENSGALG00000004469	TP53RK	20	0.030822669	0.037224723
ENSGALG00000036309	LARS2	2	0.030838542	0.037240204
ENSGALG00000004493	RSRC2	15	0.030867076	0.03727097
ENSGALG00000006565		15	0.030936243	0.037350558
ENSGALG00000039008		11	0.030939116	0.037350558
ENSGALG00000011325	NDUFA12	1	0.030981063	0.037397495
ENSGALG00000041752	CD164L2	23	0.031230294	0.037694613
ENSGALG00000013940	LNX1	4	0.031289906	0.037762825
ENSGALG00000005217	MOGAT1	9	0.031436966	0.037936553
ENSGALG00000011630	GLI2	7	0.031523155	0.038036796
ENSGALG00000014722		1	0.031583253	0.038105542
ENSGALG00000011993	RBX1	1	0.03159807	0.038119646
ENSGALG00000029444	CLK2	25	0.031847792	0.038417108
ENSGALG00000047596	SMKR1	1	0.031865553	0.03843473
ENSGALG00000012153	METTL15P1	5	0.03196349	0.038549044
ENSGALG00000029819	SCG2	9	0.031996325	0.038584827
ENSGALG00000002244	AGRP	11	0.032118739	0.038728617
ENSGALG00000011376	ANKRD9	5	0.032204919	0.038828693
ENSGALG00000052799		21	0.032388962	0.039046729
ENSGALG00000052994		27	0.032433952	0.039097101
ENSGALG00000047638		28	0.032451512	0.039114402
ENSGALG00000002388	NIPSNAP2	19	0.032466824	0.039128989
ENSGALG00000052243	CYP2C18	6	0.032815991	0.039542345
ENSGALG00000015492	PDZK1	1	0.032816287	0.039542345
ENSGALG00000001452	ATP5PB	26	0.032967389	0.039718556
ENSGALG00000017163	ACAT1	1	0.03296904	0.039718556
ENSGALG00000002321	GOT2	11	0.033070678	0.039837066
ENSGALG00000054099		15	0.033196995	0.039985277
ENSGALG00000020575	ERMN	7	0.03329546	0.040098729
ENSGALG00000008608	FETUB	9	0.033297764	0.040098729
ENSGALG00000001382	CDC2L1	21	0.033309449	0.040108839
ENSGALG00000038783		22	0.033320167	0.040117784
ENSGALG00000014920	USP14	2	0.033337561	0.040134763

ENSGALG00000016582	PBK	3	0.033367963	0.040167397
ENSGALG00000003660	FANCF	5	0.033371753	0.040167994
ENSGALG00000042132	SUGP1	28	0.033480642	0.040295081
ENSGALG00000007058	LSG1	9	0.033491417	0.040304071
ENSGALG00000029260	H-RAS	5	0.033524343	0.040339713
ENSGALG00000012457	SSTR3	1	0.03357539	0.040397151
ENSGALG00000000538	RNF113B	27	0.033715444	0.040561659
ENSGALG00000014457	ING4	1	0.033769121	0.040622227
ENSGALG00000052986		9	0.033800678	0.040656177
ENSGALG00000001791		11	0.033831436	0.040689159
ENSGALG00000053278	SUCNR1	9	0.033861026	0.040720731
ENSGALG00000031482	Pou5f3	17	0.033961927	0.040838045
ENSGALG00000035768	SCAP	2	0.033971138	0.040845093
ENSGALG00000013191	NDUFS6	2	0.033984711	0.040857383
ENSGALG00000034168	WNT5A	12	0.033991479	0.040861491
ENSGALG00000030097	RCC1	23	0.034035514	0.040910392
ENSGALG00000023616	LRRC31	9	0.034136257	0.041027441
ENSGALG00000022696	KCNE3	1	0.034218904	0.041122718
ENSGALG00000005653	NFKB2	6	0.034222986	0.04112357
ENSGALG00000008735	BFSP1	3	0.034332626	0.041251252
ENSGALG00000038952	MTFP1	15	0.034336736	0.041252125
ENSGALG00000000857	NMT1	27	0.034706712	0.041692506
ENSGALG00000015018	CASQ2	1	0.034844739	0.041854192
ENSGALG00000033996	TMEM220	18	0.034909374	0.041927699
ENSGALG00000008601	AHSG	9	0.034950625	0.041973108
ENSGALG00000029235	CPNE4	2	0.035109743	0.042160045
ENSGALG00000049385		31	0.03515122	0.042205694
ENSGALG00000006434	TSR3	14	0.035162334	0.042214882
ENSGALG00000054583	CDHR4	12	0.03521725	0.04227665
ENSGALG00000047281		31	0.03534314	0.042423598
ENSGALG00000048117	DNLZ	17	0.035479637	0.042583247
ENSGALG00000027454	CENPW	3	0.035542392	0.042654368
ENSGALG00000011687		5	0.035861051	0.043032555

ENSGALG00000037018	USP36	18	0.036072298	0.043281788
ENSGALG00000010663	TTC8	5	0.036267486	0.043511705
ENSGALG00000040665	METTL6	2	0.036504169	0.043791355
ENSGALG00000039628	AFAP1L1	13	0.036560033	0.043854056
ENSGALG00000039408	PNPLA6	30	0.036564413	0.043854996
ENSGALG00000043926		21	0.03659952	0.043892785
ENSGALG00000040807		22	0.036780268	0.044105214
ENSGALG00000051628		25	0.036809455	0.044135872
ENSGALG00000006491	ANKRD1	6	0.036949394	0.04429527
ENSGALG00000031565	NEBL	2	0.036949659	0.04429527
ENSGALG00000002046		6	0.036954479	0.044296693
ENSGALG00000000227	DPYSL2	22	0.03706567	0.044425608
ENSGALG00000050001	RNASEK	31	0.037344349	0.044755224
ENSGALG00000014140	ARFGAP3	1	0.037379333	0.044792748
ENSGALG00000033989	SLC39A14	22	0.03744176	0.044863147
ENSGALG00000052043		4	0.037455749	0.044875499
ENSGALG00000014371	LGALS8	3	0.037480011	0.044900155
ENSGALG00000053647		25	0.03752816	0.04495342
ENSGALG00000014689	FAM131B	1	0.037565252	0.04499343
ENSGALG00000002968	ZNF341	20	0.037696214	0.045145854
ENSGALG00000049431		6	0.037767566	0.045226864
ENSGALG00000042936	SYCE3	1	0.037809586	0.045272738
ENSGALG00000010537	CDKN2C	8	0.037813619	0.04527312
ENSGALG00000004503		19	0.037950873	0.045432989
ENSGALG00000013528		22	0.038074133	0.045576075
ENSGALG00000019284		1	0.038115928	0.045621627
ENSGALG00000047781	RAD9B	15	0.038204144	0.045722726
ENSGALG00000026315	RAB34	19	0.038299728	0.045832622
ENSGALG00000009655	AADAT	4	0.038312294	0.04584316
ENSGALG00000010433		8	0.038446987	0.045999815
ENSGALG00000009755	LRIG3	1	0.0385081	0.046068412
ENSGALG00000002773	SNRPC	26	0.038692962	0.046285027
ENSGALG00000006973	SH2D6	6	0.038785537	0.046391216

ENSGALG00000004343	HPD	15	0.039011422	0.046655262
ENSGALG00000009612	TGFB2	3	0.039013947	0.046655262
ENSGALG00000035350	PMEL	33	0.039033712	0.046674211
ENSGALG00000046347	FOXRED1	24	0.039037448	0.046674211
ENSGALG00000005208	S1PR1	8	0.039086843	0.046728687
ENSGALG00000023703	GIT1	19	0.039093065	0.046731543
ENSGALG00000010500	NKX6-2	6	0.03916588	0.046813996
ENSGALG00000046559		9	0.039352736	0.047032729
ENSGALG00000015762	LYRM2	3	0.039472962	0.047171795
ENSGALG00000047297	PROK1	26	0.039777569	0.047531155
ENSGALG00000010218		9	0.039923941	0.047701382
ENSGALG00000024449	RAMP2	27	0.039943787	0.047720419
ENSGALG00000043672	STAB1	12	0.04008037	0.047878902
ENSGALG00000035058	CAMKV	12	0.040105921	0.047904731
ENSGALG00000004799		19	0.040175422	0.047983046
ENSGALG00000001615	MAN2C1	10	0.040211106	0.048020962
ENSGALG00000029000	HYAL3	12	0.040311954	0.048136681
ENSGALG00000008609	PTK7	3	0.040344124	0.048170378
ENSGALG00000041613	HEATR3	11	0.040394424	0.048225714
ENSGALG00000002701	DNAJC9	6	0.040430598	0.048264175
ENSGALG00000015780	PNRC1	3	0.040468037	0.048304138
ENSGALG00000010473	PDZK1IP1	8	0.040656269	0.048524069
ENSGALG00000005623	TFIP11	15	0.040972075	0.048896203
ENSGALG00000031511	HIP1R	15	0.041094321	0.049037292
ENSGALG00000036530	PRPF19	5	0.041127331	0.04907188
ENSGALG00000041280	FOSB	32	0.041307146	0.049281608
ENSGALG00000016674	EFHC1	3	0.041345224	0.04932221
ENSGALG00000053152	FSD1	28	0.041478745	0.049476653
ENSGALG00000006597	DZIP1L	9	0.041492619	0.049487352
ENSGALG00000015806	RARS2	3	0.041495833	0.049487352
ENSGALG00000028720		1	0.041698194	0.049723822
ENSGALG00000001652	SEPT2L	15	0.041733359	0.049760889
ENSGALG00000017207	CWC15	1	0.04193077	0.049991384

ENSGALG00000016476	TTC32	3	0.041989198	0.050056148
ENSGALG00000030533	PGLS	30	0.042145795	0.050237919
ENSGALG00000007320	FXYD2	24	0.042305899	0.050423835
ENSGALG00000051119	PCP4L1	25	0.042386977	0.050515532
ENSGALG00000044433		23	0.042456701	0.050593682
ENSGALG00000006241		9	0.042505078	0.050646381
ENSGALG00000017008	RCBTB1	1	0.042548167	0.050692769
ENSGALG00000045185		5	0.042555946	0.050697084
ENSGALG00000012218	CBY1	1	0.042685554	0.050844925
ENSGALG00000016624	ELP3	3	0.042688387	0.050844925
ENSGALG00000013621	ATP6V1H	2	0.042742817	0.050904783
ENSGALG00000030028	MRPS14	8	0.042923972	0.051115537
ENSGALG00000007585	NIT1	25	0.043208986	0.051449513
ENSGALG00000008150	RASAL1	15	0.043212865	0.051449513
ENSGALG00000045634	XKR5	3	0.043377515	0.051640504
ENSGALG00000006490	SCN3B	24	0.043445475	0.05171636
ENSGALG00000017168	SLN	1	0.043460906	0.051729679
ENSGALG00000031642	ILK	1	0.043643939	0.051942464
ENSGALG00000037014	TSNARE1	2	0.043767222	0.052084105
ENSGALG00000011391	AMN	5	0.043852131	0.052180056
ENSGALG00000016351	ACOT9	1	0.04389191	0.052222293
ENSGALG00000013073	UPP1	2	0.044056657	0.052413193
ENSGALG00000027762	PGAP2	1	0.044072009	0.052426343
ENSGALG00000010463	AHSA1	5	0.044161102	0.052527199
ENSGALG00000003221	WDR61	10	0.044300223	0.052687536
ENSGALG00000049153	PDF	11	0.044337766	0.052727043
ENSGALG00000010119	CDKL2	4	0.044417239	0.052816403
ENSGALG00000036086	TAGLN2	25	0.044560062	0.052981067
ENSGALG00000003925	MYPN	6	0.044736503	0.053185665
ENSGALG00000043623	ARL14	9	0.04477493	0.05322616
ENSGALG00000004238	MRM2	14	0.044835877	0.053293415
ENSGALG00000001177	CD34	26	0.044975146	0.053453743
ENSGALG00000049119	ZNF638	4	0.045108018	0.053606439

ENSGALG00000034438	GNB3	1	0.045136592	0.053635169
ENSGALG00000045949		1	0.045170235	0.053669916
ENSGALG00000008007	ITGA11	10	0.045198332	0.053698067
ENSGALG00000048034		23	0.045323403	0.053841412
ENSGALG00000006505	ZFAND6	10	0.045329017	0.053842836
ENSGALG00000049369		5	0.04543053	0.053958158
ENSGALG00000010098	SLC6A9	8	0.045455186	0.053982184
ENSGALG00000037665	LMBR1L	33	0.04558546	0.054131624
ENSGALG00000016287	NR0B1	1	0.045750607	0.054318689
ENSGALG00000028205	PRPF3	25	0.045751902	0.054318689
ENSGALG00000048899		30	0.045843508	0.05442215
ENSGALG00000028326	HSD11B2	11	0.045870331	0.05444869
ENSGALG00000014127		4	0.045892919	0.0544702
ENSGALG00000013640	MBP	2	0.046050934	0.054652428
ENSGALG00000002603	COG4	11	0.046058463	0.054656043
ENSGALG00000036282	PRKAG1	33	0.046078695	0.054674731
ENSGALG00000002868	RPL26L1	13	0.046226794	0.05484512
ENSGALG00000010607	SPATA7	5	0.046358478	0.054996003
ENSGALG00000006776	KLHDC8B	12	0.046467784	0.055120313
ENSGALG00000010752	CDCP2	8	0.046817183	0.055529369
ENSGALG00000016042	CHAF1B	1	0.04688068	0.055599273
ENSGALG00000010402	HPGDS	4	0.046923764	0.055644958
ENSGALG00000032856	GFRA2	22	0.046986853	0.055714354
ENSGALG00000004176	ATOX1	13	0.047001886	0.05572676
ENSGALG00000004813	IARS1	12	0.047131307	0.055874771
ENSGALG00000017316	UCP3	1	0.047250626	0.05601078
ENSGALG00000049751		1	0.0474385	0.056228019
ENSGALG00000006482	FAH	10	0.047576747	0.056386399
ENSGALG00000020921		10	0.047646144	0.056463158
ENSGALG00000000223	TMEM183A	26	0.047655087	0.056468268
ENSGALG00000031520		28	0.047818997	0.056656985
ENSGALG00000008536	KLHDC3	3	0.04797823	0.056840124
ENSGALG00000047587		30	0.048122218	0.057005169

ENSGALG00000041334	SNRPD1	2	0.048313303	0.057225967
ENSGALG00000010991	ALG6	8	0.048502032	0.057443931
ENSGALG00000049420		20	0.048583416	0.057534731
ENSGALG00000035384	TMEM241	2	0.048610381	0.057557334
ENSGALG00000026945	CIDEC	11	0.048611943	0.057557334
ENSGALG00000002150	CPEB1	10	0.048661479	0.05761039
ENSGALG00000001845	PHTF1	26	0.048837069	0.057812659
ENSGALG00000016995	MED4	1	0.048876552	0.057853781
ENSGALG00000006192	USH1C	5	0.04908719	0.058097467
ENSGALG00000008294	ITPR1	12	0.049114044	0.058123609
ENSGALG00000016648		1	0.049174883	0.05818996
ENSGALG00000013101		16	0.049183838	0.058194909
ENSGALG00000007495	SLC24A1	10	0.049215142	0.058226298
ENSGALG00000043885	CFAP45	25	0.049415996	0.058458255
ENSGALG00000038265	CREB5	2	0.049433363	0.058473128
ENSGALG00000032468	SNX17	3	0.049612524	0.058679358
ENSGALG00000037307	IQCD	15	0.049703575	0.058781348
ENSGALG00000031186		1	0.049735631	0.058813553
ENSGALG00000032858	RSL24D1	10	0.049828321	0.058917448
ENSGALG00000033034	SLC25A34	21	0.049909546	0.059007766
ENSGALG00000010432	GSTZ1	5	0.049988994	0.059095967
ENSGALG00000034502	SCD5	4	0.050033339	0.059142657
ENSGALG00000031013	DHDH	31	0.050228883	0.059368046
ENSGALG00000008095	KIF23	10	0.050362804	0.059520565
ENSGALG00000010406	TMEM63C	5	0.050412122	0.059573077
ENSGALG00000037262	HOXC8	33	0.050466641	0.059631723
ENSGALG00000047567		33	0.050769435	0.059983694
ENSGALG00000052872		25	0.050798951	0.060012752
ENSGALG00000002094	TRMT2A	15	0.050804161	0.060013092
ENSGALG00000014573	NAT10	5	0.050831274	0.060039303
ENSGALG00000016801	UXS1	1	0.051285322	0.060569734
ENSGALG00000004525	TSPAN10	18	0.051370485	0.060664439
ENSGALG00000006246		9	0.051507832	0.060820743

ENSGALG00000038596	C14orf132	5	0.051844848	0.061210461
ENSGALG00000006809	POU2AF1	24	0.051847916	0.061210461
ENSGALG00000026081	RBM38	20	0.051944203	0.061318198
ENSGALG00000013165	FH	2	0.052058267	0.061446897
ENSGALG00000011476	SEPTIN11	4	0.052164666	0.061566525
ENSGALG00000054629	PDCD2L	11	0.052273766	0.061689317
ENSGALG00000053885		8	0.052361168	0.061786482
ENSGALG00000035906	YTHDC1	4	0.05237816	0.061800552
ENSGALG00000041120	METTL24	3	0.052410745	0.061833015
ENSGALG00000055000	KCTD14	1	0.052622763	0.062077143
ENSGALG00000040855	PTPRR	1	0.052642999	0.062095007
ENSGALG00000015831	SNX14	3	0.052661394	0.062110696
ENSGALG00000020647	LDLRAD1	8	0.053109066	0.062632638
ENSGALG00000050866	HSD3B1	1	0.053401292	0.062971175
ENSGALG00000038748	RSRP1	23	0.053438078	0.063008459
ENSGALG00000042822	TAX1BP1	2	0.053498939	0.063074122
ENSGALG00000030801	CCKAR	4	0.053658454	0.06325607
ENSGALG00000050005		31	0.05366744	0.063260546
ENSGALG00000014126		1	0.053751431	0.063353427
ENSGALG00000030824	SF3B5	3	0.053829097	0.063438834
ENSGALG00000017048	EXOSC8	1	0.054044519	0.063686558
ENSGALG00000012291	POLR2F	1	0.054065047	0.063704592
ENSGALG00000009559	IFT80	9	0.054187866	0.063843139
ENSGALG00000026294		9	0.054294268	0.06396232
ENSGALG00000008661	CYR61	8	0.054439726	0.064127483
ENSGALG00000004442	PPA1	6	0.054648828	0.064367578
ENSGALG00000028284	PTX3	9	0.054684363	0.064403211
ENSGALG00000001613	GNL3	12	0.054696946	0.064406484
ENSGALG00000044472		16	0.054697707	0.064406484
ENSGALG00000010125	CSGALNACT1	4	0.054717698	0.064423801
ENSGALG00000009983	NUBPL	5	0.054759465	0.064466753
ENSGALG00000019312		1	0.054837597	0.064552503
ENSGALG00000044569		16	0.054969259	0.064701242

ENSGALG00000004669	ASPA	19	0.055183334	0.064946949
ENSGALG00000051063		20	0.055425436	0.06522559
ENSGALG00000024077		1	0.055470302	0.065272089
ENSGALG00000005318	OVCH2	5	0.055653408	0.065481231
ENSGALG00000014991	HDAC2	3	0.055828927	0.065675147
ENSGALG00000032231	C4	16	0.055828992	0.065675147
ENSGALG00000005789	UQCRC1	12	0.055883464	0.065732884
ENSGALG00000009045	EBNA1BP2	8	0.056091366	0.065971065
ENSGALG00000039590		2	0.056145255	0.066028076
ENSGALG00000039022	PDE1B	33	0.056159782	0.06603879
ENSGALG00000035800	CAMKMT	3	0.056368323	0.066277624
ENSGALG00000010052	PPP2R3C	5	0.056389901	0.066296603
ENSGALG00000049395		14	0.056490771	0.06640879
ENSGALG00000001184	COQ9	11	0.056507909	0.066417363
ENSGALG00000026914	DOK3	13	0.056508958	0.066417363
ENSGALG00000006123		20	0.056743964	0.066687147
ENSGALG00000012971	CCDC170	3	0.056807348	0.066755204
ENSGALG00000037896		32	0.056900419	0.06685813
ENSGALG00000023354	FSCN2	18	0.056958509	0.066919936
ENSGALG00000012545	CYTIP	7	0.057193874	0.067189989
ENSGALG00000007299	THOC7	12	0.057298038	0.067305874
ENSGALG00000021158	SLC7A9	11	0.057346048	0.067352542
ENSGALG00000038649	DONSON	1	0.057348814	0.067352542
ENSGALG00000054250		30	0.05758149	0.067619292
ENSGALG00000049231	DNAJC22	33	0.057717753	0.067772781
ENSGALG00000036908		1	0.057850588	0.067922217
ENSGALG00000005617	NTHL1	14	0.058013562	0.068107006
ENSGALG00000003636	FBLIM1	21	0.058096272	0.06819754
ENSGALG00000031193	IGSF9	25	0.058179985	0.068289234
ENSGALG00000006064	TEKT1	19	0.05820717	0.068314565
ENSGALG00000011289	NKIRAS1	2	0.058330242	0.06845242
ENSGALG00000021443	PANX3	24	0.058338748	0.068455813
ENSGALG00000008660	CST7	3	0.058371154	0.068487248

ENSGALG00000007456	NPRL3	14	0.058388108	0.068500548
ENSGALG00000043506	NMB	10	0.05850578	0.068631997
ENSGALG00000031720	LGI1	6	0.058788655	0.068957198
ENSGALG00000032690	DDX51	15	0.058870009	0.069045982
ENSGALG00000008931	COMMD1	3	0.058972122	0.069159093
ENSGALG00000002055	HES4	21	0.059033868	0.069224846
ENSGALG00000039088	TRAPPC5	30	0.059343959	0.069581777
ENSGALG00000035080	HARS	13	0.059418795	0.069662823
ENSGALG00000007645		6	0.059676901	0.069958702
ENSGALG00000034258	C25H1ORF43	25	0.059810677	0.070108785
ENSGALG00000040619	BAAT	5	0.05991361	0.07022269
ENSGALG00000013196	ANKS6	2	0.05994336	0.070250807
ENSGALG00000052767		1	0.059974768	0.07028086
ENSGALG00000034099	ATP6V1C1	2	0.060194424	0.070531483
ENSGALG00000009415	SMOC1	5	0.060205321	0.070537473
ENSGALG00000031826	MYOT	13	0.060259873	0.070594604
ENSGALG00000002503	SFTPA2	6	0.060377654	0.070725789
ENSGALG00000002937	PSME3	27	0.060454889	0.070809459
ENSGALG00000053046		7	0.060626591	0.071003748
ENSGALG00000005385	ACTR8	12	0.060664887	0.071041776
ENSGALG00000012041	ACTR10	5	0.060816727	0.071212749
ENSGALG00000003569	TMEM130	14	0.060884955	0.071285794
ENSGALG00000035199	ODF1	2	0.060918885	0.071318672
ENSGALG00000033391	ISL2	10	0.061023715	0.071434538
ENSGALG00000047627		16	0.061042677	0.071449876
ENSGALG00000007882		24	0.061149487	0.071568026
ENSGALG00000034198	MCM10	1	0.061330078	0.071772496
ENSGALG00000013400	PDCD6	2	0.061368234	0.071810256
ENSGALG00000007374	YPEL4	5	0.061604445	0.072079742
ENSGALG00000036783		8	0.06177799	0.072275861
ENSGALG00000005487	CCDC12	2	0.06186234	0.072367601
ENSGALG00000045705	H2AX	24	0.062279345	0.072848432
ENSGALG00000008249	DNAJC2	1	0.062376175	0.072954696

ENSGALG00000006944		12	0.062584938	0.073190368
ENSGALG00000010741	MAP1LC3C	3	0.06258968	0.073190368
ENSGALG00000014570	LPCAT3	1	0.062759062	0.0733814
ENSGALG00000035461	CHORDC1	1	0.062772212	0.073389738
ENSGALG00000003909	PIGT	20	0.062793865	0.073408015
ENSGALG00000010226		8	0.063229036	0.073907592
ENSGALG00000000607	GPR37L1	26	0.063233331	0.073907592
ENSGALG00000032746	ENPP2	2	0.063301417	0.07398008
ENSGALG00000005995	SLC13A5	19	0.063659804	0.074391795
ENSGALG00000031227	ELP6	2	0.063890499	0.074654226
ENSGALG00000012118	TMEM260	5	0.064076663	0.074864579
ENSGALG00000038789	CLIC3	17	0.064384535	0.075217078
ENSGALG00000019741	PSMC3IP	27	0.064488792	0.075331658
ENSGALG00000006632	CEP63	9	0.064531676	0.07537453
ENSGALG00000012975	IQSEC3	1	0.064642707	0.075496985
ENSGALG00000023781	TREM2	26	0.064857162	0.075740195
ENSGALG00000001049		19	0.064863983	0.075740906
ENSGALG00000029223	THOC5	15	0.064980431	0.075869615
ENSGALG00000039474	ID4	2	0.065074902	0.075972643
ENSGALG00000026159	PDLIM7	13	0.065106863	0.076002679
ENSGALG00000005688	DNASE1L3	12	0.065142165	0.076036609
ENSGALG00000039034	ELP2	2	0.06526384	0.076167736
ENSGALG00000031929		9	0.065266998	0.076167736
ENSGALG00000030502	ADAMTS4	25	0.06530062	0.076199681
ENSGALG00000030200	NFU1	22	0.06543568	0.076349976
ENSGALG00000040976	ACTR6	1	0.065599066	0.07653329
ENSGALG00000001288		21	0.065607609	0.076535933
ENSGALG00000006379	SHH	2	0.065688819	0.07662334
ENSGALG00000008567		3	0.065744545	0.076681006
ENSGALG00000016581	TCEANC	1	0.065836214	0.076780579
ENSGALG00000042151		4	0.065928487	0.076880838
ENSGALG00000040070	PDIA2	14	0.066273465	0.077275735
ENSGALG00000041266	CNN1	30	0.066434951	0.077456622

ENSGALG00000029812	RAB25	25	0.066447745	0.077464131
ENSGALG00000008380	DGKZ	5	0.066557143	0.077584249
ENSGALG00000045115		30	0.066641706	0.077675397
ENSGALG00000016555	HADHB	3	0.066736735	0.077778724
ENSGALG00000010201	ABHD18	4	0.066748072	0.077784501
ENSGALG00000000977	MAPKAP1	17	0.06683602	0.077879548
ENSGALG00000002615	DEF6	26	0.066861259	0.077901512
ENSGALG00000041258		23	0.067270098	0.078370371
ENSGALG00000053552	IER5L	17	0.067319303	0.078420203
ENSGALG00000010803		2	0.067648527	0.078796187
ENSGALG00000006272		9	0.067717794	0.078869334
ENSGALG00000039346	ARHGAP39	2	0.067903451	0.079074391
ENSGALG00000035203	MCRS1	33	0.067906827	0.079074391
ENSGALG00000008287	PSMD10	4	0.068043022	0.079225417
ENSGALG00000011086	ACTA1	3	0.068061508	0.079239374
ENSGALG00000008043	CORO2B	10	0.068085133	0.079259311
ENSGALG00000004472	ASTN1	8	0.068240083	0.079432108
ENSGALG00000035996	COX6C	2	0.068587277	0.079828625
ENSGALG00000032634	CFAP20	11	0.068643111	0.079885984
ENSGALG00000035499	SKIV2L	26	0.068662962	0.07990146
ENSGALG00000041213	ARHGAP17	14	0.06914536	0.080455136
ENSGALG00000011096	ABCB10	3	0.069488724	0.080846947
ENSGALG00000054015	PRR11	19	0.069518601	0.080873992
ENSGALG00000053116	LRRC61	2	0.069536081	0.080886609
ENSGALG00000030313	RAD51	5	0.069830121	0.081220897
ENSGALG00000020084	CAPN13	3	0.06989309	0.081286384
ENSGALG00000016716	STAG3	1	0.070222339	0.081661515
ENSGALG00000008352	CKMT1B	10	0.070576799	0.082065889
ENSGALG00000005338	RPS21	20	0.070661584	0.082156641
ENSGALG00000032372	CYB5R2	5	0.070826121	0.082338094
ENSGALG00000026293	C3orf33	9	0.070831155	0.082338094
ENSGALG00000050552		17	0.070929219	0.082444229
ENSGALG00000014067	SGTA	28	0.071017703	0.08253921

ENSGALG00000003213	LGALS2	1	0.071031726	0.08254764
ENSGALG00000002630	ZNF76	26	0.07128542	0.082834569
ENSGALG00000027788	CPE	4	0.071509948	0.083087554
ENSGALG00000031170	ADCY1	2	0.071622261	0.083210121
ENSGALG00000051055	MAPK8IP2	1	0.071662947	0.083242811
ENSGALG00000044464	TEPSIN	18	0.071664052	0.083242811
ENSGALG00000005948	RPL27A	5	0.071846577	0.083446876
ENSGALG00000019114	CLDN10	1	0.071979003	0.083592722
ENSGALG00000027546	BZFP1	16	0.072317852	0.083978244
ENSGALG00000001149	ADGRG5	11	0.072516798	0.084201249
ENSGALG00000016836	TUBGCP3	1	0.072568115	0.084252811
ENSGALG00000027562	PURG	4	0.072823406	0.084539955
ENSGALG00000016364	ALKAL2	3	0.072829303	0.084539955
ENSGALG00000054424		2	0.072855206	0.084561973
ENSGALG00000011684	STAP1	4	0.072947702	0.084661273
ENSGALG00000006677	P4HA2	13	0.072991698	0.084704271
ENSGALG00000030121		15	0.07300795	0.084715067
ENSGALG00000004302	NDUFA10	7	0.073049591	0.08475532
ENSGALG00000055025		16	0.073463323	0.085227241
ENSGALG00000011834	NUDT6	4	0.073628346	0.085410563
ENSGALG00000048038		1	0.073725641	0.085515292
ENSGALG00000015112	ZNF521	2	0.073858173	0.085660868
ENSGALG00000007941	C5H11orf74	5	0.073988676	0.085804064
ENSGALG00000050888		2	0.074186904	0.086025765
ENSGALG00000001718	CCNG1	13	0.074222374	0.086058711
ENSGALG00000012732	PHACTR1	2	0.074229443	0.086058724
ENSGALG00000019716	KRT40	27	0.074524602	0.086384811
ENSGALG00000049003		25	0.074524877	0.086384811
ENSGALG00000004734		4	0.074533286	0.086386345
ENSGALG00000046984	M1AP	4	0.074838617	0.086731989
ENSGALG00000039326	HSBP1L1	2	0.075216112	0.087161191
ENSGALG00000020210	CENPU	4	0.075438638	0.08741075
ENSGALG00000047383	CCN5	20	0.075514481	0.087490314

ENSGALG00000007570		4	0.075586206	0.087565094
ENSGALG00000019061	MMP1	1	0.075706033	0.087695579
ENSGALG00000007817	EHF	5	0.075810752	0.08780854
ENSGALG00000007900	COMMD3	2	0.075861302	0.087858743
ENSGALG00000034411		31	0.076164425	0.088201427
ENSGALG00000000094	ADIPOR1	26	0.076398011	0.088463528
ENSGALG00000009373	CTSO	4	0.076581956	0.088668103
ENSGALG00000023440	GABPB1	10	0.076764452	0.088870961
ENSGALG00000054241		16	0.077039953	0.089181444
ENSGALG00000006992	MMP9	20	0.07751238	0.089719807
ENSGALG00000011324		7	0.077577789	0.089786994
ENSGALG00000010356	RAD54L	8	0.077613127	0.089819367
ENSGALG00000024378	ZNF414	28	0.077958598	0.090210609
ENSGALG00000026091	CACNG4	18	0.078200759	0.090482241
ENSGALG00000008907	PLCB4	3	0.078686772	0.091035944
ENSGALG00000004800	C1orf50	21	0.07876937	0.091122859
ENSGALG00000031411	KCNN1	28	0.078791849	0.091140216
ENSGALG00000005156		6	0.078948848	0.091313157
ENSGALG00000004002	LYPLA2	23	0.079052161	0.091423978
ENSGALG00000043198		7	0.079433108	0.091855832
ENSGALG00000012795	RIOK1	2	0.07946349	0.091882251
ENSGALG00000040340	PCYOX1L	13	0.079526851	0.091946795
ENSGALG00000012085		5	0.07998399	0.092466559
ENSGALG00000010154	GEMIN2	5	0.080188271	0.092693932
ENSGALG00000051458		33	0.080271336	0.092781154
ENSGALG00000054224		26	0.080335198	0.092846168
ENSGALG00000007185	COQ5	15	0.080373353	0.092881461
ENSGALG00000043532	SBSPON	2	0.080449747	0.092960933
ENSGALG00000036264	SCYL3	8	0.080521485	0.093035011
ENSGALG00000042415	BBS2	11	0.080569351	0.093081495
ENSGALG00000040325	EMC2	2	0.080708121	0.093232982
ENSGALG00000036356	YEATS2	9	0.080938446	0.093490193
ENSGALG00000007611	RPL35A	9	0.080951746	0.093496698

ENSGALG00000005084	TRIM37	19	0.081170859	0.093740886
ENSGALG00000020986		9	0.081234991	0.093806066
ENSGALG00000005357	COPB2	9	0.081278542	0.093847468
ENSGALG00000033038	STARD3NL	2	0.081311532	0.09387667
ENSGALG00000025876	THPO	9	0.08137424	0.093940173
ENSGALG00000017419	HPS1	6	0.08164931	0.094248797
ENSGALG00000050857	PTPN20	6	0.081710472	0.094310468
ENSGALG00000003958	PRMT3	5	0.081944988	0.094572195
ENSGALG00000054772	NAT8L	4	0.08234631	0.095026363
ENSGALG00000050178		3	0.082561899	0.095259446
ENSGALG00000002000	GRAMD2A	10	0.082563916	0.095259446
ENSGALG00000017246	ME3	1	0.082635649	0.095333188
ENSGALG00000052303		30	0.082925483	0.095658507
ENSGALG00000053077		33	0.082993139	0.095727494
ENSGALG00000040739	TXNL4A	2	0.083250562	0.096015333
ENSGALG00000048849		6	0.083295563	0.096058148
ENSGALG00000007659	HYOU1	24	0.083444745	0.096221087
ENSGALG00000002116	TEN1	18	0.083658501	0.096458449
ENSGALG00000016764	EIF5B	1	0.083736651	0.096539428
ENSGALG00000007731	DPCD	6	0.083850951	0.096662064
ENSGALG00000014121	COX5A	10	0.084034333	0.096864305
ENSGALG00000037642	SLC23A1	13	0.084067505	0.096893382
ENSGALG00000004640	MAD2L2	21	0.084300939	0.097153246
ENSGALG00000032405		16	0.084354418	0.097205691
ENSGALG00000022704		1	0.084534047	0.09740348
ENSGALG00000054968		1	0.084914017	0.097832053
ENSGALG00000032598	MYO1A	33	0.084954747	0.097869731
ENSGALG00000049157	DOK4	11	0.085154317	0.098090372
ENSGALG00000007418	CD3D	24	0.08522144	0.098158418
ENSGALG00000003128	EDEM2	20	0.085288577	0.098226468
ENSGALG00000008348	PDIA3	10	0.085815568	0.098824067
ENSGALG00000027961	PCASP2	28	0.086290786	0.099361937
ENSGALG00000041921	CWH43	4	0.086351663	0.099422647

ENSGALG00000000542	ZNF593	23	0.086490126	0.099572666
ENSGALG00000010774	SSBP3	8	0.086568229	0.099653173
ENSGALG00000053961		13	0.086621323	0.09970488
ENSGALG00000015234		2	0.086718634	0.099807467
ENSGALG00000012768	GCM2	2	0.086779707	0.099867596
ENSGALG00000051171		18	0.086787259	0.099867596
ENSGALG00000001956	OLFML3	26	0.086843474	0.099922854
ENSGALG00000004003	AIFM1	4	0.087069833	0.100173851
ENSGALG00000011859	GBE	1	0.087608432	0.100783999
ENSGALG00000033171	TGM4	2	0.087647856	0.10081984
ENSGALG00000004956	GPI	11	0.087727002	0.100901361
ENSGALG00000006173	XYLB	2	0.088191028	0.101425505
ENSGALG00000010338	CXCL13L2	4	0.088434113	0.101695477
ENSGALG00000023821	DHX58	27	0.08844384	0.101697071
ENSGALG00000032170		17	0.088594444	0.101860637
ENSGALG00000026077	ALDOC	19	0.088819376	0.102109623
ENSGALG00000052016	ADRA2B	22	0.088928912	0.10222591
ENSGALG00000050403	LYPD1	7	0.08917602	0.102500302
ENSGALG00000013156	ZNF830	2	0.089309274	0.102643791
ENSGALG00000002319	TEKT2	23	0.089452083	0.102798233
ENSGALG00000043641	JUND	28	0.089581265	0.102936986
ENSGALG00000010878	USO1	4	0.089622509	0.102974675
ENSGALG00000044226		30	0.089916459	0.103302684
ENSGALG00000003758	MREG	7	0.089990284	0.103377759
ENSGALG00000052770		10	0.090371759	0.103806204
ENSGALG00000004236	TRAF3IP1	7	0.090505118	0.103949596
ENSGALG00000015230	NME7	1	0.090605853	0.104055492
ENSGALG00000021473	FAM192A	11	0.091055879	0.104562472
ENSGALG00000034903		2	0.091092101	0.104589542
ENSGALG00000006738	CDK5RAP1	20	0.091096608	0.104589542
ENSGALG00000014066		1	0.091195469	0.104693188
ENSGALG00000041204	IGF2BP1	27	0.091355368	0.104866881
ENSGALG00000005649		6	0.091429122	0.104941663

ENSGALG00000025882	BUD13	24	0.091644436	0.105171982
ENSGALG00000017005	CAB39L	1	0.091647036	0.105171982
ENSGALG00000034510	VPS16	4	0.091671704	0.105190391
ENSGALG00000011186	ERMARD	3	0.091751574	0.105272132
ENSGALG00000010983	NPY	2	0.091821104	0.105341994
ENSGALG00000008593	ATP1B4	4	0.092451404	0.106055129
ENSGALG00000014966	HGD	1	0.092660736	0.106285264
ENSGALG00000009929	SOST	27	0.09277463	0.106405895
ENSGALG00000029301	BMP2	3	0.093075054	0.106740419
ENSGALG00000040434		27	0.093909808	0.107687603
ENSGALG00000015038	CHD1L	1	0.094074583	0.107866409
ENSGALG00000047771	PGF	5	0.094204459	0.107996926
ENSGALG00000041730	GPR20	2	0.094206127	0.107996926
ENSGALG00000030932	NKX2-3	6	0.094266503	0.108055981
ENSGALG00000012873	SERPINB5	2	0.09466929	0.108507487
ENSGALG00000007014	PYROXD2	6	0.095099277	0.108990082
ENSGALG00000005701	IP6K2	12	0.095289186	0.109197466
ENSGALG00000032723	ANKRD13B	19	0.095564116	0.109502233
ENSGALG00000010873	DHRS9	7	0.096183898	0.110202055
ENSGALG00000005614	SRRD	15	0.096256336	0.110274688
ENSGALG00000000719	RPL22	21	0.096921386	0.111026161
ENSGALG00000002845	CTNNA3	6	0.096975195	0.111077365
ENSGALG00000052460	CYB5R4	3	0.097333454	0.111477251
ENSGALG00000012756	PARPBP	1	0.097400449	0.111543503
ENSGALG00000002193		9	0.098095088	0.112328457
ENSGALG00000002044	EEF2KMT	14	0.098162136	0.112389062
ENSGALG00000009633	KATNBL1	5	0.098166449	0.112389062
ENSGALG00000041327	TRIM55	2	0.098275849	0.112503749
ENSGALG00000050309		1	0.098408852	0.112645431
ENSGALG00000021679	SF3A2	28	0.098585581	0.112837134
ENSGALG00000007157	TENT5D	4	0.09889791	0.113183989
ENSGALG00000012860	HSD11B1L	28	0.099093888	0.113397633
ENSGALG00000032838	BKJ	6	0.099395478	0.113732082

ENSGALG00000002033	NDUFB6	14	0.100081849	0.114506707
ENSGALG00000015960	RPIA	4	0.100272788	0.114714318
ENSGALG00000047005		2	0.100282122	0.114714318
ENSGALG00000026465	TMEM100	18	0.100340626	0.114770475
ENSGALG00000017179	PDGFD	1	0.100505184	0.114947914
ENSGALG00000002797	PGD	21	0.100794902	0.115268453
ENSGALG00000016967	ENOX1	1	0.101539898	0.116109535
ENSGALG00000043234	HBA1	14	0.102086919	0.1167241
ENSGALG00000001172	KBP	21	0.102611974	0.117313437
ENSGALG00000043399	EZH2	2	0.102673495	0.117372767
ENSGALG00000004804		20	0.103109238	0.117859842
ENSGALG00000037933	FSBP	2	0.103149412	0.117894712
ENSGALG00000053121		19	0.103162974	0.11789916
ENSGALG00000020590	SGF29	8	0.10324516	0.117982028
ENSGALG00000009110	LSM8	1	0.103327321	0.118064851
ENSGALG00000019030		1	0.103659692	0.118433529
ENSGALG00000029947	MMAB	15	0.103796982	0.118571276
ENSGALG00000032143	CRISPLD2	11	0.103799705	0.118571276
ENSGALG00000003046	PSMA4	10	0.104011495	0.118802076
ENSGALG00000006267	TPX2	20	0.104538712	0.11939308
ENSGALG00000024039		4	0.104588648	0.119438924
ENSGALG00000009526	VTI1B	5	0.104640238	0.119486647
ENSGALG00000009680	PAQR7	23	0.104847997	0.119712672
ENSGALG00000009421	SRSF5	5	0.104926497	0.119787147
ENSGALG00000001569	RHOC	26	0.104932872	0.119787147
ENSGALG00000003103	MST1R	12	0.105243849	0.120130898
ENSGALG00000029033		20	0.105279755	0.120160634
ENSGALG00000036452	CDK5RAP3	27	0.105298768	0.120171085
ENSGALG00000010867	TACSTD2	8	0.105331671	0.120197385
ENSGALG00000017033	MRPS31	1	0.105400288	0.120264431
ENSGALG00000003138	OXTR	12	0.105428596	0.120285475
ENSGALG00000032799		3	0.105618955	0.120491384
ENSGALG00000020754	ATP6V0E2	6	0.105710603	0.120584655

ENSGALG00000011318	DNPEP	7	0.106011601	0.120916693
ENSGALG00000006297	TRPC7	13	0.106126279	0.121036173
ENSGALG00000005293	GNG13	14	0.106208355	0.121118451
ENSGALG00000052348		22	0.106417516	0.121345625
ENSGALG00000016578	TRAPPC2	1	0.106897565	0.121881617
ENSGALG00000027269	GUCA2B	21	0.107068469	0.122065062
ENSGALG00000007168	CFAP20DC	12	0.107153725	0.122150838
ENSGALG00000040224	TIRAP	24	0.107283652	0.122287517
ENSGALG00000004394		7	0.107406262	0.12241583
ENSGALG00000001342	HCN2	28	0.107431172	0.122432777
ENSGALG00000002792	SPDEF	26	0.107710279	0.122739386
ENSGALG00000038549		3	0.108076235	0.123144896
ENSGALG00000041520	YJU2	28	0.108131617	0.123196487
ENSGALG00000000911	IL19	26	0.108407542	0.123499314
ENSGALG00000040794	CCNE2	2	0.108587132	0.123692349
ENSGALG00000045251		24	0.10914595	0.124317288
ENSGALG00000040882	HSPA4	13	0.109229496	0.124400826
ENSGALG00000040754	CYP1C1	5	0.109244388	0.124406167
ENSGALG00000053187		31	0.109430347	0.124606297
ENSGALG00000037735	CenpA	16	0.109647584	0.124842001
ENSGALG00000031895	SDK2	18	0.109696996	0.124886599
ENSGALG00000001234	CETP	11	0.109778333	0.12496753
ENSGALG00000014163	TTLL1	1	0.109806998	0.124988492
ENSGALG00000003682	ARL6IP4	15	0.110264135	0.125497115
ENSGALG00000017216		1	0.110360905	0.125595531
ENSGALG00000042730	STT3A	24	0.110965718	0.126272049
ENSGALG00000049136		33	0.111267509	0.126603652
ENSGALG00000032313	KDELR3	1	0.111434587	0.126781928
ENSGALG00000050845		12	0.111713664	0.127087583
ENSGALG00000012282	L2HGDH	5	0.111728681	0.127092808
ENSGALG00000053550	ESPNL	9	0.111907807	0.127284691
ENSGALG00000012416		1	0.111981254	0.12735635
ENSGALG00000029195	STK32A	13	0.112027117	0.127396627

ENSGALG00000008532	NELFB	17	0.112081321	0.127446381
ENSGALG00000014750		1	0.112102418	0.127458484
ENSGALG00000003751		6	0.11228065	0.127649227
ENSGALG00000049689		2	0.112409294	0.127783565
ENSGALG00000004063	SLC25A14	4	0.112459201	0.127828381
ENSGALG00000008349	LMCD1	12	0.112892073	0.128308449
ENSGALG00000001573	P2RX1	19	0.113002445	0.128421923
ENSGALG00000002329	CCL1	19	0.113406647	0.128869269
ENSGALG00000029673	TBC1D19	4	0.113773633	0.129274244
ENSGALG00000004489		11	0.113824083	0.129319516
ENSGALG00000003079	MFF	9	0.11411923	0.129642763
ENSGALG00000004946		9	0.114314017	0.129851948
ENSGALG00000026978	RPL29	12	0.114553519	0.130111882
ENSGALG00000052935	TNFRSF9	21	0.11470985	0.13027731
ENSGALG00000012150	TAB1	1	0.114808825	0.130377574
ENSGALG00000003123	CNTNAP1	27	0.114823674	0.130382292
ENSGALG00000029764		4	0.114927223	0.13048772
ENSGALG00000039786	FGFR1	22	0.115149686	0.130728131
ENSGALG00000043302	LAT2	19	0.115484292	0.131095798
ENSGALG00000003841		4	0.115514988	0.131118437
ENSGALG00000039880	RHEB	2	0.11560413	0.131207405
ENSGALG00000007886		5	0.115800987	0.131418599
ENSGALG00000021525	ISLR	10	0.115824142	0.131432645
ENSGALG00000005083	SLC5A10	14	0.115927864	0.131538102
ENSGALG00000043336	COPZ1	33	0.116169635	0.131800164
ENSGALG00000041618		2	0.116231516	0.131858101
ENSGALG00000010399	SELENOT	9	0.11643072	0.132071799
ENSGALG00000013948	RASL11B	4	0.117087703	0.132804685
ENSGALG00000028318	CDKN1A	26	0.117162003	0.132876597
ENSGALG00000029152	IMP4	9	0.117229186	0.132940426
ENSGALG00000050014		24	0.117557796	0.133300679
ENSGALG00000048129	TEX12	24	0.117587584	0.133322058
ENSGALG00000053446		11	0.117818966	0.13357198

ENSGALG00000054746		18	0.11801066	0.133770299
ENSGALG00000009803	MGST2	4	0.118015837	0.133770299
ENSGALG00000004553	SGPL1	6	0.118124546	0.133881073
ENSGALG00000021592	TLCD2	19	0.118325066	0.134095876
ENSGALG00000053412		31	0.118466224	0.13424337
ENSGALG00000039307	GNB5	10	0.11855687	0.134333604
ENSGALG00000041522	CLCF1	5	0.118579768	0.134347064
ENSGALG00000028015	HES1	9	0.119032898	0.134847917
ENSGALG00000044224		7	0.119310435	0.135149771
ENSGALG00000028409	SNRPGP15	22	0.119436764	0.135280303
ENSGALG00000029614	FAM174C	28	0.119767795	0.135642646
ENSGALG00000031597	HBAD	14	0.120167795	0.136083024
ENSGALG00000006344	GSTT1	15	0.120315194	0.136237292
ENSGALG00000041273	KIAA0319	2	0.120575462	0.136519325
ENSGALG00000045676	NELFE	26	0.120594763	0.1365285
ENSGALG00000009722	DYNC1I1	2	0.120651696	0.136580274
ENSGALG00000001883	ST6GALNAC2	18	0.120727167	0.136653022
ENSGALG00000044362	PP2D1	2	0.121305589	0.137282593
ENSGALG00000001583	TRAF1	17	0.121305885	0.137282593
ENSGALG00000008411	GHRL	12	0.121452538	0.137435804
ENSGALG00000041834	AEBP1	22	0.12146987	0.137437016
ENSGALG00000030032	SLC4A10	7	0.121476152	0.137437016
ENSGALG00000008121	CYP17A1	6	0.121736199	0.137718452
ENSGALG00000003073	RHOBTB1	6	0.121797845	0.137775408
ENSGALG00000008421	PEX16	5	0.121909284	0.137888673
ENSGALG00000001723		8	0.121954522	0.137927046
ENSGALG00000024298	ADAMTSL5	28	0.122082673	0.138059176
ENSGALG00000000585	MYL4	27	0.122310151	0.138303596
ENSGALG00000011685	CSTF3	5	0.122355688	0.138342257
ENSGALG00000001921		17	0.12237916	0.138355966
ENSGALG00000001975	TMEM120A	19	0.122574572	0.138564042
ENSGALG00000015800	ORC3	3	0.122888775	0.138906353
ENSGALG00000046714		1	0.122984351	0.138998093

ENSGALG00000050564		6	0.122992736	0.138998093
ENSGALG00000014616	MT3	11	0.123107069	0.13911441
ENSGALG00000013006	ROPN1L	2	0.123305647	0.139325897
ENSGALG00000005365	NARFL	14	0.123634882	0.139684962
ENSGALG00000002511		26	0.123881859	0.139951034
ENSGALG00000014695	CLCN1	1	0.12409174	0.140175151
ENSGALG00000038994	KPTN	32	0.124901174	0.141076426
ENSGALG00000054976	IL11	22	0.124984145	0.141157066
ENSGALG00000000866	SELENBP1	25	0.125173063	0.14135055
ENSGALG00000033530		27	0.125178646	0.14135055
ENSGALG00000001559	ZP3	10	0.125194901	0.141355813
ENSGALG00000012254	KCNJ4	1	0.125347487	0.141512317
ENSGALG00000002728	SLC16A3	18	0.125356724	0.141512317
ENSGALG00000050870	NPM2	22	0.125609816	0.1417849
ENSGALG00000051085		3	0.12584873	0.14204143
ENSGALG00000037050	FABP3	23	0.125898793	0.142084782
ENSGALG00000002556	NGF	26	0.126332474	0.142561023
ENSGALG00000016186	PDE9A	1	0.126825051	0.143103632
ENSGALG00000016094	SFXN5	4	0.126869936	0.143141032
ENSGALG00000030460	TBC1D31	2	0.126973806	0.143244968
ENSGALG00000010170		4	0.127285215	0.143582998
ENSGALG00000001701		21	0.127350579	0.143643442
ENSGALG00000037257	HLX	3	0.12765862	0.143977574
ENSGALG00000007728		15	0.128477074	0.144887251
ENSGALG00000012194		5	0.129814766	0.146382266
ENSGALG00000033411		13	0.130066719	0.146652811
ENSGALG00000040709		1	0.131060136	0.147759246
ENSGALG00000033061	CAMK2B	22	0.13162243	0.148379465
ENSGALG00000002637	RBP7	21	0.131816643	0.148584666
ENSGALG00000004545	WDR72	10	0.131977186	0.148739229
ENSGALG00000043545	PNPT1	3	0.131978162	0.148739229
ENSGALG00000054145		10	0.132239712	0.149020223
ENSGALG00000052368		1	0.132618906	0.149433724

ENSGALG00000007636	PCK1	20	0.133021502	0.149873514
ENSGALG00000036950	CASP14	22	0.133217855	0.150080875
ENSGALG00000005038	CENPI	4	0.133634204	0.150536017
ENSGALG00000050931		2	0.133701514	0.150597927
ENSGALG00000042744	RGS2	8	0.133864473	0.150767552
ENSGALG00000008998	FAM179A	3	0.134096014	0.151014381
ENSGALG00000017167	SLC35F2	1	0.134346763	0.151282793
ENSGALG00000052975	CCDC113	11	0.134369729	0.151294681
ENSGALG00000035016	ID2	3	0.134777352	0.151739635
ENSGALG00000005383	BRCC3	4	0.135113044	0.152103531
ENSGALG00000045305		1	0.135525548	0.152553823
ENSGALG00000006077	C5H11orf58	5	0.135641985	0.152670795
ENSGALG00000004986	CERCAM	17	0.136124116	0.153199312
ENSGALG00000005543		9	0.136906666	0.154065803
ENSGALG00000030118	PCGF1	4	0.137040319	0.154201975
ENSGALG00000044466		11	0.137185933	0.154351581
ENSGALG00000002800	GRK7	9	0.137440479	0.154623709
ENSGALG00000032875	ATP5F1D	28	0.137495867	0.154667149
ENSGALG00000008659	HACD1	2	0.137504461	0.154667149
ENSGALG00000000893	TAT	11	0.137676154	0.154845987
ENSGALG00000005017	DUSP13	6	0.137858286	0.155024098
ENSGALG00000043920	OTUD3	21	0.137859944	0.155024098
ENSGALG00000028172	WDR90	14	0.137998982	0.155166137
ENSGALG00000037390	GPR83L	4	0.13821971	0.155399993
ENSGALG00000047321		17	0.138582363	0.155793358
ENSGALG00000021238		14	0.13892258	0.156161429
ENSGALG00000011885	ANXA5	4	0.139058441	0.156299739
ENSGALG00000013506	MED24	27	0.139107744	0.156340743
ENSGALG00000036892	BUB1B	5	0.13924621	0.156481939
ENSGALG00000039376	DPT	1	0.139455146	0.156702294
ENSGALG00000035761		2	0.139585337	0.156834133
ENSGALG00000014264	NSUN7	4	0.140144276	0.157447632
ENSGALG00000047646	CCNT1	33	0.140580878	0.15792359

ENSGALG00000047967		4	0.140838063	0.158197928
ENSGALG00000023683	TCTN2	15	0.141410228	0.158825986
ENSGALG00000053332		14	0.14175114	0.15919422
ENSGALG00000011239	OXNAD1	2	0.142382512	0.159888559
ENSGALG00000010117	SLC25A21	5	0.142671006	0.160197769
ENSGALG00000015000	LYAR	4	0.142884959	0.160423233
ENSGALG00000040621	IL18R1	1	0.142935857	0.160453444
ENSGALG00000045908		2	0.142938187	0.160453444
ENSGALG00000037723		10	0.143338105	0.160887555
ENSGALG00000033690	RSL1D1	14	0.14335409	0.160890686
ENSGALG00000016400	RSAD2	3	0.143544083	0.161089094
ENSGALG00000037727	OSGEPL1	7	0.14368202	0.161229052
ENSGALG00000006375	TM4SF19	9	0.143696073	0.161229983
ENSGALG00000003419		15	0.143919211	0.16146549
ENSGALG00000026276	TCF15	20	0.144261104	0.16182822
ENSGALG00000032270	NCAPH	22	0.144269068	0.16182822
ENSGALG00000030407	IL6R	25	0.144464613	0.162032658
ENSGALG00000046353		2	0.144494284	0.162045225
ENSGALG00000004926	ATPAF2	14	0.144502397	0.162045225
ENSGALG00000030059	NTAQ1	2	0.144723693	0.162278462
ENSGALG00000006785	IRF1	13	0.144862863	0.162419576
ENSGALG00000001550	RBM39	20	0.144949701	0.162501995
ENSGALG00000050091		16	0.145552429	0.163162708
ENSGALG00000016910	RBM26	1	0.145581974	0.163180824
ENSGALG00000045153	SHISA3	4	0.145653217	0.163245672
ENSGALG00000027494	SPTSSB	9	0.145708618	0.163292755
ENSGALG00000010627	PRPF38A	8	0.146249298	0.163883622
ENSGALG00000047143		8	0.146409007	0.164047511
ENSGALG00000011505	MARK3	5	0.146561578	0.164203374
ENSGALG00000031067	TMEM132A	5	0.146577156	0.164205739
ENSGALG00000053511	APOLD1	1	0.146659931	0.164276882
ENSGALG00000048246		21	0.146667607	0.164276882
ENSGALG00000013606	FGL1	4	0.146719048	0.164319404

ENSGALG00000029582	EVI5	8	0.146869326	0.164468895
ENSGALG00000006834	CPB1	9	0.146879505	0.164468895
ENSGALG00000001616	CDC45	15	0.147553402	0.165208322
ENSGALG00000051033		1	0.147750926	0.165414291
ENSGALG00000001663	MAK16	22	0.148023695	0.165704454
ENSGALG00000031248	RMDN1	2	0.148183146	0.165867722
ENSGALG00000009854	NEK2	3	0.148784152	0.166525167
ENSGALG00000016509	IFT172	3	0.148926319	0.166668986
ENSGALG00000014252		1	0.148953248	0.166683825
ENSGALG00000040759	CPXM1	4	0.149182194	0.166924703
ENSGALG00000033908	SLC10A4	4	0.149201934	0.166931472
ENSGALG00000050045		8	0.14987305	0.167666951
ENSGALG00000005685		19	0.149911805	0.167694921
ENSGALG00000041533	SLC11A2	33	0.150327031	0.168140936
ENSGALG00000043727	NR4A1	33	0.150338102	0.168140936
ENSGALG00000015044	GTF3C6	3	0.150704107	0.168534823
ENSGALG00000006320		15	0.150725453	0.168543237
ENSGALG00000026983	CHRD	9	0.150955499	0.168784998
ENSGALG00000016580	SCARA5	3	0.151064544	0.168891435
ENSGALG00000045534		10	0.151285277	0.169122709
ENSGALG00000042045	IRX3	11	0.151751865	0.169628757
ENSGALG00000003851	TRIT1	23	0.151893104	0.169771071
ENSGALG00000048923	CBARP	28	0.1521319	0.170022388
ENSGALG00000048310		7	0.152155029	0.170032653
ENSGALG00000010152	TSPAN8	1	0.15224975	0.17012291
ENSGALG00000003642	PDGFA	14	0.152354183	0.170224004
ENSGALG00000024466	CCL17	11	0.152631681	0.170518424
ENSGALG00000035050		2	0.152751294	0.170636421
ENSGALG00000004869	ISCU	15	0.15278164	0.170654684
ENSGALG00000054174		28	0.153114237	0.171010524
ENSGALG00000038454		1	0.153130969	0.171013547
ENSGALG00000016173	UBASH3A	1	0.153320941	0.171208108
ENSGALG00000004290	ANGPTL1	8	0.153333268	0.171208108

ENSGALG00000036894	ABCA2	17	0.153561052	0.171446746
ENSGALG00000051361		25	0.153787054	0.17168335
ENSGALG00000041897		28	0.154310809	0.172252284
ENSGALG00000000129	TCF25	11	0.154798411	0.17278076
ENSGALG00000043448	ABLIM2	4	0.154850628	0.172823223
ENSGALG00000006974	DDX27	20	0.155143427	0.173134157
ENSGALG00000052876		16	0.155354188	0.173353492
ENSGALG00000007814		9	0.155478285	0.173476091
ENSGALG00000032606		33	0.155503924	0.173488823
ENSGALG00000019768	ACSF2	18	0.155595671	0.173575298
ENSGALG00000031164	WFDC2	20	0.155803334	0.173791058
ENSGALG00000039315	MARS	33	0.156265321	0.174290437
ENSGALG00000024296		24	0.156589592	0.174636138
ENSGALG00000000402	LOXL2	22	0.156981474	0.175057171
ENSGALG00000011747		1	0.157000965	0.175062895
ENSGALG00000041432	STUB1	14	0.157572653	0.175684286
ENSGALG00000052649	HIST1H2B5	1	0.157660691	0.175766371
ENSGALG00000028721	LMOD1	26	0.157696599	0.17579033
ENSGALG00000003010	NUP93	11	0.157992365	0.176103932
ENSGALG00000026019	ASCC1	6	0.158298732	0.176429291
ENSGALG00000010582	PPA2	4	0.158392264	0.176517401
ENSGALG00000016797	FHL2	1	0.158873897	0.177037966
ENSGALG00000014568	EMG1	1	0.159242649	0.177432662
ENSGALG00000039779	NUDT16L1	14	0.159866211	0.178111176
ENSGALG00000035337	AARS2	3	0.160040217	0.17828875
ENSGALG00000054289	GJB5	23	0.160200666	0.178451189
ENSGALG00000051488	SFI1	15	0.160215601	0.178451523
ENSGALG00000003492	PPP1R3E	22	0.16024767	0.178470939
ENSGALG00000040247	AIP	5	0.160685637	0.178942366
ENSGALG00000033162	PSMD3	27	0.160719845	0.178964116
ENSGALG00000007981	CLN6	10	0.160931627	0.179183575
ENSGALG00000017336		1	0.161196586	0.179462197
ENSGALG00000047209		1	0.161746063	0.180057495

ENSGALG00000052002	RNASEH2A	30	0.16223089	0.180580724
ENSGALG00000018439	DLX1	7	0.162309463	0.180651691
ENSGALG00000025804	CLCN2	9	0.162364277	0.180696206
ENSGALG00000016283	BEND6	3	0.16253104	0.180865289
ENSGALG00000043662	NDRG4	11	0.163170554	0.181560372
ENSGALG00000048343		11	0.163188411	0.181563674
ENSGALG00000005889	CCDC18	8	0.163248326	0.181613762
ENSGALG00000032098	ARTN	8	0.163388216	0.181752807
ENSGALG00000008407	IRAK2	12	0.163596451	0.181967846
ENSGALG00000010464	GLRX3	6	0.164168267	0.182587219
ENSGALG00000029150	AK1	17	0.164886623	0.183369445
ENSGALG00000008702	OVOL2	3	0.166586664	0.185243155
ENSGALG00000040241	NTRK3	10	0.167471458	0.186210057
ENSGALG00000004622	SPOUT1	17	0.167534279	0.186262922
ENSGALG00000039742	CYP21A1	16	0.167798864	0.186540075
ENSGALG00000045045		10	0.16812855	0.186889544
ENSGALG00000038413	SURF6	17	0.168282519	0.187043642
ENSGALG00000046650		4	0.168531786	0.187303625
ENSGALG00000046687	EPS8L3	26	0.168649096	0.187416918
ENSGALG00000006638	GRIK4	24	0.168751244	0.187513343
ENSGALG00000003011	TOM1L1	18	0.168825346	0.18757859
ENSGALG00000016477	WDR35	3	0.169193943	0.187971001
ENSGALG00000009254	ENO4	6	0.169672143	0.188485099
ENSGALG00000051812		25	0.169951275	0.188777981
ENSGALG00000000516	FANCA	11	0.17022413	0.189063838
ENSGALG00000004047	GALE	23	0.170465512	0.189314691
ENSGALG00000054572		12	0.17066718	0.189521396
ENSGALG00000000137	SNRPE	26	0.170836968	0.189692665
ENSGALG00000049966		25	0.171813471	0.190759575
ENSGALG00000043605	BEST4	8	0.172051193	0.191006118
ENSGALG00000014652	KIAA0907	25	0.172148999	0.1910973
ENSGALG00000019976		3	0.173774576	0.192884243
ENSGALG00000010229	ABCD4	5	0.174273201	0.193420093

ENSGALG00000032614	AT2	1	0.174718088	0.193896209
ENSGALG00000010275	FCF1	5	0.175309987	0.194535373
ENSGALG00000009454	ZNF277	1	0.175408252	0.194626701
ENSGALG00000053706	C14orf2	5	0.176064474	0.195337045
ENSGALG00000005790		20	0.176227868	0.195500537
ENSGALG00000036711	S100A11	25	0.176583387	0.195877114
ENSGALG00000010167	PNN	5	0.176923703	0.196236761
ENSGALG00000001963		19	0.177087788	0.196395975
ENSGALG00000000684	KLHDC8A	26	0.177099463	0.196395975
ENSGALG00000002771	CRIP1	8	0.177890689	0.197255472
ENSGALG00000054836		1	0.178288154	0.197678227
ENSGALG00000047364		30	0.179111684	0.198573265
ENSGALG00000009975	CCT2	1	0.179825982	0.199347049
ENSGALG00000003522	RBBP4	23	0.179939371	0.199454614
ENSGALG00000032108	TMEM136-1	24	0.179965012	0.199464903
ENSGALG00000004687	CENPP	12	0.180029604	0.199518356
ENSGALG00000003540	CEP95	18	0.180607311	0.200140411
ENSGALG00000005360	CA4	19	0.180792893	0.200327858
ENSGALG00000012236	DMC1	1	0.181796164	0.20142123
ENSGALG00000007887	AAGAB	10	0.182071668	0.201708146
ENSGALG00000001078		21	0.182502357	0.202166915
ENSGALG00000001992	PKLR	10	0.182625154	0.202284566
ENSGALG00000004689	PKN1	17	0.182763308	0.202419204
ENSGALG00000040857	TECTA	24	0.183060733	0.202730201
ENSGALG00000015908	COL12A1	3	0.183566975	0.203272375
ENSGALG00000050350	HIST1H2B7	1	0.184031539	0.203768303
ENSGALG00000001844	ALKBH4	19	0.184426347	0.204186911
ENSGALG00000036074	RPP40	2	0.184463167	0.204209133
ENSGALG00000039985	CDH11	11	0.184582003	0.204310231
ENSGALG00000004446	NPFFR1	6	0.184588001	0.204310231
ENSGALG00000010633	GPX7	8	0.184637435	0.204346396
ENSGALG00000009050	CAPN3	5	0.184675405	0.204369869
ENSGALG00000030844	DMTF1	1	0.185381561	0.205132714

ENSGALG00000002546	COL5A1	17	0.185917435	0.205707013
ENSGALG00000053130	MYOZ3	13	0.185938147	0.205711263
ENSGALG00000007095	CUL2	2	0.18611371	0.205886815
ENSGALG00000047448	PCSK9	8	0.186272385	0.206043653
ENSGALG00000007525		5	0.186567245	0.206351091
ENSGALG00000007152	FAM3D	12	0.187214502	0.207048202
ENSGALG00000053509	DDX11	1	0.18745944	0.207300285
ENSGALG00000029760	NRBP2	2	0.188044056	0.20792792
ENSGALG00000014432	RPS20	2	0.188101995	0.207973126
ENSGALG00000017170	ALKBH8	1	0.188294769	0.208167388
ENSGALG00000026146	CRLF1	28	0.189406674	0.209377658
ENSGALG00000047463		8	0.189530722	0.209495793
ENSGALG00000006895	CEP131	18	0.189582212	0.209533713
ENSGALG00000014581	BORCS8	28	0.189816772	0.209773942
ENSGALG00000030878	RPLP1	2	0.190112256	0.210081452
ENSGALG00000028041		4	0.190192079	0.210150613
ENSGALG00000013590		4	0.190255198	0.210201307
ENSGALG00000017169	ELMOD1	1	0.19059763	0.21056056
ENSGALG00000002203	FGF18	13	0.190979911	0.210945792
ENSGALG00000032060	GPR153	21	0.19098094	0.210945792
ENSGALG00000012836		10	0.191049101	0.211001964
ENSGALG00000009149	FKBP7	7	0.19145371	0.211429679
ENSGALG00000037067	EIF3E	2	0.191948717	0.211953856
ENSGALG00000010140	EIF2B3	8	0.191963129	0.211953856
ENSGALG00000026241	GRIN3B	28	0.192344037	0.212355202
ENSGALG00000034278	SVOP	15	0.19244757	0.21245027
ENSGALG00000029066		23	0.192779748	0.212797707
ENSGALG00000026809	SARS	26	0.192821729	0.21282478
ENSGALG00000016884	SLC15A1	1	0.193263337	0.213292892
ENSGALG00000011816	ENGASE	18	0.193288662	0.213301535
ENSGALG00000016760		1	0.193313941	0.213310125
ENSGALG00000003466		1	0.193431289	0.213420297
ENSGALG00000000452	TNFRSF10B	22	0.194093005	0.214131018

ENSGALG00000042521	MPP3	27	0.194210553	0.214241317
ENSGALG00000012715	CAP2	2	0.194241244	0.214255789
ENSGALG00000015485	ACP6	1	0.194603507	0.214635962
ENSGALG00000043307	HIBCH	7	0.194675225	0.214695642
ENSGALG00000008776	HYAL6	1	0.194747136	0.214755524
ENSGALG00000009565	EIF2S1	5	0.194764899	0.214755689
ENSGALG00000034115	DUS1L	18	0.195036164	0.215035352
ENSGALG00000007833		24	0.195711659	0.215760602
ENSGALG00000037195	TALDO1	5	0.197438292	0.217644435
ENSGALG00000019967	MTFR2	3	0.197648571	0.217856538
ENSGALG00000049692		4	0.197866229	0.218076736
ENSGALG00000004009		19	0.198438594	0.218687796
ENSGALG00000008810	FAM161A	3	0.198739764	0.218999904
ENSGALG00000043930		2	0.199889664	0.220247123
ENSGALG00000041514		17	0.202081588	0.222642162
ENSGALG00000015864	IBTK	3	0.202293446	0.222837163
ENSGALG00000053383		3	0.202295133	0.222837163
ENSGALG00000019036	SPCS2	1	0.203133153	0.223740066
ENSGALG00000004521	GPX3	13	0.203197989	0.223791264
ENSGALG00000042123	CLDN23	4	0.203225729	0.2238016
ENSGALG00000001602	NUTF2	11	0.203275946	0.223826306
ENSGALG00000002390	CA6	21	0.203284877	0.223826306
ENSGALG00000040805	RNF212	4	0.203324193	0.223847399
ENSGALG00000009212	MND1	4	0.203348521	0.223847399
ENSGALG00000003687	OGFOD2	15	0.203359111	0.223847399
ENSGALG00000033598	HIRA	15	0.203569791	0.224059077
ENSGALG00000005908	TMEM9B	5	0.204091032	0.224612506
ENSGALG00000010825	AGR2	2	0.204488668	0.225029814
ENSGALG00000031789		22	0.204858821	0.225416804
ENSGALG00000005164	MTG2	20	0.205022232	0.225576256
ENSGALG00000000769	RAB7B	26	0.20630584	0.226968069
ENSGALG00000008700	PDCD4	6	0.207580197	0.228329809
ENSGALG00000042374	PDE11A	7	0.207581066	0.228329809

ENSGALG00000029863	PHYHIPL	6	0.207688611	0.228427497
ENSGALG00000041401	SDHB	21	0.208716226	0.229537021
ENSGALG00000030767		2	0.20875945	0.22956385
ENSGALG00000012886		2	0.209007329	0.229815705
ENSGALG00000007335	KBTBD13	10	0.209521838	0.230360665
ENSGALG00000007470	POLR1D	4	0.210030175	0.23089874
ENSGALG00000012128	IMMP1L	5	0.210807696	0.231732622
ENSGALG00000043370	TAF6L	33	0.211504029	0.232477114
ENSGALG00000037812	USP43	18	0.211650008	0.232616599
ENSGALG00000051549		19	0.211936808	0.232910816
ENSGALG00000052760	ATOH7	6	0.212285982	0.23327352
ENSGALG00000006821	TNMD	4	0.213953096	0.235084266
ENSGALG00000034326	TMEM158	2	0.214073816	0.235195715
ENSGALG00000039855	SNX29	14	0.214205035	0.235318677
ENSGALG00000009368	RGS6	5	0.214284297	0.235384544
ENSGALG00000003219	TMEM231	11	0.214682066	0.235778161
ENSGALG00000054379	TSPAN32	5	0.214689168	0.235778161
ENSGALG00000021047	KIFAP3	8	0.214700642	0.235778161
ENSGALG00000051275		20	0.215002214	0.236088075
ENSGALG00000050830	SV2A	25	0.215384587	0.236486652
ENSGALG00000028815		24	0.215437739	0.236523713
ENSGALG00000035913	PPP1R27	18	0.215973891	0.237090993
ENSGALG00000015773	PTCD3	4	0.216681901	0.237833675
ENSGALG00000007003		17	0.216689436	0.237833675
ENSGALG00000006088	DCTN5	14	0.217259939	0.238438384
ENSGALG00000016980	COG3	1	0.217340156	0.238504952
ENSGALG00000005886	WSCD1	19	0.217362601	0.238508117
ENSGALG00000000251	CD79B	27	0.217497637	0.238614687
ENSGALG00000011484	CMTM8	2	0.217498862	0.238614687
ENSGALG00000001655	SPCS1	12	0.217572494	0.238673991
ENSGALG00000001916	SLIT3	13	0.217781019	0.238862012
ENSGALG00000037029		28	0.217783071	0.238862012
ENSGALG00000001108	MIER2	28	0.217809399	0.238869401

ENSGALG00000016931	BORA	1	0.217983497	0.239038833
ENSGALG00000007594	ASB12	4	0.218095016	0.239131276
ENSGALG00000053043	CARHSP1	14	0.218107022	0.239131276
ENSGALG00000007386	LUC7L3	18	0.218505864	0.239547024
ENSGALG00000002437	DIPK1B	17	0.218560729	0.239585631
ENSGALG00000036412	TMEM108	2	0.218958725	0.240000335
ENSGALG00000005099	ORM1	17	0.21938101	0.240441587
ENSGALG00000006054	CALCA	5	0.219838933	0.240921815
ENSGALG00000033510	ERBB3	33	0.219912669	0.240980963
ENSGALG00000003523	IFT52	20	0.220822814	0.241956557
ENSGALG00000039648		5	0.221560309	0.24274282
ENSGALG00000028363	SPG7	11	0.221691827	0.242865089
ENSGALG00000004722	ASPN	12	0.221818775	0.242982331
ENSGALG00000016478	MATN3	3	0.222093996	0.243261956
ENSGALG00000036283	RBM8A	25	0.222410354	0.243586584
ENSGALG00000006342	UEVLD	5	0.222788747	0.243979091
ENSGALG00000032986	CFTR	1	0.222816397	0.243987457
ENSGALG00000001258	NUMA1	1	0.222870733	0.24402504
ENSGALG00000009699	CCNL1	9	0.223196864	0.244360184
ENSGALG00000044675	MATN2	2	0.223930861	0.245133724
ENSGALG00000001709	MUSTN1	12	0.223943619	0.245133724
ENSGALG00000011690	CLBA1	5	0.224283854	0.245484114
ENSGALG00000037510	WBSCR22	19	0.225210045	0.246475727
ENSGALG00000038885	FEZ1	24	0.225388357	0.246648738
ENSGALG00000043064	EXFABP	17	0.225953934	0.247245474
ENSGALG00000010401	EIF2A	9	0.226010783	0.247285488
ENSGALG00000038266	CTXN2	10	0.226339522	0.247622952
ENSGALG00000016462	MYCN	3	0.227228011	0.248572684
ENSGALG00000028681		4	0.228181364	0.249593197
ENSGALG00000027540		28	0.228480136	0.249897586
ENSGALG00000040761	DRD4	5	0.228809852	0.250235764
ENSGALG00000043209	ADGRB2	23	0.229192505	0.250624215
ENSGALG00000030344	ISYNA1	28	0.229206153	0.250624215

ENSGALG00000031529	ERBB2	27	0.229695263	0.251136509
ENSGALG00000015395	CD200L	1	0.229875258	0.251310771
ENSGALG00000007378	FAM162A	1	0.229907567	0.251323558
ENSGALG00000006008	HOMER2	10	0.230461854	0.251906892
ENSGALG00000000558	SLC1A6	28	0.231605974	0.253134783
ENSGALG00000005291		14	0.231668288	0.253180195
ENSGALG00000004103	FAM81A	10	0.232392115	0.253948473
ENSGALG00000004070	MICALL2	14	0.232856539	0.254433173
ENSGALG00000049426	WBP1	4	0.23325936	0.254850482
ENSGALG00000038301	NIPSNAP1	15	0.234208905	0.255864992
ENSGALG00000010785	ACOT11	8	0.234263177	0.255901355
ENSGALG00000010417	VDHAP	8	0.234773078	0.256435379
ENSGALG00000011564	RUFY3	4	0.235408988	0.257106933
ENSGALG00000002984	RAB24	13	0.23629956	0.258056474
ENSGALG00000030499	MOB2	5	0.236429217	0.258174945
ENSGALG00000032656	TCF7L1	22	0.236939524	0.258709019
ENSGALG00000006144	CDK10	11	0.237029929	0.258784556
ENSGALG00000051469		30	0.23715313	0.258895883
ENSGALG00000016558	VEGFD	1	0.2384741	0.260314655
ENSGALG00000046995	NECTIN4	25	0.238542161	0.260365641
ENSGALG00000005347	ADAMTS18	11	0.238789113	0.260611856
ENSGALG00000033792		19	0.239017051	0.260837278
ENSGALG00000026039	THEM4	25	0.239892313	0.261749116
ENSGALG00000014164	BIK	1	0.239895544	0.261749116
ENSGALG00000004249	GRHL3	23	0.240030772	0.261873229
ENSGALG00000043100	SRPX	1	0.240248467	0.262087283
ENSGALG00000007124	POP5	15	0.240395506	0.262224227
ENSGALG00000034988	RDH5	33	0.240982698	0.262841223
ENSGALG00000035185		19	0.242327418	0.264284276
ENSGALG00000006644		13	0.242443062	0.26438675
ENSGALG00000008739	MINDY3	2	0.242477801	0.264400986
ENSGALG00000005973	LCMT1	14	0.242591965	0.264501818
ENSGALG00000013090	LOXL4	6	0.243679861	0.265664212

ENSGALG00000010903	RASGEF1B	4	0.243795933	0.265766992
ENSGALG00000050292		31	0.243976724	0.2659403
ENSGALG00000010157	TRAPPC6A	5	0.244104795	0.266056116
ENSGALG00000035206		33	0.244324537	0.266271817
ENSGALG00000002239	REXO5	14	0.245075358	0.267065012
ENSGALG00000013886	LDLRAD4	2	0.24509616	0.267065012
ENSGALG00000022206		5	0.245710092	0.267710048
ENSGALG00000028147	CTSK	25	0.245862865	0.267852565
ENSGALG00000043909		6	0.246122735	0.268111722
ENSGALG00000027571		1	0.246219564	0.268176517
ENSGALG00000007102	MLEC	15	0.246226205	0.268176517
ENSGALG00000034013		6	0.247261634	0.269280199
ENSGALG00000010243	PRDX1	8	0.247492037	0.269507048
ENSGALG00000001084	ATP8B3	28	0.247826265	0.269846906
ENSGALG00000008330	TMEM60	1	0.247936743	0.269943094
ENSGALG00000002952	NRG4	10	0.248129932	0.270129309
ENSGALG00000008100	RAPSN	5	0.248523026	0.270533101
ENSGALG00000003134		13	0.248625757	0.270620769
ENSGALG00000006779	PDZD3	24	0.248692489	0.270669242
ENSGALG00000035869	ATAD3A	21	0.248799734	0.270761796
ENSGALG00000008045	SLC9A8	20	0.24901385	0.270957904
ENSGALG00000021688	CD320	28	0.249024381	0.270957904
ENSGALG00000017681	POLR3K	14	0.2490846	0.270999244
ENSGALG00000004999	TAF7	4	0.249435895	0.271357234
ENSGALG00000048601		4	0.249863476	0.271798141
ENSGALG00000007512	MORC2	15	0.250007859	0.271930939
ENSGALG00000006203		20	0.250283786	0.27220678
ENSGALG00000012872	SERPINB11	2	0.250493916	0.272411017
ENSGALG00000001790	METTL23	18	0.25080283	0.272722635
ENSGALG00000010624	MEMO1	3	0.251977337	0.273975359
ENSGALG00000010413	NGB	5	0.252051856	0.274031946
ENSGALG00000007839	NCAM1	24	0.252074868	0.27403253
ENSGALG00000048179		4	0.253442704	0.275494953

ENSGALG00000038812	MRPL27	18	0.253847719	0.275910611
ENSGALG00000002016	PRKCD	12	0.253883671	0.275925092
ENSGALG00000010269	KCNAB1	9	0.25418949	0.276232839
ENSGALG00000012312	GCAT	1	0.254460551	0.276502762
ENSGALG00000016575	GPM6B	1	0.254563764	0.276582265
ENSGALG00000044449	LAG3	1	0.254579083	0.276582265
ENSGALG00000020895		20	0.255297116	0.277337646
ENSGALG00000012985	SLC6A13	1	0.256039491	0.278119333
ENSGALG00000009154	IDS	4	0.256115953	0.278177607
ENSGALG00000001531	FN3K	18	0.256251667	0.27830022
ENSGALG00000054856		4	0.256502819	0.278543418
ENSGALG00000049911		18	0.256521286	0.278543418
ENSGALG00000010864	EREG	4	0.257309062	0.279373944
ENSGALG00000044375	HDDC3	10	0.258277742	0.280400722
ENSGALG00000033351	CTHRC1	2	0.258374775	0.280481092
ENSGALG00000002311	GALK1	18	0.258678744	0.28078607
ENSGALG00000038574		18	0.259146401	0.281268655
ENSGALG00000030096		21	0.259421931	0.281542644
ENSGALG00000001812	ATCAY	28	0.260180593	0.282340866
ENSGALG00000032743	C1orf112	8	0.260544389	0.282710486
ENSGALG00000049504		31	0.261003412	0.283183361
ENSGALG00000006217	S100B	7	0.261659457	0.283869896
ENSGALG00000033207	HIBADH	2	0.262251334	0.284486701
ENSGALG00000023937	CMC4	4	0.26238087	0.284601899
ENSGALG00000008883	TCF7L2	6	0.262936605	0.28517933
ENSGALG00000012812	SVOPL	1	0.263076641	0.285305833
ENSGALG00000009811	TATDN3	3	0.263158006	0.285368691
ENSGALG00000054759	B3GALNT1	9	0.26396508	0.286215137
ENSGALG00000003812	UBE2F	7	0.263985519	0.286215137
ENSGALG00000010244	AKR1A1	8	0.264355221	0.286590487
ENSGALG00000011724	WDR91	1	0.265011221	0.287276122
ENSGALG00000047864		5	0.265072021	0.287316487
ENSGALG00000004496	TNIP1	13	0.265923636	0.288213946

ENSGALG00000013005	SNRNP48	2	0.266054897	0.288330581
ENSGALG00000039473	NOP53	32	0.266323608	0.288596138
ENSGALG00000036496	ACTN4	32	0.266455757	0.28871368
ENSGALG00000036359	RUVBL2	33	0.26654603	0.288785831
ENSGALG00000010915	IL6	2	0.267490606	0.289783471
ENSGALG00000003284	CRLF3	18	0.269164117	0.291570547
ENSGALG00000000632	CHTF8	11	0.269378456	0.291776807
ENSGALG00000006855	MELTF	9	0.269957701	0.292378242
ENSGALG00000052108		1	0.270101318	0.292507804
ENSGALG00000016502	TP53I3	3	0.270575829	0.292995655
ENSGALG00000026374	PFDN2	25	0.270836406	0.29325178
ENSGALG00000001857	C1QTNF12	21	0.270864434	0.293256087
ENSGALG00000034004	TRIM3	1	0.271078832	0.293462151
ENSGALG00000016794	MRPS9	1	0.271105121	0.293464555
ENSGALG00000046427	SAMD14	27	0.271251983	0.293597464
ENSGALG00000037826		33	0.271306548	0.293630458
ENSGALG00000008564	NSUN6	2	0.271598751	0.293920615
ENSGALG00000041232	TMEM223	33	0.271847315	0.2941635
ENSGALG00000006676	RLBP1	10	0.27227251	0.294597455
ENSGALG00000007247	TGFB1I1	15	0.272709311	0.295043889
ENSGALG00000035252	RPL7	2	0.273193876	0.295541916
ENSGALG00000017098	GTF3A	1	0.273402829	0.295741722
ENSGALG00000052271	SLC23A3	7	0.273737464	0.296077432
ENSGALG00000011224	SLC4A3	7	0.274327857	0.296689689
ENSGALG00000047684		5	0.274582163	0.296938387
ENSGALG00000008040		15	0.275509242	0.297914526
ENSGALG00000049767		30	0.275623825	0.298011998
ENSGALG00000047859	TSPAN31	33	0.276544335	0.298980766
ENSGALG00000044391	RBM11	1	0.276642204	0.299060058
ENSGALG00000009388	SUSD4	3	0.277491436	0.299951514
ENSGALG00000014526	TPI1	1	0.278023368	0.300499861
ENSGALG00000031766	PPP1R7	9	0.278192203	0.300655693
ENSGALG00000030661	STAT2	33	0.278517947	0.300981062

ENSGALG00000054286	APLN	4	0.278554718	0.300994122
ENSGALG00000010165	TCTE1	3	0.279045219	0.301497417
ENSGALG00000008198	TPCN3	3	0.279598844	0.302050059
ENSGALG00000047532		25	0.27960625	0.302050059
ENSGALG00000014412	CSTA	1	0.280362021	0.302839663
ENSGALG00000005352	GATA5	20	0.28094873	0.303446529
ENSGALG00000006749	WDR37	2	0.281034371	0.303512142
ENSGALG00000040771		17	0.281469186	0.303954812
ENSGALG00000012364	PPIL4	3	0.282655184	0.305208524
ENSGALG00000043756	PEX11G	28	0.282710004	0.305240687
ENSGALG00000000891	NSUN5	19	0.283020593	0.305548973
ENSGALG00000006782	MRPL46	10	0.283199711	0.305715279
ENSGALG00000013900	MAP7	3	0.283364419	0.305866002
ENSGALG00000001238	STX8	18	0.283942407	0.306462758
ENSGALG00000000654	TMED6	11	0.284445471	0.306978548
ENSGALG00000001366	CASP9	21	0.285252245	0.307821986
ENSGALG00000014261	UCHL1	4	0.285718989	0.308292098
ENSGALG00000014030	DNAI7	1	0.285738457	0.308292098
ENSGALG00000011962	NDNF	4	0.28592391	0.308464894
ENSGALG00000013174	RECQL	1	0.286368306	0.308916992
ENSGALG00000048024		17	0.286960142	0.309528044
ENSGALG00000021174		24	0.287455378	0.310034801
ENSGALG00000021039	HKDC1	6	0.289129289	0.311812615
ENSGALG00000004649	TRPV1	19	0.289907314	0.312624027
ENSGALG00000029023	DMRTB1	8	0.290162847	0.312871913
ENSGALG00000009473	EFCAB1	2	0.290458937	0.313163482
ENSGALG00000000277	GnRH-I	22	0.291442233	0.314195857
ENSGALG00000003650	NOL11	18	0.291996605	0.31476568
ENSGALG00000030964	LRRC20	6	0.292611826	0.31538182
ENSGALG00000045125	GP5	9	0.292619907	0.31538182
ENSGALG00000008912	ABCB1	2	0.292722834	0.315414604
ENSGALG00000016554	ACE2	1	0.292740714	0.315414604
ENSGALG00000040258	FARSA	30	0.292746202	0.315414604

ENSGALG00000006202	SCNN1B	14	0.292753799	0.315414604
ENSGALG00000039186		2	0.293253379	0.315924938
ENSGALG00000048930	CDC37	30	0.293346676	0.315997528
ENSGALG00000038416		6	0.293518267	0.316154438
ENSGALG00000005011		10	0.293927888	0.316567683
ENSGALG00000009566	IARS2	3	0.294178559	0.316809678
ENSGALG00000046164	APBB1	1	0.294238865	0.316846638
ENSGALG00000036941	CUL1	2	0.294324384	0.316910739
ENSGALG00000007286	SIRT7	18	0.294857287	0.317456504
ENSGALG00000040292		8	0.294929545	0.317506264
ENSGALG00000036971	FGF12	9	0.29520032	0.317769709
ENSGALG00000050176		19	0.295577173	0.318147287
ENSGALG00000003113	RGS14	13	0.295962612	0.318534039
ENSGALG00000053555		25	0.295992491	0.31853808
ENSGALG00000046637		3	0.296084871	0.318609375
ENSGALG00000005775		4	0.297048958	0.319618597
ENSGALG00000030988	RBPMS2	10	0.297189085	0.319741155
ENSGALG00000035580	MPP2	27	0.29935822	0.322046478
ENSGALG00000011740	MGP	1	0.29965676	0.322339204
ENSGALG00000009545	SLC25A12	7	0.300009278	0.322689938
ENSGALG00000034747	NAPA	32	0.300232187	0.322901214
ENSGALG00000006137		6	0.300723982	0.323401615
ENSGALG00000005631	TRPM2	9	0.300945014	0.323610773
ENSGALG00000015495	POU1F1	1	0.301250422	0.323910617
ENSGALG00000041182	TBX1	15	0.301843993	0.324520219
ENSGALG00000026817		14	0.302320894	0.32500429
ENSGALG00000016316	GCM1	3	0.302460487	0.325098453
ENSGALG00000002843	RILP	19	0.30246181	0.325098453
ENSGALG00000009042	ISM1	3	0.302518106	0.325130301
ENSGALG00000014490	P3H3	1	0.304395911	0.327119631
ENSGALG00000016424	CPSF3	3	0.304929955	0.327664664
ENSGALG00000008691	SNX5	3	0.305067766	0.327783862
ENSGALG00000054488		30	0.305805181	0.328547235

ENSGALG00000036106	SMPD1	1	0.305978139	0.328689435
ENSGALG00000033002	SLC7A3	4	0.305991453	0.328689435
ENSGALG00000011918	CCDC134	1	0.306042041	0.328714818
ENSGALG00000042029	SERINC4	10	0.306772696	0.329470579
ENSGALG00000031496	SPINK5	13	0.307239084	0.329942413
ENSGALG00000029480	TMEM138	5	0.307472107	0.330163575
ENSGALG00000000944	SUPT4H1	19	0.308592846	0.331337845
ENSGALG00000002273	LYRM1	14	0.309662442	0.332457001
ENSGALG00000001503	TBCD	18	0.310336138	0.333150954
ENSGALG00000054823	SLC30A3	3	0.310627861	0.333434766
ENSGALG00000006900		18	0.311461011	0.334299657
ENSGALG00000040929	C15orf62	5	0.311806673	0.334641208
ENSGALG00000010553	AIMP1	4	0.31201002	0.334829975
ENSGALG00000009552		5	0.312139524	0.334939471
ENSGALG00000010901		2	0.31237509	0.335162748
ENSGALG00000052672		19	0.312498482	0.335265639
ENSGALG00000050294		4	0.312777246	0.335535189
ENSGALG00000016571	GLRA2	1	0.313015986	0.33576176
ENSGALG00000015846	SNAP91	3	0.313910484	0.336691637
ENSGALG00000047805		30	0.314333314	0.337115498
ENSGALG00000032994	SOX17	2	0.314653081	0.337407037
ENSGALG00000005801	CHRNA4	20	0.314660495	0.337407037
ENSGALG00000000736	MATK	28	0.3157865	0.338584663
ENSGALG00000004476	SEMA3G	12	0.31618023	0.338977011
ENSGALG00000023740	HBZ	14	0.317506277	0.340368738
ENSGALG00000032319		8	0.317655243	0.340498495
ENSGALG00000015897	IMPG1	3	0.317880123	0.340709594
ENSGALG00000038923		11	0.318136398	0.340954303
ENSGALG00000053568		4	0.319280291	0.342150166
ENSGALG00000020201	CEP44	4	0.319319296	0.342161893
ENSGALG00000002931	REG4	8	0.319432189	0.342252784
ENSGALG00000051550		5	0.319519906	0.342316687
ENSGALG00000004831	RPS4Y1	4	0.319623514	0.342397602

ENSGALG00000040899	GPAT2	22	0.319851104	0.342611307
ENSGALG00000012290	SOX10	1	0.321367497	0.344205366
ENSGALG00000008367	MDK	5	0.32169491	0.344525782
ENSGALG00000052376		16	0.322000802	0.344823097
ENSGALG00000030377	MDFI	26	0.322037032	0.344831609
ENSGALG00000030115	WISP1	2	0.322092649	0.344860877
ENSGALG00000020703	GRB14	7	0.322130049	0.344870637
ENSGALG00000050375		30	0.322672641	0.345421204
ENSGALG00000038810		25	0.322725937	0.345447928
ENSGALG00000001640	RNF122	22	0.322804138	0.345501304
ENSGALG00000019552	SERPINB10	2	0.322872735	0.345544392
ENSGALG00000006412	VGLL1	4	0.322909781	0.345553709
ENSGALG00000006854	NEURL2	20	0.323298716	0.345939556
ENSGALG00000043185	MATN1	23	0.32436358	0.347048538
ENSGALG00000041555	COL1A1	27	0.324744781	0.347425912
ENSGALG00000038364	CCN3	2	0.32483749	0.347494605
ENSGALG00000050120		22	0.325536712	0.348212048
ENSGALG00000049951	SPOCK2	6	0.325732833	0.348391266
ENSGALG00000012196	MCUB	4	0.325924754	0.348565961
ENSGALG00000002067		14	0.326058511	0.348678427
ENSGALG00000042767	LPAR2	30	0.326165949	0.34876273
ENSGALG00000011290	RPL15	2	0.326323346	0.348900435
ENSGALG00000016831	PCID2	1	0.327321382	0.349936836
ENSGALG00000043799	DSCC1	2	0.327490461	0.350086901
ENSGALG00000031648		2	0.327626194	0.350201296
ENSGALG00000034284	SLC4A2	2	0.328056049	0.350630032
ENSGALG00000052646	EXD3	17	0.328212026	0.350765994
ENSGALG00000002683	DECR2	14	0.328855126	0.351422485
ENSGALG00000011190	PLAC8	4	0.329715479	0.352311001
ENSGALG00000031301	TULP1	26	0.329954334	0.35253533
ENSGALG00000006819	AGBL1	10	0.330912596	0.353528195
ENSGALG00000053560	FBXO16	3	0.331068168	0.35366341
ENSGALG00000017318	PAAF1	1	0.331448093	0.354038248

ENSGALG00000016665	FDFT1	3	0.331731299	0.354309717
ENSGALG00000015895	HTR1B	3	0.33196786	0.354531323
ENSGALG00000023843	FRMD7	4	0.332564525	0.355137437
ENSGALG00000049257		11	0.332794522	0.35532982
ENSGALG00000033656	DQX1	4	0.332802964	0.35532982
ENSGALG00000031776	NFASC	26	0.33322238	0.355746474
ENSGALG00000008114	SFXN2	6	0.333416208	0.35592224
ENSGALG00000010268		4	0.33353636	0.356019333
ENSGALG00000007244	SIRT4	15	0.334005234	0.356488605
ENSGALG00000035246		10	0.334544911	0.357033357
ENSGALG00000052887		11	0.335003345	0.357491317
ENSGALG00000007437	DPP8	10	0.336022081	0.358547058
ENSGALG00000034907	HOXB5	27	0.336836255	0.359384359
ENSGALG00000004440		15	0.337077793	0.359610599
ENSGALG00000008798	ZMYND19	17	0.33739498	0.359917497
ENSGALG00000010303		3	0.337427533	0.359920733
ENSGALG00000012631	PWP1	1	0.33772912	0.360210913
ENSGALG00000007559	PDSS1	2	0.338179554	0.360659783
ENSGALG00000002050	EXOC7	18	0.338852541	0.361345901
ENSGALG00000030812	CSNK1G1	10	0.339057503	0.361532849
ENSGALG00000037504	CFAP36	3	0.339100634	0.361547222
ENSGALG00000042419	KCTD17	1	0.339269903	0.361696068
ENSGALG00000048887		31	0.339821211	0.362252145
ENSGALG00000005258	SSTR5	14	0.340163135	0.362584938
ENSGALG00000008403	PHF21A	5	0.341456784	0.363932042
ENSGALG00000002089		6	0.342068313	0.364551957
ENSGALG00000046030		21	0.343029331	0.365544191
ENSGALG00000007539	ANKRD2	6	0.343551943	0.366069111
ENSGALG00000001363	MYBBP1A	19	0.344338208	0.366874849
ENSGALG00000042706	TMC2	2	0.344596629	0.367101341
ENSGALG00000000504	ETV7	26	0.344611002	0.367101341
ENSGALG00000047773		4	0.345133801	0.36762614
ENSGALG00000037901	EFEMP1	3	0.345325428	0.367798125

ENSGALG00000014425	NCAPG	4	0.345368618	0.367811998
ENSGALG00000006283	THAP4	9	0.345923527	0.368370792
ENSGALG00000011897	SMDT1	1	0.347339043	0.36984586
ENSGALG00000010598	KCNK10	5	0.347459449	0.369933503
ENSGALG00000035244		1	0.347482032	0.369933503
ENSGALG00000035457		13	0.349367825	0.371908669
ENSGALG00000029572	TOP1MT	2	0.349551671	0.372071892
ENSGALG00000003438	NUF2	8	0.350193726	0.372722774
ENSGALG00000011524	PPEF2	4	0.350988746	0.373536334
ENSGALG00000021020	CGNRH-R	10	0.351246616	0.373778145
ENSGALG00000014624	CERS3	10	0.351478125	0.373991864
ENSGALG00000007572	MMS19	6	0.351551181	0.374036959
ENSGALG00000031666	CCDC91	1	0.35271352	0.375208705
ENSGALG00000012420	CG-1B	1	0.352714031	0.375208705
ENSGALG00000006786	SYTL4	4	0.353645642	0.376166913
ENSGALG00000028949	CORO6	19	0.356075818	0.378718811
ENSGALG00000002561		26	0.356648474	0.379294796
ENSGALG00000010255	NASP	8	0.356989069	0.379623907
ENSGALG00000012285	BAIAP2L2	1	0.357254433	0.379872966
ENSGALG00000015808	SLC35A1	3	0.357427295	0.380023632
ENSGALG00000007241	NUBP1	14	0.358707781	0.381351816
ENSGALG00000030064		4	0.358939062	0.381564428
ENSGALG00000006009		12	0.360052608	0.382714798
ENSGALG00000034737		14	0.360151548	0.382786596
ENSGALG00000035839		1	0.360967866	0.383620778
ENSGALG00000003155	SRR	19	0.361084608	0.383711402
ENSGALG00000013759	MRPL47	9	0.361707405	0.384339729
ENSGALG00000037925	RND1	33	0.362860382	0.385531251
ENSGALG00000011349		7	0.363133504	0.38578782
ENSGALG00000005971	RIC3	5	0.363610704	0.386261135
ENSGALG00000008852	DNAJC19	9	0.364028012	0.386670751
ENSGALG00000011762	UBA6	4	0.364909953	0.387573785
ENSGALG00000009985	LSM6	4	0.36525947	0.38791122

ENSGALG00000030095	AP5S1	4	0.365403598	0.388030488
ENSGALG00000000154	DENND2D	26	0.366629028	0.389297896
ENSGALG00000011914	CENPM	1	0.367794928	0.390490192
ENSGALG00000019261	ARHGAP8	1	0.367815948	0.390490192
ENSGALG00000006122	EARS2	14	0.368654227	0.391346073
ENSGALG00000005956		6	0.369144205	0.391832097
ENSGALG00000001014	ARPC5L	17	0.369247909	0.391908057
ENSGALG00000053164		2	0.369769363	0.392427352
ENSGALG00000051258		6	0.370177294	0.392826088
ENSGALG00000043582		2	0.370319031	0.392942298
ENSGALG00000009792		4	0.370799966	0.393418377
ENSGALG00000004127		4	0.371886683	0.394537051
ENSGALG00000041973		33	0.372106226	0.394735619
ENSGALG00000007720	AIFM3	15	0.372936588	0.395582063
ENSGALG00000006205	GTF2B	8	0.37306249	0.395681189
ENSGALG00000044914	C11orf16	5	0.373751791	0.396377802
ENSGALG00000045218	MYO18B	15	0.374195513	0.396813871
ENSGALG00000033014		22	0.374737697	0.397354269
ENSGALG00000020876	AOX2	7	0.375594379	0.398228023
ENSGALG00000054125		33	0.375835555	0.398449084
ENSGALG00000039205		25	0.376040236	0.39863142
ENSGALG00000054793	NCAPH2	1	0.376087784	0.398647166
ENSGALG00000004700	NCF2	8	0.376674151	0.399233999
ENSGALG00000036327	NGEF	9	0.377049325	0.399596907
ENSGALG00000010375	SLC30A2	23	0.377895862	0.40045926
ENSGALG00000043240	ARPP21	2	0.378342297	0.400897509
ENSGALG00000050269		4	0.380923839	0.403597877
ENSGALG00000015006	MAB21L3	1	0.382872971	0.405627782
ENSGALG00000043593		1	0.383504449	0.40626149
ENSGALG00000027090		4	0.384198537	0.406961407
ENSGALG00000013883	SNRNP27	22	0.385712648	0.408529736
ENSGALG00000041833		19	0.385994756	0.408793022
ENSGALG00000011164		2	0.386457136	0.409247165

ENSGALG00000013234	HAPLN2	25	0.387275231	0.410077889
ENSGALG00000012456	RAC2	1	0.387451935	0.410229371
ENSGALG00000003570	ATIC	7	0.387917769	0.410686928
ENSGALG00000033834	TRIP13	2	0.388452315	0.411217145
ENSGALG00000009350	SFXN4	6	0.388516202	0.411249072
ENSGALG00000052883		1	0.38865242	0.411357549
ENSGALG00000007093	CYTH1	18	0.389906036	0.41264858
ENSGALG00000002485	PHKG1	19	0.390309247	0.413039459
ENSGALG00000047768		1	0.391083639	0.413823033
ENSGALG00000008517	MCTS1	4	0.392374526	0.41515295
ENSGALG00000007623	CACNA1G	18	0.392451992	0.415198884
ENSGALG00000007831	TLX1	6	0.393325651	0.416087078
ENSGALG00000015070	FIG4	3	0.394093256	0.416862937
ENSGALG00000031572		33	0.395014639	0.417801309
ENSGALG00000029912	CAMK1	12	0.395200034	0.417961143
ENSGALG00000045607	CXCL13	4	0.395566433	0.418277764
ENSGALG00000041473	DDX31	17	0.395568022	0.418277764
ENSGALG00000002992	DAW1	9	0.396256405	0.418969333
ENSGALG00000033271	PFKM	33	0.396333933	0.419014969
ENSGALG00000006719	GNAZ	15	0.396558837	0.419188993
ENSGALG00000024122	TNFRSF13B	14	0.396567295	0.419188993
ENSGALG00000017046	POSTN	1	0.398613159	0.421315036
ENSGALG00000012964	ANKH	2	0.399074252	0.421765827
ENSGALG00000036686	PLTP	20	0.399583797	0.422267745
ENSGALG00000001633	DUSP26	22	0.401188635	0.423901706
ENSGALG00000002131	NPRL2	12	0.401199515	0.423901706
ENSGALG00000049582		30	0.401728289	0.424423622
ENSGALG00000013598	SCGN	2	0.402917467	0.425614041
ENSGALG00000028519	CCR10	27	0.402924865	0.425614041
ENSGALG00000033877	KANSL2	33	0.404028643	0.426743004
ENSGALG00000048020		30	0.404286951	0.426978847
ENSGALG00000002647	PLS1	9	0.404670371	0.427346772
ENSGALG00000045436		17	0.406259524	0.428987821

ENSGALG00000006751	THY1	24	0.407345033	0.430096812
ENSGALG00000005349	MSLN	14	0.408505291	0.431284528
ENSGALG00000003369	KAT2A	27	0.409423446	0.432216457
ENSGALG00000003563	YARS	23	0.409741994	0.432515291
ENSGALG00000002837	RPL27	27	0.411807887	0.434658376
ENSGALG00000051594	FBXO46	32	0.412136143	0.434967194
ENSGALG00000002179	FBF1	18	0.412546276	0.435362363
ENSGALG00000003286	ACSBG1	10	0.412991803	0.435794812
ENSGALG00000016911	OBI1	1	0.413134864	0.435908047
ENSGALG00000039791	DOLPP1	17	0.41501494	0.437853869
ENSGALG00000050621	MRPS16	21	0.415205158	0.438016654
ENSGALG00000038299	NUDCD3	22	0.416247642	0.439078423
ENSGALG00000012135	ATF4	1	0.416653502	0.439468525
ENSGALG00000012724	PAK1IP1	2	0.416916194	0.439707564
ENSGALG00000006361	NEU4	9	0.419271611	0.442153498
ENSGALG00000015497	VGLL3	1	0.419973579	0.442855475
ENSGALG00000026516	CZIB	8	0.420284361	0.443115508
ENSGALG00000045729	HHIPL2	3	0.420292861	0.443115508
ENSGALG00000006415	CD40LG	4	0.42047952	0.443273974
ENSGALG00000010324	MRPL1	4	0.421770889	0.44459691
ENSGALG00000021529	GP1BB	15	0.421887942	0.444681853
ENSGALG00000039329		21	0.421975098	0.444735273
ENSGALG00000053468	ANKRD66	3	0.422241771	0.444977866
ENSGALG00000054014		1	0.422404462	0.445110847
ENSGALG00000047223		1	0.422530954	0.445205662
ENSGALG00000043329		9	0.422601435	0.445241449
ENSGALG00000003944	FAM189A1	10	0.423044489	0.44566973
ENSGALG00000045312	SEMA6C	25	0.424195293	0.446843474
ENSGALG00000034649	TOX3	11	0.424352344	0.446970295
ENSGALG00000023682	GTF2H3	15	0.424470869	0.447056517
ENSGALG00000021451		11	0.426268887	0.448911431
ENSGALG00000002426	EXOSC3	6	0.426644085	0.449267754
ENSGALG00000005336	GUK1	2	0.426976391	0.449578855

ENSGALG00000053854	AKR1E2	1	0.427061347	0.44962948
ENSGALG00000009269	PTGR2	5	0.427241422	0.449780233
ENSGALG00000003838		23	0.427947545	0.450484712
ENSGALG00000007383	HSPB8	15	0.428048001	0.450551561
ENSGALG00000044610	CFAP100	12	0.428603981	0.451097828
ENSGALG00000005094	GRAPL	14	0.429460266	0.45196004
ENSGALG00000030979	MKS1	19	0.430840403	0.453373353
ENSGALG00000014662	RBP5	1	0.431500096	0.454028363
ENSGALG00000010623	ORC1	8	0.431664216	0.45416186
ENSGALG00000016461	DDX1	3	0.431874814	0.454344229
ENSGALG00000017047	SUPT20H	1	0.431918535	0.454351022
ENSGALG00000029618		5	0.433330274	0.455796759
ENSGALG00000004834	COPS3	14	0.433505512	0.45594175
ENSGALG00000042119	MIP	33	0.433621868	0.456024792
ENSGALG00000041708	WNT4	21	0.437526587	0.460091564
ENSGALG00000011514	SLC25A3	1	0.438180492	0.460739457
ENSGALG00000047995		31	0.438640495	0.461183372
ENSGALG00000031707	DHH	33	0.439452317	0.461997077
ENSGALG00000030687	LGI3	22	0.441110587	0.463700439
ENSGALG00000028466		11	0.442575806	0.465200586
ENSGALG00000030356	OTOP1	4	0.444752118	0.467447856
ENSGALG00000016430	PDHA2	1	0.445010297	0.467647211
ENSGALG00000034960	MLXIPL	19	0.445018501	0.467647211
ENSGALG00000007349	RASL12	10	0.445517776	0.468131528
ENSGALG00000019358	LLPH	1	0.446396578	0.469014517
ENSGALG00000007803		6	0.447175915	0.46979286
ENSGALG00000007116	CABP1	15	0.447940121	0.470555173
ENSGALG00000037492	MGRN1	14	0.448126277	0.470671749
ENSGALG00000035804	SQSTM1	13	0.448128298	0.470671749
ENSGALG00000010675	PSMC1	5	0.448239804	0.470748314
ENSGALG00000036498	EHD2	32	0.448355642	0.470789312
ENSGALG00000050368		14	0.448356064	0.470789312
ENSGALG00000031567	GPALPP1	1	0.448992735	0.471397203

ENSGALG00000047414		14	0.449012311	0.471397203
ENSGALG00000042246		32	0.449591419	0.471964543
ENSGALG00000010997	DFNA5	2	0.449755198	0.472095827
ENSGALG00000007300	PYCR1	18	0.451122731	0.473490528
ENSGALG00000035239	GLCCI1	2	0.451190664	0.473521068
ENSGALG00000032993	FTSJ3	27	0.451493793	0.473798418
ENSGALG00000004085	ZDHHC13	5	0.451849279	0.47413066
ENSGALG00000034414	SLU7	13	0.453774604	0.476109952
ENSGALG00000008552	MAL	3	0.453918545	0.476219999
ENSGALG00000000498	ACE	27	0.455025795	0.477340576
ENSGALG00000011358	WNT6	7	0.455378129	0.477669092
ENSGALG00000015483	PNISR	3	0.455914963	0.478191066
ENSGALG00000007976		4	0.458295593	0.480646668
ENSGALG00000003114	NOG	18	0.459475721	0.481842906
ENSGALG00000006221	KIF1A	9	0.459942598	0.48229103
ENSGALG00000049225		2	0.460427651	0.482758134
ENSGALG00000008731	DZANK1	3	0.4608413	0.483150298
ENSGALG00000039572	PADI1	21	0.460971035	0.483244762
ENSGALG00000034955	DCTN2	33	0.461422393	0.483676345
ENSGALG00000030160	DRC7	11	0.461741003	0.483968714
ENSGALG00000038595	POLR2I	16	0.462511902	0.484735054
ENSGALG00000047031		33	0.463493056	0.485721602
ENSGALG00000014820	VTCN1	1	0.464088618	0.486303929
ENSGALG00000035901	GDF6	2	0.464765716	0.486971589
ENSGALG00000034042	PIAS3	25	0.464949603	0.487122402
ENSGALG00000051397		7	0.465673397	0.487838795
ENSGALG00000006688	TADA3	12	0.465861702	0.487994135
ENSGALG00000004004	PHKB	11	0.466167995	0.488273031
ENSGALG00000043861		4	0.466351581	0.488423366
ENSGALG00000027476	PLEKHG5	21	0.46724787	0.489320044
ENSGALG00000035817	CIAO1	22	0.468724412	0.490824181
ENSGALG00000017289	C12orf4	1	0.470169019	0.492294619
ENSGALG00000013899	VPS45	25	0.471144068	0.493273195

ENSGALG00000044709		31	0.473571246	0.495771804
ENSGALG00000013410	UBA3	12	0.474400988	0.496590403
ENSGALG00000016362	SH3YL1	3	0.474434643	0.496590403
ENSGALG00000032428		11	0.474615786	0.496737363
ENSGALG00000010175	HSP90AB1	3	0.474838583	0.49692789
ENSGALG00000009843	INTS7	3	0.47498606	0.497039567
ENSGALG00000049487	KCNK15	20	0.475030608	0.497043526
ENSGALG00000027874	CHAC1	5	0.475138755	0.497114024
ENSGALG00000042726	TRMT9B	4	0.475634471	0.497589971
ENSGALG00000011868	SNAPC1	5	0.475973022	0.49790143
ENSGALG00000015573	FHL5	3	0.476065636	0.497955589
ENSGALG00000016467	SMC6	3	0.476890992	0.498776108
ENSGALG00000013649	CNOT7	4	0.477095633	0.498947341
ENSGALG00000043283	LGI2	4	0.477642136	0.499476034
ENSGALG00000033378	AKTIP	11	0.478475437	0.50030452
ENSGALG00000053285		25	0.478644844	0.50043874
ENSGALG00000010233	SYNDIG1L	5	0.479408955	0.501194666
ENSGALG00000005774	NKAIN4	20	0.479572286	0.501322436
ENSGALG00000013072	AKR1B10	1	0.479642397	0.501352743
ENSGALG00000037052		23	0.479858553	0.501535688
ENSGALG00000008417	CFAP58	6	0.480034153	0.501676218
ENSGALG00000036850	DNAJA4	10	0.480117425	0.50172024
ENSGALG00000024085		22	0.481742791	0.503375598
ENSGALG00000041981	PRR7	13	0.484340411	0.506046498
ENSGALG00000005668	WSB1	19	0.484512193	0.506182604
ENSGALG00000041844	DERL2	19	0.484954748	0.506601546
ENSGALG00000025941	RGS20	2	0.485064977	0.506673286
ENSGALG00000010078	GLP1R	3	0.485129255	0.506697019
ENSGALG00000031426	TMBIM6	33	0.485814579	0.50736935
ENSGALG00000047639		22	0.486337128	0.507852498
ENSGALG00000027142	RPL32	12	0.486360504	0.507852498
ENSGALG00000015807	RWDD2B	1	0.486675664	0.508135778
ENSGALG00000003930	APBA2	10	0.486715144	0.508135778

ENSGALG00000005657	CRHR2	2	0.487211479	0.508610407
ENSGALG00000011169	PDCD2	3	0.487502256	0.508870388
ENSGALG00000010713		3	0.488021959	0.509369265
ENSGALG00000045403	R3HCC1L	6	0.488529912	0.509855792
ENSGALG00000009668	HPF1	4	0.490410935	0.511775123
ENSGALG00000010801	TMEM61	8	0.490516805	0.511841798
ENSGALG00000009261	VAX1	6	0.490720153	0.512010168
ENSGALG00000047464	FBXL22	10	0.49187113	0.513167168
ENSGALG00000002432	DYDC2	6	0.49232781	0.513599673
ENSGALG00000017368	TRNT1	12	0.492788535	0.514036326
ENSGALG00000007333	UBAP1L	10	0.493214254	0.51443639
ENSGALG00000010411	RNF13	9	0.49400661	0.515218767
ENSGALG00000042664	CBLN1	11	0.494821014	0.516024001
ENSGALG00000001062	GGT7	20	0.494977769	0.516143329
ENSGALG00000054294		31	0.495623539	0.516772517
ENSGALG00000040298	TPK1	2	0.496003712	0.517124691
ENSGALG00000035814		27	0.496204206	0.51728949
ENSGALG00000004247	MORN3	15	0.496551076	0.517606845
ENSGALG00000008763	SSX2IP	8	0.496839384	0.517863106
ENSGALG00000054877		15	0.497468325	0.518474339
ENSGALG00000002427	SMIM5	18	0.500511651	0.521601586
ENSGALG00000012710	NCS1	17	0.501418228	0.522501706
ENSGALG00000028437	TRIM62	21	0.502988867	0.524093596
ENSGALG00000004306	SYT12	5	0.503540822	0.524623878
ENSGALG00000034430	CMC2	11	0.50445954	0.525536156
ENSGALG00000016722		1	0.505776915	0.526863556
ENSGALG00000039837	CCKBR	1	0.50612756	0.527183781
ENSGALG00000030793	KCNE2	1	0.506605681	0.527629927
ENSGALG00000023772	HSPB7	21	0.506642433	0.527629927
ENSGALG00000043394	NUP188	17	0.50669404	0.527638605
ENSGALG00000047017	PHYHIP	22	0.507643625	0.528582299
ENSGALG00000050819		32	0.508575437	0.529507327
ENSGALG00000008280	PMPCB	1	0.508904641	0.52980484

ENSGALG00000051008		15	0.50904314	0.529903783
ENSGALG00000049348		8	0.511670309	0.532593143
ENSGALG00000034868	KRT7	33	0.512458468	0.533367998
ENSGALG00000044739		1	0.513329737	0.534229215
ENSGALG00000011361	CRYZ	8	0.513925371	0.534803452
ENSGALG00000010926	SPP1	4	0.516081784	0.537001638
ENSGALG00000006376	TMC3	10	0.516293055	0.537175631
ENSGALG00000041161	NT5DC4	22	0.518888333	0.539829816
ENSGALG00000013575	IFI6	2	0.519523612	0.540444621
ENSGALG00000008796	RPF1	8	0.520073177	0.540970162
ENSGALG00000015935	SMYD1	4	0.523582149	0.544573671
ENSGALG00000052464	ANKRD33	33	0.523783667	0.5447368
ENSGALG00000032619	SYPL2	26	0.524123052	0.545043273
ENSGALG00000030857	USP48	21	0.524617702	0.545511141
ENSGALG00000046375	SULT6B1	3	0.525915274	0.546813757
ENSGALG00000002528	DNAJC18	13	0.527711795	0.5486306
ENSGALG00000005493	ITGB1BP2	4	0.527815959	0.5486306
ENSGALG00000007868	BCO2	24	0.527823744	0.5486306
ENSGALG00000016392	RPS7	3	0.527842662	0.5486306
ENSGALG00000010044	KCNMB4	1	0.528795797	0.549574425
ENSGALG00000007269	GABRA3	4	0.530735151	0.551542972
ENSGALG00000026716	CELF5	28	0.531476758	0.552266585
ENSGALG00000047976	ASB16	27	0.53193709	0.552697822
ENSGALG00000005861		14	0.533540159	0.55431622
ENSGALG00000028928	LCAT	11	0.535196264	0.555989439
ENSGALG00000011439	RBMS3	2	0.536734357	0.557539787
ENSGALG00000044874		2	0.538084432	0.55889458
ENSGALG00000049859	H2AFJ	1	0.539361477	0.560173296
ENSGALG00000032329	NPM3	6	0.539938682	0.56072501
ENSGALG00000005610	SLC44A3	8	0.5401478	0.560894407
ENSGALG00000038133	SMIM3	13	0.540670908	0.561389797
ENSGALG00000045602		20	0.540722264	0.561395314
ENSGALG00000026613	S100A4	25	0.54081869	0.56144762

ENSGALG00000046988		10	0.540993437	0.561581217
ENSGALG00000005648		4	0.541256516	0.561806478
ENSGALG00000044388		13	0.543393645	0.563957901
ENSGALG00000046187	PARP12	1	0.543446345	0.563957901
ENSGALG00000012293	C22orf23	1	0.543468001	0.563957901
ENSGALG00000015822		1	0.544922059	0.56541866
ENSGALG00000003086	EXOSC10	21	0.544970748	0.565421063
ENSGALG00000049529		1	0.54518763	0.565597956
ENSGALG00000009934	NUP107	1	0.545478154	0.565851211
ENSGALG00000009433	BAG3	6	0.545863519	0.566202798
ENSGALG00000006969	USP28	24	0.548521584	0.568911507
ENSGALG00000007806	FGF16	4	0.5494112	0.569785724
ENSGALG00000005551	DKK3	5	0.55060229	0.570972421
ENSGALG00000006836	SNX21	20	0.550884652	0.571216648
ENSGALG00000021341	ADAM20	15	0.551613631	0.571923895
ENSGALG00000009023	ING3	1	0.552327233	0.572615079
ENSGALG00000002908	TRAIP	12	0.553611825	0.57389806
ENSGALG00000054473	SLC13A3	20	0.555334766	0.575635195
ENSGALG00000044612	INAVA	26	0.555499595	0.575757103
ENSGALG00000007147		15	0.555645167	0.575859033
ENSGALG00000027147		28	0.556045111	0.576224549
ENSGALG00000029788	CCK	2	0.556519976	0.576667635
ENSGALG00000006408	PIGX	9	0.556753464	0.576860553
ENSGALG00000030084	EXOSC7	2	0.560009283	0.580184654
ENSGALG00000009554	KLHL2	4	0.561093489	0.581258531
ENSGALG00000002015	SNCG	6	0.56146875	0.581597865
ENSGALG00000047196		4	0.564765344	0.58496295
ENSGALG00000042661	MVB12A	30	0.564958854	0.585113676
ENSGALG00000004878		21	0.565606621	0.5857348
ENSGALG00000034501	ATP8B2	25	0.566414754	0.586521877
ENSGALG00000052689		25	0.568660631	0.588797475
ENSGALG00000047801	GIPC3	28	0.569834488	0.589962802
ENSGALG00000003085	CDK1	6	0.570007654	0.590091979

ENSGALG00000026605	BAG2	3	0.571911773	0.591983942
ENSGALG00000033405	DBN1	13	0.571932325	0.591983942
ENSGALG00000001330	ATP5MC1	27	0.572875631	0.592909988
ENSGALG00000011104	GCG	7	0.573305497	0.593304526
ENSGALG00000035879	ITGA7	33	0.576026351	0.596069702
ENSGALG00000023335	RCHY1	4	0.576823421	0.596843854
ENSGALG00000004321	OSER1	20	0.57919279	0.599213573
ENSGALG00000007503	KMO	7	0.579211939	0.599213573
ENSGALG00000004179	ANAPC5	15	0.579431045	0.599389389
ENSGALG00000053391		25	0.580812662	0.600767627
ENSGALG00000015531		4	0.581544371	0.601473451
ENSGALG00000001696	SAG	9	0.583065893	0.602995966
ENSGALG00000053019		16	0.584235461	0.604154269
ENSGALG00000003122	ZFAND2A	14	0.584438501	0.60426174
ENSGALG00000024379		13	0.584438506	0.60426174
ENSGALG00000006436	AQP12A	9	0.585857151	0.605677144
ENSGALG00000035421	SNRPN	20	0.586120616	0.60589815
ENSGALG00000000793	VPS72	25	0.586661771	0.606406153
ENSGALG00000010182	SLC29A1	3	0.587881892	0.607615827
ENSGALG00000011372	CINP	5	0.588443275	0.608144503
ENSGALG00000031306	BHLHA15	14	0.590693873	0.610418713
ENSGALG00000044241		11	0.590985635	0.610668462
ENSGALG00000008151	WSB2	15	0.593312107	0.613020466
ENSGALG00000029823		6	0.593906316	0.613582419
ENSGALG00000000802	DHODH	11	0.594421746	0.614062895
ENSGALG00000054458	AKT2	32	0.596386015	0.616039875
ENSGALG00000028236	PRRX2	17	0.596978114	0.61659925
ENSGALG00000012190	TMEM163	7	0.598707262	0.618332852
ENSGALG00000018503	NKX6-3	22	0.599333365	0.618927053
ENSGALG00000013990	RPS12	3	0.600298668	0.619871414
ENSGALG00000010879	FGGY	8	0.600913024	0.620453256
ENSGALG00000041459	DDIT4	6	0.601360489	0.620862697
ENSGALG00000002473	GPR157	21	0.601546189	0.621001836

ENSGALG00000004702	DYNC2I2	17	0.601669469	0.621076518
ENSGALG00000010457		5	0.602042961	0.621409449
ENSGALG00000041193	NFYB	1	0.602612354	0.621909414
ENSGALG00000051473		1	0.602629355	0.621909414
ENSGALG00000052387		2	0.602904116	0.622140308
ENSGALG00000020449	COX16	5	0.604860361	0.624106151
ENSGALG00000000433		7	0.606523518	0.625769272
ENSGALG00000001604		24	0.606976516	0.626183658
ENSGALG00000047313		5	0.607091557	0.626249354
ENSGALG00000001369		26	0.607747186	0.626872636
ENSGALG00000027695	PCBP4	12	0.608072803	0.627155446
ENSGALG00000036645	KIF15	2	0.608136808	0.627168409
ENSGALG00000035949	ACAD11	2	0.608811983	0.627811613
ENSGALG00000014528	ENO2	1	0.609369804	0.628333702
ENSGALG00000001768	TENM2	13	0.609570564	0.62848756
ENSGALG00000031262		3	0.611189667	0.630103629
ENSGALG00000005558	TBL3	14	0.611572996	0.630445515
ENSGALG00000003532	DDX5	18	0.612146182	0.630983041
ENSGALG00000054317		1	0.612623169	0.631421327
ENSGALG00000008228	PPIL3	7	0.612874357	0.631626831
ENSGALG00000040355	TCF24	2	0.61301149	0.631714765
ENSGALG00000026111	SAAL1	5	0.613826272	0.63250095
ENSGALG00000050763	PBXIP1	25	0.613977447	0.632603263
ENSGALG00000040828	POLB	22	0.614598443	0.633189591
ENSGALG00000037688	MYG1	33	0.61501625	0.633566503
ENSGALG00000029166	UBTD1	6	0.616007918	0.634534472
ENSGALG00000010998	EFCAB7	8	0.616564392	0.635054032
ENSGALG00000028347	SDCBP2	20	0.616751958	0.635193566
ENSGALG00000041429	MPV17L2	28	0.617132819	0.635532134
ENSGALG00000016717	GTPBP6	1	0.618048973	0.63642185
ENSGALG00000032051	CYB561A3	5	0.618127901	0.636449375
ENSGALG00000006876	CTSA	20	0.618568932	0.636849699
ENSGALG00000029024	TCF21	3	0.619050046	0.63729122

ENSGALG00000009868	ZNF330	4	0.620370661	0.638596832
ENSGALG00000028567	MYL9	20	0.621754505	0.639967304
ENSGALG00000012546	MCM5	1	0.622420237	0.640598459
ENSGALG00000055117		2	0.624126304	0.642300137
ENSGALG00000027822		18	0.62491696	0.64305954
ENSGALG00000033231	RACGAP1	33	0.625596133	0.643704105
ENSGALG00000036796	HYLS1	24	0.62581464	0.643874602
ENSGALG00000034196	WNT2B	26	0.627092701	0.645135108
ENSGALG00000013723	OASL	12	0.628268745	0.646290459
ENSGALG00000039576	KNSTRN	5	0.628445338	0.646417581
ENSGALG00000027415	GRIN2C	18	0.629872728	0.64783114
ENSGALG00000001926	HSPB1	19	0.635333673	0.653392668
ENSGALG00000041640	TWIST1	2	0.635611038	0.653622787
ENSGALG00000034478	CCL4	19	0.637205673	0.655207352
ENSGALG00000040586	TUBA3E	26	0.638148056	0.656121026
ENSGALG00000010108		4	0.639385659	0.657338055
ENSGALG00000035086	MYL6	33	0.641267632	0.659217286
ENSGALG00000003952	RBPJL	20	0.641504968	0.659405671
ENSGALG00000007786	DOK5	20	0.641841932	0.659696424
ENSGALG00000004725		15	0.643229027	0.661066379
ENSGALG00000028817	OTX1	3	0.643391145	0.661177263
ENSGALG00000008059	ASCC2	15	0.643697252	0.661436086
ENSGALG00000016044	SIM2	1	0.644770127	0.662482696
ENSGALG00000031397	NT5C3A	2	0.646879056	0.664593557
ENSGALG00000009945	CPM	1	0.646977445	0.664638637
ENSGALG00000004588	RPS14	13	0.647527493	0.66514766
ENSGALG00000023120	SULT1B1	4	0.647642145	0.66520939
ENSGALG00000033120	SRSF7	3	0.648336894	0.665846515
ENSGALG00000004916	DRC3	14	0.648371661	0.665846515
ENSGALG00000010322	JDP2	5	0.650402505	0.667875841
ENSGALG00000013607	FBXO5	3	0.651115116	0.668551294
ENSGALG00000002001	STXBP3	8	0.651638298	0.669032146
ENSGALG00000007945	CRYAB	24	0.652638336	0.670002461

ENSGALG00000034982	CYP11A1	10	0.652732382	0.670042594
ENSGALG00000003333	GHDC	27	0.652934491	0.67019364
ENSGALG00000008666	CTLA4	7	0.653575482	0.670795106
ENSGALG00000036346	AMH	28	0.654267053	0.671448377
ENSGALG00000016863	TEX30	1	0.655166855	0.672315222
ENSGALG00000033641	NRG2	13	0.656088629	0.673204464
ENSGALG00000013626	GMNN	2	0.656582997	0.673655039
ENSGALG00000036897	SLC16A8	1	0.657649634	0.674692637
ENSGALG00000034107	TRIM63	23	0.658661105	0.675673469
ENSGALG00000001551	CCNL2	21	0.658889569	0.675850973
ENSGALG00000029817	USP22	14	0.660020036	0.676953591
ENSGALG00000003189	NFYC	23	0.661181347	0.678087658
ENSGALG00000004691	NLE1	19	0.661437006	0.678292801
ENSGALG00000029968	GADD45B	28	0.665670627	0.682576903
ENSGALG00000009619	GPR176	5	0.667334536	0.68422553
ENSGALG00000008733	LPAR3	8	0.667698025	0.68452681
ENSGALG00000038433	SPON2	4	0.667740661	0.68452681
ENSGALG00000049593		2	0.668585738	0.685335511
ENSGALG00000036257	ORMDL1	7	0.668878885	0.685578366
ENSGALG00000054988	SLC22A31	11	0.669044655	0.685690634
ENSGALG00000006040	UCKL1	20	0.669872528	0.686477535
ENSGALG00000046313	DNASE1L2	14	0.669925054	0.686477535
ENSGALG00000038231	CFAP126	25	0.670183051	0.686684196
ENSGALG00000002798	WDR59	11	0.671329268	0.687800837
ENSGALG00000009875	HMGA2	1	0.671659719	0.688081579
ENSGALG00000008861	BSPRY	17	0.672463975	0.688847623
ENSGALG00000022813	NDUFV3	1	0.673211296	0.689555219
ENSGALG00000023933	G0S2	26	0.674367926	0.690681909
ENSGALG00000031243	ABCB8	2	0.674989396	0.691260348
ENSGALG00000033322	MTHFSD	11	0.675249904	0.691469058
ENSGALG00000001314	PTGS1	17	0.6786495	0.694891951
ENSGALG00000012130	DNAJC24	5	0.6791274	0.695322898
ENSGALG00000053689		2	0.680579683	0.696751307

ENSGALG00000016332	MCPH1	3	0.68130741	0.697437767
ENSGALG00000001666	RPAIN	19	0.682200782	0.698293665
ENSGALG00000014513	CDCA3	1	0.685468809	0.701579886
ENSGALG00000040773	C9orf116	17	0.688289991	0.704408248
ENSGALG00000038708	KCNQ4	23	0.688869317	0.704941971
ENSGALG00000017031		1	0.690352549	0.706400523
ENSGALG00000040910	COA7	8	0.692011334	0.708038449
ENSGALG00000050943	RNF19B	23	0.692806111	0.708792156
ENSGALG00000040016	PPP1CA	5	0.696079217	0.712081038
ENSGALG00000011799		1	0.69675912	0.712716774
ENSGALG00000023199	HNRNPDL	4	0.697129421	0.713035738
ENSGALG00000019276		1	0.699168256	0.71506111
ENSGALG00000031824	HOOK2	30	0.699398663	0.715236761
ENSGALG00000034265	ZAR1L	1	0.700278846	0.716076818
ENSGALG00000001076	LIMK1	19	0.701038818	0.71679382
ENSGALG00000010298	DCTN6	4	0.701880831	0.717584897
ENSGALG00000001824		11	0.701952732	0.717584897
ENSGALG00000035690	RAPGEFL1	27	0.701989064	0.717584897
ENSGALG00000001998	R3HDM4	28	0.702644073	0.718194248
ENSGALG00000010461	EBF3	6	0.704815931	0.720353785
ENSGALG00000013604	VIP	3	0.707033976	0.72256016
ENSGALG00000036856	CBX5	33	0.707227366	0.722697224
ENSGALG00000025886	SUSD3	12	0.707826794	0.723249149
ENSGALG00000019663	ACBD7	2	0.708124909	0.72349313
ENSGALG00000050811	SP7	33	0.710211846	0.725564562
ENSGALG00000011970	CHADL	1	0.710523966	0.725822614
ENSGALG00000010625	SRD5A2	3	0.71088838	0.726134039
ENSGALG00000045940		3	0.710974202	0.726160868
ENSGALG00000048769		1	0.712690529	0.727793015
ENSGALG00000037395	UBTD2	13	0.712691593	0.727793015
ENSGALG00000035825		28	0.714388143	0.729464419
ENSGALG00000050132		16	0.714873173	0.729898559
ENSGALG00000003285	CDHR2	13	0.716189526	0.731181353

ENSGALG00000053543		12	0.717382083	0.732337554
ENSGALG00000035157	SOX21	1	0.718992653	0.733920252
ENSGALG00000006407		5	0.719575596	0.734453812
ENSGALG00000015204	ATG3	1	0.7197867	0.734607787
ENSGALG00000008888	IL1RAPL2	4	0.720124897	0.734891435
ENSGALG00000031758	SUGCT	2	0.72053831	0.735251787
ENSGALG00000042360	TRIM29	24	0.722906753	0.737606866
ENSGALG00000051251		1	0.723707447	0.738362053
ENSGALG00000008859	WDR31	17	0.724134792	0.738713968
ENSGALG00000050187		25	0.724173547	0.738713968
ENSGALG00000003175		17	0.728376524	0.74293918
ENSGALG00000019755		18	0.729466167	0.743988371
ENSGALG00000038110	DGKA	33	0.730225724	0.744700757
ENSGALG00000007494	LARS1	13	0.730776758	0.745200386
ENSGALG00000019796	DAP3	25	0.731152315	0.745521005
ENSGALG00000008806	RPL12	17	0.731782377	0.746101055
ENSGALG00000010089	DPH2	8	0.732129115	0.746392166
ENSGALG00000030466	CS	33	0.732959583	0.74717634
ENSGALG00000003281	GSS	20	0.733761462	0.747931241
ENSGALG00000031546		5	0.734145036	0.748259669
ENSGALG00000003821	RAMP1	7	0.734663573	0.748725589
ENSGALG00000012222	RAP1GDS1	4	0.734729069	0.748729757
ENSGALG00000009925	MTA3	3	0.737586833	0.751579162
ENSGALG00000034661	ID1	20	0.737667692	0.751598744
ENSGALG00000003510	UCK2	8	0.738600042	0.752485821
ENSGALG00000012538	NR4A2	7	0.738964893	0.752794631
ENSGALG00000003003	CCL20	9	0.739550395	0.753328151
ENSGALG00000005064	PRPSAP2	14	0.740059804	0.753784077
ENSGALG00000014016	MEX3A	25	0.740980964	0.754659279
ENSGALG00000015305	NR2E1	3	0.742085575	0.755721157
ENSGALG00000041774	TESMIN	5	0.74352394	0.757084888
ENSGALG00000051597		30	0.743548883	0.757084888
ENSGALG00000029921	CSRP2	1	0.744833031	0.758329087

ENSGALG00000006198	LSS	7	0.745755667	0.759205047
ENSGALG00000032687	PHLDA2	5	0.746741662	0.760145357
ENSGALG00000000908	ADAT1	11	0.746973259	0.760317635
ENSGALG00000041203	CRMP1	4	0.747506519	0.760796911
ENSGALG00000037856	CHL1	12	0.752866216	0.766187945
ENSGALG00000028809	CHCHD1	6	0.753360708	0.7666272
ENSGALG00000013838		5	0.75352716	0.766732593
ENSGALG00000038209	TRIM39.1	16	0.754941385	0.768107503
ENSGALG00000029768	PTGDR	5	0.755335442	0.768444309
ENSGALG00000000839	WNT11	1	0.758027456	0.771118703
ENSGALG00000037206	ST7	1	0.758992118	0.772035613
ENSGALG00000049241		22	0.760130288	0.773128845
ENSGALG00000014640	GOLPH3L	25	0.761040424	0.773989982
ENSGALG00000013720	FBXO15	2	0.761596752	0.774491176
ENSGALG00000016546	RBBP7	1	0.764952812	0.777839183
ENSGALG00000006186	RAD21L1	20	0.76525614	0.778082732
ENSGALG00000008821	NDUFA5	1	0.766034121	0.778808809
ENSGALG00000016507	FNDC4	3	0.766560744	0.779279236
ENSGALG00000001805		18	0.766970198	0.77959444
ENSGALG00000004152	C9orf78	17	0.767004516	0.77959444
ENSGALG00000008793	SEC23B	3	0.767062618	0.77959444
ENSGALG00000011192	PHF10	3	0.767529542	0.780003976
ENSGALG00000030151	LUZP2	5	0.767633902	0.780045018
ENSGALG00000013821	GEMIN6	3	0.768591683	0.7809532
ENSGALG00000029537	SNUPN	10	0.771225028	0.7835636
ENSGALG00000014435	MRPL51	1	0.772964923	0.785265898
ENSGALG00000004583	NPLOC4	18	0.775182034	0.787452682
ENSGALG00000016505	GPN1	3	0.775266813	0.787473196
ENSGALG00000020151	NMU	4	0.778440916	0.790631411
ENSGALG00000011549	ANKS1B	1	0.779848956	0.791995529
ENSGALG00000006134	HOGA1	6	0.780422028	0.792511518
ENSGALG00000031977	CACNG2	1	0.780703505	0.792731332
ENSGALG00000005489	DDX52	19	0.781933026	0.793913681

ENSGALG00000025809	TMEM161A	28	0.78215778	0.794075755
ENSGALG00000037258	MRPL32	2	0.782384542	0.79423984
ENSGALG00000008103	CEP41	1	0.782564437	0.794356325
ENSGALG00000016508		3	0.782919196	0.794650275
ENSGALG00000027070	TIMP2	18	0.784498081	0.796186541
ENSGALG00000016965	DNAJC15	1	0.78499553	0.796625093
ENSGALG00000037286	VIL1	7	0.785903637	0.79748028
ENSGALG00000007036	PITRM1	2	0.786272846	0.797788534
ENSGALG00000019509	CCDC102B	2	0.787195847	0.798658592
ENSGALG00000011114	COG2	3	0.788383674	0.799797166
ENSGALG00000000949	HBEGF	13	0.789629286	0.800994167
ENSGALG00000031359	GCK	22	0.790211633	0.801518213
ENSGALG00000041025	FOXJ1	18	0.790827677	0.802076349
ENSGALG00000004495		18	0.791661267	0.802855013
ENSGALG00000003770	ANXA2	10	0.79203543	0.803167664
ENSGALG00000001727	LZTS1	22	0.792562834	0.803635645
ENSGALG00000029714	UNC45A	10	0.793193168	0.804207908
ENSGALG00000001446	GSN	17	0.793481264	0.804433114
ENSGALG00000053659		3	0.795224921	0.80613381
ENSGALG00000040716	PRLH	7	0.795969437	0.80682146
ENSGALG00000035584	COL3A1	7	0.797084274	0.807884335
ENSGALG00000052512	NPS	6	0.798036041	0.808781768
ENSGALG00000001983		8	0.799132418	0.809825596
ENSGALG00000038652		3	0.799854481	0.81048996
ENSGALG00000006602	SYT8	5	0.800853764	0.811435096
ENSGALG00000038469	CDC25A	2	0.801296447	0.811816168
ENSGALG00000015200	DAZAP1	28	0.801476533	0.811931153
ENSGALG00000010986	GNG4	3	0.802298747	0.81269657
ENSGALG00000006130	STMN3	20	0.806418478	0.816733498
ENSGALG00000051146	TLE5	28	0.806450327	0.816733498
ENSGALG00000034282		32	0.806484977	0.816733498
ENSGALG00000051159		30	0.808069169	0.81826986
ENSGALG00000034621	KCNIP4	4	0.808217115	0.81835171

ENSGALG00000045096		14	0.808720625	0.818793539
ENSGALG00000013041	CCT5	2	0.810767681	0.820797936
ENSGALG00000004068	FAM114A2	13	0.812942568	0.822931401
ENSGALG00000012378	KATNA1	3	0.813515735	0.823443248
ENSGALG00000041822	FGF22	28	0.813921936	0.823786018
ENSGALG00000011624	PNLDC1	3	0.815660116	0.825476742
ENSGALG00000017378	CRTAC1	6	0.816357549	0.826114001
ENSGALG00000038984		23	0.816438895	0.826127755
ENSGALG00000032506	GEM	2	0.816890395	0.826516023
ENSGALG00000006140		14	0.820862301	0.830402008
ENSGALG00000006755	USP2	24	0.820867334	0.830402008
ENSGALG00000045063	LRP2BP	4	0.822206691	0.83168792
ENSGALG00000005628	COL9A3	20	0.824201718	0.833636794
ENSGALG00000052318		12	0.824330695	0.833674336
ENSGALG00000004365		15	0.824375582	0.833674336
ENSGALG00000041294	COQ2	4	0.825745569	0.834990523
ENSGALG00000017321	PLEKHB1	1	0.826173206	0.835353669
ENSGALG00000040044		32	0.827145343	0.836267261
ENSGALG00000004505	CCDC137	18	0.827734189	0.836793215
ENSGALG00000004341		8	0.828813933	0.837815311
ENSGALG00000042647		11	0.829628776	0.838569482
ENSGALG00000026380	UBE2C	20	0.83103267	0.839918878
ENSGALG00000039203	MOB1A	22	0.8313072	0.840084818
ENSGALG00000002103	TPPP3	11	0.831334652	0.840084818
ENSGALG00000025751	NMRK2	28	0.832482505	0.841175038
ENSGALG00000029509	NECAB1	2	0.833910679	0.842548302
ENSGALG00000006471	SLCO2A1	9	0.834918288	0.843496453
ENSGALG00000014607	STARD3	27	0.838953864	0.84750327
ENSGALG00000041524		2	0.842890679	0.851409664
ENSGALG00000032336		18	0.843225239	0.851677049
ENSGALG00000005763	VILL	2	0.843819931	0.852207109
ENSGALG00000010227	KRR1	1	0.845795571	0.854131638
ENSGALG00000045172	FAM166A	17	0.847904222	0.85619016

ENSGALG00000033696	RALY	20	0.848516784	0.856737756
ENSGALG00000010809	BZW2	2	0.850019913	0.858184383
ENSGALG00000027712	SLAMF8	25	0.851271179	0.85937651
ENSGALG00000006106	TNFRSF6B	20	0.851444521	0.859480342
ENSGALG00000015267	CMSS1	1	0.85255875	0.860533844
ENSGALG00000002587	CRCP	19	0.85266975	0.860574643
ENSGALG00000023395	PLIN1	10	0.854758922	0.86261178
ENSGALG00000030877	CLUAP1	14	0.855452253	0.863240033
ENSGALG00000048579		4	0.855803477	0.863522989
ENSGALG00000010958	PPFIA2	1	0.859388034	0.867068126
ENSGALG00000042967	CTPS1	23	0.859968578	0.867582068
ENSGALG00000054083	H2A-VIII	1	0.860773133	0.8683219
ENSGALG00000029449	RFC2	19	0.860902015	0.868380069
ENSGALG00000028037	FOS	5	0.864308387	0.871743913
ENSGALG00000039311	SMAGP	33	0.864931617	0.872300348
ENSGALG00000037828	SLC47A1	19	0.866393146	0.873702062
ENSGALG00000010662		4	0.866644939	0.873883703
ENSGALG00000043439	NSMCE2	2	0.867284403	0.874456192
ENSGALG00000052399		14	0.867462446	0.874563387
ENSGALG00000005554	ADIPOQ	9	0.869429391	0.876473961
ENSGALG00000009093	PNO1	3	0.871507636	0.878496413
ENSGALG00000026448	FAM96A	10	0.871877292	0.878796381
ENSGALG00000024428	C18H17ORF67	18	0.872618314	0.879470582
ENSGALG00000017097	MTIF3	1	0.873495911	0.880282308
ENSGALG00000043257		1	0.874687913	0.88141072
ENSGALG00000015849	ME1	3	0.877354934	0.884025181
ENSGALG00000033314		19	0.877468617	0.884066671
ENSGALG00000005126	DHX40	19	0.879411658	0.885951115
ENSGALG00000041435		25	0.880388963	0.886862411
ENSGALG00000035462	IREB2	10	0.88075067	0.887153484
ENSGALG00000042148	FKBP5	26	0.882619688	0.888962651
ENSGALG00000045876		4	0.882860279	0.889131525
ENSGALG00000010555	OSBPL9	8	0.885508988	0.891725396

ENSGALG00000027742		28	0.885746157	0.891890569
ENSGALG00000020943	AGXT	9	0.88621388	0.892287848
ENSGALG00000051431	RPS15A	14	0.887949366	0.893961408
ENSGALG00000028496		24	0.888345689	0.894219357
ENSGALG00000002766	MLKL	11	0.888352257	0.894219357
ENSGALG00000000994		23	0.890742979	0.896551853
ENSGALG00000033572	SORT1	26	0.891281251	0.897019587
ENSGALG00000000529	LIMD2	27	0.892362283	0.898033453
ENSGALG00000006796	SRPX2	4	0.893022297	0.898623493
ENSGALG00000035201	HSPA14	1	0.894122135	0.899598345
ENSGALG00000034078	GLT8D2	1	0.894138632	0.899598345
ENSGALG00000001214	PARD6A	11	0.894562094	0.899950133
ENSGALG00000003921	DBNDD2	20	0.895587153	0.900907033
ENSGALG00000028451	MT4	11	0.895933711	0.901181301
ENSGALG00000015685	CFAP99	4	0.896034332	0.901208167
ENSGALG00000049411	DNALI1	23	0.896506535	0.901608725
ENSGALG00000006997	BRINP1	17	0.898186409	0.903223661
ENSGALG00000004161	CCNB1	10	0.898288544	0.903251873
ENSGALG00000029401	SIX1	5	0.900424076	0.905324545
ENSGALG00000008454	NOP58	7	0.902124695	0.906959631
ENSGALG00000051645	CDC6	27	0.902897094	0.90766133
ENSGALG00000052980		22	0.905446491	0.910149139
ENSGALG00000007520	SSRP1	5	0.905621014	0.910249527
ENSGALG00000024031	CYB561	27	0.907507342	0.912070311
ENSGALG00000017302	SLCO2B1	1	0.908942432	0.913437325
ENSGALG00000009641	COL1A2	2	0.909976049	0.914400689
ENSGALG00000046731		22	0.91094663	0.915300557
ENSGALG00000052786		4	0.911318841	0.915599096
ENSGALG00000036976	DALRD3	12	0.912563879	0.91677444
ENSGALG00000022557	CLDN3	19	0.913104636	0.917242118
ENSGALG00000032278	ATF6	8	0.913637484	0.917701775
ENSGALG00000050075	TMEM35A	4	0.914874171	0.918868268
ENSGALG00000010789	PARS2	8	0.915858737	0.919781367

ENSGALG00000053025		13	0.919485855	0.923347968
ENSGALG00000040307	MORN2	3	0.921972649	0.925747168
ENSGALG00000032955	TUFT1	25	0.922026869	0.925747168
ENSGALG00000000604		23	0.922286776	0.925931878
ENSGALG00000001442	OPCML	24	0.922691722	0.926262158
ENSGALG00000004195	KIF4B	4	0.923271325	0.926767701
ENSGALG00000047182	ACHE	31	0.924998047	0.92842453
ENSGALG00000037087	RPF2	3	0.926030249	0.92938405
ENSGALG00000015528	JAKMIP1	4	0.926253691	0.929531791
ENSGALG00000041972	POP4	11	0.928628472	0.931838282
ENSGALG00000008839	ATP11B	9	0.929640258	0.9327768
ENSGALG00000043254	EPX	19	0.93084489	0.933908643
ENSGALG00000006922		24	0.931105185	0.934092934
ENSGALG00000029072	NTN3	14	0.93873485	0.941669603
ENSGALG00000004483	AHSA2	3	0.938981997	0.941840037
ENSGALG00000029617	COL17A1	6	0.939642963	0.942425488
ENSGALG00000003795	DENR	15	0.940131164	0.942837578
ENSGALG00000039989	GPR160	9	0.943141279	0.945778569
ENSGALG00000054117		25	0.94450703	0.947070248
ENSGALG00000011369	LHX8	8	0.945470556	0.947958432
ENSGALG00000001319	CAMK1G	26	0.949841159	0.952262231
ENSGALG00000014096	ARNTL2	1	0.951307162	0.953653558
ENSGALG00000031863		2	0.952822218	0.955093827
ENSGALG00000004184	SPARC	13	0.953012984	0.95520652
ENSGALG00000015860	UBE3D	3	0.953305784	0.955421456
ENSGALG00000038801	ATP5B	33	0.955689898	0.957732138
ENSGALG00000030237	PITX1	13	0.960549841	0.962523358
ENSGALG00000034344	MTHFD2	22	0.962340584	0.964238536
ENSGALG00000028023	LRRC74B	15	0.963504482	0.965325403
ENSGALG00000016473	OSR1	3	0.964359965	0.966103118
ENSGALG00000006307	NSMCE1	14	0.96616669	0.96783359
ENSGALG00000036831		32	0.966769671	0.968358055
ENSGALG00000005063	ST6GALNAC4	17	0.968577863	0.970089526

ENSGALG00000004357	ANXA6	13	0.972502294	0.97394008
ENSGALG00000028560	OC3	1	0.973983489	0.975343354
ENSGALG00000000579	VAMP3	21	0.975841182	0.977123391
ENSGALG00000013733	GTF2H5	3	0.977002433	0.978205836
ENSGALG00000011637	TCP1	3	0.978095617	0.979219957
ENSGALG00000034294	ATP6V0D2	2	0.979742599	0.980788301
ENSGALG00000005290	TRMT13	8	0.980475435	0.981441341
ENSGALG00000007305	NME1	18	0.980955034	0.981840809
ENSGALG00000028978	BTBD19	8	0.98180905	0.982614934
ENSGALG00000004362	MRPL21	5	0.981933733	0.982659062
ENSGALG00000028551		3	0.982040785	0.982685539
ENSGALG00000027369	DGCR6	15	0.98242875	0.982993086
ENSGALG00000002500	GMPPB	12	0.983526132	0.984010349
ENSGALG00000000824		27	0.984508814	0.984912699
ENSGALG00000005284	LRRC39	8	0.991596874	0.991922281
ENSGALG00000015892	IRAK1BP1	3	0.991923774	0.992167889
ENSGALG00000010258	LTBP2	5	0.992165402	0.992328172
ENSGALG00000006633	TMEM243	1	0.992603419	0.992684833
ENSGALG00000037671		1	0.994530961	0.994530961

Protein accession	Protein description	Gene name	MW [kDa]
E1C619	ATP-binding cassette transporter 1(pr	ABCA1	254.08
E1BVR2	Zinc finger protein 777-like(predicted	LOC112531692	63.871
F1P088	Perilipin 1(predicted) OS=Gallus gall	PLIN1	56.106
A0A1D5PDS3	Heterogeneous nuclear ribonucleopro	HNRNPH2	51.4
P02467	Collagen alpha-2(I) chain OS=Gallus	COL1A2	128.99
F6UW59	Apolipoprotein AIV(predicted) OS=C	APOA4	40.682
A0A1D5PKQ4	Collagen alpha-1(III) chain OS=Gallu	COL3A1	132.44
A0A1D5PYU1	Collagen alpha-1(I) chain OS=Gallus	COL1A1	137.78
E1C5A9	Cytochrome P450 2H2(predicted) OS	CYP2C23b	56.444
A0A1D5NYF3	Adipocyte-type fatty acid-binding pro	FABP4	14.921
E1BXY1	TBC1 domain family member 4(predi	TBC1D4	108.83
A0A1D5P9C9	SH3 domain binding protein 5(predic	SH3BP5	46.969
F7BYG6	Perilipin OS=Gallus gallus OX=9031	PLIN2	49.019
A0A3Q2UF48	Peptidase S1 domain-containing prote	--	20.38
A0A3Q2TXV2	CAP-Gly domain-containing protein (	LOC417131	295.81
R4GHD4	CIDE-N domain-containing protein C	CIDEC	26.309
A0A1L1RWX6	4HBT domain-containing protein OS=	ACOT13	15.486
E1BRR4	Scavenger receptor class A member 5	SCARA5	53.34
Q9PTG6	Dynactin subunit 2 OS=Gallus gallus	DCTN2	45.125
A0A3Q2U0F9	Reverse transcriptase domain-contain	--	52.429
P08287	Histone H1.11L OS=Gallus gallus OX	--	22.528
A0A3Q2UB57	Putative LOC102087994(predicted) (	--	93.171
A0A1D5P6W1	Collagen type V alpha 2 chain(predic	COL5A2	141.72
A0A3Q2U9F3	FAT atypical cadherin 4(predicted) O	FAT4	519.86
Q6PVZ5	Type II alpha-keratin IIA OS=Gallus	KRT5	62.461
F1NII7	Fibrillin 1(predicted) OS=Gallus gallu	FBN1	311.03
F1P4I7	CN hydrolase domain-containing prot	VNN1	55.314
Q6Q126	Thyroid hormone responsive OS=Gal	THRSP	14.186

E1BTT4	Hydroxyacyl-CoA dehydrogenase trif	--	50.849
F1NWS4	Aryl hydrocarbon receptor 1 beta(pre	LOC100859347	105.67
A0A3Q2UGD4	Ig-like domain-containing protein OS	--	10.5
A0A3Q2UGT8	2-iminobutanoate/2-iminopropanoate	RIDA	29.815
A0A1D5NW68	Albumin OS=Gallus gallus OX=9031	ALB	64.004
Q95601	MHC class I alpha chain 2 OS=Gallu	BFIV21	38.959
A0A3Q2UDK3	Rho guanine nucleotide exchange fac	ARHGEF6	80.766
F1NL38	Histidine-rich glycoprotein(predicted)	HRG	123.03
A0A3Q2U3N8	Gamma-glutamylaminecyclotransfera	GGACT	17.987
E1C0E3	ADAM metallopeptidase with thromb	ADAMTS19	133.02
F1NC49	AA_TRNA_LIGASE_II_ALA domai	AARSD1	45.395
E1BZE1	Alpha 2-HS glycoprotein(predicted) (	AHSG	37.2
A0A3Q2TYH6	Ig-like domain-containing protein OS	--	10.523
A0A3Q2UH20	Ig-like domain-containing protein OS	--	26.629
R4GLI9	Flavodoxin_2 domain-containing pro	NQO2	26.771
P80566	Superoxide dismutase [Cu-Zn] OS=G	SOD1	15.703
F1NG65	Nucleoside diphosphate kinase OS=G	NME4	20.646
P19121	Albumin OS=Gallus gallus OX=9031	ALB	69.918
A0A547	Trifunctional purine biosynthetic prot	GART	106.53
A0A1D5PJ72	Capping protein inhibiting regulator c	--	161.31
A0A1D5P9M6	Stromal cell derived factor 2(predicte	SDF2	23.279
D5M8S4	E3 ubiquitin-protein ligase RNF170 (	RNF170	29.629
A0A3Q2UIH0	CSD domain-containing protein OS=	CARHSP1	16.484
A0A3Q2UD12	Collagen alpha-3(VI) chain OS=Gallu	COL6A3	326.28
A0A3Q2U8S7	ANK_REP_REGION domain-contai	ANKRD28	113.15
A0A3Q2U578	Histone H3 OS=Gallus gallus OX=90	--	10.927
A0A1D5PNX5	Granulin precursor OS=Gallus gallus	--	30.264
A0A3Q2U5V5	Ig-like domain-containing protein OS	--	9.9678
P27731	Transthyretin OS=Gallus gallus OX=	TTR	16.309
E1C1B4	Galactokinase 1(predicted) OS=Gallu	GALK1	41.595
F1NI29	Enoyl-CoA hydratase OS=Gallus gall	HADHA	82.986
A0A1D5PWW6	Zinc finger AN1-type containing 2B(	LOC100858626	51.748
A0A1L1RWG9	IF rod domain-containing protein OS	KRT24	46.038

R4GG24	Aldo_ket_red domain-containing prot	AKR1E2	35.808
F1NC38	Long-chain specific acyl-CoA dehydr	ACADL	47.873
P81476	Ribonuclease CL2 OS=Gallus gallus	--	13.433
A0A1L1RUM8	Peptidase S1 domain-containing prote	CTSG	27.082
Q5ZL43	1,2-dihydroxy-3-keto-5-methylthiope	ADI1	21.674
Q5ZIY8	Ribonuclease inhibitor OS=Gallus ga	RNH1	49.863
Q8UWG7	60S ribosomal protein L6 OS=Gallus	RPL6	33.923
A0A1D5PC99	Electron transfer flavoprotein subunit	ETFA	34.217
Q5ZL56	Acyl-CoA dehydrogenase short chain	ACADS	44.614
E1BQP0	Protein phosphatase OS=Gallus gallu	PPTC7	27.13
Q6IEC5	Putative ISG12(2) protein OS=Gallus	ISG12(2)	10.281
A0A1L1RSZ1	Ribosomal protein L7 like 1(predicted	RPL7L1	26.264
A0A1D5PUK4	Protein ADP-ribosylarginine hydrolas	ADPRHL	38.411
A0A3Q2TWT6	Pericentriolar material 1 protein OS=(	PCM1	219.69
A0A1D5PZS5	Mucin-5AC (Fragment)(predicted) O	--	373.02
E1C4C6	Calponin-homology (CH) domain-con	SMTNL2	50.783
Q5ZLP2	Methylthioribulose-1-phosphate dehy	APIP	26.821
F1NHI4	Sod_Cu domain-containing protein O	SOD3	34.133
A0A1L1S0X5	60S ribosomal protein L34 OS=Gallu	RPL34	13.306
E1C5Y3	Rap-GAP domain-containing protein	RALGAPA1	237.45
A0A3Q2TUG3	TGF_BETA_2 domain-containing pro	INHBE	38.128
Q5ZLI7	UBC core domain-containing protein	UBE2H	20.655
A0A3Q3AAG5	Transcription elongation factor A3(pr	TCEA3	70.385
A0A1D5P6S2	FAA_hydrolase domain-containing pr	FAHD2A	32.872
E1C0Q5	Acetyl-CoA acetyltransferase 1(predi	ACAT1	44.09
E1C4M3	Cathepsin X OS=Gallus gallus OX=9	CTSZ	33.853
F1NT28	Inorganic diphosphatase OS=Gallus g	PPA1	32.637
D5M8S2	Maillard deglycase OS=Gallus gallus	DJ-1	19.931
F1NMF8	Teashirt zinc finger homeobox 3(prec	TSHZ3	118.53
Q6Q123	THRSP-like protein OS=Gallus gallu	MID1IP1	17.726
P40618	High mobility group protein B3 OS=(	HMGB3	23.057
A0A1D5PUX7	GPI ethanolamine phosphate transfera	PIGG	79.344
A0A3Q2U150	Acyl-CoA dehydrogenase medium ch	--	146.84

A0A3Q3AIA0	Ig-like domain-containing protein OS	--	10.562
A0A1L1RRA5	Superoxide dismutase [Cu-Zn] OS=G	SOD1	15.689
E1BXI0	Gamma-butyrobetaine hydroxylase O	BBOX1	49.425
A0A1D5PE74	Fibrillar collagen NC1 domain-contai	COL5A1	184.18
Q5ZIK2	PDZ domain-containing protein 11 O	PDZD11	15.831
A0A1D5PWT4	Thioredoxin domain-containing prote	TXN2	16.259
A0A3Q2UDS8	Sulfiredoxin OS=Gallus gallus OX=9	SRXN1	16.275
A0A3Q2TZX3	Nudix hydrolase domain-containing p	NUDT15	18.06
P84247	Histone H3.3 OS=Gallus gallus OX=	H3-IX	15.328
A0A3Q3ASF8	Dynein heavy chain 9, axonemal (Fra	--	91.242
P08288	Histone H1.11R OS=Gallus gallus O	--	21.803
A0A3Q2U0X4	C2 domain-containing protein OS=Ga	--	57.018
A0A3Q2U4T4	F-box and WD repeat domain contair	FBXW5	63.979
P08250	Apolipoprotein A-I OS=Gallus gallus	APOA1	30.68
A0A3Q3B0H9	Dolichyl-phosphate-mannose--protein	TMTC1	102.48
F1NUY1	Protein kinase domain-containing pro	DAPK2	40.986
R4GMD9	Thioredoxin domain-containing prote	TXNDC17	14.228
E1BWM3	Cor1 domain-containing protein OS=	SYCP3	27.792
A0A1D5PHZ5	Carboxypeptidase Q OS=Gallus gallu	CPQ	51.97
A0A1D5P3Y4	KOW domain-containing protein OS=	MRPL24	24.995
Q91955	Myotrophin OS=Gallus gallus OX=90	MTPN	12.886
F1NLF7	RING-type domain-containing protein	RNF11	17.416
A0A1D5P1U2	Pyruvate dehydrogenase E1 compone	PDHB	38.877
A0A1D5PQN5	IF rod domain-containing protein OS=	KRT7	53.763
Q05423	Fatty acid-binding protein, brain OS=	FABP7	14.927
F1P310	Ubiquinone biosynthesis protein OS=	COQ9	35.149
P21566	Cofilin-2 OS=Gallus gallus OX=903	CFL2	18.661
A0A3Q2U3V9	Putative LOC102086700(predicted) (	LOC100858647	12.421
A0A3Q2U5M7	Ribulose-phosphate 3-epimerase OS=	RPEL1	37.028
F1P1P5	Frataxin intermediate form OS=Gallu	FXN	18.51
F1P3Q4	SWI/SNF related, matrix associated, a	SMARCA1	117.82
A0A1D5PNU2	Apolipoprotein H OS=Gallus gallus (	APOH	41.093
A0A1L1RNR4	Kininogen 1(predicted) OS=Gallus ga	KNG1	62.02

A0A1D5P4E3	Phosphatase and actin regulator OS=(	PHACTR3	62.017
A0A3Q2U8F2	Ig-like domain-containing protein OS	--	10.222
E1BW57	Nudix hydrolase domain-containing p	NUDT5	24.57
E1C4V1	ATP synthase-coupling factor 6, mito	ATP5J	12.36
A0A3Q2U9M3	Ig-like domain-containing protein OS	--	9.7115
A0A1D5P2V1	Tetratricopeptide repeat protein 38 O	TTC38L	31.706
A0A1I7Q419	40S ribosomal protein S12 OS=Gallu	RPS12	13.529
P02612	Myosin regulatory light chain 2, smoo	--	19.845
A0A3Q3AKX3	Ig-like domain-containing protein OS	--	10.508
P07031	Acylphosphatase-2 OS=Gallus gallus	ACYP2	11.409
A0A1D5PRI6	NEDD8 OS=Gallus gallus OX=9031	NEDD8	9.591
F1NZ86	75 kDa glucose-regulated protein OS=	HSPA9	73.161
E1BT15	NEDD4-binding protein 2-like 1(prec	N4BP2L1	25.026
Q5ZK69	Proteasomal ATPase-associated facto	PAAF1	42.373
F1NHL4	Cysteinylglycine-S-conjugate dipeptic	LAP3	58.451
A0A1D5PFM8	Mitochondrial ribosomal protein L32(	MRPL32	20.572
E1BXR1	Prefoldin subunit 3 OS=Gallus gallus	LOC772071	18.737
A0A3Q2U3L5	NADB-LER4(predicted) OS=Gallus g	LOC101747680	15.766
A0A1D5PKG1	AB hydrolase-1 domain-containing pr	ABHD4	43.793
A0A1L1RTR9	Mitochondrial ribosomal protein L12(	MRPL12	20.471
A0A3Q2U7J0	Protein FAM136A OS=Gallus gallus	--	107.83
F1NJF0	Complement component 1 Q subcom	C1QBP	24.308
F1NJ37	Myosin light chain, embryonic(predic	MYL4	20.652
A0A3Q2UJX6	N-acetylglucosamine-6-phosphate dea	AMDHD2	40.63
A0A1L1RWR0	Transthyretin OS=Gallus gallus OX=	LOC112531842	16.083
F1P0W7	Mitochondrial pyruvate carrier OS=G	MPC1L	12.406
E1BX85	Glutathione-dependent dehydroascorb	GSTO2	27.223
P00940	Triosephosphate isomerase OS=Gallu	TPI1	26.62
A0A1L1RR71	Delta-1-pyrroline-5-carboxylate synth	ALDH18A1	87.386
E1C8W4	Ubiquitin carboxyl-terminal hydrolase	USP5	93.227
Q09121	Eukaryotic translation initiation facto	EIF5A1	15.889
A0A1D5PEN4	WD_REPEATS_REGION domain-co	STXBP5L	108.04
A0A1D5P6V5	Perilipin OS=Gallus gallus OX=9031	PLIN4	45.388

A0A1D5P4Y0	Tubulin polymerization promoting pr	TPPP	23.956
E1BQI7	[Heparan sulfate]-glucosamine N-sulf	NDST1	100.89
F1NV65	WD_REPEATS_REGION domain-co	DMXL1	338
A0A1D5PNG3	Eukaryotic translation initiation facto	EIF6	26.525
Q90979	Transcription factor OS=Gallus gallus	IBR	53.579
F1NT19	Superoxide dismutase OS=Gallus gal	SOD2	24.859
A0A3Q3AHU9	Phosphohistidine phosphatase(predict	LOC424401	13.06
P23668	16 kDa beta-galactoside-binding lecti	--	14.934
E1C4J8	Interferon induced protein 35(predicte	IFI35	42.884
E1C2N4	Nucleolar autoantigen 36 OS=Gallus	ZNF330	35.776
Q5ZM14	Na(+)/H(+) exchange regulatory cofa	SLC9A3R1	35.805
A0A1D5PRJ0	TPR_REGION domain-containing pr	TTC32	16
Q2I811	NK-lysin OS=Gallus gallus OX=9031	GNLY	15.231
Q5ZL27	mRNA processing body assembly fac	LSM14A	50.309
Q5ZIK4	Protein yippee-like OS=Gallus gallus	YPEL5	13.841
A0A452J805	Tropomyosin beta chain OS=Gallus g	TPM2	37.741
P41366	Vitelline membrane outer layer protei	VMO1	20.234
D0VX28	Cytochrome b-c1 complex subunit 6 (	UQCRHL	9.04
E1BXF4	Reticulocalbin-1(predicted) OS=Gallu	RCN1	37.873
E1BVP5	Aspartoacylase(predicted) OS=Gallus	ASPA	40.923
E1BW06	ACB domain-containing protein OS=	ECI2	44.414
F1NDH2	Angiotensin 1-10 OS=Gallus gallus O	AGT	51.346
A0A3Q2TTC1	Collagen IV NC1 domain-containing	COL4A2	182.5
A0A1D5PBU7	Secernin 2(predicted) OS=Gallus gall	SCRN2	46.756
A0A1D5PK48	Apolipoprotein C-III OS=Gallus gallu	APOC3	10.26
E1C043	Cytochrome c oxidase polypeptide Va	COX5A	15.968
E1BUA6	CN hydrolase domain-containing prot	VNN1	56.558
A0A3Q3A4J0	Ketohexokinase(predicted) OS=Gallu	KHK	31.637
Q6J211	E3 ubiquitin-protein ligase RNF114 (	RNF114	25.224
A0A3Q2TVK1	Sep15_SelM domain-containing prote	SELENOM	16.946
R4GFY1	Mediator of RNA polymerase II trans	MED10	16.783
A0A3Q2TXL5	Four and a half LIM domains 1(predi	FHL1	32.391
A0A3Q3AV59	Ig-like domain-containing protein OS	--	10.323

F1NY85	60S ribosomal protein L8 OS=Gallus	MRPL2	31.71
A0A1L1RYE4	C-type lectin domain-containing prote	PGR2/3	17.282
A0A3Q2UCP8	ACB domain-containing protein OS=	ACBD7	9.5809
F1N8X2	Lamin-L(III) (Fragment)(predicted) C	LMINA	51.657
P24032	Myosin regulatory light chain 2, smoo	--	19.911
O42283	10 kDa heat shock protein, mitochond	HSPE1	11.083
A0A1L1S091	Phosphatidylethanolamine-binding pr	PEBP1	20.729
A0A1D5PG19	Succinyl-CoA:glutarate-CoA transfer	SUGCT	45.344
P41263	Retinol-binding protein 4 OS=Gallus	RBP4	22.515
A0A3Q3A521	JAKMIP_CC3 domain-containing pro	JAKMIP2	90.334
F1NMS1	Mitochondrial transcription rescue fac	C3H6ORF203	26.514
F1NWF3	FGE-sulfatase domain-containing pro	SUMF2	35.08
Q9PRL8	Acyl-CoA-binding protein OS=Gallus	DBI	9.6448
E1BZE6	Heterogeneous nuclear ribonucleopro	HNRNPA3	39.54
A0A1D5P7X8	Dedicator of cytokinesis 8(predicted)	DOCK8	221.2
Q5ZKL7	Bridging integrator 2 OS=Gallus gallu	BIN2	51.179
A0A1L1RQD3	HD domain-containing protein OS=G	LOC107056801	20.66
Q07883	Growth factor receptor-bound protein	GRB2	25.076
Q98TF7	60S ribosomal protein L35 OS=Gallu	RPL35	14.551
F1NIX0	60S ribosomal protein L8 OS=Gallus	RPL8	28.022
A0A3Q3AUG8	Calumenin(predicted) OS=Gallus gal	LOC107050717	19.493
A0A1L1RZK1	Lactoylglutathione lyase OS=Gallus g	GLO1	20.553
P20763	Ig lambda chain C region OS=Gallus	--	11.361
A0A1D5NXY4	Barrier to autointegration factor 1(pre	BANF1	10.057
A0A1D5P8C3	AP_endonuc_2 domain-containing pr	HYI	43.985
P18660	60S acidic ribosomal protein P1 OS=	RPLP1	11.477
F1NIK4	Ribosomal protein L36a like(predicte	RPL36A	12.285
E1BVT3	Malate dehydrogenase OS=Gallus gal	MDH2	35.655
F1NBX1	Gluconolactonase OS=Gallus gallus (	RGN	33.215
A0A1D5P3I6	Peptidylprolyl isomerase OS=Gallus g	FKBP3	62.84
F1NNS8	Thioredoxin domain-containing prote	PRDX4	29.634
F1NHT5	Fetuin B(predicted) OS=Gallus gallus	FETUB	44.137
F1NVH2	Tubulin-specific chaperone A OS=Ga	TBCA	12.707

F1NEL0	Calcium uniporter regulatory subunit	MCUB	45.789
F1N857	J domain-containing protein OS=Gall	HSCB	26.62
A0A1D5PLH7	Thioredoxin domain-containing prote	PRDX4	29.899
Q5ZLQ3	WD_REPEATS_REGION domain-co	AAMP	45.643
A0A1D5PCY8	Dynactin subunit 1 OS=Gallus gallus	DCTN1	146.62
A0A3Q2U554	Hydroxyacylglutathione hydrolase, m	HAGH	33.435
P67966	Cysteine and glycine-rich protein 1 O	CSRP1	20.385
F1NB64	Acetyl-CoA acyltransferase 1(predicte	ACAA1	44.607
P32429	60S ribosomal protein L7a OS=Gallu	RPL7A	29.999
F1P1W1	PPFIA binding protein 2(predicted) C	PPFIBP2	105.18
F1NC46	U1-type domain-containing protein O	SCAPER	154.54
A0A1D5PMD9	Carboxylic ester hydrolase OS=Gallu	LOC769704	61.905
E1C6N5	Acetyltransferase component of pyruv	DLAT	66.522
A0A1D5NU37	Secernin 3(predicted) OS=Gallus gall	SCRN3	46.816
F1NG43	CMD domain-containing protein OS=	LOC768709	27.232
R4GHN1	Arachidonate 5-lipoxygenase activati	ALOX5AP	17.906
Q5ZL37	Succinate--CoA ligase [GDP-forming	SUCLG2	46.76
O57415	Ras-responsive element-binding prote	RREB1	178.39
A0A3Q2U1W1	DDB1 and CUL4 associated factor 8	--	26.034
F1NGJ4	PACT_coil_coil domain-containing p	PCNT	405.81
E1C039	Pleckstrin homology, MyTH4 and FE	PLEKHH2	168.92
Q5ZLV2	Peptidyl-prolyl cis-trans isomerase-lil	PPIL3	18.051
A0A1D5PZU7	Peptidyl-prolyl cis-trans isomerase O	PIN1	18.101
E1BRQ9	TFIIS N-terminal domain-containing	IWS1	77.804
A0A1D5PYL3	Katanin p60 ATPase-containing subu	KATNAL2	60.215
F1NBV0	Peroxiredoxin-6 OS=Gallus gallus O	PRDX6	25.076
A0A3Q2TYN8	Microtubule associated scaffold prote	MTUS1	51.203
A0A1D5PH20	Protein SMG8 OS=Gallus gallus OX=	SMG8	71.642
F1NLC7	Thioredoxin domain-containing prote	TXNDC12	18.562
P11183	Glycine cleavage system H protein, m	GCSH	18.01
F1NSB1	Alpha-aspartyl dipeptidase(predicted)	LOC424109	27.227
A0A1D5PYK0	Heat shock cognate 71 kDa protein O	HSPA8	69.114
Q5ZLF0	Hsc70-interacting protein OS=Gallus	ST13	40.158

A0A3Q2TXI8	Mediator complex subunit 28 OS=Ga	MED28	19.525
A0A1D5PDA1	Enoyl-CoA hydratase 1(predicted) OS	ECH1	56.388
A0A1D5PFS5	Maturin OS=Gallus gallus OX=9031	--	14.965
A0A1D5P8B8	PKS_ER domain-containing protein (	CRYZL1	38.717
Q5ZJ00	55 kDa erythrocyte membrane protein	MPP1	52.519
A0A1D5PHB1	HEXIM P-TEFb complex subunit 1 (	--	32.816
E1BSM1	[Heparan sulfate]-glucosamine N-sulf	NDST4	100.71
A0A1D5P926	ANK_REP_REGION domain-contair	GIT1	77.59
F1NIK8	SAM domain-containing protein OS=	STIM2	83.079
A0A3Q2U4P4	Ubiquitin carboxyl-terminal hydrolase	USP5	88.376
Q5ZJX7	Multivesicular body subunit 12A OS=	MVB12A	28.062
A0A3Q2UH03	Ig-like domain-containing protein OS	--	10.205
E1BZH9	Hydroxyacyl-CoA dehydrogenase(pre	HADH	34.421
A0A1D5PL36	Acetyl-CoA acyltransferase 2(predicte	ACAA2	44.293
P13538	Myosin heavy chain, skeletal muscle,	--	223.14
E1C1R8	Musashi RNA binding protein 2(pred	MSI2	36.985
A0A1L1RPR4	Aspartate aminotransferase OS=Gallu	GOT2	47.986
E1C818	H/ACA ribonucleoprotein complex su	GAR1	22.534
F1P4Q4	Cytoplasmic protein OS=Gallus gallu	NCK1	42.999
A0A3Q3AL57	Beta-1,4-galactosyltransferase(predict	B4GALT1	60.096
A0A1D5PTE2	Dynactin subunit 2 OS=Gallus gallus	DCTN2	44.579
P31335	Bifunctional purine biosynthesis prote	ATIC	64.414
A0A3Q2UKA8	Ubiquitin-associated domain-containi	UBAC1	41.749
A0A3Q2U3X0	LRRCT domain-containing protein O	LOC101750889	76.937
F1NFS8	NADB-LER1 OS=Gallus gallus OX=	LOC100857820	27.387
E1BUG9	CS domain-containing protein OS=G	NUDCD2	17.8
E1C0G0	Ubiquitin-like domain-containing pro	TBCEL	48.33
A0A1D5PH14	Dynein light chain roadblock OS=Ga	DYNLRB1	10.78
A0A3Q2UEG8	A2M domain-containing protein OS=	--	16.869
F1NG62	Nucleotide binding protein like(predi	NUBPL	34.664
F1NIM4	Peptidylprolyl isomerase OS=Gallus g	FKBP4	50.445
Q5ZMH8	AFP-like domain-containing protein (	NANS	40.144
F1NQI0	Aspartylglucosaminidase(predicted) (	AGA	36.604

P43347	Translationally-controlled tumor prot	TPT1	19.53
E1BS15	Acyl-CoA synthetase family member	ACSF2	65.311
E1BSK7	VOC domain-containing protein OS=	MCEE	19.869
Q5ZIE2	Eukaryotic translation initiation facto	EIF1AX	16.442
A0A3Q2UMV5	DNA helicase OS=Gallus gallus OX=	MCM3	89.219
E1C837	WD_REPEATS_REGION domain-co	STRN	85.073
A0A1L1RY04	ATP synthase subunit beta OS=Gallu	ATP5B	68.409
A0A1D5PET4	Cytoplasmic polyadenylation element	CPEB4	77.368
R4GIU0	Endonuclease G(predicted) OS=Gallu	ENDOG	22.176
Q2KN97	Cytospin-A OS=Gallus gallus OX=9(	SPECC1L	124.91
A3R0S3	Growth factor receptor-bound protein	GRB2	25.177
Q5ZL83	Succinate--CoA ligase [ADP/GDP-fo	SUCLG1	34.849
F1NM89	TatD DNase domain containing 3(pre	TATDN3	26.965
R4GJC4	dTMP kinase OS=Gallus gallus OX=	CMPK2	24.705
A0A1D5PTB3	GFO_IDH_MocA domain-containing	DHDH	42.606
F1NVV8	RWD domain-containing protein OS=	RWDD1	27.982
A0A1D5NW93	ATP synthase F1 subunit delta OS=G	ATP5D	16.817
E1C843	Integrator complex subunit 12 OS=Ga	INTS12	48.739
A0A1D5PUL8	Dihydrolipoamide acetyltransferase c	PDHX	53.189
E1BWS9	HYPK_UBA domain-containing prot	HYPK	14.111
A0A3Q3AFL8	Protein phosphatase 1 regulatory subu	LRRCC1	111.29
E1C810	Ribosomal protein L37 OS=Gallus ga	RPL37	11.07
A0A1D5PH07	AIR carboxylase OS=Gallus gallus O	PAICS	93.958
P67881	Cytochrome c OS=Gallus gallus OX=	CYC	11.71
Q5ZKU4	Integrator complex subunit 2 OS=Gal	INTS2	132.88
A0A1D5PTS6	Protein-L-isoaspartate O-methyltransf	PCMT1L	26.768
A0A1D5PS08	c-Myc-binding protein(predicted) OS	MYCBP	13.107
A0A1D5NZY6	Adenosine kinase OS=Gallus gallus (	ADK	38.561
E1C230	Charged multivesicular body protein	CHMP3	24.526
F1NY09	DUF1907 domain-containing protein	C1H11ORF54	34.679
F1P4A5	TPR_REGION domain-containing pr	CDC16	72.409
A0A3Q2U0Q2	HMA domain-containing protein OS=	RBP14L	28.068
A0A3Q2TUH0	Pyridoxal kinase OS=Gallus gallus O	PDXK	34.958

Q5ZL72	60 kDa heat shock protein, mitochonc	HSPD1	60.972
A0A1D5NTZ7	Mitochondrial ribosomal protein L27	MRPL27	15.048
A0A1D5PKC2	dTMP kinase OS=Gallus gallus OX=	DTYMK	24.082
F1NSS4	Acetyl-CoA C-myristoyltransferase C	SCP2	57.509
E1BWS2	NSF attachment protein gamma(predi	NAPG	35.066
A0A1L1RXA3	Ubiquitin carboxyl-terminal hydrolase	UCHL3	26.288
E1C7A7	SMB domain-containing protein OS=	VTN	51.65
A0A3Q2TXL9	Stress induced phosphoprotein 1(pred	STIP1	62.058
R4GIL5	Metalloproteinase inhibitor 2 OS=Ga	TIMP2	24.184
A0A1L1RQQ0	2-phospho-D-glycerate hydro-lyase O	ENO1	47.332
Q5ZJX9	Proteasome subunit alpha type OS=G	PSMA5	26.447
A0A1D5PK63	PX domain-containing protein OS=G	SNX12	18.93
A0A1D5P8P3	Collagen IV NC1 domain-containing	COL4A1	160.77
F1NIC5	Mitochondrial import inner membran	TIMM8A	10.686
F1NPA3	ARID domain-containing protein OS=	ARID4A	141.75
A0A1D5P2Y6	PBX homeobox interacting protein 1(	PBXIP1	67.263
A0A1D5NX25	Heterogeneous nuclear ribonucleopro	HNRNPAB	39.267
A0A1L1RK74	Exonuclease domain-containing prote	REXO2	26.65
A0A1D5NY06	PABS domain-containing protein OS	SRM	33.509
Q5ZMJ0	Peptidyl-prolyl cis-trans isomerase O	PPIF	21.707
A0A1L1S0P1	PIT 54(predicted) OS=Gallus gallus (	PIT54	50.847
F1NGU0	Metabolism of cobalamin associated	MMAA	45.921
Q07460	Eukaryotic translation initiation facto	EIF5A2	16.72
F1NHH1	Cystatin domain-containing protein C	CSTB	11.159
A0A1D5PBY3	Thioredoxin domain-containing prote	PRDX3	25.761
Q90593	Endoplasmic reticulum chaperone Bil	HSPA5	72.018
Q5ZJJ9	Osteoclast-stimulating factor 1 OS=G	OSTF1	22.296
E1C658	ATP synthase subunit d, mitochondri	ATP5H	18.343
A0A3Q2UM86	BPTI/Kunitz inhibitor domain-contai	--	6.5726
P11029	Acetyl-CoA carboxylase OS=Gallus g	ACAC	262.72
A0A1D5P9Z1	Low molecular weight cytosolic acid	ACP1	18.085
P32046	60S ribosomal protein L37a OS=Gall	RPL37A	10.247
F1NDP5	Sulfurtransferase OS=Gallus gallus C	TST	32.932

A0A1D5P0U5	Calreticulin OS=Gallus gallus OX=90	CALR	48.106
A0A1D5PT73	Cytoplasmic polyadenylation element	CPEB2	25.649
A0A1D5PML5	Kinesin-like protein OS=Gallus gallu	KIF3C	84.801
Z4YJB8	Actin-depolymerizing factor OS=Gal	DSTN	18.531
P14731	Lamin-B1 OS=Gallus gallus OX=903	LMNB1	66.529
Q5ZKG5	Low molecular weight phosphotyrosi	ACP1	18.196
Q5ZKF5	S-phase kinase-associated protein 1 C	SKP1	18.658
H9L0E7	TPR_REGION domain-containing pr	SGTA	32.265
Q7T048	AH receptor-interacting protein OS=(	AIP	37.509
A0A1D5PR40	60S ribosomal protein L17 OS=Gallu	LOC107049046	21.397
A0A1D5PEY8	Lipocln_cytosolic_FA-bd_dom doma	C8G	22.303
A0A1D5PRM3	39S ribosomal protein L9, mitochond	MRPL9	27.911
A0A1D5P470	Glutathione S-transferase OS=Gallus	LOC107050492	25.449
A0A1D5P8F9	Saposin B-type domain-containing pr	CNPY2	21.189
A0A3Q2UGE4	Mitochondrial ribosomal protein L41	MRPL41	15.188
A0A1L1RXL9	Vimentin OS=Gallus gallus OX=903	VIM	52.677
Q5ZL20	Mitochondrial ribosomal protein S11	MRPS11	19.41
O93481	Chromobox protein (CHCB2) OS=Ga	CBX3	19.777
A0A1L1RK55	GMP_PDE_delta domain-containing	UNC119B	24.464
Q02960	Macrophage migration inhibitory fact	MIF	12.484
F1NWY7	Mitochondrial ribosomal protein L42	MRPL42	17.088
O73930	Pterin-4-alpha-carbinolamine dehydra	PCBD1	11.997
Q5ZIE1	Transcription initiation factor IIE sub	GTF2E2	32.995
Q5ZL50	Profilin OS=Gallus gallus OX=9031	PFN2	15.046
Q5ZHL5	NFU1 iron-sulfur cluster scaffold hor	NFU1	26.017
P15988	Collagen alpha-2(VI) chain OS=Gallu	COL6A2	109.18
A0A1D5PV06	Protein disulfide-isomerase OS=Gallu	P4HB	57.475
A0A3Q2U969	UDP-N-acetylglucosamine pyrophosp	UAP1L1	52.27
F1P2M6	Complement regulatory soluble prote	LOC419851	79.429
P01875	Ig mu chain C region OS=Gallus gall	--	48.173
F1NVF3	Gc-globulin OS=Gallus gallus OX=9	GC	53.716
Q5ZLH8	UBC core domain-containing protein	UBE2G1	19.509
F1NYF5	WD_REPEATS_REGION domain-co	PWP1	55.703

E1BTI7	SMB domain-containing protein OS=	TINAG	52.753
E1BQ58	RRF domain-containing protein OS=(	MRRFP1	28.567
A0A3Q2UBI9	Mitochondrial ribosomal protein L40(	MRPL40	33.205
F1N8G6	SMB domain-containing protein OS=	TINAGL1	51.681
E1BQX1	DUF4097 domain-containing protein	FAM185A	40.449
E1C9J2	Mesencephalic astrocyte derived neur	MANF	20.386
F1NF64	Complement factor I(predicted) OS=(	CFI	67.214
A0A1D5PU39	LRRcap domain-containing protein C	ANP32A	31.513
F1NKG5	Dimethylargininase OS=Gallus gallus	DDAH1	34.323
Q5ZL13	Nucleoside diphosphate-linked moiety	NUDT19	42.122
P02752	Riboflavin-binding protein OS=Gallu	--	27.211
D0EKR3	Peptidyl-prolyl cis-trans isomerase OS	PPIA	17.81
A0A452J879	Non-specific serine/threonine protein	STK11	48.161
A0A1D5PX32	Serine/arginine-rich splicing factor 7(	SRSF7L	28.751
Q9I882	Protein kinase C inhibitor OS=Gallus	chPKCI	13.759
E1BS06	Ribosomal_L23eN domain-containing	RPL23A	17.656
A0A1L1RW22	CTP:phosphoethanolamine cytidylylt	PCYT2	24.375
P08106	Heat shock 70 kDa protein OS=Gallu	--	69.75
F1NLL4	NTF2 domain-containing protein OS=	NUTF2	14.403
P02259	Histone H5 OS=Gallus gallus OX=90	--	20.733
A0A1L1RTK6	Hydroxymethylbilane synthase OS=G	HMBS	37.109
A0A1D5P338	Glutathione reductase OS=Gallus gal	GSR	50.415
A0A3Q2UDK9	C2H2-type domain-containing protein	GTF2H2	41.329
A0A1D5PVF7	Transcription factor SOX-7(predicted	SOX7	41.215
F1P4H4	Thioredoxin domain containing 5(pre	TXNDC5	46.422
A0A1D5PVG1	Spectrin alpha chain, non-erythrocytic	SPTAN1	285.04
P07997	5-aminolevulinate synthase, nonspeci	ALAS1	69.948
F1P5B8	Vacuolar protein-sorting-associated p	VPS36	59.973
Q5ZKQ7	Glucose-induced degradation protein	GID8	26.814
F1P586	Splicing factor SWAP(predicted) OS=	SFSWAP	105.12
F7BYQ2	40S ribosomal protein S6 OS=Gallus	RPS6	28.682
F1NQ69	Molybdenum cofactor sulfurase OS=(	MOCOS	96.957
Q5ZHR7	Clathrin light chain OS=Gallus gallus	CLTA	23.76

A0A1D5PI10	FERM domain-containing protein OS	EPB41L3	125.84
F1P371	PITH domain-containing protein OS=	PITHD1	23.191
E1BXE9	Prefoldin subunit 6-like protein (Frag	--	60.646
A0A1D5PUI6	Cytochrome c oxidase polypeptide VI	LOC107049545	13.984
A0A1L1RKA9	Histidine-rich glycoprotein(predicted)	HRG	83.928
F1NSM1	Nudix hydrolase domain-containing p	NUDT9	40.085
F1NQ53	Signal recognition particle 19(predict	SRP19	16.099
Q5XNV3	Cytoplasmic activation-proliferation-a	CAPRIN1	77.987
P24479	Protein S100-A11 OS=Gallus gallus (	S100A11	11.413
A0A1D5P8J3	LUC7 like(predicted) OS=Gallus gall	LUC7L	32.649
Q5ZK19	Dynactin subunit 3(predicted) OS=Ga	DCTN3	21.049
A0A1D5PAY6	Anamorsin OS=Gallus gallus OX=90	CIAPIN1	34.646
F1NJ73	Adenylate kinase 2, mitochondrial OS	AK2	26.156
A0A1D5PRN9	Ribose-5-phosphate isomerase OS=G	RPIA	35.951
F1NU52	Ligand of Numb protein X 2-like(pred	LOC422320	75.324
F1NCP6	BLOC-1-related complex subunit 7 C	BORCS7	11.516
A0A1D5PI23	Dual specificity protein phosphatase (	DUSP3	20.694
F1NII4	Notchless protein homolog 1 OS=Gal	NLE1	53.467
E1C745	Protein MEF2BNB(predicted) OS=Ga	BORCS8	13.537
F1P241	CRK like proto-oncogene, adaptor pr	CRKL	21.148
P19596	Fibroblast growth factor 1 OS=Gallus	FGF1	17.321
A0A3Q3AVL7	Thioredoxin-disulfide reductase OS=(	TXNRD2	55.584
P28337	Aminomethyltransferase, mitochondr	AMT	42.058
A0A1L1RVU7	Tropomyosin alpha-3 chain isoform A	TPM3	29.022
Q5ZIR8	Mitochondrial import inner membran	TIMM9	10.483
F1P2E9	Myopalladin(predicted) OS=Gallus ga	MYPN	109.52
A0A3Q3APB3	DUF3421 domain-containing protein(	LOC101748084	23.87
A0A1D5PYK1	GRIP domain-containing protein OS=	GOLGA4	259.95
A0A1D5PWG8	14 kDa phosphohistidine phosphatase	PHPT1	13.83
A0A3Q2U481	LIM domain binding 3 OS=Gallus ga	--	30.012
F1NGH4	Death inducer-obliterator 1 OS=Gallu	--	73.093
A0A3Q2U0J0	RRM domain-containing protein OS=	MSI1	40.693
A0A1L1RLL0	TRASH domain-containing protein C	RPL24	17.688

F1NRX4	Late endosomal/lysosomal adaptor an	LAMTOR5	9.5157
A0A3Q2TZS5	Formin-binding protein 1-like OS=Ga	FNBP1L	63.789
F1NX22	Collagen alpha-1(XII) chain OS=Gall	COL12A1	333.51
F1NL99	6-pyruvoyl tetrahydrobiopterin syntha	PTS	16.749
A0A1L1RJT5	40S ribosomal protein S30 OS=Gallu	FAU	10.155
A0A1L1RPV2	Ig-like domain-containing protein OS	--	53.458
A0A3Q2TW86	CCR4-NOT transcription complex su	CNOT4	76.081
A0A1D5PM62	DUF1794 domain-containing protein	THAP4	17.676
A0A1D5PFW3	Splicing regulatory glutamic acid and	--	26.772
A0A1D5PMQ3	DNA helicase OS=Gallus gallus OX=	CHD2	212.83
A0A3Q2U1F0	Methyltransferase like 27 OS=Gallus	--	25.265
Q5ZKE7	UMP-CMP kinase OS=Gallus gallus	CMPK	22.172
A0A3Q2UD90	Prostaglandin E synthase 3 OS=Gallu	PTGES3	23.402
A0A1D5PKH3	G-rich RNA sequence binding factor	GRSF1	52.103
F1NHW3	Enhancer of rudimentary homolog OS	ERH	12.289
R4GGJ6	Migration and invasion enhancer 1(pr	MIEN1	13.28
A0A1D5PQE1	Aspartyl aminopeptidase OS=Gallus g	DNPEP	55.855
A0A1L1RZU5	GrpE protein homolog OS=Gallus ga	GRPEL1	26.527
A0A1L1RRP0	Rhodanese domain-containing protein	LOC421792	17.445
P10184	Ovoinhibitor OS=Gallus gallus OX=9	SPINK5	57.004
F1P1R3	ANK_REP_REGION domain-contain	PSMD10	24.007
P70081	Histone H4 type VIII OS=Gallus gallu	H4-VIII	11.439
F1NUU6	Choline-specific glycerophosphodiest	ENPP6	49.302
F1P4G0	Methionine aminopeptidase 2 OS=Ga	METAP2	53.006
A0A1L1RPB1	Sulfhydryl oxidase OS=Gallus gallus	GFER	21.436
P08286	Histone H1.10 OS=Gallus gallus OX=	--	22.003
A0A3Q3A2E9	RRM domain-containing protein OS=	PABPN1	33.969
A0A3Q2TZF7	Metastasis associated 1 family memb	MTA2	86.478
P41125	60S ribosomal protein L13 OS=Gallu	RPL13	24.395
Q92058	Alkaline phosphatase, tissue-nonspec	ALPL	56.76
A0A1D5P7C6	Peptidase_M24 domain-containing pr	PA2G4	43.779
A0A1D5PU00	Alpha-1-microglobulin OS=Gallus ga	AMBP	38.457
E1BQF9	Cold shock domain containing E1(pre	CSDE1	88.631

F1N986	Carbonic anhydrase OS=Gallus gallus	CA5A	36.215
Q5ZIT7	AAA domain-containing protein OS=	PSMC3	47.198
P04268	Tropomyosin alpha-1 chain OS=Gallu	TPM1	32.765
A0A3Q2TUB7	Ig-like domain-containing protein OS	LOC107054704	51.585
F1NRM8	Endoplasmic reticulum resident prote	ERP29	28.191
F1NEQ6	Proteasome subunit alpha type OS=G	PSMA6	27.47
A0A1L1RNU2	60S ribosomal protein L31 OS=Gallu	RPL31	15.63
A0A1D5NZ10	Gamma-glutamylcyclotransferase OS	GGCT	20.22
Q5ZKT8	39S ribosomal protein L15, mitochon	MRPL15	33.556
F1NN84	VPS37 C-terminal domain-containing	VPS37B	31.064
A0A3Q2U7A5	B3_4 domain-containing protein OS=	LRRC47	60.859
R4GME1	Prefoldin subunit 2(predicted) OS=Ga	PFDN2	17.616
F2Z4L4	Protein SET(predicted) OS=Gallus ga	SET	32.103
E1BWV6	Mitochondrial ribosomal protein L21(	MRPL21	21.347
A0A1D5PKG6	UBC core domain-containing protein	UBE2L3	33.444
A0A1D5P6M8	CN hydrolase domain-containing prot	NIT2	27.783
Q5ZIZ7	DnaJ heat shock protein family (Hsp4	DNAJA2	45.64
A0A1D5PD29	TGS domain-containing protein OS=(	MRPL39	40.059
Q9DDU8	Transformer-2 beta OS=Gallus gallus	TRA2B	33.798
R4GGQ4	Mitochondrial import inner membran	TIMM13	10.958
F1P4R6	Mitochondrial ribosomal protein S9(p	MRPS9	53.14
P61355	60S ribosomal protein L27 OS=Gallu	RPL27	15.798
A0A1D5PHK9	PDZ domain-containing protein OS=(	HTRA2	21.699
H9L019	Splicing factor 3b subunit 4(predicted	SF3B4	43.923
Q5ZHW8	Ribosomal protein S14(predicted) OS	RPS14	16.273
A0A1D5P9D4	Uncharacterized protein OS=Gallus g	IPO4	50.59
F1NDC2	40S ribosomal protein S23 OS=Gallu	RPS23	15.807
A0A3Q2U374	EF-hand domain-containing protein C	LOC107050604	39.914
F1NWH4	C-terminal-flanking peptide OS=Gall	TAC1	13.048
Q5ZM66	40S ribosomal protein S26 OS=Gallu	RPS26	13.031
A0A3Q3B1L2	Prefoldin subunit 6 OS=Gallus gallus	--	18.625
A0A1D5NYB2	Polyadenylate-binding protein OS=Ga	PABPC1	70.871
A0A3Q3AMS8	AAA_lid_3 domain-containing protei	LOC112531384	12.543

Q5ZHS1	Elongator complex protein 3 OS=Gal	ELP3	62.091
A0A3Q2UJW9	Vesicle-fusing ATPase OS=Gallus ga	SPATA5	134.12
F1N8W9	Dynactin 5 (p25)(predicted) OS=Gall	DCTN5	20.188
E1C1D7	4-hydroxy-2-oxoglutarate aldolase, m	HOGA1	34.898
F1NDD6	Receptor-associated protein(predictec	LRPAP1	40.783
F1NAP2	Dynactin subunit 6 OS=Gallus gallus	DCTN6	20.672
F1NKW4	SGNH_hydro domain-containing pro	IAH1	27.653
A0A1L1RU52	Tropomyosin alpha-1 chain OS=Gallu	TPM1	28.753
R4GKJ0	CXXC motif containing zinc binding	C8H1orf123	18.213
F1NVH4	PDZ domain-containing protein OS=(	PSMD9	22.729
A0A3Q2U232	Calreticulin(predicted) OS=Gallus ga	CALR3	15.191
A0A1D5P263	Abhydrolase_2 domain-containing pr	LYPLA1	24.623
A0A1D5PDE7	Peptidyl-prolyl cis-trans isomerase E	PPIE	35.5
A0A1D5PUF7	GDP-4-keto-6-deoxy-D-mannose-3,5	TSTA3	36.429
E1BX77	ClpX-type ZB domain-containing pro	CLPX	67.168
P35915	Hydroxymethylglutaryl-CoA lyase, m	HMGCL	31.436
A0A1D5NTX7	Seryl-tRNA synthetase OS=Gallus ga	SARS2	54.476
E1BWM0	TPR_REGION domain-containing pr	TTC1	33.792
A0A3Q2U9L1	Sialic acid binding Ig like lectin 1(pre	--	62.666
A0A1D5PHA6	GTP:AMP phosphotransferase AK3,	AK3	25.571
A0A1D5PEB4	Ubiquitin carboxyl-terminal hydrolase	USP43	106.6
E1C516	Pyridoxal phosphate homeostasis pro	PROSC	29.469
F1P524	Cytochrome b5 heme-binding domair	NENF	18.114
A0A1D5PZ61	FIP1-like 1 protein OS=Gallus gallus	FIP1L1	58.374
F1P0P3	Stomatin like 2 OS=Gallus gallus OX	--	41.474
R4GJL1	Protein NipSnap 3A (Fragment)(pred	NIPSNAP3A	28.153
A0A1D5NW58	COMM domain-containing protein 1	COMMD1	21.434
A0A1D5P3H0	M-phase phosphoprotein 6(predicted)	MPHOSPH6	19.526
A0A1L1S057	AIF-MLS domain-containing protein	MGARP	24.974
F1NYA9	Elongation factor 1-beta OS=Gallus g	EEF1B2	24.718
A0A1L1RLR7	RanBP2-type domain-containing prot	LOC107051192	129.46
Q5F426	Pyruvate dehydrogenase E1 compone	PDHA2	44.442
A0A3Q2UAH3	Upstream binding transcription factor	UBTF	82.314

Q6K1L7	Probable RNA-binding protein EIF1A	eif1ad	21.235
Q5F3N1	Protein-L-isoaspartate(D-aspartate) O	PCMT1	24.691
A0A1D5PI87	Hydroxysteroid 17-beta dehydrogenas	HSD17B10	43.384
E1BUJ8	CHCH domain-containing protein OS	CHCHD4	15.464
A0A1L1RT71	Phosphomevalonate kinase OS=Gallu	PMVK	21.104
F1N9R3	Rab interacting lysosomal protein(pre	RILP	45.17
E1BQN0	zf-CHCC domain-containing protein	NDUFS6	22.395
F1P5A5	28S ribosomal protein S31, mitochon	MRPS31	47.924
Q5ZMG0	D-dopachrome decarboxylase OS=Ga	DDT	12.835
A0A1D5PPU3	Beta-lactamase-like protein 2 OS=Ga	LACTB2	29.559
A0A1L1RY35	ATP synthase subunit alpha OS=Gall	ATP5A1Z	58.41
A0A1D5PN77	Probable cytosolic iron-sulfur protein	CIAO1	47.749
E1BV45	RRM domain-containing protein OS=	RNPS1	31.851
E1BT08	Heat shock 110 kDa protein OS=Gall	HSPH1	96.084
E1C861	L-methionine (R)-S-oxide reductase (	MSRB1	10.298
F1NFA8	Nsp1_C domain-containing protein C	NUP62	58.368
E1BXY8	zf-Tim10_DDP domain-containing pı	TIMM10	10.271
Q5ZHT7	Signal recognition particle 9 kDa prot	SRP9	10.08
A0A1D5PP80	DNA-directed RNA polymerases I an	POLR1C	34.815
A0A1L1RM23	NADH dehydrogenase [ubiquinone] i	NDUFS4	32.699
A0A3Q2UAF8	Synaptotagmin binding cytoplasmic F	SYNCRIP	69.609
E1BWV0	FeS_assembly_P domain-containing	FAM96B	17.791
Q5ZK84	Aldo-keto reductase family 1 membe	AKR1A1	37.076
A0A1D5P5T9	Ceramide transfer protein OS=Gallus	COL4A3BP	70.932
F1NPS5	Charged multivesicular body protein	CHMP1A	21.733
F1P141	NHL repeat containing E3 ubiquitin p	--	40.571
P13648	Lamin-A OS=Gallus gallus OX=9031	LMNA	73.164
E1C7U9	THAP-type domain-containing protei	THAP12	101.05
E1BRT9	Complex I subunit B13 OS=Gallus ga	NDUFA5	13.448
R4GJR8	Solute carrier family 25 member 51 is	--	32.865
E1BT93	Aldedh domain-containing protein OS	LOC107057078	56.79
F1NYU4	Glutathione peroxidase OS=Gallus ga	GPX4	19.345
E1BV97	RPOLD domain-containing protein C	POLR2C	31.389

F1NPJ4	Succinate dehydrogenase [ubiquinone	SDHA	72.963
A0A1D5PPN2	BTB domain containing 7(predicted)	BTBD7	120.25
Q5F3C9	SH3 domain-binding glutamic acid-ri	SH3BGRL	12.795
A0A3Q3AYZ9	Leupaxin(predicted) OS=Gallus gallu	LPXN	41.733
P20785	Collagen alpha-1(VI) chain OS=Gallu	COL6A1	107.98
F1NYE5	HABP4_PAI-RBP1 domain-containin	SERBP1	45.477
E1C0R8	Centromere protein J(predicted) OS=	CENPJ	142.55
A0A1D5PQV3	Dihydrolipoyllysine-residue succinylt	DLST	51.067
Q5ZJG3	Transcription factor BTF3 homolog 4	BTF3L4	17.232
Q5ZML4	Cysteine and histidine-rich domain-co	CHORDC1	37.37
A0A1D5P1P9	Ribonucleoprotein OS=Gallus gallus O	NHP2	18.796
Q9I993	Beta,beta-carotene 15,15'-dioxygenas	BCO1	60.384
F1NE59	Sushi, von Willebrand factor type A,	CR1	401.73
X2J6L4	Allograft inflammatory factor 1-like p	C9ORF58	16.291
F1NQ91	Pleckstrin homology domain containi	PLEKHA5	127.61
E1BWD5	Band 4.1-like protein 5 OS=Gallus ga	EPB41L5	57.376
A0A1D5P763	PITH domain-containing protein OS=	TXNL1	32.448
Q9YH06	High mobility group protein B1 OS=G	HMGB1	24.909
E1BXG9	Peptidylprolyl isomerase D(predicted	PPID	40.478
A0A1D5P2D6	Protein-tyrosine-phosphatase OS=Ga	PTPRF	212.64
Q5ZMB2	Heme-binding protein 1 OS=Gallus g	HEBP1	21.105
A0A1C7CYU2	2,3-bisphosphoglycerate 3-phosphatas	MINPP1	50.256
A0A1D5PD86	2,4-dienoyl-CoA reductase 1(predicte	DECR1	35.746
A0A3Q2TTL4	SAP30 binding protein(predicted) OS	SAP30BP	32.326
A0A3Q2U839	AAA domain-containing protein OS=	PSMC5	47.594
A0A3Q2UEL0	UBC core domain-containing protein	UBE2N	11.983
E1C578	Multiple C2 and transmembrane dom	MCTP1	104.16
A0A1D5NYD8	Vesicle-fusing ATPase OS=Gallus ga	VPS4A	49.042
Q6QLR1	Gallinacin-9 OS=Gallus gallus OX=9	GAL9	7.2784
A0A1D5PVK3	RNA binding protein fox-1 homolog	RBFOX3	38.417
A0A1L1RNH7	IF rod domain-containing protein OS=	--	62.085
Q5ZJ60	3-hydroxyisobutyryl-CoA hydrolase,	HIBCH	42.828
A0A3Q3B0Z9	PPPDE domain-containing protein OS	DESI1	18.725

P08629	Thioredoxin OS=Gallus gallus OX=9	TXN	11.7
F1NQW8	Splicing factor, arginine/serine-rich 1	SRSF1	27.791
A0A3Q2U9R9	Biotin carboxylation domain-containi	--	25.556
F1NB62	CTP:phosphoethanolamine cytidylylt	PCYT2	44.657
F1NB02	40S ribosomal protein S8 OS=Gallus	RPS8	24.054
A0A1D5NXA0	NADPH:adrenodoxin oxidoreductase	FDXR	51.246
A0A3Q3B2I1	WD_REPEATS_REGION domain-co	TSSC1	40.391
E1C3C6	V-type proton ATPase subunit G OS=	ATP6V1G1	13.552
A0A1D5PXK6	MPN domain-containing protein OS=	STAMBP	52.217
A0A1D5PXL4	Hypoxia up-regulated protein 1 OS=C	HYOU1	119.02
F1NRU5	RNA-binding protein NOB1 OS=Gal	NOB1	47.293
E1C1V7	Zinc finger and BTB domain containi	ZBTB47	33.557
Q5F424	CCT-beta OS=Gallus gallus OX=903	CCT2	57.431
A0A1L1RIQ6	Vacuolar protein sorting-associated p	VPS29	20.517
E1BTX6	Methionine adenosyltransferase 2 sub	MAT2B	37.448
Q5F4A4	Inosine-5'-monophosphate dehydroge	IMPDH2	55.558
P02605	Myosin light chain 3, skeletal muscle	--	16.71
P07341	Fructose-bisphosphate aldolase B OS	ALDOB	39.295
A0A1D5P7R1	RNA transcription, translation and tra	C5H14orf166	28.02
A0A1D5NWV2	PTB domain-containing protein OS=(	--	16.634
F1NLJ7	Alpha-mannosidase OS=Gallus gallus	MAN2C1	115.64
E1C5D2	Complex I-15 kDa OS=Gallus gallus	NDUFS5	12.651
E1C007	Protein kinase C and casein kinase su	PACSIN2	56.059
E1BY89	60S ribosomal protein L23 OS=Gallu	RPL23	14.865
Q5ZL33	Serine-threonine kinase receptor-asso	STRAP	38.14
A0A1D5NYA8	DnaJ heat shock protein family (Hsp4	DNAJA4	44.802
E1BTE0	Single stranded DNA binding protein	SSBP1	17.232
Q5ZM32	Dihydrolipoyl dehydrogenase OS=Ga	DLD	53.974
P0CB50	Peroxiredoxin-1 OS=Gallus gallus OX	PRDX1	22.314
Q9IAM7	Double-strand break repair protein M	MRE11	80.023
E1C1Y5	Ribosomal_S10 domain-containing p	MRPS10	22.733
A0A1D5P1E1	Tripeptidyl aminopeptidase OS=Gallu	TPP1	62.884
A0A3Q3AI12	Kinesin light chain OS=Gallus gallus	KLC2	61.627

Q800W4	TIA-1 OS=Gallus gallus OX=9031 G	TIA1	41.292
Q5ZK59	Ribonuclease H2 subunit B OS=Gallu	RNASEH2B	29.07
A0A1D5PD41	WD_REPEATS_REGION domain-co	WDR77	38.49
A0A3Q2UBJ7	HIT domain-containing protein OS=C	HINT1	16.915
F1NJV1	Amino_oxidase domain-containing pr	PAOX	54.298
A0A1D5NY19	Peroxin-19 OS=Gallus gallus OX=90	LOC107050261	31.589
F1P2A1	HMA domain-containing protein OS=	ATOX1	7.984
Q5ZHP2	Macro domain-containing protein OS	OARD1	20.906
F1P4D9	Myosin IE(predicted) OS=Gallus gall	MYO1E	126.79
A0A1D5P2B1	Protein quaking OS=Gallus gallus OX	QKI	36.725
F1NYI3	RNA-splicing ligase RtcB homolog C	RTCB	55.242
H9KYW5	Cathepsin S(predicted) OS=Gallus ga	CTSS	36.321
E1C4P4	Protein kinase C and casein kinase su	PACSIN1	46.207
A3RKL3	Dihydrofolate reductase OS=Gallus g	DHFR	21.78
A0A1D5P632	APOBEC1 complementation factor(p	A1CF	64.066
A0A1D5PNR0	RNA-binding protein 8A OS=Gallus	RBM8A	18.431
A0A1D5NW35	JmjC domain-containing protein OS=	KDM3B	181.42
A0A3Q2UGF7	Putative LOC102098019(predicted) C	LOC423629	24.487
F1P1V8	Integrator complex subunit 13(predic	INTS13	82.444
A0A1D5PQK9	TatD DNase domain containing 1(pre	TATDN1	31.974
E1C6T8	Eukaryotic translation initiation facto	EIF3I	36.489
Q5ZIM5	Methionine aminopeptidase 1 OS=Ga	METAP1	43.121
F1NAC6	Ubiquinol-cytochrome c reductase co	UQCRC1	55.914
E1C894	NACHT domain-containing protein C	NLRX1	111.3
A0A3Q2U825	40S ribosomal protein S28 OS=Gallu	RPS28	7.8409
A0A1D5NTY5	Transitional endoplasmic reticulum A	LOC430766	89.31
A0A3Q3AUG2	FYVE-type domain-containing protei	EEA1	161.49
F1P3K7	4a-hydroxytetrahydrobiopterin dehyd	PCBD2	11.779
F1NHT3	Spectrin alpha chain, non-erythrocyti	SPTAN1	285.32
A0A1D5PAA8	Glycogenin 1(predicted) OS=Gallus g	GYG1	37.2
E1BX03	Far upstream element binding protein	FUBP1	67.428
Q5ZJB7	Charged multivesicular body protein	CHMP7	49.779
F1NZC1	ABRA C-terminal-like protein OS=G	ABRACL	9.0534

R4GHR0	Glutaredoxin-like protein OS=Gallus	--	15.486
E1C6N0	26S proteasome regulatory subunit RI	PSMD14	34.567
R4GH86	Glutathione peroxidase OS=Gallus ga	GPX1	20.586
E1C8S9	J domain-containing protein OS=Gall	DNAJC9	29.844
A0A1L1RV30	Stromal cell derived factor 2 like 1(pr	SDF2L1	21.229
F1NAC5	DEP domain-containing mTOR-intera	DEPTOR	46.85
E1BT27	StAR related lipid transfer domain co	STARD13	108.16
E1C6C4	Dpy-30 histone methyltransferase cor	DPY30	11.26
A0A1D5PW77	Ig-like domain-containing protein OS	LOC776376	34.987
A0A1L1S0C3	Cystatin domain-containing protein C	CST3	13.71
F1NVZ3	39S ribosomal protein L23, mitochon	MRPL23	23.935
A0A1D5NWE7	Succinate dehydrogenase assembly fa	SDHAF2	18.496
Q5ZKK8	Ribosomal protein L19 OS=Gallus ga	RPL19	23.413
P02789	Ovotransferrin OS=Gallus gallus OX=	--	77.776
A0A1D5PFY7	Succinate-semialdehyde dehydrogena	ALDH5A1	57.922
F1NW58	RF_PROK_I domain-containing prote	MTRF1L	44.98
E1BRV8	D-aminoacyl-tRNA deacylase OS=Ga	DTD1	23.251
A0A1D5PFX6	RNA binding motif protein 12(predic	RBM12	93.043
F1P1L8	SERPIN domain-containing protein C	SERPINB6	43.1
A0A1D5PTF1	Striated muscle preferentially express	SPEG	362.41
A0A1D5PX97	AMP_N domain-containing protein C	PEPD	55.121
F1P0L3	HpcH_HpaI domain-containing prote	CLYBL	36.603
A0A1D5PXU4	TOG array regulator of axonemal mic	FAM179B	170.04
A0A1D5PIF2	LIM domain-containing protein OS=C	LIMS1	37.207
A0A1L1RTD8	Phytanoyl-CoA 2-hydroxylase(predic	PHYH	37.025
Q5ZMR4	RRM domain-containing protein OS=	SRSF10	31.286
A0A1D5P5R1	CN hydrolase domain-containing prot	NIT1	34.633
Q5ZJQ7	RNA polymerase II subunit A C-term	SSU72	22.574
A0A1L1RWG7	MICOS complex subunit(predicted) C	CHCHD6	46.982
A0A1D5NVM3	Endonuclease/exonuclease/phosphata	EEPD1	61.837
A0A1D5PSJ8	NADB-LER4(predicted) OS=Gallus g	LOC769668	27.405
F1NVD1	ADAM metallopeptidase with thromb	ADAMTS3	136.08
F1NN16	40S ribosomal protein S7 OS=Gallus	RPS7	22.112

A0A1D5PGY8	CS domain-containing protein OS=G	PTGES3L	18.146
F1P0K1	Rib_recp_KP_reg domain-containing	RRBP1	111.97
F1NP43	Prefoldin subunit 4 OS=Gallus gallus	PFDN4	15.4
Q98UC3	Ubiquitin fusion-degradation 1-like p	Ufd1l	34.562
F1NN91	Chromo domain-containing protein C	CDYL2	56.958
A0A3Q2U419	AU RNA binding methylglutaconyl-C	AUH	36.819
E1C5C6	Fem-1 homolog C(predicted) OS=Ga	FEM1C	68.599
Q5F411	CCT-epsilon OS=Gallus gallus OX=9	CCT5	59.714
P62149	Calmodulin OS=Gallus gallus OX=90	CALM	16.837
E1BZS2	Nucleosome assembly protein 1 like 1	NAP1L1	45.358
F1NZK8	Tr-type G domain-containing protein	EFL1	126.64
E1BYY7	3-oxoacyl-[acyl-carrier-protein] synth	OXSM	48.942
Q02020	Fibrinogen beta chain (Fragment) OS	FGB	52.678
A0A3Q2U5Q5	A-kinase anchoring protein 9(predicte	AKAP9	441.59
A0A1D5PPX8	DGCR8 microprocessor complex sub	--	87.395
A0A1D5PAQ3	UDP-N-acetylglucosamine pyrophosp	UAP1	52.515
F1NV99	Carboxylic ester hydrolase OS=Gallu	BCHE	65.818
F1NHE0	Staufen double-stranded RNA bindin	STAU1	78.076
A0A1D5P0D3	tRNA-binding domain-containing pro	AIMP1	33.866
F1NND9	RF_PROK_I domain-containing prot	MRPL58	21.962
A0A1D5NZ30	Nucleolin OS=Gallus gallus OX=903	NCL	75.399
F1NA30	Bis(5'-adenosyl)-triphosphatase OS=G	FHIT	16.88
A0A3Q2UD98	EMI domain-containing protein OS=G	--	60.46
F1NNP6	Glutaredoxin 3(predicted) OS=Gallus	GLRX3	36.574
A0A3Q2UH39	C-terminal-binding protein 2 isoform	--	60.488
Q5ZHQ1	Small ubiquitin-related modifier 3 OS	SUMO3	10.711
Q9I9K9	Glycine amidinotransferase, mitochon	GATM	48.311
R4GHY3	Abhydrolase_2 domain-containing pr	LYPLAL1	26.183
E1C4C0	Acyl carrier protein OS=Gallus gallus	NDUFAB1	16.547
E1C7J8	Mitochondrial inner membrane protea	ATP23	27.532
E1BSL7	Thioredoxin domain-containing prote	ERP44	46.976
A0A1D5NXK7	Prefoldin subunit 5(predicted) OS=G	PFDN5	17.259
E1BUR2	FCH and mu domain containing endo	--	38.212

P84172	Elongation factor Tu, mitochondrial (	TUFM	38.249
A0A3Q2U474	Ig-like domain-containing protein OS	--	10.324
A0A3Q2U784	Small nuclear ribonucleoprotein Sm I	SNRPD2	16.22
E1BVB1	60S ribosomal protein L32(predicted)	RPL32	15.916
A0A1D5PC97	SRA1 domain-containing protein OS=	SRA1	22.865
A0A1D5PYI7	U6 snRNA-associated Sm-like protei	LSM5	9.9234
A0A140T8H0	Molybdopterin synthase catalytic sub	MOCS2	17.833
Q5F425	Protein lin-7 homolog C OS=Gallus g	LIN7C	21.847
R4GLK3	26S proteasome non-ATPase regulato	PSMD4	40.533
P09648	Procathepsin L (Fragments) OS=Gall	CTSL	23.963
A0A1D5PKI0	H1.10 linker histone OS=Gallus gallu	--	22.541
P27463	Retinal dehydrogenase 1 OS=Gallus g	ALDH1A1	55.809
F1NAZ4	G protein beta subunit-like OS=Gallu	MLST8	36.092
F1NK88	Rho-associated protein kinase 2 OS=(	ROCK2	165.38
A0A3Q2U6A7	Calponin-homology (CH) domain-co	C20orf27	78.777
A0A1L1RUE3	ASW(predicted) OS=Gallus gallus O	HINTW	13.922
F1NXX2	Mitochondrial ribosomal protein S22(	MRPS22	60.098
E1C6X1	Inorganic diphosphatase OS=Gallus g	PPA2	52.868
A0A1D5PME7	Glutamate receptor OS=Gallus gallus	GRIN2A	128.9
A0A1D5NZV1	Microtubule associated protein RP/EI	MAPRE3	31.833
A0A1L1RL74	DnaJ homolog subfamily C member 3	DNAJC3	53.038
E1BQD1	ES1 protein-like protein, mitochondri	GATD3AL1	30.107
Q5Y1E8	DD1 protein OS=Gallus gallus OX=9	DD1	103.52
F1P5N7	Asparaginase-like protein 1 OS=Gallu	ASRGL1	33.492
A0A1L1RSD2	Glucuronidase beta(predicted) OS=G	GUSB	75.906
A0A3Q2UDZ5	Vacuolar protein sorting 13 homolog	VPS13C	400.61
F1NNU3	Cysteine desulfurase, mitochondrial (	NFS1	49.105
A0A3Q2UBL3	Nicotinate-nucleotide pyrophosphory	QPRT	35.474
Q90ZG0	Peptidylprolyl isomerase OS=Gallus g	FKBP12	11.973
A0A3Q2U9M9	Golgin A3(predicted) OS=Gallus gall	GOLGA3	166.48
A0A1D5PY04	CAP-Gly domain containing linker pr	CLIP2	111.87
E1BVC6	ANK_REP_REGION domain-contai	ANKRD17	245.19
A0A1D5PI06	NADH dehydrogenase [ubiquinone] f	NDUFV2	26.893

Q5ZJY1	6-phosphogluconolactonase OS=Gall	PGLS	26.213
E1C5F2	Ribonuclease T2 (Fragment)(predicte	RNASET2	31.197
E1BTU5	Ectonucleotide pyrophosphatase/phos	ENPP7	50.253
A0A3Q2U983	Lipoyl-binding domain-containing pr	PC	34.402
F1NDA1	Eukaryotic translation initiation facto	EIF2D	66.428
F1P5J8	N-acetylglucosamine-6-phosphate de	AMDHD2	44.294
Q5ZI93	TPD52 like 2(predicted) OS=Gallus g	TPD52L2	22.181
A0A1D5NZY0	Tenascin OS=Gallus gallus OX=9031	TNC	168.95
A0A1D5NX08	Carboxylic ester hydrolase OS=Gallu	CES1L2	61.895
F1NZC0	Aldose 1-epimerase OS=Gallus gallus	GALM	38.048
F1NHV9	DNA-directed RNA polymerase II sul	POLR2E	24.643
A0A3Q2U9S7	Shootin-1 OS=Gallus gallus OX=903	SHTN1	68.681
A0A3Q2TU15	TPR_REGION domain-containing pr	TTC4	42.676
Q5F3Z5	DnaJ homolog subfamily B member 6	DNAJB6	36.675
E1C958	Asparaginyl endopeptidase OS=Gallu	LGMN	49.261
A0A1L1S0V6	Eukaryotic translation initiation facto	EIF4H	25.423
A0A1D5PI81	Non-specific serine/threonine protein	DAPK1	151.83
A0A1D5PY00	CS domain-containing protein OS=G	NUDCD3	39.725
F1NT45	RRM domain-containing protein OS=	NIFK	31.416
E1BWJ8	Abl-interactor 1, transcript variant X1	ABI1	54.893
A0A3Q2UFW2	U2 snRNP auxiliary factor large subu	U2AF2	75.971
F1P3S2	Beta-MPP OS=Gallus gallus OX=903	PMPCB	53.937
E1BYS5	Cingulin like 1(predicted) OS=Gallus	CGNL1	149.16
A0A1D5PDJ8	Corrinoid adenosyltransferase OS=Ga	--	24.242
Q5ZLF8	Microtubule-associated proteins 1A/1	MAP1LC3B	14.618
P43233	Cathepsin B OS=Gallus gallus OX=9	CTSB	37.587
A0A1D5PFR7	Microtubule-associated protein 1A/1E	MAP1LC3A	14.373
A0A1L1RQ73	Myeloid leukemia factor 2(predicted)	MLF2	37.444
F1NPL9	Cytochrome c oxidase copper chapero	COX17	7.2084
A0A1D5PPL7	Eukaryotic translation elongation fact	EEF1D	32.325
A0A3Q2UM15	FIP-RBD domain-containing protein	ERC1	125.24
E1C420	3-hydroxy-3-methylglutaryl coenzyme	HMGCR	101.33
A0A1L1RUE9	Cell division cycle protein 123 homol	CDC123	36.306

Q5ZLD1	Fumarate hydratase, mitochondrial O	FH	54.299
F1NRT4	Ubiquinone biosynthesis O-methyltra	COQ3	31.18
A0A1D5NTX4	Mitochondrial ribosomal protein S28	MRPS28	30.341
Q5ZJ85	U4/U6 small nuclear ribonucleoprote	PRPF3	77.385
F1NXF1	VWFA domain-containing protein OS	VWA8	213.5
Q5ZI42	Malignant T-cell-amplified sequence	MCTS1	20.571
F1NBR7	Ubiquinone biosynthesis monooxyger	COQ6	52.733
A0A3Q3A5D4	Pleckstrin(predicted) OS=Gallus gallu	PLEK	40.282
A0A1D5PAU7	N-acyl-L-amino-acid amidohydrolase	ACY1	45.909
E1BWB7	PX domain-containing protein OS=G	HS1BP3	43.231
A0A1D5PIF7	ANK_REP_REGION domain-contair	KANK1	138.95
A0A3Q2UF49	Kin17_mid domain-containing protei	KIN	41.346
F1NL05	Poly [ADP-ribose] polymerase OS=G	PARP1	113.14
F1NPM7	RRM domain-containing protein OS=	TRA2A	32.464
A0A1D5PL30	FMR1 autosomal homolog 1(predicte	FXR1	65.263
P13216	Adrenodoxin, mitochondrial (Fragme	FDX1	15.482
A0A3Q2U8X1	Lipoyl synthase, mitochondrial OS=G	LIAS	41.439
F1NIA8	5-phosphohydroxy-L-lysine phospho-	PHYKPL	48.054
Q98TF6	60S ribosomal protein L36 OS=Gallu	RPL36	12.296
P80226	Fatty acid-binding protein, liver OS=	FABP1	14.21
A0A1D5PFR6	Sep15_SelM domain-containing prote	SELENOF	16.421
A0A1D5P8M2	Parkinson disease 7 domain-containir	PDDC1	23.585
Q90734	Alpha-actinin-4 OS=Gallus gallus O	ACTN4	104.21
F1N9L9	Ubiquitin associated and SH3 domair	UBASH3A	70.786
Q9YHT2	Succinate dehydrogenase [ubiquinone	SDHB	32.597
Q5F4A3	Acidic leucine-rich nuclear phosphop	ANP32E	28.925
E1BX68	Arf-GAP domain-containing protein	SMAP1	48.258
F1NAE9	Multimerin 1(predicted) OS=Gallus g	MMRN1	133.07
A0A3Q3AA87	Polysacc_synt_4 domain-containing p	PBDC1	48.61
F1NQ55	URI1 prefoldin like chaperone(predic	URI1	57.201
F1NE68	Aldehyde oxidase OS=Gallus gallus	AOX1	146.58
E1C962	U6 snRNA-associated Sm-like protei	LSM1	15.164
A0A1L1RRT4	Peptidoglycan recognition protein L(p	PGLYRP2	49.978

F1NXB6	Peptidase_M14 domain-containing pr	CPB2	48.912
F1P4I0	PKS_ER domain-containing protein (	CRYZ	35.298
Q5ZLW7	Putative RNA-binding protein Luc7-l	LUC7L2	47.232
P05081	Adenylate kinase isoenzyme 1 OS=G	AK1	21.683
A0A3Q2TWL3	B30.2/SPRY domain-containing prot	LOC107055470	21.548
A0A1D5PKN8	Sulfurtransferase(predicted) OS=Gall	MPST	33.222
F1NU17	Phosphoglycerate kinase OS=Gallus g	PGK2	44.666
P14448	Fibrinogen alpha chain OS=Gallus ga	FGA	82.438
A0A1L1RR91	J domain-containing protein OS=Gall	DNAJB11	40.381
F6R1X6	Small RNA binding exonuclease prot	SSB	46.316
E1BQC2	Ovotransferrin OS=Gallus gallus OX=	TF	83.489
F1NNN7	Tumor necrosis factor receptor type 1	TRADD	51.35
A0A1D5NTG1	PTS1-BP OS=Gallus gallus OX=903	PEX5	71.747
A0A1D5P6Q7	Rho GTPase activating protein 35(pre	ARHGAP35	179.86
E1C3A1	Kinesin-like protein OS=Gallus gallu	KIF5B	110.14
F1P4P9	GRIP domain-containing protein OS=	GCC2	196.15
P25207	Nuclear transcription factor Y subuni	NFYB	22.585
A0A1D5P823	Cysteine rich with EGF like domains	CRELD1	48.845
Q5ZKR4	Rab-like protein 3 OS=Gallus gallus (	RABL3	26.002
F1NA05	RAN binding protein 2(predicted) OS	--	48.199
P17153	Annexin A5 OS=Gallus gallus OX=9	ANXA5	36.198
Q6VN51	Protein phospatase 3 regulatory subur	PPP3R1	19.3
Q5ZK10	NSFL1 cofactor p47 OS=Gallus gallu	NSFL1C	40.64
F1NEA8	MYND-type domain-containing prote	PDCD2	41.619
A0A1D5P2K1	UV excision repair protein RAD23 O	RAD23A	39.373
E1BRU0	Mitotic arrest deficient 1 like 1(predic	MAD1L1	83.927
A0A3Q2TU46	Exocyst complex component OS=Gal	EXOC6B	93.32
F1NK48	PH domain-containing protein OS=G	--	23.298
Q5ZM47	Serine/threonine-protein phosphatase	PPP2CA	35.564
Q5ZJR8	Elongation of very long chain fatty ac	ELOVL6	31.268
A0A1D5PF37	39s ribosomal protein mitochondrial(	MRPL55	13.382
A0A3S5ZP68	WH1 domain-containing protein OS=	ENAH	57.154
E1BWG6	REST corepressor 1(predicted) OS=G	RCOR1	50.87

A0A1D5NXB3	RNA binding motif protein 4(predicte	LOC100859276	40.873
A0A3Q2UMJ6	Serine/threonine kinase 31(predicted)	STK31	107.82
A0A1D5NTR1	Coiled-coil domain-containing protei	CCDC6	53.297
A0A1D5P892	Serine and arginine rich splicing facto	SRSF6	40.521
A0A1L1RJI1	Cornichon family AMPA receptor au	CNIH1	18.606
A0A1L1RP44	Ribokinase OS=Gallus gallus OX=90	RBKS	30.521
E1C172	Eukaryotic elongation factor 2 kinase	EEF2K	84.766
Q8JG64	Protein disulfide-isomerase A3 OS=C	PDIA3	56.181
E1C2L5	RRM domain-containing protein OS=	ALYREF	26.695
A0A1D5PVX9	AB hydrolase-1 domain-containing pı	LOC107056877	22.526
A0A1D5PJC6	Calcineurin-like phosphoesterase don	CPPED1	35.894
A0A1D5PR69	Phospholysine phosphohistidine inorg	LHPP	28.538
E1C9H8	Protein kinase cAMP-dependent type	PRKAR2A	45.502
F1NA82	TPR_REGION domain-containing pr	SGTB	33.847
R4GI50	RNA polymerase II-associated protei	RPAP3	72.598
F1P2S1	RPOL4c domain-containing protein C	POLR2D	16.325
A0A3Q2U656	PX domain-containing protein OS=G	SNX4	46.734
R4GKF0	CHCH domain-containing protein OS	CHCHD10	9.4295
A0A3Q3AUH6	LEM domain-containing protein OS=	LEMD3	95.283
A0A3Q2UFU8	Ig-like domain-containing protein OS	--	9.5923
A0A1D5PP57	Vacuolar proton pump subunit B OS=	ATP6V1B2	55.635
O57535	Nucleoside diphosphate kinase OS=C	--	17.288
A0A3Q2TS99	S1 motif domain-containing protein (	--	8.8443
Q5F4B4	Cell-cycle related and expression-elev	CREPT	36.852
A0A1D5P8E6	Nuclear transcription factor, X-box bi	NFX1	127.9
E1BZR1	Heterogeneous nuclear ribonucleopro	HNRNPR	71.974
A0A1D5PFK3	Alpha-dystroglycan OS=Gallus gallus	DAG1	97.709
A0A1L1RWZ8	Coiled-coil domain-containing protei	CCDC58	14.298
Q5ZK02	GM2 ganglioside activator OS=Gallu	RCJMB04_14a17	12.77
E1C2U4	DNA helicase OS=Gallus gallus OX=	MCM4	96.883
Q5ZJ54	T-complex protein 1 subunit zeta OS=	CCT6	57.644
F1NXV6	Activation peptide fragment 1 OS=Ga	F2	73.106
R4GI40	LDLR chaperone MESD OS=Gallus g	MESDC2	24.563

F1NVI1	VPS37 C-terminal domain-containing	VPS37A	44.281
A0A1D5PPP2	Pseudouridine 5'-phosphatase(predict	PUDP	26.18
A0A1L1RXE9	Ubiquinol-cytochrome c reductase co	UQCRC2	45.772
A0A3Q2TXZ7	Collagen IV NC1 domain-containing	--	42.909
P21611	Beta-2-microglobulin OS=Gallus gall	B2M	13.042
E1C7I2	Mitochondrial ribosomal protein L14	MRPL14	16.265
F1NDC7	SRCR domain-containing protein OS	MARCO	49.946
F1NT10	Transcription elongation regulator 1(	TCERG1	109.94
Q5ZIA3	PX domain-containing protein OS=G	SNX3	18.755
F1NVX8	Tubulin-folding cofactor E OS=Gallu	TBCE	57.982
Q90838	Leucine zipper protein OS=Gallus ga	PTRF	41.311
A0A3Q2U9D4	Methyltranfer_dom domain-containin	METTL10	19.305
Q5ZMN1	G3BP stress granule assembly factor	G3BP1	52.44
A0A3Q2UD25	Avidin(predicted) OS=Gallus gallus (	LOC426220	18.339
R4GIQ2	60S ribosomal protein L21 OS=Gallu	RPL21	18.577
A0A3Q3AM84	Non-specific serine/threonine protein	MARK2	76.678
F1P0J0	Elongation factor G, mitochondrial O	GFM1	82.407
O42184	CAP-Gly domain-containing linker p	CLIP1	161.03
A0A1D5NZW2	Nicotinamide-nucleotide adenylyltran	NMNAT1	32.39
Q7ZT63	Proteasome subunit beta OS=Gallus g	cpsmb7	29.876
A0A1L1RLN6	Myosin light polypeptide 6 OS=Gallu	MYL6	16.838
A0A1D5P407	Serine/arginine-rich splicing factor 4	SRSF5A	32.223
O93436	Signal transducing adapter molecule 2	STAM2	52.406
Q5ZKU8	p21-activated protein kinase-interacti	PAK1IP1	41.232
Q5ZI51	ADP-ribose glycohydrolase ARH3 O	ADPRS	39.909
A0A0K0PUH6	Chemerin OS=Gallus gallus OX=903	RARRES2	18.231
A0A1D5NXH1	Poly(U)-specific endoribonuclease O	ENDOUL	33.582
E1C7P4	Complement factor H (Fragment)(pre	CFH	141.46
Q5ZIE0	Eukaryotic translation initiation facto	EIF5	49.078
P47832	60S ribosomal protein L26 (Fragment	RPL26	15.165
O42388	60S ribosomal protein L40 OS=Gallu	UBA52	14.739
F1NN92	Uridine-cytidine kinase OS=Gallus ga	UCK1	30.744
A0A1D5PRY0	Guanylate kinase OS=Gallus gallus C	GUK1	22.334

Q5ZMN0	Acidic leucine-rich nuclear phosphop	ANP32B	29.943
A0A3Q2UGH0	Trafficking protein particle complex 2	TRAPPC2L	11.965
Q5F3R2	Lysine-specific demethylase 5B OS=(	KDM5B	173.46
A0A1D5PR30	BUB1 N-terminal domain-containing	BUB1B	122.52
E1C1F2	GRIP domain-containing protein OS=	TRIP11	227.28
F1N9A6	E3 ubiquitin protein ligase OS=Gallu	RNF20	114.82
Q5ZMN4	UBR-type domain-containing protein	UBR7	47.08
P62846	40S ribosomal protein S15 OS=Gallu	RPS15	17.04
A0A3Q3B2U9	PKS_ER domain-containing protein (	LOC112529921	40.001
F1N9B5	39S ribosomal protein L50, mitochon	MRPL50	18.175
E1C214	Ubiquitin carboxyl-terminal hydrolase	USP16	93.133
Q5ZLY3	Tropomodulin 3(predicted) OS=Gallu	TMOD3	39.365
A0A1D5NZQ0	Bromodomain-containing protein 4 (F	BRD4	115.72
A0A1D5P856	STAS domain-containing protein OS=	HDHD2	28.326
E1C4W4	Aldehyde dehydrogenase (NAD(+)) (	ALDH7A1	58.053
A0A1L1RMS7	Amidohydro-rel domain-containing p	DPYS	54.765
F1N8A1	Cyclic pyranopterin monophosphate s	MOCS1	74.56
Q5ZLP4	Serine/threonine-protein phosphatase	PPP2R2A	51.726
A0A1D5PJC9	PDZ and LIM domain protein 7 OS=(	PDLIM7	39.842
A0A1D5PBD2	ATP synthase inhibitory factor subun	ATPIF1	10.456
A0A3Q3AGL8	CTP_transf_like domain-containing p	PCYT1A	42.096
A0A1D5PG36	Isocitrate dehydrogenase [NAD] subu	IDH3B	48.352
Q5ZHP5	Charged multivesicular body protein 4	CHMP4B	25.155
A0A3Q2UKP2	MG2 domain-containing protein(pred	--	20.305
A0A1L1RYK0	ATP synthase subunit gamma OS=Ga	ATP5C1	32.723
F1P0X3	Septin OS=Gallus gallus OX=9031 G	SEPTIN11	50.634
F1P3V8	DnaJ homolog subfamily C member 2	DNAJC2	71.423
Q5ZK88	Paraspeckle component 1 OS=Gallus	PSPC1	58.47
Q8UVD9	Far upstream element-binding protein	KHSRP	80.643
A0A1D5PUG8	Bridging integrator 2 OS=Gallus gallu	BIN2	51.251
P0DMQ6	Sorbitol dehydrogenase OS=Gallus ga	SORD	38.144
F1NLH9	Inosine triphosphate pyrophosphatase	ITPA	22.262
E1C2C5	Phosphatidylinositol 3-kinase regulato	PIK3R2	83.493

A0A3Q3APT7	GOLGA2L5 domain-containing prote	GOLGA2	132.57
F1NQR2	Na(+)/H(+) exchange regulatory cofa	SLC9A3R2	39.04
A0A1D5PIT4	Lipocln_cytosolic_FA-bd_dom doma	PTGDS	20.873
A0A1L1RJI4	Dehydrogenase/reductase SDR family	DHRS11	27.644
F6T844	ZPR1 zinc finger(predicted) OS=Gall	ZPR1	49.642
E1BVA1	SH3 domain-containing protein OS=(	EPS8	88.63
E1C3A9	Vigilin OS=Gallus gallus OX=9031 (	HDLBP	141.6
A0A3Q2UHT5	S-AdoMet_synt_C domain-containing	--	40.942
A0A1D5P2M3	Epidermal growth factor receptor patl	EPS15	96.732
A0A1D5PD32	RNA helicase OS=Gallus gallus OX=	DDX17	73.041
Q5ZIN1	Nuclear migration protein nudC OS=(	NUDC	39.209
A0A1D5P851	DLG associated protein 4(predicted) (	DLGAP4	31.649
F1P2Q2	UDENN domain-containing protein (	FAM45A	44.886
A0A1L1RX65	Isocitrate dehydrogenase [NAD] subu	IDH3A	39.79
Q5H7N8	Zinc finger CCCH domain-containing	ZC3H15	48.996
A0A1D5P524	Kinesin family member 1B(predicted)	KIF1B	193.34
A0A1D5P117	RNA polymerase II-associated factor	PAF1	55.497
A0A1L1RRN4	Peptidyl-prolyl cis-trans isomerase O:	PPIB	24.308
A0A1D5NV31	RRM domain-containing protein OS=	--	14.47
A0A1D5P394	DUF2052 domain-containing protein	CCDC97	62.124
Q5ZMW1	Aconitate hydratase, mitochondrial O	ACO2	85.649
A0A3Q2UCS5	Coiled-coil domain-containing protei	CCDC25	22.335
Q5ZKY2	Ubiquitin-like modifier-activating enz	ATG7	78.8
A0A452J889	Prohibitin OS=Gallus gallus OX=903	PHB	29.783
F7BFS9	WD_REPEATS_REGION domain-co	DCAF7	51.301
E1C2E9	MRP-S23 domain-containing protein	MRPS23	22.087
A0A3Q2U7L8	Ig-like domain-containing protein OS	--	9.7865
A0A3Q2U335	ADF-H domain-containing protein O:	--	13.208
Q5ZHQ8	Transcription elongation factor A1(pr	TCEA1	33.983
A0A1D5PMP3	ANK_REP_REGION domain-contai	UACA	153.92
F1NN30	Putative LOC102091205(predicted) (	NLRP1L	56.963
A0A1D5PVL4	MPN domain-containing protein OS=	EIF3F	23.239
A0A3Q3AWH1	Histone-binding protein RBBP4 OS=	RBBP4	43.481

A0A452J7Q6	Endophilin-A2 OS=Gallus gallus OX	SH3GL1	52.856
E1C2F2	Pinin OS=Gallus gallus OX=9031 GN	PNN	78.126
R4GMH5	Plasminogen OS=Gallus gallus OX=9	LOC428593	93.294
Q5ZKQ2	Quinoid dihydropteridine reductase(p	QDPR	24.894
F1NV61	Caspase 7(predicted) OS=Gallus gallu	CASP7	34.721
A0A1D5P6Q6	TARBP2 subunit of RISC loading co	--	28.049
F1NM24	DNA polymerase subunit gamma-2, n	POLG2	50.355
F1NZ84	Coagulation factor XIII B chain(predi	F13B	70.613
F1NCD7	Peptidyl-prolyl cis-trans isomerase OS	PIN4	13.182
F1NRM4	Glyco_18 domain-containing protein	CTBS	40.686
F1NP60	Mannose receptor C type 2(predicted)	MRC2	157.87
E1BTQ4	Avidin(predicted) OS=Gallus gallus C	AVDL	16.136
A0A1D5PER6	1-phosphatidylinositol 4,5-bisphospha	LOC776273	142.68
A0A1D5P2G3	Choline kinase alpha(predicted) OS=C	CHKA	51.111
Q8QGH2	Small ubiquitin-related modifier 1 OS	SUMO1	11.557
A0A1D5PBJ0	Bromodomain adjacent to zinc finger	BAZ1B	176.69
F1N9C1	NADB-LER3 OS=Gallus gallus OX=	LOC415661	27.874
E1C756	Molybdopterin molybdenumtransfera	GPHN	79.733
P53666	LIM domain kinase 2 OS=Gallus gall	LIMK2	72.467
A0A3Q2U214	A2M domain-containing protein(pred	--	12.474
A0A1D5P5J5	RAB25, member RAS oncogene fami	RAB25	22.986
A0A3Q2TS11	Xyloside xylosyltransferase 1(predict	XXYLT1	40.974
Q5ZIP2	Ragulator complex protein LAMTOR	LAMTOR3	13.68
Q5ZL57	Coatomer subunit delta OS=Gallus ga	ARCN1	57.17
A0A1D5PLP1	NAC-A/B domain-containing protein	NACA	24.873
A0A1D5PZN5	PTPRF interacting protein alpha 1(pr	PPFIA1	136.06
E1BXF8	RF_PROK_I domain-containing prote	MTRF1	52.83
A0A1D5PXI1	Multifunctional fusion protein OS=G	ALDH4A1	60.626
A0A1L1RXT6	Talin 2(predicted) OS=Gallus gallus C	TLN2	270.26
A0A1D5PXK1	39S ribosomal protein L30, mitochon	MRPL30	18.109
E1C1Q0	LIM zinc-binding domain-containing	LYAR	42.1
E1BQN9	Calcyclin-binding protein OS=Gallus	CACYBP	25.593
A0A1D5PF55	Double-stranded RNA-binding protei	STAU2	66.934

E1C377	Speckle type BTB/POZ protein like(p	SPOPL	40.621
A0A1L1RPK6	DENN domain containing 5B(predict	DENND5B	146.6
A0A1D5PHE8	RNA polymerase-associated protein I	LEO1	74.772
F1NR75	Tubulin polyglutamylase TTLL5 (Fra	TTLL5	144.89
A0A3Q2TTD8	A-kinase anchoring protein 8 like(pre	AKAP8L	80.041
A0A1D5P5X8	Tim44 domain-containing protein OS	MRPL45	46.477
E1BQM1	Cysteine rich with EGF like domains	CRELD2	39.497
A0A3Q2TWH1	Propionyl-CoA carboxylase subunit a	PCCA	73.878
F1NAX3	Glycerol-3-phosphate phosphatase OS	PGP	33.029
A0A1D5NXE4	N-acetyltransferase domain-containin	LOC107049486	19.004
E1BYZ4	Family with sequence similarity 114 r	FAM114A2	54.369
A0A3Q2TYI7	RING-type E3 ubiquitin transferase C	BRCA1	190.87
A0A3Q3AB26	Far upstream element binding protein	FUBP3	60.926
F1NV49	ATP-dependent RNA helicase DDX1	DDX1	82.526
A0A1D5PF01	PDZ domain-containing protein OS=(	NUR2L	86.944
A0A3Q2U2U7	MAPK regulated corepressor interact	FAM195A	14.141
E1BYQ6	Nucleoprotein TPR OS=Gallus gallus	TPR	276.92
A0A3Q2U596	Lamin-A OS=Gallus gallus OX=9031	LMNA	73.683
P08267	Ferritin heavy chain OS=Gallus gallu	FTH	21.092
P47838	40S ribosomal protein S6 OS=Gallus	RPS6	28.654
A0A1D5PBA6	Dynactin subunit 4(predicted) OS=Ga	DCTN4	52.43
E1C700	Laminin beta 2-like chain(predicted) (	LAMB2	179.1
Q5ZMU9	Transitional endoplasmic reticulum A	VCP	89.324
F1NZH9	FtsJ domain-containing protein OS=C	MRM2	27.423
A0A3Q2UHG8	Guanine deaminase OS=Gallus gallus	GDA	50.28
Q90855	Glutamate receptor OS=Gallus gallus	GluR1	101.69
A0A3Q3AA18	AFG1 like ATPase(predicted) OS=Ga	LACE1	51.033
F1N9Z7	Succinyl-CoA:3-ketoacid-coenzyme A	OXCT1	56.173
Q5F3D2	Heterogeneous nuclear ribonucleopro	HNRNPH3	36.656
Q5ZJ66	Probable glutamate--tRNA ligase, mi	EARS2	56.954
F1NEQ2	PPM-type phosphatase domain-conta	TAB1	54.771
E1BWG7	Parathion hydrolase-related protein O	PTER	39.056
E1BVP0	CDK-activating kinase assembly facto	MNAT1	35.763

F1NTQ2	Beta-hexosaminidase OS=Gallus gall	HEXB	63.008
F1P526	Protein phosphatase methylesterase 1	PPME1	42.609
P84175	40S ribosomal protein S12 OS=Gallu	RPS12	14.545
A0A1L1RVY5	Nucleoporin 54(predicted) OS=Gallu	NUP54	55.228
A0A3Q2U1D7	Dynamin GTPase OS=Gallus gallus (	DNM1	95.434
A0A1D5P5B6	Kinesin family member 13B(predicte	KIF13B	194.51
Q5ZL58	Small nuclear ribonucleoprotein Sm I	SNRPD3	13.916
A0A1D5PL53	Thioredoxin-disulfide reductase OS=(	TXNRD3	66.4
Q5ZK05	Toll-interacting protein OS=Gallus ga	TOLLIP	30.54
Q5ZKF6	7-methylguanosine phosphate-specifi	NT5C3B	33.109
A0A3Q2UGT3	Cadherin-2 OS=Gallus gallus OX=90	CDH2	93.905
A0A1D5NWZ8	RNA_pol_L_2 domain-containing pr	POLR2J	13.293
P58268	Zinc finger protein ubi-d4 OS=Gallus	REQ	45.883
E1BSH9	Hcy-binding domain-containing prote	BHMT2	45.01
Q98TF8	60S ribosomal protein L22 OS=Gallu	RPL22	14.729
F1P541	Ubiquitin carboxyl-terminal hydrolas	UCHL5	37.833
Q5ZK23	Glutaredoxin domain-containing prot	GLRX5	17.425
A0A1D5P973	Secretion associated Ras related GTP	SAR1B	22.391
A0A3Q2UIQ2	Zinc finger protein 438(predicted) OS	ZNF438	88.43
F1NIU3	Inter-alpha inhibitor heavy chain 2(pr	ITIH2	106.51
A0A1L1RN94	Zinc finger protein 622 OS=Gallus ga	ZNF622	41.531
A0A1D5PWF0	COMM domain-containing protein 5	COMMD5	22.965
F1NI31	Prominin-1-A(predicted) OS=Gallus	PROM1L	86.365
A0A1D5NYY8	Dipeptidyl peptidase 7(predicted) OS	DPP7	62.122
Q5ZME2	Malate dehydrogenase, cytoplasmic C	MDH1	36.543
A0A1L1RJY0	Neurocrescin(predicted) OS=Gallus g	RABEP1	90.479
F1NC26	Heat shock protein family A (Hsp70)	HSPA4L	94.75
A0A1L1RUE7	Proteasome subunit beta OS=Gallus g	LOC107049719	21.314
Q5ZJV3	DnaJ heat shock protein family (Hsp4	DNAJA1	44.706
F1N9I1	FHA domain-containing protein OS=	PPP1R8	38.758
F1NWG2	Cathepsin C OS=Gallus gallus OX=9	CTSC	59.406
Q5ZHP4	Tumor protein D52(predicted) OS=G	TPD52	19.86
F1NKJ7	AB hydrolase-1 domain-containing pr	ABHD10	31.965

Q5ZJI3	DUF1713 domain-containing protein	AURKAIP1	23.413
A0A3Q2UHI2	Taxilin alpha(predicted) OS=Gallus g	TXLNA	59.763
A1EA72	Muscleblind-like 1 isoform 5 OS=Ga	MBNL1	35.751
R4GH26	Peptidylprolyl isomerase OS=Gallus g	FKBP10	62.353
Q7ZT27	p15INK4b tumor suppressor OS=Gal	CDKN2B	14.504
F7BFT4	Eukaryotic translation initiation facto	EIF4E3	22.774
A0A3Q2UHC3	Galactocerebrosidase OS=Gallus gall	GALC	75.657
A0A1D5P411	RRM domain-containing protein OS=	FUS	49.402
F1P4M2	Non-specific serine/threonine protein	CSNK1G2	65.971
Q5ZMK7	Ubiquitin-fold modifier 1 OS=Gallus	UFM1	9.0874
Q5F3W6	14-3-3 protein gamma OS=Gallus gal	YWHAG	28.23
Q2XP49	Deoxyribonuclease II OS=Gallus gall	DNASE2	40.883
E1BV78	Fibrinogen C-terminal domain-contai	FGG	49.954
E1C7G0	Armadillo repeat containing 6(predict	ARMC6	50.875
Q5ZLE6	Eukaryotic translation initiation facto	EIF3H	39.536
A0A1D5NU81	ZP domain-containing protein OS=Ga	TGFBR3	91.689
Q8UVF8	Inhibitor of apoptosis protein 3 OS=C	IAP3	55.251
A0A1D5PSD3	Phosphodiesterase 4D interacting pro	--	83.797
R9PXM3	Ubiquitin carboxyl-terminal hydrolase	USP47	154.85
Q5ZIH1	Heterogeneous nuclear ribonucleopro	HNRNPD	29.355
E1C1J4	Tetratricopeptide repeat domain 25(pr	TTC25	72.286
E1C7F8	Rae1 protein homolog OS=Gallus gal	RAE1	47.398
A0A3Q2UH51	LUC7 like(predicted) OS=Gallus gall	LUC7L	39.224
A0A1D5PPQ2	FABP domain-containing protein OS	FABP5	31.369
Q5ZK22	Sorting nexin OS=Gallus gallus OX=	SNX32	46.609
A0A1L1RNY8	Histone H2A OS=Gallus gallus OX=9	H2AFX	15.045
A0A1D5PT30	Ribosomal_S10 domain-containing p	MRPL48	23.874
Q90679	Thymocyte nuclear protein 1 OS=Gal	THYN1	27.966
A0A3Q2UC95	VWFD domain-containing protein OS	--	452.25
F1P1A5	TRANSKETOLASE_1 domain-conta	TKTL1	68.453
E1BS97	Non-specific serine/threonine protein	PAK2	57.545
R4GLW9	Nuclear mitotic apparatus protein(pre	NUMA1	241.34
F1NII6	Ribonucloprotein OS=Gallus gallus C	--	14.074

A0A1D5PM26	p53 and DNA damage-regulated prot	PDRG1	14.925
Q08705	Transcriptional repressor CTCF OS=	CTCF	82.828
A0A1D5NZN9	PID domain-containing protein OS=C	NOS1AP	69.557
E1C908	FYN binding protein 1(predicted) OS	FYB	92.203
A0A1D5PNL6	Zinc finger MYND-type containing 1	ZMYND11	70.904
A0A3Q3B1M1	Nuclear receptor coactivator 3(predic	NCOA3	146.6
R4GJX0	Bis(5'-nucleosyl)-tetraphosphatase [a	NUDT2	16.953
A0A1L1RXN8	CoA carboxyltransferase C-terminal	MCCC2L	62.788
A0A1D5PJE2	Tetratricopeptide repeat domain 17(p	TTC17	130.31
A0A3S5ZPA4	RRM domain-containing protein OS=	SRSF11	59.575
F1NPD1	Sulfotransferase OS=Gallus gallus O	SULT	36.157
P09206	Tubulin beta-3 chain OS=Gallus gall	--	49.86
Q2ACD6	Chicken-II-type gonadotropin-releasi	MRPS26	33.243
E1C7W8	PH domain-containing protein OS=G	PHLDB2	140.16
E1BSD8	DNA-directed RNA polymerases I, II	POLR2H	17.143
A0A3Q2TZ64	RNA binding motif single stranded i	RBMS1	40.256
E1BXG0	Peptidyl-prolyl cis-trans isomerase O	PPIH	19.205
Q9PTR5	Lissencephaly-1 homolog OS=Gallus	PAFAH1B1	46.664
F1N9I5	ELAV-like protein OS=Gallus gallus	ELAVL1	36.102
F1NH79	DNA helicase OS=Gallus gallus OX=	--	130.23
A0A1D5PG55	SAP30 like OS=Gallus gallus OX=9	--	19.986
E1C726	Leucine rich repeats and immunoglob	LRIG1	130.97
Q5ZKR8	DNA helicase OS=Gallus gallus OX=	MCM6	93.302
A0A1D5P607	Mitochondrial ribosomal protein S9(	MRPS9	38.384
A0A1L1RLH7	26S proteasome regulatory subunit 4	PSMC1	56.143
A0A1D5PD87	WD_REPEATS_REGION domain-c	STRN3	79.364
Q5ZL54	KH homology domain-containing pro	KHDC4	65.051
P05153	Phosphoenolpyruvate carboxykinase,	PCK1	69.527
F1P0X7	Caspase-1 (Fragment)(predicted) OS=	CASP1	44.706
A0A3Q2U296	DDB1-and CUL4-associated factor 8	--	41.978
Q5ZJ81	Endophilin-B2 OS=Gallus gallus OX	SH3GLB2	44.888
A0A3Q3B116	Biogenesis of lysosome-related organ	BLOC1S1	14.424
A0A1D5NYJ2	Lon protease homolog, mitochondrial	LONP1	103.65

E1BRE2	NAD-dependent protein deacylase sir	SIRT5	33.956
F1P099	Sulfatase domain-containing protein (	ARSB	58.445
E1C4H6	U6 snRNA-associated Sm-like protei	LSM4	15.341
A0A1D5PDK5	Calponin-homology (CH) domain-co	IGSF9	20.599
E1C8Z9	PID domain-containing protein OS=C	DAB2	76.862
F1NMU0	Cytochrome c oxidase assembly prote	COX15	42.838
F1ND59	Heat shock protein family A (Hsp70)	HSPA13	52.117
E1C538	Nudix hydrolase domain-containing p	NUDT21	51.064
Q90835	Elongation factor 1-alpha 1 OS=Gallu	EEF1A	50.156
F1N915	THO complex subunit 7 homolog OS	THOC7	23.873
A0A1L1RZD1	Splicing factor, arginine/serine-rich 7	SRSF7	15.621
A0A1D5PQI8	Shroom family member 2(predicted) (	SHROOM2	196.26
Q5ZMV5	Actin-related protein 2/3 complex sub	ARPC5	16.452
E1BZP0	Prolyl-tRNA synthetase OS=Gallus g	PARS2	66.07
Q5ZM91	cAMP-dependent protein kinase type	PRKAR1A	43.35
F1N8Z4	RuvB-like helicase OS=Gallus gallus	RUVBL1	50.179
A0A1L1S0C5	Sorting nexin-1 OS=Gallus gallus OX	SNX1	58.092
A0A3Q2TVA9	Peptidase S1 domain-containing prote	CFD	49.504
E1C8V9	Small nuclear ribonucleoprotein G OS	SNRPGP15	8.462
F1NG46	Mitochondrial transcription factor A(	TFAM	29.966
A0A3S5ZPN3	DEAD box protein 5 OS=Gallus gallu	DDX5	67.673
A0A1D5PA73	Glycerol-3-phosphate dehydrogenase	--	13.043
Q98TA4	Mannose-binding protein OS=Gallus	MBL	27.396
F1NF89	40S ribosomal protein S24 OS=Gallu	RPS24	15.197
E1BRW8	Protein-ribulosamine 3-kinase OS=Ga	FN3K	35.058
A0A3Q2UAA5	Ig-like domain-containing protein OS	LOC107051274	12.905
A0A3Q2U3R6	Heat shock protein family H (Hsp110	HSPH1	94.351
E1C477	Ubiquilin 1(predicted) OS=Gallus gal	UBQLN1	62.998
P62764	Visinin-like protein 1 OS=Gallus gall	VSNL1	22.142
A0A1D5P8I3	Splicing factor proline and glutamine	SFPQ	69.532
F1NCC9	HMG box domain-containing protein	SMARCE1	44.862
E1C6C2	Rnmt-activating mini protein(predicte	FAM103A1	14.002
A0A3Q2UAT4	Ubiquitin-like domain-containing pro	HERPUD1	42.255

P00368	Glutamate dehydrogenase 1, mitochor	GLUD1	55.196
F1NL52	NFKB repressing factor(predicted) O	NKRF	86.335
A0A3Q2UCR0	RRM domain-containing protein OS=	CSTF2	60.154
F1NCE3	Pre-mRNA processing factor 40 hom	PRPF40A	96.981
F1NUU0	Dedicator of cytokinesis 5(predicted)	DOCK5	216.19
F1NMZ1	Membrane associated guanylate kinas	MAGI1	136.97
A0A3Q3B0L2	PrdX deacylase domain-containing pr	PRORSD1P	19.337
F1NJV0	NAD kinase 2, mitochondrial OS=Ga	NADK2	49.294
A0A3Q3A8D7	N-myc downstream regulated 1(predi	NDRG1	44.218
A0A1L1RZU7	Ribosomal RNA-processing protein 4	EXOSC2	32.437
A0A1D5PEW1	Ras suppressor protein 1(predicted) C	RSU1	31.524
E1BUT3	CD34 molecule(predicted) OS=Gallu	CD34	40.513
F1NKV0	J domain-containing protein OS=Gall	DNAJB4	38.277
F1NF87	Exocyst complex component 5 OS=G	EXOC5	98.528
E1BZN8	HGF activator(predicted) OS=Gallus	F12	57.771
E1C1I6	Aldo_ket_red domain-containing prot	LOC772271	35.955
F1P0Z3	Phospholipase B-like OS=Gallus gall	PLBD2	62.001
Q05744	Cathepsin D OS=Gallus gallus OX=9	CTSD	43.298
E1BUA8	Protein BCCIP homolog OS=Gallus g	BCCIP	34.514
Q9DDD4	Tetranectin OS=Gallus gallus OX=90	CLEC3B	22.172
F1P4X4	Mitochondrial import receptor subuni	TOMM34	51.899
F1NK34	Tight junction protein(predicted) OS=	TJP2	130.73
A0A3Q2U096	Fork-head domain-containing protein	FOXI2	12.555
A0A3Q2UEF8	RNA binding motif protein 10(predic	--	31.08
F1P2Y8	Ribosome biogenesis protein WDR12	WDR12	50.607
F1NEM3	Suppressor of cytokine signaling 4(pr	SOCS4	49.952
Q5ZKZ5	Signal recognition particle subunit SF	SRP72	74.484
F1NAW2	CD2 associated protein(predicted) OS	CD2AP	71.216
A0A1D5PZK7	PKS_AT domain-containing protein (	MCAT	43.212
A0A1L1RJR6	Methyltransferase like 3(predicted) O	METTL3	60.97
A0A1D5PYW8	Transcription and mRNA export facto	ENY2	11.013
Q5ZI72	Heterogeneous nuclear ribonucleopro	HNRNPDL	33.443
A0A3Q2U5U9	B30.2/SPRY domain-containing prot	--	19.858

F1NLF2	KH domain-containing, RNA-binding	KHDRBS1	46.525
E1C3B3	Sec2p domain-containing protein OS=	RAB3IL1	42.522
F1NGW4	WD_REPEATS_REGION domain-co	WDR5B	36.595
A0A3Q3A2G8	ELKS/RAB6-interacting/CAST famil	ERC1	117.3
A0A1D5P624	Ubiquilin 4(predicted) OS=Gallus gal	UBQLN4	64.027
A0A1L1RZJ6	ATP synthase subunit alpha OS=Gall	ATP5A1W	60.027
A0A1D5P7Q3	MRP-S28 domain-containing protein	MRPS35	44.094
A0A1L1RSB5	Sm protein F OS=Gallus gallus OX=9	SNRPF	9.7251
F1NAY8	Diphosphomevalonate decarboxylase	MVD	67.998
A0A3Q2UEZ5	Tax1 binding protein 1(predicted) OS	TAX1BP1	85.875
A0A1D5PCV7	G3BP stress granule assembly factor	G3BP2	50.743
Q5ZIR1	Endophilin-B1 OS=Gallus gallus OX	SH3GLB1	40.795
E1BXI3	Programmed cell death 5(predicted) (	PDCD5	14.219
A0A1D5PCF5	Insulin like growth factor binding pro	IGFBP7	33.074
F1NCS2	EWS RNA binding protein 1(predicte	EWSR1	72.362
A1IIE6	General transcription facter IIA subur	GTF2A1	41.754
P70082	Histone H2A.J OS=Gallus gallus OX	H2A-IX	14.016
P79781	Ubiquitin-40S ribosomal protein S27a	RPS27A	17.979
A0A1D5NYA7	Sema domain-containing protein OS=	PLXNB2	171.53
A0A1D5PXG0	Heterogeneous nuclear ribonucleopro	HNRNPK	47.102
A0A3Q2TW93	RRM domain-containing protein OS=	RALYL	32.724
Q5ZHM8	Syndecan binding protein(predicted) (	SDCBP	32.057
F1N832	EMG1 N1-specific pseudouridine me	EMG1	25.566
E1BS31	Membrane palmitoylated protein 6(pr	MPP6	66.035
Q5ZMP2	t-SNARE coiled-coil homology doma	STX7	29.342
F1NS24	zinc_ribbon_16 domain-containing pr	MIOS	98.313
Q9DG45	Pterin-4-alpha-carbinolamine dehydra	PCBD2	11.768
F1NZB0	Plexin A4 OS=Gallus gallus OX=903	--	212.39
A0A1D5PKI2	Copper homeostasis protein cutC hon	CUTC	53.346
F1NI04	N-acetylglucosamine-6-sulfatase OS=	GNS	60.635
A0A1D5PIZ1	Peptidase_M3 domain-containing pro	THOP1	78.308
F1NVJ6	RNA helicase OS=Gallus gallus OX=	DDX43	49.493
A0A3Q2TRS9	Hyaluronan binding protein 2(predicte	HABP2	61.584

Q5ZJT7	Elongin-C OS=Gallus gallus OX=903	TCEB1	12.473
A0A1D5PMT8	60S acidic ribosomal protein P2 OS=	RPLP2	11.842
F1NTC8	PDZ and LIM domain 5(predicted) O	PDLIM5	64.091
F1NLK0	Guanine nucleotide-binding protein-li	GNL3	63.983
Q5F334	Leucine-rich repeat-containing protei	LRRC59	38.665
E1BU66	60S ribosomal protein L38 OS=Gallu	RPL38	8.2178
F1NSS6	Enoyl-CoA hydratase domain contain	ECHDC2	30.801
F1NMN0	Cleavage and polyadenylation specifi	CPSF2	88.695
A0A1L1RM96	WD_REPEATS_REGION domain-co	PLRG1	57.121
F1P558	Histone-lysine N-methyltransferase S	SETD7	40.64
A0A1L1RLC7	NAD(P)-bd_dom domain-containing	HTATIP2	24.952
A0A1D5PTE8	Glia maturation factor OS=Gallus gal	GMFB	16.592
F6QGI8	Fructose-bisphosphatase OS=Gallus g	FBP1	51.83
F1NFT8	WD repeat-containing protein 61 OS=	WDR61	33.275
P67869	Casein kinase II subunit beta OS=Gal	CSNK2B	24.942
E1C0P2	Rab5 GDP/GTP exchange factor(prec	--	56.846
A0A3Q2U1Z4	28s ribosomal protein mitochondrial(	MRPS21	36.24
A0A1L1RNJ2	Enoyl-CoA hydratase domain contain	ECHDC3	31.213
E1C123	Aha1_N domain-containing protein C	AHSA1	38.045
F1NMY1	14-3-3 protein gamma OS=Gallus gal	YWHAG	28.302
F1NBT0	Serine/threonine-protein kinase 10 OS	STK10	112.5
A0A1L1RNT6	60S ribosomal protein L14 OS=Gallu	RPL14	13.946
P28173	Amidophosphoribosyltransferase OS=	PPAT	56.257
A0A3Q2UMP0	Guanine nucleotide-binding protein su	LOC420562	49.524
A0A1L1RPU0	Mitochondrial ribosomal protein L1(p	MRPL1	37.856
E1BYD2	WD_REPEATS_REGION domain-co	NUP37	36.949
A0A1L1RJL0	WD_REPEATS_REGION domain-co	THOC3	42.117
Q5ZK63	Activated RNA polymerase II transcr	SUB1	14.237
A0A1D5P908	DUF3456 domain-containing protein	CNPY3	28.116
F1P191	ADP-ribose glycohydrolase ARH3(pr	ADPRHL2	39.91
E1BZ71	THUMP domain-containing protein C	THUMPD2	45.605
A0A3Q2U1A2	Ig-like domain-containing protein OS	--	9.7514
E1BZ75	U6 snRNA-associated Sm-like protei	LSM8	10.431

F1NPD3	60S ribosomal protein L18a OS=Gall	RPL18A	17.506
E1BW98	WD_REPEATS_REGION domain-co	GNB4	37.53
E1C281	PHD finger protein 6, transcript varia	PHF6	40.951
E1C1L2	Transcription factor CP2 like 1(predic	TFCP2L1	54.71
A0A1L1RM56	Peptide-methionine (S)-S-oxide reduc	MSRA	24.677
F1NQF6	Alpha-mannosidase OS=Gallus gallus	MAN2B2	118.7
A0A1D5PAE4	PWWP domain-containing protein OS	HDGF	26.248
E1C4X4	Transcription elongation factor SPT4	SUPT4H1	13.221
A0A1D5PWV5	Ubiquitin-like protein 5 OS=Gallus g	UBL5	8.661
A0A3Q2UAF1	GRIP1-associated protein 1-like(pred	GRIPAP1	77.656
A0A3Q2UK97	Pre-mRNA-splicing factor RBM22 O	RBM22	43.484
A0A3Q2TZW3	Formin binding protein 1(predicted) (	FNBP1	64.084
A0A3Q2TZ50	Non-specific serine/threonine protein	LOC101750560	69.979
F1P3U1	MICOS complex subunit MIC60 OS=	IMMT	79.223
Q5ZJJ2	Replication protein A 70 kDa DNA-b	RPA1	68
E1C8Y9	RRM domain-containing protein OS=	SRSF3	19.329
A0A1D5P5X1	Reticulocalbin 2(predicted) OS=Gallu	RCN2	35.711
P79760	Cytochrome P450 1A4 OS=Gallus ga	CYP1A4	59.361
A0A1D5NYI2	NAD-dependent protein deacetylase (	SIRT3	38.723
E1C9C4	3-hydroxy-3-methylglutaryl coenzym	HMGCS2	51.953
Q5ZMG9	CCT-alpha OS=Gallus gallus OX=90	TCP1	60.466
Q5ZJ27	Protein Hook homolog 1 OS=Gallus g	HOOK1	82.979
A0A1L1RZK3	ANK_REP_REGION domain-contain	KANK3	105.99
Q04584	Zyxin OS=Gallus gallus OX=9031 G	ZYX	58.537
Q5ZJS2	Peroxisomal biogenesis factor 11 gam	PEX11G	25.367
Q5ZIZ2	SET and MYND domain-containing p	SMYD5	46.967
A0A1L1RQQ1	NAD(P)H-hydrate epimerase OS=Ga	APOA1BP	25.939
F1NDR9	NIF3-like protein 1 OS=Gallus gallus	NIF3L1	41.156
F1NRI3	WD repeat-containing protein 1 OS=(	WDR1	66.584
E1C0D8	Peptidase_M3 domain-containing pro	NLN	80.375
A0A3Q2UCF3	Eukaryotic translation initiation facto	EIF3G	33.647
A0A1D5P2P4	Spectrin repeat containing nuclear en	SYNE2	798.37
A0A1D5NT61	Glutamate dehydrogenase (NAD(P)(+	GLUD2	60.861

F1NBA4	Glutaredoxin-1 OS=Gallus gallus OX	GLRX	11.369
A0A1D5PNU3	WD_REPEATS_REGION domain-co	DPH7	54.384
A0A1L1RP66	AAA domain-containing protein OS=	PSMC6	45.797
Q5ZLS5	Elongation factor for RNA polymeras	ELL	68.169
E1BYN9	Pribosyltran_N domain-containing pr	PRPSAP1	42.427
A0A3Q2UMN6	CwfJ_C_1 domain-containing protein	CWF19L1	75.052
Q5ZMH9	Ribosome biogenesis protein NSA2 h	NSA2	30.052
Q04929	Adapter molecule crk OS=Gallus gall	CRK	33.805
A0A1D5PJ40	Clathrin light chain OS=Gallus gallus	CLTB	23.121
P63283	SUMO-conjugating enzyme UBC9 O	UBE2I	18.007
A0A3Q2U7V2	PDZ domain-containing protein OS=(	GIPC1	55.056
A0A1D5PKI8	Glypican 4(predicted) OS=Gallus gal	GPC4	63.086
A0A1D5PMY4	Alanine--tRNA ligase OS=Gallus gal	AARS2	104.42
F1NLV4	WD_REPEATS_REGION domain-co	GNB1	37.29
F1NKZ7	Fe2OG dioxygenase domain-containi	OGFOD3	34.252
E1C6S7	Coiled-coil and C2 domain containing	CC2D1A	97.792
F1NGI6	N-sulfoglucosamine sulfohydrolase(p	SGSH	63.513
A0A1D5PG52	Ubiquitin carboxyl-terminal hydrolase	FAM63A	50.467
A0A1L1RL78	Cathepsin L1(predicted) OS=Gallus g	CTSV	36.626
F1P3C8	WD_REPEATS_REGION domain-co	DYNC1I2	68.214
Q5ZJM9	Small ubiquitin-related modifier 2 OS	SUMO2	10.871
A0A1D5PGV4	Proteasome assembly chaperone 4(pre	PSMG4	13.372
A0A1D5NUJ3	2-oxoisovalerate dehydrogenase subu	BCKDHA	50.22
Q5ZLX3	WD_REPEATS_REGION domain-co	RBBP5	59.187
E1BUP6	Protein disulfide isomerase family A	PDIA5	77.295
A0A3Q2U2R1	C-terminal binding protein 1(predicte	CTBP1	46.845
Q5ZMI4	Major vault protein OS=Gallus gallus	MVP	93.821
A0A1D5P4W0	Regulator of chromosome condensati	RCC1	44.522
Q5ZLI2	Proteasome subunit alpha type OS=G	PSMA3	28.481
Q5ZLR5	Cytochrome b-c1 complex subunit Ri	UQCRFS1	29.386
F1P332	Methylcrotonoyl-CoA carboxylase be	MCCC2L	65.846
Q5F415	TBC1 domain family member 23 OS=	TBC1D23	76.01
F1NLA0	Glutamyl-tRNA(Gln) amidotransferas	QRSL1	57.001

F1NCZ4	28S ribosomal protein S18c, mitocho	MRPS18C	16.241
A0A1L1RZA5	Fibrinogen C-terminal domain-contai	ANGPTL3	53.534
P07850	Sulfite oxidase OS=Gallus gallus OX	SUOX	50.204
A0A1D5PZT4	GTP cyclohydrolase 1 feedback regul	GCHFR	9.6321
F1NLE4	Glutathione synthetase OS=Gallus ga	GSS	52.285
P13127	F-actin-capping protein subunit alpha	CAPZA1	32.959
A0A3Q2UID9	Golgin B1(predicted) OS=Gallus gall	GOLGB1	360.34
F1NZP0	Twinfilin-2 OS=Gallus gallus OX=9(	TWF2	39.805
A0A3Q2UB37	Malic enzyme OS=Gallus gallus OX=	ME1	72.704
Q03481	Alpha8 subunit of nicotinic acetylcho	CHRNA8	58.705
Q5ZLN5	TAR DNA-binding protein 43 OS=G	TARDBP	44.589
E1C5C5	Putative selenoprotein S(predicted) O	SELENOS	20.956
Q5ZIL3	E2 ubiquitin-conjugating enzyme OS	UBE2K	22.434
A0A1L1RKA0	DnaJ heat shock protein family (Hsp4	DNAJC7	58.878
A0A3Q2TY40	Ribosomal RNA-processing protein 4	EXOSC8	27.855
A0A1D5P102	Discs large MAGUK scaffold protein	DLG1	97.307
A0A1D5PQ66	RNA binding motif protein 5(predicte	RBM10	104.87
Q5ZML5	Protein LSM12 homolog OS=Gallus	LSM12	21.519
A0A1D5PCX2	Charged multivesicular body protein 2	CHMP2A	24.819
E1C129	Cytosolic Fe-S cluster assembly facto	NUBP1	34.542
F1NUK8	WD_REPEATS_REGION domain-co	NWD1	45.49
A0A1D5NZF2	Enhancer of mRNA-decapping protei	EDC3	56.077
Q9PTI4	RING-type E3 ubiquitin transferase C	MKRN1	52.23
A0A3Q2U9M2	Filamin B(predicted) OS=Gallus gallu	FLNB	280.76
P11722	Fibronectin OS=Gallus gallus OX=90	FN1	273.25
A0A1D5NZ72	TRAF-type zinc finger domain contai	TRAFD1	65.263
F1NBX4	60S ribosomal protein L27a OS=Gall	RPL27A	16.808
A0A1D5NYF8	GATOR complex protein SEC13 OS=	SEC13	35.18
E1C6V0	Heat shock protein beta-8 OS=Gallus	HSPB8	21.734
A0A1L1RPX5	Folate hydrolase 1(predicted) OS=Ga	FOLH1	84.017
A0A1D5PVR1	Reduced folate transporter OS=Gallus	SLC19A1	55.521
Q05826	CCAAT/enhancer-binding protein be	CEBPB	35.03
A0A3Q3AJD3	WH1 domain-containing protein OS=	--	9.4945

A0A1L1RXF5	Nucleolysin TIAR(predicted) OS=Ga	TIAL1	41.098
E1BWW2	Tumor susceptibility 101 protein(prec	TSG101	44.124
A0A1L1RJ01	Threonine synthase-like 2 OS=Gallus	THNSL2	54.456
A0A3Q2UCN7	Reverse transcriptase domain-contain	--	55.184
A0A1D5P1U9	Ubiquitin carboxyl-terminal hydrolase	USP15	109.06
A0A1D5PE41	Complex I-23kD OS=Gallus gallus O	NDUFS8	23.824
R4GI64	AAA domain-containing protein OS=	SPG7	86.074
A0A3Q2U7I7	RuvB-like helicase OS=Gallus gallus	LOC107051177	51.043
E1C4G6	CDKN2A interacting protein(predicte	CDKN2AIP	68.404
Q5ZHY5	E3 ubiquitin-protein ligase CHIP OS=	STUB1	35.661
Q5ZJZ2	60S ribosomal protein L3(predicted) (	RPL3	46.085
F1NJ25	COMM domain-containing protein 3	--	21.246
A0A1D5NTH5	Twinfilin actin binding protein 1(prec	TWF1	55.201
A0A1L1RJ88	Acetylserotonin O-methyltransferase	ASMTL	69.496
Q789A6	Nonmuscle myosin heavy chain OS=(	MYH10	229.04
E1C460	Phenazine biosynthesis like protein de	PBLD	32.205
A0A1D5PC76	AKAP7_NLS domain-containing pro	AKAP7	50.503
Q5ZHY0	Dihydroorotate dehydrogenase (quinc	DHODH	41.397
Q5ZHW3	WD_REPEATS_REGION domain-cc	BUB3	37.28
F1NEW6	Mitochondrial ribosomal protein L19(	MRPL19	29.67
E1C8P2	3'-phosphoadenosine-5'-phosphosulfa	PAPSS1	70.805
A0A1D5PYU9	Biphenyl hydrolase like(predicted) OS	BPHL	31.885
A0A1D5P583	FSA_C domain-containing protein OS	KIAA1109	548.86
A0A3Q2TS28	Protein HGH1 homolog OS=Gallus g	--	22.319
A0A1D5PTQ3	ANK_REP_REGION domain-contain	NFKBIB	34.004
Q5ZMH1	Septin-2 OS=Gallus gallus OX=9031	SEPTIN2	40.225
E1C4L9	DNA methyltransferase 1 associated p	DMAP1	53.302
A0A1D5PZS0	Pre-mRNA-processing factor 19 OS=	PRPF19	55.067
Q90631	Kinectin OS=Gallus gallus OX=9031	KTN1	155.97
A0A1D5NUQ4	Ribos_L4_asso_C domain-containing	RPL4	43.888
A0A3Q2TYL5	Ubiquitin-like modifier activating enz	--	41.235
E1BSQ5	Zinc finger FYVE-type containing 1(p	ZFYVE1	87.707
A0A3Q3AE84	PDZ domain-containing protein OS=(	GOPC	54.187

F1NCJ8	Methyltranfer_dom domain-containin	AS3MT	56.964
R4GG77	SNARE-associated protein Snapin OS	SNAPIN	14.129
F1NMJ2	Nucleoside-diphosphate kinase OS=C	AK4	19.771
F6VMK6	WD_REPEATS_REGION domain-co	WDR92	39.363
A0A1D5NXM7	MPN domain-containing protein OS=	NPLOC4	66.892
F6UW25	YTH domain-containing protein OS=	YTHDF1	55.769
F1NCV8	Endoplasmic reticulum lectin 1(predi	ERLEC1	51.177
A0A1D5P0D2	Chromosome 12 open reading frame	C33H12orf10	52.351
A0A1L1RJ78	Nucleophosmin OS=Gallus gallus OX	NPM1	43.828
E1C3M4	Adenylyltransferase and sulfurtransfe	MOCS3	50.819
E1C1R5	PUM-HD domain-containing protein	PUM3	73.009
Q5ZLN1	Phosphoglycerate mutase 1 OS=Gallu	PGAM1	28.898
A0A1D5PN67	Pyroglutamyl-peptidase I OS=Gallus	PGPEP1	23.855
F1NCQ3	Protein kinase domain-containing pro	MYLKSML	42.58
Q5F372	E3 ubiquitin-protein ligase OS=Gallu	WWP1	104.73
F1P3C2	Unk zinc finger(predicted) OS=Gallu	UNK	90.384
F1NKY8	PDZ domain-containing protein OS=(	GIPC2	34.869
F1P0L9	Protein kinase C OS=Gallus gallus O	PRKCI	52.834
A0A1D5P3Q3	Guanidinoacetate N-methyltransferas	GAMT	26.971
F1NBB0	2-methoxy-6-polyprenyl-1,4-benzoqu	COQ5	35.113
F1NDS5	Prefoldin subunit 1(predicted) OS=Ga	PFDN1	14.222
F1NEF6	Acyl-CoA dehydrogenase family mer	ACAD9	67.634
Q5ZHY2	Myelin expression factor 2(predicted)	MYEF2	65.699
A0A1D5P592	Propionyl-CoA carboxylase subunit b	PCCB	57.502
E1BVT9	Intersectin 2(predicted) OS=Gallus ga	ITSN2	198.44
A0A1D5NU66	Receptor protein-tyrosine kinase OS=	PDGFRB	120.22
F1NH93	40S ribosomal protein S20 OS=Gallu	RPS20	13.374
A0A1D5PUK8	Rab GTPase-activating protein 1-like	RABGAP1L	28.971
A0A3Q2TSX5	Heterogeneous nuclear ribonucleopro	LOC101749377	56.307
F1NTM7	Aspartate aminotransferase OS=Gallu	GOT1	45.908
R4GJ59	Seryl-tRNA synthetase OS=Gallus ga	SARS	71.294
F1NF19	Synaptic functional regulator FMR1 (	FMR1	61.838
A0A1L1RSQ1	SAM_MT_RSMB_NOP domain-con	NSUN4	44.776

A0A3Q2UCK3	Tubulin-folding cofactor C OS=Gallu	TBCC	38.813
Q5ZIE3	Signal recognition particle 14 kDa pr	SRP14	12.528
A0A1D5P6D2	COP9 signalosome complex subunit 8	COPS8	25.608
E1BQG4	G protein-coupled receptor kinase OS	GRK5	67.683
E1BV94	Female-lethal(2)D homolog OS=Gall	WTAP	44.36
F1NUQ3	Heart fatty acid binding protein OS=(	FABP3	14.844
P68497	Metallothionein OS=Gallus gallus O)	--	6.4616
E1BRX6	SKICH domain-containing protein OS	CALCOCO2	41.995
Q76KB1	Heparan sulfate 2-O-sulfotransferase	HS2ST1	41.701
A0A1D5PG47	Cathepsin H(predicted) OS=Gallus ga	CTSH	43.765
F1P1B7	Nucleosome assembly protein 1-like 4	NAP1L4	42.895
Q8JG30	Sulfotransferase family cytosolic 1B r	SULT1B1	34.073
R4GFR8	J domain-containing protein OS=Gall	DNAJB2	31.091
F1P2M3	IF3_N domain-containing protein OS	MTIF3	31.352
A0A3Q2U039	YLP motif containing 1(predicted) OS	YLPM1	248.77
A0A1D5NUG1	CGG triplet repeat binding protein 1(	CGGBP1	19.031
A0A1D5PKP4	Striatin interacting protein 1(predicte	STRIP1	93.424
F1NZ31	Myeloid derived growth factor(predic	MYDGF	18.531
H9L0I5	Mitochondrial ribosomal protein L10	MRPL10	28.99
E1BUR3	DDRGK domain-containing protein 1	DDRGK1	25.736
A0A1D5PYD2	B30.2/SPRY domain-containing prot	LOC420374	52.977
E1BS61	Rap guanine nucleotide exchange fac	RAPGEF1	117.91
A0A1D5PXQ2	Sequestosome 1(predicted) OS=Gallu	SQSTM1	46.698
P55168	Cyclin-C OS=Gallus gallus OX=9031	CCNC	33.258
E1BRR7	FMN hydroxy acid dehydrogenase do	HAO1	40.545
A0A3S5ZPP7	SH2 domain-containing protein OS=(	LCP2	61.984
Q5F485	ATP-dependent RNA helicase DDX4	DDX42	103.05
E1BRU3	RNA binding motif protein 39, transc	RBM23	58.538
A0A1L1RNQ9	Septin OS=Gallus gallus OX=9031 G	SEPT6	48
A0A1D5NT45	KxDL motif-containing protein 1 OS=	KXD1	19.896
A0A1D5PWP5	Phosphorylase b kinase regulatory sul	PHKA2	135.26
E1BZH7	Vacuolar-sorting protein SNF8 OS=C	SNF8	28.885
F1NRH6	Rho-associated protein kinase OS=Ga	ROCK1	158.67

A0A1D5PLB6	UBA domain-containing protein OS=	VPS13D	485.47
Q5ZMQ4	Dynein light intermediate chain OS=(	DYNC1LI2	53.902
Q98953	Protein S100-A6 OS=Gallus gallus O	S100A6	10.276
A0A1L1RPQ6	Tropomyosin 4(predicted) OS=Gallus	TPM4	28.639
P00356	Glyceraldehyde-3-phosphate dehydro	GAPDH	35.704
Q5F3F5	G-protein coupled receptor-associated	LMBRD2	80.29
F1P4U8	Putative LOC102087742(predicted) (	LOC100857197	70.093
Q90WA9	Fatty acid-binding protein, liver OS=(	LFABP	14.192
F1NK40	Alpha-2-macroglobulin-like 1 (Fragm	A2ML4	163.34
A0A1D5PTL7	Annexin OS=Gallus gallus OX=9031	ANXA4	35.509
F1NI93	Probable E3 ubiquitin-protein ligase r	MKRN2	46.49
A0A1D5NXF0	Calmodulin regulated spectrin associa	CAMSAP2	152.56
E1C1G5	Zinc finger CCCH-type containing 18	ZC3H18	101.36
Q8JIG5	Alpha-1-acid glycoprotein OS=Gallus	ogchi	22.321
A0A1D5PPP7	Caspase-6(predicted) OS=Gallus gall	CASP6	34.476
F1NNW2	Acyl-CoA dehydrogenase family mer	ACAD8	50.529
A0A1D5P315	Zinc finger CCCH domain-containing	ZC3H11B	72.728
F1NB70	NAD-dependent protein deacylase OS	SIRT4	35.202
A0A1D5NZ09	Polyribonucleotide nucleotidyltransfe	PNPT1	86.953
F1NAS0	SGT1 homolog, MIS12 kinetochore c	SUGT1	37.212
A0A3Q2U025	GRIP domain-containing protein OS=	GOLGA1	90.256
A0A1D5PJJ8	Huntingtin interacting protein 1(predi	HIP1	113.05
A0A3Q3AL64	RUN and FYVE domain containing 1	RUFY1	71.996
A0A1D5PZ59	Nucleoporin NUP188 (Fragment)(pre	NUP188	225.25
A0A1D5PUI1	Threonyl-tRNA synthetase OS=Gallu	TARS2	77.563
Q5F478	Serine/threonine-protein phosphatase	ANKRD44	107.4
A0A1D5PWH3	PH domain-containing protein OS=G	PLEKHA7	132.42
E1BTS4	Pre-mRNA-splicing factor SPF27 OS	BCAS2	25.934
F1NB68	Paxillin OS=Gallus gallus OX=9031	TGFB1I1	61.059
A0A1D5PH32	Actin-depolymerizing factor OS=Gal	GSN	80.979
F1NWZ1	ArfGAP with GTPase domain, ankyri	AGAP1	89.294
A0A1D5NWC3	FYVE and coiled-coil domain contain	FYCO1	176.94
A0A3Q2UFA5	RING-type E3 ubiquitin transferase C	ZNF598	94.21

F1NXN8	NADH-ubiquinone oxidoreductase 75	NDUFS1	79.594
F6R7D0	18 kDa Sin3-associated polypeptide (	SAP18	20.873
A0A1L1RVY9	Chromosome 19 open reading frame 2	C19orf25	11.683
E1BY41	Phospholipase A2 group XIIB(predict	PLA2G12B	22.614
E1BSG7	WW domain-binding protein 11(pred	WBP11	70.761
A0A3Q2U3U3	Heterogeneous nuclear ribonucleopro	HNRNPA2B1	36.259
A0A1D5PNX0	Alcohol dehydrogenase iron-containi	ADHFE1	50.485
F1NZX3	Isopentenyl-diphosphate Delta-isome	IDI2	32.772
A0A3Q2U485	DEAH-box helicase 8(predicted) OS=	DHX8	126.91
Q5ZIP3	Deoxyhypusine hydroxylase OS=Gall	DOHH	32.875
Q5ZJ46	Transcription factor GA binding prot	GABPA	51.431
E1BWU6	Sema domain-containing protein OS=	PLXNB3	214.93
A0A3Q2U5B6	Diacylglycerol kinase OS=Gallus gall	DGKZ	102.56
A0A1L1RJ69	Heparan sulfate proteoglycan 2(predi	HSPG2	395.04
F1NR99	Coproporphyrinogen oxidase OS=Ga	CPOX	39.152
F6R228	Putative transferase CAF17(predicted	IBA57	36.128
A0A1D5PEJ1	CBF domain-containing protein OS=(	CEBPZ	122.48
Q5ZJB5	Multiple coagulation factor deficiency	MCFD2	16.037
E1C396	Hepatocyte growth factor-regulated ty	HGS	84.367
A0A1D5PHS2	IF rod domain-containing protein OS=	KRT18	46.849
A0A3Q3AXB0	EF_TS domain-containing protein OS	TSFM	39.673
A0A1D5PCR1	Huntingtin interacting protein 1 relate	HIP1R	110.26
A0A1D5NZU4	Protein kinase domain-containing pro	CDC2L1	89.925
Q5ZJ65	Protein FAM76A OS=Gallus gallus (	FAM76A	34.92
Q5ZL34	Cleavage and polyadenylation specifi	CPSF6	59.359
F1P360	Cytoskeleton associated protein 4(pre	CKAP4	62.443
A0A1D5PQD8	Neurabin-1-like(predicted) OS=Gallu	PPP1R9B	151.44
F1N876	Selenide, water dikinase OS=Gallus g	SEPHS2L	42.922
A0A3Q2U1V0	Ankyrin 1(predicted) OS=Gallus gallu	ANK1	210.43
A0A3Q2UB39	GTP binding protein 10(predicted) OS	GTPBP10	41.652
R4GJM5	CBM20 domain-containing protein O	STBD1	31.967
R4GLI2	GMP reductase OS=Gallus gallus OX	GMPR	37.269
E1BRJ8	DPH-type MB domain-containing pro	DPH3P1	9.5605

A0A1D5P797	Spectrin beta chain OS=Gallus gallus	SPTBN1	272.38
A0A1L1RX69	Transmembrane protein 50B(predicte	TMEM50B	17.909
A0A1D5PG45	DnaJ heat shock protein family (Hsp4	DNAJA3	52.392
A0A3Q2TZD8	UV excision repair protein RAD23 O	RAD23B	40.975
A0A1D5PXV5	Transcription factor(predicted) OS=G	SP1	77.568
A0A1L1RU16	AAA domain-containing protein OS=	PEX6	58.332
E1C6R4	Peptidylprolyl isomerase OS=Gallus g	FKBP8	44.326
A0A1D5P3K4	NADH dehydrogenase [ubiquinone] 1	NDUFA8	20.125
A0A1D5PYN7	GCFC domain-containing protein OS	PAXBP1	103.34
E1BV20	PHD finger protein 5A(predicted) OS	PHF5A	12.405
F1P2G2	Epidermal growth factor receptor patl	EPS15L1	101.37
Q6IT44	Interleukin-18 OS=Gallus gallus OX=	IL18	22.688
F1NUW0	HIG1 domain-containing protein OS=	HIGD2A	10.094
R4GL74	MCF.2 cell line derived transforming	MCF2L	142.71
A0A1D5PF81	Regulator of microtubule dynamics 1	RMDN1	34.457
A0A1D5P8T3	Zinc-hook domain-containing protein	RAD50	142.69
A0A3Q2U403	PGAM family member 5, mitochondr	PGAM5	37.972
A0A1L1RSP5	DAZ associated protein 1(predicted) (	DAZAP1	40.699
E1C4C7	Replication protein A3(predicted) OS	RPA3	13.468
A0A1D5P0Y5	TPR_REGION domain-containing pr	--	60.558
P08110	Endoplasmin OS=Gallus gallus OX=9	HSP90B1	91.554
A0A3Q2U1C1	YLP motif containing 1(predicted) OS	YLPM1	241.45
E1BRT3	USP domain-containing protein OS=(	USP40	144.55
P83038	Histone deacetylase 4 OS=Gallus gall	HDAC4	119.47
R4GI14	CARD domain-containing protein OS	BCL10	27.126
Q5ZJA1	PCI domain-containing protein OS=G	COPS7A	30.404
F1ND14	CUGBP Elav-like family member 1 (	CELF1	51.668
F1NJH4	BRCA1-associated protein(predicted)	BRAP	63.443
A0A1D5PUU0	Receptor protein-tyrosine kinase OS=	MERTK	104.78
E1C5L3	Poly(A)-specific ribonuclease OS=Ga	CNOT6L	68.729
E1C7T7	Mitochondrial GTPase 1 OS=Gallus g	MTG1	36.317
A0A3Q2UA96	T-complex protein 1 subunit gamma (	CCT3	68.699
F1NQ96	Non-specific serine/threonine protein	TP53RK	48.003

A0A1D5PJM1	Phospholipase A2 group XIIA OS=G	--	21.374
F1NGB1	Nucleobindin 2(predicted) OS=Gallus	NUCB2	53.837
F1NIP3	Kinesin-like protein OS=Gallus gallu	KIF3A	80.733
A0A3Q2U6B2	E3 ubiquitin/ISG15 ligase TRIM25, t	LOC416147	46.165
F1NCI5	Heterogeneous nuclear ribonucleopro	RBMXL3	30.799
F6QYT5	Plus3 domain-containing protein OS=	RTF1	78.596
A0A1D5PM21	Serine/arginine repetitive matrix prot	SRRM1	98.932
A0A3Q2U3K4	Ig-like domain-containing protein OS	--	10.578
Q5ZLP8	Insulin-like growth factor 2 mRNA-b	IGF2BP3	64.372
E1BSJ2	40S ribosomal protein S21 OS=Gallu	RPS21	9.1183
A0A1D5PBC3	Adseverin OS=Gallus gallus OX=903	SCIN	78.852
A0A3Q3A8P1	Iron-sulfur cluster assembly 2 OS=Ga	--	18.339
A0A3Q2U0E4	Ig-like domain-containing protein OS	--	11.018
A0A1L1RMW4	Zinc finger protein 740(predicted) OS	ZNF740	37.14
A0A1D5PD83	p95-APP2(predicted) OS=Gallus gall	GIT2	84.518
Q5ZLL9	Breast cancer metastasis-suppressor 1	BRMS1L	37.766
A0A1D5P2B7	Nuclear factor NF-kappa-B p100 subu	NFKB2	99.17
E1BQR6	LIM and cysteine rich domains 1(pred	LMCD1	40.072
A0A1D5PD17	HEAT repeat containing 5B(predicted	HEATR5B	214.59
A0A1D5NYK3	Glutaryl-CoA dehydrogenase(predicte	GCDH	46.758
P50141	GTP cyclohydrolase 1 OS=Gallus gal	GCH1	26.115
F1P2L0	CBFD_NFYB_HMF domain-contain	POLE3	16.359
A0A3Q2UIT2	Cytosolic Fe-S cluster assembly facto	NUBP2	28.872
A0A1D5PB54	Golgin B1(predicted) OS=Gallus gall	GOLGB1	349.49
Q5ZIZ5	Non-POU domain containing, octame	NONO	54.067
A0A1D5P6H2	t-SNARE coiled-coil homology doma	SNAP29	28.102
A0A1D5PXL6	Rap-GAP domain-containing protein	--	26.295
F6T197	Voltage-dependent anion-selective ch	VDAC2	31.496
E1BXC2	S-formylglutathione hydrolase OS=G	ESD	31.559
A0A3Q3AG64	RNA helicase OS=Gallus gallus OX=	LOC423693	77.671
F1N9S5	PALP domain-containing protein OS=	SRR	25.123
E1C626	TNF receptor-associated factor OS=C	TRAF6	61.914
A0A1D5PZM0	SH3 domain-containing protein(predi	--	22.529

F1NLX8	Aryl hydrocarbon receptor OS=Gallus	AHR	96.28
E1C947	Golgin subfamily A member 5 OS=G	GOLGA5	82.892
A0A1D5PXN3	Ectonucleotide pyrophosphatase/phos	ENPP3	100.79
Q5ZIY4	Ribosome maturation protein SBDS (	SBDS	28.823
A0A1D5PWG6	Neurofibromin OS=Gallus gallus OX	NF1	317.19
A0A3Q2U4U0	Macoilin(predicted) OS=Gallus gallu	TMEM57	73.864
A0A1D5PNH0	PKS_ER domain-containing protein (	MECR	48.766
A0A1D5PBN9	Purine rich element binding protein A	PURA	34.388
E1C6A8	Pre-mRNA-splicing factor 38A OS=(	PRPF38A	37.46
F1NKY6	Copine 1(predicted) OS=Gallus gallu	CPNE1	59.721
A0A3Q2UAL0	Sperm associated antigen 1(predicted	SPAG1	65.035
A0A3Q2U6Y3	Tumor protein p53 binding protein 1(	TP53BP1	202.48
E1BX57	Ubiquitin like 7(predicted) OS=Gallu	UBL7	40.293
Q5ZIJ9	E3 ubiquitin-protein ligase MIB2 OS=	MIB2	106.34
P20136	Glutathione S-transferase 2 OS=Gallu	GSTM2	25.893
A0A3Q2TSA1	WD_REPEATS_REGION domain-co	FBXW9	32.278
Q9I9D1	Voltage-dependent anion-selective ch	VDAC2	30.197
A0A1D5PZY7	WD_REPEATS_REGION domain-co	--	45.487
A0A3Q2U8F1	Rho GTPase activating protein 17(pre	ARHGAP17	91.216
O42479	Ferrochelatase, mitochondrial OS=Ga	FECH	45.429
E1BR36	Actin like 6A(predicted) OS=Gallus g	ACTL6A	47.484
Q90635	Dihydropyrimidinase-related protein 2	DPYSL2	62.33
E1BZQ4	Acyl-CoA thioesterase 11(predicted)	ACOT11	62.619
A0A3Q2TSW2	Ubiquitin-like domain-containing pro	UBTD2	24.273
A0A1D5PYU0	Dynamin-type G domain-containing p	MFN1	83.97
A0A1D5NUM3	Mth938 domain-containing protein(pr	AAMDC	13.123
Q5ZLF7	Importin subunit alpha OS=Gallus ga	KPNA4	57.854
Q5ZID5	Ubiquitin carboxyl-terminal hydrolase	USP28	118.75
Q5ZJ56	60S ribosomal protein L7 OS=Gallus	RPL7	28.78
F1NSZ4	CYTOSOL_AP domain-containing pr	NPEPL1	56.089
E1C9D0	DAO domain-containing protein OS=	DDO	37.493
F1NQT4	SSD domain-containing protein OS=(	NPC1	143.16
A0A3Q2UDU0	KIAA0930(predicted) OS=Gallus gal	KIAA0930	41.416

F1ND19	AMP-binding domain-containing pro	ACSL4	74.505
C7G537	ADF actin binding protein OS=Gallus	COTL1	16.106
A0A1D5PZ51	Amidohydro-rel domain-containing p	DPYSL3	70.714
A0A3Q2TRY3	Complement factor H (Fragment)(pre	CFH	147.86
E1BTT7	Gal_mutarotas_2 domain-containing	GANC	104.83
A0A1D5PL07	CUE domain-containing protein OS=	AUP1	44.618
F1NBT2	Exonuclease domain-containing prote	ERI3	27.539
A0A1D5PXT0	UBC core domain-containing protein	UBE2M	20.947
A0A1D5PWK4	Leucine-rich repeat-containing protei	LRRC59	40.455
E1C6C9	Complex I-PDSW OS=Gallus gallus	NDUFB10	20.497
A0A3Q2UEW9	CCHC-type domain-containing protei	LOC112531364	54.8
P48463	Serine/threonine-protein phosphatase	PPP2CA	35.563
A0A1D5PZV2	RBR-type E3 ubiquitin transferase OS	ANKIB1	119.57
A0A3Q2UI36	Mx protein (Fragment)(predicted) OS	MX1	76.453
Q5ZJL3	Proteasome inhibitor PI31 subunit OS	PSMF1	29.142
F1NC02	Proteasome subunit alpha type OS=G	PSMA4	29.497
A0A1D5NVV7	Syntabulin(predicted) OS=Gallus gall	SYBU	72.461
Q1KNA7	GHRH/PACAP-like OS=Gallus gallu	GHRH	19.527
F1P1G7	HIV-1 Tat specific factor 1(predicted)	HTATSF1	51.078
A0A3Q3AK56	Trichoplein keratin filament-binding	TCHP	43.535
P30352	Serine/arginine-rich splicing factor 2	SRSF2	25.524
Q5ZKC1	Eukaryotic translation initiation facto	EIF2A	64.503
E1BU01	Non-specific serine/threonine protein	CDC42BPB	194.88
A0A0B5H7I8	Tripartite motif containing 25 OS=Ga	TRIM25	71.424
Q5ZK01	Protein pelota homolog OS=Gallus ga	PELO	43.507
P81475	Complement factor B-like protease (F	--	24.275
A0A3Q2UPE8	15 kDa phosphoprotein enriched in as	LOC107049501	14.955
A0A1D5PB11	Tight junction protein 1(predicted) OS	TJP1	189.49
A0A1D5PER4	Crystallin beta-gamma domain contai	AIM1L	164.52
F1N8K3	Arf-GAP domain-containing protein (	ARFGAP1	61.264
F1NL59	Caspase 9(predicted) OS=Gallus gallu	CASP9	46.861
F2Z4K7	40S ribosomal protein S3a OS=Gallu	RPS3A	29.867
F1P3P3	Rho GDP dissociation inhibitor alpha	ARHGDIA	23.262

E1BQJ6	Kinesin motor domain-containing prc	CENPE	257.85
H9L3K3	Adenosine deaminase RNA specific(p	ADAR	121.77
A0A1D5PQG2	DHHA2 domain-containing protein C	PRUNE1	55.274
A0A1L1RR51	Kinesin light chain OS=Gallus gallus	KLC1	72.041
F1NHV6	YY1 transcription factor(predicted) C	YY2	45.181
A0A1L1RWM3	SH3 domain-binding glutamic acid-ri	SH3BGRL3	10.463
F1NQJ6	Ubiquitin carboxyl-terminal hydrolase	USP3	59.063
Q5ZJV0	COP9 signalosome complex subunit 4	COPS4	46.785
Q5F352	GTPase NRas OS=Gallus gallus OX=	NRAS	21.278
A0A3Q2U7V6	TGF_BETA_2 domain-containing pro	LOC107049250	38.163
E1C027	Nuclear transcription factor, X-box bi	NFXL1	98.753
A0A3Q2UAE7	Ig-like domain-containing protein OS	--	10.941
P50890	40S ribosomal protein SA OS=Gallus	RPSA	33.021
E1BXS9	eIF-2B GDP-GTP exchange factor su	EIF2B5	81.18
F1NKI9	Circadian locomoter output cycles pro	CLOCK	90.045
F1N8D3	FHA domain-containing protein OS=	SLMAP	92.623
F1N8E6	MPN domain-containing protein OS=	EMC8	22.853
A0A1D5PTU5	Acyl-peptide hydrolase OS=Gallus ga	APEH	81.856
A0A1D5PW57	SAM domain-containing protein OS=	SAMD4A	77.934
H9L022	Scaffold attachment factor B1-like isc	SAFB	103.74
A0A3Q2U2U5	B30.2/SPRY domain-containing prote	LOC107049156	42.915
A0A3Q2UIV9	Anaphase-promoting complex subuni	ANAPC1	195.42
A0A1D5NVM2	NADH dehydrogenase [ubiquinone] 1	NDUFA2	11.071
A0A1D5PPT0	Proteasomal ubiquitin receptor ADRM	ADRM1	39.349
A0A1D5P7L6	Septin OS=Gallus gallus OX=9031 G	SEPTIN8	49.7
Q5ZHZ9	RNA binding motif protein 12B(pred	RBM12B	78.817
Q5ZMT0	14-3-3 protein epsilon OS=Gallus gal	YWHAE	29.174
A0A3Q2UGH8	Cleavage stimulation factor 50 kDa su	CSTF1	48.357
F1NUY6	N-terminal kinase-like-binding protei	GORAB	42.011
E1BY93	Joining chain of multimeric IgA and I	JCHAIN	17.946
A0A1D5P9E1	COMM domain-containing protein O	COMMD9	21.816
Q5F387	Matrin-type domain-containing protei	SF3A3	58.888
A0A3Q2UMT0	Target of Myb protein 1 OS=Gallus g	TOM1	54.725

F1P3F5	Protein numb homolog OS=Gallus ga	NUMB	63.242
Q49B65	EF hand-containing protein 1 OS=Ga	EFHD1	26.926
A0A1D5PGR2	TLE family member 1, transcriptiona	TLE1	74.159
A0A1D5P7U7	Splicing factor 3B subunit 5 OS=Gall	SF3B5	10.094
A0A1L1RLA6	LRRcap domain-containing protein C	PPP1R7	40.611
Q5F4B1	Glycerol-3-phosphate phosphatase OS	PGP	32.995
Q5ZLX2	Eukaryotic translation initiation facto	EIF2S1	36.178
A0A1D5PGT3	Brain-specific angiogenesis inhibitor	BAIAP2	54.377
A0A1D5PUQ0	Rho GTPase activating protein 25(pre	ARHGAP25	71.847
F1NGM7	Protein CASP OS=Gallus gallus OX=	CUX1	77.43
F1NMI2	DnaJ heat shock protein family (Hsp4	DNAJC6	99.526
F1P0L5	RRM domain-containing protein OS=	TRMT2A	66.976
Q5ZLS0	RRM domain-containing protein OS=	CSTF2	49.891
A0A3Q2U6D5	NifU_N domain-containing protein C	ISCU	16.672
E1BS37	Hydroxysteroid 17-beta dehydrogenas	HSD17B11	32.639
A0A3Q2U6G0	GrpE like 2, mitochondrial(predicted)	GRPEL2	16.965
Q90XD2	Agmatinase, mitochondrial OS=Gallu	AGMAT	36.488
A0A1D5PS91	Phospholipase A2 OS=Gallus gallus (	PLA2G4A	80.806
Q5ZMQ0	Armadillo repeat-containing protein 1	ARMC1	31.063
A0A1D5P307	Integrin_alpha2 domain-containing pr	ITGA3	113.76
A0A3Q2UPE2	ADF-H domain-containing protein O	LOC107056280	37.73
A0A3Q2UAE2	Poly [ADP-ribose] polymerase OS=G	PARP3	57.092
O93510	Gelsolin OS=Gallus gallus OX=9031	GSN	85.831
E1BZM3	VWFA domain-containing protein OS	INTS6L	100.84
E1BXJ6	Sedoheptulokinase (Fragment)(predic	SHPK	50.859
E1BYW9	Proteasome subunit beta OS=Gallus g	PSMB3	23.085
A0A1D5NU78	N-acetyl-alpha-glucosaminidase(pred	NAGLU	83.915
E1BYN7	Voltage-dependent anion-selective ch	VDAC1	30.706
A0A1D5P735	Matrin-type domain-containing protei	CIZ1	67.316
Q766Y2	LIM and SH3 protein OS=Gallus gall	lasp-2	31.435
A0A3Q2U743	A2M_recep domain-containing protei	--	61.085
Q5F477	Lipid droplet-associated hydrolase OS	LDAH	36.882
F1NK96	Protein disulfide-isomerase A6 OS=C	PDIA6	48.787

A0A1D5NWM4	Receptor protein-tyrosine kinase OS=	ERBB2	132.21
E1BT75	Exocyst complex component 7 OS=G	EXOC7	75.225
F1P0Y6	Malic enzyme OS=Gallus gallus OX=	ME1	63.911
F1NAB7	Complement subcomponent C1r OS=	C1R	80.506
P28497	F-actin-capping protein subunit alpha	CAPZA2	32.844
A0A1D5PIX3	Non-specific serine/threonine protein	STK3	56.214
A0A3Q2UAA1	J domain-containing protein OS=Gall	LOC107049075	37.995
A0A3Q2TVQ2	Charged multivesicular body protein	CHMP1B	20.74
F1N9B4	Protein aidB (Fragment)(predicted) O	LOC417013	70.421
E1C9F4	O-phosphoserine phosphohydrolase (	PSPH	25.143
A0A1D5P5M6	ES1 protein-like protein, mitochondri	GATD3AL1	26.794
F1NE00	Kinesin-like protein OS=Gallus gallu	KIF5C	109.46
F1NWL9	Zinc finger CCCH-type antiviral prot	ZC3HAV1	81.575
A0A1D5PUB7	PlsC domain-containing protein OS=(	AGPAT3	43.582
E1BXL5	Aminotran_1_2 domain-containing pr	CCBL1	47.887
A0A3Q3AF25	Branched-chain-amino-acid aminotra	BCAT1	41.727
A0A1D5PEI0	DNA-directed RNA polymerase subu	POLR2I	14.865
Q5ZM44	Calcineurin B homologous protein 1 (	CHP1	22.447
A0A1D5PW26	TruB_N domain-containing protein C	TRUB1	30.816
A0A3Q2U0S7	RNA helicase OS=Gallus gallus OX=	DDX3X	70.556
Q1T7C1	Centromere protein K OS=Gallus gal	CENPK	30.72
F1NW80	Golgi SNAP receptor complex memb	GOSR1	27.719
Q5ZKK2	Integrator complex subunit 9 OS=Gal	INTS9	73.762
A0A1L1RUW5	N-myc and STAT interactor(predicte	NMI	42.97
A0A3Q2TWN1	Acid sphingomyelinase-like phospho	SMPDL3A	53.569
F1NFF7	Cytochrome P450 2K1 (Fragment)(pr	CYP2AC1	56.143
Q5ZLG0	Acetoacetyl-CoA synthetase OS=Gall	AACS	74.369
A0A1D5PHJ9	1,4-alpha-glucan branching enzyme (	GBE1	79.501
F1NIE8	RanGAP1_C domain-containing prot	RANGAP1	62.946
A0A1D5P6R9	Ribosome biogenesis regulatory prote	RRS1	39.784
Q5ZL80	Heterogeneous nuclear ribonucleopro	HNRNPM	76.022
P51901	Annexin A6 OS=Gallus gallus OX=9	ANXA6	75.218
E1BRV2	Mitochondrial ribosomal protein L20	MRPL20	17.648

A0A1D5PN92	Discs large MAGUK scaffold protein	DLG3	91.073
A0A1D5PVM3	Upstream binding protein 1(predicted	UBP1	56.444
Q04861	Nuclear factor NF-kappa-B p105 subu	NFKB1	107.99
F1P573	Vitamin-K-epoxide reductase (warfar	VKORC1L1	19.765
E1C8N0	Regulator of microtubule dynamics pr	RMDN2	47.835
A0A1D5PCE2	Peptidase_M28 domain-containing pr	NAALADL2	81.471
A0A1D5PFL2	Mitoguardin 2(predicted) OS=Gallus	MIGA2	66.168
F1N849	ASD1 domain-containing protein(pre	--	143.11
A0A1D5P4L6	Inter-alpha-trypsin inhibitor heavy ch	ITIH3	71.557
E1BSM9	CAP10 domain-containing protein OS	KDELC1	57.894
O57476	Hsp90 co-chaperone Cdc37 OS=Gallu	CDC37	45.608
E1C591	PPIase cyclophilin-type domain-conta	PPIG	88.35
P27042	Lysozyme g OS=Gallus gallus OX=90	--	23.238
E1C7S2	SCP2 domain-containing protein OS=	HSDL2	44.234
Q90584	Collagen alpha-1(XVII) chain OS=Ga	COL17A1	151.2
A0A1D5P8V8	Vesicle-fusing ATPase OS=Gallus ga	VPS4B	44.518
Q5ZI08	Transcription elongation factor SPT5	SUPT5H	119.98
A0A1L1RRM0	Vesicle trafficking 1(predicted) OS=G	VTA1	42.897
A0A1D5NZS4	Glyoxylate reductase 1 homolog OS=	GLYR1	60.502
A0A3Q2UCV3	TOM1-like protein 2(predicted) OS=G	TOM1L2	57.986
F1NPQ2	Multiple inositol polyphosphate phos	MINPP1	50.199
Q5ZKV4	Cytosolic Fe-S cluster assembly facto	NUBP2	29.358
A0A1L1RQP3	Glyoxylate reductase/hydroxypyruvat	LOC100858664	35.775
A0A3Q2U9L6	E3 ubiquitin-protein ligase parkin OS	PARK2	52.483
H9KYX6	Methanethiol oxidase OS=Gallus gall	SELENBP1	52.446
E1BW99	tRNA (adenine(58)-N(1))-methyltrans	TRMT61B	47.642
F1N9W3	Glutamyl-tRNA(Gln) amidotransferas	GATB	60.159
F1NU56	40S ribosomal protein S25 OS=Gallu	RPS25	13.712
F1P3Y0	Multivesicular body subunit 12B(prec	MVB12B	31.805
Q5F420	AMP-binding domain-containing pro	ACSL1	78.561
A0A1D5P276	Receptor-interacting serine/threonine-	RIPK2	63.975
F1NJM1	NOL1/NOP2/Sun domain family mem	NSUN2	90.579
A0A3Q2UB58	La ribonucleoprotein domain family r	LARP7	67.899

F1P331	Aldo_ket_red domain-containing prot	AKR7A2	36.624
Q5ZLN0	Leucine-rich repeat-containing protei	LRRC40	67.475
A0A3Q2TUF8	VWFA domain-containing protein OS	CACNA2D1	122.36
P18942	NADH-ubiquinone oxidoreductase ch	MT-ND4L	10.727
Q5F3Z3	Ubiquitin-conjugating enzyme E2 var	UBE2V2	16.305
E1BVD1	Aldo_ket_red domain-containing prot	LOC418170	35.663
A0A1D5NTT1	5-demethoxyubiquinone hydroxylase,	COQ7	24.78
F1NAM7	3-hydroxybutyrate dehydrogenase 2(p	BDH2	26.864
F1P4F1	SH3 domain binding protein 4(predic	SH3BP4	108.27
Q5ZLQ6	14-3-3 protein beta/alpha OS=Gallus	YWHAB	27.907
A0A1D5PZ78	Rab-GAP TBC domain-containing pr	TBC1D13	46.484
A0A1L1RQM2	Enolase-phosphatase E1 OS=Gallus g	ENOPH1	33.897
E1BW94	RRM domain-containing protein OS=	SF3B6	10.618
A0A1D5P9D7	SWI/SNF related, matrix associated, a	SMARCC2	129.93
A0A3Q2TWU6	Signal transducing adaptor molecule(	STAM	46.948
Q5ZJ32	SOSS complex subunit C OS=Gallus	INIP	11.292
A0A3Q2U072	SCO cytochrome c oxidase assembly	SCO2	28.135
A0A3Q2U3Q5	Methionine adenosyltransferase OS=G	MAT2A	20.501
F1NFB8	E3 ubiquitin-protein ligase OS=Gallu	DTX3L	83.322
E1BXS8	Carboxymethylenebutenolidase homo	CMBL	28.17
Q9DEA3	Proliferating cell nuclear antigen OS=	PCNA	28.888
E1C4N7	Sorting nexin OS=Gallus gallus OX=	--	66.607
A0A1D5NW71	Host cell factor C2(predicted) OS=Ga	HCFC2	87.971
A0A3Q2TSE8	A2M_recep domain-containing protei	--	29.62
F1NNH9	TPR_REGION domain-containing pr	TOMM70	65.074
A0A3Q2UF40	Ubiquitin-like modifier-activating enz	LOC107051050	39.945
A0A1L1RRY6	THUMP domain-containing protein O	THUMPD3	54.878
A0A3Q3AGK3	Ig-like domain-containing protein OS	--	10.261
A0A1D5PM61	Metastasis associated 1 family memb	MTA2	60.883
E1BR24	Pyr_redox_2 domain-containing prote	AIFM2	40.619
A0A3Q2U8K0	Alpha-2-macroglobulin-like protein 1	--	33.165
F1NUP9	Short chain dehydrogenase/reductase	SDR16C5	33.614
F1P174	Peptidase_M3 domain-containing pro	MIPEP	89.545

Q5F3D1	Zinc finger CCHC domain-containing	ZCCHC8	68.467
A0A1D5PYB9	Peptidylprolyl isomerase OS=Gallus g	FKBP15	126.48
A0A1L1RVM1	t-SNARE coiled-coil homology doma	STX8	26.569
Q5ZMM1	Ribosomal oxygenase 1 OS=Gallus g	RIOX1	65.684
A0A3Q2U0D1	Uncharacterized protein OS=Gallus g	DCAF11	9.4779
Q5ZIF6	U1-type domain-containing protein O	ZMAT2	23.613
A0A1D5PZ01	LsmAD domain-containing protein O	ATXN2L	114.26
A0A1D5PD65	Phosphatidylinositol-4,5-bisphosphat	TMEM55A	27.938
Q5ZJ43	Exocyst complex component 8 OS=G	EXOC8	80.429
A0A1D5P4X2	Conserved oligomeric Golgi complex	COG5	104.37
A0A1D5PZ75	Sulfite oxidase OS=Gallus gallus OX	--	50.284
F1NT21	2-deoxy-D-ribose 5-phosphate aldola	DERA	34.794
A0A1D5P5D8	Endoplasmic reticulum oxidoreducta	ERO1B	51.049
F1NHH2	LLGL scribble cell polarity complex	LLGL1	134.72
R4GJB9	Protein-tyrosine-phosphatase OS=Ga	PTPRJ	153.84
A0A1D5PV36	Tetratricopeptide repeat protein 38 O	TTC38	52.415
F1NJI6	F-box only protein 6 (Fragment)(pred	FBXO44	30.659
A0A1D5NTA0	Ubiquitin thioesterase OS=Gallus gal	OTUB1	31.176
F1NE88	Ubiquitin carboxyl-terminal hydrolas	USP4	107.45
F1NNI8	39S ribosomal protein L35, mitochon	MRPL35	20.057
A0A1D5Q032	Adenylyl cyclase-associated protein	CAP1	51.079
O13156	GDNF family receptor alpha-1 OS=G	GFRA1	52.043
A0A3Q3ATN2	Nicotinamide riboside kinase 1(predi	NMRK1	23.908
F1P5J5	Alanine--tRNA ligase OS=Gallus gal	AARS	106.97
E1C4T3	PATJ crumbs cell polarity complex c	PATJ	198.27
A0A1D5NXD4	Histone domain-containing protein O	NFYC	36.489
F1P5I8	Acyl-CoA binding domain containing	ACBD3	59.568
R4GF53	G_PROTEIN_RECEP_F1_2 domain-	GRPR	43.633
Q5ZLE8	RNA helicase OS=Gallus gallus OX=	DHX38	141.04
A0A1L1S0X6	DUF1681 domain-containing protein	NECAP2	28.877
F1NWN8	5-formyltetrahydrofolate cyclo-ligase	MTHFS	22.498
A0A3Q3AJ38	Mitochondrial calcium uptake 2(pred	MICU2	47.995
Q5ZMD6	Histone H2A.Z OS=Gallus gallus OX	H2AFZ	13.583

R4GK63	GB1/RHD3-type G domain-containin	GBP	68.07
E1C003	Choline dehydrogenase OS=Gallus ga	CHDH	79.966
R4GL80	Coenzyme A synthase OS=Gallus gal	--	56.303
F1NMM3	Protein archease OS=Gallus gallus O	ZBTB8OS	19.104
A0A1D5PM49	Chondroitinsulfatase OS=Gallus gallu	--	57.283
A0A3Q2U6I0	Vacuolar protein sorting 13 homolog	VPS13C	399.05
A0A1D5P7B5	Mitochondrial ribosomal protein L3(p	MRPL3	38.615
A0A3Q2U2F9	Plectin(predicted) OS=Gallus gallus (	--	54.39
F1N9A3	ADP ribosylation factor like GTPase	ARL3	20.409
A0A3Q2UED4	MIT domain-containing protein OS=(	SPG20	63.648
Q66VY4	Splicing factor 3a subunit 2 OS=Gallu	SF3A2	35.138
A0A1D5PVF8	Tartrate-resistant acid phosphatase ty	ACP5	34.96
A0A3Q2TUC5	Glycine hydroxymethyltransferase OS	--	20.691
A0A3Q2U8J3	Dipeptidyl peptidase 9(predicted) OS	DPP9	106.47
Q98TH5	40S ribosomal protein S11 OS=Gallu	cRPS11	18.346
A0A3Q2U257	CHDCT2 domain-containing protein	--	26.359
A0A3Q2UFQ7	Coiled-coil domain containing 186(pr	CCDC186	99.477
A0A1D5PVS3	Translocase of outer mitochondrial m	TOMM6	7.896
A0A1D5P6P3	Thioredoxin-disulfide reductase OS=(	TXNRD1	54.844
F1NSY0	Serine/threonine-protein kinase RIO3	RIOK3	58.528
F1NCX2	Ubiquitin-like domain-containing pro	UBFD1	30.65
O93567	Zinc finger and BTB domain-containi	ZBTB7A	59.841
Q8AWB4	GDP-fucose protein O-fucosyltransfe	POFUT1	43.315
F1NY50	COMM domain-containing protein O	COMMD6	22.649
A0A1D5PBZ7	alpha-1,2-Mannosidase OS=Gallus ga	MAN1B1	73.961
E1C1D1	Annexin OS=Gallus gallus OX=9031	ANXA7	49.333
F1NJX8	Farnesyltransferase, CAAX box, alph	FNTA	42.011
F1NA74	Pro-apoptotic WT1 regulator(predicte	PAWR	36.061
Q5ZLB3	Helix-destabilizing protein OS=Gallu	HNRNPA1	38.166
A0A1L1RSX2	Tensin 3(predicted) OS=Gallus gallus	TNS3	158.63
E1C6C0	Mediator of ErbB2-driven cell motilit	MEMO1	31.81
A0A3Q2UI69	Sema domain-containing protein OS=	SEMA4G	89.138
A0A1D5P7M2	Retinol saturase(predicted) OS=Gallu	RETSAT	67.66

A0A1D5PTL4	Tripartite motif containing 3(predicte	TRIM3	81.298
A0A1D5PQU7	Protein kinase domain-containing pro	CAMKK2	57.104
A0A3Q3AL01	Exosome component 1(predicted) OS	EXOSC1	18.493
A0A1D5PUQ7	ATP-dependent 6-phosphofructokina	PFKM	81.719
P87362	Bleomycin hydrolase OS=Gallus gall	BLMH	52.69
A0A1D5PWD8	Dihydrolipoamide acetyltransferase c	DBT	54.097
F1NE49	Regulator of telomere elongation heli	RTEL1	148.24
F1P2A7	Exocyst complex component 2 OS=G	EXOC2	103.96
A0A1D5P1C6	Phospholipase B-like OS=Gallus gall	PLBD1	62.052
F1NM37	WD_REPEATS_REGION domain-co	TBL3	90.49
A0A1D5NU82	Tubulin gamma chain OS=Gallus gal	TUBG1	50.331
A0A1D5PD25	EF-hand domain family member D2(	EFHD2	24.563
A0A1D5PRK3	Mitochondrial ribosomal protein L13	MRPL13	20.725
A0A1D5NXI3	K(+) channel subunit beta-1 OS=Gall	KCNAB2	40.93
A0A1D5PM19	Myosin-9 OS=Gallus gallus OX=903	MYH9	226.63
A0A3Q2U3S6	Protoporphyrinogen oxidase OS=Gall	--	55.664
A0A1D5PG13	Protein transport protein Sec61 subun	SEC61G	7.7412
F1P2P4	ATP-dependent (S)-NAD(P)H-hydrat	NAXD	36.525
Q4JQQ0	Caspase-18/a OS=Gallus gallus OX=	CASP18	52.914
F1NCS6	Formyltetrahydrofolate synthetase OS	MTHFD1L	106.89
A0A3Q2U7J1	39S ribosomal protein L22, mitochon	MRPL22	23.863
F1NCZ2	Rab GDP dissociation inhibitor OS=C	GDI1	70.788
F1NLC8	Nardilysin(predicted) OS=Gallus gall	NRDC	132.4
Q5ZMF6	TNF receptor associated protein 1(pre	TRAP1	79.676
R4GM79	DNA topoisomerase I OS=Gallus gal	TOP1	88.665
A0A3Q2UQ06	Attractin(predicted) OS=Gallus gallus	ATRN	160.53
E1C568	V-SNARE coiled-coil homology dom	STXBP6	23.657
R4GM10	Fructose-bisphosphate aldolase OS=C	ALDOC	39.36
A0A0A0MQ61	Glutathione transferase OS=Gallus ga	LOC100859645	25.222
A0A3Q3A989	WD_REPEATS_REGION domain-co	WDR20	64.993
A0A1D5P4B1	NADH dehydrogenase [ubiquinone] 1	NDUFA6	14.396
E1BY36	Acetylserotonin O-methyltransferase	ASMTL	70.216
Q5ZMC0	Endothelial differentiation-related fac	EDF1	16.363

F7BGT1	Nudix hydrolase domain-containing p	NUDT1	18.07
E1BSV1	Dimethylglycine dehydrogenase(pred	DMGDH	96.461
A0A1D5PLP9	Kinesin family member 21A(predicte	KIF21A	179.27
F1NZ07	FYVE-type domain-containing protei	ZFYVE21	26.286
A0A1L1RP17	SH3 domain-containing protein OS=(	MIA3	221.69
F1NNV6	ATPase, H+ transporting, lysosomal a	ATP6AP1	50.53
F1N9N4	Epididymal secretory protein E1 OS=	NPC2	16.206
A0A1D5PD50	Zinc finger MYND-type containing 8	ZMYND8	125.52
F1NAR5	SERPIN domain-containing protein C	SERPINF2	56.973
F1NNJ3	SNW domain-containing protein 1 OS	SNW1	61.872
O93480	Chromobox protein (CHCB1) OS=Ga	CBX1	21.538
E1C4L3	Sepiapterin reductase OS=Gallus gall	SPR	28.839
F1NLY0	5-methyltetrahydrofolate--homocyste	MTR	151.08
F1NGM2	Atypical kinase COQ8A, mitochondr	COQ8A	72.489
Q5ZIQ3	Heterogeneous nuclear ribonucleopro	HNRNPK	47.278
A0A1D5PPF7	Translin-associated protein X (Fragm	TSNAX	33.243
F1NEF2	CCT-theta OS=Gallus gallus OX=903	CCT8	59.487
F1NRZ4	RNA binding motif protein 15(predic	RBM15	102.13
F1NGW2	Thiopurine S-methyltransferase OS=(	TPMT	26.828
A0A1D5NZD6	Riboflavin kinase OS=Gallus gallus (	RFK	17.935
E1C1Y0	RNA 3'-terminal phosphate cyclase C	RTCA	39.223
A0A3Q2TV88	Ig-like domain-containing protein OS	--	10.237
Q5ZJ49	Alpha-MPP OS=Gallus gallus OX=9(	PMPCA	57.547
A0A3Q2TWE1	RNA helicase OS=Gallus gallus OX=	DHX30	131.92
P98157	Low-density lipoprotein receptor-rela	LRP1	507.13
A0A1D5P5R5	Four and a half LIM domains 3(predi	FHL3	32.309
A0A3Q2UG80	Methyl-CpG binding protein 2 OS=G	--	21.52
E1C254	mRNA-capping enzyme OS=Gallus g	RNGTT	62.539
A0A1D5PI47	Ribonucleotide reductase regulatory T	RRM2B	40.782
F1NPM6	NADH dehydrogenase [ubiquinone] 1	NDUFA10	41.449
A0A3Q2TX12	Glycerol-3-phosphate dehydrogenase	GPD1L2	38.462
Q5F3T9	UDP-glucose 6-dehydrogenase OS=G	UGDH	55.064
E1C541	Mitochondrial fission factor(predicted	MFF	26.412

A0A1D6UPT3	Cellular nucleic acid-binding protein	CNBP	18.844
F1NU77	Trafficking protein particle complex 8	TRAPPC8	162.62
A0A3Q2UEL4	Phosphatidylinositol 3-kinase catalyti	PIK3C3	101.66
A0A1L1RX26	Eukaryotic peptide chain release facto	ETF1	49.03
Q90828	Cytoplasmic dynein 1 light intermedi	DYNC1LI1	55.893
A0A1D5PV03	Microtubule actin crosslinking factor	MACF1	619.32
A0A3Q2UDL9	ER membrane protein complex subun	EMC2	34.884
E1C892	MRP-L46 domain-containing protein	MRPL46	32.839
Q9PV94	Small nuclear ribonucleoprotein-asso	SNRPB	24.588
A0A3Q2TSA7	PH domain-containing protein OS=G	PLEKHA6	147.7
F1NCA2	Glycerol-3-phosphate dehydrogenase	GPD2	80.751
A0A1D5PSE2	NADB-LER4(predicted) OS=Gallus g	LOC107049164	27.475
E1BQG2	Zinc finger protein 148(predicted) OS	ZNF148	86.336
A0A1D5Q028	Deubiquitinating enzyme CYLD OS=	LOC420294	89.728
F1NNH5	Teashirt zinc finger homeobox 2(pred	TSHZ2	115.08
F1P571	Interferon gamma receptor 1(predicte	IFNGR1	48.442
A0A1D5NZF5	BHLH domain-containing protein OS	USF3	240.99
E1BXL1	Methylenetetrahydrofolate reductase (	MTHFR	74.212
F1NBF1	Beta-mannosidase OS=Gallus gallus (	MANBA	100.87
E1BVZ7	Dynein light chain Tctex-type 3(predi	DYNLT3	13.077
F1P5T2	Phosphorylase kinase OS=Gallus gall	PHKG1	57.067
F1NVA3	Formin homology 2 domain containir	FHOD1	161.37
Q5ZJ23	Argininosuccinate synthase OS=Gallu	ASS1	46.903
F1NLP7	Antithrombin-III OS=Gallus gallus O	SERPINC1	51.709
A0A1D5NUM7	Rho guanine nucleotide exchange fac	ARHGEF7	88.136
A0A3Q2UHG1	Homeobox domain-containing protein	PKNOX1	45.636
Q5ZKI1	Chloride intracellular channel protein	CLIC2	28.134
A0A1D5NYR0	N-acetyltransferase domain-containin	NAA50	17.815
A0A1D5PD08	Pyridoxal 5'-phosphate synthase OS=	PNPO	23.828
A0A3Q2TWE8	PPFIA binding protein 1(predicted) C	PPFIBP1	107.38
A0A1D5PUG2	Sarcosine dehydrogenase OS=Gallus	--	50.732
Q804X6	Coagulation factor IX OS=Gallus gal	F9	51.804
A0A1D5PV32	GYF domain-containing protein OS=	GIGYF2	147.67

A0A1D5PUG6	RNA helicase OS=Gallus gallus OX=	DDX23	95.147
A0A3Q2U942	Acyl-coenzyme A thioesterase 5 (Fra	ACOT4	45.591
Q5ZHR4	Diadenosine tetraphosphate synthetas	GARS	77.58
A0A1D5P9K6	Acyl-CoA dehydrogenase short/branc	ACADSB	47.292
A0A452J864	F-actin-capping protein subunit beta (	CAPZB	29.277
F1NXP6	Caspase 8(predicted) OS=Gallus gallu	CASP8	54.557
A0A1D5NY78	SCD domain-containing protein OS=	STAG1	114.93
A0A1D5PP82	Tripartite motif containing 28(predict	LOC107050474	90.242
A0A1L1RWF6	Calponin OS=Gallus gallus OX=9031	CNN3	36.61
R4GKS3	CHCH domain-containing protein OS	COX19	10.591
F1NKW9	26S proteasome non-ATPase regulato	PSMD5	50.384
F1NK38	T-complex protein 1 subunit eta OS=	CCT7	59.458
A0A3Q2U328	CAP-Gly domain-containing protein (	TBCB	34.914
Q5ZMM8	C-terminal-binding protein 2(predicte	CTBPL	47.985
E1C5L8	Mitochondrial ribosomal protein L44(	MRPL44	36.056
Q5ZJW4	Vesicle-trafficking protein SEC22b O	SEC22B	24.717
E1C008	WD_REPEATS_REGION domain-co	WDR37	54.895
Q58I02	Nicotinamide phosphoribosyltransfera	PBEF1	55.586
F1NIY3	Programmed cell death protein 4 OS=	PDCD4	51.723
A0A1D5Q004	Carn_acyltransf domain-containing pr	CRAT	75.361
F1NPW9	SMK-1 domain-containing protein OS	PPP4R3A	94.005
A0A1D5NYP8	SHC-transforming protein 1 OS=Gall	SHC1	67.555
A0A1D5P7X7	Peptidase_M28 domain-containing pr	NAALADL2	86.805
A0A1D5P1D0	3'-phosphoadenosine-5'-phosphosulfa	PAPSS2	67.301
F1NPI8	Pyridine nucleotide-disulfide oxidore	PYROXD1	55.219
B8ZX71	Sixth complement component OS=Ga	C6	104.72
E1C8A6	Lys-63-specific deubiquitinase OS=G	BRCC3	29.812
A0A3Q3AG59	Putative C-mannosyltransferase DPY	DPY19L1	80.116
A0A1L1RPP7	WD repeat-containing protein 44 OS=	WDR44	95.067
A0A1D5PLZ2	SERPIN domain-containing protein (	SERPIND1	55.814
Q5F486	BAG family molecular chaperone reg	BAG5	51.167
E1BU99	CN hydrolase domain-containing prot	UPB1	42.716
E1C762	D-aminoacyl-tRNA deacylase 2 OS=(	DTD2	18.652

A0A1D5P6I3	Conserved oligomeric Golgi complex	COG1	101.87
F1NMN3	Lactamase beta(predicted) OS=Gallus	LACTB	57.977
A0A1D5PXH0	AP complex subunit beta OS=Gallus	AP1B1	104.8
A0A1D5PYQ3	Tr-type G domain-containing protein	GSPT2	67.658
A0A1D6UPU5	FAD-binding FR-type domain-contai	CYBB	65.401
E1C5I8	SAM_MT_RSMB_NOP domain-con	NSUN5	50.994
F1NW85	Asparaginase OS=Gallus gallus OX=	ASPG	65.275
A0A1D5P835	DUF1681 domain-containing protein	NECAP1	29.662
E1BV47	Protein BUD31 homolog OS=Gallus	BUD31	17.026
E1C7J0	Heme oxygenase OS=Gallus gallus C	HMOX2	35.366
A0A3Q3A8P5	Collagen beta(1-O)galactosyltransfera	LOC425607	65.456
F7B5S0	Importin subunit alpha OS=Gallus ga	KPNA6	60.06
A0A1D5PGW7	Sorcin(predicted) OS=Gallus gallus C	SRI	21.849
A0A3Q2U3D5	Spliceosome associated factor 3, U4/U	SART3	98.601
A0A1D5PXP9	Transforming growth factor-beta-indu	TGFBI	74.476
P09987	Histone H1 OS=Gallus gallus OX=90	--	21.881
F1NXZ7	FAA_hydrolase domain-containing pr	FAHD1	24.711
A0A1D5NTR9	Fam20C domain-containing protein C	FAM20C	63.086
A0A1D5NXS9	Alpha-actinin-4 OS=Gallus gallus OX	ACTN4	117.28
E1C8T5	Sm domain-containing protein OS=G	LSM6	9.1275
F1NKX6	KRAP_IP3R_bind domain-containing	SSFA2	139.54
Q8AYD0	Cadherin-5 OS=Gallus gallus OX=90	CDH5	86.781
F1NLV8	Inactive tyrosine-protein kinase 7 OS=	PTK7	115.53
A0A3Q2U530	MAP7 domain-containing protein 3 is	MAP7D3	94.608
Q5F3I0	Protein PRRC1 OS=Gallus gallus OX	PRRC1	45.943
E1BY44	TGc domain-containing protein OS=C	TGM2	78.677
E1C8H2	Testis expressed 264, ER-phagy recep	TEX264	35.957
F1N8Z0	Protein pelota homolog OS=Gallus ga	PELO	130.69
Q9W694	Homeodomain transcription factor BA	BARX2B	29.844
F1P1U3	Carn_acyltransf domain-containing pr	CPT2	73.907
R4GIZ6	TIGAR OS=Gallus gallus OX=9031 C	TIGAR	30.906
A0A1D5P0U6	Family with sequence similarity 160 r	FAM160B1	86.626
A0A1L1RNV8	MICOS complex subunit(predicted) C	CHCHD3	27.447

A0A1D5PYE6	LisH domain-containing protein OS=	DCAF1	149.76
Q5ZIL1	B30.2/SPRY domain-containing prot	ASH2L	67.071
E1BW77	CD2 associated protein(predicted) OS	CD2AP	65.22
A0A3Q2U1U6	RRM domain-containing protein OS=	--	51.854
A0A1D5P832	Poly(U) binding splicing factor 60(pr	PUF60	38.696
A0A3Q3AUJ9	S-adenosylmethionine synthase(predi	LOC107049542	28.863
A0A3Q2UNB9	Letm1 RBD domain-containing prote	LOC107049895	41.792
A0A3Q2U086	Reticulon OS=Gallus gallus OX=903	RTN4	22.307
A0A1D5NWW8	Mitogen-activated protein kinase 8 in	MAPK8IP3	145.15
A0A3Q2UFZ9	Guided entry of tail-anchored protein	ASNA1	36.834
E1C898	Ankyrin repeat and sterile alpha moti	ANKS1A	121.42
Q90687	Tyrosine-protein phosphatase non-rec	PTPN11	67.982
F1ND07	Proteasome activator complex subuni	PSME4	209.79
E1BUD0	NADB-LER2 OS=Gallus gallus OX=	LOC771069	27.551
F1NMA3	Sulfotransferase OS=Gallus gallus O	SULT1B	34.061
A0A1D5PAF0	NADB-LER4(predicted) OS=Gallus	LOC107080643	15.558
A0A1D5NVZ0	Mitochondrial ribosomal protein S12	MRPS12	14.7
A0A1D5PXQ1	Nucleoporin 153(predicted) OS=Gall	NUP153	151.03
F1NEF7	Myeloid protein 1(predicted) OS=Gal	LECT2	35.216
R4GLT4	Syntrophin beta 2(predicted) OS=Gal	SNTB2	68.465
F1NYZ1	S5 DRBM domain-containing protein	MRPS5	45.688
A0A1D5PI15	AH domain-containing protein OS=G	ARFIP1	46.662
P79795	Neuropilin-1 OS=Gallus gallus OX=	NRP1	102.48
F1NDY2	Sh3 domain-binding protein 1(predict	SH3BP1	64.266
A0A3Q3ADH6	Protein kinase domain-containing pro	NRBP2	52.434
A0A1D5PE45	Dehydrogenase/reductase SDR family	DHRS4	28.261
R4GJP5	Heparosan-N-sulfate-glucuronate 5-e	GLCE	64.233
A0A1D5P1Y9	V-type proton ATPase subunit a OS=	TCIRG1	92.765
A0A3Q3AE10	Golgi SNAP receptor complex memb	GOSR2	24.738
F1NLV6	Cobl domain-containing protein OS=	COBLL1	127.67
A0A1D5PPC4	5'-nucleotidase domain containing 1(	NT5DC1	47.894
A0A1D5PQG9	Diaphanous related formin 2(predicte	DIAPH2	90.805
Q90771	Cyclin-dependent kinase OS=Gallus	cdk6	36.85

E1C430	Ribosome-releasing factor 2, mitocho	GFM2	86.288
A0A1D5NUY2	Kinesin motor domain-containing pro	KIF11	119.8
F1N8Y3	20-hydroxysteroid dehydrogenase(pre	CBR3	30.326
F1P0A1	X-prolyl aminopeptidase 1(predicted)	XPNPEP1	70.014
F1NG19	Chromosome 1 open reading frame 5	--	19.537
A0A1D5P8F7	RAB12, member RAS oncogene fam	RAB12	27.791
A0A1D5NY89	UBX domain-containing protein OS=	FAF1	73.822
F1NAQ8	Glutamine amidotransferase OS=Gall	GMPS	83.543
A0A1D5NY12	RH2 domain-containing protein OS=	SPAG9	132.1
Q8UWC5	Nuclear protein matrin 3 OS=Gallus g	MATR3	100.71
A0A3Q2U364	Phosphatase and actin regulator 4 OS	--	16.656
A0A3Q2UJ64	Cytochrome b-c1 complex subunit 7	UQCRB	13.143
A0A1D5PGS8	Ubiquitin protein ligase E3 componen	UBR5	304.46
A0A1L1RXS0	MHD domain-containing protein OS=	--	26.218
E1C7T1	SERPIN domain-containing protein C	SPIA1	48.714
E1C2S1	Talin-1 OS=Gallus gallus OX=9031	TLN1	271.91
P30374	Ribonuclease homolog OS=Gallus ga	--	15.947
Q8JIS3	D-erythrulose reductase OS=Gallus g	DER	26.167
Q5ZM25	Obg-like ATPase 1 OS=Gallus gallus	OLA1	44.778
F1NH65	Sec1 family domain containing 1(pred	SCFD1	71.367
E1BT09	Rab3 GTPase-activating protein catal	RAB3GAP1	113.57
A0A1D5P251	Microtubule associated protein 1S(pr	MAP1S	147.2
F1NM59	RNA-binding protein 27(predicted) C	RBM27	113.07
A0A1D5P604	Sorbin and SH3 domain containing 2	SORBS2	71.82
F1P1X0	HTH La-type RNA-binding domain-c	LARP4B	81.219
F1NYQ3	Non-lysosomal glucosylceramidase C	GBA2	96.065
F1NCN1	Exo/endonuclease G(predicted) OS=	EXOG	43.159
E1C2P3	Heat shock 70 kDa protein 14 OS=Ga	HSPA14	54.112
Q5ZLW0	Lymphocyte cytosolic protein 1(predi	LCP1	69.748
Q5ZMB8	26S proteasome AAA-ATPase subun	PSMC2	48.661
A0A1D5NTN7	Phospholipase A2 activating protein(	PLAA	84.94
E1BZ74	AAA domain-containing protein OS=	AFG3L2	89.39
Q6PZ02	Cysteine protease ATG4B OS=Gallus	ATG4B	44.53

A0A1D5PGL4	Myosin XVIIIA(predicted) OS=Gallu	MYO18A	228
A0A3Q3A7W7	Plectin(predicted) OS=Gallus gallus (	LOC107050990	48.159
A0A1D5PYS3	Replication factor C subunit 2 OS=Ga	RFC2	36.601
E1BQK7	Tudor domain-containing protein OS=	SMNDC1	26.654
A0A1L1RJM3	Purine nucleoside phosphorylase OS=	PNP	32.239
E1BUG3	STEAP4 metalloreductase(predicted)	STEAP4	53.433
E1C1J0	ADP-ribosylation factor-related prote	ARFRP1	22.769
A0A1D5P628	HSPA (Hsp70) binding protein 1(pred	HSPBP1	35.742
A0A1D5PWP7	Protein disulfide-isomerase A4 OS=C	PDIA4	72.678
A0A1L1RMC0	Proteasome subunit alpha type OS=G	PSMA1	29.325
A0A1D5NUK5	Adenine phosphoribosyltransferase O	APRT	19.521
A0A1D5PIC1	WD_REPEATS_REGION domain-co	ATG16L2	65.817
A0A3Q2U504	Vitamin K-dependent protein S OS=C	PROS1	74.1
F1NFJ9	Squalene synthase OS=Gallus gallus (	FDFT1	48.05
A0A3Q2UHP6	Nudix hydrolase 6 OS=Gallus gallus	--	35.277
A0A1L1RWY1	Mitochondrial ribosomal protein L47(	MRPL47	29.391
A0A1D5P5I0	RNA helicase OS=Gallus gallus OX=	IFIH1	125.03
E1BVM7	Coiled-coil domain containing 134(pr	CCDC134	26.549
A0A3Q2U2Y3	Cell division cycle and apoptosis regu	--	53.306
F1NSI7	Rab-GAP TBC domain-containing pr	TBC1D10A	53.484
A0A1D5PAT5	SWI/SNF related, matrix associated, a	SMARCC1	117.73
A0A1D5NXB2	Dishevelled segment polarity protein	DVL2	80.059
P16236	Proto-oncogene c-Rel OS=Gallus gal	REL	66.846
Q90592	Solute carrier family 2, facilitated glu	SLC2A2	57.699
A0A3Q2U9U9	Non-specific serine/threonine protein	OXSR1	58.115
F1P5I5	UBC core domain-containing protein	UBE2O	149.72
A0A1D5PH50	RBR-type E3 ubiquitin transferase OS	ARIH1	63.838
A0A1D5PJW4	Proteasome subunit alpha type OS=G	PSMA7	27.872
A0A1D5PX34	Protein-tyrosine-phosphatase OS=Ga	PTPRM	163.5
F1P3R7	UBX domain-containing protein OS=	UBXN6	49.782
F1P1Y1	Rho guanine nucleotide exchange fac	ARHGEF12	172.6
A0A1D5PS29	Elongation factor 2 OS=Gallus gallus	EEF2	95.359
F1NJU7	Tyrosine--tRNA ligase OS=Gallus ga	YARS	60.147

Q5ZM60	Cell cycle progression protein 1 OS=(	CCPG1	93.483
Q5F366	Alpha-L-iduronidase(predicted) OS=(	IDUA	71.563
E1C9H4	28S ribosomal protein S17, mitochon	MRPS17	15.502
A0A1D5PN47	Histone deacetylase 10 OS=Gallus ga	--	33.46
A0A1D5PY98	UFM1-activating enzyme OS=Gallus	UBA5	43.846
A0A1D5NXZ5	4_1_CTD domain-containing protein	--	13.914
E1BXT7	ANK_REP_REGION domain-contair	ANKHD1	270.05
F1NYK3	Methylcrotonoyl-CoA carboxylase 1(	MCCC1	83.467
E1BZJ9	Aminotran_5 domain-containing prot	SCLY	45.553
F1NRE4	Beta-galactoside alpha-2,6-sialyltrans	ST6GAL1	47.378
A0A1D6UPT4	Apoptosis inducing factor mitochond	AIFM1	75.014
A0A3Q3AHD7	MICOS complex subunit MIC60 OS=	IMMT	82.269
A0A1D5PNB0	Calcium/calmodulin-dependent prote	CAMK2D	54.205
P08641	Cadherin-1 OS=Gallus gallus OX=90	CDH1	97.782
F1P1W9	POU domain protein OS=Gallus gallu	CCDC160	57.238
A0A3Q2UC15	PPFIA binding protein 2(predicted) C	PPFIBP2	93.918
Q5ZKN2	Membrane-associated progesterone re	PGRMC1	21.282
A0A1D5PPU9	Catalase OS=Gallus gallus OX=9031	CAT	59.614
Q5ZM36	Eukaryotic initiation factor 4A-III OS	EIF4A3	46.815
A0A1D5P5L2	Coagulation factor V(predicted) OS=(	F5	229.84
F1NQ58	CENP-V/GFA domain-containing pro	CENPV	23.029
A0A1D5P7K7	Septin OS=Gallus gallus OX=9031 G	SEPT7	49.411
F1NQA6	RNA helicase OS=Gallus gallus OX=	DDX52	67.773
A0A1D5PQ96	3-hydroxyisobutyrate dehydrogenase	HIBADH	31.781
F1NMN7	Aldedh domain-containing protein OS	ALDH9A1	63.674
A0A1D5P2D5	Isoleucyl-tRNA synthetase OS=Gallu	IARS	146.56
A0A3Q2UP33	ATP binding cassette subfamily B me	ABCB6	95.931
F1NI13	BSD domain-containing protein OS=(	SYAP1	40.826
E1C4U9	BPL/LPL catalytic domain-containing	HLCS	99.152
F1NW03	PPM-type phosphatase domain-conta	PDP2	60.686
F1NCS3	Ceramidase OS=Gallus gallus OX=9(	NAAA	39.858
E1C7C1	Complement component 8 subunit be	C8B	65.365
A0A3Q2UHN7	FCH and double SH3 domains 1(pred	FCHSD1	79.919

A0A1D5PLV9	TraB domain containing(predicted) O	TRABD	37.924
Q5F3K4	WD repeat-containing protein 48 OS=	WDR48	76.313
R4GHB0	Phosphatidylinositol transfer protein,	--	30.766
A0A1L1RML3	LisH domain-containing protein OS=	TCOF1	94.368
F1NPF7	PH domain-containing protein OS=G	FERMT1	77.435
Q5ZJL5	Adenylosuccinate synthetase isozyme	ADSS2	49.454
Q90952	Serum paraoxonase/arylesterase 2 OS	PON2	39.408
A0A1D5P814	Protein kinase domain-containing pro	CAMK1D	42.211
A0A1D5PJN1	UBC core domain-containing protein	BIRC6	525.35
A0A3Q3AD64	Mesoderm induction early response p	MIER1	55.411
Q5ZK09	Protein Hikeshi OS=Gallus gallus OX	HIKESHI	21.773
A0A3S5ZP30	C-type lectin domain-containing prote	CD72	44.36
E1BVT7	Mitochondrial ribosomal protein L18	MRPL18	20.856
Q5ZLH0	NTF2-related export protein 2 OS=Ga	NXT2	16.162
A0A3Q2TRU7	IRF tryptophan pentad repeat domain-	IRF6	52.667
E1BUB6	Glutathione transferase OS=Gallus ga	GSTT1L	27.954
Q90891	Dual specificity mitogen-activated pro	MAP2K2	44.077
P50460	Cysteine and glycine-rich protein 2 O	CSRP2	20.925
A0A1L1RSJ8	Far upstream element binding protein	FUBP1	65.129
A0A1D5PFP1	Complex I-9kD OS=Gallus gallus OX	NDUFV3	10.677
A0A3Q2UCQ1	SAP domain-containing protein OS=(	LOC112530285	19.388
P07032	Acylphosphatase-1 OS=Gallus gallus	ACYP1	11.15
A0A3Q2U536	RanBP2-type domain-containing prot	LOC101751687	51.62
A0A1D5PFV9	Protein kinase domain-containing pro	SRPK1	83.694
A0A3Q3AU43	Biorientation of chromosomes in cell	--	17.125
A0A1D5PLB3	Non-lysosomal glucosylceramidase C	GBA2	89.856
E1C4M6	Ubiquitin carboxyl-terminal hydrolase	USP14	52.671
A0A1D5NXK6	UBA domain-containing protein(pred	DDI2	111.16
E1C9F5	Pitrilysin metalloproteinase 1 OS=Ga	PITRM1	117.31
Q5ZMI9	Vesicle-fusing ATPase OS=Gallus ga	VPS4B	48.698
F1P204	Multidrug resistance-associated prote	ABCC1	170.87
E1C9E6	General transcription factor IIH subur	GTF2H2	44.731
F1NRK3	Ribosomal_L7Ae domain-containing	RPP38	32.375

P08642	GTPase HRas OS=Gallus gallus OX=	HRAS	21.365
E1C6U1	Phosphatidate phosphatase OS=Gallu	LPIN2	95.88
A0A1D5PHE2	Inositol-1-monophosphatase OS=Gal	IMPA1	30.142
A0A3Q2U6Y1	Radical S-adenosyl methionine and fl	TYW1	77.865
A0A1D5PDW6	Phosphatidylinositol-3,4,5-trisphosph	INPP5D	129.28
Q00649	Heat shock protein beta-1 OS=Gallus	HSPB1	21.671
A0A1D5NYZ0	WD_REPEATS_REGION domain-co	RRP9	31.12
A0A1D5PRZ0	Golgi associated, gamma adaptin ear	GGA1	73.047
A0A1L1RVZ0	28S ribosomal protein S15, mitochon	--	20.092
Q5F371	Protein strawberry notch homolog 1 (	SBNO1	137.18
A0A3Q2UGP8	SANT domain-containing protein OS	LOC101747869	38.354
A0A3Q2TY77	RAB15, member RAS oncogene fam	RAB15	24.177
O13016	Tyrosine-protein phosphatase non-rec	PTPN1	50.333
A0A1D5PQX8	C2H2-type domain-containing protein	ZNF385D	51.564
F1NNL9	ApaG domain-containing protein OS=	POLDIP2	42.684
A0A1D5PDT3	Calcium-transporting ATPase OS=Ga	ATP2B4	124.08
A0A1D5P8H8	Histone acetyltransferase OS=Gallus	CREBBP	261.94
F1P319	Torsin 1A interacting protein 2(predic	TOR1AIP2	55.906
E1C7Z0	WD_REPEATS_REGION domain-co	UTP18	66.495
A0A3Q2TXI9	Protein kinase domain-containing pro	NEK9	113.02
E1C852	PDZ and LIM domain 1(predicted) O	PDLIM1	35.66
A0A1L1RVP3	CHCH domain-containing protein OS	CHCHD2P9	15.248
F1NK56	N-acetylneuraminate lyase OS=Gallus	NPL	36.054
A0A1D5PC90	Histidine--tRNA ligase OS=Gallus ga	HARS	56.684
E1C903	Polyadenylate-binding protein OS=Ga	PABPC4	69.629
E1BSZ0	PlsC domain-containing protein OS=(	AGPAT5	42.376
Q5F3W4	Carboxypeptidase OS=Gallus gallus (	SCPEP1	49.977
F1NBU5	ATP synthase subunit s, mitochondria	ATP5S	22.696
F1P100	ElaC homolog protein 2 OS=Gallus g	ELAC2	94.237
F1NSN1	Microtubule associated protein 9(pred	MAP9	48.849
A0A1D5PXR9	USP domain-containing protein OS=(	USP25	120.85
Q5ZLR4	Epithelial splicing regulatory protein	ESRP2	76.946
P21868	Casein kinase II subunit alpha OS=Ga	CSNK2A1	45.19

A0A3Q2UAT5	KASH domain-containing protein OS	SYNE3	106.88
A0A1L1RIY5	CIDE-N domain-containing protein C	DFFA	34.417
A0A1D5PHD7	Calcium/calmodulin dependent serine	CASK	100.82
A0A1D5Q008	Mannose-6-phosphate isomerase OS=	MPI	45.847
A0A3Q3A3H5	Solute carrier family 44 member 2(pr	LOC107050443	73.826
E1BYZ3	MICAL like 2(predicted) OS=Gallus	MICALL2	69.754
R4GGJ0	Ribosomal protein S16(predicted) OS	RPS16	16.413
P00174	Cytochrome b5 OS=Gallus gallus OX	CYB5A	15.545
E1BVG8	RAB3 GTPase activating non-catalyti	RAB3GAP2	154.51
Q8JHU1	Liver X receptor OS=Gallus gallus O	LXR	47.055
Q5ZMD4	Tripartite motif-containing protein 59	TRIM59	46.775
R4GJD6	Eukaryotic translation initiation facto	--	28.102
F1NWX6	Plasminogen OS=Gallus gallus OX=9	PLG	90.763
E1BT44	Alpha-galactosidase OS=Gallus gallu	GLA	45.942
A0A1L1RYV5	tRNA (guanine-N(7)-)-methyltransfer	WDR4	46.9
E1BU27	Aldo_ket_red domain-containing prot	AKR1D1	49.535
E1C8K3	Annexin OS=Gallus gallus OX=9031	ANXA8L1	36.732
A0A3Q3AN15	ARID domain-containing protein OS=	ARID1B	161.97
E1C136	Chloride intracellular channel protein	CLIC5	30.309
A0A1D5P6I0	DNA primase OS=Gallus gallus OX=	PRIM1	44.991
Q5ZM11	Arginine--tRNA ligase, cytoplasmic (	RARS1	75.417
A0A1D5P834	Kynureninase OS=Gallus gallus OX=	KYNU	53.218
A0A3Q2UPU0	Protein kinase domain-containing pro	--	66.894
A0A3Q2UPC8	Filamin (Fragment)(predicted) OS=G	FLNA	50.352
F1NX13	Developmentally regulated GTP bind	DRG1	40.46
A0A1D5NTS9	AH domain-containing protein OS=G	ARFIP2	43.255
F1NU68	O-GlcNAcase(predicted) OS=Gallus	MGEA5	102.33
E1BUW5	Phosphatidate cytidylyltransferase, m	TAMM41	37.556
A0A1D5PKQ7	tRNA (guanine(37)-N1)-methyltransf	TRMT5	56.533
E1BUN2	Exocyst complex component Sec8 OS	EXOC4	111.05
Q5ZMA7	Protein kinase domain-containing pro	STK26	46.804
F1P231	Ras-related protein Rap-2c(predicted)	RAP2C	20.725
F1NTI4	GTP-binding protein 8 OS=Gallus ga	GTPBP8	35.362

F1NLM0	FPG_CAT domain-containing protein	NEIL1	42.388
E1BY49	PlsC domain-containing protein OS=(	AGPAT4	44.389
Q5ZL03	GCS light chain OS=Gallus gallus O	BCAR3	30.262
A0A1D5PGB1	Galactokinase 2(predicted) OS=Gallu	GALK2	50.067
Q1KME6	Ras-related protein Rab-6A OS=Gallu	RAB6A	23.49
A0A1D5P6E8	AMP-binding domain-containing pro	CDH15	67.248
A0A3Q3A7M9	SH3 domain-containing protein OS=(	MIA2	157.35
Q5ZI69	39S ribosomal protein L37, mitochon	MRPL37	49.317
F1NBV7	PNPLA9(predicted) OS=Gallus gallu	PLA2G6	88.393
Q90Z16	Optineurin OS=Gallus gallus OX=90	OPTN	64.087
O73884	Phosphoethanolamine/phosphocholin	PHOSPHO1	30.443
A0A1D5PBP6	Ceruloplasmin(predicted) OS=Gallus	CP	130.81
Q8AWB8	Structural maintenance of chromoson	SMC3	141.57
Q5ZJD3	Large subunit GTPase 1 homolog OS	LSG1	73.83
R4GLW2	Adaptor protein, phosphotyrosine inte	APPL2	75.381
F1NLG3	TYR_PHOSPHATASE_2 domain-co	PTP4A2	19.025
A0A3Q2U863	F-box domain-containing protein OS=	FBXW9	34.984
A0A1L1RLU2	Vacuolar protein sorting-associated p	VPS33A	68.546
F1NSB6	Ankyrin repeat and FYVE domain co	ANKFY1	118.45
E1BS68	PRKCSH_1 domain-containing prote	GNPTG	34.795
A0A1D5NZA6	Exocyst complex component OS=Gal	EXOC6	87.799
A0A1D5PKJ9	RNA-binding motif protein 47 OS=G	--	42.493
Q5ZL26	Phosphoribosyl pyrophosphate syntha	PRPSAP2	40.803
E1C0L2	3-hydroxyanthranilate 3,4-dioxygenas	HAAO	33.163
A0A1D5NX34	Stabilin 2(predicted) OS=Gallus gallu	STAB2	272.74
A0A3Q2TX33	A2M domain-containing protein OS=	--	13.979
A0A1D5PN46	Fructose-bisphosphatase OS=Gallus g	FBP2	37.102
A0A1L1RSA2	Programmed cell death 6(predicted) (	PDCD6	19.692
A0A1D5NWV6	Sorting nexin 27(predicted) OS=Gallu	SNX27	60.32
A0A1D5PKH4	Fe_hyd_SSU domain-containing prot	NARFL	52.212
F1NG56	Rho GTPase activating protein 5(prec	ARHGAP5	172.09
A0A1D5P167	Folate gamma-glutamyl hydrolase OS	GGH	48.209
F1NNQ9	WD_REPEATS_REGION domain-co	PRPF4	59.806

Q5ZM30	Regulation of nuclear pre-mRNA dor	RPRD1A	30.836
A0A1D5PKK5	Fibronectin type III domain containin	FNDC3B	131.41
F1NLI6	DZF domain-containing protein OS=(	ZFR2	116.42
P62303	Small nuclear ribonucleoprotein E OS	SNRPE	10.803
A0A1D5NW78	Sperm associated antigen 17(predicte	SPAG17	198.88
A0A3Q2U734	Fibronectin type-III domain-containin	LOC422151	119.27
A0A140T8F5	Ig-like domain-containing protein OS	PIGR	70.57
F1NG68	2,4-dienoyl-CoA reductase 2(predicte	DECR2	37.275
A0A1L1RS21	DAO domain-containing protein OS=	FOXRED1	54.567
F1P156	Crystallin lambda 1(predicted) OS=G	CRYL1	34.945
A0A1D5PU43	Enoyl-CoA delta isomerase 1(predicte	ECI1	34.264
Q5ZIK9	Coatomer subunit epsilon OS=Gallus	COPE	34.369
A0A3Q2UBQ4	TPR_REGION domain-containing pr	--	20.113
E1C7Z1	Tyrosine-protein kinase OS=Gallus g	FER	94.526
A0A1D5NV75	NSF attachment protein alpha(predict	NAPA	33.244
R4GJY1	ORM1-like protein OS=Gallus gallus	ORMDL3	17.665
F1NQV5	RNA helicase OS=Gallus gallus OX=	DDX27	85.637
Q98SE2	U2snRNP auxiliary factor small subu	U2AF1	27.701
A0A1D5PV66	NIDO domain-containing protein OS	LOC100857892	29.883
E1C6A9	Cell division cycle 5 like(predicted) (	CDC5L	101.54
A0A1D5P380	EGF containing fibulin extracellular r	EFEMP1	50.653
E1C9D8	tRNA-uridine aminocarboxypropyltra	DTWD1	35.284
A0A3Q2U609	Putative aarF domain-containing prot	--	58.07
F1NMQ2	Epimerase_2 domain-containing prot	GNE	82.777
F1NEN7	Ubiquinone biosynthesis protein COQ	COQ4	29.075
Q5ZI60	Coronin OS=Gallus gallus OX=9031	CORO1C	53.207
A0A3Q2UBC4	SUN domain-containing protein OS=	SUCO	114.38
P00698	Lysozyme C OS=Gallus gallus OX=9	LYZ	16.238
A0A1D5PZ37	Sorting nexin 14(predicted) OS=Gallu	SNX14	105.1
F1NBI2	Uroporphyrinogen decarboxylase OS=	UROD	36.97
A0A1D5P8W3	Glycine N-methyltransferase OS=Gal	--	19.558
F1NVJ2	PKS_ER domain-containing protein (	LOC424430	36.649
A0A1D5PJP2	Sarcosine dehydrogenase(predicted) (	--	28.186

A0A1L1RP46	RAB1A, member RAS oncogene fam	RAB1B	22.708
F1NQF4	Heme oxygenase (biliverdin-producir	HMOX1	35.738
A0A1L1RPC6	MSP domain-containing protein OS=	VAPA	27.755
A0A1D5PEZ3	Dynactin subunit 1 OS=Gallus gallus	DCTN1	139.91
A0A1D5PLH4	Nucleoplasmin domain-containing pr	NPM3	16.367
A0A3Q2UIM1	Cytidine deaminase OS=Gallus gallus	CDA	14.996
E1C7T6	tRNA wybutosine-synthesizing protei	TYW5	37.244
A0A1D5NVH6	6-phosphofructo-2-kinase OS=Gallus	PFKFB4L	50.442
A0A1D5P8W2	Mediator of RNA polymerase II trans	MED4	28.679
F1NYZ7	GST N-terminal domain-containing p	CLIC4	28.521
A0A3Q2U186	Biogenesis of lysosome-related organ	BLOC1S5	22.875
Q5ZJ73	Protein CREG1 OS=Gallus gallus OX	CREG1	21.106
A0A3Q2UGJ5	EMAP like 4(predicted) OS=Gallus g	EML4	105.89
A0A1D5NZ55	UDP-glucose glycoprotein glucosyltra	UGGT1	170.79
A0A1L1RSV3	Beclin-1 OS=Gallus gallus OX=9031	BECN1	51.134
A0A1D6UPQ3	Threonyl-tRNA synthetase OS=Gallu	TARS	90.648
A0A1L1RSG1	Cytochrome c oxidase subunit OS=G	COX6A1	11.815
A0A1L1RIW5	IF rod domain-containing protein OS=	KRT8	60.513
F1NTT5	Rab23 GTPase(predicted) OS=Gallus	RAB23	26.836
A0A3Q2U6W6	B30.2/SPRY domain-containing prot	HNRNPUL1	92.862
A0A3Q3ASJ5	Kininogen-1a (Fragment)(predicted) (	--	109.55
A0A1D5P620	Alba domain-containing protein OS=	RPP25L	20.037
Q5ZMI5	RAB9A, member RAS oncogene fam	RAB9A	22.818
A0A3Q2U0P8	VWFA domain-containing protein OS	ITGA11	134.04
Q5ZLT2	Protein-serine/threonine kinase OS=C	PDK3	46.63
E1C5M4	APH domain-containing protein OS=	HYKK	41.417
F1NL88	PKS_ER domain-containing protein (	RTN4IP1	39.927
A0A1D5P991	ANK_REP_REGION domain-contair	RAI14	107.15
Q90617	Lysosome-associated membrane glyc	LAMP2	46.715
A0A1D5NX82	Nuclear cap-binding protein subunit 2	NCBP2	18.07
A0A1D5PJG5	Protein polybromo-1 OS=Gallus gallu	PBRM1	181.91
F1NZI4	Protein-glucosylgalactosylhydroxylys	PGGHG	78.357
A0A1D5PNJ4	GP-PDE domain-containing protein (	GDE1	33.228

A0A1D5PWK8	SCO cytochrome c oxidase assembly	SCO1	33.773
A0A3Q2TU86	HMG box domain-containing protein	TGOLN2	47.118
A0A1D5PBW1	NudC domain-containing protein 1 O	NUDCD1	83.435
A0A3Q2UE77	Protein Noxp20(predicted) OS=Gallu	FAM114A1	60.687
A0A1D5P6S9	Family with sequence similarity 234 r	FAM234A	61.494
A0A1D5PJ08	Nidogen 1(predicted) OS=Gallus gall	NID1	126.52
R4GGG5	Microfibrillar-associated protein 1 OS	MFAP1	51.969
E1BWI0	SH3 domain-containing protein OS=(	DSP	330.76
F1NT40	Secretion associated Ras related GTP	SAR1A	22.391
A0A1D5PIK2	Zinc finger CCCH-type containing 7E	ZC3H7B	113.94
A0A1D5NTW8	Laminin subunit alpha 3(predicted) O	LAMA3	381.89
E1BVU7	Sperm antigen with calponin homolog	SPECC1	86.226
A0A3Q2UL92	G-patch domain-containing protein O	RBM6	132.46
A0A3Q3B1H6	t-SNARE coiled-coil homology doma	STX2	29.982
F1NG51	Vav guanine nucleotide exchange fac	VAV2	97.47
A0A1L1RT07	Beta-N-acetylhexosaminidase OS=Ga	HEXA	70.601
A0A1L1RU05	Receptor expression-enhancing protei	REEP6	21.014
F1NYK1	Palmitoyl-protein hydrolase 1 OS=Ga	PPT1	33.857
F1NUD1	GPN-loop GTPase OS=Gallus gallus	GPN1	41.497
A0A1D5PWD7	Flotillin OS=Gallus gallus OX=9031	FLOT1	57.625
A0A3Q2UBD4	LIM zinc-binding domain-containing	--	8.5607
Q5ZLX8	MBD domain-containing protein(prec	MBD3	26.146
Q5ZM83	Mitochondrial Rho GTPase 2 OS=Ga	RHOT2	69.07
A0A1D5PQI1	Galectin OS=Gallus gallus OX=9031	LGALS3	27.253
F1NVA1	Acyl-CoA synthetase medium chain f	ACSM3	64.839
P79769	Translin OS=Gallus gallus OX=9031	TSN	25.96
E1C0J4	UPF1 RNA helicase and ATPase(prec	UPF1	123.4
A0A1D5P2Z2	Palmitoyltransferase OS=Gallus gallu	ZDHHC18	38.992
A0A1D5P786	WD repeat domain 75(predicted) OS=	WDR75	93.623
A0A1L1RUB9	AAA domain-containing protein OS=	RFC4	39.722
A0A1D5NWX3	Geranylgeranyl diphosphate synthase	GGPS1	34.472
A0A1D5NTA7	WD repeat and FYVE domain-contai	WDFY2	41.513
F1P0B2	Amyloid-beta A4 protein OS=Gallus	APP	78.541

E1C4N5	Dolichol-phosphate mannosyltransfer	DPM1	27.195
A0A3Q2UEQ5	Fission, mitochondrial 1 OS=Gallus g	--	17.663
F1NRT5	Acid phosphatase 6, lysophosphatidic	ACP6	46.821
A0A3Q2U8C2	MICOS complex subunit MIC13 OS=	C19orf70	13.47
F1NM87	Amine oxidase OS=Gallus gallus OX	IL4I1	59.241
E1C319	t-SNARE coiled-coil homology doma	STX12	30.797
E1C155	Aldedh domain-containing protein OS	ALDH6A1	54.969
A0A1D5P765	Transket_pyr domain-containing prot	BCKDHB	42.501
Q5ZMK3	Sacchrp_dh_NADP domain-containin	SCCPDH	46.854
R4GMA6	Ras homolog family member G OS=(	--	37.703
Q5ZHV2	N-alpha-acetyltransferase 35, NatC au	NAA35	83.538
A0A1D5NTB4	BRM protein(predicted) OS=Gallus g	SMARCA2	176.74
Q5ZJ38	RanBD1 domain-containing protein C	RANBP1	24.171
Q5ZIH9	Coiled-coil domain-containing protein	CCDC174	59.322
Q5ZHN3	WD repeat domain phosphoinositide-	WIPI2	47.775
A0A1L1RYW1	BCS1-like protein OS=Gallus gallus (	BCS1L	47.486
Q5ZI39	Plastin 3(predicted) OS=Gallus gallus	PLS3	70.823
F1NSW0	MSP domain-containing protein OS=	VAPB	27.305
Q5ZLN4	Flap endonuclease 1 OS=Gallus gallu	FEN1	42.983
F1N9B7	RAD23 homolog B, nucleotide excisi	RAD23B	53.54
Q5ZM48	Synaptogyrin OS=Gallus gallus OX=9	SYNGR2	25.068
F1NLS1	MRC1L-A(predicted) OS=Gallus gal	MRC1	166.46
A0A3Q3B2L3	Alpha-2-macroglobulin-like protein 1	--	52.034
Q5ZLG1	Ras-related protein Rab-18 OS=Gallu	RAB18	22.954
F1ND23	Complex I-30kD OS=Gallus gallus O	NDUFS3	29.232
A0A1D5PTF6	Family with sequence similarity 13 m	FAM13A	80.744
F1NZC2	AP-3 complex subunit mu-1 OS=Gal	AP3M1	46.996
F1NEF8	NHL repeat-containing protein 2 OS=	NHLRC2	79.539
A0A1D5PUD5	Nucleoporin 88(predicted) OS=Gallu	NUP88	77.629
A0A1D5NWU3	Active breakpoint cluster region-relat	ABR	99.016
F1NJ62	RNA helicase OS=Gallus gallus OX=	DDX24	91.668
F1NQG5	Ribosomal protein L15 OS=Gallus ga	RPL15	24.132
A0A3Q2U018	Fam20C domain-containing protein C	--	15.451

A0A1D5PL85	Selenoprotein O OS=Gallus gallus O	SELENOO	94.526
A0A1L1RWP3	EW135(predicted) OS=Gallus gallus	DMBT1L	97.774
F1NYN9	NTP_transferase domain-containing p	GMPPA	47.02
Q5ZJ25	Vacuolar protein sorting-associated p	VPS51	86.058
A0FKN5	Prestin OS=Gallus gallus OX=9031 (	SLC26A5	81.112
A0A1D5P6A0	Phosphodiesterase OS=Gallus gallus	PDE8A	92.165
A0A1D5PVT7	TGc domain-containing protein OS=(	TGM3	70.672
E1BQW2	DUF155 domain-containing protein (	RMND1	51.897
P12003	Vinculin OS=Gallus gallus OX=9031	VCL	124.56
F1NHR3	VLIG-type G domain-containing prot	GVINP1	278.74
A0A1D5PAV5	Unc-51 like kinase 4(predicted) OS=(	--	38.538
P51890	Lumican OS=Gallus gallus OX=9031	LUM	38.642
P14093	ATP synthase protein 8 OS=Gallus ga	MT-ATP8	6.3764
F1NB38	Ethylmalonyl-CoA decarboxylase OS	ECHDC1	32.516
A0A1D5PU94	C4a anaphylatoxin OS=Gallus gallus	C4A	189.99
F1NA58	Calponin-homology (CH) domain-co	--	82.606
E1BYM1	mRNA cap guanine-N7 methyltransfe	RNMT	47.54
R4GH41	Ceramidase OS=Gallus gallus OX=9(	ASAH1	44.583
A0A1D5PPQ7	Eukaryotic translation initiation facto	EIF4E	25.044
E1BYA9	PPM-type phosphatase domain-conta	ILKAP	42.675
A0A1D5PN97	Methylthioribose-1-phosphate isomer	MRI1	36.976
Q5ZMU0	28S ribosomal protein S7, mitochond	MRPS7	27.279
F1NEB5	Dihydroxyacetone phosphate acyltran	PDCD2	99.219
E1BUZ7	ATP synthase mitochondrial F1 comp	ATPAF2	33
Q08200	60S ribosomal protein L10 (Fragmen	RPL10	23.987
E1BYD1	Coiled-coil domain-containing protei	CCDC53	21.876
A0A1D5NXV3	Aspartate beta-hydroxylase OS=Gallu	--	33.792
F1P5G4	Phosducin domain-containing protein	PDCL3	27.802
A0A452J7W6	RAF proto-oncogene serine/threonine	RAF1	71.857
F1NBG8	PABS domain-containing protein OS	SMS	41.911
R4GIV8	UBC core domain-containing protein	UBE2R2	27.178
A0A1D5PD21	DENN domain containing 4A(predict	DENND4A	209.66
A0A1D5P9S3	THUMP domain-containing protein (	--	25.408

F1NWH5	Aquaglyceroporin-3 OS=Gallus gallu	AQP3	31.377
P07630	Carbonic anhydrase 2 OS=Gallus gall	CA2	29.007
A0A1D5P061	Phosphorylase b kinase regulatory sul	PHKA1	134.07
E1C231	E3 ubiquitin-protein ligase listerin OS	LTN1	199.44
F1NJF8	Alpha-galactosidase OS=Gallus gallu	NAGA	47.4
A0A1D5PQ16	Unconventional myosin-6 OS=Gallus	MYO6	145.02
Q9PU45	Radixin OS=Gallus gallus OX=9031	RDX	68.554
A0A1D5PW44	Peptidyl-prolyl cis-trans isomerase OS	PPIL1	18.095
R4GFH1	Caveolae associated protein 2(predict	PRKCDBP	43.376
F1NDL4	Nidogen 2(predicted) OS=Gallus gall	NID2	120.57
H9KZ12	Scaffold attachment factor B1(predict	SAFB2	108.41
A0A1D5PYP4	CRAL-TRIO domain-containing prot	TTPA	31.482
H9KZL2	Nicastrin OS=Gallus gallus OX=9031	NCSTN	79.495
R4GJ94	Dynamin-like 120 kDa protein, form	OPA1	115.81
A0A1D5P8P5	NTP_transferase domain-containing p	EIF2B3	48.909
A0A3Q3AKG1	Protein kinase domain-containing pro	CDK18	58.352
A0A1D5PJW3	EH domain binding protein 1(predicte	EHBP1	132.34
F1NET8	Tetratricopeptide repeat protein 36 OS	TTC36	20.52
A0A1D5P6Q5	3-hydroxyacyl-[acyl-carrier-protein] c	FASN	274.77
A0A1L1RLU1	Sulfotransferase OS=Gallus gallus OX	SULT6B1L	36.699
E1BVM8	Protein-serine/threonine phosphatase	PPM1A	42.479
A0A1D5PM40	Ras homolog, mTORC1 binding(prec	RHEB	18.792
A0A1D5NWN5	Deacetylase sirtuin-type domain-conta	SIRT2	52.217
Q5ZJ61	Phenylalanyl-tRNA synthetase beta su	FARSB	65.893
A0A1D5PTI9	Ubiquitin-protein ligase E3A OS=Ga	UBE3A	100.99
Q6EE31	T-complex protein 1 subunit theta OS	CCT8	59.374
A0A1D5PIW9	Ankyrin-3 (Fragment)(predicted) OS=	ANK3	475.9
F1NLT8	Rho GDP dissociation inhibitor beta(p	ARHGDIB	22.886
F1NDZ7	YccV-like domain-containing protein	FBXO21	70.565
Q6QLR2	Gallinacin-7 OS=Gallus gallus OX=9	GAL7	7.563
E1BVP9	Pre-mRNA-splicing factor 38B OS=G	PRPF38B	64.892
E1C2H6	Kynurenine formamidase OS=Gallus	AFMID	32.31
Q8JHU2	Farnesoid X receptor OS=Gallus gallu	FXR	54.535

A0A1L1RZN9	Vacuolar protein sorting-associated p	VPS33B	74.877
F1NLE3	Fibronectin type-III domain-containin	CRLF3	49.676
A0A1D5PL72	Ubiquitinyl hydrolase 1 OS=Gallus ga	VCPIP1	134.38
A0A3Q2U1W6	RING-type E3 ubiquitin transferase C	IFI35	125.89
F1NT93	DJ-1_PfpI domain-containing protein	GATD3A	27.3
A0A3Q2U989	Phosphoribosylformylglycinamidine s	--	14.721
F1P1G4	Ras-related protein Rab-43(predicted)	RAB43	23.762
A0A3Q2UEX2	Cysteine-S-conjugate beta-lyase 2 OS	KYAT3	47.368
A0A1D5PA36	CDK5 regulatory subunit associated p	CDK5RAP3	57.131
A0A1D5PIT3	Protein argonaute-3 OS=Gallus gallus	AGO3	95.934
Q5ZJ67	Nudix hydrolase domain-containing p	NUDT14	25.807
F1NMC3	C-1-tetrahydrofolate synthase, cytopla	MTHFD1	100.22
F1N843	Tyrosine-protein phosphatase non-rec	PTPN6	68.366
A0A1L1RNP9	Zinc finger protein 207(predicted) OS	ZNF207	47.896
P17785	Annexin A2 OS=Gallus gallus OX=9	ANXA2	38.64
F1NYI8	Lon protease homolog 2, peroxisomal	LONP2	94.678
F1ND18	Protein tyrosine phosphatase mitocho	PTPMT1	21.779
F1P179	Glutamyl-tRNA synthetase OS=Gallu	EPRS	179.86
A0A1D5PP94	Dynamin GTPase OS=Gallus gallus C	LOC430067	90.214
A0A1L1RR29	RRM domain-containing protein OS=	SLIRP	10.877
E1BS70	Otoferlin(predicted) OS=Gallus gallu	OTOF	204.17
P09324	Tyrosine-protein kinase Yes OS=Gall	YES1	60.792
F1NG88	PSI domain-containing protein OS=G	PTTG1IP	20.096
A0A1D5P287	Myosin-11 OS=Gallus gallus OX=90	MYH11	228.72
A0A1D5PVA4	AAA domain-containing protein OS=	ATAD3B	67.219
A0A1L1RYG0	BTB domain-containing protein OS=	KCTD12	32.664
F1NCM5	15-oxoprostaglandin 13-reductase OS	PTGR1	35.937
A0A1D5PAB0	Unconventional myosin-Va OS=Gallu	MYO5A	212.52
F1P372	Threonyl-tRNA synthetase OS=Gallu	TARSL2	90.262
Q6JLB2	Proteasome subunit beta OS=Gallus g	PSMB1	25.984
F1N9Q5	Protein CEPU-1(predicted) OS=Gallu	NTM	36.263
Q5ZKJ9	V-type proton ATPase subunit E 1(pr	ATP6V1E1	26.118
P18939	NADH-ubiquinone oxidoreductase ch	MT-ND4	51.145

A0A1D5PDH1	Zinc fingers and homeoboxes 3(predi	ZHX3	94.029
F1NY37	Acyl-coenzyme A oxidase OS=Gallus	ACOX1	74.513
F1NQ35	60S ribosomal protein L35a OS=Gall	RPL35A	12.583
A0A1D5PHL0	Proteasome subunit alpha type OS=G	PSMA2	25.919
F1NBW2	H(+)-transporting two-sector ATPase	ATP6V1A	68.372
F1NGU3	Leucine rich pentatricopeptide repeat	LRPPRC	155.63
A0A1I7Q403	Ubiquitin-conjugating enzyme E2 var	UBE2V1	16.463
Q5ZKU6	Calponin OS=Gallus gallus OX=9031	CNN2	33.39
A0A1D5PUQ9	Nucleoporin p58/p45 (Fragment)(pre	NUP58	57.555
Q5ZLX5	Zinc finger Ran-binding domain-cont	ZRANB2	37.863
A0A1D5P2A5	TIP120 domain-containing protein OS	CAND2	134.42
A0A1D5PG53	ATP-dependent dihydroxyacetone kin	TKFC	59.005
A0A1D5PLK6	BRO1 domain-containing protein OS	PDCD6IP	97.44
A0A3Q2U120	RAN binding protein 2(predicted) OS	LOC776067	170.98
A0A1D5PFT2	BRISC and BRCA1-A complex mem	BABAM1	49.79
Q5ZME7	YTH domain-containing protein OS=	YTHDF1	61.286
Q5ZKJ2	14_3_3 domain-containing protein OS	YWHAH	28.342
F1NPG2	Isocitrate dehydrogenase [NADP] OS	IDH1	46.602
E1BX23	Glycosyltransferase 8 domain contain	GLT8D1	42.343
A0A3Q2UHD3	Mitochondrial ribosomal protein L4 C	--	29.057
A0A1L1RPC1	Nuclear GTPase SLIP-GC (Fragment	NUGGC	86.862
F1P5I1	E3 ubiquitin-protein ligase OS=Gallu	UBR1	205.48
A0A3Q2UB48	Protein kinase domain-containing pro	TBK1	77.967
Q5ZKA4	Eukaryotic translation initiation facto	EIF3J	28.724
Q5ZI67	NHL repeat-containing protein 2 OS=	NHLRC2	79.606
A0A1D5NZS7	Nuclear factor 1 OS=Gallus gallus OX	NFIB	62.417
F1NPW0	Iodothyronine deiodinase OS=Gallus	DIO1	26.502
A0A1D5PQU1	Phenylalanine--tRNA ligase OS=Gall	FARSA	63.381
A0A1D5PXG6	USP domain-containing protein OS=G	USP12-like	42.696
E1BZ56	Proteasome assembly chaperone 2 OS	PSMG2	30.328
E1BVK8	Acyl-CoA thioesterase 8(predicted) C	ACOT8	41.398
F7BQ11	Signal transducer and activator of tra	STAT5A	86.94
R4GM98	Nucleoside diphosphate kinase OS=G	NME3	20.426

F1NS20	DUF4392 domain-containing protein	C5H14orf159	67.371
A0A1D5PGH1	Basigin OS=Gallus gallus OX=9031 (	BSG	39.823
P11799	Myosin light chain kinase, smooth mu	Mylk	210.44
P28675	Decorin OS=Gallus gallus OX=9031	DCN	39.686
P02001	Hemoglobin subunit alpha-D OS=Ga	HBAD	15.695
A0A1D5NVS0	MTSS I-BAR domain containing 1(pr	MTSS1	75.764
F1P2G6	Phosphatidylinositol glycan anchor bi	PIGT	63.76
R4GIB4	JmjC domain-containing protein OS=	JMJD7	36.419
A0A3Q2ULT0	Tripeptidyl-peptidase 2 OS=Gallus ga	TPP2	141.67
A0A1D5PQQ4	CCR4-NOT transcription complex su	CNOT10	80.869
Q5ZIY2	Putative phosphatidylinositol transfer	PITPNB	31.72
A0A3Q3AYU9	RUN and FYVE domain containing 3	RUFY3	68.342
E1C2Z5	T1-TrpRS OS=Gallus gallus OX=903	WARS	53.696
Q5ZKU5	Ras-related protein Rab-14 OS=Gallu	RAB14	23.897
Q5F3L5	Protein kinase domain-containing pro	NEK6	36.32
A0A1D5NX04	RALBP1 associated Eps domain cont	REPS1	83.991
F1NWS5	Protein kinase domain-containing pro	SCYL2	83.701
A0A3Q2U5V7	Glycerate kinase OS=Gallus gallus O	GLYCTK	52.916
Q5ZKM1	pre-rRNA 2'-O-ribose RNA methyltra	FTSJ3	95.005
A0A3Q3AN84	Mothers against decapentaplegic hom	SMAD2Z	48.927
A0A1D5NT47	Tropomyosin 4(predicted) OS=Gallus	TPM4	31.373
A0A1D5PAN6	Sorting nexin 13(predicted) OS=Gallu	SNX13	106.55
A0A1D5PV01	Carboxypeptidase OS=Gallus gallus (	CTSA	50.492
A0A3Q2UFS6	Fermitin family member 3(predicted)	--	18.572
A0A3Q2UER6	SWIM-type domain-containing protei	ZSWIM8	199.1
Q5ZKI4	Coiled-coil domain-containing protei	CCDC93	71.718
F1NRR5	Golgi associated, gamma adaptin ear	GGA3	81.786
F1NFJ5	Phosphodiesterase OS=Gallus gallus	PDE3B	121.53
A0A1D5NYB1	ATP synthase lipid-binding protein O	ATP5G2	13.746
A0A1D5PEJ0	Insulin like growth factor 2 mRNA bi	IGF2BP2	71.136
F1P463	M20_dimer domain-containing protei	CNDP2	53.064
D5LPR1	Fascin OS=Gallus gallus OX=9031 G	FSCN1	54.171
F1NZC4	Coiled-coil domain-containing protei	CCDC51	46.99

A0A1D5P8I7	Isocitrate dehydrogenase [NADP] OS	IDH2	50.482
A0A3Q3A4Z6	Olfactory receptor OS=Gallus gallus (	--	35.741
A0A3Q2U7Y4	ThiF domain-containing protein OS=(	SAE1	37.523
E1C1S5	Transcription elongation factor spt6 (	SUPT6H	199.27
F1NL82	Ethanolamine kinase 2(predicted) OS	ETNK2	44.249
E1BUD8	Protein transport protein Sec24C (Fra	SEC24C	121.68
F1NHU0	Component of oligomeric Golgi comp	COG4	87.532
F1P0U3	RAB GTPase activating protein 1(pre	RABGAP1	121.72
F1NRQ6	Glycerol kinase OS=Gallus gallus OX	GK2	57.85
F1NCE1	Eukaryotic translation initiation facto	EIF3D	63.957
P09652	Tubulin beta-4 chain OS=Gallus gallu	--	50.42
A0A1D5PYA4	LanC like 1(predicted) OS=Gallus ga	LANCL1	45.259
E1BQC5	Poly [ADP-ribose] polymerase OS=G	PARP14	206.68
A0A1D5PRV8	Mitogen-activated protein kinase OS=	MAPK11	41.661
A0A3Q2TVY3	PKS_ER domain-containing protein (	VAT1	42.453
E1BTH9	Neural Wiskott-Aldrich syndrome pro	WASL	54.776
E1C7P7	AB hydrolase-1 domain-containing pr	ABHD6	38.391
A0A3Q3AIQ1	RAB29, member RAS oncogene fami	--	21.241
R4GF38	START domain-containing protein O	LOC422154	29.668
A0A1D5P3J8	Serine hydroxymethyltransferase OS=	SHMT1	52.475
A0A1D5P893	Poly(rC) binding protein 2(predicted)	LOC426023	34.832
A0A1D5PFK7	AP4E_app_platf domain-containing p	AP4E1	127.47
Q5F495	MOB-like protein phocein OS=Gallus	MOB4	25.816
A0A1D5PYE7	Utrophin(predicted) OS=Gallus gallus	UTRN	397.25
F1N803	Complement C1s(predicted) OS=Gal	C1S	78.597
E1BS96	Actin binding LIM protein 1(predicte	ABLIM1	82.907
P07583	Beta-galactoside-binding lectin OS=(	CG-1B	15.063
A0A1D5P331	RING-type E3 ubiquitin transferase (	RC3H1	118.06
A0A1D5PYC5	Myosin-IXb (Fragment)(predicted) O	MYO9B	244.84
A0A1D5PUZ2	Glutaminyl-tRNA synthetase OS=Ga	QARS	94.011
F1NEE3	PCI domain-containing protein OS=(	PCID2	46.254
F1NYV4	Glycerophosphodiester phosphodieste	GDPD1	35.86
F1NT20	Acetyl-CoA acetyltransferase 2(predi	ACAT2	40.886

A0A1D5P3C8	SECIS binding protein 2 like OS=Gal	--	112.45
A0A1D5P6N1	WD_REPEATS_REGION domain-co	WDR43	73.063
R4GLE6	Eukaryotic translation initiation facto	EIF1	12.733
A0A3Q3AZJ6	RRM domain-containing protein OS=	RBM3	18.618
P08636	40S ribosomal protein S17 OS=Gallu	RPS17	15.538
A0A1D5NZC1	Eukaryotic translation initiation facto	EIF4G3	176.25
A0A1D5PM35	Single-stranded DNA-binding protein	SSBP3	37.694
A0A3Q2U1E2	Inverted formin-2(predicted) OS=Gal	INF2	115.08
E1C0T1	PB1 domain-containing protein OS=(	TFG	42.587
A0A3Q2TT15	tRNA (guanine(10)-N2)-methyltransf	TRMT11	52.913
F1NU38	Trafficking protein particle complex 4	TRAPPC4	31.97
F1NVJ0	4-hydroxyphenylpyruvate dioxygenas	HPD	44.963
E1C2I9	Leucyl-tRNA synthetase OS=Gallus g	LARS	134.27
E1BRI5	Abhydrolase_2 domain-containing pr	LYPLA2	25.113
Q9PSW9	Histone H2B 8 OS=Gallus gallus OX	H2B-VIII	13.964
A0A1D5PXN7	Pyridoxal dependent decarboxylase d	PDXDC1	88.224
F1NGS2	GRAM domain-containing protein OS	WBP2NL	32.534
F1NWF6	Asparagine synthetase [glutamine-hyc	ASNS	64.068
A0A1D5NTC7	GM3 synthase(predicted) OS=Gallus	ST3GAL5	42.316
A0A1D5NVF3	Sorting nexin-17 OS=Gallus gallus O	SNX17	53.024
Q5ZHT5	L51_S25_CI-B8 domain-containing p	MRPL43	16.489
A0A3Q2U5P1	Coiled-coil domain-containing protei	CCDC169	24.264
Q5ZJ39	Density-regulated protein OS=Gallus	DENR	22.158
A0A1D5NUD9	BCL2 associated transcription factor	BCLAF1	86.636
A0A1D5PF07	Protein-serine/threonine phosphatase	PPM1B	42.734
F1NV33	DNA mismatch repair protein OS=Ga	MSH2	104.9
E1BSK2	Vacuolar protein sorting-associated p	VPS18	112.57
A0A1D5P1S0	WRNPLPNID domain-containing pro	PPP4R1	100.01
A0A1D5P450	DIS3-like exonuclease 2 OS=Gallus g	DIS3L2	97.087
A0A1D5P810	ATP synthase peripheral stalk subuni	ATP5O	26.639
A0A3Q2UH50	Low density lipoprotein receptor adap	--	19.677
Q5F353	GRASP55_65 domain-containing pro	GORASP2	46.409
F1NAJ2	Peptidylprolyl isomerase domain and	PPWD1	70.999

F1P568	Prospero homeobox protein 1 OS=Ga	PROX1	82.875
E1C6F0	U1 small nuclear ribonucleoprotein C	SNRPC	17.396
P09207	Tubulin beta-6 chain OS=Gallus gallu	--	50.153
Q5ZHT1	Acyl-CoA dehydrogenase family mem	ACAD11	87.001
P21869	Casein kinase II subunit alpha' OS=G	--	41.246
E1C897	CathepsinE-A-like protein OS=Gallus	CTSEAL	45.328
A0A1D5PPF8	Heat shock 70 kDa protein 4 OS=Gal	HSPA4	94.239
A0A3Q3AV17	Enoyl-CoA hydratase OS=Gallus gall	EHHADH	79.663
F1NKQ7	Fas associated via death domain(pred	FADD	22.819
A0A1D5PCU3	RBR-type E3 ubiquitin transferase OS	ARIH2	57.318
Q92178	Calpain-2 catalytic subunit OS=Gallu	CAPN2	79.228
A0A1D5PSL2	Afadin, adherens junction formation f	AFDN	214.84
E1BZ00	NEDD8 ultimate buster 1 (Fragment)	NUB1	70.35
A0A452J839	Tropomyosin alpha-1 chain OS=Gallu	TPM1	32.865
E1C6T4	Serine/threonine-protein kinase OS=G	PRKD3	100.39
A0A1D5P668	tRNA nucleotidyl transferase 1(predic	TRNT1	49.492
A0A1D5NW43	ESCRT-II complex subunit VPS25 O	VPS25	20.404
E1C4I9	Solute carrier family 25 member 21(p	SLC25A21	32.604
A0A1D5PW63	Glutathione transferase OS=Gallus ga	GSTT1	27.929
A0A1D5P9I0	Rho guanine nucleotide exchange fac	ARHGEF11	166.71
R4GM18	Kruppel like factor 9(predicted) OS=G	KLF9	25.661
F1NC81	P2X purinoceptor OS=Gallus gallus O	P2RX4	43.299
F1NC54	SH3 domain and tetratricopeptide rep	SH3TC1	152.43
F1NAB5	Retinol binding protein 5(predicted) O	RBP5	15.888
A0A1D5PFV1	I-kappa-B kinase OS=Gallus gallus O	IKBKB	86.738
A0A1D5NXF6	DMAP_binding domain-containing p	DIP2B	168.96
A0A3Q3AZN5	Glycine N-methyltransferase (Fragme	--	7.5958
A0A3Q3AFD8	Protein transport protein sec16 OS=G	SEC16A	235.78
H9L0U6	Proteasome subunit beta OS=Gallus g	PSMB4	28.112
A0A1D5PEL3	Septin-5 OS=Gallus gallus OX=9031	SEPT5	50.779
Q5ZHM2	IK cytokine(predicted) OS=Gallus ga	IK	64.924
Q5ZKP6	Adenosine deaminase OS=Gallus gal	ADA	40.678
F1NSV0	TLC domain-containing protein OS=G	TMEM56	29.669

F1NSS7	Brix domain-containing protein 2 OS	BRIX1	40.342
A0A1D5P782	Sec3-PIP2_bind domain-containing p	EXOC1	100.53
A0A1D5NZU8	Mitochondrial ribosomal protein S18.	MRPS18A	16.126
A0A1D5PMK3	PA domain-containing protein(predic	SPPL2A	56.843
A0A3Q2UHQ2	UBC core domain-containing protein	UBE2L6	17.558
A0A3Q2U4V3	Tryptophanyl-tRNA synthetase OS=C	LOC101750690	36.743
R4GM61	DAO domain-containing protein OS=	PIPOX	55.193
E1C0F3	Ras-related protein Rab-7a(predicted)	RAB7A	23.548
A0A1D5P2Y5	P-type domain-containing protein OS	GAAGSD	104.53
A0A1D5PT46	Protein kinase domain-containing pro	MAP3K20	51.209
A0A1D5NUW2	Phosvitin OS=Gallus gallus OX=903	VTG1	210.68
F1NKY1	Alpha-methylacyl-CoA racemase(pre	AMACR	42.931
P28318	Protein MRP-126 OS=Gallus gallus (	--	14.065
R4GJZ6	Z-linked golgi phosphoprotein 3(pred	GOLPH3	33.339
Q5ZKR9	ADP ribosylation factor 4(predicted)	ARF4	20.528
F1NLW3	Mitochondrial import receptor subuni	--	15.122
A0A3S5ZPM6	Thr_synth_N domain-containing prot	THNSL1	81.855
E1C832	SMC hinge domain-containing protei	SMCHD1	229.46
Q9W719	Hypoxanthine-guanine phosphoribosy	HPRT1	24.608
Q03696	Neuronal-glial cell adhesion molecule	--	136.57
A4PBT0	Metallothionein OS=Gallus gallus OX	MT1	6.2775
A0A3Q2U2Y6	Succinate dehydrogenase cytochrome	SDHC	15.361
A0A3Q2U8W6	Peptidase_M3 domain-containing pro	MIPEP	73.001
F1NZJ6	HECT-type E3 ubiquitin transferase (	UBE3C	124.1
A0A3Q2TX69	WD_REPEATS_REGION domain-co	SEC31B	132.49
F1NR86	TIP41-like protein OS=Gallus gallus	TIPRL	31.564
A0A1D5P6L7	U-box domain-containing protein OS	UBE4A	123.31
E1C245	Microtubule actin crosslinking factor	MACF1	829.56
E1C105	Ras-related protein Rab-3 OS=Gallus	RAB3C	26.029
F1NVA9	Mitochondrial ribosomal protein L38	MRPL38	44.429
P05122	Creatine kinase B-type OS=Gallus ga	CKB	42.87
A0A1D5PHF3	Myosin phosphatase Rho interacting	MPRIP	113.58
E1C7K8	NADH dehydrogenase [ubiquinone] 1	NDUFAF4	19.957

A0A3Q2U0G4	Aspartate dehydrogenase domain-con	--	26.99
F1NIY2	Xanthine dehydrogenase OS=Gallus g	XDH	149.54
F1NL83	Cytochrome c oxidase subunit VIIb(p	COX7B	8.9201
E1C0W9	ATP binding cassette subfamily B me	ABCB7	75.147
Q5ZKC9	14-3-3 protein zeta OS=Gallus gallus	YWHAZ	27.775
Q6QLQ5	Cathelicidin-1 OS=Gallus gallus OX=	CATHL1	16.072
E1C7T9	BAR/IMD domain containing adaptor	BAIAP2L1	56.594
A0A3Q2UMJ2	Collagen alpha-3(VI) chain OS=Gallu	COL6A3	326.58
A0A3Q3AS85	LRR binding FLII interacting protein	LRRFIP2	45.001
Q5ZK87	t-SNARE coiled-coil homology doma	STX16	37.281
A0A1D5PH43	YKT6 v-SNARE homolog(predicted)	YKT6	22.423
F1NYK6	Carbonyl reductase 4(predicted) OS=	CBR4	25.153
A0A1L1RZH6	Ketimine reductase mu-crystallin OS=	CRYM	26.789
F1N8Y5	GRASP55_65 domain-containing pro	GORASP1	45.419
A0A1L1RTN0	Tensin(predicted) OS=Gallus gallus (	TNS1	185.98
F1NXI9	5'-AMP-activated protein kinase gam	PRKAG2	50.848
A0A452J7S4	Cytosolic malate dehydrogenase OS=	MDH1	57.458
E1C7U8	Chromosome 3 open reading frame 3	C1H3ORF38	35.297
E1C2E5	NAD-capped RNA hydrolase NUDT1	NUDT12	52.183
A0A1L1RT70	DNA polymerase subunit gamma-1 C	POLG	135.44
F1NT44	Component 3 of promoter of RISC O	TSN	25.976
A0A1D5P1A9	IFI30 lysosomal thiol reductase(predi	IFI30	44.602
R4GKQ7	Aldolase_II domain-containing protei	ADD3	75.403
E1C8A1	Hepatocyte growth factor-like/macrop	MST1	79.379
A0A1D5P4F3	GB1/RHD3-type G domain-containin	GBP4L	70.278
E1BWX9	Imidazolonepropionate hydrolase OS=	UROC1	61.357
F1NE09	Cytoplasmic FMR1-interacting protei	CYFIP2	145.7
F1NPH9	OMPdecase OS=Gallus gallus OX=9	UMPS	51.898
Q5ZJV4	Mini-chromosome maintenance comp	MCMBP	71.923
A0A3Q2U105	P-type Cu(+) transporter OS=Gallus g	ATP7B	157.52
A0A3Q3B2T4	HBS1-like protein OS=Gallus gallus	HBS1L	71.095
A0A3Q3A497	RanBD1 domain-containing protein (	RANBP3	55.176
A0A1D5P813	Mitogen-activated protein kinase kina	MAP4K4	119.91

Q5ZI99	Actin-related protein 2/3 complex sub	ARPC1B	41.068
A0A1D5PZ84	Dehydrogenase/reductase 3(predicted	DHRS3	33.571
F1NNP8	Bms1-type G domain-containing prot	SMG6	91.857
A0A3Q3A9Y1	Putative transcription factor PML(pre	PMLL	44.327
P63247	Receptor of activated protein C kinase	RACK1	35.076
Q5ZJJ5	AKT-interacting protein OS=Gallus g	AKTIP	33.304
O57579	Aminopeptidase Ey OS=Gallus gallus	ANPEP	109.13
A0A3Q3AGY1	BAH domain-containing protein OS=	MTA2	14.445
A0A3Q2UAJ4	Laminin subunit gamma 1(predicted)	LAMC1	175.3
A0A3Q2UL56	ZnF_CDGSH domain-containing pro	CISD3	11.65
A0A1D5PXF3	Phosphoinositide 5-phosphatase OS=	INPP5B	99.118
F1NEQ4	A2M_recep domain-containing protei	--	29.328
A0A1D5P5R0	Heat shock protein HSP 90-alpha OS	HSP90AA1	83.875
E1C6Z3	BTB domain-containing protein OS=	KCTD21	29.308
E1C6A1	Proton-translocating NAD(P)(+) trans	NNT	113.78
A0A3Q2TYX8	Adaptor protein, phosphotyrosine inte	APPL2	69.012
A0A1D5PLE4	A-kinase anchoring protein 12(predic	AKAP12	207.9
A0A3Q2TYT7	BCL2 like 15(predicted) OS=Gallus g	BCL2L15	18.138
F1P2D2	3'(2'), 5'-bisphosphate nucleotidase 1(	BPNT1	32.914
P67883	60S ribosomal protein L30 OS=Gallu	RPL30	12.814
A0A3Q2UE20	HECT-type E3 ubiquitin transferase (	HERC2	517.72
Q98932	Ras-related protein Rab-5C OS=Gallu	RAB5C	23.553
F1NV37	Cytosolic 5-nucleotidase 1b-like(pred	NT5C1B	39.921
A0A3Q2UHP7	Trafficking protein particle complex 9	TRAPPC9	109.35
A0A1D5NT82	Zinc finger CCCH domain-containing	ZC3H14	78.302
E1BSW8	Translocase of inner mitochondrial m	TIMMDC1	25.99
A0A1D5NUT3	Myb_DNA-bind_5 domain-containin	FSBP	34.41
A0A1D5P9I3	Isocitrate dehydrogenase [NAD] subu	IDH3B	40.489
Q5ZLC7	Microtubule-associated protein RP/EI	MAPRE1	29.143
A0A1D5P3Y8	Copine 3(predicted) OS=Gallus gallu	CPNE3	59.666
Q90678	Platelet-activating factor acetylhydrol	PLA2G7	47.046
A0A3Q2TXG3	SON DNA binding protein(predicted)	SON	324.45
A0A3Q2TWU3	Phosphoinositide phospholipase C OS	PLCD1	87.25

F1NUB8	Protein kinase domain-containing pro	NRBP1	59.709
A0A3Q2TTG8	GB1/RHD3-type G domain-containin	GBP1	108
A0A1L1RKK6	Zinc finger protein 639(predicted) OS	ZNF639	55.505
A0A1L1S0D8	Polypyrimidine tract-binding protein	PTBP1	59.898
A0A1L1RLH3	CARD domain-containing protein OS	CARD11	18.353
A0A3Q2UFI0	RB binding protein 6, ubiquitin ligase	RBBP6	204.25
Q5F3L3	Protein FAM234B OS=Gallus gallus	FAM234B	67.646
E1C3N9	Golgi membrane protein 1(predicted)	GOLM1	43.042
A0A1D5P0R4	Mitochondrial 18 kDa protein OS=Ga	MTFP1	17.302
E1BXT1	Haloacid dehalogenase like hydrolase	CECR5	45.753
F1NPL1	Coiled-coil domain-containing protei	ZNF830	57.891
F1NCJ9	TRUD domain-containing protein OS	PUS7	73.991
A0A3Q2TT37	Fibronectin type-III domain-containin	FNDC3A	125.61
E1C502	Autophagy_act_C domain-containing	ATG3	35.669
A0A1D5PW21	Enhancer of mRNA-decapping protei	EDC4	148.95
F1NAC8	LRRcap domain-containing protein C	SNRPA1	28.414
A0A1L1RQK6	Deleted in malignant brain tumors 1(p	LOC426820	111.7
Q5F3X1	Protein kinase C OS=Gallus gallus O.	PRKCA	76.845
A0A1D5PNX4	RAB, member RAS oncogene family	RABL6	78.032
Q5ZMC1	RNA helicase OS=Gallus gallus OX=	RP11-529K1.3	53.901
Q90YH9	Testin OS=Gallus gallus OX=9031 G	TES	47.983
Q5ZJ02	DBIRD complex subunit ZNF326 OS	ZNF326	63.54
F1NWD1	UDP-glucose 4-epimerase OS=Gallus	GALE	38.311
Q5ZM56	Serine/threonine-protein phosphatase	PTPA	36.739
F1NSU6	PKS_ER domain-containing protein (	ZADH2	39.059
A0A3Q2TV61	Vacuolar fusion protein MON1 homo	MON1A	57.595
A0A1L1RPJ2	Protein kinase domain-containing pro	MAP2K1	43.635
E1BQT7	Rho guanine nucleotide exchange fac	ARHGEF16	77.733
A0A1D5PVA3	Ribosome production factor 2 homolo	RPF2	35.077
A0A1D5PS46	Nucleoporin 133(predicted) OS=Gall	NUP133	125.4
F1P4R3	Four and a half LIM domains 2(predi	FHL2	32.43
F1NMP3	Tubulin alpha chain OS=Gallus gallu	TUBA8B	49.785
P22451	60S ribosomal protein L5 OS=Gallus	RPL5	34.091

E1BS92	Pantothenate kinase 1(predicted) OS=	PANK1	41.692
A0A3Q3AMJ1	Gamma-tubulin complex component	TUBGCP2	96.451
F1N8U3	MFS domain-containing protein(pred	--	46.654
A0A3Q2UIP8	Inositol polyphosphate 1-phosphatase	INPP1	39.805
F1P3A5	Proto-oncogene c-Rel OS=Gallus gal	REL	66.876
F1NLG8	Translation initiation factor eIF-2B su	EIF2B1	33.593
A0A1D5P6T3	Tyrosine-protein kinase receptor OS=	INSR	149.74
A0A1D5P9V4	Solute carrier family 44 member 3(pr	SLC44A3	68.383
F1P226	SUMO-activating enzyme subunit 2 (	UBA2	71.078
A0A1D5P6N3	Fam20C domain-containing protein (	FAM20A	40.453
A0A1L1RM42	Chromosome 1 open reading frame 1(	C23H1ORF109	26.531
F1NPA2	PCI domain-containing protein OS=C	PSMD11	47.406
A0A3Q2U3E6	Interleukin enhancer binding factor 3-	ILF3	90.037
A0A1D5PIZ8	IQ motif containing GTPase activatin	IQGAP3	189.62
F1NCY7	Non-specific serine/threonine protein	MAPKAPK2	44.696
P11682	Apolipoprotein B (Fragment) OS=Ga	APOB	50.847
A0A3Q2UG39	Oxysterol-binding protein OS=Gallus	OSBPL2	55.08
A0A1L1RZ25	Leucine-rich repeat-containing protei	LRRC57	26.691
E1C2I6	Ligand dependent nuclear receptor in	LRIF1	95.49
H9L201	Lactamase_B domain-containing prot	PNKD	34.482
F1P5S5	Propionate--CoA ligase OS=Gallus g	ACSS3	73.435
A0A1D5PYI9	Elongator complex protein 6 OS=Gal	ELP6	29.377
A0A1D5PJL7	SpaA domain-containing protein OS=	PM5	132.88
F1NC47	BCL2 interacting protein 1(predicted)	BNIP1	26.103
A0A3Q2TZK1	Arginyl-tRNA--protein transferase 1 (	ATE1	59.965
F1NS60	MMS19 nucleotide excision repair pr	MMS19	117.1
E1BSA4	Pituitary tumor-transforming 1 protei	PTTG1IP	21.187
Q5F3D7	U3 small nucleolar RNA-associated p	UTP15	58.392
A0A1D5PBV0	Protein CASC3 OS=Gallus gallus OX	CASC3	72.535
E1BZD7	Transmembrane protein 254(predicte	TMEM254	14.731
A0A1D5PBU0	ITI_HC_C domain-containing protein	--	29.418
Q5ZIP7	Ras-related protein Rab-5A(predicted	RAB5A	23.541
R4GLB3	Proteasome subunit beta OS=Gallus g	PSMB2	22.708

F1NU06	Mitochondrial pyruvate carrier OS=G	MPC2	15.121
A0A3Q2U3U4	Syndetin OS=Gallus gallus OX=9031	VPS50	107.47
A0A1D5P9P3	Alpha-actinin-1 OS=Gallus gallus OX	ACTN1	105.69
A0A1D5PNM2	Sorbin and SH3 domain containing 1(	SORBS1	102.81
F1N8Q9	SOS Ras/Rho guanine nucleotide exc	SOS2	152.81
F1NX57	Glyoxylate and hydroxypyruvate redu	GRHPR	35.216
F1NCB2	Sodium/hydrogen exchanger OS=Gal	SLC9A6	93.171
Q5F3P1	Protein kinase domain-containing pro	NEK7	34.574
A0A3Q2UAH8	Dedicator of cytokinesis 1(predicted)	DOCK1	213.74
F1NSQ1	Aminoacyl tRNA synthase complex-i	AIMP2	34.521
A0A1D5PE85	Non-specific serine/threonine protein	SMG1	401.87
F1NW02	Alpha-amylase OS=Gallus gallus OX	AMY1A	57.79
A0A1D5P2W2	Extended synaptotagmin 2(predicted)	ESYT2	98.307
A0A1D5PEZ5	Nucleoporin NDC1 OS=Gallus gallus	NDC1	82.942
F1NW43	Pyruvate kinase OS=Gallus gallus OX	PKLR	57.873
P16924	Prolyl 4-hydroxylase subunit alpha-1	P4HA1	59.439
Q5ZIS0	Succinate dehydrogenase [ubiquinone	SDHD	16.435
F1P531	Methylcrotonoyl-CoA carboxylase 2(	MCCC2	60.678
A0A1D5P7W0	Nuclear RNA export factor 1(predicte	NXF1	69.446
E1C7M7	Catenin alpha 1(predicted) OS=Gallu	CTNNA1	100.04
E1BSA2	EBNA1 binding protein 2(predicted)	EBNA1BP2	55.979
E1C3D2	Septin OS=Gallus gallus OX=9031 G	SEPT2	41.638
F1NY55	LEM domain-containing protein OS=	ANKLE2	103.86
F1NGP4	tRNA-dihydrouridine(47) synthase [N	DUS3L	76.498
A0A3Q2U295	Telomerase RNA component interact	C19orf43	15.798
F1NRD4	C1q domain-containing protein OS=C	ADIPOQ	26.46
A0A1D5PKA3	FAD-binding PCMH-type domain-co	D2HGDH	59.254
Q5ZIG8	AAA domain-containing protein OS=	YME1L1	81.136
A0A1D5PJ79	Lysosome-associated membrane glyc	LAMP1	43.863
A0A1D5P6B8	Cell adhesion molecule 1(predicted) (	CADM1	45.001
E1C6J9	Thy-1 antigen OS=Gallus gallus OX=	THY1	18.173
F1NT80	AAA domain-containing protein OS=	ATAD1	41.075
F1NV47	EH domain containing 4(predicted) C	EHD4	61.285

A0A1L1RKJ1	Tapasin-related(predicted) OS=Gallus	LOC100857331	47.621
Q5ZML9	Actin-histidine N-methyltransferase C	SETD3	67.195
A0A1D5PTW8	Non-specific serine/threonine protein	RPS6KA3	80.517
A0A1L1RZN7	J domain-containing protein OS=Gall	DNAJB12	39.278
A0A1D5P386	Protein C19orf12(predicted) OS=Gal	C11H19orf12	14.82
A0A1D5PHU2	Syntaxin-binding protein 1 OS=Gallu	STXBP1	68.584
Q5ZJQ4	Non-specific serine/threonine protein	SGK3	56.26
P00337	L-lactate dehydrogenase B chain OS=	LDHB	36.318
A0A1D5P7Y8	AP complex subunit beta OS=Gallus	AP2B1	105.65
F1P1Q3	RING-type E3 ubiquitin transferase C	RMND5A	44.129
Q5ZL59	UBC core domain-containing protein	UBE2D3	16.687
A0A1D5PNM0	Complex I-B22 OS=Gallus gallus OX	NDUFB9	21.327
A0A1D5PTV1	Amine oxidase OS=Gallus gallus OX	AOC3	84.309
O73790	Heterochromatin-associated protein M	SERPINB10	47.383
F1P0I2	CAP10 domain-containing protein OS	KDELC2	47.865
F1NVD4	Adenylosuccinate synthetase isozyme	ADSSL1	50.097
F1NGM0	EH domain containing 3(predicted) C	EHD3	60.895
Q6R748	Syntaxin-binding protein 1 OS=Gallu	STXBP1	67.472
Q5ZHW4	Ras-related protein Rab-5B OS=Gallu	RAB5B	23.614
F1P220	Pre-mRNA-splicing factor ISY1(pred	ISY1	32.401
F1NGX1	Integrin alpha-V OS=Gallus gallus O	ITGAV	113.97
A0A3Q3AKJ5	Nidogen 2(predicted) OS=Gallus gall	--	20.043
E1BT66	TATA-box binding protein associated	TAF15	51.251
Q6ITC7	40S ribosomal protein S13 OS=Gallu	RPS13	17.222
A0A3Q2TS63	Alpha-1,3-mannosyl-glycoprotein 2-b	--	43.075
E1BVG7	PNPLA domain-containing protein O	PNPLA8	90.697
A0A3Q2TVX8	Ubiquitin-fold modifier-conjugating e	UFC1	24.32
F1NM13	Ankyrin repeat and MYND domain-c	ANKMY2	51.648
Q5ZKW2	Protein LZIC OS=Gallus gallus OX=9	LZIC	21.47
A0A1D5PX38	WD_REPEATS_REGION domain-co	WDR55	22.246
Q5ZJK4	Serine/threonine-protein kinase 4 OS=	STK4	55.336
E1BTI0	Glycerol-3-phosphate acyltransferase	GPAM	94.208
Q9YGL9	Sarcoplasmic/endoplasmic reticulum	ATP2A3	110.54

F1P4J0	Ribosomal protein S6 kinase OS=Gal	RPS6KB1	56.153
F1NW97	Tubulin alpha chain OS=Gallus gallu	TUBA4B	50.011
A0A1D5PAN0	LRRNT domain-containing protein O	PRELP	48.976
P02542	Desmin OS=Gallus gallus OX=9031	DES	53.512
A0A1D5P6G0	DEAQ-box RNA dependent ATPase	DQX1	82.167
E1BQT4	Transporter OS=Gallus gallus OX=90	SLC6A12	68.23
E1C296	LLGL domain-containing protein OS=	LLGL2	115.53
A0A3Q2TSK1	Mitochondrial import inner membran	TIMM50	26.284
P84169	26S proteasome non-ATPase regulato	PSMD13	42.751
A0A1D5PJP0	SH3 and PX domains 2B(predicted) O	SH3PXD2B	94.425
A0A3Q2UAX9	U3 small nucleolar ribonucleoprotein	MPHOSPH10	75.73
A0A3Q3A8Z1	Transmembrane protein 135 OS=Gall	TMEM135	72.802
A0A1D5P778	YTH domain-containing protein OS=	YTHDF2	62.505
A0A1D5PV23	RING-type domain-containing protei	PEX10	34.9
A0A3Q2U278	Structural maintenance of chromosom	SMC1A	25.792
F1N845	Protein C10 OS=Gallus gallus OX=90	C1H12ORF57	13.243
Q5ZKU1	DCN1-like protein 1 OS=Gallus gallu	DCUN1D1	30.109
F1NNU5	Copper transporter OS=Gallus gallus	SLC31A1	20.315
Q5ZKD1	Choline/ethanolaminephosphotransfe	CEPT1	46.097
A0A1D5PP99	Cordon-bleu WH2 repeat protein(pre	COBL	148.11
E1BTT8	L-lactate dehydrogenase OS=Gallus g	LDHA	36.502
E1BYJ6	Zinc finger BED-type containing 1(pr	DHRSX	37.265
Q5F4B3	Trimethyllysine dioxygenase, mitoch	TMLHE	48.656
F1NJU1	Rho guanine nucleotide exchange fac	ARHGEF18	130.65
E1C3X0	Trafficking protein particle complex s	TRAPPC6A	17.94
A0A1D5NYA0	NIPSNAP domain-containing protein	NIPSNAP1	32.584
E1C588	DH domain-containing protein OS=G	ARHGEF17	155.99
Q5F3L1	Ribosomal protein S6 kinase alpha-5	RPS6KA5	89.039
F1NGA6	Ectonucleoside triphosphate diphosph	ENTPD7	60.526
E1BYS4	Carboxypeptidase D OS=Gallus gallu	CPD	150.77
A0A3Q2TWF2	Late endosomal/lysosomal adaptor an	LAMTOR1	17.687
E1BVR7	PPM-type phosphatase domain-conta	PPM1G	57.7
P21760	Extracellular fatty acid-binding protei	EXFABP	20.201

A0A1D5PSK3	ER membrane protein complex subur	EMC1	109.8
A0A3Q3AJQ2	PNKD metallo-beta-lactamase domai	--	12.641
E1BTY5	Mitochondrial import inner membran	TIMM21	35.262
Q5ZKA2	Isoleucine--tRNA ligase, mitochondri	IARS2	112.01
A0A3Q2U6J3	Dysferlin(predicted) OS=Gallus gallu	DYSF	236.46
F1NC27	Ras-related protein Rab-21 OS=Gallu	RAB21	23.55
E1BQI6	alpha-1,2-Mannosidase OS=Gallus ga	EDEM3	99.263
Q5ZLL8	Glycerol-3-phosphate acyltransferase	GPAT3	50.112
A0A3Q2UG65	PWWP domain-containing protein OS	HDGFRP3	20.518
R4GK38	Sulfate anion transporter 1 OS=Gallu	SLC26A1	79.278
Q5F4B2	Switch-associated protein 70 OS=Gal	SWAP70	68.411
P52162	Protein max OS=Gallus gallus OX=9	MAX	18.184
A0A1D5PA60	Methionine--tRNA ligase, cytoplasmi	MARS	100.23
P36377	SPARC OS=Gallus gallus OX=9031	SPARC	34.06
R4GII9	Beta_elim_lyase domain-containing p	LOC101747660	45.482
F1NSI3	Cytochrome b5 heme-binding domair	CYB5B	16.185
A0A1D5P4W8	Stabilin 1(predicted) OS=Gallus gallu	STAB1	286.24
A0A3Q2UF15	Elongator complex protein 4 OS=Gal	ELP4	52.031
A0A1D5PXH4	Glutamine--fructose-6-phosphate trar	GFPT1	76.777
A0A3Q2TZX0	Conserved oligomeric Golgi complex	COG8	64.655
F1NU71	Mitochondrial import inner membran	TIMM44	51.264
P28701	Retinoic acid receptor RXR-gamma (	RXRG	51.233
F1NXD7	SEC23 interacting protein(predicted)	SEC23IP	108.38
A0A3Q2U340	Cytoplasmic linker associated protein	CLASP1	161.5
E1C455	Queuosine salvage protein OS=Gallus	CZH9orf64	39.061
F1N9K2	p52 pro-apototic protein(predicted) O	MRPS30	51.596
A0A3Q2UP12	Non-specific serine/threonine protein	KALRN	333.65
F1NM85	Mitochondrial import inner membran	TIMM17B	17.557
E3VVM5	Elongation of very long chain fatty ac	ELOVL1	33.89
F1P550	ATP-binding domain-containing prot	DPH6	78.321
A0A1D5PQW2	Phosphomannomutase OS=Gallus ga	PMM1	31.267
A0A1D5PZF2	Vacuolar protein sorting-associated p	VPS41	99.033
E1BYI2	Cystatin domain-containing protein C	CST7	16.854

F1NAM2	Lamin-B1 OS=Gallus gallus OX=903	LMNB1	73.638
A4F5B6	TAP binding protein OS=Gallus gallu	TPN	45.516
A0A1D5P8H6	Spermatogenesis associated serine ric	SPATS2	57.545
E1C3K7	PNN interacting serine and arginine r	PNISR	93.449
P18937	NADH-ubiquinone oxidoreductase ch	MT-ND2	38.333
A0A3Q2U7B8	Membrane-associated guanylate kinas	MAGI3	159.92
Q9I8D6	T-complex protein 1 subunit delta OS	tcp-1	57.736
A0A1D5PJM6	Nucleoporin 214(predicted) OS=Gall	NUP214	206.89
A0A1D5P5Y0	BCAS3 domain-containing protein O	BCAS3	85.765
R4GLV4	FHA domain-containing protein OS=	MKI67	284.2
F1NHD9	Exocyst complex component 3 OS=G	EXOC3	86.181
F1P4X5	DBC1 domain-containing protein OS	--	62.966
E1C866	AP-2 complex subunit alpha OS=Gal	AP2A2	104.15
F1NIP5	Ribose-phosphate diphosphokinase O	PRPS1L1	34.747
F1P1L1	Dipeptidyl peptidase 9(predicted) OS	DPP9	98.449
A0A1D5PKQ1	Queuine tRNA-ribosyltransferase cata	QTRT1	43.364
F1NM36	Phosphoglucomutase 5(predicted) OS	PGM5	62.703
A3F963	Muscleblind-like 2 isoform 4 OS=Ga	MBNL2	38.253
A0A3Q2U2L2	Vacuolar protein sorting-associated p	VPS16	91.359
Q90ZD4	Interferon regulatory factor 7 OS=Gal	IRF-3	54.44
F1NDT9	Acid phosphatase 2, lysosomal(predic	ACP2	48.193
A0A1D5PB96	DUF3504 domain-containing protein	QRICH1	83.909
E1C0E0	Trafficking protein particle complex s	TRAPPC13	44.365
Q5F356	Phosphatidylinositol 5-phosphate 4-k	PIP4K2A	46.091
F1NI07	Insulin like growth factor binding pro	--	66.794
Q9W6U9	Glycoprotein 130 OS=Gallus gallus O	gp130	102.49
E1BT61	NEDD8-activating enzyme E1 catalyt	UBA3	49.214
A0A1D5NUN3	Propionate--CoA ligase OS=Gallus ga	ACSS2	77.206
E1BYS6	Protein kinase C OS=Gallus gallus O	PKN2	117.31
Q5F464	Lipoma-preferred partner homolog O	LPP	65.139
A0A1D5PWG1	EWS RNA binding protein 1(predicte	EWSR1	76.895
A0A1D5P5B2	Butyryl-CoA dehydrogenase OS=Gal	IVD	45.845
Q5F408	Cysteine--tRNA ligase, cytoplasmic O	CARS1	85.8

E1C878	Glucosamine-6-phosphate isomerase	GNPDA2	31.108
F1NNX7	Ankyrin 3(predicted) OS=Gallus gallu	ANK3	213.46
F1NMS0	RAC-PK-alpha OS=Gallus gallus OX	AKT1	54.003
Q5ZMN3	Prohibitin-2 OS=Gallus gallus OX=9	PHB2	33.336
A0A1D5PRH9	MARVEL domain-containing protein	MAL2	19.863
Q5ZM58	Actin related protein 1A(predicted) O	ACTR1A	42.645
A0A1D5NYI5	CULLIN_2 domain-containing protei	CUL5	77.078
A0A1D5PCW2	Ras-specific guanine nucleotide-relea	RALGPS2	62.199
A0A3Q2UPN7	HECT domain-containing protein(pre	LOC424588	92.203
A0A3Q2UPM7	Peroxisomal biogenesis factor 11 beta	PEX11B	22.181
F1NQC6	Oxoglutarate dehydrogenase (succiny	DHTKD1	98.361
F1NV68	Importin subunit alpha OS=Gallus ga	KPNA3	57.928
F1NN63	Phosphoglucomutase 1(predicted) OS	PGM1	61.548
A0A1D5NWL9	17-beta-hydroxysteroid dehydrogenas	HSD17B4	79.292
F1NTA4	ATP-binding cassette sub-family B m	ABCB5	145.41
Q5ZLG9	GATOR complex protein WDR59 OS	WDR59	109.88
E1BYZ5	Pannexin_like domain-containing pro	LRRC8D	97.787
A0A1D5PRI4	MMS19 nucleotide excision repair pr	MMS19	111.45
A0A1D5PL02	DEP domain containing MTOR intera	DEPDC6	45.491
A0A1D5PMV3	Fam20C domain-containing protein C	--	27.85
F1NG21	SpoU_sub_bind domain-containing p	--	43.885
A0A1D5NZM5	Regulator of chromosome condensati	RCC2	56.649
A0A1L1RMP5	Poly(ADP-ribose) polymerase family	PARP9	86.625
A0A1D5NZM0	HABP4_PAI-RBP1 domain-containin	SERBP1	47.228
F1P3M9	Complement regulatory membrane pr	C4BPA	48.736
E1C1G0	Phosphatidylinositol-4-phosphate 3-k	PIK3C2A	191.53
A0A3Q2U2C6	CLPTM1 regulator of GABA type A	--	31.088
A0A1D5NU70	Family with sequence similarity 91 m	FAM91A1	94.425
Q5ZL42	Basic leucine zipper and W2 domain-	BZW2	47.842
Q5ZK33	Mitochondrial proton/calcium exchan	LETM1	85.848
Q7SZI5	Raftlin OS=Gallus gallus OX=9031 C	RFTN1	65.695
E1BUF6	Actin filament associated protein 1 lil	AFAP1L2	91.773
F1NPI6	UBA_e1_C domain-containing protei	UBA6	112.59

A0A1D5PY54	LanC like 2(predicted) OS=Gallus ga	LANCL2	51.615
A0A3S5ZP89	SWI/SNF related, matrix associated, a	SMARCA5	120.59
E1BV80	LIM zinc finger domain containing 2(	LIMS2	28.793
R4GKJ9	G_PROTEIN_RECEP_F1_2 domain-	FFAR4	39.395
Q9W6J2	Glutathione transferase OS=Gallus ga	GSTA4	25.44
P02112	Hemoglobin subunit beta OS=Gallus	HBB	16.466
A0A1D5PQ50	GlcNAc-1-P transferase OS=Gallus g	DPAGT1	45.855
A0A3Q2TVA4	Aldedh domain-containing protein OS	LOC107050351	35.176
F1P298	Amidohydrolase domain-containing p	AMDHD1	47.609
A0A1D5P0Y1	O-GlcNAc transferase subunit p110 O	OGT	115.51
E1C0R9	ER membrane protein complex subun	EMC7	26.68
A0A1D5PNU7	Neuroplastin(predicted) OS=Gallus g	NPTN	31.322
A0A1D5PRG9	Inositol hexakisphosphate and diphos	PPIP5K2	134.41
F1NUX4	Serine/threonine-protein kinase TOR	MTOR	286.72
P56517	Histone deacetylase 1 OS=Gallus gall	HDAC1	54.938
Q6PKI8	Phe-4-monooxygenase OS=Gallus ga	PAH	51.089
F1NPT0	Dynamin-1-like protein OS=Gallus ga	DNM1L	77.684
F1NKB6	ATP-dependent 6-phosphofructokinas	PFKL	85.145
A0A1D5PPD3	Ubiquitin carboxyl-terminal hydrolase	USP7	128.61
F1NCP7	RAD50 interactor 1(predicted) OS=G	RINT1	87.913
E1BX02	26S proteasome non-ATPase regulato	PSMD2	100.01
F6UK13	39S ribosomal protein L28, mitochon	MRPL28	29.583
F1NXR8	Luc7-like 3(predicted) OS=Gallus gal	LUC7L3	51.44
A0A3Q2U4F3	Ribonuclease P/MRP subunit p30(pre	RPP30	40.543
P14154	Gap junction alpha-1 protein OS=Gal	GJA1	43.125
A0A3Q2UFP4	Platelet and endothelial cell adhesion	PECAM1	80.213
F1N9S7	Annexin OS=Gallus gallus OX=9031	ANXA1	38.556
E1C1E9	Acyl-coenzyme A oxidase OS=Gallus	ACOX3	79.956
Q5F362	Cell cycle control protein 50A OS=G	TMEM30A	41.448
A0A3Q2U9B9	LisH domain-containing protein OS=	KIAA1468	146.71
A0A1D5PVC3	6-O-methylguanine-DNA methyltrans	MGMT	21.708
E1BYE0	Sulfotransferase OS=Gallus gallus OX	LOC415852	33.189
F1NZ68	NIPSNAP domain-containing protein	NIPSNAP2	38.173

A0A1D5PJI9	Vacuolar protein sorting 13 homolog	VPS13A	360.66
A0A1D5NTN8	Hydrolase_4 domain-containing prote	MGLL	33.338
A0A1D5P356	ATP binding cassette subfamily B me	ABCB8	78.811
F1NCY5	E3 UFM1-protein ligase 1 OS=Gallus	UFL1	89.261
A0A1D5PIF5	E3 ubiquitin-protein ligase OS=Gallu	NEDD4	104.36
A0A3Q2U809	Solute carrier family 12 member 4(pr	SLC12A4	117.62
Q6B842	Carnitine O-palmitoyltransferase OS=	CPT1A	87.884
A0A3Q2UEG5	B30.2/SPRY domain-containing prote	--	25.69
A0A1D5PB77	Bile acid-CoA:amino acid N-acyltran	BAAT	44.307
A0A3Q3A7P6	40S ribosomal protein S15a OS=Gall	RPS15A	14.855
F1NYT7	Prenylcys_lyase domain-containing p	PCYOX1	56.066
Q5ZJI7	Actin-related protein 2/3 complex sub	ARPC1A	41.689
Q5ZME8	WD40 repeat-containing protein SMU	SMU1	57.497
Q91348	6-phosphofructo-2-kinase/fructose-2,	--	54.403
E1BT00	V-type proton ATPase subunit D OS=	ATP6V1D	25.653
F1NHX3	LEM domain-containing protein OS=	LEMD3	97.121
F1NMA2	Sulfotransferase OS=Gallus gallus OX	SULT1E1	34.36
A0A1D5PJF2	Acyl-CoA synthetase medium chain f	ACSM5	65.077
E1C266	Tyrosine--tRNA ligase OS=Gallus ga	YARS2	53.54
A0A1D5PP61	Coiled-coil domain containing 90B(p	CCDC90B	27.853
A0A1D5NY37	GTPase KRas(predicted) OS=Gallus	KRAS	21.452
Q5ZM96	Queuine tRNA-ribosyltransferase acc	QTRT2	47.064
Q5ZLB0	RNA helicase OS=Gallus gallus OX=	DDX47	50.244
A0A1D5P0D0	Alkyl transferase OS=Gallus gallus C	--	38.82
A0A1D5P8Q3	Papilin, proteoglycan like sulfated gly	PAPLN	95.042
A0A1D6UPS7	AB hydrolase-1 domain-containing pr	EPHX2	66.831
A0A1D5PFY6	HTH La-type RNA-binding domain-c	LARP1	118.61
F1NY18	Unc-13 homolog B(predicted) OS=Ga	UNC13B	179.13
A0A1D5P6W8	Lysine-specific histone demethylase (	KDM1A	90.539
F1NZB4	Nucleoporin 210(predicted) OS=Gall	NUP210	212.03
E1BSI6	L51_S25_CI-B8 domain-containing p	MRPS25	20.074
A0A3Q3ARC0	Oxoglutarate dehydrogenase (succiny	LOC107054982	164.1
F1NCA1	Cyclin K(predicted) OS=Gallus gallu	CCNK	65.559

E1C2S9	R3H domain containing 1(predicted)	R3HDM1	98.64
A0A1D5PKH0	Receptor protein-tyrosine kinase OS=	EGFR	136.47
F1P3T4	GOLD domain-containing protein OS	TMED3	24.138
E1C3L4	E3 ubiquitin-protein ligase OS=Gallu	WWP2	104.09
Q5F3J5	Proteasome activator complex subuni	PSME3	29.481
A0A1D5P722	von Willebrand factor OS=Gallus gal	VWF	303.13
A0A1D5NX91	IRF-2BP1_2 domain-containing prote	C5H14orf4	76.513
A0A1D5P482	Integrator complex subunit 8(predicte	INTS8	112.94
Q5ZKP4	Trafficking protein particle complex s	TRAPPC2	16.586
A0A1D5PPA8	AdoHcyase_NAD domain-containing	AHCY	47.735
Q5QHR9	Tumor necrosis factor alpha convertir	ADAM17	93.595
P09244	Tubulin beta-7 chain OS=Gallus gallu	--	49.67
Q92074	Beta-1,4-galactosyltransferase OS=Ga	CKI	40.928
E1BS63	Multidrug resistance protein 1 OS=Ga	ABCB1	141.98
A0A3Q2UEG2	Catenin delta 1(predicted) OS=Gallus	CTNND1	104
A0A1D5P0B9	Exosome component 7(predicted) OS	EXOSC7	31.645
A0A3Q2UEE3	Isochorismatase domain-containing p	LOC107050412	50.112
A0A3Q2U8X6	Dynein light chain Tctex-type 1(predi	DYNLT1	12.408
A0A1D5P179	Complex I-B16.6 OS=Gallus gallus C	NDUFA13	16.777
E1BRR6	Scavenger receptor class B member 2	SCARB2	53.906
E1BSH7	Dynamin-type G domain-containing p	MFN2	86.735
A0A1D5PPQ9	KIAA1324 like(predicted) OS=Gallus	KIAA1324L	99.444
F1NC48	Mitochondrial nucleoid factor 1 OS=(	C26H6ORF125	15.069
A0A3Q3A829	MHC_II_alpha domain-containing pr	--	10.853
P01994	Hemoglobin subunit alpha-A OS=Gal	HBAA	15.429
F1NG23	Nucleoredoxin (Fragment)(predicted)	--	14.804
F1NTR0	Malonyl-CoA decarboxylase(predicte	MLYCD	56.178
Q90997	Transferrin receptor protein 1 OS=Ga	TFRC	85.658
F1NLQ2	N-acylneuraminate cytidylyltransferas	CMAS	46.247
Q5ZJW2	NADH dehydrogenase [ubiquinone] 1	NDUFB8	21.945
A0A1D5PAM1	Checkpoint and tumor suppressor pro	CHEK2	59.043
A0A1D5P8M4	Pancreatic progenitor cell differentiat	C2H8ORF22	9.2032
A0A1D5PZA1	ATP-dependent helicase ATRX OS=(	ATRX	269.91

F1NHD8	Programmed cell death 6(predicted) (	PDCD6	20.721
F1NT48	Protein transport protein sec16 OS=G	SEC16B	107.99
Q5ZJX6	ATP binding cassette subfamily E me	ABCE1	67.355
E1C9H5	Aminotran_1_2 domain-containing pı	AADAT	47.634
F1NPN3	SERPIN domain-containing protein (	SERPINA10	51.261
A0A1D5P5T8	Poly(A) polymerase OS=Gallus gallu	PAPOLB	82.897
A0A3S5ZPJ0	Serine/threonine-protein phosphatase	PPP6C	62.241
F1NWE2	Integrin alpha FG-GAP repeat contai	ITFG1	61.607
F1NY25	Citrate hydro-lyase OS=Gallus gallus	ACO1	98.087
A0A1D5PJH1	Adaptin_N domain-containing protei	--	27.744
F1NZA1	Tubulin tyrosine ligase like 12(predic	TTLL12	72.813
E1BY22	Methylmalonyl-CoA isomerase OS=(	MUT	82.764
F1P2T2	NADPH--cytochrome P450 reductase	POR	76.968
E1C0C3	J domain-containing protein OS=Gall	SEC63	87.525
F1NPS0	Nucleoporin 155(predicted) OS=Gall	NUP155	154.14
F1P458	Sulfhydryl oxidase OS=Gallus gallus	QSOX2	76.078
P12275	Arylamine N-acetyltransferase, liver i	--	32.914
A0A3Q2TWK3	V-set and immunoglobulin domain co	VSIG4	41.182
F1N826	Interleukin-1 receptor-associated kina	IRAK2	76.691
E1C8R7	5'-3' exoribonuclease 1(predicted) OS	XRN1	200.96
E1BW15	RNA helicase OS=Gallus gallus OX=	DDX21	75.196
A0A1D5NZ06	40S ribosomal protein S27 OS=Gallu	RPS27	9.461
A0A1D5PXD0	Tr-type G domain-containing protein	EEFSEC	60.714
A0A3Q3AUC8	Tetratricopeptide repeat and ankyrin ı	TRANK1	254
F1NQ45	WD_REPEATS_REGION domain-co	CDC40	65.697
A0A1D5P0T9	Fatty acid hydroxylase domain-contai	SC5D	33.924
E1BZ86	Calcium/calmodulin-dependent prote	CAMK2G	51.549
E1BV11	Septin OS=Gallus gallus OX=9031 G	SEPT14	49.496
F1NIS2	Transmembrane protein 119 OS=Gall	--	25.492
A0A452J7V2	Ras-related protein Rab-2A OS=Gallı	RAB2A	22.651
E1C080	RAB8B, member RAS oncogene fam	RAB8B	22.944
F1NYJ6	WD_REPEATS_REGION domain-co	GEMIN5	170.54
E1BX04	Coiled-coil domain-containing protei	PPP1R21	88.851

E1C840	Stonin-2 OS=Gallus gallus OX=9031	STON2	99.406
F1P5E2	WD repeat and FYVE domain-contai	WDFY2	45.014
E1BTB7	C1q domain-containing protein OS=(	--	66.759
E1BSA7	SEC24 homolog A, COPII coat comp	SEC24A	120.02
A0A1D5PB75	Solute carrier family 12 member 9(pr	SLC12A9	97.855
F1NYG7	2-hydroxyacyl-CoA lyase 1(predicted	HACL1	62.008
Q5ZMQ2	Actin, cytoplasmic 2 OS=Gallus gallu	ACTG1	41.792
A0A1D5NWK1	Rhophilin Rho GTPase binding prote	RHPN2	59.73
E1C0T6	PH domain-containing protein OS=G	FERMT2	80.663
A0A1D5PB71	Anaphase-promoting complex subuni	ANAPC4	94.593
A0A1D5PIJ9	Peptide-methionine (R)-S-oxide redu	MSRB3	19.709
F1NHQ3	Ethanolaminephosphotransferase 1 (F	SELENOI	43.635
A0A2Z5EM90	NADB-LER5 OS=Gallus gallus OX=	NADB-LER5	27.434
A0A1D5PSP4	Nicotinate phosphoribosyltransferase	NAPRT	57.272
F1NN49	Delta-aminolevulinic acid dehydratas	ALAD	55.253
Q5ZJN2	Ras-related protein Rab-11A OS=Gal	RAB11A	24.438
A0A1D5NV93	Pyrroline-5-carboxylate reductase OS	PYCRL	28.481
Q5ZJN4	Ubiquitin carboxyl-terminal hydrolas	USP10	86.555
E1C525	HRDC domain-containing protein OS	EXOSC10	103.9
A0A1L1RKI2	SEH1 like nucleoporin(predicted) OS	SEH1L	39.784
Q5ZIZ3	Ubiquinol-cytochrome c reductase co	UQCC1	34.343
Q90623	Protein phosphatase 1 regulatory subu	PPP1R12A	111.6
F1NZH6	Peroxisomal trans-2-enoyl-CoA redu	PECR	31.848
F1P0P4	Signal recognition particle subunit SF	SRP68	68.831
F1NBV3	DAO domain-containing protein OS=	L2HGDH	48.609
A0A1L1RMV7	Retinal dehydrogenase 2 OS=Gallus g	ALDH1A2	54.774
Q5ZJQ6	Aspartate--tRNA ligase, cytoplasmic	DARS	57.189
A0A1D5NUQ6	Matrix AAA peptidase interacting pro	C7H2ORF47	27.837
R4GFF0	Galactose-1-phosphate uridylyltransfe	GALT	28.411
E1BWA9	Protein FAM3C OS=Gallus gallus O	FAM3C	24.81
E1C345	Transmembrane 4 L six family memb	TM4SF4	21.217
F1P591	Conserved oligomeric Golgi complex	COG6	67.074
A0A1D5PRM0	Canalicular multispecific organic anio	ABCC3	159.83

E1C0V1	Glucosamine 6-phosphate N-acetyltra	GNPNAT1	21.378
E1BQB7	Sphingomyelin phosphodiesterase 4(p	SMPD4	94.796
A0A1D5PIQ3	Flavin-containing monooxygenase OS	--	14.425
A0A1D5PH35	Cleavage and polyadenylation specifi	CPSF3	72.196
P79784	Ectonucleoside triphosphate diphosph	ENTPD2	54.533
Q5ZM33	Heterochromatin protein 1-binding pr	HP1BP3	61.851
Q5F471	Serine/threonine-protein phosphatase	PPP6R3	98.309
F1NDZ9	Calcium homeostasis endoplasmic ret	CHERP	114.5
E1BWX1	Annexin OS=Gallus gallus OX=9031	ANXA6	86.382
A0A1D5P3B1	60S ribosomal protein L11 OS=Gallu	RPL11	20.233
A0A1D5PF61	RIC8 guanine nucleotide exchange fa	RIC8B	60.535
Q5ZLD4	Transmembrane protein 11, mitochon	TMEM11	21.734
A0A1D5NU19	WD repeat domain 11(predicted) OS=	WDR11	136.09
E1BTG1	60S ribosomal protein L12 OS=Gallu	RPL12	17.737
E1BVF8	V-type proton ATPase subunit OS=G	ATP6V0D1	40.321
P42558	GTP-binding nuclear protein Ran OS	RAN	24.427
F1NW83	MOB kinase activator 1B OS=Gallus	--	35.225
A0A1D5NXC6	Plug_translocon domain-containing p	SEC61A2	52.229
Q98TX3	Programmed cell death protein 4 OS=	PDCD4	51.707
A0A1D5NWQ3	SRP54 domain-containing protein OS	SRPRA	71.197
F1NKQ0	F-box domain-containing protein OS=	FBXL17	49.48
E1C988	Oxysterol-binding protein OS=Gallus	OSBPL1A	106.31
A0A3Q2UDT6	Aminopeptidase puromycin sensitive(	NPEPPS	124.22
A0A1D5PDV6	Ribosomal protein S19(predicted) OS	RPS19	16.074
A0A3Q2TSD4	C-type lectin domain-containing prote	FCER2	29.78
A0A3Q2TWJ0	Protein-ribulosamine 3-kinase OS=Ga	FN3KRP	31.577
O93327	Core histone macro-H2A.1 OS=Gallu	MACROH2A1	39.656
E1BSR5	PWI domain-containing protein OS=(	--	41.138
A0A3Q2U7R0	SON DNA binding protein(predicted)	SON	309.66
A0A3Q2U0J5	Retinoblastoma-associated protein OS	RB1	109.98
A0A1D5PLZ3	NADP-retinol dehydrogenase OS=Ga	RDH10	37.919
A0A1D5PN56	Torsin-1A-interacting protein 1 (Frag	TOR1AIP1	23.958
A0A1D5PW68	Eukaryotic translation initiation facto	EIF3B	92.309

A0A3Q2U7I2	Tetratricopeptide repeat protein 39B (	TTC39B	58.403
Q90WD0	Actin-related protein 3 OS=Gallus ga	ACTR3	47.421
A0A3Q3ATV6	Inositol 1,4,5-trisphosphate receptor t	ITPR2	297.94
Q5ZL97	ELMO domain-containing protein OS	ELMO1	83.79
A0A3Q2U8Y5	DUF4430 domain-containing protein	TCN2	59.98
Q5ZI57	Trafficking protein particle complex s	TRAPPC3	20.276
Q5ZHN4	Protein XRP2 OS=Gallus gallus OX=	RP2	39.722
A0A1D5NY28	DNA-directed RNA polymerase subu	POLR1A	175.96
F1N891	Protein kinase domain-containing pro	PEAK1	192.54
F1NGR3	NADB-LER5(predicted) OS=Gallus g	LOC415664	27.699
Q9YGW6	Ezrin OS=Gallus gallus OX=9031 GN	EZR	69.366
A0A1D5NY15	Agrin OS=Gallus gallus OX=9031 G	AGRN	222.31
A0A1D5P0A1	UTP--glucose-1-phosphate uridylyltra	UGP2	56.834
A0A1D5PSZ3	SET binding factor 1(predicted) OS=(	SBF1	213.24
F1P0W8	Glycerol-3-phosphate dehydrogenase	GPD1L	38.589
A0A1L1RRI7	Alpha-1,3-glucosyltransferase OS=Ga	ALG8	60.05
A0A1D5NT70	Glutathione transferase OS=Gallus ga	LOC395611	25.346
F1N863	JmjC domain-containing protein OS=	HIF1AN	39.701
F1NM39	Histone deacetylase 2 OS=Gallus gall	HDAC2	55.304
P00523	Proto-oncogene tyrosine-protein kinas	SRC	60.009
A0A1D5PB38	Hephaestin(predicted) OS=Gallus gal	HEPH	131.03
A0A1L1RKU5	Solute carrier family 12 member 7(pr	SLC12A7	120.3
F1NU16	Splicing factor 3a subunit 1(predicted	SF3A1	88.486
F1NLG7	UTP6 small subunit processome com	UTP6	70.856
A0A1D5PI59	Platelet-activating factor acetylhydrol	PAFAH2	42.307
E1BZL0	Ubiquitinyl hydrolase 1 OS=Gallus ga	USP24	292.26
F1NVD2	Fibronectin type-II domain-containing	IGF2R	281.88
R4GH80	NF-kappa-B inhibitor alpha OS=Gallu	NFKBIA	35.372
E1C5W8	E3 ubiquitin-protein ligase RNF123(p	RNF123	147.57
E1C2F9	IQ motif containing GTPase activatin	IQGAP2	190.44
A0A3Q2UGF2	BCL2/adenovirus E1B 19 kDa protein	BNIP3L	36.646
A0A3Q2U4J3	Sema domain-containing protein OS=	PLXNB2	202.21
F1P451	AMP-binding domain-containing pro	ACSL3	79.33

E1BTF5	DAGKc domain-containing protein C	AGK	41.132
A0A3Q2UBG0	N-acetylglucosamine kinase(predicte	LOC107051359	21.829
E1C0K5	Milk fat globule-EGF factor 8 protein	MFGE8	53.146
A0A1L1RR20	Ribonuclease P/MRP subunit p40(pre	RPP40	38.851
P14092	ATP synthase subunit a OS=Gallus g	MT-ATP6	24.797
A0A3Q2U9X6	PX domain-containing protein OS=G	SNX8	47.22
A0A1D5PZT0	alpha-1,2-Mannosidase OS=Gallus ga	MAN1A2	73.074
A0A3Q2U3M3	DEC-205 protein(predicted) OS=Gall	LY75	191.65
Q2IAL7	Cathelicidin-2 OS=Gallus gallus OX=	CATHL2	16.974
E1BQN6	Protein kinase C OS=Gallus gallus O	PRKCZ	68.248
A0A1D5PSF4	BAAT_C domain-containing protein	ACOT4	24.499
E1BXK3	Glycogen [starch] synthase OS=Gallu	GYS2	81.049
A0A3Q2TXI6	AIG1-type G domain-containing prot	GIMAP1-GIMAP	28.639
F1NSC1	ATP synthase subunit b OS=Gallus g	ATP5PB	51.109
A0A1D5PL06	RAP domain-containing protein OS=	TBRG4	71.396
A0A1D5PA50	VASt domain-containing protein OS=	GRAMD1C	52.066
F1NJU5	Complement C8 alpha chain(predicte	C8A	67.724
A0A1D5PMH0	Vacuolar protein sorting 45 homolog(	VPS45	45.062
E1BZ19	DNA topoisomerase 2 OS=Gallus gal	TOP2B	179.46
R9PXP1	Tudor domain-containing protein 7 O	TDRD7	123.03
F1NJ23	Laminin subunit beta-1 OS=Gallus ga	LAMB1	196.89
E1C1P7	HERV-H LTR-associating 2(predicte	HHLA2	41.538
A0A1L1RXR5	Complex III subunit 8 OS=Gallus gal	UQCRQ	9.6178
E1BUH7	Transmembrane protein 43(predicted)	TMEM43	44.867
Q5F3Z2	CAP10 domain-containing protein OS	POGLUT1	45.782
A0A1D5P1W7	Importin N-terminal domain-containi	KPNB1	97.332
F1NRW5	Nuclear pore complex protein Nup85	NUP85	94.948
E1BZ62	WD_REPEATS_REGION domain-co	LRWD1	71.723
A0A1D5PAZ7	Aminopeptidase OS=Gallus gallus O	ENPEP	107.92
F1NNU7	Acyl-CoA thioesterase 9(predicted) C	ACOT9	46.734
A0A3Q2UAT0	Rab proteins geranylgeranyltransferas	CHM	73.102
F1NKZ2	MAP7 domain containing 2(predicted	MAP7D2	77.301
Q6B344	Annexin OS=Gallus gallus OX=9031	ANXA6	75.226

A0A1D5P0E2	Protein transport protein SEC23 OS=	SEC23A	86.186
Q5F3L4	Protein DENND6A OS=Gallus gallus	DENND6A	67.05
F1NUS2	Prolyl endopeptidase OS=Gallus gallu	PREP	95.025
A0A1D5PLE1	ATP binding cassette subfamily C me	ABCC6	169.5
E1BWN6	Coronin OS=Gallus gallus OX=9031	CORO2A	58.935
A0A1D5PY15	Coronin OS=Gallus gallus OX=9031	LOC107056441	58.502
F1P0Z4	L-serine ammonia-lyase OS=Gallus g	SDSL	46.548
Q5ZKD0	Lysophosphatidylglycerol acyltransfe	LPGAT1	42.634
Q5ZIG3	Solute carrier family 25 member 46 C	SLC25A46	44.641
E1BX21	HDAC_interact domain-containing pr	SIN3A	145.59
F1NCK8	Glycophorin C OS=Gallus gallus OX	GYPC	10.071
A0A1D5P6F4	IgGFc-binding protein (Fragment)(pr	LOC429249	289.27
Q5ZIH5	Suppressor of IKBKE 1(predicted) O	SIKE1	18.235
E1C6K1	t-SNARE coiled-coil homology doma	STX19	34.237
E1BZ79	Mevalonate kinase OS=Gallus gallus	MVK	41.882
F1NUQ5	Eukaryotic translation initiation facto	EIF4E2	27.107
A0A1D5NWK3	Squalene monooxygenase OS=Gallus	SQLE	63.889
A0A1D5PZL4	HECT-type E3 ubiquitin transferase (	NEDD4L	111.79
A0A3Q2TT14	Ras interacting protein 1(predicted) C	LOC107050411	111.13
Q5ZKQ5	Protein GOLM2 OS=Gallus gallus O	GOLM2	42.497
E1C7K1	Family with sequence similarity 98 m	FAM98B	47.601
F1P4F3	Lymphocyte antigen 86 OS=Gallus ga	LY86	18.015
E1C378	Histamine N-methyltransferase(predi	HNMT	32.887
A0A1D5P9F9	Complement C3(predicted) OS=Gallu	C3	198.98
F1NP58	Pescadillo homolog OS=Gallus gallus	PES1	68.3
F1NPL8	Glycogen synthase kinase-3 beta OS=	GSK3A	44.71
Q5ZKI3	NOT2_3_5 domain-containing protei	CNOT2	57.374
E1BVP1	Eukaryotic translation initiation facto	EIF5B	137.75
A0A1D5P9P4	BTB domain-containing protein OS=	KCTD12	35.196
A0A1D5NZG5	TRMT1-like protein OS=Gallus gallu	TRMT1L	85.366
A0A3Q2TZT0	Inositol-3-phosphate synthase OS=Ga	ISYNA1	60.599
Q56I99	Mothers against decapentaplegic hom	SMAD5	52.228
A0A1D5PVT8	NAD(+) kinase OS=Gallus gallus OX	NADK	48.999

P21642	Phosphoenolpyruvate carboxykinase [	PCK2	71.106
A0A1D5NVJ9	Transporter associated with antigen p	--	36.535
P42324	Hippocalcin-like protein 1 OS=Gallus	HPCAL1	22.269
A0A1D5NV94	AP-2 complex subunit alpha OS=Gal	AP2A2	107.03
E1C7J9	RNA cytidine acetyltransferase OS=G	NAT10	114.51
F1NGR5	ADP dependent glucokinase(predicte	ADPGK	52.965
A0A3S5ZPL8	SPATS2-like(predicted) OS=Gallus g	SPATS2L	61.591
A0A1D5PAE0	ZP domain-containing protein OS=Ga	ENG	78.278
A0A3Q2U8P4	GB1/RHD3-type G domain-containin	GBP	65.139
E1C3Y2	Homogentisate 1,2-dioxygenase OS=	HGD	49.465
E1C7Y1	Component of oligomeric Golgi comp	COG2	83.841
F1NE72	Lysine--tRNA ligase OS=Gallus gallu	KARS	67.864
E1C3U6	COP9 signalosome complex subunit 2	COPS2	51.596
F1NUL3	ATPase H(+)-transporting lysosomal	ATP6AP2	39.071
A0A1D5PC23	Transaldolase OS=Gallus gallus OX=	TALDO1	37.582
E1C6W8	Myotubularin phosphatase domain-co	MTMR9LP	62.363
A0A1D5PZJ4	CCT-theta OS=Gallus gallus OX=903	CCT8	60.254
O73775	Fibulin-1 OS=Gallus gallus OX=9031	FBLN1	78.137
A0A1L1RPT9	Ubiquitin carboxyl-terminal hydrolase	FAM188A	50.238
Q08393	Glutathione S-transferase OS=Gallus	--	25.413
F1NHD1	Staufen double-stranded RNA binding	STAU1	75.433
F1NFL6	Phosvitin OS=Gallus gallus OX=9031	VTG2	205.15
F1NY29	Electron transfer flavoprotein-ubiquin	ETFDH	68.714
F1NVZ1	WD repeat and FYVE domain-contai	WDFY1	46.45
A0A3Q2U1Z0	Ribosome biogenesis protein BOP1 O	BOP1	81.255
F1NER5	Glyco_18 domain-containing protein	CHID1	45.026
E1BZT9	Acetyl-coenzyme A synthetase OS=G	ACSS1B	76.389
A0A3Q2U8R2	FERM, ARHGEF and pleckstrin dom	FARP1	115.77
E1BTY4	Sorting nexin OS=Gallus gallus OX=	SNX9	67.093
A0A3Q3AVD8	Rab-GAP TBC domain-containing pr	EVI5	90.6
P13914	Arylamine N-acetyltransferase, pineal	--	33.663
A0A3Q2U9K8	Phosphoenolpyruvate carboxykinase (	PCK2	45.096
A0A1D5NTC9	PNPLA7(predicted) OS=Gallus gallu	PNPLA7	141.77

A0A1D5PG82	Golgi integral membrane protein 4(pr	GOLIM4	75.939
F1NG10	Actin related protein 10(predicted) OS	ACTR10	46.267
F1N8T0	Nuclear export mediator factor(predic	NEMF	121.72
A0A1I7Q3Y8	Sodium/potassium-transporting ATPa	ATP1B3	30.366
A0A3Q2U3Y3	Calponin OS=Gallus gallus OX=9031	CNN1	28.407
A0A1L1RQR8	Chromosome 22 open reading frame 3	C22orf39	12.467
E1C3Q6	Peptidase_M28 domain-containing pr	ERMP1	77.078
Q5ZIX3	Chromatin modification-related prote	MEAF6	20.458
A0A1D5PTM0	UBC core domain-containing protein	UBE2J1	32.985
Q5ZMT4	GOLD domain-containing protein OS	TMED5	25.674
E1BT18	AMP-binding domain-containing pro	SLC27A4	89.291
Q5ZLM5	Transmembrane 9 superfamily memb	TM9SF2L	74.082
P20135	Glutathione S-transferase theta-1 OS=	GSTT1	29.822
F1NCV7	S-methyl-5'-thioadenosine phosphory	MTAP	30.848
A0A1D5P570	Acyl-CoA dehydrogenase family mer	ACAD11	87.123
Q5I9D4	Galectin OS=Gallus gallus OX=9031	LGALS8	35.587
A0A1D5PLN6	MIF4G domain-containing protein OS	PAIP1	42.227
A0A3Q2U9X9	Delta-sagatoxin-srs1a-like isoform x1	LOC426064	22.896
F1P4F4	Signal sequence receptor subunit alph	SSR1	32.172
A0A1D5PE83	Microsomal prostaglandin E synthase	PTGES2	39.617
E1C1X1	Transmembrane protein 126A(predict	TMEM126A	23.254
A0A1D5PXD8	Argonaute RISC component 1(predic	AGO1	97.231
A0A3Q2U8D9	J domain-containing protein OS=Gall	DNAJC11	59.053
E1C218	Dedicator of cytokinesis 7(predicted)	DOCK7	232
Q9DF58	Integrin-linked protein kinase OS=Ga	ILK	51.518
A0A1D5PK79	Neuroblastoma amplified sequence(p	NBAS	255.06
E1C4J9	SH3 and PX domains 2B(predicted) O	SH3PXD2B	96.438
A0A3Q2TZW5	BTB domain-containing protein(pred	--	7.9531
A0A3Q2UDP3	Dedicator of cytokinesis 10(predicted	DOCK10	241.28
F1N9S3	Protein FAM98A(predicted) OS=Gal	FAM98A	58.636
E1C6S5	Canalicular multispecific organic anio	ABCC2	172.17
A0A3Q2UJU4	Solute carrier family 25 member 24(p	SLC25A24	48.671
Q5ZLZ6	Monocarboxylate transporter 1 OS=G	SLC16A1	54.306

R4GHJ7	Phosphorylase b kinase regulatory sul	PHKB	122.19
A0A1L1RQ91	Glucose-6-phosphate isomerase OS=(	GPI	62.13
F1NNN0	F-box domain-containing protein OS=	FBXL8	43.722
P34065	Proteasome subunit beta type-5 (Frag	PSMB5	27.045
E1BZM5	Component of oligomeric Golgi comp	COG7	86.647
A0A3Q3AW40	TPR_REGION domain-containing pr	UNC45A	94.301
A0A1D5P4E9	Niban apoptosis regulator 1(predicted	FAM129A	108.48
F1N8N7	Integrin beta OS=Gallus gallus OX=9	ITGB1	88.618
E1BXD0	General transcription factor IIF subun	GTF2F2	29.919
E1BSC0	RNA helicase OS=Gallus gallus OX=	--	119.15
A0A3Q2UC12	Platelet-activating factor acetylhydrol	PAFAH1B2	26.806
F1NTP6	Solute carrier family 25 member 28(p	SLC25A28	41.252
P63270	Actin, gamma-enteric smooth muscle	ACTG2	41.876
F1NN38	Palmitoyltransferase OS=Gallus gallu	ZDHHC6	40.583
F1P327	Dehydrogenase/reductase 13(predicte	DHRS13	34.844
A0A1L1RUT1	Coatomer subunit beta' OS=Gallus ga	COPB2	103.14
A0A1D5PPC9	Tetraspanin 13(predicted) OS=Gallus	TSPAN13	21.11
F1P3J5	Lipase OS=Gallus gallus OX=9031 G	LIPA	45.281
A0A1D5PAR5	Tubulin alpha chain OS=Gallus gallu	TUBA3E	49.956
Q5ZK62	Arf-GAP with coiled-coil, ANK repe	ACAP2	88.453
A0A1D5NZF8	Ovotransferrin OS=Gallus gallus OX	MELTF	78.354
P18936	NADH-ubiquinone oxidoreductase ch	MT-ND1	36.077
A0A1D5PHB6	RNA helicase OS=Gallus gallus OX=	DHX36	112.65
Q9W6H0	Mimecan OS=Gallus gallus OX=903	OGN	33.179
A0A1D5PPY9	Tetraspanin OS=Gallus gallus OX=9(	TSPAN33	36.785
F1NT58	Diablo homolog, mitochondrial OS=(	DIABLO	26.041
A0A1D5PKL6	cGMP-dependent protein kinase OS=	PRKG1	51.58
A0A3Q2U4H1	Extended synaptotagmin 1(predicted)	ESYT1	114.18
A0A1D5PNV7	Transmembrane protein 245(predicte	TMEM245	95.113
P18943	Cytochrome c oxidase subunit 1 OS=	MT-CO1	57.051
Q90WR6	Sulfotransferase OS=Gallus gallus O	SULT1C	36.25
F1P0Z7	ADP ribosylation factor like GTPase	ARL8B	21.509
E1C1B8	CCR4-NOT transcription complex su	CNOT1	266.81

R4GLJ4	Transmembrane 6 superfamily memb	--	39.274
E1BVN1	Metaxin 2(predicted) OS=Gallus gall	MTX2	31.18
A0A3Q2TUG2	Ecm29 proteasome adaptor and scaffo	--	204.43
Q08392	Glutathione S-transferase OS=Gallus	--	25.298
A0A3Q2TWG2	Helicase C-terminal domain-containi	--	59.205
A0A1D5NYW5	Peptidylprolyl isomerase OS=Gallus g	FKBP11	10.977
A0A1D5NZW0	Zinc finger protein 36 C3H1 type-like	ZFP36L2	50.606
A0A3Q2UPL1	Ankyrin 1(predicted) OS=Gallus gallu	ANK1	194.78
A0A1D5NT29	Methionyl-tRNA formyltransferase, n	MTFMT	41.775
A0A1D5PWH6	THO complex subunit 5 homolog OS	THOC5	78.612
Q5XKY5	5-aminoimidazole-4-carboxamide rib	PURH	64.402
F1N970	Retinol dehydrogenase 14(predicted)	RDH14	35.627
A0A3Q2TYM7	Putative aarF domain-containing prot	--	55.524
Q04678	FACT complex subunit SSRP1 OS=G	SSRP1	80.025
E1BQU4	Vesicle-fusing ATPase OS=Gallus ga	NSF	82.176
R4GLH9	Protein kinase domain-containing pro	--	103.01
A0A3Q3AG75	Glycine hydroxymethyltransferase OS	--	48.563
E1C5B1	ATP-binding cassette sub-family G m	ABCG2	77.374
R4GFN8	Sugar phosphate phosphatase OS=Ga	ARMT1	50.849
A0A452J855	Tudor-interacting repair regulator pro	NUDT16L1	33.829
A0A3Q3AB56	Glutamate--cysteine ligase OS=Gallu	GCLC	66.809
A0A1L1S044	AIG1-type G domain-containing prote	LOC420368	42.744
Q5F412	Dynein light chain OS=Gallus gallus	DYL1	10.334
A0A3Q2UG75	Ras-responsive element-binding prote	RREB1	187.42
Q5ZK34	Coiled-coil domain containing 12(pre	CCDC12	19.982
A0A1D5PC75	FA_desaturase domain-containing pro	SCD	65.033
A0A1D5PBV9	Glutamine synthetase OS=Gallus gall	GLUL	42.199
F1P1P3	Apoptotic protease-activating factor 1	APAF1	142.29
Q9DDD0	Neurexin-1 (Fragment) OS=Gallus ga	NRXN1	150.07
F1NG44	ZnF_CDGSH domain-containing prot	CISD1	11.864
F1NZA2	Translocator protein(predicted) OS=G	TSPO	18.576
E1C1M8	Syntaxin-18 OS=Gallus gallus OX=9	STX18	38.387
A0A1D6UPS9	Splicing factor 3b subunit 3(predicted	SF3B3	135.49

F1NS88	Isochorismatase domain-containing p	ISOC1	31.727
Q6W8X3	High mobility group A1b OS=Gallus	HMGA1	10.429
F6SU35	Ribosomal protein OS=Gallus gallus	RPL10A	24.708
A0A3Q2UDN9	Progestin and adipoQ receptor family	PAQR7	35.831
A0A3Q2TSE6	Thioredox_DsbH domain-containing	SPATA20	82.272
Q5F3C6	Serine/threonine protein phosphatase	PPP2R5C	61.138
A0A1D5PHS9	UPF2 regulator of nonsense mediated	UPF2	147.74
F1NDH1	Arginyl-tRNA--protein transferase 1 (	ATE1	54.901
E1C1L6	Ectonucleoside triphosphate diphospl	ENTPD5	47.96
Q5ZKB9	Probable ATP-dependent RNA helica	DDX6	54.417
A0A3Q3B0N1	Importin N-terminal domain-containi	IPO9	112.95
A0A1D5PFS2	Ras-related GTP-binding protein OS=	RRAGA	36.553
Q5ZK71	DNA topoisomerase OS=Gallus gallu	TOP3B	96.686
F1NZZ2	Laminin subunit alpha 5(predicted) O	LAMA5	405.46
A0A3Q3AXF8	RCR-type E3 ubiquitin transferase OS	MYCBP2	505.48
Q5F3T2	Septin-type G domain-containing pro	SEPTIN9	65.523
A0A1D5P201	2-phosphoxylose phosphatase 1(predi	PXYLP1	55.726
F1NX83	4-alpha-glucanotransferase OS=Gallu	AGL	174.43
A0A1D5PCW9	Ribonuclease L(predicted) OS=Gallus	RNASEL	78.058
A0A1D5P360	Tyrosine-protein kinase OS=Gallus g	LYN	56.074
F1NPF0	Protein-synthesizing GTPase OS=Gal	EIF2S3	51.103
E1BVV9	Cactin OS=Gallus gallus OX=9031 G	CACTIN	85.765
A0A1D5P441	Protein-tyrosine-phosphatase OS=Ga	PTPRC	131.72
F1NHN4	Motile sperm domain containing 2(pr	MOSPD2	59.795
P08251	Sodium/potassium-transporting ATPa	ATP1B1	34.94
F1NY76	Poly(ADP-ribose) glycohydrolase OS	PARG	107.51
A0A1D5PIH4	UDP-glucose glycoprotein glucosyltra	UGGT1	174.71
Q5ZJM3	Peptide-N(4)-(N-acetyl-beta-glucosar	NGLY1	73.902
P0C1H5	Histone H2B 7 OS=Gallus gallus OX	H2B-VII	13.964
A0A1L1RLR2	IST1 homolog OS=Gallus gallus OX=	IST1	39.739
R4GGZ2	NDUFA4 mitochondrial complex ass	NDUFA4	9.497
P41239	Tyrosine-protein kinase CSK OS=Ga	CSK	50.751
Q5ZL39	Serine/threonine-protein phosphatase	PPP1CC	36.999

A0A1D5PBN1	CD44 antigen OS=Gallus gallus OX=	CD44	43.191
F1NUY5	UBX domain-containing protein OS=	FAF2	52.621
F1NI41	GPI-anchor transamidase OS=Gallus	PIGK	44.434
F1NJY3	Ig-like domain-containing protein OS	ILDR1	65.711
Q5ZIG2	Pre-mRNA-splicing factor SLU7 OS=	SLU7	65.364
O73885	Heat shock cognate 71 kDa protein O	HSPA8	70.826
Q5ZHX7	NADH-cytochrome b5 reductase 2 O	CYB5R2	33.607
A0A3Q2U245	HECT and RLD domain containing E	--	12.434
Q5ZKG8	Golgi to ER traffic protein 4 homolog	GET4	34.648
F1NVR6	N(alpha)-acetyltransferase 15, NatA a	NAA15	101.05
A0A1D5P3R3	Protein phosphatase 6 regulatory subu	PPP6R2	103.79
Q805F9	DNA damage-binding protein 1 OS=(	DDB1	126.95
Q5F339	Holocytochrome c-type synthase OS=	HCCS	31.438
F1N9U0	PRP6 homolog OS=Gallus gallus OX	PRPF6	106.8
E1BWC6	Mitochondrial calcium uptake 1(predi	MICU1	54.741
F1NTD6	Activating signal cointegrator 1 comp	ascc3	251.85
A0A1D5NZ00	Sec1 family domain containing 2(pre	SCFD2	69.805
F1NR71	MPN domain-containing protein OS=	PSMD7	36.905
A0A3Q3AJL9	Deleted in malignant brain tumors 1 p	DMBT1	57.653
E1C0W7	ERO1-like protein alpha(predicted) C	ERO1A	52.926
F1NT57	Aldo_ket_red domain-containing prot	AKR1B1	37.057
R4GFT6	SUN domain-containing protein OS=	SUN2	66.348
E1C2V9	Arf-GAP domain-containing protein (	ARFGAP2	57.362
Q5ZII9	Tuftelin-interacting protein 11 OS=G	TFIP11	95.573
F1N988	Clustered mitochondria protein homo	CLUH	171.72
A0A3Q2UAU6	Nipped-B protein OS=Gallus gallus (	LOC427025	289.4
A0A3Q2TVC7	Peroxin-14 OS=Gallus gallus OX=90	PEX14	39.402
A0A1D5PGJ1	Phosphatidylinositol transfer protein a	PITPNA	30.112
F1NHZ7	Solute carrier family 30 member 1 OS	--	55.993
F1NSP8	Heterogeneous nuclear ribonucleopro	HNRNPU	88.77
A0A3Q2U958	Coenzyme A synthase OS=Gallus gal	--	54.898
E1C2K7	TBC1 domain family member 9B(pre	TBC1D9B	138.03
A0A1D5PJH0	Putative ergosterol biosynthetic prote	C5H14orf1	15.72

E1BWW7	Transmembrane protein 177 OS=Gall	TMEM177	34.044
E1BTA0	Catechol O-methyltransferase OS=Ga	COMT	29.788
F1NB57	Serine/threonine-protein kinase PLK	PLK2	76.506
Q5ZK40	SWI/SNF-related matrix-associated a	SMARCB1	44.495
E1BZ96	Solute carrier organic anion transport	SLCO2A1	70.13
A0A1D5P277	Chloride channel protein OS=Gallus g	CLCN3	87.84
E1C4M0	40S ribosomal protein S2 OS=Gallus	RPS2	30.673
A0A1D5PCE6	Protein kinase domain-containing pro	SCYL3	77.084
A0A3Q2U2T0	Tetraspanin OS=Gallus gallus OX=90	TSPAN18	27.831
A0A1D5P086	Nuclear factor 1 OS=Gallus gallus O	NFIC	60.448
F1NPB8	Aquarius intron-binding spliceosomal	AQR	174.26
A0A3Q2UB91	ELMO domain-containing protein OS	ELMO2	82.861
A0A452J830	Importin subunit alpha OS=Gallus ga	KPNA1	60.107
A0A3Q2UIV3	3'-5' exonuclease domain-containing p	EXD2	66.744
Q9DEQ6	Eukaryote initiation factor 2 beta OS=	EIF2B	38.436
Q02391	Golgi apparatus protein 1 OS=Gallus	GLG1	129.71
A0A1D5NX47	GB1/RHD3-type G domain-containin	ATL2	65.439
P0CF65	Protein cereblon OS=Gallus gallus O	CRBN	51.118
A0A3Q2U5C1	Gluconokinase OS=Gallus gallus OX	IDNK	20.062
P18938	NADH-ubiquinone oxidoreductase ch	MT-ND3	13.12
A0A1D5P365	Neuroblastoma amplified sequence(p	NBAS	269.69
Q5ZJ03	Guanine nucleotide-binding protein s	GNG2	7.8781
Q5ZJF1	COP9 signalosome complex subunit 3	COPS3	47.962
A0A1D5PY39	VPS35 endosomal protein-sorting fac	C14H16orf62	109.32
H9L0D7	Wiskott-Aldrich syndrome protein fa	WASF2	54.485
A0A3Q2ULN8	Trinucleotide repeat containing adapt	TNRC6B	187.65
A0A3Q2UE34	AIRS_C domain-containing protein O	--	32.538
E1C576	Tetratricopeptide repeat domain 37(p	TTC37	174.63
A0A1D5PAF9	AP-3 complex subunit beta OS=Gallu	AP3B1	124.44
E1BRV1	Zinc finger ZZ-type and EF-hand don	--	326.03
F1NH33	Aldehyde dehydrogenase OS=Gallus	ALDH3A2	54.092
A0A1D5P4Y1	Ski2 like RNA helicase(predicted) OS	SKIV2L	108.13
A0A1D5PY19	Amino_oxidase domain-containing pr	LOXL4	62.069

A0A1D5P7Y2	Tetraspanin 13(predicted) OS=Gallus	TSPAN13	22.536
A0A1D5PKV6	Xylulose kinase OS=Gallus gallus OX	XYLB	58.348
A0A3Q3ASX6	Calcium activated nucleotidase 1(pre	CANT1	44.478
F1NBT7	Solute carrier family 43 member 3(pr	SLC43A3	76.455
P02659	Apovitellenin-1 OS=Gallus gallus OX	--	11.966
Q5ZJA7	Oxoglutarate dehydrogenase (succiny	LOC107054982	115.18
A0A1D5PN41	Maleylacetoacetate isomerase OS=Ga	GSTZ1	23.977
A0A3Q2UIK1	Glycogen [starch] synthase OS=Gallu	LOC107050456	97.893
E1BRH0	CDK5 regulatory subunit-associated p	CDKAL1	65.681
A0A1D5PSM9	GLTP domain-containing protein OS=	GLTP	24.064
Q02977	Proto-oncogene tyrosine-protein kinas	YRK	60.001
A0A1D5P1D5	U-box domain-containing protein OS	UBE4B	133.28
Q5ZKX6	NEDD8-conjugating enzyme UBE2F	UBE2F	21.229
E1BR42	Dedicator of cytokinesis 2(predicted)	DOCK2	212.93
A0A3Q2U8J0	S4 RNA-binding domain-containing p	IMP3	21.187
A0A3Q3AW32	Adenosine deaminase OS=Gallus gal	CECR1	53.854
A0A1D5PD38	Cysteine sulfinic acid decarboxylase(	CSAD	55.277
A0A3Q2UAW9	GIMAP family P-loop NTPase domai	--	14.748
A1IMF0	Ubiquitin carboxyl-terminal hydrolase	UCH-L1	25.11
E1C2G1	Transcription factor 25(predicted) OS	TCF25	77.089
F1NPE8	Receptor-type tyrosine-protein phosp	PTPRA	92.177
F1N903	26S proteasome non-ATPase regulato	PSMD6	45.577
F1NL71	Coiled-coil domain-containing protein	CCDC47	55.659
F1NMJ9	Tyrosine-protein kinase OS=Gallus g	JAK1	133.01
A0A3Q2UIJ5	Delta-sagatoxin-srs1a-like isoform x1	LOC769729	22.853
E1BW97	AP-3 complex subunit beta OS=Gallu	AP3B1	122.23
E1BWU0	Peroxisomal biogenesis factor 11 alph	PEX11A	46.004
F1NW17	Extended synaptotagmin 2(predicted)	ESYT2	95.977
E1BT32	Rhomboid domain-containing protein	RHBDD1	36.808
Q5ZJV5	Cytochrome c oxidase subunit 4 OS=	COX4I1	19.631
A0A1D5P4B4	FERM domain-containing protein OS	EPB41L4B	32.886
A0A3Q2UG91	Notch receptor 2(predicted) OS=Gall	NOTCH2	251.03
F1NS44	RING-type E3 ubiquitin transferase C	UHRF2	91.993

A0A3Q2TRS4	Myotubularin-related protein 14 isofo	MTMR14	64.367
A0A1D5PDJ9	Unk like zinc finger(predicted) OS=G	UNKL	75.021
F1NVV0	CID domain-containing protein OS=(	PCF11	186.62
A0A1L1RXQ1	TPR_REGION domain-containing pr	CDC23	67.7
E1C4B8	Phosphomannomutase OS=Gallus ga	PMM2	28.282
Q5ZKQ8	ADP-ribosylation factor-like protein 8	ARL8A	21.444
A0A1L1RZW1	Voltage-dependent anion-selective ch	VDAC3	30.403
Q5ZLD7	Vacuolar protein sorting-associated p	VPS53	93.704
F1P212	Thioredoxin domain-containing prote	TMX3	54.043
F1NYS9	Mitogen-activated protein kinase kina	MAP3K5	141.7
A0A1D5NTG0	MPN domain-containing protein OS=	COPS5	37.752
E1BW35	Ectoderm-neural cortex protein 2 OS=	KLHL25	66.193
A5HUL2	MHC class II beta chain 1 OS=Gallus	BLB1	29.246
A0A1D5PYI1	AdoHcyase_NAD domain-containing	AHCYL1	59.258
A0A3Q2UCJ8	ARVCF(predicted) OS=Gallus gallus	ARVCF	99.25
E1C7F4	Epoxide hydratase OS=Gallus gallus	EPHX1L	55.611
Q2PUH1	Acetyl-CoA carboxylase kinase OS=(	PRKAA1	64.098
A0A1L1RQR5	Mitochondrial ribosomal protein S2(p	MRPS2	21.546
Q5ZL18	N-fatty-acyl-amino acid synthase/hyd	PM20D1	57.161
F1ND93	Pentatricopeptide repeat domain 3(pre	PTCD3	48.977
F1NSK8	ADP-ribosylarginine hydrolase(predic	ADPRH	42.135
A0A1D5P3W4	Solute carrier family 27 member 5(pr	SLC27A2	64.133
F1P4X9	DUF3677 domain-containing protein	INTS1	246.85
F1P3Q8	Crooked neck pre-mRNA splicing fac	CRNKL1	83.171
E1BQ95	CD93 molecule(predicted) OS=Gallu	CD248	58.828
E1BYM5	U4/U6.U5 small nuclear ribonucleopr	SNRNP27	18.844
F7BQ59	Nuclear transcription factor Y subuni	NFYA	32.287
F1NX32	Glycine cleavage system P protein OS	GLDC	111.8
A0A1D5P1J7	ATP synthase membrane subunit f OS	ATP5J2	10.308
A0A1D5PXN4	Protein kinase C delta type OS=Gallu	PRKCD	78.479
A0A1D5PW67	PKS_ER domain-containing protein (	TP53I3	35.849
A0A1D5PF08	Calnexin(predicted) OS=Gallus gallu	CANX	68.368
F1NBW0	Mitochondrial poly(A) polymerase(pr	MTPAP	63.806

E1BSE6	RAP domain-containing protein OS=	FASTKD2	77.344
P09482	Neuronal acetylcholine receptor subu	CHRNA4	70.837
A0A1D5NW46	Histone deacetylase 2 OS=Gallus gall	HDAC2	51.938
F1NYW0	Protein tyrosine phosphatase non-rece	PTPN2	66.131
F1NZX7	Sorting nexin-2 OS=Gallus gallus OX	SNX2	66.529
F1NPX4	Vacuolar fusion protein CCZ1 homol	CCZ1	55.136
Q5ZML0	BRISC and BRCA1-A complex mem	BABAM2	43.429
F1NI85	Catechol-O-methyltransferase domain	COMTD1	38.528
A0A1D5PCA7	E3 ubiquitin-protein ligase TRIP12 O	TRIP12	223.41
F1NH49	Nuclear pore complex protein OS=Ga	NUP107	105.45
F1NQJ1	Alanine--glyoxylate aminotransferase	AGXT2	52.433
A0A3Q2U067	Sideroflexin 4(predicted) OS=Gallus	SFXN4	45.389
F1NZL2	Dysferlin(predicted) OS=Gallus gallu	DYSF	238.45
F1P1V6	AB hydrolase-1 domain-containing pr	ABHD2	48.033
F1NZD3	RRM domain-containing protein OS=	RBMXL1	41.551
F1NNL8	Translocator protein 2(predicted) OS=	TSPO2	18.597
Q9DGN6	Endothelin-converting enzyme 1 OS=	ECE-1	84.985
A0A1L1RPD1	Amidase domain-containing protein (	FAAH	64.147
F1NIB1	Rap1 GTPase-GDP dissociation stimu	RAP1GDS1	66.27
A0A1D5NYV3	DIS3 homolog, exosome endoribonuc	DIS3	101.32
E1C5Y7	Phospholipase A2 receptor 1(predicte	PLA2R1	168.59
A0A3Q2U8Q1	MAM domain containing 4(predicted	MAMDC4	140.68
A0A3Q3A4Y1	Polypeptide N-acetylgalactosaminyltr	GALNT2	58.81
Q5ZLS3	E3 ubiquitin-protein ligase BRE1A O	RNF20	114.81
A0A3Q2UAJ0	LRR binding FLII interacting protein	LRRFIP1	103.71
E1BQH9	UDP-glucose glycoprotein glucosyltra	UGGT2	178.99
E1C6E8	Palmdelphin(predicted) OS=Gallus ga	PALMD	62.768
F1NVW7	Complex I-B14.7 OS=Gallus gallus (	NDUFA11	14.143
E1C6G6	Signal recognition particle 54 kDa pro	SRP54	55.696
F1NI89	S-(hydroxymethyl)glutathione dehydr	ADH4	39.605
A0A3Q3AA34	Eukaryotic translation initiation facto	EIF4G1	172.44
A0A1L1RRE8	HIT domain-containing protein OS=(	HINT3	18.599
A0A3S5ZPJ3	Checkpoint and tumor suppressor pro	CHEK2	54.328

F1P4K4	Aldedh domain-containing protein OS	ALDH8A1	53.204
E1BRL5	Myotubularin OS=Gallus gallus OX=	MTM1	69.72
A0A1L1RUX2	RNA helicase OS=Gallus gallus OX=	EIF4A2	42.389
A0A3Q2U7V5	Transmembrane protein 14C (Fragme	LOC420860	10.986
A0A1D5P3A0	Mic1 domain-containing protein OS=	C2H18ORF8	77.267
Q8JFP1	Eukaryotic initiation factor 4A-II OS=	EIF4A2	46.371
E1BSX0	Aminotran_5 domain-containing prot	AGXT	50.938
Q5ZLA0	Glucosamine-6-phosphate isomerase	GNPDA1	32.264
O93295	Ectonucleoside triphosphate diphosph	ENTPD8	54.034
E1BRK4	Glycos_transf_1 domain-containing p	ALG1	52.301
A0A3Q2UEL1	Phosphopantothenoylcysteine synthet	PPCS	43.713
A0A1D5P7X9	Cytochrome b-c1 complex subunit 7 (	UQCRB	13.499
F1NY56	Diacylglycerol kinase OS=Gallus gall	DGKQ	100.49
A0A1D5PIU8	LPS responsive beige-like anchor pro	LRBA	316.67
A0A1L1RN32	Deleted in malignant brain tumors 1(p	LOC426820	134.34
Q5ZJ64	Eukaryotic translation initiation facto	EIF3M	42.591
A0A1D5PVC0	Nipped-B protein OS=Gallus gallus O	LOC427439	291.3
A0A1D5PXG5	SEC24 homolog B, COPII coat comp	SEC24B	130.77
A0A3Q3A649	Solute carrier family 25 member 42(p	SLC25A42	36.291
F1NQA5	Smg4_UPF3 domain-containing prote	UPF3B	56.926
A0A3Q3B2M9	TOG domain-containing protein OS=	GCN1	290.57
F1NVF2	Protein kinase domain-containing pro	MAP2K6	37.653
F1NBC2	Arylacetamide deacetylase(predicted)	AADAC	44.608
A0A1D5P5T1	Eukaryotic translation initiation facto	EIF3K	25.13
Q5ZIF3	Ufm1-specific protease 2 OS=Gallus	UFSP2	52.098
F1NME3	Lysophosphatidylcholine acyltransfer	LPCAT1	55.134
A0A1D5PMR8	RRM domain-containing protein OS=	CPSF7	58.917
A0A1D5P3L6	Pre-mRNA processing factor 39(pred	PRPF39	64.047
A0A3Q2UD05	DNA-directed RNA polymerase OS=	--	35.035
F1NB65	Arachidonate 5-lipoxygenase(predicte	ALOX5	86.209
A0A1D5P3M6	Cyclin and CBS domain divalent met	CNNM4	70.5
F1P337	Sorting nexin OS=Gallus gallus OX=	SNX5	45.681
A0A3Q2U347	Phosvitin OS=Gallus gallus OX=903	VTG3	187

A5HUL8	MHC class II M beta chain 2 OS=Gal	DMB2	27.887
E1BZ22	Corticosteroid 11-beta-dehydrogenase	HSD11B1b	32.936
E1BXS1	BCL2 like 13(predicted) OS=Gallus g	BCL2L13	51.179
F1NY52	LIM domain 7(predicted) OS=Gallus	LMO7	183.74
F1NBA8	Importin N-terminal domain-containi	IPO7	119.43
F1P4D5	Store-operated calcium entry-associat	SARAF	38.877
F1NBM0	2-oxoglutarate and iron-dependent ox	OGFOD1	60.862
A0A3Q3APC4	E3 ubiquitin-protein ligase UBR4 iso	--	100.77
A0A1L1RTK2	Torsin family 1 member B OS=Gallu	--	44.748
Q5ZLX0	Cytochrome b5 heme-binding domair	PGRMC2	21.641
A0A3Q2TXM3	Putative H+ transporting F1 ATP syn	ATP5E	5.7928
A0A1D5NYK7	Moesin OS=Gallus gallus OX=9031 I	--	68.37
A0A3Q3A7Y5	AP-2 complex subunit sigma OS=Ga	CLAPS2	31.042
A0A1D5PK62	Coatomer subunit zeta OS=Gallus ga	COPZ1	33.762
A0A3Q2U6U8	Oxoglutarate dehydrogenase (succiny	LOC107054982	165.8
Q5F3B5	G protein subunit alpha q(predicted) (	GNAQ	42.234
F1P160	Dedicator of cytokinesis 3(predicted)	DOCK3	230.85
A0A1D5NZQ7	Peroxidase OS=Gallus gallus OX=90	PXDN	177.5
F1NJM8	IF rod domain-containing protein OS	SYNM	182.39
A0A1D5PW05	Peroxiredoxin-like 2 activated in M-(	FAM213A	24.887
A0A3Q2U0D5	Atp-binding cassette sub-family f me	ABCF2	69.689
Q5F3R8	Ras-related protein Rab-11B(predicte	RAB11B	24.521
A0A1D5PRS6	Death associated protein 3(predicted)	DAP3	45.576
A0A1D5PUM1	MYB binding protein 1a(predicted) C	MYBBP1A	137.34
A0A1D5P6M2	Suppressor of tumorigenicity 14 prote	ST14	92.007
Q5ZLD9	FIST_C domain-containing protein O	FBXO22	43.256
A0A1D5PF77	AdoHcyase_NAD domain-containing	AHCYL2	56.696
Q5ZMG7	AP-1 complex subunit mu-1 OS=Gal	AP1M1	48.556
E1C310	Endoplasmic reticulum transmembrai	BCAP29	27.765
O93467	GTP-binding protein OS=Gallus gallı	cRhoA	21.782
E1BRU9	Carn_acyltransf domain-containing p	CROT	70.06
A0A3Q3B0X4	EXPERA domain-containing protein	LOC100857622	24.018
A0A1L1RRH5	Ig-like domain-containing protein OS	BF1	35.953

A0A1L1RR94	Prosaposin OS=Gallus gallus OX=90	PSAP	57.16
F1NW41	Cationic amino acid transporter 2 OS=	SLC7A2	37.798
E1BVN3	Phospholipid-transporting ATPase OS	ATP8B4	134.05
F1NTD7	Prostaglandin F2 receptor inhibitor(pr	PTGFRN	98.139
A0A1D5NYA6	Calcium/calmodulin-dependent prote	CAMK2B	60.194
A0A3Q2UA27	Hook microtubule tethering protein 3	HOOK3	63.395
E1C115	Eukaryotic translation initiation facto	EIF4EBP1	12.5
A0A3Q2TWJ4	Receptor protein-tyrosine kinase OS=	EPHA5	94.467
A0A1D5PGI3	Sidoreflexin OS=Gallus gallus OX=9	SFXN1	35.675
F1NLD7	Double-stranded RNA-activated prote	EIF2AK2	62.318
A0A1D5PVC4	CCR4-NOT transcription complex su	CNOT9	33.601
A0A140T8G9	BH3-interacting domain death agonis	BID	21.677
A0A3Q2TU25	Dihydropyrimidine dehydrogenase [N	DPYD	111.34
A0A1D5P3U4	PH domain-containing protein OS=G	FERMT2	81.019
A0A1D5PLX0	Coatomer subunit gamma OS=Gallus	COPG2	97.557
A0A3Q2U2C2	Cytochrome P450 2G19(predicted) O	LOC112530469	59.3
A0A1D5PVW3	Cornichon family AMPA receptor au	CNIH4	16.049
R4GHW9	Band 4.1 OS=Gallus gallus OX=9031	EPB41	120.19
A0A3Q3AC33	Alpha-1,4 glucan phosphorylase OS=	LOC107049660	18.682
Q5ZI49	Ribose-phosphate pyrophosphokinase	PRPS2	35.663
A0A452J7Z9	RING-CH-type domain-containing pr	MARCH5	31.334
E1BSP8	SEC24 homolog D, COPII coat comp	SEC24D	113.85
A0A1D5NXP9	Ephexin1(predicted) OS=Gallus gallu	NGEF	62.167
F1NW50	V-type proton ATPase subunit H OS=	ATP6V1H	55.478
Q5ZIT5	Ras-related protein Rab-10 OS=Gallu	RAB10	22.549
A0A3Q2UHS1	Junctional adhesion molecule 3(predi	JAM3	33.204
A0A3Q2TXM8	Myotubularin phosphatase domain-co	MTMR10	88.341
A0A1D5P3K0	Terminal uridylyl transferase 7(predic	ZCCHC6	172.13
E1BRX4	Sidoreflexin OS=Gallus gallus OX=9	SFXN5	40.234
A0A3Q2U5G8	Transcriptional coactivator p100 (Fra	--	39.877
E1BQK9	CULLIN_2 domain-containing protei	CUL4B	101.26
F1NQI9	Chromosome 17 open reading frame '	C18H17orf75	32.61
A0A3Q2UC82	RAS p21 protein activator 2(predicte	RASA2	90.396

E1C0Z8	Maspardin OS=Gallus gallus OX=90:	SPG21	35.273
F1P4A7	Eukaryotic translation initiation facto	EIF4G2	101.8
F1NGJ3	SEC7 domain-containing protein OS=	GBF1	207.87
A0A3Q2UI39	E3 ubiquitin-protein ligase HACE1 C	HACE1	97.965
Q5ZMV7	WD repeat-containing protein 82 OS=	WDR82	35.105
E1C3I1	Zinc finger C3HC-type containing 1(p	ZC3HC1	53.228
A0A1D5NW27	Tubulin alpha chain OS=Gallus gallu	TUBA1A	50.135
Q5F4A0	Suf domain-containing protein OS=G	CSTF3	83.071
A0A1D5P804	Protein odr-4 homolog OS=Gallus ga	C8H1orf27	49.382
F1NAT2	Cap-specific mRNA (nucleoside-2'-O	CMTR1	106.64
A0A1D5PAP3	Carbonic anhydrase OS=Gallus gallus	CA7	29.287
A0A1D5NWJ4	SLD3 domain-containing protein (Fra	C5H15orf52	61.756
Q02092	Growth hormone receptor OS=Gallus	GHR	68.572
A0A1D5NU72	PDZ domain-containing protein OS=(	SYNJ2BP	15.457
A0A3Q3ALZ9	SAM domain, SH3 domain and nucle	SAMSN1	40.217
F1NV36	ZP domain-containing protein OS=Ga	LINC00954	102.3
Q5ZIF1	Adipocyte plasma membrane-associat	APMAP	46.115
Q5F418	26S proteasome non-ATPase regulato	PSMD1	106.06
A0A1L1RMH9	Transmembrane protein 120A(predict	TMEM120A	40.421
E1C6U2	Complement component 7(predicted)	C7	93.015
A0A1L1RLH8	Brix domain-containing protein OS=(	RPF1	38.815
F1NXH1	MOSC domain-containing protein OS	MARC1	37.414
P50594	Protein mago nashi homolog OS=Gal	MAGOH	17.105
F1NXB9	Component of oligomeric Golgi comp	COG3	93.941
A0A1L1RXY9	116 kDa U5 small nuclear ribonucleo	EFTUD2	104.38
A0A1D5PVM0	Vitellogenin domain-containing prote	MTTP	99.966
A0A1D5P2F3	Hexokinase OS=Gallus gallus OX=9(	HK2	98.885
F1NFM3	Myotubularin phosphatase domain-co	MTMR9	63.526
F1NM67	Integrin beta OS=Gallus gallus OX=9	ITGB2	85.465
A0A1D5PEH7	Solute carrier family 27 member 5(pr	SLC27A2	68.832
A0A1L1RUR2	FAD synthase OS=Gallus gallus OX=	FLAD1	66.334
A0A1D5P285	Sigma 1-type opioid receptor OS=Ga	SIGMAR1	18.306
A0A3Q2UDV6	Ig-like domain-containing protein OS	NPTN	20.718

A0A1D5PMZ4	TYR_PHOSPHATASE_2 domain-co	PTP4A3	19.747
Q5F3Z1	CTP synthase 2 OS=Gallus gallus OX	CTPS2	66.068
Q91000	Trans Golgi network protease furin O	FURIN	86.63
F1NFY6	Histone deacetylase OS=Gallus gallus	HDAC8	40.529
F1P0Y0	Cytoskeleton-associated protein 5(pre	CKAP5	225.35
Q5ZL55	Charged multivesicular body protein (	CHMP6	23.258
A0A3Q3B169	Family with sequence similarity 91 m	FAM91A1	94.596
Q5F422	SAP domain-containing protein OS=(	DEK	42.163
A0A1D5PN32	Annexin OS=Gallus gallus OX=9031	ANXA11	52.687
A0A3Q2U926	Asp-B-Hydro_N domain-containing p	ASPH	37.277
P81628	Endoplasmic reticulum resident prote	ERP29	25.373
F1NH26	AMP_N domain-containing protein C	XPNPEP3	56.286
R4GGM5	GOLD domain-containing protein OS	TMED7	36.748
F1N9D0	alpha-1,2-Mannosidase OS=Gallus ga	MAN1A1	72.262
E1C388	ATP-dependent RNA helicase DHX2	DHX29	154.86
Q646T7	Peptidylprolyl isomerase OS=Gallus g	FKBP51	50.427
A0A1D5P140	Spen family transcriptional repressor(	SPEN	398.26
F1NLM6	Serine/threonine protein phosphatase	PPP2R5E	54.699
Q9YH14	ATP synthase subunit e, mitochondria	rbf	8.3387
Q5ZM57	Very-long-chain (3R)-3-hydroxyacyl-	HACD3	42.835
A0A1D5PVG8	Prolylcarboxypeptidase(predicted) OS	PRCP	54.686
A0A3Q2TSV4	SAFB like transcription modulator OS	--	53.818
E1BQ97	Two pore segment channel 1(predicte	CATSPER4	94.845
A0A3Q2UGX4	SUN domain-containing protein OS=	SUN1	89.356
E1C593	Exportin-T OS=Gallus gallus OX=90	XPOT	109.82
Q5ZIF4	Solute carrier family 25 member 13(p	SLC25A13	74.102
F1NEQ3	RNA helicase OS=Gallus gallus OX=	DDX18	73.837
A0A1D5NTS3	Phospholipid-transporting ATPase OS	ATP11C	128.79
A0A1D5NV85	Nucleoporin 160(predicted) OS=Gall	NUP160	100.23
F1NCB0	DNA-directed RNA polymerase subu	POLR2B	133.82
A0A1D5PV15	Nudix hydrolase domain-containing p	NUDT7	27.7
Q5ZLW3	Dymeclin OS=Gallus gallus OX=903	DYM	76.16
A0A3Q2UCD7	Centrosomal protein 290(predicted) C	--	20.166

R4GIW4	X-prolyl aminopeptidase 2(predicted)	XPNPEP2	75.788
F1P1R2	RNA binding motif protein 19(predic	RBM19	107.87
E1BSI4	Charged multivesicular body protein	CHMP5	24.602
F1NNT4	COMM domain-containing protein O	COMMD4	22.093
A0A1D5PBM6	Complex I-49kD OS=Gallus gallus O	NDUFS2	84.612
A0A1D5PDA2	CULLIN_2 domain-containing protei	CUL2	90.379
R4GGD2	Transmembrane protein 38B(predicte	TMEM38B	31.751
F1NBY5	Rab-GAP TBC domain-containing pr	TBC1D15	77.024
F1ND15	J domain-containing protein OS=Gall	DNAJC8	29.614
F1NQG0	Wolframin ER transmembrane glycop	WFS1	100.26
R4GGY0	NAD(P)(+)--arginine ADP-ribosyltra	ART7C	33.995
E1C1P9	Mitochondrial ribosomal protein S27	MRPS27	46.74
A0A3Q2TS12	RAN binding protein 2(predicted) OS	LOC776067	187.94
A0A1D5P560	Integrin beta OS=Gallus gallus OX=9	ITGB5	88.377
F1NAP1	Eukaryotic translation initiation facto	EIF2B2	59.135
A0A3Q2UPQ7	CHDCT2 domain-containing protein	--	25.354
A0A3Q3A8B1	Ras and Rab interactor 2(predicted) O	RIN2	49.821
F1NHG6	Family with sequence similarity 162	FAM162A	17.086
E1BWJ5	Ubiquitinyl hydrolase 1 OS=Gallus g	USP9Y	290.16
A0A1D5P6C6	Beta-1-syntrophin(predicted) OS=Gal	SNTB1	56.746
F1P0T4	Heterogeneous nuclear ribonucleopro	HNRNPLL	60.147
Q5F3N9	Protein ILRUN OS=Gallus gallus OX	ILRUN	31.976
A0A3Q3A6F5	Sema domain-containing protein OS=	PLXND1	210.41
A0A3Q2U5X1	Annexin OS=Gallus gallus OX=9031	ANXA13	34.973
F1N9G6	Glutathione S-transferase kappa OS=	GSTK1	25.353
Q5ZIF2	Delta(24)-sterol reductase OS=Gallus	DHCR24	60.122
Q91025	Vitellogenin-3 (Fragments) OS=Gallu	VTG3	38.15
A0A3Q2TUR0	Coxsackievirus and adenovirus recep	TSTD1	36.828
A0A3Q3A5X4	SWIB domain-containing protein OS	SMARCD2	44.605
A0A1D5PIQ5	Mitogen-activated protein kinase OS=	MAPK14	41.458
F1NJK8	Protein arginine methyltransferase 8(	PRMT1	45.253
A0A1D5PFI2	Calpain 5(predicted) OS=Gallus gallu	CAPN5	73.317
F1NIZ5	Alpha-1B adrenergic receptor OS=Ga	ADRA1B	57.013

F1NKQ3	Zinc finger and BTB domain containi	ZBTB20	73.901
E1BQE4	RNA binding motif protein 26(predic	RBM26	110.06
A0A1D5PR96	RAP2B, member of RAS oncogene fa	--	13.557
F1P4R1	Epoxide hydrolase OS=Gallus gallus	EPHX1	53.191
F1NBL3	Ras-related protein Rab OS=Gallus ga	RAB32	24.346
A0A1D5PPZ5	TLDc domain-containing protein OS=	TLDC1	52.099
A0A3Q2U421	VPS26 endosomal protein sorting fac	DSCR3	32.255
A0A1D5PEK3	Calcium-activated neutral proteinase	CAPN1	81.435
A0A3Q2U9H1	CRAL_TRIO_N domain-containing p	--	11.694
F1NZZ7	VPS8 subunit of CORVET complex(	VPS8	157.16
A0A1D5P8M7	Lipase maturation factor OS=Gallus g	LMF2	81.593
E1C9A8	DUF3730 domain-containing protein	FOCAD	200.31
F1P1K3	Arp2/3 complex 34 kDa subunit OS=	ARPC2	34.401
A0A1L1RWX8	Vacuolar protein sorting-associated p	VPS35	91.74
Q5F4B9	ATP binding cassette subfamily D me	ABCD3	75.534
P18940	NADH-ubiquinone oxidoreductase ch	MT-ND5	66.373
F1NSZ5	Laminin subunit alpha 4(predicted) O	LAMA4	202.47
A0A3Q3AZ85	TGc domain-containing protein OS=(	F13A1	83.75
A0A452J7V1	Zinc transporter 5 OS=Gallus gallus (	SLC30A5	77.11
E1BVM0	Transmembrane protein 199(predicte	TMEM199	21.317
E1BXT5	TP53RK binding protein(predicted) (	TPRKB	19.282
F1NV60	Ectonucleoside triphosphate diphosph	ENTPD8L	46.043
E1C735	S-adenosylmethionine synthase OS=(	MAT1A	43.752
F1NPU9	Polypeptide N-acetylgalactosaminyltr	GALNT7	72.303
F1NDD1	Sidoreflexin OS=Gallus gallus OX=9	SFXN2	35.44
F1NGG4	Putative corticotropin-releasing horm	CRHR2	48.07
F6UZB4	Peptide-O-fucosyltransferase OS=Gal	POFUT2	42.261
O13113	Dolichyl-diphosphooligosaccharide--p	DAD1	12.926
A0A3Q3AQD4	Angiomotin_C domain-containing pro	AMOTL2	77.925
A0A1D5PM72	Hydroxysteroid 17-beta dehydrogenas	HSD17B12	42.67
Q6JHU8	Prolyl 3-hydroxylase 1 OS=Gallus ga	P3H1	81.675
E1C857	Tetraspanin OS=Gallus gallus OX=9(	TSPAN6	27.827
E1C456	Mannosyltransferase OS=Gallus gallu	ALG9	69.272

Q5ZMA6	Glutamine-dependent NAD(+) synthe	NADSYN1	79.226
F1NPK5	Microsomal triglyceride transfer prote	MTTPL	96.74
Q02440	Unconventional myosin-Va OS=Gallu	MYO5A	212.38
Q5ZHV7	SPRY domain-containing protein 7 O	SPRYD7	21.735
F1NB10	PCI domain-containing protein OS=G	GPS1	55.25
P18944	Cytochrome c oxidase subunit 2 OS=	MT-CO2	25.568
F1NHC7	Receptor protein-tyrosine kinase OS=	EPHB2	111.03
Q5ZIQ8	Negative elongation factor complex m	NELFCD	65.665
F1NUY4	GH3 domain containing(predicted) O	GHDC	63.242
P47836	40S ribosomal protein S4 OS=Gallus	RPS4	29.594
A0A1D5NVI1	60S ribosomal protein L9 OS=Gallus	RPL9	21.835
A0A3Q3AGI3	RRP12-like protein OS=Gallus gallus	RRP12	135.12
F1P073	Chloride channel CLIC-like protein 1	CLCC1	58.728
A0A1D5P655	Cytoplasmic FMR1-interacting protei	CYFIP1	145.2
A0A1D5PLJ0	Ras-related GTP-binding protein OS=	RRAGC	44.899
A0A1D5PTS3	Immediate early response 3-interactin	IER3IP1	8.8995
A0A1D5PXZ4	Bromo domain-containing protein OS	ATAD2B	162.67
R4GJ64	Transmembrane protein 161A(predict	TMEM161A	53.731
E1C0Z0	Helicase with zinc finger 2(predicted)	HELZ2	316.16
Q5ZIZ4	Cytosolic purine 5'-nucleotidase OS=	NT5C2	65.894
A0A1D5PDR0	Terpene cyclase/mutase family memb	LSS	85.351
E1C8Y3	Actin-related protein 2/3 complex sub	ARPC3	20.549
F1NJK4	AB hydrolase-1 domain-containing pr	SERHL	34.17
F1NBL6	Importin N-terminal domain-containi	XPO5	136.7
Q5ZLC9	Golgin subfamily A member 7 OS=G	GOLGA7	15.762
E1BTS5	Syntaxin binding protein 3(predicted)	STXBP3	67.664
A0A1D5P9D0	MOB kinase activator 1A(predicted)	MOB1A	25.077
F1NAK4	TOG domain-containing protein OS=	GCN1	293.38
Q5F413	Stromal membrane-associated protein	SMAP2	46.314
F1NIH3	Neutrophil cytosolic factor 2(predicte	NCF2	57.783
E1BR29	Mitochondrial import inner membran	TIMM23B	21.701
E1C0E1	FMN hydroxy acid dehydrogenase do	HAO2	42.047
F1NW73	ATP binding cassette subfamily A me	ABCA8	184.78

A0A1L1RQM9	WD_REPEATS_REGION domain-co	AAAS	85.99
F1NV02	Apolipoprotein B OS=Gallus gallus (	APOB	523.35
E1BVQ3	SEC7 domain-containing protein OS=	ARFGEF2	201.95
A0A1D5PQE8	G protein subunit alpha 13(predicted)	GNA13	43.776
A0A1L1RJ91	EW135(predicted) OS=Gallus gallus	--	104.06
A0A3Q3A8S0	Gamma-tubulin complex component	TUBGCP3	86.065
A0A1D5PV09	NCK associated protein 1 like(predict	NCKAP1L	126.76
A0A3Q2TWP3	Diaphanous homologue(predicted) OS	--	45.669
H9L0N7	CID domain-containing protein OS=(	RPRD2	149.88
Q5ZI89	Trafficking protein particle complex s	TRAPPC11	128.55
A0A1D5P4Q6	Insulin degrading enzyme(predicted)	IDE	111.6
F1P077	Mannosyltransferase OS=Gallus gallu	ALG12	55.778
Q5ZMU6	Serine/threonine-protein phosphatase	PPP4R2	44.962
A0A3Q2UA92	Phosphatidylinositol-3,4,5-trisphosph	TPTE	58.554
Q800K9	Surfeit locus protein 4 OS=Gallus gal	SURF4	30.619
A0A1D5PEB3	Signal transducer and activator of tran	STAT2	96.208
Q5ZLL6	Methylsterol monooxygenase 1 OS=(	MSMO1	35.485
F1NMD8	Sphingosine-1-phosphate lyase 1(pred	SGPL1	61.053
Q5ZLS8	Integrator complex subunit 10 OS=Ga	INTS10	81.831
E1C0P9	U2 snRNP associated SURP domain	U2SURP	120.63
A0A1D5PNK3	Solute carrier family 25 member 11(p	SLC25A11	33.165
A0A3Q2TV46	Rab-GAP TBC domain-containing pr	TBC1D22A	62.676
A0A3Q3ANR7	GB1/RHD3-type G domain-containin	ATL2	61.157
Q6XFR1	Integrin-associated protein OS=Gallu	CD47	34.936
A0A1D5PG05	5'-nucleotidase domain containing 4(p	NT5DC4	60.866
E1BVB6	Cytochrome P450 3A80(predicted) O	CYP3A4	61.56
A0A1L1RPU6	Transmembrane protein 53(predicted)	TMEM53	33.409
F1NTJ5	Myosin IB(predicted) OS=Gallus gall	MYO1B	129.13
F1NW95	NADH dehydrogenase [ubiquinone] 1	NDUFA12	16.945
A0A1D5PNZ9	Phosphatidylinositol 4-kinase alpha(p	PI4KA	232.54
F1NID0	CNH domain-containing protein OS=	VPS39	100.56
A0A1D5P062	SAM domain-containing protein OS=	STIM1	76.41
A5HUL6	MHC class II M alpha chain OS=Gall	DMA	28.373

Q6B7Z6	Exosome complex component RRP45	EXOSC9	49.374
Q90611	72 kDa type IV collagenase OS=Gallu	MMP2	74.94
F1N8X8	Tight junction protein 3(predicted) OS	TJP3	115.06
A0A1D5P7X3	Macrophage-capping protein OS=Gal	CAPG	36.05
A0A1D5PPZ3	Tetraspanin OS=Gallus gallus OX=90	CD82	28.958
F1P1G9	Phosphatidylinositol-glycan biosynthe	--	27.104
P31395	Stathmin OS=Gallus gallus OX=9031	STMN1	17.082
A0A1D5PJA6	3-phosphoinositide-dependent protein	PDPK1	60.318
A0A1D5PHP0	Ubiquitin specific peptidase 19(predic	USP19	150.68
E1BYG4	Cystathionine beta-synthase OS=Gall	CBSL	62.751
A0A3Q2U611	Leuk-A4-hydro_C domain-containing	RNPEP	102.25
E1C7A6	Peptidase_M48 domain-containing pr	OMA1	63.837
A0A3Q2U562	MFS domain-containing protein OS=	--	20.974
F1NIN3	Phosphatidylserine decarboxylase pro	PISD	46.539
A0A1D5PNG4	WASH complex subunit 5 OS=Gallus	KIAA0196	131.14
F1P5J7	Alkylglycerone-phosphate synthase C	AGPS	70.749
E1BQU2	Phosphoacetylglucosamine mutase O	PGM3	59.899
F1NEB6	Cytochrome P450 family 46 subfamil	CYP46A1	57.607
E1BV83	Nucleoporin 205(predicted) OS=Gall	NUP205	228.33
E1BTM6	Aa_trans domain-containing protein (	SLC38A10	135.89
A0A3Q2TUJ1	CLK4-associating serine/arginine rich	--	121.85
Q5ZJ29	Ornithine--oxo-acid aminotransferase	OAT	48.342
A0A3S5ZPB7	Phosphate carrier protein, mitochondr	SLC25A3	39.901
A0A1D5PD98	Complement C5(predicted) OS=Gallu	C5	185.27
Q5ZJK3	Signal transducer and activator of tran	STAT1	87.243
A0A1D5P8A7	Eukaryotic translation initiation facto	EIF2B4	36.716
A0A1D5PFB7	Myosin motor domain-containing pro	MYO7L2	193.01
A0A1D5PSB4	Ubiquitin protein ligase E3 componen	--	109.44
A0A1D5PNT8	VPS10 domain-containing protein OS	SORT1	83.553
Q90733	COUP transcription factor 2 OS=Gall	NR2F2	45.427
A0A3Q2UDS6	Interferon-induced transmembrane pr	LOC107053353	12.319
A0A1D5NW32	Importin N-terminal domain-containi	IPO11	112.14
Q5F3Q6	O-phosphoseryl-tRNA(Sec) selenium	SEPSECS	53.841

E1C5A3	Serine/threonine protein phosphatase	PPP2R5A	55.284
O93417	Caspase-3 OS=Gallus gallus OX=903	CASP3	31.675
E1C7Y6	Foie-gras_1 domain-containing protei	TRAPPC10	143.46
E1C093	Phosphatidylinositol-4,5-bisphosphat	PIK3CG	128.01
A0A3Q2UFB1	SH3 domain-containing protein OS=(	LOC107050955	52.566
Q6WNG8	Heterogeneous nuclear ribonucleopro	HNRNPH2	56.566
A0A3Q2UNM0	HTH La-type RNA-binding domain-c	LARP1	108.48
F1N8U1	Mitochondrial ribosomal protein S36	MRPS36	11.645
F1P574	NTP_transferase domain-containing p	GMPPB	39.435
F1NCH3	HECT domain-containing protein OS	HERC4	86.424
A0A1D5PN24	Protein kinase domain-containing pro	CAMK1	41.213
Q5ZJA5	Signal recognition particle receptor su	SRPRB	28.146
Q5F3A1	Translocation protein SEC62 OS=Ga	SEC62	45.965
F6TYJ6	Metaxin OS=Gallus gallus OX=9031	MTX3	33.783
F1NKL4	Dynein cytoplasmic 1 heavy chain 1(p	DYNC1H1	533.17
E1BWS4	Aminoacyl-tRNA hydrolase OS=Gall	PTRH2	18.29
Q5ZJN1	Replication termination factor 2 OS=(	RTF2	34.222
A0A1D5P927	PHD-type domain-containing protein	RSF1	158.46
Q90929	Nuclear factor 1 (Fragment) OS=Gall	cNFI-A6	42.973
E1BV90	Dopa decarboxylase(predicted) OS=C	DDC	55.028
A0A3Q3AGE2	Scribble planar cell polarity protein(p	--	17.301
A0A3Q2U913	Oxysterol-binding protein OS=Gallus	OSBPL9	81.43
P84023	Mothers against decapentaplegic hom	SMAD3	48.251
A0A3Q2U400	Tankyrase_bdg_C domain-containing	TNKS1BP1	177.5
A0A3Q2U6S8	Oxysterol-binding protein OS=Gallus	OSBPL10	82.236
F1P1M5	ABC1 domain-containing protein OS	ADCK2	69.149
A0A3Q2U525	Trans-2,3-enoyl-CoA reductase(predi	LOC107050163	34.815
Q5ZJV9	CCR4-NOT transcription complex su	CNOT7	32.745
H9KZT7	DZF domain-containing protein OS=(	ILF2	54.304
F1NWK2	Cytochrome c oxidase subunit 7A2(p	COX7A2	9.5963
A0A1D5PKL2	RanBP2-type domain-containing prot	--	60.448
Q5F3H4	Proteasome assembly chaperone 1 OS	PSMG1	31.475
O57389	2',3'-cyclic-nucleotide 3'-phosphodies	CNP	47.236

A0A1D5PNP0	Saposin B-type domain-containing pr	AOAH	72.172
E1BTM0	Inter-alpha-trypsin inhibitor heavy ch	ITIH5	106.96
E1C6A6	AT-hook containing transcription fac	AHCTF1	252.64
Q5ZKA6	WASH complex subunit 1 OS=Gallu	WASHC1	51.682
E1BSN3	Trans-3-hydroxy-L-proline dehydrata	L3HYPDH	38.675
Q5ZIV5	Programmed cell death protein 10 OS	PDCD10	24.701
F1P5L4	Elongator complex protein 1 OS=Gal	IKBKAP	150.4
F1N993	Mtr4 exosome RNA helicase(predicte	SKIV2L2	133.52
F1NW06	Tetraspanin OS=Gallus gallus OX=9(	CD81	26.259
E1BSS3	Eukaryotic translation initiation facto	EIF3A	164.38
F1P1I4	Serine incorporator 3(predicted) OS=	SERINC3	52.488
A0A1D5P2H7	Putative LOC102093731(predicted) (	LOC424740	33.581
Q5ZM62	Atp-binding cassette sub-family f mer	ABCF2	71.243
E1BVC0	Peroxisomal membrane protein PEX1	PEX16	28.005
F1NH08	UDPGT domain-containing protein C	UGT8	61.114
F1NT94	Histone acetyltransferase OS=Gallus	KAT6A	225.54
A0A3Q3A5J9	Cytochrome P450 family 39 subfamil	CYP39A1	52.911
Q5F361	TBC domain-containing protein kinas	TBCK	100.45
F1NC51	Ubiquinol-cytochrome c reductase, cc	UQCR10	7.1261
A0A1D5NWY3	Unconventional myosin-Ic OS=Gallu	MYO1C	120.51
Q92071	Integrin beta OS=Gallus gallus OX=9	ITGB3	86.115
F1NNT7	Brush border myosin IB(predicted) O	MYO1F	126.16
F1NMK5	Fmp27_GFWDK domain-containing	KIAA0100	254.22
A0A1D5PM08	Growth arrest specific 2 like 1(predic	GAS2L1	78.333
F1NTV6	UDP-glucose glycoprotein glucosyltra	UGGT1	172.42
A0A1D5NYS9	Oxysterol-binding protein OS=Gallus	OSBPL11	84.513
P47990	Xanthine dehydrogenase/oxidase OS=	XDH	149.61
A0A1D5PBC6	Asparagine--tRNA ligase OS=Gallus	NARS	64.115
A0A1D5PET1	Non-specific serine/threonine protein	PIK3R4	159.74
R4GLG2	Solute carrier family 25 member 20(p	SLC25A20	32.839
A0A3Q2U9R2	GST N-terminal domain-containing p	--	6.7626
A0A1D5PVE4	Helicase_PWI domain-containing prc	--	69.579
E1C3I5	Solute carrier family 25 member 29(p	SLC25A29	33.101

A0A1D5PDA8	GTPase IMAP family member 5 OS=	GIMAP6	29.053
A5HUJ4	Tripartite motif protein 7 OS=Gallus	TRIM7.2	56.542
F1NK98	Tubulin-specific chaperone D OS=Ga	TBCD	113.68
A0A1D5P0J6	Tryptophan 2,3-dioxygenase OS=Gal	TDO2	55.502
A0A1D5P9L6	J domain-containing protein OS=Gall	DNAJC5	22.097
E1BW14	Family with sequence similarity 126 r	--	13.642
E1C9K0	TPR_REGION domain-containing pr	CTR9	131.35
A0A1D5PUV1	Rho guanine nucleotide exchange fac	ARHGEF12	170.5
E1C6F1	J domain-containing protein OS=Gall	DNAJC12	22.957
A0A3Q2TV38	Filamin (Fragment)(predicted) OS=G	FLNA	43.293
F1NV16	Protein TSSC4 OS=Gallus gallus OX	TSSC4	36.825
P26697	Glutathione S-transferase 3 OS=Gallu	--	26.326
A0A1D5PB12	TruB_N domain-containing protein C	TRUB1	24.806
Q5ZLM0	Parafibromin OS=Gallus gallus OX=9	CDC73	60.697
R4GL60	ENTH domain-containing protein OS	CLINT1	65.991
F1NLF1	Hydroxysteroid dehydrogenase-like p	HSDL1	37.591
F1NSA8	RAP1A, member of RAS oncogene fa	RAP1A	21.001
R4GF68	SZT2 subunit of KICSTOR complex(	SZT2	395.56
A0A3Q2U8W3	TNase-like domain-containing protei	--	24.587
A0A1D5PHT9	GTPase activating protein and VPS9	GAPVD1	160.57
F1NCZ3	Multidrug resistance-associated prote	ABCC13	144.43
F1N804	Sema domain-containing protein OS=	PLXNA1	213.13
Q5ZMR9	Exportin-4 OS=Gallus gallus OX=90	XPO4	130.32
A0A3Q2U068	Heterogeneous nuclear ribonucleopro	HNRNPU	85.564
A0A3Q2UJJ7	Retinol dehydrogenase 12-like(predic	LOC107056277	46.502
F1NB52	Coatomer subunit gamma OS=Gallus	COPG1	97.611
A0A1L1RPY6	Eukaryotic translation initiation facto	EIF3E	52.148
A0A1D5PW25	L-type lectin-like domain-containing	LMAN2	37.538
A0A3Q2UF74	Mannosyl-oligosaccharide glucosidas	MOGS	91.966
E1BUS2	Nop domain-containing protein OS=C	NOP58	59.266
Q5ZHP8	tRNA N(3)-methylcytidine methyltra	METTL2	42.729
E1C358	Thioredoxin domain-containing prote	TXNDC11	104.7
A0A1D5P206	Angiomotin_C domain-containing pr	AMOTL1	106.73

F1NUL5	RAN binding protein 10(predicted) O	RANBP10	66.859
F1NTV5	DUF1279 domain-containing protein	FAM210B	19.807
E1C5G9	Tyrosine aminotransferase OS=Gallus	TAT	50.551
A0A3Q2U4Y9	Non-specific serine/threonine protein	CDC42BPA	190.09
F1P2S9	Gamma-tubulin complex component	TUBGCP3	98.757
A0A1D5PHL1	RNA binding motif protein 14(predic	RBM14	77.306
E1BSN7	Alpha-1,4 glucan phosphorylase OS=	PYGB	96.759
H9L3L2	Coatomer subunit alpha OS=Gallus g	COPA	138.52
Q5F351	MPN domain-containing protein OS=	FAM175B	39.594
Q5ZJZ6	Nuclear cap-binding protein subunit 1	NCBP1	92.563
A0A3Q2U4K3	SEC63 domain-containing protein OS	LOC112530170	26.483
F1NHI3	RNA helicase OS=Gallus gallus OX=	DHX15	87.685
A0A1D5PDG4	GNAS complex locus(predicted) OS=	GNAS	44.382
R4GFT9	Guanine nucleotide-binding protein su	GNG12	8.0904
A0A3Q2U774	Niban apoptosis regulator 2(predicted	FAM129B	92.849
A0A1L1RY74	Mago-bind domain-containing protein	PYM1	23.027
A0A3Q2UK47	Protein transport protein Sec31A OS=	SEC31A	125.15
A0A3Q3AYR7	t-SNARE coiled-coil homology doma	STX5	38.392
E1C4Z6	von Willebrand factor OS=Gallus gal	VWF	312.61
A0A1D5PIG8	V-SNARE coiled-coil homology dom	VAMP4	16.313
F1NNV7	MICOS complex subunit OS=Gallus	APOO	23.914
F1P581	Hexose-6-phosphate dehydrogenase/g	H6PD	89.264
F1NDV3	Nucleoporin 133(predicted) OS=Gall	NUP133	131.51
R4GLS1	ZnF_CDGSH domain-containing pro	CISD2	15.247
E1BRL4	Synaptosomal-associated protein OS=	SNAP23	23.603
F1P4E5	Geranylgeranyl transferase type-2 sub	RABGGTB	36.826
F1NBE3	Aminotran_1_2 domain-containing pr	GCAT	45.197
A0A3Q2U1X8	Microsomal signal peptidase 25 kDa s	--	14.937
A0A1D5P768	TPR_REGION domain-containing pr	NAA25	112.3
E1C3F8	Membrane palmitoylated protein 5(pr	MPP5	77.034
Q5ZL91	Integrator complex subunit 7 OS=Gal	INTS7	106.53
E1BS94	Alpha-L-fucosidase OS=Gallus gallus	FUCA2	52.405
F1NZW9	Solute carrier family 35 member A3(p	SLC35A3	36.078

F1NSQ4	Mitochondrial Rho GTPase OS=Gallu	RHOT1	70.894
A0A3Q3AHG4	Importin N-terminal domain-containi	TNPO1	101.99
A0A3Q2UES1	FERM_M domain-containing protein	--	37.754
A0A1D5PFL9	Nuclear pore complex protein Nup93	NUP93	93.598
F1NJ69	General vesicular transport factor p11	USO1	106.13
A0A3Q2UBF5	Non-specific protein-tyrosine kinase	PTK2	118.98
A0A3Q2TTF9	Vacuolar protein sorting-associated p	VPS11	97.115
Q5ZLB8	Ras-related protein Rab-22A(predicte	RAB22A	21.846
A0A1D5PJH5	Clathrin heavy chain OS=Gallus gallu	CLTCL1	182.81
F1P4X7	7-dehydrocholesterol reductase(predi	DHCR7	55.2
E1BQ44	Complex I-MWFE OS=Gallus gallus	NDUFA1	8.2116
A0A1D5P384	Complex I-B18 OS=Gallus gallus OX	NDUFB7	18.215
A0A3Q3AHL7	eIF-3c_N domain-containing protein	--	16.508
A0A1D5PNS9	6-phosphofructo-2-kinase OS=Gallus	PFKFB4L	59.274
A0A1D5NTM8	Protein-tyrosine-phosphatase OS=Ga	PTPRG	159.8
E1BSI5	THO complex subunit 2 OS=Gallus g	THOC2	170.87
P00789	Calpain-1 catalytic subunit OS=Gallu	--	80.351
A0A3Q2TXP3	Transmembrane protein 181(predicte	TMEM181	54.565
A0A1D5PEE1	Oxysterol-binding protein OS=Gallus	OSBP	95.175
Q5ZIE6	NEDD8-activating enzyme E1 regula	NAE1	60.483
A0A1D5PBD8	Nuclear pore complex protein Nup96	NUP98	192.9
F1P353	Ribosome assembly factor mrt4 OS=	MRTO4	27.357
A0A1D5PMG4	Exportin-7 OS=Gallus gallus OX=90	XPO7	121.47
Q6LC82	GTPase cRac1A OS=Gallus gallus O	cRac1A	21.45
Q5ZIA5	Coatomer subunit beta OS=Gallus ga	COPB1	107.12
A0A1L1RM52	VPS26, retromer complex componen	VPS26A	37.995
E1C645	Complex I-B14.5a OS=Gallus gallus	NDUFA7	12.542
E1BZT5	MPN domain-containing protein OS=	PRPF8	273.63
Q5ZIW8	Ras-related protein R-Ras2(predicted	RRAS	23.237
A0A1D5PJR5	Arginine--tRNA ligase, cytoplasmic	RARS	75.471
F1NEK9	2-amino-3-carboxymuconate-6-semia	ACMSD	36.531
A0A1L1RKT0	Glycylpeptide N-tetradecanoyltransfe	NMT1	56.865
Q5ZKD7	Putative helicase MOV-10 OS=Gallu	MOV10	109.05

E1C8A2	POTRA domain-containing protein C	SAMM50	52.22
A0A1D5P2L3	CULLIN_2 domain-containing protei	CUL1	89.69
F1NBD0	PHB domain-containing protein OS=	ERLIN2	38.216
R4GH36	Small nuclear ribonucleoprotein poly	SNRPB2	25.607
P24802	Procollagen-lysine,2-oxoglutarate 5-d	PLOD1	84.317
P23991	Alcohol dehydrogenase 1 OS=Gallus	ADH1	39.807
A0A1D5PDC1	Clathrin heavy chain OS=Gallus gallu	CLTCL1	190.8
Q9I8D0	V-type proton ATPase 116 kDa subu	ATP6V0A1	95.983
Q5ZKZ6	3-ketodihydrosphingosine reductase(	KDSR	24.536
F1P0I1	DExD/H-box helicase 60(predicted)	DDX60	210.92
F1NPQ0	PCI domain-containing protein OS=C	PSMD12	53.008
Q5ZJH9	H/ACA ribonucleoprotein complex su	DKC1	58.15
E1BRA6	DnaJ homolog subfamily C member 1	DNAJC10	91.494
Q5ZJJ7	Claudin domain containing 1(predicte	CLDND1	27.999
A0A3Q2U7Y6	WAPL domain-containing protein OS	WAPL	130.48
A0A3Q2U8D7	Endoplasmic reticulum junction form	LNPK	47.229
A0A1D5P868	ASCH domain-containing protein OS	TRIP4	65.42
Q5ZJH2	Nicalin OS=Gallus gallus OX=9031	NCLN	62.422
F1NW23	Clathrin heavy chain OS=Gallus gallu	CLTC	191.61
A0A1D6UPU6	Dolichyl-diphosphooligosaccharide--	RPN2	73.598
E1BVT0	Small monomeric GTPase OS=Gallus	RALB	23.348
F1NPH4	VPS26, retromer complex componen	VPS26B	39.272
A0A1D5P822	OCIA domain-containing protein OS=	OCIAD2	27.387
R4GKP0	MARVEL domain-containing protein	MARVELD2	56.932
A0A3Q2TXT4	WH2 domain-containing protein OS=	WASH1	52.529
F1NC11	WD_REPEATS_REGION domain-co	WDR3	105.58
E1BTS3	Electron transfer flavoprotein regulat	ETFRF1	10.303
F1NFQ9	Receptor protein-tyrosine kinase OS=	TEK	114.53
E1C771	FGGY carbohydrate kinase domain co	FGGY	60.069
A0A1D5PG75	Gamma-glutamyltransferase 5(predic	GGT5	60.222
A0A1D5P6D5	Diacylglycerol kinase OS=Gallus gall	DGKA	79.139
A0A1D5NYX9	Putative mitochondrial ribosomal pro	MRPS14	14.979
A0A3Q3ALB3	Mitogen-activated protein kinase OS=	MAPK3	40.416

H9L0G3	Mothers against decapentaplegic hom	LOC769000	47.849
E1C4Y3	Calcineurin B homologous protein 3 (	TESC	25.023
Q5ZI87	Tubulin-specific chaperone D OS=Ga	TBCD	113.66
R4GGG4	Cytochrome P450 family 2 subfamily	CYP2U1	59.518
A0A1D5NT33	WD_REPEATS_REGION domain-co	TBL2	31.068
A0A1D5PS55	Suppressor of tumorigenicity 7 protei	ST7	57.478
R4GJU7	ST6 N-acetylgalactosaminide alpha-2	MXRA7	9.4535
A0A1D5PLS2	Citrate synthase OS=Gallus gallus OX	CS	52.056
E1BRT4	Protein MGR2 homolog OS=Gallus g	ROMO1	8.2428
Q90W83	Aldo-keto reductase OS=Gallus gallu	akr	36.472
E1C7G8	Dimethylaniline monooxygenase [N-(	DM5L	60.445
A0A3Q3APH5	Thromboxane A synthase 1(predicted	TBXAS1	57.317
E1C757	Bile salt export pump OS=Gallus gall	ABCB11	148.57
A0A1D5P629	Nop domain-containing protein OS=(	NOP56	59.832
F1P4D7	PRP4 pre-mRNA-processing factor 4	PRPF4B	126.81
F1NPJ8	Glutathione peroxidase(predicted) OS	GPX3	23.324
F1P1M3	Exocyst complex component 3 OS=G	EXOC3L4	81.201
A0A3Q3A8N6	CUE domain-containing protein OS=	ASCC2	80.196
A0A1L1RZT1	Plasmalemma vesicle-associated prot	PLVAP	47.011
A0A1D5PXS5	TPR_REGION domain-containing pr	TRAPPC12	82.498
F1N992	Lipid phosphate phosphohydrolase 1	PLPP1	31.806
A0A1D5PY21	FUN14 domain containing 2(predicte	FUNDC2	19.94
F1NDU7	Transmembrane serine protease 6(pre	TMPRSS6	86.108
Q71RI7	Guanine nucleotide-binding protein G	GNA11	42.058
A0A3Q2UIZ8	Histone deacetylase 10(predicted) OS	--	24.864
A0A1D5NZ43	Ceramide synthase 5(predicted) OS=(	CERS5	45.515
F1P1C3	SEC7 domain-containing protein OS=	IQSEC1	130.88
A0A1D5PH79	EH domain containing 2(predicted) C	LOC107049991	60.561
A0A1D5P5V5	C4a anaphylatoxin OS=Gallus gallus	C4	183.3
A0A1D5NV65	Phosphoglucomutase 2(predicted) OS	PGM2	68.034
A0A1D5PJ54	Metadherin(predicted) OS=Gallus gal	--	14.166
A0A3Q3A502	Solute carrier organic anion transport	SLCO1B3	79.141
A0A3Q2UAF4	Nuclear GTPase SLIP-GC (Fragment	C3H8ORF80	43.642

A0A1D5PCD1	Flotillin OS=Gallus gallus OX=9031	FLOT2	47.236
F1NJ94	PHB domain-containing protein OS=	ERLIN1	38.907
E1BYB2	ANK_REP_REGION domain-contain	KIDINS220	195.43
A0A1L1RN08	L-type lectin-like domain-containing	LMAN1L	56.518
A0A1D5NXL7	PCI domain-containing protein OS=C	PSMD3	59.352
A0A3Q2U3C3	Transmembrane 9 superfamily memb	TM9SF3	63.018
Q5ZMG8	Rho-related GTP-binding protein Rho	RHOGL	21.28
A0A1D5P4Y9	FA_desaturase domain-containing pro	FADS6	39.526
A0A1D5P1T0	Small monomeric GTPase OS=Gallus	RALA	23.548
F1N8B6	Inositol-polyphosphate 5-phosphatase	INPP5K	53.727
S5TQ07	Zinc finger protein 36 C3H1 type-like	Zfp36l1	36.02
A0A1D5PW58	Arf-GAP domain-containing protein	ARFGAP3	61.839
A0A1D5PND5	Phosphorylase b kinase regulatory sul	PHKB	113.76
Q5ZLB2	ADP-ribosylation factor-like 6-intera	ARL6IP1	23.566
E1BVB0	ADP ribosylation factor like GTPase	ARL1	20.369
E1BYQ3	CULLIN_2 domain-containing protei	CUL3	88.974
F1P0L2	Transmembrane 9 superfamily memb	TM9SF2	74.257
E1BQJ4	C2 domain-containing protein OS=Ga	C2CD5	125.68
A0A3Q2TXJ3	RanBD1 domain-containing protein(p	--	20.187
A0A1D5P2W8	TOG domain-containing protein OS=	RANBP6	89.238
A0A1D5PLU6	Zw10 kinetochore protein(predicted)	ZW10	88.564
A0A3Q3AXS8	2-hydroxyacyl-CoA lyase 2 OS=Gallu	ILVBL	70.886
A0A3Q2U005	AIG1-type G domain-containing prot	LOC112531693	49.177
P50147	Guanine nucleotide-binding protein C	GNAI2	40.577
E1C8U1	Nucleoporin NUP53 OS=Gallus gallu	LOC107056600	34.491
F1NEI8	Poly(A)-specific ribonuclease PARN	PARN	73.168
E1C6X2	Fibronectin type-II domain-containing	SEL1L	88.375
A0A1D5P7Q4	Coronin OS=Gallus gallus OX=9031	CORO7	128.59
H9L0X6	2'-5' oligoadenylate synthase OS=Gal	OASL	55.266
F1ND55	Aldolase_II domain-containing protei	ADD1	81.003
P09653	Tubulin beta-5 chain OS=Gallus gallu	--	49.971
A0A1D5P2V5	Cation-transporting ATPase(predicted	ATP13A1	139.9
A0A3Q3APV9	Transmembrane protein 214(predicted	TMEM214	66.982

A0A3Q3ATB4	LYR motif-containing protein 4(predi	LYRM4	10.434
F1NH69	Bms1-type G domain-containing prot	BMS1	148.1
A0A1D5PLR9	Protein-tyrosine-phosphatase OS=Ga	PTPRK	161.89
A0A1D5NVP8	Pleckstrin homology, MyTH4 and FE	PLEKHH1	158.4
F1NE58	Geranylgeranyl transferase type I subu	PGGT1B	41.789
E1C0F1	Dolichyl-diphosphooligosaccharide--]	RPN1	77
A0A1D5PU80	Sodium/potassium-transporting ATPa	ATP1A1	112.46
F1NPN5	SERPIN domain-containing protein C	SPIA3	47.58
A0A3Q3B0A5	IRF-2BP1_2 domain-containing prote	IRF2BP2	98.581
Q5ZM72	Fatty acyl-CoA reductase 1 OS=Gallu	FAR1	59.449
P79996	Mitogen-activated protein kinase 9 O	MAPK9	44.008
A0A1L1RTX2	C-type lectin domain-containing prote	CLC2DL5	26.585
A0A1D5NVY0	Up-regulated during skeletal muscle g	USMG5	6.7187
Q5ZLL0	Vacuolar ATPase assembly integral n	VMA21	11.578
A0A1D5PN07	Rho GTPase activating protein 1(prec	ARHGAP1	50.4
Q5ZMG2	Claudin OS=Gallus gallus OX=9031	CLDN1	22.626
F1P418	Diphthine methyl ester synthase OS=(	DPH5	31.846
A0A1D5NVP6	Neuronal pentraxin receptor(predicted	NPTXR	50.736
F1P040	HGF/SF receptor OS=Gallus gallus C	MET	154.38
A0A3Q2TXN1	Zona pellucida sperm-binding protein	--	49.726
F1NPA9	DNA-(apurinic or apyrimidinic site) l	RPS3	26.719
Q5ZL38	SAGA-associated factor 29 OS=Gallu	SGF29	33.111
E1C764	Protein kinase domain-containing pro	BMP2K	124.12
A0A1D5PLU5	Tetratricopeptide repeat domain 31(pı	TTC31	57.036
A0A1D5PWB9	AP-3 complex subunit delta OS=Gall	AP3D1	101.23
A0A1D5PX68	Solute carrier family 25 member 1(pr	SLC25A1	33.907
F1NEU9	TBC1 domain family member 24 OS=	TBC1D24	62.991
A0A3Q2TTG9	Membrane-spanning 4-domains subfa	--	22.639
A0A1D5PBF6	FLII actin remodeling protein(predict	FLII	140.64
A0A1L1RXM8	GDT1 family protein OS=Gallus gall	TMEM165	34.107
F1NYM3	Complex I-20kD OS=Gallus gallus O	NDUFS7	24.29
E1BRC0	Vacuolar protein sorting-associated p	VPS54	110.45
F1NNV2	AMP-binding domain-containing pro	ACSL5	75.606

Q5ZHM5	Protein RER1 OS=Gallus gallus OX=	RER1	23.032
Q5ZMW3	Apoptosis inhibitor 5 OS=Gallus gall	API5	58.608
A0A1L1RJP3	Solute carrier organic anion transport	SLCO4A1	62.929
R4GI80	Synaptopodin(predicted) OS=Gallus g	SYNPO	117.82
F1NCU9	Paraoxonase OS=Gallus gallus OX=9	PON1	39.334
A0A1D5NTU6	Protein argonaute-2 OS=Gallus gallus	AGO2	97.831
A0A1L1RME0	Death domain-containing protein OS=	THOC1	76.36
Q5ZMD1	14-3-3 protein theta OS=Gallus gallus	YWHAQ	27.782
E1C959	Inhibitor of nuclear factor kappa-B ki	CWF19L1	55.976
A0A1D5NWJ7	Cytochrome c oxidase subunit 7A2 li	COX7A2L	12.614
A0A1D5P039	Rab-GAP TBC domain-containing pr	TBC1D5	89.293
A0A1D5P1L0	SH3 domain-containing protein OS=C	BCAR1	98.897
E1BWM5	FUN14 domain-containing protein 1 (	FUNDC1	17.279
A0A3Q3AJC5	Receptor protein-tyrosine kinase OS=	MST1R	141.76
A0A1D5PGQ5	AdoHcyase_NAD domain-containing	AHCYL1	61.573
Q5ZII8	Tetraspanin OS=Gallus gallus OX=90	CD151	28.636
E1C530	Centrosomal protein 78(predicted) OS	CEP78	88.44
P02707	Hepatic lectin OS=Gallus gallus OX=	--	24.216
F1NWX1	Alpha-1,3/1,6-mannosyltransferase A	ALG2	46.659
Q6DV79	Signal transducer and activator of tra	STAT3	88.17
Q5F4C5	Ras-related protein Rab-35(predicted)	RAB35	23.125
Q5ZJ69	AT-rich interactive domain-containin	ARID5B	132.72
E1BQM9	Dpy-19 like C-mannosyltransferase 3	DPY19L3	77.158
F1NIK3	Developmentally regulated GTP bind	DRG2	40.728
Q5ZMJ6	ADP/ATP translocase OS=Gallus gal	SLC25A4	32.968
E1BVW0	Protein kinase domain-containing pro	CSNK1D	46.762
A0A3Q3A5B2	Lipase maturation factor 1(predicted)	--	18.808
A0A3Q2U3M9	Round spermatid basic protein 1 like(	RSBN1L	53.181
A0A1D5PL17	Poly [ADP-ribose] polymerase OS=G	PARP4	181.46
A0A1D5P6G2	N-alpha-acetyltransferase 40 OS=Gal	NAA40	27.164
F1NVX7	Transmembrane 9 superfamily memb	TM9SF4	77.3
A0A1D5PY29	SCD domain-containing protein OS=	STAG2	144.52
Q5ZKX9	ER lumen protein-retaining receptor 2	KDELR2	24.456

A0M8T8	Caveolin OS=Gallus gallus OX=9031	CAV1	20.468
Q06066	Y-box-binding protein 1 OS=Gallus g	YBX1	35.799
F1P5U6	Cytochrome P-450(predicted) OS=Ga	CYP2C18	55.702
Q5F4C4	Leucine-rich repeat protein SHOC-2 (	SHOC2	59.162
F1NF25	Neutral cholesterol ester hydrolase 1(	NCEH1	46.154
F1NHL2	TIP120 domain-containing protein OS	CAND1	136.36
R4GHH2	WASH complex subunit 4(predicted)	WASHC4	136.04
A0A1D5PS95	RING-type domain-containing protei	RNF213	589.25
E1C6G9	NCK associated protein 1(predicted)	NCKAP1	128.74
R4GJH2	Complex I-B15 OS=Gallus gallus OX	NDUFB4	18.701
P13731	Serpin H1 OS=Gallus gallus OX=903	SERPINH1	45.682
F1NM78	Kynurenine 3-monooxygenase OS=G	KMO	55.77
E1BU39	Acyl-coenzyme A thioesterase 1 (Fra	LOC423222	43.339
A0A1D5NUX8	Solute carrier family 25 member 10(p	SLC25A10	35.382
R4GFY2	Thioredoxin domain-containing prote	TMX4	41.43
Q5F3V3	Sister chromatid cohesion protein PD	PDS5A	150.13
P53488	Actin-related protein 2 OS=Gallus ga	ACTR2	44.701
E1BQH1	Folliculin OS=Gallus gallus OX=903	FLCN	64.849
Q5ZLM1	YTH domain-containing protein OS=	YTHDF3	63.56
P19966	Transgelin OS=Gallus gallus OX=90	TAGLN	22.314
Q25QX5	BASH/BLNK N-terminal associated	BNAS1	26.713
Q5F398	E3 ubiquitin-protein transferase MAE	MAEA	45.388
A0A452J801	DnaJ homolog subfamily C member	DNAJC16	89.281
A0A1D5P5J0	FoP_duplication domain-containing p	CHTOP	26.154
F1NKR5	WD_REPEATS_REGION domain-co	WDR36	98.286
A0A1D5PD02	UBA_e1_C domain-containing protei	--	53.351
Q5ZL74	Vesicle-associated membrane protein	VAMP7	24.681
F1NIL3	ATP synthase subunit OS=Gallus gal	ATP5L	11.855
F1NNN4	Cytochrome P450 4B1-like(predicted	CYP4A22	58.696
Q5ZJ96	Aminotran_1_2 domain-containing pr	SPTLC2	62.735
Q5ZIU9	Zinc transporter ZIP9 OS=Gallus gall	SLC39A9	32.191
E1C784	Calcium-transporting ATPase OS=Ga	ATP2B4	129.51
E1BYW5	Cytochrome P450 family 4 subfamily	CYP4V2	61.514

F1NKL7	Holliday junction recognition protein	HJURP	76.044
A0A1D5PSQ6	Cytochrome P450 2C8(predicted) OS	LOC101749846	73.332
Q5ZI00	Epimerase domain-containing protein	NDUFA9	43.079
Q5ZK03	Protein transport protein Sec23A OS=	SEC23A	86.303
Q5ZHZ0	Spliceosome RNA helicase DDX39B	DDX39B	49.003
A0A1D5PQN0	Catenin beta 1(predicted) OS=Gallus	CTNNB1	78.529
A0A1D5NVZ1	Family with sequence similarity 120A	FAM120C	122.65
A0A1D5PZG1	DAO domain-containing protein OS=	DAO	39.334
Q800L1	Surfeit locus protein 1 OS=Gallus gal	SURF1	35.21
R4GI86	Calcium-binding protein 39(predicted	CAB39	39.899
A0A1D5PN05	COX6C domain-containing protein C	COX6C	8.7914
A0A1D5PA09	V-type proton ATPase subunit C OS=	ATP6V1C1	44.14
A0A1D5PHR1	ATP binding cassette subfamily D me	ABCD2	82.228
E1C279	3Beta_HSD domain-containing prote	NSDHL	38.925
Q5ZI04	CYFIP-related Rac1 interactor A OS=	CYRIA	37.313
A0A3Q2UB17	Polypeptide N-acetylgalactosaminyltr	GALNT2	64.417
A0A3Q2TUW7	Cell division control protein 42 homo	CDC42	21.703
E1BV75	Dehydrogenase/reductase 7(predicted	DHRS7	37.72
P28687	Signal peptidase complex subunit 3 C	SPC22	20.23
Q5F337	Ras-related C3 botulinum toxin subst	RAC2	21.418
A0A1L1RIZ2	Lysophosphatidylcholine acyltransfer	LPCAT2	73.498
A0A1D5PQF7	E2F_TDP domain-containing protein	E2F8	81.083
F1NCC7	Fatty acid hydroxylase domain-contai	AGMO	57.982
F1NAM6	GB1/RHD3-type G domain-containin	ATL1	62.964
E1BVP6	Heat shock protein family A (Hsp70)	HSPA12A	74.675
F1NM57	Acyl-CoA-binding domain-containing	ACBD5	54.457
A0A3S5ZPT2	Cytohesin-1 isoform A(predicted) OS	CYTH1	46.314
Q5ZK61	Complex I-SGDH OS=Gallus gallus (	NDUFB5	21.357
A0A3Q2TZC7	Solute carrier family 40 protein OS=(	SLC40A1	57.32
A0A1D5PTH2	Cytochrome P450 family 20 subfamil	CYP20A1	52.427
A0A1D5NZ71	J domain-containing protein OS=Gall	DNAJC13	255.09
A0A1L1RWI4	Cytochrome P450 2K1 (Fragment)(pr	CYP2AC2	56.972
A0A1D5P4S8	6-phosphofructokinase OS=Gallus ga	PFKP	91.57

A0A3Q2UB31	Protocadherin 1(predicted) OS=Gallu	PCDH1	121.31
R4GMB6	Farnesyl pyrophosphate synthase OS=	FDPS	40.45
F1NNS1	Dolichyl-diphosphooligosaccharide--]	DDOST	48.765
A0A1D5P5L5	Methyltransf_11 domain-containing p	LOC107048987	30.318
A0A1D5PXL0	Beta-galactosidase OS=Gallus gallus	GLB1	73.445
F1NDA9	AP-1 complex subunit gamma OS=G	AP1G1	91.412
A0A1L1RZC3	Ig-like domain-containing protein OS	VSIG10L	48.388
F1NYW8	Fumarylacetoacetase OS=Gallus gallu	FAH	46.548
A0A3Q3AEK7	Myosin ID(predicted) OS=Gallus gall	MYO1D	112.43
F1P2B1	Torsin OS=Gallus gallus OX=9031 G	TOR1A	39.222
Q5ZIN0	Lysophosphatidylserine lipase ABHD	ABHD12	43.855
A0A1D5P4Q8	Jagunal homolog 1(predicted) OS=Ga	TTLL8	20.853
A0A1D5P4N1	Solute carrier family 41 member 3(pr	SLC41A3	48.612
P62758	Neurocalcin-delta OS=Gallus gallus (	NCALD	22.261
Q5ZHP3	Cilia- and flagella-associated protein	CFAP20	22.734
A0A1L1RJ89	CAAX prenyl protease OS=Gallus ga	ZMPSTE24	53.532
Q5ZIZ0	6-phosphogluconate dehydrogenase, (	PGD	53.292
A0A1D5PL84	Leucyl and cystinyl aminopeptidase(p	LNPEP	115.35
Q5ZJ26	Tankyrase_bdg_C domain-containing	RCJMB04_21h11	35.803
A0A1D5PDV1	Transportin 3 OS=Gallus gallus OX=	--	64.092
A0A3Q2UAY3	FXYD domain-containing ion transpo	LOC112530976	9.0033
A0A3Q2UIA4	SH2 domain-containing protein OS=(	STAT6	21.083
A0A1D5P1I5	Anoctamin OS=Gallus gallus OX=90	ANO1	117.31
Q5ZM59	FXYD domain-containing ion transpo	FXYD6	10.212
Q5ZKB0	GOLD domain-containing protein OS	TMED2	22.602
F1NUR7	DnaJ heat shock protein family (Hsp4	DNAJC1	62.73
F1NB92	Aminopeptidase OS=Gallus gallus O	ERAP1	102.45
R9PXN6	Cochlin OS=Gallus gallus OX=9031	COCH	59.428
A6NAB8	Delta-6 fatty acid desaturase OS=Gal	FADS2	51.99
F1NTZ6	DUF3504 domain-containing protein	KIAA1958	79.495
A0A3Q2TYK9	Calcium uniporter protein OS=Gallus	MCU	34.679
A0A1D5PMA0	Solute carrier organic anion transport	SLCO1A2	72.21
E1C7K7	N-acetylated alpha-linked acidic dipe	NAALAD2	83.626

Q5F428	Eukaryotic translation initiation facto	EIF3L	66.682
F1NEX4	OGFr_N domain-containing protein (	OGFR	80.192
F1NL85	Dolichyl-diphosphooligosaccharide--]	MAGT1	36.776
A0A1D5PGF0	Growth hormone inducible transmem	GHITM	36.579
F1NV57	Protein tyrosine phosphatase non-rece	PTPN9	66.206
Q5ZIW5	Protein VAC14 homolog OS=Gallus	VAC14	87.822
F1NCU8	DnaJ homolog subfamily C member 2	DNAJC25	41.592
F1NTH9	MFS domain-containing protein OS=	SLC37A4	48.703
Q90847	G protein alpha subunit OS=Gallus ga	Galphai3	40.517
Q5F470	Ras-related protein Rab-8A OS=Gallu	RAB8A	23.522
Q5ZKJ6	CD200 molecule(predicted) OS=Gall	CD200	28.259
E1C2C3	SF3b1 domain-containing protein OS	SF3B1	145.72
A0A1L1RZ64	3-hydroxy-3-methylglutaryl coenzyme	HMGCS1	57.668
R4GFF1	UPF0235 protein C15orf40 (Fragmen	--	16.173
A0A1D5P106	STEAP3 metalloreductase(predicted)	STEAP3	54.631
A0A1L1RNG8	Lysophosphatidylcholine acyltransfer	LPCAT3	57.214
P50146	Guanine nucleotide-binding protein C	GNAI1	40.378
F1NZ23	DNA excision repair protein ERCC-3	ERCC3	89.061
A0A3Q3AU66	Fatty acid 2-hydroxylase OS=Gallus g	FA2H	39.463
Q5F4B6	RRM domain-containing protein OS=	POLDIP3	45.914
A0A1D5PQK3	STE20-related kinase adapter protein	STRADA	41.685
E1BW50	DUF1716 domain-containing protein	CTNNBL1	65.153
R4GGF3	SCP domain-containing protein OS=(	GLIPR2	16.922
B3TZC1	PNPLA7 OS=Gallus gallus OX=9031	PNPLA7	147.73
A0A1D5P648	Retinol dehydrogenase 16 (Fragment)	RDH16	45.432
A0A1D5PTW3	Hydroxysteroid 17-beta dehydrogenas	HSD17B7	33.832
A0A1D5PV96	PIPK domain-containing protein OS=	PIP4K2B	47.422
A0A3Q2UEC2	Golgin subfamily B member 1(predic	--	62.439
F1P3T3	Mitochondrial fission regulator 1 like	MTFR1L	31.439
E1C5V6	Acyl-coenzyme A oxidase OS=Gallus	ACOX2	75.493
P26990	ADP-ribosylation factor 6 OS=Gallus	ARF6	20.096
A0A1L1RKB7	Calcium-transporting ATPase OS=Ga	ATP2A2	114.78
A0A1D5PGF6	TLC domain-containing protein OS=(	TRAM1L1	57.818

A0A3Q2U0C6	Dual specificity phosphatase 22(predi	--	12.037
E1BTN3	Ras-associating domain-containing pı	RASSF7	49.883
Q9PSX7	Rho-related GTP-binding protein Rhc	RHOC	21.973
A0A3Q2UIM7	Alpha-mannosidase OS=Gallus gallu:	MAN2A1	131.99
A0A1D5NXR2	Endoplasmic reticulum transmembrai	BCAP31	27.228
E1BUU9	Dolichyl-phosphate beta-glucosyltran	ALG5	37.259
E1C0X6	Iodotyrosine dehalogenase 1 OS=Gal	IYD	32.055
P47826	60S acidic ribosomal protein P0 OS=	RPLP0	34.285
E1BQZ4	TGc domain-containing protein OS=(	EPB42	79.196
F1NE33	Formimidoyltetrahydrofolate cyclode	FTCD	59.183
P67962	Casein kinase I isoform alpha OS=Ga	CSNK1A1	38.9
F1P2P3	Cysteinyl-tRNA synthetase OS=Gallu	CARS2	52.711
F6R8U4	Guided entry of tail-anchored protein:	WRB	19.158
A0A3Q2UBH5	Dehydrogenase/reductase 7B(predicte	DHRS7B	33.823
F1NLT1	RNA polymerase II associated proteir	RPAP1	160.67
F1NA76	GOLD domain-containing protein OS	TMED10	24.803
E1C5H3	SUN domain-containing protein OS=	--	22.347
Q5ZHX1	Ras-related protein Rap-1b OS=Gallu	RAP1B	20.825
A0A1D5Q007	Dolichyl-phosphate-mannose--proteir	POMT2	79.857
F6UZR6	3-beta-hydroxysterol Delta (14)-reduc	LBR	73.37
A0A1L1RZ69	Lanosterol 14alpha-demethylase (Fra	CYP51A1	55.952
A0A1D5P6U6	SEC7 domain-containing protein OS=	ARFGEF1	208.29
P21872	Trifunctional purine biosynthetic prot	GART	106.54
A0A1D5PTN0	60S ribosome subunit biogenesis prot	NIP7	18.137
A0A3Q2TYD3	Protein kinase domain-containing pro	MYLK4	79.474
E1C2I3	Endoplasmic reticulum-golgi interme	ERGIC1	32.693
F1P010	Actin-related protein 2/3 complex sul	ARPC4	19.706
A0A3Q3AC89	N6-L-threonylcarbamoyladenine synt	--	14.91
F1NMB3	UDP-glucuronosyltransferase OS=Ga	UGT2A1	60.643
A5HUL4	MHC class II beta chain 2 OS=Gallus	BLB2	29.088
E1BYN0	Sulfatase domain-containing protein (	ARSD	66.495
Q5ZLH1	RPA_C domain-containing protein O	RPA2	28.863
E1BV44	Chromosome segregation 1-like prote	CSE1L	110.44

E1BQV4	ER membrane protein complex subur	EMC3	30.099
E1C796	Transformation/transcription domain	TRRAP	435.11
A0A1D5PM44	Regulator of microtubule dynamics 3	RMDN3	51.694
E1C488	XPG_I_2 domain-containing protein	FAM120B	78.351
F1NI39	Transmembrane protein 87A(predicte	TMEM87B	60.614
A0A1D5P2K7	Mediator complex subunit 23 OS=Ga	--	157.66
F1NE32	Motile sperm domain-containing prot	MOSPD1	24.424
Q5ZLT7	Basic leucine zipper and W2 domain-	BZW1	48.019
A0A1D5NUY6	NADH dehydrogenase [ubiquinone] 1	NDUFC2	12.774
A0A1L1RIN1	Calcium load-activated calcium chan	TMCO1	21.296
A0A1D5NTZ8	Leucyl-tRNA synthetase OS=Gallus g	LARS2	104.42
F1NPV8	Bystin like(predicted) OS=Gallus gall	BYSL	48.773
E1C297	Microsomal glutathione S-transferase	MGST3	15.594
Q5ZKJ0	Cleft lip and palate transmembrane pr	CLPTM1L	61.648
E1BQX4	Secretory carrier-associated membran	SCAMP4	26.104
A0A3Q2UCU2	6.8 kDa mitochondrial proteolipid (Fr	C14orf2	6.2372
A0A1D5P2Q1	SAC domain-containing protein OS=	SACM1L	67.45
F1NQ46	Amidase domain-containing protein (	FAAHL	57.07
A0A1D5PJA5	Dipeptidyl peptidase 4 OS=Gallus ga	DPP4	86.395
A0A1L1RUV1	Udp-glucuronosyltransferase 1-8-like	--	38.001
A0A3Q2UCZ2	Huntingtin(predicted) OS=Gallus gall	HTT	342.46
F1NZQ9	Histidine ammonia-lyase OS=Gallus	HAL	72.965
P02640	Villin-1 OS=Gallus gallus OX=9031	VIL1	92.478
A0A1D5PTS7	FERM domain-containing protein OS	--	65.623
A0A3Q3B220	Tetraspanin OS=Gallus gallus OX=9(	CD63	24.306
A0A1D5P3Y2	Serine/threonine-protein phosphatase	PPP3CA	53.094
A0A3Q2UCG1	Heparan sulfate proteoglycan 2(predi	HSPG2	408.94
E1BYN2	Zinc finger AN1-type containing 6(pr	ZFAND6	22.749
F1NN08	ADP-ribosylation factor 1 (Fragment)	ARF1	20.697
F1ND46	RING-type domain-containing protei	RNF175	37.763
A0A1D5PLV3	cAMP-dependent protein kinase OS=	PRKACB	39.367
A0A3Q2U787	Reticulon OS=Gallus gallus OX=903	RTN3	25.988
F1P5V9	Parvin alpha(predicted) OS=Gallus ga	PARVA	38.396

A0A3Q2TWQ7	VWFD domain-containing protein OS	--	30.028
Q5ZLG7	ADP/ATP translocase OS=Gallus gal	SLC25A6	32.748
A0A1D5P8P6	5'-AMP-activated protein kinase subu	PRKAG1	31.329
A0A452J822	PRA1 family protein OS=Gallus gallu	ARL6IP5	21.564
E1BYF1	Cystathionine gamma-lyase OS=Gallu	CTH	43.898
A0A1D5PXK5	Calcium-transporting ATPase OS=Ga	ATP2B2	138.29
F1NGM1	Phosphatidylinositol-3,5-bisphosphate	MTMR1	74.21
E1C4N0	S10_plectin domain-containing prote	RPS10	18.91
A0A1D5PAE8	Leukotriene A(4) hydrolase OS=Gallu	LTA4H	76.639
A0A1D5P553	Aldehyde dehydrogenase OS=Gallus	ALDH3B2	55.225
E5G6H7	ADP-ribosyl cyclase/cyclic ADP-ribo	BST1	35.528
F1NXB0	Procollagen-lysine 5-dioxygenase OS	PLOD2	81.272
Q5ZMF8	DIRP domain-containing protein OS=	LIN9	51.344
A0A1D5PL70	HEMCAM(predicted) OS=Gallus gal	MCAM	69.001
R4GIH6	Aa_trans domain-containing protein (	SLC38A3	55.561
A0A3Q2TT45	Adaptin_N domain-containing protei	--	17.617
A0A1D5PXY6	Tripartite motif containing 23(predict	TRIM23	73.859
A0A1D5P8H7	Importin N-terminal domain-containi	XPO1	123.16
A0A1D5PJ47	Alpha-1,6-mannosyl-glycoprotein 2-b	MGAT2	52.848
F1NT14	Glutathione transferase OS=Gallus ga	MGST1	17.284
E1BTF7	1-acyl-sn-glycerol-3-phosphate acyltr	AGPAT2	23.407
F1NWX7	Protein transport protein Sec61 subun	SEC61B	10.014
Q5ZK57	Hexosyltransferase OS=Gallus gallus	B3GNT2	45.971
E1C667	Ladinin 1(predicted) OS=Gallus gallu	LAD1	44.802
A0A3Q2UKY5	Transmembrane protein 82 OS=Gallu	--	34.909
A0A1D5PGC1	F5/8 type C domain-containing protei	AEBP1	136.3
R4GKC8	PDZ and LIM domain protein 7 OS=(	PDLIM7	46.011
A0A3Q3B1P7	Anoctamin OS=Gallus gallus OX=90	ANO1	112.98
Q5ZJL1	Polypeptide N-acetylgalactosaminyltr	GALNT1	64.072
F1N859	Transmembrane protein 97 OS=Gallu	TMEM97	19.762
F1NSL8	Phosphatidylinositol glycan anchor bi	PIGS	60.237
A0A1D5P888	Serine/threonine-protein phosphatase	PPP1CA	39.101
F1NKR0	GDNF family receptor alpha 3(predic	GFRA3	47.443

A0A1D5PSP0	Solute carrier family 25 member 22(p	SLC25A22	34.774
E1BYE9	Elongation of very long chain fatty ac	ELOVL2	34.632
Q5ZIR9	E3 ubiquitin-protein ligase RNF185 (	RNF185	20.828
A0A3Q2UD21	JC7(predicted) OS=Gallus gallus OX	NUCKS1	32.625
A0A3Q2TW68	AMP deaminase OS=Gallus gallus O.	AMPD2	101.83
Q5ZMQ3	Signal peptidase complex catalytic su	SEC11A	20.631
A0A1D5PK00	Histocompatibility minor 13(predicte	HM13	63.517
E1BT94	Complex I-B17 OS=Gallus gallus OX	NDUFB6	14.917
A0A1D5PCU1	ADP/ATP translocase OS=Gallus gal	SLC25A5	32.864
Q5ZKF4	High mobility group protein 20A OS=	HMG20A	40.12
A0A3Q3AUY5	Secretory carrier-associated membran	SCAMP3	32.436
F1NQY0	Beta globin protein 1(predicted) OS=	VEZF1	61.141
Q5F3N4	Disintegrin and metalloproteinase doi	ADAM10	84.611
A0A1D5PLG6	VIR_N domain-containing protein OS	KIAA1429	202.92
A0A3Q2UC30	Periostin(predicted) OS=Gallus gallus	POSTN	92.76
Q5ZMP6	AP-2 complex subunit mu OS=Gallus	AP2M1	49.389
A0A1D5PJW1	Dimethylaniline monooxygenase [N-(	FMO3	60.291
A0A1D5PL92	Ig-like domain-containing protein(pre	TIMD4	59.999
F1NM98	Protein phosphatase 1 regulatory subu	--	35.06
Q90927	Nuclear factor 1 OS=Gallus gallus O.	cNFI-A4	54.643
F1NX01	Polypeptide N-acetylgalactosaminyltr	GALNT10	63.83
A0A1D5PNK7	Calcium-transporting ATPase OS=Ga	ATP2C1	105.4
A0A3Q2UHW3	Guanine nucleotide-binding protein s	--	42.52
E1C7F5	Acetyl-coenzyme A transporter 1(pre	SLC33A1	65.524
F1NZY9	NADH-cytochrome b5 reductase OS=	CYB5R1	34.088
P51641	Ciliary neurotrophic factor receptor su	CNTFR	40.307
P79762	Zona pellucida sperm-binding protein	ZP3	46.765
R4GL09	MRC1L-B protein(predicted) OS=Ga	MMR1L4	165.52
E1BTV1	PHB domain-containing protein OS=	STOM	30.813
O73888	Hematopoietic prostaglandin D synth	HPGDS	22.73
E1BTT1	Signal-regulatory protein(predicted) (	TPPNR1L	69.252
E1C5U6	Villin-1 OS=Gallus gallus OX=9031	VILL	97.229
A0A1D5PFT8	ENTH domain-containing protein OS	PICALM	66.254

Q5ZMC2	Unconventional myosin-Ig OS=Gallu	MYO1G	114.97
A0A1L1RZL5	DZF domain-containing protein OS=(	ILF2	45.408
F1NF85	Phosphatidylethanolamine N-methylt	PEMT	26.597
A0A1D5PSW5	Protein MON2 homolog OS=Gallus g	MON2	188.91
Q5ZMR3	Protein-L-isoaspartate O-methyltrans	PCMTD1	40.923
Q75R65	Tyrosine-protein kinase JAK2 OS=Ga	JAK2	129.85
Q5ZLG8	Transmembrane anterior posterior tra	TAPT1	65.775
A0A3Q2UJM6	Malectin domain-containing protein (	MLEC	27.708
A0A1D5P4J2	ERGIC and golgi 3(predicted) OS=Ga	ERGIC3	43.557
E1BTL3	Retinol dehydrogenase 12(predicted)	RDH11	36.068
A0A1D5PP23	ELMO domain-containing protein OS	ELMO3	82.611
A0A3Q2U252	Pyr_redox_2 domain-containing prote	--	42.256
E1C8L7	Leukotriene C4 synthase(predicted) (	LTC4SL	16.364
F1P1M1	UDP-glucuronosyltransferase OS=Ga	UGT1A1	59.519
E1C1V3	Junction plakoglobin OS=Gallus gallu	JUP	82.125
A0A1D6UPT6	Complex I-MNLL OS=Gallus gallus	NDUFB1	6.9219
F1NYA2	Eukaryotic translation initiation facto	EIF4H	27.635
A0A1L1RKY9	Caldesmon OS=Gallus gallus OX=90	CALD1	60.154
E1C4U7	Complex I-B12 OS=Gallus gallus OX	NDUFB3	11.154
A0A1D5PRA3	Signal peptidase complex catalytic su	SEC11C	20.234
Q5ZLA7	Dolichyl-diphosphooligosaccharide--]	STT3A	80.595
R4GKS2	acidPPc domain-containing protein O	PLPP2	59.183
F1NTK1	Phosphoserine aminotransferase OS=	PSAT1	40.544
A0A1D5PJL3	Amino_oxidase domain-containing pr	MAOA	57.99
F1P151	Clat_adaptor_s domain-containing pr	AP3S1	21.595
E1BTE8	Chromosome 1 open reading frame 2	C8H1ORF21	13.855
F1NZJ2	Hexokinase OS=Gallus gallus OX=9(	HK1	86.985
F1NV09	Epithelial cell adhesion molecule OS=	EPCAM	33.611
A0A1D5NY13	AIP3 domain-containing protein OS=	SRCIN1	128.62
Q5ZM18	Nucleolar GTP-binding protein 1 OS=	GTPBP4	73.934
Q5ZKX0	Dolichyl-diphosphooligosaccharide--]	STT3B	88.543
R4GF62	28S ribosomal protein S6, mitochond	MRPS6	41.453
E1C699	Complex I-9kD OS=Gallus gallus OX	NDUFV3	42.814

F1NI43	Long-chain-fatty-acid--CoA ligase AC	ACSBG2	80.987
A0A1D5PTF3	EI24 autophagy associated transmeml	EI24	39.054
A0A1D5PVU7	Integrin_alpha2 domain-containing pr	ITGA7	122.73
A0A3Q2U888	Rhomboid domain-containing protein	RHBDF2	91.308
F1NWD9	G protein-coupled receptor 107(predi	GPR107	62.569
A0A1L1RRG6	Cytochrome P450 2J2(predicted) OS=	CYP2J22	56.372
A0A1D5PWU4	Ankyrin repeat domain 13A(predicted	ANKRD13A	57.66
A0A3S5ZP86	Cytochrome b5 heme-binding domain	FADS1	59.075
A0A3Q2UAD4	Dihydroxyacetone phosphate acyltran	PDCD2	71.605
F1NER9	Glycoprotein IIIb OS=Gallus gallus C	CD36	52.637
F1NIW6	TraB domain containing(predicted) O	TRABD	41.877
F1NM35	Lipase domain-containing protein OS	PLA1A	50.069
F1NZS9	Thymopoietin isoform x1(predicted) (	TMPO	50.171
A0A3Q2U831	NPR3 like, GATOR1 complex subun	NPRL3	63.989
R4GGX6	Mitochondrial import receptor subuni	--	6.5839
F1NRQ2	Protein NDRG3 OS=Gallus gallus OX	LOC107057310	41.071
A0A3Q2UI63	Plug_translocon domain-containing p	SEC61A1	46.072
E1BZ66	UBC core domain-containing protein	UBE2J2	28.917
A0A1D5PRR1	t-SNARE coiled-coil homology doma	VTI1A	25.036
A0A5H1ZRJ9	Reversion-inducing cysteine-rich prot	RECK	98.72
A0A1D5PKV8	Protein SON(predicted) OS=Gallus g	SON	48.895
Q5ZLP5	SAP domain-containing protein OS=(	SARNP	23.462
A0A1D5PA28	Methyltransf_11 domain-containing p	METTL7A	27.918
A0A3Q2UGC6	Solute carrier family 25 member 10(p	SLC25A10	55.425
R4GH27	WH2 domain-containing protein OS=	LMOD1	69.047
A0A1D5NWH9	CSD domain-containing protein OS=(	YBX3	30.873
Q5ZKL6	Lysophospholipid acyltransferase 2 O	mboat2	59.552
Q5ZKN1	Cyclin-dependent kinase 9 OS=Gallu	CDK9	42.802
E1BTX4	UBX domain-containing protein 7 OS	UBXN7	51.783
A0A1D5NYP5	Cytochrome P450 family 7 subfamily	--	56.975
F1NBN1	Plug_translocon domain-containing p	SEC61A1	52.278
Q5ZJU8	Tumor necrosis factor alpha-induced	TNFAIP8L1	21.535
A0A1D5PM46	Rap-GAP domain-containing protein	TSC2	202.07

Q5ZJR3	Oligosaccharyltransferase complex su	OSTC	16.743
F1P2W9	CULLIN_2 domain-containing protei	CUL2	80.4
A0A3Q3AK07	Transmembrane protein with metallo	--	48.674
A0A1D5PMA9	Laminin beta 2-like chain(predicted)	LAMB2	193.96
E1C5T6	Transmembrane protein 35B(predicte	TMEM35B	14.808
A0A1L1RSY0	Tripartite motif containing 35(predict	TRIM44	56.115
E1C3S1	Monocarboxylate transporter 8(predic	SLC16A2	57.598
A0A3Q2U1W2	Glutamine amidotransferase type-1 do	--	10.797
Q5MNV6	Zinc transporter 7 OS=Gallus gallus	SLC30A7	41.989
A0A3Q2U797	TFIIS N-terminal domain-containing	LOC100859302	50.106
A0A1D5NUR5	Syntrophin alpha 1(predicted) OS=Ga	SNTA1	51.678
Q9PU47	PDZ and LIM domain protein 3 OS=	PDLIM3	34.365
A0A1D5P8V6	UBA domain-containing protein OS=	UBAP2L	113.92
A0A3Q2UHC9	Sterile alpha motif domain containing	--	26.199
Q91049	Occludin OS=Gallus gallus OX=9031	OCLN	55.863
A0A1D5PND8	Aminotran_1_2 domain-containing pr	GPT2	60.015
Q5ZIC5	Longin domain-containing protein OS	SEC22A	35.104
A0A1D5PM87	RAD21 cohesin complex component(	RAD21	72.05
F1NNP0	Cytochrome p450 4b1-like(predicted)	CYP4B7	57.556
Q5ZKA3	Pre-mRNA-splicing factor CWC22 h	CWC22	108.67
A0A1D5PYG2	F-box domain-containing protein OS=	FBXL20	47.405
A0A1D5P8S6	Cysteine-rich and transmembrane dor	--	11.886
F1N9C5	Transmembrane protein 209 OS=Gall	TMEM209	62.99
E1C8M8	(S)-3-amino-2-methylpropionate trans	ABAT	56.552
F1NSV6	Sodium/potassium-transporting ATPa	ATP1B1	34.924
A0A3Q3A395	L-type lectin-like domain-containing	LMAN2L	38.709
A0A1D5PT04	Nucleolar protein 6 OS=Gallus gallus	NOL6	128.99
F1NY54	Thyroid hormone receptor associated	THRAP3	109.88
E1BVP2	Phospholipase OS=Gallus gallus OX=	PLD1	118.99
R4GH54	Cytochrome c oxidase assembly prote	COX20	12.915
A0A1L1RPI1	BHLH domain-containing protein OS	SREBF1	110.91
A0A3Q2UPB5	PUM-HD domain-containing protein(	--	63.907
A0A3Q3ARU3	Ras association (RalGDS/AF-6) and p	RAPH1	134.33

A0A1D5NWM7	Ribosomal L1 domain containing 1(p	RSL1D1	49.601
A0A1D5NW88	Microtubule-associated protein OS=C	MAP4	111.7
Q5F3B9	Succinate--CoA ligase [ADP-forming	SUCLA2	49.997
D2Z1L9	LIM and SH3 domain protein 1 OS=(	LASP1	29.655
F1NJG4	Cytochrome P450 CYP2D49 OS=Ga	CYP2D6	57.946
F1NMM5	AB hydrolase-1 domain-containing p	ABHD12B	39.931
A0A3Q3AQG0	Oxysterol-binding protein OS=Gallus	OSBPL8	91.077
Q5ZMK5	Bifunctional arginine demethylase an	JMJD6	47.759
Q5F3U9	Sister chromatid cohesion protein PD	PDS5B	161.02
E1BRU7	Hexokinase OS=Gallus gallus OX=9(	HKDC1	102.19
P05180	Cytochrome P450 2H1 OS=Gallus ga	CYP2H1	56.261
A0A3Q2U968	VWFA domain-containing protein O	ITGAD	110.93
A0A3Q2UF08	PWWP domain-containing protein O	HDGFRP2	71.927
E1C004	Carbonate dehydratase IV OS=Gallus	CA4	36.159
Q802T2	Dolichyl pyrophosphate Man9GlcNA	ALG6	57.437
P0C7A1	Cytosolic endo-beta-N-acetylglucosa	ENGASE	80.888
A0A1D5P0N6	Anoctamin OS=Gallus gallus OX=90	ANO10	74.926
F1NK09	DUF1741 domain-containing protein	C6H10orf76	78.608
A0A1L1RLF0	Cytochrome P450 family 2 subfamily	CYP2W2	56.915
A0A1L1RU55	Adenine phosphoribosyltransferase O	APRT	19.292
A0A3Q2U2T3	Putative proline-rich protein 21 (Frag	--	53.421
A0A1D5PPH4	Protein kinase domain-containing pro	STK25	48.091
R4GLP0	Cytochrome c oxidase polypeptide V	COX7C	15.062
A0A3Q2UML3	Acyl-coenzyme A thioesterase 1-like	LOC423222	46.297
A0A1D5PLN3	Transmembrane protein 14C (Fragme	TMEM14C	11.084
E1BTJ1	Glycylpeptide N-tetradecanoyltransfe	NMT2	56.809
E1C250	ANK_REP_REGION domain-contai	KANK4	107.59
F1NG47	Protein kinase C OS=Gallus gallus O	PRKCE	84.643
Q5ZMF9	Derlin OS=Gallus gallus OX=9031 G	DERL1	28.909
F1NXX9	Bactericidal permeability-increasing	LBP	52.779
A0A1D5NXB4	Pappalysin 2(predicted) OS=Gallus g	PAPPA2	177.74
A0A3Q2U701	BZIP domain-containing protein OS=	BATF3	15.637
A0A3Q2UKN9	Chromosome 18 open reading frame	CZH18ORF32	22.289

A0A1D5PS70	PP28 domain-containing protein OS=	PDAP1	21.129
A0A1D5NW15	Protein kinase domain-containing pro	AAK1	48.982
P62207	Serine/threonine-protein phosphatase	PPP1CB	37.186
E1C3Y3	Tetraspanin OS=Gallus gallus OX=9(	TSPAN8	26.925
A0A3Q2U3I1	ENTH domain-containing protein OS	EPN2	52.482
Q5ZM65	Phosphatidylserine synthase 1 OS=Ga	PTDSS1	55.51
E1BWX4	Lysine ketoglutarate reductase OS=G	AASS	102.95
A0A1D5NXV6	Spermatogenesis-defective protein 39	VIPAS39	56.802
Q5F370	15-oxoprostaglandin 13-reductase OS	PTGR2	37.61
E1C944	acidPPc domain-containing protein O	PLPP3	35.146
F1NRI9	Sulfotransferase OS=Gallus gallus OX	LOC421740	35
E1BQC0	MFS domain-containing protein OS=	SLC22A18	45.829
F1P296	Cystine/glutamate transporter (Fragm	CGTL	52.783
P12276	Fatty acid synthase OS=Gallus gallus	FASN	274.78
O57604	Podocalyxin OS=Gallus gallus OX=9	PODXL	61.014
E1C2H4	LEM domain-containing protein OS=	LEMD2	77.059
A0A3Q2U3G1	Peroxisomal biogenesis factor 11 gam	PEX11G	24.26
P21265	Adenylosuccinate lyase OS=Gallus ga	ADSL	54.652
F1NYL5	Invariant chain(predicted) OS=Gallus	CD74	24.812
R4GJK4	Signal sequence receptor subunit gam	SSR3	21.216
A0A1D5PAQ0	Ig-like domain-containing protein OS	--	10.386
A0A1D5P1R5	Unc-79 homolog, NALCN channel co	UNC79	290.46
A0A1D5NVC3	Calpain catalytic domain-containing p	CAPN7	84.737
A0A1D5PCQ6	Mannose-6-phosphate receptor, cation	M6PR	34.578
E1BVC8	Secretory carrier-associated membran	SCAMP1	48.477
R4GJN4	Heat shock 70 kDa protein 12B (Frag	GBE	68.776
F1NH92	MICOS complex subunit OS=Gallus	APOOL	29.693
F1NBV2	Arf-GAP domain-containing protein (	AGFG1	56.031
A0A1D5PMQ4	EMAP like 6(predicted) OS=Gallus g	EML6	218.12
A0A1L1RXA5	Receptor expression-enhancing protei	REEP5	37.839
E1C896	Selenoprotein K OS=Gallus gallus OX	SELENOK	10.158
A0A1D5PZZ1	Cytochrome P450 2J2(predicted) OS=	CYP2J21	56.489
A0A1D5PI58	SERPIN domain-containing protein C	OVALX	43.627

F1N9R1	Catenin alpha 3(predicted) OS=Gallu	CTNNA3	100.05
R4GJX3	Interferon-inducible transmembrane p	LOC770612	14.947
E1BST8	Phosphatidylinositol glycan anchor bi	PIGU	49.775
F1NMG8	Unspecific monooxygenase OS=Gallu	CYP1A2	60.282
A0A3Q2UFX3	IGv domain-containing protein OS=C	--	10.083
Q5ZLH4	Transmembrane protein 230 OS=Gall	TMEM230	13.174
A0A1D5PAB6	Transmembrane protein 19 OS=Gallu	TMEM19	36.866
E1C4J6	Solute carrier family 51 alpha subunit	SLC51A	37.721
Q8JIY0	Syndecan OS=Gallus gallus OX=903	SDC2	22.084
A0A3Q2UIS4	Kinesin motor domain-containing pro	KIF16B	232.89
A0A3Q2U0F7	Sidoreflexin OS=Gallus gallus OX=9	SFXN3	35.378
A0A1D5Q006	Protein CDV3 homolog OS=Gallus g	CDV3	21.64
P27003	Protein S100-A10 OS=Gallus gallus (	S100A10	11.29
A0A1L1RV18	Microsomal signal peptidase 25 kDa s	--	12.637
F6U467	Very-long-chain (3R)-3-hydroxyacyl-	HACD2	27.943
A0A1D5PY43	G kinase anchoring protein 1(predicte	GKAP1	41.882
E1C5S3	Solute carrier organic anion transporte	SLCO2B1	72.711
A0A1L1RY50	ENTH domain-containing protein OS	LOC772071	65.555
A0A1D5PUE2	Unc-50 inner nuclear membrane RNA	UNC50	30.29
A0A1L1RTK9	RRM domain-containing protein OS=	EIF4B	97.948
F1P203	MARVEL domain-containing protein	CMTM4	23.567
E1C7L9	Solute carrier family 38 member 4(pre	SLC38A4	62.303
E1BYB1	BCL2 like 14(predicted) OS=Gallus g	BCL2L14	37.392
F1NX66	Phospholipid scramblase OS=Gallus	PLSCR1	33.665
P01012	Ovalbumin OS=Gallus gallus OX=90	SERPINB14	42.881
A0A1D5PRD7	Glucocorticoid receptor OS=Gallus g	NR3C1	83.994
A0A1D5PHH8	DCS-1 OS=Gallus gallus OX=9031 (	DCPS	26.996
A0A3Q2UGJ2	STAT_int domain-containing protein	LOC112530959	43.014
F1NTZ0	PKS_ER domain-containing protein (	LOC100857280	39.568
Q5ZML6	Protein FAM210A OS=Gallus gallus	FAM210A	30.449
F1NZR2	ENTH domain-containing protein OS	EPN3	67.84
P80389	Antimicrobial peptide CHP1 OS=Gal	--	4.4804
A0A3Q3B025	LIM zinc-binding domain-containing	LIMA1	85.113

A0A1L1RQ64	UBX domain-containing protein 4 isc	UBXN4	16.099
E1C3D3	ERGIC and golgi 2(predicted) OS=Ga	ERGIC2	42.521
F1NHA6	G_PROTEIN_RECEP_F3_4 domain-	GPRC5C	46.214
A0A1D5PBJ1	Cytochrome b reductase 1 OS=Gallus	CYBRD1	31.979
F1P4N9	Periostin(predicted) OS=Gallus gallus	POSTN	90.643
F1N8J7	LIM zinc-binding domain-containing	CRIP1	8.5998
E3VVZ8	Elongation of very long chain fatty ac	ELOVL5	35.182
Q5F3Z0	Transmembrane protein 106B(predict	TMEM106B	30.949
F1NYH8	Ena/VASP-like protein OS=Gallus ga	EVL	44.758
E1C0E5	Transmembrane protein 9B OS=Gallu	TMEM9B	21.326
A0A3Q2UGE9	BAT2_N domain-containing protein (	PRRC2C	297.78
Q5F389	WW domain-containing oxidoreducta	WWOX	46.729
A0A3Q2TS16	Non-specific serine/threonine protein	MARK1	80.012
E1BTL9	60S ribosomal export protein NMD3	NMD3	57.566
A0A3Q2TTB1	PDZ domain-containing protein OS=(	HTRA1	38.847
P01014	Ovalbumin-related protein Y OS=Gal	SERPINB14B	43.772
A0A1D5PAP2	Ribosomal RNA processing 1B(predi	RRP1B	84.456
E1C1G8	Fibulin-5 OS=Gallus gallus OX=9031	FBLN5	49.66
A0A1D5PHG1	Receptor-binding cancer antigen expr	EBAG9	13.269
A0A1D5PUU2	LisH domain-containing protein OS=	NOLC1	72.417
F1NZV4	CAP-ZIP_m domain-containing prote	FAM21C	146.21
Q5F384	Protein YIPF3 OS=Gallus gallus OX=	YIPF3	36.942
F1P4I3	V-SNARE coiled-coil homology dom	VAMP3	11.687
A0A1D5P8Y3	Non-specific serine/threonine protein	PAK4	71.586
A0A1D5P637	6-phosphofructo-2-kinase OS=Gallus	PFKFB3	59.067
A0A3Q3ARC5	Complex I-9kD OS=Gallus gallus OX	NDUFV3	39.234
Q6URH5	Gap junction protein OS=Gallus gallu	GJB1	31.787
A0A1D5PUF3	Progestin and adipoQ receptor family	PAQR9	45.098
A0A1L1RP39	UTP--glucose-1-phosphate uridylyltra	UGP2	55.633
Q90987	Stathmin-2 OS=Gallus gallus OX=90	STMN2	20.882
A0A3Q2U7R8	Galectin OS=Gallus gallus OX=9031	LGALS2	14.702
E1C4M9	L-type amino acid transporter 4 OS=(	SLC43A2	62.018
Q5ZKR7	Long-chain-fatty-acid--CoA ligase A(	ACSBG2	84.206

A0A3Q2TTC4	DUF1394 domain-containing protein	FAM49B	36.849
R4GKH5	MARVEL domain-containing protein	SYPL1	25.905
A0A1D5PD62	Reticulon OS=Gallus gallus OX=903	RTN4	36.171
A0A1D5PFJ2	Calpain inhibitor OS=Gallus gallus C	CAST	78.061
F1NT18	Cytochrome P450(predicted) OS=Gal	CYP3A5	58.247
A0A1L1RJB6	Anion exchange protein OS=Gallus g	SLC4A1	100.52
A0A1D5PIK5	PL48 domain-containing protein OS=	FAM65A	121.52
E1BTE2	SERPIN domain-containing protein C	SERPINB5	42.645
R4GLP5	Integrator complex subunit 3(predicte	INTS3	113.14
P15979	Class I histocompatibility antigen, F1	--	38.246
Q5ZK43	Trimeric intracellular cation channel t	TMEM38A	33.247
O73895	Tapasin OS=Gallus gallus OX=9031	TAPBP	45.455
P01013	Ovalbumin-related protein X (Fragme	SERPINB14C	26.291
P46158	Gallinacin-2 OS=Gallus gallus OX=9	GAL2	7.1415
A0A1D5PHQ9	Amine oxidase OS=Gallus gallus OX	AOC1	93.908
P33150	Cadherin-13 OS=Gallus gallus OX=9	CDH13	78.38
A0A1D5PI43	Amine oxidase OS=Gallus gallus OX	MAOB	58.574
A0A1D5P9X2	Tropomyosin beta chain OS=Gallus g	TPM2	35.635
F1NV32	Secretory carrier-associated membran	SCAMP2	36.969
A0A3Q2U1B0	Periostin(predicted) OS=Gallus gallus	POSTN	94.204
F1P0V0	CYCLIN domain-containing protein C	CCNYL1	40.58
A0A1D5PAE7	Elongin-A OS=Gallus gallus OX=903	TCEB3	87.916
A0A1D5PS26	NADB-LER4(predicted) OS=Gallus g	--	15.701
F1N8J8	Cysteine rich protein 2(predicted) OS	CRIP2	25.971
P12957	Caldesmon OS=Gallus gallus OX=90	CALD1	88.746
Q01841	Protein-glutamine gamma-glutamyltra	TGM2	77.969
Q5ZHQ6	Acyl-CoA-binding domain-containing	ACBD5	54.454
A0A1D5PR60	Metadherin(predicted) OS=Gallus gal	MTDH	41.374
F1NGQ9	Frizzled-1 OS=Gallus gallus OX=903	FZD1	63.101
E1C1N8	Tudor domain-containing protein OS=	AKAP1	95.474
A0A3Q2TY54	PLD phosphodiesterase domain-conta	--	22.87
A0A3Q2UHE4	SH3 domain-containing protein OS=C	CTTN	58.021
F1NU55	SH3 domain-containing protein OS=C	CTTN	58.043

Q9YHT1	Succinate dehydrogenase [ubiquinone	SDHA	72.93
F1NH24	Leucine-rich repeat-containing protei	LRRC9	127.35
A0A1D5NV58	BAT2_N domain-containing protein (	PRRC2C	293.35
Q5F3I1	Fibrinogen C-terminal domain-contai	FGL2	50.241
F1NMQ3	PEST proteolytic signal-containing nu	PCNP	19.103
A0A3Q3A7M3	CD99 molecule (Xg blood group)(pre	CD99	18.269
P23614	Brain acid soluble protein 1 homolog	BASP1	25.438
E1BTF4	SERPIN domain-containing protein C	OVALY	43.813
A0A1D5PEB6	Claudin OS=Gallus gallus OX=9031	CLDN15	24.377
Q5ZMV0	SH3 domain-containing protein OS=(	HCLS1	57.246
A0A1D5P2D3	SUI1 domain-containing protein OS=	EIF1B	12.823
F1NWJ8	SH3 domain binding glutamate rich p	--	7.6384
A0A1D5PFA1	Zinc finger C2HC-type containing 1A	ZC2HC1A	32.803
A0A1D5PDE6	Myristoylated alanine-rich C-kinase s	MARCKS	27.643
P26932	Calponin-1 OS=Gallus gallus OX=90	CNN1	32.36
P00504	Aspartate aminotransferase, cytoplasr	GOT1	45.935

Table S8. Proteins quantified by hepatic proteome

Con	HS-I	HS-II	HS-I/Con Ratio	HS-I/Con P value
0.3285	0.7390	2.0055	2.2500	0.5230
0.4000	0.5105	2.1410	1.2760	0.1057
0.4680	0.5000	2.0760	1.0680	0.4733
0.5743	0.8545	1.8590	1.4880	0.1300
0.6525	0.7125	1.9203	1.0920	0.8539
0.6760	0.7480	1.8150	1.1070	0.8744
0.6830	0.7860	1.8010	1.1510	0.6027
0.7103	0.7173	1.8077	1.0100	0.9777
0.7103	0.8098	1.7613	1.1400	0.7416
0.7365	0.7563	1.7240	1.0270	0.7651
0.7155	0.8738	1.6100	1.2210	0.3340
0.7295	1.0015	1.6120	1.3730	0.1642
0.7608	0.7745	1.6383	1.0180	0.7643
0.6620	1.0360	1.3655	1.5650	0.1062
0.7150	0.8595	1.4610	1.2020	0.5034
0.7020	0.9375	1.4230	1.3350	0.0651
0.7600	0.9375	1.4867	1.2340	0.1309
0.6540	1.1893	1.2760	1.8180	0.0026
0.6290	1.2895	1.2180	2.0500	0.0507
0.7845	0.7425	1.5070	0.9460	0.2231
0.6745	1.2210	1.2770	1.8100	0.0412
0.5860	1.3650	1.1045	2.3290	0.0267
0.9155	0.7495	1.7150	0.8190	0.5390
0.7860	0.7420	1.4425	0.9440	
0.5670	1.4250	1.0240	2.5130	0.4444
0.8318	0.8360	1.4673	1.0050	0.9268
0.7773	1.0038	1.3530	1.2910	0.5300
0.8738	0.7798	1.5043	0.8920	0.8752

0.8298	0.8980	1.4270	1.0820	0.3500
0.6420	1.3280	1.1000	2.0690	0.0190
0.6360	1.3245	1.0885	2.0830	0.0558
0.7933	0.9790	1.3510	1.2340	0.0635
0.7748	1.0145	1.3180	1.3090	0.0075
0.7855	0.8823	1.3350	1.1230	0.9004
0.6503	1.3320	1.1023	2.0480	0.0126
0.8830	0.9145	1.4810	1.0360	0.7395
0.8233	0.9215	1.3680	1.1190	0.3697
0.7585	1.0405	1.2550	1.3720	0.1098
0.7910	0.9500	1.3065	1.2010	0.0898
0.7925	1.0215	1.3053	1.2890	0.0265
0.7830	1.1000	1.2850	1.4050	0.0152
0.8430	0.9258	1.3787	1.0980	0.7937
0.8038	1.0165	1.3123	1.2650	0.2085
0.8580	0.9695	1.3950	1.1300	0.0356
0.8070	1.0190	1.3020	1.2630	0.0388
0.7975	1.0350	1.2827	1.2980	0.0060
0.7490	1.1583	1.1973	1.5460	0.3623
0.8340	1.0290	1.3290	1.2340	0.0833
0.8265	0.9653	1.3170	1.1680	0.2820
0.8030	0.9750	1.2715	1.2140	0.4014
0.8768	0.9015	1.3863	1.0280	0.6676
0.8165	0.9370	1.2865	1.1480	0.5765
0.8230	1.0540	1.2970	1.2810	0.0553
0.6883	1.2965	1.0807	1.8840	0.0039
0.8798	0.8748	1.3713	0.9940	0.9067
0.8798	0.8700	1.3683	0.9890	0.8886
0.8220	1.0023	1.2757	1.2190	0.0403
0.8813	0.8610	1.3673	0.9770	0.6856
0.8570	0.9308	1.3280	1.0860	0.1680
0.7980	1.0190	1.2280	1.2770	0.0081
0.8758	0.8465	1.3473	0.9670	0.7044

0.7770	1.1168	1.1940	1.4370	0.0232
0.8475	0.9525	1.3020	1.1240	0.0439
0.8545	1.0185	1.3100	1.1920	0.2428
0.8235	1.0660	1.2590	1.2940	0.2684
0.8238	1.0340	1.2593	1.2550	0.0423
0.8750	0.8975	1.3377	1.0260	0.8510
0.7073	1.2438	1.0780	1.7590	0.0003
0.8400	0.9618	1.2797	1.1450	0.0040
0.8413	0.9835	1.2803	1.1690	0.0374
0.8605	0.9440	1.3067	1.0970	0.2640
0.8118	1.0295	1.2317	1.2680	0.2810
0.8193	1.0353	1.2410	1.2640	0.0295
0.8455	0.9598	1.2813	1.1350	0.0560
0.8425	0.9210	1.2735	1.0930	0.5654
0.8735	0.9855	1.3210	1.1280	0.4866
0.8910	0.8813	1.3470	0.9890	0.8333
0.8140	1.0405	1.2293	1.2780	0.0057
0.8500	0.9005	1.2830	1.0590	0.6988
0.7248	1.2388	1.0930	1.7090	0.0000
0.7050	1.2755	1.0620	1.8090	0.0900
0.8773	0.9055	1.3187	1.0320	0.9509
0.7895	1.0715	1.1860	1.3570	0.0081
0.9040	0.9390	1.3570	1.0390	0.5186
0.8420	1.0020	1.2603	1.1900	0.5705
0.8735	0.9355	1.3080	1.0710	0.2792
0.8448	1.0110	1.2613	1.1970	0.0099
0.8708	0.9283	1.3003	1.0660	0.1478
0.8273	1.0258	1.2340	1.2400	0.0104
0.8655	1.0105	1.2910	1.1680	0.4507
0.9473	0.8503	1.4110	0.8980	0.1930
0.8143	1.0710	1.2103	1.3150	0.0020
0.8585	1.0305	1.2710	1.2000	0.0713
0.8533	0.9795	1.2617	1.1480	0.0524

0.7520	1.1945	1.1090	1.5880	0.3231
0.8535	0.9978	1.2587	1.1690	0.0142
0.9210	0.8313	1.3573	0.9030	0.2795
0.9003	0.8780	1.3257	0.9750	0.8094
0.8690	0.9825	1.2747	1.1310	0.3715
0.8865	0.9885	1.2980	1.1150	0.1377
0.9410	0.8045	1.3743	0.8550	0.2874
0.8630	0.9958	1.2540	1.1540	0.0164
0.7280	1.2650	1.0560	1.7380	0.0530
0.9140	0.9440	1.3230	1.0330	0.7161
0.8413	1.0278	1.2157	1.2220	0.0039
0.7935	1.1585	1.1460	1.4600	0.0121
0.8675	1.0315	1.2530	1.1890	0.3624
0.8960	0.9253	1.2933	1.0330	0.6040
0.7990	1.0950	1.1505	1.3700	0.0903
0.7305	1.2750	1.0510	1.7450	0.5033
0.8850	0.9335	1.2717	1.0550	0.5350
0.8300	1.0260	1.1910	1.2360	0.0736
0.8730	0.9740	1.2510	1.1160	0.2154
0.9030	0.9065	1.2943	1.0040	0.8964
0.8815	0.9575	1.2617	1.0860	0.0438
0.9330	0.9120	1.3340	0.9770	0.8777
0.8755	0.9743	1.2477	1.1130	0.0212
0.9300	0.9625	1.3250	1.0350	0.9404
0.9663	0.7915	1.3773	0.8190	0.3554
0.8438	1.0190	1.2013	1.2080	0.0270
0.8765	0.9815	1.2470	1.1200	0.1194
0.8315	1.0480	1.1817	1.2600	0.1965
0.8460	1.0348	1.2020	1.2230	0.0211
0.8988	0.9288	1.2707	1.0330	0.5666
0.8308	1.0735	1.1727	1.2920	0.0579
0.8755	0.9783	1.2360	1.1170	0.1095
0.8808	0.9768	1.2440	1.1090	0.4921

0.8970	0.9965	1.2660	1.1110	0.6042
0.9083	0.9050	1.2817	0.9960	0.8882
0.8548	1.0153	1.2040	1.1880	0.0483
0.8805	0.9588	1.2407	1.0890	0.1378
0.8743	0.9803	1.2313	1.1210	0.4643
0.8945	0.9915	1.2550	1.1080	0.6526
0.8460	1.0135	1.1865	1.1980	0.3524
0.8935	0.9515	1.2530	1.0650	0.6441
0.7923	1.1605	1.1097	1.4650	0.1285
0.8755	0.9708	1.2263	1.1090	0.1426
0.8785	0.9800	1.2310	1.1160	0.1351
0.8815	0.9748	1.2347	1.1060	0.0203
0.8285	1.0545	1.1595	1.2730	0.0329
0.8520	0.9935	1.1925	1.1660	0.2073
0.8830	0.9508	1.2360	1.0770	0.1837
0.9043	0.9265	1.2660	1.0250	0.5918
0.8880	0.9768	1.2420	1.1000	0.1087
0.8103	1.1420	1.1327	1.4090	0.0828
0.8693	0.9988	1.2147	1.1490	0.1462
0.9230	0.8865	1.2897	0.9600	0.3931
0.8970	0.9555	1.2510	1.0650	0.4406
0.8960	0.9555	1.2493	1.0660	0.1892
0.7913	1.1658	1.1020	1.4730	0.0323
0.9360	0.9350	1.3020	0.9990	0.9860
0.8968	0.9523	1.2460	1.0620	0.5288
0.8780	1.0345	1.2190	1.1780	0.0275
0.9323	0.8673	1.2917	0.9300	0.5972
0.8575	1.0205	1.1877	1.1900	0.0143
0.8803	1.0015	1.2193	1.1380	0.1493
0.8855	0.9220	1.2265	1.0410	0.5352
0.8670	0.9850	1.2000	1.1360	0.0608
0.8960	1.0075	1.2400	1.1240	0.4127
0.9303	0.8753	1.2863	0.9410	0.4924

0.8883	0.9425	1.2263	1.0610	0.2603
0.8550	1.0050	1.1795	1.1750	0.2404
0.8675	1.0175	1.1970	1.1730	0.0737
0.8935	0.9595	1.2330	1.0740	0.3471
0.8690	0.9710	1.1980	1.1170	0.4589
0.8768	0.9845	1.2087	1.1230	0.0123
0.8923	0.9608	1.2293	1.0770	0.4965
0.8730	0.9813	1.2023	1.1240	0.2181
0.9010	0.9518	1.2400	1.0560	0.4176
0.8790	0.9955	1.2087	1.1330	0.0565
0.8903	0.9325	1.2243	1.0470	0.3670
0.9050	0.9940	1.2440	1.0980	0.3394
0.9670	0.8885	1.3300	0.9190	0.7098
0.8595	0.9980	1.1810	1.1610	0.1830
0.8790	0.9973	1.2073	1.1350	0.0757
0.8943	0.9590	1.2287	1.0720	0.5736
0.8625	0.9910	1.1845	1.1490	0.0067
0.8760	1.0038	1.2027	1.1460	0.1948
0.8865	0.9678	1.2153	1.0920	0.2400
0.9038	0.9633	1.2390	1.0660	0.1288
0.8955	0.9428	1.2270	1.0530	0.6091
0.8908	0.9520	1.2193	1.0690	0.4626
0.8768	0.9773	1.1987	1.1150	0.5602
0.8663	1.0043	1.1837	1.1590	0.0193
0.8753	1.0053	1.1947	1.1490	0.3000
0.9075	0.9700	1.2390	1.0690	0.0323
0.8840	0.9583	1.2057	1.0840	0.4021
0.8853	1.0045	1.2073	1.1350	0.0007
0.9705	0.8845	1.3240	0.9110	0.3407
0.8638	1.0363	1.1763	1.2000	0.0296
0.8365	1.0943	1.1387	1.3080	0.0073
0.9128	0.9285	1.2420	1.0170	0.8340
0.8055	1.1365	1.0940	1.4110	0.0943

0.8785	1.0410	1.1933	1.1850	0.1608
0.7865	1.2060	1.0670	1.5330	0.1974
0.8688	1.0235	1.1790	1.1780	0.0968
0.9220	0.9293	1.2503	1.0080	0.9461
0.8975	0.9725	1.2163	1.0840	0.5468
0.8795	1.0120	1.1910	1.1510	0.0848
0.8808	1.0150	1.1927	1.1520	0.0081
0.8605	1.0463	1.1643	1.2160	0.0174
0.8853	1.0058	1.1973	1.1360	0.2482
0.8713	1.0273	1.1777	1.1790	0.0688
0.8793	0.9963	1.1887	1.1330	0.0983
0.8878	0.9905	1.2003	1.1160	0.1694
0.8635	1.0405	1.1663	1.2050	0.0027
0.9068	0.9585	1.2237	1.0570	0.9479
0.8445	1.0595	1.1395	1.2550	0.2916
0.8885	0.9485	1.1985	1.0680	0.6242
0.8960	0.9740	1.2083	1.0870	0.0911
0.9365	0.9615	1.2630	1.0270	0.7555
0.8005	1.1835	1.0780	1.4780	0.0057
0.8150	1.1588	1.0980	1.4220	0.0001
0.8778	1.0070	1.1823	1.1470	0.1493
0.8920	1.0020	1.2013	1.1230	0.0537
0.8735	1.0390	1.1753	1.1890	0.2518
0.8593	1.0663	1.1560	1.2410	0.0157
0.8773	1.0118	1.1803	1.1530	0.0221
0.8835	0.9635	1.1887	1.0910	0.0478
0.7883	1.1923	1.0597	1.5130	0.0001
0.8770	1.0133	1.1783	1.1550	0.0372
0.8188	1.1343	1.0993	1.3850	0.0272
0.9325	0.9540	1.2523	1.0230	0.8416
0.8783	1.0133	1.1790	1.1540	0.0008
0.8398	1.0875	1.1250	1.2950	0.0033
0.8858	1.0113	1.1873	1.1420	0.1504

0.9495	0.7980	1.2725	0.8400	0.6044
0.8935	0.9885	1.1963	1.1060	0.0247
0.9523	0.8648	1.2750	0.9080	0.4745
0.8830	1.0123	1.1813	1.1460	0.0901
0.8830	1.0103	1.1817	1.1440	0.2657
0.8758	1.0248	1.1670	1.1700	0.0755
0.9765	0.7378	1.3013	0.7560	0.1059
0.9130	0.9520	1.2140	1.0430	0.4411
0.7950	1.1813	1.0563	1.4860	0.0005
0.8580	1.0905	1.1400	1.2710	0.4262
0.8855	0.9700	1.1765	1.0950	0.7160
0.8868	1.0238	1.1787	1.1540	0.0040
0.9020	0.9763	1.1983	1.0820	0.0910
0.9253	0.9373	1.2300	1.0130	0.9122
0.8753	0.9938	1.1623	1.1350	0.0175
0.8595	1.0633	1.1407	1.2370	0.0765
0.9165	0.9568	1.2160	1.0440	0.2670
0.8310	1.1195	1.1020	1.3470	0.2710
0.8595	1.0405	1.1400	1.2110	0.2004
0.9180	0.9450	1.2170	1.0290	0.4672
0.9405	0.9545	1.2470	1.0150	0.8167
0.8780	0.9960	1.1630	1.1340	0.6184
0.9033	0.9468	1.1967	1.0480	0.5236
0.8820	0.9925	1.1675	1.1250	0.5166
0.9013	0.9835	1.1933	1.0910	0.0478
0.8593	1.0678	1.1370	1.2430	0.0028
0.9105	1.0070	1.2050	1.1060	0.2015
0.9510	0.9400	1.2580	0.9880	0.8929
0.9175	0.9493	1.2127	1.0350	0.6032
0.8668	1.0370	1.1447	1.1960	0.0105
0.8690	1.0275	1.1477	1.1820	0.1064
0.8935	1.0135	1.1803	1.1340	0.1520
0.9015	0.9585	1.1910	1.0630	0.4029

0.8370	1.0990	1.1045	1.3130	0.0038
0.8780	1.0385	1.1590	1.1830	0.0030
0.8903	1.0028	1.1753	1.1260	0.2623
0.9205	0.9900	1.2140	1.0760	0.4311
0.9538	0.8655	1.2573	0.9070	0.0467
0.9118	0.9663	1.2010	1.0600	0.4392
0.8875	0.9805	1.1680	1.1050	0.1530
0.8915	0.9690	1.1730	1.0870	0.3234
0.9060	1.0160	1.1920	1.1210	0.4171
0.9400	0.9405	1.2360	1.0010	0.9964
0.9673	0.8570	1.2723	0.8860	0.2518
0.6715	1.4378	0.8820	2.1410	0.2901
0.8943	0.9983	1.1743	1.1160	0.0822
0.8985	0.9915	1.1793	1.1040	0.0548
0.9050	1.0190	1.1880	1.1260	0.0649
0.9140	0.9640	1.2000	1.0550	0.3876
0.8753	1.0338	1.1480	1.1810	0.0165
0.8868	0.9933	1.1633	1.1200	0.1387
0.9115	0.9730	1.1960	1.0670	0.4425
0.9140	1.0060	1.1990	1.1010	0.2665
0.9513	0.9293	1.2477	0.9770	0.6980
0.8765	1.0135	1.1495	1.1560	0.7616
0.9063	0.9783	1.1883	1.0790	0.1235
0.8475	1.0873	1.1100	1.2830	0.0075
0.8573	1.1098	1.1230	1.2950	0.0731
0.8833	1.0320	1.1567	1.1680	0.0978
0.9030	0.9940	1.1827	1.1010	0.1307
0.9148	0.9618	1.1987	1.0510	0.3711
0.8340	1.1375	1.0920	1.3640	0.0665
0.8635	1.0460	1.1300	1.2110	0.0719
0.8990	0.9880	1.1770	1.0990	0.1667
0.8998	0.9840	1.1780	1.0940	0.0678
0.9073	0.9723	1.1873	1.0720	0.2712

0.8818	1.0220	1.1527	1.1590	0.1478
0.8993	1.0003	1.1747	1.1120	0.3142
0.9113	0.9588	1.1900	1.0520	0.3435
0.9338	0.9318	1.2193	0.9980	0.9454
1.0300	0.7270	1.3447	0.7060	0.0707
0.8915	0.9760	1.1635	1.0950	0.2811
0.9018	0.9913	1.1763	1.0990	0.0566
0.8885	0.9880	1.1575	1.1120	0.0095
0.9163	0.9715	1.1927	1.0600	0.0895
0.9503	0.8935	1.2373	0.9400	0.7295
0.8950	0.9965	1.1645	1.1130	0.2586
0.9098	0.9830	1.1837	1.0810	0.0877
0.9253	0.9608	1.2037	1.0380	0.5648
0.9623	0.8705	1.2520	0.9050	0.4218
0.8440	1.0388	1.0973	1.2310	0.1038
0.9148	0.9805	1.1893	1.0720	0.3809
0.9298	0.9260	1.2087	0.9960	0.9108
0.8945	1.0420	1.1620	1.1650	0.3499
0.9083	0.9533	1.1800	1.0500	0.2292
0.9250	0.9538	1.2017	1.0310	0.7788
0.8430	1.1080	1.0945	1.3140	0.0319
0.8675	1.0543	1.1260	1.2150	0.0278
0.9173	0.9708	1.1893	1.0580	0.6269
0.9325	0.9473	1.2093	1.0160	0.7097
0.9510	0.9455	1.2330	0.9940	0.9469
0.8963	1.0188	1.1617	1.1370	0.0221
0.9133	0.9690	1.1837	1.0610	0.2108
0.8808	1.0525	1.1403	1.1950	0.0023
0.8825	1.0075	1.1430	1.1420	0.2737
0.8963	1.0133	1.1610	1.1310	0.1272
0.9010	0.9690	1.1660	1.0750	0.3728
0.9315	0.9203	1.2050	0.9880	0.8739
0.8928	1.0015	1.1547	1.1220	0.0208

0.8970	0.9910	1.1600	1.1050	0.0853
0.9100	0.9665	1.1770	1.0620	0.5449
0.9333	0.9480	1.2067	1.0160	0.4343
0.9083	0.9780	1.1733	1.0770	0.0084
0.8868	1.0210	1.1450	1.1510	0.0461
0.8888	1.0473	1.1477	1.1780	0.0004
0.9093	0.9935	1.1737	1.0930	0.1590
0.9238	0.9580	1.1923	1.0370	0.3850
0.9773	0.8458	1.2620	0.8650	0.3300
0.8773	1.0405	1.1313	1.1860	0.0349
0.9118	0.9748	1.1760	1.0690	0.1574
0.9145	0.9750	1.1800	1.0660	0.0226
0.9185	0.9465	1.1850	1.0300	0.9540
0.9218	0.9805	1.1890	1.0640	0.1087
0.9270	0.9055	1.1955	0.9770	0.7449
0.9340	0.9190	1.2040	0.9840	0.9532
0.9458	0.9153	1.2190	0.9680	0.5106
0.9505	0.9570	1.2250	1.0070	0.8289
0.9700	0.9480	1.2500	0.9770	0.8739
0.8855	1.0365	1.1407	1.1710	0.0123
0.9320	0.9388	1.2000	1.0070	0.8424
0.8970	1.0425	1.1540	1.1620	0.1955
0.8975	0.9900	1.1553	1.1030	0.2245
0.9415	0.9440	1.2113	1.0030	0.9493
0.9070	1.0033	1.1667	1.1060	0.0111
0.9140	0.9755	1.1750	1.0670	0.3148
0.9143	0.9798	1.1760	1.0720	0.1456
0.9180	0.9645	1.1803	1.0510	0.0215
0.9975	0.7395	1.2830	0.7410	0.3646
0.8405	1.1178	1.0797	1.3300	0.0698
0.8845	1.0430	1.1367	1.1790	0.0515
0.8603	1.0723	1.1047	1.2460	0.0003
0.8938	1.0253	1.1473	1.1470	0.0332

0.9038	1.0033	1.1603	1.1100	0.2405
0.9115	1.0240	1.1700	1.1230	0.1925
0.8988	1.0153	1.1527	1.1300	0.0052
0.9250	0.9448	1.1870	1.0210	0.7685
0.9275	0.8985	1.1900	0.9690	0.7804
0.9025	1.0130	1.1567	1.1220	0.3317
0.9035	1.0050	1.1587	1.1120	0.0749
0.9183	0.9380	1.1773	1.0220	0.4737
0.9490	0.9145	1.2163	0.9640	0.8599
0.8708	1.0590	1.1153	1.2160	0.0008
0.9178	0.9763	1.1743	1.0640	0.3669
0.9415	0.9340	1.2047	0.9920	0.8908
0.8720	1.0640	1.1153	1.2200	0.0871
0.9260	0.9600	1.1840	1.0370	0.3855
0.9455	0.9108	1.2090	0.9630	0.5316
1.0703	0.6343	1.3690	0.5930	0.0767
0.8730	1.0895	1.1160	1.2480	0.0204
0.9088	1.0035	1.1613	1.1040	0.3766
0.9123	0.9903	1.1657	1.0860	0.1925
0.9138	0.9705	1.1673	1.0620	0.0916
0.9515	0.9120	1.2163	0.9580	0.3029
0.9123	0.9908	1.1650	1.0860	0.0714
0.9200	0.9365	1.1745	1.0180	0.5727
0.9040	0.9870	1.1537	1.0920	0.1811
0.9443	0.9340	1.2050	0.9890	0.7770
0.9708	0.8965	1.2377	0.9240	0.6817
0.8978	1.0205	1.1440	1.1370	0.0681
0.9210	0.9875	1.1733	1.0720	0.3438
0.8650	1.0765	1.1010	1.2450	0.0523
0.9108	0.9993	1.1597	1.0970	0.2387
0.9203	0.9808	1.1717	1.0660	0.2723
0.9243	1.0030	1.1763	1.0850	0.1640
0.9263	0.9660	1.1790	1.0430	0.4067

0.9320	0.9460	1.1860	1.0150	0.9973
0.9140	0.9680	1.1625	1.0590	0.6577
0.9238	0.9635	1.1750	1.0430	0.4095
0.9460	0.9380	1.2030	0.9920	0.9087
0.9070	1.0083	1.1527	1.1120	0.5785
0.9133	0.9840	1.1607	1.0770	0.2271
0.8620	1.0795	1.0950	1.2520	0.0777
0.9343	0.9495	1.1863	1.0160	0.5562
0.9455	0.9090	1.2007	0.9610	0.6609
0.8495	1.1165	1.0783	1.3140	0.0650
0.9010	0.9830	1.1430	1.0910	0.3085
0.9125	0.9943	1.1580	1.0900	0.1005
0.8870	1.0230	1.1250	1.1530	0.1003
0.9305	0.9430	1.1795	1.0130	0.9354
0.8945	1.0408	1.1330	1.1630	0.0434
0.9050	1.0293	1.1463	1.1370	0.0045
0.9103	1.0018	1.1530	1.1010	0.3118
0.9283	0.9673	1.1763	1.0420	0.7052
0.9288	0.9648	1.1763	1.0390	0.4672
0.7558	1.2780	0.9570	1.6910	0.0026
0.9023	1.0063	1.1423	1.1150	0.0457
0.9145	0.9970	1.1573	1.0900	0.2180
0.9215	1.0100	1.1670	1.0960	0.5947
0.9225	0.9335	1.1680	1.0120	0.8177
0.9420	0.9108	1.1930	0.9670	0.6544
0.9465	0.9365	1.1987	0.9890	0.8307
0.9585	0.9095	1.2130	0.9490	0.5109
0.8928	1.0398	1.1293	1.1650	0.0331
0.9320	0.9570	1.1793	1.0270	0.6709
0.9735	0.9290	1.2310	0.9540	0.1130
0.8480	1.1550	1.0720	1.3620	0.0273
0.8813	1.0565	1.1143	1.1990	0.1932
0.9408	0.9453	1.1890	1.0050	0.9647

0.9040	0.9910	1.1420	1.0960	0.6891
0.9238	0.9750	1.1663	1.0550	0.1312
0.9275	0.9280	1.1715	1.0010	0.9339
0.9400	0.9500	1.1873	1.0110	0.6351
0.9420	0.8960	1.1900	0.9510	0.9518
0.8908	1.0450	1.1243	1.1730	0.0877
0.9020	1.0108	1.1383	1.1210	0.0140
0.9350	0.9525	1.1800	1.0190	0.8264
0.9853	0.8588	1.2430	0.8720	0.4474
0.9015	0.9955	1.1370	1.1040	0.4714
0.9295	0.9728	1.1720	1.0470	0.4643
0.9338	0.9593	1.1773	1.0270	0.6234
0.9375	0.9380	1.1813	1.0010	0.9820
0.8998	1.0350	1.1330	1.1500	0.0688
0.8888	1.0525	1.1177	1.1840	0.0576
0.9143	0.9938	1.1503	1.0870	0.1401
0.8818	1.0660	1.1080	1.2090	0.0448
0.9123	0.9948	1.1470	1.0900	0.2450
0.9403	0.9445	1.1817	1.0050	0.8738
0.9400	0.9570	1.1820	1.0180	0.7685
0.8055	1.2250	1.0120	1.5210	0.2571
0.9183	0.9910	1.1530	1.0790	0.0538
0.9338	0.9833	1.1727	1.0530	0.1381
0.9395	0.9288	1.1797	0.9890	0.8521
0.9490	0.9278	1.1917	0.9780	0.6320
0.8800	1.0435	1.1040	1.1860	0.2791
0.9265	0.9745	1.1623	1.0520	0.5233
0.9320	0.9715	1.1697	1.0420	0.3296
0.9300	0.9558	1.1667	1.0280	0.5316
0.9318	0.9688	1.1683	1.0400	0.4999
0.9615	0.9530	1.2060	0.9910	0.8751
0.8723	1.0875	1.0930	1.2470	0.0488
0.8233	1.1913	1.0307	1.4470	0.0002

0.9468	0.9410	1.1853	0.9940	0.8835
0.9835	0.8615	1.2310	0.8760	0.0746
0.9928	0.8368	1.2433	0.8430	0.2888
0.9153	1.0038	1.1447	1.0970	0.1050
0.8970	1.0388	1.1217	1.1580	0.1691
0.9200	1.0230	1.1500	1.1120	0.4865
0.9228	0.9838	1.1537	1.0660	0.2562
0.9565	0.8755	1.1960	0.9150	0.5235
0.9245	0.9853	1.1543	1.0660	0.0611
0.8480	1.1343	1.0587	1.3380	0.0246
0.9100	0.9900	1.1353	1.0880	0.1213
0.9178	0.9930	1.1453	1.0820	0.0938
0.9405	0.9640	1.1740	1.0250	0.4678
0.9420	0.9785	1.1753	1.0390	0.1409
0.9445	0.9473	1.1787	1.0030	0.9846
0.9233	0.9913	1.1517	1.0740	0.1864
0.9288	0.9655	1.1583	1.0400	0.4159
0.9455	0.9433	1.1787	0.9980	0.9593
0.9790	0.8705	1.2210	0.8890	0.2109
0.9395	0.9553	1.1707	1.0170	0.9174
0.9425	0.9618	1.1740	1.0200	0.6740
0.8055	1.2220	1.0030	1.5170	0.0006
0.9133	1.0030	1.1373	1.0980	0.2827
0.9213	0.9918	1.1467	1.0770	0.1159
0.9238	0.9720	1.1497	1.0520	0.3460
0.9278	0.9728	1.1550	1.0490	0.8129
0.9495	0.9353	1.1820	0.9850	0.7319
0.9548	0.9233	1.1887	0.9670	0.5663
0.8418	1.1490	1.0470	1.3650	0.0001
0.8573	1.1313	1.0660	1.3200	0.0606
0.9173	1.0120	1.1413	1.1030	0.1295
0.9175	1.0035	1.1413	1.0940	0.3047
0.9225	0.9955	1.1473	1.0790	0.1125

0.9455	0.9870	1.1760	1.0440	0.8817
0.9193	0.9990	1.1423	1.0870	0.0603
0.9568	0.9240	1.1893	0.9660	0.7171
0.9210	1.0275	1.1440	1.1160	0.2855
0.9250	0.9910	1.1487	1.0710	0.6448
0.9363	0.9785	1.1630	1.0450	0.3217
0.9153	1.0045	1.1360	1.0980	0.0605
0.9190	1.0000	1.1407	1.0880	0.1880
0.9518	0.9208	1.1813	0.9670	0.4497
0.9588	0.9238	1.1897	0.9630	0.4437
0.9288	0.9870	1.1520	1.0630	0.1078
0.9298	0.9828	1.1530	1.0570	0.2404
0.9555	0.9353	1.1847	0.9790	0.6982
0.9608	0.9273	1.1913	0.9650	0.3109
0.9055	1.0165	1.1223	1.1230	0.0086
0.9205	1.0008	1.1403	1.0870	0.0509
0.9358	0.9685	1.1593	1.0350	0.0865
0.9555	0.9700	1.1840	1.0150	0.1079
0.9593	0.9268	1.1883	0.9660	0.6497
0.9318	0.9533	1.1533	1.0230	0.2969
0.9505	0.9775	1.1770	1.0280	0.6520
0.8743	1.0785	1.0813	1.2340	0.0020
0.9078	1.0068	1.1227	1.1090	0.0655
0.9348	1.0110	1.1567	1.0820	0.2059
0.9370	1.0028	1.1593	1.0700	0.2389
0.8780	1.0750	1.0850	1.2240	0.3341
0.8940	1.0568	1.1047	1.1820	0.0012
0.9193	0.9813	1.1360	1.0670	0.2367
0.9518	0.9390	1.1763	0.9870	0.8108
0.8940	1.0570	1.1040	1.1820	0.0023
0.9410	0.9618	1.1617	1.0220	0.6227
0.9418	0.9660	1.1627	1.0260	0.6007
0.9813	0.8883	1.2117	0.9050	0.0244

0.8710	1.1130	1.0750	1.2780	0.0573
0.9375	0.9730	1.1570	1.0380	0.5270
0.9070	1.0065	1.1180	1.1100	0.3509
0.9325	0.9858	1.1493	1.0570	0.4754
0.9543	0.9270	1.1770	0.9710	0.7880
0.9035	1.0170	1.1135	1.1260	0.2415
0.9218	1.0078	1.1357	1.0930	0.0178
0.9278	0.9905	1.1427	1.0680	0.4536
0.9333	0.9738	1.1500	1.0430	0.1798
0.9448	0.9575	1.1643	1.0130	0.7966
0.9020	1.0230	1.1105	1.1340	0.3232
0.9380	0.9925	1.1550	1.0580	0.3687
0.9410	0.9588	1.1587	1.0190	0.7563
0.9260	1.0085	1.1393	1.0890	0.0882
0.9400	0.9570	1.1567	1.0180	0.6612
0.9430	0.9610	1.1603	1.0190	0.6489
0.9530	0.9910	1.1720	1.0400	0.2506
0.9528	0.9433	1.1723	0.9900	0.8553
0.9845	0.8800	1.2107	0.8940	0.3935
0.9088	1.0355	1.1167	1.1390	0.0030
0.9205	0.9760	1.1310	1.0600	0.9246
0.9265	0.9945	1.1390	1.0730	0.0310
0.9318	0.9918	1.1453	1.0640	0.4521
0.9445	0.9245	1.1605	0.9790	0.9259
0.9448	0.9585	1.1613	1.0150	0.7008
0.9125	1.0225	1.1203	1.1210	0.0272
0.9303	0.9908	1.1423	1.0650	0.4434
0.9458	0.9573	1.1617	1.0120	0.7784
0.8630	1.1228	1.0590	1.3010	0.0342
0.9160	0.9948	1.1243	1.0860	0.2922
0.9200	0.9860	1.1290	1.0720	0.5288
0.9253	0.9958	1.1353	1.0760	0.0112
0.9715	0.9165	1.1917	0.9430	0.4166

0.9920	0.8055	1.2170	0.8120	0.3244
0.9180	0.9910	1.1247	1.0800	0.1265
0.9203	1.0040	1.1273	1.0910	0.0507
0.9240	1.0285	1.1320	1.1130	0.1408
0.9335	0.9798	1.1433	1.0500	0.2759
0.9380	0.9740	1.1487	1.0380	0.5777
0.9493	0.9493	1.1617	1.0000	0.9785
0.9528	0.9583	1.1660	1.0060	0.8829
0.8950	1.0680	1.0943	1.1930	0.0848
0.8998	1.0253	1.1007	1.1390	0.0445
0.9005	1.0250	1.1013	1.1380	0.1203
0.9165	1.0090	1.1207	1.1010	0.1179
0.9313	0.9900	1.1393	1.0630	0.2997
0.9345	0.9615	1.1425	1.0290	0.9710
0.9450	0.9600	1.1557	1.0160	0.7435
0.9543	0.9375	1.1667	0.9820	0.7239
0.9755	0.9348	1.1933	0.9580	0.5344
0.9035	1.0363	1.1037	1.1470	0.0417
0.9130	1.0055	1.1155	1.1010	0.1529
0.9355	0.9775	1.1430	1.0450	0.2494
0.9508	0.9493	1.1620	0.9980	0.9506
0.9210	1.0113	1.1250	1.0980	0.2518
0.9248	1.0003	1.1287	1.0820	0.1645
0.9378	0.9803	1.1450	1.0450	0.4636
0.9550	0.9765	1.1660	1.0230	0.7662
0.8513	1.1543	1.0383	1.3560	0.0797
0.9613	0.9215	1.1723	0.9590	0.6103
0.8913	1.0708	1.0863	1.2010	0.0059
0.9190	0.9995	1.1207	1.0880	0.0442
0.8785	1.1055	1.0700	1.2580	0.1533
0.8928	1.0708	1.0873	1.1990	0.0051
0.9038	1.0420	1.1010	1.1530	0.0092
0.9298	0.9960	1.1327	1.0710	0.4135

0.9540	0.9598	1.1620	1.0060	0.8393
0.9585	0.9760	1.1670	1.0180	0.8251
0.9603	0.9535	1.1697	0.9930	0.9433
0.9683	0.9198	1.1793	0.9500	0.6531
0.9803	0.9108	1.1940	0.9290	0.7085
1.0945	0.5775	1.3330	0.5280	0.0614
1.1315	0.7065	1.3780	0.6240	0.4445
0.9158	1.0110	1.1147	1.1040	0.0246
0.9435	0.9660	1.1487	1.0240	0.4952
0.9548	0.9553	1.1623	1.0010	0.9931
0.9578	0.9403	1.1653	0.9820	0.7296
0.8653	1.1338	1.0520	1.3100	0.1439
0.8965	1.0765	1.0900	1.2010	0.3714
0.9088	1.0348	1.1050	1.1390	0.1977
0.9125	1.0105	1.1095	1.1070	0.1872
0.9245	1.0290	1.1240	1.1130	0.4390
0.9573	0.9535	1.1637	0.9960	0.8735
0.9353	0.9918	1.1363	1.0600	0.3225
0.9465	0.9650	1.1500	1.0200	0.6641
0.9495	0.9795	1.1540	1.0320	0.6994
0.9140	1.0078	1.1093	1.1030	0.5238
0.9245	0.9745	1.1220	1.0540	0.3227
0.9288	1.0038	1.1277	1.0810	0.1075
0.9325	1.0230	1.1320	1.0970	0.5559
0.9535	0.9440	1.1573	0.9900	0.7836
0.9123	1.0223	1.1067	1.1210	0.0240
0.9160	1.0253	1.1113	1.1190	0.0019
0.9330	1.0175	1.1320	1.0910	0.0595
0.9368	0.9870	1.1360	1.0540	0.3134
0.9515	0.9185	1.1540	0.9650	0.3482
0.9650	0.8905	1.1710	0.9230	0.6304
0.9123	1.0348	1.1060	1.1340	0.1425
0.9265	1.0033	1.1233	1.0830	0.0591

0.9325	0.9878	1.1303	1.0590	0.1859
0.9423	0.9633	1.1417	1.0220	0.7809
0.9450	0.9715	1.1457	1.0280	0.7059
0.9640	0.9398	1.1680	0.9750	0.6891
0.8830	1.0953	1.0697	1.2400	0.0013
0.9135	1.0385	1.1060	1.1370	0.0853
0.9270	0.9965	1.1223	1.0750	0.1285
0.9315	0.9703	1.1280	1.0420	0.5747
0.9365	0.9650	1.1340	1.0300	0.4950
0.9465	0.9643	1.1467	1.0190	0.7959
0.9575	0.9085	1.1600	0.9490	0.6095
0.9745	0.9480	1.1800	0.9730	0.8142
0.9283	1.0038	1.1233	1.0810	0.2111
0.9315	0.9730	1.1270	1.0450	0.3010
0.9418	0.9795	1.1397	1.0400	0.3701
0.9888	0.8765	1.1963	0.8860	0.1276
1.0075	0.8955	1.2190	0.8890	0.3445
0.9115	1.0245	1.1023	1.1240	0.0032
0.9163	1.0120	1.1077	1.1050	0.0168
0.9240	0.9918	1.1173	1.0730	0.0644
0.9295	1.0338	1.1240	1.1120	0.0263
0.9305	1.0120	1.1253	1.0880	0.0554
0.9488	0.9643	1.1473	1.0160	0.7526
0.9273	1.0173	1.1203	1.0970	0.0537
0.9343	0.9983	1.1290	1.0690	0.1718
0.9388	0.9728	1.1337	1.0360	0.4306
0.9580	0.9410	1.1577	0.9820	0.7815
0.9603	0.9553	1.1597	0.9950	0.8876
0.9060	1.0223	1.0937	1.1280	0.0665
0.9240	0.9838	1.1157	1.0650	0.3075
0.9318	0.9925	1.1250	1.0650	0.0113
0.9640	0.9848	1.1633	1.0220	0.7670
0.9790	0.9105	1.1817	0.9300	0.0149

0.9840	0.9390	1.1880	0.9540	0.4068
1.0085	0.8995	1.2170	0.8920	0.4458
0.9350	0.9803	1.1277	1.0480	0.2925
0.9373	0.9913	1.1300	1.0580	0.2386
0.9375	0.9833	1.1307	1.0490	0.5918
0.9395	0.9758	1.1333	1.0390	0.6454
0.9545	0.9353	1.1507	0.9800	0.5851
0.9043	1.0295	1.0900	1.1390	0.0406
0.9178	1.0360	1.1060	1.1290	0.0810
0.9468	0.9703	1.1407	1.0250	0.7392
0.9470	0.9815	1.1413	1.0360	0.1335
0.9523	0.9625	1.1470	1.0110	0.9054
0.9548	0.9443	1.1507	0.9890	0.8841
0.9195	1.0310	1.1070	1.1210	0.0703
0.9285	0.9948	1.1177	1.0710	0.0313
0.9418	0.9813	1.1337	1.0420	0.3971
0.8645	1.1340	1.0400	1.3120	0.3369
0.9205	1.0265	1.1070	1.1150	0.0676
0.9265	0.9900	1.1150	1.0690	0.3942
0.9395	1.0093	1.1303	1.0740	0.1249
0.9438	0.9730	1.1353	1.0310	0.5592
0.9598	0.9663	1.1547	1.0070	0.7605
0.9450	0.9745	1.1360	1.0310	0.2691
0.9175	1.0490	1.1020	1.1430	0.3505
0.9205	1.0323	1.1053	1.1210	0.4661
0.9288	1.0085	1.1153	1.0860	0.2200
0.9488	0.9720	1.1393	1.0250	0.5915
0.9123	1.0423	1.0943	1.1430	0.4503
0.9200	1.0035	1.1040	1.0910	0.4972
0.9568	0.9518	1.1477	0.9950	0.9501
0.9665	0.9010	1.1595	0.9320	0.3685
0.9115	1.0448	1.0933	1.1460	0.0129
0.9225	1.0285	1.1060	1.1150	0.0331

0.9370	0.9955	1.1230	1.0620	0.3343
0.9370	0.9690	1.1230	1.0340	0.5086
0.9405	0.9803	1.1277	1.0420	0.5150
0.9525	0.9640	1.1417	1.0120	0.5259
0.9598	0.9513	1.1503	0.9910	0.8984
0.9760	0.9560	1.1700	0.9800	0.9383
0.7660	1.4100	0.9180	1.8410	0.7316
0.9330	0.9800	1.1180	1.0500	0.6740
0.9343	0.9953	1.1193	1.0650	0.3503
0.9513	0.9650	1.1397	1.0140	0.8235
0.9665	0.9280	1.1583	0.9600	0.6798
0.9700	0.9640	1.1620	0.9940	0.9481
0.8680	1.1245	1.0390	1.2960	0.0022
0.9150	1.0253	1.0950	1.1200	0.1859
0.9260	1.0120	1.1087	1.0930	0.0175
0.9305	0.9865	1.1140	1.0600	0.3457
0.9400	0.9665	1.1250	1.0280	0.6232
0.9613	0.9463	1.1503	0.9840	0.7125
0.9653	0.9560	1.1553	0.9900	0.9266
0.9145	1.0230	1.0940	1.1190	0.2597
0.9193	1.0430	1.0993	1.1350	0.0214
0.9385	1.0033	1.1220	1.0690	0.0870
0.9390	1.0180	1.1230	1.0840	0.2702
0.9405	0.9700	1.1250	1.0310	0.7176
0.9423	0.9980	1.1267	1.0590	0.5191
0.9470	0.9505	1.1325	1.0040	0.9906
0.9475	0.9883	1.1330	1.0430	0.4157
0.9545	0.9675	1.1413	1.0140	0.7884
0.9558	0.9550	1.1427	0.9990	0.9803
1.0000	0.8310	1.1955	0.8310	0.1613
0.8768	1.1073	1.0473	1.2630	0.0651
0.8810	1.1085	1.0530	1.2580	0.1135
0.9335	0.9853	1.1153	1.0550	0.2270

0.9400	1.0155	1.1230	1.0800	0.3997
0.9458	0.9603	1.1303	1.0150	0.7326
0.9760	0.9575	1.1660	0.9810	0.6341
0.9363	0.9933	1.1183	1.0610	0.3097
0.9378	0.9990	1.1200	1.0650	0.6597
0.9480	0.9760	1.1323	1.0300	0.4393
1.0450	0.8445	1.2480	0.8080	0.0829
0.9318	0.9995	1.1113	1.0730	0.1401
0.9453	0.9888	1.1280	1.0460	0.4281
0.9523	0.9323	1.1357	0.9790	0.6265
0.9188	1.0155	1.0953	1.1050	0.0035
0.9295	1.0135	1.1080	1.0900	0.1729
0.9308	1.0010	1.1097	1.0750	0.3669
0.9550	0.9753	1.1380	1.0210	0.3205
0.8650	1.1435	1.0305	1.3220	0.2113
0.9013	1.0700	1.0737	1.1870	0.0983
0.9108	1.0070	1.0843	1.1060	0.1609
0.9115	0.9675	1.0855	1.0610	0.3681
0.9270	1.0055	1.1037	1.0850	0.2150
0.9360	1.0025	1.1150	1.0710	0.0476
0.9790	0.9158	1.1657	0.9350	0.1822
0.9815	0.9490	1.1690	0.9670	0.5007
1.0020	0.8683	1.1930	0.8670	0.2515
0.9395	0.9690	1.1177	1.0310	0.5890
0.9525	0.9420	1.1330	0.9890	0.8360
1.0105	0.9030	1.2020	0.8940	0.1412
0.8938	1.0738	1.0630	1.2010	0.0322
0.9155	1.0465	1.0883	1.1430	0.0719
0.9178	1.0395	1.0910	1.1330	0.0968
0.9183	1.0285	1.0917	1.1200	0.0983
0.9218	1.0113	1.0960	1.0970	0.0600
0.9283	0.9903	1.1033	1.0670	0.1299
0.9353	1.0033	1.1123	1.0730	0.3794

0.9393	0.9735	1.1170	1.0360	0.5632
0.9475	0.9808	1.1267	1.0350	0.7033
0.9650	0.9465	1.1473	0.9810	0.6112
0.9085	1.0573	1.0797	1.1640	0.0174
0.9373	1.0035	1.1130	1.0710	0.4439
0.9388	0.9933	1.1153	1.0580	0.2536
0.9395	0.9903	1.1160	1.0540	0.5637
0.9430	0.9970	1.1203	1.0570	0.1150
0.9598	0.9253	1.1403	0.9640	0.4431
0.9070	1.0615	1.0770	1.1700	0.0544
0.9125	1.0493	1.0833	1.1500	0.0768
0.9425	0.9903	1.1187	1.0510	0.2328
0.9538	0.9720	1.1323	1.0190	0.9126
0.9550	0.9573	1.1337	1.0020	0.9837
0.9680	0.9725	1.1490	1.0050	0.7401
0.9168	1.0445	1.0873	1.1390	0.0130
0.9345	1.0183	1.1087	1.0900	0.0240
0.9410	1.0008	1.1157	1.0630	0.5272
0.9420	0.9730	1.1170	1.0330	0.7307
0.9430	0.9695	1.1180	1.0280	0.2753
0.9453	0.9888	1.1207	1.0460	0.5251
0.9545	0.8845	1.1320	0.9270	0.5626
0.9100	1.0450	1.0785	1.1480	0.5215
0.9338	1.0125	1.1067	1.0840	0.0971
0.9343	1.0000	1.1070	1.0700	0.0106
0.9585	0.9638	1.1360	1.0050	0.9114
0.9690	0.9315	1.1480	0.9610	0.0443
0.9173	1.0048	1.0860	1.0950	0.0065
0.9455	0.9795	1.1193	1.0360	0.7211
0.9595	0.9500	1.1360	0.9900	0.5898
0.9200	1.0190	1.0885	1.1080	0.2123
0.9225	1.0310	1.0915	1.1180	0.4986
0.9450	0.9938	1.1167	1.0520	0.0656

0.9453	0.9740	1.1177	1.0300	0.4382
0.9568	0.9708	1.1313	1.0150	0.6699
0.7975	1.2510	0.9420	1.5690	0.0003
0.9125	1.0378	1.0777	1.1370	0.0232
0.9315	1.0230	1.1000	1.0980	0.0879
0.9320	0.9985	1.1010	1.0710	0.6829
1.0290	0.8258	1.2153	0.8020	0.0526
1.0353	0.8160	1.2230	0.7880	0.1136
0.9230	1.0445	1.0893	1.1320	0.0340
0.9335	1.0005	1.1017	1.0720	0.3404
0.9395	0.9830	1.1085	1.0460	0.7945
0.9633	0.9458	1.1370	0.9820	0.9046
0.9000	1.0735	1.0615	1.1930	0.3203
0.9430	1.0263	1.1117	1.0880	0.0776
0.9675	0.9560	1.1407	0.9880	0.7865
0.9775	0.9635	1.1520	0.9860	0.8700
0.9965	0.9365	1.1750	0.9400	0.7256
0.9180	1.0340	1.0815	1.1260	0.3560
0.9360	0.9915	1.1025	1.0590	0.2536
0.9453	0.9540	1.1137	1.0090	0.8453
0.9613	0.9630	1.1327	1.0020	0.9409
0.9628	0.9833	1.1337	1.0210	0.4604
0.9958	0.9018	1.1733	0.9060	0.0803
0.9253	1.0203	1.0887	1.1030	0.1194
0.9488	0.9933	1.1167	1.0470	0.6033
0.9625	0.9573	1.1333	0.9950	0.9589
0.9325	1.0085	1.0965	1.0820	0.6762
0.9555	0.9505	1.1235	0.9950	0.9252
0.9833	0.9400	1.1563	0.9560	0.6602
0.9855	0.9190	1.1593	0.9330	0.0092
0.9265	0.9990	1.0890	1.0780	0.5360
0.9283	1.0233	1.0910	1.1020	0.6146
0.9295	1.0310	1.0920	1.1090	0.2606

0.9358	1.0190	1.0993	1.0890	0.1115
0.9388	1.0088	1.1033	1.0750	0.0985
0.9630	0.9380	1.1315	0.9740	0.9739
0.9915	0.9038	1.1650	0.9110	0.1263
0.9483	0.9838	1.1137	1.0370	0.3481
0.9593	0.9828	1.1260	1.0240	0.4481
0.9225	1.0345	1.0823	1.1210	0.0349
0.9235	0.9790	1.0830	1.0600	0.1974
0.9390	1.0080	1.1013	1.0730	0.0763
0.9555	0.9728	1.1207	1.0180	0.9006
1.0038	0.8790	1.1777	0.8760	0.0809
0.9200	1.0550	1.0780	1.1470	0.2333
0.9620	0.9718	1.1270	1.0100	0.5337
0.9635	0.9613	1.1290	0.9980	0.9410
0.9650	0.9825	1.1310	1.0180	0.7508
0.9690	0.9500	1.1360	0.9800	0.6527
0.9808	0.9285	1.1493	0.9470	0.4876
0.9030	1.0783	1.0570	1.1940	0.0428
0.9128	1.0695	1.0687	1.1720	0.0076
0.9248	1.0310	1.0830	1.1150	0.0863
0.9323	1.0118	1.0920	1.0850	0.1945
0.9378	1.0113	1.0983	1.0780	0.2061
0.9443	0.9798	1.1060	1.0380	0.4790
0.9450	1.0210	1.1070	1.0800	0.5112
0.9530	0.9873	1.1157	1.0360	0.2932
0.9705	0.9465	1.1367	0.9750	0.4879
0.9865	0.8855	1.1550	0.8980	0.0945
0.9960	0.8995	1.1667	0.9030	0.1287
0.9095	1.0570	1.0640	1.1620	0.1683
0.9320	1.0060	1.0905	1.0790	0.5694
0.9580	0.9500	1.1210	0.9920	0.8429
0.9615	0.9660	1.1250	1.0050	0.9136
0.9788	0.9275	1.1450	0.9480	0.6767

0.9208	1.0413	1.0767	1.1310	0.2036
0.9253	1.0340	1.0813	1.1180	0.1717
0.9618	0.9610	1.1243	0.9990	0.9648
0.9803	0.9300	1.1460	0.9490	0.5729
0.9908	0.9105	1.1587	0.9190	0.7677
0.9308	1.0293	1.0867	1.1060	0.0215
0.9340	1.0188	1.0910	1.0910	0.0238
0.9568	0.9413	1.1177	0.9840	0.9163
0.9588	0.9690	1.1200	1.0110	0.8828
0.9883	0.9115	1.1543	0.9220	0.0296
0.8913	1.1030	1.0403	1.2380	0.0416
0.9355	1.0360	1.0920	1.1070	0.2195
0.9455	0.9870	1.1030	1.0440	0.3176
0.9583	0.9853	1.1180	1.0280	0.1965
0.9595	0.9580	1.1200	0.9980	0.9129
0.9643	0.9705	1.1253	1.0060	0.6319
0.9898	0.9125	1.1547	0.9220	0.0737
0.9150	1.0535	1.0665	1.1510	0.1931
0.9220	1.0435	1.0747	1.1320	0.0194
0.9315	1.0270	1.0857	1.1030	0.0756
0.9443	1.0013	1.1010	1.0600	0.4619
0.9470	0.9760	1.1040	1.0310	0.5092
0.9598	0.9715	1.1193	1.0120	0.7363
0.9750	0.9755	1.1370	1.0010	0.9464
0.9808	0.9483	1.1437	0.9670	0.2340
0.9863	0.9190	1.1497	0.9320	0.1800
0.9930	0.9460	1.1580	0.9530	0.6119
0.9305	1.0323	1.0837	1.1090	0.0176
0.9495	0.9948	1.1063	1.0480	0.1040
0.9535	0.9868	1.1100	1.0350	0.7488
0.9748	0.9390	1.1347	0.9630	0.4799
0.9768	0.9598	1.1370	0.9830	0.6474
0.9780	0.9348	1.1387	0.9560	0.5387

1.0025	0.8840	1.1673	0.8820	0.0628
1.1195	0.7525	1.3030	0.6720	0.6367
0.9315	1.0140	1.0835	1.0890	0.1909
0.9833	0.9205	1.1433	0.9360	0.0600
0.8795	1.1260	1.0220	1.2800	0.1094
0.9208	1.0518	1.0703	1.1420	0.0072
0.9360	0.9888	1.0880	1.0560	0.0896
0.9475	1.0015	1.1010	1.0570	0.3752
0.9710	0.9335	1.1283	0.9610	0.2935
0.9258	1.0408	1.0743	1.1240	0.0939
0.9328	1.0433	1.0830	1.1180	0.1756
0.9328	1.0815	1.0833	1.1590	0.0085
0.9443	0.9848	1.0960	1.0430	0.3526
0.9460	1.0210	1.0980	1.0790	0.1378
0.9483	1.0018	1.1013	1.0560	0.0817
0.9605	0.9735	1.1153	1.0140	0.8688
0.9698	0.9480	1.1260	0.9780	0.7236
1.0113	0.8755	1.1743	0.8660	0.0518
1.0125	0.9135	1.1760	0.9020	0.0365
0.9290	1.0378	1.0780	1.1170	0.4985
0.9400	0.9913	1.0907	1.0550	0.0954
0.9430	1.0223	1.0940	1.0840	0.0897
0.9515	1.0125	1.1040	1.0640	0.0301
0.9710	0.9658	1.1263	0.9950	0.8840
0.9768	0.9445	1.1333	0.9670	0.7263
0.9768	0.9338	1.1333	0.9560	0.2184
0.9843	0.9193	1.1420	0.9340	0.3099
0.8998	1.0973	1.0425	1.2200	0.3371
0.9220	1.0475	1.0683	1.1360	0.2381
0.9420	1.0285	1.0920	1.0920	0.4616
0.9425	0.9970	1.0923	1.0580	0.1906
0.9430	1.0133	1.0933	1.0740	0.0344
0.9683	0.9600	1.1223	0.9910	0.8656

0.9830	0.9288	1.1390	0.9450	0.4864
0.9520	1.0218	1.1027	1.0730	0.1059
0.9565	0.9765	1.1080	1.0210	0.3471
0.9605	0.9935	1.1120	1.0340	0.9224
0.9675	0.9768	1.1203	1.0100	0.8800
0.9855	0.9303	1.1413	0.9440	0.4616
0.8988	1.0560	1.0397	1.1750	0.1763
0.9720	0.9545	1.1243	0.9820	0.2857
0.9778	0.9228	1.1310	0.9440	0.3251
1.0300	0.8875	1.1920	0.8620	0.3078
1.1018	0.6870	1.2750	0.6240	0.0791
0.9533	0.9968	1.1023	1.0460	0.7532
0.9703	0.9233	1.1213	0.9520	0.3253
0.9865	0.8883	1.1407	0.9000	0.3150
0.9063	1.0665	1.0470	1.1770	0.0053
0.9265	1.0340	1.0700	1.1160	0.1481
0.9455	1.0140	1.0917	1.0720	0.0438
0.9500	0.9895	1.0970	1.0420	0.3810
0.9515	0.9978	1.0990	1.0490	0.5030
0.9553	1.0530	1.1037	1.1020	0.0491
0.9648	0.9453	1.1147	0.9800	0.8134
0.9835	0.9450	1.1363	0.9610	0.3760
1.0115	0.8950	1.1680	0.8850	0.1104
1.0153	0.8380	1.1730	0.8250	0.0855
0.9260	1.0460	1.0690	1.1300	0.0879
0.9575	0.9898	1.1047	1.0340	0.5397
0.9590	0.9778	1.1063	1.0200	0.9941
0.9860	0.9930	1.1380	1.0070	0.7921
0.9928	0.9278	1.1460	0.9350	0.0044
0.9133	1.0828	1.0530	1.1860	0.0174
0.9378	1.0243	1.0817	1.0920	0.1142
0.9455	1.0240	1.0900	1.0830	0.1912
0.9675	0.9668	1.1153	0.9990	0.9940

0.9450	1.0125	1.0890	1.0710	0.3294
0.9515	0.9993	1.0960	1.0500	0.2040
0.9518	1.0008	1.0960	1.0510	0.5299
0.9515	1.0185	1.0960	1.0700	0.3682
0.9618	0.9700	1.1077	1.0090	0.8230
0.9775	0.9295	1.1265	0.9510	0.4899
0.9840	0.9070	1.1335	0.9220	0.2833
0.9200	1.0425	1.0593	1.1330	0.0262
0.9255	0.9248	1.0650	0.9990	0.8085
0.9275	1.0385	1.0680	1.1200	0.0907
0.9570	1.0030	1.1013	1.0480	0.4780
0.9775	0.9293	1.1247	0.9510	0.1601
1.0235	0.9000	1.1780	0.8790	0.0730
0.8900	1.1250	1.0235	1.2640	0.0992
0.8993	1.0955	1.0340	1.2180	0.0236
0.9233	1.0453	1.0620	1.1320	0.0135
0.9315	1.0495	1.0710	1.1270	0.3376
0.9615	0.9818	1.1053	1.0210	0.6238
0.9655	0.9560	1.1100	0.9900	0.9460
0.9688	0.9723	1.1140	1.0040	0.9728
0.9690	0.9688	1.1143	1.0000	0.9510
0.9385	1.0055	1.0785	1.0710	0.6540
0.9950	0.9163	1.1430	0.9210	0.3471
0.9015	1.0928	1.0353	1.2120	0.0114
0.9575	0.9845	1.0997	1.0280	0.5665
0.9765	0.9443	1.1213	0.9670	0.6504
0.9768	0.9598	1.1213	0.9830	0.6024
0.9973	0.9235	1.1450	0.9260	0.1018
0.9993	0.9150	1.1467	0.9160	0.0083
0.9995	0.9420	1.1470	0.9420	0.5412
0.9118	1.0600	1.0457	1.1630	0.0867
0.9263	1.0500	1.0620	1.1340	0.2271
0.9650	0.9780	1.1067	1.0130	0.7258

0.9813	0.9558	1.1257	0.9740	0.4997
0.9930	0.9118	1.1387	0.9180	0.1209
0.8625	1.1738	0.9880	1.3610	0.0981
0.8655	1.1675	0.9917	1.3490	0.0085
0.9173	1.0550	1.0510	1.1500	0.0650
0.9325	1.0305	1.0690	1.1050	0.3477
0.9683	0.9780	1.1093	1.0100	0.7852
0.9695	0.9490	1.1110	0.9790	0.7102
0.9810	0.9485	1.1240	0.9670	0.3080
0.9855	0.9443	1.1290	0.9580	0.2660
0.9893	0.9188	1.1333	0.9290	0.1590
1.0370	0.8830	1.1880	0.8510	0.0478
0.9320	1.1095	1.0670	1.1900	0.0183
0.9405	1.0285	1.0773	1.0940	0.2752
0.9418	0.9943	1.0787	1.0560	0.3147
0.9508	1.0075	1.0883	1.0600	0.3160
0.9588	0.9985	1.0977	1.0410	0.5064
0.9638	0.9740	1.1037	1.0110	0.8393
0.9888	0.9638	1.1317	0.9750	0.6406
1.0280	0.8980	1.1770	0.8740	0.3200
0.9403	1.0160	1.0757	1.0810	0.0075
0.9458	1.0305	1.0820	1.0900	0.0339
0.9498	1.0355	1.0863	1.0900	0.1028
0.9605	0.9938	1.0990	1.0350	0.2419
0.9498	0.9990	1.0860	1.0520	0.1793
0.9615	0.9863	1.0987	1.0260	0.7232
0.9693	0.9725	1.1077	1.0030	0.8851
0.9730	0.9605	1.1120	0.9870	0.7523
0.9855	0.9178	1.1263	0.9310	0.0887
1.0250	0.9000	1.1720	0.8780	0.1921
0.9125	1.0775	1.0425	1.1810	0.2036
0.9548	1.0063	1.0900	1.0540	0.3433
0.9808	0.9538	1.1200	0.9720	0.3267

0.9955	0.9210	1.1370	0.9250	0.1372
0.9960	0.9045	1.1377	0.9080	0.1579
0.9208	1.0648	1.0510	1.1560	0.2398
0.9215	1.0635	1.0513	1.1540	0.0026
0.9220	1.0890	1.0520	1.1810	0.1690
0.9605	0.9885	1.0957	1.0290	0.2115
0.9690	0.9930	1.1060	1.0250	0.4859
0.9725	0.9820	1.1100	1.0100	0.9449
0.9813	0.9740	1.1197	0.9930	0.8028
0.9843	0.9493	1.1227	0.9640	0.5692
0.9925	0.8915	1.1327	0.8980	0.3581
0.9945	0.9245	1.1343	0.9300	0.4492
1.0038	0.9050	1.1450	0.9020	0.1608
1.0140	0.9190	1.1570	0.9060	0.2405
1.0185	0.8753	1.1617	0.8590	0.1375
1.1530	0.6975	1.3160	0.6050	0.0313
0.9150	1.0403	1.0430	1.1370	0.5020
0.9180	1.0393	1.0467	1.1320	0.0171
0.9210	1.0643	1.0500	1.1560	0.0799
0.9370	1.0395	1.0680	1.1090	0.2498
0.9365	1.0250	1.0680	1.0950	0.1336
0.9500	0.9965	1.0830	1.0490	0.7189
0.9520	1.0188	1.0857	1.0700	0.4850
0.9553	0.9918	1.0893	1.0380	0.4350
0.9608	0.9760	1.0950	1.0160	0.7836
0.9665	0.9515	1.1015	0.9840	0.7470
0.9750	0.9365	1.1115	0.9610	0.7994
0.9145	1.0685	1.0413	1.1680	0.0228
0.9525	1.0005	1.0847	1.0500	0.1365
0.9653	0.9545	1.0997	0.9890	0.8961
0.9870	0.9645	1.1240	0.9770	0.8251
0.9925	0.9353	1.1307	0.9420	0.2453
1.0165	0.8953	1.1573	0.8810	0.1009

0.9125	1.0825	1.0385	1.1860	0.5802
0.9223	1.0668	1.0497	1.1570	0.1300
0.9480	1.0270	1.0790	1.0830	0.0135
0.9635	0.9665	1.0960	1.0030	0.9847
0.9653	0.9823	1.0983	1.0180	0.5706
0.9870	0.9665	1.1237	0.9790	0.7191
0.9895	0.9458	1.1257	0.9560	0.4287
0.9038	1.0818	1.0273	1.1970	0.0014
0.9310	1.0165	1.0590	1.0920	0.2733
0.9333	1.0423	1.0613	1.1170	0.0291
0.9465	0.9795	1.0760	1.0350	0.5736
0.9543	1.0043	1.0847	1.0520	0.1502
0.9648	0.9615	1.0970	0.9970	0.9674
0.9725	0.9643	1.1060	0.9920	0.6122
0.9773	0.9630	1.1110	0.9850	0.8667
0.9838	0.9475	1.1187	0.9630	0.3821
0.9905	0.9200	1.1260	0.9290	0.1630
1.0005	0.9140	1.1380	0.9140	0.2514
1.0305	0.8428	1.1720	0.8180	0.0814
0.9085	1.0805	1.0320	1.1890	0.0952
0.9570	0.9980	1.0873	1.0430	0.4597
0.9665	0.9450	1.0980	0.9780	0.8359
0.9730	0.9758	1.1057	1.0030	0.8728
0.9915	0.9310	1.1267	0.9390	0.0533
0.9315	1.0248	1.0573	1.1000	0.0232
0.9563	0.9990	1.0850	1.0450	0.5175
0.9760	0.9455	1.1080	0.9690	0.6162
0.9980	0.9038	1.1323	0.9060	0.4850
1.0070	0.9235	1.1433	0.9170	0.0509
0.9323	1.0513	1.0570	1.1280	0.0732
0.9610	0.9790	1.0900	1.0190	0.8015
0.9670	0.9825	1.0967	1.0160	0.7366
1.0263	0.8708	1.1637	0.8480	0.0109

0.9438	1.0328	1.0690	1.0940	0.2395
0.9590	0.9830	1.0865	1.0250	0.8242
0.9745	0.9680	1.1040	0.9930	0.8214
0.9870	0.9478	1.1183	0.9600	0.5174
0.9453	1.0235	1.0703	1.0830	0.1541
0.9573	1.0168	1.0837	1.0620	0.3542
0.9580	0.9728	1.0847	1.0150	0.7130
0.9618	0.9923	1.0873	1.0320	0.4118
0.9633	0.9903	1.0890	1.0280	0.5320
0.9748	1.0015	1.1020	1.0270	0.4594
0.9750	0.9945	1.1030	1.0200	0.8991
0.9860	0.9478	1.1150	0.9610	0.3948
0.9965	0.9228	1.1273	0.9260	0.2056
0.8983	1.1183	1.0150	1.2450	0.0994
0.9310	1.0580	1.0517	1.1360	0.0169
0.9675	0.9618	1.0937	0.9940	0.8111
0.9988	0.9210	1.1287	0.9220	0.2723
0.9303	1.0515	1.0500	1.1300	0.0051
0.9395	1.0345	1.0605	1.1010	0.6317
0.9550	0.9895	1.0783	1.0360	0.4926
0.9675	1.0030	1.0920	1.0370	0.7553
0.9775	0.9840	1.1040	1.0070	0.8009
1.0025	0.9180	1.1317	0.9160	0.2898
0.9218	1.0808	1.0400	1.1720	0.0379
0.9480	1.0430	1.0690	1.1000	0.1344
0.9520	1.0015	1.0737	1.0520	0.4992
0.9573	0.9935	1.0797	1.0380	0.2168
0.9665	0.9930	1.0900	1.0270	0.4697
0.9673	0.9905	1.0913	1.0240	0.6265
0.9783	0.9630	1.1030	0.9840	0.4834
0.9948	0.9590	1.1223	0.9640	0.7644
1.0430	0.8338	1.1770	0.7990	0.0768
0.9560	1.0238	1.0777	1.0710	0.0117

0.9700	0.9975	1.0930	1.0280	0.7594
0.9865	0.9725	1.1120	0.9860	0.5808
0.9980	0.9325	1.1243	0.9340	0.1603
1.0045	0.8815	1.1325	0.8780	0.0121
1.1105	0.7720	1.2510	0.6950	0.1269
0.9615	1.0135	1.0830	1.0540	0.3711
0.9700	1.0000	1.0920	1.0310	0.6279
0.9855	0.9235	1.1093	0.9370	0.0690
0.9860	0.9675	1.1100	0.9810	0.8912
0.9898	0.9533	1.1140	0.9630	0.3908
1.0065	0.9385	1.1330	0.9320	0.3165
0.9518	1.0093	1.0710	1.0600	0.3878
0.9590	0.9918	1.0790	1.0340	0.6910
0.9620	0.9948	1.0827	1.0340	0.3424
0.9620	0.9990	1.0827	1.0380	0.3826
0.9675	0.9985	1.0880	1.0320	0.9365
0.9693	0.9863	1.0903	1.0180	0.5110
0.9713	0.9795	1.0923	1.0080	0.9189
0.9710	0.9980	1.0923	1.0280	0.4478
0.9765	0.9603	1.0990	0.9830	0.6114
0.9913	0.9408	1.1153	0.9490	0.2117
0.9940	0.9355	1.1187	0.9410	0.0781
1.0595	0.8620	1.1920	0.8140	0.0482
0.9428	1.0508	1.0600	1.1150	0.6338
0.9615	0.9915	1.0810	1.0310	0.5161
0.9723	0.9648	1.0930	0.9920	0.8938
0.9735	0.9865	1.0940	1.0130	0.6580
0.9908	0.9328	1.1133	0.9410	0.1605
1.0185	0.9250	1.1450	0.9080	0.5418
0.9848	0.9485	1.1057	0.9630	0.2883
0.9865	0.9353	1.1080	0.9480	0.0726
0.9910	0.9320	1.1133	0.9400	0.1887
1.0010	0.9483	1.1243	0.9470	0.3316

0.9400	1.0325	1.0550	1.0980	0.2516
0.9690	0.9758	1.0870	1.0070	0.9654
0.9855	0.9315	1.1060	0.9450	0.1832
1.0080	0.9405	1.1310	0.9330	0.1150
1.1158	0.6978	1.2523	0.6250	0.0350
0.9263	1.0680	1.0383	1.1530	0.3285
0.9533	1.0253	1.0687	1.0760	0.0796
0.9580	1.0215	1.0740	1.0660	0.1931
0.9680	1.0050	1.0850	1.0380	0.6392
0.9740	0.9810	1.0923	1.0070	0.8759
0.8985	1.1113	1.0067	1.2370	0.0110
0.9485	1.0220	1.0625	1.0770	0.1571
0.9755	0.9800	1.0923	1.0050	0.9139
0.9765	0.9763	1.0933	1.0000	0.9758
0.9765	0.9728	1.0937	0.9960	0.9040
0.9818	0.9640	1.0993	0.9820	0.6762
0.9893	0.9575	1.1080	0.9680	0.5308
0.9900	0.9558	1.1087	0.9650	0.1345
0.9905	0.9273	1.1090	0.9360	0.2333
0.9925	0.9523	1.1120	0.9590	0.3719
1.0090	0.9365	1.1300	0.9280	0.4446
1.0005	0.9090	1.1200	0.9090	0.8755
1.0600	0.8575	1.1860	0.8090	0.1997
0.9595	0.9775	1.0725	1.0190	0.6197
0.9715	0.9768	1.0860	1.0050	0.8321
0.9795	0.9798	1.0947	1.0000	0.9969
1.0208	0.8920	1.1413	0.8740	0.0817
0.8780	1.1430	0.9810	1.3020	0.1616
0.9788	0.9705	1.0933	0.9920	0.8425
0.9940	0.9665	1.1100	0.9720	0.5272
0.9298	1.0595	1.0373	1.1400	0.4576
0.9538	1.0185	1.0643	1.0680	0.0304
0.9573	1.0183	1.0687	1.0640	0.0438

0.9600	0.9573	1.0713	0.9970	0.9096
0.9703	0.9970	1.0830	1.0280	0.6203
0.9835	0.9605	1.0980	0.9770	0.3795
1.0825	0.7755	1.2083	0.7160	0.0407
0.9313	1.0658	1.0380	1.1440	0.1214
0.9390	1.0500	1.0470	1.1180	0.2052
0.9578	1.0123	1.0680	1.0570	0.2699
0.9725	0.9735	1.0840	1.0010	0.9642
0.9763	0.9740	1.0883	0.9980	0.9360
0.9953	0.9478	1.1093	0.9520	0.1713
1.0165	0.9335	1.1330	0.9180	0.1433
0.9365	1.0580	1.0430	1.1300	0.2449
0.9630	0.9973	1.0727	1.0360	0.3328
0.9658	1.0005	1.0757	1.0360	0.5238
0.9853	0.9465	1.0980	0.9610	0.2178
0.9875	0.9543	1.1000	0.9660	0.5466
0.9893	0.9578	1.1020	0.9680	0.5120
0.9938	0.9323	1.1070	0.9380	0.5157
0.9968	0.9500	1.1103	0.9530	0.0201
0.9965	0.9278	1.1103	0.9310	0.0691
1.0148	0.8850	1.1303	0.8720	0.2545
0.9120	1.1198	1.0153	1.2280	0.3231
0.9183	1.0698	1.0223	1.1650	0.2289
0.9315	1.0730	1.0367	1.1520	0.0190
0.9420	1.0505	1.0480	1.1150	0.1873
0.9488	1.0023	1.0560	1.0560	0.6584
0.9770	0.9920	1.0870	1.0150	0.3945
0.9815	0.9560	1.0920	0.9740	0.6921
0.9865	0.9453	1.0980	0.9580	0.6761
1.0068	0.9145	1.1210	0.9080	0.0387
1.0145	0.9335	1.1290	0.9200	0.0144
1.0198	0.8920	1.1353	0.8750	0.1066
1.0315	0.8765	1.1477	0.8500	0.1514

0.9418	1.0720	1.0473	1.1380	0.0215
0.9830	0.9675	1.0927	0.9840	0.5664
0.9950	0.9275	1.1060	0.9320	0.2761
0.9995	0.8950	1.1113	0.8950	0.0032
1.0060	0.9340	1.1190	0.9280	0.2064
0.9590	1.0035	1.0655	1.0460	0.3325
0.9620	1.0188	1.0683	1.0590	0.0292
0.9623	1.0068	1.0693	1.0460	0.1549
0.9708	0.9685	1.0787	0.9980	0.9265
0.9893	0.9725	1.0987	0.9830	0.6520
0.9928	0.9458	1.1030	0.9530	0.5299
0.9930	0.9260	1.1035	0.9330	0.5473
1.0228	0.8923	1.1363	0.8720	0.1782
1.0250	0.9455	1.1390	0.9220	0.1733
0.9498	1.0270	1.0543	1.0810	0.1643
0.9550	1.0270	1.0600	1.0750	0.4371
0.9573	1.0120	1.0630	1.0570	0.0968
0.9795	0.9778	1.0877	0.9980	0.9855
0.9870	0.9455	1.0955	0.9580	0.5644
0.9920	0.9665	1.1010	0.9740	0.8352
0.9998	0.9110	1.1093	0.9110	0.0180
1.0083	0.9030	1.1187	0.8960	0.0264
0.9050	1.1060	1.0040	1.2220	0.0505
0.9410	1.0490	1.0435	1.1150	0.4007
0.9613	0.9693	1.0660	1.0080	0.8346
0.9635	1.0180	1.0690	1.0570	0.3455
0.9753	0.9775	1.0813	1.0020	0.9362
0.9775	0.9490	1.0840	0.9710	0.2542
0.9845	0.9645	1.0920	0.9800	0.7213
0.9880	0.9765	1.0960	0.9880	0.9457
0.9905	0.9868	1.0980	0.9960	0.9967
1.0003	0.9008	1.1097	0.9010	0.0343
1.0348	0.8913	1.1477	0.8610	0.0068

0.9665	0.9998	1.0713	1.0340	0.6043
0.9710	0.9825	1.0760	1.0120	0.9778
0.9773	0.9630	1.0827	0.9850	0.4434
0.9825	0.9760	1.0890	0.9930	0.7895
0.9865	0.9385	1.0927	0.9510	0.3645
0.9635	1.0140	1.0670	1.0520	0.6822
0.9653	0.9898	1.0683	1.0250	0.3105
0.9673	0.9863	1.0710	1.0200	0.7831
0.9725	0.9888	1.0767	1.0170	0.6996
0.9755	0.9848	1.0797	1.0090	0.9793
0.9833	0.9673	1.0883	0.9840	0.6834
0.9968	0.9445	1.1033	0.9480	0.0859
1.0105	0.9198	1.1183	0.9100	0.0393
1.0138	0.9218	1.1223	0.9090	0.4641
1.0780	0.7350	1.1930	0.6820	0.0291
0.9435	1.0468	1.0433	1.1090	0.0826
0.9558	1.0265	1.0570	1.0740	0.4667
0.9635	0.9950	1.0660	1.0330	0.3609
0.9820	0.9758	1.0857	0.9940	0.9098
1.0080	0.9225	1.1153	0.9150	0.0126
1.0188	0.9018	1.1270	0.8850	0.0607
0.9773	0.9863	1.0803	1.0090	0.8060
0.9783	0.9823	1.0807	1.0040	0.9490
0.9878	0.9625	1.0913	0.9740	0.5988
1.0008	0.9398	1.1060	0.9390	0.1824
1.0020	0.9388	1.1070	0.9370	0.2464
1.0295	0.8885	1.1377	0.8630	0.2298
0.9525	1.0135	1.0517	1.0640	0.2299
0.9535	1.0205	1.0525	1.0700	0.4461
0.9595	0.9815	1.0597	1.0230	0.7225
0.9633	1.0043	1.0633	1.0430	0.1738
0.9650	0.9955	1.0655	1.0320	0.9862
0.9855	0.9683	1.0883	0.9820	0.6102

0.9928	0.9640	1.0957	0.9710	0.1958
1.0070	0.9308	1.1113	0.9240	0.0785
1.0238	0.9050	1.1303	0.8840	0.0592
1.0473	0.8575	1.1567	0.8190	0.0011
0.9570	0.9885	1.0560	1.0330	0.7399
0.9663	0.9975	1.0657	1.0320	0.6791
0.9685	1.0073	1.0687	1.0400	0.2708
0.9730	0.9938	1.0733	1.0210	0.6732
0.9888	0.9458	1.0910	0.9570	0.0895
0.9978	0.9560	1.1003	0.9580	0.5764
1.0000	0.9560	1.1030	0.9560	0.7820
1.0060	0.9450	1.1093	0.9390	0.1159
0.9378	1.0533	1.0333	1.1230	0.0009
0.9470	1.0370	1.0440	1.0950	0.0755
0.9493	1.0335	1.0457	1.0890	0.1084
0.9635	1.0125	1.0617	1.0510	0.3513
0.9698	0.9988	1.0690	1.0300	0.6595
0.9713	1.0010	1.0707	1.0310	0.5451
0.9838	1.0035	1.0843	1.0200	0.7302
0.9920	1.0125	1.0935	1.0210	0.7813
1.0385	0.9030	1.1440	0.8700	0.2204
0.8730	1.1865	0.9610	1.3590	0.0044
0.9290	1.0720	1.0230	1.1540	0.2225
0.9373	1.0553	1.0320	1.1260	0.0297
0.9640	1.0053	1.0613	1.0430	0.2208
0.9830	0.9758	1.0827	0.9930	0.8596
0.9903	0.9593	1.0900	0.9690	0.3257
0.9973	0.9273	1.0980	0.9300	0.2069
1.0660	0.8265	1.1740	0.7750	0.0031
0.9555	0.9970	1.0510	1.0430	0.1212
0.9653	1.0005	1.0620	1.0370	0.7903
1.0025	0.9363	1.1027	0.9340	0.6007
1.0038	0.9500	1.1037	0.9460	0.0405

0.8693	1.1710	0.9553	1.3470	0.0051
1.0133	0.9188	1.1140	0.9070	0.3290
1.0288	0.8905	1.1303	0.8660	0.0030
0.9130	1.1175	1.0025	1.2240	0.0688
0.9445	1.0670	1.0367	1.1300	0.0177
0.9533	1.0405	1.0467	1.0920	0.0785
0.9723	0.9613	1.0680	0.9890	0.7081
0.9833	0.9778	1.0797	0.9940	0.8795
0.9860	0.9733	1.0830	0.9870	0.6796
0.9890	0.9410	1.0863	0.9510	0.2594
0.9985	0.9435	1.0967	0.9450	0.3859
1.0023	0.9075	1.1007	0.9050	0.0438
1.0200	0.9300	1.1200	0.9120	0.4515
1.0240	0.9100	1.1243	0.8890	0.1148
0.9885	1.0035	1.0847	1.0150	0.6781
1.0098	0.9415	1.1073	0.9320	0.1066
1.0245	0.8958	1.1240	0.8740	0.0676
0.8575	1.1770	0.9400	1.3730	0.7608
0.9263	1.1013	1.0150	1.1890	0.0003
0.9463	1.0225	1.0370	1.0810	0.2318
0.9685	1.0140	1.0613	1.0470	0.2116
0.9828	0.9870	1.0767	1.0040	0.8505
0.9885	0.9535	1.0830	0.9650	0.3264
1.0543	0.7790	1.1557	0.7390	0.0052
0.9395	1.0605	1.0290	1.1290	0.0220
0.9580	1.0190	1.0493	1.0640	0.1910
0.9583	1.0188	1.0497	1.0630	0.0173
0.9588	1.0378	1.0497	1.0820	0.1670
0.9793	0.9793	1.0723	1.0000	0.9990
0.9890	0.9525	1.0833	0.9630	0.1893
1.0095	0.8595	1.1057	0.8510	0.0195
1.0250	0.9085	1.1223	0.8860	0.0264
0.9385	1.1010	1.0267	1.1730	0.0118

0.9408	1.0490	1.0290	1.1150	0.4602
0.9610	1.0145	1.0515	1.0560	0.3266
0.9703	0.9950	1.0617	1.0260	0.3139
0.9705	0.9615	1.0620	0.9910	0.8054
0.9883	0.9720	1.0810	0.9840	0.7179
0.9945	0.9735	1.0880	0.9790	0.6468
1.0050	0.9373	1.0990	0.9330	0.1842
1.0050	0.9485	1.0990	0.9440	0.0446
1.0173	0.9190	1.1130	0.9030	0.0387
1.0193	0.9138	1.1153	0.8960	0.0429
0.9520	1.0420	1.0403	1.0950	0.1902
0.9575	1.0325	1.0470	1.0780	0.0872
0.9618	1.0000	1.0517	1.0400	0.4633
0.9925	0.9640	1.0843	0.9710	0.6286
0.9350	1.0670	1.0210	1.1410	0.3985
0.9520	1.0415	1.0397	1.0940	0.0826
0.9583	1.0188	1.0467	1.0630	0.1695
0.9725	1.0090	1.0620	1.0380	0.7774
0.9790	0.9935	1.0690	1.0150	0.8089
1.0015	0.9490	1.0937	0.9480	0.2863
1.0380	0.9055	1.1340	0.8720	0.1228
0.9315	1.0815	1.0160	1.1610	0.3565
0.9395	1.0435	1.0253	1.1110	0.1507
0.9623	1.0238	1.0497	1.0640	0.5141
0.9723	1.0025	1.0610	1.0310	0.7190
0.9743	0.9828	1.0627	1.0090	0.8360
0.9913	0.9735	1.0810	0.9820	0.9367
0.9303	1.0388	1.0137	1.1170	0.0063
0.9640	0.9980	1.0507	1.0350	0.3247
0.9670	0.9853	1.0540	1.0190	0.5367
0.9695	1.0050	1.0570	1.0370	0.5008
0.9753	0.9998	1.0633	1.0250	0.5468
0.9760	1.0060	1.0640	1.0310	0.4949

0.9785	0.9995	1.0670	1.0210	0.8330
1.0180	0.9365	1.1100	0.9200	0.7336
1.0335	0.9155	1.1270	0.8860	0.0155
0.8898	1.1583	0.9687	1.3020	0.0895
0.9845	0.9900	1.0720	1.0060	0.9783
0.9853	0.9790	1.0730	0.9940	0.8087
0.9865	0.9800	1.0740	0.9930	0.8469
1.0045	0.9660	1.0937	0.9620	0.3524
1.0245	0.9240	1.1160	0.9020	0.4594
1.0250	0.9290	1.1160	0.9060	0.4385
1.0460	0.8655	1.1393	0.8270	0.0300
1.0568	0.8370	1.1507	0.7920	0.0493
0.9505	1.0250	1.0343	1.0780	0.0728
0.9735	1.0048	1.0593	1.0320	0.2278
0.9768	0.9973	1.0627	1.0210	0.7275
0.9768	1.0108	1.0627	1.0350	0.6811
0.9775	0.9875	1.0637	1.0100	0.7810
0.9815	0.9785	1.0683	0.9970	0.9572
0.9893	0.9733	1.0760	0.9840	0.7163
0.9930	0.9505	1.0800	0.9570	0.5745
0.9955	0.9528	1.0830	0.9570	0.2590
1.0045	0.9433	1.0933	0.9390	0.0218
1.0140	0.9320	1.1033	0.9190	0.5191
1.0268	0.8980	1.1170	0.8750	0.1500
1.0330	0.8920	1.1237	0.8640	0.1963
1.0475	0.8965	1.1400	0.8560	0.2409
0.9453	1.0445	1.0277	1.1050	0.0226
0.9640	1.0048	1.0483	1.0420	0.3073
0.9670	1.0130	1.0510	1.0480	0.6934
0.9750	0.9940	1.0595	1.0190	0.9036
0.9750	0.9875	1.0595	1.0130	0.9927
0.9748	1.0013	1.0600	1.0270	0.6872
0.9853	0.9793	1.0713	0.9940	0.9185

0.9870	0.9780	1.0730	0.9910	0.7485
0.9920	0.9855	1.0780	0.9930	0.8226
0.9235	1.0885	1.0027	1.1790	0.0116
0.9310	1.0885	1.0110	1.1690	0.0838
0.9570	1.0203	1.0390	1.0660	0.0849
0.9573	1.0268	1.0400	1.0730	0.0144
0.9725	1.0070	1.0563	1.0350	0.2244
0.9788	0.9763	1.0630	0.9970	0.9223
1.0040	0.9630	1.0900	0.9590	0.2442
1.0240	0.9330	1.1120	0.9110	0.1870
0.9428	1.0450	1.0233	1.1080	0.0162
0.9563	1.0110	1.0373	1.0570	0.2924
0.9728	1.0148	1.0557	1.0430	0.3988
0.9755	0.9905	1.0580	1.0150	0.8931
0.9748	0.9488	1.0580	0.9730	0.7559
0.9825	0.9830	1.0657	1.0010	0.9945
0.9905	0.9575	1.0750	0.9670	0.1800
0.9915	0.9658	1.0753	0.9740	0.6332
0.9943	0.9513	1.0783	0.9570	0.0015
1.0035	0.9488	1.0890	0.9450	0.1262
1.0110	0.9373	1.0967	0.9270	0.3104
1.0695	0.8025	1.1603	0.7500	0.0955
0.7600	1.3390	0.8240	1.7620	0.1089
0.8765	1.1865	0.9503	1.3540	0.0088
0.9740	0.9955	1.0560	1.0220	0.3033
0.9743	0.9930	1.0563	1.0190	0.6017
0.9820	0.9890	1.0643	1.0070	0.8465
0.9820	0.9593	1.0643	0.9770	0.4407
0.9925	0.9670	1.0757	0.9740	0.5376
0.8838	1.1713	0.9570	1.3250	0.0012
0.9670	1.0185	1.0477	1.0530	0.1837
0.9685	1.0165	1.0493	1.0500	0.5508
0.9730	1.0090	1.0537	1.0370	0.5240

1.0000	0.9723	1.0830	0.9720	0.3891
0.9518	1.0445	1.0300	1.0970	0.1761
0.9550	1.0425	1.0333	1.0920	0.0436
0.9653	1.0233	1.0443	1.0600	0.3001
0.9653	0.9940	1.0447	1.0300	0.4046
0.9685	1.0230	1.0480	1.0560	0.3729
0.9813	1.0030	1.0617	1.0220	0.6042
1.0013	0.9538	1.0837	0.9530	0.3259
1.0035	0.9365	1.0860	0.9330	0.2852
0.9480	1.0178	1.0247	1.0740	0.2967
0.9490	1.0590	1.0263	1.1160	0.4841
0.9643	1.0208	1.0420	1.0590	0.0539
0.9700	1.0180	1.0490	1.0490	0.3881
0.9713	1.0255	1.0497	1.0560	0.4037
0.9865	0.9685	1.0665	0.9820	0.9093
1.0155	0.9465	1.0980	0.9320	0.3844
1.0305	0.9000	1.1143	0.8730	0.3341
1.1085	0.7065	1.1985	0.6370	0.0305
0.9345	1.0848	1.0097	1.1610	0.0106
0.9700	1.0423	1.0477	1.0740	0.0595
0.9860	1.0030	1.0650	1.0170	0.7421
0.9903	0.9743	1.0690	0.9840	0.7669
1.0288	0.8900	1.1107	0.8650	0.0155
0.9205	1.1075	0.9933	1.2030	0.0005
0.9700	1.0178	1.0470	1.0490	0.4060
0.9813	0.9943	1.0583	1.0130	0.7724
0.9848	0.9855	1.0623	1.0010	0.9852
0.9848	0.9840	1.0627	0.9990	0.9147
1.0070	0.9398	1.0867	0.9330	0.0775
0.9415	1.0555	1.0150	1.1210	0.2960
0.9490	1.0490	1.0233	1.1050	0.0202
0.9500	1.0148	1.0240	1.0680	0.0825
0.9540	1.0440	1.0285	1.0940	0.1448

0.9565	1.0428	1.0307	1.0900	0.1247
0.9575	1.0700	1.0320	1.1170	0.3746
0.9770	1.0055	1.0530	1.0290	0.7051
0.9818	0.9563	1.0583	0.9740	0.5301
1.0460	0.9010	1.1280	0.8610	0.0240
1.0503	0.8735	1.1317	0.8320	0.1677
1.2173	0.4398	1.3117	0.3610	0.0165
0.9660	0.9890	1.0403	1.0240	0.7643
0.9740	1.0000	1.0490	1.0270	0.8150
0.9843	0.9930	1.0600	1.0090	0.8405
0.9930	0.9400	1.0690	0.9470	0.2936
0.9925	0.9983	1.0693	1.0060	0.9448
1.0113	0.9378	1.0890	0.9270	0.0308
1.0160	0.9450	1.0940	0.9300	0.7172
0.9350	1.0795	1.0060	1.1550	0.3413
0.9445	1.0620	1.0163	1.1240	0.4119
0.9750	1.0088	1.0487	1.0350	0.6585
0.9855	0.9900	1.0600	1.0050	0.9377
0.9853	0.9873	1.0603	1.0020	0.9236
0.9200	1.0845	0.9890	1.1790	0.1390
0.9558	1.0438	1.0277	1.0920	0.0344
1.0055	0.9670	1.0810	0.9620	0.6130
1.0785	0.8408	1.1597	0.7800	0.2598
0.8900	1.1485	0.9560	1.2900	0.0182
0.9288	1.0753	0.9973	1.1580	0.1110
0.9680	1.0238	1.0397	1.0580	0.7061
0.9718	0.9600	1.0433	0.9880	0.7976
1.0000	0.9535	1.0740	0.9540	0.3699
1.0250	0.9235	1.1013	0.9010	0.0625
0.9450	1.0600	1.0140	1.1220	0.1914
0.9620	1.0365	1.0320	1.0770	0.2860
0.9630	1.0333	1.0330	1.0730	0.1030
0.9740	0.9923	1.0450	1.0190	0.6006

0.9880	1.0120	1.0600	1.0240	0.7451
0.9938	0.9813	1.0663	0.9870	0.7984
1.0468	0.8908	1.1227	0.8510	0.3061
1.0520	0.8698	1.1287	0.8270	0.1692
0.9545	1.0508	1.0230	1.1010	0.0322
0.9605	1.0385	1.0300	1.0810	0.0474
0.9625	1.0303	1.0317	1.0700	0.0500
0.9750	1.0198	1.0453	1.0460	0.6584
0.9933	0.9715	1.0650	0.9780	0.7459
1.0025	0.9543	1.0747	0.9520	0.6422
1.0170	0.9510	1.0900	0.9350	0.1777
1.0180	0.9495	1.0910	0.9330	0.1297
1.0448	0.8613	1.1197	0.8240	0.0056
1.0728	0.8368	1.1500	0.7800	0.2433
0.9580	1.0265	1.0257	1.0720	0.3119
0.9690	1.0160	1.0373	1.0490	0.1783
1.0360	0.8625	1.1095	0.8330	0.4261
0.9805	0.9955	1.0495	1.0150	0.5048
0.9845	0.9873	1.0533	1.0030	0.8732
0.9955	0.9673	1.0653	0.9720	0.3535
1.0043	0.9618	1.0743	0.9580	0.6398
1.0083	0.9518	1.0787	0.9440	0.5272
1.0080	0.9500	1.0790	0.9420	0.1932
0.9473	1.0370	1.0130	1.0950	0.3437
0.9515	1.0105	1.0170	1.0620	0.4906
0.9860	0.9865	1.0540	1.0010	0.9985
1.0075	0.9580	1.0770	0.9510	0.4899
1.0263	0.9145	1.0967	0.8910	0.0017
1.0373	0.8770	1.1083	0.8460	0.0566
1.0460	0.8868	1.1177	0.8480	0.1808
0.9563	1.0528	1.0213	1.1010	0.3453
0.9605	1.0415	1.0257	1.0840	0.0006
0.9695	1.0073	1.0353	1.0390	0.2489

0.9855	0.9893	1.0527	1.0040	0.8202
1.0110	0.9473	1.0797	0.9370	0.2599
1.0165	0.9540	1.0860	0.9390	0.4006
1.0260	0.9230	1.0960	0.9000	0.0569
1.0420	0.8600	1.1125	0.8250	0.2063
1.0473	0.8618	1.1187	0.8230	0.0061
0.9508	1.0463	1.0143	1.1000	0.0128
0.9595	1.0510	1.0233	1.0950	0.1903
0.9633	1.0343	1.0280	1.0740	0.2870
0.9803	0.9985	1.0457	1.0190	0.7161
0.9915	0.9665	1.0580	0.9750	0.5017
0.9933	0.9938	1.0597	1.0010	0.9573
0.9965	0.9753	1.0633	0.9790	0.6636
1.0060	0.9500	1.0735	0.9440	0.4179
0.9528	1.0325	1.0153	1.0840	0.4224
0.9690	1.0205	1.0330	1.0530	0.8073
0.9695	1.0260	1.0337	1.0580	0.1243
0.9735	1.0003	1.0377	1.0270	0.3914
0.9838	0.9810	1.0490	0.9970	0.9585
0.9908	0.9825	1.0557	0.9920	0.8552
0.9920	1.0108	1.0570	1.0190	0.4201
1.0075	0.9713	1.0743	0.9640	0.4930
1.0085	0.9508	1.0747	0.9430	0.3734
1.0435	0.9100	1.1120	0.8720	0.2994
1.0453	0.8940	1.1143	0.8550	0.0459
1.0460	0.8958	1.1147	0.8560	0.1427
1.0468	0.8700	1.1163	0.8310	0.0242
1.0508	0.8710	1.1200	0.8290	0.0698
0.9470	1.0710	1.0085	1.1310	0.3527
0.9508	1.0663	1.0127	1.1210	0.2625
0.9665	1.0265	1.0290	1.0620	0.5651
0.9895	0.9908	1.0537	1.0010	0.9769
1.0025	0.9760	1.0680	0.9740	0.4536

1.0145	0.9408	1.0807	0.9270	0.0528
0.9873	0.9905	1.0503	1.0030	0.9802
1.0100	0.9520	1.0750	0.9430	0.1893
1.0245	0.9000	1.0900	0.8780	0.0569
1.0965	0.7833	1.1663	0.7140	0.0199
0.9590	1.0485	1.0190	1.0930	0.4229
0.9963	0.9388	1.0590	0.9420	0.4937
0.9965	0.9833	1.0597	0.9870	0.3958
0.9980	0.9655	1.0610	0.9670	0.1208
1.0085	0.9310	1.0723	0.9230	0.0848
1.0620	0.9035	1.1290	0.8510	0.0134
1.0675	0.8760	1.1350	0.8210	0.0062
0.9563	1.0523	1.0157	1.1000	0.1606
0.9670	1.0350	1.0265	1.0700	0.4665
0.9758	0.9965	1.0360	1.0210	0.7178
1.0125	0.9280	1.0757	0.9170	0.1185
1.0135	0.9425	1.0763	0.9300	0.3673
1.0240	0.9028	1.0873	0.8820	0.0597
1.0260	0.9210	1.0897	0.8980	0.4140
0.9000	1.1850	0.9550	1.3170	0.2000
0.9848	1.0035	1.0447	1.0190	0.6862
0.9930	0.9715	1.0540	0.9780	0.8009
0.9973	0.9793	1.0580	0.9820	0.6402
1.0010	0.9800	1.0620	0.9790	0.2491
1.0028	0.9670	1.0640	0.9640	0.3704
1.0063	0.9630	1.0673	0.9570	0.2357
1.0055	0.9625	1.0673	0.9570	0.1899
1.0090	0.9675	1.0710	0.9590	0.6086
1.0180	0.9495	1.0800	0.9330	0.6226
0.9365	1.1015	0.9930	1.1760	0.0177
0.9695	1.0330	1.0275	1.0650	0.3343
0.9725	1.0235	1.0310	1.0520	0.1690
0.9793	1.0130	1.0377	1.0340	0.4404

0.9843	1.0013	1.0430	1.0170	0.8380
0.9963	0.9675	1.0560	0.9710	0.5363
1.0065	0.9735	1.0670	0.9670	0.4672
1.0093	0.9350	1.0697	0.9260	0.3188
1.0363	0.8870	1.0980	0.8560	0.0047
1.0838	0.8143	1.1490	0.7510	0.0097
1.0858	0.8240	1.1507	0.7590	0.0104
0.9293	1.0965	0.9840	1.1800	0.7528
0.9575	1.0583	1.0143	1.1050	0.3533
0.9790	1.0133	1.0367	1.0350	0.5343
0.9893	0.9793	1.0473	0.9900	0.8522
0.9908	0.9918	1.0493	1.0010	0.9388
0.9938	0.9868	1.0520	0.9930	0.9394
1.0005	0.9690	1.0600	0.9690	0.7459
0.9650	1.0375	1.0210	1.0750	0.0944
0.9965	0.9838	1.0540	0.9870	0.7616
1.0080	0.9615	1.0667	0.9540	0.6945
1.0203	0.9228	1.0793	0.9040	0.4385
0.9305	1.1090	0.9840	1.1920	0.1576
0.9493	1.0603	1.0037	1.1170	0.0065
0.9525	1.0698	1.0067	1.1230	0.3221
0.9775	1.0170	1.0330	1.0400	0.6022
0.9995	0.9620	1.0560	0.9620	0.7932
1.0050	0.9570	1.0620	0.9520	0.2522
1.0090	0.9345	1.0667	0.9260	0.1855
1.0348	0.9253	1.0933	0.8940	0.2785
0.9400	1.0820	0.9930	1.1510	0.4984
0.9680	1.0345	1.0220	1.0690	0.6125
0.9793	1.0143	1.0343	1.0360	0.4122
0.9873	0.9725	1.0427	0.9850	0.7320
0.9935	0.9850	1.0495	0.9910	0.9075
0.9955	0.9730	1.0517	0.9770	0.7461
1.0075	0.9435	1.0637	0.9360	0.3228

1.0113	0.9523	1.0683	0.9420	0.0888
1.0150	0.9558	1.0720	0.9420	0.1194
1.0238	0.9278	1.0810	0.9060	0.6435
0.9545	1.0678	1.0067	1.1190	0.0650
0.9570	1.0568	1.0100	1.1040	0.2225
0.9878	1.0000	1.0420	1.0120	0.7169
0.9898	1.0168	1.0447	1.0270	0.6676
0.9935	0.9833	1.0480	0.9900	0.6941
1.0013	0.9760	1.0563	0.9750	0.5260
1.0025	0.9848	1.0573	0.9820	0.4320
1.0760	0.7980	1.1350	0.7420	0.0395
0.9665	1.0340	1.0187	1.0700	0.4263
0.9875	0.9545	1.0410	0.9670	0.2945
0.9900	0.9910	1.0430	1.0010	0.9700
0.9218	1.1110	0.9703	1.2050	0.0186
0.9753	1.0263	1.0273	1.0520	0.5254
0.9763	1.0240	1.0277	1.0490	0.0111
0.9808	1.0163	1.0327	1.0360	0.4402
0.9835	0.9648	1.0360	0.9810	0.2459
0.9998	0.9808	1.0530	0.9810	0.7850
1.0025	0.9645	1.0553	0.9620	0.1991
1.0263	0.9303	1.0803	0.9060	0.1756
0.9458	1.0548	0.9947	1.1150	0.2398
0.9550	1.0555	1.0047	1.1050	0.3534
0.9560	1.0535	1.0060	1.1020	0.0728
0.9605	1.0500	1.0100	1.0930	0.0417
0.9798	1.0065	1.0303	1.0270	0.5836
1.0330	0.9315	1.0870	0.9020	0.5147
0.8998	1.1615	0.9457	1.2910	0.0025
0.9573	1.0593	1.0060	1.1070	0.2323
0.9590	1.0548	1.0077	1.1000	0.0752
0.9740	1.0300	1.0240	1.0570	0.1887
0.9805	0.9905	1.0303	1.0100	0.8164

0.9963	0.9935	1.0470	0.9970	0.9593
1.0088	0.9608	1.0600	0.9520	0.1194
1.0465	0.9033	1.0997	0.8630	0.1134
0.9470	1.0510	0.9940	1.1100	0.4220
0.9613	1.0418	1.0097	1.0840	0.2592
0.9730	1.0328	1.0217	1.0610	0.1949
0.9755	1.0170	1.0247	1.0430	0.3888
0.9775	1.0230	1.0260	1.0470	0.6470
0.9800	1.0070	1.0290	1.0280	0.8878
0.9875	0.9970	1.0373	1.0100	0.7465
0.9935	1.0215	1.0433	1.0280	0.9034
0.9960	0.9950	1.0460	0.9990	0.9695
1.0155	0.9335	1.0660	0.9190	0.7167
0.9758	1.0278	1.0237	1.0530	0.2583
0.9845	0.9820	1.0323	0.9970	0.9596
0.9915	0.9808	1.0397	0.9890	0.8003
1.0070	0.9645	1.0560	0.9580	0.6393
1.0115	0.9735	1.0610	0.9620	0.8895
1.0140	0.9350	1.0640	0.9220	0.3744
1.0165	0.9665	1.0660	0.9510	0.7121
1.1058	0.7913	1.1603	0.7160	0.0143
0.9858	0.9875	1.0333	1.0020	0.9727
0.9980	0.9640	1.0463	0.9660	0.5066
1.0018	0.9678	1.0497	0.9660	0.1262
1.0058	0.9618	1.0540	0.9560	0.4209
1.0215	0.9275	1.0710	0.9080	0.1378
1.0395	0.8950	1.0890	0.8610	0.2219
1.0465	0.8700	1.0967	0.8310	0.0040
1.0585	0.8713	1.1097	0.8230	0.0500
1.0718	0.8500	1.1233	0.7930	0.0101
0.9725	1.0385	1.0187	1.0680	0.2030
0.9793	1.0130	1.0253	1.0340	0.3503
0.9865	1.0033	1.0333	1.0170	0.4324

0.9880	1.0095	1.0340	1.0220	0.8512
0.9980	0.9885	1.0447	0.9900	0.8647
0.9990	0.9838	1.0460	0.9850	0.5760
1.0055	0.9785	1.0530	0.9730	0.7897
1.0243	0.9570	1.0720	0.9340	0.1726
1.0580	0.8905	1.1073	0.8420	0.0281
0.9835	1.0120	1.0290	1.0290	0.8434
0.9875	0.9965	1.0327	1.0090	0.6651
0.9990	0.9800	1.0445	0.9810	0.8259
1.0058	1.0043	1.0517	0.9990	0.9779
1.0300	0.9310	1.0777	0.9040	0.2248
1.0735	0.8483	1.1233	0.7900	0.2451
0.9730	1.0340	1.0170	1.0630	0.0679
0.9780	1.0250	1.0220	1.0480	0.6346
0.9870	1.0070	1.0313	1.0200	0.7041
1.0008	0.9825	1.0453	0.9820	0.7393
1.0010	0.9783	1.0457	0.9770	0.6314
0.9768	1.0028	1.0200	1.0270	0.3920
1.0345	0.9370	1.0800	0.9060	0.0030
1.0948	0.8048	1.1433	0.7350	0.0033
1.1120	0.7340	1.1610	0.6600	0.7315
0.9915	1.0010	1.0345	1.0100	0.9538
0.9933	1.0008	1.0363	1.0080	0.8550
1.0023	0.9545	1.0453	0.9520	0.1327
1.0083	0.9780	1.0520	0.9700	0.5265
1.0115	0.9645	1.0547	0.9540	0.3354
0.9930	0.9925	1.0343	0.9990	0.9649
0.9948	0.9955	1.0367	1.0010	0.9712
1.0085	0.9655	1.0505	0.9570	0.6381
1.0090	0.9548	1.0510	0.9460	0.5282
1.0090	0.9745	1.0513	0.9660	0.3551
1.0120	0.9733	1.0543	0.9620	0.2467
1.0213	0.9468	1.0637	0.9270	0.0325

1.0225	0.9533	1.0650	0.9320	0.0447
1.0285	0.9318	1.0720	0.9060	0.1554
1.0300	0.9305	1.0730	0.9030	0.0011
1.0415	0.8848	1.0853	0.8490	0.0575
0.9605	1.0328	1.0003	1.0750	0.5658
0.9775	0.9930	1.0180	1.0160	0.7754
0.9825	1.0145	1.0227	1.0330	0.3440
0.9838	1.0235	1.0237	1.0400	0.4452
1.0075	0.9685	1.0485	0.9610	0.4289
1.0198	0.9408	1.0613	0.9230	0.1328
1.0250	0.9585	1.0670	0.9350	0.7197
1.0295	0.9350	1.0713	0.9080	0.1885
1.0600	0.8855	1.1030	0.8350	0.1143
1.0645	0.8408	1.1087	0.7900	0.0056
0.9383	1.1125	0.9757	1.1860	0.0450
0.9550	1.0600	0.9930	1.1100	0.5512
0.9663	1.0485	1.0050	1.0850	0.4852
0.9810	1.0195	1.0200	1.0390	0.6296
0.9880	1.0125	1.0280	1.0250	0.5921
0.9970	0.9943	1.0370	0.9970	0.9479
1.0483	0.8940	1.0907	0.8530	0.4437
1.0510	0.8903	1.0930	0.8470	0.8187
1.0790	0.8448	1.1217	0.7830	0.0643
0.9420	1.1048	0.9787	1.1730	0.1731
0.9708	1.0565	1.0087	1.0880	0.1715
0.9878	1.0048	1.0267	1.0170	0.6818
1.0035	0.9865	1.0430	0.9830	0.8460
1.0060	0.9758	1.0450	0.9700	0.4249
1.0085	0.9655	1.0480	0.9570	0.7565
1.2115	0.5773	1.2587	0.4760	0.0090
0.9410	1.0678	0.9767	1.1350	0.0413
0.9473	1.0890	0.9837	1.1500	0.1036
0.9893	0.9858	1.0267	0.9960	0.9323

0.9915	1.0048	1.0293	1.0130	0.4798
1.0098	0.9608	1.0480	0.9510	0.2416
0.9610	1.0555	0.9965	1.0980	0.7185
0.9728	1.0053	1.0090	1.0330	0.2199
0.9960	0.9713	1.0327	0.9750	0.3959
0.9968	1.0010	1.0333	1.0040	0.9771
1.0038	1.0133	1.0407	1.0090	0.9248
1.0205	0.9610	1.0580	0.9420	0.6014
1.0235	0.9448	1.0610	0.9230	0.4916
1.0245	0.9553	1.0627	0.9320	0.5878
1.0685	0.7995	1.1080	0.7480	0.2449
0.9385	1.1180	0.9725	1.1910	0.2724
0.9873	0.9975	1.0223	1.0100	0.7001
0.9895	1.0078	1.0253	1.0180	0.9091
0.9980	0.9768	1.0340	0.9790	0.6975
0.9985	0.9918	1.0343	0.9930	0.9510
1.0110	0.9715	1.0470	0.9610	0.5023
1.0120	0.9685	1.0480	0.9570	0.3995
1.0215	0.9795	1.0585	0.9590	0.0385
1.0305	0.9515	1.0680	0.9230	0.2033
0.9610	1.0525	0.9950	1.0950	0.3346
1.0008	0.9893	1.0353	0.9890	0.8402
1.0028	0.9665	1.0383	0.9640	0.7287
1.0053	0.9785	1.0407	0.9730	0.4218
1.0355	0.9360	1.0720	0.9040	0.3802
1.0465	0.9120	1.0833	0.8710	0.5203
0.9083	1.1338	0.9390	1.2480	0.0271
0.9288	1.1083	0.9603	1.1930	0.1230
1.0098	0.9453	1.0443	0.9360	0.2749
1.0280	0.9515	1.0630	0.9260	0.0916
1.0388	0.9148	1.0743	0.8810	0.0010
0.9470	1.0925	0.9787	1.1540	0.1026
0.9773	1.0355	1.0097	1.0600	0.6168

0.9885	0.9910	1.0210	1.0030	0.7358
1.0045	0.9805	1.0380	0.9760	0.6250
1.0073	0.9803	1.0407	0.9730	0.5605
0.8930	1.1565	0.9220	1.2950	0.1823
0.9150	1.1108	0.9440	1.2140	0.0345
0.9880	1.0155	1.0200	1.0280	0.7508
0.9918	0.9873	1.0233	0.9950	0.9228
0.9928	1.0060	1.0250	1.0130	0.8548
0.9980	0.9908	1.0303	0.9930	0.9249
1.0035	0.9880	1.0360	0.9850	0.7837
1.0048	1.0125	1.0370	1.0080	0.7873
1.0628	0.8873	1.0963	0.8350	0.0165
0.8738	1.2300	0.9007	1.4080	0.0219
0.9648	1.0520	0.9950	1.0900	0.0724
0.9720	1.0420	1.0020	1.0720	0.4932
0.9945	0.9775	1.0250	0.9830	0.7125
0.9958	0.9818	1.0267	0.9860	0.7012
0.9978	0.9893	1.0283	0.9910	0.7881
1.0010	0.9895	1.0317	0.9890	0.6895
1.0130	0.9313	1.0440	0.9190	0.2162
1.0250	0.9580	1.0570	0.9350	0.1838
1.1020	0.8385	1.1360	0.7610	0.0435
0.9590	1.0728	0.9877	1.1190	0.0661
0.9615	1.1025	0.9905	1.1470	0.1069
0.9838	1.0205	1.0137	1.0370	0.7051
0.9880	1.0130	1.0180	1.0250	0.8417
0.9935	1.0070	1.0230	1.0140	0.8160
0.9948	1.0195	1.0243	1.0250	0.5597
0.9965	1.0023	1.0263	1.0060	0.9427
1.0023	0.9660	1.0327	0.9640	0.6977
1.0095	0.9850	1.0400	0.9760	0.7596
1.0465	0.9255	1.0777	0.8840	0.0120
1.0610	0.8980	1.0930	0.8460	0.3309

0.9670	1.0678	0.9947	1.1040	0.0860
0.9888	1.0178	1.0177	1.0290	0.4218
0.9935	1.0065	1.0220	1.0130	0.6189
0.9985	0.9990	1.0277	1.0010	0.9968
1.0000	0.9970	1.0287	0.9970	0.9299
0.9210	1.1318	0.9467	1.2290	0.0171
0.9563	1.0438	0.9830	1.0920	0.1260
0.9705	1.0365	0.9980	1.0680	0.0604
0.9790	1.0285	1.0060	1.0510	0.4061
0.9935	1.0005	1.0210	1.0070	0.6039
0.9985	0.9983	1.0267	1.0000	0.9468
1.0005	0.9975	1.0290	0.9970	0.9605
1.0025	0.9880	1.0310	0.9860	0.8078
1.0298	0.9675	1.0590	0.9400	0.3155
1.0760	0.8275	1.1065	0.7690	0.0861
1.0900	0.8275	1.1200	0.7590	0.0045
0.9900	1.0145	1.0167	1.0250	0.5288
1.0198	0.9635	1.0473	0.9450	0.4003
1.0250	0.9673	1.0527	0.9440	0.3502
1.0328	0.9328	1.0607	0.9030	0.3284
1.0405	0.9438	1.0687	0.9070	0.0872
1.0493	0.9058	1.0780	0.8630	0.0402
1.0660	0.8728	1.0943	0.8190	0.0210
0.9438	1.1040	0.9683	1.1700	0.1012
0.9898	0.9888	1.0157	0.9990	0.9867
0.9923	0.9850	1.0177	0.9930	0.7905
0.9990	1.0125	1.0247	1.0140	0.7032
0.9985	0.9660	1.0247	0.9670	0.6951
1.0210	0.9613	1.0477	0.9410	0.1301
0.9405	1.1070	0.9640	1.1770	0.1482
0.9470	1.1060	0.9707	1.1680	0.0164
0.9630	1.0713	0.9867	1.1120	0.1255
0.9845	1.0330	1.0090	1.0490	0.6741

0.9915	1.0120	1.0160	1.0210	0.6358
1.0025	0.9228	1.0280	0.9200	0.0293
1.0105	0.9943	1.0357	0.9840	0.6577
1.0293	0.9453	1.0550	0.9180	0.1799
1.0480	0.8995	1.0740	0.8580	0.2789
1.0488	0.9103	1.0753	0.8680	0.0172
1.0653	0.8695	1.0923	0.8160	0.0007
0.9255	1.0843	0.9480	1.1720	0.1999
0.9323	1.1158	0.9547	1.1970	0.3167
0.9483	1.0925	0.9707	1.1520	0.0649
1.0058	0.9848	1.0300	0.9790	0.1904
0.9633	1.0865	0.9853	1.1280	0.0338
0.9943	1.0008	1.0170	1.0070	0.8673
1.0025	0.9945	1.0253	0.9920	0.8705
1.0800	0.8760	1.1050	0.8110	0.0656
0.9660	1.0913	0.9870	1.1300	0.1279
0.9698	1.0503	0.9913	1.0830	0.3635
0.9788	1.0015	1.0000	1.0230	0.3019
0.9985	1.0040	1.0203	1.0060	0.9279
1.0140	0.9800	1.0360	0.9660	0.6867
1.0238	0.9435	1.0463	0.9220	0.0874
1.0350	0.9340	1.0573	0.9020	0.1579
0.9730	1.0398	0.9933	1.0690	0.2286
0.9838	1.0153	1.0047	1.0320	0.6519
1.0110	0.9765	1.0320	0.9660	0.7016
1.0135	0.9795	1.0350	0.9660	0.3710
1.0213	0.9820	1.0430	0.9620	0.2332
1.0235	0.9600	1.0450	0.9380	0.7631
1.0265	0.9475	1.0485	0.9230	0.2507
1.0415	0.9270	1.0633	0.8900	0.0205
0.9840	1.0435	1.0040	1.0600	0.8724
0.9965	1.0080	1.0160	1.0120	0.8341
1.0268	0.9783	1.0477	0.9530	0.5419

0.9200	1.1050	0.9375	1.2010	0.4090
0.9618	1.0830	0.9797	1.1260	0.2773
0.9625	1.0460	0.9810	1.0870	0.1548
0.9855	1.0205	1.0040	1.0360	0.1369
0.9978	0.9753	1.0167	0.9770	0.8765
1.0095	0.9850	1.0290	0.9760	0.7303
1.0195	0.9560	1.0390	0.9380	0.1494
1.0313	0.9505	1.0503	0.9220	0.1126
1.0348	0.9210	1.0543	0.8900	0.0505
1.0470	0.9303	1.0673	0.8880	0.0757
1.0515	0.9390	1.0713	0.8930	0.0073
1.0863	0.8415	1.1067	0.7750	0.0194
0.9528	1.0948	0.9697	1.1490	0.2162
0.9530	1.0745	0.9700	1.1270	0.0125
0.9555	1.0940	0.9727	1.1450	0.0084
0.9845	1.0305	1.0020	1.0470	0.7107
0.9903	1.0218	1.0083	1.0320	0.5487
1.0063	0.9690	1.0247	0.9630	0.5455
1.0115	0.9795	1.0300	0.9680	0.5096
1.0433	0.9290	1.0623	0.8900	0.1223
1.0623	0.8868	1.0813	0.8350	0.0390
0.9135	1.1305	0.9290	1.2380	0.2303
0.9985	1.0040	1.0150	1.0060	0.9956
0.9993	1.0100	1.0160	1.0110	0.7819
1.0050	0.9953	1.0223	0.9900	0.7855
1.0088	0.9840	1.0263	0.9750	0.6688
1.0210	0.9890	1.0387	0.9690	0.2560
1.0738	0.8640	1.0917	0.8050	0.0576
1.0878	0.8765	1.1060	0.8060	0.1938
0.9740	1.0465	0.9900	1.0740	0.5112
0.9810	1.0133	0.9970	1.0330	0.5343
0.9875	1.0175	1.0030	1.0300	0.9258
1.0050	0.9675	1.0207	0.9630	0.4414

1.0053	0.9953	1.0213	0.9900	0.7111
1.0358	0.9453	1.0527	0.9130	0.1050
1.0400	0.9315	1.0563	0.8960	0.0230
0.9725	1.0465	0.9870	1.0760	0.2687
0.9733	1.0380	0.9883	1.0670	0.0182
0.9765	1.0578	0.9910	1.0830	0.2205
0.9785	1.0355	0.9930	1.0580	0.4263
0.9875	1.0360	1.0020	1.0490	0.1861
0.9945	0.9990	1.0093	1.0050	0.9149
0.9953	1.0173	1.0100	1.0220	0.6444
1.0018	1.0008	1.0170	0.9990	0.9264
1.0110	0.9875	1.0260	0.9770	0.6131
1.0233	0.9650	1.0387	0.9430	0.1713
1.0305	0.9600	1.0460	0.9320	0.6192
1.0318	0.9323	1.0477	0.9040	0.1552
1.0398	0.9320	1.0550	0.8960	0.0803
0.9723	1.0555	0.9857	1.0860	0.2518
0.9875	1.0170	1.0017	1.0300	0.3216
0.9955	1.0163	1.0090	1.0210	0.1599
0.9990	1.0173	1.0130	1.0180	0.6934
0.9993	1.0083	1.0133	1.0090	0.7822
0.9995	1.0085	1.0137	1.0090	0.7432
1.0020	0.9790	1.0163	0.9770	0.7982
1.0060	0.9813	1.0200	0.9750	0.4671
1.0183	0.9733	1.0320	0.9560	0.0355
1.0323	0.9363	1.0467	0.9070	0.0434
1.0395	0.9235	1.0540	0.8880	0.1373
1.0443	0.9105	1.0587	0.8720	0.0751
0.9865	1.0318	0.9990	1.0460	0.5338
0.9893	1.0240	1.0017	1.0350	0.6239
1.0065	1.0010	1.0193	0.9950	0.8521
1.0240	0.9680	1.0370	0.9450	0.5856
1.0798	0.8113	1.0940	0.7510	0.0100

0.9553	1.0830	0.9670	1.1340	0.1430
0.9625	1.0793	0.9740	1.1210	0.1766
0.9860	1.0450	0.9980	1.0600	0.5653
0.9933	1.0243	1.0053	1.0310	0.3470
1.0203	0.9740	1.0330	0.9550	0.2160
1.0275	0.9650	1.0400	0.9390	0.1666
1.0443	0.9310	1.0563	0.8920	0.1127
1.0760	0.8845	1.0890	0.8220	0.2072
1.0875	0.8853	1.1003	0.8140	0.0583
1.0928	0.8530	1.1063	0.7810	0.0060
1.1050	0.8415	1.1180	0.7620	0.1092
0.9900	1.0193	1.0010	1.0300	0.5240
0.9923	1.0110	1.0033	1.0190	0.7510
1.0018	1.0175	1.0127	1.0160	0.2396
1.0055	0.9955	1.0170	0.9900	0.8933
1.0428	0.9168	1.0547	0.8790	0.0897
1.0480	0.9310	1.0600	0.8880	0.0268
1.0748	0.8718	1.0863	0.8110	0.0095
0.9638	1.0758	0.9733	1.1160	0.2147
0.9780	1.0380	0.9880	1.0610	0.6253
0.9913	1.0095	1.0013	1.0180	0.6437
1.0035	1.0048	1.0137	1.0010	0.9515
1.0105	0.9863	1.0210	0.9760	0.4260
1.0210	0.9680	1.0315	0.9480	0.2748
1.0233	0.9658	1.0330	0.9440	0.0240
1.0513	0.8945	1.0617	0.8510	0.2230
1.0720	0.8745	1.0830	0.8160	0.0194
0.9820	1.0540	0.9910	1.0730	0.1302
0.9915	1.0340	1.0005	1.0430	0.1994
1.0028	0.9845	1.0113	0.9820	0.4918
1.0043	1.0020	1.0133	0.9980	0.9314
1.0275	0.9653	1.0370	0.9390	0.2898
1.1115	0.7780	1.1220	0.7000	0.0244

1.1955	0.6420	1.2063	0.5370	0.0460
0.9845	1.0103	0.9927	1.0260	0.6676
0.9898	1.0268	0.9977	1.0370	0.4565
0.9915	1.0085	0.9993	1.0170	0.6745
1.0078	1.0033	1.0160	0.9960	0.8946
1.0145	0.9675	1.0230	0.9540	0.3251
1.0170	0.9468	1.0250	0.9310	0.1188
1.0233	0.9713	1.0313	0.9490	0.4140
1.0288	0.9695	1.0367	0.9420	0.1847
1.0400	0.9275	1.0480	0.8920	0.3239
0.9800	1.0525	0.9867	1.0740	0.3105
0.9945	1.0540	1.0010	1.0600	0.6326
1.0010	1.0125	1.0080	1.0110	0.9901
1.0105	0.9948	1.0180	0.9840	0.3794
1.0105	0.9890	1.0180	0.9790	0.7693
1.0215	0.9735	1.0290	0.9530	0.4349
1.0355	0.9310	1.0430	0.8990	0.5348
0.9483	1.0500	0.9537	1.1070	0.0138
0.9918	1.0338	0.9977	1.0420	0.6952
1.0033	1.0125	1.0090	1.0090	0.8582
1.0035	1.0063	1.0100	1.0030	0.9094
1.0080	0.9980	1.0140	0.9900	0.9264
1.0195	0.9838	1.0253	0.9650	0.6107
1.0200	0.9815	1.0260	0.9620	0.5159
1.0245	0.9645	1.0310	0.9410	0.1129
1.0340	0.9440	1.0405	0.9130	0.1391
1.0403	0.9620	1.0467	0.9250	0.1518
0.9685	1.0618	0.9737	1.0960	0.0369
0.9805	1.0675	0.9855	1.0890	0.8242
0.9815	1.0380	0.9860	1.0580	0.2376
0.9865	1.0270	0.9913	1.0410	0.3579
1.0393	0.9408	1.0443	0.9050	0.1609
1.0498	0.9233	1.0547	0.8790	0.1399

1.0495	0.9490	1.0550	0.9040	0.0209
0.9543	1.0833	0.9580	1.1350	0.0145
0.9793	1.0468	0.9830	1.0690	0.1357
1.0018	0.9903	1.0053	0.9890	0.7172
1.0065	0.9803	1.0110	0.9740	0.3298
1.0163	0.9625	1.0203	0.9470	0.4410
1.0205	0.9760	1.0250	0.9560	0.3753
1.0425	0.9353	1.0470	0.8970	0.1954
1.0483	0.8778	1.0523	0.8370	0.0466
1.0510	0.9270	1.0550	0.8820	0.5147
0.9808	1.0433	0.9837	1.0640	0.0893
0.9955	1.0188	0.9983	1.0230	0.3473
0.9978	0.9628	1.0007	0.9650	0.7033
1.0003	1.0145	1.0037	1.0140	0.8797
1.0058	1.0080	1.0087	1.0020	0.9248
1.0058	1.0000	1.0090	0.9940	0.7906
1.0095	1.0025	1.0125	0.9930	0.9262
1.0103	0.9918	1.0130	0.9820	0.6300
1.0120	0.9885	1.0150	0.9770	0.8254
1.0168	0.9750	1.0193	0.9590	0.4648
1.0205	0.9780	1.0233	0.9580	0.4288
1.0493	0.8908	1.0527	0.8490	0.0611
0.9810	1.0625	0.9825	1.0830	0.2541
0.9848	1.0418	0.9870	1.0580	0.5391
0.9888	1.0363	0.9903	1.0480	0.4486
1.0018	1.0125	1.0037	1.0110	0.9022
1.0060	1.0080	1.0080	1.0020	0.9987
1.0065	1.0055	1.0085	0.9990	0.9548
1.0190	0.9780	1.0210	0.9600	0.2705
0.9683	1.0790	0.9697	1.1140	0.1360
0.9715	1.0855	0.9720	1.1170	0.4320
0.9768	1.0483	0.9777	1.0730	0.0322
0.9800	1.0635	0.9810	1.0850	0.2862

0.9833	1.0425	0.9840	1.0600	0.1936
0.9883	1.0260	0.9890	1.0380	0.3434
0.9953	1.0285	0.9967	1.0330	0.0919
1.0098	0.9928	1.0110	0.9830	0.3423
1.0095	1.0070	1.0110	0.9980	0.9858
1.0145	0.9898	1.0153	0.9760	0.5398
1.0153	1.0070	1.0167	0.9920	0.8424
1.0173	0.9825	1.0180	0.9660	0.4669
1.0180	0.9805	1.0193	0.9630	0.0742
1.0248	0.9925	1.0253	0.9690	0.5443
1.0300	0.9615	1.0310	0.9330	0.6128
1.0305	0.9605	1.0320	0.9320	0.1580
1.0313	0.9603	1.0327	0.9310	0.0309
1.0435	0.9325	1.0447	0.8940	0.0849
1.0748	0.8933	1.0757	0.8310	0.2386
1.0770	0.8845	1.0777	0.8210	0.2441
0.9893	1.0235	0.9890	1.0350	0.7544
0.9980	1.0135	0.9980	1.0160	0.7944
1.0105	0.9675	1.0100	0.9570	0.3356
1.0190	0.9565	1.0190	0.9390	0.1899
1.0498	0.9215	1.0497	0.8780	0.1211
1.0545	0.9260	1.0540	0.8780	0.2872
1.0680	0.8965	1.0677	0.8390	0.0601
1.1040	0.8298	1.1037	0.7520	0.0009
0.9810	1.0415	0.9800	1.0620	0.2824
0.9913	1.0408	0.9907	1.0500	0.4066
0.9913	1.0355	0.9907	1.0450	0.6911
0.9935	1.0505	0.9927	1.0570	0.6267
0.9985	1.0285	0.9975	1.0300	0.9373
1.0060	1.0183	1.0047	1.0120	0.6798
1.0090	1.0023	1.0077	0.9930	0.8303
1.0125	0.9935	1.0110	0.9810	0.8051
1.0123	0.9945	1.0113	0.9820	0.5910

1.0195	0.9855	1.0180	0.9670	0.6713
1.0263	0.9853	1.0250	0.9600	0.2691
1.0310	0.9130	1.0300	0.8860	0.2472
1.0370	0.9490	1.0357	0.9150	0.0306
1.0383	0.9530	1.0370	0.9180	0.0109
1.0470	0.9358	1.0460	0.8940	0.1980
0.9475	1.0975	0.9460	1.1580	0.4002
0.9818	1.0555	0.9797	1.0750	0.7147
0.9880	1.0515	0.9865	1.0640	0.1450
0.9905	1.0093	0.9883	1.0190	0.6819
0.9965	1.0250	0.9947	1.0290	0.5213
1.0018	1.0330	0.9997	1.0310	0.3156
1.0320	0.9575	1.0297	0.9280	0.0570
1.0683	0.8970	1.0663	0.8400	0.2180
0.9978	0.9755	0.9947	0.9780	0.4887
1.0063	1.0160	1.0030	1.0100	0.9600
1.0225	0.9770	1.0193	0.9560	0.4208
1.1263	0.7785	1.1230	0.6910	0.0135
0.9390	1.1363	0.9350	1.2100	0.3529
0.9940	1.0303	0.9897	1.0360	0.4246
1.0048	0.9885	1.0010	0.9840	0.7763
1.0280	0.9885	1.0243	0.9620	0.2791
1.0348	0.9380	1.0303	0.9060	0.3283
1.0645	0.9155	1.0600	0.8600	0.2135
0.9125	1.2135	0.9075	1.3300	0.0343
0.9590	1.0663	0.9540	1.1120	0.0777
0.9885	1.0275	0.9840	1.0390	0.7134
1.0043	1.0093	0.9993	1.0050	0.8010
1.0143	0.9793	1.0097	0.9650	0.2588
1.0185	0.9588	1.0137	0.9410	0.3136
1.0223	0.9815	1.0173	0.9600	0.4457
1.0263	0.9723	1.0210	0.9470	0.3583
1.0343	0.9570	1.0293	0.9250	0.3488

1.0710	0.9040	1.0660	0.8440	0.0911
0.9260	1.1250	0.9200	1.2150	0.0478
0.9455	1.1138	0.9400	1.1780	0.0550
0.9923	1.0163	0.9863	1.0240	0.2605
0.9970	1.0280	0.9913	1.0310	0.7105
1.0048	1.0243	0.9983	1.0190	0.6292
1.0050	1.0313	0.9987	1.0260	0.3832
1.0073	1.0118	1.0013	1.0040	0.8530
1.0338	0.9783	1.0277	0.9460	0.4247
1.0948	0.8305	1.0877	0.7590	0.0024
1.0120	1.0125	1.0050	1.0000	0.9728
1.0130	1.0080	1.0060	0.9950	0.9352
1.0135	1.0035	1.0065	0.9900	0.8885
1.0283	0.9713	1.0210	0.9450	0.1830
1.0328	0.9345	1.0260	0.9050	0.5434
1.0490	0.9258	1.0420	0.8830	0.0743
0.9328	1.1450	0.9250	1.2280	0.4910
0.9780	1.0438	0.9700	1.0670	0.3377
1.0015	1.0350	0.9930	1.0330	0.0724
1.0020	1.0053	0.9943	1.0030	0.9090
1.0170	0.9905	1.0093	0.9740	0.2831
1.0288	0.9665	1.0203	0.9390	0.0966
1.0555	0.9295	1.0470	0.8810	0.2530
1.0763	0.8815	1.0677	0.8190	0.1089
1.0935	0.8338	1.0843	0.7620	0.0622
0.9725	1.0370	0.9640	1.0660	0.7821
0.9770	1.0233	0.9680	1.0470	0.1918
1.0120	1.0088	1.0027	0.9970	0.9062
1.0138	0.9963	1.0050	0.9830	0.8995
1.0183	0.9935	1.0093	0.9760	0.2516
1.0188	1.0068	1.0100	0.9880	0.4209
1.0203	0.9928	1.0110	0.9730	0.1446
1.0310	0.9670	1.0220	0.9380	0.4044

1.0358	0.9593	1.0263	0.9260	0.1867
1.0563	0.9190	1.0463	0.8700	0.0927
1.0695	0.8865	1.0600	0.8290	0.1990
1.0968	0.8440	1.0873	0.7700	0.0277
0.9548	1.0888	0.9457	1.1400	0.4884
0.9570	1.0938	0.9477	1.1430	0.2808
0.9930	0.9995	0.9833	1.0070	0.8694
0.9933	1.0458	0.9837	1.0530	0.4170
0.9973	1.0230	0.9870	1.0260	0.5262
1.0083	1.0058	0.9980	0.9980	0.9064
1.0420	0.9505	1.0320	0.9120	0.3984
1.0525	0.9245	1.0415	0.8780	0.0740
1.0603	0.9153	1.0493	0.8630	0.0014
0.9455	1.1020	0.9353	1.1660	0.1973
0.9990	1.0403	0.9880	1.0410	0.4701
1.0025	0.9743	0.9913	0.9720	0.6169
1.0115	0.9673	1.0007	0.9560	0.2277
1.0265	0.9933	1.0157	0.9680	0.5652
1.0300	0.9918	1.0190	0.9630	0.1950
1.0475	0.9345	1.0360	0.8920	0.2309
1.0528	0.9285	1.0417	0.8820	0.0409
1.0910	0.8360	1.0790	0.7660	0.0592
1.0975	0.8680	1.0850	0.7910	0.1012
0.9300	1.1340	0.9187	1.2190	0.2089
0.9933	1.0240	0.9817	1.0310	0.5332
1.0008	0.9843	0.9883	0.9840	0.6835
1.0033	1.0190	0.9917	1.0160	0.5736
1.0058	1.0150	0.9940	1.0090	0.6183
1.0400	0.9520	1.0280	0.9150	0.5053
1.0430	0.9990	1.0300	0.9580	0.6584
1.0610	0.9080	1.0485	0.8560	0.1694
0.9883	1.0298	0.9753	1.0420	0.3471
0.9905	1.0375	0.9780	1.0470	0.2479

0.9955	1.0240	0.9830	1.0290	0.6918
0.9963	1.0383	0.9837	1.0420	0.2946
0.9970	1.0258	0.9837	1.0290	0.3237
1.0005	1.0220	0.9870	1.0210	0.7784
1.0190	1.0003	1.0053	0.9820	0.8978
1.0468	0.9453	1.0333	0.9030	0.4610
1.0515	0.9445	1.0380	0.8980	0.2550
0.9908	1.0283	0.9770	1.0380	0.3708
1.0020	1.0053	0.9880	1.0030	0.9736
1.0055	1.0213	0.9913	1.0160	0.7633
1.0070	1.0010	0.9933	0.9940	0.8190
1.0158	1.0005	1.0013	0.9850	0.6123
1.0205	1.0083	1.0067	0.9880	0.7013
1.0420	0.9308	1.0270	0.8930	0.0197
1.0545	0.9380	1.0400	0.8900	0.2823
1.0700	0.8880	1.0550	0.8300	0.1824
0.9800	1.0653	0.9650	1.0870	0.0909
1.0000	1.0220	0.9850	1.0220	0.7804
1.0053	0.9968	0.9903	0.9920	0.7632
1.0195	0.9855	1.0040	0.9670	0.7286
1.0388	0.9583	1.0233	0.9230	0.0300
1.0640	0.9168	1.0483	0.8620	0.0030
1.0930	0.8425	1.0765	0.7710	0.0879
0.9648	1.0605	0.9493	1.0990	0.1137
0.9903	1.0403	0.9740	1.0500	0.4148
0.9940	1.0420	0.9777	1.0480	0.4876
1.0145	0.9753	0.9987	0.9610	0.3608
1.0340	0.9305	1.0175	0.9000	0.2038
0.9465	1.0985	0.9300	1.1610	0.1747
0.9770	1.0523	0.9603	1.0770	0.2265
0.9908	1.0448	0.9740	1.0550	0.6071
0.9948	1.0258	0.9777	1.0310	0.6785
1.0088	1.0090	0.9913	1.0000	0.9783

1.0180	0.9970	1.0010	0.9790	0.8150
1.0280	0.9825	1.0105	0.9560	0.6676
1.0330	0.9725	1.0155	0.9410	0.5192
1.0395	0.9580	1.0220	0.9220	0.3435
1.0510	0.9373	1.0327	0.8920	0.2307
0.9715	1.0205	0.9540	1.0500	0.1644
0.9800	1.0648	0.9627	1.0860	0.1339
0.9908	1.0665	0.9727	1.0760	0.1381
1.0028	1.0380	0.9850	1.0350	0.6295
1.0140	0.9985	0.9960	0.9850	0.3642
1.0200	1.0010	1.0015	0.9810	0.7853
1.0353	0.9625	1.0170	0.9300	0.4872
1.0398	0.9718	1.0207	0.9350	0.2309
1.0525	0.9325	1.0340	0.8860	0.0207
1.0000	0.9930	0.9813	0.9930	0.8752
1.0073	1.0190	0.9877	1.0120	0.9056
1.0345	0.9705	1.0147	0.9380	0.2012
1.0415	0.9558	1.0213	0.9180	0.2287
1.0620	0.9340	1.0420	0.8790	0.0201
1.0710	0.8910	1.0510	0.8320	0.2996
1.0115	1.0040	0.9910	0.9930	0.8990
1.0210	0.9868	1.0007	0.9660	0.6604
1.0270	0.9845	1.0065	0.9590	0.2738
1.0470	0.9490	1.0257	0.9060	0.2693
1.1170	0.8385	1.0950	0.7510	0.1417
0.9985	1.0145	0.9780	1.0160	0.8184
1.0180	0.9903	0.9967	0.9730	0.5019
1.0430	0.9480	1.0207	0.9090	0.0479
1.0438	0.9510	1.0217	0.9110	0.0348
1.0630	0.9250	1.0410	0.8700	0.0117
0.9295	1.1170	0.9090	1.2020	0.6547
0.9985	1.0555	0.9770	1.0570	0.6768
1.0045	1.0175	0.9820	1.0130	0.8848

1.0045	1.0365	0.9825	1.0320	0.3079
1.0305	0.9745	1.0080	0.9460	0.5008
1.0348	0.9723	1.0117	0.9400	0.2300
1.0370	0.9650	1.0140	0.9310	0.1918
1.0450	0.9530	1.0220	0.9120	0.3761
1.0603	0.9295	1.0367	0.8770	0.3034
1.0653	0.9170	1.0417	0.8610	0.0100
0.9890	1.0400	0.9660	1.0520	0.2949
0.9965	1.0275	0.9740	1.0310	0.3296
1.0093	1.0180	0.9860	1.0090	0.9099
1.0098	1.0175	0.9870	1.0080	0.8790
1.0143	0.9993	0.9910	0.9850	0.6691
1.0170	0.9845	0.9940	0.9680	0.0530
1.0190	1.0030	0.9955	0.9840	0.8857
1.0230	0.9793	0.9997	0.9570	0.1283
1.0270	0.9885	1.0030	0.9630	0.5370
1.0348	0.9520	1.0110	0.9200	0.3400
1.0388	0.9283	1.0147	0.8940	0.1269
1.0645	0.9250	1.0400	0.8690	0.1208
1.0705	0.9078	1.0463	0.8480	0.0099
1.0853	0.8488	1.0603	0.7820	0.0038
1.1003	0.8658	1.0750	0.7870	0.1249
0.9985	1.0598	0.9750	1.0610	0.4251
1.0128	1.0120	0.9880	0.9990	0.9893
1.0140	1.0045	0.9900	0.9910	0.9311
1.0153	1.0095	0.9913	0.9940	0.9374
1.0178	1.0048	0.9933	0.9870	0.8086
1.0195	0.9938	0.9947	0.9750	0.8356
1.0620	1.0290	1.0370	0.9690	0.5207
1.2485	0.6620	1.2180	0.5300	0.3550
0.9440	1.1060	0.9200	1.1720	0.2655
1.0093	0.9918	0.9843	0.9830	0.7856
1.0110	1.0180	0.9860	1.0070	0.8962

1.0128	0.9870	0.9870	0.9750	0.3651
1.0175	1.0115	0.9925	0.9940	0.8446
1.0190	0.9670	0.9937	0.9490	0.2533
1.0210	0.9988	0.9953	0.9780	0.6834
1.0483	0.9483	1.0223	0.9050	0.2616
1.0578	0.9298	1.0313	0.8790	0.0638
0.9528	1.0973	0.9277	1.1520	0.0576
0.9695	1.0688	0.9440	1.1020	0.0094
1.0198	1.0003	0.9930	0.9810	0.5759
1.0240	0.9840	0.9970	0.9610	0.7236
1.0275	0.9830	1.0010	0.9570	0.2454
1.0305	0.9813	1.0033	0.9520	0.2240
1.0310	0.9788	1.0040	0.9490	0.2331
1.0508	0.9613	1.0230	0.9150	0.2425
1.0575	0.9285	1.0295	0.8780	0.0760
0.8715	1.2675	0.8483	1.4540	0.0265
0.9448	1.1103	0.9193	1.1750	0.6792
0.9718	1.0900	0.9453	1.1220	0.0271
1.0105	1.0095	0.9830	0.9990	0.9697
1.0310	0.9750	1.0030	0.9460	0.4877
1.0315	0.9905	1.0040	0.9600	0.5745
1.0325	0.9915	1.0043	0.9600	0.8598
1.0365	0.9798	1.0087	0.9450	0.4900
1.0740	0.9003	1.0453	0.8380	0.1279
0.9793	1.0418	0.9520	1.0640	0.0362
0.9815	1.0535	0.9540	1.0730	0.6704
1.0193	0.9978	0.9910	0.9790	0.6342
1.0200	1.0115	0.9910	0.9920	0.9234
1.0265	0.9970	0.9975	0.9710	0.3256
1.0333	0.9723	1.0043	0.9410	0.4071
1.0460	0.9635	1.0167	0.9210	0.1919
0.9898	1.0525	0.9613	1.0630	0.3937
0.9920	1.0335	0.9630	1.0420	0.7618

1.0000	1.0090	0.9710	1.0090	0.9641
1.0130	1.0245	0.9840	1.0110	0.9557
1.0163	1.0160	0.9863	1.0000	0.9661
1.0165	1.0150	0.9870	0.9990	0.9854
1.0188	1.0205	0.9893	1.0020	0.9404
1.0285	0.9848	0.9983	0.9570	0.5622
1.0293	0.9720	0.9993	0.9440	0.3829
1.0415	0.9868	1.0110	0.9470	0.0425
1.0560	0.9523	1.0250	0.9020	0.0716
0.9355	1.1260	0.9070	1.2040	0.1312
1.0030	1.0353	0.9730	1.0320	0.6926
1.0195	0.9818	0.9893	0.9630	0.6106
1.0323	0.9738	1.0010	0.9430	0.0373
1.0350	0.9765	1.0040	0.9430	0.6501
1.0445	0.9705	1.0130	0.9290	0.4251
0.9958	1.0465	0.9647	1.0510	0.3733
1.0025	1.0230	0.9710	1.0200	0.7518
1.0070	1.0218	0.9760	1.0150	0.6297
1.0160	1.0058	0.9850	0.9900	0.7575
1.0183	1.0070	0.9867	0.9890	0.7690
1.0210	0.9920	0.9890	0.9720	0.7576
1.0240	1.0763	0.9923	1.0510	0.4069
1.0435	0.9680	1.0117	0.9280	0.0876
1.0643	0.9315	1.0317	0.8750	0.7388
0.9725	1.0983	0.9410	1.1290	0.1253
0.9880	1.0635	0.9567	1.0760	0.2373
1.0008	1.0388	0.9690	1.0380	0.5282
1.0105	1.0200	0.9785	1.0090	0.9015
1.0115	1.0200	0.9787	1.0080	0.8117
1.0195	1.0040	0.9873	0.9850	0.7288
1.0310	0.9895	0.9985	0.9600	0.7606
1.0315	0.9875	0.9990	0.9570	0.7274
1.0423	0.9593	1.0090	0.9200	0.0476

1.0565	0.9405	1.0230	0.8900	0.0402
1.0715	0.9145	1.0370	0.8530	0.3341
1.0783	0.9153	1.0433	0.8490	0.1327
1.0870	0.8995	1.0520	0.8280	0.3965
1.1300	0.8665	1.0940	0.7670	0.2889
0.9768	1.0865	0.9447	1.1120	0.2394
0.9905	1.0320	0.9580	1.0420	0.4834
0.9950	1.0590	0.9620	1.0640	0.3151
0.9968	1.0515	0.9637	1.0550	0.3641
0.9988	1.0610	0.9653	1.0620	0.4844
1.0023	1.0203	0.9690	1.0180	0.6136
1.0033	1.0180	0.9700	1.0150	0.7017
1.0053	1.0100	0.9717	1.0050	0.8403
1.0090	1.0090	0.9760	1.0000	0.9921
1.0105	1.0053	0.9767	0.9950	0.8053
1.0165	1.0178	0.9833	1.0010	0.9449
1.0255	1.0100	0.9920	0.9850	0.9693
1.0475	0.9345	1.0133	0.8920	0.2772
1.0718	0.9300	1.0367	0.8680	0.4343
1.0938	0.8878	1.0580	0.8120	0.0058
1.1033	0.8553	1.0663	0.7750	0.0577
0.8280	1.2830	0.8000	1.5500	0.0465
0.9455	1.1100	0.9130	1.1740	0.0525
0.9853	1.0673	0.9517	1.0830	0.2018
0.9850	1.0575	0.9520	1.0740	0.4358
1.0430	0.9643	1.0080	0.9240	0.0646
1.0445	0.9660	1.0090	0.9250	0.0989
0.9595	1.0878	0.9260	1.1340	0.5922
0.9735	1.0880	0.9393	1.1180	0.0278
0.9798	1.0573	0.9450	1.0790	0.1347
0.9803	1.1065	0.9463	1.1290	0.0704
0.9928	1.0553	0.9580	1.0630	0.0878
1.0005	1.0235	0.9653	1.0230	0.7638

1.0095	1.0115	0.9740	1.0020	0.9791
1.0105	0.9933	0.9747	0.9830	0.7280
1.0280	0.9835	0.9920	0.9570	0.2744
1.0380	0.9755	1.0017	0.9400	0.2336
1.0860	0.8975	1.0480	0.8260	0.1132
0.9788	1.1038	0.9437	1.1280	0.1997
0.9970	1.0660	0.9610	1.0690	0.1981
1.0265	0.9870	0.9900	0.9620	0.6005
1.0360	0.9705	0.9990	0.9370	0.6319
1.0410	0.9688	1.0033	0.9310	0.0547
1.0445	0.9435	1.0073	0.9030	0.0137
1.0475	0.9693	1.0097	0.9250	0.2451
1.0578	0.9543	1.0197	0.9020	0.1059
1.0615	0.9355	1.0235	0.8810	0.1551
0.9390	1.1285	0.9040	1.2020	0.4697
1.0015	1.0380	0.9640	1.0360	0.1183
1.0098	0.9845	0.9723	0.9750	0.4558
1.0183	0.9915	0.9807	0.9740	0.5566
1.0215	0.9935	0.9840	0.9730	0.7912
1.0258	1.0050	0.9880	0.9800	0.7558
1.0310	0.9913	0.9933	0.9610	0.7136
1.0518	0.9630	1.0130	0.9160	0.3017
1.0525	0.9500	1.0140	0.9030	0.0236
1.1500	0.7970	1.1080	0.6930	0.2061
0.9783	1.0753	0.9410	1.0990	0.2230
0.9958	1.0468	0.9583	1.0510	0.6257
1.0043	1.0625	0.9660	1.0580	0.4904
1.0415	0.9675	1.0020	0.9290	0.3490
1.0508	0.9535	1.0110	0.9070	0.1031
1.0510	0.9555	1.0110	0.9090	0.1795
1.0640	0.9450	1.0240	0.8880	0.0147
0.9830	1.0698	0.9443	1.0880	0.5165
0.9930	1.0830	0.9545	1.0910	0.5313

1.0208	1.0110	0.9807	0.9900	0.8320
1.0320	0.9785	0.9920	0.9480	0.7900
1.0378	0.9788	0.9973	0.9430	0.5343
1.0800	0.8883	1.0383	0.8220	0.1034
1.1020	0.8485	1.0585	0.7700	0.1903
1.1180	0.8393	1.0740	0.7510	0.0257
1.1555	0.7310	1.1110	0.6330	0.0702
0.9685	1.1068	0.9300	1.1430	0.2712
1.0008	1.0260	0.9610	1.0250	0.2844
1.0303	0.9938	0.9893	0.9650	0.5428
1.0325	0.9985	0.9910	0.9670	0.7563
1.0970	0.8835	1.0530	0.8050	0.3001
1.0988	0.8700	1.0543	0.7920	0.0027
0.9800	1.0615	0.9400	1.0830	0.0568
0.9835	1.0830	0.9430	1.1010	0.0678
0.9928	1.0668	0.9517	1.0750	0.4479
1.0280	0.9965	0.9863	0.9690	0.6335
1.0320	0.9965	0.9900	0.9660	0.5745
1.0365	0.9960	0.9940	0.9610	0.7127
1.0455	0.9728	1.0023	0.9300	0.5510
1.0940	0.8905	1.0490	0.8140	0.0563
1.1435	0.8155	1.0970	0.7130	0.0031
0.9548	1.1310	0.9150	1.1850	0.1029
0.9693	1.0975	0.9287	1.1320	0.0813
0.9863	1.0598	0.9447	1.0750	0.2137
0.9975	1.0245	0.9560	1.0270	0.7720
1.0155	1.0140	0.9730	0.9990	0.9331
1.0268	0.9975	0.9837	0.9720	0.6779
1.0340	0.9878	0.9910	0.9550	0.2494
1.0510	0.9520	1.0070	0.9060	0.3049
1.0528	1.0023	1.0083	0.9520	0.0121
1.0575	0.9425	1.0130	0.8910	0.4288
1.0670	0.9278	1.0217	0.8690	0.0200

0.9908	1.0625	0.9477	1.0720	0.0510
1.0018	1.0425	0.9590	1.0410	0.5448
1.0125	0.9913	0.9690	0.9790	0.7214
1.0258	0.9960	0.9813	0.9710	0.2624
1.0355	0.9918	0.9907	0.9580	0.6984
1.0658	0.9948	1.0203	0.9330	0.0440
0.9705	1.0895	0.9280	1.1230	0.1582
0.9750	1.0853	0.9320	1.1130	0.0785
1.0075	1.0310	0.9633	1.0230	0.7410
1.0168	1.0218	0.9720	1.0050	0.8693
1.0205	1.0275	0.9760	1.0070	0.8570
1.0488	0.9353	1.0027	0.8920	0.0546
1.1385	0.8185	1.0880	0.7190	0.2538
0.9860	1.0525	0.9420	1.0670	0.2848
1.0030	1.0290	0.9580	1.0260	0.5742
1.0045	1.0240	0.9590	1.0190	0.8456
1.0063	1.0203	0.9607	1.0140	0.7247
1.0443	0.9100	0.9970	0.8710	0.0352
1.0493	0.9633	1.0023	0.9180	0.1956
1.0660	0.8960	1.0180	0.8410	0.2349
1.1273	0.8200	1.0760	0.7270	0.0101
0.9700	1.0975	0.9257	1.1310	0.0432
0.9840	1.0768	0.9383	1.0940	0.1813
1.0118	1.0188	0.9653	1.0070	0.6589
1.0240	1.0098	0.9773	0.9860	0.7195
1.0263	1.0053	0.9793	0.9800	0.7603
1.0300	1.0028	0.9830	0.9740	0.1919
1.0650	0.9530	1.0160	0.8950	0.0418
1.0738	0.9180	1.0243	0.8550	0.0110
1.0820	0.9053	1.0327	0.8370	0.0618
1.0973	0.8740	1.0467	0.7970	0.0408
0.9440	1.1450	0.8993	1.2130	0.0037
0.9805	1.0735	0.9343	1.0950	0.3149

0.9943	1.0425	0.9473	1.0490	0.4167
0.9993	1.0090	0.9520	1.0100	0.8635
1.0030	1.0195	0.9557	1.0160	0.6567
1.0090	1.0143	0.9613	1.0050	0.9612
1.0198	0.9898	0.9717	0.9710	0.5966
1.0320	0.9830	0.9830	0.9530	0.5715
1.0338	0.9990	0.9847	0.9660	0.7252
1.0350	0.9915	0.9867	0.9580	0.3741
1.0650	0.9258	1.0150	0.8690	0.0415
1.0685	0.9300	1.0180	0.8700	0.0476
1.0813	0.9080	1.0303	0.8400	0.3407
1.1950	0.6958	1.1393	0.5820	0.1201
0.9833	1.0738	0.9363	1.0920	0.4673
1.0030	1.0670	0.9547	1.0640	0.0736
1.0105	1.0318	0.9617	1.0210	0.6270
1.0140	0.9983	0.9650	0.9840	0.7592
1.0228	1.0095	0.9737	0.9870	0.8849
1.0465	0.9608	0.9960	0.9180	0.1651
1.0475	0.9663	0.9973	0.9220	0.1369
1.0568	0.9345	1.0063	0.8840	0.0085
1.1030	0.8905	1.0497	0.8070	0.0065
0.9525	1.1333	0.9060	1.1900	0.0277
0.9790	1.0665	0.9310	1.0890	0.6627
0.9895	1.0485	0.9410	1.0600	0.4675
0.9965	1.0700	0.9477	1.0740	0.1160
1.0250	1.0200	0.9750	0.9950	0.9176
1.0285	1.0153	0.9783	0.9870	0.6772
1.0335	0.9935	0.9833	0.9610	0.1066
1.0390	0.9770	0.9880	0.9400	0.0943
1.0475	0.9620	0.9960	0.9180	0.5358
1.0600	0.9460	1.0080	0.8920	0.3605
1.0790	0.9115	1.0260	0.8450	0.2837
1.1045	0.8555	1.0505	0.7750	0.0138

0.9293	1.1830	0.8830	1.2730	0.0051
0.9703	1.1055	0.9217	1.1390	0.0513
1.0120	1.0340	0.9613	1.0220	0.8498
1.0130	1.0200	0.9620	1.0070	0.9848
1.0138	1.0160	0.9627	1.0020	0.9382
1.0253	1.0005	0.9743	0.9760	0.5655
1.0343	0.9825	0.9830	0.9500	0.0214
1.0473	0.9705	0.9950	0.9270	0.1300
1.0878	0.8733	1.0333	0.8030	0.0246
1.0948	0.8583	1.0403	0.7840	0.0220
1.1138	0.8490	1.0580	0.7620	0.0010
0.9465	1.1135	0.8983	1.1760	0.0583
0.9965	1.0820	0.9460	1.0860	0.1927
1.0165	1.0295	0.9643	1.0130	0.6938
1.0273	1.0068	0.9747	0.9800	0.6541
1.0585	0.9548	1.0043	0.9020	0.0277
1.1035	0.8595	1.0475	0.7790	0.0415
0.9560	1.1660	0.9060	1.2200	0.0299
0.9905	1.0785	0.9393	1.0890	0.4559
0.9948	1.0633	0.9427	1.0690	0.3109
1.0088	1.0470	0.9563	1.0380	0.0662
1.0200	1.0545	0.9670	1.0340	0.6543
1.0263	1.0388	0.9733	1.0120	0.7715
1.0263	0.9948	0.9733	0.9690	0.3787
1.0275	0.9910	0.9740	0.9640	0.3621
1.0355	0.9933	0.9813	0.9590	0.2900
1.0530	0.9555	0.9985	0.9070	0.6601
1.0870	0.9200	1.0303	0.8460	0.0089
0.9993	1.0563	0.9460	1.0570	0.1011
1.0030	1.0385	0.9503	1.0350	0.7096
1.0220	1.0030	0.9680	0.9810	0.4069
1.0263	1.0180	0.9717	0.9920	0.8796
1.0340	1.0025	0.9790	0.9700	0.7423

1.0355	0.9328	0.9803	0.9010	0.2181
1.0358	0.9638	0.9810	0.9300	0.2138
1.0385	0.9878	0.9830	0.9510	0.1925
1.1045	0.8760	1.0460	0.7930	0.0814
1.1578	0.7525	1.0963	0.6500	0.0068
0.9845	1.0658	0.9313	1.0830	0.2899
0.9908	1.0558	0.9370	1.0660	0.1145
0.9948	1.0788	0.9407	1.0840	0.1049
1.0188	1.0245	0.9633	1.0060	0.8015
1.0230	1.0300	0.9680	1.0070	0.7572
1.0255	0.9988	0.9703	0.9740	0.1808
1.0263	1.0085	0.9707	0.9830	0.8061
1.0345	1.0133	0.9787	0.9790	0.2222
1.0900	0.8905	1.0313	0.8170	0.2295
1.1243	0.8230	1.0637	0.7320	0.0475
0.9933	1.0508	0.9387	1.0580	0.2533
1.0085	1.0220	0.9530	1.0130	0.8747
1.0140	1.0203	0.9580	1.0060	0.8101
1.0228	1.0193	0.9667	0.9970	0.9148
1.0353	0.9753	0.9787	0.9420	0.3025
1.0390	0.9755	0.9820	0.9390	0.5314
1.0438	0.9843	0.9860	0.9430	0.3978
1.0465	0.9660	0.9890	0.9230	0.2385
1.0493	0.9825	0.9920	0.9360	0.5847
1.0535	0.9408	0.9953	0.8930	0.0075
1.1308	0.8345	1.0680	0.7380	0.0599
0.9840	1.0783	0.9287	1.0960	0.0795
1.0035	1.0485	0.9470	1.0450	0.6864
1.0108	1.0200	0.9537	1.0090	0.8254
1.0150	1.0395	0.9577	1.0240	0.2069
1.0165	1.0285	0.9597	1.0120	0.9653
1.0215	1.0308	0.9643	1.0090	0.7556
1.0345	0.9983	0.9770	0.9650	0.5811

1.0370	0.9300	0.9790	0.8970	0.5191
1.0690	0.9368	1.0093	0.8760	0.2200
0.9398	1.1615	0.8863	1.2360	0.0021
1.0028	1.0445	0.9453	1.0420	0.0710
1.0210	1.0213	0.9627	1.0000	0.9934
1.0218	1.0130	0.9633	0.9910	0.8692
1.0418	0.9763	0.9820	0.9370	0.1867
1.0650	0.9425	1.0040	0.8850	0.2105
1.0723	0.9333	1.0110	0.8700	0.0404
1.2130	0.6703	1.1440	0.5530	0.0147
0.9865	1.0718	0.9290	1.0860	0.1831
1.0150	1.0280	0.9563	1.0130	0.8453
1.0290	1.0035	0.9697	0.9750	0.4787
1.0375	0.9690	0.9770	0.9340	0.2735
1.0440	0.9728	0.9837	0.9320	0.0535
1.0860	0.9010	1.0230	0.8300	0.0081
1.0985	0.8890	1.0347	0.8090	0.0175
0.9935	1.0670	0.9350	1.0740	0.1000
1.0193	0.9970	0.9593	0.9780	0.6084
1.0245	1.0195	0.9643	0.9950	0.9404
1.0283	1.0090	0.9673	0.9810	0.8658
1.0300	0.9818	0.9693	0.9530	0.3784
1.0543	0.9485	0.9923	0.9000	0.0113
1.1670	0.7738	1.0980	0.6630	0.0090
0.9845	1.0015	0.9250	1.0170	0.7230
1.0010	1.0560	0.9410	1.0550	0.5859
1.0070	1.0545	0.9467	1.0470	0.6141
1.0108	1.0435	0.9500	1.0320	0.3518
1.0255	1.0153	0.9640	0.9900	0.7744
1.0295	1.0055	0.9680	0.9770	0.7234
1.0398	1.0103	0.9773	0.9720	0.6540
1.0408	0.9878	0.9783	0.9490	0.2856
1.0413	0.9950	0.9790	0.9560	0.5124

1.0505	0.9688	0.9877	0.9220	0.2991
1.0535	0.9740	0.9903	0.9250	0.3862
1.0720	0.9128	1.0080	0.8510	0.0853
1.1140	0.8363	1.0470	0.7510	0.1987
0.9330	1.1695	0.8760	1.2530	0.1190
0.9703	1.1165	0.9107	1.1510	0.2083
0.9910	1.0550	0.9310	1.0650	0.3549
1.0140	1.0320	0.9520	1.0180	0.8617
1.0160	1.0405	0.9543	1.0240	0.4557
1.0193	1.0245	0.9570	1.0050	0.8669
1.0250	1.0260	0.9620	1.0010	0.9644
1.0320	1.0010	0.9690	0.9700	0.4203
1.0338	1.0015	0.9707	0.9690	0.3154
1.0353	0.9900	0.9723	0.9560	0.2263
1.0380	1.0065	0.9745	0.9700	0.5074
1.1095	0.8715	1.0420	0.7850	0.4338
1.0028	1.0585	0.9403	1.0560	0.0210
1.0093	1.0355	0.9470	1.0260	0.8438
1.0298	1.0090	0.9657	0.9800	0.7196
1.0300	0.8510	0.9665	0.8260	0.1176
1.0503	0.9730	0.9853	0.9260	0.0470
1.0705	0.9420	1.0045	0.8800	0.0631
1.0718	0.9493	1.0057	0.8860	0.0331
1.0773	0.9295	1.0100	0.8630	0.0183
1.0940	0.9025	1.0260	0.8250	0.4348
1.1050	0.8560	1.0360	0.7750	0.0125
1.1085	0.8765	1.0400	0.7910	0.1367
1.1583	0.7913	1.0860	0.6830	0.0413
1.0160	1.0275	0.9520	1.0110	0.8382
1.0168	1.0353	0.9530	1.0180	0.7620
1.0208	1.0393	0.9567	1.0180	0.6003
1.0258	0.9878	0.9610	0.9630	0.3894
1.0273	1.0155	0.9630	0.9890	0.7774

1.0293	1.0083	0.9643	0.9800	0.5647
1.0425	1.0030	0.9770	0.9620	0.3349
1.0478	0.9570	0.9813	0.9130	0.0631
1.0595	0.9580	0.9930	0.9040	0.0435
1.0003	1.0630	0.9367	1.0630	0.2182
1.0090	1.0530	0.9440	1.0440	0.5838
1.0198	1.0280	0.9540	1.0080	0.8360
1.0240	1.0070	0.9580	0.9830	0.7972
1.0263	1.0100	0.9607	0.9840	0.7907
1.0295	1.0065	0.9640	0.9780	0.3224
1.0305	1.0063	0.9650	0.9760	0.8178
1.0340	1.0248	0.9683	0.9910	0.9325
1.0388	0.9770	0.9727	0.9410	0.2284
1.0410	0.9810	0.9740	0.9420	0.5385
1.0465	0.9888	0.9800	0.9450	0.1883
1.0718	0.9350	1.0033	0.8720	0.0840
1.0808	0.9143	1.0117	0.8460	0.0607
0.9720	1.0805	0.9090	1.1120	0.4421
0.9795	1.0895	0.9157	1.1120	0.3398
1.0015	1.0995	0.9360	1.0980	0.4821
1.0215	1.0123	0.9553	0.9910	0.7671
1.0425	1.0020	0.9750	0.9610	0.7632
1.0500	0.9840	0.9820	0.9370	0.2767
1.0540	0.9505	0.9860	0.9020	0.0243
1.0640	0.9515	0.9953	0.8940	0.1386
1.0935	0.8673	1.0227	0.7930	0.2222
1.1403	0.7595	1.0663	0.6660	0.0507
0.9968	1.0668	0.9313	1.0700	0.5330
1.0233	1.0233	0.9553	1.0000	0.9813
1.0235	1.0225	0.9560	0.9990	0.9773
1.0285	1.0150	0.9610	0.9870	0.7791
1.0325	1.0030	0.9643	0.9710	0.5715
1.0450	0.9880	0.9763	0.9450	0.4848

1.0520	0.9630	0.9830	0.9150	0.5749
1.0653	0.9393	0.9953	0.8820	0.0007
1.1425	0.8118	1.0670	0.7110	0.3650
1.1683	0.7583	1.0913	0.6490	0.0687
1.0023	1.0518	0.9350	1.0490	0.2608
1.0233	0.9923	0.9550	0.9700	0.4789
1.0295	1.0150	0.9603	0.9860	0.7088
1.0313	0.9995	0.9620	0.9690	0.7749
1.0320	0.9853	0.9627	0.9550	0.3301
1.0435	1.0015	0.9735	0.9600	0.5430
1.0655	0.9600	0.9940	0.9010	0.0695
0.9573	1.1485	0.8920	1.2000	0.2807
0.9960	1.0665	0.9280	1.0710	0.1197
1.0195	1.0243	0.9503	1.0050	0.8621
1.0275	1.0275	0.9573	1.0000	0.9478
1.0285	1.0155	0.9590	0.9870	0.7972
1.0290	1.0000	0.9590	0.9720	0.6914
1.0465	0.9980	0.9750	0.9540	0.1526
1.0495	0.9925	0.9780	0.9460	0.7404
1.0533	1.0040	0.9813	0.9530	0.2723
1.0890	0.9113	1.0153	0.8370	0.0096
1.0965	0.8960	1.0223	0.8170	0.1777
1.1628	0.7470	1.0837	0.6420	0.0093
1.2210	0.6335	1.1375	0.5190	0.1362
1.0020	1.0495	0.9327	1.0470	0.1785
1.0025	1.0410	0.9330	1.0380	0.5098
1.0125	1.0790	0.9425	1.0660	0.1717
1.0235	1.0028	0.9530	0.9800	0.3709
1.0253	1.0218	0.9547	0.9970	0.9191
1.0265	1.0285	0.9560	1.0020	0.9148
1.0373	1.0033	0.9660	0.9670	0.5816
1.0413	0.9830	0.9690	0.9440	0.3578
1.0570	0.9660	0.9840	0.9140	0.1506

1.0808	0.9255	1.0057	0.8560	0.0038
1.1728	0.7530	1.0923	0.6420	0.0041
0.9748	1.1085	0.9070	1.1370	0.0870
0.9978	1.0655	0.9277	1.0680	0.4734
1.0078	1.0475	0.9370	1.0390	0.1204
1.0168	1.0553	0.9457	1.0380	0.3839
1.0165	1.0183	0.9457	1.0020	0.9246
1.0188	1.0150	0.9473	0.9960	0.9545
1.0278	1.0218	0.9553	0.9940	0.9048
1.0410	1.0070	0.9683	0.9670	0.2371
1.0640	0.9455	0.9900	0.8890	0.3456
1.0848	0.9208	1.0093	0.8490	0.1300
1.1093	0.8778	1.0313	0.7910	0.0181
1.1233	0.8560	1.0447	0.7620	0.2097
0.9835	1.0695	0.9140	1.0870	0.4052
1.0088	1.0525	0.9373	1.0430	0.1515
1.0103	1.0515	0.9390	1.0410	0.2741
1.0178	1.0378	0.9450	1.0200	0.6600
1.0235	1.0140	0.9510	0.9910	0.9079
1.0385	1.0025	0.9643	0.9650	0.1763
1.0438	0.9948	0.9700	0.9530	0.8352
1.0520	0.9748	0.9770	0.9270	0.1815
1.0545	0.9760	0.9793	0.9260	0.2988
1.0878	0.8950	1.0107	0.8230	0.0189
0.9615	1.1010	0.8920	1.1450	0.1742
0.9650	1.0965	0.8960	1.1360	0.4749
1.0093	1.0638	0.9370	1.0540	0.3753
1.0198	1.0410	0.9460	1.0210	0.5441
1.0258	1.0255	0.9520	1.0000	0.9755
1.1363	0.8385	1.0543	0.7380	0.0109
1.1525	0.7968	1.0690	0.6910	0.0051
0.9823	1.0923	0.9107	1.1120	0.4723
1.0158	1.0435	0.9417	1.0270	0.7150

1.0325	1.0150	0.9573	0.9830	0.7734
1.0400	1.0008	0.9637	0.9620	0.1623
1.0545	0.9708	0.9773	0.9210	0.2854
1.0915	0.9085	1.0115	0.8320	0.1386
1.1348	0.8375	1.0523	0.7380	0.0490
1.0160	1.0235	0.9410	1.0070	0.8801
1.0185	0.9928	0.9427	0.9750	0.9777
1.0258	1.0070	0.9503	0.9820	0.3878
1.0273	1.0115	0.9513	0.9850	0.7464
1.0400	1.0190	0.9630	0.9800	0.7594
1.0493	1.0160	0.9713	0.9680	0.7915
1.0083	1.0535	0.9330	1.0450	0.3939
1.0115	1.0605	0.9353	1.0480	0.8187
1.0140	1.0290	0.9380	1.0150	0.8568
1.0283	1.0248	0.9507	0.9970	0.9592
1.0383	0.9933	0.9600	0.9570	0.1554
1.0613	0.9718	0.9813	0.9160	0.2874
1.0635	0.9690	0.9835	0.9110	0.1770
1.0863	0.9210	1.0047	0.8480	0.0753
1.0860	0.9190	1.0050	0.8460	0.5022
1.1990	0.7005	1.1097	0.5840	0.1234
1.0090	1.0335	0.9320	1.0240	0.6997
1.0130	1.0270	0.9360	1.0140	0.4504
1.0203	1.0373	0.9430	1.0170	0.7705
1.0238	0.9870	0.9457	0.9640	0.3851
1.0283	1.0225	0.9500	0.9940	0.8587
1.0390	1.0070	0.9597	0.9690	0.5077
1.0510	0.9730	0.9710	0.9260	0.0773
1.0545	0.9875	0.9745	0.9360	0.4668
1.0730	0.9653	0.9910	0.9000	0.0152
1.0753	0.9275	0.9940	0.8630	0.0344
1.0805	0.8380	0.9983	0.7760	0.0177
1.0890	0.9170	1.0065	0.8420	0.4174

0.9908	1.0843	0.9147	1.0940	0.1132
1.0030	1.0450	0.9260	1.0420	0.7376
1.0270	1.0345	0.9483	1.0070	0.9267
1.0323	1.0278	0.9523	0.9960	0.8811
1.0595	0.9653	0.9777	0.9110	0.0157
1.0615	0.9553	0.9800	0.9000	0.5460
1.0878	0.9195	1.0037	0.8450	0.0520
1.0980	0.9000	1.0140	0.8200	0.1675
1.1110	0.8645	1.0253	0.7780	0.0187
0.9948	1.0833	0.9167	1.0890	0.2670
1.0218	1.0598	0.9420	1.0370	0.2272
1.0280	1.0355	0.9477	1.0070	0.8720
1.0570	0.9763	0.9747	0.9240	0.2494
1.0730	0.9185	0.9893	0.8560	0.0993
1.0963	0.8540	1.0110	0.7790	0.1678
1.0025	1.0455	0.9230	1.0430	0.5545
1.0030	1.0675	0.9235	1.0640	0.1555
1.0095	1.0330	0.9300	1.0230	0.7155
1.0215	1.0423	0.9410	1.0200	0.4377
1.0340	1.0370	0.9520	1.0030	0.9467
1.0485	0.9950	0.9655	0.9490	0.4834
1.0715	0.9425	0.9873	0.8800	0.0517
1.0773	0.9320	0.9917	0.8650	0.1507
1.0783	0.9335	0.9927	0.8660	0.2308
0.9743	1.1193	0.8963	1.1490	0.0565
0.9968	1.0828	0.9170	1.0860	0.3001
1.0278	1.0068	0.9457	0.9800	0.4029
1.0318	1.0030	0.9493	0.9720	0.4978
1.0470	0.9960	0.9637	0.9510	0.2344
1.0595	0.9705	0.9743	0.9160	0.0351
1.0620	0.9295	0.9773	0.8750	0.0172
1.0645	0.9410	0.9793	0.8840	0.0746
1.0798	0.9358	0.9933	0.8670	0.0787

1.0210	0.9908	0.9387	0.9700	0.4447
1.0325	1.0175	0.9487	0.9850	0.8464
1.0353	1.0188	0.9510	0.9840	0.7623
1.0355	0.9925	0.9520	0.9580	0.8264
1.0388	1.0100	0.9550	0.9720	0.1901
1.0508	0.9890	0.9653	0.9410	0.3266
1.0628	0.9673	0.9763	0.9100	0.0460
1.0760	0.9500	0.9890	0.8830	0.3394
1.1180	0.8508	1.0280	0.7610	0.0388
1.0245	1.0285	0.9410	1.0040	0.9348
1.0278	1.0265	0.9430	0.9990	0.9937
1.0278	1.0290	0.9433	1.0010	0.9848
1.0280	1.0123	0.9440	0.9850	0.6144
1.0290	1.0510	0.9445	1.0210	0.8068
1.0373	0.9923	0.9527	0.9570	0.4000
1.0695	0.9655	0.9820	0.9030	0.0239
1.1865	0.7730	1.0890	0.6510	0.0064
0.9993	1.0685	0.9163	1.0690	0.4512
1.0213	1.0648	0.9360	1.0430	0.4759
1.0208	1.0415	0.9363	1.0200	0.3530
1.0380	1.0315	0.9515	0.9940	0.6856
1.0473	1.0090	0.9603	0.9630	0.1047
1.0615	0.9675	0.9733	0.9110	0.3852
1.0653	0.9540	0.9770	0.8960	0.3130
1.1265	0.8460	1.0330	0.7510	0.0777
0.9845	1.0720	0.9020	1.0890	0.4629
1.0065	1.0425	0.9220	1.0360	0.7007
1.0180	1.0085	0.9320	0.9910	0.6462
1.0650	0.9525	0.9760	0.8940	0.0899
1.0703	0.9660	0.9807	0.9030	0.1711
1.0980	0.8750	1.0053	0.7970	0.0995
1.0985	0.8105	1.0060	0.7380	0.1298
1.0120	1.0355	0.9260	1.0230	0.6636

1.0333	1.0020	0.9453	0.9700	0.7155
1.0593	0.9800	0.9693	0.9250	0.0267
1.0635	0.9805	0.9730	0.9220	0.4014
1.0848	0.9520	0.9923	0.8780	0.0041
1.0945	0.9140	1.0010	0.8350	0.0486
1.1065	0.9198	1.0130	0.8310	0.0536
0.9660	1.1010	0.8830	1.1400	0.2066
0.9770	1.1550	0.8930	1.1820	0.1950
1.0460	0.9870	0.9560	0.9440	0.8420
1.0523	1.0118	0.9617	0.9620	0.4124
1.1080	0.8875	1.0130	0.8010	0.2744
1.0285	1.0105	0.9390	0.9820	0.7268
1.0433	1.0243	0.9520	0.9820	0.6424
1.0478	1.0040	0.9563	0.9580	0.2779
1.0603	0.9513	0.9677	0.8970	0.0226
1.0635	0.9303	0.9707	0.8750	0.2184
1.0695	0.9798	0.9763	0.9160	0.0860
1.0700	0.9665	0.9765	0.9030	0.0336
1.0690	0.9705	0.9765	0.9080	0.4077
1.1050	0.9040	1.0083	0.8180	0.0469
1.1698	0.8053	1.0680	0.6880	0.0022
1.1928	0.8183	1.0887	0.6860	0.0088
1.0105	1.0235	0.9220	1.0130	0.7707
1.0333	1.0288	0.9423	0.9960	0.8620
1.0343	1.0278	0.9430	0.9940	0.8649
1.0415	1.0075	0.9497	0.9670	0.5820
1.0430	0.9973	0.9517	0.9560	0.1228
1.0480	1.0128	0.9560	0.9660	0.5250
1.0795	0.9530	0.9850	0.8830	0.1637
1.0820	0.9470	0.9870	0.8750	0.2057
1.1528	0.8045	1.0513	0.6980	0.1964
0.9700	1.1090	0.8833	1.1430	0.5693
1.0070	1.0680	0.9170	1.0610	0.1729

1.0105	1.0640	0.9207	1.0530	0.4906
1.0203	1.0448	0.9290	1.0240	0.6991
1.0350	1.0198	0.9433	0.9850	0.7751
1.0393	0.9848	0.9463	0.9480	0.3996
1.0525	1.0080	0.9585	0.9580	0.2498
1.0533	0.9910	0.9590	0.9410	0.0707
1.0538	0.9873	0.9603	0.9370	0.0481
1.0630	0.9885	0.9680	0.9300	0.1633
1.0698	0.9780	0.9747	0.9140	0.0458
1.0083	1.0685	0.9173	1.0600	0.4558
1.0310	1.0035	0.9380	0.9730	0.8301
1.0343	1.0248	0.9410	0.9910	0.6939
1.0798	0.9425	0.9827	0.8730	0.0097
1.0815	0.9318	0.9840	0.8620	0.0447
1.0915	0.9810	0.9935	0.8990	0.1460
0.9040	1.1870	0.8220	1.3130	0.1740
0.9665	1.1035	0.8790	1.1420	0.2961
1.0025	1.0515	0.9110	1.0490	0.4683
1.0035	1.0990	0.9120	1.0950	0.7858
1.0040	1.0465	0.9130	1.0420	0.5236
1.0180	1.0428	0.9250	1.0240	0.3756
1.0380	0.9960	0.9440	0.9600	0.7037
1.0483	1.0060	0.9527	0.9600	0.0910
1.0483	0.9775	0.9530	0.9330	0.0934
1.0560	0.9690	0.9600	0.9180	0.5508
1.0570	1.0615	0.9610	1.0040	0.9388
1.0870	0.9365	0.9885	0.8620	0.1336
1.0905	0.9268	0.9917	0.8500	0.1036
1.1050	0.8965	1.0040	0.8110	0.1496
0.9555	1.2065	0.8680	1.2630	0.1594
1.0085	1.0638	0.9157	1.0550	0.4965
1.0168	1.0673	0.9237	1.0500	0.3832
1.0235	1.0208	0.9290	0.9970	0.8889

1.0463	0.9953	0.9500	0.9510	0.6311
1.0500	1.0420	0.9530	0.9920	0.8986
1.1048	0.9015	1.0030	0.8160	0.0610
1.1865	0.7910	1.0775	0.6670	0.1466
0.9928	1.0975	0.9000	1.1060	0.4254
1.0020	1.0805	0.9083	1.0780	0.1931
1.0370	1.0000	0.9410	0.9640	0.4247
1.0403	1.0050	0.9440	0.9660	0.4532
1.0470	1.0008	0.9497	0.9560	0.3704
1.0515	1.0303	0.9540	0.9800	0.4881
1.0530	1.0200	0.9547	0.9690	0.4683
1.0650	0.9560	0.9660	0.8980	0.5230
1.0670	0.9485	0.9680	0.8890	0.0746
1.0868	0.9325	0.9860	0.8580	0.0026
1.1153	0.8955	1.0117	0.8030	0.0374
1.1755	0.7900	1.0660	0.6720	0.1650
1.0055	1.0403	0.9110	1.0350	0.7620
1.0263	1.0468	0.9297	1.0200	0.6546
1.0433	0.9883	0.9457	0.9470	0.0326
1.0618	0.9655	0.9617	0.9090	0.0463
1.0888	0.9058	0.9860	0.8320	0.0436
1.0975	0.9053	0.9943	0.8250	0.0153
1.1075	0.8915	1.0030	0.8050	0.2592
1.1130	0.8845	1.0080	0.7950	0.2207
1.1263	0.8660	1.0207	0.7690	0.0527
1.1798	0.7658	1.0683	0.6490	0.0369
1.0405	1.0358	0.9420	0.9950	0.9422
1.0453	1.0010	0.9457	0.9580	0.5023
1.0540	0.9888	0.9537	0.9380	0.2149
1.0673	0.9768	0.9657	0.9150	0.1409
1.0993	0.9348	0.9953	0.8500	0.3609
1.0093	1.0600	0.9127	1.0500	0.0486
1.0525	0.9793	0.9513	0.9300	0.6906

1.0563	0.9688	0.9550	0.9170	0.1773
1.0588	0.9820	0.9570	0.9280	0.1116
1.0703	0.9783	0.9670	0.9140	0.0495
1.0715	0.9690	0.9683	0.9040	0.0198
1.0945	0.9140	0.9890	0.8350	0.4150
1.1180	0.8880	1.0110	0.7940	0.0747
0.9940	1.0645	0.8980	1.0710	0.2699
1.0135	1.0473	0.9150	1.0330	0.8297
1.0135	1.0398	0.9157	1.0260	0.2685
1.0270	1.0745	0.9270	1.0460	0.7737
1.0315	1.0355	0.9310	1.0040	0.9789
1.0433	1.0258	0.9420	0.9830	0.2992
1.0638	0.9788	0.9607	0.9200	0.2389
0.9713	1.1510	0.8760	1.1850	0.4305
1.0140	1.0420	0.9150	1.0280	0.8368
1.0353	0.9985	0.9340	0.9650	0.5229
1.0405	1.0185	0.9383	0.9790	0.5144
1.0470	0.9885	0.9445	0.9440	0.2548
1.0478	1.0318	0.9447	0.9850	0.5084
1.0545	0.9933	0.9517	0.9420	0.4055
1.0850	0.9220	0.9783	0.8500	0.0435
1.1823	0.7833	1.0660	0.6630	0.0663
1.3430	0.5390	1.2110	0.4010	0.1696
1.0128	1.0620	0.9120	1.0490	0.3093
1.0353	1.0290	0.9327	0.9940	0.9223
1.0408	1.0075	0.9373	0.9680	0.4117
1.0490	1.0000	0.9453	0.9530	0.2320
1.0765	0.9405	0.9700	0.8740	0.0696
1.1355	0.8748	1.0233	0.7700	0.0070
1.0000	1.1240	0.9000	1.1240	0.5985
1.0190	1.0540	0.9170	1.0340	0.7190
1.0453	1.0135	0.9407	0.9700	0.5455
1.0575	0.9885	0.9520	0.9350	0.3945

1.0665	0.9730	0.9603	0.9120	0.0994
1.0803	0.9580	0.9723	0.8870	0.0508
1.1128	0.8410	1.0013	0.7560	0.1355
1.1473	0.8270	1.0323	0.7210	0.1674
1.0015	1.0815	0.9000	1.0800	0.5086
1.0115	1.0420	0.9090	1.0300	0.7523
1.0235	1.0595	0.9197	1.0350	0.4144
1.0350	1.0198	0.9303	0.9850	0.4339
1.0405	0.9953	0.9350	0.9570	0.3054
1.0495	0.9530	0.9430	0.9080	0.1699
1.0485	0.9855	0.9430	0.9400	0.4711
1.0515	0.9788	0.9457	0.9310	0.1407
1.1080	0.9015	0.9960	0.8140	0.0135
1.1155	0.8895	1.0030	0.7970	0.3267
1.1235	0.8733	1.0100	0.7770	0.0024
1.1775	0.7850	1.0590	0.6670	0.3611
1.0365	1.0270	0.9310	0.9910	0.8923
1.0550	0.9928	0.9473	0.9410	0.3373
1.1065	0.9120	0.9940	0.8240	0.2159
1.1108	0.8578	0.9973	0.7720	0.0647
1.0233	1.0570	0.9177	1.0330	0.5582
1.0313	1.0388	0.9247	1.0070	0.8762
1.0578	0.9858	0.9490	0.9320	0.3981
1.0805	0.9510	0.9690	0.8800	0.0109
1.0925	0.9390	0.9805	0.8590	0.1861
1.0323	1.0313	0.9247	0.9990	0.9994
1.0425	0.9985	0.9340	0.9580	0.2244
1.0588	0.9918	0.9490	0.9370	0.0289
1.0870	0.9405	0.9740	0.8650	0.5871
1.0925	0.9483	0.9790	0.8680	0.0138
1.0980	0.9030	0.9840	0.8220	0.4251
1.1395	0.8785	1.0213	0.7710	0.0039
1.0015	1.0973	0.8967	1.0960	0.7578

1.0325	1.0303	0.9243	0.9980	0.9279
1.0458	1.0175	0.9360	0.9730	0.7879
1.0550	1.0205	0.9440	0.9670	0.0580
1.0553	1.0250	0.9443	0.9710	0.4683
1.0585	0.9805	0.9470	0.9260	0.6007
1.0605	0.9995	0.9493	0.9420	0.1849
1.0693	0.9725	0.9573	0.9100	0.2711
1.0715	0.9545	0.9590	0.8910	0.4409
1.0835	0.9380	0.9700	0.8660	0.5976
1.0855	0.9605	0.9710	0.8850	0.3445
1.0873	0.9435	0.9730	0.8680	0.0166
1.1210	0.8795	1.0030	0.7850	0.2680
1.1395	0.8580	1.0200	0.7530	0.0463
1.1425	0.8405	1.0223	0.7360	0.0113
0.9955	1.1395	0.8895	1.1450	0.1845
1.0213	1.0515	0.9130	1.0300	0.6227
1.0345	1.0413	0.9243	1.0070	0.8807
1.0360	1.0095	0.9260	0.9740	0.2799
1.0438	1.0453	0.9327	1.0010	0.9877
1.0493	0.9893	0.9383	0.9430	0.2895
1.0558	0.9700	0.9433	0.9190	0.0167
1.0840	0.9630	0.9690	0.8880	0.0142
1.0983	0.9290	0.9813	0.8460	0.0142
1.0188	1.0640	0.9093	1.0440	0.5682
1.0385	0.9970	0.9270	0.9600	0.3638
1.0435	1.0115	0.9320	0.9690	0.0572
1.0453	1.0160	0.9337	0.9720	0.4781
1.0600	1.0125	0.9470	0.9550	0.1930
1.0600	0.9700	0.9470	0.9150	0.7040
1.0700	0.9703	0.9553	0.9070	0.1249
1.0868	0.9453	0.9707	0.8700	0.0239
1.0950	0.9365	0.9780	0.8550	0.4246
1.1273	0.8755	1.0067	0.7770	0.1102

1.1675	0.7980	1.0420	0.6840	0.1083
1.1688	0.8163	1.0437	0.6980	0.2910
1.1915	0.7470	1.0645	0.6270	0.0676
1.2115	0.7035	1.0820	0.5810	0.2015
0.9080	1.2050	0.8100	1.3270	0.4179
0.9865	1.1535	0.8800	1.1690	0.0353
1.0280	1.0505	0.9173	1.0220	0.3739
1.0335	1.0110	0.9220	0.9780	0.7788
1.0413	1.0238	0.9287	0.9830	0.8132
1.0483	1.0108	0.9350	0.9640	0.4680
1.0500	1.0315	0.9365	0.9820	0.6857
1.0535	1.0230	0.9395	0.9710	0.0433
1.0550	0.9815	0.9410	0.9300	0.2123
1.0618	0.9958	0.9467	0.9380	0.2133
1.0625	1.0035	0.9480	0.9440	0.1769
1.0730	0.9550	0.9570	0.8900	0.1711
1.0850	0.9355	0.9680	0.8620	0.2038
1.1285	0.8725	1.0065	0.7730	0.0111
0.9810	1.0940	0.8740	1.1150	0.2835
1.0295	1.0780	0.9175	1.0470	0.5723
1.0313	1.0450	0.9183	1.0130	0.6316
1.0413	1.0268	0.9273	0.9860	0.6137
1.0443	1.0040	0.9307	0.9610	0.1984
1.0440	1.0225	0.9307	0.9790	0.6922
1.0530	1.0200	0.9385	0.9690	0.8340
1.0540	1.0240	0.9395	0.9720	0.8270
1.0565	0.9960	0.9413	0.9430	0.0491
1.0715	0.9585	0.9547	0.8950	0.2007
1.0723	0.9743	0.9553	0.9090	0.2271
1.1410	0.8560	1.0167	0.7500	0.0005
1.2050	0.7605	1.0740	0.6310	0.0367
1.0165	1.0665	0.9047	1.0490	0.3999
1.0475	0.9998	0.9323	0.9540	0.1548

1.0853	0.9520	0.9660	0.8770	0.0104
1.1033	0.8673	0.9823	0.7860	0.0424
1.0273	1.0493	0.9130	1.0210	0.4083
1.0380	1.0273	0.9227	0.9900	0.5781
1.0378	1.0303	0.9227	0.9930	0.9251
1.0413	1.0348	0.9260	0.9940	0.7981
1.0620	1.0035	0.9437	0.9450	0.1027
1.0650	1.0100	0.9465	0.9480	0.4567
1.0655	0.9710	0.9470	0.9110	0.5200
0.9963	1.0993	0.8850	1.1030	0.0301
1.0078	1.0873	0.8953	1.0790	0.3461
1.0410	1.0455	0.9247	1.0040	0.8928
1.0443	1.0145	0.9273	0.9720	0.5202
1.0495	1.0068	0.9320	0.9590	0.4706
1.0880	0.9280	0.9660	0.8530	0.5171
1.0940	0.9360	0.9710	0.8560	0.0434
1.1030	0.9085	0.9790	0.8240	0.5011
0.9725	1.1325	0.8630	1.1650	0.1525
0.9808	1.1378	0.8700	1.1600	0.5272
0.9883	1.0948	0.8763	1.1080	0.2491
1.0510	1.0340	0.9320	0.9840	0.7942
1.0585	0.9600	0.9387	0.9070	0.1123
1.0740	0.9330	0.9523	0.8690	0.0096
1.0910	0.9510	0.9675	0.8720	0.2612
1.0938	0.9290	0.9707	0.8490	0.1185
1.0298	1.0588	0.9123	1.0280	0.9736
1.0305	1.0195	0.9130	0.9890	0.8624
1.0310	1.0345	0.9130	1.0030	0.9550
1.0385	1.0363	0.9197	0.9980	0.9533
1.0608	0.9903	0.9393	0.9340	0.0401
1.0663	0.9860	0.9443	0.9250	0.2815
1.0690	0.9995	0.9475	0.9350	0.4432
1.0288	1.0400	0.9103	1.0110	0.7737

1.0353	1.0165	0.9163	0.9820	0.4733
1.0545	1.0050	0.9337	0.9530	0.3693
1.0715	0.9620	0.9480	0.8980	0.5193
0.9000	1.2718	0.7957	1.4130	0.0012
0.9145	1.2443	0.8087	1.3610	0.0503
1.0003	1.0850	0.8847	1.0850	0.5649
1.0145	1.0775	0.8970	1.0620	0.3138
1.0180	1.0850	0.9000	1.0660	0.1282
1.0315	1.0510	0.9120	1.0190	0.8025
1.0395	1.0143	0.9193	0.9760	0.6481
1.0423	1.0313	0.9213	0.9890	0.8077
1.0575	0.9815	0.9350	0.9280	0.2283
1.0638	0.9910	0.9403	0.9320	0.1923
1.0730	0.9955	0.9485	0.9280	0.4557
1.0740	0.9838	0.9493	0.9160	0.0510
0.9483	1.1903	0.8370	1.2550	0.0525
1.0290	1.0483	0.9090	1.0190	0.5577
1.0343	1.0300	0.9137	0.9960	0.8930
1.0398	1.0495	0.9177	1.0090	0.5326
1.0470	0.9970	0.9240	0.9520	0.4758
1.0518	1.0073	0.9287	0.9580	0.1759
1.0818	0.9608	0.9557	0.8880	0.0482
1.0858	0.9370	0.9583	0.8630	0.0008
1.0920	0.9585	0.9640	0.8780	0.0435
1.1030	0.9170	0.9740	0.8310	0.1950
1.1270	0.8838	0.9950	0.7840	0.0047
1.1753	0.7908	1.0377	0.6730	0.0624
1.0253	1.0228	0.9047	0.9980	0.9504
1.0280	1.0435	0.9070	1.0150	0.9142
1.0510	1.0080	0.9273	0.9590	0.4125
1.1890	0.7865	1.0490	0.6610	0.0727
0.9995	1.1078	0.8810	1.1080	0.0202
1.0153	1.0630	0.8943	1.0470	0.5290

1.0328	1.0235	0.9097	0.9910	0.9214
1.0335	1.0485	0.9110	1.0150	0.5775
1.0505	1.0108	0.9250	0.9620	0.4042
1.0513	1.0150	0.9267	0.9660	0.0699
1.0535	1.0125	0.9277	0.9610	0.5118
1.0625	1.0170	0.9365	0.9570	0.5934
1.0878	0.9448	0.9583	0.8690	0.3123
1.1040	0.9345	0.9730	0.8460	0.0505
1.0055	1.1055	0.8853	1.0990	0.7199
1.0215	1.0585	0.8987	1.0360	0.4309
1.0413	1.0205	0.9167	0.9800	0.6599
1.0465	1.0248	0.9210	0.9790	0.2627
1.0603	0.9998	0.9333	0.9430	0.0043
1.0613	0.9980	0.9340	0.9400	0.3679
1.0720	1.0175	0.9430	0.9490	0.4794
1.1245	0.8980	0.9900	0.7990	0.2745
1.0073	1.0705	0.8853	1.0630	0.3006
1.0083	1.0943	0.8867	1.0850	0.2363
1.0203	1.0748	0.8970	1.0530	0.2572
1.0408	1.0363	0.9153	0.9960	0.9144
1.0543	0.9900	0.9270	0.9390	0.3047
1.0550	1.0290	0.9277	0.9750	0.5092
1.0768	0.9735	0.9463	0.9040	0.0010
1.0810	0.9855	0.9500	0.9120	0.1049
1.2430	0.7400	1.0930	0.5950	0.1583
1.0185	1.0605	0.8940	1.0410	0.4979
1.0195	1.0345	0.8950	1.0150	0.7426
1.0350	1.0205	0.9090	0.9860	0.8225
1.0555	1.0098	0.9263	0.9570	0.2407
1.0630	0.9850	0.9333	0.9270	0.2013
1.0643	0.9943	0.9340	0.9340	0.2118
1.0680	0.9888	0.9377	0.9260	0.2966
1.0685	0.9888	0.9387	0.9250	0.0449

1.0765	0.9525	0.9450	0.8850	0.6083
1.0800	0.9543	0.9487	0.8840	0.0617
1.0845	0.9623	0.9527	0.8870	0.1908
1.0915	0.9458	0.9583	0.8660	0.0818
1.1083	0.8813	0.9727	0.7950	0.0009
1.1190	0.8865	0.9830	0.7920	0.3446
1.1658	0.7880	1.0230	0.6760	0.0036
1.0133	1.0840	0.8887	1.0700	0.1176
1.0193	1.0760	0.8937	1.0560	0.2034
1.0213	1.0643	0.8953	1.0420	0.3018
1.0565	0.9855	0.9270	0.9330	0.2402
1.0735	0.9723	0.9417	0.9060	0.0923
1.0755	0.9965	0.9435	0.9270	0.1794
1.0875	0.9668	0.9540	0.8890	0.3802
1.0938	0.9260	0.9597	0.8470	0.0198
1.1130	0.9180	0.9765	0.8250	0.4950
1.0093	1.0740	0.8843	1.0640	0.5606
1.0315	1.0235	0.9040	0.9920	0.9100
1.0358	1.0388	0.9077	1.0030	0.8581
1.0503	1.0363	0.9203	0.9870	0.7822
1.0553	0.9993	0.9240	0.9470	0.1506
1.0685	0.9910	0.9360	0.9270	0.5488
1.1045	0.9395	0.9670	0.8510	0.0856
1.1070	0.9260	0.9700	0.8360	0.0321
0.9478	1.1658	0.8290	1.2300	0.1780
1.0080	1.1350	0.8820	1.1260	0.1253
1.0365	1.0643	0.9067	1.0270	0.6469
1.0440	1.0578	0.9137	1.0130	0.9119
1.0518	1.0395	0.9203	0.9880	0.8401
1.0533	1.0340	0.9220	0.9820	0.6937
1.0955	0.9605	0.9585	0.8770	0.4068
1.0990	0.9560	0.9620	0.8700	0.4987
1.1120	0.9025	0.9730	0.8120	0.2073

1.0145	1.0330	0.8870	1.0180	0.7502
1.0293	1.0710	0.9000	1.0410	0.3365
1.0393	1.0243	0.9080	0.9860	0.6229
1.0465	1.0333	0.9143	0.9870	0.8169
1.0505	1.0530	0.9180	1.0020	0.9599
1.0518	1.0190	0.9197	0.9690	0.5463
1.0633	1.0015	0.9297	0.9420	0.3077
1.0825	0.9815	0.9465	0.9070	0.5100
1.0303	1.0613	0.8993	1.0300	0.4630
1.0395	1.0155	0.9070	0.9770	0.7280
1.0415	1.0368	0.9097	0.9950	0.8749
1.0425	1.0460	0.9100	1.0030	0.9047
1.0615	1.0068	0.9270	0.9480	0.2912
1.0688	1.0035	0.9333	0.9390	0.1329
1.0735	0.9743	0.9367	0.9080	0.0661
1.0790	0.9800	0.9417	0.9080	0.1136
1.0083	1.1088	0.8797	1.1000	0.1727
1.0185	1.0828	0.8880	1.0630	0.4861
1.0300	1.0270	0.8980	0.9970	0.9854
1.0365	1.0483	0.9040	1.0110	0.8167
1.0445	1.0140	0.9110	0.9710	0.8984
1.0450	1.0353	0.9113	0.9910	0.8664
1.0490	1.0165	0.9147	0.9690	0.4180
1.0525	1.0028	0.9173	0.9530	0.2070
1.0523	1.0235	0.9177	0.9730	0.1786
1.1055	0.9140	0.9640	0.8270	0.2382
1.1370	0.8790	0.9915	0.7730	0.0070
1.1403	0.8968	0.9947	0.7860	0.0501
1.0355	1.0150	0.9020	0.9800	0.8615
1.0400	1.0398	0.9053	1.0000	0.9838
1.0495	1.0095	0.9140	0.9620	0.4144
1.0595	1.0110	0.9230	0.9540	0.5262
1.0655	0.9988	0.9280	0.9370	0.2760

1.0980	0.9595	0.9560	0.8740	0.3458
1.1080	0.9250	0.9650	0.8350	0.5641
1.2370	0.6980	1.0777	0.5640	0.0074
1.0305	1.0488	0.8967	1.0180	0.7495
1.0325	1.0255	0.8980	0.9930	0.7599
1.0493	1.0243	0.9133	0.9760	0.7555
1.0618	1.0058	0.9240	0.9470	0.1126
1.0758	0.9660	0.9363	0.8980	0.0506
1.0780	0.9940	0.9375	0.9220	0.1565
1.0815	0.9810	0.9410	0.9070	0.7207
1.0960	0.9330	0.9540	0.8510	0.0957
1.1230	0.9010	0.9773	0.8020	0.0964
0.9880	1.0925	0.8590	1.1060	0.5059
1.0248	1.0663	0.8907	1.0400	0.1891
1.0260	1.0755	0.8913	1.0480	0.3839
1.0365	1.0195	0.9010	0.9840	0.7905
1.0530	1.0495	0.9155	0.9970	0.9698
1.0568	1.0165	0.9187	0.9620	0.2800
1.0860	0.9463	0.9437	0.8710	0.0120
1.0998	0.9365	0.9553	0.8520	0.0385
1.1018	0.9378	0.9570	0.8510	0.1568
1.1580	0.8560	1.0067	0.7390	0.0014
1.0383	1.0295	0.9010	0.9920	0.8410
1.0400	1.0395	0.9027	1.0000	0.9991
1.0413	1.0333	0.9040	0.9920	0.8414
1.0500	1.0585	0.9115	1.0080	0.9137
1.0513	1.0313	0.9127	0.9810	0.6490
1.0668	0.9890	0.9260	0.9270	0.1312
1.0835	0.9915	0.9400	0.9150	0.4294
1.0863	0.9650	0.9430	0.8880	0.1327
1.1060	0.9490	0.9605	0.8580	0.3875
1.0348	1.0553	0.8970	1.0200	0.8700
1.0365	1.0338	0.8987	0.9970	0.9080

1.0518	1.0148	0.9117	0.9650	0.4226
1.0555	1.0003	0.9147	0.9480	0.3813
1.0575	1.0430	0.9165	0.9860	0.8716
1.0630	0.9835	0.9220	0.9250	0.0657
1.0995	0.9265	0.9530	0.8430	0.1264
1.1023	0.9613	0.9560	0.8720	0.0459
1.1230	0.9058	0.9740	0.8070	0.0017
1.1370	0.8955	0.9853	0.7880	0.0030
0.9795	1.2050	0.8480	1.2300	0.2742
1.0338	1.0365	0.8953	1.0030	0.9307
1.0405	1.0500	0.9007	1.0090	0.8793
1.0463	1.0370	0.9057	0.9910	0.8326
1.0520	1.0465	0.9113	0.9950	0.9153
1.0533	1.0263	0.9117	0.9740	0.5853
1.0553	0.9933	0.9137	0.9410	0.2930
1.0690	1.0048	0.9253	0.9400	0.0459
1.0790	0.9985	0.9340	0.9250	0.4541
1.1070	0.9295	0.9590	0.8400	0.1506
1.1575	0.8545	1.0020	0.7380	0.0983
1.0135	1.0835	0.8767	1.0690	0.1499
1.0220	1.0420	0.8840	1.0200	0.7360
1.0293	1.0675	0.8903	1.0370	0.3195
1.0338	1.0510	0.8943	1.0170	0.7068
1.0350	1.0528	0.8957	1.0170	0.6146
1.0353	1.0495	0.8957	1.0140	0.8103
1.0403	1.0543	0.9003	1.0130	0.9564
1.0868	0.9668	0.9403	0.8900	0.0383
1.1430	0.8630	0.9890	0.7550	0.1539
1.1835	0.7970	1.0240	0.6730	0.0809
1.0120	1.0580	0.8740	1.0450	0.6043
1.0328	1.0485	0.8923	1.0150	0.7592
1.0460	1.0388	0.9040	0.9930	0.8787
1.0593	1.0123	0.9153	0.9560	0.1275

1.0635	1.0018	0.9187	0.9420	0.3339
1.0638	1.0098	0.9193	0.9490	0.0075
1.0653	0.9985	0.9203	0.9370	0.1538
1.0693	1.0013	0.9233	0.9360	0.1670
1.0728	1.0000	0.9263	0.9320	0.5646
1.0723	0.9943	0.9267	0.9270	0.2602
1.0965	0.9175	0.9477	0.8370	0.0568
1.1025	0.9280	0.9530	0.8420	0.0322
1.1075	0.9270	0.9570	0.8370	0.1349
1.1225	0.9295	0.9695	0.8280	0.2344
1.1228	0.8840	0.9703	0.7870	0.0037
1.1400	0.8540	0.9853	0.7490	0.0177
1.2263	0.7048	1.0597	0.5750	0.0204
1.0128	1.0238	0.8740	1.0110	0.8049
1.0140	1.0848	0.8747	1.0700	0.4223
1.0330	1.0230	0.8910	0.9900	0.9929
1.0433	1.0968	0.9007	1.0510	0.7687
1.0470	1.0680	0.9040	1.0200	0.5167
1.0760	1.0025	0.9290	0.9320	0.0125
1.0790	1.0075	0.9315	0.9340	0.5146
1.1103	0.9368	0.9580	0.8440	0.0309
1.1300	0.9463	0.9747	0.8370	0.0085
1.1758	0.7983	1.0150	0.6790	0.0033
1.0510	1.0010	0.9060	0.9520	0.3394
1.0553	1.0100	0.9097	0.9570	0.3201
1.0635	0.9968	0.9163	0.9370	0.1731
1.0840	0.9875	0.9347	0.9110	0.0267
1.1535	0.8530	0.9940	0.7390	0.0767
1.0165	1.0505	0.8750	1.0330	0.6821
1.0418	1.0278	0.8973	0.9870	0.7851
1.0480	1.0080	0.9020	0.9620	0.3839
1.0688	0.9803	0.9203	0.9170	0.2695
1.0738	0.9945	0.9240	0.9260	0.1929

1.0848	0.9743	0.9343	0.8980	0.0821
1.1050	0.9555	0.9515	0.8650	0.1932
1.1135	0.9003	0.9587	0.8080	0.0109
0.9610	1.1335	0.8260	1.1800	0.1533
0.9855	1.0980	0.8480	1.1140	0.1733
1.0100	1.1080	0.8687	1.0970	0.5813
1.0670	1.0055	0.9180	0.9420	0.1391
1.0705	0.9735	0.9210	0.9090	0.2309
1.0838	0.9165	0.9323	0.8460	0.2283
1.1500	0.8643	0.9890	0.7520	0.0007
1.0190	1.0560	0.8753	1.0360	0.3085
1.0350	1.0678	0.8887	1.0320	0.7080
1.0420	1.0835	0.8950	1.0400	0.7649
1.0495	1.0280	0.9020	0.9800	0.6748
1.0735	0.9948	0.9227	0.9270	0.1029
1.0760	0.9790	0.9240	0.9100	0.3747
1.0770	0.9705	0.9257	0.9010	0.3330
1.0875	0.9828	0.9340	0.9040	0.0069
1.0960	0.9670	0.9420	0.8820	0.0277
1.1335	0.9035	0.9740	0.7970	0.0644
1.1478	0.8670	0.9857	0.7550	0.0729
0.9990	1.0720	0.8570	1.0730	0.8195
1.0400	1.0323	0.8920	0.9930	0.8939
1.0498	1.0323	0.9007	0.9830	0.6686
1.0595	0.9940	0.9090	0.9380	0.3863
1.0705	0.9750	0.9183	0.9110	0.0232
1.0833	0.9785	0.9293	0.9030	0.1250
1.0845	0.9535	0.9310	0.8790	0.2398
1.0923	0.9390	0.9377	0.8600	0.0653
1.0945	0.9605	0.9393	0.8780	0.0142
1.0985	0.9720	0.9430	0.8850	0.0359
1.2333	0.7163	1.0587	0.5810	0.0027
1.0245	1.0240	0.8780	1.0000	0.9982

1.0315	1.0450	0.8837	1.0130	0.6734
1.0395	1.0555	0.8910	1.0150	0.4727
1.0478	1.0410	0.8977	0.9940	0.8923
1.0555	1.0443	0.9047	0.9890	0.7600
1.0665	1.0390	0.9140	0.9740	0.5794
1.0695	1.0115	0.9170	0.9460	0.3832
1.0760	0.9688	0.9220	0.9000	0.0923
1.0765	0.9895	0.9230	0.9190	0.1915
1.0803	0.9868	0.9260	0.9130	0.1625
1.0848	0.9760	0.9293	0.9000	0.0530
1.0885	0.9663	0.9333	0.8880	0.3895
1.1020	0.9573	0.9440	0.8690	0.4055
1.1215	0.9245	0.9615	0.8240	0.0288
1.0080	1.1610	0.8625	1.1520	0.6280
1.0355	1.0795	0.8867	1.0420	0.5595
1.0393	1.0495	0.8900	1.0100	0.7736
1.0420	1.0698	0.8920	1.0270	0.7196
1.0455	1.0140	0.8950	0.9700	0.3015
1.0730	0.9958	0.9183	0.9280	0.2962
1.0805	1.0065	0.9247	0.9320	0.2079
1.1758	0.8203	1.0067	0.6980	0.0293
0.9960	1.0955	0.8520	1.1000	0.8521
1.0035	1.1318	0.8577	1.1280	0.1773
1.0165	1.0693	0.8690	1.0520	0.4761
1.0310	1.0355	0.8810	1.0040	0.8793
1.0660	0.9848	0.9113	0.9240	0.1812
1.0745	0.9923	0.9190	0.9230	0.5554
1.0893	0.9895	0.9317	0.9080	0.0100
1.1275	0.9068	0.9637	0.8040	0.1039
1.1305	0.9245	0.9663	0.8180	0.0238
1.1338	0.9035	0.9697	0.7970	0.0149
1.1445	0.8680	0.9790	0.7580	0.0466
0.9835	1.1075	0.8400	1.1260	0.1738

1.0355	1.0628	0.8840	1.0260	0.6926
1.0543	1.0270	0.9000	0.9740	0.5401
1.0758	0.9935	0.9183	0.9240	0.0129
1.0785	1.0210	0.9215	0.9470	0.5314
1.0950	0.9753	0.9357	0.8910	0.1117
1.0983	0.9540	0.9377	0.8690	0.0428
1.1283	0.8388	0.9637	0.7430	0.0393
1.1343	0.8830	0.9690	0.7780	0.0143
1.0175	1.0255	0.8680	1.0080	0.8934
1.0213	1.0955	0.8713	1.0730	0.4534
1.0403	1.0618	0.8873	1.0210	0.6571
1.0533	1.0258	0.8987	0.9740	0.2961
1.0593	1.0260	0.9033	0.9690	0.4754
1.0603	1.0235	0.9047	0.9650	0.4595
1.0753	0.9875	0.9170	0.9180	0.2029
1.0753	0.9965	0.9170	0.9270	0.2944
1.1060	0.9635	0.9430	0.8710	0.0978
1.1898	0.7713	1.0153	0.6480	0.0069
0.9550	1.1390	0.8140	1.1930	
1.0000	1.1173	0.8517	1.1170	0.0662
1.0205	1.0555	0.8690	1.0340	0.7323
1.0370	1.0550	0.8833	1.0170	0.7700
1.0470	1.0320	0.8917	0.9860	0.7763
1.0690	1.0223	0.9110	0.9560	0.2284
1.0775	0.9925	0.9183	0.9210	0.0467
0.9858	1.1350	0.8393	1.1510	0.1248
0.9980	1.1415	0.8493	1.1440	0.3134
1.0590	0.9968	0.9013	0.9410	0.3677
1.0673	0.9923	0.9080	0.9300	0.0736
1.0685	0.9915	0.9090	0.9280	0.0910
1.0725	0.9793	0.9123	0.9130	0.0091
1.1003	0.9818	0.9363	0.8920	0.1119
1.1105	0.9990	0.9450	0.9000	0.3846

1.1178	0.9128	0.9507	0.8170	0.1456
1.0440	1.0515	0.8877	1.0070	0.8937
1.0715	1.0030	0.9103	0.9360	0.1008
1.0755	1.0025	0.9147	0.9320	0.4718
1.0980	0.9550	0.9335	0.8700	0.8931
1.1010	0.9448	0.9363	0.8580	0.0352
1.1110	0.9595	0.9440	0.8640	0.2365
1.1345	0.8860	0.9640	0.7810	0.0584
1.1380	0.8765	0.9670	0.7700	0.2637
1.0390	1.0525	0.8823	1.0130	0.6873
1.0455	1.0505	0.8877	1.0050	0.9778
1.0480	1.0435	0.8897	0.9960	0.9794
1.0535	1.0160	0.8940	0.9640	0.6852
1.0585	1.0075	0.8983	0.9520	0.6087
1.0665	0.9863	0.9053	0.9250	0.5777
1.0675	0.9965	0.9060	0.9330	0.3615
1.0850	1.0063	0.9207	0.9270	0.0696
1.0390	1.0683	0.8810	1.0280	0.6984
1.0575	1.0273	0.8967	0.9710	0.4414
1.0615	1.0275	0.9000	0.9680	0.5268
1.0708	1.0100	0.9077	0.9430	0.4606
1.0710	0.9760	0.9080	0.9110	0.5489
1.0845	1.0043	0.9197	0.9260	0.0942
1.0890	0.9770	0.9237	0.8970	0.0418
1.0978	0.9915	0.9307	0.9030	0.1007
1.1045	0.9870	0.9365	0.8940	0.1101
1.1160	0.8490	0.9460	0.7610	0.1288
0.8590	1.3625	0.7277	1.5860	0.1967
1.0508	1.0450	0.8900	0.9950	0.9113
1.0528	1.0445	0.8920	0.9920	0.8315
1.1133	0.9115	0.9430	0.8190	0.0012
1.1195	0.9238	0.9477	0.8250	0.0612
1.1200	0.9008	0.9483	0.8040	0.2441

0.9905	1.1040	0.8380	1.1150	0.3510
1.0455	1.0473	0.8840	1.0020	0.9832
1.0540	1.0363	0.8920	0.9830	0.7697
1.0610	1.0235	0.8980	0.9650	0.1888
1.0625	1.0140	0.8987	0.9540	0.3997
1.0633	1.0535	0.8993	0.9910	0.7753
1.0895	0.9880	0.9217	0.9070	0.0967
1.0928	0.9720	0.9250	0.8890	0.1965
1.0960	0.8605	0.9270	0.7850	0.0463
1.1130	0.9418	0.9413	0.8460	0.0012
1.1303	0.8645	0.9557	0.7650	0.0213
0.9895	1.0980	0.8360	1.1100	0.3373
1.0308	1.0213	0.8710	0.9910	0.9921
1.0325	1.0385	0.8720	1.0060	0.8991
1.0340	1.0753	0.8733	1.0400	0.3120
1.0545	1.0318	0.8913	0.9780	0.7142
1.0565	1.0035	0.8930	0.9500	0.0595
1.0645	1.0338	0.8997	0.9710	0.7745
1.0678	1.0183	0.9027	0.9540	0.5634
1.0690	0.9855	0.9030	0.9220	0.4829
1.0705	0.9770	0.9050	0.9130	0.1527
1.0803	0.9948	0.9133	0.9210	0.1946
1.0923	0.9733	0.9233	0.8910	0.0904
1.1185	0.8833	0.9457	0.7900	0.3510
1.0023	1.1260	0.8460	1.1230	0.1618
1.0640	1.0380	0.8977	0.9760	0.5636
1.0885	1.0060	0.9185	0.9240	0.2190
1.0928	0.9540	0.9227	0.8730	0.0101
1.0973	0.9623	0.9263	0.8770	0.1103
1.1118	0.9680	0.9383	0.8710	0.1452
0.9985	1.1318	0.8417	1.1330	0.1762
1.0553	1.0015	0.8897	0.9490	0.4170
1.0593	1.0340	0.8930	0.9760	0.4974

1.0715	1.0115	0.9030	0.9440	0.5371
1.0715	0.9953	0.9033	0.9290	0.1885
1.0718	1.0198	0.9040	0.9510	0.1879
1.0723	1.0098	0.9040	0.9420	0.6114
1.0725	1.0128	0.9043	0.9440	0.4817
1.0810	0.9948	0.9110	0.9200	0.2519
1.1245	0.9075	0.9480	0.8070	0.3292
0.9325	1.1860	0.7850	1.2720	0.0113
1.0465	1.0423	0.8817	0.9960	0.9571
1.0515	1.0125	0.8850	0.9630	0.4356
1.0545	0.9878	0.8883	0.9370	0.4898
1.0750	1.0053	0.9053	0.9350	0.1373
1.0855	1.0145	0.9140	0.9350	0.6942
1.0943	0.9773	0.9213	0.8930	0.2190
1.0998	0.9590	0.9260	0.8720	0.0086
0.8033	1.4773	0.6753	1.8390	0.0435
1.0475	1.0473	0.8813	1.0000	0.9215
1.0608	1.0278	0.8923	0.9690	0.6957
1.0673	1.0278	0.8980	0.9630	0.4545
1.0883	0.9913	0.9153	0.9110	0.1677
1.0890	0.9610	0.9153	0.8820	0.0416
1.0890	0.9585	0.9160	0.8800	0.0616
1.0913	0.9808	0.9173	0.8990	0.0390
1.2315	0.7465	1.0360	0.6060	0.1131
1.2385	0.7130	1.0415	0.5760	0.0778
1.0325	1.0718	0.8673	1.0380	0.8454
1.0463	1.0390	0.8787	0.9930	0.9341
1.0620	1.0138	0.8923	0.9550	0.4260
1.0643	1.0125	0.8940	0.9510	0.3858
1.0643	1.0150	0.8943	0.9540	0.3868
1.0890	0.9798	0.9143	0.9000	0.0399
1.0935	0.9953	0.9180	0.9100	0.0820
1.0930	0.9795	0.9180	0.8960	0.0106

1.1015	0.9823	0.9253	0.8920	0.0765
1.1980	0.7935	1.0060	0.6620	0.0206
1.0250	1.0765	0.8603	1.0500	0.2894
1.0353	1.0773	0.8687	1.0410	0.5523
1.0413	1.0600	0.8733	1.0180	0.8074
1.0535	1.0090	0.8840	0.9580	0.6173
1.0620	1.0283	0.8907	0.9680	0.1830
1.0755	1.0885	0.9025	1.0120	0.7978
1.0785	0.9775	0.9053	0.9060	0.0010
1.0878	0.9983	0.9127	0.9180	0.0236
1.0885	0.9723	0.9133	0.8930	0.0876
1.1045	0.9795	0.9265	0.8870	0.4959
1.1115	0.9645	0.9330	0.8680	0.3117
1.1300	0.8883	0.9483	0.7860	0.0983
1.1325	0.9230	0.9505	0.8150	0.3439
1.1345	0.8900	0.9520	0.7840	0.0126
0.9800	1.1145	0.8210	1.1370	0.2778
0.9880	1.1463	0.8277	1.1600	0.1856
1.0485	1.0180	0.8790	0.9710	0.3231
1.0635	1.0120	0.8910	0.9520	0.2927
1.0648	1.0163	0.8927	0.9540	0.1871
1.0685	0.9880	0.8950	0.9250	0.5252
1.0943	0.9655	0.9170	0.8820	0.0981
1.1135	0.9655	0.9335	0.8670	0.4252
1.1475	0.8928	0.9620	0.7780	0.2768
1.0085	1.0780	0.8440	1.0690	0.0395
1.0375	1.0335	0.8680	0.9960	0.9408
1.0418	1.0138	0.8717	0.9730	0.5056
1.0453	1.0755	0.8750	1.0290	0.7591
1.0540	1.0300	0.8820	0.9770	0.3417
1.0633	1.0128	0.8903	0.9530	0.4264
1.0743	1.0098	0.8997	0.9400	0.1336
1.0795	1.0008	0.9033	0.9270	0.2239

1.0925	1.0050	0.9145	0.9200	0.6927
1.0958	0.9833	0.9170	0.8970	0.0229
1.1003	0.9650	0.9210	0.8770	0.2996
1.1173	0.9368	0.9353	0.8380	0.1118
1.1443	0.8423	0.9573	0.7360	0.0075
1.2335	0.7668	1.0330	0.6220	0.0056
0.9805	1.1733	0.8197	1.1970	0.0314
1.0325	1.0385	0.8630	1.0060	0.9856
1.0533	1.0220	0.8803	0.9700	0.5936
1.0693	1.0360	0.8937	0.9690	0.4126
1.0808	0.9970	0.9040	0.9230	0.0879
1.0988	0.9833	0.9190	0.8950	0.0241
1.1023	0.9648	0.9210	0.8750	0.0037
1.1243	0.9418	0.9400	0.8380	0.0272
1.1465	0.8990	0.9580	0.7840	0.0562
1.0430	1.0665	0.8707	1.0230	0.5055
1.0638	1.0470	0.8880	0.9840	0.6793
1.0643	1.0243	0.8890	0.9620	0.3550
1.0655	1.0070	0.8893	0.9450	0.2957
1.1030	0.9638	0.9213	0.8740	0.0217
1.1138	0.9565	0.9300	0.8590	0.1283
1.1280	0.9350	0.9413	0.8290	0.0194
1.1348	0.9058	0.9477	0.7980	0.0387
1.1580	0.8600	0.9670	0.7430	0.0280
1.0510	1.0465	0.8770	0.9960	0.9306
1.0620	0.9995	0.8860	0.9410	0.4072
1.0765	1.0063	0.8980	0.9350	0.3900
1.0955	0.9873	0.9133	0.9010	0.1298
1.1265	0.9060	0.9390	0.8040	0.1512
1.1975	0.7875	0.9990	0.6580	0.0923
1.0040	1.0760	0.8360	1.0720	0.4524
1.0483	1.0585	0.8737	1.0100	0.8100
1.0520	1.0840	0.8760	1.0300	0.8313

1.0573	1.0408	0.8807	0.9840	0.8475
1.0758	1.0075	0.8960	0.9370	0.2915
1.1150	0.9265	0.9290	0.8310	0.0266
1.1865	0.8275	0.9885	0.6970	0.0905
1.0090	1.1720	0.8395	1.1620	0.4182
1.0265	1.0525	0.8540	1.0250	0.3900
1.0528	1.0415	0.8763	0.9890	0.8696
1.0585	1.0065	0.8810	0.9510	0.4564
1.0645	0.9920	0.8860	0.9320	0.6653
1.0765	1.0120	0.8957	0.9400	0.4655
1.0775	1.0078	0.8967	0.9350	0.1733
1.0895	0.9910	0.9063	0.9100	0.0876
1.0940	1.0110	0.9100	0.9240	0.2036
1.0998	0.9730	0.9147	0.8850	0.1710
1.1003	0.9378	0.9157	0.8520	0.0106
1.1008	0.9868	0.9160	0.8960	0.0409
1.0125	1.0420	0.8410	1.0290	0.0586
1.0230	1.0580	0.8500	1.0340	0.6747
1.0255	1.0560	0.8520	1.0300	0.4518
1.0465	1.1020	0.8695	1.0530	0.7356
1.0595	1.0120	0.8800	0.9550	0.5110
1.0595	1.0438	0.8803	0.9850	0.7919
1.0860	0.9585	0.9020	0.8830	0.1028
1.1005	0.9683	0.9140	0.8800	0.0685
1.1668	0.8005	0.9690	0.6860	0.0276
1.2805	0.6653	1.0647	0.5200	0.0002
1.0398	1.0780	0.8627	1.0370	0.7691
1.0480	1.0488	0.8697	1.0010	0.9851
1.0560	1.0110	0.8770	0.9570	0.3718
1.0730	1.0353	0.8907	0.9650	0.4787
1.0758	1.0020	0.8933	0.9310	0.1486
1.0770	1.0073	0.8937	0.9350	0.4924
1.0838	1.0030	0.8993	0.9250	0.1384

1.0910	1.0165	0.9050	0.9320	0.4209
1.0950	0.9748	0.9083	0.8900	0.5573
1.0985	0.9975	0.9120	0.9080	0.5800
1.1590	0.8610	0.9620	0.7430	0.0439
1.0268	1.0663	0.8507	1.0380	0.6858
1.0290	1.0530	0.8530	1.0230	0.7335
1.0715	1.0158	0.8887	0.9480	0.1737
1.0763	1.0093	0.8927	0.9380	0.4510
1.0875	0.9650	0.9020	0.8870	0.1847
1.1148	0.9453	0.9247	0.8480	0.1285
1.1320	0.9005	0.9380	0.7950	0.1049
1.1355	0.9315	0.9410	0.8200	0.2070
1.2328	0.7485	1.0223	0.6070	0.0265
1.0130	1.0745	0.8390	1.0610	0.4701
1.0465	1.0285	0.8670	0.9830	0.8449
1.0695	0.9975	0.8860	0.9330	0.7286
1.0923	0.9813	0.9040	0.8980	0.0793
1.0920	1.0240	0.9045	0.9380	0.5397
1.2200	0.7695	1.0097	0.6310	0.0009
1.0495	1.0615	0.8683	1.0110	0.8809
1.0675	0.9960	0.8830	0.9330	0.1180
1.0820	1.0123	0.8950	0.9360	0.0629
1.0855	1.0035	0.8973	0.9240	0.0220
1.0870	1.0250	0.8993	0.9430	0.4069
1.0960	0.9848	0.9060	0.8980	0.4051
1.0980	0.9860	0.9085	0.8980	0.1008
1.1180	0.9738	0.9247	0.8710	0.5159
1.0415	1.0165	0.8600	0.9760	0.5399
1.0818	1.0045	0.8937	0.9290	0.2614
1.0840	1.0018	0.8957	0.9240	0.2490
1.0950	1.0095	0.9040	0.9220	0.4212
1.2295	0.7445	1.0160	0.6060	0.2176
1.3050	0.6510	1.0780	0.4990	0.0349

1.0185	1.0710	0.8400	1.0520	0.5879
1.0280	1.1365	0.8485	1.1060	0.9392
1.0350	1.1315	0.8540	1.0930	0.0708
1.0460	1.0653	0.8633	1.0180	0.8617
1.0635	1.0388	0.8777	0.9770	0.5113
1.0803	1.0088	0.8910	0.9340	0.3974
1.0883	0.9768	0.8980	0.8980	0.0704
1.0900	0.9940	0.8997	0.9120	0.1185
1.0938	0.9553	0.9023	0.8730	0.0010
1.0960	0.9560	0.9040	0.8720	0.1971
1.1130	0.9450	0.9183	0.8490	0.0078
1.1245	0.9150	0.9280	0.8140	0.0536
1.2035	0.8060	0.9930	0.6700	0.0834
0.9935	1.1200	0.8190	1.1270	0.0059
1.0365	1.0828	0.8543	1.0450	0.5074
1.0575	1.0543	0.8710	0.9970	0.9445
1.0668	1.0393	0.8787	0.9740	0.3867
1.0760	1.0183	0.8867	0.9460	0.3649
1.0845	0.9645	0.8940	0.8890	0.0247
1.0928	0.9898	0.9003	0.9060	0.2441
1.0935	0.9585	0.9010	0.8770	0.4201
1.1045	1.0250	0.9100	0.9280	0.4181
1.1050	0.9980	0.9100	0.9030	0.0728
1.1185	0.9493	0.9217	0.8490	0.1158
1.1843	0.8250	0.9763	0.6970	0.0138
1.1965	0.8190	0.9860	0.6840	0.1633
1.2213	0.7743	1.0063	0.6340	0.0176
1.0138	1.1025	0.8340	1.0880	0.1672
1.0555	1.0338	0.8687	0.9790	0.6484
1.0713	1.0215	0.8820	0.9540	0.1648
1.0818	1.0110	0.8900	0.9350	0.4257
1.0923	0.9900	0.8993	0.9060	0.0570
1.0928	0.9915	0.8997	0.9070	0.2091

1.1460	0.9230	0.9430	0.8050	0.2207
1.0320	1.0510	0.8480	1.0180	0.5914
1.0485	1.0350	0.8620	0.9870	0.7362
1.0590	1.0100	0.8700	0.9540	0.5741
1.0635	1.0418	0.8747	0.9800	0.5733
1.0753	0.9938	0.8843	0.9240	0.1445
1.0773	1.0050	0.8860	0.9330	0.0600
1.0838	1.0265	0.8903	0.9470	0.6976
1.0920	0.9803	0.8980	0.8980	0.0051
1.0995	0.9798	0.9033	0.8910	0.0221
1.1043	0.9735	0.9073	0.8820	0.1108
1.1240	0.9385	0.9240	0.8350	0.0231
1.1265	0.9125	0.9260	0.8100	0.0625
1.1810	0.8518	0.9710	0.7210	0.1099
1.2808	0.7163	1.0533	0.5590	0.0067
1.0350	1.1370	0.8500	1.0990	0.6407
1.0498	1.0538	0.8617	1.0040	0.9297
1.0633	1.0373	0.8733	0.9760	0.6406
1.0853	1.0158	0.8907	0.9360	0.2292
1.0870	0.9785	0.8920	0.9000	0.3747
1.0903	1.0123	0.8950	0.9280	0.0264
1.0998	0.9785	0.9030	0.8900	0.0722
1.1015	1.0090	0.9040	0.9160	0.4122
1.1265	0.9125	0.9250	0.8100	0.1668
1.1443	0.9013	0.9400	0.7880	0.1147
1.1605	0.8670	0.9530	0.7470	0.0453
1.2538	0.6800	1.0293	0.5420	0.0412
1.0688	0.9898	0.8767	0.9260	0.2545
1.0723	1.0233	0.8797	0.9540	0.1463
1.0745	1.0148	0.8813	0.9440	0.1595
1.0898	0.9723	0.8937	0.8920	0.1118
1.0160	1.0750	0.8320	1.0580	0.1902
1.0210	1.0655	0.8360	1.0440	0.2931

1.0310	1.0750	0.8440	1.0430	0.3325
1.0405	1.0405	0.8520	1.0000	0.9548
1.0510	1.0125	0.8610	0.9630	0.6397
1.0575	1.0513	0.8657	0.9940	0.9948
1.0588	1.0560	0.8673	0.9970	0.9081
1.0610	1.0635	0.8690	1.0020	0.8916
1.0615	1.0490	0.8697	0.9880	0.8581
1.0650	1.0415	0.8723	0.9780	0.5248
1.0843	0.9723	0.8877	0.8970	0.2010
1.1600	0.9035	0.9495	0.7790	0.1468
1.0123	1.0860	0.8277	1.0730	0.1328
1.0160	1.0720	0.8310	1.0550	0.4654
1.0385	1.0435	0.8490	1.0050	0.8948
1.0648	1.0285	0.8713	0.9660	0.3673
1.0878	1.0175	0.8893	0.9350	0.2614
1.0965	1.0018	0.8970	0.9140	0.1886
1.1125	0.9900	0.9100	0.8900	0.0771
1.1125	0.9895	0.9105	0.8890	0.4657
1.1210	0.9213	0.9173	0.8220	0.0448
1.2365	0.7498	1.0110	0.6060	0.0075
1.0210	1.0810	0.8340	1.0590	0.0262
1.0575	1.0540	0.8643	0.9970	0.9844
1.0580	1.0453	0.8643	0.9880	0.8845
1.0603	1.0210	0.8663	0.9630	0.2839
1.0695	1.0250	0.8737	0.9580	0.2487
1.0705	1.0725	0.8750	1.0020	0.9984
1.0800	1.0565	0.8820	0.9780	0.9331
1.0920	1.0015	0.8917	0.9170	0.0716
1.0973	0.9885	0.8967	0.9010	0.0131
1.1290	0.9545	0.9225	0.8450	0.5331
1.1333	0.9235	0.9260	0.8150	0.0525
1.0355	1.0880	0.8453	1.0510	0.1398
1.0713	1.0385	0.8747	0.9690	0.7971

1.0750	0.9905	0.8770	0.9210	0.5133
1.0778	1.0178	0.8793	0.9440	0.5453
1.1685	0.8795	0.9540	0.7530	0.0318
1.2065	0.7790	0.9840	0.6460	0.1954
1.0515	1.0660	0.8567	1.0140	0.7982
1.0643	1.0463	0.8673	0.9830	0.7727
1.0800	0.9950	0.8797	0.9210	0.0296
1.0818	1.0135	0.8813	0.9370	0.3995
1.0850	1.0115	0.8847	0.9320	0.2538
1.0855	1.0218	0.8850	0.9410	0.2690
1.1015	0.9793	0.8973	0.8890	0.0933
1.1135	0.9465	0.9070	0.8500	0.0146
1.0588	1.0550	0.8613	0.9960	0.9434
1.0603	1.0600	0.8633	1.0000	0.9697
1.0753	1.0280	0.8750	0.9560	0.3694
1.0860	0.9853	0.8840	0.9070	0.1255
1.1250	0.9710	0.9160	0.8630	0.4642
1.1390	0.8995	0.9270	0.7900	0.1170
1.2198	0.7823	0.9927	0.6410	0.0281
1.0583	1.0538	0.8600	0.9960	0.9115
1.0620	1.0060	0.8630	0.9470	0.5097
1.0783	1.0118	0.8767	0.9380	0.1215
1.0843	1.0170	0.8810	0.9380	0.1379
1.1025	0.9828	0.8963	0.8910	0.0719
1.1163	0.9705	0.9077	0.8690	0.0206
1.1205	0.9760	0.9105	0.8710	0.4359
1.1220	0.9775	0.9120	0.8710	0.0008
1.0628	1.0330	0.8633	0.9720	0.6439
1.0755	1.0248	0.8737	0.9530	0.5017
1.0773	1.0120	0.8743	0.9390	0.0679
1.0870	0.9948	0.8830	0.9150	0.5307
1.0923	1.0785	0.8873	0.9870	0.9004
1.0975	0.9955	0.8917	0.9070	0.5701

1.1030	0.9918	0.8953	0.8990	0.0259
1.1205	0.9760	0.9100	0.8710	0.5029
1.1215	0.9330	0.9103	0.8320	0.0027
1.1290	0.9135	0.9170	0.8090	0.3529
1.1415	0.8990	0.9270	0.7880	0.0316
1.1605	0.8710	0.9420	0.7510	0.0047
1.1648	0.8550	0.9460	0.7340	0.0098
0.9605	1.1795	0.7790	1.2280	0.7920
1.0468	1.0625	0.8487	1.0150	0.8800
1.0833	1.0208	0.8780	0.9420	0.2574
1.0863	1.0018	0.8810	0.9220	0.0823
1.1260	0.8970	0.9130	0.7970	0.1159
1.0493	1.0595	0.8500	1.0100	0.8969
1.0790	0.9820	0.8740	0.9100	0.4028
1.0973	0.9730	0.8887	0.8870	0.0453
1.0993	0.9910	0.8903	0.9020	0.2010
1.1185	0.9625	0.9057	0.8610	0.2004
1.2148	0.8240	0.9840	0.6780	0.0031
0.9760	1.1375	0.7900	1.1650	0.2182
1.0030	1.1468	0.8117	1.1430	0.2722
1.0545	1.0210	0.8530	0.9680	0.9293
1.0600	1.0608	0.8573	1.0010	0.9439
1.0830	0.9845	0.8760	0.9090	0.1897
1.0973	1.0158	0.8880	0.9260	0.1673
1.1188	0.9408	0.9047	0.8410	0.1100
1.1190	0.9790	0.9055	0.8750	0.5301
1.1580	0.8830	0.9370	0.7630	0.2897
1.0270	1.0650	0.8300	1.0370	0.2346
1.0435	1.0765	0.8437	1.0320	0.6997
1.0670	1.0350	0.8627	0.9700	0.2643
1.0680	1.0433	0.8633	0.9770	0.7282
1.0768	1.0285	0.8697	0.9550	0.5180
1.0795	0.9830	0.8720	0.9110	0.6443

1.0963	0.9965	0.8853	0.9090	0.1434
1.0978	0.9880	0.8867	0.9000	0.0642
1.1015	0.9833	0.8903	0.8930	0.0911
1.1045	0.9460	0.8920	0.8560	0.5743
1.1265	0.9750	0.9105	0.8660	0.3003
1.1350	0.9545	0.9175	0.8410	0.3305
1.1943	0.8328	0.9650	0.6970	0.0138
1.0485	1.0330	0.8460	0.9850	0.7756
1.0913	1.0068	0.8807	0.9230	0.1420
1.1300	0.9190	0.9120	0.8130	0.2589
1.1530	0.9210	0.9310	0.7990	0.2256
0.9045	1.2470	0.7290	1.3790	0.1767
1.0490	1.1205	0.8450	1.0680	0.4683
1.0543	1.0478	0.8493	0.9940	0.8809
1.0595	1.0190	0.8540	0.9620	0.5419
1.0643	1.0525	0.8573	0.9890	0.7914
1.0860	1.0168	0.8753	0.9360	0.1780
1.0918	1.0203	0.8800	0.9350	0.1131
1.1290	0.9138	0.9097	0.8090	0.0168
1.1440	0.8955	0.9220	0.7830	0.0661
1.1730	0.8895	0.9460	0.7580	0.1683
1.0580	1.0695	0.8520	1.0110	0.8692
1.0733	1.0340	0.8640	0.9630	0.2705
1.0788	1.0210	0.8687	0.9460	0.4025
1.0938	0.9875	0.8810	0.9030	0.0294
1.1060	0.9900	0.8900	0.8950	0.1181
1.1150	0.9868	0.8977	0.8850	0.0312
1.1280	0.9740	0.9075	0.8630	0.0544
1.1555	0.9203	0.9297	0.7960	0.0897
0.9605	1.1505	0.7720	1.1980	0.0376
1.0145	1.0833	0.8153	1.0680	0.2559
1.0518	1.0763	0.8453	1.0230	0.7911
1.0620	1.0995	0.8535	1.0350	0.4719

1.0728	1.0388	0.8620	0.9680	0.6114
1.0855	0.9925	0.8730	0.9140	0.7995
1.0925	0.9700	0.8780	0.8880	0.3527
1.1040	0.9343	0.8880	0.8460	0.0042
1.1058	0.9885	0.8890	0.8940	0.0141
1.1115	0.9713	0.8940	0.8740	0.0980
1.1170	0.9955	0.8980	0.8910	0.1567
1.1240	0.9545	0.9033	0.8490	0.0120
1.1293	0.9485	0.9077	0.8400	0.0485
1.1578	0.8843	0.9307	0.7640	0.0386
0.7680	1.5740	0.6167	2.0490	0.1264
1.0175	1.0760	0.8170	1.0570	0.8525
1.0630	1.0413	0.8537	0.9800	0.6527
1.0870	1.0130	0.8723	0.9320	0.3289
1.0920	0.9700	0.8770	0.8880	0.3661
1.1085	0.9833	0.8903	0.8870	0.0898
1.1405	0.9015	0.9160	0.7900	0.1326
1.1408	0.9255	0.9160	0.8110	0.0200
1.0478	1.0513	0.8407	1.0030	0.9855
1.0520	1.0598	0.8433	1.0070	0.7908
1.1188	0.9648	0.8977	0.8620	0.1040
1.2798	0.6845	1.0263	0.5350	0.0016
1.0388	1.0955	0.8323	1.0550	0.4038
1.0568	1.0660	0.8467	1.0090	0.9186
1.0655	1.0550	0.8533	0.9900	0.8534
1.0793	1.0335	0.8650	0.9580	0.4691
1.0843	1.0203	0.8687	0.9410	0.2471
1.0938	1.0085	0.8763	0.9220	0.2859
1.1065	0.9520	0.8860	0.8600	0.1637
1.1070	0.9625	0.8867	0.8690	0.0258
1.1198	0.9633	0.8973	0.8600	0.1853
1.1235	0.9865	0.8995	0.8780	0.1582
1.1525	0.9123	0.9237	0.7920	0.0644

1.2145	0.7848	0.9733	0.6460	0.0355
1.2248	0.7870	0.9807	0.6430	0.0001
1.2255	0.7835	0.9813	0.6390	0.0153
1.0740	1.0000	0.8590	0.9310	0.3993
1.0888	1.0298	0.8707	0.9460	0.3459
1.1025	1.0385	0.8820	0.9420	0.2032
1.1060	1.0035	0.8843	0.9070	0.2669
1.1348	0.9470	0.9080	0.8350	0.0032
1.1485	0.9153	0.9190	0.7970	0.0034
1.1528	0.9378	0.9217	0.8130	0.0119
0.9298	1.2873	0.7430	1.3850	0.0835
1.0880	1.0130	0.8697	0.9310	0.4394
1.0995	0.9715	0.8790	0.8840	0.2757
1.1175	0.9665	0.8923	0.8650	0.1197
1.1550	0.9080	0.9233	0.7860	0.0259
1.1770	0.8540	0.9410	0.7260	0.0022
1.0605	1.0185	0.8460	0.9600	0.7446
1.0918	1.0160	0.8710	0.9310	0.3065
1.0925	1.0055	0.8713	0.9200	0.0784
1.1065	0.9730	0.8833	0.8790	0.0004
1.1188	0.9663	0.8927	0.8640	0.0926
1.1285	0.9805	0.9010	0.8690	0.2446
1.1368	0.9338	0.9077	0.8210	0.0991
1.1413	0.9520	0.9110	0.8340	0.4408
1.0265	1.1100	0.8180	1.0810	0.3183
1.0445	1.0710	0.8323	1.0250	0.8309
1.0810	1.0150	0.8620	0.9390	0.5875
1.0830	0.9918	0.8627	0.9160	0.3100
1.0945	0.9818	0.8723	0.8970	0.0373
1.0998	1.0155	0.8770	0.9230	0.0187
1.1035	0.9595	0.8790	0.8700	0.1391
1.1175	0.9620	0.8910	0.8610	0.3747
1.1588	0.8973	0.9240	0.7740	0.0513

1.1598	0.9000	0.9243	0.7760	0.1115
1.2325	0.8658	0.9823	0.7020	0.0129
1.0560	1.0738	0.8407	1.0170	0.7636
1.0783	1.0630	0.8580	0.9860	0.7971
1.0848	1.0265	0.8640	0.9460	0.4499
1.0945	1.0113	0.8713	0.9240	0.1005
1.1025	0.9945	0.8777	0.9020	0.2851
1.1083	0.9860	0.8817	0.8900	0.0400
1.0453	1.0735	0.8310	1.0270	0.8260
1.0618	1.0648	0.8443	1.0030	0.9899
1.0678	1.0295	0.8487	0.9640	0.5006
1.0713	1.0495	0.8520	0.9800	0.6004
1.0855	1.0185	0.8633	0.9380	0.0190
1.0885	1.0270	0.8653	0.9440	0.2937
1.0970	1.0088	0.8723	0.9200	0.3274
1.1040	0.9943	0.8780	0.9010	0.2109
1.1185	0.9400	0.8890	0.8400	0.0352
1.0345	1.0620	0.8210	1.0270	0.4434
1.0365	1.0565	0.8230	1.0190	0.7742
1.0540	1.0345	0.8370	0.9810	0.8623
1.0580	1.0768	0.8397	1.0180	0.8362
1.0575	1.0743	0.8397	1.0160	0.8766
1.0640	1.0543	0.8443	0.9910	0.8659
1.0745	1.0865	0.8530	1.0110	0.9984
1.0748	1.0285	0.8530	0.9570	0.5654
1.0830	1.0288	0.8597	0.9500	0.3456
1.0850	1.0293	0.8613	0.9490	0.4023
1.0860	1.0345	0.8627	0.9530	0.2674
1.0925	1.0525	0.8670	0.9630	0.4763
1.1008	0.9823	0.8740	0.8920	0.2064
1.1480	0.8930	0.9120	0.7780	0.2528
1.1865	0.8658	0.9423	0.7300	0.0405
1.1960	0.8793	0.9493	0.7350	0.1452

1.0598	1.0553	0.8400	0.9960	0.9703
1.0688	1.0518	0.8480	0.9840	0.7647
1.0745	1.0025	0.8520	0.9330	0.3216
1.0770	1.0465	0.8543	0.9720	0.4605
1.0843	1.0220	0.8597	0.9430	0.2799
1.1030	0.9915	0.8747	0.8990	0.1063
1.1325	0.9483	0.8977	0.8370	0.0433
1.1348	0.9480	0.9003	0.8350	0.0922
1.0350	1.1100	0.8193	1.0720	0.3240
1.0645	1.0685	0.8427	1.0040	0.9497
1.0878	1.0275	0.8620	0.9450	0.3136
1.1010	0.9825	0.8720	0.8920	0.1194
1.1030	0.9988	0.8737	0.9050	0.2868
1.1055	1.0015	0.8757	0.9060	0.0784
1.1355	0.9735	0.8995	0.8570	0.1376
1.1400	0.9665	0.9030	0.8480	0.1847
1.1750	0.8990	0.9310	0.7650	0.1605
0.9880	1.1285	0.7820	1.1420	0.0685
1.0605	1.0260	0.8390	0.9670	0.3767
1.0655	1.0538	0.8433	0.9890	0.8351
1.0770	1.0410	0.8520	0.9670	0.6522
1.0883	0.9788	0.8610	0.8990	0.2195
1.0993	0.9890	0.8700	0.9000	0.2560
1.1093	0.9675	0.8773	0.8720	0.0369
1.1110	0.9868	0.8793	0.8880	0.0978
1.1135	1.0043	0.8813	0.9020	0.2000
1.1170	1.0415	0.8840	0.9320	0.3475
1.1190	0.9825	0.8850	0.8780	0.3103
1.1520	0.9218	0.9117	0.8000	0.0095
1.2023	0.8208	0.9507	0.6830	0.0017
1.0488	1.0938	0.8283	1.0430	0.2990
1.0650	1.0628	0.8410	0.9980	0.9221
1.0690	1.0670	0.8447	0.9980	0.9764

1.0755	1.0035	0.8500	0.9330	0.0586
1.0930	1.0153	0.8640	0.9290	0.3548
1.1015	0.9853	0.8703	0.8940	0.0370
1.1013	1.0153	0.8703	0.9220	0.1494
1.0788	1.0403	0.8513	0.9640	0.5354
1.0830	0.9935	0.8550	0.9170	0.2624
1.0865	1.0268	0.8570	0.9450	0.5146
1.0890	0.9860	0.8590	0.9050	0.6007
1.0895	0.9840	0.8600	0.9030	0.1765
1.0965	1.0263	0.8653	0.9360	0.1644
1.0978	1.0200	0.8667	0.9290	0.2136
1.1093	0.9903	0.8753	0.8930	0.2488
1.1730	0.8410	0.9250	0.7170	0.0190
0.9875	1.1300	0.7780	1.1440	0.4535
1.0555	1.0010	0.8313	0.9480	0.4428
1.0640	1.0240	0.8380	0.9620	0.7125
1.0645	1.0630	0.8387	0.9990	0.9775
1.0910	1.0170	0.8597	0.9320	0.2059
1.0935	0.9750	0.8620	0.8920	0.5014
1.1068	0.9998	0.8723	0.9030	0.0688
1.1070	1.0158	0.8727	0.9180	0.2530
1.1083	0.9935	0.8733	0.8960	0.1928
1.1120	1.0220	0.8760	0.9190	0.5259
1.1178	0.9483	0.8813	0.8480	0.0274
1.0585	1.0863	0.8327	1.0260	0.8032
1.0700	1.0115	0.8420	0.9450	0.6008
1.0845	1.0315	0.8537	0.9510	0.4901
1.0855	1.0153	0.8543	0.9350	0.1923
1.0923	1.0085	0.8600	0.9230	0.0917
1.0963	1.0128	0.8623	0.9240	0.1441
1.0980	1.0043	0.8637	0.9150	0.1809
1.1028	1.0068	0.8677	0.9130	0.0822
1.1165	0.9795	0.8790	0.8770	0.0824

1.1200	1.0105	0.8810	0.9020	0.3190
1.1625	0.8770	0.9150	0.7540	0.2067
1.1870	0.8663	0.9337	0.7300	0.0103
1.2320	0.7820	0.9693	0.6350	0.0217
1.0970	1.0003	0.8620	0.9120	0.0766
1.0993	1.0075	0.8640	0.9170	0.2184
1.2900	0.6940	1.0137	0.5380	0.0267
1.3315	0.6330	1.0460	0.4750	0.1425
1.0360	1.0655	0.8130	1.0280	0.7815
1.0513	1.1030	0.8257	1.0490	0.5046
1.0713	1.0435	0.8413	0.9740	0.6127
1.0760	1.0075	0.8450	0.9360	0.3020
1.0810	1.0383	0.8490	0.9600	0.7200
1.0913	1.0378	0.8570	0.9510	0.3355
1.0968	1.0180	0.8607	0.9280	0.1143
1.0990	0.9865	0.8630	0.8980	0.1083
1.1065	1.0073	0.8690	0.9100	0.0254
1.1135	0.9943	0.8737	0.8930	0.0460
1.1350	0.9598	0.8910	0.8460	0.0648
1.1613	0.9008	0.9113	0.7760	0.0000
1.2220	0.7900	0.9590	0.6460	0.2745
1.0333	1.1200	0.8097	1.0840	0.6141
1.0803	1.0133	0.8467	0.9380	0.4610
1.0813	1.0250	0.8473	0.9480	0.3602
1.0813	1.0480	0.8477	0.9690	0.6063
1.0875	1.0268	0.8530	0.9440	0.4009
1.0885	1.0560	0.8535	0.9700	0.7603
1.0993	0.9793	0.8620	0.8910	0.0614
1.1008	1.0098	0.8630	0.9170	0.5027
1.1318	0.9668	0.8873	0.8540	0.0239
1.1440	0.8975	0.8970	0.7850	0.0687
1.0410	1.0560	0.8150	1.0140	0.8017
1.0460	1.0818	0.8187	1.0340	0.7406

1.0880	1.0118	0.8517	0.9300	0.3662
1.1098	1.0005	0.8690	0.9020	0.1359
1.1390	0.9398	0.8923	0.8250	0.2000
1.1415	0.9140	0.8940	0.8010	0.3820
1.1500	0.9323	0.9000	0.8110	0.0034
1.2065	0.8353	0.9450	0.6920	0.0479
1.2115	0.9045	0.9490	0.7470	0.0468
1.2233	0.7920	0.9580	0.6470	0.0002
1.0720	1.0230	0.8380	0.9540	0.3398
1.1015	1.0105	0.8613	0.9170	0.3836
1.0160	1.0885	0.7930	1.0710	0.7091
1.0773	1.0165	0.8417	0.9440	0.1835
1.0933	1.0113	0.8540	0.9250	0.1868
1.0980	1.0215	0.8570	0.9300	0.2870
1.1055	0.9975	0.8630	0.9020	0.1147
1.1288	0.9753	0.8820	0.8640	0.0031
1.2615	0.7430	0.9855	0.5890	0.0553
1.2695	0.7295	0.9920	0.5750	0.0582
1.0585	1.0310	0.8260	0.9740	0.0003
1.0963	1.0433	0.8553	0.9520	0.2497
1.1105	0.9993	0.8667	0.9000	0.3329
1.1190	0.9473	0.8727	0.8470	0.0386
1.1440	0.9393	0.8920	0.8210	0.0742
1.2448	0.7808	0.9713	0.6270	0.0119
1.0670	1.0488	0.8307	0.9830	0.7897
1.0755	1.0105	0.8380	0.9400	0.1335
1.0850	1.0380	0.8453	0.9570	0.4821
1.0908	1.0278	0.8497	0.9420	0.4477
1.0950	0.9815	0.8530	0.8960	0.4402
1.0995	1.0098	0.8560	0.9180	0.1650
1.1040	1.0530	0.8595	0.9540	0.7570
1.1213	0.9778	0.8733	0.8720	0.1378
1.1273	0.9755	0.8787	0.8650	0.0240

1.1528	0.9255	0.8977	0.8030	0.0720
1.1845	0.8525	0.9230	0.7200	0.0731
1.2218	0.8075	0.9520	0.6610	0.0016
1.2445	0.7660	0.9700	0.6160	0.1106
1.0465	1.0968	0.8137	1.0480	0.9901
1.0740	1.0125	0.8360	0.9430	0.3640
1.0890	1.0018	0.8477	0.9200	0.0210
1.0910	1.0175	0.8483	0.9330	0.1721
1.1160	1.0070	0.8680	0.9020	0.2101
1.1178	0.9720	0.8697	0.8700	0.0207
1.1350	0.9895	0.8825	0.8720	0.2271
1.1468	0.9528	0.8927	0.8310	0.0307
1.1660	0.9340	0.9070	0.8010	0.0233
1.2033	0.8423	0.9363	0.7000	0.0032
1.2140	0.8415	0.9440	0.6930	0.0504
0.8530	1.3365	0.6630	1.5670	0.2563
0.9973	1.1783	0.7747	1.1810	0.3033
1.0665	1.0200	0.8290	0.9560	0.6891
1.0940	1.0145	0.8500	0.9270	0.3029
1.1073	0.9998	0.8607	0.9030	0.0552
1.1128	0.9758	0.8643	0.8770	0.0653
1.1138	1.0148	0.8653	0.9110	0.0609
1.1215	0.9815	0.8717	0.8750	0.2241
1.1673	0.9033	0.9070	0.7740	0.0014
1.1768	0.9298	0.9147	0.7900	0.0077
1.0750	1.0603	0.8337	0.9860	0.5851
1.0818	1.0458	0.8390	0.9670	0.7006
1.0848	1.0305	0.8417	0.9500	0.2985
1.0890	1.0403	0.8450	0.9550	0.4963
1.0978	0.9990	0.8517	0.9100	0.2572
1.1120	1.0080	0.8630	0.9060	0.2107
1.1620	0.8965	0.9020	0.7720	0.0138
1.0543	1.0850	0.8173	1.0290	0.7790

1.0600	1.0145	0.8210	0.9570	0.1759
1.1035	0.9735	0.8550	0.8820	0.5424
1.1095	1.0430	0.8595	0.9400	0.6440
1.1265	0.9803	0.8730	0.8700	0.1386
1.1330	0.9633	0.8783	0.8500	0.0226
1.1600	0.9500	0.8985	0.8190	0.2752
1.1335	0.9253	0.8777	0.8160	0.0094
1.1655	0.8825	0.9020	0.7570	0.1155
1.1953	0.8615	0.9247	0.7210	0.0040
1.2370	0.7840	0.9577	0.6340	0.0267
1.2390	0.7725	0.9590	0.6230	0.2377
0.9858	1.1910	0.7623	1.2080	0.0093
1.1235	0.9420	0.8690	0.8380	0.1293
1.1520	0.9020	0.8900	0.7830	0.1149
1.1515	0.9023	0.8903	0.7840	0.0120
1.0905	1.0313	0.8420	0.9460	0.5948
1.0948	1.0223	0.8447	0.9340	0.1186
1.0965	0.9820	0.8460	0.8960	0.2689
1.0970	1.0420	0.8473	0.9500	0.3001
1.1000	1.0175	0.8497	0.9250	0.2311
1.1123	1.0023	0.8587	0.9010	0.1058
1.1170	0.9903	0.8627	0.8870	0.0471
1.1363	0.9580	0.8777	0.8430	0.0544
1.1860	0.9105	0.9150	0.7680	0.0383
1.0288	1.1253	0.7927	1.0940	0.2889
1.0938	1.0205	0.8437	0.9330	0.2631
1.0968	1.0068	0.8457	0.9180	0.0998
1.1023	1.0070	0.8503	0.9140	0.1637
1.1043	1.0210	0.8513	0.9250	0.1120
1.1483	0.9365	0.8853	0.8160	0.0269
1.1540	0.9605	0.8895	0.8320	0.4406
0.9910	1.1355	0.7630	1.1460	0.2301
1.0810	1.0965	0.8325	1.0140	0.8752

1.0915	1.0830	0.8405	0.9920	0.7532
1.0935	1.0323	0.8420	0.9440	0.3892
1.1063	1.0240	0.8517	0.9260	0.4628
1.1125	0.9595	0.8570	0.8620	0.1700
1.1430	0.9825	0.8805	0.8600	0.2844
1.3270	0.6738	1.0220	0.5080	0.0035
1.0543	1.0935	0.8110	1.0370	0.5349
1.0840	1.0465	0.8333	0.9650	0.4907
1.0985	1.0248	0.8447	0.9330	0.0395
1.1115	0.9973	0.8543	0.8970	0.1177
1.1140	0.9978	0.8567	0.8960	0.0546
1.1185	1.0015	0.8607	0.8950	0.0179
1.1325	0.9880	0.8707	0.8720	0.0222
1.1320	0.9548	0.8710	0.8430	0.0299
1.1615	0.8900	0.8930	0.7660	0.1703
1.2280	0.8280	0.9440	0.6740	0.0195
1.2453	0.7858	0.9580	0.6310	0.0030
1.2523	0.7478	0.9627	0.5970	0.0061
1.2605	0.7755	0.9690	0.6150	0.1229
1.2745	0.7335	0.9800	0.5760	0.0404
1.0855	1.0260	0.8337	0.9450	0.2455
1.1000	1.0193	0.8443	0.9270	0.3266
1.1135	0.9850	0.8550	0.8850	0.3315
1.1205	0.9983	0.8603	0.8910	0.1276
1.1200	0.9375	0.8603	0.8370	0.0086
1.1755	0.9250	0.9030	0.7870	0.2021
1.0830	1.0448	0.8310	0.9650	0.6912
1.0933	1.0303	0.8387	0.9420	0.1248
1.0998	0.9910	0.8437	0.9010	0.3681
1.1035	0.9805	0.8460	0.8890	0.4232
1.1640	0.9465	0.8930	0.8130	0.2202
1.0485	1.1103	0.8037	1.0590	0.4414
1.0703	1.0720	0.8200	1.0020	0.9491

1.1075	1.0098	0.8487	0.9120	0.1430
1.1100	0.9868	0.8500	0.8890	0.0907
1.1150	0.9610	0.8540	0.8620	0.0258
1.1190	0.9875	0.8573	0.8820	0.0910
1.1215	0.9910	0.8590	0.8840	0.0760
1.1265	0.9678	0.8627	0.8590	0.0074
1.1370	1.0015	0.8715	0.8810	0.2164
1.1555	0.9025	0.8850	0.7810	0.0752
1.1555	0.9780	0.8855	0.8460	0.1967
1.0765	1.0348	0.8237	0.9610	0.6034
1.0878	1.0450	0.8320	0.9610	0.4890
1.0988	0.9978	0.8403	0.9080	0.0194
1.1145	1.0015	0.8530	0.8990	0.0469
1.1195	1.0335	0.8565	0.9230	0.6241
1.1250	0.9828	0.8607	0.8740	0.0262
1.1335	0.9678	0.8673	0.8540	0.0552
1.1418	0.9550	0.8740	0.8360	0.0217
1.1885	0.9020	0.9095	0.7590	0.2135
1.1108	1.0098	0.8483	0.9090	0.0115
1.1145	1.0130	0.8513	0.9090	0.0056
1.1398	0.9613	0.8713	0.8430	0.0592
1.0905	0.9950	0.8320	0.9120	0.3392
1.0910	1.0283	0.8323	0.9420	0.2271
1.1003	1.0198	0.8400	0.9270	0.0983
1.1045	1.0203	0.8430	0.9240	0.1931
1.1048	1.0235	0.8433	0.9260	0.1198
1.1183	0.9963	0.8527	0.8910	0.0684
1.1370	0.9685	0.8677	0.8520	0.0111
1.1468	0.9498	0.8750	0.8280	0.0136
1.0315	1.0935	0.7860	1.0600	0.3196
1.0785	1.0633	0.8217	0.9860	0.9178
1.0813	1.0513	0.8240	0.9720	0.6982
1.0988	1.0185	0.8370	0.9270	0.2306

1.1035	1.0235	0.8410	0.9280	0.2317
1.1098	1.0090	0.8460	0.9090	0.2045
1.1225	0.9900	0.8557	0.8820	0.0877
1.1235	0.9703	0.8563	0.8640	0.0080
1.1555	0.9343	0.8807	0.8090	0.0416
1.0480	1.1135	0.7977	1.0620	0.5792
1.0770	1.0320	0.8200	0.9580	0.3341
1.0838	1.0065	0.8247	0.9290	0.3575
1.0898	1.0340	0.8293	0.9490	0.4829
1.1023	1.0145	0.8387	0.9200	0.4357
1.1048	1.0368	0.8403	0.9380	0.2326
1.1213	0.9983	0.8533	0.8900	0.1557
1.1290	0.9765	0.8597	0.8650	0.1151
1.1403	0.9623	0.8677	0.8440	0.1422
1.1798	0.9085	0.8983	0.7700	0.0005
1.1868	0.8833	0.9037	0.7440	0.0103
1.2540	0.7650	0.9540	0.6100	0.0531
1.1038	1.0098	0.8383	0.9150	0.1751
1.1073	1.0063	0.8417	0.9090	0.0496
1.1135	1.0163	0.8463	0.9130	0.1046
1.1183	1.0005	0.8500	0.8950	0.1130
1.1195	0.9780	0.8507	0.8740	0.0327
1.1255	0.9513	0.8550	0.8450	0.1157
1.1885	0.8858	0.9027	0.7450	0.0893
1.2270	0.8315	0.9330	0.6780	0.0142
1.0885	1.0030	0.8260	0.9210	0.1459
1.1130	1.0600	0.8445	0.9520	0.4912
1.1180	1.0013	0.8483	0.8960	0.2781
1.0270	1.1305	0.7787	1.1010	0.4817
1.0755	1.0613	0.8157	0.9870	0.8133
1.0955	0.9895	0.8300	0.9030	0.5497
1.1115	0.9660	0.8430	0.8690	0.2793
1.1258	0.9783	0.8537	0.8690	0.0620

1.2130	0.8250	0.9190	0.6800	0.0522
1.3000	0.6980	0.9855	0.5370	0.0012
1.0505	1.0560	0.7950	1.0050	0.9313
1.0720	1.0260	0.8120	0.9570	0.5942
1.0933	1.0405	0.8273	0.9520	0.5284
1.1000	1.0260	0.8330	0.9330	0.6083
1.1270	1.0240	0.8535	0.9090	0.6840
1.1555	0.9470	0.8747	0.8200	0.0128
1.1815	0.8995	0.8943	0.7610	0.0063
1.2060	0.8405	0.9130	0.6970	0.2391
1.0635	1.0933	0.8040	1.0280	0.6809
1.0853	1.0618	0.8207	0.9780	0.8737
1.1113	1.0013	0.8397	0.9010	0.1808
1.1440	0.9408	0.8650	0.8220	0.0088
1.1590	0.8765	0.8767	0.7560	0.0583
1.1693	0.9253	0.8843	0.7910	0.1054
1.1848	0.9303	0.8953	0.7850	0.1000
1.1870	0.9200	0.8970	0.7750	0.0587
1.2005	0.8693	0.9077	0.7240	0.0073
1.0525	1.0565	0.7950	1.0040	0.9399
1.0753	1.0725	0.8117	0.9970	0.9049
1.1063	1.0248	0.8357	0.9260	0.4072
1.1250	1.0300	0.8495	0.9160	0.5101
1.1283	0.9780	0.8523	0.8670	0.0830
1.1560	0.9765	0.8725	0.8450	0.1795
1.1615	0.9153	0.8767	0.7880	0.0577
1.1815	0.9020	0.8923	0.7630	0.0020
1.1940	0.8570	0.9020	0.7180	0.0824
1.0858	1.0185	0.8190	0.9380	0.4452
1.0960	1.0313	0.8260	0.9410	0.4685
1.0975	1.0403	0.8273	0.9480	0.2633
1.1263	0.9898	0.8490	0.8790	0.2477
1.1375	0.9548	0.8573	0.8390	0.0291

1.1588	0.9403	0.8733	0.8110	0.1469
1.1618	0.9308	0.8757	0.8010	0.0056
1.0275	1.0935	0.7740	1.0640	0.4713
1.1048	1.0330	0.8320	0.9350	0.2121
1.1133	1.0060	0.8387	0.9040	0.0647
1.1135	1.0000	0.8390	0.8980	0.0804
1.1210	1.0008	0.8443	0.8930	0.0320
1.1215	1.0055	0.8443	0.8970	0.1278
1.1440	0.9605	0.8620	0.8400	0.0747
1.1950	0.9060	0.8995	0.7580	0.2352
1.2185	0.8290	0.9180	0.6800	0.3372
1.2260	0.8220	0.9227	0.6700	0.0095
1.0820	1.1150	0.8140	1.0300	0.9410
1.0915	1.0478	0.8207	0.9600	0.6748
1.0925	1.0273	0.8213	0.9400	0.3256
1.1145	1.0070	0.8383	0.9040	0.0421
1.1268	0.9898	0.8473	0.8780	0.0746
1.2345	0.8948	0.9283	0.7250	0.0582
1.2370	0.7930	0.9300	0.6410	0.0661
1.1063	1.0143	0.8310	0.9170	0.1028
1.1290	0.9855	0.8483	0.8730	0.0328
1.1298	0.9923	0.8490	0.8780	0.3188
1.1343	0.9953	0.8513	0.8770	0.0685
1.1565	0.9418	0.8683	0.8140	0.0150
1.2525	0.7945	0.9405	0.6340	0.0705
1.0755	1.0790	0.8067	1.0030	0.9508
1.1113	1.0320	0.8337	0.9290	0.3603
1.1270	1.0020	0.8457	0.8890	0.4931
1.1553	0.9645	0.8670	0.8350	0.1352
1.1783	0.9115	0.8840	0.7740	0.0237
1.2830	0.7315	0.9620	0.5700	0.0306
1.1043	1.0193	0.8267	0.9230	0.1280
1.1113	1.0105	0.8323	0.9090	0.2132

1.1155	1.0130	0.8350	0.9080	0.0611
1.1660	0.8940	0.8730	0.7670	0.1829
1.0500	1.0710	0.7850	1.0200	0.9812
1.0510	1.1165	0.7863	1.0620	0.6008
1.0590	1.0450	0.7920	0.9870	0.8849
1.0900	1.0305	0.8150	0.9450	0.2456
1.1563	0.9445	0.8653	0.8170	0.0344
1.1848	0.8988	0.8863	0.7590	0.0778
1.2638	0.7640	0.9447	0.6050	0.0599
1.0518	1.0810	0.7860	1.0280	0.7401
1.0705	1.0880	0.7997	1.0160	0.8039
1.1198	1.0238	0.8367	0.9140	0.8591
1.1220	0.9975	0.8387	0.8890	0.0220
1.1243	1.0015	0.8393	0.8910	0.0359
1.1430	0.9585	0.8540	0.8390	0.0067
1.1638	0.9305	0.8693	0.8000	0.0431
1.1795	0.8795	0.8810	0.7460	0.0344
1.1998	0.8845	0.8967	0.7370	0.0310
1.0570	1.0265	0.7880	0.9710	0.9032
1.1053	1.0485	0.8240	0.9490	0.8455
1.1045	1.0233	0.8243	0.9260	0.1051
1.1060	0.9825	0.8250	0.8880	0.2274
1.1405	0.9635	0.8510	0.8450	0.0023
1.0720	1.0315	0.7990	0.9620	0.3173
1.1040	1.0015	0.8220	0.9070	0.1153
1.1163	0.9983	0.8317	0.8940	0.0970
1.1243	0.9988	0.8380	0.8880	0.2355
1.1458	0.9663	0.8533	0.8430	0.1173
1.0470	1.0908	0.7793	1.0420	0.4624
1.1060	1.0303	0.8223	0.9320	0.3741
1.1060	1.0360	0.8223	0.9370	0.2724
1.0570	1.0940	0.7850	1.0350	0.4671
1.0688	1.0870	0.7937	1.0170	0.7796

1.1085	0.9920	0.8237	0.8950	0.1776
1.1520	0.9413	0.8563	0.8170	0.0005
1.1780	0.9475	0.8755	0.8040	0.2706
1.3295	0.6650	0.9875	0.5000	0.0393
1.0220	1.1290	0.7580	1.1050	0.0081
1.0983	1.0458	0.8147	0.9520	0.4036
1.1185	0.9598	0.8297	0.8580	0.0284
1.1348	0.9703	0.8420	0.8550	0.0015
1.1400	1.0205	0.8460	0.8950	0.5319
1.1460	0.9535	0.8500	0.8320	0.0615
1.1600	0.9255	0.8607	0.7980	0.0289
1.2068	0.8730	0.8953	0.7230	0.1102
1.0230	1.1030	0.7580	1.0780	0.1608
1.0955	0.9985	0.8120	0.9110	0.5096
1.1130	1.0540	0.8250	0.9470	0.4415
1.1670	0.9358	0.8647	0.8020	0.0090
1.0825	1.0195	0.8010	0.9420	0.2584
1.0975	1.0365	0.8117	0.9440	0.4916
1.1128	1.0483	0.8237	0.9420	0.3373
1.1265	1.0085	0.8333	0.8950	0.1099
1.1295	0.9958	0.8360	0.8820	0.0278
1.1410	1.0250	0.8440	0.8980	0.3650
1.1095	0.9810	0.8200	0.8840	0.1166
1.1105	1.0295	0.8207	0.9270	0.1085
1.1105	1.0300	0.8210	0.9280	0.4795
1.1145	1.0243	0.8240	0.9190	0.2172
1.1648	0.8828	0.8603	0.7580	0.0127
1.0550	1.0620	0.7790	1.0070	0.9730
1.1228	0.9948	0.8290	0.8860	0.3962
1.1250	0.9640	0.8300	0.8570	0.1606
1.1300	1.0115	0.8340	0.8950	0.0900
1.1383	0.9753	0.8397	0.8570	0.0796
1.1460	0.9738	0.8460	0.8500	0.0665

1.2500	0.8180	0.9220	0.6540	0.0237
1.3163	0.7283	0.9710	0.5530	0.0105
0.9445	1.2620	0.6963	1.3360	0.2740
1.0780	1.0285	0.7940	0.9540	0.3019
1.0955	1.0515	0.8070	0.9600	0.3860
1.1143	1.0133	0.8217	0.9090	0.0710
1.1283	1.0030	0.8317	0.8890	0.0944
1.1305	1.0078	0.8327	0.8910	0.1341
1.1310	1.0085	0.8333	0.8920	0.0627
1.1483	0.9903	0.8467	0.8620	0.0556
1.3035	0.7265	0.9607	0.5570	0.0185
1.0810	1.0323	0.7957	0.9550	0.5804
1.1085	1.0950	0.8155	0.9880	0.9077
1.1113	1.0053	0.8183	0.9050	0.2078
1.1343	0.9915	0.8353	0.8740	0.0519
1.1380	0.9858	0.8380	0.8660	0.0881
1.1293	0.9863	0.8303	0.8730	0.0276
1.1760	0.9260	0.8640	0.7870	0.0113
1.2178	0.8620	0.8953	0.7080	0.0059
1.2403	0.7438	0.9120	0.6000	0.0647
1.0945	1.0145	0.8033	0.9270	0.1712
1.1170	1.0235	0.8203	0.9160	0.2145
1.1238	1.0130	0.8247	0.9010	0.1247
1.4518	0.4690	1.0657	0.3230	0.0031
1.0900	1.0130	0.7990	0.9290	0.0301
1.0915	1.0115	0.8000	0.9270	0.3595
1.0963	1.0405	0.8033	0.9490	0.2881
1.1048	1.0188	0.8097	0.9220	0.1187
1.1293	1.0293	0.8277	0.9110	0.3660
1.1500	0.9280	0.8430	0.8070	0.0637
1.1548	0.9678	0.8467	0.8380	0.0681
1.1580	0.9545	0.8490	0.8240	0.2068
1.1678	0.9513	0.8557	0.8150	0.0052

1.1950	0.9355	0.8765	0.7830	0.3641
1.0890	1.0618	0.7967	0.9750	0.7478
1.1178	1.0283	0.8180	0.9200	0.2420
1.1235	0.9920	0.8220	0.8830	0.1473
1.1390	0.9795	0.8337	0.8600	0.0174
1.1513	0.9540	0.8423	0.8290	0.0181
1.1523	0.9600	0.8433	0.8330	0.0301
1.0880	1.0268	0.7957	0.9440	0.6027
1.1118	1.0173	0.8123	0.9150	0.2738
1.1195	1.0358	0.8180	0.9250	0.1593
1.1188	1.0210	0.8183	0.9130	0.2309
1.1280	0.9880	0.8247	0.8760	0.0326
1.1493	0.9468	0.8400	0.8240	0.0077
1.1943	0.9478	0.8727	0.7940	0.0653
1.1000	1.0005	0.8030	0.9100	0.2978
1.1060	0.9895	0.8070	0.8950	0.3906
1.1080	1.0225	0.8090	0.9230	0.2226
1.1105	1.0333	0.8110	0.9300	0.3183
1.1243	1.0058	0.8203	0.8950	0.0551
1.1350	1.0013	0.8287	0.8820	0.1292
1.1660	0.9105	0.8510	0.7810	0.2193
1.3785	0.5830	1.0065	0.4230	0.0187
1.1013	1.0443	0.8030	0.9480	0.5716
1.1608	0.9438	0.8463	0.8130	0.0614
1.2353	0.8523	0.9010	0.6900	0.0137
1.3450	0.6985	0.9805	0.5190	0.9687
1.0845	1.0235	0.7900	0.9440	0.4486
1.1173	1.0393	0.8133	0.9300	0.2839
1.1255	0.9670	0.8190	0.8590	0.0571
1.1318	1.0198	0.8237	0.9010	0.0484
1.1320	0.9880	0.8243	0.8730	0.0769
1.1583	0.9365	0.8427	0.8090	0.0031
1.1783	0.9468	0.8573	0.8040	0.0185

1.2093	0.8608	0.8803	0.7120	0.0119
1.0878	1.0648	0.7903	0.9790	0.7378
1.1048	1.0380	0.8037	0.9400	0.1700
1.1178	1.0078	0.8123	0.9020	0.3948
1.1385	0.9895	0.8277	0.8690	0.0570
1.1405	1.0105	0.8293	0.8860	0.0618
1.1483	0.9713	0.8353	0.8460	0.2318
1.0545	1.1485	0.7657	1.0890	0.4172
1.1100	1.0230	0.8053	0.9220	0.1979
1.1103	1.0305	0.8063	0.9280	0.1658
1.1168	1.0260	0.8107	0.9190	0.3701
1.1210	1.0218	0.8140	0.9110	0.3551
1.1423	0.9863	0.8290	0.8630	0.1526
1.1615	0.9285	0.8430	0.7990	0.2447
1.1045	0.9970	0.8010	0.9030	0.3345
1.1268	1.0138	0.8170	0.9000	0.0849
1.1385	1.0063	0.8250	0.8840	0.1624
1.1405	0.9793	0.8263	0.8590	0.0685
1.3353	0.6775	0.9683	0.5070	0.1551
1.0905	1.0040	0.7900	0.9210	0.1427
1.1368	1.0008	0.8227	0.8800	0.2265
1.1630	0.9995	0.8420	0.8590	0.3767
1.1660	0.9940	0.8440	0.8520	0.1892
1.3120	0.7050	0.9500	0.5370	0.0308
1.0933	1.0760	0.7903	0.9840	0.6553
1.1250	1.0203	0.8137	0.9070	0.1117
1.1340	1.0013	0.8197	0.8830	0.0908
1.1490	0.9688	0.8310	0.8430	0.1069
1.1648	0.9615	0.8417	0.8250	0.1560
1.2965	0.7440	0.9375	0.5740	0.0899
1.0755	1.0395	0.7760	0.9670	0.6139
1.0815	1.0738	0.7810	0.9930	0.9397
1.0970	1.0740	0.7923	0.9790	0.9291

1.0998	1.0318	0.7940	0.9380	0.3416
1.1020	0.9985	0.7960	0.9060	0.4739
1.1128	1.0070	0.8037	0.9050	0.2602
1.1165	1.0308	0.8060	0.9230	0.3275
1.1575	0.9658	0.8357	0.8340	0.1050
1.2150	0.9055	0.8770	0.7450	0.1347
1.1103	1.0438	0.8003	0.9400	0.2072
1.1210	1.0095	0.8087	0.9010	0.2557
1.1238	1.0053	0.8107	0.8950	0.2006
1.1485	0.9770	0.8277	0.8510	0.2156
1.1560	0.9190	0.8340	0.7950	0.4423
1.1718	0.9548	0.8443	0.8150	0.0128
1.1698	0.9405	0.8423	0.8040	0.0580
1.0880	1.1430	0.7825	1.0510	0.4413
1.1413	0.9723	0.8207	0.8520	0.1230
1.1495	0.9808	0.8270	0.8530	0.0408
1.1683	0.9520	0.8400	0.8150	0.0285
1.2035	0.9220	0.8650	0.7660	0.7031
1.2253	0.8563	0.8807	0.6990	0.0501
1.3205	0.7093	0.9493	0.5370	0.0306
1.1033	1.0545	0.7920	0.9560	0.6363
1.1665	0.9568	0.8370	0.8200	0.1213
1.1738	0.9388	0.8427	0.8000	0.3160
1.0230	1.0900	0.7340	1.0650	0.4100
1.0450	1.1363	0.7490	1.0870	0.7665
1.1055	1.0710	0.7927	0.9690	0.6964
1.2220	0.8905	0.8765	0.7290	0.4105
1.2410	0.8660	0.8880	0.6980	0.0222
1.2505	0.8465	0.8950	0.6770	0.1361
1.0855	1.0678	0.7757	0.9840	0.8627
1.1330	0.9978	0.8103	0.8810	0.1162
1.1355	0.9845	0.8123	0.8670	0.0504
1.1595	0.9698	0.8293	0.8360	0.0697

1.1720	0.9895	0.8375	0.8440	0.4108
1.3430	0.6670	0.9600	0.4970	0.0414
1.1195	0.9830	0.7990	0.8780	0.0042
1.1370	1.0058	0.8110	0.8850	0.0416
1.1648	1.0055	0.8310	0.8630	0.0187
1.1723	0.9463	0.8357	0.8070	0.0163
1.0528	1.1768	0.7497	1.1180	0.3496
1.1310	1.0145	0.8057	0.8970	0.0376
1.0475	1.1540	0.7443	1.1020	0.2063
1.0508	1.1303	0.7473	1.0760	0.4882
1.0860	1.0300	0.7720	0.9480	0.4994
1.1520	1.0245	0.8193	0.8890	0.1772
1.1725	0.9970	0.8340	0.8500	0.2111
1.0865	1.0295	0.7710	0.9480	0.1835
1.1083	1.0560	0.7870	0.9530	0.4935
1.1188	1.0433	0.7940	0.9330	0.1923
1.1320	1.0720	0.8040	0.9470	0.7769
1.1548	0.9845	0.8197	0.8530	0.0048
1.1680	0.9175	0.8290	0.7860	0.0372
1.2095	0.8510	0.8583	0.7040	0.0271
1.0995	1.0405	0.7790	0.9460	0.4070
1.1293	1.0065	0.8003	0.8910	0.0821
1.1683	0.9755	0.8280	0.8350	0.5001
1.1673	0.9648	0.8280	0.8270	0.0054
1.0093	1.2235	0.7143	1.2120	0.4881
1.1078	1.0530	0.7847	0.9510	0.3939
1.1198	1.0100	0.7930	0.9020	0.0489
1.1453	0.9978	0.8110	0.8710	0.1012
1.1638	0.9685	0.8237	0.8320	0.2217
1.1710	0.9573	0.8287	0.8170	0.2932
1.2110	0.9590	0.8577	0.7920	0.0612
1.0130	1.1355	0.7160	1.1210	0.1213
1.1248	0.9858	0.7950	0.8760	0.1729

1.1343	1.0080	0.8020	0.8890	0.0829
1.1615	0.9555	0.8210	0.8230	0.0821
1.1585	0.9775	0.8173	0.8440	0.0299
1.1810	0.8920	0.8340	0.7550	0.4360
1.1678	0.8933	0.8230	0.7650	0.0118
1.0955	1.0195	0.7710	0.9310	0.1150
1.1115	1.0548	0.7830	0.9490	0.3927
1.1140	1.0580	0.7847	0.9500	0.2493
1.1210	1.0190	0.7887	0.9090	0.2418
1.1783	0.9473	0.8290	0.8040	0.0471
1.1835	0.9870	0.8335	0.8340	0.4370
1.1675	0.9770	0.8213	0.8370	0.0885
1.1423	0.9648	0.8020	0.8450	0.2192
1.1580	0.9778	0.8127	0.8440	0.0264
1.1950	0.9665	0.8385	0.8090	0.0643
1.0720	1.0540	0.7520	0.9830	0.8911
1.1175	1.0445	0.7837	0.9350	0.2496
1.1225	1.0553	0.7867	0.9400	0.5390
1.1458	0.9960	0.8030	0.8690	0.2987
1.1620	1.0058	0.8140	0.8660	0.1246
1.2100	0.8975	0.8487	0.7420	0.0389
1.0913	1.0928	0.7637	1.0010	0.9488
1.1735	0.9095	0.8220	0.7750	0.1945
1.1930	0.9045	0.8350	0.7580	0.0253
1.0450	1.0890	0.7300	1.0420	0.8476
1.1885	0.8895	0.8310	0.7480	0.3038
1.2315	0.8300	0.8610	0.6740	0.1183
1.3085	0.7655	0.9145	0.5850	0.0009
1.1258	1.0423	0.7853	0.9260	0.1125
1.1303	1.0330	0.7893	0.9140	0.2398
1.1575	1.0008	0.8077	0.8650	0.0653
1.1635	0.9770	0.8123	0.8400	0.1233
1.1768	0.9540	0.8217	0.8110	0.0340

1.2385	0.8265	0.8650	0.6670	0.0001
1.1265	1.0153	0.7857	0.9010	0.1428
1.1405	1.0153	0.7947	0.8900	0.1513
1.1763	0.8720	0.8197	0.7410	0.0037
1.0768	1.1003	0.7493	1.0220	0.7602
1.1955	0.8840	0.8320	0.7390	0.1579
1.2575	0.8160	0.8750	0.6490	0.0452
1.1090	1.0398	0.7707	0.9380	0.2298
1.1403	0.9945	0.7927	0.8720	0.0680
1.1840	0.9040	0.8230	0.7640	0.1349
1.1865	0.9458	0.8243	0.7970	0.0788
1.2090	0.8685	0.8400	0.7180	0.0362
1.1175	0.9955	0.7750	0.8910	0.2694
1.1805	0.9225	0.8190	0.7810	0.0090
1.1945	0.8915	0.8290	0.7460	0.0465
1.2160	0.8565	0.8440	0.7040	0.1060
1.1200	1.0493	0.7760	0.9370	0.3955
1.1363	1.0063	0.7870	0.8860	0.1371
1.1360	1.0113	0.7873	0.8900	0.0794
1.1450	1.0505	0.7930	0.9170	0.4294
1.1468	1.0113	0.7950	0.8820	0.2670
1.1465	0.9518	0.7950	0.8300	0.0373
1.1910	0.9393	0.8243	0.7890	0.0664
1.3040	0.7370	0.9020	0.5650	0.0482
1.1960	0.9820	0.8260	0.8210	0.3607
1.2085	0.8675	0.8350	0.7180	0.1175
1.2265	0.8460	0.8470	0.6900	0.0266
1.0615	1.0605	0.7320	0.9990	0.9418
1.1130	1.0630	0.7677	0.9550	0.5781
1.1370	0.9695	0.7850	0.8530	0.0268
1.2280	0.9235	0.8475	0.7520	0.2597
1.2910	0.7813	0.8913	0.6050	0.0066
1.3430	0.7255	0.9260	0.5400	0.0454

1.1403	1.0300	0.7853	0.9030	0.1941
1.2230	0.8855	0.8430	0.7240	0.0904
1.2293	0.8770	0.8467	0.7130	0.0511
1.3740	0.6533	0.9460	0.4750	0.0208
1.1010	1.0230	0.7560	0.9290	0.5328
1.1270	1.0383	0.7740	0.9210	0.3164
1.1465	1.0118	0.7873	0.8820	0.1425
1.1513	1.0080	0.7910	0.8760	0.0808
1.1523	0.9930	0.7917	0.8620	0.0429
1.1835	0.9145	0.8130	0.7730	0.2476
1.2500	0.8283	0.8590	0.6630	0.0216
1.3645	0.6560	0.9370	0.4810	0.0392
1.1498	1.0205	0.7890	0.8880	0.1964
1.1678	0.9855	0.8007	0.8440	0.0782
1.2605	0.8040	0.8650	0.6380	0.0411
1.1458	1.0125	0.7850	0.8840	0.0381
1.2230	0.8898	0.8383	0.7280	0.1387
1.2615	0.8775	0.8640	0.6960	0.3273
1.3550	0.6700	0.9280	0.4940	0.0361
1.1640	1.0320	0.7965	0.8870	0.2380
0.9485	1.2520	0.6480	1.3200	0.4643
1.1340	0.9795	0.7750	0.8640	0.0342
1.1410	1.0418	0.7790	0.9130	0.3439
1.1468	1.0115	0.7830	0.8820	0.1642
1.1665	0.9775	0.7967	0.8380	0.0534
1.1970	0.8905	0.8180	0.7440	0.1677
1.0093	1.2535	0.6880	1.2420	0.6984
1.0910	1.0410	0.7440	0.9540	0.5453
1.1528	0.9825	0.7857	0.8520	0.1193
1.1700	0.9785	0.7980	0.8360	0.0629
1.1998	0.9365	0.8177	0.7810	0.1563
1.2360	0.8415	0.8430	0.6810	0.0635
1.2705	0.7993	0.8660	0.6290	0.0222

1.1675	0.8995	0.7950	0.7700	0.0125
1.2210	0.9830	0.8310	0.8050	0.1407
1.1288	1.0333	0.7677	0.9150	0.4465
1.1475	1.0143	0.7807	0.8840	0.1798
1.1948	0.9403	0.8123	0.7870	0.0906
1.2300	0.9390	0.8370	0.7630	0.3514
1.1405	1.0335	0.7747	0.9060	0.2546
1.1425	1.0295	0.7760	0.9010	0.1291
1.1858	0.9620	0.8050	0.8110	0.0330
1.0970	1.0325	0.7440	0.9410	0.5785
1.1660	0.9908	0.7897	0.8500	0.1302
1.1783	0.9848	0.7970	0.8360	0.0137
1.1338	1.0433	0.7653	0.9200	0.2503
1.1415	1.0305	0.7703	0.9030	0.2470
1.2003	0.9490	0.8100	0.7910	0.0114
1.1190	1.0698	0.7547	0.9560	0.5068
1.3280	0.7575	0.8955	0.5700	0.0098
1.3715	0.6975	0.9240	0.5090	0.0661
1.1550	1.0123	0.7760	0.8760	0.1389
1.1130	1.0660	0.7467	0.9580	0.4449
1.1398	1.0420	0.7640	0.9140	0.2335
1.1595	0.9505	0.7770	0.8200	0.0094
1.1283	1.0483	0.7547	0.9290	0.3726
1.1555	1.0275	0.7727	0.8890	0.3716
1.2398	0.8753	0.8290	0.7060	0.1677
1.4013	0.6533	0.9380	0.4660	0.1255
1.2125	0.9033	0.8100	0.7450	0.0070
1.2185	0.8998	0.8140	0.7380	0.0260
1.0850	1.0595	0.7240	0.9760	0.8267
1.1110	1.0175	0.7410	0.9160	0.4526
1.1605	0.9948	0.7737	0.8570	0.0907
1.2033	0.9390	0.8030	0.7800	0.1463
1.3635	0.7095	0.9090	0.5200	0.0243

1.1760	0.9965	0.7833	0.8470	0.0921
1.2055	0.9385	0.8023	0.7790	0.0324
1.2630	0.8110	0.8410	0.6420	0.0141
1.1465	0.9705	0.7620	0.8460	0.0488
1.2280	0.9540	0.8170	0.7770	0.1822
1.2395	0.9295	0.8245	0.7500	0.1802
1.1725	0.9843	0.7783	0.8390	0.0089
1.1908	0.9613	0.7910	0.8070	0.0792
1.2335	0.9380	0.8195	0.7600	0.3808
1.1555	1.0150	0.7663	0.8780	0.0803
1.1795	0.9260	0.7820	0.7850	0.0690
1.2375	0.8605	0.8200	0.6950	0.3757
1.3370	0.6900	0.8867	0.5160	0.0179
1.0995	1.0315	0.7280	0.9380	0.7263
1.1150	1.0888	0.7387	0.9760	0.8083
1.2925	0.8033	0.8557	0.6210	0.0029
1.0885	1.0365	0.7200	0.9520	0.3133
1.0960	1.0440	0.7240	0.9530	0.4191
1.2275	0.9580	0.8110	0.7800	0.0863
1.3105	0.7445	0.8660	0.5680	0.0964
1.3268	0.7535	0.8773	0.5680	0.0086
1.3810	0.6835	0.9130	0.4950	0.0820
1.0910	1.1318	0.7197	1.0370	0.7259
1.1968	0.9563	0.7893	0.7990	0.0268
1.2520	0.8285	0.8260	0.6620	0.0329
1.3780	0.6745	0.9080	0.4890	0.0242
1.1635	0.9953	0.7653	0.8550	0.1332
1.2985	0.7635	0.8540	0.5880	0.1001
1.3463	0.7223	0.8857	0.5360	0.0014
1.1875	0.9723	0.7790	0.8190	0.1304
1.2125	1.0510	0.7955	0.8670	0.1979
1.2965	0.8370	0.8500	0.6460	0.1420
1.3158	0.7723	0.8637	0.5870	0.0370

1.1498	1.0345	0.7533	0.9000	0.2594
1.0520	1.0705	0.6880	1.0180	0.5616
1.2255	0.9725	0.8015	0.7940	0.0389
1.2540	0.7800	0.8200	0.6220	0.0957
1.0285	1.1345	0.6720	1.1030	0.6199
1.1220	1.0065	0.7300	0.8970	0.5985
1.3098	0.7858	0.8527	0.6000	0.0275
0.9883	1.2358	0.6420	1.2500	0.3742
1.1540	1.0420	0.7500	0.9030	0.2375
1.2133	0.9383	0.7887	0.7730	0.0209
1.2200	0.8765	0.7930	0.7180	0.1346
1.2500	0.8380	0.8120	0.6700	0.0118
1.3570	0.6563	0.8820	0.4840	0.0006
1.1540	1.1015	0.7495	0.9550	0.7278
1.3338	0.7510	0.8657	0.5630	0.0043
1.0515	1.2013	0.6813	1.1420	0.3880
1.1165	1.0200	0.7230	0.9140	0.3954
1.1430	0.9825	0.7410	0.8600	0.2323
1.2568	0.8405	0.8140	0.6690	0.0609
1.3215	0.7825	0.8563	0.5920	0.0015
1.1300	1.0050	0.7310	0.8890	0.1680
1.1945	0.9095	0.7720	0.7610	0.2854
1.2495	0.8350	0.8050	0.6680	0.2294
1.1538	1.0440	0.7420	0.9050	0.2675
1.2210	0.9283	0.7847	0.7600	0.0917
1.2305	0.9160	0.7913	0.7440	0.0690
1.2538	0.8855	0.8057	0.7060	0.0043
1.4073	0.5338	0.9050	0.3790	0.0026
1.1320	1.0760	0.7270	0.9510	0.3607
1.1363	1.0638	0.7300	0.9360	0.5883
1.2720	0.8125	0.8160	0.6390	0.1110
1.0165	1.1685	0.6520	1.1500	0.5714
1.3070	0.8460	0.8375	0.6470	0.1751

1.4430	0.6055	0.9255	0.4200	0.0084
1.3915	0.7173	0.8900	0.5150	0.0045
1.1433	1.0643	0.7307	0.9310	0.6916
1.3540	0.7008	0.8657	0.5180	0.0066
1.1920	0.9678	0.7597	0.8120	0.0412
1.1128	1.0898	0.7080	0.9790	0.7756
1.1655	0.9625	0.7410	0.8260	0.0130
1.2215	0.8850	0.7770	0.7250	0.1914
1.2570	0.9325	0.7995	0.7420	0.1937
1.1923	0.9860	0.7573	0.8270	0.2176
1.2520	0.9003	0.7930	0.7190	0.0367
1.2285	0.8795	0.7760	0.7160	0.0343
1.3460	0.7105	0.8510	0.5280	0.1403
0.8665	1.3690	0.5460	1.5800	0.2779
1.2068	1.0005	0.7607	0.8290	0.0315
1.0775	1.0845	0.6780	1.0060	0.9258
1.1330	1.0135	0.7080	0.8950	0.6416
1.2083	0.9690	0.7547	0.8020	0.1228
1.2325	0.9238	0.7697	0.7490	0.0689
1.3095	0.8685	0.8175	0.6630	0.2317
1.3590	0.7785	0.8475	0.5730	0.0268
1.3033	0.8255	0.8123	0.6330	0.0028
1.1238	1.0993	0.6990	0.9780	0.7308
1.0415	1.1410	0.6470	1.0960	0.4614
1.2110	1.0330	0.7525	0.8530	0.4037
1.2715	0.9295	0.7890	0.7310	0.2517
1.4310	0.6235	0.8887	0.4360	0.0042
1.3210	0.8450	0.8195	0.6400	0.1340
1.1508	1.0665	0.7080	0.9270	0.5313
1.2508	0.9198	0.7690	0.7350	0.0303
1.6205	0.3495	0.9970	0.2160	0.0853
1.1180	1.1175	0.6863	1.0000	0.9654
1.3030	0.8370	0.8003	0.6420	0.0036

1.3355	0.7365	0.8200	0.5510	0.3389
1.3670	0.7363	0.8397	0.5390	0.0319
1.3945	0.6958	0.8557	0.4990	0.0010
1.2885	0.8075	0.7900	0.6270	0.0576
1.3600	0.7460	0.8343	0.5490	0.0017
1.4738	0.5978	0.9017	0.4060	0.0060
1.1250	1.0965	0.6867	0.9750	0.9271
1.2440	0.9270	0.7587	0.7450	0.0830
1.6690	0.2515	1.0180	0.1510	0.0021
1.2765	0.8200	0.7770	0.6420	0.2262
1.0925	1.1623	0.6637	1.0640	0.2942
1.3045	0.8360	0.7903	0.6410	0.0095
1.0935	1.0730	0.6620	0.9810	0.8513
1.1685	1.0270	0.7050	0.8790	0.5805
1.2453	0.9370	0.7513	0.7520	0.0043
1.3770	0.7075	0.8307	0.5140	0.0017
1.2650	0.9660	0.7615	0.7640	0.3632
1.1570	1.0773	0.6957	0.9310	0.6331
1.2600	0.9105	0.7577	0.7230	0.0095
1.1490	1.0500	0.6890	0.9140	0.3287
1.2605	0.8565	0.7560	0.6790	0.5263
1.1843	1.0260	0.7093	0.8660	0.3760
1.3288	0.8048	0.7947	0.6060	0.0418
1.2020	1.0013	0.7170	0.8330	0.0992
1.2820	0.8495	0.7620	0.6630	0.0229
1.1903	1.0283	0.7043	0.8640	0.3566
1.2453	0.9318	0.7373	0.7480	0.1126
1.4283	0.6593	0.8460	0.4620	0.0145
1.2183	0.9900	0.7197	0.8130	0.1086
1.2973	0.8608	0.7667	0.6640	0.0231
1.3305	0.8128	0.7860	0.6110	0.0315
1.2410	1.0050	0.7327	0.8100	0.1578
1.4490	0.6885	0.8555	0.4750	0.1249

1.2495	0.9260	0.7340	0.7410	0.0240
1.3265	0.8280	0.7777	0.6240	0.0015
1.1090	1.0610	0.6490	0.9570	0.7571
1.1925	1.0328	0.6973	0.8660	0.3512
1.2200	0.9095	0.7120	0.7450	0.6354
1.2795	0.8330	0.7460	0.6510	0.2733
1.1495	1.0130	0.6680	0.8810	0.2480
1.2820	0.8760	0.7447	0.6830	0.1009
1.3873	0.7318	0.8050	0.5270	0.0168
1.1725	0.9885	0.6790	0.8430	0.2560
1.3033	0.8655	0.7540	0.6640	0.0262
1.3858	0.6563	0.8023	0.4740	0.0044
1.3020	0.8045	0.7520	0.6180	0.2573
1.2130	1.0100	0.6993	0.8330	0.0547
1.2043	1.0375	0.6937	0.8620	0.1543
1.4828	0.6005	0.8503	0.4050	0.0376
1.2930	0.9515	0.7380	0.7360	0.4775
1.3190	0.8340	0.7537	0.6320	0.0076
1.4335	0.6790	0.8177	0.4740	0.0027
1.4615	0.5858	0.8330	0.4010	0.0022
1.3765	0.7760	0.7757	0.5640	0.0037
1.3325	0.8150	0.7480	0.6120	0.0027
1.3760	0.7438	0.7720	0.5410	0.0044
1.2478	0.9290	0.6953	0.7450	0.0053
1.2680	0.9503	0.7053	0.7490	0.3476
1.3805	0.7720	0.7643	0.5590	0.0171
1.3375	0.8323	0.7400	0.6220	0.0099
1.3853	0.7728	0.7643	0.5580	0.0075
1.2355	0.9145	0.6780	0.7400	0.5433
1.3468	0.8338	0.7360	0.6190	0.0067
1.5063	0.6005	0.8217	0.3990	0.0026
1.1813	1.0918	0.6440	0.9240	0.4777
1.4818	0.6250	0.8073	0.4220	0.0033

1.4800	0.6850	0.8020	0.4630	0.0226
1.3255	0.8708	0.7163	0.6570	0.0664
1.4420	0.6455	0.7790	0.4480	0.2097
1.3360	0.7885	0.7200	0.5900	0.1074
1.4085	0.6885	0.7560	0.4890	0.1863
1.4678	0.5983	0.7887	0.4080	0.0157
1.3800	0.7280	0.7390	0.5280	0.3333
1.3423	0.8070	0.7173	0.6010	0.0102
1.3105	0.9068	0.6987	0.6920	0.1601
1.4445	0.7030	0.7697	0.4870	0.0026
1.4840	0.6940	0.7910	0.4680	0.0012
1.2810	0.8805	0.6810	0.6870	0.4329
1.3938	0.7815	0.7387	0.5610	0.0011
1.1095	1.0955	0.5860	0.9870	0.5505
1.4090	0.6990	0.7380	0.4960	0.1833
1.4420	0.8395	0.7555	0.5820	0.3387
1.4733	0.6543	0.7717	0.4440	0.0020
1.4595	0.6385	0.7630	0.4370	0.0387
1.3470	0.7840	0.7020	0.5820	0.1440
1.5250	0.6465	0.7945	0.4240	0.1130
1.5425	0.5875	0.8033	0.3810	0.0009
1.3863	0.8040	0.7190	0.5800	0.0043
1.3415	0.8765	0.6917	0.6530	0.0452
1.5235	0.5550	0.7860	0.3640	0.1103
1.2750	0.8895	0.6560	0.6980	0.0077
1.4350	0.6740	0.7350	0.4700	0.1263
1.4400	0.6843	0.7303	0.4750	0.0016
1.3960	0.8000	0.7063	0.5730	0.1959
1.3630	0.7940	0.6870	0.5830	0.4600
1.6540	0.4153	0.8340	0.2510	0.0047
1.4255	0.6905	0.7160	0.4840	0.3109
1.4795	0.6860	0.7430	0.4640	0.0007
1.1395	1.0735	0.5710	0.9420	0.4846

1.4145	0.7858	0.7033	0.5550	0.0069
1.5270	0.6820	0.7555	0.4470	0.0375
1.3790	0.7615	0.6800	0.5520	0.1618
1.5748	0.5525	0.7760	0.3510	0.0020
1.3903	0.8425	0.6847	0.6060	0.0092
0.9185	1.3630	0.4500	1.4840	0.2335
1.2325	0.9560	0.5950	0.7760	0.3307
1.3163	0.9483	0.6363	0.7200	0.0595
1.2495	0.9420	0.5960	0.7540	0.0689
1.1735	1.1393	0.5573	0.9710	0.9074
1.2955	0.8840	0.6070	0.6820	0.2326
1.4195	0.8860	0.6620	0.6240	0.2308
1.1130	1.1245	0.5170	1.0100	0.4732
1.3293	0.9425	0.6180	0.7090	0.1111
1.2893	1.0063	0.5983	0.7800	0.1413
1.4505	0.6985	0.6660	0.4820	0.0190
1.2420	1.0895	0.5690	0.8770	0.5518
1.4730	0.6715	0.6740	0.4560	0.0268
1.4808	0.7343	0.6723	0.4960	0.0041
1.5533	0.6400	0.6983	0.4120	0.0002
1.6165	0.6070	0.7280	0.3760	0.0818
1.6880	0.5185	0.7465	0.3070	0.0315
1.5415	0.6608	0.6757	0.4290	0.4202
1.5840	0.5918	0.6910	0.3740	0.0003
1.5355	0.5955	0.6400	0.3880	0.0768
1.2990	0.9095	0.5410	0.7000	0.4104
1.3305	0.9545	0.5513	0.7170	0.8567
1.6250	0.5173	0.6723	0.3180	0.0197
1.3895	0.8065	0.5640	0.5800	0.2123
1.8585	0.3380	0.7340	0.1820	0.0381
1.5490	0.7990	0.6090	0.5160	0.2047
1.7165	0.5350	0.6660	0.3120	0.0139
1.6585	0.4975	0.6390	0.3000	0.1892

1.3955	1.0820	0.5340	0.7750	0.9305
1.0460	1.2655	0.3990	1.2100	0.6125
1.6800	0.4575	0.6370	0.2720	0.1459
1.4975	0.7030	0.5480	0.4690	0.1361
1.7420	0.4845	0.6243	0.2780	0.0069
1.6335	0.5345	0.5780	0.3270	0.1807
1.7255	0.5085	0.6107	0.2950	0.0059
1.2865	0.9715	0.4060	0.7550	0.3074
1.6318	0.6605	0.5070	0.4050	0.0036
1.7638	0.4810	0.5340	0.2730	0.0045
1.8003	0.4825	0.5433	0.2680	0.0061
1.7135	0.4825	0.5070	0.2820	0.2181
1.9260	0.4120	0.5705	0.2140	0.0533
1.9018	0.4143	0.4780	0.2180	0.0072
1.9655	0.4585	0.4380	0.2330	0.0701
1.4250	1.1845	0.3170	0.8310	0.9660

HS-II/Con Ratio	HS-II/Con P value	HS-II/HS-I Ratio	HS-II/HS-I P value
6.1050	0.1612	2.7140	0.4307
5.3520	0.2928	4.1940	0.3695
4.4360	0.3726	4.1520	0.3964
3.2370	0.0070	2.1760	0.0049
2.9430	0.0275	2.6950	0.0520
2.6850	0.0001	2.4260	0.0154
2.6370	0.0307	2.2910	0.0673
2.5450	0.0876	2.5200	0.0923
2.4800	0.5014	2.1750	0.6319
2.3410	0.1014	2.2800	0.1009
2.2500	0.0056	1.8430	0.0501
2.2100		1.6100	
2.1540	0.0118	2.1150	0.0006
2.0630	0.0703	1.3180	0.1832
2.0430	0.4006	1.7000	0.5486
2.0270	0.0724	1.5180	0.1849
1.9560	0.0225	1.5860	0.0839
1.9510	0.0013	1.0730	0.2454
1.9360		0.9450	
1.9210	0.2765	2.0300	0.2468
1.8930		1.0460	
1.8850	0.3884	0.8090	0.5906
1.8730		2.2880	
1.8350		1.9440	0.0066
1.8060		0.7190	
1.7640	0.0844	1.7550	0.0705
1.7410	0.0497	1.3480	0.3570
1.7220	0.1642	1.9290	0.0901

1.7200	0.0013	1.5890	0.0101
1.7130	0.2442	0.8280	0.5285
1.7110	0.5482	0.8220	0.6236
1.7030	0.0000	1.3800	0.0233
1.7010	0.0012	1.2990	0.0238
1.7000	0.4578	1.5130	0.5352
1.6950	0.0351	0.8280	0.4537
1.6770		1.6190	
1.6620	0.0224	1.4850	0.0198
1.6550	0.0631	1.2060	0.3328
1.6520	0.0094	1.3750	0.0396
1.6470	0.0131	1.2780	0.0658
1.6410		1.1680	
1.6350	0.1098	1.4890	0.2025
1.6330	0.0266	1.2910	0.2337
1.6260		1.4390	
1.6130	0.0129	1.2780	0.0562
1.6080	0.0004	1.2390	0.0380
1.5990	0.1359	1.0340	0.7130
1.5940		1.2920	
1.5930	0.0479	1.3640	0.0213
1.5830	0.0992	1.3040	0.1734
1.5810	0.0051	1.5380	0.0005
1.5760	0.4579	1.3730	0.6115
1.5760		1.2310	
1.5700	0.0014	0.8340	0.3139
1.5590	0.0010	1.5680	0.0033
1.5550	0.0245	1.5730	0.0327
1.5520	0.0038	1.2730	0.0172
1.5520	0.0113	1.5880	0.0099
1.5500	0.0029	1.4270	0.0164
1.5390	0.0141	1.2050	0.0644
1.5380	0.1860	1.5920	0.2294

1.5370	0.0362	1.0690	0.6334
1.5360	0.0020	1.3670	0.0092
1.5330		1.2860	
1.5290		1.1810	
1.5290	0.0065	1.2180	0.0683
1.5290	0.0389	1.4900	0.0654
1.5240	0.0042	0.8670	0.0865
1.5230	0.0005	1.3310	0.0045
1.5220	0.0019	1.3020	0.0064
1.5180	0.0112	1.3840	0.0342
1.5170	0.0410	1.1960	0.3481
1.5150	0.0023	1.1990	0.1323
1.5150	0.0079	1.3350	0.0317
1.5120	0.1376	1.3830	0.2417
1.5120		1.3400	
1.5120	0.0169	1.5290	0.0136
1.5100	0.0016	1.1810	0.0178
1.5090	0.1440	1.4250	0.1060
1.5080	0.0053	0.8820	0.1680
1.5060	0.2124	0.8330	0.5391
1.5030	0.0440	1.4560	0.1359
1.5020	0.0001	1.1070	0.0668
1.5010		1.4450	
1.4970	0.3076	1.2580	0.5945
1.4970	0.0042	1.3980	0.0150
1.4930	0.0065	1.2480	0.0844
1.4930	0.0012	1.4010	0.0018
1.4920	0.0020	1.2030	0.0709
1.4920		1.2780	
1.4900	0.0026	1.6600	0.0042
1.4860	0.0002	1.1300	0.0484
1.4800		1.2330	
1.4790	0.0056	1.2880	0.0399

1.4750	0.3125	0.9280	0.8726
1.4750	0.0050	1.2620	0.0547
1.4740	0.0095	1.6330	0.0064
1.4730	0.0386	1.5100	0.0723
1.4670	0.0843	1.2970	0.1585
1.4640		1.3130	
1.4610	0.0077	1.7080	0.0175
1.4530	0.0149	1.2590	0.0746
1.4510		0.8350	
1.4470		1.4010	
1.4450	0.0020	1.1830	0.0479
1.4440		0.9890	
1.4440		1.2150	
1.4430	0.0042	1.3980	0.0012
1.4400	0.0723	1.0510	0.1674
1.4390	0.2451	0.8240	0.8967
1.4370	0.0160	1.3620	0.0365
1.4350	0.0712	1.1610	0.2189
1.4330	0.0192	1.2840	0.0451
1.4330	0.0211	1.4280	0.0074
1.4310	0.0002	1.3180	0.0022
1.4300		1.4630	
1.4250	0.0002	1.2810	0.0022
1.4250		1.3770	
1.4250	0.1361	1.7400	0.0313
1.4240	0.0031	1.1790	0.0901
1.4230	0.0060	1.2710	0.0383
1.4210	0.0625	1.1280	0.3678
1.4210	0.0036	1.1620	0.1066
1.4140	0.0021	1.3680	0.0073
1.4120	0.0058	1.0920	0.4841
1.4120	0.0031	1.2630	0.0015
1.4120	0.0151	1.2740	0.1262

1.4110		1.2700	
1.4110	0.1087	1.4160	0.0204
1.4090	0.0023	1.1860	0.1009
1.4090	0.0002	1.2940	0.0079
1.4080	0.0523	1.2560	0.2415
1.4030		1.2660	
1.4020	0.0666	1.1710	0.3470
1.4020	0.1113	1.3170	0.0713
1.4010	0.1494	0.9560	0.9071
1.4010	0.0060	1.2630	0.0027
1.4010	0.0100	1.2560	0.0689
1.4010	0.0002	1.2670	0.0016
1.4000	0.0012	1.1000	0.1572
1.4000	0.0430	1.2000	0.2125
1.4000	0.0007	1.3000	0.0090
1.4000	0.0026	1.3660	0.0011
1.3990	0.0015	1.2720	0.0058
1.3980	0.2368	0.9920	0.8785
1.3970	0.0222	1.2160	0.0307
1.3970	0.0048	1.4550	0.0009
1.3950	0.0009	1.3090	0.0144
1.3940	0.0003	1.3080	0.0015
1.3930	0.1653	0.9450	0.6619
1.3910		1.3930	
1.3890	0.0233	1.3080	0.0299
1.3880		1.1780	
1.3860	0.0525	1.4890	0.0167
1.3850	0.0007	1.1640	0.0402
1.3850	0.0135	1.2180	0.0026
1.3850	0.0782	1.3300	0.1216
1.3840	0.0030	1.2180	0.0082
1.3840		1.2310	
1.3830	0.0305	1.4700	0.0565

1.3810	0.0043	1.3010	0.0126
1.3800	0.0922	1.1740	0.1992
1.3800	0.0156	1.1760	0.2128
1.3800	0.0040	1.2850	0.0092
1.3790	0.0494	1.2340	0.2375
1.3790	0.0012	1.2280	0.0116
1.3780	0.0284	1.2800	0.0839
1.3770	0.0172	1.2250	0.0525
1.3760	0.0007	1.3030	0.0130
1.3750	0.0041	1.2140	0.0049
1.3750	0.0013	1.3130	0.0006
1.3750		1.2520	
1.3750		1.4970	
1.3740	0.0279	1.1830	0.1981
1.3740	0.0022	1.2110	0.0115
1.3740	0.0677	1.2810	0.1585
1.3730	0.0054	1.1950	0.0135
1.3730	0.0099	1.1980	0.0984
1.3710	0.0066	1.2560	0.0479
1.3710	0.0005	1.2860	0.0025
1.3700	0.0775	1.3020	0.0966
1.3690	0.0491	1.2810	0.0097
1.3670	0.0330	1.2270	0.1999
1.3660	0.0009	1.1790	0.0164
1.3650	0.0427	1.1880	0.1241
1.3650	0.0026	1.2770	0.0066
1.3640	0.0071	1.2580	0.0532
1.3640	0.0023	1.2020	0.0213
1.3640		1.4970	
1.3620	0.0125	1.1350	0.0546
1.3610	0.0369	1.0410	0.7757
1.3610	0.6145	1.3380	0.6531
1.3580	0.2284	0.9630	0.7991

1.3580	0.0411	1.1460	0.1541
1.3570		0.8850	
1.3570	0.0893	1.1520	0.3396
1.3560	0.0341	1.3460	0.0370
1.3550	0.0156	1.2510	0.0945
1.3540	0.0009	1.1770	0.0793
1.3540	0.0005	1.1750	0.0144
1.3530	0.0038	1.1130	0.2067
1.3530	0.0513	1.1900	0.0218
1.3520	0.0155	1.1460	0.0632
1.3520	0.0059	1.1930	0.0508
1.3520	0.0224	1.2120	0.0128
1.3510	0.0018	1.1210	0.0720
1.3500	0.0768	1.2770	0.2689
1.3490	0.0012	1.0760	0.6353
1.3490	0.0811	1.2640	0.1193
1.3490	0.0059	1.2410	0.0055
1.3490		1.3140	
1.3470		0.9110	
1.3470	0.0030	0.9480	0.2524
1.3470	0.0033	1.1740	0.1502
1.3470	0.0104	1.1990	0.0438
1.3460	0.0968	1.1310	0.4966
1.3450	0.0144	1.0840	0.2979
1.3450	0.0109	1.1670	0.0581
1.3450	0.0008	1.2340	0.0006
1.3440	0.0034	0.8890	0.0378
1.3440	0.0005	1.1630	0.0510
1.3430	0.0096	0.9690	0.8724
1.3430	0.0110	1.3130	0.0409
1.3420	0.0033	1.1640	0.0419
1.3400	0.0044	1.0340	0.4347
1.3400	0.0063	1.1740	0.1232

1.3400	0.2775	1.5950	0.1498
1.3390	0.0025	1.2100	0.0272
1.3390	0.0333	1.4740	0.0510
1.3380	0.0222	1.1670	0.0254
1.3380	0.0240	1.1700	0.2834
1.3330	0.0019	1.1390	0.1593
1.3330	0.0980	1.7640	0.0246
1.3300	0.0165	1.2750	0.0107
1.3290	0.0056	0.8940	0.0962
1.3290		1.0450	
1.3290	0.2910	1.2130	0.5335
1.3290	0.0021	1.1510	0.0331
1.3290	0.0002	1.2270	0.0036
1.3290	0.0499	1.3120	0.2065
1.3280	0.0037	1.1700	0.0241
1.3270	0.0148	1.0730	0.4376
1.3270	0.0008	1.2710	0.0062
1.3260	0.3366	0.9840	0.9182
1.3260	0.1410	1.0960	0.5245
1.3260	0.0094	1.2880	0.0065
1.3260		1.3060	
1.3250	0.2228	1.1680	0.5669
1.3250	0.0039	1.2640	0.0196
1.3240	0.0519	1.1760	0.3120
1.3240	0.0010	1.2130	0.0033
1.3230	0.0004	1.0650	0.1889
1.3230		1.1970	
1.3230		1.3380	
1.3220	0.0392	1.2770	0.0340
1.3210	0.0002	1.1040	0.1294
1.3210	0.0299	1.1170	0.2384
1.3210	0.0055	1.1650	0.0859
1.3210	0.0072	1.2430	0.0460

1.3200	0.0218	1.0050	0.9228
1.3200	0.0121	1.1160	0.2101
1.3200	0.0777	1.1720	0.1872
1.3190		1.2260	
1.3180	0.0033	1.4530	0.0008
1.3170	0.0244	1.2430	0.0563
1.3160	0.0511	1.1910	0.1423
1.3160	0.0891	1.2110	0.2132
1.3160		1.1730	
1.3150		1.3140	
1.3150	0.0375	1.4850	0.0262
1.3130	0.3659	0.6130	0.6216
1.3130	0.0048	1.1760	0.0564
1.3130	0.0088	1.1890	0.0636
1.3130		1.1660	
1.3130	0.0063	1.2450	0.0054
1.3120	0.0025	1.1110	0.1572
1.3120	0.0068	1.1710	0.0385
1.3120	0.2055	1.2290	0.3164
1.3120		1.1920	
1.3120	0.0041	1.3430	0.0098
1.3110	0.4433	1.1340	0.7577
1.3110	0.0126	1.2150	0.0450
1.3100	0.0231	1.0210	0.7192
1.3100	0.2667	1.0120	0.9348
1.3100	0.0066	1.1210	0.3003
1.3100	0.0063	1.1900	0.0742
1.3100	0.0085	1.2460	0.0362
1.3090		0.9600	
1.3090	0.0306	1.0800	0.2572
1.3090	0.0047	1.1910	0.0279
1.3090	0.0011	1.1970	0.0238
1.3090	0.0301	1.2210	0.0718

1.3070	0.0058	1.1280	0.1831
1.3060	0.0749	1.1740	0.1840
1.3060	0.0137	1.2410	0.0032
1.3060	0.0033	1.3090	0.0045
1.3060	0.2223	1.8500	0.0285
1.3050	0.2063	1.1920	0.3277
1.3050	0.0013	1.1870	0.0323
1.3030	0.1372	1.1720	0.2914
1.3020	0.0118	1.2280	0.0329
1.3020	0.2731	1.3850	0.1313
1.3010	0.0655	1.1690	0.1402
1.3010	0.0020	1.2040	0.0217
1.3010	0.0096	1.2530	0.0067
1.3010	0.0883	1.4380	0.0395
1.3000	0.0858	1.0560	0.7380
1.3000	0.0269	1.2130	0.0983
1.3000	0.0010	1.3050	0.0010
1.2990		1.1150	
1.2990	0.0070	1.2380	0.0075
1.2990	0.0104	1.2600	0.0713
1.2980	0.1561	0.9880	0.8993
1.2980	0.0029	1.0680	0.4288
1.2970	0.0632	1.2250	0.1683
1.2970	0.0000	1.2770	0.0017
1.2970		1.3040	
1.2960	0.0019	1.1400	0.0612
1.2960	0.0056	1.2220	0.0357
1.2950	0.0035	1.0830	0.2375
1.2950	0.0574	1.1340	0.2561
1.2950	0.0037	1.1460	0.1430
1.2940	0.0483	1.2030	0.1020
1.2940	0.0101	1.3090	0.0017
1.2930	0.0045	1.1530	0.0556

1.2930	0.0047	1.1710	0.0280
1.2930	0.0432	1.2180	0.1395
1.2930	0.0007	1.2730	0.0003
1.2920	0.0008	1.2000	0.0018
1.2910	0.0058	1.1210	0.1440
1.2910	0.0007	1.0960	0.0883
1.2910	0.0075	1.1810	0.0068
1.2910	0.0022	1.2450	0.0073
1.2910	0.2095	1.4920	0.0488
1.2900	0.0016	1.0870	0.1908
1.2900	0.0017	1.2060	0.0259
1.2900	0.0003	1.2100	0.0029
1.2900	0.0146	1.2520	0.1535
1.2900	0.0014	1.2130	0.0049
1.2900	0.2839	1.3200	0.2594
1.2890	0.1039	1.3100	0.0387
1.2890	0.0058	1.3320	0.0030
1.2890		1.2800	
1.2890		1.3190	
1.2880	0.0001	1.1000	0.1194
1.2880	0.1179	1.2780	0.0117
1.2870		1.1070	
1.2870	0.0014	1.1670	0.0945
1.2870	0.0260	1.2830	0.0318
1.2860	0.0118	1.1630	0.0506
1.2860	0.0077	1.2050	0.0175
1.2860	0.0008	1.2000	0.0062
1.2860	0.0004	1.2240	0.0008
1.2860	0.1320	1.7350	0.1576
1.2850	0.2063	0.9660	0.7826
1.2850	0.0029	1.0900	0.3025
1.2840	0.0012	1.0300	0.2872
1.2840	0.0192	1.1190	0.2512

1.2840	0.0068	1.1570	0.1294
1.2840		1.1430	
1.2830	0.0062	1.1350	0.0873
1.2830	0.0135	1.2560	0.0213
1.2830	0.2948	1.3240	0.2663
1.2820	0.0157	1.1420	0.3147
1.2820	0.0095	1.1530	0.0971
1.2820	0.0313	1.2550	0.0380
1.2820	0.1800	1.3300	0.0536
1.2810	0.0002	1.0530	0.1149
1.2800	0.0244	1.2030	0.0253
1.2800	0.0054	1.2900	0.0010
1.2790	0.1662	1.0480	0.8293
1.2790	0.0012	1.2330	0.0011
1.2790	0.0171	1.3270	0.0077
1.2790	0.6604	2.1580	0.0890
1.2780		1.0240	
1.2780	0.1547	1.1570	0.3178
1.2780	0.0267	1.1770	0.0344
1.2780	0.0099	1.2030	0.0272
1.2780	0.0055	1.3340	0.0009
1.2770	0.0252	1.1760	0.0793
1.2770	0.1312	1.2540	0.1420
1.2760	0.0023	1.1690	0.0478
1.2760	0.0018	1.2900	0.0034
1.2750	0.5482	1.3810	0.4079
1.2740	0.0015	1.1210	0.0891
1.2740	0.0535	1.1880	0.0619
1.2730	0.2349	1.0230	0.9957
1.2730	0.0227	1.1610	0.0343
1.2730	0.0106	1.1950	0.0148
1.2730	0.0024	1.1730	0.0234
1.2730	0.0143	1.2200	0.0079

1.2730	0.0984	1.2540	0.1962
1.2720	0.1775	1.2010	0.1533
1.2720	0.0109	1.2200	0.0139
1.2720	0.1399	1.2830	0.1686
1.2710	0.0475	1.1430	0.3131
1.2710	0.0166	1.1800	0.0459
1.2700	0.0057	1.0140	0.8313
1.2700	0.0032	1.2490	0.0025
1.2700	0.0568	1.3210	0.0966
1.2690	0.0992	0.9660	0.8165
1.2690	0.0203	1.1630	0.1559
1.2690	0.0029	1.1650	0.0399
1.2680	0.0455	1.1000	0.2967
1.2680	0.0792	1.2510	0.1972
1.2670	0.0027	1.0890	0.1970
1.2670	0.0001	1.1140	0.0189
1.2670	0.1430	1.1510	0.3084
1.2670	0.2580	1.2160	0.2580
1.2670	0.0033	1.2190	0.0297
1.2660	0.1347	0.7490	0.1083
1.2660	0.0029	1.1350	0.0469
1.2660	0.0160	1.1610	0.1194
1.2660		1.1550	
1.2660	0.5411	1.2510	0.5631
1.2660	0.0375	1.3100	0.0166
1.2660	0.0140	1.2800	0.0205
1.2660	0.0171	1.3340	0.0032
1.2650	0.0153	1.0860	0.3883
1.2650	0.0007	1.2320	0.0213
1.2650		1.3250	
1.2640		0.9280	
1.2640	0.1426	1.0550	0.5429
1.2640	0.0212	1.2580	0.0438

1.2630	0.0045	1.1520	0.4528
1.2630	0.0015	1.1960	0.0122
1.2630	0.0618	1.2620	0.3440
1.2630	0.0029	1.2500	0.0027
1.2630	0.4202	1.3280	0.0957
1.2620	0.0535	1.0760	0.2207
1.2620	0.0000	1.1260	0.0205
1.2620	0.0198	1.2390	0.0362
1.2620	0.2038	1.4470	0.0210
1.2610	0.1036	1.1420	0.2260
1.2610	0.0218	1.2050	0.0218
1.2610	0.0054	1.2270	0.0045
1.2600	0.0050	1.2590	0.0043
1.2590	0.0020	1.0950	0.2115
1.2580	0.0136	1.0620	0.3470
1.2580	0.0621	1.1580	0.2388
1.2570	0.1015	1.0390	0.7658
1.2570	0.1239	1.1530	0.2851
1.2570	0.1131	1.2510	0.1083
1.2570	0.0328	1.2350	0.0065
1.2560	0.3540	0.8260	0.4406
1.2560	0.0001	1.1630	0.0057
1.2560	0.0068	1.1930	0.0180
1.2560	0.0269	1.2700	0.0604
1.2560	0.0278	1.2840	0.0285
1.2550	0.4001	1.0580	0.8287
1.2550	0.0021	1.1930	0.0544
1.2550	0.0019	1.2040	0.0195
1.2540	0.0135	1.2210	0.0388
1.2540	0.0344	1.2060	0.0103
1.2540		1.2650	
1.2530	0.1007	1.0050	0.9782
1.2520	0.0062	0.8650	0.0069

1.2520	0.0027	1.2600	0.0061
1.2520	0.2127	1.4290	0.0609
1.2520	0.2924	1.4860	0.1429
1.2510	0.0051	1.1400	0.0516
1.2500	0.0127	1.0800	0.4707
1.2500		1.1240	
1.2500	0.0128	1.1730	0.0898
1.2500	0.1644	1.3660	0.1704
1.2490	0.0029	1.1720	0.0093
1.2480	0.0765	0.9330	0.3824
1.2480	0.0136	1.1470	0.0411
1.2480	0.0005	1.1530	0.0285
1.2480	0.0043	1.2180	0.0080
1.2480	0.0013	1.2010	0.0050
1.2480	0.0008	1.2440	0.0096
1.2470	0.0103	1.1620	0.0639
1.2470	0.0340	1.2000	0.0845
1.2470	0.0243	1.2500	0.0181
1.2470	0.2343	1.4030	0.1093
1.2460	0.0720	1.2260	0.1573
1.2460	0.0061	1.2210	0.0135
1.2450	0.0171	0.8210	0.0501
1.2450	0.3634	1.1340	0.6537
1.2450	0.0002	1.1560	0.0245
1.2450	0.0293	1.1830	0.0798
1.2450	0.0879	1.1870	0.3145
1.2450	0.0004	1.2640	0.0139
1.2450	0.0306	1.2870	0.0481
1.2440	0.0050	0.9110	0.0745
1.2440	0.2276	0.9420	0.7298
1.2440	0.0038	1.1280	0.0632
1.2440	0.0333	1.1370	0.2355
1.2440	0.0010	1.1530	0.0154

1.2440		1.1910	
1.2430	0.0030	1.1430	0.0405
1.2430	0.1199	1.2870	0.1375
1.2420		1.1130	
1.2420	0.1542	1.1590	0.2424
1.2420	0.0020	1.1890	0.0209
1.2410	0.0000	1.1310	0.0264
1.2410	0.0111	1.1410	0.1370
1.2410	0.0132	1.2830	0.0035
1.2410	0.0128	1.2880	0.0161
1.2400	0.0194	1.1670	0.0572
1.2400	0.0023	1.1730	0.0284
1.2400	0.0249	1.2670	0.0098
1.2400	0.0014	1.2850	0.0007
1.2390	0.0012	1.1040	0.0269
1.2390	0.0042	1.1390	0.0339
1.2390	0.0018	1.1970	0.0060
1.2390		1.2210	
1.2390	0.0564	1.2820	0.1030
1.2380	0.0017	1.2100	0.0010
1.2380		1.2040	
1.2370	0.0038	1.0030	0.9231
1.2370	0.0014	1.1150	0.0897
1.2370	0.0066	1.1440	0.0358
1.2370	0.0018	1.1560	0.0591
1.2360	0.1059	1.0090	0.9215
1.2360	0.0010	1.0450	0.2330
1.2360	0.0024	1.1580	0.0286
1.2360	0.0043	1.2530	0.0623
1.2350	0.0039	1.0440	0.3156
1.2350	0.0137	1.2080	0.0131
1.2350	0.0104	1.2040	0.0141
1.2350	0.0087	1.3640	0.0026

1.2340		0.9660	
1.2340	0.0491	1.1890	0.0226
1.2330	0.1813	1.1110	0.4853
1.2330	0.1489	1.1660	0.1467
1.2330	0.0943	1.2700	0.0314
1.2320	0.0900	1.0950	0.4445
1.2320	0.0142	1.1270	0.0793
1.2320	0.0842	1.1540	0.1723
1.2320	0.0004	1.1810	0.0038
1.2320	0.0016	1.2160	0.0080
1.2310	0.0743	1.0860	0.5106
1.2310	0.0183	1.1640	0.0948
1.2310	0.0329	1.2090	0.0676
1.2300	0.0034	1.1300	0.0203
1.2300	0.0033	1.2090	0.0036
1.2300	0.0832	1.2070	0.0883
1.2300		1.1830	
1.2300	0.0375	1.2430	0.0403
1.2300	0.1792	1.3760	0.0128
1.2290	0.0068	1.0780	0.2184
1.2290	0.3648	1.1590	0.6160
1.2290	0.0005	1.1450	0.0127
1.2290	0.1106	1.1550	0.1951
1.2290	0.3886	1.2550	0.2569
1.2290	0.0153	1.2120	0.0045
1.2280	0.0072	1.0960	0.1226
1.2280	0.0299	1.1530	0.1907
1.2280	0.0170	1.2140	0.0050
1.2270	0.0789	0.9430	0.5548
1.2270	0.0047	1.1300	0.1260
1.2270	0.1324	1.1450	0.0957
1.2270	0.0110	1.1400	0.0539
1.2270	0.0706	1.3000	0.0206

1.2270	0.5213	1.5110	0.2758
1.2250	0.0131	1.1350	0.0538
1.2250	0.0377	1.1230	0.1428
1.2250		1.1010	
1.2250	0.0250	1.1670	0.0297
1.2250	0.1861	1.1790	0.2961
1.2240	0.0021	1.2240	0.0002
1.2240	0.0032	1.2170	0.0059
1.2230	0.0031	1.0250	0.7089
1.2230	0.0077	1.0740	0.2212
1.2230	0.0130	1.0740	0.4477
1.2230	0.0042	1.1110	0.1431
1.2230	0.0110	1.1510	0.0689
1.2230	0.2306	1.1880	0.4749
1.2230	0.0049	1.2040	0.0154
1.2230	0.0152	1.2440	0.0291
1.2230	0.0645	1.2770	0.0160
1.2220	0.0023	1.0650	0.3052
1.2220	0.0318	1.1090	0.2030
1.2220	0.0022	1.1690	0.0239
1.2220	0.0018	1.2240	0.0047
1.2210	0.0844	1.1120	0.1837
1.2210	0.0100	1.1280	0.0440
1.2210	0.0077	1.1680	0.0805
1.2210		1.1940	
1.2200	0.1114	0.9000	0.5740
1.2200	0.1153	1.2720	0.0674
1.2190	0.0314	1.0150	0.7890
1.2190	0.0029	1.1210	0.0064
1.2180		0.9680	
1.2180	0.0210	1.0150	0.8239
1.2180	0.0712	1.0570	0.6027
1.2180	0.0102	1.1370	0.1278

1.2180	0.0362	1.2110	0.0294
1.2180		1.1960	
1.2180	0.0297	1.2270	0.0023
1.2180	0.1156	1.2820	0.0194
1.2180	0.5770	1.3110	0.4225
1.2180	0.7118	2.3080	0.1648
1.2180		1.9500	
1.2170	0.0006	1.1030	0.0636
1.2170	0.0026	1.1890	0.0023
1.2170	0.0012	1.2170	0.0053
1.2170	0.0025	1.2390	0.0138
1.2160	0.3754	0.9280	0.7426
1.2160		1.0130	
1.2160	0.0406	1.0680	0.5509
1.2160	0.1887	1.0980	0.4982
1.2160		1.0920	
1.2160	0.0211	1.2200	0.0325
1.2150	0.0055	1.1460	0.0835
1.2150	0.0306	1.1920	0.0444
1.2150	0.1299	1.1780	0.1507
1.2140	0.0804	1.1010	0.4913
1.2140	0.0240	1.1510	0.0580
1.2140	0.0736	1.1230	0.2390
1.2140		1.1070	
1.2140	0.0028	1.2260	0.0005
1.2130	0.0009	1.0830	0.1164
1.2130	0.0001	1.0840	0.0196
1.2130		1.1130	
1.2130	0.0039	1.1510	0.0282
1.2130	0.5853	1.2560	0.5134
1.2130	0.4761	1.3150	0.2784
1.2120	0.0933	1.0690	0.3662
1.2120	0.1429	1.1200	0.3710

1.2120	0.0023	1.1440	0.0425
1.2120	0.0077	1.1850	0.0697
1.2120	0.0011	1.1790	0.0506
1.2120	0.0507	1.2430	0.0884
1.2110	0.0133	0.9770	0.5101
1.2110	0.0357	1.0650	0.2892
1.2110	0.0025	1.1260	0.0347
1.2110	0.0012	1.1630	0.0894
1.2110	0.0043	1.1750	0.0170
1.2110	0.0450	1.1890	0.0568
1.2110	0.4821	1.2770	0.3716
1.2110		1.2450	
1.2100	0.0049	1.1190	0.1131
1.2100	0.0108	1.1580	0.0201
1.2100	0.0039	1.1640	0.0097
1.2100	0.0113	1.3650	0.0071
1.2100		1.3610	
1.2090	0.0027	1.0760	0.0806
1.2090	0.0005	1.0950	0.0676
1.2090	0.0158	1.1270	0.0445
1.2090	0.0023	1.0870	0.1250
1.2090	0.0000	1.1120	0.0467
1.2090	0.0067	1.1900	0.0202
1.2080	0.0017	1.1010	0.0393
1.2080	0.0017	1.1310	0.0296
1.2080	0.0279	1.1650	0.0365
1.2080	0.0414	1.2300	0.0187
1.2080	0.0029	1.2140	0.0139
1.2070	0.0294	1.0700	0.4125
1.2070	0.0371	1.1340	0.0497
1.2070	0.0054	1.1340	0.0169
1.2070	0.2502	1.1810	0.0883
1.2070	0.0271	1.2980	0.0088

1.2070		1.2650	
1.2070		1.3530	
1.2060	0.0192	1.1500	0.0368
1.2060	0.0763	1.1400	0.2223
1.2060	0.1272	1.1500	0.2081
1.2060	0.0625	1.1610	0.1489
1.2060	0.0204	1.2300	0.0217
1.2050	0.0023	1.0590	0.2729
1.2050	0.0003	1.0680	0.2873
1.2050	0.0390	1.1760	0.1350
1.2050	0.0001	1.1630	0.0015
1.2050	0.0141	1.1920	0.0572
1.2050	0.1673	1.2190	0.1143
1.2040	0.0036	1.0740	0.3270
1.2040	0.0052	1.1240	0.0277
1.2040	0.0023	1.1550	0.0245
1.2030		0.9170	
1.2030	0.0259	1.0780	0.3034
1.2030	0.2572	1.1260	0.3806
1.2030	0.0669	1.1200	0.2402
1.2030	0.0075	1.1670	0.0224
1.2030	0.0203	1.1950	0.0203
1.2020	0.0018	1.1660	0.0112
1.2010		1.0510	
1.2010	0.1459	1.0710	0.5783
1.2010	0.0237	1.1060	0.0830
1.2010	0.0205	1.1720	0.0342
1.2000	0.2063	1.0500	0.7486
1.2000	0.3240	1.1000	0.5410
1.2000	0.1170	1.2060	0.0917
1.2000	0.3272	1.2870	0.2380
1.1990	0.0147	1.0470	0.2217
1.1990	0.0049	1.0750	0.1856

1.1990	0.0228	1.1280	0.1026
1.1990	0.0086	1.1590	0.0192
1.1990	0.0276	1.1500	0.0478
1.1990	0.0028	1.1840	0.0020
1.1990	0.0559	1.2090	0.0450
1.1990		1.2240	
1.1980	0.9528	0.6510	0.7924
1.1980	0.1279	1.1410	0.3694
1.1980	0.0488	1.1250	0.1752
1.1980	0.0211	1.1810	0.0530
1.1980	0.1101	1.2480	0.0151
1.1980		1.2050	
1.1970	0.0350	0.9240	0.1817
1.1970	0.1082	1.0680	0.3556
1.1970	0.0032	1.0960	0.0952
1.1970	0.0975	1.1290	0.2170
1.1970	0.0682	1.1640	0.1358
1.1970	0.0294	1.2160	0.0206
1.1970	0.0707	1.2090	0.0243
1.1960	0.1570	1.0690	0.5076
1.1960	0.0028	1.0540	0.3296
1.1960	0.0019	1.1180	0.0089
1.1960		1.1030	
1.1960	0.1407	1.1600	0.0709
1.1960	0.0060	1.1290	0.2172
1.1960	0.1400	1.1910	0.2313
1.1960	0.0427	1.1460	0.1199
1.1960	0.0149	1.1800	0.0188
1.1960	0.0013	1.1970	0.0011
1.1960	0.1449	1.4390	0.0155
1.1950	0.1695	0.9460	0.5055
1.1950		0.9500	
1.1950	0.0022	1.1320	0.0310

1.1950		1.1060	
1.1950	0.0066	1.1770	0.0181
1.1950		1.2180	
1.1940	0.0277	1.1260	0.0574
1.1940	0.0076	1.1210	0.3412
1.1940	0.0415	1.1600	0.0364
1.1940		1.4780	
1.1930	0.0040	1.1120	0.0492
1.1930	0.0164	1.1410	0.0676
1.1930	0.0675	1.2180	0.0757
1.1920	0.0009	1.0790	0.0158
1.1920	0.0102	1.0930	0.2082
1.1920	0.1067	1.1090	0.1961
1.1920	0.0018	1.1670	0.0062
1.1910	0.3679	0.9010	0.3235
1.1910	0.1593	1.0030	0.9766
1.1910	0.0838	1.0770	0.3184
1.1910	0.2626	1.1220	0.4524
1.1910	0.0198	1.0980	0.2431
1.1910	0.0040	1.1120	0.0040
1.1910	0.0390	1.2730	0.0076
1.1910		1.2320	
1.1910	0.5695	1.3740	0.2388
1.1900	0.0097	1.1530	0.0871
1.1900	0.2013	1.2030	0.2245
1.1900		1.3310	
1.1890	0.0559	0.9900	0.8893
1.1890	0.0531	1.0400	0.4821
1.1890	0.0024	1.0500	0.5053
1.1890	0.0563	1.0610	0.3715
1.1890	0.0033	1.0840	0.0611
1.1890	0.0091	1.1140	0.0026
1.1890	0.1118	1.1090	0.1260

1.1890	0.0141	1.1470	0.0791
1.1890	0.3528	1.1490	0.2428
1.1890	0.0091	1.2120	0.0160
1.1880	0.0029	1.0210	0.6831
1.1880	0.0615	1.1090	0.3255
1.1880	0.0650	1.1230	0.2105
1.1880	0.0135	1.1270	0.1662
1.1880	0.0329	1.1240	0.0780
1.1880	0.0261	1.2320	0.0203
1.1870	0.0302	1.0150	0.7714
1.1870	0.0265	1.0320	0.6946
1.1870	0.0797	1.1300	0.2056
1.1870	0.0145	1.1650	0.1635
1.1870	0.0374	1.1840	0.0594
1.1870		1.1810	
1.1860	0.0004	1.0410	0.3826
1.1860	0.0008	1.0890	0.0248
1.1860	0.0281	1.1150	0.2690
1.1860	0.1896	1.1480	0.1129
1.1860	0.0595	1.1530	0.0759
1.1860	0.0740	1.1330	0.1562
1.1860	0.2750	1.2800	0.2551
1.1850	0.1261	1.0320	0.8145
1.1850	0.0270	1.0930	0.2561
1.1850	0.0010	1.1070	0.0044
1.1850	0.0295	1.1790	0.0331
1.1850	0.0193	1.2320	0.0083
1.1840	0.0014	1.0810	0.0719
1.1840	0.0221	1.1430	0.1880
1.1840	0.0005	1.1960	0.0006
1.1830	0.4767	1.0680	0.8020
1.1830	0.2311	1.0590	0.7247
1.1820	0.0053	1.1240	0.0146

1.1820	0.0205	1.1480	0.0647
1.1820	0.0787	1.1650	0.0965
1.1810		0.7530	
1.1810	0.0071	1.0380	0.3897
1.1810	0.0158	1.0750	0.2344
1.1810	0.1410	1.1030	0.5388
1.1810	0.1442	1.4720	0.0300
1.1810	0.3346	1.4990	0.0537
1.1800	0.0148	1.0430	0.3538
1.1800	0.1580	1.1010	0.3854
1.1800	0.1071	1.1280	0.4583
1.1800	0.1750	1.2020	0.0329
1.1790	0.0657	0.9890	0.9790
1.1790	0.0184	1.0830	0.1475
1.1790	0.0093	1.1930	0.0511
1.1790		1.1960	
1.1790		1.2550	
1.1780	0.0737	1.0460	0.6947
1.1780	0.1650	1.1120	0.3223
1.1780	0.0468	1.1670	0.0622
1.1780	0.0285	1.1760	0.0170
1.1780	0.0067	1.1530	0.0164
1.1780	0.1592	1.3010	0.0490
1.1770	0.0145	1.0670	0.3830
1.1770	0.2219	1.1240	0.2793
1.1770	0.0378	1.1840	0.0027
1.1760	0.4679	1.0870	0.0347
1.1760	0.0359	1.1820	0.1756
1.1760	0.1127	1.2300	0.0686
1.1760	0.0013	1.2620	0.0003
1.1750	0.1299	1.0900	0.5070
1.1750	0.2566	1.0660	0.5881
1.1750	0.1140	1.0590	0.5540

1.1750	0.0021	1.0790	0.2017
1.1750	0.0141	1.0940	0.0356
1.1750	0.8681	1.2060	0.8780
1.1750	0.0960	1.2890	0.0090
1.1740	0.0239	1.1320	0.0528
1.1740	0.0184	1.1460	0.0321
1.1730	0.0050	1.0460	0.3144
1.1730		1.1060	
1.1730	0.1225	1.0930	0.3761
1.1730	0.0270	1.1520	0.1877
1.1730	0.0128	1.3400	0.0180
1.1720		1.0220	
1.1720	0.0004	1.1600	0.0014
1.1720	0.0442	1.1750	0.0938
1.1720		1.1510	
1.1720		1.1960	
1.1720	0.1381	1.2380	0.0274
1.1710	0.0210	0.9800	0.8286
1.1710	0.0001	0.9990	0.9935
1.1710	0.0321	1.0500	0.1952
1.1710	0.0960	1.0790	0.2464
1.1710	0.0422	1.0860	0.2901
1.1710	0.0335	1.1290	0.0775
1.1710		1.0840	
1.1710	0.0049	1.1300	0.0108
1.1710	0.0189	1.2010	0.0028
1.1710	0.0972	1.3040	0.0243
1.1710	0.0200	1.2970	0.0054
1.1700	0.1144	1.0070	0.8921
1.1700	0.4668	1.0840	0.7404
1.1700	0.0428	1.1800	0.0047
1.1700	0.0833	1.1650	0.0767
1.1700	0.2036	1.2350	0.0520

1.1690	0.4723	1.0340	0.9742
1.1690	0.2377	1.0460	0.7869
1.1690	0.0536	1.1700	0.0585
1.1690	0.0960	1.2320	0.0425
1.1690	0.7853	1.2730	0.6188
1.1680	0.0533	1.0560	0.3599
1.1680	0.0044	1.0710	0.0532
1.1680	0.2547	1.1870	0.1385
1.1680	0.0716	1.1560	0.0897
1.1680	0.0048	1.2660	0.0023
1.1670	0.1173	0.9430	0.5583
1.1670		1.0540	
1.1670	0.0310	1.1180	0.0302
1.1670	0.0011	1.1350	0.0006
1.1670	0.0004	1.1690	0.0016
1.1670	0.0003	1.1600	0.0002
1.1670	0.2986	1.2650	0.1141
1.1660	0.0942	1.0120	0.8228
1.1660	0.0212	1.0300	0.5432
1.1660	0.0068	1.0570	0.3206
1.1660	0.1039	1.1000	0.3069
1.1660	0.3136	1.1310	0.4098
1.1660	0.0590	1.1520	0.0777
1.1660		1.1660	
1.1660	0.0373	1.2060	0.0117
1.1660	0.2678	1.2510	0.0995
1.1660		1.2240	
1.1650	0.0030	1.0500	0.2716
1.1650	0.0132	1.1120	0.0329
1.1640	0.3291	1.1250	0.4110
1.1640	0.0583	1.2080	0.0026
1.1640	0.0756	1.1850	0.0648
1.1640	0.0794	1.2180	0.1028

1.1640	0.0919	1.3210	0.0076
1.1640		1.7320	
1.1630	0.4062	1.0690	0.7204
1.1630	0.0402	1.2420	0.0137
1.1620		0.9080	
1.1620	0.0107	1.0180	0.5558
1.1620	0.0404	1.1000	0.1070
1.1620	0.0639	1.0990	0.2167
1.1620	0.0079	1.2090	0.0120
1.1610	0.1374	1.0320	0.6623
1.1610	0.0578	1.0380	0.6792
1.1610	0.0033	1.0020	0.9459
1.1610	0.0125	1.1130	0.0708
1.1610		1.0750	
1.1610	0.0003	1.0990	0.0201
1.1610	0.1509	1.1460	0.2319
1.1610	0.1191	1.1880	0.1627
1.1610	0.0428	1.3410	0.0028
1.1610		1.2870	
1.1600	0.4372	1.0390	0.8725
1.1600	0.0026	1.1000	0.0375
1.1600	0.0046	1.0700	0.2430
1.1600		1.0900	
1.1600	0.0241	1.1660	0.0692
1.1600	0.1373	1.2000	0.0631
1.1600	0.0106	1.2140	0.0040
1.1600	0.1876	1.2420	0.0636
1.1590	0.2740	0.9500	0.9209
1.1590	0.1819	1.0200	0.7997
1.1590		1.0620	
1.1590	0.0018	1.0960	0.0632
1.1590	0.0107	1.0790	0.0273
1.1590	0.1328	1.1690	0.0831

1.1590	0.1668	1.2260	0.1821
1.1580	0.0714	1.0790	0.2758
1.1580	0.0009	1.1350	0.0069
1.1580		1.1190	
1.1580	0.1841	1.1470	0.1187
1.1580	0.1678	1.2270	0.0407
1.1570	0.3124	0.9850	0.8172
1.1570	0.0047	1.1780	0.0021
1.1570	0.1285	1.2260	0.0419
1.1570		1.3430	
1.1570	0.9847	1.8560	0.3148
1.1560	0.1640	1.1060	0.3618
1.1560	0.0048	1.2150	0.0116
1.1560	0.2225	1.2840	0.1248
1.1550	0.0004	0.9820	0.7199
1.1550	0.1147	1.0350	0.5578
1.1550	0.0032	1.0770	0.0310
1.1550	0.0125	1.1090	0.0843
1.1550	0.0174	1.1010	0.1999
1.1550	0.0459	1.0480	0.4201
1.1550	0.1218	1.1790	0.1348
1.1550	0.1247	1.2020	0.0871
1.1550	0.1198	1.3050	0.0353
1.1550	0.1600	1.4000	0.0317
1.1540	0.0602	1.0220	0.7199
1.1540	0.0303	1.1160	0.0800
1.1540	0.3494	1.1320	0.4968
1.1540		1.1460	
1.1540	0.0043	1.2350	0.0008
1.1530	0.0425	0.9730	0.5703
1.1530	0.0705	1.0560	0.3327
1.1530		1.0640	
1.1530	0.0202	1.1540	0.0042

1.1520	0.0159	1.0760	0.3367
1.1520	0.0267	1.0970	0.0696
1.1520	0.0791	1.0950	0.2801
1.1520		1.0760	
1.1520	0.0049	1.1420	0.0306
1.1520	0.3026	1.2120	0.2062
1.1520	0.3850	1.2500	0.1906
1.1510	0.0139	1.0160	0.7559
1.1510	0.5313	1.1520	0.3499
1.1510	0.2084	1.0280	0.7424
1.1510	0.0078	1.0980	0.2275
1.1510	0.0273	1.2100	0.0029
1.1510		1.3090	
1.1500	0.0502	0.9100	0.3306
1.1500	0.2454	0.9440	0.5862
1.1500	0.0092	1.0160	0.7418
1.1500		1.0200	
1.1500	0.0864	1.1260	0.1736
1.1500	0.6209	1.1610	0.5853
1.1500	0.1196	1.1460	0.1481
1.1500	0.0728	1.1500	0.0020
1.1490	0.4992	1.0730	0.6889
1.1490	0.2191	1.2470	0.1162
1.1480	0.1002	0.9470	0.3434
1.1480	0.0181	1.1170	0.1184
1.1480	0.1456	1.1880	0.1725
1.1480	0.0110	1.1680	0.0171
1.1480	0.0241	1.2400	0.0081
1.1480	0.0674	1.2530	0.0112
1.1480		1.2180	
1.1470	0.4720	0.9860	0.7978
1.1470	0.2439	1.0110	0.9040
1.1470	0.0166	1.1320	0.0042

1.1470	0.0061	1.1780	0.0172
1.1470	0.0090	1.2490	0.0053
1.1460	0.3656	0.8420	0.2902
1.1460	0.2498	0.8490	0.1077
1.1460	0.0333	0.9960	0.9924
1.1460	0.0938	1.0370	0.6993
1.1460	0.0102	1.1340	0.0133
1.1460	0.1042	1.1710	0.0145
1.1460	0.0154	1.1850	0.0010
1.1460	0.0853	1.1960	0.0282
1.1460	0.0681	1.2340	0.0398
1.1460		1.3450	
1.1450		0.9620	
1.1450	0.2601	1.0470	0.6409
1.1450	0.0415	1.0850	0.2469
1.1450	0.0385	1.0800	0.1125
1.1450	0.1122	1.0990	0.2641
1.1450	0.0789	1.1330	0.0728
1.1450	0.0642	1.1740	0.0353
1.1450		1.3110	
1.1440	0.0214	1.0590	0.2593
1.1440	0.4316	1.0500	0.8507
1.1440	0.0430	1.0490	0.4885
1.1440	0.0971	1.1060	0.1836
1.1430	0.0025	1.0870	0.0493
1.1430	0.0693	1.1140	0.1556
1.1430	0.2463	1.1390	0.4455
1.1430	0.0185	1.1580	0.0042
1.1430	0.0158	1.2270	0.0048
1.1430		1.3020	
1.1420	0.0127	0.9680	0.7659
1.1420	0.0007	1.0830	0.1857
1.1420	0.0620	1.1740	0.0350

1.1420	0.0430	1.2350	0.0023
1.1420	0.1127	1.2580	0.0043
1.1410	0.2427	0.9870	0.9503
1.1410	0.0025	0.9890	0.7492
1.1410	0.2886	0.9660	0.7911
1.1410	0.0367	1.1080	0.0908
1.1410		1.1140	
1.1410		1.1300	
1.1410	0.0351	1.1500	0.0595
1.1410	0.0395	1.1830	0.1047
1.1410	0.2850	1.2710	0.0129
1.1410	0.1967	1.2270	0.0506
1.1410	0.1681	1.2650	0.0464
1.1410		1.2590	
1.1410	0.1008	1.3270	0.0364
1.1410		1.8870	
1.1400	0.5587	1.0030	0.9810
1.1400	0.0222	1.0070	0.8192
1.1400	0.1373	0.9870	0.7418
1.1400	0.2751	1.0270	0.7642
1.1400	0.0576	1.0420	0.4485
1.1400	0.5260	1.0870	0.6061
1.1400	0.0261	1.0660	0.3991
1.1400	0.0102	1.0980	0.0785
1.1400	0.0562	1.1220	0.1557
1.1400	0.1505	1.1580	0.1761
1.1400	0.2460	1.1870	0.3189
1.1390	0.0665	0.9750	0.6740
1.1390	0.0276	1.0840	0.0690
1.1390	0.0999	1.1520	0.0032
1.1390		1.1650	
1.1390	0.1088	1.2090	0.0258
1.1390	0.2031	1.2930	0.0180

1.1380	0.3559	0.9590	0.9365
1.1380	0.2301	0.9840	0.8757
1.1380		1.0510	
1.1380	0.2494	1.1340	0.2986
1.1380	0.0888	1.1180	0.1596
1.1380	0.0664	1.1630	0.0098
1.1380	0.0295	1.1900	0.0398
1.1370	0.0494	0.9500	0.3928
1.1370		1.0420	
1.1370	0.0093	1.0180	0.6265
1.1370	0.0066	1.0990	0.1859
1.1370	0.0551	1.0800	0.1159
1.1370	0.1361	1.1410	0.1022
1.1370	0.0032	1.1470	0.0012
1.1370	0.1740	1.1540	0.2560
1.1370	0.1083	1.1810	0.0236
1.1370	0.1034	1.2240	0.0289
1.1370	0.1873	1.2450	0.0762
1.1370	0.6256	1.3910	0.1837
1.1360	0.2043	0.9550	0.3491
1.1360	0.0529	1.0900	0.1596
1.1360	0.5259	1.1620	0.4445
1.1360	0.0736	1.1330	0.3993
1.1360	0.2907	1.2100	0.1234
1.1350	0.0251	1.0320	0.4775
1.1350	0.1800	1.0860	0.3217
1.1350	0.5104	1.1720	0.4242
1.1350	0.3324	1.2530	0.0463
1.1350	0.0794	1.2380	0.0073
1.1340	0.1264	1.0050	0.8935
1.1340	0.0852	1.1130	0.2162
1.1340	0.1906	1.1160	0.2699
1.1340	0.1025	1.3360	0.0033

1.1330	0.2661	1.0350	0.6993
1.1330	0.5178	1.1050	0.6093
1.1330		1.1400	
1.1330	0.1435	1.1800	0.0397
1.1320	0.0046	1.0460	0.4605
1.1320	0.1076	1.0660	0.4573
1.1320	0.0492	1.1150	0.0968
1.1310	0.0298	1.0960	0.1390
1.1310	0.0383	1.1000	0.1354
1.1310	0.0263	1.1000	0.0990
1.1310		1.1090	
1.1310	0.0610	1.1760	0.0346
1.1310	0.2952	1.2220	0.1353
1.1300	0.3748	0.9080	0.5044
1.1300	0.0056	0.9940	0.9150
1.1300	0.0340	1.1370	0.0248
1.1300	0.1053	1.2250	0.0412
1.1290	0.0871	0.9990	0.9430
1.1290	0.1809	1.0250	0.8307
1.1290	0.1490	1.0900	0.0865
1.1290		1.0890	
1.1290		1.1220	
1.1290	0.0947	1.2330	0.0428
1.1280	0.2970	0.9620	0.6268
1.1280		1.0250	
1.1280	0.1306	1.0720	0.4311
1.1280	0.0078	1.0870	0.0074
1.1280	0.0275	1.0980	0.1359
1.1280	0.0194	1.1020	0.0605
1.1280	0.0998	1.1450	0.0786
1.1280	0.2786	1.1700	0.0297
1.1280	0.4801	1.4120	0.0847
1.1270	0.0643	1.0530	0.3379

1.1270		1.0960	
1.1270		1.1430	
1.1270	0.1285	1.2060	0.0379
1.1270	0.4555	1.2850	0.1850
1.1270		1.6200	
1.1260		1.0690	
1.1260		1.0920	
1.1260	0.0711	1.2010	0.0114
1.1260		1.1470	
1.1260	0.0530	1.1690	0.0346
1.1260		1.2070	
1.1250	0.3500	1.0610	0.6132
1.1250	0.3654	1.0880	0.2771
1.1250	0.0457	1.0880	0.0906
1.1250	0.0275	1.0840	0.2130
1.1250		1.0900	
1.1250	0.0298	1.1060	0.0761
1.1250	0.2349	1.1150	0.2758
1.1250	0.0331	1.0950	0.0727
1.1250	0.0114	1.1440	0.0175
1.1250	0.0726	1.1860	0.0081
1.1250	0.0634	1.1960	0.0087
1.1250		1.3830	
1.1240	0.3943	1.0090	0.8520
1.1240	0.0085	1.0900	0.1490
1.1240	0.1687	1.1330	0.2415
1.1240	0.0487	1.1090	0.0391
1.1240	0.0446	1.1940	0.0004
1.1240		1.2380	
1.1230	0.0207	1.1660	0.0210
1.1230	0.0078	1.1850	0.0021
1.1230	0.1473	1.1950	0.0513
1.1230	0.0512	1.1860	0.0072

1.1220	0.4959	1.0220	0.9262
1.1220	0.0088	1.1140	0.1615
1.1220	0.4546	1.1870	0.2954
1.1220		1.2030	
1.1220	0.5083	1.7950	0.0200
1.1210	0.3618	0.9720	0.8935
1.1210	0.0375	1.0420	0.2933
1.1210		1.0510	
1.1210		1.0800	
1.1210	0.2776	1.1130	0.1846
1.1200	0.2393	0.9060	0.1920
1.1200	0.3707	1.0400	0.7371
1.1200	0.1721	1.1150	0.1621
1.1200	0.2706	1.1200	0.2219
1.1200	0.2433	1.1240	0.2454
1.1200	0.0771	1.1400	0.0567
1.1200	0.0513	1.1570	0.0086
1.1200	0.1042	1.1600	0.0392
1.1200	0.0509	1.1960	0.0430
1.1200	0.2209	1.1680	0.1364
1.1200		1.2070	
1.1190	0.6715	1.2320	0.2118
1.1190		1.3830	
1.1180	0.1233	1.0970	0.1309
1.1180	0.0211	1.1120	0.0087
1.1180	0.0358	1.1170	0.0500
1.1180	0.1600	1.2800	0.0004
1.1170		0.8580	
1.1170	0.0337	1.1270	0.0457
1.1170		1.1480	
1.1160	0.7529	0.9790	0.8505
1.1160	0.0944	1.0450	0.4654
1.1160	0.0351	1.0500	0.3193

1.1160	0.0036	1.1190	0.0992
1.1160	0.6483	1.0860	0.7631
1.1160	0.0908	1.1430	0.0510
1.1160	0.5734	1.5580	0.0480
1.1150	0.2735	0.9740	0.8056
1.1150		0.9970	
1.1150	0.3390	1.0550	0.6702
1.1150	0.2256	1.1140	0.3181
1.1150	0.0160	1.1170	0.0754
1.1150	0.0077	1.1700	0.0162
1.1150		1.2140	
1.1140		0.9860	
1.1140	0.0013	1.0760	0.1019
1.1140	0.1105	1.0750	0.2428
1.1140	0.0089	1.1600	0.0066
1.1140	0.2089	1.1530	0.1536
1.1140	0.0448	1.1510	0.0278
1.1140	0.3635	1.1870	0.2046
1.1140	0.0709	1.1690	0.0237
1.1140	0.1300	1.1970	0.0160
1.1140	0.2934	1.2770	0.0356
1.1130	0.8287	0.9070	0.6197
1.1130	0.3900	0.9560	0.7514
1.1130	0.0845	0.9660	0.3760
1.1130		0.9980	
1.1130	0.3168	1.0540	0.6841
1.1130		1.0960	
1.1130	0.1061	1.1420	0.0531
1.1130	0.2913	1.1620	0.0620
1.1130	0.1802	1.2260	0.0382
1.1130		1.2090	
1.1130	0.1383	1.2730	0.0454
1.1130	0.3178	1.3090	0.0147

1.1120	0.1487	0.9770	0.6156
1.1120	0.0025	1.1290	0.0060
1.1120	0.2997	1.1920	0.1772
1.1120	0.0701	1.2420	0.0082
1.1120	0.2729	1.1980	0.1077
1.1110	0.3140	1.0620	0.4944
1.1110	0.0020	1.0490	0.1356
1.1110	0.0563	1.0620	0.2294
1.1110	0.0209	1.1140	0.0398
1.1110	0.0325	1.1300	0.0649
1.1110	0.1887	1.1660	0.0716
1.1110	0.3726	1.1920	0.0330
1.1110	0.4080	1.2740	0.0271
1.1110		1.2050	
1.1100	0.1496	1.0270	0.5108
1.1100		1.0320	
1.1100	0.0329	1.0500	0.0676
1.1100	0.3030	1.1120	0.2156
1.1100	0.1948	1.1590	0.0851
1.1100		1.1390	
1.1100	0.1465	1.2180	0.0174
1.1100	0.1711	1.2390	0.0260
1.1090		0.9080	
1.1090	0.4206	0.9950	0.9461
1.1090	0.0808	1.1000	0.0214
1.1090		1.0500	
1.1090	0.0149	1.1060	0.0279
1.1090	0.3307	1.1420	0.2479
1.1090	0.1161	1.1320	0.0470
1.1090		1.1220	
1.1090	0.2265	1.1130	0.0726
1.1090	0.0955	1.2320	0.0030
1.1090	0.1313	1.2880	0.0112

1.1080	0.1908	1.0720	0.0760
1.1080	0.4900	1.0950	0.6193
1.1080	0.1095	1.1240	0.0880
1.1080		1.1160	
1.1080	0.1344	1.1640	0.0328
1.1070		1.0520	
1.1070	0.0120	1.0790	0.0066
1.1070	0.1151	1.0860	0.2275
1.1070	0.1345	1.0890	0.2355
1.1070	0.2487	1.0960	0.4092
1.1070	0.0354	1.1250	0.0251
1.1070	0.0558	1.1680	0.0089
1.1070	0.5062	1.2160	0.1998
1.1070	0.2691	1.2180	0.1760
1.1070	0.9438	1.6230	0.3957
1.1060	0.0689	0.9970	0.9739
1.1060	0.2138	1.0300	0.7712
1.1060	0.0215	1.0710	0.1148
1.1060	0.2357	1.1130	0.2993
1.1060	0.1535	1.2090	0.0157
1.1060	0.3558	1.2500	0.0631
1.1050	0.1713	1.0950	0.0491
1.1050	0.0206	1.1000	0.0861
1.1050	0.2288	1.1340	0.1189
1.1050	0.2177	1.1770	0.0615
1.1050	0.1971	1.1790	0.0109
1.1050	0.2897	1.2800	0.0463
1.1040	0.1612	1.0380	0.4896
1.1040	0.2966	1.0310	0.2881
1.1040	0.1823	1.0800	0.1543
1.1040	0.0218	1.0590	0.2274
1.1040	0.4002	1.0700	0.7308
1.1040	0.0586	1.1240	0.0719

1.1040	0.0236	1.1370	0.0097
1.1040	0.2465	1.1940	0.0355
1.1040	0.3057	1.2490	0.0208
1.1040	0.1020	1.3490	0.0032
1.1030		1.0680	
1.1030	0.2947	1.0680	0.4991
1.1030	0.0826	1.0610	0.2465
1.1030	0.1358	1.0800	0.1097
1.1030	0.0034	1.1540	0.0005
1.1030	0.2545	1.1510	0.0642
1.1030	0.2846	1.1540	0.5317
1.1030	0.1080	1.1740	0.0344
1.1020	0.0141	0.9810	0.4554
1.1020	0.0808	1.0070	0.6761
1.1020	0.0369	1.0120	0.7915
1.1020	0.0580	1.0490	0.3693
1.1020	0.1571	1.0700	0.2206
1.1020	0.0734	1.0700	0.1497
1.1020	0.1766	1.0810	0.3685
1.1020	0.3699	1.0800	0.4915
1.1020		1.2670	
1.1010	0.2016	0.8100	0.0402
1.1010	0.3403	0.9540	0.7329
1.1010	0.0385	0.9780	0.6419
1.1010	0.0764	1.0560	0.0943
1.1010	0.0323	1.1100	0.0895
1.1010	0.2557	1.1360	0.1386
1.1010	0.0964	1.1840	0.0129
1.1010	0.3148	1.4200	0.0052
1.1000		1.0540	
1.1000	0.6260	1.0610	0.7435
1.1000	0.4701	1.1780	0.3218
1.1000	0.1436	1.1620	0.0470

1.0990	0.2622	0.8160	0.0435
1.0990	0.2023	1.2130	0.1341
1.0990	0.1271	1.2690	0.0029
1.0980	0.1650	0.8970	0.1741
1.0980	0.1753	0.9720	0.5015
1.0980	0.1679	1.0060	0.9650
1.0980	0.0859	1.1110	0.0698
1.0980	0.0476	1.1040	0.1067
1.0980	0.0449	1.1130	0.0547
1.0980	0.1292	1.1540	0.0381
1.0980	0.2540	1.1620	0.1284
1.0980	0.1437	1.2130	0.0038
1.0980		1.2040	
1.0980	0.2183	1.2360	0.0097
1.0970	0.1375	1.0810	0.2418
1.0970	0.2577	1.1760	0.0622
1.0970	0.3185	1.2550	0.0402
1.0960		0.7990	
1.0960	0.0411	0.9220	0.0359
1.0960	0.0465	1.0140	0.7699
1.0960	0.0314	1.0470	0.2296
1.0960	0.0298	1.0910	0.0049
1.0960	0.2286	1.1360	0.1474
1.0960	0.3287	1.4840	0.0151
1.0950		0.9700	
1.0950	0.1476	1.0300	0.6092
1.0950	0.0568	1.0300	0.4574
1.0950	0.1704	1.0110	0.8765
1.0950	0.0042	1.0950	0.0037
1.0950	0.0900	1.1370	0.0112
1.0950	0.3333	1.2860	0.0248
1.0950	0.3353	1.2350	0.0613
1.0940	0.3338	0.9320	0.3083

1.0940	0.6094	0.9810	0.8945
1.0940	0.1474	1.0360	0.4876
1.0940	0.0403	1.0670	0.0939
1.0940	0.1678	1.1050	0.0709
1.0940	0.2048	1.1120	0.1071
1.0940		1.1180	
1.0940	0.2255	1.1730	0.0556
1.0940	0.1156	1.1590	0.0216
1.0940	0.0840	1.2110	0.0055
1.0940	0.0769	1.2210	0.0026
1.0930	0.1415	0.9980	0.9879
1.0930		1.0140	
1.0930	0.1057	1.0520	0.1961
1.0930	0.1265	1.1250	0.1849
1.0920		0.9570	
1.0920	0.0555	0.9980	0.9920
1.0920	0.1261	1.0270	0.5692
1.0920		1.0530	
1.0920		1.0760	
1.0920	0.1503	1.1520	0.0094
1.0920		1.2520	
1.0910	0.4503	0.9390	0.6871
1.0910	0.1923	0.9830	0.8383
1.0910	0.1604	1.0250	0.7329
1.0910	0.3606	1.0580	0.4448
1.0910	0.0305	1.0810	0.1274
1.0910	0.5423	1.1100	0.3872
1.0900	0.1051	0.9760	0.5334
1.0900	0.0395	1.0530	0.2683
1.0900	0.0304	1.0700	0.1662
1.0900	0.2004	1.0520	0.2226
1.0900	0.1904	1.0640	0.2632
1.0900		1.0580	

1.0900		1.0680	
1.0900		1.1850	
1.0900		1.2310	
1.0890	0.5693	0.8360	0.2969
1.0890	0.1763	1.0830	0.3481
1.0890	0.0132	1.0960	0.0578
1.0890	0.0879	1.0960	0.1466
1.0890	0.1107	1.1320	0.0310
1.0890		1.2080	
1.0890		1.2010	
1.0890	0.4284	1.3160	0.0270
1.0890	0.8372	1.3750	0.2378
1.0880	0.1284	1.0090	0.8529
1.0880	0.0142	1.0540	0.1513
1.0880	0.2265	1.0660	0.3463
1.0880	0.2872	1.0510	0.5473
1.0880	0.1203	1.0770	0.2154
1.0880	0.2455	1.0920	0.2048
1.0880	0.2637	1.1060	0.2713
1.0880	0.4957	1.1360	0.3358
1.0880	0.0805	1.1370	0.0120
1.0880	0.1336	1.1590	0.0294
1.0880	0.3858	1.1840	0.2635
1.0880	0.4687	1.2440	0.1107
1.0880	0.5427	1.2600	0.1833
1.0880		1.2720	
1.0870	0.2221	0.9840	0.7581
1.0870	0.1206	1.0430	0.2828
1.0870	0.2243	1.0380	0.7346
1.0870	0.2976	1.0660	0.5821
1.0870	0.4560	1.0730	0.6895
1.0870	0.4538	1.0590	0.6496
1.0870	0.1753	1.0940	0.0530

1.0870	0.0555	1.0970	0.0139
1.0870	0.0301	1.0940	0.0351
1.0860	0.3047	0.9210	0.2818
1.0860	0.3398	0.9290	0.2586
1.0860	0.0511	1.0180	0.5600
1.0860	0.0828	1.0130	0.7588
1.0860	0.0247	1.0490	0.2388
1.0860	0.1661	1.0890	0.2475
1.0860		1.1320	
1.0860		1.1920	
1.0850	0.0569	0.9790	0.4469
1.0850	0.1550	1.0260	0.6228
1.0850	0.2374	1.0400	0.6207
1.0850	0.5571	1.0680	0.3888
1.0850	0.2838	1.1150	0.2692
1.0850	0.4896	1.0840	0.4929
1.0850	0.4223	1.1230	0.2832
1.0850	0.1979	1.1130	0.0846
1.0850	0.0434	1.1340	0.0103
1.0850	0.0998	1.1480	0.0009
1.0850	0.2197	1.1700	0.0516
1.0850	0.5923	1.4460	0.0159
1.0840		0.6150	
1.0840	0.4192	0.8010	0.0085
1.0840	0.2772	1.0610	0.4028
1.0840	0.0332	1.0640	0.1000
1.0840	0.0096	1.0760	0.0763
1.0840	0.0013	1.1100	0.0295
1.0840	0.3538	1.1120	0.2205
1.0830	0.2768	0.8170	0.0113
1.0830	0.1182	1.0290	0.3880
1.0830	0.2503	1.0320	0.6667
1.0830	0.1863	1.0440	0.4327

1.0830	0.0878	1.1140	0.0527
1.0820	0.2603	0.9860	0.8571
1.0820	0.0057	0.9910	0.8536
1.0820	0.1813	1.0210	0.6587
1.0820	0.0782	1.0510	0.2783
1.0820		1.0240	
1.0820	0.1065	1.0580	0.1471
1.0820	0.4325	1.1360	0.2161
1.0820	0.2573	1.1600	0.1484
1.0810	0.3083	1.0070	0.9107
1.0810	0.6269	0.9690	0.7843
1.0810	0.0127	1.0210	0.5031
1.0810		1.0300	
1.0810	0.2309	1.0240	0.7072
1.0810	0.5116	1.1010	0.0781
1.0810		1.1600	
1.0810	0.6237	1.2380	0.1612
1.0810	0.2430	1.6960	0.0168
1.0800	0.1908	0.9310	0.1593
1.0800	0.0910	1.0050	0.8638
1.0800	0.2849	1.0620	0.2245
1.0800	0.4278	1.0970	0.4354
1.0800	0.0606	1.2480	0.0056
1.0790	0.1492	0.8970	0.0706
1.0790	0.2963	1.0290	0.7535
1.0790	0.1233	1.0640	0.1483
1.0790	0.0354	1.0780	0.0511
1.0790	0.0105	1.0800	0.3426
1.0790	0.1356	1.1560	0.0136
1.0780		0.9620	
1.0780	0.1136	0.9760	0.4691
1.0780	0.0082	1.0090	0.7945
1.0780	0.3539	0.9850	0.8105

1.0780	0.1877	0.9880	0.8362
1.0780	0.2643	0.9640	0.7603
1.0780	0.2036	1.0470	0.5468
1.0780	0.1802	1.1070	0.0582
1.0780		1.2520	
1.0780	0.4326	1.2960	0.0911
1.0780	0.6916	2.9830	0.0319
1.0770	0.0948	1.0520	0.4830
1.0770	0.3307	1.0490	0.6137
1.0770	0.3900	1.0670	0.3227
1.0770	0.4443	1.1370	0.1996
1.0770	0.0959	1.0710	0.2785
1.0770	0.3711	1.1610	0.0755
1.0770		1.1580	
1.0760		0.9320	
1.0760	0.5132	0.9570	0.8135
1.0760	0.2444	1.0400	0.6013
1.0760	0.1933	1.0710	0.2845
1.0760	0.0352	1.0740	0.4624
1.0750		0.9120	
1.0750	0.0772	0.9850	0.6597
1.0750		1.1180	
1.0750	0.6847	1.3790	0.0681
1.0740		0.8320	
1.0740	0.3856	0.9280	0.3008
1.0740	0.1365	1.0160	0.7873
1.0740	0.1759	1.0870	0.0093
1.0740	0.1513	1.1260	0.0631
1.0740	0.1522	1.1930	0.0177
1.0730		0.9570	
1.0730	0.4907	0.9960	0.9226
1.0730	0.2221	1.0000	0.9782
1.0730	0.1452	1.0530	0.3532

1.0730		1.0470	
1.0730	0.1780	1.0870	0.1762
1.0730	0.7478	1.2600	0.2190
1.0730	0.5119	1.2980	0.0614
1.0720	0.1077	0.9740	0.3448
1.0720		0.9920	
1.0720	0.1280	1.0010	0.9830
1.0720	0.5675	1.0250	0.6798
1.0720	0.3636	1.0960	0.3620
1.0720	0.4134	1.1260	0.1507
1.0720		1.1460	
1.0720		1.1490	
1.0720	0.3733	1.3000	0.0086
1.0720	0.7834	1.3740	0.2465
1.0710	0.3856	0.9990	0.9870
1.0710	0.1087	1.0210	0.5716
1.0710	0.8355	1.2860	0.4506
1.0700	0.3481	1.0540	0.4292
1.0700	0.1158	1.0670	0.1355
1.0700	0.1156	1.1010	0.0163
1.0700	0.4904	1.1170	0.2617
1.0700	0.5438	1.1330	0.2574
1.0700	0.5024	1.1360	0.1795
1.0690	0.4810	0.9770	0.8424
1.0690	0.0795	1.0060	0.8866
1.0690	0.5732	1.0680	0.5948
1.0690	0.4669	1.1240	0.1615
1.0690	0.1824	1.1990	0.0073
1.0690	0.3655	1.2640	0.0211
1.0690	0.6378	1.2600	0.1429
1.0680	0.0548	0.9700	0.8446
1.0680	0.0303	0.9850	0.4943
1.0680	0.0996	1.0280	0.5384

1.0680	0.0869	1.0640	0.1152
1.0680	0.1747	1.1400	0.0738
1.0680		1.1380	
1.0680	0.3321	1.1870	0.0075
1.0680	0.7836	1.2940	0.3277
1.0680	0.3913	1.2980	0.0137
1.0670	0.0229	0.9690	0.2941
1.0670	0.4435	0.9740	0.6230
1.0670	0.2112	0.9940	0.9623
1.0670	0.5424	1.0470	0.6422
1.0670	0.0435	1.0950	0.1186
1.0670	0.4144	1.0660	0.3050
1.0670	0.3527	1.0900	0.2853
1.0670	0.7594	1.1300	0.5500
1.0660	0.3956	0.9830	0.9146
1.0660		1.0120	
1.0660	0.1047	1.0070	0.7619
1.0660	0.1889	1.0370	0.4524
1.0660	0.1926	1.0690	0.1617
1.0660	0.5510	1.0740	0.5023
1.0660	0.3760	1.0460	0.5561
1.0660	0.2061	1.1060	0.2035
1.0660	0.2733	1.1300	0.1389
1.0660		1.2220	
1.0660	0.6128	1.2460	0.0932
1.0660	0.5295	1.2440	0.0892
1.0660	0.4015	1.2830	0.0088
1.0660	0.4223	1.2860	0.0862
1.0650	0.5289	0.9420	0.6405
1.0650	0.6290	0.9500	0.5453
1.0650	0.6035	1.0020	0.9831
1.0650	0.0455	1.0640	0.2994
1.0650		1.0940	

1.0650	0.2438	1.1490	0.0330
1.0640	0.4203	1.0600	0.4711
1.0640	0.2654	1.1290	0.0171
1.0640	0.8419	1.2110	0.4636
1.0640	0.7682	1.4890	0.0773
1.0630		0.9720	
1.0630	0.4940	1.1280	0.1974
1.0630	0.0921	1.0780	0.0438
1.0630	0.0967	1.0990	0.0404
1.0630	0.2512	1.1520	0.0398
1.0630	0.3495	1.2500	0.0036
1.0630		1.2960	
1.0620	0.5820	0.9650	0.6102
1.0620	0.4490	0.9920	0.8719
1.0620	0.3796	1.0400	0.4628
1.0620	0.2423	1.1590	0.0059
1.0620	0.6692	1.1420	0.3049
1.0620	0.4567	1.2040	0.0200
1.0620	0.6356	1.1830	0.2247
1.0610	0.5606	0.8060	0.2940
1.0610	0.1586	1.0410	0.4100
1.0610	0.7646	1.0850	0.6531
1.0610	0.1728	1.0800	0.0115
1.0610		1.0840	
1.0610	0.2699	1.1000	0.0824
1.0610	0.1471	1.1080	0.0340
1.0610	0.0644	1.1090	0.0080
1.0610		1.1070	
1.0610		1.1370	
1.0600	0.0093	0.9010	0.0407
1.0600	0.7255	0.9950	0.9340
1.0600	0.0689	1.0070	0.8066
1.0600	0.3506	1.0240	0.4887

1.0600	0.3011	1.0420	0.5572
1.0600	0.3367	1.0910	0.2666
1.0600		1.0960	
1.0600	0.4187	1.1440	0.1498
1.0600	0.2995	1.2380	0.0022
1.0600	0.7781	1.4110	0.0531
1.0600	0.5712	1.3960	0.0283
1.0590	0.6155	0.8970	0.9160
1.0590	0.7055	0.9590	0.7303
1.0590	0.4171	1.0230	0.6552
1.0590	0.3956	1.0700	0.4526
1.0590	0.3938	1.0580	0.1408
1.0590	0.4847	1.0660	0.3465
1.0590	0.5903	1.0940	0.4497
1.0580	0.6942	0.9840	0.8592
1.0580	0.1553	1.0710	0.2165
1.0580	0.5351	1.1090	0.2209
1.0580	0.7440	1.1700	0.2820
1.0570	0.5256	0.8870	0.3909
1.0570	0.4806	0.9470	0.4102
1.0570	0.5399	0.9410	0.6351
1.0570		1.0160	
1.0570	0.6888	1.0980	0.5710
1.0570		1.1100	
1.0570	0.4575	1.1410	0.0974
1.0570	0.5752	1.1820	0.1263
1.0560		0.9180	
1.0560		0.9880	
1.0560	0.5797	1.0200	0.8633
1.0560	0.2440	1.0720	0.0576
1.0560	0.8106	1.0650	0.7907
1.0560	0.6118	1.0810	0.4106
1.0560	0.3969	1.1270	0.0726

1.0560	0.7830	1.1220	0.4681
1.0560	0.1389	1.1220	0.0492
1.0560	0.8351	1.1650	0.5229
1.0550	0.2503	0.9430	0.3370
1.0550	0.4600	0.9560	0.6216
1.0550	0.2721	1.0420	0.1267
1.0550	0.0955	1.0270	0.6222
1.0550	0.1472	1.0660	0.0699
1.0550	0.0810	1.0820	0.1260
1.0550	0.4486	1.0740	0.3076
1.0550	0.8940	1.4220	0.2586
1.0540	0.6669	0.9850	0.8734
1.0540		1.0910	
1.0540	0.2086	1.0520	0.0670
1.0530	0.7320	0.8730	0.2618
1.0530	0.4870	1.0010	0.9734
1.0530	0.1025	1.0040	0.9104
1.0530	0.2348	1.0160	0.7489
1.0530	0.0585	1.0740	0.0064
1.0530	0.3648	1.0740	0.3376
1.0530	0.1046	1.0940	0.0190
1.0530	0.4799	1.1610	0.0789
1.0520	0.5146	0.9430	0.5617
1.0520	0.5948	0.9520	0.5080
1.0520	0.2142	0.9550	0.4295
1.0520	0.3936	0.9620	0.4706
1.0520	0.1130	1.0240	0.6595
1.0520		1.1670	
1.0510	0.3874	0.8140	0.0174
1.0510	0.0204	0.9500	0.5294
1.0510	0.3523	0.9550	0.1494
1.0510	0.6152	0.9940	0.9210
1.0510	0.1121	1.0400	0.4006

1.0510	0.6704	1.0540	0.5781
1.0510	0.2961	1.1030	0.0519
1.0510	0.8256	1.2170	0.2423
1.0500	0.7213	0.9460	0.4770
1.0500	0.4641	0.9690	0.6396
1.0500	0.2932	0.9890	0.7986
1.0500	0.5295	1.0080	0.9582
1.0500		1.0030	
1.0500	0.5895	1.0220	0.8384
1.0500	0.0921	1.0400	0.1512
1.0500	0.9472	1.0210	0.9746
1.0500	0.2621	1.0510	0.2987
1.0500	0.8294	1.1420	0.4926
1.0490	0.2128	0.9960	0.9530
1.0490	0.4352	1.0510	0.4380
1.0490	0.3791	1.0600	0.3709
1.0490	0.6819	1.0950	0.4001
1.0490		1.0900	
1.0490	0.8619	1.1380	0.5687
1.0490		1.1030	
1.0490	0.7172	1.4660	0.0305
1.0480	0.0702	1.0460	0.2393
1.0480	0.3667	1.0850	0.3213
1.0480	0.3318	1.0850	0.1472
1.0480	0.4860	1.0960	0.2348
1.0480	0.3912	1.1550	0.0649
1.0480	0.5858	1.2170	0.0304
1.0480	0.7239	1.2610	0.0667
1.0480	0.6597	1.2740	0.0182
1.0480	0.4364	1.3220	0.0061
1.0470	0.4727	0.9810	0.6856
1.0470	0.2074	1.0120	0.6534
1.0470	0.1588	1.0300	0.2322

1.0470		1.0240	
1.0470	0.4670	1.0570	0.3067
1.0470	0.4032	1.0630	0.3186
1.0470		1.0760	
1.0470	0.1492	1.1200	0.0791
1.0470	0.5635	1.2430	0.0245
1.0460		1.0170	
1.0460	0.0980	1.0360	0.2031
1.0460	0.5005	1.0660	0.4017
1.0460	0.6991	1.0470	0.6660
1.0460	0.6148	1.1580	0.0682
1.0460	0.7416	1.3240	0.1669
1.0450	0.0009	0.9840	0.5694
1.0450	0.5723	0.9970	0.9835
1.0450	0.5344	1.0240	0.7653
1.0450	0.3524	1.0640	0.2029
1.0450	0.2917	1.0690	0.1360
1.0440	0.1253	1.0170	0.5590
1.0440		1.1530	
1.0440	0.7232	1.4210	0.0169
1.0440	0.9248	1.5820	0.6174
1.0430	0.5238	1.0330	0.7549
1.0430	0.0472	1.0360	0.4027
1.0430	0.4409	1.0950	0.0869
1.0430	0.2527	1.0760	0.2408
1.0430	0.3196	1.0930	0.1202
1.0420	0.1236	1.0420	0.1641
1.0420	0.3230	1.0410	0.2611
1.0420	0.6122	1.0880	0.0350
1.0420	0.6502	1.1010	0.1830
1.0420	0.5631	1.0790	0.3090
1.0420	0.1966	1.0830	0.0737
1.0420	0.3364	1.1230	0.0211

1.0420	0.2767	1.1170	0.0064
1.0420	0.7656	1.1510	0.2676
1.0420	0.2633	1.1530	0.0043
1.0420	0.6348	1.2270	0.0504
1.0410	0.8721	0.9690	0.8218
1.0410	0.6112	1.0250	0.7886
1.0410	0.2142	1.0080	0.8311
1.0410	0.1908	1.0000	0.9645
1.0410	0.1935	1.0830	0.1847
1.0410	0.3120	1.1280	0.0574
1.0410		1.1130	
1.0410	0.6982	1.1460	0.2049
1.0410		1.2460	
1.0410	0.7547	1.3190	0.0389
1.0400	0.7270	0.8770	0.2418
1.0400		0.9370	
1.0400	0.6600	0.9590	0.7481
1.0400		1.0000	
1.0400	0.7423	1.0150	0.9108
1.0400	0.4482	1.0430	0.4627
1.0400	0.7191	1.2200	0.0999
1.0400	0.6315	1.2280	0.0120
1.0400	0.7992	1.3280	0.0681
1.0390	0.7885	0.8860	0.3834
1.0390	0.4809	0.9550	0.4698
1.0390	0.5158	1.0220	0.5766
1.0390		1.0570	
1.0390	0.3485	1.0710	0.0331
1.0390	0.9020	1.0850	0.7583
1.0390	0.7870	2.1800	0.1802
1.0380	0.4733	0.9150	0.1680
1.0380	0.7937	0.9030	0.3200
1.0380	0.5061	1.0420	0.4457

1.0380	0.0848	1.0240	0.2851
1.0380	0.2746	1.0910	0.1226
1.0370	0.7254	0.9440	0.8332
1.0370	0.1571	1.0040	0.9041
1.0370	0.0658	1.0630	0.1150
1.0370	0.4529	1.0320	0.6269
1.0370	0.4034	1.0270	0.6790
1.0370		1.1010	
1.0370	0.6721	1.1230	0.2832
1.0370	0.7352	1.1120	0.2205
1.0370		1.3860	
1.0360	0.5974	0.8700	0.3093
1.0360	0.3259	1.0250	0.3213
1.0360	0.6056	1.0170	0.7732
1.0360	0.5823	1.0590	0.3455
1.0360	0.5645	1.0430	0.1939
1.0360	0.7486	1.0780	0.4834
1.0360	0.3668	1.0820	0.1952
1.0360	0.5632	1.0810	0.2660
1.0360	0.5467	1.1220	0.0211
1.0350		0.9450	
1.0350	0.5893	1.0470	0.3771
1.0350	0.7078	1.0740	0.5067
1.0350	0.3801	1.0640	0.0146
1.0350	0.6705	1.1450	0.1959
1.0350	0.8284	1.1880	0.4371
1.0340	0.9073	0.8280	0.1683
1.0340	0.6943	0.8670	0.2739
1.0340	0.5432	1.1050	0.0481
1.0340		1.1170	
1.0340	0.6065	1.1740	0.0381
1.0330	0.6019	0.8960	0.2145
1.0330	0.7749	0.9750	0.7453

1.0330		1.0300	
1.0330	0.7119	1.0590	0.5040
1.0330	0.6599	1.0620	0.4530
1.0320		0.7970	
1.0320	0.9197	0.8500	0.2184
1.0320		1.0040	
1.0320	0.5891	1.0370	0.5152
1.0320	0.7526	1.0190	0.8742
1.0320	0.7254	1.0400	0.6563
1.0320		1.0490	
1.0320	0.2452	1.0240	0.4671
1.0320	0.6989	1.2360	0.0255
1.0310	0.7367	0.7320	0.0564
1.0310	0.6203	0.9460	0.2220
1.0310		0.9620	
1.0310		1.0490	
1.0310	0.4599	1.0460	0.3079
1.0310	0.3031	1.0400	0.2580
1.0310	0.4244	1.0430	0.2928
1.0310	0.4783	1.1210	0.1343
1.0310		1.1030	
1.0310		1.3550	
1.0300	0.3634	0.9210	0.2407
1.0300	0.6035	0.8980	0.2237
1.0300	0.6911	0.9930	0.9781
1.0300		1.0050	
1.0300	0.5794	1.0160	0.7498
1.0300	0.4495	1.0050	0.8874
1.0300	0.6921	1.0240	0.7420
1.0300	0.7349	1.0690	0.5215
1.0300		1.0560	
1.0300	0.4683	1.1640	0.0176
1.0300		1.2170	

1.0290	0.6774	0.9320	0.2226
1.0290	0.3833	1.0000	0.9902
1.0290		1.0150	
1.0290	0.4713	1.0290	0.5422
1.0290	0.4628	1.0320	0.2818
1.0280	0.8499	0.8360	0.1107
1.0280	0.6293	0.9420	0.0870
1.0280	0.5277	0.9630	0.4551
1.0280		0.9780	
1.0280	0.4320	1.0200	0.5686
1.0280	0.4559	1.0280	0.6601
1.0280	0.5730	1.0320	0.5142
1.0280	0.8764	1.0440	0.7817
1.0280	0.5940	1.0950	0.0873
1.0280	0.9071	1.3370	0.1991
1.0280	0.8764	1.3530	0.0493
1.0270	0.7009	1.0020	0.9929
1.0270	0.7938	1.0870	0.4154
1.0270	0.6274	1.0880	0.0768
1.0270	0.9446	1.1370	0.6687
1.0270	0.6599	1.1320	0.0427
1.0270	0.6378	1.1900	0.0264
1.0270	0.9519	1.2540	0.3276
1.0260	0.6720	0.8770	0.2021
1.0260	0.4118	1.0270	0.2389
1.0260	0.0759	1.0330	0.3811
1.0260	0.5321	1.0120	0.6992
1.0260	0.8990	1.0610	0.7012
1.0260	0.4565	1.0900	0.0234
1.0250	0.9395	0.8710	0.3687
1.0250	0.9033	0.8780	0.2387
1.0250	0.7150	0.9210	0.3071
1.0250	0.8098	0.9770	0.8681

1.0250		1.0040	
1.0250	0.6219	1.1140	0.0924
1.0250	0.7224	1.0420	0.5614
1.0250	0.6095	1.1160	0.0166
1.0250	0.8513	1.1940	0.3384
1.0250	0.7583	1.1810	0.0674
1.0250	0.8323	1.2560	0.0372
1.0240		0.8740	
1.0240	0.7289	0.8560	0.4581
1.0240	0.7963	0.8880	0.1658
1.0240	0.3709	1.0460	0.1318
1.0230	0.6443	0.9070	0.0180
1.0230	0.4929	1.0160	0.7081
1.0230	0.7222	1.0310	0.6036
1.0230		1.2610	
1.0220	0.9044	0.9040	0.3103
1.0220	0.6954	0.9440	0.5282
1.0220	0.5640	0.9990	0.9495
1.0220	0.4137	1.0160	0.7097
1.0220	0.7420	1.0570	0.5530
1.0220	0.6310	1.1090	0.0251
1.0220	0.8132	1.1320	0.2091
1.0210	0.7877	0.9550	0.4795
1.0210	0.6709	0.9900	0.9128
1.0210	0.8148	1.0570	0.5036
1.0210		1.0570	
1.0210	0.3191	1.0620	0.0887
1.0210	0.8658	1.0890	0.3954
1.0210	0.7308	1.1070	0.1007
1.0210	0.6442	1.1470	0.0027
1.0200	0.8866	0.9620	0.9540
1.0200	0.8935	1.0080	0.9723
1.0200	0.7866	1.0710	0.3580

1.0190	0.8493	0.8480	0.4752
1.0190	0.7577	0.9050	0.4402
1.0190	0.8299	0.9380	0.4602
1.0190	0.4613	0.9840	0.5425
1.0190	0.7904	1.0420	0.5391
1.0190	0.7904	1.0450	0.4829
1.0190	0.7232	1.0870	0.0397
1.0190	0.7938	1.1050	0.0851
1.0190	0.7777	1.1450	0.0647
1.0190	0.8801	1.1470	0.1879
1.0190	0.7432	1.1410	0.0460
1.0190	0.8551	1.3150	0.0361
1.0180	0.7992	0.8860	0.0920
1.0180		0.9030	
1.0180	0.6443	0.8890	0.0082
1.0180		0.9720	
1.0180	0.8265	0.9870	0.7720
1.0180	0.7172	1.0570	0.3659
1.0180	0.7366	1.0520	0.3526
1.0180	0.7283	1.1440	0.0451
1.0180	0.9282	1.2190	0.1417
1.0170		0.8220	
1.0170	0.9116	1.0110	0.9307
1.0170	0.8335	1.0060	0.9637
1.0170	0.4927	1.0270	0.5102
1.0170	0.8296	1.0430	0.6448
1.0170	0.5937	1.0500	0.0549
1.0170	0.9670	1.2640	0.2318
1.0170	0.8115	1.2620	0.0109
1.0160		0.9460	
1.0160	0.6903	0.9840	0.8061
1.0160		0.9860	
1.0160	0.7416	1.0550	0.1451

1.0160	0.8098	1.0260	0.6755
1.0160	0.7612	1.1140	0.1522
1.0160	0.8556	1.1340	0.1112
1.0150		0.9430	
1.0150	0.8347	0.9520	0.3627
1.0150	0.8242	0.9370	0.3448
1.0150		0.9590	
1.0150	0.7120	0.9670	0.4028
1.0150	0.8429	1.0100	0.8926
1.0150	0.8974	0.9930	0.8560
1.0150	0.6907	1.0160	0.8037
1.0150		1.0390	
1.0150	0.8041	1.0760	0.2199
1.0150		1.0900	
1.0150	0.8044	1.1240	0.1545
1.0150	0.7647	1.1320	0.0710
1.0140	0.7840	0.9340	0.0260
1.0140	0.6509	0.9850	0.6890
1.0140	0.3990	0.9930	0.5009
1.0140	0.7920	0.9960	0.8930
1.0140	0.7175	1.0050	0.9039
1.0140	0.7634	1.0050	0.9127
1.0140	0.9911	1.0380	0.8638
1.0140	0.6699	1.0390	0.2246
1.0140	0.6285	1.0600	0.1047
1.0140	0.7184	1.1180	0.0677
1.0140	0.8649	1.1410	0.1893
1.0140	0.8409	1.1630	0.0409
1.0130	0.9143	0.9680	0.7286
1.0130	0.8787	0.9780	0.7757
1.0130	0.6089	1.0180	0.5106
1.0130		1.0710	
1.0130	0.9193	1.3490	0.0150

1.0120	0.7533	0.8930	0.2316
1.0120	0.8934	0.9020	0.1257
1.0120	0.7979	0.9550	0.5975
1.0120	0.8806	0.9820	0.7376
1.0120	0.8364	1.0610	0.2138
1.0120		1.0780	
1.0120	0.9911	1.1350	0.3344
1.0120		1.2310	
1.0120	0.9405	1.2430	0.1308
1.0120	0.9821	1.2970	0.0656
1.0120		1.3290	
1.0110	0.9773	0.9820	0.7756
1.0110	0.7863	0.9920	0.9259
1.0110	0.5450	0.9950	0.7935
1.0110		1.0220	
1.0110	0.8904	1.1500	0.1132
1.0110		1.1390	
1.0110	0.8956	1.2460	0.0326
1.0100	0.8536	0.9050	0.2051
1.0100		0.9520	
1.0100	0.8689	0.9920	0.8609
1.0100	0.9241	1.0090	0.9171
1.0100	0.6777	1.0350	0.2741
1.0100	0.7277	1.0660	0.2822
1.0100	0.9080	1.0700	0.2639
1.0100	0.9396	1.1870	0.2774
1.0100	0.9740	1.2380	0.0919
1.0090	0.9332	0.9400	0.4660
1.0090	0.7365	0.9680	0.2503
1.0090	0.8723	1.0270	0.5425
1.0090	0.9251	1.0110	0.8937
1.0090	0.9416	1.0740	0.4327
1.0090	0.9348	1.4420	0.0520

1.0090	0.8854	1.8790	0.0438
1.0080	0.8341	0.9830	0.7549
1.0080	0.8517	0.9720	0.5011
1.0080	0.8280	0.9910	0.8539
1.0080	0.8651	1.0130	0.8110
1.0080	0.8749	1.0570	0.2213
1.0080	0.8363	1.0830	0.0600
1.0080	0.9048	1.0620	0.2431
1.0080	0.8167	1.0690	0.0918
1.0080	0.9196	1.1300	0.3230
1.0070	0.8816	0.9370	0.1576
1.0070	0.8814	0.9500	0.6709
1.0070		0.9960	
1.0070	0.6853	1.0230	0.2801
1.0070		1.0290	
1.0070		1.0570	
1.0070	0.9657	1.1200	0.7609
1.0060	0.9678	0.9080	0.1652
1.0060	0.9806	0.9650	0.7566
1.0060	0.8600	0.9970	0.9691
1.0060	0.8817	1.0040	0.9212
1.0060		1.0160	
1.0060	0.9247	1.0420	0.5816
1.0060	0.8515	1.0450	0.4848
1.0060	0.9884	1.0690	0.5968
1.0060	0.8859	1.1020	0.1063
1.0060	0.8703	1.0880	0.0338
1.0050	0.8590	0.9170	0.0860
1.0050	0.9398	0.9230	0.8386
1.0050		0.9500	
1.0050	0.9596	0.9650	0.5472
1.0050	0.9287	1.1100	0.1317
1.0050	0.9419	1.1420	0.1118

1.0050	0.9457	1.1120	0.0932
1.0040	0.9035	0.8840	0.0197
1.0040	0.8108	0.9390	0.2163
1.0040	0.9721	1.0150	0.8023
1.0040	0.7755	1.0310	0.2444
1.0040	0.9090	1.0600	0.2853
1.0040		1.0500	
1.0040	0.9999	1.1190	0.3284
1.0040	0.8640	1.1990	0.0736
1.0040		1.1380	
1.0030	0.9363	0.9430	0.1546
1.0030	0.9251	0.9800	0.5099
1.0030	0.9314	1.0390	0.5094
1.0030	0.9538	0.9890	0.9253
1.0030	0.9306	1.0010	0.9901
1.0030	0.9194	1.0090	0.7612
1.0030	0.9439	1.0100	0.4800
1.0030	0.9540	1.0210	0.6320
1.0030		1.0270	
1.0030	0.9602	1.0450	0.6763
1.0030	0.9450	1.0460	0.4787
1.0030	0.9543	1.1820	0.2303
1.0020	0.9731	0.9250	0.1455
1.0020	0.9091	0.9470	0.4486
1.0020	0.9973	0.9560	0.5669
1.0020	0.9968	0.9910	0.9227
1.0020	0.9775	1.0000	0.9797
1.0020	0.9598	1.0030	0.9421
1.0020	0.9616	1.0440	0.3685
1.0010	0.9479	0.8990	0.1065
1.0010	0.9601	0.8950	0.1795
1.0010	0.9933	0.9330	0.1497
1.0010	0.9660	0.9220	0.2692

1.0010	0.9516	0.9440	0.4486
1.0010	0.9503	0.9640	0.5918
1.0010	0.9814	0.9690	0.4925
1.0010	0.9415	1.0180	0.3146
1.0010	0.9984	1.0040	0.9918
1.0010	0.9972	1.0260	0.6123
1.0010	0.9929	1.0100	0.8772
1.0010	0.8839	1.0360	0.9000
1.0010	0.9840	1.0400	0.2488
1.0010	0.9708	1.0330	0.5464
1.0010		1.0720	
1.0010	0.9726	1.0740	0.2094
1.0010	0.9971	1.0750	0.1652
1.0010	0.9893	1.1200	0.1453
1.0010	0.9666	1.2040	0.3482
1.0010	0.9917	1.2180	0.3027
1.0000	0.9465	0.9660	0.7830
1.0000	0.9174	0.9850	0.7812
1.0000	0.9537	1.0440	0.5424
1.0000	0.9547	1.0650	0.3986
1.0000	0.8836	1.1390	0.4913
1.0000		1.1380	
1.0000	0.9852	1.1910	0.0604
1.0000	0.9883	1.3300	0.0037
0.9990	0.9550	0.9410	0.3383
0.9990	0.9922	0.9520	0.4589
0.9990	0.9372	0.9570	0.7306
0.9990	0.9956	0.9450	0.6666
0.9990	0.9663	0.9700	0.9271
0.9990	0.9741	0.9870	0.6895
0.9990	0.9715	1.0050	0.8810
0.9990		1.0180	
0.9990	0.9878	1.0170	0.5912

0.9990	0.9745	1.0330	0.5869
0.9990	0.9802	1.0400	0.2762
0.9990		1.1280	
0.9990	0.9350	1.0910	0.2030
0.9990	0.9393	1.0880	0.1792
0.9990	0.9767	1.1180	0.1455
0.9980		0.8620	
0.9980	0.9302	0.9280	0.7301
0.9980	0.9641	0.9380	0.1960
0.9980	0.9359	0.9790	0.7385
0.9980	0.9510	0.9700	0.5868
0.9980	0.9605	0.9680	0.2112
0.9980	0.8752	1.0750	0.5980
0.9980	0.9376	1.1890	0.0772
0.9970	0.8984	1.0200	0.8432
0.9970	0.9994	0.9870	0.9637
0.9970	0.9605	1.0430	0.5276
0.9970	0.8453	1.4430	0.1036
0.9960	0.9608	0.8230	0.4168
0.9960	0.9063	0.9610	0.4875
0.9960	0.9707	1.0130	0.7531
0.9960	0.8964	1.0360	0.3596
0.9960	0.9537	1.0980	0.4247
0.9960		1.1580	
0.9950	0.9490	0.7480	0.0442
0.9950	0.9056	0.8950	0.1904
0.9950		0.9580	
0.9950	0.7660	0.9900	0.6871
0.9950	0.8313	1.0310	0.3572
0.9950	0.9544	1.0570	0.3597
0.9950	0.9480	1.0370	0.4513
0.9950	0.9168	1.0500	0.6457
0.9950	0.9953	1.0760	0.2788

0.9950		1.1790	
0.9940		0.8180	
0.9940	0.9614	0.8440	0.0459
0.9940	0.8706	0.9710	0.4199
0.9940	0.8806	0.9640	0.7115
0.9940	0.8469	0.9750	0.6522
0.9940	0.8690	0.9680	0.5644
0.9940	0.9013	0.9900	0.7068
0.9940	0.8676	1.0510	0.5229
0.9940	0.8818	1.3100	0.0452
0.9930	0.9262	0.9930	0.9083
0.9930	0.8642	0.9980	0.9958
0.9930	0.9221	1.0030	0.9569
0.9930	0.8540	1.0510	0.3510
0.9930	0.9361	1.0980	0.1656
0.9930	0.9237	1.1260	0.1350
0.9920	0.9839	0.8080	0.5357
0.9920	0.9101	0.9290	0.2862
0.9920	0.5367	0.9590	0.0299
0.9920	0.8787	0.9890	0.7585
0.9920	0.8253	1.0190	0.4114
0.9920	0.7683	1.0560	0.1823
0.9920		1.1260	
0.9920	0.9660	1.2110	0.1278
0.9920	0.9943	1.3010	0.0422
0.9910	0.9770	0.9300	0.7341
0.9910	0.7151	0.9460	0.2277
0.9910	0.6628	0.9940	0.9277
0.9910	0.9438	1.0090	0.9821
0.9910	0.6060	1.0160	0.4881
0.9910	0.5418	1.0030	0.7703
0.9910	0.6584	1.0180	0.4200
0.9910		1.0570	

0.9910	0.9227	1.0700	0.0407
0.9910	0.8773	1.1390	0.3613
0.9910	0.8617	1.1960	0.1931
0.9910	0.8708	1.2880	0.0902
0.9900	0.9878	0.8690	0.4955
0.9900	0.9551	0.8660	0.2734
0.9900	0.8449	0.9840	0.7771
0.9900	0.9014	0.9410	0.3834
0.9900	0.5539	0.9650	0.4359
0.9900	0.6241	0.9920	0.8253
0.9900		1.0860	
0.9900	0.7326	1.1270	0.1230
0.9900	0.7738	1.1460	0.0208
0.9890	0.9767	0.8490	0.1387
0.9890	0.8683	0.9500	0.3748
0.9890	0.8748	1.0180	0.6902
0.9890	0.7824	1.0350	0.4653
0.9890	0.8933	1.0230	0.5512
0.9890	0.7189	1.0270	0.3590
0.9890	0.7066	1.1090	0.2843
0.9890	0.8560	1.1220	0.0822
0.9890	0.8875	1.2910	0.4339
0.9890		1.2500	
0.9880	0.9074	0.8100	0.2589
0.9880	0.8022	0.9590	0.5287
0.9880	0.8122	1.0040	0.9823
0.9880	0.7796	0.9730	0.5036
0.9880	0.6429	0.9790	0.3922
0.9880		1.0800	
0.9880	0.9155	1.0310	0.7040
0.9880	0.9089	1.1550	0.2516
0.9870	0.7368	0.9470	0.2601
0.9870	0.7907	0.9430	0.1359

0.9870		0.9600	
0.9870	0.8193	0.9470	0.4632
0.9870	0.6894	0.9590	0.0269
0.9870	0.8342	0.9660	0.6761
0.9870	0.9538	1.0050	0.9098
0.9870	0.8530	1.0930	0.6977
0.9870		1.0990	
0.9860	0.8065	0.9500	0.3432
0.9860	0.8535	0.9830	0.8389
0.9860	0.8259	0.9710	0.6604
0.9860	0.5961	0.9920	0.8079
0.9860	0.5859	1.0010	0.9648
0.9860	0.7149	0.9980	0.9675
0.9860	0.6505	1.1030	0.0761
0.9860		1.1090	
0.9860	0.8614	1.1880	0.2039
0.9850	0.6853	0.9060	0.0794
0.9850	0.9243	0.9640	0.5608
0.9850	0.5877	0.9940	0.8353
0.9850		1.0190	
0.9850	0.6243	1.0680	0.1388
0.9850	0.5782	1.1440	0.0210
0.9850	0.8744	1.2780	0.2265
0.9840	0.8005	0.8950	0.1633
0.9840	0.8185	0.9360	0.4237
0.9840	0.8141	0.9380	0.3921
0.9840	0.8120	1.0240	0.8679
0.9840	0.8719	1.0930	0.6616
0.9830		0.8470	
0.9830	0.7570	0.9130	0.1881
0.9830	0.7093	0.9320	0.5659
0.9830	0.8619	0.9530	0.4946
0.9830	0.8000	0.9820	0.8251

0.9830	0.8721	1.0040	0.9815
0.9830	0.8729	1.0280	0.6611
0.9830	0.6674	1.0440	0.6570
0.9830		1.0670	
0.9830	0.8334	1.1020	0.2924
0.9820		0.9350	
0.9820	0.8099	0.9040	0.1473
0.9820	0.2926	0.9120	0.1182
0.9820	0.8095	0.9490	0.5435
0.9820		0.9970	
0.9820	0.8139	1.0000	0.9981
0.9820	0.8702	1.0570	0.5531
0.9820	0.7963	1.0500	0.2600
0.9820	0.5760	1.1090	0.0369
0.9810	0.7421	0.9880	0.8350
0.9810	0.8631	0.9690	0.7761
0.9810	0.6420	1.0460	0.5019
0.9810	0.7829	1.0690	0.3401
0.9810		1.1160	
0.9810	0.7203	1.1800	0.3577
0.9800		0.9870	
0.9800	0.3078	1.0140	0.7946
0.9800	0.8583	1.0220	0.9514
0.9800	0.8011	1.0810	0.4582
0.9800		1.3060	
0.9790		0.9640	
0.9790	0.4638	1.0060	0.8614
0.9790	0.2910	1.0770	0.0972
0.9790	0.6818	1.0740	0.1116
0.9790	0.7405	1.1250	0.1832
0.9780		0.8140	
0.9780	0.8584	0.9260	0.6536
0.9780		0.9650	

0.9780	0.0614	0.9480	0.1479
0.9780		1.0340	
0.9780	0.4110	1.0410	0.4139
0.9780		1.0510	
0.9780	0.8533	1.0720	0.4252
0.9780	0.8347	1.1150	0.5839
0.9780	0.6971	1.1360	0.0634
0.9770		0.9290	
0.9770		0.9480	
0.9770	0.8922	0.9690	0.7676
0.9770	0.7489	0.9700	0.5650
0.9770	0.6320	0.9920	0.8734
0.9770	0.2441	1.0100	0.6291
0.9770	0.7927	0.9930	0.9672
0.9770	0.5091	1.0210	0.5440
0.9770	0.7434	1.0150	0.8901
0.9770	0.7928	1.0620	0.6039
0.9770	0.7858	1.0930	0.5044
0.9770		1.1240	
0.9770	0.7183	1.1530	0.0668
0.9770	0.7835	1.2490	0.0399
0.9770	0.9419	1.2420	0.0613
0.9760	0.7292	0.9200	0.4235
0.9760	0.5981	0.9760	0.5953
0.9760		0.9860	
0.9760	0.9316	0.9820	0.7803
0.9760	0.7388	0.9890	0.7941
0.9760	0.8647	1.0010	0.9830
0.9760	0.6277	1.0080	0.9038
0.9760		1.8400	
0.9750		0.8320	
0.9750	0.7131	0.9930	0.9255
0.9750	0.2120	0.9690	0.5245

0.9750	0.3501	1.0000	0.9926
0.9750	0.4784	0.9810	0.3021
0.9750	0.6019	1.0280	0.6250
0.9750	0.6825	0.9970	0.9329
0.9750	0.7569	1.0780	0.7160
0.9750	0.6899	1.1090	0.3942
0.9740	0.6992	0.8450	0.0352
0.9740	0.5581	0.8830	0.0602
0.9740	0.6345	0.9930	0.8689
0.9740		1.0130	
0.9740		1.0180	
0.9740	0.5865	1.0230	0.6229
0.9740	0.5222	1.0260	0.6028
0.9740	0.7960	1.0640	0.4531
0.9740	0.8050	1.1090	0.5147
0.9730	0.8649	0.6690	0.0239
0.9730	0.7345	0.8280	0.5752
0.9730	0.4893	0.8670	0.0247
0.9730		0.9740	
0.9730		1.0290	
0.9730	0.8121	1.0140	0.9468
0.9730	0.9289	1.0130	0.9359
0.9730	0.7595	1.0300	0.6063
0.9730	0.7988	1.1610	0.4718
0.9720	0.5194	0.9140	0.1065
0.9720		0.9060	
0.9720	0.5928	0.9930	0.9191
0.9720	0.7531	0.9800	0.9060
0.9720	0.2756	1.0010	0.9672
0.9720	0.7113	1.0330	0.7067
0.9720	0.4979	1.0550	0.3790
0.9710	0.5803	0.9130	0.3064
0.9710		0.9320	

0.9710	0.6834	0.9620	0.7493
0.9710	0.8014	0.9600	0.7908
0.9710	0.2657	0.9710	0.5631
0.9710	0.5472	0.9720	0.4513
0.9710	0.5666	0.9690	0.5369
0.9710	0.6520	1.0140	0.7955
0.9710	0.6465	1.0280	0.6842
0.9710	0.2183	1.0250	0.0898
0.9710	0.6480	1.0760	0.3711
0.9700		0.8060	
0.9700	0.7334	0.9400	0.4618
0.9700	0.7465	1.0080	0.8837
0.9700	0.3444	1.0280	0.4892
0.9700		1.0280	
0.9700		1.0440	
0.9690	0.2683	0.9220	0.2270
0.9690		0.9490	
0.9690	0.4175	0.9550	0.1354
0.9690	0.4777	0.9790	0.5153
0.9690	0.4197	0.9800	0.7273
0.9690		0.9970	
0.9690	0.6292	0.9220	0.3040
0.9690	0.4794	1.0450	0.0950
0.9690	0.9471	1.1080	0.4077
0.9680	0.4681	0.8570	0.1028
0.9680	0.5298	0.9000	0.1464
0.9680	0.6087	0.9330	0.3970
0.9680	0.4686	0.9590	0.5151
0.9680	0.6371	0.9590	0.3816
0.9680	0.5519	0.9830	0.7082
0.9680	0.7814	1.0090	0.9331
0.9680	0.8085	1.0120	0.9488
0.9680	0.1585	1.0520	0.2192

0.9680		1.0880	
0.9680	0.8606	1.1340	0.4842
0.9680	0.6922	1.1400	0.4288
0.9680		1.1700	
0.9680		1.2630	
0.9670	0.6840	0.8690	0.1451
0.9670	0.5660	0.9280	0.1749
0.9670	0.7244	0.9080	0.1069
0.9670	0.5829	0.9160	0.2768
0.9670	0.4408	0.9100	0.3222
0.9670	0.5937	0.9500	0.3418
0.9670	0.4056	0.9530	0.2228
0.9670	0.2903	0.9620	0.1540
0.9670		0.9670	
0.9670	0.1659	0.9720	0.3513
0.9670	0.9362	0.9660	0.8527
0.9670	0.8824	0.9820	0.7480
0.9670	0.6970	1.0840	0.5820
0.9670	0.8248	1.1150	0.7487
0.9670	0.6010	1.1920	0.0470
0.9670	0.8186	1.2470	0.1198
0.9660		0.6240	
0.9660		0.8230	
0.9660	0.5440	0.8920	0.1270
0.9660	0.6949	0.9000	0.2308
0.9660	0.2319	1.0450	0.1895
0.9660	0.1046	1.0450	0.2080
0.9650	0.6445	0.8510	0.5221
0.9650	0.6599	0.8630	0.0985
0.9650	0.6124	0.8940	0.1227
0.9650	0.6489	0.8550	0.0708
0.9650	0.4225	0.9080	0.0229
0.9650	0.5613	0.9430	0.5330

0.9650	0.3980	0.9630	0.6183
0.9650	0.5968	0.9810	0.7784
0.9650	0.4521	1.0090	0.8405
0.9650	0.0553	1.0270	0.5652
0.9650		1.1680	
0.9640	0.6221	0.8550	0.2053
0.9640	0.5375	0.9020	0.1006
0.9640		1.0030	
0.9640		1.0290	
0.9640	0.3959	1.0360	0.3223
0.9640	0.1330	1.0680	0.0825
0.9640	0.6619	1.0420	0.6576
0.9640	0.5632	1.0690	0.4526
0.9640	0.3983	1.0940	0.3073
0.9630		0.8010	
0.9630	0.2487	0.9290	0.0440
0.9630	0.3482	0.9880	0.5429
0.9630	0.5901	0.9890	0.8193
0.9630		0.9900	
0.9630	0.6239	0.9830	0.8553
0.9630	0.7538	1.0020	0.9497
0.9630	0.7327	1.0520	0.7223
0.9630		1.0670	
0.9630		1.3900	
0.9620	0.6375	0.8750	0.1152
0.9620	0.7814	0.9160	0.4432
0.9620	0.7772	0.9090	0.2557
0.9620		1.0360	
0.9620	0.5740	1.0600	0.4007
0.9620	0.1495	1.0580	0.3282
0.9620	0.3441	1.0840	0.0681
0.9610	0.9427	0.8830	0.2221
0.9610	0.8332	0.8810	0.4501

0.9610	0.5262	0.9700	0.6672
0.9610		1.0140	
0.9610	0.6828	1.0190	0.7801
0.9610	0.6756	1.1690	0.3688
0.9610	0.8068	1.2470	0.0779
0.9610	0.7165	1.2800	0.1142
0.9610	0.8052	1.5200	0.4480
0.9600	0.7162	0.8400	0.2652
0.9600	0.3414	0.9370	0.1946
0.9600	0.3426	0.9960	0.9894
0.9600	0.6525	0.9920	0.9250
0.9600		1.1920	
0.9600	0.4955	1.2120	0.0402
0.9590		0.8860	
0.9590	0.3154	0.8710	0.0140
0.9590	0.7357	0.8920	0.3753
0.9590	0.5138	0.9900	0.9439
0.9590	0.4611	0.9930	0.9626
0.9590	0.7226	0.9980	0.9737
0.9590	0.7164	1.0300	0.7761
0.9590		1.1780	
0.9590		1.3450	
0.9580	0.7231	0.8090	0.1012
0.9580	0.6189	0.8460	0.0837
0.9580	0.1920	0.8910	0.0894
0.9580		0.9330	
0.9580	0.0639	0.9600	0.0549
0.9580	0.6374	0.9860	0.8578
0.9580	0.4145	1.0030	0.9742
0.9580		1.0580	
0.9580	0.5184	1.0060	0.9849
0.9580	0.7988	1.0750	0.5258
0.9580	0.3715	1.1010	0.0103

0.9570	0.2344	0.8920	0.0440
0.9570	0.4840	0.9200	0.3632
0.9570	0.5003	0.9780	0.8216
0.9570	0.3451	0.9850	0.7011
0.9570	0.7143	0.9990	0.9903
0.9570	0.2230	1.0260	0.4806
0.9560	0.6760	0.8520	0.0557
0.9560	0.5606	0.8590	0.0603
0.9560	0.4829	0.9340	0.3564
0.9560	0.4748	0.9510	0.4047
0.9560	0.4422	0.9500	0.2127
0.9560	0.4953	1.0720	0.3062
0.9560		1.3290	
0.9550		0.8950	
0.9550		0.9310	
0.9550		0.9370	
0.9550	0.0512	0.9420	0.1507
0.9550	0.4619	1.0960	0.0886
0.9550	0.6019	1.0410	0.5593
0.9550		1.1360	
0.9550	0.5493	1.3120	0.0589
0.9540	0.5207	0.8430	0.0446
0.9540	0.5851	0.8710	0.1029
0.9540	0.1459	0.9480	0.1025
0.9540	0.3567	0.9680	0.5702
0.9540	0.5835	0.9740	0.7582
0.9540	0.0669	0.9800	0.1705
0.9540	0.3757	1.0660	0.2203
0.9540	0.4297	1.1160	0.1709
0.9540	0.4607	1.1410	0.2142
0.9540	0.5381	1.1980	0.2373
0.9530	0.3999	0.7850	0.0082
0.9530	0.5404	0.8700	0.2337

0.9530	0.3959	0.9090	0.1186
0.9530	0.7608	0.9440	0.6029
0.9530	0.1478	0.9370	0.1006
0.9530	0.6234	0.9480	0.6174
0.9530	0.5517	0.9820	0.8317
0.9530		1.0000	
0.9530	0.6246	0.9860	0.9602
0.9530	0.2277	0.9950	0.9569
0.9530	0.4876	1.0960	0.2029
0.9530		1.0950	
0.9530	0.8746	1.1350	0.3049
0.9530	0.8130	1.6380	0.2798
0.9520	0.6227	0.8720	0.3002
0.9520	0.2428	0.8950	0.0064
0.9520	0.2694	0.9320	0.1543
0.9520	0.3769	0.9670	0.5602
0.9520	0.5528	0.9650	0.5195
0.9520	0.4075	1.0370	0.5902
0.9520	0.3133	1.0320	0.5807
0.9520	0.4143	1.0770	0.2153
0.9520	0.5602	1.1790	0.1947
0.9510	0.5840	0.7990	0.1392
0.9510		0.8730	
0.9510		0.8970	
0.9510	0.3254	0.8860	0.0343
0.9510	0.3162	0.9560	0.0460
0.9510	0.4230	0.9640	0.5428
0.9510	0.0205	0.9900	0.6835
0.9510		1.0110	
0.9510		1.0350	
0.9510		1.0660	
0.9510		1.1260	
0.9510	0.4938	1.2280	0.0985

0.9500	0.4845	0.7460	0.0126
0.9500	0.4526	0.8340	0.0045
0.9500	0.6919	0.9300	0.5278
0.9500		0.9430	
0.9500	0.5605	0.9480	0.4968
0.9500	0.2844	0.9740	0.6154
0.9500	0.1708	1.0010	0.9880
0.9500	0.3651	1.0250	0.6568
0.9500	0.6500	1.1830	0.5185
0.9500	0.6353	1.2120	0.2566
0.9500	0.5845	1.2460	0.1202
0.9490	0.4199	0.8070	0.0250
0.9490	0.3404	0.8740	0.0297
0.9490	0.3330	0.9370	0.1237
0.9490	0.4729	0.9680	0.5613
0.9490	0.3294	1.0520	0.4313
0.9490	0.7601	1.2190	0.4740
0.9480	0.3849	0.7770	0.0504
0.9480	0.7019	0.8710	0.3377
0.9480	0.3043	0.8870	0.1765
0.9480	0.1554	0.9130	0.0223
0.9480	0.2849	0.9170	0.3510
0.9480	0.1389	0.9370	0.2316
0.9480	0.3981	0.9780	0.6303
0.9480	0.0846	0.9830	0.6840
0.9480	0.3023	0.9880	0.8152
0.9480	0.7806	1.0450	0.9167
0.9480	0.4023	1.1200	0.0210
0.9470	0.1617	0.8960	0.0356
0.9470	0.5803	0.9150	0.4003
0.9470		0.9650	
0.9470	0.4108	0.9540	0.7003
0.9470	0.7726	0.9770	0.8587

0.9470	0.5542	1.0510	0.4577
0.9470	0.2817	1.0180	0.7126
0.9470	0.1367	0.9950	0.8721
0.9470		1.1940	
0.9470	0.5907	1.4570	0.0565
0.9460	0.4455	0.8740	0.1015
0.9460	0.4488	0.8880	0.1467
0.9460	0.3784	0.8720	0.0524
0.9460	0.1058	0.9400	0.0734
0.9460	0.0913	0.9400	0.0627
0.9460	0.1296	0.9720	0.4425
0.9460	0.5578	0.9620	0.5929
0.9460	0.1707	0.9660	0.3877
0.9460	0.7399	1.1580	0.4606
0.9460	0.6804	1.2920	0.3464
0.9450	0.4161	0.8930	0.1835
0.9450		0.9320	
0.9450	0.0856	0.9390	0.0469
0.9450	0.1735	0.9480	0.1945
0.9450	0.3362	1.0040	0.8833
0.9450		1.0070	
0.9450	0.4606	1.0020	0.9680
0.9450		1.0240	
0.9450	0.6726	1.0100	0.9305
0.9450	0.1957	1.0580	0.2735
0.9450	0.7599	1.2800	0.0499
0.9440	0.4098	0.8610	0.0134
0.9440	0.0314	0.9030	0.3701
0.9440	0.1825	0.9350	0.1021
0.9440	0.1307	0.9210	0.0780
0.9440	0.2857	0.9330	0.5673
0.9440	0.1554	0.9360	0.0681
0.9440	0.2950	0.9790	0.8188

0.9440	0.6659	1.0530	0.8072
0.9440	0.6133	1.0770	0.3186
0.9430	0.3247	0.7630	0.0021
0.9430	0.0107	0.9050	0.0064
0.9430	0.1786	0.9430	0.1799
0.9430	0.3136	0.9510	0.3116
0.9430	0.3375	1.0060	0.9560
0.9430		1.0650	
0.9430	0.3421	1.0830	0.2520
0.9430	0.6179	1.7070	0.1805
0.9420	0.3107	0.8670	0.0916
0.9420	0.4514	0.9300	0.2867
0.9420	0.1973	0.9660	0.3824
0.9420	0.1396	1.0080	0.8769
0.9420	0.1945	1.0110	0.7553
0.9420	0.7098	1.1350	0.5890
0.9420	0.3118	1.1640	0.0002
0.9410	0.3072	0.8760	0.0485
0.9410	0.3524	0.9620	0.4943
0.9410	0.4124	0.9460	0.4152
0.9410	0.6058	0.9590	0.6146
0.9410	0.3801	0.9870	0.8191
0.9410	0.2202	1.0460	0.4298
0.9410	0.5650	1.4190	0.0891
0.9400		0.9240	
0.9400	0.4931	0.8910	0.3507
0.9400	0.2990	0.8980	0.2863
0.9400	0.2573	0.9100	0.1528
0.9400	0.1121	0.9500	0.3686
0.9400	0.4344	0.9630	0.7504
0.9400	0.2449	0.9670	0.7144
0.9400	0.2100	0.9900	0.8823
0.9400	0.4272	0.9840	0.8588

0.9400	0.4614	1.0200	0.6507
0.9400	0.4319	1.0170	0.7948
0.9400	0.5213	1.1040	0.3999
0.9400	0.7781	1.2520	0.3953
0.9390	0.5733	0.7490	0.1740
0.9390	0.6043	0.8160	0.1284
0.9390		0.8820	
0.9390	0.5604	0.9220	0.4729
0.9390	0.1294	0.9170	0.0594
0.9390	0.3454	0.9340	0.2252
0.9390	0.2661	0.9380	0.1736
0.9390	0.1019	0.9680	0.4272
0.9390	0.3087	0.9690	0.5244
0.9390	0.0413	0.9820	0.6950
0.9390	0.5374	0.9680	0.7534
0.9390		1.1960	
0.9380	0.2134	0.8880	0.0511
0.9380	0.4483	0.9150	0.5033
0.9380	0.2864	0.9570	0.6180
0.9380	0.7267	1.1360	0.6855
0.9380	0.1792	1.0130	0.6508
0.9380	0.2023	1.0660	0.3041
0.9380	0.3146	1.0590	0.0700
0.9380	0.2336	1.0870	0.0921
0.9380		1.1370	
0.9380	0.3252	1.2100	0.0374
0.9380		1.1870	
0.9380	0.7187	1.3730	0.1527
0.9370		0.9270	
0.9370	0.5826	0.9210	0.1668
0.9370	0.2768	0.9210	0.0513
0.9370	0.1988	0.9730	0.6055
0.9370	0.1794	0.9480	0.4774

0.9370	0.2127	0.9560	0.2669
0.9370	0.6711	0.9740	0.8331
0.9370	0.1313	1.0250	0.5341
0.9370	0.0585	1.0370	0.4340
0.9360	0.2077	0.8810	0.0029
0.9360	0.5495	0.8960	0.1185
0.9360	0.4754	0.9280	0.2686
0.9360		0.9510	
0.9360	0.3642	0.9510	0.2904
0.9360	0.0058	0.9580	0.1095
0.9360	0.5720	0.9590	0.6411
0.9360	0.8426	0.9450	0.6840
0.9360	0.2151	0.9960	0.9483
0.9360		0.9930	
0.9360	0.1960	0.9910	0.8688
0.9360	0.1402	1.0730	0.3772
0.9360	0.3965	1.1070	0.2725
0.9350		0.8410	
0.9350	0.7701	0.8400	0.0991
0.9350	0.7467	0.8510	0.1858
0.9350	0.0575	0.9440	0.2116
0.9350	0.4082	0.9730	0.9156
0.9350	0.2610	0.9980	0.9677
0.9350	0.2530	1.0370	0.5899
0.9350	0.5035	1.0460	0.6080
0.9350	0.7570	1.1790	0.4051
0.9350	0.7765	1.4040	0.0983
0.9340	0.4966	0.8730	0.2036
0.9340	0.2323	0.9340	0.1127
0.9340	0.0403	0.9350	0.0128
0.9340	0.4011	0.9470	0.4443
0.9340	0.4189	0.9610	0.5647
0.9340	0.4418	0.9880	0.8487

0.9340		1.0210	
0.9340	0.1276	1.0600	0.2253
0.9340	0.9668	1.3140	0.2579
0.9340	0.6071	1.4390	0.2865
0.9330	0.0518	0.8890	0.0486
0.9330	0.1961	0.9620	0.5402
0.9330	0.0675	0.9460	0.2321
0.9330	0.5540	0.9620	0.4960
0.9330	0.1694	0.9770	0.5070
0.9330	0.0310	0.9720	0.6884
0.9330	0.1849	1.0350	0.2670
0.9320	0.6514	0.7770	0.2299
0.9320	0.2286	0.8700	0.0090
0.9320	0.1100	0.9280	0.0102
0.9320	0.1619	0.9320	0.4399
0.9320	0.1998	0.9440	0.3533
0.9320		0.9590	
0.9320		0.9770	
0.9320	0.5915	0.9850	0.9818
0.9320	0.3413	0.9770	0.7083
0.9320	0.2590	1.1140	0.1750
0.9320	0.6002	1.1410	0.4207
0.9320	0.6813	1.4510	0.0111
0.9320	0.7048	1.7960	0.2228
0.9310	0.2441	0.8890	0.0793
0.9310		0.8960	
0.9310	0.2091	0.8730	0.0337
0.9310	0.0475	0.9500	0.0891
0.9310	0.1929	0.9340	0.4299
0.9310	0.1410	0.9300	0.0564
0.9310	0.3127	0.9630	0.6436
0.9310	0.1098	0.9860	0.8710
0.9310	0.0795	1.0190	0.7553

0.9310	0.2397	1.0870	0.1345
0.9310	0.4765	1.4510	0.0625
0.9300	0.3052	0.8180	0.0549
0.9300	0.6389	0.8710	0.0561
0.9300	0.0181	0.8950	0.0041
0.9300	0.1672	0.8960	0.0487
0.9300	0.0851	0.9290	0.0165
0.9300	0.3780	0.9330	0.4139
0.9300	0.1262	0.9350	0.2738
0.9300	0.0174	0.9620	0.1660
0.9300		1.0470	
0.9300	0.3875	1.0960	0.4519
0.9300	0.2080	1.1750	0.1087
0.9300	0.7514	1.2200	0.4572
0.9290		0.8550	
0.9290	0.0975	0.8910	0.0211
0.9290	0.0748	0.8930	0.0164
0.9290	0.1743	0.9110	0.0258
0.9290		0.9380	
0.9290	0.0700	0.9620	0.2974
0.9290	0.7610	0.9750	0.8672
0.9290	0.3122	1.0020	0.9970
0.9290	0.3207	1.0030	0.9524
0.9290	0.3759	1.1290	0.2306
0.9280		0.8100	
0.9280		0.8170	
0.9280	0.3824	0.8810	0.0768
0.9280	0.0277	0.9090	0.0380
0.9280	0.1497	0.9280	0.2500
0.9280	0.5136	1.2570	0.2406
0.9280	0.4954	1.3420	0.0713
0.9270	0.3441	0.8340	0.2762
0.9270	0.4382	0.9020	0.1252

0.9270	0.2435	0.9430	0.2449
0.9270	0.2489	0.9630	0.4962
0.9270	0.4701	1.0070	0.9835
0.9270	0.5400	1.1130	0.5059
0.9270	0.5477	1.2570	0.1027
0.9260		0.9190	
0.9260	0.8386	0.9500	0.8009
0.9260	0.1208	0.9440	0.2235
0.9260	0.3717	0.9410	0.4073
0.9260	0.2245	0.9450	0.5504
0.9260	0.5170	0.9560	0.5861
0.9250	0.1573	0.8860	0.0975
0.9250	0.7033	0.8820	0.5713
0.9250		0.9120	
0.9250	0.3066	0.9280	0.2519
0.9250	0.0724	0.9670	0.3754
0.9250	0.3681	1.0100	0.9015
0.9250	0.4911	1.0150	0.9165
0.9250	0.2791	1.0910	0.2696
0.9250		1.0940	
0.9250	0.7013	1.5840	0.2352
0.9240		0.9020	
0.9240		0.9110	
0.9240	0.4589	0.9090	0.2824
0.9240	0.0901	0.9580	0.2952
0.9240	0.0606	0.9290	0.0094
0.9240	0.1584	0.9530	0.4770
0.9240		0.9980	
0.9240	0.3333	0.9870	0.8514
0.9240	0.0849	1.0270	0.2022
0.9240	0.2851	1.0720	0.3666
0.9240	0.5171	1.1910	0.1925
0.9240	0.3048	1.0980	0.6216

0.9230	0.2574	0.8440	0.0627
0.9230		0.8860	
0.9230	0.0382	0.9170	0.2065
0.9230	0.0809	0.9270	0.3619
0.9230	0.0069	1.0130	0.6558
0.9230	0.6510	1.0260	0.8480
0.9230	0.0973	1.0920	0.3103
0.9230		1.1270	
0.9230	0.5286	1.1860	0.2530
0.9220	0.2970	0.8460	0.0977
0.9220	0.0213	0.8890	0.0215
0.9220	0.2629	0.9150	0.1821
0.9220	0.2581	0.9980	0.9951
0.9220	0.3146	1.0770	0.3198
0.9220	0.5331	1.1840	0.5471
0.9210		0.8830	
0.9210	0.5192	0.8650	0.3049
0.9210		0.9000	
0.9210	0.1385	0.9030	0.0639
0.9210	0.4555	0.9180	0.0738
0.9210	0.2081	0.9700	0.7443
0.9210	0.1666	1.0480	0.2527
0.9210	0.1822	1.0640	0.4866
0.9210	0.5285	1.0630	0.5381
0.9200	0.0981	0.8010	0.0207
0.9200	0.3838	0.8470	0.1030
0.9200	0.2310	0.9390	0.3223
0.9200	0.0506	0.9460	0.3274
0.9200	0.2323	0.9680	0.5513
0.9200	0.0380	1.0040	0.9266
0.9200	0.0391	1.0510	0.3399
0.9200	0.0469	1.0410	0.5375
0.9200	0.3130	1.0620	0.5647

0.9190	0.0297	0.9470	0.3517
0.9190	0.3923	0.9320	0.4757
0.9190	0.2467	0.9330	0.3812
0.9190		0.9590	
0.9190	0.0624	0.9460	0.1621
0.9190	0.3308	0.9760	0.7151
0.9190	0.0672	1.0090	0.8221
0.9190	0.2383	1.0410	0.7111
0.9190	0.4924	1.2080	0.2037
0.9180	0.3142	0.9150	0.1882
0.9180	0.2871	0.9190	0.1975
0.9180	0.5784	0.9170	0.5629
0.9180	0.1084	0.9330	0.1438
0.9180	0.3939	0.8990	0.1746
0.9180	0.1847	0.9600	0.5436
0.9180	0.3175	1.0170	0.8476
0.9180		1.4090	
0.9170	0.1803	0.8580	0.1067
0.9170	0.3943	0.8790	0.1533
0.9170	0.0458	0.8990	0.0221
0.9170	0.0243	0.9220	0.0079
0.9170	0.0572	0.9520	0.1796
0.9170	0.4642	1.0060	0.9105
0.9170	0.3519	1.0240	0.7728
0.9170	0.6148	1.2210	0.4275
0.9160		0.8410	
0.9160		0.8840	
0.9160		0.9240	
0.9160	0.1461	1.0250	0.6346
0.9160	0.3202	1.0150	0.5453
0.9160	0.2558	1.1490	0.3227
0.9160	0.6714	1.2410	0.5118
0.9150		0.8940	

0.9150	0.3457	0.9430	0.4694
0.9150	0.0939	0.9890	0.8157
0.9150	0.2559	0.9920	0.9554
0.9150	0.0014	1.0420	0.2673
0.9150		1.0950	
0.9150	0.4479	1.1010	0.3946
0.9140		0.8020	
0.9140	0.4592	0.7730	0.0888
0.9140		0.9690	
0.9140	0.1286	0.9500	0.1099
0.9140		1.1410	
0.9130		0.9290	
0.9130	0.0800	0.9290	0.1438
0.9130	0.1251	0.9530	0.1796
0.9130	0.1436	1.0170	0.7447
0.9130	0.4589	1.0430	0.8877
0.9130	0.0783	0.9970	0.9553
0.9130	0.3677	1.0100	0.9383
0.9130	0.4966	1.0060	0.9810
0.9130	0.3129	1.1150	0.1072
0.9130	0.5038	1.3260	0.1092
0.9130	0.5712	1.3300	0.0625
0.9120	0.3058	0.9010	0.0750
0.9120	0.0003	0.9160	0.0525
0.9120	0.0702	0.9180	0.0429
0.9120	0.0438	0.9430	0.4807
0.9120	0.0426	0.9540	0.2321
0.9120	0.0488	0.9440	0.4385
0.9120	0.2107	1.0340	0.6074
0.9120	0.3635	1.0420	0.3326
0.9120	0.7112	1.3070	0.5102
0.9110	0.3844	0.7970	0.3596
0.9110	0.1269	0.8590	0.0575

0.9110	0.4631	0.8650	0.1902
0.9110	0.2606	0.8890	0.1908
0.9110	0.4739	0.9250	0.7939
0.9110	0.2408	0.9610	0.7062
0.9110	0.2337	0.9510	0.4792
0.9110	0.0436	0.9680	0.2331
0.9110	0.1115	0.9730	0.5518
0.9110	0.2791	0.9790	0.7667
0.9110	0.0458	0.9970	0.9527
0.9100	0.2272	0.8590	0.0693
0.9100		0.9350	
0.9100	0.0087	0.9180	0.0021
0.9100	0.0510	1.0430	0.2022
0.9100	0.1871	1.0560	0.3511
0.9100	0.0055	1.0130	0.7934
0.9090		0.6930	
0.9090		0.7970	
0.9090		0.8660	
0.9090	0.7344	0.8300	0.6450
0.9090		0.8720	
0.9090	0.0671	0.8870	0.0431
0.9090		0.9480	
0.9090	0.0233	0.9470	0.1289
0.9090	0.1027	0.9750	0.6482
0.9090		0.9910	
0.9090	0.2999	0.9050	0.0779
0.9090	0.5661	1.0560	0.8208
0.9090	0.3225	1.0700	0.4031
0.9090		1.1200	
0.9080	0.6704	0.7190	0.3197
0.9080	0.3145	0.8610	0.1269
0.9080	0.1915	0.8650	0.0477
0.9080	0.0458	0.9100	0.0539

0.9080	0.3798	0.9550	0.8052
0.9080	0.2455	0.9150	0.3607
0.9080	0.3509	1.1130	0.2360
0.9080	0.7055	1.3620	0.6083
0.9070	0.2194	0.8200	0.1577
0.9070	0.2222	0.8410	0.0177
0.9070		0.9410	
0.9070	0.0710	0.9390	0.1830
0.9070	0.0425	0.9490	0.3100
0.9070	0.0223	0.9260	0.0877
0.9070	0.0317	0.9360	0.1743
0.9070		1.0100	
0.9070	0.3338	1.0210	0.8436
0.9070	0.1040	1.0570	0.2955
0.9070	0.3460	1.1300	0.0963
0.9070		1.3490	
0.9060	0.0952	0.8760	0.2630
0.9060	0.1238	0.8880	0.0403
0.9060	0.0136	0.9570	0.2244
0.9060	0.0545	0.9960	0.9330
0.9060	0.3143	1.0890	0.2994
0.9060	0.0332	1.0980	0.1782
0.9060		1.1250	
0.9060		1.1400	
0.9060	0.4941	1.1790	0.0739
0.9060	0.6077	1.3950	0.3275
0.9050	0.1352	0.9090	0.0696
0.9050	0.0964	0.9450	0.4968
0.9050	0.0572	0.9650	0.4019
0.9050	0.1516	0.9890	0.8254
0.9050	0.6044	1.0650	0.7510
0.9040	0.0535	0.8610	0.0095
0.9040	0.5931	0.9710	0.5842

0.9040	0.1745	0.9860	0.5409
0.9040	0.1425	0.9750	0.4661
0.9040	0.1052	0.9880	0.7669
0.9040	0.0385	0.9990	0.9844
0.9040		1.0820	
0.9040		1.1390	
0.9030		0.8440	
0.9030	0.4312	0.8740	0.4394
0.9030	0.0524	0.8810	0.0349
0.9030	0.1082	0.8630	0.3632
0.9030	0.1236	0.8990	0.3247
0.9030	0.0013	0.9180	0.0081
0.9030	0.0769	0.9820	0.8472
0.9020	0.1375	0.7610	0.2689
0.9020		0.8780	
0.9020	0.1113	0.9350	0.1784
0.9020	0.0377	0.9210	0.0414
0.9020	0.1460	0.9550	0.3298
0.9020	0.0588	0.9160	0.0627
0.9020	0.0911	0.9580	0.6440
0.9020	0.0769	1.0610	0.3710
0.9020	0.5516	1.3610	0.2529
0.9020		2.2470	
0.9010	0.2501	0.8590	0.1193
0.9010	0.0917	0.9060	0.0193
0.9010	0.0696	0.9300	0.2241
0.9010	0.0572	0.9450	0.3911
0.9010	0.0585	1.0310	0.6543
0.9010	0.2848	1.1700	0.0473
0.9000	0.4676	0.8010	0.3831
0.9000		0.8700	
0.9000	0.0340	0.9280	0.3608
0.9000	0.1728	0.9630	0.6727

0.9000	0.0686	0.9870	0.8087
0.9000	0.0426	1.0150	0.7650
0.9000	0.6027	1.1910	0.3705
0.9000	0.6757	1.2480	0.2917
0.8990	0.0921	0.8320	0.1376
0.8990		0.8720	
0.8990	0.0635	0.8680	0.0193
0.8990	0.0054	0.9120	0.0097
0.8990	0.0503	0.9390	0.1067
0.8990	0.1216	0.9900	0.8536
0.8990		0.9570	
0.8990	0.0375	0.9660	0.4562
0.8990	0.1316	1.1050	0.2983
0.8990	0.4933	1.1280	0.5999
0.8990	0.1446	1.1570	0.1101
0.8990		1.3490	
0.8980	0.1158	0.9070	0.2285
0.8980	0.1760	0.9540	0.4990
0.8980	0.3558	1.0900	0.4588
0.8980	0.4256	1.1630	0.1685
0.8970	0.0668	0.8680	0.0839
0.8970	0.0220	0.8900	0.0765
0.8970	0.2129	0.9630	0.7500
0.8970	0.0230	1.0190	0.7209
0.8970	0.1337	1.0440	0.6636
0.8960	0.0541	0.8970	0.0194
0.8960		0.9350	
0.8960	0.0097	0.9570	0.0000
0.8960		1.0360	
0.8960	0.0986	1.0320	0.6290
0.8960		1.0900	
0.8960	0.3475	1.1630	0.2402
0.8950	0.2682	0.8170	0.4525

0.8950	0.0561	0.8970	0.1332
0.8950	0.3827	0.9200	0.6853
0.8950	0.2603	0.9250	0.3884
0.8950	0.0075	0.9210	0.1031
0.8950	0.5154	0.9660	0.6805
0.8950	0.1421	0.9500	0.3561
0.8950	0.1890	0.9840	0.8914
0.8950		1.0050	
0.8950		1.0340	
0.8950	0.3690	1.0110	0.8028
0.8950	0.1780	1.0310	0.6672
0.8950		1.1400	
0.8950		1.1890	
0.8950	0.4487	1.2160	0.2690
0.8940	0.3182	0.7810	0.0367
0.8940	0.0962	0.8680	0.0667
0.8940	0.0173	0.8880	0.0568
0.8940		0.9170	
0.8940	0.0542	0.8920	0.0841
0.8940	0.0957	0.9490	0.5079
0.8940	0.0009	0.9730	0.4014
0.8940	0.0307	1.0060	0.8214
0.8940	0.0146	1.0560	0.3536
0.8930	0.2145	0.8550	0.1058
0.8930	0.0046	0.9300	0.1900
0.8930		0.9210	
0.8930	0.1690	0.9190	0.2634
0.8930	0.0358	0.9350	0.2185
0.8930		0.9760	
0.8930	0.2922	0.9850	0.7868
0.8930	0.0545	1.0270	0.7026
0.8930	0.1125	1.0440	0.7731
0.8930	0.4461	1.1500	0.3918

0.8930		1.3060	
0.8930	0.8533	1.2790	0.2927
0.8930	0.2702	1.4250	0.1270
0.8930	0.7081	1.5380	0.3896
0.8920		0.6720	
0.8920	0.6439	0.7630	0.3884
0.8920	0.0176	0.8730	0.0033
0.8920		0.9120	
0.8920	0.1576	0.9070	0.0508
0.8920	0.0351	0.9250	0.3067
0.8920	0.1171	0.9080	0.0544
0.8920	0.1884	0.9180	0.2813
0.8920		0.9590	
0.8920	0.0144	0.9510	0.3501
0.8920	0.2016	0.9450	0.4635
0.8920		1.0020	
0.8920		1.0350	
0.8920	0.4459	1.1540	0.4075
0.8910		0.7990	
0.8910	0.4296	0.8510	0.2224
0.8910	0.0887	0.8790	0.0660
0.8910	0.0372	0.9030	0.0505
0.8910	0.0219	0.9270	0.0683
0.8910	0.1114	0.9100	0.2345
0.8910	0.2756	0.9200	0.8971
0.8910	0.2355	0.9170	0.5714
0.8910	0.0760	0.9450	0.3478
0.8910	0.1128	0.9960	0.9908
0.8910	0.2089	0.9810	0.8229
0.8910	0.3112	1.1880	0.2328
0.8910		1.4120	
0.8900	0.1850	0.8480	0.1027
0.8900	0.0119	0.9330	0.1014

0.8900	0.0492	1.0150	0.7886
0.8900	0.3163	1.1330	0.2827
0.8890	0.0206	0.8700	0.0165
0.8890	0.0387	0.8980	0.0436
0.8890	0.2576	0.8960	0.3695
0.8890	0.0079	0.8950	0.0017
0.8890	0.0040	0.9400	0.1495
0.8890	0.2818	0.9370	0.5135
0.8890		0.9750	
0.8880	0.1565	0.8050	0.0367
0.8880	0.2292	0.8230	0.0590
0.8880	0.0065	0.8840	0.0139
0.8880	0.1458	0.9140	0.2775
0.8880	0.0662	0.9260	0.1752
0.8880		1.0410	
0.8880	0.0683	1.0370	0.5898
0.8880		1.0780	
0.8870		0.7620	
0.8870	0.2766	0.7650	0.2896
0.8870	0.1650	0.8000	0.0611
0.8870	0.1444	0.9010	0.0046
0.8870	0.0672	0.9780	0.6308
0.8870	0.0760	1.0210	0.7361
0.8870	0.3280	1.0170	0.9005
0.8870	0.0445	1.0450	0.6326
0.8860	0.2360	0.8620	0.5870
0.8860		0.8960	
0.8860		0.8830	
0.8860	0.1791	0.8870	0.2486
0.8860	0.0750	0.9490	0.3769
0.8860	0.0474	0.9580	0.6227
0.8860	0.0260	0.9480	0.5729
0.8850	0.0036	0.8750	0.0236

0.8850	0.0110	0.9010	0.0492
0.8850	0.1617	0.9290	0.4131
0.8850		0.9850	
0.8840	0.3924	0.6260	0.0351
0.8840	0.3799	0.6500	0.0286
0.8840	0.3670	0.8150	0.2879
0.8840	0.0043	0.8320	0.0289
0.8840	0.0833	0.8290	0.0455
0.8840	0.2191	0.8680	0.0789
0.8840	0.0620	0.9060	0.0415
0.8840	0.0292	0.8930	0.0071
0.8840		0.9530	
0.8840	0.0401	0.9490	0.4526
0.8840	0.1520	0.9530	0.6920
0.8840	0.0007	0.9650	0.4788
0.8830	0.1618	0.7030	0.0271
0.8830	0.0223	0.8670	0.0019
0.8830	0.0094	0.8870	0.1381
0.8830	0.0002	0.8740	0.0001
0.8830		0.9270	
0.8830	0.0161	0.9220	0.0728
0.8830	0.0279	0.9950	0.9482
0.8830	0.1229	1.0230	0.8306
0.8830	0.1202	1.0060	0.9365
0.8830		1.0620	
0.8830	0.2467	1.1260	0.3018
0.8830	0.6266	1.3120	0.3002
0.8820	0.0607	0.8850	0.1019
0.8820		0.8690	
0.8820	0.0640	0.9200	0.0886
0.8820		1.3340	
0.8810	0.1539	0.7950	0.0402
0.8810	0.1613	0.8410	0.0807

0.8810	0.1244	0.8890	0.0516
0.8810	0.0083	0.8690	0.0072
0.8810	0.0209	0.9150	0.1717
0.8810	0.0012	0.9130	0.0077
0.8810	0.0687	0.9160	0.3077
0.8810	0.0009	0.9210	0.3938
0.8810	0.3753	1.0140	0.8806
0.8810	0.1397	1.0410	0.6098
0.8800	0.7783	0.8010	0.4650
0.8800	0.0980	0.8490	0.0153
0.8800	0.0443	0.8980	0.1345
0.8800	0.0018	0.8990	0.0031
0.8800	0.0048	0.9340	0.0732
0.8800	0.1008	0.9360	0.3592
0.8800	0.2227	0.9270	0.4313
0.8800	0.3996	1.1020	0.4031
0.8790	0.0876	0.8270	0.0157
0.8790	0.2979	0.8100	0.1119
0.8790	0.0868	0.8350	0.0270
0.8790	0.0197	0.8830	0.0326
0.8790	0.0232	0.9360	0.4022
0.8790	0.0198	0.9020	0.1119
0.8790	0.0089	0.9720	0.4027
0.8790	0.2608	0.9640	0.6880
0.8790		1.4770	
0.8780	0.1662	0.8430	0.0387
0.8780		0.8650	
0.8780		0.8910	
0.8780	0.0130	0.9170	0.1001
0.8780	0.0211	0.9480	0.4460
0.8780	0.0843	0.9390	0.3773
0.8780	0.1757	0.9480	0.2663
0.8780	0.0107	0.9490	0.0568

0.8780		0.9920	
0.8780	0.0564	0.9940	0.9227
0.8780	0.2192	0.9900	0.8270
0.8780	0.1134	1.0130	0.8581
0.8780	0.0332	1.1040	0.1528
0.8780	0.6267	1.1090	0.4988
0.8780	0.1946	1.2980	0.1193
0.8770	0.0264	0.8200	0.0014
0.8770	0.1252	0.8310	0.0592
0.8770	0.0301	0.8410	0.0074
0.8770		0.9410	
0.8770	0.0498	0.9690	0.3677
0.8770	0.0698	0.9470	0.3624
0.8770	0.3700	0.9870	0.9277
0.8770	0.0393	1.0360	0.6444
0.8770	0.3315	1.0640	0.7698
0.8760	0.3115	0.8230	0.1739
0.8760		0.8830	
0.8760	0.0592	0.8740	0.0494
0.8760	0.0286	0.8880	0.1508
0.8760	0.0189	0.9250	0.1632
0.8760	0.3958	0.9450	0.4823
0.8760	0.0380	1.0290	0.6524
0.8760	0.0650	1.0480	0.5131
0.8750	0.3435	0.7110	0.1449
0.8750	0.1218	0.7770	0.0126
0.8750	0.0493	0.8520	0.0422
0.8750	0.1786	0.8640	0.2146
0.8750	0.0663	0.8850	0.0217
0.8750	0.0194	0.8920	0.0840
0.8750	0.2975	0.9980	0.9911
0.8750	0.5595	1.0060	0.9970
0.8750		1.0780	

0.8740		0.8590	
0.8740	0.1265	0.8400	0.0920
0.8740	0.0446	0.8870	0.0633
0.8740	0.0777	0.8850	0.1480
0.8740	0.2621	0.8720	0.1414
0.8740	0.0539	0.9030	0.0578
0.8740	0.0428	0.9280	0.2528
0.8740	0.2340	0.9640	0.8495
0.8730	0.0467	0.8470	0.0089
0.8730		0.8930	
0.8730	0.0322	0.8770	0.1160
0.8730	0.1103	0.8700	0.0036
0.8730	0.0761	0.9210	0.1871
0.8730	0.0373	0.9300	0.2479
0.8730	0.0265	0.9610	0.4794
0.8730	0.0309	0.9610	0.4906
0.8720	0.0315	0.7930	0.0230
0.8720	0.3453	0.8200	0.0014
0.8720		0.8740	
0.8720	0.1432	0.8620	0.0390
0.8720		0.8980	
0.8720	0.0342	0.8800	0.0446
0.8720	0.0026	0.9000	0.0453
0.8720	0.0530	0.9150	0.1341
0.8720	0.0322	0.8970	0.0745
0.8720		1.0550	
0.8720	0.4092	1.1280	0.5154
0.8720	0.1249	1.1090	0.2894
0.8710		0.8890	
0.8710	0.0150	0.8710	0.0222
0.8710	0.0965	0.9050	0.1435
0.8710	0.1585	0.9130	0.3478
0.8710	0.0097	0.9290	0.2875

0.8710	0.3667	0.9960	0.9755
0.8710		1.0430	
0.8710	0.4284	1.5440	0.0993
0.8700	0.0491	0.8550	0.0539
0.8700		0.8760	
0.8700	0.0776	0.8920	0.2231
0.8700	0.0001	0.9190	0.0633
0.8700	0.0099	0.9690	0.5481
0.8700	0.0499	0.9430	0.0949
0.8700	0.6217	0.9590	0.7997
0.8700		1.0230	
0.8700	0.2295	1.0850	0.5493
0.8690		0.7860	
0.8690	0.0073	0.8350	0.0004
0.8690	0.0192	0.8290	0.0130
0.8690		0.8840	
0.8690	0.1387	0.8720	0.1358
0.8690	0.0023	0.9040	0.0421
0.8690	0.0407	0.9970	0.9523
0.8690	0.0471	1.0200	0.7054
0.8690	0.0519	1.0210	0.8062
0.8690	0.1014	1.1760	0.0377
0.8680	0.0481	0.8750	0.0456
0.8680	0.0112	0.8680	0.0046
0.8680	0.0308	0.8750	0.1442
0.8680	0.5181	0.8610	0.4814
0.8680	0.0244	0.8850	0.0183
0.8680	0.0096	0.9360	0.2531
0.8680	0.2271	0.9480	0.7191
0.8680	0.0317	0.9770	0.8216
0.8680	0.3874	1.0120	0.9507
0.8670	0.1044	0.8500	0.2050
0.8670	0.0279	0.8690	0.1526

0.8670	0.0221	0.8980	0.0406
0.8670	0.1358	0.9140	0.1655
0.8670	0.0215	0.8790	0.3322
0.8670		0.9370	
0.8670		1.0290	
0.8670	0.0536	0.9950	0.9314
0.8670	0.0119	1.0750	0.1891
0.8670	0.0170	1.1000	0.1413
0.8660	0.3941	0.7040	0.1076
0.8660	0.0087	0.8640	0.0048
0.8660	0.2519	0.8580	0.2071
0.8660	0.0193	0.8730	0.0258
0.8660	0.1016	0.8710	0.0931
0.8660	0.0131	0.8880	0.0606
0.8660	0.1235	0.9200	0.3390
0.8660	0.0004	0.9210	0.0415
0.8660		0.9350	
0.8660	0.0434	1.0320	0.7505
0.8660	0.3162	1.1730	0.0353
0.8650	0.0168	0.8090	0.0049
0.8650		0.8480	
0.8650	0.0165	0.8340	0.0006
0.8650	0.0375	0.8510	0.0208
0.8650	0.0215	0.8510	0.0205
0.8650	0.3779	0.8530	0.1445
0.8650	0.2396	0.8540	0.3106
0.8650	0.0160	0.9730	0.6664
0.8650		1.1460	
0.8650		1.2850	
0.8640		0.8260	
0.8640	0.1550	0.8510	0.1099
0.8640	0.0653	0.8700	0.2089
0.8640	0.0029	0.9040	0.0072

0.8640	0.0038	0.9170	0.3150
0.8640	0.0019	0.9100	0.0155
0.8640	0.1044	0.9220	0.3557
0.8640	0.1075	0.9220	0.3977
0.8640	0.2849	0.9260	0.1110
0.8640	0.0635	0.9320	0.3414
0.8640	0.0504	1.0330	0.6699
0.8640		1.0270	
0.8640	0.2631	1.0320	0.8668
0.8640	0.3527	1.0430	0.5978
0.8640	0.1061	1.0980	0.2415
0.8640	0.1708	1.1540	0.2350
0.8640	0.4893	1.5040	0.0710
0.8630	0.0093	0.8540	0.0067
0.8630	0.1213	0.8060	0.1132
0.8630		0.8710	
0.8630	0.5977	0.8210	0.3351
0.8630	0.1327	0.8460	0.0903
0.8630	0.0485	0.9270	0.1528
0.8630	0.2100	0.9250	0.1105
0.8630	0.0592	1.0230	0.8043
0.8630	0.0775	1.0300	0.7455
0.8630	0.1687	1.2720	0.0890
0.8620		0.9050	
0.8620	0.0249	0.9010	0.0391
0.8620	0.0021	0.9190	0.1569
0.8620	0.0096	0.9460	0.0550
0.8620		1.1650	
0.8610		0.8330	
0.8610	0.0439	0.8730	0.1098
0.8610		0.8950	
0.8610	0.0624	0.9390	0.4393
0.8610	0.0577	0.9290	0.4038

0.8610	0.0117	0.9590	0.5734
0.8610	0.2516	0.9960	0.9540
0.8610	0.1242	1.0650	0.5552
0.8600		0.7290	
0.8600		0.7720	
0.8600	0.4006	0.7840	0.1592
0.8600	0.0122	0.9130	0.1218
0.8600		0.9460	
0.8600	0.2533	1.0170	0.8825
0.8600	0.0427	1.1440	0.0992
0.8590	0.0167	0.8290	0.0180
0.8590	0.0539	0.8320	0.0654
0.8590	0.2878	0.8260	0.1099
0.8590	0.0753	0.8770	0.0915
0.8590	0.0328	0.9280	0.2486
0.8590		0.9440	
0.8590	0.1632	0.9540	0.7605
0.8590	0.0008	0.9500	0.0651
0.8590	0.0169	0.9740	0.6227
0.8590	0.0426	1.0780	0.2761
0.8590	0.2117	1.1370	0.4428
0.8580		0.7990	
0.8580	0.0374	0.8640	0.0239
0.8580	0.0584	0.8730	0.1276
0.8580		0.9140	
0.8580	0.0003	0.9420	0.1976
0.8580	0.0252	0.9500	0.5023
0.8580		0.9760	
0.8580	0.0447	0.9990	0.9830
0.8580	0.0103	0.9780	0.6520
0.8580	0.0543	0.9700	0.4242
0.8580	0.1781	1.4780	0.0260
0.8570		0.8570	

0.8570	0.0225	0.8460	0.0159
0.8570	0.0092	0.8440	0.0065
0.8570	0.0263	0.8620	0.0397
0.8570	0.0284	0.8660	0.0238
0.8570	0.0890	0.8800	0.1812
0.8570	0.0849	0.9070	0.1121
0.8570	0.0896	0.9520	0.5297
0.8570	0.0278	0.9330	0.4000
0.8570	0.0081	0.9380	0.3830
0.8570	0.0226	0.9520	0.4906
0.8570	0.1671	0.9660	0.9506
0.8570	0.4617	0.9860	0.8174
0.8570	0.4642	1.0400	0.9205
0.8560	0.3319	0.7430	0.3069
0.8560	0.1061	0.8210	0.0805
0.8560	0.0150	0.8480	0.0191
0.8560	0.2778	0.8340	0.1421
0.8560		0.8830	
0.8560	0.0958	0.9220	0.2475
0.8560	0.0163	0.9190	0.3032
0.8560	0.0173	1.2270	0.2040
0.8550		0.7780	
0.8550	0.1068	0.7580	0.0499
0.8550	0.0120	0.8130	0.0252
0.8550		0.8510	
0.8550	0.0763	0.9250	0.3200
0.8550	0.2976	0.9260	0.3469
0.8550	0.0151	0.9420	0.2343
0.8550	0.3288	1.0630	0.6369
0.8550	0.0625	1.0450	0.5548
0.8550	0.0379	1.0730	0.2935
0.8550		1.1280	
0.8540		0.7580	

0.8540	0.0965	0.8320	0.0570
0.8540	0.0261	0.8760	0.0439
0.8540	0.0001	0.9240	0.0456
0.8540	0.0815	0.9030	0.3457
0.8540	0.0485	0.9590	0.5870
0.8540	0.0621	0.9830	0.8150
0.8540	0.2673	1.1490	0.3023
0.8540	0.0686	1.0970	0.4203
0.8530		0.8460	
0.8530	0.2080	0.7950	0.0689
0.8530	0.0541	0.8360	0.0305
0.8530	0.0071	0.8760	0.0048
0.8530	0.0273	0.8800	0.0611
0.8530	0.0259	0.8840	0.0615
0.8530	0.0621	0.9290	0.1170
0.8530	0.0418	0.9200	0.4205
0.8530	0.0908	0.9790	0.4953
0.8530	0.1434	1.3160	0.1174
0.8520		0.7150	
0.8520	0.0016	0.7620	0.0037
0.8520		0.8230	
0.8520	0.1038	0.8370	0.0297
0.8520	0.0150	0.8640	0.0266
0.8520	0.0009	0.8910	0.0391
0.8520	0.0859	0.9250	0.3686
0.8510	0.1138	0.7400	0.0087
0.8510	0.3381	0.7440	0.0299
0.8510	0.0752	0.9040	0.1266
0.8510	0.0058	0.9150	0.0440
0.8510	0.0401	0.9170	0.2280
0.8510	0.0013	0.9320	0.0775
0.8510	0.0163	0.9540	0.5554
0.8510	0.3901	0.9460	0.6863

0.8510	0.0066	1.0420	0.6501
0.8500	0.0037	0.8440	0.0166
0.8500	0.0035	0.9080	0.1069
0.8500	0.0571	0.9120	0.4580
0.8500	0.8396	0.9770	0.9171
0.8500	0.0443	0.9910	0.8525
0.8500	0.1377	0.9840	0.8654
0.8500		1.0880	
0.8500		1.1030	
0.8490	0.0059	0.8380	0.0019
0.8490	0.0035	0.8450	0.0468
0.8490	0.1773	0.8530	0.0627
0.8490	0.2107	0.8800	0.1378
0.8490	0.2301	0.8920	0.2218
0.8490	0.2837	0.9180	0.2515
0.8490	0.0415	0.9090	0.1769
0.8490	0.0055	0.9150	0.0718
0.8480	0.3563	0.8250	0.1828
0.8480	0.0076	0.8730	0.0111
0.8480	0.0211	0.8760	0.0538
0.8480	0.0528	0.8990	0.2467
0.8480		0.9300	
0.8480	0.0019	0.9160	0.0817
0.8480	0.0046	0.9450	0.3594
0.8480	0.0056	0.9390	0.3522
0.8480	0.0618	0.9490	0.4284
0.8480		1.1140	
0.8470	0.9486	0.5340	0.0655
0.8470	0.1325	0.8520	0.1977
0.8470	0.0736	0.8540	0.0699
0.8470	0.0184	1.0350	0.5939
0.8470	0.1469	1.0260	0.8418
0.8470	0.3328	1.0530	0.6565

0.8460		0.7590	
0.8460	0.0101	0.8440	0.0200
0.8460	0.0122	0.8610	0.1324
0.8460	0.0007	0.8770	0.0015
0.8460	0.0263	0.8860	0.0123
0.8460	0.0426	0.8540	0.0646
0.8460	0.0568	0.9330	0.3145
0.8460	0.0078	0.9520	0.7142
0.8460		1.0770	
0.8460	0.0027	1.0000	0.9924
0.8460	0.1834	1.1050	0.3292
0.8450		0.7610	
0.8450	0.3088	0.8530	0.2333
0.8450		0.8400	
0.8450	0.0030	0.8120	0.0030
0.8450	0.0404	0.8640	0.0130
0.8450		0.8900	
0.8450	0.2165	0.8700	0.2860
0.8450	0.1227	0.8860	0.2701
0.8450		0.9160	
0.8450	0.0472	0.9260	0.0401
0.8450	0.0220	0.9180	0.3814
0.8450	0.0756	0.9490	0.4243
0.8450	0.5708	1.0710	0.7617
0.8440	0.1471	0.7510	0.0269
0.8440	0.0835	0.8650	0.1011
0.8440	0.0342	0.9130	0.1414
0.8440	0.0245	0.9670	0.5599
0.8440	0.0235	0.9630	0.6247
0.8440	0.1367	0.9690	0.6429
0.8430	0.0714	0.7440	0.0070
0.8430	0.0469	0.8880	0.2562
0.8430	0.0065	0.8640	0.0000

0.8430	0.1203	0.8930	0.5009
0.8430	0.0175	0.9080	0.1653
0.8430	0.0105	0.8860	0.0247
0.8430	0.2103	0.8950	0.4282
0.8430	0.0509	0.8930	0.2274
0.8430	0.0372	0.9160	0.1903
0.8430		1.0450	
0.8420		0.6620	
0.8420	0.0228	0.8460	0.0059
0.8420		0.8740	
0.8420	0.1139	0.8990	0.5054
0.8420	0.0034	0.9010	0.0375
0.8420	0.0800	0.9010	0.6840
0.8420	0.0515	0.9430	0.6531
0.8420	0.0020	0.9660	0.3694
0.8410	0.7059	0.4570	0.0418
0.8410	0.2854	0.8420	0.0745
0.8410	0.0473	0.8680	0.1703
0.8410	0.0067	0.8740	0.0287
0.8410	0.0062	0.9230	0.3076
0.8410	0.0709	0.9520	0.5450
0.8410		0.9560	
0.8410	0.0186	0.9350	0.3330
0.8410		1.3880	
0.8410	0.5263	1.4610	0.3477
0.8400	0.3049	0.8090	0.2669
0.8400	0.1413	0.8460	0.1833
0.8400	0.0060	0.8800	0.1215
0.8400	0.0034	0.8830	0.1717
0.8400	0.0624	0.8810	0.1459
0.8400	0.0020	0.9330	0.2393
0.8400	0.0045	0.9220	0.2167
0.8400	0.0012	0.9370	0.0471

0.8400	0.0169	0.9420	0.3058
0.8400	0.2003	1.2680	0.2261
0.8390	0.0530	0.7990	0.0340
0.8390	0.0845	0.8060	0.0789
0.8390	0.0823	0.8240	0.0322
0.8390		0.8760	
0.8390	0.0011	0.8660	0.0013
0.8390	0.0944	0.8290	0.0837
0.8390	0.0287	0.9260	0.2226
0.8390	0.0024	0.9140	0.0808
0.8390	0.0207	0.9390	0.4491
0.8390	0.0466	0.9460	0.8178
0.8390	0.0548	0.9670	0.8253
0.8390	0.2599	1.0680	0.6617
0.8390	0.1071	1.0300	0.8382
0.8390	0.0190	1.0700	0.4174
0.8380		0.7370	
0.8380	0.2797	0.7220	0.0001
0.8380		0.8630	
0.8380	0.0329	0.8800	0.0888
0.8380	0.0068	0.8780	0.0175
0.8380		0.9060	
0.8380	0.0521	0.9500	0.5276
0.8380	0.1782	0.9670	0.8788
0.8380	0.4441	1.0780	0.6398
0.8370		0.7830	
0.8370		0.8400	
0.8370	0.0102	0.8600	0.0223
0.8370	0.0698	0.8140	0.0736
0.8370	0.0016	0.8560	0.0019
0.8370	0.0111	0.8790	0.0960
0.8370	0.0018	0.8910	0.0444
0.8370	0.0081	0.9030	0.1145

0.8370	0.0820	0.9100	0.8204
0.8370	0.0242	0.9330	0.2504
0.8370	0.0345	0.9540	0.8592
0.8370	0.0961	0.9980	0.9563
0.8370	0.1883	1.1370	0.2725
0.8370	0.1492	1.3470	0.0577
0.8360	0.1891	0.6990	0.0181
0.8360		0.8310	
0.8360	0.0080	0.8610	0.0507
0.8360	0.0039	0.8630	0.0124
0.8360	0.0010	0.9070	0.0927
0.8360	0.0028	0.9350	0.1004
0.8360	0.0017	0.9550	0.2884
0.8360	0.0960	0.9980	0.9450
0.8360	0.3479	1.0660	0.7450
0.8350	0.0121	0.8160	0.0126
0.8350	0.0049	0.8480	0.0266
0.8350	0.0050	0.8680	0.0464
0.8350	0.0054	0.8830	0.0670
0.8350	0.0305	0.9560	0.4050
0.8350	0.0808	0.9720	0.7013
0.8350	0.0133	1.0070	0.9132
0.8350	0.0661	1.0460	0.5406
0.8350		1.1240	
0.8340	0.0147	0.8380	0.0066
0.8340		0.8860	
0.8340	0.0185	0.8920	0.2351
0.8340	0.0197	0.9250	0.4165
0.8340		1.0360	
0.8340		1.2690	
0.8330		0.7770	
0.8330	0.0158	0.8250	0.0074
0.8330	0.2655	0.8080	0.1167

0.8330	0.0821	0.8460	0.1437
0.8330	0.0353	0.8890	0.0032
0.8330		1.0030	
0.8330	0.3581	1.1950	0.3674
0.8320	0.4424	0.7160	0.2155
0.8320		0.8110	
0.8320	0.0386	0.8410	0.0480
0.8320	0.0377	0.8750	0.2116
0.8320		0.8930	
0.8320	0.0464	0.8850	0.1902
0.8320	0.0395	0.8900	0.1934
0.8320	0.0508	0.9150	0.1542
0.8320	0.0510	0.9000	0.1403
0.8320	0.0653	0.9400	0.5953
0.8320	0.0070	0.9760	0.6335
0.8320	0.0087	0.9280	0.2697
0.8310		0.8070	
0.8310		0.8030	
0.8310		0.8070	
0.8310	0.3180	0.7890	0.3070
0.8310		0.8700	
0.8310	0.0071	0.8430	0.1479
0.8310	0.0452	0.9410	0.4705
0.8310	0.0184	0.9440	0.4886
0.8310	0.0775	1.2100	0.2101
0.8310	0.3447	1.6000	0.1050
0.8300	0.2172	0.8000	0.1708
0.8300	0.0482	0.8290	0.0677
0.8300		0.8670	
0.8300	0.0051	0.8600	0.0565
0.8300	0.0172	0.8920	0.1240
0.8300	0.2010	0.8870	0.3964
0.8300	0.0586	0.8970	0.1433

0.8300	0.1050	0.8900	0.3426
0.8300	0.3785	0.9320	0.6926
0.8300	0.2680	0.9140	0.5703
0.8300		1.1170	
0.8290	0.1049	0.7980	0.0473
0.8290		0.8100	
0.8290	0.0111	0.8750	0.0659
0.8290	0.1286	0.8840	0.2015
0.8290		0.9350	
0.8290	0.0654	0.9780	0.8606
0.8290		1.0420	
0.8290	0.0249	1.0100	0.8991
0.8290	0.3570	1.3660	0.0535
0.8280		0.7810	
0.8280		0.8430	
0.8280		0.8880	
0.8280	0.0155	0.9210	0.0591
0.8280	0.2837	0.8830	0.4802
0.8280	0.1929	1.3120	0.0713
0.8270	0.0140	0.8180	0.0356
0.8270		0.8870	
0.8270	0.0046	0.8840	0.0150
0.8270	0.0024	0.8940	0.0290
0.8270	0.0041	0.8770	0.2146
0.8270	0.1226	0.9200	0.6283
0.8270	0.0195	0.9210	0.2101
0.8270	0.4145	0.9500	0.8577
0.8260		0.8460	
0.8260	0.0881	0.8900	0.3510
0.8260	0.0080	0.8940	0.1888
0.8260	0.2214	0.8950	0.4241
0.8260	0.3856	1.3650	0.3172
0.8260		1.6560	

0.8250		0.7840	
0.8250	0.6742	0.7470	0.6931
0.8250	0.0409	0.7550	0.0263
0.8250	0.0311	0.8100	0.0656
0.8250	0.0092	0.8450	0.0025
0.8250	0.0681	0.8830	0.2203
0.8250	0.0132	0.9190	0.2769
0.8250	0.0038	0.9050	0.1314
0.8250	0.0005	0.9450	0.1220
0.8250		0.9460	
0.8250	0.0019	0.9720	0.5440
0.8250		1.0140	
0.8250		1.2320	
0.8240		0.7310	
0.8240	0.0432	0.7890	0.0019
0.8240	0.0594	0.8260	0.1063
0.8240	0.0055	0.8450	0.0201
0.8240	0.0159	0.8710	0.0332
0.8240	0.0039	0.9270	0.2336
0.8240	0.1482	0.9100	0.3623
0.8240		0.9400	
0.8240	0.1269	0.8880	0.3432
0.8240	0.0397	0.9120	0.1552
0.8240	0.0820	0.9710	0.7516
0.8240	0.1960	1.1830	0.3937
0.8240	0.4430	1.2040	0.5079
0.8240	0.1646	1.3000	0.1527
0.8230	0.1107	0.7560	0.0366
0.8230	0.0100	0.8400	0.0243
0.8230	0.0033	0.8630	0.0013
0.8230	0.0754	0.8800	0.2292
0.8230	0.0197	0.9080	0.2023
0.8230	0.0350	0.9070	0.2188

0.8230	0.1982	1.0220	0.8829
0.8220		0.8070	
0.8220		0.8330	
0.8220		0.8610	
0.8220	0.0040	0.8400	0.0000
0.8220	0.0072	0.8900	0.0574
0.8220	0.0032	0.8820	0.0015
0.8220	0.2097	0.8670	0.1111
0.8220	0.0029	0.9160	0.0317
0.8220	0.0116	0.9220	0.2249
0.8220	0.0581	0.9320	0.2547
0.8220	0.0113	0.9850	0.8112
0.8220		1.0150	
0.8220	0.3236	1.1400	0.3617
0.8220	0.3693	1.4710	0.0157
0.8210	0.2444	0.7480	0.2515
0.8210	0.0036	0.8180	0.0051
0.8210	0.0118	0.8420	0.0268
0.8210	0.0098	0.8770	0.0605
0.8210		0.9120	
0.8210	0.0010	0.8840	0.0089
0.8210	0.0313	0.9230	0.1942
0.8210	0.0823	0.8960	0.3839
0.8210		1.0140	
0.8210	0.1698	1.0430	0.7073
0.8210		1.0990	
0.8210	0.5096	1.5140	0.0861
0.8200	0.0154	0.8860	0.2061
0.8200	0.0107	0.8600	0.0190
0.8200	0.0006	0.8690	0.0257
0.8200	0.0234	0.9190	0.1724
0.8190		0.7740	
0.8190		0.7850	

0.8190	0.0312	0.7850	0.0111
0.8190	0.0258	0.8190	0.2872
0.8190		0.8500	
0.8190	0.1577	0.8230	0.0829
0.8190	0.0225	0.8210	0.1482
0.8190	0.1157	0.8170	0.1091
0.8190	0.0682	0.8290	0.1514
0.8190	0.0060	0.8380	0.0242
0.8190	0.0243	0.9130	0.2698
0.8190	0.1928	1.0510	0.7727
0.8180	0.0081	0.7620	0.0035
0.8180		0.7750	
0.8180		0.8140	
0.8180	0.0052	0.8470	0.0136
0.8180	0.0160	0.8740	0.0206
0.8180	0.0051	0.8950	0.1960
0.8180	0.0295	0.9190	0.2286
0.8180	0.1711	0.9200	0.6733
0.8180	0.0529	0.9960	0.9743
0.8180	0.1439	1.3480	0.1360
0.8170		0.7720	
0.8170	0.0202	0.8200	0.0033
0.8170	0.0714	0.8270	0.1105
0.8170	0.0094	0.8490	0.0068
0.8170	0.0044	0.8520	0.0045
0.8170	0.2249	0.8160	0.2674
0.8170	0.7328	0.8350	0.5208
0.8170	0.0019	0.8900	0.0594
0.8170	0.0014	0.9070	0.0535
0.8170	0.2760	0.9660	0.9742
0.8170	0.0574	1.0030	0.9597
0.8160	0.0158	0.7770	0.0076
0.8160	0.1187	0.8420	0.0251

0.8160		0.8850	
0.8160	0.0967	0.8640	0.1421
0.8160	0.1652	1.0850	0.2257
0.8160		1.2630	
0.8150	0.0008	0.8040	0.0115
0.8150	0.0496	0.8290	0.0513
0.8150	0.0019	0.8840	0.0008
0.8150	0.0198	0.8700	0.1000
0.8150	0.0153	0.8750	0.0714
0.8150	0.0126	0.8660	0.0034
0.8150	0.0416	0.9160	0.1024
0.8150	0.0109	0.9580	0.5134
0.8140	0.0755	0.8160	0.1604
0.8140	0.0355	0.8140	0.0144
0.8140	0.0157	0.8510	0.0450
0.8140	0.0038	0.8970	0.1707
0.8140	0.1474	0.9430	0.7953
0.8140		1.0310	
0.8140	0.3537	1.2690	0.4957
0.8130	0.0023	0.8160	0.0001
0.8130		0.8580	
0.8130	0.0168	0.8660	0.0321
0.8130	0.0029	0.8660	0.0099
0.8130	0.0163	0.9120	0.2663
0.8130	0.0049	0.9350	0.2255
0.8130	0.0198	0.9330	0.7394
0.8130	0.0343	0.9330	0.2190
0.8120	0.0090	0.8360	0.0394
0.8120	0.0137	0.8530	0.1951
0.8120	0.0080	0.8640	0.0286
0.8120	0.1635	0.8880	0.2181
0.8120	0.0618	0.8230	0.0474
0.8120	0.1995	0.8960	0.4923

0.8120	0.0053	0.9030	0.0526
0.8120	0.1570	0.9320	0.7525
0.8120	0.0061	0.9760	0.5329
0.8120		1.0040	
0.8120		1.0310	
0.8120		1.0820	
0.8120	0.0853	1.1060	0.2295
0.8110		0.6600	
0.8110	0.0134	0.7990	0.0472
0.8110	0.0564	0.8600	0.0903
0.8110	0.0070	0.8790	0.0793
0.8110	0.2330	1.0180	0.9463
0.8100	0.0040	0.8020	0.0206
0.8100		0.8900	
0.8100	0.0039	0.9130	0.0980
0.8100	0.0398	0.8980	0.0546
0.8100	0.1251	0.9410	0.5615
0.8100	0.1804	1.1940	0.2262
0.8090		0.6950	
0.8090	0.1391	0.7080	0.0221
0.8090		0.8350	
0.8090	0.0314	0.8080	0.0020
0.8090		0.8900	
0.8090	0.0002	0.8740	0.0713
0.8090	0.0864	0.9620	0.4080
0.8090	0.4457	0.9250	0.6588
0.8090		1.0610	
0.8080		0.7790	
0.8080	0.0947	0.7840	0.0256
0.8080	0.0077	0.8330	0.0124
0.8080	0.0255	0.8280	0.0369
0.8080	0.0082	0.8460	0.0593
0.8080		0.8870	

0.8080	0.0102	0.8880	0.0708
0.8080	0.0028	0.8970	0.0588
0.8080	0.0008	0.9060	0.1904
0.8080		0.9430	
0.8080	0.1218	0.9340	0.5948
0.8080	0.0145	0.9610	0.8547
0.8080	0.0115	1.1590	0.1931
0.8070		0.8190	
0.8070	0.0112	0.8750	0.0188
0.8070		0.9920	
0.8070	0.1708	1.0110	0.9179
0.8060		0.5850	
0.8060	0.0733	0.7540	0.0267
0.8060	0.0023	0.8110	0.0012
0.8060		0.8380	
0.8060	0.0002	0.8150	0.0171
0.8060	0.0057	0.8610	0.0022
0.8060	0.0007	0.8630	0.0118
0.8060	0.0289	0.9960	0.9510
0.8060		1.0300	
0.8060	0.2776	1.0640	0.4853
0.8050	0.0136	0.7970	0.0168
0.8050	0.0321	0.8360	0.0614
0.8050	0.0153	0.8510	0.0781
0.8050	0.0011	0.8920	0.0544
0.8050	0.0030	0.8990	0.2447
0.8050	0.0218	0.9100	0.1353
0.8050	0.0198	0.9320	0.1935
0.8050	0.0195	1.0100	0.8445
0.8040		0.6710	
0.8040	0.0241	0.7530	0.0174
0.8040	0.0388	0.7850	0.0213
0.8040	0.0404	0.7760	0.0220

0.8040	0.0429	0.8300	0.0376
0.8040		0.8800	
0.8040		0.9050	
0.8040	0.0024	0.9500	0.3410
0.8040	0.0017	0.8990	0.0423
0.8040	0.0470	0.9200	0.3318
0.8040	0.0739	0.9020	0.2417
0.8040	0.0070	0.9460	0.1904
0.8040	0.0068	0.9570	0.6484
0.8040	0.0031	1.0520	0.5294
0.8030	0.8142	0.3920	0.0718
0.8030		0.7590	
0.8030	0.0215	0.8200	0.0112
0.8030	0.0477	0.8610	0.2091
0.8030		0.9040	
0.8030	0.0441	0.9060	0.0998
0.8030		1.0160	
0.8030	0.0210	0.9900	0.9108
0.8020	0.0558	0.8000	0.0746
0.8020	0.0027	0.7960	0.0017
0.8020	0.0507	0.9300	0.3973
0.8020	0.2191	1.4990	0.0835
0.8010	0.0123	0.7600	0.0023
0.8010	0.0393	0.7940	0.0535
0.8010	0.0657	0.8090	0.1877
0.8010	0.0053	0.8370	0.0539
0.8010	0.0092	0.8510	0.0715
0.8010	0.0369	0.8690	0.1617
0.8010		0.9310	
0.8010	0.0146	0.9210	0.2019
0.8010	0.0352	0.9320	0.5949
0.8010	0.0081	0.9120	0.2611
0.8010	0.0124	1.0130	0.8657

0.8010	0.1103	1.2400	0.3041
0.8010	0.0798	1.2460	0.1064
0.8010	0.2475	1.2520	0.1672
0.8000		0.8590	
0.8000	0.0149	0.8460	0.0580
0.8000	0.0387	0.8490	0.0987
0.8000	0.0265	0.8810	0.1486
0.8000	0.0018	0.9590	0.4381
0.8000	0.0085	1.0040	0.9439
0.8000	0.0057	0.9830	0.8579
0.7990	0.3607	0.5770	0.0404
0.7990	0.0249	0.8590	0.1865
0.7990		0.9050	
0.7990	0.0020	0.9230	0.4763
0.7990	0.0245	1.0170	0.8532
0.7990		1.1020	
0.7980		0.8310	
0.7980	0.0330	0.8570	0.0756
0.7980	0.0002	0.8670	0.0288
0.7980	0.0007	0.9080	0.0164
0.7980	0.0202	0.9240	0.3274
0.7980	0.0544	0.9190	0.3621
0.7980	0.0393	0.9720	0.8600
0.7980	0.3812	0.9570	0.8766
0.7970	0.0444	0.7370	0.0078
0.7970	0.0313	0.7770	0.0695
0.7970	0.1000	0.8490	0.1263
0.7970	0.0206	0.8700	0.1364
0.7970	0.0100	0.8890	0.1124
0.7970	0.0019	0.8640	0.0079
0.7970		0.9160	
0.7970	0.3088	0.9260	0.4999
0.7970	0.0373	1.0300	0.7538

0.7970	0.2055	1.0270	0.8380
0.7970	0.1253	1.1350	0.3206
0.7960	0.0245	0.7830	0.0076
0.7960	0.0104	0.8070	0.0816
0.7960	0.0320	0.8420	0.0306
0.7960	0.0022	0.8620	0.0187
0.7960	0.0245	0.8830	0.3318
0.7960	0.0014	0.8940	0.0714
0.7950	0.0815	0.7740	0.0586
0.7950	0.0170	0.7930	0.0333
0.7950	0.0133	0.8240	0.0192
0.7950	0.0137	0.8120	0.0338
0.7950	0.0008	0.8480	0.0035
0.7950	0.0043	0.8430	0.0229
0.7950	0.0104	0.8650	0.2005
0.7950	0.0110	0.8830	0.2728
0.7950		0.9460	
0.7940		0.7730	
0.7940		0.7790	
0.7940		0.8090	
0.7940	0.1016	0.7800	0.0558
0.7940	0.0296	0.7820	0.0577
0.7940	0.0083	0.8010	0.0367
0.7940	0.0935	0.7850	0.2940
0.7940	0.0278	0.8290	0.0490
0.7940	0.0034	0.8360	0.0706
0.7940	0.0122	0.8370	0.1095
0.7940	0.0014	0.8340	0.0057
0.7940	0.0152	0.8240	0.0287
0.7940	0.0356	0.8900	0.2083
0.7940		1.0210	
0.7940	0.1459	1.0880	0.3912
0.7940	0.2506	1.0800	0.6681

0.7930	0.0079	0.7960	0.0004
0.7930	0.0121	0.8060	0.0670
0.7930		0.8500	
0.7930	0.0066	0.8160	0.0044
0.7930	0.0085	0.8410	0.0086
0.7930	0.0036	0.8820	0.1160
0.7930	0.0189	0.9470	0.4204
0.7930	0.0391	0.9500	0.5349
0.7920	0.0238	0.7380	0.0232
0.7920	0.0421	0.7890	0.0352
0.7920	0.0235	0.8390	0.0321
0.7920	0.0049	0.8880	0.1597
0.7920	0.0227	0.8750	0.3082
0.7920	0.0006	0.8740	0.0441
0.7920	0.0570	0.9240	0.3230
0.7920	0.0740	0.9340	0.6007
0.7920	0.0596	1.0360	0.7611
0.7910		0.6930	
0.7910		0.8180	
0.7910	0.0085	0.8000	0.0039
0.7910	0.0258	0.8180	0.0605
0.7910	0.0085	0.8800	0.2173
0.7910	0.0088	0.8800	0.2520
0.7910	0.0062	0.9070	0.2441
0.7910	0.0272	0.8910	0.0827
0.7910	0.0214	0.8780	0.1024
0.7910	0.0538	0.8490	0.0407
0.7910	0.0378	0.9010	0.4296
0.7910	0.0098	0.9890	0.8536
0.7910	0.0191	1.1580	0.1325
0.7900	0.0520	0.7570	0.0257
0.7900	0.0084	0.7910	0.0626
0.7900	0.0298	0.7920	0.0311

0.7900		0.8470	
0.7900	0.0273	0.8510	0.1179
0.7900	0.0042	0.8830	0.0311
0.7900	0.0001	0.8570	0.0391
0.7890	0.0273	0.8180	0.0170
0.7890		0.8610	
0.7890	0.0135	0.8350	0.1429
0.7890		0.8710	
0.7890		0.8740	
0.7890	0.0406	0.8430	0.1234
0.7890	0.0022	0.8500	0.0499
0.7890	0.0167	0.8840	0.3234
0.7890	0.0987	1.1000	0.4927
0.7880		0.6880	
0.7880	0.0136	0.8310	0.1001
0.7880		0.8180	
0.7880	0.1489	0.7890	0.1700
0.7880	0.0083	0.8450	0.0764
0.7880		0.8840	
0.7880	0.0083	0.8730	0.0525
0.7880	0.0151	0.8590	0.1510
0.7880	0.0383	0.8790	0.1440
0.7880	0.0785	0.8570	0.3564
0.7880	0.0590	0.9290	0.4910
0.7870	0.0453	0.7670	0.0583
0.7870		0.8320	
0.7870	0.0084	0.8280	0.1355
0.7870	0.0022	0.8420	0.0083
0.7870	0.0084	0.8530	0.0612
0.7870	0.0047	0.8510	0.0158
0.7870	0.0149	0.8600	0.0175
0.7870	0.0472	0.8620	0.1920
0.7870	0.0410	0.8970	0.1679

0.7870	0.0282	0.8720	0.2500
0.7870		1.0430	
0.7870	0.1029	1.0780	0.4261
0.7870	0.1517	1.2400	0.1438
0.7860	0.0048	0.8620	0.0055
0.7860	0.0118	0.8580	0.1498
0.7860	0.4009	1.4610	0.3219
0.7860		1.6520	
0.7850		0.7630	
0.7850	0.0132	0.7490	0.0059
0.7850	0.0019	0.8060	0.0192
0.7850		0.8390	
0.7850	0.0692	0.8180	0.0860
0.7850	0.0026	0.8260	0.0195
0.7850	0.0044	0.8450	0.0118
0.7850	0.0017	0.8750	0.1058
0.7850	0.0021	0.8630	0.0295
0.7850	0.0074	0.8790	0.0925
0.7850	0.0077	0.9280	0.4684
0.7850	0.0090	1.0120	0.9051
0.7850		1.2140	
0.7840	0.1617	0.7230	0.0736
0.7840	0.0264	0.8360	0.1721
0.7840	0.0154	0.8270	0.0872
0.7840	0.0166	0.8090	0.0370
0.7840	0.0208	0.8310	0.0663
0.7840	0.0466	0.8080	0.1551
0.7840	0.0149	0.8800	0.1477
0.7840	0.1332	0.8550	0.0742
0.7840	0.0002	0.9180	0.1382
0.7840		0.9990	
0.7830		0.7720	
0.7830	0.0595	0.7570	0.0409

0.7830	0.0170	0.8420	0.0702
0.7830	0.0103	0.8690	0.0530
0.7830	0.0632	0.9500	0.8408
0.7830		0.9780	
0.7830	0.0480	0.9650	0.6487
0.7830	0.1677	1.1310	0.4307
0.7830	0.1553	1.0490	0.7363
0.7830	0.1137	1.2100	0.2641
0.7820	0.0086	0.8190	0.0308
0.7820	0.0503	0.8520	0.1797
0.7810		0.7290	
0.7810	0.0076	0.8280	0.0361
0.7810	0.0126	0.8440	0.0847
0.7810	0.0004	0.8390	0.0871
0.7810	0.0019	0.8650	0.0672
0.7810	0.0092	0.9040	0.1591
0.7810	0.4055	1.3260	0.4687
0.7810		1.3600	
0.7800		0.8010	
0.7800	0.0012	0.8200	0.0030
0.7800	0.1218	0.8670	0.2112
0.7800	0.0122	0.9210	0.1104
0.7800	0.0204	0.9500	0.6506
0.7800	0.1467	1.2440	0.1938
0.7790	0.0668	0.7920	0.1755
0.7790		0.8290	
0.7790	0.0233	0.8140	0.0095
0.7790	0.0077	0.8270	0.0524
0.7790		0.8690	
0.7790	0.0063	0.8480	0.0902
0.7790	0.2647	0.8160	0.3712
0.7790	0.0089	0.8930	0.3780
0.7790	0.0005	0.9010	0.1232

0.7790	0.0382	0.9700	0.8136
0.7790		1.0830	
0.7790	0.1290	1.1790	0.4308
0.7790		1.2660	
0.7780	0.1110	0.7420	0.3918
0.7780		0.8260	
0.7780	0.0024	0.8460	0.0153
0.7780	0.0017	0.8340	0.0259
0.7780	0.0189	0.8620	0.0585
0.7780	0.0001	0.8950	0.0816
0.7780	0.0773	0.8920	0.2632
0.7780	0.0063	0.9370	0.3243
0.7780	0.0182	0.9710	0.0053
0.7780	0.0649	1.1120	0.3730
0.7780	0.0040	1.1220	0.3096
0.7770		0.4960	
0.7770	0.0579	0.6570	0.0473
0.7770		0.8130	
0.7770	0.0334	0.8380	0.0677
0.7770	0.0032	0.8610	0.0503
0.7770	0.0323	0.8860	0.2079
0.7770	0.0050	0.8530	0.0128
0.7770	0.0067	0.8880	0.4213
0.7770	0.0358	1.0040	0.9685
0.7770	0.0041	0.9840	0.8241
0.7760	0.0005	0.7860	0.0007
0.7760	0.0210	0.8020	0.0251
0.7760	0.0051	0.8170	0.0186
0.7760	0.0026	0.8120	0.0380
0.7760	0.0075	0.8530	0.1231
0.7760	0.0132	0.8560	0.0892
0.7760	0.0556	1.0060	0.9984
0.7750	0.0343	0.7530	0.0264

0.7750		0.8090	
0.7750		0.8780	
0.7750	0.0773	0.8240	0.3171
0.7750	0.0198	0.8910	0.2903
0.7750	0.0023	0.9120	0.2688
0.7750	0.3709	0.9460	0.8081
0.7740	0.0076	0.9490	0.5156
0.7740		1.0220	
0.7740	0.0236	1.0730	0.0913
0.7740	0.2863	1.2220	0.6139
0.7740		1.2410	
0.7730	0.0132	0.6400	0.0009
0.7730		0.9230	
0.7730		0.9870	
0.7730	0.0202	0.9870	0.8666
0.7720	0.0299	0.8160	0.1340
0.7720	0.0019	0.8260	0.0075
0.7720		0.8620	
0.7720	0.0059	0.8130	0.0107
0.7720	0.0196	0.8350	0.0445
0.7720	0.0031	0.8570	0.0421
0.7720	0.0020	0.8710	0.0329
0.7720	0.0070	0.9160	0.3918
0.7720	0.2582	1.0050	0.9654
0.7710	0.0242	0.7040	0.0160
0.7710	0.0104	0.8270	0.0178
0.7710	0.0018	0.8400	0.0062
0.7710	0.0058	0.8440	0.0268
0.7710	0.0020	0.8340	0.0177
0.7710	0.0199	0.9450	0.5773
0.7710	0.0348	0.9260	0.8246
0.7700		0.6720	
0.7700	0.4873	0.7590	0.1382

0.7700	0.0072	0.7760	0.0116
0.7700	0.0096	0.8160	0.0494
0.7700	0.0286	0.8320	0.1828
0.7700		0.8930	
0.7700	0.0768	0.8960	0.5046
0.7700	0.2484	1.5170	0.1152
0.7690	0.0069	0.7420	0.0035
0.7690	0.0093	0.7960	0.0053
0.7690	0.0145	0.8240	0.0490
0.7690	0.0046	0.8570	0.1363
0.7690	0.0007	0.8590	0.0359
0.7690	0.0003	0.8590	0.0140
0.7690	0.0043	0.8810	0.0965
0.7690	0.0330	0.9120	0.2560
0.7690		1.0030	
0.7690	0.1387	1.1400	0.3232
0.7690	0.0676	1.2190	0.0789
0.7690	0.1540	1.2870	0.0899
0.7690		1.2500	
0.7690	0.3069	1.3360	0.3693
0.7680	0.0082	0.8130	0.0115
0.7680	0.0020	0.8280	0.0892
0.7680		0.8680	
0.7680	0.0001	0.8620	0.1337
0.7680	0.0001	0.9180	0.1909
0.7680	0.0023	0.9760	0.9187
0.7670	0.0297	0.7950	0.0072
0.7670	0.0007	0.8140	0.0078
0.7670	0.0499	0.8510	0.3284
0.7670		0.8630	
0.7670	0.3106	0.9430	0.7932
0.7660	0.0536	0.7240	0.0335
0.7660	0.0292	0.7650	0.0177

0.7660	0.0068	0.8400	0.0109
0.7660	0.0039	0.8610	0.1119
0.7660		0.8890	
0.7660	0.0016	0.8680	0.1198
0.7660	0.0047	0.8670	0.0835
0.7660	0.0104	0.8910	0.1635
0.7660	0.0539	0.8700	0.2796
0.7660		0.9810	
0.7660	0.0031	0.9050	0.4005
0.7650	0.0169	0.7960	0.1313
0.7650	0.0135	0.7960	0.0409
0.7650	0.0054	0.8420	0.0239
0.7650	0.0044	0.8520	0.0164
0.7650	0.2016	0.8290	0.4610
0.7650	0.0054	0.8760	0.0158
0.7650	0.0034	0.8960	0.2980
0.7650	0.0014	0.9150	0.2258
0.7650	0.0641	1.0080	0.9240
0.7640	0.0006	0.8400	0.0018
0.7640	0.0144	0.8400	0.0592
0.7640	0.0305	0.9060	0.1564
0.7630		0.8360	
0.7630	0.0009	0.8090	0.0173
0.7630	0.0015	0.8240	0.0093
0.7630	0.0130	0.8260	0.0202
0.7630	0.0020	0.8240	0.0057
0.7630	0.0076	0.8560	0.0446
0.7630	0.0022	0.8960	0.1499
0.7630	0.0123	0.9210	0.3028
0.7620		0.7190	
0.7620	0.1323	0.7730	0.1360
0.7620	0.0221	0.7840	0.0408
0.7620	0.0017	0.8220	0.0390

0.7620	0.0041	0.8220	0.0069
0.7620	0.0018	0.8380	0.1098
0.7620	0.0083	0.8640	0.0493
0.7620	0.0041	0.8830	0.0218
0.7620	0.0565	0.9430	0.5578
0.7610	0.0379	0.7160	0.0480
0.7610		0.7950	
0.7610	0.0087	0.8190	0.0281
0.7610	0.0175	0.8020	0.0565
0.7610	0.0738	0.8270	0.0782
0.7610	0.0012	0.8110	0.0098
0.7610	0.0070	0.8550	0.1260
0.7610	0.0176	0.8800	0.1856
0.7610	0.0413	0.9020	0.4001
0.7610	0.0054	0.9890	0.8383
0.7610	0.0283	1.0230	0.8438
0.7610		1.2470	
0.7600	0.0019	0.8300	0.0499
0.7600	0.0007	0.8360	0.0174
0.7600	0.0031	0.8330	0.0163
0.7600	0.0151	0.8500	0.0741
0.7600	0.0000	0.8700	0.0652
0.7600	0.0069	0.8990	0.3798
0.7600	0.1415	1.0190	0.9224
0.7600	0.3810	1.1220	0.8109
0.7590		0.8240	
0.7590	0.2886	0.7970	0.3702
0.7590	0.0330	0.8470	0.0768
0.7580	0.0440	0.6890	0.0235
0.7580	0.0145	0.7690	0.1039
0.7580		0.8390	
0.7580		0.8730	
0.7580	0.0082	0.8730	0.1291

0.7580		1.1140	
0.7580	0.4459	1.4120	0.5204
0.7570		0.7530	
0.7570		0.7910	
0.7570	0.0191	0.7950	0.0234
0.7570	0.0773	0.8120	0.4539
0.7570	0.0287	0.8330	0.6589
0.7570	0.0019	0.9240	0.2516
0.7570	0.0005	0.9940	0.9957
0.7570		1.0860	
0.7560	0.0459	0.7350	0.0517
0.7560	0.0648	0.7730	0.0206
0.7560	0.0145	0.8390	0.0583
0.7560	0.0050	0.9190	0.2799
0.7560	0.0342	1.0000	0.9480
0.7560	0.0118	0.9560	0.8676
0.7560	0.0062	0.9620	0.8679
0.7560	0.0046	0.9750	0.7573
0.7560	0.0011	1.0440	0.6416
0.7550		0.7520	
0.7550	0.0094	0.7570	0.0698
0.7550	0.0044	0.8150	0.1631
0.7550	0.1012	0.8250	0.2626
0.7550	0.0007	0.8720	0.1345
0.7550	0.0284	0.8930	0.3546
0.7550	0.0448	0.9580	0.5888
0.7550	0.0300	0.9890	0.8294
0.7550		1.0530	
0.7540	0.0247	0.8040	0.0070
0.7540	0.0236	0.8010	0.0180
0.7540	0.0054	0.7950	0.0089
0.7540	0.0126	0.8580	0.2762
0.7540	0.0019	0.8980	0.1947

0.7540	0.0555	0.9290	0.6973
0.7540	0.0040	0.9410	0.3866
0.7530		0.7080	
0.7530	0.0033	0.8050	0.0160
0.7530	0.0008	0.8340	0.0216
0.7530	0.0013	0.8390	0.0283
0.7530	0.0006	0.8440	0.0190
0.7530	0.0029	0.8400	0.0252
0.7530	0.0226	0.8970	0.2305
0.7530	0.0324	0.9930	0.9746
0.7530		1.1070	
0.7530	0.1087	1.1220	0.4430
0.7520	0.0971	0.7300	0.2596
0.7520	0.0226	0.7830	0.1258
0.7520	0.0060	0.8000	0.0172
0.7520	0.0040	0.8330	0.0369
0.7520	0.0051	0.8560	0.1084
0.7520	0.0199	1.0380	0.7476
0.7520		1.1730	
0.7510	0.0029	0.8190	0.0348
0.7510	0.0038	0.8610	0.0684
0.7510	0.0972	0.8560	0.2034
0.7510	0.0003	0.8550	0.0658
0.7510	0.0017	0.9220	0.2932
0.7510	0.4448	1.1840	0.8122
0.7500	0.0021	0.7480	0.0326
0.7500	0.0159	0.8080	0.1000
0.7500	0.1784	0.8440	0.3795
0.7500	0.1213	0.8990	0.3489
0.7500	0.0028	0.9700	0.8167
0.7500		1.3150	
0.7490	0.0029	0.8110	0.0189
0.7490	0.0032	0.8240	0.0812

0.7490	0.0011	0.8240	0.0171
0.7490		0.9770	
0.7480		0.7330	
0.7480	0.0357	0.7040	0.0328
0.7480		0.7580	
0.7480	0.0036	0.7910	0.0104
0.7480	0.0022	0.9160	0.3505
0.7480	0.1008	0.9860	0.8310
0.7480	0.3492	1.2360	0.3070
0.7470	0.0005	0.7270	0.0086
0.7470	0.0145	0.7350	0.0018
0.7470	0.4027	0.8170	0.3301
0.7470	0.0012	0.8410	0.0042
0.7470	0.0007	0.8380	0.0392
0.7470	0.0018	0.8910	0.0807
0.7470	0.0070	0.9340	0.5220
0.7470		1.0020	
0.7470	0.1189	1.0140	0.9832
0.7460		0.7680	
0.7460	0.1718	0.7860	0.0328
0.7460	0.0072	0.8060	0.0144
0.7460		0.8400	
0.7460	0.0000	0.8830	0.0157
0.7450		0.7750	
0.7450	0.0014	0.8210	0.0281
0.7450	0.0050	0.8330	0.0860
0.7450	0.0120	0.8390	0.1282
0.7450	0.0221	0.8830	0.3237
0.7440	0.0063	0.7140	0.0018
0.7440	0.0100	0.7980	0.0054
0.7440	0.0017	0.7940	0.0224
0.7430		0.7180	
0.7430	0.0269	0.7300	0.0318

0.7430	0.0056	0.8300	0.0551
0.7430	0.0002	0.9100	0.0395
0.7430	0.0683	0.9240	0.6642
0.7430	0.2367	1.4850	0.2380
0.7420		0.6710	
0.7420	0.0148	0.7790	0.0484
0.7420	0.0002	0.8640	0.0789
0.7420	0.0022	0.8680	0.0488
0.7420	0.0788	0.8290	0.3865
0.7420	0.0028	0.8910	0.3015
0.7420	0.0138	0.9300	0.1418
0.7420	0.1323	1.0260	0.7149
0.7410		0.6870	
0.7410		0.8130	
0.7410	0.0572	0.7830	0.0532
0.7410	0.0008	0.9240	0.3555
0.7400		0.7860	
0.7400	0.0110	0.7830	0.0065
0.7400	0.0314	0.7860	0.0731
0.7400	0.0009	0.8260	0.0294
0.7400	0.0004	0.8400	0.0127
0.7400	0.0860	0.8230	0.2567
0.7390		0.8360	
0.7390	0.0131	0.7970	0.0326
0.7390	0.0656	0.7970	0.1759
0.7390	0.0063	0.8040	0.0422
0.7390	0.0176	0.9750	0.7858
0.7380		0.7340	
0.7380	0.0636	0.8330	0.0499
0.7380		0.8610	
0.7380	0.0035	0.8250	0.0448
0.7380	0.0003	0.8610	0.1024
0.7380	0.0018	0.8690	0.1142

0.7380	0.2272	1.1270	0.8659
0.7380	0.2206	1.3330	0.2285
0.7370	0.1942	0.5520	0.0617
0.7370		0.7720	
0.7370	0.0085	0.7670	0.0257
0.7370	0.0008	0.8110	0.0193
0.7370	0.0017	0.8290	0.0405
0.7370	0.0088	0.8260	0.0386
0.7370	0.0005	0.8260	0.0208
0.7370	0.0132	0.8550	0.0700
0.7370	0.2121	1.3220	0.2002
0.7360	0.0064	0.7710	0.0169
0.7360	0.3235	0.7450	0.3999
0.7360	0.0018	0.8140	0.0388
0.7360	0.0026	0.8420	0.0734
0.7360	0.0094	0.8500	0.0564
0.7350	0.0003	0.8420	0.0347
0.7350	0.0793	0.9330	0.5182
0.7350	0.0381	1.0390	0.7345
0.7350	0.3001	1.2260	0.6602
0.7340	0.0037	0.7920	0.0217
0.7340	0.0104	0.8010	0.0154
0.7340	0.0280	0.8140	0.1253
0.7340	0.2948	2.2720	0.1354
0.7330		0.7890	
0.7330		0.7910	
0.7330	0.0131	0.7720	0.0440
0.7330	0.0028	0.7950	0.0153
0.7330	0.0133	0.8040	0.0588
0.7330		0.9080	
0.7330	0.0079	0.8750	0.1510
0.7330		0.8890	
0.7330	0.0086	0.9000	0.2398

0.7330	0.4143	0.9370	0.8321
0.7320	0.0096	0.7500	0.0018
0.7320	0.0055	0.7960	0.0530
0.7320	0.0178	0.8290	0.0490
0.7320	0.0008	0.8510	0.0536
0.7320	0.0001	0.8830	0.0748
0.7320	0.0037	0.8780	0.0657
0.7310	0.0469	0.7750	0.0255
0.7310	0.0094	0.7990	0.0580
0.7310	0.0009	0.7900	0.0183
0.7310	0.0014	0.8020	0.0472
0.7310	0.0007	0.8350	0.0397
0.7310	0.0003	0.8870	0.0683
0.7310	0.0425	0.9210	0.4135
0.7300		0.8030	
0.7300		0.8160	
0.7300	0.0032	0.7910	0.0214
0.7300	0.0104	0.7850	0.0338
0.7300	0.0062	0.8160	0.0633
0.7300	0.0412	0.8280	0.1041
0.7300		0.9350	
0.7300	0.4891	1.7260	0.4872
0.7290	0.0068	0.7690	0.0637
0.7290	0.0245	0.8970	0.4278
0.7290	0.0144	1.0570	0.6559
0.7290	0.7953	1.4040	0.0277
0.7280		0.7720	
0.7280	0.0022	0.7830	0.0258
0.7280		0.8470	
0.7280	0.0005	0.8080	0.0135
0.7280	0.0072	0.8340	0.0718
0.7280	0.0006	0.9000	0.0743
0.7280	0.0044	0.9060	0.2971

0.7280	0.0199	1.0230	0.7707
0.7270	0.0092	0.7420	0.0095
0.7270	0.0020	0.7740	0.0080
0.7270	0.0433	0.8060	0.0902
0.7270	0.0047	0.8360	0.0521
0.7270	0.0017	0.8210	0.0142
0.7270	0.0051	0.8600	0.5064
0.7260	0.0668	0.6670	0.0015
0.7260	0.0041	0.7870	0.0117
0.7260	0.0065	0.7820	0.0099
0.7260	0.0188	0.7900	0.1021
0.7260	0.0036	0.7970	0.1290
0.7260	0.0152	0.8410	0.0281
0.7260	0.0747	0.9080	0.7523
0.7250		0.8030	
0.7250	0.0050	0.8060	0.0432
0.7250	0.0101	0.8200	0.0738
0.7250	0.0000	0.8440	0.0668
0.7250	0.5947	1.4290	0.2986
0.7240		0.7870	
0.7240	0.0507	0.8220	0.2209
0.7240	0.0163	0.8420	0.3872
0.7240	0.1014	0.8490	0.2550
0.7240		1.3480	
0.7230	0.0016	0.7350	0.0021
0.7230	0.0125	0.7980	0.0447
0.7230	0.0005	0.8190	0.0393
0.7230	0.0251	0.8580	0.1437
0.7230	0.0112	0.8750	0.4434
0.7230	0.4217	1.2600	0.6889
0.7220		0.7470	
0.7220	0.0153	0.7270	0.0105
0.7220	0.3726	0.7380	0.4445

0.7220	0.0031	0.7700	0.0289
0.7220		0.7970	
0.7220	0.0120	0.7980	0.0408
0.7220	0.0034	0.7820	0.0249
0.7220	0.0179	0.8650	0.1516
0.7220	0.0122	0.9690	0.8574
0.7210	0.0023	0.7670	0.0092
0.7210	0.0093	0.8010	0.1107
0.7210	0.0033	0.8060	0.0504
0.7210	0.0236	0.8470	0.1822
0.7210		0.9080	
0.7210	0.0009	0.8840	0.1012
0.7200	0.0381	0.8960	0.2632
0.7190	0.0757	0.6850	0.0429
0.7190	0.0178	0.8440	0.0979
0.7190	0.0062	0.8430	0.0343
0.7190	0.0035	0.8820	0.2762
0.7190	0.6203	0.9380	0.7909
0.7190	0.0462	1.0290	0.7066
0.7190	0.1564	1.3390	0.2371
0.7180	0.0131	0.7510	0.0666
0.7180	0.0365	0.8750	0.3221
0.7180	0.2032	0.8980	0.0989
0.7170		0.6730	
0.7170	0.0234	0.6590	0.1148
0.7170	0.0326	0.7400	0.0341
0.7170	0.4085	0.9840	0.8946
0.7160	0.1452	1.0250	0.9275
0.7160	0.0533	1.0570	0.7467
0.7150	0.0136	0.7260	0.0220
0.7150	0.0016	0.8120	0.0301
0.7150	0.0026	0.8250	0.0620
0.7150	0.0039	0.8550	0.1415

0.7150	0.0778	0.8460	0.4492
0.7150		1.4390	
0.7140		0.8130	
0.7130	0.0015	0.8060	0.0072
0.7130	0.0007	0.8260	0.0280
0.7130	0.0068	0.8830	0.1548
0.7120	0.0209	0.6370	0.0260
0.7120	0.0006	0.7940	0.0074
0.7110	0.0267	0.6450	0.0146
0.7110	0.0399	0.6610	0.0115
0.7110		0.7500	
0.7110	0.0067	0.8000	0.0211
0.7110	0.0048	0.8370	0.1881
0.7100		0.7490	
0.7100	0.0020	0.7450	0.0315
0.7100	0.0035	0.7610	0.0021
0.7100	0.1229	0.7500	0.5728
0.7100	0.0001	0.8330	0.0063
0.7100		0.9040	
0.7100	0.0076	1.0090	0.8595
0.7090	0.0077	0.7490	0.0168
0.7090	0.0028	0.7950	0.0370
0.7090	0.2739	0.8490	0.6749
0.7090	0.0130	0.8580	0.1270
0.7080	0.1310	0.5840	0.1037
0.7080	0.0020	0.7450	0.0034
0.7080	0.0001	0.7850	0.0035
0.7080	0.0007	0.8130	0.0810
0.7080	0.0375	0.8500	0.2162
0.7080	0.0575	0.8660	0.5565
0.7080	0.0603	0.8940	0.4871
0.7070		0.6310	
0.7070	0.0013	0.8060	0.1059

0.7070	0.0028	0.7960	0.0528
0.7070	0.0037	0.8590	0.2252
0.7060	0.0148	0.8360	0.0678
0.7060		0.9350	
0.7050	0.0015	0.9210	0.4449
0.7040		0.7560	
0.7040	0.0091	0.7420	0.0051
0.7040	0.0014	0.7420	0.0064
0.7040	0.0010	0.7740	0.0368
0.7040	0.0054	0.8750	0.2042
0.7040	0.1002	0.8440	0.5715
0.7030	0.0028	0.8410	0.1758
0.7020	0.0263	0.8310	0.3386
0.7020	0.0030	0.8310	0.0147
0.7020	0.0260	0.8680	0.0109
0.7010		0.7130	
0.7010	0.0022	0.7500	0.0032
0.7010	0.0118	0.7450	0.0121
0.7010	0.0006	0.8060	0.2330
0.7010	0.0466	0.8090	0.1568
0.7010	0.0272	0.9460	0.7286
0.7000	0.0266	0.6990	0.0064
0.7000		0.9040	
0.7000	0.0020	0.9230	0.5650
0.6990		0.6700	
0.6990		0.9340	
0.6990		1.0370	
0.6990	0.1203	1.1950	0.3551
0.6980	0.0044	0.7530	0.0062
0.6980	0.0188	0.7640	0.0478
0.6980	0.0007	0.8070	0.0443
0.6980	0.0080	0.8310	0.1606
0.6980	0.0007	0.8610	0.1986

0.6980		1.0470	
0.6970	0.0016	0.7740	0.0186
0.6970	0.0004	0.7830	0.0267
0.6970	0.0039	0.9400	0.5791
0.6960	0.0716	0.6810	0.0170
0.6960		0.9410	
0.6960	0.1128	1.0720	0.8216
0.6950	0.0035	0.7410	0.0102
0.6950	0.0013	0.7970	0.0166
0.6950		0.9100	
0.6950	0.0181	0.8720	0.3256
0.6950		0.9670	
0.6940		0.7790	
0.6940	0.0088	0.8880	0.2144
0.6940		0.9300	
0.6940		0.9850	
0.6930	0.0043	0.7400	0.0099
0.6930	0.0002	0.7820	0.0357
0.6930	0.0015	0.7790	0.0137
0.6930	0.0197	0.7550	0.0267
0.6930	0.0090	0.7860	0.0803
0.6930	0.0017	0.8350	0.0850
0.6920	0.0200	0.8780	0.1498
0.6920		1.2240	
0.6910	0.1393	0.8410	0.5535
0.6910		0.9630	
0.6910		1.0010	
0.6900		0.6900	
0.6900	0.0062	0.7220	0.0144
0.6900		0.8100	
0.6900	0.0876	0.9180	0.7045
0.6900	0.0405	1.1410	0.3590
0.6900	0.1223	1.2760	0.0343

0.6890	0.0020	0.7620	0.0367
0.6890	0.0309	0.9520	0.8822
0.6890	0.0097	0.9650	0.9310
0.6890	0.2170	1.4480	0.0826
0.6870		0.7390	
0.6870	0.0055	0.7450	0.0430
0.6870	0.0036	0.7780	0.0638
0.6870	0.0066	0.7850	0.0392
0.6870	0.0029	0.7970	0.0280
0.6870		0.8890	
0.6870	0.0042	1.0370	0.6949
0.6870		1.4280	
0.6860	0.0073	0.7730	0.0874
0.6860	0.0026	0.8120	0.0777
0.6860		1.0760	
0.6850	0.0010	0.7750	0.0119
0.6850	0.0737	0.9420	0.8159
0.6850	0.2675	0.9850	0.9738
0.6850		1.3850	
0.6840	0.0043	0.7720	0.0808
0.6830		0.5180	
0.6830		0.7910	
0.6830	0.0037	0.7480	0.0649
0.6830	0.0014	0.7740	0.0591
0.6830	0.0107	0.8150	0.0397
0.6830		0.9190	
0.6820	0.0208	0.5490	0.1600
0.6820		0.7150	
0.6820	0.0075	0.8000	0.0422
0.6820	0.0015	0.8160	0.1156
0.6820	0.0901	0.8730	0.6332
0.6820		1.0020	
0.6820	0.0243	1.0840	0.4562

0.6810		0.8840	
0.6810	0.0026	0.8450	0.2194
0.6800	0.0471	0.7430	0.1250
0.6800	0.0037	0.7700	0.0232
0.6800	0.0423	0.8640	0.0355
0.6800	0.2326	0.8910	0.5903
0.6790	0.0041	0.7500	0.0123
0.6790	0.0015	0.7540	0.0132
0.6790	0.0083	0.8370	0.0630
0.6780		0.7210	
0.6770	0.0009	0.7970	0.0724
0.6760	0.0005	0.8090	0.0356
0.6750	0.0046	0.7340	0.0207
0.6750	0.0294	0.7480	0.0842
0.6750	0.0003	0.8540	0.0853
0.6740	0.0063	0.7050	0.0194
0.6740	0.2361	1.1820	0.6503
0.6740	0.1984	1.3250	0.4439
0.6720	0.0061	0.7670	0.0490
0.6710	0.0001	0.7000	0.0024
0.6700	0.0051	0.7330	0.0191
0.6700		0.8170	
0.6690	0.0071	0.7200	0.0022
0.6690	0.0306	0.7520	0.0891
0.6690	0.1381	0.9470	0.8733
0.6690	0.2907	1.4360	0.5686
0.6680	0.0011	0.8970	0.2548
0.6680	0.0035	0.9050	0.4242
0.6670		0.6830	
0.6670		0.7280	
0.6670	0.0002	0.7780	0.0363
0.6670	0.0495	0.8550	0.1472
0.6670	0.2855	1.2810	0.5733

0.6660	0.0387	0.7860	0.1066
0.6660	0.0040	0.8550	0.1774
0.6660		1.0370	
0.6650		0.7850	
0.6650	0.0074	0.8560	0.3579
0.6650	0.0216	0.8870	0.4907
0.6640	0.0014	0.7910	0.0043
0.6640	0.0102	0.8230	0.0721
0.6640	0.0840	0.8740	0.6847
0.6630	0.0010	0.7550	0.0061
0.6630		0.8440	
0.6630		0.9530	
0.6630	0.0491	1.2850	0.3537
0.6620		0.7060	
0.6620	0.0220	0.6780	0.0274
0.6620	0.0328	1.0650	0.7269
0.6610		0.6950	
0.6610		0.6930	
0.6610	0.0732	0.8470	0.3328
0.6610		1.1630	
0.6610	0.0179	1.1640	0.3266
0.6610	0.2563	1.3360	0.5236
0.6600	0.0157	0.6360	0.0111
0.6600	0.0006	0.8250	0.0930
0.6600		0.9970	
0.6590	0.2008	1.3460	0.1701
0.6580	0.0052	0.7690	0.0385
0.6580		1.1190	
0.6580	0.0390	1.2260	0.3427
0.6560	0.0074	0.8010	0.1096
0.6560	0.0388	0.7570	0.1332
0.6560	0.1562	1.0160	0.9628
0.6560	0.1425	1.1180	0.6362

0.6550	0.0039	0.7280	0.0107
0.6540		0.6430	
0.6540	0.0031	0.8240	0.0663
0.6540		1.0510	
0.6530		0.5920	
0.6510		0.7250	
0.6510	0.0226	1.0850	0.6527
0.6500	0.5196	0.5200	0.0739
0.6500	0.0049	0.7200	0.0048
0.6500	0.0008	0.8410	0.1505
0.6500		0.9050	
0.6500		0.9690	
0.6500	0.0539	1.3440	0.2017
0.6490	0.0393	0.6800	0.1511
0.6490	0.0148	1.1530	0.3968
0.6480	0.0150	0.5670	0.0218
0.6480		0.7090	
0.6480		0.7540	
0.6480	0.0317	0.9680	0.9869
0.6480	0.0002	1.0940	0.3997
0.6470		0.7270	
0.6460		0.8490	
0.6440		0.9640	
0.6430	0.0105	0.7110	0.0069
0.6430	0.0042	0.8450	0.3911
0.6430	0.0168	0.8640	0.3338
0.6430	0.0106	0.9100	0.5054
0.6430	0.1443	1.6960	0.1629
0.6420	0.0000	0.6760	0.0020
0.6420	0.0057	0.6860	0.0614
0.6420		1.0040	
0.6410		0.5580	
0.6410	0.0142	0.9900	0.9589

0.6410	0.2559	1.5280	0.3370
0.6400	0.0747	1.2410	0.2271
0.6390	0.0652	0.6870	0.2319
0.6390	0.0623	1.2350	0.1417
0.6370	0.0244	0.7850	0.1235
0.6360	0.0537	0.6500	0.1736
0.6360		0.7700	
0.6360		0.8780	
0.6360	0.0018	0.8570	0.4730
0.6350	0.0202	0.7680	0.1131
0.6330	0.0287	0.8810	0.4234
0.6320		0.8820	
0.6320		1.1980	
0.6300		0.3990	
0.6300	0.0035	0.7600	0.0272
0.6290		0.6250	
0.6250		0.6990	
0.6250	0.0156	0.7790	0.1155
0.6240	0.0243	0.8330	0.0862
0.6240	0.1121	0.9410	0.8327
0.6240	0.1153	1.0890	0.7167
0.6230	0.0098	0.9840	0.7933
0.6220	0.0062	0.6360	0.0394
0.6210		0.5670	
0.6210	0.0140	0.7280	0.1882
0.6210	0.0016	0.8490	0.5699
0.6210	0.0926	1.4250	0.3576
0.6200	0.0132	0.9700	0.9435
0.6150	0.0097	0.6640	0.0449
0.6150	0.0148	0.8360	0.1072
0.6150		2.8530	
0.6140	0.0020	0.6140	0.0059
0.6140	0.0013	0.9560	0.7265

0.6140		1.1130	
0.6140	0.0029	1.1400	0.5074
0.6140	0.0027	1.2300	0.2040
0.6130		0.9780	
0.6130	0.0231	1.1180	0.4951
0.6120	0.0897	1.5080	0.0266
0.6100	0.0203	0.6260	0.0023
0.6100	0.0092	0.8180	0.2612
0.6100	0.4482	4.0480	0.3947
0.6090		0.9480	
0.6070	0.0016	0.5710	0.0001
0.6060	0.0053	0.9450	0.7192
0.6050		0.6170	
0.6030	0.4217	0.6860	0.9048
0.6030	0.0103	0.8020	0.1460
0.6030	0.0245	1.1740	0.4008
0.6020	0.1883	0.7880	0.4969
0.6010	0.0132	0.6460	0.0002
0.6010	0.0019	0.8320	0.0407
0.6000	0.0037	0.6560	0.0010
0.6000		0.8830	
0.5990	0.0146	0.6910	0.0283
0.5980	0.0338	0.9870	0.9776
0.5970	0.0014	0.7160	0.0128
0.5940	0.0025	0.8970	0.5480
0.5920	0.0314	0.6850	0.0893
0.5920	0.0029	0.7910	0.3298
0.5920	0.1307	1.2830	0.3839
0.5910	0.0030	0.7270	0.0967
0.5910	0.0002	0.8910	0.4362
0.5910	0.0032	0.9670	0.9846
0.5900	0.0162	0.7290	0.0240
0.5900	0.2058	1.2430	0.3918

0.5870	0.0095	0.7930	0.1729
0.5860	0.0003	0.9390	0.5853
0.5850		0.6120	
0.5850	0.0195	0.6750	0.0025
0.5840		0.7830	
0.5830		0.8960	
0.5810		0.6590	
0.5810	0.0434	0.8500	0.3141
0.5800	0.0590	1.1000	0.7072
0.5790		0.6870	
0.5790	0.0022	0.8710	0.4419
0.5790	0.0348	1.2230	0.2136
0.5780		0.9350	
0.5770	0.0014	0.6920	0.0250
0.5760	0.0024	0.6690	0.0159
0.5730	0.2507	1.4160	0.3188
0.5710	0.2235	0.7760	0.2693
0.5710	0.0018	0.9040	0.4908
0.5700	0.0362	1.2040	0.4402
0.5700	0.0713	1.4220	0.1897
0.5640	0.0000	1.0000	0.8816
0.5610	0.0029	0.9180	0.4265
0.5610	0.0198	1.0380	0.7447
0.5570	0.0017	0.7480	0.0377
0.5560	0.1026	0.7420	0.2782
0.5540	0.0249	0.9900	0.9844
0.5530	0.0020	0.8890	0.5259
0.5520	0.0005	0.9890	0.9143
0.5490		0.7410	
0.5470	0.0012	0.8830	0.5058
0.5460	0.0314	1.3680	0.1554
0.5450	0.0085	0.5900	0.0257
0.5450	0.0540	1.2920	0.3540

0.5420	0.1163	1.1710	0.6618
0.5400	0.0177	0.8230	0.4157
0.5400		1.2070	
0.5390		0.9130	
0.5370		1.0980	
0.5370	0.0050	1.3180	0.2840
0.5360		1.0150	
0.5340	0.0013	0.8890	0.5332
0.5330	0.0331	0.7710	0.3358
0.5330	0.0019	1.0950	0.4175
0.5330	0.1008	1.1400	0.6823
0.5320		0.7730	
0.5300	0.0018	0.9450	0.5795
0.5280		0.5350	
0.5240		1.0560	
0.5240	0.2117	0.9000	0.8735
0.5240	0.0112	1.1790	0.2538
0.5230		1.1950	
0.5210		0.8950	
0.5210	0.1862	1.2290	0.6747
0.5210	0.0373	1.3670	0.1674
0.5190	0.0024	0.8940	0.2941
0.5160	0.0047	0.7890	0.3253
0.5160		1.4160	
0.5150		0.7370	
0.5120		1.0910	
0.5070	0.0031	1.0670	0.5978
0.5060	0.1589	0.8830	0.9088
0.5040		0.8650	
0.5040	0.1703	2.0080	0.2729
0.5020		1.0370	
0.5020	0.0049	1.0830	0.2638
0.5010		0.5320	

0.4970	0.0009	0.8950	0.6286
0.4950	0.1534	1.1080	0.8816
0.4930		0.8930	
0.4930	0.0264	1.4050	0.2819
0.4920	0.0055	0.8130	0.1982
0.4900		0.3300	
0.4830		0.6220	
0.4830	0.0023	0.6710	0.0390
0.4770		0.6330	
0.4750	0.1225	0.4890	0.0227
0.4690		0.6870	
0.4660	0.0473	0.7470	0.4474
0.4650		0.4600	
0.4650	0.0010	0.6560	0.1500
0.4640	0.0041	0.5950	0.0003
0.4590		0.9530	
0.4580	0.0227	0.5220	0.1987
0.4580		1.0040	
0.4540	0.0016	0.9160	0.6632
0.4500	0.0035	1.0910	0.7332
0.4500	0.0929	1.1990	0.6877
0.4420	0.2121	1.4400	0.6068
0.4380	0.5315	1.0230	0.9166
0.4360	0.0083	1.1680	0.3735
0.4170		1.0750	
0.4160		0.5950	
0.4140	0.3666	0.5780	0.3600
0.4140	0.1050	1.3000	0.1909
0.4060		0.6990	
0.3950	0.1683	2.1720	0.3361
0.3930	0.0688	0.7620	0.5303
0.3880	0.1029	1.2450	0.6889
0.3850		1.2840	

0.3830	0.4056	0.4940	0.1774
0.3810		0.3150	
0.3790		1.3920	
0.3660		0.7800	
0.3580	0.0416	1.2890	0.1730
0.3540		1.0810	
0.3540	0.0236	1.2010	0.3357
0.3160		0.4180	
0.3110	0.0031	0.7680	0.2791
0.3030	0.0175	1.1100	0.4602
0.3020	0.0218	1.1260	0.4172
0.2960		1.0510	
0.2960	0.1022	1.3850	0.6349
0.2510	0.0299	1.1540	0.6461
0.2230		0.9550	
0.2220	0.4642	0.2680	0.4372

Index	Compounds	Class I	Class II	Q1 (Da)
MEDP1796	Perillyl alcohol	Alcohol and amines	Alcohols	153.1259609
MADN0577	D-inositol 4-phospha	Alcohol and amines	Alcohols	259.0213
MEDP1194	Calcitriol	Alcohol and amines	Alcohols	417.3
MEDP2027	D-Mannosamine	Alcohol and amines	Amines	180.08759
MADP0490	1-amino-3,3-diethoxy	Alcohol and amines	Amines	148.1327042
MADN0511	(S)-2-Amino-3-(7-chl	Alcohol and amines	Amines	237.0417968
MEDP1067	Palmitoyl N-isopropy	Alcohol and amines	Amines	320.29827
MEDP2143	Diethanolamine lauri	Alcohol and amines	Amines	288.2541005
MEDP1040	N-(2-hydroxyethyl)st	Alcohol and amines	Amines	328.3189
MADP0051	Diethanolamine	Alcohol and amines	Amines	106.0863
MADP0283	N-Acetylhistamine	Alcohol and amines	Amines	154.0969
MADP0557	N1-Acetylspermidine	Alcohol and amines	Amines	188.1744
MADP0583	Trimethylamine N-O	Alcohol and amines	Amines	76.0761494
MADP0347	Agmatine	Alcohol and amines	Polyamines	131.1284
MEDP2038	3-O-Methyldopa	Alcohol and amines	Polyamines	212.0939723
MADP0156	Oxidopamine	Alcohol and amines	Polyamines	170.0812
MEDP1212	1,3-Dicyclohexylurea	Alcohol and amines	Polyamines	225.195717
MEDP2066	Triethylenetetramine	Alcohol and amines	Polyamines	147.1603596
MEDP0601	Spermidine	Alcohol and amines	Polyamines	146.1578976
MEDP1196	Biotinamide	Alcohol and amines	Polyamines	244.1082639
MEDP1197	Hypotaurocyamine	Alcohol and amines	Polyamines	152
MADP0571	Diethyl phosphate	Aldehyde,Ketones,E	Esters	155.0461533
MEDN1064	N-arachidene glycine	Aldehyde,Ketones,E	Esters	360.253894
MADN0224	N-Acetyl-DL-serine	Amino acid and Its r	Amino acid and Its r	146.0448
MADP0203	L- -Glutamyl-L-gluta	Amino acid and Its r	Amino acid and Its r	277.1031
MADP0185	N-(Phosphonomethyl	Amino acid and Its r	Amino acid and Its r	230.043
MADP0195	Securinine	Amino acid and Its r	Amino acid and Its r	240.1006
MEDP0886	N-Amidino-L-Aspart	Amino acid and Its r	Amino acid derivati	176.4

MADP0544	(+/-)-High Proline	Amino acid and Its r	Amino acid derivativ	130.0866
MEDP1942	N-Methy-4-Hydroxy-	Amino acid and Its r	Amino acid derivativ	146.0812
MEDN0061	N-Phenylacetylglycin	Amino acid and Its r	Amino acid derivativ	192.0660932
MEDN0720	N-(2-Methylbenzoyl)	Amino acid and Its r	Amino acid derivativ	192.0660932
MADP0091	5-Aminovaleric Acid	Amino acid and Its r	Amino acid derivativ	118.0863
MADP0098	N-γ-Acetyl-N-2-Forn	Amino acid and Its r	Amino acid derivativ	265.1183
MEDN1684	S-Ribosyl-L-homocy:	Amino acid and Its r	Amino acid derivativ	266.0901
MEDN1861	N-Myristoylglycine	Amino acid and Its r	Amino acid derivativ	284.2222085
MADN0316	N-Alpha-Acetyl-L-A:	Amino acid and Its r	Amino acid derivativ	173.0563
MEDN1518	Acetylvaline	Amino acid and Its r	Amino acid derivativ	158.0841269
MADP0138	N-Acetyl-L-Leucine	Amino acid and Its r	Amino acid derivativ	174.1125
MEDP1916	N-Acetyl-L-phenylal:	Amino acid and Its r	Amino acid derivativ	208.0951
MEDP0637	Phe-Pro	Amino acid and Its r	Amino acid derivativ	263.131743
MEDP1968	L-Lysine-Butanoic A	Amino acid and Its r	Amino acid derivativ	235.1
MADN0171	N-Acetyl-L-Glutamic	Amino acid and Its r	Amino acid derivativ	188.0564
MEDP2303	6-Diazo-5-oxo-L-nor	Amino acid and Its r	Amino acid derivativ	172.0714489
MEDP0876	O-Succinyl-L-Homos	Amino acid and Its r	Amino acid derivativ	220.2
MEDP2131	L-Isserine	Amino acid and Its r	Amino acid derivativ	106.0496782
MADP0031	N6-Acetyl-L-Lysine	Amino acid and Its r	Amino acid derivativ	189.1234
MEDP0611	N-Acetyl-L-Histidine	Amino acid and Its r	Amino acid derivativ	198.0800412
MEDP2302	N-Acetyl-DL-methio	Amino acid and Its r	Amino acid derivativ	192.0695412
MEDP2084	L-Homophenylalanin	Amino acid and Its r	Amino acid derivativ	180.1026729
MADP0269	N-Methylalanine	Amino acid and Its r	Amino acid derivativ	104.07
MADP0485	N-Ethylglycine	Amino acid and Its r	Amino acid derivativ	104.0701594
MADN0015	N-Acetyl-L-Tyrosine	Amino acid and Its r	Amino acid derivativ	222.0772
MEDP1177	N-Methyl-α-aminoisc	Amino acid and Its r	Amino acid derivativ	235.2
MADN0289	N-Acetyl-beta-alanin	Amino acid and Its r	Amino acid derivativ	130.0509
MADN0336	N-Acetyl-L-alanine	Amino acid and Its r	Amino acid derivativ	130.0507
MADN0118	N-Acetylglycine	Amino acid and Its r	Amino acid derivativ	116.0353
MEDP1914	N-(1-Deoxy-1-fructo:	Amino acid and Its r	Amino acid derivativ	358
MADP0573	Nα-Acetyl-L-Arginin	Amino acid and Its r	Amino acid derivativ	217.1304739
MEDP1126	(R)-(-)-2-phenylglyci	Amino acid and Its r	Amino acid derivativ	120.0726939
MADP0531	N-acetyl-D-phenylala	Amino acid and Its r	Amino acid derivativ	206.0826153

MADP0024	L-Carnosine	Amino acid and Its r	Amino acid derivativ	227.1139
MEDP2132	1-amino-1-cyclobutai	Amino acid and Its r	Amino acid derivativ	116.0707284
MADP0331	L-Methionine sulfoxi	Amino acid and Its r	Amino acid derivativ	166.053
MADP0553	Gly-Tyr	Amino acid and Its r	Amino acid derivativ	239.1033
MEDN1686	3-Methylaspartate	Amino acid and Its r	Amino acid derivativ	146.0465
MADP0092	6-Aminocaproic Acic	Amino acid and Its r	Amino acid derivativ	132.1019
MADP0150	N-Acetylthreonine	Amino acid and Its r	Amino acid derivativ	162.0761
MADP0167	N,N-Dimethylarginin	Amino acid and Its r	Amino acid derivativ	203.1497
MEDN1832	Docosahexaenoic Ac	Amino acid and Its r	Amino acid derivativ	384.2549
MEDN1246	3-(pyrazol-1-yl) -L-al	Amino acid and Its r	Amino acid derivativ	154.0620148
MADP0032	N-Acetylaspartate	Amino acid and Its r	Amino acid derivativ	176.0554
MADN0583	N-glutarylglycine	Amino acid and Its r	Amino acid derivativ	188.0563
MADP0547	O-Acetyl-L-homoseri	Amino acid and Its r	Amino acid derivativ	162.076
MEDN1890	L-Histidinol	Amino acid and Its r	Amino acid derivativ	140.0825
MADP0258	Xanthurenic Acid	Amino acid and Its r	Amino acid derivativ	206.0434
MADN0074	2-Aminoisobutyric A	Amino acid and Its r	Amino acid derivativ	102.056
MADP0104	γ-Aminobutyric Acid	Amino acid and Its r	Amino acid derivativ	104.0706
MADP0569	L-Homoarginine	Amino acid and Its r	Amino acid derivativ	189.1342093
MADN0159	N-Acetylornithine	Amino acid and Its r	Amino acid derivativ	173.0937
MEDP1973	D-Proline betaine	Amino acid and Its r	Amino acid derivativ	144.1016
MADP0360	7-Methyluric Acid	Amino acid and Its r	Amino acid derivativ	183.0508
MADP0234	Cis-4-Hydroxy-D-Prc	Amino acid and Its r	Amino acid derivativ	132.0643
MEDP1662	N6,N6,N6-Trimethyl-	Amino acid and Its r	Amino acid derivativ	189.15915
MEDP0587	N-Alpha-acetyl-L-lys	Amino acid and Its r	Amino acid derivativ	189.1160924
MEDP1970	L-Methionine methyl	Amino acid and Its r	Amino acid derivativ	164.07398
MEDP1933	Cyclopentylglycine	Amino acid and Its r	Amino acid derivativ	144.098
MEDP1974	1-Methylpiperidine-2	Amino acid and Its r	Amino acid derivativ	144.098
MADN0542	N-Acetyltyramine	Amino acid and Its r	Amino acid derivativ	178.0863382
MADN0007	3-Hydroxy-3-Methyl	Amino acid and Its r	Amino acid derivativ	161.0455
MEDN1480	N-Arachidonoyl-L-A	Amino acid and Its r	Amino acid derivativ	374.26665
MADP0359	1-Methyluric Acid	Amino acid and Its r	Amino acid derivativ	183.0508
MADP0337	N-Methyl-4-aminobu	Amino acid and Its r	Amino acid derivativ	118.0865
MADN0016	N-Acetylneuraminic .	Amino acid and Its r	Amino acid derivativ	308.0987

MEDP0087	L-Alanyl-L-Lysine	Amino acid and Its r	Amino acid derivativ	218.1
MADP0036	Gly-Leu	Amino acid and Its r	Amino acid derivativ	189.1234
MADP0130	Nα-Acetyl-L-glutami	Amino acid and Its r	Amino acid derivativ	189.087
MADN0549	N-acetyl-DL-tryptoph	Amino acid and Its r	Amino acid derivativ	245.0934594
MEDP1307	(2S,3S)-3-methylphe	Amino acid and Its r	Amino acid derivativ	180.1008879
MEDP1963	S-(Methyl)glutathion	Amino acid and Its r	Amino acid derivativ	322.10673
MADP0043	Trans-4-Hydroxy-L-P	Amino acid and Its r	Amino acid derivativ	132.0655
MADP0546	Cis-L-3-hydroxyproli	Amino acid and Its r	Amino acid derivativ	132.0657
MEDP0664	L-Tyrosine methyl es	Amino acid and Its r	Amino acid derivativ	196.2
MADN0592	Cysteine sulfinic acid	Amino acid and Its r	Amino acid derivativ	152.0017
MADP0315	2-Phenylglycine	Amino acid and Its r	Amino acid derivativ	152.07
MADP0039	S-(5-Adenosy)-L-Ho	Amino acid and Its r	Amino acid derivativ	385.1289
MADP0030	N,N-Dimethylglycine	Amino acid and Its r	Amino acid derivativ	104.0706
MADN0092	Homogentisic Acid	Amino acid and Its r	Amino acid derivativ	167.035
MADP0018	3-N-Methyl-L-Histid	Amino acid and Its r	Amino acid derivativ	170.0924
MEDP0891	L-Tryptophanamide	Amino acid and Its r	Amino acid derivativ	204.3
MADN0596	Serine alanine	Amino acid and Its r	Amino acid derivativ	175.0725
MADP0096	Kynurenic Acid	Amino acid and Its r	Amino acid derivativ	190.04
MADP0095	Kynurenine	Amino acid and Its r	Amino acid derivativ	209.1
MADP0028	L-Theanine	Amino acid and Its r	Amino acids	175.1077
MEDN0049	L-Saccharopine	Amino acid and Its r	Amino acids	275.1243364
MADP0523	N(6),N(6)-Dimethyl-	Amino acid and Its r	Amino acids	175.1432049
MADN0009	L-Pyroglutamic Acid	Amino acid and Its r	Amino acids	128.0353
MADP0177	N-Formylglycine	Amino acid and Its r	Amino acids	104.0342
MADN0021	Sarcosine	Amino acid and Its r	Amino acids	88.0404
MADN0001	Glycine	Amino acid and Its r	Amino acids	74.0247
MADN0011	β-Alanine	Amino acid and Its r	Amino acids	88.0404
MADP0119	Methylcysteine	Amino acid and Its r	Amino acids	136.2
MADP0005	L-Alanine	Amino acid and Its r	Amino acids	90.055
MADP0012	L-Proline	Amino acid and Its r	Amino acids	116.0706
MADP0011	L-Phenylalanine	Amino acid and Its r	Amino acids	166.0863
MADP0175	L-Norvaline	Amino acid and Its r	Amino acids	118.0863
MADP0218	L-Leucine	Amino acid and Its r	Amino acids	132.1009

MADP0014	L-Valine	Amino acid and Its r	Amino acids	118.0863
MADP0003	L-Lysine	Amino acid and Its r	Amino acids	147.1128
MADN0004	L-Isoleucine	Amino acid and Its r	Amino acids	130.0873
MADP0235	L-Norleucine	Amino acid and Its r	Amino acids	132.1009
MADP0013	L-Serine	Amino acid and Its r	Amino acids	106.0499
MADP0006	L-Aspartic Acid	Amino acid and Its r	Amino acids	134.0448
MADP0025	L-Glutamine	Amino acid and Its r	Amino acids	147.0764
MADN0187	3-Aminoisobutanoic .	Amino acid and Its r	Amino acids	102.056
MADN0006	L-Tryptophan	Amino acid and Its r	Amino acids	203.0826
MADP0008	L-Histidine	Amino acid and Its r	Amino acids	156.0768
MADP0010	L-Methionine	Amino acid and Its r	Amino acids	150.0583
MADP0330	L-Allothreonine	Amino acid and Its r	Amino acids	120.0656
MADN0003	L-Glutamic Acid	Amino acid and Its r	Amino acids	146.0459
MADP0135	L-Threonine	Amino acid and Its r	Amino acids	120.0655
MADP0023	L-Asparagine Anhydı	Amino acid and Its r	Amino acids	133.0608
MADP0007	L-Citrulline	Amino acid and Its r	Amino acids	176.103
MADP0259	L-Tyrosine	Amino acid and Its r	Amino acids	182.0801
MADN0208	DL-2-Methylglutami	Amino acid and Its r	Amino acids	160.0615
MEDP1509	Glu-Met	Amino acid and Its r	Small Peptide	279
MEDP1511	Met-Glu	Amino acid and Its r	Small Peptide	279
MADP0403	Pro-Met	Amino acid and Its r	Small Peptide	247.1116
MEDP1913	N(Alpha)-Acetyl-Eps	Amino acid and Its r	Small Peptide	243.13
MADN0437	His-Ala	Amino acid and Its r	Small Peptide	225.0994
MADN0013	Glyc-Pro	Amino acid and Its r	Small Peptide	171.0775
MEDP1863	Gly-His	Amino acid and Its r	Small Peptide	195.0845
MADN0636	γ-Glu-Gln	Amino acid and Its r	Small Peptide	274.1041
MADP0616	Tyr-Cys	Amino acid and Its r	Small Peptide	285.090377
MEDP1936	L-Aspartic acid-O-di	Amino acid and Its r	Small Peptide	458.1
MADP0602	Ser-Pro	Amino acid and Its r	Small Peptide	203.1022421
MADN0655	Asn-Pro	Amino acid and Its r	Small Peptide	228.0983
MADN0466	cyclo(glu-glu)	Amino acid and Its r	Small Peptide	257.0771
MEDN1207	Cysteinyldopa	Amino acid and Its r	Small Peptide	315.0651072
MADP0592	Met-Hyp	Amino acid and Its r	Small Peptide	263.1061

MADP0377	Glu-Val	Amino acid and Its r	Small Peptide	247.1286
MEDN0561	N-Acetyl-Asp-Glu	Amino acid and Its r	Small Peptide	303.0828655
MADP0420	Cyclo(Ala-Pro)	Amino acid and Its r	Small Peptide	169.0963
MADP0621	Arg-Glu	Amino acid and Its r	Small Peptide	304.1611556
MADP0209	Glu-Gln	Amino acid and Its r	Small Peptide	276.1183
MADN0439	Ala-Glu	Amino acid and Its r	Small Peptide	217.081
MADP0418	Pro-Glu-Val	Amino acid and Its r	Small Peptide	344.1826
MADN0464	Met-Val	Amino acid and Its r	Small Peptide	247.1118
MEDP1679	Glu-Val-Phe	Amino acid and Its r	Small Peptide	394.19665
MEDP1367	Ala-Tyr	Amino acid and Its r	Small Peptide	253.118
MADN0635	Met-Asp	Amino acid and Its r	Small Peptide	263.0703
MEDP1683	Leu-Leu-Leu	Amino acid and Its r	Small Peptide	358.26927
MEDP1935	Ile-Asp	Amino acid and Its r	Small Peptide	247.1288
MADN0598	Leu-Gly	Amino acid and Its r	Small Peptide	187.109
MADP0618	Gly-Ala-Pro-Met-Phe	Amino acid and Its r	Small Peptide	621.3252541
MEDP1851	Asp-Tyr	Amino acid and Its r	Small Peptide	297.1082
MEDP1859	Gln-Phe	Amino acid and Its r	Small Peptide	276.1299
MADP0385	cyclo(pro-pro)	Amino acid and Its r	Small Peptide	195.1134
MADP0565	Glu-Phe-Ala	Amino acid and Its r	Small Peptide	366.1658
MADP0558	Leu-Asn	Amino acid and Its r	Small Peptide	246.1444
MADN0597	Aspartyl valine	Amino acid and Its r	Small Peptide	231.0983
MADN0651	Asn-Gly	Amino acid and Its r	Small Peptide	188.0678
MADN0443	Asp-Gly	Amino acid and Its r	Small Peptide	189.0504
MADN0418	Cysteine glutathione (	Amino acid and Its r	Small Peptide	425.078
MEDP1924	Cyclo(Pro-Glu)	Amino acid and Its r	Small Peptide	227.09
MADP0627	Tyr-Pro	Amino acid and Its r	Small Peptide	279.1328063
MADP0312	Pro-Gly	Amino acid and Its r	Small Peptide	173.0918
MADN0515	Asp-Leu	Amino acid and Its r	Small Peptide	245.1127649
MEDP1860	Glu-Leu	Amino acid and Its r	Small Peptide	261.1434
MADP0399	Pro-Ser	Amino acid and Its r	Small Peptide	203.1021
MADN0435	Phe-Ala-Gly	Amino acid and Its r	Small Peptide	292.1295
MEDP1884	Pro-His	Amino acid and Its r	Small Peptide	235.1168
MADN0438	cyclo(pro-tyr)	Amino acid and Its r	Small Peptide	259.1088

MADP0229	Anserine	Amino acid and Its r Small Peptide	241.1296
MADN0467	cyclo(gly-glu)	Amino acid and Its r Small Peptide	185.0561
MEDP1676	Phe-Val-Asp	Amino acid and Its r Small Peptide	380.18039
MEDP1672	Leu-Leu-Glu	Amino acid and Its r Small Peptide	374.22752
MADN0650	Asn-Asp	Amino acid and Its r Small Peptide	246.0734
MADP0581	Pro-Lys	Amino acid and Its r Small Peptide	244.1668
MADP0404	Pro-Ala	Amino acid and Its r Small Peptide	187.108
MEDP1896	Val-Pro-Ala	Amino acid and Its r Small Peptide	286.1759
MADN0638	Asn-Glu	Amino acid and Its r Small Peptide	260.0886
MADN0453	Val-Val	Amino acid and Its r Small Peptide	215.1401
MADP0414	Gly-Thr	Amino acid and Its r Small Peptide	177.0866
MADP0622	Arg-Asn	Amino acid and Its r Small Peptide	289.1633138
MEDP1894	Tyr-Leu	Amino acid and Its r Small Peptide	295.1606
MADN0654	Asn-Lys	Amino acid and Its r Small Peptide	259.1405
MADP0395	Asp-Ile	Amino acid and Its r Small Peptide	247.129
MADP0600	Arg-Phe	Amino acid and Its r Small Peptide	322.1884
MEDP1678	Thr-Val-Leu	Amino acid and Its r Small Peptide	314.2075
MEDP1893	Thr-Val-Ile	Amino acid and Its r Small Peptide	314.2075
MEDP1666	Leu-Val-Ser	Amino acid and Its r Small Peptide	318.20155
MEDP1861	Glu-Tyr	Amino acid and Its r Small Peptide	311.1233
MEDP1673	Ile-Ile-Thr	Amino acid and Its r Small Peptide	346.232
MADP0425	Pro-Glu	Amino acid and Its r Small Peptide	245.1143
MADN0456	Ile-Phe	Amino acid and Its r Small Peptide	277.1556
MADP0632	Arg-Ser	Amino acid and Its r Small Peptide	262.1499807
MADP0556	Met-Pro	Amino acid and Its r Small Peptide	247.1121
MADP0382	Pro-Leu	Amino acid and Its r Small Peptide	229.1552
MADP0408	Glu-Cit	Amino acid and Its r Small Peptide	305.1444
MADP0308	Gly-Gly	Amino acid and Its r Small Peptide	133.0604
MADP0402	Trp-Val	Amino acid and Its r Small Peptide	304.166
MADN0639	Trp-Ala	Amino acid and Its r Small Peptide	274.1197
MADP0376	Arg-Leu	Amino acid and Its r Small Peptide	288.2047
MADN0645	Asn-Ala	Amino acid and Its r Small Peptide	202.0828
MADP0398	Lys-Ile	Amino acid and Its r Small Peptide	260.1795

MEDP1867	Gly-Pro-Leu	Amino acid and Its r	Small Peptide	286.1757
MEDP1675	Leu-Leu-Gly	Amino acid and Its r	Small Peptide	302.20653
MADN0634	Asn-Val	Amino acid and Its r	Small Peptide	230.1137
MADN0450	Arg-Val	Amino acid and Its r	Small Peptide	272.1718
MADN0434	Trp-Gly	Amino acid and Its r	Small Peptide	260.0997
MADP0633	Arg-Thr	Amino acid and Its r	Small Peptide	276.1671729
MADP0634	Leu-Glu	Amino acid and Its r	Small Peptide	261.146
MADP0590	Gly-Lys	Amino acid and Its r	Small Peptide	204.1327
MADP0631	Arg-Met	Amino acid and Its r	Small Peptide	306.1590245
MADP0640	Asn-Ser	Amino acid and Its r	Small Peptide	220.092
MADP0322	Val-Tyr	Amino acid and Its r	Small Peptide	281.1502
MEDP1674	Val-Val-Val	Amino acid and Its r	Small Peptide	316.22197
MADP0401	Trp-Ile	Amino acid and Its r	Small Peptide	318.1806
MADP0607	Trp-Ser	Amino acid and Its r	Small Peptide	292.1310111
MEDP1870	Ile-Lys	Amino acid and Its r	Small Peptide	260.195
MADP0374	Ile-Met	Amino acid and Its r	Small Peptide	263.1438
MADP0396	Ile-Glu	Amino acid and Its r	Small Peptide	261.1457
MEDP1022	Thr-Phe	Amino acid and Its r	Small Peptide	267.1243321
MEDP1023	Trp-Glu	Amino acid and Its r	Small Peptide	334.1303767
MADP0625	Arg-Ile	Amino acid and Its r	Small Peptide	288.203448
MADP0388	Glu-Ile	Amino acid and Its r	Small Peptide	261.1436729
MADP0612	Arg-Ala	Amino acid and Its r	Small Peptide	246.1568849
MADN0652	Hyp-Ile	Amino acid and Its r	Small Peptide	243.1351
MADP0022	Glutathione Oxidized	Amino acid and Its r	Small Peptide	613.1592
MADP0432	Glu-Gly	Amino acid and Its r	Small Peptide	205.0822474
MADP0428	Val-Glu	Amino acid and Its r	Small Peptide	247.1294
MADP0601	Phe-Tyr	Amino acid and Its r	Small Peptide	329.1492912
MADN0447	Val-Met	Amino acid and Its r	Small Peptide	247.1118
MEDP1507	Phe-Asn	Amino acid and Its r	Small Peptide	280
MEDP1681	Leu-Gly-Leu	Amino acid and Its r	Small Peptide	302.20743
MADN0595	Tyr-Val	Amino acid and Its r	Small Peptide	279.1362
MADP0427	Lys-Leu	Amino acid and Its r	Small Peptide	260.1973
MADP0383	Leu-Leu	Amino acid and Its r	Small Peptide	245.1867

MADP0608	Trp-Thr	Amino acid and Its r	Small Peptide	306.1459018
MADP0393	Ile-Ile	Amino acid and Its r	Small Peptide	245.1865
MADP0430	Glu-His	Amino acid and Its r	Small Peptide	285.1194
MADP0392	Ile-Leu	Amino acid and Its r	Small Peptide	245.186
MADP0373	Leu-Ile	Amino acid and Its r	Small Peptide	245.1867
MADP0567	Proline Hydroxyproli	Amino acid and Its r	Small Peptide	229.1182
MADP0378	Pro-Val	Amino acid and Its r	Small Peptide	215.1394
MADN0200	Ala-Gln	Amino acid and Its r	Small Peptide	216.099
MADN0012	Ala-Ala	Amino acid and Its r	Small Peptide	159.0775
MADP0560	Ala-Arg	Amino acid and Its r	Small Peptide	246.1546
MEDP1512	Phe-Val	Amino acid and Its r	Small Peptide	265
MADP0610	Thr-Arg	Amino acid and Its r	Small Peptide	276.1671554
MADP0332	Ala-Phe	Amino acid and Its r	Small Peptide	237.1243
MEDP1911	Phe-Trp	Amino acid and Its r	Small Peptide	352.16505
MADN0441	Val-Phe-Ala	Amino acid and Its r	Small Peptide	334.177
MADP0339	Leu-Phe	Amino acid and Its r	Small Peptide	279.1712
MEDP1670	Gly-Gly-Phe	Amino acid and Its r	Small Peptide	280.12872
MEDP1926	Cyclo(Pro-Phe)	Amino acid and Its r	Small Peptide	245.12
MADN0454	Ala-Ile	Amino acid and Its r	Small Peptide	201.1225
MADP0636	Asn-Asn	Amino acid and Its r	Small Peptide	247.1035
MADN0440	Val-Gly	Amino acid and Its r	Small Peptide	173.0912
MADP0423	Leu-Trp	Amino acid and Its r	Small Peptide	318.1819
MADP0629	Tyr-Thr	Amino acid and Its r	Small Peptide	283.1298055
MADP0623	Thr-His	Amino acid and Its r	Small Peptide	257.1243402
MADP0384	Val-Leu	Amino acid and Its r	Small Peptide	231.1723
MADP0429	Val-Ile	Amino acid and Its r	Small Peptide	231.1706
MADN0262	Gly-Val	Amino acid and Its r	Small Peptide	173.0928
MADP0570	Met-Gln	Amino acid and Its r	Small Peptide	278.1174
MEDP1682	Leu-Gly-Phe	Amino acid and Its r	Small Peptide	336.19221
MADP0389	Ile-Trp	Amino acid and Its r	Small Peptide	318.1811
MADN0465	Thr-Val	Amino acid and Its r	Small Peptide	217.1181
MADP0409	Ser-Phe-Ala	Amino acid and Its r	Small Peptide	324.1563
MADN0461	Ile-Ala	Amino acid and Its r	Small Peptide	201.1236

MADN0451	Ile-Tyr	Amino acid and Its r	Small Peptide	293.1497
MADP0397	Leu-Thr	Amino acid and Its r	Small Peptide	233.15
MADP0400	Ser-Val	Amino acid and Its r	Small Peptide	205.1185
MEDP1667	Phe-Glu	Amino acid and Its r	Small Peptide	295.12701
MEDP1866	Gly-Phe-Phe	Amino acid and Its r	Small Peptide	370.173
MADN0457	Ile-Val	Amino acid and Its r	Small Peptide	229.155
MADP0536	gamma-D-GLU-GLY	Amino acid and Its r	Small Peptide	205.0817537
MADN0446	Leu-Tyr	Amino acid and Its r	Small Peptide	293.1494
MADP0410	Lys-Val	Amino acid and Its r	Small Peptide	246.1823
MEDP1508	Phe-Met	Amino acid and Its r	Small Peptide	297
MADN0460	His-Ile	Amino acid and Its r	Small Peptide	267.1433
MADP0162	Leu-Ala	Amino acid and Its r	Small Peptide	203.139
MADP0406	Ile-Thr	Amino acid and Its r	Small Peptide	233.15
MEDP1680	Thr-Leu-Ile	Amino acid and Its r	Small Peptide	346.23344
MADP0584	Phe-Gly	Amino acid and Its r	Small Peptide	223.1083
MADP0390	Ile-Arg	Amino acid and Its r	Small Peptide	288.204
MADN0463	Val-Asp	Amino acid and Its r	Small Peptide	231.0979
MADN0631	Gln-Gln	Amino acid and Its r	Small Peptide	273.1195
MADP0548	Asp-Arg	Amino acid and Its r	Small Peptide	290.1464
MADN0315	Leu-Val	Amino acid and Its r	Small Peptide	229.1557
MADP0577	Phe-Lys	Amino acid and Its r	Small Peptide	294.1825
MADP0579	Lys-Ser	Amino acid and Its r	Small Peptide	234.1446
MEDP1889	Ser-Phe	Amino acid and Its r	Small Peptide	253.1177
MADP0343	Gly-Ile	Amino acid and Its r	Small Peptide	189.1227
MADP0594	Pro-Thr	Amino acid and Its r	Small Peptide	217.1172
MADP0588	Lys-Met	Amino acid and Its r	Small Peptide	278.1503
MADP0509	Gly-Gln	Amino acid and Its r	Small Peptide	204.0972635
MADN0445	Val-Lys	Amino acid and Its r	Small Peptide	244.166
MADP0407	Thr-Ile	Amino acid and Its r	Small Peptide	233.1491
MADP0611	Thr-Asp	Amino acid and Its r	Small Peptide	235.092402
MADP0624	Arg-Lys	Amino acid and Its r	Small Peptide	303.2137634
MADN0641	Val-Gln	Amino acid and Its r	Small Peptide	244.1302
MADN0452	Ile-Gly	Amino acid and Its r	Small Peptide	187.1075

MADP0596	Ser-Arg	Amino acid and Its r	Small Peptide	262.151
MADP0391	Pyr-Glu	Amino acid and Its r	Small Peptide	259.0916
MADP0620	Arg-Arg	Amino acid and Its r	Small Peptide	331.216619
MADN0642	Val-Asn	Amino acid and Its r	Small Peptide	230.1147
MADP0394	Ile-Ser	Amino acid and Its r	Small Peptide	219.133
MADP0614	Tyr-Asn	Amino acid and Its r	Small Peptide	296.1238677
MADP0426	Val-Arg	Amino acid and Its r	Small Peptide	274.1883
MADP0379	Thr-Leu	Amino acid and Its r	Small Peptide	233.1502
MEDP1888	Ser-Leu	Amino acid and Its r	Small Peptide	219.1333
MADP0635	Thr-Ser	Amino acid and Its r	Small Peptide	207.0964
MADP0405	Glu-Ser	Amino acid and Its r	Small Peptide	235.0919054
MADP0603	Ser-Ser	Amino acid and Its r	Small Peptide	193.0821074
MADP0574	Ile-Gln	Amino acid and Its r	Small Peptide	260.1615
MADP0586	Ser-Gln	Amino acid and Its r	Small Peptide	234.1084
MADP0613	Thr-Glu	Amino acid and Its r	Small Peptide	249.1082472
MADP0554	Asp-Ala	Amino acid and Its r	Small Peptide	205.0806
MADP0220	Glutathione Reduced	Amino acid and Its r	Small Peptide	308.0925
MADN0580	AG-183	Benzene and substitu	Benzene and substitu	267.0518
MEDP2274	BW-245C	Benzene and substitu	Benzene and substitu	369.2397
MEDN1885	3,5-Dimethoxypheno	Benzene and substitu	Benzene and substitu	153.0556236
MEDP2286	Tripterin	Benzene and substitu	Benzene and substitu	451.2838705
MEDP2137	5-Phenyl-1-pentanol	Benzene and substitu	Benzene and substitu	165.1272013
MEDN0541	DL-3,4-Dihydroxyph	Benzene and substitu	Benzene and substitu	169.0501088
MEDN1814	Dihydrodaidzein	Benzene and substitu	Benzene and substitu	255.0659546
MEDP0361	4-Hydroxybenzaldeh	Benzene and substitu	Benzene and substitu	123.04
MADP0559	CAY10574	Benzene and substitu	Benzene and substitu	219.0991
MADN0222	Daidzein	Benzene and substitu	Benzene and substitu	313.0773
MADP0198	4-Propylphenol	Benzene and substitu	Benzene and substitu	178.1218
MEDN0739	4-Hydroxybenzyl alc	Benzene and substitu	Benzene and substitu	123.0446295
MEDN1770	2-Naphthalenesulfoni	Benzene and substitu	Benzene and substitu	207.0124705
MEDN1837	2,6-Di-tert-butyl-4-(h	Benzene and substitu	Benzene and substitu	235.168
MEDN0554	2-(4-Hydroxyphenyl)	Benzene and substitu	Benzene and substitu	137.0602796
MEDP2104	3-aminoquinoline	Benzene and substitu	Benzene and substitu	145.0757936

MEDP1033	Benzaldehyde	Benzene and substitu	Benzene and substitu	107.0484
MEDP1003	1-Hydroxylamino-2-p	Benzene and substitu	Benzene and substitu	120.0726939
MEDP2099	4,5-Diaminofluoresce	Benzene and substitu	Benzene and substitu	363.0894108
MEDP2139	4-Chloroaniline	Benzene and substitu	Benzene and substitu	128.0261739
MEDP0798	1-Naphthylamine	Benzene and substitu	Benzene and substitu	144.1
MEDP2229	α-CEHC	Benzene and substitu	Benzene and substitu	279.1593549
MEDN1516	6-Hydroxyflavone (6-	Benzene and substitu	Benzene and substitu	237.0564742
MEDP2113	2-Phenylpropylamine	Benzene and substitu	Benzene and substitu	136.111547
MEDP2255	Chrysoeriol	Benzene and substitu	Benzene and substitu	301.0723
MEDN1878	Trolox	Benzene and substitu	Benzene and substitu	249.1134922
MEDP1898	3-Hydroxyphenylurea	Benzene and substitu	Benzene and substitu	153.06458
MEDP2245	2-Amino-4-methylph	Benzene and substitu	Benzene and substitu	124.0759
MEDP1035	alpha-hexylcinnamald	Benzene and substitu	Benzene and substitu	217.1586301
MADN0537	Monophenyl phospha	Benzene and substitu	Benzene and substitu	173.0015071
MEDP2082	N-(3-Hydroxypropyl)	Benzene and substitu	Benzene and substitu	206.0811767
MADP0561	Metrizamide	Benzene and substitu	Benzene and substitu	789.8619
MEDN1876	4-Hydroxy-3-methox	Benzene and substitu	Benzene and substitu	183.0655
MADP0495	3-aminobenzamide	Benzene and substitu	Benzene and substitu	137.0708926
MEDP2236	URB937	Benzene and substitu	Benzene and substitu	355.1652812
MEDP2271	N-arachidonic acid-L	Benzene and substitu	Benzene and substitu	392.2805
MEDP1830	Ethyl 4-Methoxycinn	Benzene and substitu	Phenolic acids	207.1020801
MEDN1213	2-(4-hydroxyphenyl)	Benzene and substitu	Phenolic acids	165.0603756
MEDN0086	Terephthalic Acid	Benzene and substitu	Phenolic acids	165.0188087
MADN0424	3-Hydroxyanthranilic	Benzene and substitu	Phenolic acids	152.0362
MEDN1706	3-(2-Hydroxyphenyl)	Benzene and substitu	Phenolic acids	165.0603594
MADN0270	Phthalic Acid	Benzene and substitu	Phenolic acids	165.0188
MADN0411	3-Amino-4-Hydroxyb	Benzene and substitu	Phenolic acids	152.0351
MEDP1218	3-Amino-2-menacarb	Benzene and substitu	Phenolic acids	170.1
MEDN0092	3,4-Dihydroxybenzen	Benzene and substitu	Phenolic acids	167.1
MEDP1242	4-tert-butylbenzoic ac	Benzene and substitu	Phenolic acids	179.10617
MEDN1549	Hydroumbellic acid	Benzene and substitu	Phenolics	181.0546485
MADP0271	3-Methoxytyramine	Benzene and substitu	Phenolics	168.1015
MEDP0745	Creosol	Benzene and substitu	Phenolics	139.2

MEDN0115	Chenodeoxycholic A	Bile acids	Bile acids	391.2839454
MEDN1899	Deoxycholic acid	Bile acids	Bile acids	391.2845173
MEDN1906	3-Epideoxycholic aci	Bile acids	Bile acids	391.2854728
MEDN1154	Cholic acid	Bile acids	Bile acids	407.2816526
MEDN1059	Tauroursodeoxycholi	Bile acids	Bile acids	498.2885821
MEDN1100	Taurohyodeoxycholic	Bile acids	Bile acids	498.2885821
MEDN1126	Gamma-Mercholic A	Bile acids	Bile acids	407.2794195
MEDN0106	Taurochenodesoxych	Bile acids	Bile acids	498.2889592
MEDN1077	alpha-Muricholic acid	Bile acids	Bile acids	407.279268
MEDN1104	7-ketolithocholic acid	Bile acids	Bile acids	389.2680449
MEDN1113	12-ketolithocholic ac	Bile acids	Bile acids	389.2680449
MEDN1141	Apocholic acid	Bile acids	Bile acids	389.2680449
MEDN1581	Taurohyocholic acid	Bile acids	Bile acids	498.1955
MEDN1907	Taurine-α-ratcholate	Bile acids	Bile acids	498.1955
MEDN1902	beta-Muricholic acid	Bile acids	Bile acids	407.2796792
MADN0571	(2R)-Glycerol-O-β-D	Carbohydrates and I	Carboxylic acids and	253.0935
MADN0610	D-Gulonic acid γ-lact	Carbohydrates and I	Carboxylic acids and	177.0402187
MEDN1897	L-Gulose	Carbohydrates and I	Carboxylic acids and	179.056179
MADN0527	L-Iditol	Carbohydrates and I	Carboxylic acids and	181.0718863
MEDN1653	D-Glucose 1,6-bisphc	Carbohydrates and I	Phosphate sugars	338.9888
MEDN1057	2-Methyl-d-erythritol	Carbohydrates and I	Phosphate sugars	276.9878552
MEDN1221	D-Mannose 6-phosph	Carbohydrates and I	Phosphate sugars	259.0219186
MADN0397	Sucrose 6′-monophos	Carbohydrates and I	Phosphate sugars	421.076
MEDN1614	D-Arabinose 5-phosp	Carbohydrates and I	Phosphate sugars	229.0113
MEDN0498	Ribulose-5-Phosphate	Carbohydrates and I	Phosphate sugars	229.0113539
MEDN0641	Ribose 1-phosphate	Carbohydrates and I	Phosphate sugars	229.0113539
MEDN0657	D-Xylulose 5-phosph	Carbohydrates and I	Phosphate sugars	229.0113539
MEDN0506	N-Acetylglucosamine	Carbohydrates and I	Phosphate sugars	300.0484677
MADN0129	D-Fructose 6-Phosph	Carbohydrates and I	Phosphate sugars	271.0099
MEDN0502	2-Deoxyribose 1-Pho	Carbohydrates and I	Phosphate sugars	213.0164393
MEDN0461	D-Sedoheptuiose 7-P	Carbohydrates and I	Phosphate sugars	287.0168332
MADN0054	D-Glucose 6-Phospha	Carbohydrates and I	Phosphate sugars	259.0224
MADP0292	Glucosamine	Carbohydrates and I	Sugar acids	180.086

MEDN0531	D-Glucarate	Carbohydrates and I	Sugar acids	209
MEDN1314	Mucic Acid	Carbohydrates and I	Sugar acids	209
MADN0174	Gluconolactone	Carbohydrates and I	Sugar acids	177.0405
MADN0587	2-keto-D-gluconic ac	Carbohydrates and I	Sugar acids	193.0348
MADN0065	L-Gulonolactone	Carbohydrates and I	Sugar acids	177.0405
MADN0399	D-Galacturonic Acid	Carbohydrates and I	Sugar acids	193.0358
MADN0243	D-Glucoronic Acid	Carbohydrates and I	Sugar acids	193.0347
MADN0563	2-Azidoethyl 2-(Acet	Carbohydrates and I	Sugar acids	289.1155204
MADN0184	Dl-Threitol	Carbohydrates and I	Sugar Alcoholss	121.0506
MADN0046	D-Sorbitol	Carbohydrates and I	Sugar Alcoholss	181.0718
MADN0172	Xylitol	Carbohydrates and I	Sugar Alcoholss	151.0612
MADN0185	1,5-Anhydro-D-gluci	Carbohydrates and I	Sugar Alcoholss	163.0612
MADN0161	D-Mannitol	Carbohydrates and I	Sugar Alcoholss	181.0718
MADN0023	Galactitol	Carbohydrates and I	Sugar Alcoholss	181.0718
MADN0045	D-Arabinitol	Carbohydrates and I	Sugar Alcoholss	151.0612
MADN0153	Inositol	Carbohydrates and I	Sugar Alcoholss	179.0561
MADN0396	Galactinol	Carbohydrates and I	Sugar Alcoholss	341.1077
MEDP1246	2,4-diacetamino-2,4,6	Carbohydrates and I	Sugar derivatives	247.1
MADN0143	Methyl beta-D-galact	Carbohydrates and I	Sugar derivatives	193.0718
MADN0225	Sedoheptulose	Carbohydrates and I	Sugars	209.0651
MEDN1757	Isomaltotriose	Carbohydrates and I	Sugars	503.1602996
MEDN1758	dextrin	Carbohydrates and I	Sugars	503.1602996
MEDN0570	L-Erythrulose	Carbohydrates and I	Sugars	119.0344587
MADN0221	D-Quinovose	Carbohydrates and I	Sugars	223.0815
MEDN0539	3'-Sialyllactose	Carbohydrates and I	Sugars	632.203828
MADN0060	Maltose	Carbohydrates and I	Sugars	341.1089
MADN0061	Maltotriose	Carbohydrates and I	Sugars	503.1618
MADN0051	D-Trehalose	Carbohydrates and I	Sugars	341.1089
MEDP1844	alpha-D-Glucopyrano	Carbohydrates and I	Sugars	343.1229
MADN0370	D-(+)-Cellobiose	Carbohydrates and I	Sugars	341.1076
MADN0223	meso-Erythritol	Carbohydrates and I	Sugars	103.0398
MEDN1761	D-(+)-talose	Carbohydrates and I	Sugars	179.056179
MEDN1762	D-Allose	Carbohydrates and I	Sugars	179.056179

MADN0058	L-Fucose	Carbohydrates and I	Sugars	163.0612
MEDP1990	Melibiose	Carbohydrates and I	Sugars	381.0784
MADN0181	D-(+)-sucrose	Carbohydrates and I	Sugars	341.1089
MADN0053	D-Erythronolactone	Carbohydrates and I	Sugars	117
MADP0082	Nicotinamide-N-Oxid	CoEnzyme and vitar	CoEnzyme and vitar	139.0503
MEDP0240	Pyridoxine	CoEnzyme and vitar	CoEnzyme and vitar	170.1
MEDP0243	4-Oxoretinol	CoEnzyme and vitar	CoEnzyme and vitar	301.2
MADN0070	Riboflavin	CoEnzyme and vitar	CoEnzyme and vitar	375.1307
MADN0213	Pantothenic acid	CoEnzyme and vitar	CoEnzyme and vitar	218.1024
MADP0083	Nicotinic Acid	CoEnzyme and vitar	CoEnzyme and vitar	124.0388
MADP0086	Trigonelline	CoEnzyme and vitar	CoEnzyme and vitar	138.0553
MADP0279	Thiamine	CoEnzyme and vitar	CoEnzyme and vitar	265.111
MADN0069	Pantothenol	CoEnzyme and vitar	CoEnzyme and vitar	204.1237
MADN0066	L-Ascorbate	CoEnzyme and vitar	CoEnzyme and vitar	175
MEDP0389	Pantetheine	CoEnzyme and vitar	CoEnzyme and vitar	279.13
MEDP0408	11-Cis-Retinol	CoEnzyme and vitar	CoEnzyme and vitar	287.3
MADP0256	Nicotinamide	CoEnzyme and vitar	CoEnzyme and vitar	123.0568
MEDN1712	Flavin Mononucleoti	CoEnzyme and vitar	CoEnzyme and vitar	455.0964214
MEDP1705	(E)-Guggulsterone	CoEnzyme and vitar	CoEnzyme and vitar	313.2171856
MEDP1499	All trans-Retinal	CoEnzyme and vitar	CoEnzyme and vitar	285
MEDP0143	Biotin	CoEnzyme and vitar	CoEnzyme and vitar	245.09
MEDP0895	Dethiobiotin	CoEnzyme and vitar	CoEnzyme and vitar	215.3
MADP0480	NADP+	CoEnzyme and vitar	CoEnzyme and vitar	744.0822592
MEDP0539	Ubiquinone-1	CoEnzyme and vitar	CoEnzyme and vitar	251.1205091
MEDP1523	Carnitine C15	FA	CAR	386.3265
MEDP1424	Carnitine C9:0	FA	CAR	302.2
MEDP1519	Carnitine C12:3	FA	CAR	338.2326
MEDP1411	Carnitine C13:0	FA	CAR	358.2588
MEDP1403	Carnitine C15:1	FA	CAR	384.3108
MEDP1395	Carnitine C17:0	FA	CAR	414.357774
MEDP1406	Carnitine C14:1	FA	CAR	370.2968065
MEDP1377	Carnitine C20:1-OH	FA	CAR	470.384
MEDP1416	Carnitine C11:0	FA	CAR	330.263902

MEDP1430	Carnitine C7:0	FA	CAR	274.1649
MEDP1402	Carnitine C14:1-OH	FA	CAR	386.2917215
MEDP1390	Carnitine C18:1	FA	CAR	426.3578
MEDP1398	Carnitine C16:1	FA	CAR	398.326494
MEDP1405	Carnitine C14:0	FA	CAR	372.31055
MEDP1414	Carnitine C12:1	FA	CAR	342.2639
MEDP1525	Carnitine C14:2:DC	FA	CAR	398.2537
MEDP1419	Carnitine C10:0	FA	CAR	316.2496915
MEDP1373	Carnitine C22:2	FA	CAR	480.4047
MEDP1656	Carnitine C16-OH	FA	CAR	416.33703
MEDP1388	Carnitine C18:3-OH	FA	CAR	438.3
MEDP1529	Carnitine C18:1-OH	FA	CAR	442.3527
MEDP1397	Carnitine C16:0	FA	CAR	400.3439215
MEDP1084	Carnitine C12:0	FA	CAR	344.2709223
MEDP1427	Carnitine C8:0	FA	CAR	288.1442
MEDP1407	Carnitine C14:2	FA	CAR	368.2795
MEDP1441	Carnitine C5:1	FA	CAR	244.1543
MADP0078	L-Carnitine	FA	CAR	162
MEDP1387	Carnitine C19:2	FA	CAR	438.3578
MEDP1531	Carnitine C18-OH	FA	CAR	444.3684
MEDP1392	Carnitine C18:3	FA	CAR	422.3265
MEDP1289	Carnitine C6:0	FA	CAR	260.1762853
MEDP1391	Carnitine C18:2	FA	CAR	424.342149
MEDP1526	Carnitine C16:2-OH	FA	CAR	412.3058
MEDP1433	Carnitine ph-C1	FA	CAR	266.1387
MEDP1399	Carnitine C16:2	FA	CAR	396.3125115
MEDP1434	Carnitine C4:DC	FA	CAR	262.1285
MEDP1386	Carnitine C18:2-OH	FA	CAR	440.3371
MEDP1417	Carnitine C10:1-OH	FA	CAR	330.2
MEDP1428	Carnitine C8:1	FA	CAR	286.2013
MEDP1422	Carnitine C8-OH	FA	CAR	304.2
MADP0267	Carnitine C2:0	FA	CAR	204.1225
MADP0268	Carnitine isoC4:0	FA	CAR	232.1536

MEDP0618	Carnitine 2-methyl-C	FA	CAR	246.1627082
MEDP1440	Carnitine C5:0	FA	CAR	246.1627082
MEDP1442	Carnitine C4:0	FA	CAR	232.154344
MEDP1444	FFA(18:5)	FA	FFA	275.2
MEDN1139	DL-2-hydroxystearic	FA	FFA	299.258645
MEDN0807	FFA(14:0)	FA	FFA	227.2011301
MEDN0394	FFA(20:2)	FA	FFA	307.2637304
MEDN1805	FFA(16:1)	FA	FFA	253.2176
MEDN1409	12-Hydroxyoctadecar	FA	FFA	299.2609653
MEDN0385	FFA(12:0)	FA	FFA	199.16983
MEDP0634	FFA(20:1)	FA	FFA	311.2871805
MEDN1112	16-Hydroxyhexadeca	FA	FFA	271.2273449
MEDN1148	2-hydroxyhexadecan	FA	FFA	271.2273449
MEDN1840	Pinolenic acid	FA	FFA	277.2177
MEDN0383	FFA(18:2)	FA	FFA	279.2324303
MEDN0381	FFA(16:0)	FA	FFA	255.2324303
MEDN1069	FFA(18:4)	FA	FFA	275.2011301
MEDN1079	FFA(22:6)	FA	FFA	327.2324303
MEDP2226	Bovinic acid	FA	FFA	281.2482082
MEDN0494	8,15-Dihete	FA	FFA	335.2222595
MEDN1156	FFA(20:4)	FA	FFA	303.2
MEDN0378	FFA(18:3)	FA	FFA	277.2167802
MEDN1159	FFA(15:1)	FA	FFA	239.2011301
MEDN0395	FFA(20:3)	FA	FFA	305.2480803
MEDN1854	9,10-dihydroxysteari	FA	FFA	315.2548
MEDN1406	Tridecanedioic acid	FA	FFA	243.1605522
MEDN0604	Tetradecanedioic aci	FA	FFA	257.1753093
MEDN0658	Hexadecanedioic aci	FA	FFA	285.2066094
MEDN0768	13-oxoODE	FA	Oxidized lipids	293.2116948
MEDN0784	9-oxoODE	FA	Oxidized lipids	293.2116948
MEDN0771	15-OxoETE	FA	Oxidized lipids	317.2116948
MEDN1426	9(S),12(S),13(S)-TriI	FA	Oxidized lipids	329.2334368
MEDN1035	13(R)-HODE	FA	Oxidized lipids	295.2

MEDN1446	11-HEDE	FA	Oxidized lipids	323.5
MEDN1447	15-HEDE	FA	Oxidized lipids	323.5
MEDN0350	(±)15-HETE	FA	Oxidized lipids	319.2273449
MEDN0760	(±)5-HETE	FA	Oxidized lipids	319.2273449
MEDN0763	(±)9-HETE	FA	Oxidized lipids	319.2273449
MEDN1054	Tetranor-12(R)-HETI	FA	Oxidized lipids	265.1803947
MEDN1144	9(S)-HODE	FA	Oxidized lipids	295.2
MEDN0751	(±)12-HETE	FA	Oxidized lipids	319.2273449
MEDN0375	13-HOTrE	FA	Oxidized lipids	293.2116948
MEDN0779	5-HETrE	FA	Oxidized lipids	321.2429949
MEDN1422	8(S)-HETrE	FA	Oxidized lipids	321.2429949
MEDN1423	15(S)-HETrE	FA	Oxidized lipids	321.2429949
MEDN0765	11,12-EET	FA	Oxidized lipids	319.2273449
MEDN1078	cis-EODA	FA	Oxidized lipids	297.2429397
MEDN0391	DHA	FA	Oxidized lipids	327.2324303
MEDN0390	EPA	FA	Oxidized lipids	301.2167802
MEDN0398	AA	FA	Oxidized lipids	303.2324303
MEDN0767	12,13-EpOME	FA	Oxidized lipids	295.2273449
MEDN0783	9,10-EpOME	FA	Oxidized lipids	295.2273449
MEDN1463	(±)19(20)-DiHDPE(A	FA	Oxidized lipids	361.2402432
MEDN0376	9,10-DiHOME	FA	Oxidized lipids	313.2379096
MEDN1081	12,13-DiHOME	FA	Oxidized lipids	313.2379096
MEDN1428	20-COOH-AA	FA	Oxidized lipids	333.2074847
MEDN1462	(±)13(14)-DiHDPE(A	FA	Oxidized lipids	361.2402432
MEDN1438	(±)8(9)-DiHET	FA	Oxidized lipids	337.2395767
MEDN1439	(±)11(12)-DiHET	FA	Oxidized lipids	337.2395767
MEDN0777	5,6-DiHETrE	FA	Oxidized lipids	337.2379096
MEDP1834	Glycerol Tributyrate	GL	TG	303.1805878
MEDN0372	LPA(0:0/16:0)	GP	LPA	409.2363
MEDN1600	LPA(16:0/0:0)	GP	LPA	409.2363
MEDN1643	LPA(18:1/0:0)	GP	LPA	435.2492
MEDP1335	LPC(20:5/0:0)	GP	LPC	542.3157118
MEDP1336	LPC(0:0/20:5)	GP	LPC	542.3157118

MEDP1696	LPC(18:4/0:0)	GP	LPC	516.309017
MEDP1702	LPC(0:0/20:1)	GP	LPC	550.37
MEDP0338	LPC(16:0/0:0)	GP	LPC	496.33
MEDP1326	LPC(20:1/0:0)	GP	LPC	550.37
MEDP1346	LPC(0:0/16:0)	GP	LPC	496.331013
MEDP1701	LPC(0:0/18:1)	GP	LPC	522.3465016
MEDN0370	LPA(0:0/18:0)	GP	LPC	437.2668403
MEDP1333	LPC(0:0/20:4)	GP	LPC	544.3314584
MEDP1334	LPC(20:4/0:0)	GP	LPC	544.3314584
MEDP1322	LPC(22:5/0:0)	GP	LPC	570.3469171
MADP0289	Phosphocholine	GP	LPC	184.073
MEDP0434	LPC(17:0/0:0)	GP	LPC	510.1
MEDP0336	LPC(0:0/14:0)	GP	LPC	468.3
MEDP1691	LPC(0:0/17:0)	GP	LPC	510.35275
MEDP1902	LPC(16:2/0:0)	GP	LPC	492
MEDP1700	LPC(0:0/18:0)	GP	LPC	524.3629362
MEDP0537	LPC(O-16:0/2:0)	GP	LPC	524.36
MEDP1686	LPE(15:0/0:0)	GP	LPC	440.27671
MEDP0494	LPC(15:0/0:0)	GP	LPC	482.3
MEDP1699	LPC(0:0/16:1)	GP	LPC	494.3146631
MEDP1146	LPC(O-18:0/0:0)	GP	LPC	510.3914
MEDP1659	LPC(O-0:0/18:0)	GP	LPC	510.3914
MEDP1693	LPC(12:0/0:0)	GP	LPC	440.277717
MEDP1658	LPC(O-18:1/0:0)	GP	LPC	508.37615
MEDP1337	LPC(18:0/0:0)	GP	LPC	524.3629362
MEDP1697	LPC(14:1/0:0)	GP	LPC	466.293367
MEDP1339	LPC(18:1/0:0)	GP	LPC	522.3465016
MEDP1348	LPC(16:1/0:0)	GP	LPC	494.3146631
MEDP1331	LPC(0:0/20:3)	GP	LPC	546.3468047
MEDP1332	LPC(20:3/0:0)	GP	LPC	546.3468047
MEDP1345	LPC(18:3/0:0)	GP	LPC	518.316194
MEDP1321	LPC(0:0/22:5)	GP	LPC	570.3472654
MEDP0495	LPC(0:0/18:2)	GP	LPC	520.3302244

MEDP1341	LPC(18:2/0:0)	GP	LPC	520.3302244
MEDP1692	LPC(0:0/15:0)	GP	LPC	482.32233
MEDP1328	LPC(20:2/0:0)	GP	LPC	548.3610577
MEDP1330	LPC(0:0/20:2)	GP	LPC	548.3610577
MEDP1343	LPC(0:0/18:3)	GP	LPC	518.316194
MEDP1318	LPC(0:0/22:4)	GP	LPC	572.3625327
MEDP1319	LPC(22:4/0:0)	GP	LPC	572.3625327
MEDP1695	LPC(O-16:1/0:0)	GP	LPC-O	480.34387
MEDN1485	Glycerophospho-N-A	GP	LPE	500.27735
MEDN1276	LPE(20:4/0:0)	GP	LPE	500.2801187
MEDN0368	LPE(14:0/0:0)	GP	LPE	424.2464393
MEDP1875	LPE(17:1/0:0)	GP	LPE	466.2853
MEDN0362	LPE(18:1/0:0)	GP	LPE	478.2933894
MEDN1287	LPE(0:0/16:1)	GP	LPE	450.2642726
MEDN1273	LPE(0:0/20:3)	GP	LPE	502.2954782
MEDN1274	LPE(20:3/0:0)	GP	LPE	502.2954782
MEDN0366	LPE(16:0/0:0)	GP	LPE	452.2777394
MEDN1285	LPE(0:0/16:0)	GP	LPE	452.2777394
MEDN1267	LPE(0:0/22:5)	GP	LPE	526.2957649
MEDN1268	LPE(22:5/0:0)	GP	LPE	526.2957649
MEDN1597	Phosphatidylethanola	GP	LPE	436.24715
MEDN1271	LPE(0:0/20:2)	GP	LPE	504.3105507
MEDN1272	LPE(20:2/0:0)	GP	LPE	504.3105507
MEDP0638	LPE(16:1/0:0)	GP	LPE	452.2698892
MEDN1264	LPE(0:0/22:4)	GP	LPE	528.3100019
MEDN1265	LPE(22:4/0:0)	GP	LPE	528.3100019
MEDN1277	LPE(0:0/20:5)	GP	LPE	498.2627113
MEDN1278	LPE(20:5/0:0)	GP	LPE	498.2627113
MEDN1269	LPE(0:0/22:6)	GP	LPE	524.2793485
MEDN1270	LPE(22:6/0:0)	GP	LPE	524.2793485
MEDN1283	LPE(0:0/18:3)	GP	LPE	474.2636247
MEDN1284	LPE(18:3/0:0)	GP	LPE	474.2636247
MEDN1282	LPE(0:0/18:2)	GP	LPE	478.32518

MEDP1904	LPE(18:2/0:0)	GP	LPE	478.32518
MEDN0364	LPE(18:0/0:0)	GP	LPE	480.3090395
MEDN1279	LPE(0:0/18:0)	GP	LPE	480.3090395
MEDN0360	LPG(18:1/0:0)	GP	LPG	509.2879697
MEDN0356	LPS(22:6/0:0)	GP	LPS	568.2675686
MADP0464	PC(24:0)	GP	PC	608.46
MADP0443	PC(16:1/P-18:1)	GP	PC	742.57
MADN0486	PC(16:1e/9-HODE)	GP	PC	816.57
MADP0448	PC(20:3(8Z,11Z,14Z	GP	PC	838.62
MADP0475	PC(18:1(9Z)/18:1(9Z	GP	PC	785.59
MADP0473	PC(18:0/22:6(4Z,7Z,	GP	PC	834.6
MADP0469	PC(16:0/18:2)	GP	PC	758.57
MADP0445	PC(18:1(9Z)/18:3(6Z	GP	PC	782.57
MADP0456	PC(22:6(4Z,7Z,10Z,1	GP	PC	860.61
MADP0457	PC(22:5(4Z,7Z,10Z,1	GP	PC	820.62
MADP0442	PC(16:0/16:0)	GP	PC	734.56
MADP0468	PC(16:0/22:6)	GP	PC	806.56
MADP0478	PC(18:0/18:1)	GP	PC	810.59
MADP0436	PC(20:4(8Z,11Z,14Z	GP	PC	794.6
MADP0447	PC(22:2(13Z,16Z)/15	GP	PC	800.61
MADP0440	PC(15:0/15:0)	GP	PC	706
MADP0449	PC(20:3(8Z,11Z,14Z	GP	PC	756.54
MADP0451	PC(16:1/16:1)	GP	PC	730.53813
MEDP1096	PC(12:0/12:0)	GP	PC	548.4
MEDP1068	PC(8:0/8:0)	GP	PC	510.3
MADP0438	PC(O-16:0/20:5(5Z,8	GP	PC-O	766.575
MADP0477	PC(O-16:0/20:3)	GP	PC-O	792.59
MEDP1221	PC(O-16:0/O-1:0)	GP	PC-O	496.3
MEDP1165	PC(O-16:0/O-2:0)	GP	PC-O	510.3914
MEDP1657	PC(O-16:0/0:0)	GP	PC-O	482.35971
MADP0453	PC(P-18:1(11Z)/22:2	GP	PC-P	824.64
MADP0474	PC(P-18:0/20:4)	GP	PC-P	794.6
MADN0482	PE(16:1e/14-HDoHE	GP	PE	762.51

MADN0469	PE(16:0/20:4)	GP	PE	738.51
MADP0472	PE(16:0/18:2(9Z,12Z	GP	PE	716.52
MADP0227	O-Phosphorylethanol	GP	PE	142.0255
MADN0472	PE-NMe2(18:1(9Z)/1	GP	PE	770.568
MADN0479	PI(34:2)	GP	PI	833.52
MADN0474	PI(36:2)	GP	PI	861.55
MADN0475	PS(18:0/18:0)	GP	PS	790.54
MADP0110	L-Pipecolic acid	Heterocyclic compo	Heterocyclic compo	130.0863
MADP0369	2-Acetylfuran	Heterocyclic compo	Heterocyclic compo	111.0446
MADP0202	Indole-3-acetonitrile	Heterocyclic compo	Heterocyclic compo	174.1017
MEDP1808	Trans-Zeatin	Heterocyclic compo	Heterocyclic compo	220.1214421
MADP0508	3-(imidazol-4-yl)prop	Heterocyclic compo	Heterocyclic compo	141.0646196
MADP0497	Ectoine	Heterocyclic compo	Heterocyclic compo	143.0810291
MEDP1060	Hydroxyquinoline	Heterocyclic compo	Heterocyclic compo	146.05122
MEDN1656	Hydroxypiperazic aci	Heterocyclic compo	Heterocyclic compo	145.09854
MADN0578	3-pyrimidin-2-yl-prop	Heterocyclic compo	Heterocyclic compo	151.0509
MADN0602	Pyrimidine-2-carboxy	Heterocyclic compo	Heterocyclic compo	123.0189
MADN0564	2-pyrimidine acetic a	Heterocyclic compo	Heterocyclic compo	137.0346594
MADP0540	2-pyrimidin-2-yl-prop	Heterocyclic compo	Heterocyclic compo	153.0656
MADP0323	5-Aminoimidazole-4-	Heterocyclic compo	Heterocyclic compo	127.0611
MEDP1211	N-acetylpyrrolidine	Heterocyclic compo	Heterocyclic compo	114.0661792
MADP0503	4-Amino-5-hydroxym	Heterocyclic compo	Heterocyclic compo	140.0816468
MADP0309	4,6-Diamino-5-forma	Heterocyclic compo	Heterocyclic compo	154.0729
MADP0313	N1,N8-diacetylsperm	Heterocyclic compo	Heterocyclic compo	230.187
MEDN1039	Indole-4-carboxaldeh	Heterocyclic compo	Indole and Its deriva	144.0449638
MEDP0276	Indole-3-Carboxaldel	Heterocyclic compo	Indole and Its deriva	146.1
MEDP1127	1-Acetylindole	Heterocyclic compo	Indole and Its deriva	160.1
MADP0303	Imidazoleacetic acid	Heterocyclic compo	Indole and Its deriva	127.0498
MEDN0656	Indoleacetaldehyde	Heterocyclic compo	Indole and Its deriva	158.0606139
MEDN0562	Indole-3-carbinol	Heterocyclic compo	Indole and Its deriva	146.0606139
MEDN1150	Indole-3-carboxylic a	Heterocyclic compo	Indole and Its deriva	160.0398785
MEDP0529	Indole-3-acetamide	Heterocyclic compo	Indole and Its deriva	175.079313
MEDP2298	Indole-3-acetic acid	Heterocyclic compo	Indole and Its deriva	176.0705

MEDN0263	5-Hydroxyindole-3-A	Heterocyclic compo	Indole and Its deriva	190.0504432
MEDN1514	5-hydroxyindole-2-ca	Heterocyclic compo	Indole and Its deriva	176.0361
MEDN0523	Indole-3-lactic acid	Heterocyclic compo	Indole and Its deriva	204.0660932
MEDP2007	3-Methyloxindole	Heterocyclic compo	Indole and Its deriva	148.0759
MEDP0273	5-Hydroxytryptophol	Heterocyclic compo	Indole and Its deriva	178.08
MEDP1098	Methoxyindoleacetic	Heterocyclic compo	Indole and Its deriva	206.0720614
MADN0543	5-fluoroindole-2-carb	Heterocyclic compo	Indole and Its deriva	178.030646
MEDP1063	Indole-5-carboxylic a	Heterocyclic compo	Indole and Its deriva	162.0452919
MADN0272	4-Pyridoxic Acid	Heterocyclic compo	Pteridines and deriva	182.0458
MEDP0670	2,6-Dimethylpyridine	Heterocyclic compo	Pteridines and deriva	108.2
MADP0236	2,4-Dihydroxypteridi	Heterocyclic compo	Pteridines and deriva	165.0395
MADP0266	1,4-Dihydro-1-Methy	Heterocyclic compo	Pteridines and deriva	153.0645
MEDP0898	Pyridoxal	Heterocyclic compo	Pteridines and deriva	168.3
MADP0367	2-Picoline	Heterocyclic compo	Pteridines and deriva	94.0692
MADN0196	2,4-Dihydroxypyridir	Heterocyclic compo	Pteridines and deriva	110.0247
MADN0318	3-Hydroxypicolinic a	Heterocyclic compo	Pteridines and deriva	138.0193
MEDN1484	Ethionamide	Heterocyclic compo	Pteridines and deriva	165.04111
MADN0113	Biopterin	Heterocyclic compo	Pteridines and deriva	236.09
MEDP0443	10-Formyl-Thf	Heterocyclic compo	Pteridines and deriva	474.2
MADN0237	6-Hydroxynicotinic A	Heterocyclic compo	Pteridines and deriva	138.0194
MADP0250	6-Methylnicotinamid	Heterocyclic compo	Pteridines and deriva	137.0743
MADP0354	Pterine	Heterocyclic compo	Pteridines and deriva	164.0568
MEDP1652	10-Formyl-7,8-dihydr	Heterocyclic compo	Pteridines and deriva	472.15763
MADN0516	Pyridine-3,4-dicarbox	Heterocyclic compo	Pyridine and pyridin	166.0142386
MADP0551	4-Oxo-4-(3-pyridyl)b	Heterocyclic compo	Pyridine and pyridin	164.0706
MEDP1758	Etiocholanone	Hormones and horm	Hormones and horm	291.2318545
MEDP1710	11-Ketoetiocholanolc	Hormones and horm	Hormones and horm	305.2117785
MADP0282	L-Phenylephrine	Hormones and horm	Hormones and horm	168.101
MEDP0184	3,3',5-Triiodo-L-Thyı	Hormones and horm	Hormones and horm	651.7
MEDP1500	16-Dehydroprogester	Hormones and horm	Hormones and horm	313
MEDP1272	20,26-dihydroxyecdy	Hormones and horm	Hormones and horm	497.3
MEDP0186	L-Thyroxine	Hormones and horm	Hormones and horm	777.687
MEDN1613	Cytochalasin-H	Hormones and horm	Hormones and horm	474.2559

MADP0532	2-amino-6-methylme	Nucleotide And Its	Nucleotide and Its r	182.0497059
MADP0499	6-Methylaminopurine	Nucleotide And Its	Nucleotide and Its r	150.0773429
MADP0506	2-amino-6-chloropuri	Nucleotide And Its	Nucleotide and Its r	170.023
MADP0520	2-Chloroadenosine	Nucleotide And Its	Nucleotide and Its r	302.0648797
MADP0580	2,2'-Cyclouridine	Nucleotide And Its	Nucleotide and Its r	227.0670025
MADP0591	Tubercidin	Nucleotide And Its	Nucleotide and Its r	267.1098467
MADN0570	6'-Sialyllactose	Nucleotide And Its	Nucleotide and Its r	632.2058
MEDP2221	6-Chloropurine Nucle	Nucleotide And Its	Nucleotide and Its r	287.0545956
MADP0585	4-Deoxyuridine	Nucleotide And Its	Nucleotide and Its r	229.0814701
MADN0573	Adenosine 2'-Phosph	Nucleotide And Its	Nucleotide and Its r	346.0561
MADN0628	Adenosine 5'-diphosp	Nucleotide And Its	Nucleotide and Its r	426.0236377
MADN0624	Cytidine-5-Monopho	Nucleotide And Its	Nucleotide and Its r	322.0446089
MADN0568	Uridine 5′-diphospho	Nucleotide And Its	Nucleotide and Its r	579.0279
MADN0627	Guanosine-5'-monopl	Nucleotide And Its	Nucleotide and Its r	362.0509443
MADP0190	Cytidine 5'-Diphosph	Nucleotide And Its	Nucleotide and Its r	489.1123
MEDN1045	Xanthopterin	Nucleotide And Its r	Nucleotide and Its r	178.0376
MADP0061	7-Methylxanthine	Nucleotide And Its r	Nucleotide and Its r	167.0563
MEDP1295	Cytarabine	Nucleotide And Its r	Nucleotide and Its r	244.0826219
MADN0027	1-Methylxanthine	Nucleotide And Its r	Nucleotide and Its r	165.0417
MEDN1612	Cytidine 2',3'-cyclic r	Nucleotide And Its r	Nucleotide and Its r	304.0343
MADP0318	Nicotinamide ribosid	Nucleotide And Its r	Nucleotide and Its r	255.0979
MADP0160	Ancitabine	Nucleotide And Its r	Nucleotide and Its r	226.0829
MADP0065	Cytidine	Nucleotide And Its r	Nucleotide and Its r	244.0933
MEDP1960	Isopentenyladenine-7	Nucleotide And Its r	Nucleotide and Its r	366.2
MADP0344	5-methylcytidine	Nucleotide And Its r	Nucleotide and Its r	258.1087
MADP0059	5-Methylcytosine	Nucleotide And Its r	Nucleotide and Its r	126.0663
MEDP1953	Eritadenine	Nucleotide And Its r	Nucleotide and Its r	254.0887
MADN0031	Adenine	Nucleotide And Its r	Nucleotide and Its r	134.0477
MADP0278	6-Dimethylaminopuri	Nucleotide And Its r	Nucleotide and Its r	164.0929
MADP0270	8-Azaguanine	Nucleotide And Its r	Nucleotide and Its r	153.0514
MADN0030	5-Hydroxymethylurac	Nucleotide And Its r	Nucleotide and Its r	141.0307
MADN0374	5-Hydroxy-2'-deoxyu	Nucleotide And Its r	Nucleotide and Its r	243.0631
MADP0149	N6-Succinyl Adenosi	Nucleotide And Its r	Nucleotide and Its r	384.1153

MEDP1950	2'-Deoxyinosine-5'-m	Nucleotide And Its r	Nucleotide and	Its r	333.052
MEDP1100	2-Aminomethylpyrim	Nucleotide And Its r	Nucleotide and	Its r	110.07047
MADN0124	B-Pseudouridine	Nucleotide And Its r	Nucleotide and	Its r	243.0627
MADP0076	Uridine	Nucleotide And Its r	Nucleotide and	Its r	245.0773
MADN0028	Xanthine	Nucleotide And Its r	Nucleotide and	Its r	151.0257
MEDP0177	Thymine	Nucleotide And Its r	Nucleotide and	Its r	127
MEDP0560	Dihydrouracil	Nucleotide And Its r	Nucleotide and	Its r	115.0429274
MEDP0175	Pyrimidine	Nucleotide And Its r	Nucleotide and	Its r	81.2
MEDP1654	1-Methyladenosine	Nucleotide And Its r	Nucleotide and	Its r	282.11921
MADP0067	Cytosine	Nucleotide And Its r	Nucleotide and	Its r	112.0503
MADN0128	Guanine	Nucleotide And Its r	Nucleotide and	Its r	150.0417
MEDP1788	N4-Acetylcytidine tri	Nucleotide And Its r	Nucleotide and	Its r	526.4311014
MADP0056	2-Hydroxy-6-Aminop	Nucleotide And Its r	Nucleotide and	Its r	152.0563
MADN0343	3'-UMP	Nucleotide And Its r	Nucleotide and	Its r	323.0283
MADN0034	Hypoxanthine	Nucleotide And Its r	Nucleotide and	Its r	135.0317
MADP0133	6-O-Methylguanine	Nucleotide And Its r	Nucleotide and	Its r	166.0723
MADP0077	B-Nicotinamide Mon	Nucleotide And Its r	Nucleotide and	Its r	335.0643
MADP0316	N4-Acetylcytidine	Nucleotide And Its r	Nucleotide and	Its r	286.1034
MADP0063	Adenosine	Nucleotide And Its r	Nucleotide and	Its r	268.1043
MADN0220	Deoxyribose 5-phosp	Nucleotide And Its r	Nucleotide and	Its r	273.0367
MADN0121	2-(Dimethylamino)G	Nucleotide And Its r	Nucleotide and	Its r	310.1157
MEDN0141	2'-Deoxyuridine	Nucleotide And Its r	Nucleotide and	Its r	227.0668215
MEDN1506	3-Methyluridine	Nucleotide And Its r	Nucleotide and	Its r	257.0781011
MADP0326	1-Methylguanosine	Nucleotide And Its r	Nucleotide and	Its r	298.1147
MEDN1501	2'-O-Methyluridine	Nucleotide And Its r	Nucleotide and	Its r	257.0801844
MEDN1602	3-Deoxyguanosine	Nucleotide And Its r	Nucleotide and	Its r	266.0893
MADP0107	7-Methylguanine	Nucleotide And Its r	Nucleotide and	Its r	166.0723
MADP0106	2'-Deoxyadenosine	Nucleotide And Its r	Nucleotide and	Its r	252.1093
MADP0320	1-Methylinosine	Nucleotide And Its r	Nucleotide and	Its r	283.1042
MEDP0461	2-Methylguanosine	Nucleotide And Its r	Nucleotide and	Its r	298.1
MEDP1072	7-methylguanosine	Nucleotide And Its r	Nucleotide and	Its r	298.1
MEDP1202	5'-Deoxy-5'-fluoroad	Nucleotide And Its r	Nucleotide and	Its r	270.1
MEDP1954	N6-(2-Hydroxyethyl)	Nucleotide And Its r	Nucleotide and	Its r	312.1302

MADN0119	Cyclic Amp	Nucleotide And Its r	Nucleotide and Its r	328.0457
MADN0035	Purine	Nucleotide And Its r	Nucleotide and Its r	119.0367
MEDP1956	2-Aminopurine	Nucleotide And Its r	Nucleotide and Its r	136.06177
MEDP1093	Allopurinol	Nucleotide And Its r	Nucleotide and Its r	137.0481928
MEDN0602	Deoxyguanosine 5'-m	Nucleotide And Its r	Nucleotide and Its r	346.0552844
MEDP1077	2'-O-methyladenosine	Nucleotide And Its r	Nucleotide and Its r	348.1
MEDN0537	ADP-ribose	Nucleotide And Its r	Nucleotide and Its r	558.0638737
MADN0029	3'-Aenylic Acid	Nucleotide And Its r	Nucleotide and Its r	346.0557
MEDP1010	Coenzyme II (β-NAD	Nucleotide And Its r	Nucleotide and Its r	744
MADN0032	Flavin Adenine Dinu	Nucleotide And Its r	Nucleotide and Its r	784.1497
MADP0346	5-hydroxymethyl-2'-d	Nucleotide And Its r	Nucleotide and Its r	258.1089
MEDN0624	5'-Adenylyl sulfate(A	Nucleotide And Its r	Nucleotide and Its r	426.0120993
MEDP0176	Thymidine	Nucleotide And Its r	Nucleotide and Its r	243.09
MADP0069	Guanosine	Nucleotide And Its r	Nucleotide and Its r	284.0993
MEDP1949	5-Aminoimidazole ril	Nucleotide And Its r	Nucleotide and Its r	296.0671
MADP0057	3-Methylxanthine	Nucleotide And Its r	Nucleotide and Its r	167.0563
MEDP0401	5'-Deoxy-5'-(Methylt	Nucleotide And Its r	Nucleotide and Its r	298
MADP0142	Adenosine 5'-Monopl	Nucleotide And Its r	Nucleotide and Its r	348.0703
MADP0345	2'-O-methylguanosine	Nucleotide And Its r	Nucleotide and Its r	298.1153
MADP0143	Inosine 5'-monophosp	Nucleotide And Its r	Nucleotide and Its r	349.0543
MEDN0534	Deoxyguanosine diph	Nucleotide And Its r	Nucleotide and Its r	426.021615
MADP0226	Xanthosine	Nucleotide And Its r	Nucleotide and Its r	285.0828
MADP0188	Cytidine 5'-monopho	Nucleotide And Its r	Nucleotide and Its r	615.1544
MEDP1162	1-Methylguanine	Nucleotide And Its r	Nucleotide and Its r	166.07051
MADP0206	Cyclic ADP ribose	Nucleotide And Its r	Nucleotide and Its r	542.0667
MADP0072	Nicotinic Acid Adeni	Nucleotide And Its r	Nucleotide and Its r	664.1163
MADP0054	2'-Deoxyadenosine-5'	Nucleotide And Its r	Nucleotide and Its r	332.0749
MEDN0573	GDP-L-fucose	Nucleotide And Its r	Nucleotide and Its r	588.0744384
MEDN0538	UDP-glucose	Nucleotide And Its r	Nucleotide and Its r	565.0472206
MEDN1087	UDP-D-galactose	Nucleotide And Its r	Nucleotide and Its r	565.0472206
MEDN0530	Pimelic acid	Organic acid And Its	Organic acid And Its	159.0697581
MADN0147	Oxaloacetic acid	Organic acid And Its	Organic acid And Its	131
MADN0091	Hippuric Acid	Organic acid And Its	Organic acid And Its	178.051

MEDN1312	2-Hydroxyphenylacet	Organic acid And Its	Organic acid And Its	151
MEDN1315	4-Hydroxy-3-methylb	Organic acid And Its	Organic acid And Its	151
MADN0084	Azelaic Acid	Organic acid And Its	Organic acid And Its	187.0976
MADN0180	(R)-2-Hydroxy-3-phe	Organic acid And Its	Organic acid And Its	165.0557
MEDN1311	2-Hydroxycaprylic ac	Organic acid And Its	Organic acid And Its	159
MEDN0206	Citramalic Acid	Organic acid And Its	Organic acid And Its	147.0293734
MEDN1052	2-Hydroxyglutaric ac	Organic acid And Its	Organic acid And Its	147.0293734
MADN0177	Guanidineacetic Acid	Organic acid And Its	Organic acid And Its	116.0465
MADN0122	5-Hydroxyhexanoic A	Organic acid And Its	Organic acid And Its	131.0714
MADN0043	Pyruvic Acid	Organic acid And Its	Organic acid And Its	87.0088
MADP0089	4-Acetamidobutyric A	Organic acid And Its	Organic acid And Its	146.0812
MEDP2018	3-phenylbutyric acid	Organic acid And Its	Organic acid And Its	165.0905497
MEDN0338	DL-3-Phenyllactic ac	Organic acid And Its	Organic acid And Its	165.0551942
MADN0114	Ethylmalonic acid	Organic acid And Its	Organic acid And Its	131.035
MEDN1235	L-2-amino-6-oximeli	Organic acid And Its	Organic acid And Its	188.0567506
MADP0090	4-Guanidinobutyric A	Organic acid And Its	Organic acid And Its	146.0924
MEDN1499	2-Hydroxyhexanoic a	Organic acid And Its	Organic acid And Its	131.0719
MADN0421	2,6-Dihydroxybenzoi	Organic acid And Its	Organic acid And Its	153.019
MEDN1328	L-Leucic acid	Organic acid And Its	Organic acid And Its	131
MEDN0284	2-Hydroxy-4-methylv	Organic acid And Its	Organic acid And Its	131.0708442
MADN0042	Cis-Aconitic Acid	Organic acid And Its	Organic acid And Its	173.0092
MADN0108	Subericacid	Organic acid And Its	Organic acid And Its	173.0819
MADP0101	Urocanic Acid	Organic acid And Its	Organic acid And Its	139.0502
MADN0037	Pyrroloquinoline Qui	Organic acid And Its	Organic acid And Its	329.0051
MADN0064	D-Glyceric Acid	Organic acid And Its	Organic acid And Its	105.0193
MADN0150	2,4-Hexadienoic acid	Organic acid And Its	Organic acid And Its	111.0452
MEDN1098	2-ethyl-2-hydroxybut	Organic acid And Its	Organic acid And Its	131.07071
MEDN1621	Isocitric acid	Organic acid And Its	Organic acid And Its	173.0087
MADN0088	Dl-2-Aminooctanoic	Organic acid And Its	Organic acid And Its	158.1186
MADN0134	Dl-3,4-Dihydroxymai	Organic acid And Its	Organic acid And Its	183
MADN0323	Dihydrocaffeic Acid	Organic acid And Its	Organic acid And Its	181.0499
MADN0164	Iminodiacetic acid	Organic acid And Its	Organic acid And Its	132.0302
MEDN1226	2-amino-4-oxovaleric	Organic acid And Its	Organic acid And Its	130.0504432

MADN0107	Shikimic Acid	Organic acid And Its	Organic acid And Its	173.0455
MADN0191	3-Methacrylic acid	Organic acid And Its	Organic acid And Its	99.0452
MEDN1504	3-Hydroxycinnamic a	Organic acid And Its	Organic acid And Its	163.0443643
MADN0155	3-Hydroxypropionic a	Organic acid And Its	Organic acid And Its	167.035
MADN0238	Uric acid	Organic acid And Its	Organic acid And Its	167.0208
MADN0190	2,5-Furandicarboxylic	Organic acid And Its	Organic acid And Its	154.9986
MADN0111	3-(3-Hydroxyphenyl)	Organic acid And Its	Organic acid And Its	165.0557
MADN0198	Maleamic acid	Organic acid And Its	Organic acid And Its	114.0197
MADN0080	3-Hydroxybutyric aci	Organic acid And Its	Organic acid And Its	103.0401
MEDP2008	2-Hydroxy-4-(methyl	Organic acid And Its	Organic acid And Its	151.0418621
MADN0360	Tricarballylic acid	Organic acid And Its	Organic acid And Its	175.0247
MADP0224	L-2-Aminobutyric ac	Organic acid And Its	Organic acid And Its	104.695
MADN0376	(R)-3-Hydroxybutanc	Organic acid And Its	Organic acid And Its	103.0405
MEDP1247	1-deoxyvaleric acid	Organic acid And Its	Organic acid And Its	235.2
MADN0095	L-Lactic Acid	Organic acid And Its	Organic acid And Its	89.0244
MEDP1857	Eicosanoyl-EA	Organic acid And Its	Organic acid And Its	356.352
MADN0041	α-Ketoglutaric Acid	Organic acid And Its	Organic acid And Its	145.0142
MADN0219	1-Hydroxy-2-Naphth	Organic acid And Its	Organic acid And Its	187.0412
MEDN0678	Ethylsalicylate	Organic acid And Its	Organic acid And Its	165.0551942
MEDN0342	Sebacate	Organic acid And Its	Organic acid And Its	201.1127091
MEDN1320	2-Hydroxy-2-Methyl	Organic acid And Its	Organic acid And Its	117
MADN0104	Pyrrole-2-Carboxylic	Organic acid And Its	Organic acid And Its	110.0247
MADN0132	2-Oxoadipic acid	Organic acid And Its	Organic acid And Its	159.0299
MADN0083	Adipic Acid	Organic acid And Its	Organic acid And Its	145.0506
MEDN1630	Ferulic acid	Organic acid And Its	Organic acid And Its	193.0502
MADP0093	Creatinine	Organic acid And Its	Organic acid And Its	114.0662
MEDP0627	Isonicotinic acid	Organic acid And Its	Organic acid And Its	124.0320284
MADN0163	Ureidosuccinic acid	Organic acid And Its	Organic acid And Its	175.04
MADN0079	3-Hydroxy-3-Methyl	Organic acid And Its	Organic acid And Its	117.0557
MADP0213	p-(Phenylazo)benzen	Organic acid And Its	Organic acid And Its	263.0436
MADN0148	Thiodiglycolic Acid	Organic acid And Its	Organic acid And Its	148.9914
MADN0156	3-Hydroxyglutaric ac	Organic acid And Its	Organic acid And Its	147.0299
MADN0186	4-Hydroxy-2-Oxoglu	Organic acid And Its	Organic acid And Its	161.0092

MEDP2002	5-Aminolevulinate	Organic acid And Its	Organic acid And Its	132.0655421
MADN0427	D-Malic acid	Organic acid And Its	Organic acid And Its	133.0147
MADN0010	Allantoin	Organic acid And Its	Organic acid And Its	157.0367
MEDP0885	Mesoxalate	Organic acid And Its	Organic acid And Its	119
MADP0328	3-Amino-4-methylpe	Organic acid And Its	Organic acid And Its	132.1023
MEDP2024	Dehydroascorbic acid	Organic acid And Its	Organic acid And Its	175.023348
MADN0086	Creatine	Organic acid And Its	Organic acid And Its	130.0627
MADN0089	Glutaric Acid	Organic acid And Its	Organic acid And Its	131.035
MADN0097	L-Dihydroorotic Acid	Organic acid And Its	Organic acid And Its	157.0255
MADN0133	1,2-Ethanediol, 1-(3-	Organic acid And Its	Organic acid And Its	263
MEDP1231	Folic acid	Organic acid And Its	Organic acid And Its	474.2
MADP0208	Proline betaine	Organic acid And Its	Organic acid And Its	144.101
MADN0365	2,6-Dimethoxybenzo	Organic acid And Its	Organic acid And Its	181.05
MEDN0334	Mandelic Acid	Organic acid And Its	Organic acid And Its	151.0395441
MADN0328	3-Ureidopropionic ac	Organic acid And Its	Organic acid And Its	131.0457
MEDN1009	3-Hydroxyphenylacet	Organic acid And Its	Organic acid And Its	151.0395441
MEDN1566	Octadecanedioic acid	Organic acid And Its	Organic acid And Its	313.2429372
MEDN1533	Dimethylmalonic aci	Organic acid And Its	Organic acid And Its	131.0356986
MADN0077	Methylsuccinic acid	Organic acid And Its	Organic acid And Its	131.035
MADN0141	Fumaric Acid	Organic acid And Its	Organic acid And Its	115.0037
MADP0207	4-(Aminomethyl)ben	Organic acid And Its	Organic acid And Its	152.0702
MADN0245	2-Aminoadipic Acid	Organic acid And Its	Organic acid And Its	160.0608
MEDP1132	3-Carboxypropyltrim	Organic acid And Its	Organic acid And Its	146.1084
MADN0140	3-Methyl-2-Oxobutar	Organic acid And Its	Organic acid And Its	115
MADN0206	2-Hydroxy-3-Methyll	Organic acid And Its	Organic acid And Its	117.0557
MEDN0499	Argininosuccinic aci	Organic acid And Its	Organic acid And Its	289.1148343
MADN0040	Succinic Acid	Organic acid And Its	Organic acid And Its	117.0193
MEDP2108	3-(4-fluorobenzoyl)p	Organic acid And Its	Organicacid and Its	197.0600447
MEDP2230	8-iso-15-keto Prostag	Organic acid And Its	Organicacid and Its	353.2331362
MEDN1886	(S)-2-Hydroxy-3-phe	Organic acid And Its	Organicacid and Its	165.0562824
MEDN1856	(R)-3-Hydroxymyrist	Organic acid And Its	Organicacid and Its	243.196518
MEDN1717	Tropine	Organic acid And Its	Organicacid and Its	165.0559984
MADN0591	3-Hydroxyvalproic ac	Organic acid And Its	Organicacid and Its	159.102

MADN0530	2-Methoxyacetic acid	Organic acid And Its	Organicacid and Its	89.02469427
MEDP2121	1-aminopentadecane	Organic acid And Its	Organicacid and Its	228.2687416
MADN0616	cis,cis-Muconic acid	Organic acid And Its	Organicacid and Its	141.0156815
MADN0520	3,5-Dichlorosalicylic	Organic acid And Its	Organicacid and Its	204.9464517
MEDN1722	Piperic acid	Organic acid And Its	Organicacid and Its	165.019214
MADN0524	3-Amino-5-hydroxyb	Organic acid And Its	Organicacid and Its	152.0350297
MEDN1719	Tetradecyl phosphoni	Organic acid And Its	Organicacid and Its	277.195613
MEDN1714	Isokaurenoic acid	Organic acid And Its	Organicacid and Its	301.2168199
MEDP2283	ST-638	Organic acid And Its	Organicacid and Its	355.1117
MADP0563	(2-Hydroxyethyl)pho:	Organic acid And Its	Organicacid and Its	127.0148
MADN0574	α-Hydroxyglutaric A	Organic acid And Its	Organicacid and Its	147.0296
MEDP2223	Cork oximate	Organic acid And Its	Organicacid and Its	205.1184598
MADN0512	(+)-Camphoric acid	Organic acid And Its	Organicacid and Its	199.0975218
MADN0504	Mesoaconic acid	Organic acid And Its	Organicacid and Its	129.0182
MADN0505	Sulfoacetic acid	Organic acid And Its	Organicacid and Its	138.9700797
MADN0513	4-toluenesulfonic aci	Organic acid And Its	Organicacid and Its	171.0117315
MADP0498	3-Guanidinopropioni	Organic acid And Its	Organicacid and Its	132.076206
MADN0609	L-Cysteic acid	Organic acid And Its	Organicacid and Its	167.9959342
MADN0617	2-Methylpentanedioi	Organic acid And Its	Organicacid and Its	145.0503115
MADN0608	2-Hydroxybutyric aci	Organic acid And Its	Organicacid and Its	103.039166
MADN0618	2-Methyllactic acid	Organic acid And Its	Organicacid and Its	103.0392742
MEDN0615	Carbamoyl phosphate	Organic acid And Its	Phosphoric acids	139.9749089
MEDP2266	D-myo-Inositol-1,3,4	Organic acid And Its	Phosphoric acids	420.97
MEDP2265	2-Amino-3-phosphon	Organic acid And Its	Phosphoric acids	170.021
MEDN0588	Glucerol 3-phosphate	Organic acid And Its	Phosphoric acids	171.0058746
MEDN1239	5-O-(1-carboxyvinyl)	Organic acid And Its	Phosphoric acids	323.0284738
MADN0073	2-Aminoethanesulfor	Organic acid And Its	Sulfonic acids	124.0074
MADN0152	Methanesulfonic acid	Organic acid And Its	Sulfonic acids	94.9808
MADN0361	Isethionic acid	Organic acid And Its	Sulfonic acids	124.9911
MADN0072	2-Aminoethanesulfin	Organic acid And Its	Sulfonic acids	108.0125
MADN0508	Morpholine ethanesu	Organic acid And Its	Sulfonic acids	194.0489842
MADP0021	Betaine	Others	Others	118.1
MEDP1988	Hydroxyurea	Others	Others	123.0435

MEDP1076	Kojibiose	Others	Others	325.1112
MEDP2136	Abietic acid	Others	Others	303.2325511
MADP0454	SM(d18:1/20:0)	SL	SM	759.63
MADP0444	SM(d16:1/24:1(15Z))	SL	SM	785.65
MADP0452	SM(d18:1/22:0)	SL	SM	787.66
MADP0446	SM(d17:1/24:1(15Z))	SL	SM	799.66
MADP0470	SM(d18:1/18:0)	SL	SM	731.6
MADP0459	SM(d18:1/18:1(9Z))	SL	SM	729.58
MADP0433	SM(d18:1/16:0)	SL	SM	703.58
MEDP0871	Dihydro-D-sphingosi	SL	SM	302.5
MEDP1685	Phytosphingosine	SL	SM	318.30022
MEDP1051	Sphingosine	SL	SM	300.28885
MEDP1698	Sphingosyl-phosphoc	SL	SM	506.31171
MEDN1083	Sphingosine 1-phospl	SL	SM	378.2
MADP0284	Sn-Glycero-3-Phosph	Tryptamines,Cholin	Cholines	258.1103
MADP0192	Methacholine	Tryptamines,Cholin	Cholines	160.132
MEDP1255	Urobilin	Tryptamines,Cholin	Pigments	591.3
MEDP1045	Biliverdin	Tryptamines,Cholin	Pigments	583.2
MEDP0212	N-Acetyl-5-Hydroxyt	Tryptamines,Cholin	Tryptamines	219
MADP0080	Tryptamine	Tryptamines,Cholin	Tryptamines	161.1073

Table S9. Metabolites quantified by hepatic metabolome

Molecular Weight (Da)	Ionization model	Formula	Con
152.120115	[M+H]+	C10H16O	7388.5000
260.14	[M-H]-	C6H13O9P	5042633.3333
416.32903	[M+H]+	C27H44O3	103991.3333
179.079374	[M+H]+	C6H13NO5	38265.7500
147.22g	[M+H]+	C7H17NO2	369556.6667
238.67	[M-H]-	C11H11ClN2O2	2412100.0000
297.3031649	[M+Na]+	C19H39NO	26892.3333
287.44	[M+H]+	C16H33NO3	105520.3333
327.313729	[M+H]+	C20H41NO2	64114.0000
105.07898	[M+H]+	C4H11NO2	47982.1667
153.1	[M+H]+	C7H11N3O	############
187.28	[M+H]+	C9H21N3O	589858.3333
75.11	[M+H]+	C3H9NO	938329.6667
130.121846	[M+H]+	C5H14N4	699186.6667
211.084459	[M+H]+	C10H13NO4	111805.6667
169.07389	[M+H]+	C8H11NO3	1354951.6667
224.1889	[M+H]+	C13H24N2O	46100.8333
146.153146	[M+H]+	C6H18N4	510606.6667
145.1578976	[M+H]+	C7H19N3	1577550.0000
243.10413	[M+H]+	C10H17N3O2S	239151.6667
151.04153	[M+H]+	C3H9N3O2S	1324145.0000
154.1	[M+H]+	C4H11O4P	3171680.0000
361.261677	[M-H]-	C22H35NO3	63694.0333
147.05315	[M-H]-	C5H9NO4	323718.3333
276.09575	[M+H]+	C10H16N2O7	28466166.6667
169.014	[M+H]+	C3H8NO5P	53121.1667
217.1103	[M+H]+	C13H15NO2	207423.3333
175.0593	[M+H]+	C5H9N3O4	118223.8333

129.07843	[M+H]+	C6H11NO2	20947500.0000
145.0738932	[M+H]+	C6H11NO3	105402.3333
193.074	[M-H]-	C10H11NO3	7138.7000
193.073898	[M-H]-	C10H11NO3	7138.7000
117.07898	[M+H]+	C5H11NO2	70402.0000
264.11101	[M+H]+	C13H16N2O4	43461.1667
267.0776583	[M-H]-	C10H20N2O4	286248.5000
285.42	[M-H]-	C16H31NO3	94469.3333
174.064056	[M-H]-	C6H10N2O4	29137.8333
159.089544	[M-H]-	C7H13NO3	98682.0000
173.10519	[M+H]+	C8H15NO3	618911.6667
207.1	[M+H]+	C11H13NO3	648615.0000
262.131743	[M+H]+	C14H18N2O3	357211.6667
234.1579572	[M+H]+	C10H22N2O4	############
189.06372	[M-H]-	C7H11NO5	374756.6667
171.15	[M+H]+	C6H9N3O3	195135.0000
219.0742872	[M+H]+	C8H13NO6	100240.5000
105.09	[M+H]+	C3H7NO3	7888766.6667
188.11609	[M+H]+	C8H16N2O3	198774.3333
197.0800412	[M+H]+	C8H11N3O3	493040.0000
191.25	[M+H]+	C7H13NO3S	62069.6667
179.094629	[M+H]+	C10H13NO2	34632.1667
103.063332	[M+H]+	C4H9NO2	############
103.12	[M+H]+	C4H9NO2	############
223.08446	[M-H]-	C11H13NO4	79294.0000
117.078979	[2M+H]+	C5H11NO2	############
131.0582432	[M-H]-	C5H9NO3	149408.5000
131.0582	[M-H]-	C5H9NO3	139816.6667
117.04259	[M-H]-	C4H7NO3	109937.8333
327.13125	[M+H]+	C15H21NO7	25295.8333
216.24	[M+H]+	C8H16N4O3	201730.0000
137.084064	[M-H2O+H]+	C8H11NO	2640083.3333
207.089544	[M+H]+	C11H13NO3	305358.3333

226.10659	[M+H]+	C9H14N4O3	7144916.6667
115.13	[M+H]+	C5H9NO2	############
165.045964	[M+H]+	C5H11NO3S	960175.0000
238.09481	[M+H]+	C11H14N2O4	49606.3333
147.0531578	[M-H]-	C5H9NO4	8041150.0000
131.09463	[M+H]+	C6H13NO2	26643.1667
161.06881	[M+H]+	C6H11NO4	132985.5000
202.14243	[M+H]+	C8H18N4O2	############
385.26224	[M-H]-	C24H35NO3	4816.8150
155.06946	[M-H]-	C6H9N3O2	4562500.0000
175.04807	[M+H]+	C6H9NO5	135040.8333
189.17	[M-H]-	C7H11NO5	44309.1667
161.06826	[M+H]+	C6H11NO4	742091.6667
141.17	[M-H]-	C6H11N3O	19655.3333
205.0375077	[M+H]+	C10H7NO4	534158.3333
103.06333	[M-H]-	C4H9NO2	103045.3333
103.06333	[M+H]+	C4H9NO2	6871716.6667
188.23	[M+H]+	C7H16N4O2	############
174.10099	[M-H]-	C7H14N2O3	555981.6667
144.09207	[M]+	C7H14NO2+	2122216.6667
182.044	[M+H]+	C6H6N4O3	247588.3333
131.05824	[M+H]+	C5H9NO3	190035.0000
188.1525	[M+H]+	C9H20N2O2	10101400.0000
188.1160924	[M+H]+	C8H16N2O3	9222633.3333
163.0666997	[M+H]+	C6H13NO2S	524461.6667
143.0946287	[M+H]+	C7H13NO2	823348.3333
143.09464	[M+H]+	C7H13NO2	823348.3333
179.094629	[M-H]-	C10H13NO2	22733.7500
162.05283	[M-H]-	C6H10O5	607336.6667
375.2773	[M-H]-	C23H37NO3	10266.6117
182.044	[M+H]+	C6H6N4O3	92825.1667
117.078979	[M+H]+	C5H11NO2	2249266.6667
309.10598	[M-H]-	C11H19NO9	1193935.0000

217.14	[M+H]+	C9H19N3O3	98945.0000
188.11609	[M+H]+	C8H16N2O3	5275783.3333
188.07971	[M+H]+	C7H12N2O4	4469750.0000
246.100443	[M-H]-	C13H14N2O3	11950.1667
179.09462	[M+H]+	C10H13NO2	1092000.0000
321.09946	[M+H]+	C11H19N3O6S	564466.6667
131.05824	[M+H]+	C5H9NO3	11489716.6667
131.05769	[M+H]+	C5H9NO3	2264483.3333
195.0895433	[M+H]+	C10H13NO3	3056400.0000
153.01013	[M-H]-	C3H7NO4S	284225.0000
151.063329	[M+H]+	C8H9NO2	212857.0000
384.12159	[M+H]+	C14H20N6O5S	1716190.0000
103.06333	[M+H]+	C4H9NO2	10477783.3333
168.04226	[M-H]-	C8H8O4	40192.3333
169.08513	[M+H]+	C7H11N3O2	1794016.6667
203.1058621	[M+H]+	C11H13N3O	41930.5000
176.08026	[M-H]-	C6H12N2O4	487490.0000
189.043	[M+H]+	C10H7NO3	1653930.0000
208.085	[M+H]+	C10H12N2O3	3766283.3333
174.10044	[M+H]+	C7H14N2O3	965233.3333
276.132	[M-H]-	C11H20N2O6	251426.6667
174.136828	[M+H]+	C8H18N2O2	33859500.0000
129.04259	[M-H]-	C5H7NO3	172250.0000
103.02694	[M+H]+	C3H5NO3	############
89.04768	[M-H]-	C3H7NO2	7327650.0000
75.03203	[M-H]-	C2H5NO2	2937866.6667
89.04768	[M-H]-	C3H7NO2	7340166.6667
135.0354	[M+H]+	C4H9NO2S	2878533.3333
89.04768	[M+H]+	C3H7NO2	39325000.0000
115.06333	[M+H]+	C5H9NO	############
165.07898	[M+H]+	C9H11NO2	############
117.07898	[M+H]+	C5H11NO2	51308833.3333
131.09464	[M+H]+	C6H13NO2	18335500.0000

117.07898	[M+H]+	C5H11NO2	############
146.10553	[M+H]+	C6H14N2O2	############
131.09463	[M-H]-	C6H13NO2	74688666.6667
131.0946	[M+H]+	C6H13NO2	17207666.6667
105.04259	[M+H]+	C3H7NO3	70794500.0000
133.03751	[M+H]+	C4H7NO4	95669833.3333
146.06914	[M+H]+	C5H10N2O3	############
103.06333	[M-H]-	C4H9NO2	102872.8333
204.08988	[M-H]-	C11H12N2O2	1882383.3333
155.06948	[M+H]+	C6H9N3O2	56275666.6667
149.05105	[M+H]+	C5H11NO2S	21576500.0000
119.058244	[M+H]+	C4H9NO3	220471.6667
147.05316	[M-H]-	C5H9NO4	############
119.05824	[M+H]+	C4H9NO3	19778166.6667
132.05349	[M+H]+	C4H8N2O3	15541333.3333
175.09569	[M+H]+	C6H13N3O3	728218.3333
181.074	[M+H]+	C9H11NO3	36343333.3333
161.06881	[M-H]-	C6H11NO4	120404.0000
278.0936427	[M+H]+	C10H18N2O5S	1349013.3333
278.0936427	[M+H]+	C10H18N2O5S	1349013.3333
246.33	[M+H]+	C10H18N2O3S	1271076.6667
242.12666	[M+H]+	C11H18N2O4	55840.5000
226.1	[M-H]-	C9H14N4O3	139822.8333
172.08479	[M-H]-	C7H12N2O3	222914.3333
212.090941	[M+H-H2O]+	C8H12N4O3	27973.8333
275.26	[M-H]-	C10H17N3O6	89433.8333
284.33	[M+H]+	C12H16N2O4S	373718.3333
457.1431545	[M+H]+	C16H27NO14	259542.6667
202.21	[M+H]+	C8H14N2O4	57314.6667
229.25	[M-H]-	C9H15N3O4	19351.4667
258.08573	[M-H]-	C10H14N2O6	116452.6667
316.07287	[M-H]-	C12H16N2O6S	217820.0000
262.09818	[M+H]+	C10H18N2O4S	60116.1667

246.26	[M+H]+	C10H18N2O5	30127.6500
304.0906655	[M-H]-	C11H16N2O8	54012.3333
168.19	[M+H]+	C8H12N2O2	21037.1667
303.32	[M+H]+	C11H21N5O5	419726.6667
275.11173	[M+H]+	C10H17N3O6	2733683.3333
218.21	[M-H]-	C8H14N2O5	341075.0000
343.17	[M+H]+	C15H25N3O6	140977.8333
248.12001	[M-H]-	C10H20N2O3S	22656.3333
393.1899	[M+H]+	C19H27N3O6	50562.0000
252.111008	[M+H]+	C12H16N2O4	407025.0000
264.3	[M-H]-	C9H16N2O5S	1061963.3333
357.262757	[M+H]+	C18H35N3O4	61055.0000
246.1214904	[M+H]+	C10H18N2O5	1437003.3333
188.11664	[M-H]-	C8H16N2O3	331935.0000
619.8	[M+H]+	C29H45N7O6S	155728.1667
296.100838	[M+H]+	C13H16N2O6	261566.6667
293.137557	[M-H2O+H]+	C14H19N3O4	61240.1667
194.23	[M+H]+	C10H14N2O2	18997.8667
C17H23N3O6	[M+H]+	C17H23N3O6	35696.3333
245.13701	[M+H]+	C10H19N3O4	280720.0000
232.10647	[M-H]-	C9H16N2O5	40825.3333
189.17	[M-H]-	C6H11N3O4	107623.6667
190.15	[M-H]-	C6H10N2O5	16470.3833
426.0879051	[M-H]-	C13H22N4O8S2	2536833.3333
227.1033	[M+H]+	C10H14N2O4	389896.6667
278.3	[M+H]+	C14H18N2O4	694701.6667
172.084793	[M+H]+	C7H12N2O3	1257071.6667
246.26	[M-H]-	C10H18N2O5	69389.1667
260.137215	[M+H]+	C11H20N2O5	6315116.6667
202.21	[M+H]+	C8H14N2O4	933171.6667
293.14	[M-H]-	C14H19N3O4	10283.7000
252.122241	[M+H-H2O]+	C11H16N4O3	145411.6667
260.29	[M-H]-	C14H16N2O3	52559.5000

240.12224	[M+H]+	C10H16N4O3	12893066.6667
186.06461	[M-H]-	C7H10N2O4	74672.8333
379.1743	[M+H]+	C18H25N3O6	52010.3333
373.2213	[M+H]+	C17H31N3O6	120987.3333
247.22	[M-H]-	C8H13N3O6	131804.6667
243.15774	[M+H]+	C11H21N3O3	339831.6667
186.21	[M+H]+	C8H14N2O3	6309616.6667
285.168857	[M+H]+	C13H23N3O4	32109.8333
261.23	[M-H]-	C9H15N3O6	274596.6667
216.15	[M-H]-	C10H20N2O3	85212.3333
176.17	[M+H]+	C6H12N2O4	150952.8333
288.3	[M+H]+	C10H20N6O4	345590.0000
294.157958	[M+H]+	C15H22N2O4	154115.0000
260.29	[M-H]-	C10H20N4O4	32089.6667
246.12	[M+H]+	C10H18N2O5	102592.1667
321.17954	[M+H]+	C15H23N5O3	65216.5000
331.2107	[M+H-H2O]+	C15H29N3O5	143103.3333
331.210722	[M+H-H2O]+	C15H29N3O5	143103.3333
317.195	[M+H]+	C14H27N3O5	555505.0000
310.116488	[M+H]+	C14H18N2O6	3681133.3333
345.2264	[M+H]+	C16H31N3O5	96600.0000
244.24	[M+H]+	C10H16N2O5	12363800.0000
278.35	[M-H]-	C15H22N2O3	583131.6667
261.279	[M+H]+	C9H19N5O4	699410.0000
245.09544	[M+H]+	C10H18N2O3S	47734.5000
228.29	[M+H]+	C11H20N2O3	4179816.6667
304.13828	[M+H]+	C11H20N4O6	157731.0000
132.053493	[M+H]+	C4H8N2O3	315723.3333
303.36	[M+H]+	C16H21N3O3	80649.0000
275.3	[M-H]-	C14H17N3O3	21797.0000
287.36	[M+H]+	C12H25N5O3	610206.6667
203.2	[M-H]-	C7H13N3O4	177750.1667
259.35	[M+H]+	C12H25N3O3	2618850.0000

285.168857	[M+H]+	C13H23N3O4	35433.5000
301.20015	[M+H]+	C14H27N3O4	190761.6667
231.25	[M-H]-	C9H17N3O4	48579.6667
273.33	[M-H]-	C11H23N5O3	24101.6667
261.28	[M-H]-	C13H15N3O3	38198.6667
275.31	[M+H]+	C10H21N5O4	1140771.6667
260.29	[M+H]+	C11H20N2O5	1600061.6667
203.12644	[M+H]+	C8H17N3O3	753293.3333
305.4	[M+H]+	C11H23N5O3S	123200.0000
219.2	[M+H]+	C7H13N3O5	87848.1667
280.142308	[M+H]+	C14H20N2O4	744013.3333
315.2158	[M+H]+	C15H29N3O4	63006.8333
317.17	[M+H]+	C17H23N3O3	483191.6667
291.3	[M+H]+	C14H17N3O4	5095300.0000
259.189592	[M+H]+	C12H25N3O3	544786.6667
262.37	[M+H]+	C11H22N2O3S	1149070.0000
260.29	[M+H]+	C11H20N2O5	1393246.6667
266.1266571	[M+H]+	C13H18N2O4	92948.3333
333.1325	[M+H]+	C16H19N3O5	588061.6667
287.36	[M+H]+	C12H25N5O3	649448.3333
260.13722	[M+H]+	C11H20N2O5	1448365.0000
245.28	[M+H]+	C9H19N5O3	6237533.3333
244.31	[M-H]-	C11H20N2O4	77337.3333
612.15196	[M+H]+	C20H32N6O12S2	68862666.6667
204.07	[M+H]+	C7H12N2O5	443750.0000
246.26	[M+H]+	C10H18N2O5	628530.0000
328.4	[M+H]+	C18H20N2O4	534005.0000
248.34	[M-H]-	C10H20N2O3S	334956.6667
279.121906	[M+H]+	C13H17N3O4	126061.8333
301.2002	[M+H]+	C14H27N3O4	72434.5000
280.14286	[M-H]-	C14H20N2O4	12643.3333
259.35	[M+H]+	C12H25N3O3	253533.3333
244.33	[M+H]	C12H24N2O3	5853850.0000

305.33	[M+H]+	C15H19N3O4	284043.3333
244.33	[M+H]+	C12H24N2O3	5986283.3333
284.11152	[M+H]+	C11H16N4O5	16661500.0000
244.33	[M+H]+	C12H24N2O3	5671983.3333
244.33	[M+H]+	C12H24N2O3	5854650.0000
228.11046	[M+H]+	C10H16N2O4	1603915.0000
214.26	[M+H]+	C10H18N2O3	2188800.0000
217.10626	[M-H]-	C8H15N3O4	949416.6667
160.08479	[M-H]-	C6H12N2O3	153381.6667
245.14824	[M+H]+	C9H19N5O3	4279700.0000
264.1473925	[M+H]+	C14H20N2O3	437226.6667
275.31	[M+H]+	C10H21N5O4	2250316.6667
236.116093	[M+H]+	C12H16N2O3	5002050.0000
351.158292	[M+H]+	C20H21N3O3	154575.0000
335.18	[M-H]-	C17H25N3O4	19745.6667
278.163043	[M+H]+	C15H22N2O3	8675483.3333
279.1219	[M+H]+	C13H17N3O4	291616.6667
245.128	[M+H]+	C14H16N2O2	42300.0000
202.25	[M-H]-	C9H18N2O3	20566.8333
246.24	[M+H]+	C8H14N4O5	85725.1667
174.2	[M-H]-	C7H14N2O3	44064.6667
317.38	[M+H]+	C17H23N3O3	664725.0000
282.29	[M+H]+	C13H18N2O5	292165.0000
256.26	[M+H]+	C10H16N4O4	1730783.3333
230.3	[M+H]+	C11H22N2O3	14785833.3333
230.3	[M+H]+	C11H22N2O3	14115333.3333
174.1004	[M-H]-	C7H14N2O3	92136.6667
277.10908	[M+H]+	C10H19N3O4S	299385.0000
335.1845	[M+H]+	C17H25N3O4	75746.0000
317.38	[M+H]+	C17H23N3O3	724388.3333
218.12721	[M-H]-	C9H18N2O4	38504.0000
323.15	[M+H]+	C15H21N3O5	32423.1667
202.25	[M-H]-	C9H18N2O3	190155.0000

294.35	[M-H]-	C15H22N2O4	330260.0000
232.14	[M+H]+	C10H20N2O4	2337183.3333
204.22	[M+H]+	C8H16N2O4	2882683.3333
294.1216	[M+H]+	C14H18N2O5	2890150.0000
260.1585	[M+H]+	C20H23N3O4	43553.5000
230.3	[M-H]-	C11H22N2O3	200760.0000
204.074623	[M+H]+	C7H12N2O5	1330196.6667
294.35	[M-H]-	C15H22N2O4	318818.3333
245.32	[M+H]+	C11H23N3O3	2029183.3333
296.1194635	[M+H]+	C14H20N2O3S	314678.3333
268.31	[M-H]-	C12H20N4O3	300271.6667
202.13174	[M+H]+	C9H18N2O3	1627350.0000
232.14	[M+H]+	C10H20N2O4	2389466.6667
345.2264	[M+H]+	C16H31N3O5	71768.0000
222.09989	[M+H]+	C11H14N2O3	12077216.6667
287.36	[M+H]+	C12H25N5O3	2243916.6667
232.23	[M-H]-	C9H16N2O5	749851.6667
274.12827	[M-H]-	C10H18N4O5	223260.0000
289.13807	[M+H]+	C10H19N5O5	5596600.0000
230.1630426	[M-H]-	C11H22N2O3	235333.3333
293.17339	[M+H]+	C15H23N3O3	111688.3333
233.13701	[M+H]+	C9H19N3O4	1437526.6667
522.3567	[M+H]+	C12H16N2O4	3596150.0000
188.116093	[M+H]+	C8H16N2O3	2153666.6667
216.11046	[M+H]+	C9H16N2O4	13894816.6667
277.14547	[M+H]+	C11H23N3O3S	37666.1667
203.090607	[M+H]+	C7H13N3O4	634940.0000
245.32	[M-H]-	C11H23N3O3	557840.0000
232.14	[M+H]+	C10H20N2O4	630843.3333
234.21	[M+H]+	C8H14N2O6	301648.3333
302.37	[M+H]+	C12H26N6O3	1632716.6667
245.28	[M-H]-	C10H19N3O4	458000.0000
188.22	[M-H]-	C8H16N2O3	52724.0000

261.14316	[M+H]+	C9H19N5O4	2239100.0000
258.08519	[M+H]+	C10H14N2O6	138950.3333
330.39	[M+H]+	C12H26N8O3	152683.1667
231.25	[M-H]-	C9H17N3O4	183885.0000
218.25	[M+H]+	C9H18N2O4	481538.3333
295.29	[M+H]+	C13H17N3O5	98281.6667
273.33	[M+H]+	C11H23N5O3	2346200.0000
232.28	[M+H]+	C10H20N2O4	661535.0000
233.1489	[M+H]+	C9H18N2O4	1876083.3333
206.2	[M+H]+	C7H14N2O5	328866.6667
234.08	[M+H]+	C8H14N2O6	392905.0000
192.17	[M+H]+	C6H12N2O5	504466.6667
259.15266	[M+H]+	C11H21N3O4	1160548.3333
233.10062	[M+H]+	C8H15N3O5	267528.3333
248.23	[M+H]+	C9H16N2O6	682791.6667
204.07407	[M+H]+	C7H12N2O5	1330641.6667
307.084	[M+H]+	C10H17N3O6S	4411233.3333
268.23	[M-H]-	C13H8N4O3	248788.3333
368.5	[M+H]+	C19H32N2O5	6308.8833
154.16	[M-H]-	C8H10O3	4079.5867
450.61	[M+H]+	C29H38O4	7025.4333
164.24	[M+H]+	C11H16O	24231.3333
170.0579088	[M-H]-	C8H10O4	13874.7667
256.25	[M-H]-	C15H12O4	17704.6500
122.037	[M+H]+	C7H6O2	111997.3333
218.22	[M+H]+	C9H10N6O	404323.3333
254.057909	[M-H]-	C15H10O4	8138.5500
136.08882	[M+H]+	C9H12O	1437133.3333
124.0524	[M-H]-	C7H8O2	43874.5000
208.019415	[M-H]-	C10H8O3S	682460.0000
236.17818	[M-H]-	C15H24O2	548160.0000
138.0680796	[M-H]-	C8H10O2	153815.0000
144.068748	[M+H]+	C9H8N2	930003.3333

106.0418648	[M+H]+	C7H6O	11469416.6667
137	[M-H2O+H]+	C8H11NO	2640083.3333
362.090273	[M+H]+	C20H14N2O5	42552.8333
127.57	[M+H]+	C6H6ClN	95526.8333
143.0735	[M+H]+	C10H9N	707308.3333
278.34	[M+H]+	C16H22O4	32748.1667
238.062995	[M-H]-	C15H10O3	28267.5000
135.104799	[M+H]+	C9H13N	268320.0000
300.26	[M+H]+	C16H12O6	23975.0000
250.29	[M-H]-	C14H18O4	12292.0333
152.058578	[M+H]+	C7H8N2O2	9615350.0000
123.06787	[M+H]+	C7H9NO	936466.6667
216.1514	[M+H]+	C15H20O	76197.1667
174.008197	[M-H]-	C6H7O4P	82716.5000
205.073894	[M+H]+	C11H11NO3	1300950.0000
788.85357	[M+H]+	C18H22I3N3O8	167301.6667
184.07411	[M-H]-	C9H12O4	24883.7333
136.15	[M+H]+	C7H8N2O	738786.6667
354.4	[M+H]+	C20H22N2O4	60722.3333
391.5	[M+H]+	C23H37NO4	12516.9500
206.23776	[M+H]+	C12H14O3	25621.3000
166.062995	[M-H]-	C9H10O3	15368.5500
166.027	[M-H]-	C8H6O4	177553.3333
152.0362	[M-H]-	C7H7NO3	90520.3333
166.062995	[M-H]-	C9H10O3	40968666.6667
166.027	[M-H]-	C8H6O4	191415.0000
153.042594	[M-H]-	C7H7NO3	4076300.0000
187.0633	[M-H2O+H]+	C11H9NO2	422756.6667
168.042	[M-H]-	C8H8O4	20390.2667
178.09937	[M+H]+	C11H14O2	66040.3333
182.05791	[M-H]-	C9H10O4	13932.5283
167.0946287	[M+H]+	C9H13NO2	47780.6667
138.0681	[M+H]+	C8H10O2	302521.6667

392.293	[M-H]-	C24H40O4	1761006.6667
392.293	[M-H]-	C24H40O4	1917981.6667
392.29266	[M-H]-	C24H40O4	32100.2667
408.2875744	[M-H]-	C24H40O5	1046306.6667
499.2968	[M-H]-	C26H45NO6S	1024295.1667
499.29621	[M-H]-	C26H45NO6S	1024295.1667
408.2876	[M-H]-	C24H40O5	1111571.8333
499.297	[M-H]-	C26H45NO6S	1128736.1667
408.2876	[M-H]-	C24H40O5	703426.6667
390.277	[M-H]-	C24H38O4	114919.3333
390.277	[M-H]-	C24H38O4	114919.3333
390.277	[M-H]-	C24H38O4	114919.3333
515.291674	[M-H-H2O]-	C26H45NO7S	405054.6667
515.2917	[M-H-H2O]-	C26H45NO7S	405054.6667
408.2876	[M-H]-	C24H40O5	388186.1667
254.23	[M-H]-	C9H18O8	1081739.6667
178.14	[M-H]-	C6H10O6	1012341.6667
180.16	[M-H]-	C6H12O6	3180983.3333
182.07904	[M-H]-	C6H14O6	1375783.3333
339.99605	[M-H]-	C6H14O12P2	34364.3333
277.995655	[M-H]-	C5H12O9P2	673670.0000
260.029722	[M-H]-	C6H13O9P	8219166.6667
422.082547	[M-H]-	C12H23O14P	27734.1667
230.0191538	[M-H]-	C5H11O8P	5915466.6667
230.0191538	[M-H]-	C5H11O8P	8465550.0000
230.0191538	[M-H]-	C5H11O8P	8465550.0000
230.0191538	[M-H]-	C5H11O8P	8465550.0000
301.0562676	[M-H]-	C8H16NO9P	5101083.3333
272.01715	[M-H]-	C6H11Na2O9P	110678.3333
214.0242392	[M-H]-	C5H11O7P	2203896.6667
425.9299	[M-Ba]-	C7H13BaO10P	131697.8333
260.02972	[M-H]-	C6H13O9P	10069400.0000
179.079374	[M+H]+	C6H13NO5	40237.7000

210.0375673	[M-H]-	C6H10O8	109720.1667
210.0375673	[M-H]-	C6H10O8	109720.1667
178.04774	[M-H]-	C6H10O6	502591.6667
194.0432	[M-H]-	C6H10O7	332711.6667
178.04774	[M-H]-	C6H10O6	1265786.6667
194.042655	[M-H]-	C6H10O7	443956.6667
194.043	[M-H]-	C6H10O7	1720316.6667
290.122636	[M-H]-	C10H18N4O6	111045.5000
122.05791	[M-H]-	C4H10O4	19885.8333
182.07904	[M-H]-	C6H14O6	967275.0000
152.06848	[M-H]-	C5H12O5	7419316.6667
164.06848	[M-H]-	C6H12O5	179163.3333
182.07904	[M-H]-	C6H14O6	1445003.3333
182.07904	[M-H]-	C6H14O6	937840.0000
152.06848	[M-H]-	C5H12O5	7275766.6667
180.06339	[M-H]-	C6H12O6	22101166.6667
342.116215	[M-H]-	C12H22O11	87154.3333
246.12154	[M+H]+	C10H18N2O5	1448546.6667
194.07904	[M-H]-	C7H14O6	154114.5500
210.073	[M-H]-	C7H14O7	96800.6667
504.4	[M-H]-	C18H32O16	24527.2000
504.4	[M-H]-	C18H32O16	24527.2000
120.0422587	[M-H]-	C4H8O4	7254.7500
164.06847	[M-H]-	C6H12O5	184208.3333
633.2116281	[M-H]-	C23H39NO19	85969.1667
342.11622	[M-H]-	C12H22O11	36959.2333
504.16904	[M-H]-	C18H32O16	146629.8333
342.11622	[M-H]-	C12H22O11	30919.1667
342.11621	[M+H]+	C12H22O11	114484.0000
342.116215	[M-H]-	C12H22O11	62752.1667
122.057909	[M-H]-	C4H10O4	1340010.0000
180.16	[M-H]-	C6H12O6	3180983.3333
180.16	[M-H]-	C6H12O6	3180983.3333

164.06848	[M-H]-	C6H12O5	154330.0000
342.1162115	[M+K]+	C12H22O11	144763.8333
342.11622	[M-H]-	C12H22O11	106719.3333
118.027	[M-H]-	C4H6O4	2455733.3333
138.043	[M+H]+	C6H6N2O2	261853.3333
169.074	[M+H]+	C8H11NO3	71871.3333
300.209	[M+H]+	C20H28O2	55591.0833
376.138	[M-H]-	C17H20N4O6	593631.6667
219.11067	[M-H]-	C9H17NO5	13101566.6667
123.03148	[M+H]+	C6H5NO2	657188.3333
137.048	[M+H]+	C7H7NO2	3412523.3333
265.1123	[M]+	C12H17N4OS+	62786833.3333
205.131	[M-H]-	C9H19NO4	12690166.6667
176.0321	[M-H]-	C6H8O6	178745.0000
278.13	[M+H]+	C11H22N2O4S	93862500.0000
286.23	[M+H]+	C20H30O	1307613.3333
122.1	[M+H]+	C6H6N2O	############
456.104618	[M-H]-	C17H21N4O9P	120068.0000
312.20893	[M+H]+	C21H28O2	219343.6667
284.2140155	[M+H]+	C20H28O	144248.6667
244.088	[M+H]+	C10H16N2O3S	1933800.0000
214.1317425	[M+H]+	C10H18N2O3	66969.1667
744.4	[M]+	C21H29N7O17P3+	5164150.0000
250.1205091	[M+H]+	C14H18O4	67135.1667
385.319209	[M+H]+	C22H43NO4	9.0000
301.225309	[M+H]+	C16H31NO4	10447.6483
337.225309	[M+H]+	C19H31NO4	26343.9667
357.287909	[M+H]+	C20H39NO4	42384.4017
383.303559	[M+H]+	C22H41NO4	11591.3333
413.350509	[M+H]+	C24H47NO4	28627.5833
369.287909	[M+H]+	C21H39NO4	552014.6667
469.376724	[M+H]+	C27H51NO5	42628.5833
329.256609	[M+H]+	C18H35NO4	14706.6333

273.194009	[M+H]+	C14H27NO4	16027.9333
385.282824	[M+H]+	C21H39NO5	79923.6667
425.350509	[M+H]+	C25H47NO4	9759316.6667
397.319209	[M+H]+	C23H43NO4	1382330.0000
371.303559	[M+H]+	C21H41NO4	773300.0000
341.256609	[M+H]+	C19H35NO4	92221.6667
397.246439	[M+H]+	C21H35NO6	1379116.6667
315.240959	[M+H]+	C17H33NO4	260419.0000
479.397459	[M+H]+	C29H53NO4	376380.0000
415.329774	[M+H]+	C23H45NO5	160891.1667
437.314124	[M+H]+	C25H43NO5	20867.4333
441.345424	[M+H]+	C25H47NO5	169768.6667
399.334859	[M+H]+	C23H45NO4	3329723.3333
343.272259	[M+H]+	C19H37NO4	29119.3000
287.209659	[M+H]+	C15H29NO4	107124.8333
367.272259	[M+H]+	C21H37NO4	104030.1667
243.147059	[M+H]+	C12H21NO4	116533.5000
161.105	[M+H]+	C7H15NO3	67668333.3333
437.350509	[M+H]+	C26H47NO4	1586366.6667
443.361074	[M+H]+	C25H49NO5	370774.1667
421.319209	[M+H]+	C25H43NO4	66592.3167
259.178359	[M+H]+	C13H25NO4	397758.1667
423.334859	[M+H]+	C25H45NO4	3221058.3333
411.298474	[M+H]+	C23H41NO5	31236.4500
265.131409	[M+H]+	C14H19NO4	27017.3333
395.303559	[M+H]+	C23H41NO4	115939.0000
261.121239	[M+H]+	C11H19NO6	1443661.6667
439.329774	[M+H]+	C25H45NO5	60679.5000
329.220224	[M+H]+	C17H31NO5	96542.0000
285.194009	[M+H]+	C15H27NO4	46136.0333
303.204574	[M+H]+	C15H29NO5	175566.3333
203.1158	[M+H]+	C9H17NO4	############
231.1471	[M+H]+	C11H21NO4	46693333.3333

245.162709	[M+H]+	C12H23NO4	3930636.6667
245.162709	[M+H]+	C12H23NO4	3930636.6667
231.147059	[M+H]+	C11H21NO4	5136235.0000
274.19273	[M+H]+	C18H26O2	12006.3667
300.2664	[M-H]-	C18H36O3	5938.3833
228.37	[M-H]-	C14H28O2	3033.2750
308.27	[M-H]-	C20H36O2	9847.2500
254.22513	[M-H]-	C16H30O2	55734000.0000
300.266445	[M-H]-	C18H36O3	12120.1500
200.17	[M-H]-	C12H24O2	390190.0000
310.2871805	[M+H]+	C20H38O2	3611.9333
272.2351449	[M-H]-	C16H32O3	311940.0000
272.2351	[M-H]-	C16H32O3	311940.0000
278.4	[M-H]-	C18H30O2	96866.5000
280.24	[M-H]-	C18H32O2	6067766.6667
256.24	[M-H]-	C16H32O2	22111166.6667
276.208921	[M-H]-	C18H28O2	32198.9000
328.2402	[M-H]-	C22H32O2	2326800.0000
280.4	[M+H]+	C18H32O2	27926.0333
336.2300595	[M-H]-	C20H32O4	3016916.6667
304.2402303	[M-H]-	C20H32O2	123148.3333
278.22	[M-H]-	C18H30O2	27020.1667
240.2089	[M-H]-	C15H28O2	114878.3333
306.25	[M-H]-	C20H34O2	6969.5500
316.5	[M-H]-	C18H36O4	252815.0000
244.16746	[M-H]-	C13H24O4	241658.3333
258.18311	[M-H]-	C14H26O4	372823.8333
286.2144094	[M-H]-	C16H30O4	1610033.3333
294.2194948	[M-H]-	C18H30O3	24520.8500
294.2194948	[M-H]-	C18H30O3	24520.8500
318.2194948	[M-H]-	C20H30O3	4107.7500
330.240624	[M-H]-	C18H34O5	168106.0000
296.2351449	[M-H]-	C18H32O3	143346.8333

324.266445	[M-H]-	C20H36O3	3325.7867
324.266445	[M-H]-	C20H36O3	3325.7867
320.2351	[M-H]-	C20H32O3	14566.0333
320.2351449	[M-H]-	C20H32O3	13616.1833
320.2351449	[M-H]-	C20H32O3	13616.1833
266.188182	[M-H]-	C16H26O3	2798350.0000
296.2351449	[M-H]-	C18H32O3	9932.9833
320.2351449	[M-H]-	C20H32O3	13283.7833
294.1	[M-H]-	C18H30O3	3689.4867
322.2508	[M-H]-	C20H34O3	23520.8167
322.250795	[M-H]-	C20H34O3	23520.8167
322.250795	[M-H]-	C20H34O3	23520.8167
320.2351449	[M-H]-	C20H32O3	49672.6667
298.250795	[M-H]-	C18H34O3	123506.0000
328.2402	[M-H]-	C22H32O2	2326800.0000
302.22	[M-H]-	C20H30O2	174114.6667
304.2402	[M-H]-	C20H32O2	1721716.6667
296.2351449	[M-H]-	C18H32O3	7731.6167
296.2351449	[M-H]-	C18H32O3	7731.6167
362.24571	[M-H]-	C22H34O4	6891.8000
314.2457	[M-H]-	C18H34O4	2086945.0000
314.2457	[M-H]-	C18H34O4	2086945.0000
334.214409	[M-H]-	C20H30O4	25822.8500
362.24571	[M-H]-	C22H34O4	12185.3667
338.24571	[M-H]-	C20H34O4	193032.1667
338.24571	[M-H]-	C20H34O4	193032.1667
338.2457096	[M-H]-	C20H34O4	142479.8333
302.36	[M+H]+	C15H26O6	572258.6667
410.243342	[M-H]-	C19H39O7P	336491.6667
410.2433401	[M-H]-	C19H39O7P	336491.6667
436.2589903	[M-H]-	C21H41O7P	379716.6667
541.316841	[M+H]+	C28H48NO7P	108352.5000
541.316841	[M+H]+	C28H48NO7P	108352.5000

515.301192	[M+H]+	C26H46NO7P	18428.7167
549.379441	[M+H]+	C28H56NO7P	2771166.6667
495.332491	[M+H]+	C24H50NO7P	#############
549.379441	[M+H]+	C28H56NO7P	1435968.3333
495.332491	[M+H]+	C24H50NO7P	#############
521.348141	[M+H]+	C26H52NO7P	#############
438.274642	[M-H]-	C21H43O7P	166299.1667
543.332491	[M+H]+	C28H50NO7P	#############
543.332491	[M+H]+	C28H50NO7P	#############
569.348141	[M+H]+	C30H52NO7P	7134566.6667
184.0739	[M+H]+	C5H15NO4P+	#############
509.348141	[M+H]+	C25H52NO7P	59938500.0000
467.301191	[M+H]+	C22H46NO7P	1456085.0000
509.3481	[M+H]+	C25H52NO7P	#############
491.30119	[M+H]+	C24H46NO7P	93049.1667
523.363791	[M+H]+	C26H54NO7P	#############
523.363791	[M+H]+	C26H54NO7P	#############
439.269891	[M+H]+	C20H42NO7P	32065.5000
481.316841	[M+H]+	C23H48NO7P	2194766.6667
493.316841	[M+H]+	C24H48NO7P	27062350.0000
509.384526	[M+H]+	C26H56NO6P	1178631.6667
509.384526	[M+H]+	C26H56NO6P	1178631.6667
439.269892	[M+H]+	C20H42NO7P	75212.8333
507.368876	[M+H]+	C26H54NO6P	9910616.6667
523.363791	[M+H]+	C26H54NO7P	49095000.0000
465.285542	[M+H]+	C22H44NO7P	312123.3333
521.348141	[M+H]+	C26H52NO7P	#############
493.316841	[M+H]+	C24H48NO7P	3234466.6667
545.348141	[M+H]+	C28H52NO7P	6136350.0000
545.348141	[M+H]+	C28H52NO7P	6136350.0000
517.316841	[M+H]+	C26H48NO7P	294708.3333
569.348141	[M+H]+	C30H52NO7P	31331500.0000
519.332491	[M+H]+	C26H50NO7P	#############

519.332491	[M+H]+	C26H50NO7P	############
481.3168	[M+H]+	C23H48NO7P	928076.6667
547.363791	[M+H]+	C28H54NO7P	2694283.3333
547.363791	[M+H]+	C28H54NO7P	2694283.3333
517.316841	[M+H]+	C26H48NO7P	100242.6667
571.363791	[M+H]+	C30H54NO7P	5629183.3333
571.363791	[M+H]+	C30H54NO7P	5629183.3333
479.3376	[M+H]+	C24H50NO6P	1222605.0000
501.285	[M-H]-	C25H44NO7P	26402166.6667
501.285541	[M-H]-	C25H44NO7P	27184500.0000
425.254241	[M-H]-	C19H40NO7P	120792.8333
465.2855393	[M+H]+	C22H44NO7P	227106.6667
479.301191	[M-H]-	C23H46NO7P	25448500.0000
451.269891	[M-H]-	C21H42NO7P	2026041.6667
503.301191	[M-H]-	C25H46NO7P	445281.6667
503.301191	[M-H]-	C25H46NO7P	445281.6667
453.285541	[M-H]-	C21H44NO7P	49066500.0000
453.285541	[M-H]-	C21H44NO7P	49066500.0000
527.301191	[M-H]-	C27H46NO7P	4449733.3333
527.301191	[M-H]-	C27H46NO7P	4449733.3333
437.6	[M-H]-	C21H44NO6P	64643.3333
505.316841	[M-H]-	C25H48NO7P	405675.0000
505.316841	[M-H]-	C25H48NO7P	405675.0000
451.269891	[M+H]+	C21H42NO7P	130945.5000
529.316841	[M-H]-	C27H48NO7P	1360991.6667
529.316841	[M-H]-	C27H48NO7P	1360991.6667
499.269891	[M-H]-	C25H42NO7P	83961.0000
499.269891	[M-H]-	C25H42NO7P	83961.0000
525.285541	[M-H]-	C27H44NO7P	3783350.0000
525.285541	[M-H]-	C27H44NO7P	3783350.0000
475.269891	[M-H]-	C23H42NO7P	282876.6667
475.269891	[M-H]-	C23H42NO7P	282876.6667
477.285541	[M+H]+	C23H44NO7P	73658333.3333

477.285541	[M+H]+	C23H44NO7P	73658333.3333
481.316841	[M-H]-	C23H48NO7P	4745400.0000
481.316841	[M-H]-	C23H48NO7P	4745400.0000
510.295772	[M-H]-	C24H47O9P	39164.6667
569.275371	[M-H]-	C28H44NO9P	71866.3333
607.45769	[M+H]+	C32H66NO7P	85019.3333
741.56724	[M+H]+	C42H80NO7P	49730.8333
757.56216	[M+CH3COOH-H]-	C42H80NO8P	6365.1000
837.62476	[M+H]+	C48H88NO8P	21972.0667
785.59346	[M+H]+	C44H84NO8P	260565.5000
833.59346	[M+H]+	C48H84NO8P	24042.1000
757.56216	[M+H]+	C42H80NO8P	6724750.0000
781.56216	[M+H]+	C44H80NO8P	1632658.3333
859.60911	[M+H]+	C50H86NO8P	4940.6667
819.61419	[M+H]+	C48H86NO7P	22805.7500
733.56216	[M+H]+	C40H80NO8P	137777.6667
805.56216	[M+H]+	C46H80NO8P	941988.3333
787.60911	[M+Na]+	C44H86NO8P	133702.5000
793.59854	[M+H]+	C46H84NO7P	30780.0833
799.60911	[M+H]+	C45H86NO8P	172379.0000
705.53086	[M+H]+	C38H76NO8P	27709.7500
755.54651	[M+H]+	C42H78NO8P	10268400.0000
729.53086	[M+H]+	C40H76NO8P	2719470.0000
565.374356	[M-H2O+H]+	C28H56NO8P	735698.3333
509.311756	[M+H]+	C24H48NO8P	140875.0000
765.56724	[M+H]+	C44H80NO7P	48760.3333
769.59854	[M+Na]+	C44H84NO7P	47880.1667
495.368876	[M+H]+	C25H54NO6P	############
509.384526	[M+H]+	C26H56NO6P	1178631.6667
481.353226	[M+H]+	C24H52NO6P	20772500.0000
823.64549	[M+H]+	C48H90NO7P	69561.1667
793.59854	[M+H]+	C46H84NO7P	38005.8333
763.51521	[M-H]-	C43H74NO8P	3552.3650

739.51521	[M-H]-	C41H74NO8P	16741.7500
715.51521	[M+H]+	C39H74NO8P	72604.0000
141.0191	[M+H]+	C2H8NO4P	4156283.3333
771.57781	[M-H]-	C43H82NO8P	142500.8333
834.52583	[M-H]-	C43H79O13P	10607.2383
862.55713	[M-H]-	C45H83O13P	167974.6667
791.56764	[M-H]-	C42H82NO10P	11606.5633
[truncated: 90,381 more chars]
