# Supplementary material for: Mechanisms of hepatic steatosis in chickens: integrated analysis of the host genome, molecular phenomics and gut microbiome
Source: Gigascience. 2024 Jun 5;13:giae023. doi: 10.1093/gigascience/giae023 (PMC11152177; doi:10.1093/gigascience/giae023)
Supplement: giae023_Response_to_Reviewer_Comments_Original_Submission [file giae023_response_to_reviewer_comments_original_submission.pdf]

Author's Response To Reviewer Comments

Dear Editor,
We are grateful to you and reviewers for your valuable comments and suggestions on our manuscript (GIGA-D-23-00122). We have made all necessary revisions to improve the quality of our manuscript.
In the present study, we mainly focus on the specific mechanism of host genetics and gut microbiota in the regulation of hepatic steatosis, and further identify three molecular regulation routes and reported the microbiability of hepatic steatosis for the first time. Specific microbial taxa whose abundances are significantly associated with hepatic steatosis are identified.
In the revised manuscript, we have changed these words like “plasma”, “Con” and “corrected” to “serum”, “Ctrl”, and “adjusted”. At the introduction section, we added several cited references to the heritability of hepatic steatosis, supplemented a more detailed interpretation regarding “gut-liver axis”, and underscored the importance and rational of choosing chicken as research model. At materials and methods section, we have added information on the diets and the classification of hepatic steatosis. Detailed description of eQTL mapping, SMR, microbiability estimation and LEfSe analysis have been provided. With respect to the results section, we have supplemented the beta value of Wilcoxon rank-sum test and the characterization of gut microbiota in diverse segments. Besides, we have rearranged the paragraph of three molecular regulation routes for hepatic steatosis and modified the content of the compensatory roles of Lactobacillus in alleviating hepatic steatosis. The discussion section have been considerably modified with assistance from experts in animal genetics and nutrition. Meanwhile, all Figures and Tables, including supplementary files, were double-checked. In addition, the modified manuscript was further revised by a professional scientific editing service, Edanz China, in the aspect of English grammar, usage, and overall readability. The modifications are highlighted in the revised manuscript. The responses to the comments are listed below.
We believe that we have carefully addressed all the comments and hope that the modifications meet with your approval.
Thank you again and we look forward to hearing from you soon.
Sincerely,
Ning Yang, Ph.D.
Professor of Poultry Genetics and Breeding
China Agricultural University
Beijing 100193, China
Tel: +86-10-6273 1351
E-mail: nyang@cau.edu.cn; cjsun@cau.edu.cn

Response to comments from Anonymous Referee #1
Introduction
1.1.Introduction: The Introduction is lengthy but lacks cited references, and specific and important details related to research on host genetics, gut microbiota, and hepatic steatosis. More words are needed to underscore the importance as well as the rationale for choosing farm chicken as research model. Thus, I suggest rewriting and shortening the introduction section to include necessary references, provide more detailed information, and offer a clear rationale for conducting the study.
A1.1: We have revised the introduction section to emphasize the significance and rationale behind selecting the chicken as a research model. Unnecessary descriptions have been removed, and essential references have been added. See lines 86-89.
1.2.Example 1: Line 43-44 "A fatty liver is strongly influenced by host genetics with heritability ranging from 0.38 to 0.70." The statement on heritability lacks a cited reference and lacks information on the studied hosts (e.g., human, mice, or chicken).
A1.2: We have included additional details, supplemented the cited references, and added information about the host. See lines 43-45.
1.3.Example 2: Line 44-46, lack of references of the two mentioned genes (PNPLA3 and TM6SF2).
A1.3: The relevant references for PNPLA3 and TM6SF2 have been added. See line 47.
1.4.Example 3: line 55-57 The description of the “gut-liver axis” needs more depth, as this is much more

than the basic description you have provided “gut microbiota-derived products directly to the liver and the liver feedback route of metabolites to the gut.”

A1.4: To address the reviewer’s concerns clearly, we have enhanced the interpretation of the “gut-liver axis”. The liver and intestines bi-directionally communicate through three pathways: the bile duct, portal vein, and systemic circulation, collectively forming the “gut-liver axis”. Additional details regarding the “gut-liver axis” have been provided. See lines 56-61 and 64-73.

1.5.Example 4: line 57-59 “Gut microbiota ferment indigestible carbohydrates and proteins, thereby yielding metabolites that play a crucial role in gut homeostasis and liver substrate metabolism and functions [5-7].” The sentence provided very general information, the roles of gut microbiota are vague and lacks specificity. E.g., “what were the specific liver substrate metabolism and functions?”

A1.5: We have included a comprehensive interpretation of the impact of gut microbiota on various aspects, including gut microbial ecology, host digestion and absorption, and metabolic regulation. See lines 56-61 and 64-73.

1.6.Example 5: Line 62, the term "chicken lipid metabolism regulation model" as well as the “regulation model” mentioned in below sections require clarification. What is a regulation model?

A1.6: We apologize for inadequately clarifying this point, and relevant details have now been provided in the revised manuscript. The term “regulation model” implied that chickens were utilized as model animals to investigate the regulatory mechanisms of hepatic steatosis. Chickens have been employed as an animal model organisms in various research areas due to their biological characteristics, ease of breeding, and resemblance to other vertebrates, including humans. Additional supplements have been included in the revised manuscript. See lines 77-80.

1.7.Example 6: line 70-73. Add references to support the claim that farm animals have more genetic variations than mouse models. “patricianly suitable” should be “particularly suitable”.

A1.7: The relevant references have been added and this mistake has been rectified. See lines 86-89 and 91.

Materials and Methods

1.8.Please provide more details on experimental design: “Birds were generated from two hatches and reared in individual cages under similar conditions.”

A1.8: Additional information related to this statement has been added in the revised manuscript. See lines 482-484.

1.9.Please provide more details on the "basic diet" mentioned in the experimental design. Specify whether the diet is corn-based, wheat-based, or another type.

A1.9: The details of the diet have been outlined in Table 2. See lines 484 and 486-489.

1.10.Clarify how the control (Con, N = 217, healthy), mild hepatic steatosis (HS-Ⅰ, N = 265), and severe hepatic steatosis (HS-Ⅱ, N = 204) groups were defined.

A1.10: Additional details regarding the classification of hepatic steatosis have been provided. Individuals were classified into three categories based on the degree of fatty liver: lipid droplets constituting less than 10% of the area in the control group (Ctrl), 10% to 50% in the mild–moderate group (HS-I), and more than 50% in the severe group (HS-II). These relevant details have been included in the revised manuscript. See lines 517-521.

1.11.eQTL mapping and mendelian randomization analysis: Line 599-600:

The instrumental variables in the mendelian randomization analysis (MR) are very important and they must be independent of each other. Please explain how instrumental variables for summary data-based MR analysis were defined, ensuring their independence from each other. Describe how these variables were identified as independently associated with the exposure factor.

A1.11: Figure 6A presents potential causal relationships among SNPs, mRNA) and phenotypes. Differential gene expression serves as the casual link between an eQTL and the associated phenotype. The top eQTL-based SMR analysis employs SNPs as instrumental variables to assess causal associations between a risk factor (gene expression levels) and an outcome (HSC). Large-scale eQTL mapping was conducted to identify SNPs associated with gene expression levels. Among the identified eQTLs, those exhibiting the most significant associations based on statistical criteria (top eQTLs) were selected. Ensuring independence of instrumental variables from each other is essential to fulfill the assumptions of Mendelian Randomization. Assessment of the degree of linkage disequilibrium (LD) between SNPs is crucial, with the removal of SNPs in high LD with the top associated eQTL. The SMR incorporates instrumental variable estimation to effectively integrate independent GWAS and eQTL summary data, as depicted in Figure 6B. We have expanded this section, please see lines 624-638.

1.12.Heritability and microbiability estimation: Line 626

Only the batches and the first five host genetic principal components were corrected when estimating the phenotypic variance explained by the gut microbiota (microbiability estimation). However, gut microbial composition could also be partially influenced by host genetics. In human studies, PRS or SNPs that associated with the investigated phenotype are also considered as covariates when estimating microbiability (doi:10.1038/nature25973). Please provide some description to explain this.

A1.12: The SNPs that had significant or suggestive effects on HSC were identified, pruned for linkage disequilibrium (LD), and then the independent SNPs were selected to recalculate the PCs using PLINK. The first four PCs, the top five host genetic PCs, and the batches were considered as covariates in the model, as described by Wen et al (Wen et al., 2019; Wen et al., 2021). Subsequently, the estimates of microbiability were 0.25 for the duodenum, 0.00 for the jejunum, 0.07 for the ileum, 0.02 for the cecum, and 0.09 for the feces, similar to our previously outcomes. As there were no notable disparities between these two sets of results and for practicality of concurrently estimating microbiability for all recorded phenotypes, we consistently included the batches and the first five host genetic PCs in our comprehensive analysis. Please see lines 685-694.

1.13.Widely targeted metabolome: Line 682

Specify the number of liver tissue samples subjected to targeted metabolome.

A1.13: The specific number of the liver samples in this aspect has been classified. See lines 713-714.

1.14.Statistical Analysis: line712-731

Given the non-normally distributed nature of microbiota abundance profiles, clarify whether any data transformations were performed before using ANOVA to determine differences in gut microbiota.

A1.14: We made a mistake here and have rectified it. ANOVA was only used to a determine the differences in hepatic gene expression levels and biochemical indicators, not microbial abundance. See line 765.

1.15.Explain the rationale for using Spearman's rank correlation for taxa with occurrence between 30% to 60% but Pearson correlation coefficient for those with occurrence ≥ 60% of samples. Provide a clear explain for this differentiation on correlation methods.

A1.15: The selection between Spearman’s rank correlation and Pearson correlation was based on the nature of the data under analysis, specifically, whether the traits are binary (categorical with two outcomes) or quantitative (continuous).

Binary traits are characterized by a limited number of possible outcomes(usually two), and may not follow to a linear relationship. Spearman’s rank correlation, a non-parametric measure, evaluates the strength and direction of monotonic relationships without assuming linearity. Spearman's correlation is based on the ranks of the data rather than the actual values. Spearman's correlation assesses the consistency of rank increase between variables as their values increase, making it suitable for evaluating associations in scenarios where the relationship may be nonlinear or ordinal.

Pearson correlation is well-suited for quantitative traits that are assumed to have a linear relationship. It measures the strength and direction of the linear association between two variables, assuming a linear relationship and measuring how well the data conform to a straight line. When analyzing quantitative traits, Pearson correlation provides insight into how closely the data points align with a linear trend, capturing both the direction and strength of the linear relationship.

In summary, the selection between Spearman’s rank correlation and Pearson correlation depends on the nature of the traits. Spearman’s rank correlation is favored for binary traits or contexts where a monotonic relationship is more suitable, while the Pearson correlation coefficient is more appropriate for quantitative traits with an assumed linear relationship. The paramount consideration is to employ a correlation method that aligns with the underlying characteristics and distribution of the data.

Results

1.16.Hepatic steatosis classification and phenotype characterization. Line 90-125.

Please revise the following sentence for better clarity and understanding. “The abovementioned quantitative phenotypes mostly had positive correlations with HSC from 0.176 with HFFA to 0.426 with HCF ($P_{adj} < 0.05$, post-hoc Wilcoxon rank-sum test, Supplementary Figure S1).”

Clarify the differences in cutoffs between the applied “hepatic steatosis classification (HSC) model” and the "extreme hepatic steatosis classification (eHSC) model." Provide a clear explanation for the choice of 30-40% for eHS-I and 90% for eHS-II compared to HS-I and HS-II.

A1.16: We have added in the Materials and Methods section that “Individuals were categorized into three levels based on the severity of fatty liver: lipid droplets constituting less than 10% of the area in the control group (Ctrl), 10% to 50% in mild–moderate group (HS-I), and more than 50% in severe group (HS-II). For extremely typical individuals that could represent each group (nearly no lipid droplets in Ctrl,

30% - 40% in HS-I and over 90% lipid droplets in HS-II), oil red O staining was performed to verify lipid droplet accumulation." See lines 127-129 and 517-521.
1.17.Genetic determinants of hepatic steatosis. Line 127-142.
Please clarify why the authors introduced the variable abdominal fat percentage (AFP) as a covariate in the GWA analysis of HSC instead of using AFW directly, given that AFW had the highest heritability (h2) among the variables.
A1.17: The abdominal fat percentage (AFP) was calculated based on the body weight (BW) and abdominal fat weight (AFW). AFP was considered a superior covariate compared to AFW, because it takes into account the relative proportion of abdominal fat in relation to the entire body weight. While AFW provided information on the absolute amount of abdominal fat, AFP normalized this measure by considering the individual's overall body weight.
This normalization is particularly beneficial when dealing with variations in body size or weight among individuals. Using AFP as a covariate allowed for a more standardized assessment of the impact of abdominal fat on the outcome of interest. It helped to mitigate the potential confounding effects of body weight variations, ensuring that the influence of abdominal fat was evaluated independently of overall body size.
In contrast, using AFW alone might not adequately account for differences in body weight among subjects, potentially leading to biased or confounded results in statistical analyses. Although AFW displayed the highest heritability at 0.48 among the variables, the heritability of AFP also reached a notable level, standing at 0.46.
In summary, AFP, derived from the ratio of abdominal fat weight to body weight, offers a more robust and standardized measure for statistical modeling and analysis, making it a superior covariate compared to using AFW alone.
1.18.Why did the authors not directly study the associations between AFW (who had the highest h2) and genetic variants, especially when discussing the heritability comparison in Figure 2A.
A1.18: Abdominal fat constitutes another significant form of lipid deposition. We have conducted a relevant study that mainly focus on AFW, and the manuscript is currently being prepared.
1.19.As only Padjust<0.05 was considered as significant, please just condense the results by deleting "538 and 1797 genes that were significantly positively and negatively correlated with hepatic steatosis, which were identified by a $P < 0.05$."
A1.19: This unnecessary statement has been removed.
1.20.The authors applied Spearman'rank correlation analysis and identify 217 (98+119) genes significantly associated with hepatic steatosis. Then they used ANOVA and validated 83 of the 217 were significantly differed between three hepatic steatosis groups. Next, they stated "Among 232 candidate genes, 48 were reported to be involved in hepatic steatosis, NAFLD, or hepatic lipid metabolism in humans and rats….". Explain how the number of 232 candidate genes comes out? Additionally, provide information on how many of the 48 annotated genes were part of the 83 genes consistently identified by both Spearman's rank correlation analysis and ANOVA.
A1.20: Spearman's rank-based correlation (SRC) analysis was conducted to explore the relationship between the gene expression and hepatic steatosis classifications. We identified 98 genes that exhibited a significant positive correlation and 119 genes that displayed a significant negative correlation with hepatic steatosis after FDR correction (Padj < 0.05). Additionally, ANOVA analysis identified 98 significantly differentially expressed genes (Padj < 0.05, FDR correction), with 83 of them (84.7%) aligning with the genes identified by SRC. The total number of genes, 232, corresponds to the combined results from SRC and ANOVA analyses. In other words, 98 (SRC, positive) + 119 (SRC, negative) + (98-83) (ANOVA specific) = 232. Besides, 15 of the 48 annotated genes were part of the 83 genes consistently identified by both Spearman's rank correlation analysis and ANOVA, which was supplemented in the manuscript. See lines 201 and 203-205.

1.21.Please also remove the statement "which could be used as a DEG dataset to interpret molecular 190 regulation of hepatic steatosis to some extent.", as it appears more discussion-like than a results.
A1.21: This unnecessary statement has been deleted.
1.22.Multiple omics data reveal the molecular regulation mechanism for hepatic steatosis. Line 192-203. This section is more like a summary but not results. Readers could hardly get the information that how phosphatidylcholine (PC) and fatty acid β-oxidation activity are related to hepatic steatosis. Consider integrating this paragraph with the following sections (Line 204-235, Line 236-254, and Line 255-274) to provide a cohesive narrative of key findings on PEMT/phosphatidylcholine (PC) and fatty acid β-oxidation

activity, and their links to the progression of hepatic steatosis.
A1.22: We have integrated this paragraph with the corresponding sections. See lines 219-222, 255-256 and 276.
1.23.Microbiome signatures of hepatic steatosis and their association with host genetics Line 276-282. Much more details are needed to describe the overall composition of the gut microbiota of chickens, not just the number of ASVs, such as location differences among segments, top abundant taxon (ref: Microbiome 10, 127 (2022). https://doi.org/10.1186/s40168-022-01319-7).
A1.23: The alpha and beta diversities in five sampling sites have been supplemented in the revised manuscript, along with the dominant taxa in the phylum and genus level. See lines 305-318.
1.24.Microbiability estimation. Line 282-299
Why the actual m2 (0.15~0.27) was significantly higher than the simulated m2 (average ranged from 0.02 to 0.04, $P < 0.05$, Supplementary Fig. S5A). Discuss the potential confounding impacts of genetics on gut microbiota.
A1.24: It seemed that the concept of microbiability was not interpreted clearly. Similar to heritability, microbiability (m2) was defined as the relative proportion of the total variance attributable to the gut microbial community. It offered a holistic perspective on the collective impact of the microbiota on phenotypes, with minimal association with host genetics except for their interaction. Higher m2 values indicated a more influential role of the microbial community in shaping the target phenotype. The significant difference observed between the actual m2 values (ranging from 0.15 to 0.27) and the simulated m2 values (average ranging from 0.02 to 0.04, $P < 0.05$, Supplementary Fig. S5A) suggested that the microbiability estimates were not attributable to random chance. This discrepancy implies that the observed microbiability genuinely reflects the associations between gut microbiota and the hepatic steatosis-related phenotypes.
Concerning the potential confounding impacts of genetics on gut microbiota, host genetics can play a substantial role in shaping the composition and function of the gut microbiome. The genetic influence on the gut microbiota can arise through various mechanisms, including host genetic factors that affect immune function, mucosal structure, and metabolic processes. Additionally, host genetic variants may directly influence the abundance and diversity of certain microbial taxa. The complex interplay between host genetics and the gut microbiota is an active area of research. To better understand and control for the potential confounding effects of genetics, future studies could consider incorporating genetic data into their analyses. This might involve conducting microbial heritability and microbial GWAS.
1.25.Identification of crucial microbes for hepatic steatosis. Line 300-332
Line 307-308. Address the inconsistency between the methods described in Line 307-308 and the Methods section.
A1.25: We apologize for the lack of clarity in the statement “consistent with the microbiability estimation”. What we meant to convey was "Given that the m2 for hepatic steatosis is highest in the duodenum, we focused on the duodenal microbiota". We utilized m2 to assess the contribution of the microbial communities in each intestinal segment to hepatic steatosis. The m2 value of the duodenum reached 0.26, significantly higher than that of other segments, indicating a more profound impact of duodenal microbiota on hepatic steatosis. We have added more precise information in the manuscript. See lines 343-345.
1.26.Line 316. Include a description of the LEfSe analysis in the Methods section.
A1.26: Additional details regarding the LEfSe analysis has been included in the Methods section. See lines 775-784.
1.27.The authors reported microbial associations with HSC, HTG and HCF levels, but why not the associations between AFW and microbial profiles?
A1.27: We apologize for omitting this section in the results. The microbial associations with AFW, hepatic and serum biochemical indicators were calculated, and Figure 5 has been updated to include the associations between AFW and microbes added.
1.28.Lactobacillus might play compensatory roles in alleviating hepatic steatosis. Line 333-359
Modify the whole section as a lot of sentences are more suitable for discussions rather than presentation of results.
A1.28: This section has been revised, and additional data has been included to further support our findings. See lines 372-385.
Discussion
1.29.The current content appears lengthy, lacking a clear emphasis on key points. I recommend the authors refining it to enhance coherence and focus, making it evident to readers why these findings contribute to the broader understanding of hepatic steatosis.

A1.23: We restructured the logic of our Discussion section and refined the content.

Response to comments from Anonymous Referee #2
Throughout
2.1.In this research article, Sun et al. made great effort to investigate mechanisms of hepatic steatosis in chickens through the utilization of multi-omics integrated analysis methods. The study presents intriguing findings that contribute to our understanding of the development and prevention of hepatic steatosis in chickens, particularly through its exploration of three key aspects of molecular regulation routes. However, one prominent area that requires attention is the lack of further verification of the identified pathways. This constitutes a major limitation of the study. To ensure the robustness of the findings, it is crucial to conduct additional pathway verification experiments or employ complementary approaches.
A2.1: Thank you for your suggestions. This study mainly focused on the integrated analysis of large-scale omics data, providing crucial insights for molecular research in this field.
Title
2.2.The word 'Molecular phenomes' in the title does not appear in PubMed or Google Scholar. It seems that the author intended to refer to molecular phenomics or molecular phenome?
A2.2: We intended to refer to molecular phenomics. It has been corrected. See line 4.
Introduction
2.3.The referrals about heritability being 0.38 to 0.7 lack reference, and these reports must be conditioned on a specific cohort setup. Authors shall clearly state so. Besides, the authors should provide more detailed information and additional references to support the points made in lines 38-46.
A2.3: We carefully reviewed the literature and incorporated additional references on the heritability of fatty liver into the revised Introduction section. The cited references have been expanded to include host information. See lines 43-45.

2.4.Conduct more comprehensive literature review particularly about how the hepatic steatosis in chicken is a good study model or approxy of human’s equivalent problem. This will elevate the scientific impact of this work.
A2.4: Chickens, especially the domestic chicken, have been used as model organisms in various research areas because of their biological characteristics, ease of breeding, and similarity to other vertebrates, including humans. Additional supplements have been included in the revised manuscript. See lines 86-89.
Results
2.5.Wilcoxon rank-sum test also yields beta (median of difference), which shall be reported in the results and marked on the figures. This applies to all the Wilcoxon tests in this study.
A2.5: The Wilcoxon rank-sum test yields beta value and P value, which have been included in the revised manuscript.
2.6.In addition, not sure, for example, “from 4.9 to 7.4”, is it mean or median value? This applies to all the mentions that were based on Wilcoxon tests.
A2.6: The values were mean values in each group.
2.7.It is unclear how the extreme hepatic steatosis classification (eHSC) model was established, and it is not mentioned in the Methods section. Why were HTG, PTG, HCF, HFFAs, PHDL and a few other short-listed here in particular, what about other relevant metabolites?
A2.7: We have supplemented in the Materials and Methods section that “Individuals were categorized into three levels based on the severity of fatty liver: lipid droplets constituting less than 10% of the area in the control group (Con), 10% to 50% in mild–moderate group (HS-I), and more than 50% in severe group (HS-II). For extremely typical individuals that could represent each group (nearly no lipid droplets in Con, 30% - 40% in HS-I and over 90% lipid droplets in HS-II), oil red O staining was performed to verify lipid droplet accumulation.” See lines 517-521.
Hepatic steatosis, commonly known as fatty liver disease, refers to the accumulation of fat in liver cells. Hepatic steatosis involves the accumulation of various biochemical indicators, including hepatic TG, FFA, CF and serum TG, TC, HDL, LDL and VLDL. Besides, TBAs in the liver and serum can influence the lipid metabolism. Therefore, we comprehensively characterized these biochemical indicators using commercial kits to provide a more detailed understanding of the condition and its metabolic consequences. Concerning other metabolites, widely-targeted metabolome was performed and relevant metabolites were qualified in the following Results section.
2.8.Figure 1H shows that HTG performed better in the eHSC model compared to the HSC model (Figure

1B). However, the results of eCon and eHS-I regarding HCF in the eHSC model performed worse than in the HSC model (Figure 1C). Please provide an explanation for this phenomenon.
A2.8: The individuals involved in the eHSC model were selected based on the lipid droplet accumulation in the H&E liver histology. This method of classifying hepatic steatosis is internationally standardized. The biochemical indicators exhibit a certain trend (increase or decrease) with the severity of hepatic steatosis, but this trend is not a completely linear relationship.
2.9.At the first glance, I thought "it is quite a stretch to derive the three regulation routes from three aspects (fig. 3A), unless authors can provide more data evidence." Then, I realized that authors explained the three aspects one by one afterwards. This arrangement is a bit strange and confusing, lack of signal words. Authors shall think a better way or order to represent the data.
A2.9: We are sorry to make the paragraph confusing. We have integrated this paragraph with the corresponding sections. See lines 219-222, 255-256 and 276.
2.10.In fig. 3b, why to not use scientific notations.
A2.10: We updated the Fig. 3 and scientific notations were widely used in Fig. 3B to 3H.
2.11.In fig. 3, the representation of "0, 1, 2" on X-axis shall be put in the figure legend, and significance of involved comparisons shall be marked with "*" in the figure (this applies to fig 5B and C as well). This will make the figure easier to read.
A2.11: The representation of "0, 1, 2" on X-axis has been demonstrated in the figure legend and the significance level has been marked with "*" in the figure.
2.12."con" is not a standard abbrev of control, but "ctrl" is.
A2.12: Thank you for your suggestion. We have replaced it with "ctrl".
Materials and Methods
2.13.Authors shall explicitly introduce how to compute microbiability (m2), instead of just referring to a cited publication.
A2.13: We have provided the detailed information in Methods and Materials section. See lines 685-694.
2.14."FDR corrected P-value" is not a standard expression.
A2.14: We have changed "corrected" to "adjusted" in our revised manuscript. See line 645.
2.15.Suggest to deposit all the computational scripts to a publicly assessable platform, such as github and code ocean.
A2.15: Thank you for your suggestion. We will consider it.
Discussion
2.16.Consider changing the verb in line 392 and 427, 'propose' and 'suggest', to the past tense. Please ensure that there are no tense issues in other parts as well.
A2.16: The verbs "propose" has been changed to the past tense, and the entire revised manuscript has been thoroughly reviewed. See line 412.
Conclusion
2.17.should be written in the past tense
A2.17: The Conclusion section has been rewritten in the past tense. See lines 467-476.

2.18.It's interesting that three molecular regulation routes for hepatic steatosis was elucidated through the application of multi-omics integrated analysis methods. However, it remains essential to ascertain the extent to which these pathways, individually or collectively, align with the actual biological context.
A2.18: Insights were derived from the integrated analysis of multiple omics data. Considering hepatic steatosis as a complex trait, it is genetically regulated by polygenes. Therefore, we posited that these regulatory pathways collectively influence the development of fatty liver disease. However, it is essential to acknowledge the temporal dimension, with certain pathways potentially acting in a sequential manner.
