# Supplementary material for: Mechanisms of hepatic steatosis in chickens: integrated analysis of the host genome, molecular phenomics and gut microbiome
Source: Gigascience. 2024 Jun 5;13:giae023. doi: 10.1093/gigascience/giae023 (PMC11152177; doi:10.1093/gigascience/giae023)
Supplement: giae023_Response_to_Reviewer_Comments_Revision_1 [file giae023_response_to_reviewer_comments_revision_1.pdf]

Author's Response To Reviewer Comments

Dear Editor,
We are grateful to you and reviewers for your valuable comments and suggestions on our manuscript (GIGA-D-23-00122). We have made all necessary revisions to improve the quality of our manuscript.
In the revised manuscript, we provided a more detailed interpretation regarding “gut-liver axis” at the introduction section, and shortened the lengthy description. With respect to results, we have added the specific meaning of the values in each group and interpreted the phenomenon derived from HTG and HCF. Furthermore, we have refined the depiction of the three regulatory routes deduced from multi-omics data for hepatic steatosis. Besides, we have deposited our scripts to Github at https://github.com/FangrenLan97/gigascience. Meanwhile, all figures and tables, including supplementary files, were double-checked.
The modifications are highlighted in the revised manuscript. The responses to the comments are listed below. We believe that we have carefully addressed all the comments and hope that the modifications meet with your approval.
Thank you again and we look forward to hearing from you soon.
Sincerely,
Ning Yang, Ph.D.
Professor of Poultry Genetics and Breeding
China Agricultural University
Beijing 100193, China
Tel: +86-10-6273 1351
E-mail: nyang@cau.edu.cn; cjsun@cau.edu.cn

Response to comments from Anonymous Referee #1
Introduction
1.1.Line56-73 —a very general description (not suitable for a research paper), consider replacing it with specific details on how gut microbiota are involved in gut-liver axis, promoting metabolic disorders, including liver disease (extending Line 74-Line 75).
A1.1: We have revised the introduction section to specifically describe the complex crosstalk along the gut-liver axis. See lines 60-68.
1.2.Line91-97 —modify the very lengthy description like " Chickens are ideal for large-scale studies due to their high reproductive capacity and low cost. Fatty liver in chickens leads to significant declines in egg production and quality, making it a major cause of non-infectious mortality. Therefore, studying the molecular control of fatty liver in chickens is scientifically valuable and economically important, offering potential for molecular breeding to reduce the condition."
A1.2: We have modified these sentences as the reviewer suggested. See lines 89-94.
1.3.Line98-104—provide specific details and key findings in your research, rather than the general description. (i.e., doi.org/10.1016/j.xcrm.2024.101431)
A1.3: This section has been revised. See lines 95-104.

Response to comments from Anonymous Referee #2
2.1.In addition, not sure, for example, “from 4.9 to 7.4”, is it mean or median value? This applies to all the mentions that were based on Wilcoxon tests.
-----shall indicate in the manuscript.
A2.1: The specific meaning of the values in this aspect has been interpreted. See lines 112-126.
2.2.Figure 1H shows that HTG performed better in the eHSC model compared to the HSC model (Figure 1B). However, the results of eCon and eHS-I regarding HCF in the eHSC model performed worse than in the HSC model (Figure 1C). Please provide an explanation for this phenomenon.
-----what I meant was to interpret for biological findings.
A2.2: The phenomenon described could be attributed to several factors. The eHSC model's stricter criteria for classification may lead to a more accurate representation of extreme hepatic steatosis cases (eCtrl and

eHS-II), as individuals in these groups exhibit more pronounced lipid droplet accumulation. However, this strictness may also result in a less precise classification of intermediate cases (eCtrl and eHS-I), where the boundaries between groups become more ambiguous. Moreover, HTG levels may be a more sensitive indicator of extreme hepatic steatosis compared to HCF. HTG specifically measures the amount of triglycerides in the liver, which are directly related to the presence of lipid droplets. In contrast, HCF may encompass a wider range of lipid species, some of which may not be as closely linked to the severity of hepatic steatosis.
2.3.At the first glance, I thought "it is quite a stretch to derive the three regulation routes from three aspects (fig. 3A), unless authors can provide more data evidence." Then, I realized that authors explained the three aspects one by one afterwards. This arrangement is a bit strange and confusing, lack of signal words. Authors shall think a better way or order to represent the data.
-----still lack of signal words, and the three aspects are mingled with other sections. Authors shall definitely figure out a better way to present this in a structured manner.
A2.3: This part of results was restructured to enhance the clarity and logical coherence. See lines 219-297.
2.4.In fig. 3b, why to not use scientific notations.
----- on the basis of 10, using "e" to represent 10 is not a formal representation
A2.4: Fig. 3 was updated, and scientific notations were used in Fig. 3B to 3H.
2.5.In fig. 3, the representation of "0, 1, 2" on X-axis shall be put in the figure legend, and significance of involved comparisons shall be marked with "*" in the figure (this applies to fig 5B and C as well). This will make the figure easier to read.
---- the representation of "0, 1, 2" on X-axis shall be put in the figure legend, i.e., what do "0,1,2" represent?
A2.5: For Figure B-H, 0 refers to the eCtrl group, 1 for eHS-I, and 2 for eHS-II. The representation of "0, 1, 2" on X-axis has been demonstrated in the figure legend. See lines 1185-1186.
2.6."FDR corrected P-value" is not a standard expression.
---- shall write like "at a FDR of 0.05 by adjusting p-values using the Benjamini- Hochberg (to check whether this is indeed your case) procedure".
A2.6: We have revised this sentence. See line 644-645.
2.7.Suggest to deposit all the computational scripts to a publicly assessable platform, such as github and code ocean.
---- shall make it happen.
A2.7: Thank you for your suggestion. We have deposited our scripts of eQTL mapping and SMR at Github (https://github.com/FangrenLan97/gigascience).
2.8.It's interesting that three molecular regulation routes for hepatic steatosis was elucidated through the application of multi-omics integrated analysis methods. However, it remains essential to ascertain the extent to which these pathways, individually or collectively, align with the actual biological context.
---- not properly addressed. This is also relevant to A2.1 where authors make excuse by saying this study merely focused on integrated analysis, however, some types of validation or verification shall be made, could be from literature or from another customized analysis.
A2.8: Thank you for your comments. In our study, we not only integrated multi-omics data to validate the findings but also utilized assay kits to measure diverse biochemical indicators and metabolites for experimental confirmation of the multi-omics results. Specifically, when exploring the relationship between hepatic steatosis and FFA, metabolic profilesrevealed that the quantity of HFFAs increased significantly with the severity of hepatic steatosis (Fig. 3B), which was verified by ELISA assay kits for 673 individuals (Fig. 1C). Moreover, we integrated metabolome data and hepatic ELISA to verify the relationship between hepatic steatosis and metabolites (MDA and 4-HNE) derived from the peroxidation of FFA. Besides, biochemical indicators (TG, TC, TBA and FFA) in liver and serum (686 samples for each), coupled with metabolite abundances involved in methionine cycle (folic acid, Met, SAMe, Hcy, SAH) in liver and duodenal chyme (10 samples/group) were determined with ELISA assay kits.
2.9.It's Table S2: what headers stand for, and it is sloppy to use "af, allele1", and "E-1" to represent numbers considering that this can be simply written as 0.xx.
A2.9: We have modified these headers and used "allele1, allele2, allel1 frequency" instead. See Table S2.
