# Supplementary material for: Mechanisms of hepatic steatosis in chickens: integrated analysis of the host genome, molecular phenomics and gut microbiome
Source: Gigascience. 2024 Jun 5;13:giae023. doi: 10.1093/gigascience/giae023 (PMC11152177; doi:10.1093/gigascience/giae023)
Supplement: giae023_Response_to_Reviewer_Comments_Revision_2 [file giae023_response_to_reviewer_comments_revision_2.pdf]

Author's Response To Reviewer Comments

1.1.The logic within the Introduction section, particularly between lines 54 to 78, needs further enhancement for improved clarity and coherence. This section requires extensive rewriting. Please focus and concentrate more specifically on the specific context of your research (why liver disease, and why your focused on gut-liver axis), avoiding overly general, or unrelated information. i.e., (1) Since your study does not include functional analysis of TMAO-producing microbial genes, such background on the relationship between TMAO, gut microbiota, and metabolic disorders appears out of context. I recommend removing it and substantially reducing text describing those unrelated content. (2) line 54-55, 55-56, lines 68-70 provide very general information.
A1.1: We have rewritten this section to specifically describe the details on how gut microbiota are involved in gut-liver axis, thus promoting liver disease. See lines 54-73.
