# Supplementary material for: Mechanisms of hepatic steatosis in chickens: integrated analysis of the host genome, molecular phenomics and gut microbiome
Source: Gigascience. 2024 Jun 5;13:giae023. doi: 10.1093/gigascience/giae023 (PMC11152177; doi:10.1093/gigascience/giae023)
Supplement: giae023_Response_to_Reviewer_Comments_Revision_3 [file giae023_response_to_reviewer_comments_revision_3.pdf]

Author's Response To Reviewer Comments

1.GigaScience only allows 3 co-first authors and 2 corresponding authors at most, please make sure the number meets our requirements.
A1: The number of co-first authors and corresponding authors have been corrected to 3 and 2, respectively.
2.Some changes have been made and some comments added in the manuscript, please check all the changes and resolve all comments.
A2: The changes have been carefully checked and accepted, and all the references mentioned and abbreviations have been added in the manuscript. However, we did calculate SimpleM threshold for GWAS significance level, thus no changes was made here.
