# Supplementary material for: Mechanisms of hepatic steatosis in chickens: integrated analysis of the host genome, molecular phenomics and gut microbiome
Source: Gigascience. 2024 Jun 5;13:giae023. doi: 10.1093/gigascience/giae023 (PMC11152177; doi:10.1093/gigascience/giae023)
Supplement: giae023_Reviewer_1_Report_Original_Submission — Huanzi Zhong, Ph.D. -- 11/15/2023 [file giae023_reviewer_1_report_original_submission.pdf]

Reviewer Report

Title: Mechanisms of hepatic steatosis in chickens: integrated analysis of the host genome, molecular phenomics and gut microbiome

Version: Original Submission **Date:** 11/15/2023

Reviewer name: Huanzi Zhong, Ph.D.

Reviewer Comments to Author:

In this manuscript, the authors have applied multi-omics technologies, including host genome sequencing, liver transcriptome, proteome, metabolome, and 16S rRNA gene amplicon sequencing to investigate their interaction patterns and potential impacts on hepatic steatosis in hens. They reported similarly high host heritability (h2=0.25) and duodenal microbiability (m2=0.26) of hepatic steatosis in chicken and revealed potential links between specific host genomic variants, gene expressions, methionine cycle-related metabolites, and gut Lactobacillus (duodenal) in regulating hepatic steatosis. The paper is generally well written with comprehensive description of the methods and figure data.
The major and minor comments are listed below:
Introduction: The Introduction is lengthy but lacks cited references, and specific and important details related to research on host genetics, gut microbiota, and hepatic steatosis. More words are needed to underscore the importance as well as the rationale for choosing farm chicken as research model. Thus, I suggest rewriting and shortening the introduction section to include necessary references, provide more detailed information, and offer a clear rationale for conducting the study.
Example 1: Line 43-44 "A fatty liver is strongly influenced by host genetics with heritability ranging from 0.38 to 0.70." The statement on heritability lacks a cited reference and lacks information on the studied hosts (e.g., human, mice, or chicken).
Example 2: Line 44-46, lack of references of the two mentioned genes (PNPLA3 and TM6SF2).
Example 3: line 55-57 The description of the "gut-liver axis" needs more depth, as this is much more than the basic description you have provided "gut microbiota-derived products directly to the liver and the liver feedback route of metabolites to the gut."
Example 4: line 57-59 "Gut microbiota ferment indigestible carbohydrates and proteins, thereby yielding metabolites that play a crucial role in gut homeostasis and liver substrate metabolism and functions [5-7]." The sentence provided very general information, the roles of gut microbiota are vague and lacks specificity. E.g., "what were the specific liver substrate metabolism and functions?"
Example 5: Line 62, the term "chicken lipid metabolism regulation model" as well as the "regulation model" mentioned in below sections require clarification. What is a regulation model?
Example 6: line 70-73. Add references to support the claim that farm animals have more genetic variations than mouse models. "patricianly suitable" should be "particularly suitable".
Material and Methods:
Experimental Design: Line 463-486
1) Please provide more details on experimental design: "Birds were generated from two hatches and reared in individual cages under similar conditions."

2) Please provide more details on the "basic diet" mentioned in the experimental design. Specify whether the diet is corn-based, wheat-based, or another type.
3) Clarify how the control (Con, N = 217, healthy), mild hepatic steatosis (HS-â… , N = 265), and severe hepatic steatosis (HS-â…¡, N = 204) groups were defined.
eQTL mapping and mendelian randomization analysis: Line 599-600:
The instrumental variables in the mendelian randomization analysis (MR) are very important and they must be independent of each other. Please explain how instrumental variables for summary data-based MR analysis were defined, ensuring their independence from each other. Describe how these variables were identified as independently associated with the exposure factor.
Heritability and microbiability estimation: Line 626
Only the batches and the first five host genetic principal components were corrected when estimating the phenotypic variance explained by the gut microbiota (microbiability estimation). However, gut microbial composition could also be partially influenced by host genetics. In human studies, PRS or SNPs that associated with the investigated phenotype are also considered as covariates when estimating microbiability (doi:10.1038/nature25973). Please provide some description to explain this.
Widely targeted metabolome: Line 682
Specify the number of liver tissue samples subjected to targeted metabolome.
Statistical Analysis: line712-731
Given the non-normally distributed nature of microbiota abundance profiles, clarify whether any data transformations were performed before using ANOVA to determine differences in gut microbiota.
Explain the rationale for using Spearman's rank correlation for taxa with occurrence between 30% to 60% but Pearson correlation coefficient for those with occurrence â‰¥ 60% of samples. Provide a clear explain for this differentiation on correlation methods.
Result:
Hepatic steatosis classification and phenotype characterization. Line 90-125.
Please revise the following sentence for better clarity and understanding. "The abovementioned quantitative phenotypes mostly had positive correlations with HSC from 0.176 with HFFA to 0.426 with HCF (Padj < 0.05, post-hoc Wilcoxon rank-sum test, Supplementary Figure S1)."
Clarify the differences in cutoffs between the applied "hepatic steatosis classification (HSC) model" and the "extreme hepatic steatosis classification (eHSC) model." Provide a clear explanation for the choice of 30-40% for eHS-I and 90% for eHS-II compared to HS-I and HS-II.
Genetic determinants of hepatic steatosis. Line 127-142.
Please clarify why the authors introduced the variable abdominal fat percentage (AFP) as a covariate in the GWA analysis of HSC instead of using AFW directly, given that AFW had the highest heritability (h2) among the variables.
Why did the authors not directly study the associations between AFW (who had the highest h2) and genetic variants, especially when discussing the heritability comparison in Figure 2A.
Crucial genes for hepatic steatosis. Line169-191
1) As only Padjust<0.05 was considered as significant, please just condense the results by deleting "538 and 1797 genes that were significantly positively and negatively correlated with hepatic steatosis, which were identified by a P < 0.05."
2) The authors applied Spearman'rank correlation analysis and identify 217 (98+119) genes significantly

associated with hepatic steatosis. Then they used ANOVA and validated 83 of the 217 were significantly differed between three hepatic steatosis groups. Next, they stated "Among 232 candidate genes, 48 were reported to be involved in hepatic steatosis, NAFLD, or hepatic lipid metabolism in humans and rats….".
Explain how the number of 232 candidate genes comes out? Additionally, provide information on how many of the 48 annotated genes were part of the 83 genes consistently identified by both Spearman's rank correlation analysis and ANOVA.
3) Please also remove the statement "which could be used as a DEG dataset to interpret molecular 190 regulation of hepatic steatosis to some extent.", as it appears more discussion-like than a results.
Multiple omics data reveal the molecular regulation mechanism for hepatic steatosis. Line 192-203.
This section is more like a summary but not results. Readers could hardly get the information that how phosphatidylcholine (PC) and fatty acid Î²-oxidation activity are related to hepatic steatosis. Consider integrating this paragraph with the following sections (Line 204-235, Line 236-254, and Line 255-274) to provide a cohesive narrative of key findings on PEMT/phosphatidylcholine (PC) and fatty acid Î²-oxidation activity, and their links to the progression of hepatic steatosis.
Microbiome signatures of hepatic steatosis and their association with host genetics Line 276-282.
Much more details are needed to describe the overall composition of the gut microbiota of chickens, not just the number of ASVs, such as location differences among segments, top abundant taxon (ref: Microbiome 10, 127 (2022). https://doi.org/10.1186/s40168-022-01319-7).
Microbiability estimation. Line 282-299
Why the actual m2 (0.15~0.27) was significantly higher than the simulated m2 (average ranged from 0.02 to 0.04, $P < 0.05$, Supplementary Fig. S5A). Discuss the potential confounding impacts of genetics on gut microbiota.
Identification of crucial microbes for hepatic steatosis. Line 300-332
1) Line 307-308. Address the inconsistency between the methods described in Line 307-308 and the Methods section.
2) Line 316. Include a description of the LEfSe analysis in the Methods section.
3) The authors reported microbial associations with HSC, HTG and HCF levels, but why not the associations between AFW and microbial profiles?
Lactobacillus might play compensatory roles in alleviating hepatic steatosis.
Line 333-359
Modify the whole section as a lot of sentences are more suitable for discussions rather than presentation of results.
Discussion: The current content appears lengthy, lacking a clear emphasis on key points. I recommend the authors refining it to enhance coherence and focus, making it evident to readers why these findings contribute to the broader understanding of hepatic steatosis.

Methods

Are the methods appropriate to the aims of the study, are they well described, and are necessary controls included? Choose an item.

Conclusions

Are the conclusions adequately supported by the data shown? Choose an item.

Reporting Standards

Does the manuscript adhere to the journal's guidelines on minimum standards of reporting? Choose an item.

Choose an item.

Statistics

Are you able to assess all statistics in the manuscript, including the appropriateness of statistical tests used? Choose an item.

Quality of Written English

Please indicate the quality of language in the manuscript: Choose an item.

Declaration of Competing Interests

Please complete a declaration of competing interests, considering the following questions:

- Have you in the past five years received reimbursements, fees, funding, or salary from an organisation that may in any way gain or lose financially from the publication of this manuscript, either now or in the future?
- Do you hold any stocks or shares in an organisation that may in any way gain or lose financially from the publication of this manuscript, either now or in the future?
- Do you hold or are you currently applying for any patents relating to the content of the manuscript?
- Have you received reimbursements, fees, funding, or salary from an organization that holds or has applied for patents relating to the content of the manuscript?
- Do you have any other financial competing interests?
- Do you have any non-financial competing interests in relation to this paper?

If you can answer no to all of the above, write 'I declare that I have no competing interests' below. If your reply is yes to any, please give details below.

I declare that I have no competing interests.

I agree to the open peer review policy of the journal. I understand that my name will be included on my report to the authors and, if the manuscript is accepted for publication, my named report including any attachments I upload will be posted on the website along with the authors' responses. I agree for my

report to be made available under an Open Access Creative Commons CC-BY license (http://creativecommons.org/licenses/by/4.0/). I understand that any comments which I do not wish to be included in my named report can be included as confidential comments to the editors, which will not be published.

Choose an item.

To further support our reviewers, we have joined with Publons, where you can gain additional credit to further highlight your hard work (see: https://publons.com/journal/530/gigascience). On publication of this paper, your review will be automatically added to Publons, you can then choose whether or not to claim your Publons credit. I understand this statement.

Yes Choose an item.
