# Supplementary material for: Mechanisms of hepatic steatosis in chickens: integrated analysis of the host genome, molecular phenomics and gut microbiome
Source: Gigascience. 2024 Jun 5;13:giae023. doi: 10.1093/gigascience/giae023 (PMC11152177; doi:10.1093/gigascience/giae023)
Supplement: giae023_Reviewer_1_Report_Revision_1 — Huanzi Zhong, Ph.D. -- 11/15/2023 [file giae023_reviewer_1_report_revision_1.pdf]

Reviewer Report

Title: Mechanisms of hepatic steatosis in chickens: integrated analysis of the host genome, molecular phenomics and gut microbiome

Version: Revision 1 **Date:** 2/20/2024

Reviewer name: Huanzi Zhong, Ph.D.

Reviewer Comments to Author:

The authors have effectively addressed most of my previous concerns. However, the introduction still requires substantial revision to focus and specify its content more directly to your research field (a few examples are provided below).
1) Line56-Line73 â€”a very general description (not suitable for a research paper), consider replacing it with specific details on how gut microbiota are involved in gut-liver axis, promoting metabolic disorders, including liver disease (extending Line 74-Line 75).
2ï¼‰Line91-97 â€”modify the very lengthy description like " Chickens are ideal for large-scale studies due to their high reproductive capacity and low cost. Fatty liver in chickens leads to significant declines in egg production and quality, making it a major cause of non-infectious mortality. Therefore, studying the molecular control of fatty liver in chickens is scientifically valuable and economically important, offering potential for molecular breeding to reduce the condition."
3) Line98-104â€”provide specific details and key findings in your research, rather than the general description. (i.e., doi.org/10.1016/j.xcrm.2024.101431)
Please do carefully modify it to provide a clear, concise, and focused framework for the rest of the manuscript, setting the stage for your important research contributions.

Methods

Are the methods appropriate to the aims of the study, are they well described, and are necessary controls included? Choose an item.

Conclusions

Are the conclusions adequately supported by the data shown? Choose an item.

Reporting Standards

Does the manuscript adhere to the journal's guidelines on minimum standards of reporting? Choose an item.

Choose an item.

Statistics

Are you able to assess all statistics in the manuscript, including the appropriateness of statistical tests used? Choose an item.

Quality of Written English

Please indicate the quality of language in the manuscript: Choose an item.

Declaration of Competing Interests

Please complete a declaration of competing interests, considering the following questions:

- Have you in the past five years received reimbursements, fees, funding, or salary from an organisation that may in any way gain or lose financially from the publication of this manuscript, either now or in the future?
- Do you hold any stocks or shares in an organisation that may in any way gain or lose financially from the publication of this manuscript, either now or in the future?
- Do you hold or are you currently applying for any patents relating to the content of the manuscript?
- Have you received reimbursements, fees, funding, or salary from an organization that holds or has applied for patents relating to the content of the manuscript?
- Do you have any other financial competing interests?
- Do you have any non-financial competing interests in relation to this paper?

If you can answer no to all of the above, write 'I declare that I have no competing interests' below. If your reply is yes to any, please give details below.

I declare that I have no competing interests

I agree to the open peer review policy of the journal. I understand that my name will be included on my report to the authors and, if the manuscript is accepted for publication, my named report including any attachments I upload will be posted on the website along with the authors' responses. I agree for my report to be made available under an Open Access Creative Commons CC-BY license (http://creativecommons.org/licenses/by/4.0/). I understand that any comments which I do not wish to be included in my named report can be included as confidential comments to the editors, which will not be published.

Choose an item.

To further support our reviewers, we have joined with Publons, where you can gain additional credit to further highlight your hard work (see: https://publons.com/journal/530/gigascience). On publication of this paper, your review will be automatically added to Publons, you can then choose whether or not to claim your Publons credit. I understand this statement.

Yes Choose an item.
